# An interferon signature identified by RNA-sequencing of mammary tissues varies across the estrous cycle and is predictive of metastasis-free survival

**DOI:** 10.18632/oncotarget.2148

**Published:** 2014-06-30

**Authors:** Antoine M. Snijders, Sasha Langley, Jian-Hua Mao, Sandhya Bhatnagar, Kathleen A. Bjornstad, Chris J. Rosen, Alvin Lo, Yurong Huang, Eleanor A. Blakely, Gary H. Karpen, Mina J. Bissell, Andrew J. Wyrobek

**Affiliations:** ^1^ Life Sciences Division, Lawrence Berkeley National Laboratory, Berkeley, CA

**Keywords:** estrous cycle, mammary gland, RNA-sequencing, Type-1 interferon, low-dose ionizing radiation (LDIR), immunity, breast cancer, genetic susceptibility

## Abstract

The concept that a breast cancer patient's menstrual stage at the time of tumor surgery influences risk of metastases remains controversial. The scarcity of comprehensive molecular studies of menstrual stage-dependent fluctuations in the breast provides little insight. To gain a deeper understanding of the biological changes in mammary tissue and blood during the menstrual cycle and to determine the influence of environmental exposures, such as low-dose ionizing radiation (LDIR), we used the mouse to characterize estrous-cycle variations in mammary gene transcripts by RNA-sequencing, peripheral white blood cell (WBC) counts and plasma cytokine levels. We identified an estrous-variable and hormone-dependent gene cluster enriched for Type-1 interferon genes. Cox regression identified a 117-gene signature of interferon-associated genes, which correlated with lower frequencies of metastasis in breast cancer patients. LDIR (10cGy) exposure had no detectable effect on mammary transcripts. However, peripheral WBC counts varied across the estrous cycle and LDIR exposure reduced lymphocyte counts and cytokine levels in tumor-susceptible mice. Our finding of variations in mammary Type-1 interferon and immune functions across the estrous cycle provides a mechanism by which timing of breast tumor surgery during the menstrual cycle may have clinical relevance to a patient's risk for distant metastases.

## INTRODUCTION

The concept that the menstrual stage of a breast cancer patient at the time of surgery for tumor removal can influence surgical success and survival is of obvious clinical interest, but the evidence remains controversial [1-6]. A number of studies concluded that surgery in the luteal phase of the menstrual cycle (the time between ovulation and menses) was correlated with increased survival [7-12]. Further studies showed increased survival in women with high levels of progesterone, which is associated with the luteal phase [13, 14]. However, many reports, including several prospective studies, failed to demonstrate a survival difference between surgeries performed at differing menstrual stages [1, 15-17]. Multiple reasons can account for these conflicting results such as inadequately sized cohorts, non-standardized menstrual phases and reliance on patient records for menstrual cycle data in retrospective studies, which could be unreliable. Also, patients whose primary tumor has already metastasized or patients with micrometastases without presenting with clinical disease at the time of surgery might gain no benefit from surgery at a particular menstrual stage. Clearly, the absence of a foundational understanding of normal variations in gene expression in the mammary gland across the estrous cycle in controllable model systems makes it difficult to resolve this controversy, and to determine what to measure other than hormonal status and menstrual stage.

The evidence from animal models, although limited, provides substantial support for the concept that estrous stages vary in their sensitivity for tumor metastasis and in their sensitivity to mammary carcinogens. In C3HeB/FeJ mice, there was a doubling of metastasis free survival when mammary tumors were surgically removed near estrus versus post estrus [18]. When melanoma cells were injected into the tail vein of C57BL/6 mice at proestrus or metestrus there was no difference in metastatic burden in the lung, but ~1/3 of the mice injected in metestrus developed ovarian metastasis compared to none after injection in proestrus [19]. Also, the risk for carcinogen-induced mammary cancers appears to differ depending on the estrus stage at the time of exposure in animal models [20, 21]. In Wistar rats the highest incidence of mammary tumorigenesis was observed after 2.6 Gy whole body X-ray exposure followed by treatment with diethylstilbestrol as a promoting agent in diestrus and proestrus [20]. In contrast, the mammary tumor incidence in Sprague Dawley rats exposed to a single dose of N-methyl-N-nitrosourea was significantly lower in diestrus compared to proestrus and estrus [21]. Given these observations in animal models, it is apparent that there is a need for a deeper understanding of natural variations in cellular, hormonal and molecular components and mammary tissue mechanisms across the menstrual cycle, to identify estrous-stage windows of susceptibility for environmental exposures, and of differential risk for distant metastases.

There are dramatic morphological, cellular and molecular changes within the mammary gland as females progress through the cycle, which are well characterized in rodent models [22, 23]. The murine estrous cycle consists of four stages (proestrus, estrus, metestrus and diestrus), which repeats every 4-5 days, making the mouse a practical model system for molecular studies of the mammalian estrous cycle. At the start of proestrus, which corresponds to the follicular phase in humans, estrogen levels surge causing increases in progesterone secretion and luteinizing hormone (LH) and follicle-stimulating hormone (FSH), which initiates ovulation. Hormone levels return to baseline when ovulation occurs in estrus, and progesterone levels increase sharply in diestrus at the beginning of the post-ovulation phase, corresponding to the luteal phase in humans. During the estrous cycle mammary glands undergo extensive expansion and reorganization through a highly coordinated process of proliferation and apoptosis, which is under tight hormonal control [22, 24].

The immune system has been proposed as a mediating mechanism in surgical outcome and patient survival and the metastatic spread of breast cancer cells [25]. Furthermore, estrous stage-dependent natural killer cell activity was correlated with breast cancer metastatic potential [26]. Building towards a more foundational understanding of mammary physiology and hormonal changes across the estrous cycle, and the role of the immune system in breast cancer, research presented here identifies estrous variable genes in the mouse mammary gland and estrous variation in peripheral white blood cells counts and plasma cytokines and white blood cells.

The immune system is highly sensitive to damage from exposure to ionizing radiation [27]. With the exception of radiation therapy, human exposures to high doses of ionizing radiation are fortunately rare. However, exposures to low-dose ionizing radiation (LDIR) are common and pervasive from medical diagnostic and therapeutic procedures, indoor air and soil, and nuclear industries and fuel storage and from air travel. Even though ionizing radiation is considered a weak carcinogen compared to certain chemical agents, it is a well-documented risk factor for human breast cancer. However, risk estimates from LDIR exposures suffer from statistical uncertainties and an insufficient molecular understanding of tissue responses and genetic susceptibility [28-31]. Lymphoid tissues and circulating lymphocytes are extremely sensitive to radiation, and small doses can lead to cellular depletion, although enhancement of the immune response by LDIR has also been observed [32, 33]. Interestingly, low lymphocyte counts have been shown to be independent predictors of cancer patient survival [34, 35] suggesting that LDIR may play a role in advancing the metastatic spread of cancer cells by depressing immune-surveillance mechanisms under some conditions. However, potential tissue responses of LDIR exposure that are associated with metastatic risks remain poorly understood.

We undertook a comprehensive approach to identify molecular fluctuations across the menstrual cycle in the murine mammary gland, as well as fluctuations in white blood cells in the context of exposure to LDIR as an indicator of immune sensitivity. Specifically, we used the mouse model to characterize (1) the variations in gene expression in mammary glands (MG) as females transitioned through the normal estrous cycle, and (2) the variations in peripheral white blood cell (WBC) counts across normal estrous and after exposure to LDIR (10 cGy) in strains that are sensitive (BALB/c) and resistant (C57BL/6) to radiation-induced mammary tumors. Our investigation identified several temporal clusters of estrous-variable genes from which we developed a Type-1 interferon expression signature that is correlated with lower probabilities of metastasis in breast cancer patients. We also found that peripheral WBC counts varied across the estrus cycle, and that 10cGy LDIR exposure reduced lymphocyte counts in the cancer-susceptible (BALB/c) but not the cancer-resistant (C57BL/6) murine genotype highlighting the importance of genetic susceptibility in exposed populations.

## RESULTS

### Estrous-variable expression profiles in mammary glands of BALB/c and C57BL/6 mice

**Figure 1:**
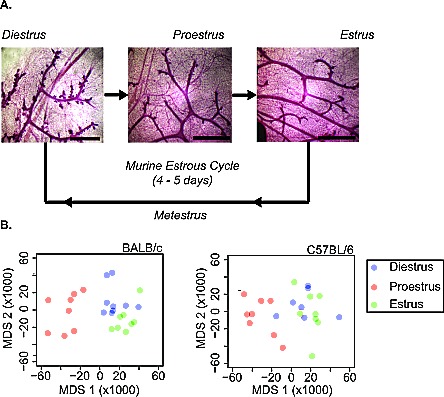
Mammary gland morphology changes with estrous cycle stage A. Morphology of whole-mounted carmine-alum stained fourth mammary gland of 16-week-old female BALB/c mice at the diestrus, proestrus and estrus stages of the estrous cycle. Diestrus is characterized by lobuloalveolar budding, which is not seen at the other stages. B. To determine overall similarity of replicates from each time point, metric multidimensional scaling (MDS) was applied to gene-level normalized counts from all non-outlier replicates of the RNA-seq mapped mammary gland transcript reads from BALB/c (left) and C57BL/6 (right) mice at diestrus (blue), proestrus (red) and estrus (green) stage. The first two MDS axes explain 29.0% and 11.2% of the variability in the BALB/c dissimilarity matrix and 22.4% and 13.2% for C57BL/6.

Individual female mice were assigned to diestrus, proestrus and estrus stages by vaginal cytology (Figure S1). The histological variation in the mammary epithelial tree architecture across the stages of estrous is illustrated in Figure 1A. MGs of BALB/c and C57BL/6 mice in diestrus, proestrus and estrus stages were excised and analyzed by RNAseq (12 mice per stage per strain; inguinal lymph nodes were removed before analyses). The overall similarity of replicates from each time point was determined using metric multidimensional scaling (MDS) of gene-level normalized counts from all non-outlier replicates. By this metric, the proestrus stage of the estrous cycle was the most different from the diestrus and estrus stages (Figure 1B). The MG expression profiles in diestrus resembled those in estrus, suggesting that the major morphological difference between these two stages (Figure 1A) is not accompanied by a major difference in gene expression.

**Figure 2:**
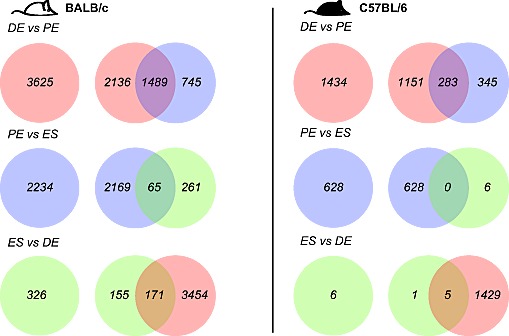
Robust gene transcript changes in proestrus compared to estrus and diestrus Differentially expressed genes were identified across the estrous cycle in BALB/c mice in white (left), C57BL/6 in black (right) based on fold-change +/−1.2 fold and FDR 5% (DE, diestrus; PE, proestrus; ES, estrus).

**Figure 3:**
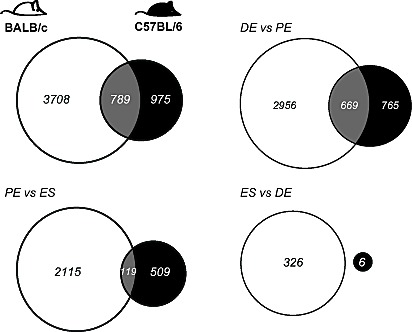
Mammary gland gene transcript levels depend on genotype and estrous stage Genotype comparisons of genes that show differential expression across the estrous cycle in BALB/c mice (in white) and C57BL/6 (in black), based on +/−1.2 fold change and 5% FDR. Overlapping transcripts are identified in gray. (DE, diestrus; PE, proestrus; ES, estrus).

Expressed transcripts were compared pairwise between stages (1.2 fold cut-off, FDR 5%, Figure 2; Tables S2 and S3). BALB/c mice showed more transcript changes among stages than C57BL/6. For BALB/c and C57BL/6, the largest change in expression occurred from diestrus to proestrus (3625 and 1434 genes, respectively). Many genes whose expression changed from proestrus-to-estrus were the same as those that changed from diestrus-to-proestrus genes (1489 (41%) for BALB/c; 283 (20%) for C57BL/6, respectively). For the genes that changed in both transitions, the direction of change (up or down) from diestrus to proestrus was opposite to the direction of change from proestrus to estrus; i.e. genes upregulated from diestrus to proestrus were downregulated from proestrus to diestrus, and vice versa. There was significant conservation between the two strains in genes that varied in their expression across the estrus cycle (Figure 3). For the diestrus to proestrus transition, there was a 669-gene overlap between the strains (BALB/c, 18% of 3625 genes; C57BL/6, 47% of 1434 genes). For the proestrus to estrus transition, there was only a 119-gene overlap (BALB/c, 5% of 2234 genes; C57BL/6, 19% of 628 genes). No genes with altered expression levels between estrus and diestrus were conserved.

**Figure 4:**
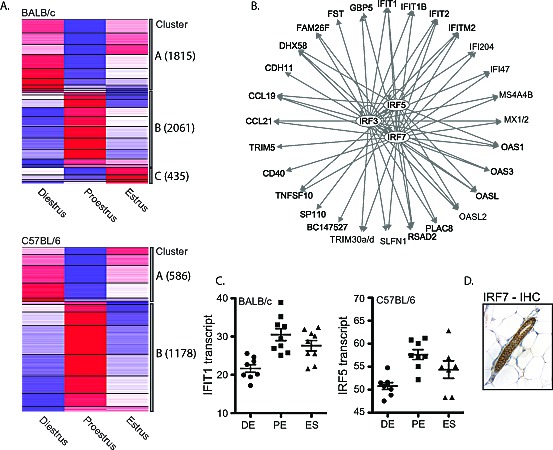
Significant upregulation of IRF7-regulated genes in BALB/c and C57BL/6 mammary glands in proestrus A. Cluster analysis of all unique genes whose transcript levels changed significantly between any two stages of estrous in BALB/c (top) and C57BL/6 (bottom). Two main clusters were identified; genes with decreased expression in proestrus (Cluster A) and genes with increased expression in proestrus (Cluster B). Increased expression is indicated in red, while decreased expression is indicated in blue. B. Upstream transcriptional regulator analyses of overlapping genes in Cluster B between the two strains revealed a significant association with IRF3, IRF5 and IRF7. C. Two examples of interferon-regulated genes IFIT1 and IRF5, which show significant upregulation in proestrus in BALB/c and C57BL/6 mammary gland tissues compared to diestrus, respectively (IFIT1: 1.9-fold, q=0.129; IRF5: 1.3-fold q=0). D. A representative image of IHC analysis of IRF7 protein in the mouse mammary gland.

The estrous-variable genes of both genotypes were assigned to 2 main clusters: genes that were downregulated (Cluster A) or upregulated (Cluster B) in proestrus compared to diestrus and estrus, respectively (Figure 4A). Cluster A (and Cluster C observed only in BALB/c) was significantly enriched for genes associated with lipid metabolism (5.16E-18<p<2.17E-02) and small molecule biochemistry (5.16E-18<p<2.17E-02). The significant upstream transcriptional regulators of the intersection of BALB/c and C57BL/6 Cluster A genes include: SREBF2, PPARA, SREBF1, and PPARG among others (Table 1, Table S4 and Figure S5). Notably, a large number of Cluster A genes in BALB/c, but not C57BL/6, were found to be associated with breast cancer (225 genes; p=1.04E-13) and transcriptionally regulated by TP53 (171 genes; p=8.14E-22). Of particular interest is the enrichment in BALB/c Cluster A of genes involved in the oxidative stress response (p=2.09E-08) including Superoxide Dismutase 1, 2 and 3. We observed decreased transcript levels of SOD1, SOD2 and SOD3 in proestrus of BALB/c mice, but not C57BL/6 (Figure S6). These data are supported by observations that sex steroids can modulate SOD activity and suggest that BALB/c mice may be more vulnerable to oxidative damage in the mammary gland during proestrus [36-38].

**Table 1:** Upstream transcriptional regulators associated with Clusters A and B in BALB/c and C57BL/6

Cluster A Upstream Regulators	p-value	Target Molecules	Cluster B Upstream Regulators	p-value	Target Molecules
SREBF2	1.33E-13	13	IRF7	2.70E-17	24
PPARA	3.26E-10	21	IRF3	7.64E-17	23
MYC	1.03E-09	30	TRIM24	5.78E-15	18
SREBF1	1.79E-09	14	STAT1	3.43E-13	24
PPARGC1A	2.82E-09	13	IRF5	1.00E-10	12
PPARG	2.19E-08	18	IRF1	1.15E-06	12
ESRRA	2.65E-08	12	NFATC2	1.56E-06	12
HIF1A	5.46E-08	16	STAT3	1.60E-06	20
EPAS1	2.83E-07	11	SPI1	4.81E-06	12
HTT	3.47E-07	23	STAT4	2.13E-05	13
FOXO1	6.48E-07	14	SMARCA4	4.35E-05	15
CEBPA	1.52E-05	14	NFKB1	8.29E-05	12
NFE2L2	8.60E-05	13			

Cluster B genes were enriched for antimicrobial and inflammatory responses in both genotypes (2.95E-22<p<5.37E-10) under the upstream regulation of three major transcriptional regulators: IRF3, IRF5 and IRF7 (p<1.00E-10; Figure 4B, 4C, Table 1 and Table S4). IRF3, 5 and 7 coordinately regulate the Type-1 interferon response, which is an integral part of the innate immune response to viral and bacterial infections. Immunohistochemical analyses revealed IRF7 protein expression predominantly located in the mammary ductal epithelium in both BALB/c and C57BL/6 mice (Figure 4D).

Clusters A and B from both genotypes were compared to an interferome database consisting of 3747 human and 2825 mouse interferon-regulated genes [39]. Both clusters were enriched for interferon regulated genes (Cluster A: 27% for BALB/c (492/1815); 23% in C57BL/6 (135/586); Cluster B: 36% for BALB/c (741/2061); 43% in C57BL/6 (506/1178). The majority (>70%) of interferon-regulated genes in both clusters were of the Type 1 category, providing supporting evidence for an estrus-variable Type-1 interferon response in the mouse mammary gland.

A single acute LDIR exposure had little effect on gene expression in MGs of BALB/c and C57BL/6 mice, and no detectable effect on the interferon clusters. Mice were irradiated in diestrus (10 cGy or sham; n=12 per strain per treatment), and MGs were collected three days later at proestrus for RNAseq analyses. Using strict selection criteria (1.2 fold-change and FDR 5%) yielded no known modulated genes in BALB/c mice and only 11 in C57BL/6 (Table S5).

### Hormonal treatment induced Type-1 interferon-related expression in MGs of oophorectomized mice

**Figure 5:**
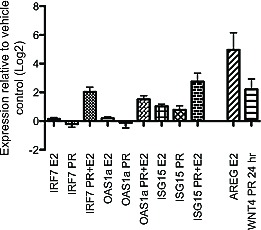
Mammary gland interferon response depends on combined estradiol and progesterone treatment of oophorectomized mice Gene expression of IRF7, ISG15 and OAS1a was measured in mammary glands of adult oophorectomized BALB/c mice injected with progesterone, estradiol, or a combination of both hormones relative to vehicle-injected mice. Gene expression of AREG and WNT4 was measured as positive controls for estradiol and progesterone treatment, respectively.

To test the hypothesis that the common estrus-variable Type-1 interferon response is under hormonal regulation, we treated non-cycling oophorectomized BALB/c mice with estradiol, progesterone or both hormones together and measured mammary expression of IRF7 and two interferon stimulated genes OAS1a and ISG15. Expression of AREG and WNT4, known downstream targets of estradiol and progesterone, respectively, were upregulated in mammary tissues after hormone treatment (Figure 5). ISG15 was found to be upregulated by either estradiol or progesterone alone (~2-fold). However, IRF7 and OAS1a gene expression was not significantly modulated by estradiol or progesterone treatment alone, but combined hormone treatment resulted in robust upregulation of IRF7 (~4-fold, p<0.002), ISG15 (~7-fold, p=0.02) and OAS1a (3-fold, p=0.02) (Figure 5). Together these data suggest that the upregulation of Type-1 interferon genes in Cluster B at proestrus is hormonally regulated and dependent on the presence of both estradiol and progesterone, which are both known to be increased during proestrus in the rodent estrous cycle.

### Tumor Type-1 interferon signature correlates with metastasis free survival in human breast cancer patients

**Figure 6:**
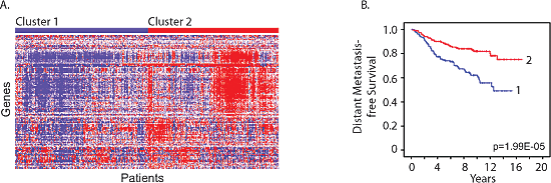
Estrous cycle gene signature correlates with human breast cancer metastasis-free survival A. Cluster diagram of breast tumor gene expression levels of the shared 117-gene protective signature in human breast cancer patients (Figure 6A; GSE6532) identified two main clusters. B. Patients with high expression of these 117 genes (Cluster 2) had a significant increase in metastasis free survival compared to patients with lower levels (Cluster 1) (log rank test p=1.99E-05).

We next determined whether the expression levels of mouse cluster B genes in human breast tumor tissue were associated with metastasis-free survival in human breast cancer patients. Stepwise Cox regression was applied to the overlapping set of cluster B genes between the two genotypes to identify a smaller signature associated with increased metastasis-free survival. Among the Cluster B genes, we identified a signature of 117 genes, which were individually associated with increased metastasis-free survival (Table S6). This signature was enriched for interferon-regulated genes (63%). Expression levels of this protective signature in patient breast tumor samples were used to cluster human breast cancer patients (Figure 6A). The patients with higher expression of the 117-gene signature had a significant longer duration of metastasis free survival compared to patients with lower levels (Figure 6B; log rank test p=1.99E-05) providing further support for an innate estrous-variable mechanism in the mammary gland involving genes associated with suppressing metastasis in human patients.

### Estrous-variability and LDIR-induced reduction in WBCs and cytokines in susceptible BALB/c but not relatively resistant C57BL/6 mice

**Figure 7:**
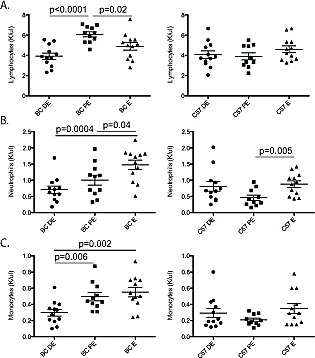
Circulating white blood cells fluctuate across the estrous cycle A five-part complete white blood cell differential was measured (K/microliter) for neutrophils, lymphocytes, monocytes, eosinophils and basophils in BALB/c (BC; n=12 for each stage) and C57BL/6 (C57; n=12 for each stage) mice across diestrus (DE), proestrus (PE) and estrus (ES) stages. Significance was determined using a non-paired T-test. A. Blood lymphocyte levels were significantly increased in proestrus compared to diestrus and estrus in BALB/c mice but not C57BL/6 mice. B. Blood neutrophil levels were significantly increased in estrus compared to proestrus and diestrus in BALB/c mice. However, in C57BL/6 mice a significant increase was observed in neutrophil levels in estrus compared to proestrus. C. Blood monocyte levels were increased in estrus and proestrus compared to diestrus in BALB/c mice. No change in monocyte levels was observed in C57BL/6.

The levels of the major subtypes of peripheral WBCs and several cytokines varied across the estrus cycle in unirradiated BALB/c, but not in C57BL/6 mice (Figure 7). In unexposed BALB/c mice (a) lymphocyte counts were higher in proestrus than in diestrus (Figure 7A; p<0.0001) or estrus (p=0.02 proestrus vs estrus) and (b) neutrophil and monocyte counts were higher in estrous than diestrus (Figure 7B; p=0.0004 and 7C; p=0.002, respectively), and intermediate values were observed in proestrus. Blood plasma concentrations of 32 cytokines were measured in females in diestrus and estrus (n=10 per stage per strain; ~10-week-old). Cytokine concentrations were highly correlated between strains with a slope of unity (Figure S7A; R^2^=0.998). Eotaxin and IL-12-p40 showed estrous-variation in BALB/c but not C57BL/6 mice (Figure S7B and C; p<0.001). Twenty-one cytokines were slightly more concentrated in C57BL/6 than in BALB/c mice (p<0.05; 1.3-2.3-fold; Figure S7A), but none of the 32 cytokines showed estrus variation in C57BL/6 mice.

**Figure 8:**
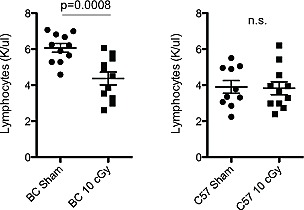
Low dose radiation exposure decreases blood lymphocyte counts in BALB/c Circulating blood lymphocyte levels in BALB/c (left) and C57BL/6 (right) mice three days after exposure to 10 cGy X-ray irradiation or sham (n=10-11 per strain). Significance was tested using an unpaired T-test.

Three days after LDIR treatment (10 cGy X-ray) BALB/c mice but not C57BL/6, showed significant depressions in lymphocyte counts (1.4-fold; p=0.0008; Figure 8) and cytokine levels (Figure S8). LDIR exposure reduced blood plasma concentrations of Eotaxin, TNF-α and MCP1 (1.3-fold; p <=0.01; Figure S8), with a trend for IL-12 depression (Figure S8; p=0.08). In summary, among the cytokines, Eotaxin and IL-12 showed estrous variation in unirradiated mice as well as LDIR-induced depressions in BALB/c, but there were no differences across estrus in C57BL/6 mice.

## DISCUSSION

### Estrous-variable expression profiles in mammary glands of mice are enriched for metabolism and interferon-related genes

We present new evidence in support of the hypothesis that the menstrual stage at the time of tumor surgery may influence personal risk for breast cancer metastases. Using the mouse, we identified (1) variations in MG gene expression as females transitioned through the normal estrous cycle, and (2) variations in peripheral WBC counts across normal estrous and after exposure to LDIR in radiation-induced mammary-tumor-susceptible strain of mice, but not detected in the tumor-resistant strain. RNA-seq analyses identified two major temporal clusters of estrous-variable genes, (1) a cluster that was down regulated in proestrous and associated with glucose and lipid metabolism, and (2) a cluster that was upregulated in proestrous and enriched for Type-1 interferon genes.

During lactation, the mammary gland is one of the most active lipid generating organs and this process is tightly regulated by the endocrine milieu [40, 41]. Increased expression of genes associated with lipid metabolism during diestrus is consistent with mammary alveolar development in this phase of the estrous cycle in preparation for a potential pregnancy and lactation after parturition; when pregnancy does not occur, these alveolar buds regress before the next estrous cycle.

Interferons are produced in response to pathogens including bacteria, viruses or parasites. Type-1 interferons bind to the interferon-α receptor, which leads to activation of interferon stimulated genes (ISGs) through JAK-STAT signaling [42]. The interferon response is tightly regulated to ensure proper clearance of pathogens without inducing autoimmune disease or excessive tissue damage [43], and the transcription factor IRF7 is considered the master regulator of the Type-1 interferon response [44]. Consistent with the literature, we found that estrous-variable cluster B was regulated by IRF7, a known regulator of Type-1 interferon mediated immune responses whose expression was previously associated with metastatic risk in patients [45]. We confirmed the hormonal regulation of IRF7 expression in oophorectomized mice. From the interferon cluster, we developed a 117-gene signature that is associated with lower levels of metastasis in breast cancer patients. LDIR exposure, our model agent of low-dose environmental stress, had no detectable effect on the expression of the interferon cluster in either strain of mice. However, exposure to LDIR reduced the peripheral blood lymphocyte counts and cytokine levels in the tumor-susceptible, but not in the tumor-resistant genotype.

### Innate estrous-variable Type-1 interferon response in mammary gland may suppress breast cancer metastasis

**Figure 9:**
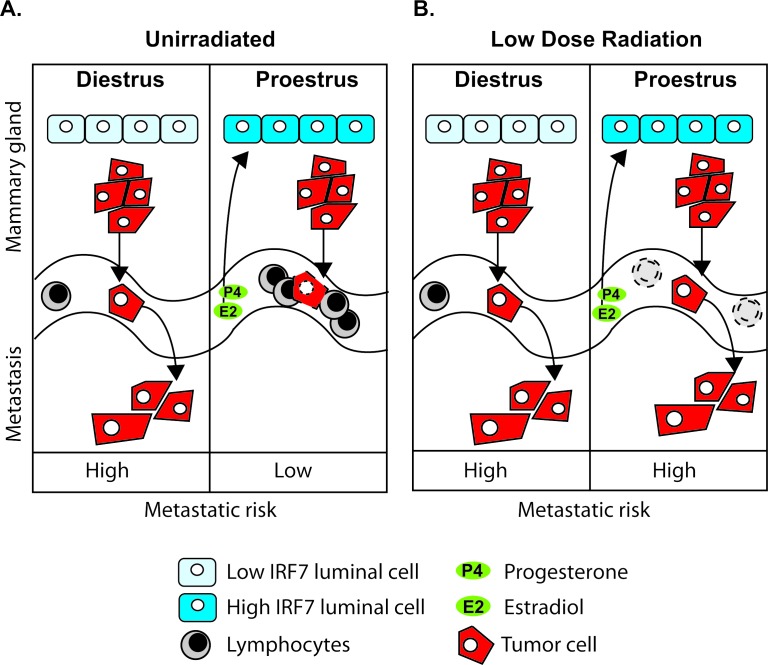
Schematic of estrous-variable and LDIR effects on metastatic risks in mammary tumor susceptible individuals A. The production of IRF7 by mammary epithelial cells spikes in proestrus compared to estrus and diestrus (the schematic for estrus is not shown since it is the same as diestrus). Our model predicts that the increased IRF7 expression and interferon signature in mammary tissues in proestrus is dependent on the presence of both estradiol and progesterone, and together with the increase in peripheral blood immune cells in proestrus, reduces risk for metastasis (low metastatic risk). B. After low-dose radiation exposure (LDIR) BALB/c mice have a reduction in peripheral blood lymphocytes; which was not observed in tumor resistant C57BL/6 mice. The interferon signature in proestrus is unaffected by LDIR in both strains. Our model predicts that the radiation induced lymphocyte depression results in a loss of the metastasis protective effect in proestrus due to immune suppression (high metastatic risk).

Our results suggest a model for how estrous-variable Type-1 interferon functions in the mammary gland might interact with genotype-variable and estrous-variable immune functions in peripheral blood to yield differing risks for metastases of nascent transformed MG cells at various stages of the estrous cycle (Figure 9). Our findings indicate that the production of IRF7 by MG epithelial cells spikes in proestrous compared to estrous and diestrus (Figure 9A), resulting in immune recognition and reduced risk for metastasis. The protection against metastases in proestrus is predicted from our findings in the BALB/c and C57BL/6 genotypes. We also showed that BALB/c mice exposed to LDIR have a reduction in peripheral blood lymphocytes (Figure 9B), and our model predicts that BALB/c will have lost the metastasis protective effect at proestrus due to immune suppression. Furthermore, our model predicts that metastatic risk will be increased in humans who experience other causes of immune suppression such as physiological distress, medical treatments, and other xenobiotic exposures. Our findings point to an innate, estrous-dependent mechanism in the mammary gland for fighting infections and suppressing cancer metastasis. This provides mechanistic support for the concept that the phase of a woman's menstrual cycle during breast cancer treatments, such as surgery or radiation could affect longterm survival.

We employed an *in vivo* approach to investigate whether estrous cycling affects baseline transcript expression in mouse mammary tissues, and LDIR responses in both mammary tissue and peripheral blood. RNA-seq transcript analyses identified significant estrous stage dependent enrichment for genes involved in antimicrobial and inflammatory responses in the mammary glands of both BALB/c and C57BL/6 mice. The majority of genes were found to be upregulated during proestrus and significantly associated with transcription regulators IRF3, IRF5 and IRF7, which regulate the Type-1 interferon response, a potent antiviral and antibacterial mediator of innate immunity. We associated upregulation of this interferon signature with increased metastasis-free survival in human breast cancer patients. Our finding for IRF7 is consistent with a recent study that used a mouse model of breast cancer metastasis to show that bone metastases were suppressed in the presence of IRF7 expression, and that this phenomenon depended on interferon signaling and functional immune cells [45]. Our results show that interferon-regulated genes fluctuate with estrous cycle in the mammary gland and that a Type-1 interferon signature is associated with metastasis free survival, independent of metastatic organ site.

These findings raise the important question of whether the human breast has an innate protective interferon-related mechanism that is activated during each menstrual cycle to prevent infections and suppress metastases. A recent RNA-seq study of normal human breast tissues from a small number of healthy premenopausal individuals in two phases of the menstrual cycle did not report fluctuations in interferon related genes as a function of estrous stage [46]. A more detailed comparison between this data set and ours is warranted, using a common RNA-seq analyses method to provide a valid comparison.

We found no expression of Type-1 interferon ligands in the mammary gland, consistent with previous reports [47], which suggests that the estrous cycle dependent interferon response in the mammary gland is mediated by a distantly produced ligand, or is activated through another pathway. For example, the uterus expresses higher levels of interferon epsilon, which protects against viral and bacterial infections, while the cervix, vagina and ovary express lower levels [47]. These authors showed that uterine IFNε expression was estrous cycle stage dependent with the highest expression in estrus, and that expression was induced after exogenous estrogen administration in oophorectomized mice, raising the possibility that uterine IFNε could act distantly on the mammary gland.

Our results show that the interferon response in the mammary gland is induced by the combined treatment with estrogen and progesterone. Prior studies have demonstrated that the estrogen and progesterone receptors can have synergistic and sometimes inhibitory cross-talk in transcriptional regulation [48, 49]. Further research is needed to elucidate the mechanism for estrogen and progesterone interactions in the induction of IFN-related gene expression.

### LDIR decreases peripheral blood lymphocytes and cytokines

We found surprisingly few LDIR induced transcript changes in MG tissue after the single exposure of 10 cGy. In contrast, our previous study with repeated LDIR exposures (four weekly exposures of 7.5 cGy), identified major LDIR responses in BALB/c and C57BL/6 mice up to one month after exposure [50]. Pre-exposure differences in baseline expressions in the MGs of these two strains of mice were enriched in stress response and metabolism genes, and differences in expression after LDIR exposure were in immune response (four hours post exposure) and in proliferation genes (one month post exposure). The discrepancy in the number of LDIR responsive genes between these two studies could be due to different LDIR exposure regimen, sample sizes and sampling times after exposure. The prior study used four animals per group and was not controlled for estrous staging, while our current study was controlled for estrous stage and included up to twelve animals in each experimental condition. Another possibility for finding fewer LDIR response genes in our current study is that we measured the response at 3 days after irradiation at proestrus, which may have missed the early response genes. Also, proestrus is characterized by rapid fluctuations in hormone levels, potentially overwhelming any low-dose expression response. Further studies of the effects of LDIR dose-regimen and time-course sampling in estrous-staged and hormone-controlled animals are needed to address these issues.

Peripheral lymphocyte reduction is common after radiation exposure and depletion kinetics are used for radiation biodosimetry [51, 52]. We found a 1.4-fold decrease in the number of lymphocytes in BALB/c mice after a 10 cGy exposure, but not in C57BL/6 mice. Along with lymphocyte reduction, we observed lower plasma concentrations of the inflammatory cytokines MCP-1, TNFα, Eotaxin and IL-12 in BALB/c mice. TNFα stimulates MCP-1 expression and secretion in a variety of cell types including monocytes, macrophages and endothelial cells. Increased TNFα and MCP-1 expression can promote breast cancer progression, through direct binging to the TNF receptor and through recruitment of macrophages to tumor sites, respectively [53, 54]. Eotaxin is regulated by estrogen and is necessary for eosinophil homing to the uterine stroma during the estrous cycle [55]. IL-12 has been reported to have antitumor effects in a number of tumor models [56-58]. A study on the role of adaptive immunity in tumor dormancy showed that 20% of mice treated with a chemical carcinogen developed tumors. However, when the remaining non-tumor bearing mice were treated with an anti IL-12 (p40), 60% of mice developed tumors [59]. IL-12 and Type-1 interferons regulate innate and Th1-cell-mediated immune responses [60]. IL-12 treatment of BALB/c mice carrying the activated oncogene Her-2/neu delayed mammary tumor onset and reduced tumor multiplicity [61]. Furthermore, intra-tumoral IL-12 delivery resulted in accumulation of IRF7 in tumor cells, which correlated with tumor regression [62]. These data suggest a possible role for IL-12 in mediating the Type-1 interferon response that we observed in the mammary gland during proestrus.

In conclusion, our findings in mice point to a novel, endogenous, estrous stage variable, interferon-based mechanism in the mammary gland that is consistent with the evidence that interferon-based mechanisms are associated with protection against breast cancer metastasis and infectious diseases in humans. Our comparative analyses of two genetically diverse inbred strains of mice with different cancer- and radiation- susceptibilities suggest that this protective mechanism may be dampened at certain stages of the normal estrous cycle, and may also be dampened in sensitive individuals by immune suppression of peripheral blood lymphocytes and inflammatory cytokines after exposure to immunosuppressive agents, such as ionizing radiation. These findings provide a mechanistic framework for the clinical controversy that the timing of breast tumor removal and treatment during the menstrual cycle may influence a patient's risk for metastases. Our findings suggest that metastatic risk at the time of surgery may be increased in patients with suppressed immune systems due to estrous stage, physiological distress, drug and radiation treatments or other exposures. Lastly, our study provides a comprehensive tabulation of estrous variable genes in the mammary glands of young adult mice. This dataset together with as yet unpublished additional data is a foundational resource for future studies to identify estrous stage-dependent mechanisms of differential risk for tumor progression and metastasis, and to identify estrous-stage windows of susceptibility or resistance to cancer therapy, ionizing radiation exposures, and environmental agents that may confer differential risks for breast cancer metastases.

## METHODS

### Mouse Estrous Cycle Determination and Irradiation

Female, virgin BALB/c and C57BL/6 mice (~8 weeks old; Jackson Laboratories) were acclimated for 1 week, and the study was carried out in strict accordance with the Guide for the Care and Use of Laboratory Animals of the National Institutes of Health. The animal use protocol was approved by the Animal Welfare and Research Committee of the Lawrence Berkeley National Laboratory (Protocol File Number 271003). Vaginal cytology was used to determine the stage of the estrus cycle between 9 and 11 weeks of age. Animals were staged in the late morning and no more than twice per week for two weeks. Briefly, the tip of a plastic pipette filled with 20 μL of PBS was gently inserted into the vagina and flushed three times. The final flush was collected and placed on a glass slide and the estrous cycle stage was determined microscopically by the types and relative numbers of cells present based on the following criteria: diestrus stage (DE) contains mostly polymorphonuclear leukocytes (PMNs) and few epithelial cells, proestrus (PE) stage contains mostly nucleated and cornified epithelial cells, estrus (ES) stage contains mostly cornified epithelial cells and the metestrus stage contains mostly cornified epithelial cells, PMNs and a few nucleated epithelial cells (Figure S1). To study effects of LDIR on transcript expression 24 female BALB/c and 24 female C57BL/6 mice were selected in diestrus. For each strain (n=12 per group), mice were exposed to 10 cGy or sham, using a Precision X-ray Inc RAD320 320 kVp X-ray machine, operated at 300 kV, 2 mA (dose rate of 196 mGy/min). Mice were returned to their cage and left undisturbed for 3 days, then euthanized and tissues were harvested. For each strain, tissues were harvested from 12 mice in diestrus (unirradiated), 12 mice in proestrus (sham irradiated), 12 mice in proestrus (10 cGy) and 12 mice in estrus (unirradiated).

### RNA Isolations

The pair of 4th mammary glands was harvested and the inguinal lymph node as well as a second lymph node often present in the distal part of the inguinal mammary gland were removed. Mammary tissues were snap frozen in liquid nitrogen within 10 minutes of euthanasia. Total RNA was isolated by homogenizing the frozen tissue in Trizol reagent (Invitrogen) followed by phase separation using chloroform. RNA was further purified utilizing the RNeasy mini kit (Qiagen; 74104) and DNA was removed using RNase-free DNase (Qiagen; 79254). RNA samples with an RNA integrity number greater than 8 were used for further analyses.

### RNA-Sequencing

Plate-based sample preparation was performed on the PerkinElmer Sciclone NGS robotic liquid handling system, where purified mRNA is converted into cDNA library templates of known strand origin for sequencing on the Illumina Sequencer platform. First, mRNA in the total RNA sample was purified using Invitrogen's DynaBeads mRNA purification kit, which utilizes magnetic beads with bound poly-T oligos to select and purify poly-A containing mRNA molecules. Following purification, mRNA was chemically fragmented with 10X fragmentation solution (Ambion) at 70°C for 3 minutes to generate fragments ranging in size between 250 to 300bp. The fragmented RNA was purified using AMpure SPRI beads (Agencourt) using a ratio of 160:100 beads volume to RNA. The RNA fragments were synthesized into first-strand cDNA using SuperScript II Reverse Transcriptase (Invitrogen) and random hexamer primers (MBI Fermentas) with Actinomycin D in the master-mix to further ensure strand specificity by preventing spurious DNA dependent synthesis during the first-strand cDNA synthesis. First-strand synthesis thermocycler conditions were as follows: incubation at 42°C for 50 minutes and an inactivation step at 70°C for 10 minutes. This step was followed by a purification using AMpure SPRI beads at a ratio of 140/100 beads volume to cDNA and second-strand cDNA synthesis using DNA Polymerase I (Invitrogen), RNase H (Invitrogen), and a nucleotide mix containing dUTP (Roche) that incorporates dUTP in place of dTTP at 16°C for 60 minutes. The ds cDNA fragments were purified using AMpure SPRI beads at a ratio of 75/100 beads volume to cDNA and a second purification using a ratio of 140/100. Using a DNA sample prep kit designed for Illumina sequencing, the purified ds cDNA fragments were then end-repaired to be blunt-ended, phosphorylated for the addition of a single A base tail, and finally ligation of the Illumina adapters, which include unique barcode sequences that allow for the multiplexing of the sample libraries in sequencing. To ensure the strand specificity of the cDNA library templates, AmpErase UNG (Uracil N-glycosylase, Applied Biosystems) is added to the ds cDNA library fragments (37°C for 15 minutes) to cleave and degrade the strand containing dUTP. The ss cDNA is then enriched using 10 cycles of PCR with Illumina TruSeq primers and purified using AMpure SPRI beads at a ratio of 90/100 beads volume to ds cDNA to create the final cDNA library for sequencing. The prepared sample libraries were quantified using KAPA Biosystem's next-generation sequencing library qPCR kit on a Roche LightCycler 480 real-time PCR instrument. The quantified sample libraries were pooled and prepared for sequencing on the Illumina HiSeq sequencing platform utilizing a TruSeq paired-end cluster kit, v3, and Illumina's cBot instrument to generate clustered flowcells for sequencing. Sequencing of the flowcells was performed on the Illumina HiSeq2000 sequencer using a TruSeq SBS sequencing kit, v3, following a 2x100 indexed run recipe.

### RNA-Seq Analyses

### Read mapping and counting

Sequencing data is available from http://genome.jgi.doe.gov/Nonliniradiation/Nonliniradiation.info.html (JGI project ID: 1016164; 1016167; 1008162; 1008165). RNA-seq reads were mapped to the mm10 genome (downloaded from UCSC) using TopHat v2.0.9 [63] with a mate-inner-distance of 80bp and all other parameters default. For each replicate, per-gene counts of uniquely mapped reads were computed using HTSeq v0.5.4p5 [64] and Gencode M2 [65] annotations. Normalized replicate counts were generated using the samr package function, samr.norm.data() [66].

### Differential Gene Expression Analysis

Differential gene expression analysis was conducted in a pairwise manner for 3 time points and for irradiated and control samples. Gene-level counts were computed on independent biological replicates. For each experimental condition, DESeq v1.14.0 [67] was used to assess the within-condition variation observed across biological replicates (Figure S2). To do this, we used leave-one-out analysis, and ran DESeq to identify differentially expressed genes with a given biological replicate within a condition against all other replicates of the same condition. In this setting, our null hypothesis is that, after correcting for multiple testing, the given replicate should show no statistically significant differentially expressed genes. However, in each condition, we found that one to three of our twelve biological replicates returned hundreds of differentially expressed genes. We manually reviewed each of these, and found that gene expression levels were consistent neither with the majority of replicates nor with each other. This may owe to the challenge of timing or tissue dissection. Hence, we excluded these outlier replicates from downstream analysis (Figure S2). Additionally, sequencing failed for 3 replicates in 3 conditions, and these were also removed from further analysis (Table S1). Counts from the remaining replicates served as the input for differential expression analysis using the samr package [68]. This package is more statistically conservative than DESeq, and uses a nonparametric model to assess gene expression [66]. It is not as widely used as DESeq because it requires five or more biological replicates per condition, and few studies achieve this depth of measurement (and hence we could not use this package for our leave-one-out analysis described above). The SAMseq() function was called with response type “two class unpaired” and False Discovery Rate (FDR) = 1 (Tables S2 and S3). Genes with ≥ 1.20 or ≤ 0.83 fold change and with q-values ≤ 0.05 were designated as differentially expressed and used for downstream analysis. In low-count regimes, particularly when a given gene has fewer than ~10 reads in several treatment replicates and most control replicates are zero (or the reverse), samr demonstrates a known failure case, and returns extreme (> +/−10^4^) fold-change values and small, inaccurate p-values. We removed these genes after exhaustive manual vetting. Consistency of samr differential expression analysis was examined for BALB/c and C57BL/6 at each time point comparison. Data were subjected to 10 rounds of sampling without replacement (5 replicates per condition). Samr analysis was then performed on subsampled data, and results were checked for consistency of fold change and reported p-values using IDR v1.1.1. (Figures S3 and S4) [68]. Strain and time point intersections were generated in R v3.0.3 [69] and plotted using the VennDiagram package, v1.6.5 [70].

### Replicate visualization and expression pattern clustering

To examine the overall similarity of replicates from each time point, metric multidimensional scaling (MDS) was applied to gene-level normalized counts from all non-outlier replicates. MDS was performed using the cmdscale() function in R with a Manhattan distance metric. For clustering of genes by expression pattern across time points, the union of all genes passing differential expression significance thresholds at minimally one time point comparison was taken for each strain. Clustering was then performed on per-gene median normalized replicate counts for these sets. Values were standardized to put them on a common scale before clustering. Affinity propagation clustering was performed using the apcluster package with q = 0.1. Clusters were visualized using the gplots package v2.12.1 [71].

### Differential Blood Counts

After euthanasia, blood was collected by cardiac puncture into an EDTA coated syringe and transferred to a centrifuge tube. A small amount of blood (25 μL) was removed into a separate tube and placed on a rocker for 10 minutes before blood count analyses, and the remaining blood was immediately centrifuged (1200 rpm; 10 minutes) to isolate plasma. The Hemavet HV950FS (Drew Scientific) was operated following the manufacturer's recommended protocols. A clean cycle and blank were run prior to the mouse quality control reagent (Drew Scientific) on each day of the experiment. A five-part complete WBC differential was measured (K/μL) for WBC, neutrophil, lymphocyte, monocyte, eosinophil and basophil counts. All Hemavet blood samples were analyzed approximately 10 minutes after collection.

### Plasma Cytokine Measurements

MILLIPLEX®MAP magnetic bead panel kits (Millipore; MCYTMAG-70K-PX32) were used for high-throughput quantification of the following 32 Mouse Cytokines/Chemokines: Eotaxin, G-CSF, GM-CSF, IFNγ, IL-1α, IL-1β, IL-2, IL-3, IL-4, IL-5, IL-6, IL-7, IL-9, IL-10, IL-12(p40), IL-12(p70), IL-13, IL-15, IL-17, IP-10, KC, LIF, LIX, MCP-1, M-CSF, MIG, MIP-1α, MIP-1β, MIP-2, RANTES, TNFα, and VEGF. MagPix® equipment (Luminex Corp., Austin, TX) was utilized to read magnetic beads. Mouse cytokine standards and quality controls samples were used for each 96-well plate run. Individual mouse plasma samples (25 μL/well) were diluted 1:2 in Assay Buffer, run in duplicate following the manufacture's protocol for running and analyzing samples. Cytokine/chemokine concentration for each sample was obtained based on the median fluorescent intensity from standard curves established using the MILLIPLEX®Analyst 5.1 software analysis program. In this program, a 5-parameter logistic or spline curve-fitting method was used to the Median Fluorescent Intensity of the standards were fitted with for calculating cytokine/chemokine concentrations in samples.

### Hormone Treatment and Quantitative RT-PCR

Oophorectomized BALB/c mice (Jackson Laboratories) at ten weeks of age were injected subcutaneously with one of four treatments: (1) a mixture of 1 μg 17β-estradiol (Sigma, E8875) and 1 mg progesterone (Sigma, P0130), (2) 1 mg progesterone alone, (3) 1 μg 17β-estradiol alone or (4) vehicle (sesame oil) for four consecutive days. Mice were euthanized on the fifth day and mammary gland tissues were harvested for RNA isolation. Mammary gland RNA isolations are the same as described earlier. Quantitative RT-PCR analysis was performed following standard methods using GAPDH as endogenous control.

### Cox regression

Expression levels of human orthologs of overlapping genes in cluster B in mammary gland tissue of BALB/c and C57BL/6 mice were identified in breast cancer samples of patients from a curated breast cancer data set (GSE6532; [72]). A single variable Cox proportional-hazards regression analysis was used to identify a set of genes in which the expression is significantly associated with distant metastasis-free survival in this cohort of breast cancer patients (p-value <0.05). Then the patients were classified into two groups using centroid linkage based on this set of genes. A Kaplan-Meier survival curve for distant metastasis-free survival was generated for two groups of patients. Log-rank test was performed to compare the difference in distant metastasis-free survival between patients in the two groups.

## SUPPLEMENTARY MATERIAL FIGUREs AND TABLES











## Abbreviations

Mammary gland (MG)
low-dose ionizing radiation (LDIR)
white blood cell (WBC)
polymorphonuclear leukocytes (PMNs)
diestrus stage (DE)
proestrus stage (PE)
estrus stage (ES)
multidimensional scaling (MDS)
False Discovery Rate (FDR)
