# Supplementary material for: An interferon signature identified by RNA-sequencing of mammary tissues varies across the estrous cycle and is predictive of metastasis-free survival
Source: Oncotarget. 2014 Jun 30;5(12):4011–25. doi: 10.18632/oncotarget.2148 (PMC4147302; doi:10.18632/oncotarget.2148)
Supplement: Supplementary file 1 [file oncotarget-05-4011-s001.pdf]

An interferon signature identified by RNA-sequencing of mammary tissues varies across the estrous cycle and is predictive of metastasis-free survival

Supplementary materials

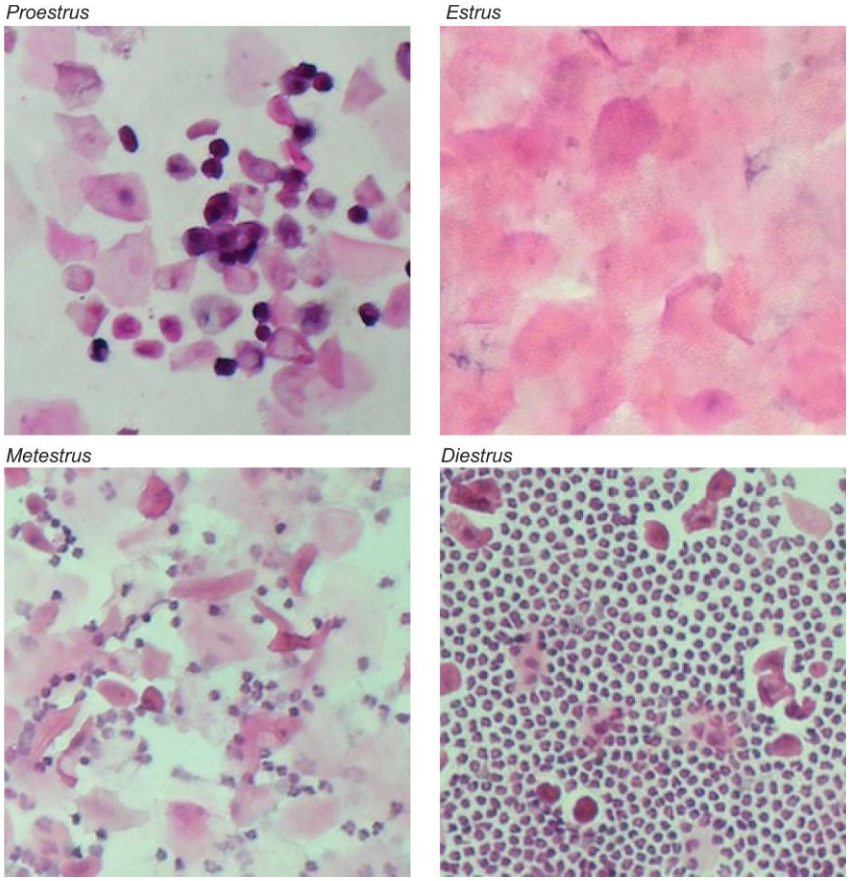

Figure S1: Vaginal cytology representing estrous cycle stages. Vaginal cytology was used to determine the stage of the estrus cycle between 9 and 11 weeks of age. Estrous cycle stage was determined microscopically by the types and relative numbers of cells present based on the following criteria: proestrus (PE) stage contains mostly nucleated and cornified epithelial cells, estrus (ES) stage contains mostly cornified epithelial cells, metestrus stage contains mostly cornified epithelial cells, PMNs and a few nucleated epithelial cells and the diestrus stage (DE) contains mostly polymorphonuclear leukocytes (PMNs) and few epithelial cells.

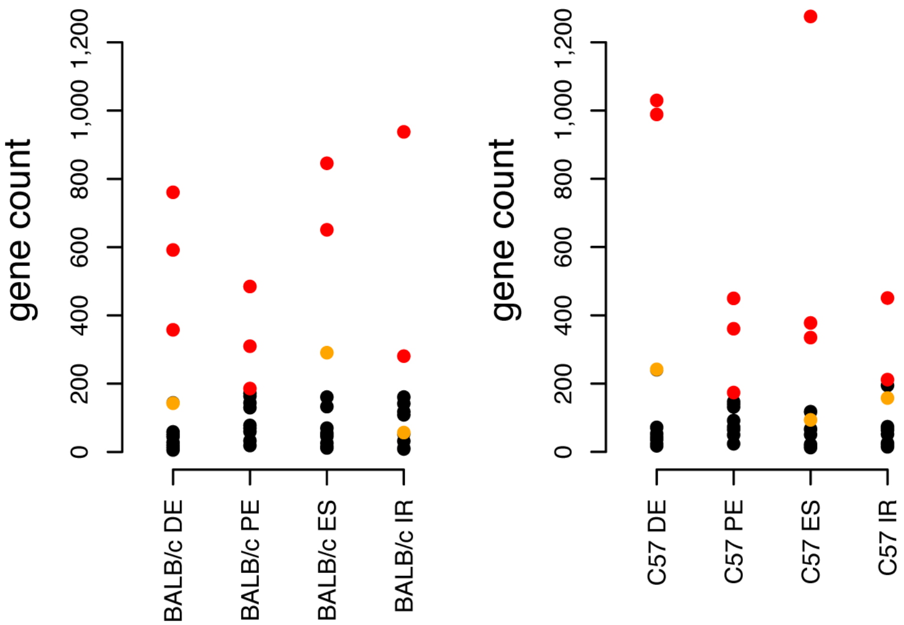

Figure S2: Identification of outlier replicates based on mappable reads and replicate consistency. We conducted within-replicate analysis of sample variation using DESeq. Each replicate was analyzed as "treatment" with the remaining replicates serving as "controls." The total number of genes identified as differentially expressed (p-value ≤ 0.01) were considered for each replicate. Replicates showing the largest within-replicate variation (red) and those with exceptionally low total mapped reads (orange, see Table S1) were filtered from downstream analysis.

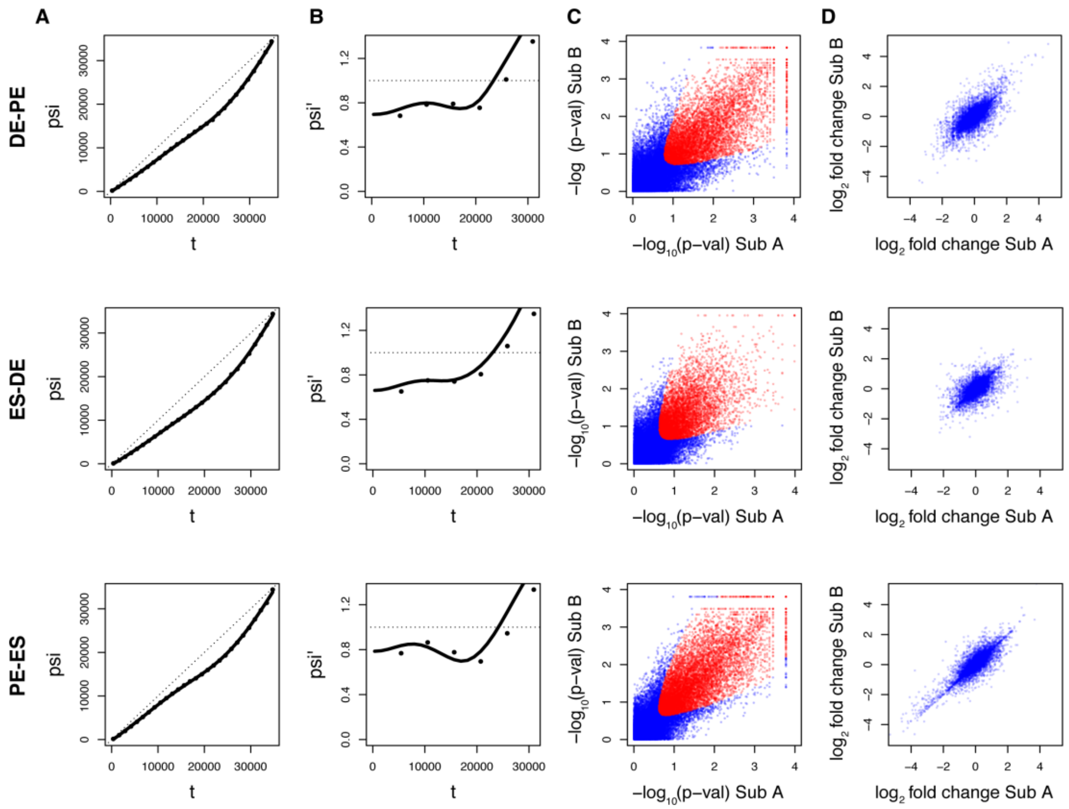

Figure S3: Pairwise comparisons of samr differential expression analysis results for subsampled data from C57BL/6. Data were subjected to 10 rounds of sampling (5 replicates) for each time course comparison point. Differential expression analysis was performed, and results were examined for consistency of p-values and fold change. Representative pairwise comparisons of (A) correspondence curves, (B) change of correspondence curves, (C) IDR decision boundaries and (D) log_2 fold changes for two independent subsamples from each time point comparison are shown.

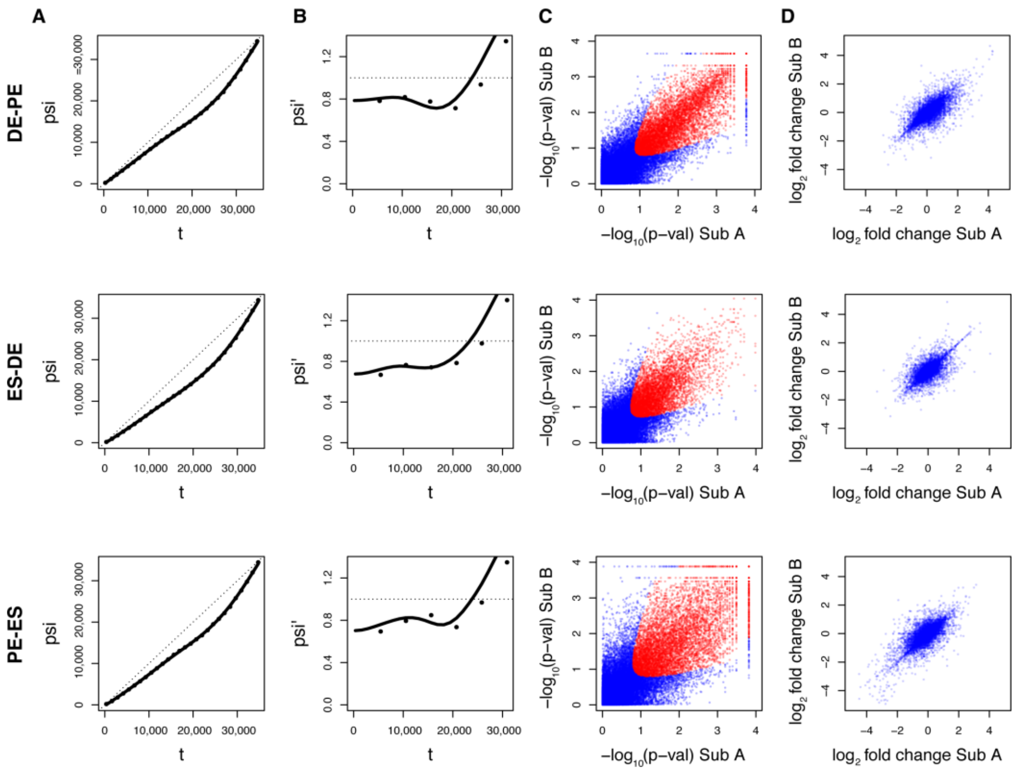

Figure S4: Pairwise comparisons of samr differential expression analysis results for subsampled data from BALB/c. Data were subjected to 10 rounds of sampling (5 replicates) for each time course comparison point. Differential expression analysis was performed, and results were examined for consistency of p-values and fold change. Representative pairwise comparisons of (A) correspondence curves, (B) change of correspondence curves, (C) IDR decision boundaries and (D) $\log_2$ fold changes for two independent subsamples from each time point comparison are shown.

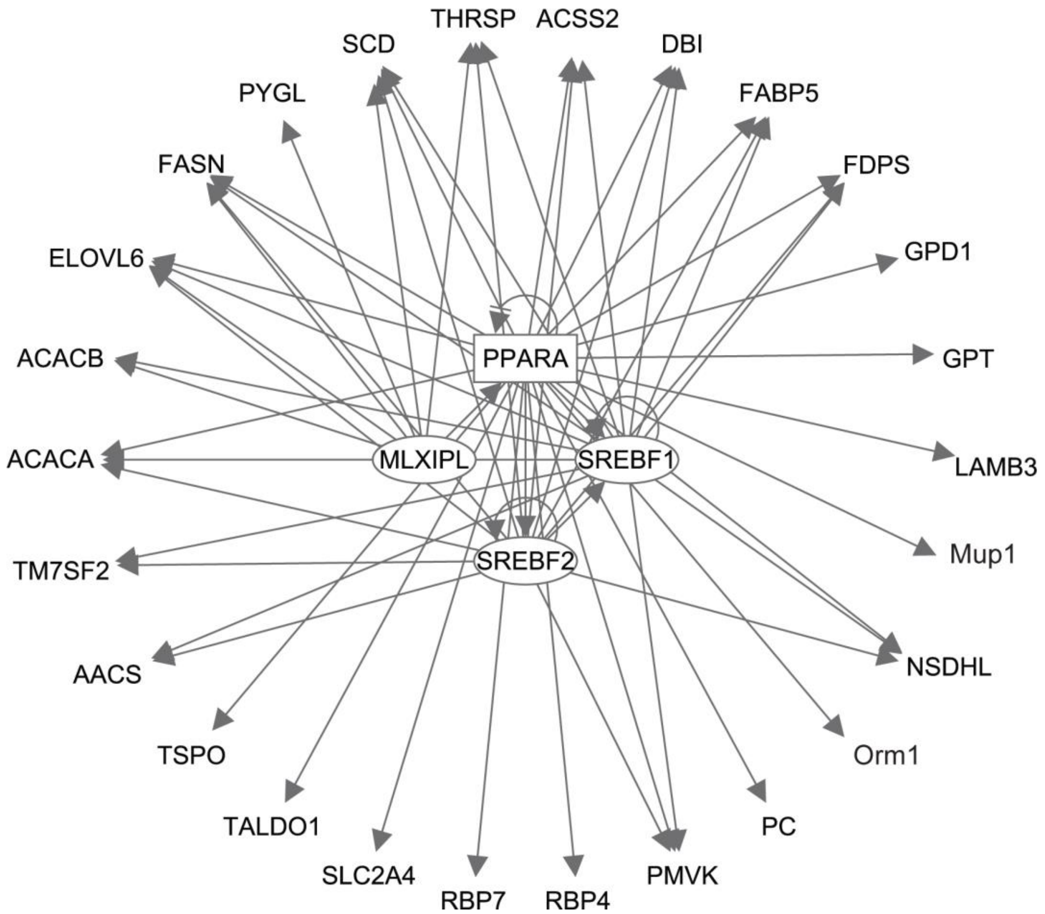

Figure S5: Glucose and lipid metabolism genes fluctuate during the estrous cycle in the BALB/c and C57BL/6 mammary glands. Upstream transcriptional regulator analyses using Ingenuity Pathway Analyses (IPA) of overlapping genes in Cluster A between the two strains revealed a significant association with PPARA, MLXIPL, SREBF1 and SREBF2 involved in metabolic processes.

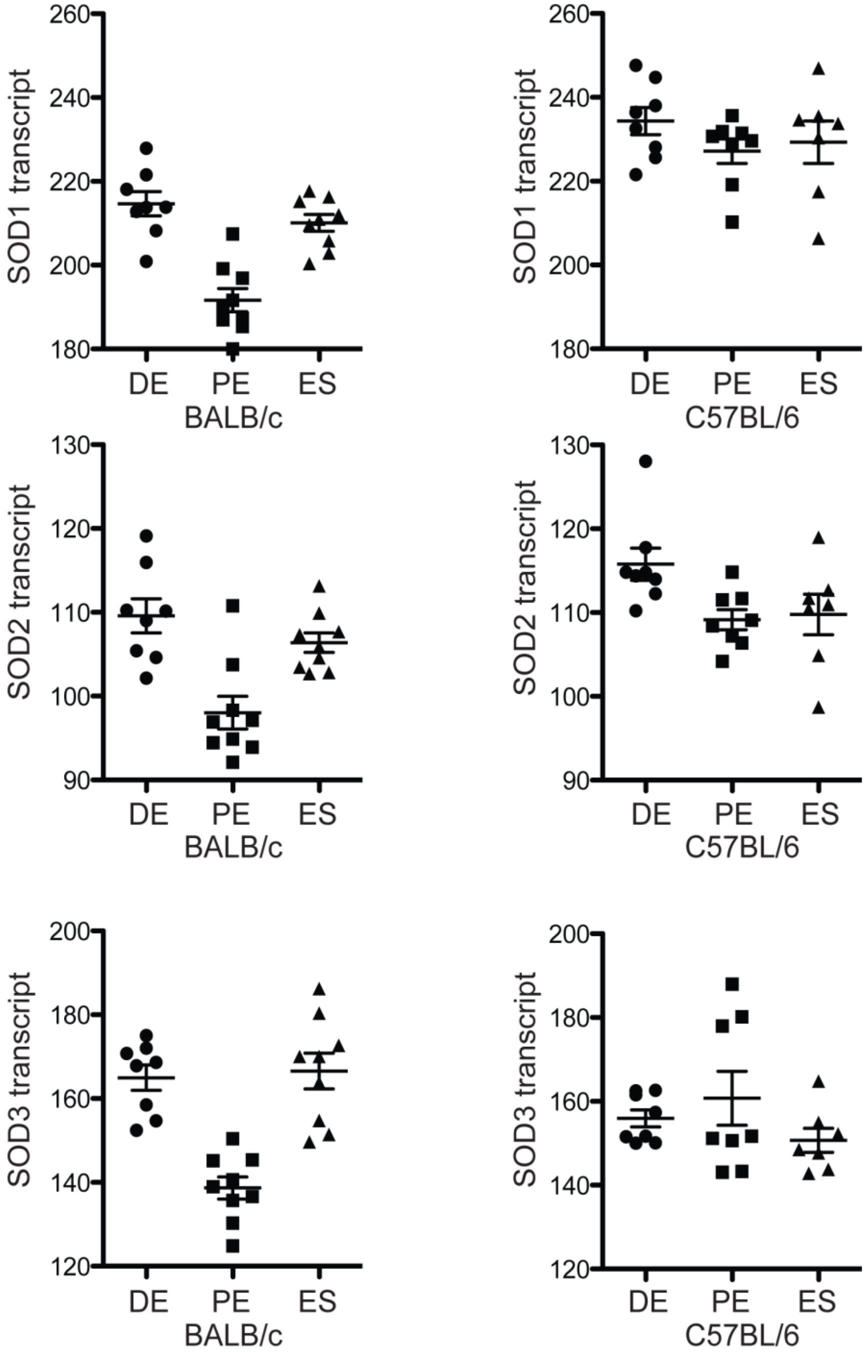

Figure S6: Significant downregulation of superoxide dismutase in mammary glands of BALB/c mice in proestrus. Transcript levels of SOD1, SOD2 and SOD3 were significantly decreased in proestrus of BALB/c mice compared to diestrus (average fold-change 1.3 and $q<0.1$) and estrus (average fold-change 1.3 and $q<0.4$), but not C57BL/6 (DE, diestrus; PE, proestrus; ES, estrus).

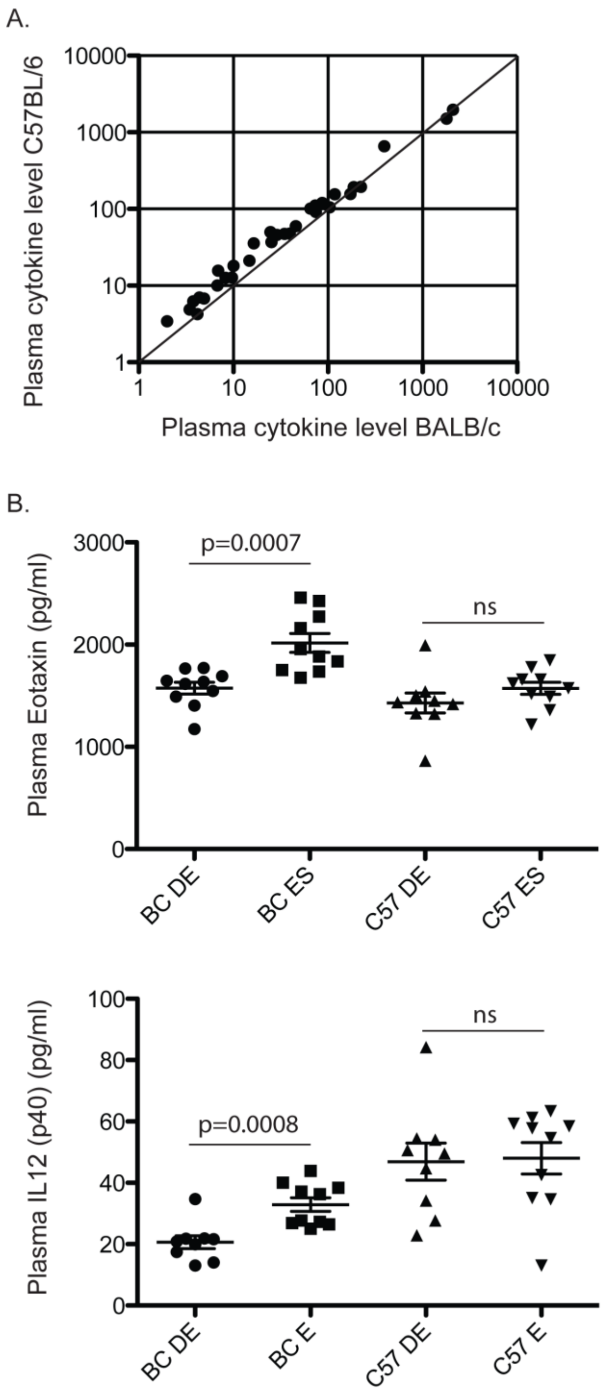

Figure S7: Plasma levels of Eotaxin and IL12-p40 fluctuate with estrous stage in BALB/c. Plasma levels of 32 cytokines were measured in BALB/c and C57BL/6 mice in diestrus and estrus. A. Plasma cytokine levels were highly correlated between the two strains (R2=0.998), and significantly higher levels were observed in C57BL/6 mice for 21/32 cytokines ($p<0.05$). B. Plasma concentrations of eotaxin (top) and IL12-p40 (bottom) were significantly increased in BALB/c mice, but not C57BL/6 mice, in mice in estrus compared to mice in diestrus. (BC, BALB/c; C57, C57BL/6; DE, diestrus; ES, estrus; ns, not significant)

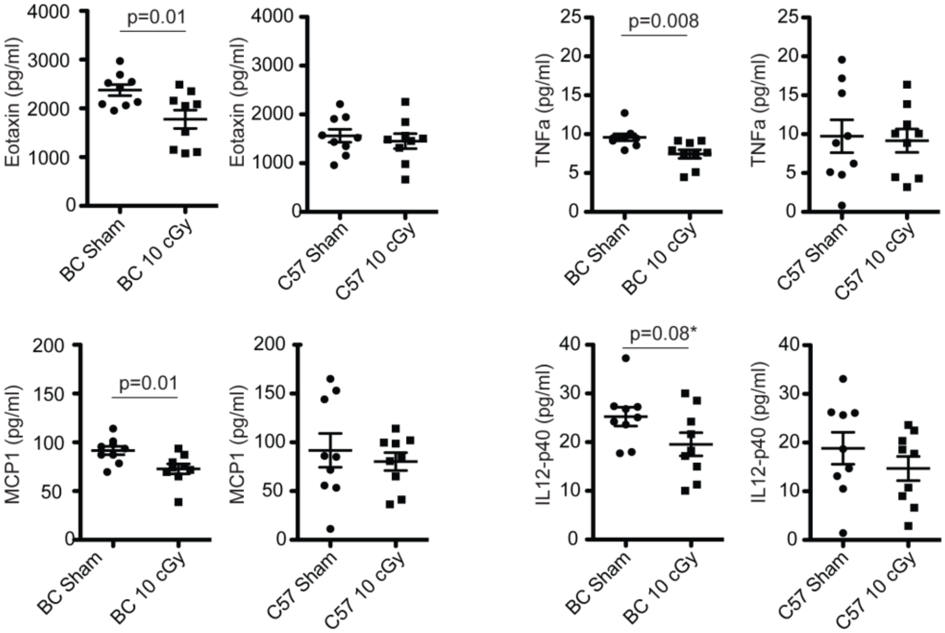

Figure S8: Low-dose radiation-induced changes in blood cytokine levels in BALB/c. Plasma cytokine concentrations of eotaxin, TNFa, MCP1 and IL12-p40 in BALB/c and C57Bl/6 mice three days after exposure to 10 cGy X-ray irradiation or sham (n=9 per strain). Significance was tested using a non-paired T-test. Asterisk indicates borderline significance. (BC, BALB/c; C57, C57BL/6)
