# Supplementary material for: An interferon signature identified by RNA-sequencing of mammary tissues varies across the estrous cycle and is predictive of metastasis-free survival
Source: Oncotarget. 2014 Jun 30;5(12):4011–25. doi: 10.18632/oncotarget.2148 (PMC4147302; doi:10.18632/oncotarget.2148)
Supplement: Supplementary file 2 [file oncotarget-05-4011-s002.pdf]

Table S1

replicate ID	strain	time	input pairs	concordant_pairs(%)		pairs_multi(%)	discordant_pairs(%)	left_mapped	left(%)	left_multi(%)	right_mapped	right(%)	right_multi(%)
HOYS.6477.3.45129.CAGATC	BALB/c	DE	34568767	22435446	64.7	4.1	0.3	24472443	70.8	4.8	23629166	68.4	4.9
HOYT.6477.6.45132.CCGTCC	BALB/c	DE	32784843	21097347	64.1	3.3	0.3	23170931	70.7	4	22454681	68.5	4.1
HOZG.6477.4.45130.GATCAG	BALB/c	DE	28512871	10485629	36.7	3.3	0.2	11505161	40.4	3.7	10998029	38.6	3.8
HOZO.6477.2.45128.TTAGGC	BALB/c	DE	45083584	29649675	65.5	4.9	0.3	32344577	71.7	5.7	31253579	69.3	5.8
HOZT.6588.1.48245.CTATAC	BALB/c	DE	46103666	29798905	64.4	3.7	0.3	33051181	71.7	4.5	31439611	68.2	4.5
HOZU.6466.2.44838.CACCGG	BALB/c	DE	48072767	30164365	62.6	3.7	0.3	32717843	68.1	4.4	32165801	66.9	4.5
HOZW.6478.1.45118.GTTTCG	BALB/c	DE	41022645	26878253	65.3	3.3	0.4	29397722	71.7	4.1	28617732	69.8	4.1
HOZX.6477.5.45131.CTTGTA	BALB/c	DE	46576212	25602835	54.8	4.1	0.3	28186771	60.5	4.8	27549646	59.1	4.9
HOZY.6466.2.44838.CACTCA	BALB/c	DE	38381114	25166226	65.4	3.6	0.3	27142067	70.7	4.4	26748134	69.7	4.4
HOZZ.6478.1.45118.GAGTGG	BALB/c	DE	28352609	18231076	64.1	3.2	0.3	20091434	70.9	3.9	19496055	68.8	4
HPAA.6477.5.45131.AGTTCC	BALB/c	DE	32741189	17777905	54.1	3.1	0.3	19611638	59.9	3.8	19174076	58.6	3.8
HPAB.6477.3.45129.ACAGTG	BALB/c	DE	46865310	29359868	62.5	3	0.3	32404149	69.1	3.8	31097866	66.4	3.8
HPAN.6478.2.45119.GGTAGC	BALB/c	ES	33433468	21863963	65.2	4	0.3	23702912	70.9	4.7	23072084	69	4.7
HPAO.6477.7.45133.GTCCGC	BALB/c	ES	41676665	28473489	68.1	3.9	0.3	30906295	74.2	4.6	30009071	72	4.7
HPAP.6478.3.45120.ATTCCT	BALB/c	ES	47879271	31485992	65.5	3.6	0.3	34415522	71.9	4.4	33220095	69.4	4.5
HPAS.6466.4.44840.CCAACA	BALB/c	ES	45885956	29031621	63	3.9	0.4	32193456	70.2	4.6	30836179	67.2	4.7
HPAT.6466.6.44842.TCATTC	BALB/c	ES	45069638	29699620	65.6	3.6	0.4	32091617	71.2	4.4	31604625	70.1	4.4
HPAU.6478.2.45119.ACTGAT	BALB/c	ES	34316978	15142771	44	3.6	0.2	16219151	47.3	4.1	15823987	46.1	4.2
HPAW.6466.5.44841.TACAGC	BALB/c	ES	44566852	28597446	63.9	4.4	0.4	31340933	70.3	5.1	30332584	68.1	5.2
HPAY.6466.3.44839.CATGGC	BALB/c	ES	44473648	30975800	69.4	4	0.4	33366156	75	4.8	32832634	73.8	4.8
HPAZ.6466.7.44843.AAAGCA	BALB/c	ES	49529694	34609264	69.6	3.4	0.4	37222597	75.2	4.2	36787269	74.3	4.3
HPBA.6588.1.48245.GACGAC	BALB/c	ES	40196689	24721926	61.4	4.1	0.2	26812157	66.7	4.7	25804775	64.2	4.7
HPBB.6466.6.44842.TCGAAG	BALB/c	ES	44418819	29205735	65.5	3.2	0.3	31599305	71.1	4	31066083	69.9	4
HPBC.6466.7.44843.TCGGCA	BALB/c	ES	41578786	28727089	68.9	3.9	0.3	30818315	74.1	4.7	30407375	73.1	4.8
NOUH.6599.6.48810.AAACAT	BALB/c	LDIR	38285061	25884351	67.3	3.5	0.5	27705190	72.4	4.3	27892245	72.9	4.3

NOUO.6599.2.48806.CAGGCG	BALB/c	LDIR	38137856	24538306	64.1	3.8	0.3	26285514	68.9	4.5	26268774	68.9	4.6
NOUP.6599.4.48808.TATAAT	BALB/c	LDIR	35230171	22779346	64.4	3.4	0.4	24128407	68.5	4.1	24606756	69.8	4.2
NOUS.6599.2.48806.CATTTT	BALB/c	LDIR	51811835	34505489	66.2	4	0.6	37014774	71.4	4.9	37065085	71.5	5
NOUX.6588.7.48251.ATGAGC	BALB/c	LDIR	48963901	34889017	71	3.9	0.3	37132243	75.8	4.7	36826801	75.2	4.8
NPNW.6588.5.48249.GTAGAG	BALB/c	LDIR	16893964	11607645	68.5	3.5	0.4	12482634	73.9	4.1	12319179	72.9	4.2
NPNX.6599.3.48807.CGGAAT	BALB/c	LDIR	38405057	25114191	65.1	4	0.5	26961855	70.2	4.8	26815448	69.8	4.8
NPNY.6599.5.48809.TCCCGA	BALB/c	LDIR	46959769	27752222	58.9	4	0.3	29472313	62.8	4.7	30315625	64.6	4.9
NPNZ.6588.5.48249.GTGAAA	BALB/c	LDIR	44156283	31318023	70.7	3.6	0.4	33459762	75.8	4.4	33114451	75	4.5
NPOA.6588.7.48251.CAAAAG	BALB/c	LDIR	38393711	26931447	69.9	3.9	0.4	28882418	75.2	4.7	28555380	74.4	4.7
NPOB.6588.3.48247.TAGCTT	BALB/c	LDIR	50574474	35316947	69.6	3.8	0.4	37720189	74.6	4.6	37462875	74.1	4.7
NPON.6588.4.48248.ATGTCA	BALB/c	PE/SHAM	41921748	30644503	72.8	4.1	0.4	32523915	77.6	4.8	32421399	77.3	4.9
NPOS.6588.2.48246.GCCAAT	BALB/c	PE/SHAM	43591147	30012150	68.6	4.6	0.3	31989917	73.4	5.3	31810506	73	5.4
NPOT.6599.3.48807.CTCAGA	BALB/c	PE/SHAM	28087769	18283885	64.9	4	0.4	19741524	70.3	4.8	19486097	69.4	4.9
NPOU.6599.1.48804.CACGAT	BALB/c	PE/SHAM	35926538	23870769	66.2	4.2	0.4	25616920	71.3	5	25656724	71.4	5
NPOW.6588.6.48250.CGTACG	BALB/c	PE/SHAM	33026607	22503481	67.9	3.3	0.4	24154479	73.1	4	23842119	72.2	4.1
NPOX.6588.3.48247.GGCTAC	BALB/c	PE/SHAM	42408066	30082026	70.6	3.9	0.4	32075524	75.6	4.7	31870760	75.2	4.8
NPOY.6588.5.48249.GTGGCC	BALB/c	PE/SHAM	62075459	43052538	69.1	3.8	0.3	46078121	74.2	4.6	45535818	73.4	4.6
NPPA.6588.2.48246.TGACCA	BALB/c	PE/SHAM	46567986	32855300	70.3	3.6	0.4	34960871	75.1	4.3	34710037	74.5	4.4
NPPB.6588.4.48248.AGTCAA	BALB/c	PE/SHAM	33731836	25041673	73.9	3.8	0.4	26460967	78.4	4.6	26431949	78.4	4.7
NPPC.6599.1.48804.CAACTA	BALB/c	PE/SHAM	41735905	27826322	66.4	4	0.4	29659138	71.1	4.7	29768446	71.3	4.8
NPPG.6599.3.48807.CTAGCT	BALB/c	PE/SHAM	40460464	26910511	66.2	3.8	0.4	28941096	71.5	4.6	28799400	71.2	4.7
NPPH.6584.2.48166.CGATGT	BALB/c	PE/SHAM	77195030	42787174	55.2	3.4	0.4	47501999	61.5	4.1	47033394	60.9	4.2
HOYU.6477.6.45132.ATGTCA	C57BL/6	DE	29413001	19278849	65.4	3.8	0.2	20941760	71.2	4.5	20199946	68.7	4.6
HOYW.6477.3.45129.GCCAAT	C57BL/6	DE	42666022	29148016	68.2	4.4	0.2	31426444	73.7	5.1	30317887	71.1	5.1
HOYX.6478.3.45120.CAACTA	C57BL/6	DE	49294912	33787035	68.4	4.5	0.2	36521749	74.1	5.3	35222892	71.5	5.4
HOYY.6477.7.45133.GTGGCC	C57BL/6	DE	34981545	24750710	70.6	4.1	0.2	26830058	76.7	5.1	25934397	74.1	5.1
HOYZ.6477.2.45128.TGACCA	C57BL/6	DE	45476137	31211976	68.5	4.7	0.2	33654894	74	5.5	32508519	71.5	5.6

HOZA.6466.4.44840.CTAGCT	C57BL/6	DE	50559699	35324214	69.7	4.7	0.2	38052180	75.3	5.5	36812281	72.8	5.5
HOZB.6478.1.45118.CGTACG	C57BL/6	DE	42811344	29241080	68.2	4.5	0.2	31396542	73.3	5.3	30535640	71.3	5.4
HOZC.6477.5.45131.AGTCAA	C57BL/6	DE	32300965	18209611	56.2	4.7	0.3	19973393	61.8	5.4	19914170	61.7	5.5
HOZH.6588.1.48245.CTCAGA	C57BL/6	DE	24157259	16201833	66.9	4.6	0.2	17937139	74.3	5.3	16897557	69.9	5.3
HOZN.6466.2.44838.CACGAT	C57BL/6	DE	43674870	30321872	69.3	4.5	0.2	32477836	74.4	5.4	31933739	73.1	5.4
HOZS.6477.2.45128.CGATGT	C57BL/6	DE	48009769	32190287	66.9	4.4	0.2	34928180	72.8	5.1	33611446	70	5.2
HPAC.6466.3.44839.CAGGCG	C57BL/6	ES	33276153	17687919	53.1	3.9	0.1	18720933	56.3	4.5	18381171	55.2	4.5
HPAG.6466.5.44841.TATAAT	C57BL/6	ES	43251636	28609588	66	3.7	0.2	31081565	71.9	4.5	30024483	69.4	4.5
HPAH.6466.7.44843.AAACAT	C57BL/6	ES	24873801	11012761	44.2	4.6	0.3	11667645	46.9	5.2	11504512	46.3	5.3
HPAX.6478.3.45120.CAAAAG	C57BL/6	ES	45532864	31252365	68.5	4.5	0.3	33835996	74.3	5.3	32632029	71.7	5.4
HPBG.6466.4.44840.CGGAAT	C57BL/6	ES	44151856	30095390	68	5	0.3	32644030	73.9	5.9	31429247	71.2	5.9
HPBH.6477.4.45130.TAGCTT	C57BL/6	ES	53834037	32673875	60.6	4.6	0.2	35791834	66.5	5.3	34195162	63.5	5.4
HPBN.6477.7.45133.GTGAAA	C57BL/6	ES	39917884	28014353	70	4.2	0.2	30364883	76.1	5	29305925	73.4	5.1
HPBO.6478.2.45119.ATGAGC	C57BL/6	ES	55213548	38779843	70.1	3.7	0.2	41553170	75.3	4.5	40337890	73.1	4.6
HPBP.6466.3.44839.CATTTT	C57BL/6	ES	35747354	26290931	73.3	4.1	0.3	28131054	78.7	4.9	27575106	77.1	4.9
HPBS.6466.6.44842.TCCCGA	C57BL/6	ES	45947225	32153778	69.8	4.7	0.2	34122379	74.3	5.4	33636944	73.2	5.5
HPBT.6477.6.45132.GTAGAG	C57BL/6	ES	5478907	2363309	43	4.4	0.3	2570994	46.9	4.8	2471263	45.1	4.9
HPBU.6466.5.44841.TAATCG	C57BL/6	ES	45365864	30421040	66.9	3.7	0.3	33093929	72.9	4.5	31952304	70.4	4.6
NOUC.6588.5.48249.GTCCGC	C57BL/6	LDIR	45065672	32862298	72.7	4.3	0.3	34805495	77.2	5.1	34359498	76.2	5.2
NOUG.6588.6.48250.GGTAGC	C57BL/6	LDIR	39233480	26788602	68.1	4.8	0.3	28200671	71.9	5.5	27936656	71.2	5.6
NOUT.6599.5.48809.TCATTC	C57BL/6	LDIR	56964082	35720598	62.6	4.2	0.2	37652635	66.1	5	38409611	67.4	5.1
NOUU.6599.5.48809.TCGGCA	C57BL/6	LDIR	37319929	23064966	61.6	4.3	0.3	24390440	65.4	5.1	24818646	66.5	5.2
NOUW.6599.4.48808.GACGAC	C57BL/6	LDIR	25142523	17505068	69.4	4.7	0.3	18293463	72.8	5.5	18598529	74	5.6
NOUY.6599.4.48808.TACAGC	C57BL/6	LDIR	44515731	30641574	68.6	3.9	0.3	32186888	72.3	4.7	32668640	73.4	4.9
NOUZ.6599.5.48809.TCGAAG	C57BL/6	LDIR	44576410	27521624	61.6	4	0.3	29130627	65.3	4.8	29688597	66.6	4.9
NPNS.6599.6.48810.AAAGCA	C57BL/6	LDIR	43224241	31281428	72.1	3.9	0.3	33037261	76.4	4.8	33138143	76.7	4.9
NPNT.6599.2.48806.CATGGC	C57BL/6	LDIR	39364429	27900900	70.7	4.8	0.3	29373601	74.6	5.6	29358028	74.6	5.7

NPNU.6588.7.48251.ACTGAT	C57BL/6	LDIR	30229548	22172312	73.1	3.6	0.3	23544786	77.9	4.4	23245741	76.9	4.4
NPOC.6599.2.48806.CCAACA	C57BL/6	LDIR	47901071	33136644	69	4	0.2	34993122	73.1	4.8	34944615	73	4.9
NPOG.6588.7.48251.ATTCCT	C57BL/6	LDIR	40986802	31243230	76	4.4	0.2	32854105	80.2	5.2	32542634	79.4	5.3
NPOH.6588.2.48246.ACAGTG	C57BL/6	PE/SHAM	52830015	38658336	73	3.7	0.3	41176768	77.9	4.6	40738173	77.1	4.6
NPOO.6584.2.48166.TTAGGC	C57BL/6	PE/SHAM	66491903	39648501	59.4	4.7	0.4	43525227	65.5	5.6	43009122	64.7	5.7
NPOP.6588.3.48247.CTTGTA	C57BL/6	PE/SHAM	48804776	36176325	73.9	4	0.3	38203228	78.3	4.9	37845151	77.5	4.9
NPOZ.6588.3.48247.GATCAG	C57BL/6	PE/SHAM	30465859	22237015	72.8	4.2	0.2	23546083	77.3	5	23310733	76.5	5.1
NPPN.6588.2.48246.CAGATC	C57BL/6	PE/SHAM	35531886	26039748	73.1	4	0.2	27549730	77.5	4.8	27311108	76.9	4.9
NPPP.6588.6.48250.GAGTGG	C57BL/6	PE/SHAM	61964343	45091873	72.6	4	0.3	47895947	77.3	4.9	47213410	76.2	5
NPPS.6588.4.48248.AGTTCC	C57BL/6	PE/SHAM	36897129	28427869	76.8	4	0.3	30082309	81.5	4.8	29852150	80.9	4.9
NPPT.6599.1.48804.CACTCA	C57BL/6	PE/SHAM	41486785	28976567	69.6	4.4	0.3	30750949	74.1	5.2	30680656	74	5.3
NPPU.6588.6.48250.GTTTCG	C57BL/6	PE/SHAM	43037093	31414347	72.8	4.2	0.3	33229319	77.2	5	32810263	76.2	5.1
NPPW.6599.1.48804.CACCGG	C57BL/6	PE/SHAM	54364059	37602751	69	4.4	0.2	39768424	73.2	5.2	39746909	73.1	5.3
NPPX.6599.3.48807.CTATAC	C57BL/6	PE/SHAM	31744595	22250844	69.9	4.4	0.3	23592625	74.3	5.1	23408540	73.7	5.2

Table S2

ensID	external_gene_id	gene_biotype	C57BL6_DE_PE_Fold_Change	C57BL6_DE_PE_qval	C57BL6_ES_DE_Fold_Change	C57BL6_ES_DE_qval	C57BL6_PE_ES_Fold_Change	C57BL6_PE_ES_qval	C57BL6_Sham_IR_Fold_Change	C57BL6_Sham_IR_qval
ENSMUSG00000000001	Gnai3	protein_coding	1.139	0	NA	NA	0.869	0.72	1.003	95.273
ENSMUSG00000000003	Pbsn	protein_coding	1	82.982	1	92.931	1	79.554	1	94.016
ENSMUSG00000000028	Cdc45	protein_coding	0.953	74.745	0.978	95.504	1.07	85.921	1.149	83.276
ENSMUSG00000000031	H19	lincRNA	0.54	8.751	1.336	82.904	1.385	37.259	1.217	69.965
ENSMUSG00000000037	Scml2	protein_coding	1.262	16.436	0.932	96.138	0.852	18.539	0.962	89.72
ENSMUSG00000000049	Apoh	protein_coding	1.025	81.178	1.062	92.474	0.919	73.428	0.916	94.174
ENSMUSG00000000056	Narf	protein_coding	1.092	8.685	0.993	97.263	0.921	18.539	1.039	82.328
ENSMUSG00000000058	Cav2	protein_coding	0.968	44.562	0.95	88.551	NA	NA	1.042	88.111
ENSMUSG00000000078	Klf6	protein_coding	1.083	39.851	0.955	95.708	0.968	64.427	1.047	87.088
ENSMUSG00000000085	Scmh1	protein_coding	1.017	58.896	1.052	96.138	0.936	49.217	0.923	83.724
ENSMUSG00000000088	Cox5a	protein_coding	0.922	26.77	1.089	82.904	0.999	79.325	1.054	83.067
ENSMUSG00000000093	Tbx2	protein_coding	0.747	8.139	0.935	91.361	1.427	21.522	1.092	75.198
ENSMUSG00000000094	Tbx4	protein_coding	2.116	36.686	0.493	64.123	NA	NA	1.317	52.324
ENSMUSG00000000103	Zfy2	protein_coding	1	84.738	1	94.597	1	81.032	1	95.832
ENSMUSG00000000120	Ngfr	protein_coding	1.703	5.016	1.254	86.34	0.468	1.061	4.021	63.708
ENSMUSG00000000125	Wnt3	protein_coding	1	75.196	1	95.504	1	85.792	1	89.892
ENSMUSG00000000126	Wnt9a	protein_coding	1.213	16.124	1.036	82.904	0.791	4.521	0.858	85.493
ENSMUSG00000000127	Fer	protein_coding	1.105	22.871	NA	NA	0.895	18.336	1.123	40.024
ENSMUSG00000000131	Xpo6	protein_coding	1.051	15.85	1.035	85.985	0.919	3.147	NA	NA
ENSMUSG00000000134	Tfe3	protein_coding	0.994	45.229	1.069	60.309	0.942	16.051	1.054	44.679
ENSMUSG00000000142	Axin2	protein_coding	0.997	71.915	1.113	72.546	0.898	29.451	0.966	91.625
ENSMUSG00000000148	Brat1	protein_coding	0.967	35.646	NA	NA	1.049	25.425	1.013	91.525
ENSMUSG00000000149	Gna12	protein_coding	1.121	8.365	0.949	88.345	0.942	13.848	0.973	96.139
ENSMUSG00000000154	Slc22a18	protein_coding	0.919	17.839	0.921	91.955	1.182	36.388	0.931	91.105
ENSMUSG00000000159	Igsf5	protein_coding	0.951	72	0.846	83.58				

1.244 80.015 0.859 85.636
ENSMUSG00000000167 Pih1d2 protein_coding 0.756 29.972
1.098 91.584 1.205 66.043 1.172 83.067
ENSMUSG00000000168 Dlat protein_coding 0.832 1.175 1.008 96.68 1.19
13.047 1.042 92.762
ENSMUSG00000000171 Sdhd protein_coding 0.98 78.299 1.04
90.454 0.983 81.388 0.969 96.358
ENSMUSG00000000182 Fgf23 protein_coding 1 81.823 1
87.282 1 85.134 1 93.584
ENSMUSG00000000183 Fgf6 protein_coding 1 62.574 1
94.102 1 87.786 1 92.675
ENSMUSG00000000184 Ccnd2 protein_coding 0.995 76.968 1.079
82.904 0.928 39.755 1.014 61.984
ENSMUSG00000000194 Gpr107 protein_coding 1.039 13.8 NA NA
0.948 21.852 0.997 83.067
ENSMUSG00000000197 Nalcn protein_coding 0.943 60.707 1.086
92.931 0.978 76.388 0.892 94.608
ENSMUSG00000000202 Btbd17 protein_coding 2.412 0.355 0.543
40.053 0.764 69.445 0.955 93.722
ENSMUSG00000000204 Slfn4 protein_coding 2.87 1.129 1.189 95.504
0.291 16.051 2.147 49.806
ENSMUSG00000000215 Ins2 protein_coding 1 73.487 NA NA
1 76.923 1 90.805
ENSMUSG00000000216 Scnn1g protein_coding 1.013 53.809
1.449 89.494 0.679 28.669 1.253 96.183
ENSMUSG00000000223 Drp2 protein_coding 0.986 52.9 0.949 88.881
1.065 26.912 1.002 90.463
ENSMUSG00000000244 Tspan32 protein_coding 1.438 0.2 0.923
85.793 0.749 11.997 1.055 93.117
ENSMUSG00000000253 Gmpr protein_coding 1.134 35.748 1.046
91.643 0.84 9.746 1.004 93.964
ENSMUSG00000000263 Glra1 protein_coding 0.603 24 1.115 96.407
1.481 45.035 0.884 94.297
ENSMUSG00000000266 Mid2 protein_coding 1.096 4.925 0.94 79.807
0.971 44.865 0.942 83.724
ENSMUSG00000000275 Trim25 protein_coding 1.067 26.77 0.97
77.944 0.961 86.552 0.94 85.493
ENSMUSG00000000276 Dgke protein_coding 0.875 4.268 0.914 81.284
1.251 7.635 0.9 83.724
ENSMUSG00000000278 Scpep1 protein_coding 1.244 0 0.965
59.946 0.834 2.563 1.042 94.752
ENSMUSG00000000282 Mnt protein_coding 0.918 24.577 0.993
94.597 1.092 36.011 0.974 95.559
ENSMUSG00000000290 Itgb2 protein_coding 1.726 0 0.796 61.883
0.728 14.206 1.093 67.691
ENSMUSG00000000295 Hddc2 protein_coding 1.003 81.465 1.011
96.013 0.987 86.602 0.973 93.261
ENSMUSG00000000296 Tpd52l1 protein_coding 0.95 84.865
0.893 87.919 1.175 76.923 1.012 93.584
ENSMUSG00000000301 Pemt protein_coding 0.86 22.237 1.185
71.617 0.981 66.647 1.049 76.831
ENSMUSG00000000303 Cdh1 protein_coding 1.083 67.163 0.889
48.565 1.041 69.825 0.823 84.29
ENSMUSG00000000305 Cdh4 protein_coding 1.459 17.336 0.727

75.052 0.941 39.245 1.122 91.269
ENSMUSG00000000308 Ckmt1 protein_coding 0.958 78.299 1.125
92.111 0.927 88.601 0.938 92.181
ENSMUSG00000000317 Bcl6b protein_coding 0.885 39.174 1.103
82.904 1.021 85.792 0.907 90.157
ENSMUSG00000000318 Clec10a protein_coding 1.278 3.536 0.808
58.64 0.966 65.688 1.034 92.903
ENSMUSG00000000320 Alox12 protein_coding 1.391 6.399 1.24
97.263 0.578 9.573 1.062 95.74
ENSMUSG00000000325 Arvcf protein_coding 1.031 80.815 0.997
96.138 0.975 87.971 0.969 92.181
ENSMUSG00000000326 Comt protein_coding 0.771 0.987 1.077 83.104
1.2 15.602 0.979 96.287
ENSMUSG00000000339 Rtca protein_coding 0.997 71.674 1.003
91.065 0.996 88.41 1.01 96.071
ENSMUSG00000000340 Dbt protein_coding 1.153 18.556 0.933
95.088 0.932 44.678 0.892 90.157
ENSMUSG00000000346 Dazap2 protein_coding 1.15 0.577 0.976
95.504 0.892 0.879 0.961 84.29
ENSMUSG00000000355 Mcts1 protein_coding 0.991 75.858 1.026
94.597 0.984 84.504 1.051 64.513
ENSMUSG00000000359 Rem1 protein_coding 1.191 18.635 0.903
90.046 0.931 23.938 1.11 31.133
ENSMUSG00000000374 Trappc10 protein_coding 0.979 49.944
1.042 81.039 0.982 86.727 1.027 90.397
ENSMUSG00000000378 Ccm2 protein_coding 1.061 21.836 0.984
91.643 0.957 15.376 0.991 95.593
ENSMUSG00000000384 Tbrg4 protein_coding 0.983 84.865 1.054
82.904 0.966 27.994 1.003 96.248
ENSMUSG00000000385 Tmprss2 protein_coding 0.774 61.334
0.977 83.876 1.317 24.791 0.629 95.92
ENSMUSG00000000386 Mx1 protein_coding 1.283 3.536 1.142 97.263
0.679 7.742 1.731 46.412
ENSMUSG00000000392 Fap protein_coding 1.345 2.651 0.916 78.51
0.809 23.938 1.058 91.872
ENSMUSG00000000394 Gcg protein_coding 1 61.725 1
91.83 1 82.161 1 92.31
ENSMUSG00000000399 Ndufa9 protein_coding 0.965 34.795
1.016 93.146 1.02 56.931 0.982 94.99
ENSMUSG00000000402 Egfl6 protein_coding 1.054 55.939 1.089
90.29 0.869 87.744 0.881 88.552
ENSMUSG00000000409 Lck protein_coding 0.91 51.089 1.15
71.98 0.954 73.874 0.836 85.636
ENSMUSG00000000416 Cttnbp2 protein_coding 0.967 84.534
1.207 71.617 0.857 16.129 0.965 96.287
ENSMUSG00000000420 Galnt1 protein_coding 1.102 1.323 0.978
85.418 0.928 9.494 1.088 46.846
ENSMUSG00000000435 Myf5 protein_coding 1 81.925 1
95.196 1 52.658 1 87.134
ENSMUSG00000000439 Mkrn2 protein_coding 1.117 7.501 0.928 96.216
0.96 15.169 0.988 96.086
ENSMUSG00000000440 Pparg protein_coding 1.078 51.297 1.048
93.707 0.88 20.009 0.977 94.001
ENSMUSG00000000441 Raf1 protein_coding 1.023 64.714 1.059

64.304 0.925 3.073 1.014 93.132
ENSMUSG00000000486 1-Sep protein_coding 0.954 76.396 0.965
96.763 1.088 74.325 0.89 83.952
ENSMUSG00000000489 Pdgfb protein_coding 0.879 19.985 0.989
96.652 1.15 44.515 0.918 83.724
ENSMUSG00000000530 Acvrl1 protein_coding 1.013 84.977
0.961 93.908 1.027 78.155 1.047 74.193
ENSMUSG00000000531 Grasp protein_coding 0.911 52.258 0.989
94.848 1.108 44.125 0.988 93.584
ENSMUSG00000000532 Acvr1b protein_coding 1.05 36.438
0.996 93.469 0.958 67.962 1.061 52.324
ENSMUSG00000000538 Tom1l2 protein_coding 0.994 81.925
0.992 95.504 1.012 72.378 0.982 88.053
ENSMUSG00000000544 Gpa33 protein_coding 0.868 79.709 1.441
89.014 0.801 74.179 0.826 92.675
ENSMUSG00000000552 Zfp385a protein_coding 0.943 10.29 0.984
84.126 1.074 62.307 1.127 43.605
ENSMUSG00000000555 Itga5 protein_coding 1.04 46.097 1.051
96.843 0.915 53.7 1.138 31.133
ENSMUSG00000000560 Gabra2 protein_coding 0.618 21.393
1.312 91.643 1.236 48.772 0.755 83.952
ENSMUSG00000000561 Wdr77 protein_coding 1.025 64.714 0.925
81.443 1.053 86.475 1.048 86.133
ENSMUSG00000000562 Adora3 protein_coding 1.17 57.655
1.133 97.155 0.755 58.271 1.095 83.067
ENSMUSG00000000563 Atp5f1 protein_coding 0.937 39.953
0.973 92.355 1.091 40.511 1.015 90.455
ENSMUSG00000000567 Sox9 protein_coding 0.796 9.508 1.038 96.407
1.21 21.201 0.958 84.29
ENSMUSG00000000568 Hnrnpd protein_coding 1.053 33.325
1.195 79.411 0.794 11.314 0.984 94.567
ENSMUSG00000000579 Dynlt1c protein_coding 0.881 27.202
1.022 82.904 1.106 79.426 1.068 96.183
ENSMUSG00000000581 C1d protein_coding 1.045 38.386 1.011
93.654 0.947 57.819 1.041 70.36
ENSMUSG00000000594 Gm2a protein_coding 1.104 16.124 1.036
96.407 0.874 9.288 0.897 83.724
ENSMUSG00000000600 Krit1 protein_coding 0.961 36.259 1.019
83.104 1.023 87.103 1.047 83.067
ENSMUSG00000000605 Clcn4-2 protein_coding 0.981 49.047
0.989 96.216 1.028 62.648 1.008 95.877
ENSMUSG00000000606 Vmn2r88 protein_coding 1 81.465 1
96.68 NA NA 1 95.593
ENSMUSG00000000617 Grm6 protein_coding 1 83.169 1
88.704 1 80.899 1 95.593
ENSMUSG00000000627 Sema4f protein_coding 1.073 32.238
1.389 62.898 0.672 1.58 0.886 93.316
ENSMUSG00000000628 Hk2 protein_coding 0.927 27.968 1.053
84.126 1.026 70.328 1.116 57.075
ENSMUSG00000000631 Myo18a protein_coding 0.884 8.139 1.053
95.431 1.073 18.797 1.067 72.616
ENSMUSG00000000632 Sez6 protein_coding 0.671 38.555 NA NA
1.333 33.787 0.887 83.724
ENSMUSG00000000673 Haao protein_coding 1.102 78.054 0.79

54.06 1.145 22.131 1.11 85.577
ENSMUSG00000000682 Cd52 protein_coding 1.306 2.567 1.151 91.584
0.662 9.456 0.961 93.264
ENSMUSG00000000686 Abhd15 protein_coding 1.032 85.77 0.977
92.914 0.993 72.378 0.988 96.184
ENSMUSG00000000690 Hoxb6 protein_coding 0.907 37.248 1.165
81.127 0.947 86.985 1.024 94.904
ENSMUSG00000000693 Loxl3 protein_coding 1.072 16.959 1
93.654 0.933 58.516 1.09 57.1
ENSMUSG00000000701 Tcl1b5 protein_coding 1 83.523 1
91.361 1 86.666 1 93.316
ENSMUSG00000000706 Btn1a1 protein_coding 0.918 63.736
1.099 82.904 0.994 69.445 0.963 93.316
ENSMUSG00000000708 Kat2b protein_coding 1.091 29.596 0.872
58.64 1.045 62.995 0.971 92.31
ENSMUSG00000000711 Rab5b protein_coding 0.977 59.358 1.014
87.185 1.012 65.8 0.967 84.19
ENSMUSG00000000730 Dnmt3l protein_coding 0.469 7.58 1.64
80.277 1.297 58.616 1.839 67.691
ENSMUSG00000000731 Aire protein_coding 1.21 49.868 0.813
83.321 1.019 85.464 0.978 96.287
ENSMUSG00000000732 Icosl protein_coding 1.082 49.202 1.071
82.904 0.86 17.258 1.208 35.053
ENSMUSG00000000738 Spg7 protein_coding 0.923 1.422 1.016 93.899
1.068 7.482 1.026 70.539
ENSMUSG00000000739 Sult5a1 protein_coding 1.622 3.393 0.618
35.072 0.994 79.524 0.958 95.676
ENSMUSG00000000740 Rpl13 protein_coding 0.986 73.292 1.018
92.545 0.999 55.512 1.022 92.748
ENSMUSG00000000743 Chmp1a protein_coding 0.974 42.722
0.953 73.166 1.08 17.662 1.041 83.067
ENSMUSG00000000751 Rpa1 protein_coding 1.017 40.333 0.965
87.998 1.017 77.294 1.143 0
ENSMUSG00000000753 Serpinf1 protein_coding 1.032 60.168 NA
NA 0.947 80.163 1.043 82.091
ENSMUSG00000000759 Tubgcp3 protein_coding 0.948 17.898
1.007 88.345 1.047 21.201 0.974 84.19
ENSMUSG00000000776 Polr3d protein_coding 0.955 74.8 1.082
90.29 0.969 66.753 1.096 78.546
ENSMUSG00000000782 Tcf7 protein_coding 1.186 56.311 0.943
95.708 0.896 67.648 0.934 95.593
ENSMUSG00000000787 Ddx3x protein_coding 1.078 9.983 0.946 79.475
0.982 53.224 1.009 96.183
ENSMUSG00000000791 Il12rb1 protein_coding 0.844 71.674
1.589 72.546 0.746 24.163 1.116 87.705
ENSMUSG00000000792 Slc5a5 protein_coding 1.109 81.823
1.262 72.642 0.712 49.453 0.958 91.872
ENSMUSG00000000794 Kcnn3 protein_coding 0.996 82.092 0.989
96.407 1.018 73.531 1.2 60.968
ENSMUSG00000000804 Usp32 protein_coding 1.082 15.662 0.947
55.71 NA NA 0.921 83.724
ENSMUSG00000000805 Car4 protein_coding 0.856 27 0.836 82.073
1.395 15.688 0.939 86.699
ENSMUSG00000000811 Txnrd3 protein_coding 1.034 72 0.98

95.809 0.988 46.956 0.975 96.086
ENSMUSG00000000817 Fasl protein_coding 1.22 79.277 0.783
92.404 1.045 80.381 1.562 92.381
ENSMUSG00000000823 Znf512b protein_coding 0.891 1.031 0.952
97.155 1.182 7.964 1.007 91.335
ENSMUSG00000000826 Dnajc5 protein_coding 1.053 16.124
1.014 96.948 0.933 8.634 1.01 71.305
ENSMUSG00000000827 Tpd52l2 protein_coding 0.97 39.712
1.043 85.756 0.99 85.921 1.154 31.133
ENSMUSG00000000838 Fmr1 protein_coding 1.043 49.517 0.986
94.848 0.969 72.464 1.124 84.246
ENSMUSG00000000861 Bcl11a protein_coding 1.121 81.76 0.953
96.68 0.937 60.614 0.788 83.724
ENSMUSG00000000869 Il4 protein_coding 1.275 57.949 0.736 75.4
1.063 75.809 1.091 94.19
ENSMUSG00000000876 Pxmp4 protein_coding 0.924 12.084 NA NA
1.087 43.481 1.009 91.078
ENSMUSG00000000881 Dlg3 protein_coding 0.92 39.712 1.028
93.908 1.055 31.317 1.039 91.525
ENSMUSG00000000884 Gnb1l protein_coding 1.058 75.761 1.023
96.843 0.921 88.693 0.979 79.268
ENSMUSG00000000889 Dbh protein_coding 1 64.589 1
96.68 1 84.504 1 85.629
ENSMUSG00000000901 Mmp11 protein_coding 0.924 85.355 0.809
86.861 1.339 67.163 0.883 83.724
ENSMUSG00000000902 Smarcb1 protein_coding 0.963 44.562
1.048 79.864 0.99 55.9 1.048 57.075
ENSMUSG00000000903 Vpreb3 protein_coding 0.563 74.299 NA
NA 1.633 57.907 0.971 96.183
ENSMUSG00000000915 Hip1r protein_coding 0.89 41.021 0.975
94.96 1.151 41.331 1.171 76.831
ENSMUSG00000000916 Nsun5 protein_coding 1.009 84.564 0.987
93.146 0.999 65.902 0.954 86.699
ENSMUSG00000000934 Top1mt protein_coding 0.808 3.101 1.027
92.545 1.207 6.069 1.023 90.177
ENSMUSG00000000938 Hoxa10 protein_coding 1.13 19.893
0.985 92.793 0.895 19.449 0.893 84.29
ENSMUSG00000000942 Hoxa4 protein_coding 0.806 17.839 1.087
78.705 1.137 86.985 1.004 94.904
ENSMUSG00000000948 Snrpn protein_coding 0.868 2.277 0.967 94.48
1.188 7.024 1.094 60.968
ENSMUSG00000000957 Mmp14 protein_coding 1.373 7.397 0.784 61.883
0.93 27.86 0.835 90.805
ENSMUSG00000000958 Slc7a7 protein_coding 1.249 0 NA NA
0.817 1.061 1.025 86.445
ENSMUSG00000000959 Oxa1l protein_coding 1.009 81.24 0.989 93.765
1.003 87.274 1.008 95.877
ENSMUSG00000000976 Heatr6 protein_coding 1.014 61.725
1.047 92.253 0.944 52.087 0.995 94.904
ENSMUSG00000000982 Ccl3 protein_coding 1.058 85.857 1.104
93.654 0.857 85.352 0.875 93.261
ENSMUSG00000000983 Wfdc18 protein_coding 0.957 59.028
0.817 75.345 1.281 22.131 0.923 95.74
ENSMUSG00000001014 Icam4 protein_coding 1.222 68.485 1.175

97.049 0.697 59.922 0.356 83.724
ENSMUSG00000001016 Ilf2 protein_coding 0.932 17.062 NA NA
1.073 23.668 1.027 49.514
ENSMUSG00000001017 Chtop protein_coding 0.94 14.075 1.029
83.104 1.035 22.994 0.995 92.691
ENSMUSG00000001018 Snapin protein_coding 0.995 72.139
1.009 91.532 0.999 71.232 0.971 94.904
ENSMUSG00000001020 S100a4 protein_coding 1.643 0 0.826
75.493 0.737 3.969 1.277 44.679
ENSMUSG00000001023 S100a5 protein_coding 1 71.516 1
88.704 1 87.274 1 94.654
ENSMUSG00000001025 S100a6 protein_coding 1.327 0.999 0.981
95.301 0.768 5.336 1.002 92.903
ENSMUSG00000001027 Scn4a protein_coding 7.081 5.481 1.097 84.126
0.129 0.72 0.909 95.92
ENSMUSG00000001029 Icam2 protein_coding 0.937 79.709 1.176
75.21 0.907 24.918 0.906 83.724
ENSMUSG00000001034 Mapk7 protein_coding 1.024 81.24 1 90.116
0.976 56.365 0.999 96.183
ENSMUSG00000001036 Epn2 protein_coding 0.992 76.968 0.951
92.137 1.059 60.104 0.972 94.904
ENSMUSG00000001039 B9d1 protein_coding 0.897 36.945 1.038
96.335 1.074 75.809 1.046 80.573
ENSMUSG00000001052 Sec24b protein_coding 1.008 68.727
1.035 83.104 0.959 23.938 1.078 92.018
ENSMUSG00000001053 N4bp3 protein_coding 0.748 2.822 1.045 92.561
1.278 10.819 0.996 87.992
ENSMUSG00000001054 Rmnd5b protein_coding 0.955 47.266
1.041 82.76 1.003 87.898 1.087 55.841
ENSMUSG00000001056 Nhp2 protein_coding 0.957 39.174 0.912
69.175 1.145 13.174 1.051 79.268
ENSMUSG00000001062 Vps9d1 protein_coding 1.034 71.763
0.953 96.216 1.016 61.743 1.003 87.088
ENSMUSG00000001065 Zfp276 protein_coding 0.946 52.9 1.031
82.904 1.021 63.919 0.998 92.181
ENSMUSG00000001076 C1ql4 protein_coding 1 62.158 1
95.431 1 80.899 1 94.608
ENSMUSG00000001082 Mfsd10 protein_coding 1.034 45.096
1.039 87.609 0.93 17.381 0.992 92.31
ENSMUSG00000001089 Luzp1 protein_coding 0.993 61.49 1.019 95.504
0.99 83.607 1 93.132
ENSMUSG00000001095 Slc13a2 protein_coding 0.592 19.023
1.342 85.985 1.252 83.925 0.856 86.699
ENSMUSG00000001098 Kctd10 protein_coding 1.016 30.984
0.944 84.017 1.041 69.134 1.027 85.786
ENSMUSG00000001100 Poldip2 protein_coding 0.936 15.76 1.014
82.904 1.055 87.274 1.033 95.35
ENSMUSG00000001105 Ift20 protein_coding 1.04 33.325 1.059
79.411 0.91 2.144 0.995 92.903
ENSMUSG00000001119 Col6a1 protein_coding 1.102 16.539
0.943 91.275 0.962 55.701 1.053 58.333
ENSMUSG00000001120 Pcbp3 protein_coding 1.066 14.194 0.986
86.861 0.951 54.037 1.108 83.581
ENSMUSG00000001123 Lgals9 protein_coding 1.081 5.481 0.999

96.216 0.926 15.904 1.383 43.377
ENSMUSG00000001127 Araf protein_coding 0.941 10.739 0.983
91.361 1.083 11.782 0.981 93.964
ENSMUSG00000001128 Cfp protein_coding 1.668 0.2 0.725 33.183
0.824 40.19 1.12 90.125
ENSMUSG00000001131 Timp1 protein_coding 1.672 9.695 0.97 96.407
0.617 27.675 1.776 33.269
ENSMUSG00000001134 Uxt protein_coding 0.891 7.887 0.987 95.35
1.137 18.208 1.109 85.786
ENSMUSG00000001138 Cnnm3 protein_coding 0.926 49.368 1
94.367 1.077 60.5 0.988 91.625
ENSMUSG00000001143 Lman2l protein_coding 0.907 12.309
1.046 72.642 1.056 35.894 0.956 85.493
ENSMUSG00000001150 Mcm3ap protein_coding 0.951 11.128
0.948 82.671 1.108 8.209 0.986 87.735
ENSMUSG00000001151 Pcnt protein_coding 0.999 68.846 0.947
85.671 1.054 44.787 0.985 96.071
ENSMUSG00000001156 Mxd1 protein_coding 1.029 79.165 1.057
82.219 0.917 39.528 0.912 86.699
ENSMUSG00000001157 Gmcl1 protein_coding 1.04 30.984 1.015
96.847 0.946 30.024 0.961 84.29
ENSMUSG00000001158 Snrnp27 protein_coding 0.906 10.143
1.067 84.126 1.032 51.977 1.03 83.067
ENSMUSG00000001166 Oas1c protein_coding 1.071 47.174 0.906
96.013 1.032 78.045 1.017 94.608
ENSMUSG00000001168 Oas1h protein_coding 0.52 58.802 2.333
84.832 0.824 83.158 1.101 96.248
ENSMUSG00000001173 Ocrl protein_coding 1.12 1.967 0.95 64.624
0.941 25.625 1.048 31.133
ENSMUSG00000001175 Calm1 protein_coding 0.906 28.998 1.102
72.642 1.004 60.233 1.218 44.882
ENSMUSG00000001211 Agpat3 protein_coding 1.026 74.958
0.991 91.643 0.981 38.434 0.938 83.724
ENSMUSG00000001225 Slc26a3 protein_coding 1 63.126 1
96.847 1 81.857 1 96.358
ENSMUSG00000001227 Sema6b protein_coding 1.133 41.454
1.018 94.48 0.869 88.203 0.918 95.273
ENSMUSG00000001228 Uhrf1 protein_coding 1.376 55.124 0.892
90.138 0.813 83.607 1.639 46.412
ENSMUSG00000001229 Dpp9 protein_coding 0.96 40.568 1.076
67.843 0.966 46.575 1.064 44.213
ENSMUSG00000001240 Ramp2 protein_coding 1.064 71.516 0.921
91.83 NA NA 0.955 86.301
ENSMUSG00000001247 Lsr protein_coding 0.945 73.292 0.91
74.851 1.163 45.035 0.972 93.264
ENSMUSG00000001248 Gramd1a protein_coding 0.887 4.874 1.015
83.104 1.11 26.179 1.01 87.134
ENSMUSG00000001249 Hpn protein_coding 0.873 51.089 0.957
95.211 1.198 60.104 1.006 94.654
ENSMUSG00000001260 Gabrg1 protein_coding 1 81.657 1
95.211 1 86.475 1 96.071
ENSMUSG00000001270 Ckb protein_coding 1.206 5.527 0.976 96.763
0.851 1.061 0.959 87.218
ENSMUSG00000001280 Sp1 protein_coding 1.012 74.745 0.991

95.809	0.991	66.425	0.946	83.724
ENSMUSG00000001281	Itgb7	protein_coding	0.976	85.857	1.092
95.431	0.94	70.214	0.995	88.245
ENSMUSG00000001285	Myg1	protein_coding	1.01	77.763	0.969
85.95	1.018	57.907	0.982	88.657
ENSMUSG00000001288	Rarg	protein_coding	0.933	66.652	1.099
80.775	0.975	75.967	0.996	94.904
ENSMUSG00000001289	Pfdn5	protein_coding	1.044	67.374	0.852
11.228	1.127	25.808	1.02	90.397
ENSMUSG00000001300	Efnb2	protein_coding	0.892	53.138	1.141
79.864	0.982	80.5	0.954	86.445
ENSMUSG00000001305	Rrp15	protein_coding	1.011	77.763	1.011
90.138	0.975	70.328	1.091	40.894
ENSMUSG00000001313	Rnd2	protein_coding	0.999	83.523	1.02
96.407	0.984	74.853	0.96	92.561
ENSMUSG00000001323	Srr	protein_coding	0.918	30.136	1.073
75.62	1.019	79.776	1.034	83.067
ENSMUSG00000001333	Sync	protein_coding	1.292	20.296	1.218
60.309	0.637	0.72	0.947	94.825
ENSMUSG00000001334	Fndc5	protein_coding	1.289	10.595	1.35
86.34	0.572	1.342	1.278	65.175
ENSMUSG00000001348	Acp5	protein_coding	0.938	16.183	1.085
60.309	0.983	43.426	0.961	83.724
ENSMUSG00000001349	Cnn1	protein_coding	0.911	19.595	1.2
60.309	0.912	50.523	1.05	87.088
ENSMUSG00000001366	Fbxo9	protein_coding	0.954	39.174	1.088
71.617	0.959	51.715	1.001	93.264
ENSMUSG00000001376	Ccdc132	protein_coding	0.953	81.465
0.989	95.088	1.06	64.305	1.046	71.305
ENSMUSG00000001379	Apbb3	protein_coding	0.85	15.06	0.946	83.733
1.247	13.082	1.034	93.964
ENSMUSG00000001380	Hars	protein_coding	0.958	24.748	0.99
94.48	1.055	29.302	1.17	31.133
ENSMUSG00000001383	Zmat2	protein_coding	0.977	51.089	1
95.708	1.023	56.125	1.031	56.339
ENSMUSG00000001403	Ube2c	protein_coding	5.276	7.397	1.153	86.277
0.165	0.72	0.978	96.086
ENSMUSG00000001415	Smg5	protein_coding	0.946	19.023	1.101
71.617	0.958	67.339	0.989	94.654
ENSMUSG00000001416	Cct3	protein_coding	0.818	10.903	1.1
96.407	1.109	28.997	1.218	31.133
ENSMUSG00000001417	Rhbg	protein_coding	1.707	2.569	0.866	94.848
0.679	3.943	0.572	83.724
ENSMUSG00000001418	0610031J06Rik	protein_coding	1.001	84.534
1.009	83.104	0.991	68.82	1.012	96.086
ENSMUSG00000001419	Mef2d	protein_coding	0.949	39.712	1.073
75.21	0.978	73.874	0.969	85.493
ENSMUSG00000001420	Tmem79	protein_coding	0.692	1.488	1.072
92.931	1.348	8.007	0.977	87.735
ENSMUSG00000001424	Snd1	protein_coding	0.84	1.017	0.996	95.735
1.195	5.864	1.172	0
ENSMUSG00000001435	Col18a1	protein_coding	0.949	48.036	0.97
91.361	1.082	56.125	1.383	40.894
ENSMUSG00000001436	Slc19a1	protein_coding	0.843	1.175	1.132

81.54 1.051 43.586 0.903 83.724
ENSMUSG00000001440 Kpnb1 protein_coding 1.02 74.745 0.989
95.708 0.989 70.214 1.066 43.377
ENSMUSG00000001441 Npepps protein_coding 1.031 62.689
0.939 71.98 1.031 66.647 1.009 80.497
ENSMUSG00000001444 Tbx21 protein_coding 0.773 45.502 1.006
94.597 1.284 39.925 1.013 93.584
ENSMUSG00000001445 Mrpl10 protein_coding 0.984 54.176
1.006 95.301 1.01 77.17 1.022 82.091
ENSMUSG00000001467 Cyp51 protein_coding 0.901 59.358 0.956
91.897 1.16 60.789 1.286 46.412
ENSMUSG00000001472 Tcf25 protein_coding 0.979 36.945 0.976
95.35 1.049 49.898 1.02 94.904
ENSMUSG00000001473 Tubb6 protein_coding 1.133 11.459 1.17
82.904 0.755 6.858 1.36 31.133
ENSMUSG00000001482 Def8 protein_coding 0.973 85.857 1.026
96.763 1.004 85.58 1.061 59.394
ENSMUSG00000001493 Meox1 protein_coding 0.735 2.009 0.965 96.68
1.411 7.365 1.085 58.721
ENSMUSG00000001496 Nkx2-1 protein_coding 1 85.832 1
95.35 1 79.554 1 94.654
ENSMUSG00000001497 Pax9 protein_coding 0.793 86.843 1.183
94.597 1.068 84.626 0.851 93.734
ENSMUSG00000001504 Irx2 protein_coding 1.167 32.676 0.837
68.115 1.021 84.229 0.903 84.19
ENSMUSG00000001506 Col1a1 protein_coding 1.017 71.763 0.95
91.897 1.037 81.032 1.277 31.133
ENSMUSG00000001507 Itga3 protein_coding 0.83 12.309 1.052
96.68 1.149 23.334 1.002 95.273
ENSMUSG00000001508 Sgca protein_coding 2.915 5.481 1.351 95.088
0.255 0.879 0.993 95.35
ENSMUSG00000001510 Dlx3 protein_coding 1.659 25.944 1.046
94.102 0.577 45.093 1.267 40.024
ENSMUSG00000001517 Foxm1 protein_coding 1.202 32.058 0.949
86.538 0.877 71.734 1.648 63.114
ENSMUSG00000001518 Itfg2 protein_coding 0.92 7.916 0.993 96.843
1.091 43.842 1.008 83.067
ENSMUSG00000001520 Nrip2 protein_coding 1.153 35.024 1.081
89.494 0.804 2.563 0.873 84.29
ENSMUSG00000001521 Tulp3 protein_coding 1.043 51.189 1.076
79.261 0.889 2.453 1.039 83.067
ENSMUSG00000001524 Gtf2h4 protein_coding 0.973 64.589
1.007 95.431 1.017 88.274 1.014 91.985
ENSMUSG00000001525 Tubb5 protein_coding 0.982 83.726 1.12
82.904 0.909 38.534 1.236 31.133
ENSMUSG00000001542 Ell2 protein_coding 1.122 41.177 0.999
93.765 0.89 42.895 1.006 83.067
ENSMUSG00000001552 Jup protein_coding 0.891 5.686 0.969 85.418
1.161 9.334 0.948 87.331
ENSMUSG00000001555 Fkbp10 protein_coding 1.118 10.595
1.089 93.146 0.821 19.552 1.293 31.133
ENSMUSG00000001558 Klhl10 protein_coding 1.309 51.297
0.722 85.116 1.054 81.032 1.082 91.928
ENSMUSG00000001566 Mnx1 protein_coding 1 73.487 1

96.847 1 87.786 1 95.593
ENSMUSG00000001569 Nom1 protein_coding 1.053 32.873 0.918
61.883 1.034 79.554 1.005 91.335
ENSMUSG00000001576 Ergic1 protein_coding 0.905 5.084 1.133
60.309 0.972 63.504 1.047 87.088
ENSMUSG00000001583 Tnk1 protein_coding 0.923 50.519 0.951
90.378 1.143 34.403 0.933 91.625
ENSMUSG00000001588 Acap1 protein_coding 0.865 34.954 1.112
87.282 1.04 57.907 0.954 92.31
ENSMUSG00000001604 Tcea3 protein_coding 1.434 32.411 0.84
85.671 0.83 15.904 0.825 94.654
ENSMUSG00000001622 Csn3 protein_coding 0.861 36.823 0.917
77.944 1.267 19.106 1.306 80.77
ENSMUSG00000001627 Ifrd1 protein_coding 0.933 57.014 0.833
28.822 1.287 5.864 0.975 90.854
ENSMUSG00000001630 Stk38l protein_coding 1.049 33.099
1.035 86.277 0.922 8.626 0.958 87.735
ENSMUSG00000001632 Brpf1 protein_coding 1.001 75.567 1.01
95.735 0.99 88.693 0.979 93.685
ENSMUSG00000001642 Akr1b3 protein_coding 0.876 3.009 1.006
82.904 1.136 52.8 1.085 60.466
ENSMUSG00000001655 Hoxc13 protein_coding 1 86.843 1
89.713 1 69.825 1 92.381
ENSMUSG00000001656 Hoxc11 protein_coding 0.972 74.299
1.121 96.68 0.918 83.275 1.212 81.56
ENSMUSG00000001657 Hoxc8 protein_coding 0.916 17.705 1.1
60.309 0.992 59.383 1.09 92.381
ENSMUSG00000001661 Hoxc6 protein_coding 0.817 8.302 1.259 60.309
0.974 55.9 0.992 91.372
ENSMUSG00000001663 Gstt1 protein_coding 0.933 80.608 1.187
79.055 0.9 48.133 1.136 64.905
ENSMUSG00000001665 Gstt3 protein_coding 1.209 37.702 0.845
67.513 0.981 73.309 0.759 87.735
ENSMUSG00000001666 Ddt protein_coding 0.974 78.299 1.013
93.863 1.012 87.898 1.011 94.384
ENSMUSG00000001672 Marveld3 protein_coding 0.983 70.597
0.883 65.819 1.153 36.578 0.892 83.724
ENSMUSG00000001674 Ddx18 protein_coding 1.041 34.439 0.966
79.475 0.992 80.5 1.053 46.412
ENSMUSG00000001687 Ubl3 protein_coding 1.007 84.564 1.068
72.546 0.931 17.258 0.969 86.254
ENSMUSG00000001700 Gramd3 protein_coding 1.114 16.124
1.025 96.138 0.874 13.551 0.993 95.593
ENSMUSG00000001707 Eef1e1 protein_coding 0.989 83.263
1.195 88.136 0.843 70.214 1.02 86.133
ENSMUSG00000001729 Akt1 protein_coding 1.056 35.371 0.973
96.843 0.968 14.079 1.01 90.463
ENSMUSG00000001739 Cldn15 protein_coding 0.765 1.031 1.079
92.561 1.207 10.819 1.047 93.964
ENSMUSG00000001741 Il16 protein_coding 1.027 38.386 1.081
95.211 0.897 34.21 1.037 95.273
ENSMUSG00000001750 Tcirg1 protein_coding 0.991 84.397
1.021 97.288 0.99 87.173 1.054 92.018
ENSMUSG00000001751 Naglu protein_coding 0.989 82.982 1.056

82.904	0.956	29.765	1.039	88.512
ENSMUSG00000001755	Coasy	protein_coding	0.891	11.519	1.048
92.561	1.073	48.451	0.96	88.505
ENSMUSG00000001761	Smo	protein_coding	1.028	45.61	1.055	97.155
0.919	29.451	0.961	86.991
ENSMUSG00000001763	Tspan33	protein_coding	0.903	34.572
1.123	94.367	0.987	57.286	0.732	86.991
ENSMUSG00000001767	Crnkl1	protein_coding	1.018	68.16	1.004
95.211	0.982	53.224	1.03	74.193
ENSMUSG00000001768	Rin2	protein_coding	1.026	72.655	0.95
74.483	1.026	78.864	0.969	85.493
ENSMUSG00000001773	Folh1	protein_coding	1.667	12.549	0.682
53.434	0.878	68.82	0.907	94.153
ENSMUSG00000001774	Chordc1	protein_coding	1.023	79.433
1.094	96.948	0.892	86.258	1.055	67.169
ENSMUSG00000001783	Rtcb	protein_coding	1.022	86.474	0.906
41.268	1.078	14.597	1.096	0
ENSMUSG00000001785	Pwp1	protein_coding	1.054	18.388	0.999
87.041	0.944	48.851	1.07	76.831
ENSMUSG00000001786	Fbxo7	protein_coding	1.153	1.828	0.891	70.864
0.974	37.906	0.982	96.086
ENSMUSG00000001794	Capns1	protein_coding	0.966	16.325
1.086	85.985	0.954	79.325	0.976	93.132
ENSMUSG00000001802	Lrp3	protein_coding	0.797	0.987	0.984	95.708
1.275	5.864	0.956	83.724
ENSMUSG00000001804	Dsg4	protein_coding	1	85.728	1
83.733	1	55.352	1	93.612
ENSMUSG00000001815	Evx2	protein_coding	1	86.57	1	95.504
1	78.713	1	96.071
ENSMUSG00000001819	Hoxd13	protein_coding	1	73.734	1
90.378	1	88.161	1	95.74
ENSMUSG00000001823	Hoxd12	protein_coding	1	72.139	1
93.654	1	83.397	1	92.748
ENSMUSG00000001827	Folr1	protein_coding	0.946	75.567	0.889
84.594	1.191	48.451	1.247	59.464
ENSMUSG00000001829	Clpb	protein_coding	0.959	40.786	0.96
77.944	1.085	25.425	1.023	92.561
ENSMUSG00000001833	7-Sep	protein_coding	1.086	21.146	0.936
91.897	0.984	55.214	1.123	35.053
ENSMUSG00000001844	Zdhhc4	protein_coding	0.915	12.309
1.056	81.039	1.036	50.851	1.018	95.559
ENSMUSG00000001847	Rac1	protein_coding	1.061	0.2	0.985	83.58
0.959	12.41	1.018	83.067
ENSMUSG00000001855	Nup214	protein_coding	1.022	29.536	NA
NA	0.966	55.701	NA	NA
ENSMUSG00000001864	Aif1l	protein_coding	0.879	25.111	NA	NA
1.148	28.603	0.874	90.611
ENSMUSG00000001865	Cpa3	protein_coding	1.601	0	0.967	81.134
0.647	6.858	1.137	94.752
ENSMUSG00000001870	Ltbp1	protein_coding	1.129	0	1.024	86.604
0.862	12.948	1.076	96.192
ENSMUSG00000001891	Ugp2	protein_coding	0.966	49.621	1.014
92.931	1.015	84.724	1.099	46.846
ENSMUSG00000001909	Trmt1	protein_coding	0.953	43.167	0.919

30.872	1.138	6.202	0.994	91.625
ENSMUSG00000001910	Nacc1	protein_coding	0.951	8.139	1.043	81.039
1.007	53.449	1.052	32.045
ENSMUSG00000001911	Nfix	protein_coding	1.123	5.016	1.099	66.01	0.81
0.72	0.947	83.724
ENSMUSG00000001918	Slc1a5	protein_coding	0.723	0.987	1.087
81.54	1.269	10.76	0.996	95.593
ENSMUSG00000001924	Uba1	protein_coding	0.913	1.017	1.054	71.617
1.035	49.384	1.085	49.239
ENSMUSG00000001930	Vwf	protein_coding	1.262	0	0.818	0
0.969	49.453	0.968	90.157
ENSMUSG00000001942	Siae	protein_coding	1.224	0.984	0.956	63.773
0.851	14.079	0.934	84.29
ENSMUSG00000001943	Vsig2	protein_coding	0.785	1.031	0.956	88.705
1.335	5.864	1.092	72.369
ENSMUSG00000001946	Esam	protein_coding	0.9	3.641	1.01	93.899
1.102	24.791	0.981	92.31
ENSMUSG00000001948	Spa17	protein_coding	0.827	28.514	1.248
68.96	0.968	73.598	0.964	95.877
ENSMUSG00000001962	Fam50a	protein_coding	1.039	71.06	0.984
87.185	0.975	44.019	1.033	60.968
ENSMUSG00000001964	Emd	protein_coding	1.025	35.024	0.99
92.355	0.989	62.791	1.048	87.7
ENSMUSG00000001983	Taco1	protein_coding	1.023	75.196	0.936
85.242	1.045	62.995	1.04	59.208
ENSMUSG00000001985	Grik3	protein_coding	1.255	16.74	0.92	96.847
0.867	34.21	0.789	83.724
ENSMUSG00000001986	Gria3	protein_coding	1.489	5.109	0.875	79.807
0.768	28.489	1.148	52.804
ENSMUSG00000001988	Npas1	protein_coding	0.711	68.846	NA	NA
1.104	65.567	0.798	87.496
ENSMUSG00000001995	Sipa1l2	protein_coding	1.1	18.635
0.999	96.013	0.912	17.898	1.022	96.184
ENSMUSG00000001998	Ap4e1	protein_coding	0.941	76.135	0.944
69.175	1.128	17.662	1.007	96.184
ENSMUSG00000001999	Blvra	protein_coding	1.012	81.657	1.002
97.263	0.988	87.744	1.163	31.133
ENSMUSG00000002006	Pdzd4	protein_coding	1.146	25.207	0.858
79.807	1.018	88.274	0.979	83.067
ENSMUSG00000002007	Srpk3	protein_coding	1.371	27.545	1.356
72.642	0.537	1.898	1.158	92.564
ENSMUSG00000002010	Idh3g	protein_coding	0.975	40.217	0.981
93.899	1.045	69.825	1.003	95.92
ENSMUSG00000002012	Pnck	protein_coding	1.222	2.352	0.937	95.088
0.874	15.376	0.98	84.29
ENSMUSG00000002014	Ssr4	protein_coding	1.02	41.454	1.044
96.268	0.94	52.087	1.031	75.81
ENSMUSG00000002015	Bcap31	protein_coding	0.935	7.58	1.055
88.136	1.012	78.155	0.993	95.877
ENSMUSG00000002017	Fam98a	protein_coding	0.93	73.292
1.134	82.904	0.948	52.658	1.062	40.024
ENSMUSG00000002020	Ltbp2	protein_coding	0.731	1.867	1.228	39.442
1.111	38.099	0.89	83.724
ENSMUSG00000002028	Kmt2a	protein_coding	1.009	70.213	NA	NA

1.058 58.915 0.87 83.724
ENSMUSG00000002031 Ift46 protein_coding 0.92 13.199 0.93
63.619 1.171 7.635 0.96 88.387
ENSMUSG00000002032 Tmem25 protein_coding 1.054 64.193
1.029 96.216 0.922 88.076 0.903 92.181
ENSMUSG00000002033 Cd3g protein_coding 1.192 56.085 1.114
94.887 0.754 62.791 0.87 89.892
ENSMUSG00000002043 Trappc6a protein_coding 0.872 20.369
1.036 96.216 1.112 19.452 0.952 84.29
ENSMUSG00000002052 Supt6 protein_coding 0.995 62.689 1.029
91.532 0.975 87.336 1.006 93.132
ENSMUSG00000002055 Spag5 protein_coding 1.288 49.868 0.746
73.766 1.042 87.744 1.305 74.047
ENSMUSG00000002057 Foxn1 protein_coding 1 86.006 1
92.545 1 62.995 1 91.335
ENSMUSG00000002058 Unc119 protein_coding 0.893 48.354 1.01
96.847 1.11 57.227 1.096 86.209
ENSMUSG00000002059 Rab34 protein_coding 0.94 10.143 1.12
81.039 0.952 84.434 1.028 88.512
ENSMUSG00000002064 Sdf2 protein_coding 0.988 74.482 1.019
95.196 NA NA 0.977 92.181
ENSMUSG00000002068 Ccne1 protein_coding 0.925 69.943 0.883
93.908 1.219 74.082 1.071 77.331
ENSMUSG00000002076 Hsf2bp protein_coding 1.027 66.58 0.67
55.71 1.454 41.806 0.918 92.675
ENSMUSG00000002083 Bbc3 protein_coding 0.872 38.286 1.122
79.411 1.023 86.144 0.882 85.493
ENSMUSG00000002100 Mybpc3 protein_coding 2.828 28.395
0.831 97.263 0.426 18.965 1.041 86.445
ENSMUSG00000002102 Psmc3 protein_coding 0.901 1.175 1.017 92.111
1.094 7.635 1.113 31.133
ENSMUSG00000002103 Acp2 protein_coding 0.981 86.333 1.035
95.735 0.984 81.525 1.048 78.546
ENSMUSG00000002104 Rapsn protein_coding 1.004 51.826 0.968
88.881 1.025 78.713 1.205 83.276
ENSMUSG00000002105 Slc39a13 protein_coding 0.987 79.577
1.084 71.617 0.933 1.342 1.051 92.018
ENSMUSG00000002107 Celf2 protein_coding 1.078 21.146 0.961
83.876 0.966 69.134 0.969 85.493
ENSMUSG00000002108 Nr1h3 protein_coding 0.846 5.967 0.994 96.897
1.189 20.572 1.035 94.654
ENSMUSG00000002109 Ddb2 protein_coding 1.152 14.205 0.915
88.881 0.944 35.559 0.874 86.991
ENSMUSG00000002111 Spi1 protein_coding 1.473 0.664 0.835 82.848
0.81 12.41 1.099 73.939
ENSMUSG00000002129 Sf3a1 protein_coding 1.074 13.105 0.995
95.735 0.938 7.948 1.059 56.339
ENSMUSG00000002147 Stat6 protein_coding 0.994 81.01 1.047 88.923
0.96 69.974 0.974 88.505
ENSMUSG00000002190 Clgn protein_coding 1.597 85.946 0.563
96.138 1.114 87.744 1.156 92.018
ENSMUSG00000002204 Napsa protein_coding 1.239 5.109 0.991 95.088
0.812 28.489 0.968 92.691
ENSMUSG00000002205 Vrk3 protein_coding 1.023 69.777 1.02

96.897	0.961	35.559	0.991	88.505
ENSMUSG00000002210	Smg9	protein_coding	0.858	6.952	1.056	89.494
1.099	32.776	1.067	56.863
ENSMUSG00000002221	Paxip1	protein_coding	0.993	62.158
1.043	83.104	0.967	73.598	0.983	88.245
ENSMUSG00000002222	Rmnd5a	protein_coding	0.893	30.877
0.909	83.733	1.229	10.76	0.974	86.991
ENSMUSG00000002227	Mov10	protein_coding	1.076	17.582	1.212
75.612	0.769	0.945	0.961	96.086
ENSMUSG00000002228	Ppm1j	protein_coding	0.928	57.544	1.048
83.104	1.025	78.713	0.866	83.724
ENSMUSG00000002233	Rhoc	protein_coding	0.979	69.24	1.008	94.767
1.014	85.921	0.987	91.872
ENSMUSG00000002240	4930432K09Rik	protein_coding	1	82.732
1	93.046	1	85.792	1	94.608
ENSMUSG00000002249	Tead3	protein_coding	0.872	2.142	1.068	92.062
1.077	14.131	0.972	86.699
ENSMUSG00000002250	Ppard	protein_coding	0.987	71.142	1.107
71.617	0.918	17.898	0.946	91.291
ENSMUSG00000002257	Def6	protein_coding	0.862	10.739	0.984
91.361	1.175	9.301	1.047	95.273
ENSMUSG00000002265	Peg3	protein_coding	2.025	2.969	0.801	60.678
0.617	9.347	0.724	84.29
ENSMUSG00000002266	Zim1	protein_coding	0.959	75.115	0.891
95.504	1.168	81.976	1.064	90.805
ENSMUSG00000002274	Metrn	protein_coding	1.085	34.439	1.045
82.904	0.879	12.907	1.029	74.896
ENSMUSG00000002279	Lmf1	protein_coding	1.002	71.763	1.005
87.604	0.993	86.144	0.898	83.952
ENSMUSG00000002280	Narfl	protein_coding	0.91	8.122	1.001	95.708
1.093	15.411	1.01	91.525
ENSMUSG00000002289	Angptl4	protein_coding	0.836	55.939
0.924	54.06	1.292	9.176	0.964	94.99
ENSMUSG00000002297	Dbf4	protein_coding	1.091	49.517	0.877
83.58	1.047	85.935	1.19	60.466
ENSMUSG00000002307	Daxx	protein_coding	1.006	78.744	0.947
81.443	1.051	60.5	1.159	56.863
ENSMUSG00000002308	Cd320	protein_coding	0.881	57.227	1.121
80.277	1.009	87.519	1.204	73.401
ENSMUSG00000002319	Ipo4	protein_coding	0.954	52.258	1.024
92.561	1.02	72.695	1.117	57.075
ENSMUSG00000002320	Tm9sf1	protein_coding	1.032	25.735
1.027	89.434	0.944	14.262	1.013	83.067
ENSMUSG00000002324	Rec8	protein_coding	0.798	28.804	1.216
96.763	1.027	46.575	0.865	94.904
ENSMUSG00000002325	Irf9	protein_coding	1.105	43.654	1.012
95.211	0.896	48.577	1.035	72.826
ENSMUSG00000002326	Gmpr2	protein_coding	1.042	38.682	1.009
96.268	0.953	71.004	1.016	83.067
ENSMUSG00000002329	Mdp1	protein_coding	0.971	49.368	1.058
72.546	0.975	56.365	1.043	78.379
ENSMUSG00000002332	Dhrs1	protein_coding	0.989	54.87	1.035	87.609
0.977	83.275	1.036	69.918
ENSMUSG00000002341	Ncan	protein_coding	0.168	0.987	1.677	86.277

3.54 5.964 0.861 95.273
ENSMUSG00000002342 Tmem161a protein_coding 0.922 1.017 0.975
93.654 1.107 6.202 0.991 90.463
ENSMUSG00000002343 Armc6 protein_coding 1.009 81.647 1.088
80.806 0.906 30.827 1.258 68.502
ENSMUSG00000002345 2310045N01Rik protein_coding 0.897 4.73
1.073 82.904 1.037 36.011 1.007 93.316
ENSMUSG00000002346 Slc25a42 protein_coding 1.199 15.602
1.141 72.546 0.729 0.72 1.197 63.114
ENSMUSG00000002365 Snx9 protein_coding 1.165 0.767 0.973 91.897
0.88 0.879 1.025 93.156
ENSMUSG00000002372 Ranbp3 protein_coding 0.965 20.369
0.992 94.597 1.044 23.668 1.04 73.368
ENSMUSG00000002379 Ndufa11 protein_coding 0.712 0.987 1.178
67.462 1.194 24.375 1.182 68.696
ENSMUSG00000002384 Bmp8b protein_coding 0.799 53.679 0.912
90.256 1.368 35.13 1.061 71.653
ENSMUSG00000002393 Nr2f6 protein_coding 0.961 47.266 1.121
80.775 0.93 77.294 0.962 84.29
ENSMUSG00000002395 NA NA NA NA 0.964 81.731 1.036
48.304 0.924 83.724
ENSMUSG00000002396 Ocel1 protein_coding 0.851 22.731 1.065
89.434 1.107 39.925 0.93 83.724
ENSMUSG00000002409 Dyrk1b protein_coding 0.802 3.07 1.125
83.104 1.106 40.575 1.038 95.676
ENSMUSG00000002413 Braf protein_coding 0.987 80.608 1.037
96.843 0.973 87.519 0.926 83.724
ENSMUSG00000002416 Ndufb2 protein_coding 0.91 16.02 1.007
95.708 1.094 35.13 1.01 95.74
ENSMUSG00000002428 Hltf protein_coding 0.992 67.374 0.971
76.978 1.039 55.352 0.982 93.316
ENSMUSG00000002455 Prpf6 protein_coding 1.026 36.124 0.975
75.345 1.001 86.144 1.038 72.826
ENSMUSG00000002458 Rgs19 protein_coding 0.993 71.06 1.17 72.642
0.86 7.742 1.041 91.525
ENSMUSG00000002459 Rgs20 protein_coding 0.901 64.893 1.03
96.68 1.079 75.155 0.815 84.29
ENSMUSG00000002475 Abhd3 protein_coding 0.96 75.115 1.093
96.68 0.953 84.896 0.836 84.29
ENSMUSG00000002477 Snrpd1 protein_coding 1.072 47.174
0.899 65.165 1.037 80.089 1.144 31.133
ENSMUSG00000002486 Tchp protein_coding 0.896 10.29 1.006 93.707
1.107 24.07 1.023 93.457
ENSMUSG00000002489 Tiam1 protein_coding 1.327 0.461 1.041 96.68
0.726 10.726 1.053 95.877
ENSMUSG00000002496 Tsc2 protein_coding 0.932 1.815 1.03 86.34
1.042 25.425 1.066 43.377
ENSMUSG00000002500 Rpl3l protein_coding 6.176 7.255 1.089 90.116
0.149 0.72 1.057 94.016
ENSMUSG00000002504 Slc9a3r2 protein_coding 0.888 14.427 1.01
95.504 1.119 32.776 1.044 92.675
ENSMUSG00000002524 Puf60 protein_coding 0.942 1.343 1.013 81.039
1.048 11.348 1.027 53.923
ENSMUSG00000002546 Golga2 protein_coding 1.055 35.787

0.935 70.29 1.013 84.724 1.021 85.786
ENSMUSG00000002550 Uck1 protein_coding 1.149 44.694 0.989
96.216 0.88 38.534 0.873 85.493
ENSMUSG00000002565 Scin protein_coding 1.171 4.675 1.05 95.088
0.811 15.63 1.017 91.372
ENSMUSG00000002578 Ikzf4 protein_coding 1.529 1.493 0.945 87.147
0.694 11.314 0.945 85.493
ENSMUSG00000002580 Mien1 protein_coding 0.845 1.379 1.038 87.185
1.134 20.315 0.985 96.248
ENSMUSG00000002588 Pon1 protein_coding 0.668 5.311 1.252 78.705
1.197 41.331 0.855 85.493
ENSMUSG00000002602 Axl protein_coding 1.041 32.873 1.036
95.35 0.928 63.075 1.091 44.882
ENSMUSG00000002603 Tgfb1 protein_coding 1.183 7.553 0.981 93.654
0.862 6.858 0.99 92.52
ENSMUSG00000002608 Ccdc97 protein_coding 0.894 4.993 1.032
79.411 1.079 26.912 1.073 83.067
ENSMUSG00000002617 Zfp40 protein_coding 1.095 18.314 0.916
94.48 1 39.755 1.113 71.653
ENSMUSG00000002625 Akap8l protein_coding 0.843 0.987 0.985
96.216 1.198 5.864 0.943 86.699
ENSMUSG00000002635 Pdcd2l protein_coding 1.056 24.253
0.975 82.947 0.97 86.112 0.885 83.724
ENSMUSG00000002658 Gtf2f1 protein_coding 0.971 41.021 1.01
92.931 1.019 79.554 1.063 52.366
ENSMUSG00000002660 Clpp protein_coding 0.881 7.574 1.125 83.104
1.01 77.17 0.987 92.762
ENSMUSG00000002661 Alkbh7 protein_coding 0.931 45.229
1.194 65.165 0.9 27.994 0.952 86.699
ENSMUSG00000002668 Dennd1c protein_coding 1.152 30.788
0.906 73.166 0.955 88.076 0.925 90.611
ENSMUSG00000002679 Med6 protein_coding 1.043 20.296 1.012
90.29 0.948 10.602 1.058 62.594
ENSMUSG00000002688 Prkd1 protein_coding 0.925 34.305 1.037
95.735 1.043 42.199 0.966 94.904
ENSMUSG00000002699 Lcp2 protein_coding 1.325 0.2 1.051 92.561
0.719 2.817 1.083 80.573
ENSMUSG00000002718 Cse1l protein_coding 1.024 67.608 1.03
79.261 0.948 23.48 1.011 73.597
ENSMUSG00000002728 Naa20 protein_coding 1.078 57.655 NA NA
0.937 31.887 1.001 83.276
ENSMUSG00000002731 Prkra protein_coding 1.062 19.893 0.96
73.166 0.983 80.5 1.022 93.964
ENSMUSG00000002732 Fkbp7 protein_coding 1.025 41.211 1.005
93.654 0.969 68.584 1.073 57.1
ENSMUSG00000002733 Plekha3 protein_coding 0.982 74.8 1.068
71.222 0.955 14.788 0.987 95.273
ENSMUSG00000002741 Ykt6 protein_coding 0.952 42.722 1.074
75.62 0.974 68.584 1.113 56.079
ENSMUSG00000002748 Baz1b protein_coding 0.973 32.557 1.001
83.464 1.027 84.626 1.074 50.452
ENSMUSG00000002763 Pex6 protein_coding 0.893 9.508 1.056 89.996
1.062 27.047 0.954 84.29
ENSMUSG00000002767 Mrpl2 protein_coding 0.828 1.965 1.07 85.501

1.13 26.498 1.019 90.455
ENSMUSG00000002768 Mea1 protein_coding 0.826 0.987 1.08 82.809
1.124 6.512 1.045 67.691
ENSMUSG00000002769 Gnmt protein_coding 0.767 34.954 NA NA
1.174 36.95 0.94 83.724
ENSMUSG00000002771 Grin2d protein_coding 0.854 41.33 1.115
72.642 1.052 78.954 1.037 90.125
ENSMUSG00000002778 Kdelr1 protein_coding 1.087 0.999 0.986
91.275 0.931 14.262 0.999 90.157
ENSMUSG00000002781 Tmem143 protein_coding 0.891 3.623 1.112
82.904 1.008 51.596 0.904 85.947
ENSMUSG00000002791 Pdxk-ps pseudogene 1.226 58.896 0.637
81.731 1.277 80.015 0.769 87.651
ENSMUSG00000002797 Ggct protein_coding 1.014 63.126 1.059
88.092 0.926 48.577 1.121 66.068
ENSMUSG00000002799 Jag2 protein_coding 0.829 17.898 1.139
83.104 1.059 34.403 0.914 88.505
ENSMUSG00000002803 Btbd6 protein_coding 1.024 72.655 0.976
96.763 1.002 49.217 1.031 91.625
ENSMUSG00000002804 Nudt14 protein_coding 0.835 34.305
1.039 91.532 1.147 26.179 1.048 92.181
ENSMUSG00000002808 Epdr1 protein_coding 1.059 30.788 1.088
96.68 0.868 24.163 1.213 33.269
ENSMUSG00000002812 Flii protein_coding 0.965 25.551 1.034
82.904 1.006 78.369 1.015 91.807
ENSMUSG00000002814 Top3a protein_coding 0.945 34.954 0.883
38.923 1.198 5.964 1.051 68.696
ENSMUSG00000002820 Atg4d protein_coding 0.918 19.595 1.003
92.561 1.082 47.935 1.021 96.183
ENSMUSG00000002825 Qtrt1 protein_coding 0.771 9.508 0.958 77.598
1.358 6.202 1.11 58.557
ENSMUSG00000002831 Plin4 protein_coding 1.021 84.738 1.02
96.843 0.957 74.535 1.521 83.067
ENSMUSG00000002833 Hdgfrp2 protein_coding 0.874 2.174 1.009
96.138 1.138 7.365 1.074 68.696
ENSMUSG00000002835 Chaf1a protein_coding 1.007 79.277
1.078 87.452 0.919 79.625 1.243 46.412
ENSMUSG00000002844 Adprh protein_coding 1.022 37.091 1.046
93.27 0.936 25.052 1.13 40.894
ENSMUSG00000002845 Tmem39a protein_coding 0.979 84.534
1.089 80.775 0.939 21.331 1.059 71.653
ENSMUSG00000002846 Timmdc1 protein_coding 0.942 34.162
1.067 83.104 0.996 88.729 1.052 83.067
ENSMUSG00000002847 Pla1a protein_coding 1.204 6.23 0.884 81.134
0.942 45.799 1.157 0
ENSMUSG00000002870 Mcm2 protein_coding 1.029 84.168 0.969
91.361 1.005 88.161 1.318 31.133
ENSMUSG00000002871 Tpra1 protein_coding 0.898 4.941 1.037 97.263
1.072 19.609 1.008 90.628
ENSMUSG00000002881 Nab1 protein_coding 1.038 71.06 1.028 83.104
0.939 22.864 1.024 90.463
ENSMUSG00000002885 Cd97 protein_coding 1.06 48.462 1.015
92.253 0.932 40.338 1.085 83.067
ENSMUSG00000002897 Il17ra protein_coding 1.041 33.099 0.97

				91.361	0.986	46.32	1.712	62.594	
ENSMUSG00000002900	Lamb1	protein_coding	0.936	65.388	0.923	45.262	1.156	28.055	1.107 82.85
ENSMUSG00000002908	Kcnn1	protein_coding	1.3	8.253	0.999	91.361	0.767	12.41	0.935 91.291
ENSMUSG00000002910	Arrdc2	protein_coding	1.115	73.734	0.818	59.396	1.098	52.432	0.979 93.457
ENSMUSG00000002930	Ppp1r17	protein_coding	1	81.925	1	97.263	1	80.163	NA NA
ENSMUSG00000002944	Cd36	protein_coding	1.034	84.738	NA	NA	0.963	85.464	0.938 86.699
ENSMUSG00000002948	Map2k7	protein_coding	0.916	18.907	1.066	84.999	1.026	55.81	0.981 91.525
ENSMUSG00000002949	Timm44	protein_coding	0.916	3.101	0.983	95.504	1.11	5.864	1.015 83.581
ENSMUSG00000002957	Ap2a2	protein_coding	1.071	0.355	0.955	88.704	0.979	45.573	1.135 52.982
ENSMUSG00000002963	Pnkp	protein_coding	0.967	38.195	0.981	94.597	1.052	34.807	1.03 69.708
ENSMUSG00000002968	Med25	protein_coding	0.938	14.221	1.101	71.98	0.967	86.346	0.985 96.183
ENSMUSG00000002980	Bcam	protein_coding	1.023	85.728	1.003	91.643	0.975	82.903	0.982 86.991
ENSMUSG00000002981	Clptm1	protein_coding	0.963	18.379	1.078	71.617	0.964	66.201	0.985 86.699
ENSMUSG00000002983	Relb	protein_coding	1.068	61.171	1.08	82.904	0.868	12.571	0.83 83.724
ENSMUSG00000002984	Tomm40	protein_coding	0.94	8.122	1.072	82.904	0.991	62.648	0.958 92.564
ENSMUSG00000002985	Apoe	protein_coding	0.999	82.352	1.06	97.263	NA	NA	0.952 83.952
ENSMUSG00000002992	Apoc2	protein_coding	0.801	47.266	1.22	95.735	1.019	81.032	1.002 86.991
ENSMUSG00000002996	Hbp1	protein_coding	1.048	34.861	0.946	60.678	1.007	82.436	0.995 87.542
ENSMUSG00000002997	Prkar2b	protein_coding	0.867	18.907	1.056	97.155	1.087	28.055	0.924 88.797
ENSMUSG00000003031	Cdkn1b	protein_coding	1.006	73.585	1.036	93.654	0.962	67.163	0.889 86.699
ENSMUSG00000003032	Klf4	protein_coding	1.13	86.333	0.923	67.941	0.961	57.286	0.944 95.74
ENSMUSG00000003033	Ap1m1	protein_coding	0.979	48.354	1.079	71.617	0.948	39.245	1.065 83.067
ENSMUSG00000003037	Rab8a	protein_coding	1.064	10.391	1.001	91.275	0.939	40.19	1.005 91.928
ENSMUSG00000003038	Hmgn2	protein_coding	1.123	1.395	0.962	74.483	0.927	23.1	1.136 35.053
ENSMUSG00000003039	Fam32a	protein_coding	0.996	72.139	0.951	60.806	1.056	20.968	0.987 94.153
ENSMUSG00000003051	Elf3	protein_coding	0.767	9.508	0.985	95.088	1.323	13.047	0.897 85.493
ENSMUSG00000003053	Cyp2c29	protein_coding	1	84.819	1	94.262	1	81.976	1 95.593
ENSMUSG00000003062	Stard3nl	protein_coding	1.306	0.2	0.915				

38.699	0.839	13.551	1.106	32.698
ENSMUSG00000003068	Stk11	protein_coding	0.96	20.116	1.057
83.104	0.984	72.148	0.948	85.493
ENSMUSG00000003070	Efna2	protein_coding	1.244	11.316	1.17
86.387	0.685	7.329	0.873	88.955
ENSMUSG00000003072	Atp5d	protein_coding	0.861	3.401	1.079	82.904
1.077	32.433	0.973	94.904
ENSMUSG00000003099	Ppp5c	protein_coding	0.907	2.822	NA	NA
1.066	8.339	1.014	80.497
ENSMUSG00000003119	Cdk12	protein_coding	0.986	64.893	1.027
84.999	0.99	72.464	0.991	94.001
ENSMUSG00000003123	Lipe	protein_coding	0.88	4.385	1.014	80.775
1.121	41.331	1.047	91.372
ENSMUSG00000003131	Pafah1b2	protein_coding	1.047	3.151	NA	NA
0.927	6.858	1.007	83.067
ENSMUSG00000003134	Tbc1d8	protein_coding	1.07	80.688
0.819	24.911	1.139	23.604	0.945	93.612
ENSMUSG00000003135	Cnot11	protein_coding	0.995	66.129
1.045	96.68	0.962	77.888	0.935	86.445
ENSMUSG00000003153	Slc2a3	protein_coding	1.117	72.372
0.937	93.469	0.958	87.336	1.145	95.273
ENSMUSG00000003154	Foxj2	protein_coding	0.984	55.303	1.059
71.617	0.957	45.573	0.981	90.157
ENSMUSG00000003161	Sri	protein_coding	1.063	2.655	0.935	61.883
1.005	87.336	1.029	59.394
ENSMUSG00000003166	Dgcr2	protein_coding	0.968	38.286	1.04
83.104	0.992	84.724	1.046	81.251
ENSMUSG00000003178	Mical3	protein_coding	1.025	74.516
1.076	93.27	0.905	59.125	0.915	87.182
ENSMUSG00000003184	Irf3	protein_coding	0.908	1.031	1.014	90.895
1.081	20.572	1.005	96.183
ENSMUSG00000003190	Bcl2l12	protein_coding	0.895	3.763	1.027
82.809	1.084	28.74	1.001	94.99
ENSMUSG00000003198	Zfp959	protein_coding	1.141	26.77	0.933
79.475	0.934	87.173	0.99	95.676
ENSMUSG00000003199	Mpnd	protein_coding	0.853	0.987	1.088	81.54	1.08
7.635	0.991	91.625
ENSMUSG00000003200	Sh3gl1	protein_coding	0.988	85.832
1.026	89.494	0.981	60.614	1.063	58.721
ENSMUSG00000003206	Ebi3	protein_coding	1.325	8.996	1.105	96.68	0.68
13.551	0.806	84.19
ENSMUSG00000003208	Ccdc94	protein_coding	1.003	82.705	NA
NA	0.995	84.434	1.032	92.181
ENSMUSG00000003226	Ranbp2	protein_coding	0.934	38.983
0.954	92.355	1.119	29.819	1.041	85.786
ENSMUSG00000003227	Edar	protein_coding	1.031	69.489	NA	NA
0.66	71.734	1.105	96.086
ENSMUSG00000003228	Grk5	protein_coding	1.012	86.145	1.013
96.843	0.977	86.666	0.927	91.217
ENSMUSG00000003233	Dvl3	protein_coding	0.934	24.344	1.118
71.617	0.96	63.075	0.923	83.724
ENSMUSG00000003234	Abcf3	protein_coding	1.031	69.089	0.969
85.671	1.002	86.602	1.033	74.193
ENSMUSG00000003235	Eif2b5	protein_coding	0.996	82.982

0.988 81.134 1.014 36.578 1.042 52.324
ENSMUSG00000003269 Cyth2 protein_coding 0.924 13.74 1.066 81.127
1.017 57.286 0.95 86.699
ENSMUSG00000003271 Sult2b1 protein_coding 0.782 54.604
0.855 72.576 1.488 27.732 0.773 86.699
ENSMUSG00000003273 Car11 protein_coding 0.949 72.985 1.04
88.923 1.012 79.554 0.901 91.269
ENSMUSG00000003279 Dlgap1 protein_coding 1.294 9.983 0.993
97.155 0.775 11.504 0.946 93.316
ENSMUSG00000003282 Plag1 protein_coding 0.776 6.677 1.097 86.156
1.168 30.645 0.854 85.493
ENSMUSG00000003283 Hck protein_coding 1.399 6.135 0.941 93.654
0.76 11.116 1.471 72.204
ENSMUSG00000003299 Mrpl4 protein_coding 0.878 1.175 1.049 79.367
1.082 25.966 1.082 79.268
ENSMUSG00000003308 Keap1 protein_coding 0.95 21.176 1.023
87.609 1.029 66.269 1.032 42.736
ENSMUSG00000003309 Ap1m2 protein_coding 0.881 49.621 0.84
70.864 1.349 30.561 0.862 84.29
ENSMUSG00000003316 Glg1 protein_coding 1.033 1.815 0.988 68.115
0.982 28.489 1.022 93.156
ENSMUSG00000003341 Atp8b3 protein_coding 5.594 33.325
0.215 90.053 0.828 36.499 0.784 90.157
ENSMUSG00000003344 Btbd2 protein_coding 0.952 30.136 0.984
94.96 1.065 24.07 1.003 91.291
ENSMUSG00000003345 Csnk1g2 protein_coding 0.952 56.477
1.008 97.155 1.044 59.612 1.007 93.316
ENSMUSG00000003346 Abhd17a protein_coding 0.951 61.887
1.033 88.136 1.021 87.336 0.958 84.29
ENSMUSG00000003348 Mob3a protein_coding 1.122 14.205 0.984
92.793 0.906 3.432 1.067 40.894
ENSMUSG00000003352 Cacnb3 protein_coding 0.909 21.957
0.995 88.705 1.106 17.998 0.774 86.699
ENSMUSG00000003354 Ccdc65 protein_coding 1.164 33.555 0.69
64.555 1.243 74.686 1.353 93.261
ENSMUSG00000003355 Fkbp11 protein_coding 1.114 57.949 0.95
96.847 0.944 56.365 1.158 67.691
ENSMUSG00000003360 Ddx23 protein_coding 0.959 20.116 1.005
93.654 1.036 28.979 0.995 92.12
ENSMUSG00000003363 Pld3 protein_coding 1.178 9.435 0.919 55.71
0.924 60.614 0.879 88.053
ENSMUSG00000003378 Grik5 protein_coding 0.85 10.143 1.029
94.96 1.144 13.356 0.884 83.724
ENSMUSG00000003379 Cd79a protein_coding 1.084 83.968 0.852
91.361 1.081 70.9 0.732 84.29
ENSMUSG00000003380 Rabac1 protein_coding 0.919 18.379
1.069 83.104 1.021 69.315 0.89 83.724
ENSMUSG00000003382 Etv3 protein_coding 1.02 55.462 0.946
74.483 1.031 70.623 0.977 95.593
ENSMUSG00000003402 Prkcsh protein_coding 0.923 10.739
1.029 95.504 1.055 22.444 1.026 94.904
ENSMUSG00000003410 Elavl3 protein_coding 1.179 72 0.954
92.545 0.885 86.985 0.66 86.699
ENSMUSG00000003411 Rab3b protein_coding 1.068 62.689 0.995

96.847 0.942 58.271 0.981 95.832
ENSMUSG00000003418 St8sia6 protein_coding 1.057 76.827 NA
NA 0.933 87.971 0.869 87.182
ENSMUSG00000003420 Fcgrt protein_coding 1.118 1.626 0.897 56.781
0.999 79.255 1.009 93.261
ENSMUSG00000003421 Nosip protein_coding 0.926 12.309 1.037
91.776 1.042 48.451 0.983 86.991
ENSMUSG00000003423 Pih1d1 protein_coding 0.916 7.207 0.969
90.053 1.125 12.35 1.013 95.877
ENSMUSG00000003429 Rps11 protein_coding 1.09 2.651 0.979 54.245
0.937 57.819 1.033 95.35
ENSMUSG00000003435 Supt5 protein_coding 0.985 61.043 0.952
37.376 1.062 9.86 1.041 52.982
ENSMUSG00000003437 Paf1 protein_coding 0.96 7.58 1.009 93.27
1.029 35.894 1.016 77.331
ENSMUSG00000003438 Timm50 protein_coding 0.93 12.084
1.059 83.464 1.017 76.754 0.98 91.372
ENSMUSG00000003444 Med29 protein_coding 0.978 75.858 1.002
90.712 1.024 42.75 1.036 72.826
ENSMUSG00000003452 Bicd1 protein_coding 0.938 75.115 1.029
93.654 1.037 55.512 0.965 94.384
ENSMUSG00000003458 Ncstn protein_coding 1.046 13.633 0.974
86.604 0.979 35.559 1.015 83.067
ENSMUSG00000003464 Pex19 protein_coding 0.881 3.07 1.052 85.756
1.074 27.047 1.006 91.525
ENSMUSG00000003469 Phyhip protein_coding 0.678 25.111
0.921 89.713 1.595 10.819 1.185 86.209
ENSMUSG00000003476 Crhr2 protein_coding 1.851 16.539 1.445
79.055 0.374 1.48 0.857 93.722
ENSMUSG00000003477 Inmt protein_coding 1.36 74.745 0.547
68.115 1.346 38.975 0.955 93.685
ENSMUSG00000003484 Cyp4f18 protein_coding 1.445 7.908 0.69
24.911 1.006 74.686 0.758 86.991
ENSMUSG00000003500 Impdh1 protein_coding 0.853 1.031 0.987
86.861 1.189 5.864 1.025 92.018
ENSMUSG00000003505 Psg18 protein_coding 1 77.615 1
95.504 1 82.161 1 92.018
ENSMUSG00000003518 Dusp3 protein_coding 1.114 15.662 0.988
95.708 0.911 23.607 1.094 91.525
ENSMUSG00000003526 Prodh protein_coding 0.731 44.467 1.044
93.654 1.312 41.235 1.116 93.016
ENSMUSG00000003527 Dgcr14 protein_coding 0.986 65.106
0.938 79.807 1.081 28.301 0.985 89.827
ENSMUSG00000003528 Slc25a1 protein_coding 0.402 0.987 1.389
89.014 1.793 7.635 0.917 94.99
ENSMUSG00000003531 Dgcr6 protein_coding 0.852 8.405 NA NA
1.128 27.475 0.989 88.387
ENSMUSG00000003534 Ddr1 protein_coding 0.9 18.907 1.044
97.155 1.066 37.741 0.952 87.218
ENSMUSG00000003541 Ier3 protein_coding 0.925 68.66 1.103 84.768
NA NA 1.141 77.854
ENSMUSG00000003545 Fosb protein_coding 0.748 51.447 1.216
92.931 1.1 73.759 0.769 93.261
ENSMUSG00000003546 Klc4 protein_coding 0.827 3.401 1.009 97.155

1.198 9.502 0.976 85.493
ENSMUSG00000003549 Ercc1 protein_coding 0.84 0.987 0.95 84.858
1.249 5.864 0.942 96.183
ENSMUSG00000003555 Cyp17a1 protein_coding 1.03 85.655
1.015 97.288 0.955 88.693 0.7 91.105
ENSMUSG00000003559 As3mt protein_coding 1.143 11.638 0.991
94.848 0.881 21.852 1.035 62.594
ENSMUSG00000003573 Homer3 protein_coding 1.081 52.799 0.96
92.137 0.964 87.898 0.972 92.12
ENSMUSG00000003575 Crtc1 protein_coding 1.032 51.189 1.042
87.029 0.93 9.288 0.972 84.19
ENSMUSG00000003581 Rnf215 protein_coding 0.969 46.413
1.033 96.407 1 84.626 0.991 95.273
ENSMUSG00000003585 Sec14l2 protein_coding 0.848 52.9 0.98
90.378 1.201 32.524 0.909 88.053
ENSMUSG00000003604 Aven protein_coding 0.868 2.524 0.97 96.216
1.188 6.202 1.045 76.347
ENSMUSG00000003617 Cp protein_coding 0.935 63.283 0.977
90.256 1.095 45.035 1 90.805
ENSMUSG00000003623 Crot protein_coding 0.991 53.679 1.019
83.464 0.99 71.004 1.028 83.067
ENSMUSG00000003644 Rps6ka1 protein_coding 1.009 69.089
0.989 79.261 1.005 65.034 0.996 94.174
ENSMUSG00000003657 Calb2 protein_coding 1 86.57 1 96.268
1 82.685 1 89.72
ENSMUSG00000003660 Snrnp200 protein_coding 1.064 15.85 0.94
59.946 0.996 67.962 1.018 87.7
ENSMUSG00000003662 Ciao1 protein_coding 0.904 5.311 0.986 95.708
1.12 12.35 1.091 50.74
ENSMUSG00000003665 Has1 protein_coding 1.426 16.124 1.152
90.092 0.608 9.563 1.621 57.952
ENSMUSG00000003680 Taf6l protein_coding 0.981 81.925 1.077
85.756 0.944 61.288 1.051 95.877
ENSMUSG00000003721 Insig2 protein_coding 0.973 63.736
0.937 62.692 1.092 15.049 0.981 89.525
ENSMUSG00000003731 Kpna6 protein_coding 1.009 41.454 1.087
71.617 0.911 2.294 0.986 84.29
ENSMUSG00000003746 Man1a protein_coding 1.132 9.435 0.97 92.404
0.912 40.338 0.934 87.705
ENSMUSG00000003752 Itpkc protein_coding 1 82.231 0.984
89.996 1.016 43.185 0.931 83.724
ENSMUSG00000003762 Adck4 protein_coding 0.823 0.987 1.009 94.887
1.202 5.864 1.111 68.696
ENSMUSG00000003778 Brd8 protein_coding 0.948 2.524 0.947 59.946
1.116 5.864 1.023 60.466
ENSMUSG00000003779 Kif20a protein_coding 1.135 61.171 0.91
73.094 0.966 88.274 1.094 79.268
ENSMUSG00000003808 Farsa protein_coding 0.92 5.204 1.017 91.584
1.064 24.375 1.045 55.485
ENSMUSG00000003809 Gcdh protein_coding 0.92 63.126 1.049
91.456 1.035 84.896 1.02 96.183
ENSMUSG00000003810 Mast2 protein_coding 1.018 82.982 1.015
82.904 0.965 21.666 1.118 36.988
ENSMUSG00000003812 Dnase2a protein_coding 1.149 16.124

0.907 79.261 0.956 32.082 0.948 93.261
ENSMUSG00000003813 Rad23a protein_coding 0.868 0.987 1.084
71.98 1.061 17.662 1.094 40.024
ENSMUSG00000003814 Calr protein_coding 0.94 35.646 1.091
82.904 0.971 85.464 1.081 55.841
ENSMUSG00000003824 Syce2 protein_coding 1.197 21.539 NA NA
0.818 25.512 1.075 95.777
ENSMUSG00000003847 Nfat5 protein_coding 0.94 41.759 1.028
97.155 1.035 43.185 0.905 83.724
ENSMUSG00000003848 Nob1 protein_coding 0.897 14.403 1.008
95.196 1.108 28.603 1.08 72.826
ENSMUSG00000003849 Nqo1 protein_coding 1.048 73.734 1.005
90.68 0.951 44.019 1.014 96.183
ENSMUSG00000003863 Ppfia3 protein_coding 1.071 83.263
0.805 85.116 1.156 43.481 0.809 85.493
ENSMUSG00000003865 Gys1 protein_coding 0.903 9.341 1.122 79.579
0.989 84.961 1.043 89.152
ENSMUSG00000003868 Ruvbl2 protein_coding 0.898 3.641 0.981
93.27 1.132 8.466 1.009 94.654
ENSMUSG00000003872 Lin7b protein_coding 0.776 41.759 1.35
79.367 0.957 75.353 0.805 91.269
ENSMUSG00000003873 Bax protein_coding 0.959 71.915 1.016
95.431 1.026 88.461 1.076 66.468
ENSMUSG00000003882 Il7r protein_coding 1.28 22.349 1.036
95.431 0.753 25.625 0.793 92.181
ENSMUSG00000003923 Tfam protein_coding 1.103 1.292 0.977 75.052
0.928 12.948 1.037 64.513
ENSMUSG00000003929 Zfp81 protein_coding 1.053 42.134 0.947
71.331 1.005 84.896 1.048 95.74
ENSMUSG00000003934 Efnb3 protein_coding 1.018 73.487 1.018
83.104 0.962 65.688 1.03 96.183
ENSMUSG00000003948 Mmd protein_coding 0.822 0.987 1.095 72.642
1.113 27.732 0.905 94.001
ENSMUSG00000003949 Hlf protein_coding 1 56.66 1.082 83.104
0.921 33.971 1.078 77.331
ENSMUSG00000003955 Fam162a protein_coding 0.954 67.334 1.1
79.055 0.954 62.791 0.995 86.699
ENSMUSG00000003970 Rpl8 protein_coding 0.993 82.231 0.977
74.483 1.029 61.743 1.018 89.052
ENSMUSG00000003992 Ssbp2 protein_coding 1.027 45.746 0.958
90.053 1.018 79.776 1.013 94.82
ENSMUSG00000004018 Fancl protein_coding 0.919 75.115 1.042
91.643 NA NA 1.029 90.865
ENSMUSG00000004031 Brinp2 protein_coding 0.469 34.162
1.958 87.609 1.084 80.381 1.454 76.831
ENSMUSG00000004032 Gstm5 protein_coding 0.984 72.546 NA NA
0.983 73.759 1.03 94.384
ENSMUSG00000004035 Gstm7 protein_coding 0.832 42.722 1.151
83.104 1.041 84.724 0.868 85.493
ENSMUSG00000004040 Stat3 protein_coding 0.941 55.641 NA NA
1.031 46.206 1.057 89.052
ENSMUSG00000004043 Stat5a protein_coding 0.975 83.09 1.037
95.809 0.989 64.155 1.379 69.965
ENSMUSG00000004044 Ptrf protein_coding 0.877 12.624 1.046

90.454 1.092 19.609 0.96 95.273
ENSMUSG00000004054 Map3k11 protein_coding 0.982 51.56 1.045
83.104 0.975 83.397 0.973 86.991
ENSMUSG00000004056 Akt2 protein_coding 1.041 49.202 0.999
97.263 0.958 46.071 0.944 85.493
ENSMUSG00000004069 Dnaja3 protein_coding 0.96 62.437 NA
NA 1.023 72.853 1.03 88.657
ENSMUSG00000004070 Hmox2 protein_coding 1.112 0.976 0.975 71.98
0.923 23.86 1.045 74.047
ENSMUSG00000004071 Cdip1 protein_coding 0.892 3.07 1.006 94.48
1.111 9.86 0.961 87.182
ENSMUSG00000004085 Zak protein_coding 1.157 2.784 1.067 79.29
0.812 0.72 0.98 91.872
ENSMUSG00000004096 Cwc15 protein_coding 0.968 10.955 0.981
85.921 1.053 16.666 1.062 51.908
ENSMUSG00000004098 Col5a3 protein_coding 0.932 32.378
0.963 96.843 1.111 53.134 0.95 86.133
ENSMUSG00000004099 Dnmt1 protein_coding 0.995 67.477 0.939
44.268 1.073 18.797 1.053 56.863
ENSMUSG00000004100 Ppan protein_coding 0.888 32.557 NA NA
1.1 33.042 1.106 75.198
ENSMUSG00000004105 Angptl2 protein_coding 1.152 9.805 1.116
72.642 0.78 7.329 1.103 71.305
ENSMUSG00000004110 Cacna1e protein_coding 3.769 0.999 0.526
60.678 0.506 14.079 1.081 95.877
ENSMUSG00000004113 Cacna1b protein_coding 0.789 66.406
1.224 90.116 1.038 83.719 2.025 31.133
ENSMUSG00000004127 Trmt10a protein_coding 1.097 46.875
0.872 70.864 1.048 68.425 0.99 92.381
ENSMUSG00000004151 Etv1 protein_coding 0.939 43.816 0.944
79.807 1.123 17.662 1.13 42.736
ENSMUSG00000004187 Kifc2 protein_coding 0.896 8.365 1.019 93.863
1.094 23.454 0.785 84.29
ENSMUSG00000004207 Psap protein_coding 1.129 1.493 0.917 39.547
0.961 39.079 0.978 92.181
ENSMUSG00000004221 Ikbkg protein_coding 0.948 11.973 0.937
56.027 1.126 5.964 0.98 86.699
ENSMUSG00000004231 Pax2 protein_coding 1 71.36 1 94.597
1 67.163 1 96.183
ENSMUSG00000004233 Wars2 protein_coding 0.996 74.8 0.947 92.404
1.061 70.623 1.069 68.354
ENSMUSG00000004263 Atn1 protein_coding 0.971 42.922 1.088
60.309 0.947 27.994 0.928 84.29
ENSMUSG00000004264 Phb2 protein_coding 0.955 28.804 NA NA
1.046 53.449 0.978 90.157
ENSMUSG00000004266 Ptpn6 protein_coding 1.243 0.944 0.93 84.594
0.866 3.718 1.042 67.691
ENSMUSG00000004267 Eno2 protein_coding 1.199 9.259 0.971 96.378
0.86 0.879 1.015 81.251
ENSMUSG00000004268 Emg1 protein_coding 0.91 7.916 1.003 93.899
1.091 28.997 1.048 70.624
ENSMUSG00000004270 Lpcat3 protein_coding 0.953 48.146
0.975 79.807 1.074 31.975 0.958 83.724
ENSMUSG00000004285 Atp6v1f protein_coding 0.934 3.895 NA NA

1.1 26.663 0.991 91.372
ENSMUSG00000004317 Clcn5 protein_coding 1.177 2.784 0.953 88.897
0.889 37.906 1.085 62.594
ENSMUSG00000004319 Clcn3 protein_coding 1.017 82.092 0.955
66.09 1.028 28.301 0.973 95.273
ENSMUSG00000004328 Hif3a protein_coding 0.976 60.707 0.558
51.115 1.833 12.35 1.173 83.276
ENSMUSG00000004341 Gpx6 protein_coding 1 67.163 1
96.138 1 74.686 1 96.183
ENSMUSG00000004344 Gpx5 protein_coding 1 86.199 1
96.216 1 83.158 1 96.192
ENSMUSG00000004347 Pde1c protein_coding 1.163 63.534 1.122
82.904 0.768 15.904 0.838 85.493
ENSMUSG00000004356 Utp20 protein_coding 0.916 56.477 0.943
91.361 1.155 56.931 1.111 62.594
ENSMUSG00000004359 Spic protein_coding 1.267 64.956 1.209
95.211 0.65 57.446 0.63 85.493
ENSMUSG00000004360 9330159F19Rik protein_coding 0.711 33.745
0.986 91.897 1.419 24.375 0.82 83.724
ENSMUSG00000004364 Cul3 protein_coding 1.126 2.047 0.984 88.345
0.903 4.963 1.015 76.347
ENSMUSG00000004366 Sst protein_coding 1 81.657 1
95.504 1 75.809 1 93.734
ENSMUSG00000004371 Il11 protein_coding 1.138 81.925 1.164
85.756 0.754 46.956 0.823 95.72
ENSMUSG00000004383 Large protein_coding 0.837 17.276 1.161
79.261 1.023 74.082 1.026 91.372
ENSMUSG00000004393 Ddx56 protein_coding 1.075 27.32 0.944 83.321
0.988 69.134 1.022 79.713
ENSMUSG00000004394 Tmed4 protein_coding 0.991 82.543 1.02
95.431 0.992 83.811 0.985 91.65
ENSMUSG00000004415 Col26a1 protein_coding 0.796 45.229
1.466 90.116 0.856 88.693 1.343 85.629
ENSMUSG00000004446 Bid protein_coding 1.025 72.27 1.113 71.617
0.874 9.456 0.867 83.724
ENSMUSG00000004451 Ralb protein_coding 1.07 20.296 1.021
83.104 0.916 2.333 1.023 60.466
ENSMUSG00000004455 Ppp1cc protein_coding 1.12 0 1.036
82.904 0.859 0.72 0.993 90.611
ENSMUSG00000004460 Dnajb11 protein_coding 0.935 36.034
1.025 95.211 1.046 25.425 1.126 38.624
ENSMUSG00000004462 Tbccd1 protein_coding 0.993 78.909
1.022 82.904 0.984 54.037 0.995 94.904
ENSMUSG00000004473 Clec11a protein_coding 1.299 1.712 NA NA
0.771 3.863 1.058 83.067
ENSMUSG00000004500 Zfp324 protein_coding 0.913 54.043
1.129 79.579 0.968 47.641 0.999 95.35
ENSMUSG00000004508 Gab2 protein_coding 1.1 17.761 0.917
70.971 0.993 69.134 0.95 86.445
ENSMUSG00000004530 Coro1c protein_coding 1.163 0.461 0.936
85.116 0.915 2.361 1.053 62.918
ENSMUSG00000004535 Tax1bp1 protein_coding 0.983 82.705
0.984 92.545 1.036 69.002 1.058 42.736
ENSMUSG00000004540 Psg17 protein_coding 1.144 69.943 1.351

96.407 0.647 88.601 1.276 83.581
ENSMUSG00000004542 Psg19 protein_coding 1 67.163 1
95.504 NA NA 1 93.156
ENSMUSG00000004552 Ctse protein_coding 2.009 0.355 0.731 59.946
0.679 9.494 2.064 95.273
ENSMUSG00000004558 Ndrg2 protein_coding 0.984 81.196 0.935
86.277 1.089 75.809 1.14 69.404
ENSMUSG00000004561 Mettl17 protein_coding 0.901 23.556 0.97
83.392 1.141 23.334 1.042 94.297
ENSMUSG00000004562 Arhgef40 protein_coding 0.91 10.514
1.053 83.104 1.045 33.508 1.046 71.855
ENSMUSG00000004565 Pnpla6 protein_coding 0.898 2.009 0.999
93.817 1.112 5.864 0.954 85.493
ENSMUSG00000004567 Mcoln1 protein_coding 0.978 29.454
1.036 79.579 0.988 82.575 1.003 95.273
ENSMUSG00000004568 Arhgef18 protein_coding 0.942 18.795
1.055 79.579 1.004 84.83 0.996 92.181
ENSMUSG00000004591 Pkn2 protein_coding 1.11 2.512 0.96 88.705
0.94 8.634 0.949 83.724
ENSMUSG00000004609 Cd33 protein_coding 1.716 0.577 0.742 81.731
0.787 21.852 0.976 92.675
ENSMUSG00000004610 Etfb protein_coding 0.974 52.018 1.048
83.104 0.981 82.575 0.978 93.612
ENSMUSG00000004612 Nkg7 protein_coding 0.817 60.339 1.476
64.304 0.829 57.091 0.85 86.699
ENSMUSG00000004613 Lim2 protein_coding 1 82.982 1
95.196 1 87.971 1 95.273
ENSMUSG00000004626 Stxbp2 protein_coding 0.889 7.71 1.007
94.848 1.113 8.209 0.993 93.156
ENSMUSG00000004630 Pcp2 protein_coding 0.44 3.641 NA NA
1.999 9.301 1.573 63.018
ENSMUSG00000004631 Sgce protein_coding 1.088 2.969 1.017 95.708
0.902 10.003 0.964 96.358
ENSMUSG00000004633 Chn2 protein_coding 1.123 13.363 0.998
96.268 0.893 59.125 0.969 91.372
ENSMUSG00000004637 Wwox protein_coding 1.072 22.349 1.086
96.407 0.861 13.848 1.039 95.559
ENSMUSG00000004642 Slbp protein_coding 1.121 4.052 0.996 87.313
0.898 27.321 1.062 71.653
ENSMUSG00000004651 Tyr protein_coding 1 78.054 1
87.147 1 64.024 1 92.564
ENSMUSG00000004654 Ghrhr protein_coding 1 86.199 1
96.407 NA NA 1 93.016
ENSMUSG00000004655 Aqp1 protein_coding 1.035 68.955 0.956
93.908 1.013 66.043 0.98 92.389
ENSMUSG00000004661 Arid3b protein_coding 0.942 44.787
1.081 83.104 0.983 87.686 0.929 91.625
ENSMUSG00000004665 Cnn2 protein_coding 1.131 2.651 1.036 84.999
0.852 0.72 1.022 83.581
ENSMUSG00000004667 Polr2e protein_coding 0.952 32.557
0.968 92.793 1.081 66.269 1.008 94.904
ENSMUSG00000004677 Myo9b protein_coding 1.027 47.847 0.968
91.275 1 87.898 1.016 57.075
ENSMUSG00000004698 Hdac9 protein_coding 1.858 0.461 NA NA

0.602 0.72 1.176 59.464
ENSMUSG00000004707 Ly9 protein_coding 1.484 1.242 0.985 91.661
0.681 10.726 1.193 91.492
ENSMUSG00000004709 Cd244 protein_coding 1.188 64.401 1.276
71.617 0.661 11.314 0.839 87.542
ENSMUSG00000004730 Emr1 protein_coding 1.622 0.2 0.894 59.946
0.688 17.258 1.088 93.685
ENSMUSG00000004748 Mtfp1 protein_coding 0.828 11.232 1.248
79.055 0.963 79.554 1.014 93.734
ENSMUSG00000004768 Rab23 protein_coding 1.064 20.032 0.981
95.504 0.957 47.641 0.996 93.964
ENSMUSG00000004771 Rab11a protein_coding 1.094 3.713 0.955
90.256 0.957 6.935 1.046 61.984
ENSMUSG00000004788 Eif2b2 protein_coding 0.934 23.556
0.958 88.691 1.117 20.315 1.01 92.389
ENSMUSG00000004789 Dlst protein_coding 0.995 52.018 1.066
83.104 0.94 87.744 0.988 90.177
ENSMUSG00000004791 Pgf protein_coding 0.77 41.33 NA NA
1.139 42.199 1.245 68.696
ENSMUSG00000004798 Ulk2 protein_coding 1.043 36.195 1.003
93.27 0.956 14.079 0.988 92.561
ENSMUSG00000004814 Ccl24 protein_coding 1.527 9.259 0.746 66.09 0.88
53.7 0.995 92.52
ENSMUSG00000004815 Dgkq protein_coding 0.935 4.055 0.999 96.847
1.067 8.209 0.982 84.29
ENSMUSG00000004821 Tmed11 protein_coding 1 86.658 1
95.941 1 86.666 1 93.261
ENSMUSG00000004837 Grap protein_coding 0.931 30.352 0.956
90.287 1.125 19.609 1.043 57.952
ENSMUSG00000004843 Chmp2b protein_coding 1.016 79.433
0.961 87.452 1.025 54.163 1.002 95.273
ENSMUSG00000004846 Plod3 protein_coding 0.885 7.887 1.052 77.618
1.075 65.235 1.097 67.691
ENSMUSG00000004849 Ap1s1 protein_coding 0.87 1.031 1.021 84.999
1.127 19.452 1.07 55.054
ENSMUSG00000004864 Mapk13 protein_coding 0.875 48.939
0.812 64.123 1.406 14.449 0.908 84.29
ENSMUSG00000004865 Srpk1 protein_coding 0.956 38.286 0.986
88.705 1.059 37.741 0.985 94.19
ENSMUSG00000004880 Lbr protein_coding 1.037 12.396 0.957
96.138 1.007 42.376 1.031 88.657
ENSMUSG00000004885 Crabp2 protein_coding 1.014 86.464
1.078 96.68 0.914 69.445 0.996 96.183
ENSMUSG00000004891 Nes protein_coding 0.877 36.034 1.152
85.985 0.991 82.778 0.972 95.832
ENSMUSG00000004892 Bcan protein_coding 0.909 62.437 1.507
89.996 0.729 77.17 0.5 90.95
ENSMUSG00000004894 Hapln2 protein_coding 1.215 81.178
1.008 96.216 0.818 88.349 0.438 86.699
ENSMUSG00000004895 Prcc protein_coding 1.049 79.277 1.021
82.904 0.932 7.948 0.962 90.95
ENSMUSG00000004896 Rrnad1 protein_coding 0.945 21.957
1.025 88.136 1.025 59.274 0.972 86.991
ENSMUSG00000004897 Hdgf protein_coding 0.891 2.009 1.045 83.104

1.075 13.356 1.019 71.305
ENSMUSG00000004902 Slc25a18 protein_coding 0.502 12.436 0.76
81.443 2.619 5.864 0.892 95.273
ENSMUSG00000004929 Thop1 protein_coding 0.992 58.077 0.918
72.399 1.099 15.688 1.059 53.923
ENSMUSG00000004931 Apba3 protein_coding 0.818 3.559 1.11 74.663
1.103 30.292 1.006 93.132
ENSMUSG00000004933 Matk protein_coding 1.089 65.622 0.919
88.897 0.996 62.648 0.983 88.657
ENSMUSG00000004934 Pias4 protein_coding 0.991 77.497 1.027
96.013 0.985 87.336 0.993 86.991
ENSMUSG00000004936 Map2k1 protein_coding 0.955 52.258
1.036 83.104 1.007 78.954 1.094 53.923
ENSMUSG00000004937 Sgta protein_coding 0.884 1.031 1.062 88.823
1.063 23.454 1.026 94.153
ENSMUSG00000004945 Tmem242 protein_coding 0.878 4.115 0.973
93.654 1.171 7.24 1.049 83.067
ENSMUSG00000004947 Dtx2 protein_coding 1.129 46.533 1.134
80.277 0.782 3.718 0.96 86.991
ENSMUSG00000004951 Hspb1 protein_coding 0.684 0.987 0.969 93.994
1.502 5.864 0.994 95.593
ENSMUSG00000004952 Rasa4 protein_coding 1.195 23.254 0.993
96.847 0.842 11.902 0.858 86.991
ENSMUSG00000004961 Syt5 protein_coding 1.139 84.738 NA NA
0.935 87.274 0.927 88.505
ENSMUSG00000004980 Hnrnpa2b1 protein_coding 0.987 84.534
0.993 93.654 1.018 75.527 1.082 31.133
ENSMUSG00000004988 Fxyd4 protein_coding 1.642 5.848 0.798 95.708
0.761 22.009 1.103 89.428
ENSMUSG00000004994 Ccdc130 protein_coding 0.92 10.514
0.981 86.071 1.107 9.176 1.014 89.428
ENSMUSG00000004996 Mri1 protein_coding 0.989 84.452 1.011
97.288 0.996 87.173 1.047 86.445
ENSMUSG00000005034 Prkacb protein_coding 1.127 0.461 0.962
79.807 0.924 1.705 0.998 95.273
ENSMUSG00000005043 Sgsh protein_coding 1.098 13.734 1.006
95.504 0.908 12.948 0.948 86.699
ENSMUSG00000005045 Chd5 protein_coding 0.999 85.283 1.081
83.104 0.923 45.573 0.949 86.991
ENSMUSG00000005054 Cstb protein_coding 1.034 58.38 1.081 60.309
0.896 4.723 0.99 91.625
ENSMUSG00000005057 Sh2b2 protein_coding 0.715 1.031 1.092 83.104
1.28 11.665 1.103 96.183
ENSMUSG00000005069 Pex5 protein_coding 0.923 7.118 1.022 93.899
1.057 14.376 0.969 83.724
ENSMUSG00000005078 Jkamp protein_coding 1.078 13.363 1.032
85.985 0.896 2.563 0.916 77.331
ENSMUSG00000005087 Cd44 protein_coding 1.093 25.207 1.065
93.899 0.858 27.675 1.052 59.394
ENSMUSG00000005089 Slc1a2 protein_coding 1.054 85.391 NA
NA 0.83 88.729 0.674 96.248
ENSMUSG00000005102 Eif2ak4 protein_coding 0.997 72.139
0.965 91.361 1.034 27.533 0.993 93.316
ENSMUSG00000005103 Wdr1 protein_coding 1.123 1.689 NA NA

0.935 3.123 1.093 79.442
ENSMUSG00000005107 Slc2a9 protein_coding 1.21 13.734
1.052 97.155 0.783 8.192 0.984 96.287
ENSMUSG00000005124 Wisp1 protein_coding 1.163 40.635 1.311
88.533 0.656 14.206 0.859 91.291
ENSMUSG00000005125 Ndrg1 protein_coding 1.084 32.873 0.935
78.471 0.987 80.5 0.986 94.904
ENSMUSG00000005131 4930550C14Rik protein_coding 1.194 47.847
0.881 96.216 0.95 69.974 0.911 96.287
ENSMUSG00000005142 Man2b1 protein_coding 1.114 10.595
0.947 61.883 0.944 82.685 0.988 96.071
ENSMUSG00000005148 Klf5 protein_coding 0.765 15.456 1.041
96.763 1.25 34.807 0.939 85.493
ENSMUSG00000005150 Wdr83 protein_coding 0.886 3.07 1.035 88.092
1.095 11.518 1.028 76.467
ENSMUSG00000005161 Prdx2 protein_coding 0.929 17.664 1.024
97.155 1.05 29.777 1.054 70.539
ENSMUSG00000005198 Polr2a protein_coding 0.943 33.002 0.96
87.857 1.099 30.107 0.999 91.65
ENSMUSG00000005202 Shbg protein_coding 1 85.728 1
96.843 1 74.535 1 96.071
ENSMUSG00000005204 Senp3 protein_coding 0.903 0.987 1.029 82.904
1.079 20.315 1.036 74.047
ENSMUSG00000005225 Plekha8 protein_coding 1.018 29.057
1.034 84.768 0.951 12.571 1.178 65.558
ENSMUSG00000005232 G6pc2 protein_coding 1 85.728 1
95.941 1 84.669 1 95.35
ENSMUSG00000005233 Spc25 protein_coding 1.266 27.113 0.866
78.471 0.914 84.071 1.403 77.621
ENSMUSG00000005237 NA NA NA NA 1.191 95.809 0.868
60.96 0.848 85.493
ENSMUSG00000005251 Ripk4 protein_coding 0.977 83.418 0.898
55.265 1.14 20.315 0.997 95.74
ENSMUSG00000005262 Ufd1l protein_coding 0.923 6.484 0.942 86.538
1.145 6.202 1.104 48.119
ENSMUSG00000005267 Zfp287 protein_coding 0.88 32.378
1.133 83.104 1.007 79.897 1.036 95.273
ENSMUSG00000005268 Prlr protein_coding 0.922 53.679 0.847
79.475 1.276 43.185 0.915 86.699
ENSMUSG00000005299 Letm1 protein_coding 0.886 0.987 1.059 87.609
1.068 25.966 1.004 88.797
ENSMUSG00000005312 Ubqln1 protein_coding 0.953 37.531
0.975 97.155 1.074 55.109 0.982 94.654
ENSMUSG00000005320 Fgfr4 protein_coding 0.829 23.556 1.067
93.863 1.132 43.185 1.311 72.826
ENSMUSG00000005338 Cadm3 protein_coding 1.068 34.439 1.012
94.367 0.922 46.32 1.04 93.722
ENSMUSG00000005339 Fcer1a protein_coding 1.538 16.959
0.706 82.848 0.916 54.521 1.091 96.071
ENSMUSG00000005354 Txn2 protein_coding 0.872 2.009 1.081 71.617
1.059 39.471 1.092 83.067
ENSMUSG00000005355 Casp14 protein_coding 1 84.263 1
97.288 1 80.634 1 92.748
ENSMUSG00000005357 Slc1a6 protein_coding 1.67 81.178

1.415 92.793 0.424 57.091 1.057 96.183
ENSMUSG00000005360 Slc1a3 protein_coding 0.884 64.014 1.27
93.707 0.893 73.531 0.952 85.493
ENSMUSG00000005362 Crbn protein_coding 1.026 58.38 0.969 75.345
1.001 68.643 0.97 87.735
ENSMUSG00000005364 Il5ra protein_coding 2.272 39.075 0.488
74.633 0.899 82.161 0.849 91.335
ENSMUSG00000005370 Msh6 protein_coding 1.043 45.096 0.948
93.654 1.011 75.637 1.009 93.612
ENSMUSG00000005371 Fbxo11 protein_coding 1.096 5.253 1.002
92.062 0.911 1.58 0.962 87.735
ENSMUSG00000005373 Mlxipl protein_coding 0.809 27.202
1.365 60.309 0.907 32.082 1.094 74.193
ENSMUSG00000005374 Tbl2 protein_coding 0.914 46.693 1.102
71.222 0.994 80.163 1.032 84.31
ENSMUSG00000005378 Wbscr22 protein_coding 0.881 1.031 1.026
80.775 1.104 17.412 1.039 91.011
ENSMUSG00000005397 Nid1 protein_coding 0.913 56.477 0.94
78.51 1.161 53.134 1.128 59.441
ENSMUSG00000005410 Mcm5 protein_coding 1.192 47.847 1.139
90.895 0.737 60.233 1.598 31.133
ENSMUSG00000005413 Hmox1 protein_coding 1.289 0.435 0.899 72.399
0.859 9.746 1.293 43.377
ENSMUSG00000005417 Mprip protein_coding 0.913 3.009 0.93 69.611
1.175 10.819 0.973 83.724
ENSMUSG00000005442 Cic protein_coding 0.986 76.968 1.031
91.776 0.983 69.445 0.986 88.505
ENSMUSG00000005447 Pafah1b3 protein_coding 0.969 57.133
0.948 91.361 NA NA 0.919 85.493
ENSMUSG00000005465 Il27ra protein_coding 1.167 47.174
0.968 96.843 0.882 23.607 1.169 54.153
ENSMUSG00000005469 Prkaca protein_coding 0.913 14.427
1.104 71.617 NA NA 0.943 95.405
ENSMUSG00000005470 Asf1b protein_coding 1.272 17.582 1.031
92.404 0.764 49.453 1.655 40.894
ENSMUSG00000005474 Myl10 protein_coding 1 77.291 1
97.288 1 74.853 1 91.872
ENSMUSG00000005481 Ddx39 protein_coding 0.959 33.745 1.003
87.604 1.033 72.148 1.026 67.691
ENSMUSG00000005483 Dnajb1 protein_coding 0.957 77.407
1.181 83.104 0.886 42.895 1.03 86.209
ENSMUSG00000005501 Usp40 protein_coding 1.034 62.574 0.92
60.678 1.05 43.842 1.001 93.045
ENSMUSG00000005503 Evx1 protein_coding 1 77.407 NA NA
NA NA 1 96.358
ENSMUSG00000005505 Kbtbd4 protein_coding 1.03 41.454
1.002 93.994 0.969 18.539 1.034 92.381
ENSMUSG00000005506 Celf1 protein_coding 1.043 41.454 1.014
95.809 0.943 52.658 0.98 96.287
ENSMUSG00000005510 Ndufs3 protein_coding 0.918 13.952
1.026 96.897 1.057 20.315 0.971 93.261
ENSMUSG00000005514 Por protein_coding 0.882 2.009 0.979 96.138
1.161 20.572 1.064 95.273
ENSMUSG00000005533 Igf1r protein_coding 0.981 83.169 0.991

93.094 1.032 77.17 0.993 84.29
ENSMUSG00000005534 Insr protein_coding 0.978 61.334 0.977
94.597 1.046 53.587 0.9 83.724
ENSMUSG00000005540 Fcer2a protein_coding 0.713 75.567
1.035 87.282 1.353 83.857 1.227 92.31
ENSMUSG00000005547 Cyp2a5 protein_coding 1 86.145 1
95.504 1 81.976 1 93.156
ENSMUSG00000005566 Trim28 protein_coding 1.055 12.855 NA
NA 0.939 11.116 1.039 84.246
ENSMUSG00000005575 Ube2m protein_coding 0.903 5.491 1.105 72.642
NA NA 1.008 92.762
ENSMUSG00000005580 Adcy9 protein_coding 1.083 10.391 1.005
95.708 0.919 16.719 0.955 83.724
ENSMUSG00000005583 Mef2c protein_coding 1.39 0.461 0.991 92.253
0.724 0.72 0.944 95.74
ENSMUSG00000005609 Ctr9 protein_coding 0.82 18.487 1.152
60.309 1.061 45.94 0.994 85.786
ENSMUSG00000005610 Eif4g2 protein_coding 1.02 58.709 1.03
87.185 0.949 20.751 1.042 78.379
ENSMUSG00000005611 Mrvi1 protein_coding 1.262 0.984 1.098 83.104
0.721 0.72 1.01 91.872
ENSMUSG00000005615 Pcyt1a protein_coding 1.054 6.958 1.078
72.642 0.879 0.72 1.107 31.133
ENSMUSG00000005621 Zfp592 protein_coding 1.153 20.942
0.956 95.088 0.909 21.331 1.005 94.904
ENSMUSG00000005625 Psmd4 protein_coding 0.878 0.987 1.018 93.529
1.115 5.964 1.123 49.239
ENSMUSG00000005628 Tmod4 protein_coding 2.829 8.537 1.205 82.904
0.294 0.72 0.897 96.248
ENSMUSG00000005640 Insrr protein_coding 1.504 34.439 1.243
85.756 0.533 15.63 0.99 82.85
ENSMUSG00000005649 Cabp5 protein_coding 1 80.408 1
94.848 1 84.308 1 92.381
ENSMUSG00000005656 Snx6 protein_coding 1.307 0 0.954 67.739
0.803 2.087 1.052 62.594
ENSMUSG00000005667 Mthfd2 protein_coding 0.896 52.726
1.112 83.464 1.006 81.032 1.23 43.377
ENSMUSG00000005672 Kit protein_coding 0.971 86.172 0.875
75.618 1.176 62.307 1.002 96.086
ENSMUSG00000005674 Tomm40l protein_coding 0.874 5.778 1.108
72.546 1.033 67.562 NA NA
ENSMUSG00000005677 Nr1i3 protein_coding 0.687 3.559 1.066 86.156
1.369 15.688 0.913 91.625
ENSMUSG00000005681 Apoa2 protein_coding 1.096 86.843 0.819
89.713 1.11 61.418 0.654 84.29
ENSMUSG00000005682 Pan2 protein_coding 0.962 26.77 0.974 90.378
1.068 30.645 0.958 83.724
ENSMUSG00000005683 Cs protein_coding 0.876 1.6 1.052 73.092
1.084 53.587 0.996 93.261
ENSMUSG00000005686 Ampd3 protein_coding 1.103 30.984 0.847
13.578 1.072 21.462 1.018 92.018
ENSMUSG00000005687 Bcas2 protein_coding 1.117 1.689 0.917 0
0.976 88.203 0.981 95.35
ENSMUSG00000005696 Sh2d1a protein_coding 1.108 47.174

1.145 96.68 0.789 59.922 0.564 83.724
ENSMUSG00000005698 Ctcf protein_coding 1.066 0.355 1.029 90.092
0.913 0.72 0.985 84.19
ENSMUSG00000005699 Pard6a protein_coding 0.95 47.266
0.941 90.287 1.112 43.586 1.007 96.358
ENSMUSG00000005705 Agrp protein_coding 0.653 56.477 0.712
90.287 2.149 41.6 1.606 90.463
ENSMUSG00000005716 Pvalb protein_coding 8.083 6.853 0.967 83.819
0.128 0.72 0.872 96.248
ENSMUSG00000005718 Tfap4 protein_coding 0.879 28.998 0.991
81.134 1.143 15.241 0.861 84.29
ENSMUSG00000005732 Ranbp1 protein_coding 0.995 76.968
1.049 83.104 0.96 54.037 1.011 67.169
ENSMUSG00000005763 Cd247 protein_coding 0.817 21.957 1.118
87.029 1.092 56.708 0.97 90.611
ENSMUSG00000005774 Rfx5 protein_coding 0.941 44.922 0.975
88.705 1.087 51.596 0.916 85.493
ENSMUSG00000005779 Psmb4 protein_coding 0.92 5.962 1.016 96.407
1.069 34.403 1.076 48.119
ENSMUSG00000005800 Mmp8 protein_coding 3.074 21.599 0.223
70.971 1.461 86.144 0.65 93.156
ENSMUSG00000005802 Slc30a4 protein_coding 1.111 0.577 0.977
85.418 0.922 9.901 1.038 95.877
ENSMUSG00000005803 Sqrdl protein_coding 1.081 17.336 0.987
82.848 0.94 49.967 0.951 85.493
ENSMUSG00000005804 Bloc1s6 protein_coding 1.062 6.683 0.987
82.947 0.952 48.133 1.004 96.071
ENSMUSG00000005813 Metap1 protein_coding 1.013 79.853
1.032 96.407 0.953 74.179 1.015 89.498
ENSMUSG00000005823 Gpr108 protein_coding 0.966 28.804
0.994 91.584 1.042 66.928 0.975 84.29
ENSMUSG00000005824 Tnfsf14 protein_coding 1.101 48.288
0.973 80.052 0.933 71.734 1.156 95.74
ENSMUSG00000005836 Gata6 protein_coding 0.967 81.52 NA NA
1.055 78.293 1.191 95.35
ENSMUSG00000005846 Rsl1d1 protein_coding 1.157 23.493
0.901 74.483 0.96 74.853 1.055 59.464
ENSMUSG00000005864 Cnga2 protein_coding 0.721 74.482 0.782
93.899 1.777 79.897 1.118 95.273
ENSMUSG00000005871 Apc protein_coding 1.041 75.196 0.941
94.48 1.017 87.744 1.137 92.018
ENSMUSG00000005873 Reep5 protein_coding 0.973 28.998 1.08
71.617 0.952 48.387 1.02 87.134
ENSMUSG00000005881 Ergic3 protein_coding 0.913 33.415 0.99
84.594 1.108 28.055 1.022 96.248
ENSMUSG00000005882 Uqcc1 protein_coding 0.938 52.258 NA NA
1.061 61.171 1.056 83.067
ENSMUSG00000005883 Spo11 protein_coding 1.435 86.199 0.784
85.075 0.885 69.315 0.589 83.724
ENSMUSG00000005886 Ncoa2 protein_coding 0.963 25.85 1.03 86.793
1.008 69.315 0.984 85.493
ENSMUSG00000005891 Prl4a1 protein_coding 1 84.397 NA
NA 1 82.74 1 92.466
ENSMUSG00000005893 Nr2c2 protein_coding 0.95 44.787 0.956

69.611	1.096	10.76	1	94.567
ENSMUSG00000005897	Nr2c1	protein_coding	1.057	15.85	1.012	96.763
0.933	15.904	1.012	96.287
ENSMUSG00000005899	Smpd4	protein_coding	1.006	78.054	0.983
96.013	1.011	83.811	1.04	62.918
ENSMUSG00000005907	Pex1	protein_coding	0.958	43.816	0.966
88.704	1.078	25.455	1.02	83.067
ENSMUSG00000005936	Kctd20	protein_coding	1.07	21.278	NA
NA	0.937	18.539	0.958	87.735
ENSMUSG00000005947	Itgae	protein_coding	0.841	65.106	1.214
92.062	0.98	84.724	0.987	96.183
ENSMUSG00000005949	Ctns	protein_coding	1.292	0.2	NA	NA
0.758	0.945	1.074	83.276
ENSMUSG00000005950	P2rx5	protein_coding	0.898	60.003	1.097
85.756	NA	NA	0.874	84.29
ENSMUSG00000005951	Shpk	protein_coding	1.01	86.112	1.061
82.904	0.931	22.009	0.986	96.287
ENSMUSG00000005952	Trpv1	protein_coding	0.835	23.641	1.339
60.309	0.892	83.811	0.934	95.405
ENSMUSG00000005958	Ephb3	protein_coding	0.772	13.062	1.166
86.793	1.111	29.777	0.916	95.877
ENSMUSG00000005968	Tuft1	protein_coding	1.325	14.966	1.068
95.504	0.705	42.376	1.022	93.964
ENSMUSG00000005973	Rcn1	protein_coding	1.082	46.097	0.938
93.094	0.981	68.584	1.164	38.2
ENSMUSG00000005980	Dnase1	protein_coding	0.67	11.128
1.201	93.146	1.247	26.978	0.922	92.31
ENSMUSG00000005981	Trap1	protein_coding	0.953	10.955	0.988
96.216	1.058	18.565	1.029	86.445
ENSMUSG00000005982	Naa60	protein_coding	0.922	60.707	1.103
81.039	0.982	73.531	1.123	48.177
ENSMUSG00000005983	1700037C18Rik	protein_coding	0.93	41.759
0.958	93.908	1.122	39.471	0.847	83.724
ENSMUSG00000005986	Ankrd13d	protein_coding	0.853	16.02	1.013
96.847	1.158	20.968	0.728	83.724
ENSMUSG00000005994	Tyrp1	protein_coding	1.316	30.497	0.537
74.851	1.412	62.307	1.374	95.273
ENSMUSG00000006005	Tpr	protein_coding	0.946	72.985	0.947
62.71	1.115	30.292	1.101	51.967
ENSMUSG00000006010	BC003331	protein_coding	1.076	51.737
0.939	61.883	0.991	60.104	1.047	64.513
ENSMUSG00000006014	Prg4	protein_coding	1.356	2.352	0.689	35.072
1.072	80.015	1.01	96.358
ENSMUSG00000006019	Dhx34	protein_coding	0.932	38.827	0.981
95.504	1.089	28.979	0.98	94.001
ENSMUSG00000006021	Kptn	protein_coding	0.834	3.231	1.093	71.617
1.1	37.259	1.034	96.183
ENSMUSG00000006024	Napa	protein_coding	0.957	10.514	1.056
61.032	0.989	79.776	1.027	83.067
ENSMUSG00000006050	Sra1	protein_coding	0.849	12.785	1.117
72.642	1.057	55.512	1.099	72.616
ENSMUSG00000006056	Calcoco2	protein_coding	1	72.655	1
95.708	1	68.425	1	95.72
ENSMUSG00000006057	Atp5g1	protein_coding	1	75.399	1.14

71.617 0.875 7.339 1.077 58.721
ENSMUSG00000006058 Snf8 protein_coding 0.914 8.122 0.989 79.894
1.107 9.567 1.046 60.466
ENSMUSG00000006095 Tbcb protein_coding 0.815 1.488 1.054 80.806
1.158 13.709 1.112 46.412
ENSMUSG00000006127 Inpp5k protein_coding 1.102 20.659
0.931 88.705 0.976 53.224 0.973 85.493
ENSMUSG00000006134 Crkl protein_coding 1.091 1.412 1.03 88.092
0.891 0.72 0.993 91.372
ENSMUSG00000006154 Eps8l1 protein_coding 0.754 26.013
0.811 35.072 1.629 6.512 1.044 95.676
ENSMUSG00000006169 Clint1 protein_coding 1.15 0 0.938
84.017 0.923 20.511 1.018 87.447
ENSMUSG00000006179 Prss16 protein_coding 0.75 26.77 0.859
84.858 1.553 16.666 0.985 92.181
ENSMUSG00000006191 Cdkal1 protein_coding 1.165 0.435 0.995
95.504 0.86 2.294 1.016 95.74
ENSMUSG00000006200 Rhox6 protein_coding 1 68.955 1
95.708 1 86.346 1 93.722
ENSMUSG00000006204 5430419D17Rik protein_coding 0.426 40.217
NA NA 2.378 62.307 5.07 46.412
ENSMUSG00000006205 Htra1 protein_coding 1.194 11.316 1.007
92.062 0.834 15.169 1.182 53.923
ENSMUSG00000006215 Zbtb17 protein_coding 0.978 60.003
0.923 88.705 1.109 34.403 0.997 84.29
ENSMUSG00000006218 Fam131c protein_coding 0.821 64.589
1.082 95.088 1.128 58.414 0.847 83.952
ENSMUSG00000006219 Fblim1 protein_coding 1.044 45.307
1.038 85.756 0.921 15.778 0.948 84.29
ENSMUSG00000006221 Hspb7 protein_coding 1.636 20.799 0.903
95.708 0.677 44.019 1.16 78.042
ENSMUSG00000006235 Epor protein_coding 0.917 72.139 0.954
87.919 1.14 57.907 1.152 57.952
ENSMUSG00000006241 Ccdc159 protein_coding 1.015 62.888
1.033 85.501 0.954 77.294 0.912 86.699
ENSMUSG00000006262 Mob1b protein_coding 1.074 38.195 0.956
92.545 0.972 59.383 0.961 83.724
ENSMUSG00000006269 Atp6v1b1 protein_coding 0.772 9.081 1.066
93.27 1.213 36.95 0.988 86.699
ENSMUSG00000006273 Atp6v1b2 protein_coding 1.11 0 1.006
84.858 0.891 4.23 0.988 94.654
ENSMUSG00000006276 Eps15l1 protein_coding 0.936 7.218 1.045
95.431 1.018 34.285 1.062 61.984
ENSMUSG00000006281 Tep1 protein_coding 1.008 86.74 0.959 72.399
1.032 70.623 1.035 79.268
ENSMUSG00000006288 Ttc5 protein_coding 0.996 85.59 NA NA
1.015 88.601 0.953 86.991
ENSMUSG00000006289 Osgep protein_coding 0.974 75.567 1.076
79.864 0.956 46.956 1.005 96.139
ENSMUSG00000006299 Aamp protein_coding 0.901 0.987 NA NA
1.109 6.449 1.042 85.577
ENSMUSG00000006301 Tmbim1 protein_coding 1.058 16.124
0.914 61.883 1.038 88.203 0.96 83.724
ENSMUSG00000006304 Arpc2 protein_coding 1.113 0 1.013 96.216

0.887 0.72 1.192 42.736
ENSMUSG00000006307 Kmt2b protein_coding 0.912 1.031 1.038 83.104
1.058 13.082 0.951 83.952
ENSMUSG00000006310 Zbtb32 protein_coding 0.844 23.319
1.003 96.68 1.178 29.777 0.96 94.468
ENSMUSG00000006311 Etv2 protein_coding 1 67.867 1
96.268 1 88.729 1 95.35
ENSMUSG00000006313 Upk1a protein_coding 0.84 34.162 1.333
83.104 0.894 88.657 1.25 74.047
ENSMUSG00000006315 Tmem147 protein_coding 0.946 17.401
1.079 82.103 0.981 87.744 1.016 96.086
ENSMUSG00000006333 Rps9 protein_coding 0.971 81.01 0.952 21.315
1.082 16.808 1 92.181
ENSMUSG00000006335 Tfpt protein_coding 0.875 3.623 0.979 78.471
1.163 5.864 1.099 49.514
ENSMUSG00000006342 Susd2 protein_coding 1.61 0.2 0.896 86.538
0.695 1.898 0.871 86.445
ENSMUSG00000006344 Ggt5 protein_coding 1.223 19.893 0.869
72.399 0.942 58.516 1.114 65.175
ENSMUSG00000006345 Ggt1 protein_coding 1.03 81.01 0.851 60.678
1.139 44.787 1.128 76.347
ENSMUSG00000006356 Crip2 protein_coding 0.768 0.987 1.044 91.643
1.247 5.864 0.977 91.105
ENSMUSG00000006360 Crip1 protein_coding 1.188 0.984 0.98 93.654
0.858 19.236 1.203 31.133
ENSMUSG00000006362 Cbfa2t3 protein_coding 0.982 67.334
1.036 83.104 0.98 66.753 0.936 86.699
ENSMUSG00000006369 Fbln1 protein_coding 1.153 36.195 0.987
90.576 0.875 65.688 0.876 85.493
ENSMUSG00000006373 Pgrmc1 protein_coding 1.069 13.633
0.981 92.404 0.955 38.434 0.953 86.991
ENSMUSG00000006378 Gcat protein_coding 0.883 19.83 1.023 88.136
1.104 58.616 0.954 92.181
ENSMUSG00000006386 Tek protein_coding 1.142 16.74 0.87 78.877
1.004 85.21 0.894 83.724
ENSMUSG00000006389 Mpl protein_coding 0.658 68.353 1.702
91.643 0.889 88.161 0.711 87.705
ENSMUSG00000006390 Elovl1 protein_coding 1.004 84.294
1.037 83.104 0.96 49.453 1.001 74.896
ENSMUSG00000006392 Med8 protein_coding 0.936 65.869 1.083
89.494 NA NA 1.059 82.091
ENSMUSG00000006395 Hyi polymorphic_pseudogene 1.062 36.686
0.944 92.474 0.998 74.535 0.981 96.358
ENSMUSG00000006398 Cdc20 protein_coding 1.309 41.177 0.685
59.946 1.118 86.258 1.436 80.497
ENSMUSG00000006403 Adamts4 protein_coding 1.023 81.925
1.052 95.196 0.932 84.669 1.01 93.734
ENSMUSG00000006411 Pvrl4 protein_coding 0.885 13.74 0.918 89.996
1.228 16.26 0.901 84.29
ENSMUSG00000006412 Pfdn2 protein_coding 1.021 57.38 0.989 82.947
0.988 72.378 0.95 84.29
ENSMUSG00000006418 Rnf114 protein_coding 1.103 21.836
0.898 60.678 1.011 88.693 0.99 95.273
ENSMUSG00000006423 C330007P06Rik protein_coding 0.912 35.894

1.003 91.532 1.089 24.383 0.958 95.593
ENSMUSG00000006435 Neurl1a protein_coding 2.421 8.102 1.327
83.104 0.31 0.72 0.833 96.183
ENSMUSG00000006442 Srm protein_coding 0.884 64.893 1.088
82.904 NA NA 1.274 57.075
ENSMUSG00000006445 Epha2 protein_coding 0.802 1.703 1.067 82.809
1.163 7.855 1.049 68.502
ENSMUSG00000006456 Rbm14 protein_coding 1.184 4.825 0.973 83.104
0.869 1.898 1.036 90.397
ENSMUSG00000006457 Actn3 protein_coding 13.176 5.253 0.811
91.776 0.094 0.72 0.761 91.65
ENSMUSG00000006462 A530013C23Rik lincRNA 0.991 86.843
0.741 62.025 1.357 22.517 0.995 93.457
ENSMUSG00000006463 Zdhhc24 protein_coding 1.012 82.705 NA
NA 0.982 84.552 0.989 95.593
ENSMUSG00000006464 Bbs1 protein_coding 1.103 10.595 1.031
91.643 0.881 2.563 0.98 90.611
ENSMUSG00000006469 Slc34a3 protein_coding 0.377 11.519
1.782 71.98 1.492 69.825 1.212 96.248
ENSMUSG00000006471 Ndor1 protein_coding 0.894 1.449 1.01 97.263
1.108 7.635 0.996 92.762
ENSMUSG00000006476 Nsmf protein_coding 0.972 41.617 0.962
95.735 1.064 59.612 0.998 96.184
ENSMUSG00000006488 Prl7a1 protein_coding 1 82.543 1
95.504 NA NA 1 93.734
ENSMUSG00000006490 Prl8a9 protein_coding 1 76.042 1
87.313 1 87.274 1 91.372
ENSMUSG00000006494 Pdk1 protein_coding 0.827 2.559 0.929 79.807
1.302 15.602 0.909 90.611
ENSMUSG00000006498 Ptbp1 protein_coding 0.962 53.138 1.116
40.99 0.932 9.573 1.11 69.708
ENSMUSG00000006517 Mvd protein_coding 0.818 37.82 1.207 76.086
1.015 87.971 1.122 71.653
ENSMUSG00000006519 Cyba protein_coding 1.261 0.355 0.893 78.471
0.887 23.212 1.029 78.546
ENSMUSG00000006522 Itih3 protein_coding 3.221 6.23 0.516 85.671
0.6 37.617 0.797 90.691
ENSMUSG00000006526 Tmem110 protein_coding 0.877 4.825 1.026
96.984 1.113 14.449 0.988 91.372
ENSMUSG00000006527 Sfmbt1 protein_coding 1.043 29.057
0.997 85.95 0.962 75.353 1 96.071
ENSMUSG00000006538 Ihh protein_coding 0.621 43.167 1.258
96.68 1.282 66.043 0.895 96.287
ENSMUSG00000006542 Prkag3 protein_coding 2.503 14.205
1.559 81.127 0.256 2.687 0.857 95.559
ENSMUSG00000006546 Cryba2 protein_coding 1 83.823 1
96.847 1 82.778 1 90.397
ENSMUSG00000006567 Atp7b protein_coding 1.244 66.944 0.836
97.129 0.96 60.233 1.81 83.067
ENSMUSG00000006570 Defb2 protein_coding 1 80.688 1
96.407 1 79.325 1 96.183
ENSMUSG00000006574 Slc4a1 protein_coding 1.993 6.23 0.907
89.277 0.55 9.573 0.823 85.947
ENSMUSG00000006575 Rundc3a protein_coding 1.065 34.439

1.014 96.013 0.927 50.184 0.79 84.29
ENSMUSG00000006576 Slc4a3 protein_coding 0.961 77.763
1.001 90.712 1.039 56.125 1.018 84.31
ENSMUSG00000006585 Cdt1 protein_coding 1.05 68.16 0.956 86.071
0.991 84.626 1.282 57.075
ENSMUSG00000006586 Runx1t1 protein_coding 1.441 6.61 1.056
96.763 0.659 3.969 0.855 84.29
ENSMUSG00000006587 Snai3 protein_coding 1.002 85.832 1.188
95.735 0.841 68.82 1.849 91.335
ENSMUSG00000006589 Aprt protein_coding 1.091 33.099 0.93
84.017 0.987 74.179 1.119 64.513
ENSMUSG00000006599 Gtf2h1 protein_coding 1.075 21.539
0.954 53.434 0.977 76.388 1.04 72.826
ENSMUSG00000006611 Hfe protein_coding 0.883 3.848 1.059 79.411
1.065 35.894 0.941 92.561
ENSMUSG00000006638 Abhd1 processed_transcript 1.003 76.968
1.006 95.809 NA NA 0.887 83.724
ENSMUSG00000006641 Slc5a6 protein_coding 1.029 84.263 NA
NA 1.016 80.749 1.037 92.762
ENSMUSG00000006642 Tcf23 protein_coding 1.176 83.263 0.703
73.766 1.205 36.839 0.908 90.455
ENSMUSG00000006651 Aplp1 protein_coding 0.985 74.147 1.059
95.708 0.956 73.874 0.923 85.493
ENSMUSG00000006673 Qrich1 protein_coding 1.031 26.338
1.016 83.104 0.953 7.742 1.023 93.132
ENSMUSG00000006675 P4htm protein_coding 1.067 66 1.002 96.407
0.935 58.271 NA NA
ENSMUSG00000006676 Usp19 protein_coding 0.905 0.987 1.013 79.367
1.085 13.956 0.986 96.086
ENSMUSG00000006678 Pola1 protein_coding 1.107 26.136 1.055
96.897 0.854 23.938 1.19 44.679
ENSMUSG00000006699 Cdc42 protein_coding 1.011 83.263 1.026
83.104 0.966 39.755 1.069 31.133
ENSMUSG00000006705 Pknox1 protein_coding 1.079 25.207
1.134 85.756 0.818 19.236 1.033 78.379
ENSMUSG00000006711 D130043K22Rik protein_coding 0.642 82.891
0.482 84.594 3.23 53.587 4.417 83.865
ENSMUSG00000006715 Gmnn protein_coding 0.94 86.333 NA NA
NA NA 1.462 59.394
ENSMUSG00000006717 Acot13 protein_coding 1.019 78.054 NA
NA 0.986 77.704 0.983 91.625
ENSMUSG00000006720 Zfp184 protein_coding 1.138 71.896
0.989 95.504 0.885 87.744 0.729 84.19
ENSMUSG00000006724 Cyp27b1 protein_coding 0.732 77.407 0.98
88.897 1.395 48.451 1.468 63.708
ENSMUSG00000006728 Cdk4 protein_coding 0.959 44.467 0.969
88.691 1.073 28.979 1.024 68.696
ENSMUSG00000006731 B4galnt1 protein_coding 0.717 5.686 0.88
49.859 1.589 5.864 0.847 85.493
ENSMUSG00000006732 Mettl1 protein_coding 0.94 54.176
1.047 90.116 1.017 87.274 1.07 79.442
ENSMUSG00000006736 Tspan31 protein_coding 0.961 42.034
1.046 71.98 0.997 80.749 1.049 52.324
ENSMUSG00000006740 Kif5b protein_coding 0.963 24.344 0.969

96.68 1.073 58.414 1.078 61.753
ENSMUSG00000006763 Saal1 protein_coding 1.124 7.65 0.969 85.921
0.918 16.719 1.021 83.276
ENSMUSG00000006764 Tph2 protein_coding 0.356 14.075 1.707
84.768 1.649 71.615 1.36 88.755
ENSMUSG00000006777 Krt23 protein_coding 0.97 55.124 0.876
81.731 1.179 79.897 1.081 95.72
ENSMUSG00000006782 Cnp protein_coding 1.069 19.477 0.898
72.399 1.045 83.538 1.02 96.287
ENSMUSG00000006784 Ttc25 protein_coding 0.398 1.15 1.121 96.407
2.232 6.916 1.209 93.584
ENSMUSG00000006800 Sulf2 protein_coding 1.14 31.732 0.921
93.654 0.949 58.775 0.977 92.52
ENSMUSG00000006818 Sod2 protein_coding 0.902 2.822 1.064 71.617
1.042 72.148 1.11 89.498
ENSMUSG00000006850 Tmco6 protein_coding 0.879 5.311 1.009 94.89
1.123 20.968 1.03 92.018
ENSMUSG00000006906 Stambp protein_coding 1.038 43.344
1.007 94.848 0.957 46.071 0.957 96.183
ENSMUSG00000006920 Ezh1 protein_coding 1.148 1.009 0.961 64.123
0.903 13.848 1.011 90.611
ENSMUSG00000006930 Hap1 protein_coding 0.88 24.817 1.027
96.68 1.11 38.26 0.836 85.493
ENSMUSG00000006931 Leprel4 protein_coding 1.072 64.401
0.868 54.427 1.072 34.285 1.033 65.558
ENSMUSG00000006932 Ctnnb1 protein_coding 1.034 13.363
1.001 89.277 0.967 24.523 0.982 84.19
ENSMUSG00000006941 Eif1b protein_coding 1.08 15.972 1.072
75.108 0.865 1.061 1.041 85.577
ENSMUSG00000006958 Chrd protein_coding 0.869 11.232 1.017
97.288 1.134 34.727 0.972 94.608
ENSMUSG00000006998 Psmd2 protein_coding 0.907 3.559 1.019 93.899
1.079 15.411 1.129 40.024
ENSMUSG00000007021 Syngr3 protein_coding 0.859 61.633
0.854 92.545 1.357 39.471 0.897 94.016
ENSMUSG00000007029 Vars protein_coding 0.903 11.384 0.955
79.475 1.158 8.007 0.994 96.183
ENSMUSG00000007030 Vwa7 protein_coding 0.991 83.333 1.008
96.378 0.998 78.045 0.95 90.157
ENSMUSG00000007033 Hspa1l protein_coding 1.077 49.868
1.083 97.049 0.856 42.376 0.845 93.156
ENSMUSG00000007034 Slc44a4 protein_coding 1.197 51.826
1.001 89.277 0.835 66.201 1.144 79.268
ENSMUSG00000007035 Msh5 protein_coding 1.053 59.358 0.907
95.504 1.048 57.637 0.989 95.72
ENSMUSG00000007036 Abhd16a protein_coding 0.949 10.143
1.148 80.775 0.918 72.269 0.976 93.612
ENSMUSG00000007038 Neu1 protein_coding 1.081 7.553 0.916 62.322
1.009 88.274 0.952 84.29
ENSMUSG00000007039 Ddah2 protein_coding 1.049 44.694 NA NA
0.972 52.213 1.03 83.067
ENSMUSG00000007041 Clic1 protein_coding 1.082 7.174 0.939 39.547
0.986 85.21 1.138 31.133
ENSMUSG00000007050 Lsm2 protein_coding 1.021 86.351 1.075

82.904 0.909 34.21 1.042 83.067
ENSMUSG00000007080 Pole protein_coding 1.082 52.799 1.017
97.155 0.908 56.625 1.382 42.736
ENSMUSG00000007097 Atp1a2 protein_coding 0.883 30.035
1.007 95.301 1.123 62.648 1.031 95.273
ENSMUSG00000007107 Atp1a4 protein_coding 1.976 61.171
0.762 92.545 0.662 81.388 1.618 64.513
ENSMUSG00000007122 Casq1 protein_coding 6.431 8.685 1.194 85.756
0.13 0.72 0.987 95.676
ENSMUSG00000007207 Stx1a protein_coding 1.047 86.006 1.149
72.546 0.833 8.472 1 91.372
ENSMUSG00000007209 Ceacam9 protein_coding 1 75.399 NA
NA 1 88.461 1 93.457
ENSMUSG00000007216 Zfp775 protein_coding 1.026 68.16 0.944
79.475 1.031 78.864 1.017 96.183
ENSMUSG00000007279 Scube2 protein_coding 1.436 62.574
1.018 92.111 0.68 40.794 1.228 94.904
ENSMUSG00000007338 Mrpl49 protein_coding 0.896 53.679
1.099 83.104 1.016 87.519 1.084 51.901
ENSMUSG00000007379 Dennd2c protein_coding 1.069 37.702
1.133 87.029 0.826 9.456 1.105 76.697
ENSMUSG00000007411 Mark3 protein_coding 1.003 84.263 0.994
95.708 1.004 83.538 1.039 74.193
ENSMUSG00000007415 Gatad1 protein_coding 0.944 32.378
1.112 60.309 0.95 32.297 0.953 86.991
ENSMUSG00000007440 Pcdha2 protein_coding 1.121 64.401
0.825 92.545 1.079 81.032 1.073 89.052
ENSMUSG00000007457 2310003L06Rik protein_coding 1 43.167
1 90.68 1 84.724 1 92.018
ENSMUSG00000007458 M6pr protein_coding 1.064 12.72 1.009 96.763
0.93 14.206 0.987 96.248
ENSMUSG00000007476 Lrrc8a protein_coding 1.225 32.058
0.863 75.345 0.941 53.7 1.004 95.273
ENSMUSG00000007480 Mc5r protein_coding 1.024 82.891 1.023
95.088 0.953 78.155 0.745 91.372
ENSMUSG00000007564 Ppp2r1a protein_coding 0.956 7.881 1.031
71.617 1.017 83.719 1.061 81.56
ENSMUSG00000007570 Fance protein_coding 0.923 18.487 0.974
92.137 1.109 21.873 0.991 94.19
ENSMUSG00000007589 Tinf2 protein_coding 0.933 36.259 1.038
97.049 1.031 54.809 1.045 82.091
ENSMUSG00000007591 Tssk4 protein_coding 0.965 47.174 0.947
84.594 1.093 36.95 1.02 93.156
ENSMUSG00000007594 Hapln4 protein_coding 0.839 27.968
1.843 60.309 0.644 32.963 1.124 73.401
ENSMUSG00000007603 Dus3l protein_coding 0.836 0.987 1.03 92.111
1.161 5.864 1.051 91.525
ENSMUSG00000007610 Gtpbp3 protein_coding 0.981 64.284 0.99
90.287 1.03 35.673 0.973 94.468
ENSMUSG00000007613 Tgfbr1 protein_coding 1.028 59.527
1.056 82.904 0.92 15.376 0.957 84.19
ENSMUSG00000007617 Homer1 protein_coding 0.952 84.397
1.196 83.104 0.879 23.938 1.081 55.841
ENSMUSG00000007646 Rad51c protein_coding 1.317 41.177

0.995 82.073 0.764 78.045 1.099 60.466
ENSMUSG00000007653 Gabrb2 protein_coding 2.667 7.332 1.107
92.561 0.339 1.705 0.737 87.331
ENSMUSG00000007655 Cav1 protein_coding 0.827 1.698 1.085 72.546
1.113 27.89 1.046 72.116
ENSMUSG00000007656 Arpp19 protein_coding 0.998 56.907
1.023 96.335 0.976 36.248 1.003 95.74
ENSMUSG00000007659 Bcl2l1 protein_coding 1.016 74.482
0.792 20.174 1.245 10.281 1.125 78.546
ENSMUSG00000007670 Khsrp protein_coding 0.95 37.248 1.075
82.904 0.98 73.027 1.004 92.263
ENSMUSG00000007682 Dio2 protein_coding 1.335 28.693 0.945
96.847 0.794 39.245 1.356 93.612
ENSMUSG00000007721 Ccdc124 protein_coding 0.834 2.009 1.124
82.904 1.067 45.208 0.977 94.82
ENSMUSG00000007739 Cct4 protein_coding 1.065 25.026 0.915
47.716 1.026 32.524 1.02 60.31
ENSMUSG00000007777 0610009B22Rik protein_coding 1.008 76.546
0.975 95.088 1.015 84.83 0.96 86.991
ENSMUSG00000007783 Cpt1c protein_coding 1.107 10.595 0.991
92.931 0.908 2.889 1.077 78.948
ENSMUSG00000007805 Twist2 protein_coding 1.117 25.207
0.965 96.843 0.93 21.666 0.956 92.31
ENSMUSG00000007812 Zfp655 protein_coding 1.109 3.825 1.001
90.256 0.902 11.997 0.918 83.724
ENSMUSG00000007815 Rhoa protein_coding 1 84.294 1.006
92.793 0.993 75.637 1.113 48.119
ENSMUSG00000007817 Zmiz1 protein_coding 0.946 49.944 1.092
64.304 0.966 88.657 1.141 64.513
ENSMUSG00000007827 Ankrd26 protein_coding 1.047 73.734
1.058 82.904 0.904 33.137 1.019 92.018
ENSMUSG00000007833 Aldh16a1 protein_coding 0.928 48.354
0.959 78.51 1.124 34.403 1.032 85.629
ENSMUSG00000007836 Hnrnpa0 protein_coding 1.043 81.125
1.064 84.007 0.903 71.004 0.847 85.493
ENSMUSG00000007837 Prrg2 protein_coding 0.938 23.923 1.031
79.367 1.034 85.935 0.959 85.493
ENSMUSG00000007850 Hnrnph1 protein_coding 1.02 86.145
1.018 96.847 0.961 75.353 1.008 93.722
ENSMUSG00000007867 Ift43 protein_coding 0.955 38.827 0.945
90.053 1.106 47.935 1.073 67.691
ENSMUSG00000007872 Id3 protein_coding 0.855 18.731 1.028
96.407 1.135 24.985 1.15 51.967
ENSMUSG00000007877 Tcap protein_coding 4.288 12.396 1.197
86.34 0.195 1.871 1.023 95.273
ENSMUSG00000007880 Arid1a protein_coding 1.024 68.16 1.123
66.01 0.868 0.879 0.933 83.724
ENSMUSG00000007888 Crlf1 protein_coding 0.42 1.239 1.471 79.864
1.616 12.703 1.39 64.905
ENSMUSG00000007891 Ctsd protein_coding 0.806 1.274 0.945 96.843
1.313 6.506 1.071 32.698
ENSMUSG00000007892 Rplp1 protein_coding 0.958 78.299 1.083
85.756 0.961 59.922 1.051 89.052
ENSMUSG00000007907 Cabs1 protein_coding 1 83.263 1

95.301 1 84.896 1 95.676
ENSMUSG00000007908 Hmgcll1 protein_coding 0.971 78.639
1.049 96.407 0.985 73.874 0.898 87.496
ENSMUSG00000007946 Phox2a protein_coding 0.772 20.369
1.033 89.996 1.25 28.74 0.882 96.287
ENSMUSG00000007950 Abhd8 protein_coding 0.978 71.06 1.135 90.389
0.9 30.024 1.19 44.882
ENSMUSG00000007987 Rabl5 protein_coding 1.017 83.333 0.946
81.731 1.039 51.977 1.037 56.863
ENSMUSG00000007989 Fzd3 protein_coding 1.034 80.146 1.011
96.847 0.958 83.158 0.915 85.493
ENSMUSG00000008028 1700008O03Rik protein_coding 0.767 31.921
1.043 96.268 1.251 58.915 0.619 85.493
ENSMUSG00000008035 Mid1ip1 protein_coding 0.784 22.481
1.184 82.904 1.074 80.634 1.13 92.381
ENSMUSG00000008036 Ap2s1 protein_coding 0.918 16.652 1.058
71.617 1.033 83.857 1.062 64.513
ENSMUSG00000008090 Fgfrl1 protein_coding 0.88 2.174 1.038
82.904 1.096 23.604 0.88 91.372
ENSMUSG00000008129 4930432K21Rik protein_coding 0.851 65.199
NA NA 1.385 62.648 1.201 87.7
ENSMUSG00000008136 Fhl2 protein_coding 1.18 39.295 0.969
96.216 0.875 49.733 0.981 83.276
ENSMUSG00000008140 Emc10 protein_coding 0.872 13.239 1.088
83.104 1.055 60.5 0.991 87.651
ENSMUSG00000008153 Clstn3 protein_coding 0.787 3.688 1.235
80.806 1.025 83.275 0.963 86.699
ENSMUSG00000008167 Fbxw9 protein_coding 0.937 25.492 0.999
94.767 1.068 38.926 1.044 92.018
ENSMUSG00000008193 Spib protein_coding 0.696 39.712 1.796
64.304 0.801 38.799 1.05 93.156
ENSMUSG00000008200 Fnbp4 protein_coding 0.971 11.707 0.967
90.576 1.063 22.994 0.984 95.72
ENSMUSG00000008206 Cers4 protein_coding 0.898 19.365 1.013
95.431 1.1 42.522 1.137 71.305
ENSMUSG00000008226 Scrn3 protein_coding 1.012 78.909 0.955
94.48 1.035 59.274 1.02 60.31
ENSMUSG00000008301 Phax protein_coding 1.091 5.313 0.934 40.463
0.979 86.985 1.065 63.708
ENSMUSG00000008305 Tle1 protein_coding 0.868 57.544 1.023
95.35 1.126 41.235 1.029 96.086
ENSMUSG00000008307 1700109H08Rik protein_coding 0.798 38.286
0.911 54.06 1.371 6.506 1.034 92.561
ENSMUSG00000008318 Relt protein_coding 1.358 6.23 0.968 84.017
0.761 40.794 1.017 92.691
ENSMUSG00000008333 Snrpb2 protein_coding 1.192 0 0.905
34.058 0.928 23.212 1.009 93.734
ENSMUSG00000008348 Ubc protein_coding 0.81 8.365 0.921 77.944
1.344 5.864 1.024 76.831
ENSMUSG00000008373 Prpf31 protein_coding 0.949 75.567 1
84.017 1.055 39.439 1.094 44.679
ENSMUSG00000008384 Sertad1 protein_coding 0.845 51.089
1.169 82.904 1.009 68.584 0.989 92.561
ENSMUSG00000008393 Carhsp1 protein_coding 1.052 54.717

1.046 82.904 0.909 3.234 1.061 59.464
ENSMUSG00000008398 Elk3 protein_coding 1.03 68.16 1.126 79.29
0.864 11.751 0.956 87.651
ENSMUSG00000008429 Herpud2 protein_coding 1.045 21.146
1.033 88.092 0.926 4.338 0.925 83.724
ENSMUSG00000008435 Rdh13 protein_coding 1.058 57.655 0.93
71.889 1.011 79.554 1.02 83.067
ENSMUSG00000008438 Adam21 protein_coding 0.799 73.487
0.718 90.576 1.744 63.801 0.632 86.991
ENSMUSG00000008450 Nutf2 protein_coding 0.942 38.555 1.115
71.617 0.953 35.815 1.115 46.846
ENSMUSG00000008475 Arpc5 protein_coding 1.146 0.461 0.951 91.361
0.913 2.294 0.965 85.493
ENSMUSG00000008482 Rnf151 protein_coding 0.901 69.715
1.472 96.138 0.755 72.378 0.754 94.904
ENSMUSG00000008489 Elavl2 protein_coding 0.848 54.87 1.346
83.104 0.874 84.669 1.136 93.132
ENSMUSG00000008496 Pou2f2 protein_coding 0.972 50.913
1.166 94.848 0.883 59.125 0.965 86.445
ENSMUSG00000008540 Mgst1 protein_coding 1.145 6.958 0.901 92.631
0.968 30.468 0.974 96.358
ENSMUSG00000008575 Nfib protein_coding 0.934 29.725 0.999
96.335 1.072 76.225 1.009 94.904
ENSMUSG00000008590 Htr3b protein_coding 1 86.145 1
92.931 1 86.258 1 94.608
ENSMUSG00000008601 Rab25 protein_coding 0.86 45.728 0.831
38.699 1.399 18.411 1.12 93.612
ENSMUSG00000008604 Ubqln4 protein_coding 0.985 78.744
1.097 62.898 0.927 0.879 0.999 95.777
ENSMUSG00000008658 Rbfox1 protein_coding 8.536 7.397 1.671
92.561 0.07 0.72 0.638 95.593
ENSMUSG00000008668 Rps18 protein_coding 0.994 85.655 0.946
23.703 1.06 22.366 1.059 72.826
ENSMUSG00000008682 Rpl10 protein_coding 1.082 10.858 0.922
59.946 1.001 87.898 1.039 72.369
ENSMUSG00000008683 Rps15a protein_coding 1.111 27.731
0.887 40.946 1.018 83.066 1.089 49.769
ENSMUSG00000008690 Ncaph2 protein_coding 0.952 4.941 0.976
72.712 1.079 8.93 1.04 51.967
ENSMUSG00000008730 Hipk1 protein_coding 0.996 73.487 0.966
68.818 1.042 22.93 1.016 94.904
ENSMUSG00000008734 Gprc5b protein_coding 0.935 25.111
1.095 61.943 0.971 44.865 0.976 90.289
ENSMUSG00000008763 Man1a2 protein_coding 1.06 22.205
0.962 53.79 0.976 76.768 1.017 89.498
ENSMUSG00000008789 Ceacam5 protein_coding 1 84.661 1
95.708 NA NA 1 94.904
ENSMUSG00000008822 Acyp1 protein_coding 0.97 49.047 0.979
85.418 1.051 37.764 0.938 94.904
ENSMUSG00000008845 Cd163 protein_coding 1.024 65.733 0.826
30.872 1.183 34.285 1.127 80.497
ENSMUSG00000008855 Hdac5 protein_coding 1.013 78.054 1.014
90.29 0.971 45.799 1.02 86.991
ENSMUSG00000008859 Rala protein_coding 0.976 28.514 1.056

75.612 0.966 60.96 1.017 59.394
ENSMUSG00000008874 Clec3a protein_coding 1 86.006 1
94.848 1 87.898 1 95.74
ENSMUSG00000008892 Vdac3 protein_coding 1.016 56.311 0.96
94.848 1.023 71.999 1.138 36.988
ENSMUSG00000008932 Slc1a7 protein_coding 1.864 4.874 1.045
97.155 0.515 18.539 0.627 84.29
ENSMUSG00000008958 Vps72 protein_coding 0.926 17.062 1.051
95.735 1.03 43.481 0.98 84.29
ENSMUSG00000008976 Gabpa protein_coding 1.121 1.161 0.965 74.483
0.921 2.563 0.959 83.724
ENSMUSG00000008999 Bmp7 protein_coding 0.756 6.34 1.232 71.617
1.069 64.305 1.126 84.31
ENSMUSG00000009013 Dynll1 protein_coding 1.011 50.377
1.128 72.642 0.878 6.751 1.015 93.156
ENSMUSG00000009030 Pdcl protein_coding 1.028 83.169 1.014
83.104 0.96 51.055 0.933 83.724
ENSMUSG00000009035 Tmem184b protein_coding 0.972 75.567
1.181 60.309 0.868 7.742 0.98 90.805
ENSMUSG00000009047 Gm5965 protein_coding 1 85.857 1
96.216 1 80.163 1 91.783
ENSMUSG00000009070 Rtdr1 protein_coding 1 81.01 1 96.652
1 83.719 1 90.674
ENSMUSG00000009073 Nf2 protein_coding 0.944 11.726 1.064
71.98 0.999 83.925 1.02 89.052
ENSMUSG00000009075 Cabp7 protein_coding 0.989 75.858 0.906
95.504 1.116 55.109 0.795 86.991
ENSMUSG00000009076 Zmat5 protein_coding 0.896 37.531 0.963
96.216 1.161 29.568 1.162 51.901
ENSMUSG00000009079 Ewsr1 protein_coding 0.981 55.641 0.952
43.505 1.072 5.864 1.026 63.708
ENSMUSG00000009090 Ap1b1 protein_coding 1.035 37.702 0.967
89.996 0.996 85.134 1.047 83.067
ENSMUSG00000009092 Derl3 protein_coding 1.193 15.456 0.984
95.088 0.85 27.86 0.98 87.651
ENSMUSG00000009093 Gstt4 protein_coding 1.326 81.178 1.014
96.68 0.744 63.919 0.827 85.493
ENSMUSG00000009097 Tbx1 protein_coding 1.259 13.557 1.112
97.263 0.716 19.765 0.88 86.699
ENSMUSG00000009108 Gnat2 protein_coding 0.67 62.888 1.331
92.062 1.12 83.925 1.352 83.067
ENSMUSG00000009112 Bcl2l13 protein_coding 0.946 38.827
1.034 90.29 1.017 56.497 1.036 86.209
ENSMUSG00000009114 2610028H24Rik protein_coding 1.054 76.135
1.283 89.494 0.741 71.734 0.511 83.724
ENSMUSG00000009115 Spatc1l protein_coding 0.716 52.9 0.774
90.053 1.801 60.104 2.236 83.067
ENSMUSG00000009145 Dqx1 protein_coding 0.919 34.795 0.917
83.321 1.188 24.07 1.054 90.157
ENSMUSG00000009185 Ccl8 protein_coding 1.496 0.461 NA NA
0.592 13.848 1.457 44.213
ENSMUSG00000009207 Lnp protein_coding 1.179 10.464 0.968
95.504 0.876 15.376 1.045 82.85
ENSMUSG00000009210 2310007L24Rik protein_coding 1.681 19.746

0.85 91.361 0.7 31.676 0.7 94.297
ENSMUSG00000009214 Tmem8c protein_coding 1 78.299 1
96.68 1 84.896 1 96.071
ENSMUSG00000009246 Trpm5 protein_coding 0.918 58.077 0.782
95.708 1.394 50.091 1.019 96.183
ENSMUSG00000009248 Ascl2 protein_coding 1.441 9.259 0.851 79.261
0.811 39.755 1.165 77.331
ENSMUSG00000009281 Rarres2 protein_coding 1 75.298
0.943 90.256 1.061 53.134 0.991 92.381
ENSMUSG00000009291 Pttg1ip protein_coding 0.986 84.564
1.011 96.407 1.005 87.744 1.027 78.948
ENSMUSG00000009292 Trpm2 protein_coding 0.969 75.567 0.915
81.731 1.122 50.636 1.027 74.193
ENSMUSG00000009293 Ube2g2 protein_coding 0.973 83.333
0.966 85.418 1.065 56.497 1.121 32.698
ENSMUSG00000009356 Lpo protein_coding 1.7 68.727 0.504
90.138 1.169 87.744 0.452 84.19
ENSMUSG00000009376 Met protein_coding 0.915 21.957 1.038
88.092 1.052 65.567 0.885 86.991
ENSMUSG00000009378 Slc16a12 protein_coding 0.808 26.013
0.907 90.744 1.358 30.645 1.311 54.265
ENSMUSG00000009394 Syn2 protein_coding 0.416 25.111 1.939
71.98 1.242 41.235 4.821 40.024
ENSMUSG00000009406 Elk1 protein_coding 0.89 1.274 1.115 61.943
1.011 75.009 1.017 95.273
ENSMUSG00000009418 Nav1 protein_coding 1.043 72.372 0.918
73.766 1.039 73.759 0.977 88.505
ENSMUSG00000009470 Tnpo1 protein_coding 1.021 82.231 0.893
64.123 1.098 31.761 1.027 95.832
ENSMUSG00000009487 Otog protein_coding 1 85.129 1
92.561 1 83.158 1 92.181
ENSMUSG00000009535 Rnmt protein_coding 0.979 79.709 0.955
55.265 1.071 9.301 NA NA
ENSMUSG00000009545 Kcnq1 protein_coding 1.203 17.761 0.983
87.282 0.846 8.626 0.907 92.181
ENSMUSG00000009549 Srp14 protein_coding 0.93 1.867 0.99 87.452
1.081 6.506 1.041 42.736
ENSMUSG00000009551 6330409D20Rik protein_coding 0.685 22.237
1.025 97.129 1.422 39.471 0.933 85.493
ENSMUSG00000009555 Cdk9 protein_coding 1.005 81.196 NA NA
0.988 85.21 0.971 85.493
ENSMUSG00000009563 Tor2a protein_coding 1.004 78.744 1.056
81.039 0.943 39.755 0.954 88.505
ENSMUSG00000009566 Fpgs protein_coding 0.914 27.836 0.92
85.116 1.188 10.819 1.065 74.896
ENSMUSG00000009569 Mkl2 protein_coding 1.069 76.042 0.888
66.09 1.054 43.129 0.982 94.153
ENSMUSG00000009575 Cbx5 protein_coding 0.966 64.589 1.068
82.904 0.973 53.224 1.137 90.674
ENSMUSG00000009580 Odam protein_coding 1 84.977 1
85.418 1 46.575 1 94.904
ENSMUSG00000009585 Apobec3 protein_coding 0.889 5.946 0.95
90.378 1.185 5.964 0.913 83.724
ENSMUSG00000009596 Taf7l protein_coding 1 57.227 NA NA

							1	82.778	1	96.184
ENSMUSG00000009614	Sardh	protein_coding	0.84	0.987	1.087	71.617	1.095	21.201	1.148	42.736
ENSMUSG00000009621	Vav2	protein_coding	1.087	10.687	0.952	85.793	0.967	25.688	0.998	88.505
ENSMUSG00000009628	Tex15	protein_coding	0.984	70.862	0.888	95.088	1.148	67.823	0.68	90.805
ENSMUSG00000009630	Ppp2cb	protein_coding	1.012	64.193	0.939	72.399	1.049	72.378	1.06	90.674
ENSMUSG00000009633	G0s2	protein_coding	0.85	35.646	1.858	60.309	0.632	4.963	0.978	87.7
ENSMUSG00000009640	Dmap1	protein_coding	0.964	40.786	0.959	93.817	1.083	27.732	1.023	86.209
ENSMUSG00000009646	Pla2g12b	protein_coding	1	84.819	1	96.897	1	69.583	1	96.287
ENSMUSG00000009647	Mcu	protein_coding	1.104	28.693	1.023	91.643	0.883	17.258	1.074	43.377
ENSMUSG00000009654	Oit3	protein_coding	1.414	35.499	1.352	96.68	0.522	27.86	1.068	95.72
ENSMUSG00000009670	Tex11	protein_coding	1.221	78.299	0.672	90.256	1.22	74.325	0.87	76.697
ENSMUSG00000009681	Bcr	protein_coding	1.049	32.873	0.989	95.088	0.966	25.688	0.951	83.724
ENSMUSG00000009687	Fxyd5	protein_coding	1.141	7.65	0.98	95.504	0.893	10.726	1.199	50.452
ENSMUSG00000009731	Kcnd1	protein_coding	1.177	7.908	1.059	90.092	0.801	3.863	1.275	32.698
ENSMUSG00000009733	Tfcp2	protein_coding	0.995	84.049	0.983	95.941	NA	NA	0.952	85.493
ENSMUSG00000009739	Pou6f1	protein_coding	1.018	67.608	0.919	93.094	1.07	80.015	0.989	86.699
ENSMUSG00000009741	Ubp1	protein_coding	0.94	12.624	0.993	96.984	1.068	36.839	1.021	89.152
ENSMUSG00000009772	Nuak2	protein_coding	1.024	63.385	0.808	34.058	1.211	44.515	1.186	55.841
ENSMUSG00000009828	Ick	protein_coding	1.069	19.132	0.975	82.671	0.958	76.388	0.996	95.74
ENSMUSG00000009863	Sdhb	protein_coding	0.877	2.5	1.099	71.617	1.036	58.616	0.974	89.525
ENSMUSG00000009876	Cox4i2	protein_coding	0.89	44.922	1.58	71.617	0.713	49.733	0.87	84.29
ENSMUSG00000009894	Snap47	protein_coding	0.949	21.637	1.057	72.546	0.992	78.642	1.037	77.331
ENSMUSG00000009900	Wnt3a	protein_coding	1	83.418	1	96.407	1	86.084	1	96.192
ENSMUSG00000009905	Kdsr	protein_coding	1.134	1.161	0.988	97.155	0.896	1.92	0.914	75.345
ENSMUSG00000009907	Vps4b	protein_coding	1.038	14.59	0.97	60.678	0.99	84.83	0.954	86.699
ENSMUSG00000009927	Rps25	protein_coding	0.92	38.827	1.033	96.268	1.051	31.975	1.048	83.067
ENSMUSG00000009941	Nxf2	protein_coding	1.248	30.984	1.81	93.574	0.441	14.262	0.623	85.493
ENSMUSG00000009995	Taz	protein_coding	0.929	1.727	0.952	62.322				

1.133 5.864 0.971 86.445
ENSMUSG00000010021 Kif19a protein_coding 1.015 81.01 0.897
93.654 1.093 74.325 1.078 60.466
ENSMUSG00000010025 Aldh3a2 protein_coding 1.03 61.171
0.979 90.046 0.992 77.704 1.039 80.77
ENSMUSG00000010044 Zmynd10 protein_coding 0.869 49.047 1.47
77.085 0.782 47.641 0.801 90.397
ENSMUSG00000010045 Tmem115 protein_coding 0.93 12.181
1.048 82.904 1.023 58.414 0.982 91.979
ENSMUSG00000010047 Hyal2 protein_coding 1.009 67.991 1.029
88.092 0.964 25.052 1.018 93.584
ENSMUSG00000010048 Ifrd2 protein_coding 0.872 55.641 1.065
83.104 1.074 74.325 1.2 44.882
ENSMUSG00000010051 Hyal1 protein_coding 1.102 55.462 1.084
71.617 0.834 1.898 1.063 48.119
ENSMUSG00000010054 Tusc2 protein_coding 0.919 9.081 1.135 40.99
0.959 29.765 0.98 93.964
ENSMUSG00000010057 Nprl2 protein_coding 0.89 18.731 1.147
72.546 NA NA 1.061 67.691
ENSMUSG00000010064 Slc38a3 protein_coding 0.904 57.544
1.507 67.843 0.729 23.607 1.318 69.918
ENSMUSG00000010066 Cacna2d2 protein_coding 0.821 44.787 0.68
79.894 1.785 28.979 1.592 78.948
ENSMUSG00000010067 Rassf1 protein_coding 0.957 28.584
1.061 79.055 0.986 65.902 1.037 65.558
ENSMUSG00000010080 Epn3 protein_coding 0.866 39.953 0.839
42.923 1.373 16.446 0.87 88.245
ENSMUSG00000010095 Slc3a2 protein_coding 1.217 4.825 0.924
79.475 0.887 41.506 0.904 85.493
ENSMUSG00000010097 Nxf1 protein_coding 0.825 1.15 1.076 83.104
1.127 9.301 1.049 50.452
ENSMUSG00000010110 Stx5a protein_coding 0.942 8.614 1.06 72.642
1.003 84.229 0.971 86.699
ENSMUSG00000010122 Slc47a1 protein_coding 1.073 83.418
0.622 93.654 1.494 71.232 1.464 83.067
ENSMUSG00000010142 Tnfrsf13b protein_coding 1.607 0.355 0.848
71.98 0.736 4.759 1.209 86.133
ENSMUSG00000010154 Spire2 protein_coding 1.024 78.428 0.95
88.897 1.029 83.066 1.107 80.187
ENSMUSG00000010175 Prox1 protein_coding 1.141 29.536 1.209
85.756 0.726 13.551 1.004 95.74
ENSMUSG00000010205 Raver1 protein_coding 0.995 82.891
1.075 72.642 0.936 22.735 1.015 80.324
ENSMUSG00000010277 2610507B11Rik protein_coding 1.067 10.687
1.038 83.104 0.902 1.342 1.018 93.261
ENSMUSG00000010290 AI597479 protein_coding 1.101 10.595
1.024 88.923 0.887 1.342 0.965 90.611
ENSMUSG00000010307 Tmem86a protein_coding 1.208 5.313 0.898
73.461 0.922 56.822 1.345 86.301
ENSMUSG00000010311 Optc protein_coding 0.846 43.435 1.117
91.643 1.055 58.616 1.198 70.539
ENSMUSG00000010342 Tex14 protein_coding 1.552 15.972 0.781
66.651 NA NA 1.031 81.061
ENSMUSG00000010358 Ifi35 protein_coding 1.069 64.193 1.069

82.904	0.876	24.523	1.154	57.1
ENSMUSG00000010362	Rdm1	protein_coding	1.052	11.197	0.898
49.859	1.055	82.74	0.878	83.724
ENSMUSG00000010376	Nedd8	protein_coding	0.886	5.962	NA	NA
1.093	15.602	1.108	48.119
ENSMUSG00000010392	Gosr1	protein_coding	1.057	10.391	0.995
88.705	0.951	30.468	0.963	83.724
ENSMUSG00000010406	Mrpl52	protein_coding	0.914	40.422	1
95.301	1.093	38.099	1.118	63.708
ENSMUSG00000010435	Spaca7	protein_coding	1	83.418	1
96.001	1	82.903	1	96.086
ENSMUSG00000010453	Kansl3	protein_coding	1.006	64.714
1.002	97.155	0.99	61.743	1.006	95.273
ENSMUSG00000010461	Eya4	protein_coding	1.809	1.828	0.914	95.301
0.604	1.705	0.833	94.19
ENSMUSG00000010476	Ebf3	protein_coding	0.876	26.77	1.084	79.29
1.048	85.921	1.176	79.713
ENSMUSG00000010492	Gm16119	antisense	0.725	10.955	1.313
83.104	1.049	57.637	0.9	96.183
ENSMUSG00000010505	Myt1	protein_coding	0.873	86.333	0.704
73.766	1.631	32.776	0.497	87.331
ENSMUSG00000010517	Faf1	protein_coding	0.951	76.686	0.973
77.598	1.075	30.561	1.093	38.624
ENSMUSG00000010529	Gm266	protein_coding	0.83	30.877	0.826
85.116	1.46	20.085	1.037	93.457
ENSMUSG00000010538	Tsacc	protein_coding	1.013	85.355	0.954
96.268	1.033	86.258	0.81	86.699
ENSMUSG00000010554	Mettl16	protein_coding	1.012	72	1.102
87.609	0.896	52.087	0.982	93.734
ENSMUSG00000010601	Apol7a	protein_coding	1.454	56.907
0.644	62.322	1.07	52.8	0.788	86.991
ENSMUSG00000010607	Pigyl	protein_coding	0.909	9.599	1.017	82.904
1.079	59.612	0.958	84.29
ENSMUSG00000010608	Rbm25	protein_coding	0.947	8.139	0.984	60.678
1.073	5.864	0.997	83.067
ENSMUSG00000010609	Psen2	protein_coding	1.011	74.482	1.103
79.411	0.899	50.184	0.87	83.724
ENSMUSG00000010651	Acaa1b	protein_coding	1.002	86.006
0.829	91.532	1.204	79.625	0.733	83.724
ENSMUSG00000010660	Plcd1	protein_coding	0.894	11.726	1.107
79.411	1.013	56.931	0.979	95.593
ENSMUSG00000010663	Fads1	protein_coding	1.066	46.875	1.139
60.309	0.825	2.563	0.956	96.192
ENSMUSG00000010721	Lmbr1	protein_coding	1.13	0.976	0.975	79.261
0.904	8.313	0.977	85.636
ENSMUSG00000010751	Tnfrsf22	protein_coding	1.36	9.435	1.068
86.156	0.689	3.943	0.833	84.29
ENSMUSG00000010755	Cars	protein_coding	0.883	3.325	1	96.216
1.129	11.518	1.109	0
ENSMUSG00000010760	NA	NA	NA	NA	0.625	90.046	1.3
66.647	0.817	84.29
ENSMUSG00000010796	Asz1	protein_coding	1	84.977	1
94.887	NA	NA	1	93.722
ENSMUSG00000010797	Wnt2	protein_coding	1.338	15.178	1.039

95.504 0.717 60.96 1.043 96.183
ENSMUSG00000010825 Grid2ip protein_coding 0.895 68.846
1.269 91.584 0.881 85.757 1.215 72.204
ENSMUSG00000010830 Kdelr3 protein_coding 1.381 8.365 1.026
90.092 0.706 1.58 1.192 44.882
ENSMUSG00000010911 Apip protein_coding 0.864 13.74 1.012 96.216
1.142 24.791 1.061 80.573
ENSMUSG00000010914 Pdhx protein_coding 0.836 1.698 1.014 96.378
1.179 16.666 1.014 95.676
ENSMUSG00000010936 Vac14 protein_coding 0.929 30.136 1.061
81.54 1.01 81.388 1.077 71.653
ENSMUSG00000011008 Mcoln2 protein_coding 2.391 0.2 0.817
90.712 0.512 1.061 1.212 59.394
ENSMUSG00000011034 Slc5a1 protein_coding 1.007 86.351
0.982 90.389 1.009 69.583 0.858 83.724
ENSMUSG00000011052 1700049J03Rik antisense 1 77.497 1
88.897 1 68.129 1 94.016
ENSMUSG00000011096 Akt1s1 protein_coding 0.909 16.472
1.101 82.904 0.996 77.888 0.942 90.611
ENSMUSG00000011114 Tbrg1 protein_coding 1.004 75.196 1.023
90.744 0.969 71.999 1.084 46.846
ENSMUSG00000011148 Adssl1 protein_coding 2.162 6.281 0.994
96.216 0.464 1.705 0.888 94.904
ENSMUSG00000011154 1700026D08Rik protein_coding 1.03 52.429
0.674 82.671 1.437 79.554 2.155 60.31
ENSMUSG00000011158 Brf1 protein_coding 0.985 85.203 0.97
75.993 1.049 45.208 1.006 92.748
ENSMUSG00000011171 Vipr2 protein_coding 1.347 32.676 0.731
61.883 1.018 87.971 1.117 93.964
ENSMUSG00000011179 Odc1 protein_coding 1.056 35.499 1.031
93.574 0.915 36.499 1.176 40.024
ENSMUSG00000011254 Thg1l protein_coding 1.022 81.24 1.009 96.216
0.968 82.685 0.99 92.263
ENSMUSG00000011256 Adam19 protein_coding 1.247 14.966
1.005 97.263 0.796 23.212 0.885 83.952
ENSMUSG00000011257 Pabpc4 protein_coding 1.031 20.444 0.99
90.287 0.981 55.701 1.053 52.324
ENSMUSG00000011263 Exoc3l2 protein_coding 0.944 56.477
1.207 71.617 0.878 20.751 1.056 95.559
ENSMUSG00000011267 Zfp296 protein_coding 0.867 58.264
1.064 88.705 1.08 41.331 1.232 90.177
ENSMUSG00000011305 Plin5 protein_coding 1.216 42.255 0.951
96.216 0.865 54.25 1.278 82.85
ENSMUSG00000011306 Sugp1 protein_coding 0.99 65.106 0.923
61.883 1.094 12.831 1.047 82.66
ENSMUSG00000011350 Gm5893 processed_transcript 1 82.543
1 96.897 1 73.531 1 93.316
ENSMUSG00000011382 Dhdh protein_coding 0.881 14.645 1.031
93.574 1.098 49.601 0.959 89.498
ENSMUSG00000011427 Zfp790 protein_coding 0.994 80.146
1.014 97.155 0.988 86.831 1.068 58.557
ENSMUSG00000011589 Fsd1 protein_coding 0.791 37.82 1.059 95.431
1.194 64.624 0.825 90.674
ENSMUSG00000011632 Pinlyp protein_coding 2.634 53.228

0.497 87.322 0.759 72.464 0.824 94.19
ENSMUSG00000011658 Fuz protein_coding 0.939 30.035 0.997
91.361 1.07 28.055 0.934 86.699
ENSMUSG00000011751 Sptbn4 protein_coding 0.845 22.92 0.87
91.361 1.364 28.74 0.82 85.493
ENSMUSG00000011752 Pgam1 protein_coding 0.904 3.325 1.05 79.055
1.055 59.612 1.1 91.872
ENSMUSG00000011831 Evi5 protein_coding 1.044 70.36 0.957 79.475
1.002 61.171 0.975 96.287
ENSMUSG00000011832 Evi5l protein_coding 0.974 48.146 1.06
79.864 0.969 49.217 1.004 92.018
ENSMUSG00000011837 Snapc2 protein_coding 0.958 51.447
1.011 96.984 1.035 67.823 1.015 93.722
ENSMUSG00000011877 Git1 protein_coding 0.959 36.259 1.016
92.253 1.025 62.307 0.969 92.31
ENSMUSG00000011884 Gltp protein_coding 1.084 18.067 NA NA
0.892 25.052 1.009 91.928
ENSMUSG00000011958 Bnip2 protein_coding 1.003 63.126 0.943
73.766 1.055 43.481 1.079 40.024
ENSMUSG00000011960 NA NA NA NA 0.998 90.116 0.999
57.091 0.954 83.724
ENSMUSG00000012017 Scarf2 protein_coding 1.02 81.125
1.026 96.68 0.955 80.5 0.922 86.991
ENSMUSG00000012042 4930579F01Rik protein_coding 1 85.391
1 95.504 1 85.58 1 94.297
ENSMUSG00000012076 Brms1l protein_coding 0.993 55.303 1.05
86.387 0.959 61.288 1.034 44.882
ENSMUSG00000012114 Med15 protein_coding 0.999 77.113 1.013
89.494 0.988 71.734 0.995 91.525
ENSMUSG00000012117 Dhdds protein_coding 0.935 13.199 1.046
83.104 1.024 56.125 1.042 71.653
ENSMUSG00000012123 Aim1l protein_coding 0.801 50.768 1.046
96.216 1.19 52.574 0.836 89.892
ENSMUSG00000012126 Ubxn11 protein_coding 0.97 61.633
0.929 73.766 1.107 26.912 0.984 92.181
ENSMUSG00000012187 Mogat1 protein_coding 0.847 4.825 1.126
71.98 1.049 44.125 0.958 96.183
ENSMUSG00000012282 Wnt8a protein_coding 0.779 47.174 NA NA
1.871 40.575 1.03 95.676
ENSMUSG00000012296 Tjap1 protein_coding 0.971 28.998 1.033
91.643 0.997 77.17 NA NA
ENSMUSG00000012350 Ehf protein_coding 1.24 4.362 0.883 48.565
0.915 59.125 0.821 84.29
ENSMUSG00000012396 Nanog protein_coding 0.72 80.608 1.17
92.062 1.189 81.169 1.446 81.56
ENSMUSG00000012405 Rpl15 protein_coding 1.094 5.657 0.957 85.116
0.955 40.338 1.072 71.653
ENSMUSG00000012422 Tmem167 protein_coding 1.038 66 1.061
83.104 0.91 32.963 1.007 69.965
ENSMUSG00000012428 Steap4 protein_coding 0.975 71.674
1.296 60.309 0.79 8.626 1.01 95.273
ENSMUSG00000012429 Mplkip protein_coding 0.889 7.218 1.029
96.847 1.096 18.208 0.914 83.724
ENSMUSG00000012443 Kif11 protein_coding 1.14 60.435 0.809

61.883 1.088 78.498 1.374 57.1
ENSMUSG00000012483 Rpa3 protein_coding 0.952 76.135 0.994
90.138 1.053 70.1 1.082 93.413
ENSMUSG00000012519 Mlkl protein_coding 1.039 45.746 1.135
82.809 0.845 27.994 1.194 44.679
ENSMUSG00000012520 Phox2b protein_coding 1 79.277 NA
NA 1 86.258 1 93.612
ENSMUSG00000012535 Tnpo3 protein_coding 1.078 21.599 0.979
87.857 0.95 51.443 1.006 90.455
ENSMUSG00000012609 Ttll5 protein_coding 0.945 25.492 1.065
71.617 0.992 87.431 1.04 71.305
ENSMUSG00000012640 Zfp715 protein_coding 1.147 19.315
0.974 96.843 0.893 8.907 0.93 86.699
ENSMUSG00000012705 Retn protein_coding 0.739 1.017 1.116 63.504
1.213 18.565 0.935 84.29
ENSMUSG00000012777 Acpt protein_coding 0.476 3.325 1.22 96.68
1.723 7.24 1.016 95.72
ENSMUSG00000012819 Cdh23 protein_coding 0.852 33.906 1.12
79.411 1.045 86.258 0.824 83.724
ENSMUSG00000012848 Rps5 protein_coding 1.064 17.336 0.953
53.434 0.983 88.657 1.009 94.567
ENSMUSG00000012889 Podnl1 protein_coding 0.987 64.014
0.985 96.68 1.03 80.015 0.802 92.31
ENSMUSG00000013033 Lphn1 protein_coding 0.969 43.495 1.119
77.598 0.919 80.163 1.063 42.736
ENSMUSG00000013076 Amotl1 protein_coding 0.952 37.248
1.074 79.411 0.976 73.531 0.968 84.29
ENSMUSG00000013083 2200002J24Rik protein_coding 1 86.112
1 93.574 1 73.598 1 93.156
ENSMUSG00000013089 Etv5 protein_coding 0.948 52.018 1.01
86.793 1.041 79.554 1.339 43.377
ENSMUSG00000013150 Gfod2 protein_coding 0.992 48.036 0.96
96.216 1.048 73.759 0.841 83.724
ENSMUSG00000013155 Enkd1 protein_coding 0.897 55.939 NA NA
1.125 66.269 1.114 93.722
ENSMUSG00000013158 4933405L10Rik protein_coding 0.601 52.258
1.791 84.999 0.929 86.258 0.897 96.287
ENSMUSG00000013160 Atp6v0d1 protein_coding 0.943 33.415
1.049 82.904 1.006 84.626 1.094 50.452
ENSMUSG00000013236 Ptprs protein_coding 1.095 16.249 1.025
87.029 0.891 1.705 1.042 76.831
ENSMUSG00000013275 Slc41a1 protein_coding 1.013 52.429 1.1
72.546 0.896 3.432 0.941 83.724
ENSMUSG00000013338 Fer1l4 protein_coding 0.881 53.679
0.655 28.832 1.736 19.106 0.855 86.991
ENSMUSG00000013353 4931406B18Rik protein_coding 1 82.982
1 95.708 1 84.896 1 95.72
ENSMUSG00000013367 Iglon5 protein_coding 1.68 1.323 0.92
78.877 0.646 2.189 1.106 84.31
ENSMUSG00000013415 Igf2bp1 protein_coding 0.452 14.221
2.192 71.617 1.004 88.693 1.076 95.273
ENSMUSG00000013418 B4galnt2 protein_coding 1.07 82.705 1.07
96.216 0.872 74.082 0.638 90.289
ENSMUSG00000013419 Zfp651 protein_coding 1.104 15.178

1.046 90.389 0.867 7.329 1.064 53.923
ENSMUSG00000013465 Nelfb protein_coding 0.991 71.674 1.015
96.847 0.993 68.129 0.99 86.991
ENSMUSG00000013483 Card14 protein_coding 1.015 60.435
0.794 40.053 1.238 24.375 0.93 96.192
ENSMUSG00000013495 Tmem175 protein_coding 1.018 54.44 1.011
93.908 0.974 60.614 0.987 86.991
ENSMUSG00000013523 Bcas1 protein_coding 1.035 74.147 0.973
96.013 0.995 71.734 0.926 86.699
ENSMUSG00000013539 Tango2 protein_coding 0.956 45.728
0.945 68.115 1.106 21.522 1.069 74.047
ENSMUSG00000013584 Aldh1a2 protein_coding 1.794 0 0.702
54.06 0.795 25.052 1.115 63.018
ENSMUSG00000013593 Ndufs2 protein_coding 0.937 10.739 NA
NA 1.072 29.302 1.007 95.832
ENSMUSG00000013611 Snx31 protein_coding 1.131 76.546 1.624
79.29 0.545 23.1 0.554 84.29
ENSMUSG00000013622 Atraid protein_coding 0.988 59.358
0.988 96.68 1.023 86.144 0.962 90.611
ENSMUSG00000013629 Cad protein_coding 0.846 39.953 0.956
95.941 1.228 56.708 1.167 67.691
ENSMUSG00000013643 Lypd8 protein_coding 1 52.799 1
92.137 1 83.857 1 95.832
ENSMUSG00000013646 Sh3bp5l protein_coding 0.918 11.769
1.062 79.367 1.028 55.81 0.969 86.699
ENSMUSG00000013662 Atad1 protein_coding 1.148 0.461 0.924 68.818
0.944 13.643 1.008 92.748
ENSMUSG00000013663 Pten protein_coding 1.051 28.395 1.08
92.111 0.881 16.719 0.947 86.991
ENSMUSG00000013668 4933402N03Rik protein_coding 1 74.299
1 97.155 1 84.83 1 91.525
ENSMUSG00000013698 Pea15a protein_coding 0.996 86.199 1.05
89.996 0.953 69.974 1.076 48.119
ENSMUSG00000013701 Timm23 protein_coding 0.968 48.036
1.023 86.387 1.012 70.623 1.007 92.52
ENSMUSG00000013707 Tnfaip8l2 protein_coding 1.489 0.355 0.904
55.71 0.741 30.827 0.965 94.99
ENSMUSG00000013736 Trnt1 protein_coding 1.073 7.959 0.931 53.434
0.999 82.778 1.037 61.316
ENSMUSG00000013766 Ly6g6e protein_coding 0.925 58.264
0.795 82.848 1.363 43.481 0.987 88.387
ENSMUSG00000013787 Ehmt2 protein_coding 0.992 64.284 1.013
96.013 0.993 70.623 0.993 94.904
ENSMUSG00000013822 Elof1 protein_coding 0.942 18.731 1.068
71.617 0.997 87.744 1.002 91.217
ENSMUSG00000013833 Med16 protein_coding 0.911 21.176 1.072
83.104 1.026 57.637 1.025 94.904
ENSMUSG00000013846 St3gal1 protein_coding 0.941 85.499
1.054 82.904 1.01 44.865 1.399 50.452
ENSMUSG00000013858 Tmem259 protein_coding 0.957 14.086
1.058 79.411 0.989 88.657 0.887 85.493
ENSMUSG00000013878 Rnf170 protein_coding 1.105 5.527 0.984
97.288 0.915 6.858 0.982 96.183
ENSMUSG00000013921 Clip3 protein_coding 1.149 42.255 0.922

93.908 0.945 39.245 1.025 95.777
ENSMUSG00000013928 Thap8 protein_coding 0.76 55.641 NA NA
1.194 58.915 0.989 88.053
ENSMUSG00000013973 Dedd protein_coding 0.997 80.815 0.995
95.211 1.007 78.045 0.96 83.724
ENSMUSG00000013974 1810033B17Rik protein_coding 1.09 55.789
NA NA 0.98 55.214 1.179 96.086
ENSMUSG00000013997 Nit1 protein_coding 0.961 13.883 0.992
96.407 1.051 12.35 0.958 83.724
ENSMUSG00000014030 Pax5 protein_coding 0.782 83.418 0.539
85.95 2.365 46.575 1.721 87.7
ENSMUSG00000014039 Prdm15 protein_coding 1.066 57.38 0.946
94.48 0.989 86.727 1.012 93.734
ENSMUSG00000014074 Rnf168 protein_coding 0.981 84.452
1.043 83.104 0.976 34.592 0.996 94.654
ENSMUSG00000014075 Tctex1d2 protein_coding 1.159 19.746
0.921 72.399 0.939 87.898 0.974 86.699
ENSMUSG00000014077 Chp1 protein_coding 1.003 74.147 1.119
87.609 0.89 21.852 1.014 93.117
ENSMUSG00000014104 Sval2 protein_coding 1 82.732 1
95.708 1 84.724 1 93.457
ENSMUSG00000014158 Trpv4 protein_coding 1.075 21.146 0.896
64.555 1.037 87.103 1.091 94.904
ENSMUSG00000014164 Klhl3 protein_coding 1.014 72.85 0.97 94.597
1.016 85.58 0.852 83.724
ENSMUSG00000014177 Tvp23b protein_coding 1.137 7.492 0.893
21.315 0.986 84.724 1.008 89.498
ENSMUSG00000014195 Dnajc7 protein_coding 1.05 3.498 0.904
13.578 1.049 23.604 0.987 84.29
ENSMUSG00000014198 Zfp385c protein_coding 1.291 82.732
0.953 96.68 0.814 87.686 1.224 68.502
ENSMUSG00000014226 Cacybp protein_coding 0.953 54.983
1.133 96.216 0.925 86.144 1.025 60.466
ENSMUSG00000014232 Cluap1 protein_coding 0.985 63.283 0.95
53.434 1.071 13.259 1.033 61.984
ENSMUSG00000014243 Zswim7 protein_coding 0.971 64.589
1.038 84.832 0.991 81.388 0.883 83.724
ENSMUSG00000014245 Pigl protein_coding 0.916 33.745 0.924
67.513 1.182 6.202 0.92 83.724
ENSMUSG00000014294 Ndufa2 protein_coding 0.857 3.07 1.008
92.111 1.157 18.411 1.029 70.539
ENSMUSG00000014301 Pam16 protein_coding 0.823 0.987 1.076 79.864
1.13 9.86 1.025 96.358
ENSMUSG00000014303 Glis2 protein_coding 1.025 42.134 1.073
73.092 0.909 6.68 1.003 92.748
ENSMUSG00000014313 Cox6c protein_coding 0.989 78.538 0.994
92.111 1.014 86.666 1.012 92.018
ENSMUSG00000014329 Bicc1 protein_coding 1.2 1.412 0.938 78.877
0.886 14.409 1.124 58.557
ENSMUSG00000014349 Ube2z protein_coding 1.017 76.546 0.985
95.211 0.999 82.903 1.047 72.826
ENSMUSG00000014351 Gip protein_coding 1 78.744 1
96.407 1 87.37 1 96.183
ENSMUSG00000014353 Tmem87b protein_coding 0.941 73.585

0.912 59.946 1.167 7.964 1.057 58.721
ENSMUSG00000014355 Anapc1 protein_coding 1.039 23.254
1.004 92.355 0.96 56.365 1.01 94.19
ENSMUSG00000014361 Mertk protein_coding 1.069 78.054 0.697
30.872 1.339 32.433 1.27 72.369
ENSMUSG00000014402 Tsg101 protein_coding 0.911 2.174 1.029
82.904 1.067 15.185 1.039 67.169
ENSMUSG00000014418 Hps5 protein_coding 0.967 62.437 1.046
93.899 0.986 87.971 1.066 57.952
ENSMUSG00000014426 Map3k4 protein_coding 1.105 7.959 1.101
79.261 0.82 1.764 0.906 83.724
ENSMUSG00000014444 Piezo1 protein_coding 0.912 38.286
1.057 79.411 1.038 86.475 0.978 84.29
ENSMUSG00000014453 Blk protein_coding 1.192 76.546 1.346
83.104 0.621 22.279 0.626 90.854
ENSMUSG00000014470 Rnf166 protein_coding 0.988 81.925
0.999 96.847 1.012 87.744 0.982 93.045
ENSMUSG00000014496 Ankrd28 protein_coding 0.994 81.01 0.946
67.513 1.061 14.169 0.999 96.183
ENSMUSG00000014498 Ankrd52 protein_coding 0.911 3.009 1.011
87.609 1.086 20.572 1.027 93.016
ENSMUSG00000014503 Pkd2l2 protein_coding 1.037 41.211
1.033 96.68 0.935 44.865 0.91 85.493
ENSMUSG00000014504 Srp19 protein_coding 1 85.092 0.983
97.263 1.019 80.749 1.082 59.464
ENSMUSG00000014529 4930511M11Rik protein_coding 1 82.732
1 96.001 1 77.532 1 96.071
ENSMUSG00000014542 Clec4f protein_coding 1 69.777 1
96.013 1 85.935 1 96.358
ENSMUSG00000014547 Wdfy2 protein_coding 1.07 30.788 0.968
95.088 0.96 46.575 1.026 82.85
ENSMUSG00000014550 Zfyve20 protein_coding 1.048 3.644 1.001
95.708 0.954 6.858 1.092 48.119
ENSMUSG00000014551 Mrps25 protein_coding 0.921 13.239
1.036 93.899 1.051 28.997 1.047 53.923
ENSMUSG00000014554 Dguok protein_coding 1.021 32.676 0.977
78.51 1.004 84.669 1.01 93.685
ENSMUSG00000014592 Camta1 protein_coding 1.043 61.725
1.033 92.561 0.93 39.245 0.954 83.952
ENSMUSG00000014599 Csf1 protein_coding 1.183 2.969 1.016 86.34
0.833 1.764 1.479 86.301
ENSMUSG00000014601 Strip1 protein_coding 0.924 3.07 1.084
40.99 0.997 84.071 0.971 85.493
ENSMUSG00000014602 Kif1a protein_coding 0.845 13.199 1.069
92.793 1.109 32.433 0.932 90.611
ENSMUSG00000014603 Alx3 protein_coding 1.188 81.647 0.632
79.475 1.337 36.95 1.163 89.108
ENSMUSG00000014606 Slc25a11 protein_coding 0.979 45.894
1.048 89.996 0.977 86.985 0.997 96.248
ENSMUSG00000014609 Chrne protein_coding 1.579 57.133 1.917
72.546 0.33 11.314 0.724 96.183
ENSMUSG00000014633 Cmc2 protein_coding 0.94 34.954 0.99
87.282 1.072 69.825 1.062 84.31
ENSMUSG00000014668 Chfr protein_coding 1.037 83.169 0.997

96.984 0.968 87.898 0.985 94.654
ENSMUSG00000014686 Ceacam16 protein_coding 1.147 54.717
0.648 59.946 1.343 66.647 0.681 83.724
ENSMUSG00000014704 Hoxa2 protein_coding 0.889 43.495 0.95
95.504 1.182 34.807 1 83.067
ENSMUSG00000014747 Ankrd53 protein_coding 0.565 14.403
1.674 73.092 1.054 83.607 0.773 88.387
ENSMUSG00000014748 Tex261 protein_coding 1.032 42.255
1.085 60.309 0.894 0.72 0.988 86.991
ENSMUSG00000014763 Fam120b protein_coding 0.944 29.21 1.038
90.952 1.018 65.567 1.033 79.268
ENSMUSG00000014767 Tbp protein_coding 1.005 70.121 1.012
95.196 0.983 59.922 0.945 88.053
ENSMUSG00000014769 Psmb1 protein_coding 0.994 83.169 1.031
88.881 0.976 73.309 1.023 64.513
ENSMUSG00000014771 Pdcd2 protein_coding 1.124 1.188 0.898 53.434
0.985 59.726 0.944 83.724
ENSMUSG00000014773 Dll1 protein_coding 0.993 85.655 1.083
82.904 0.931 15.465 0.886 83.724
ENSMUSG00000014776 Nol3 protein_coding 0.648 1.343 1.073 76.086
1.431 18.208 1.261 68.696
ENSMUSG00000014778 Fhod1 protein_coding 0.836 0.987 1.048 87.029
1.136 6.506 1.011 93.964
ENSMUSG00000014782 Plekhg4 protein_coding 1.04 81.647
0.969 95.211 0.993 87.274 1.461 76.697
ENSMUSG00000014786 Slc9a5 protein_coding 0.853 26.013
0.914 88.704 1.284 22.444 0.994 96.086
ENSMUSG00000014791 Elmo3 protein_coding 0.836 0.987 0.963 93.817
1.244 5.864 0.985 88.955
ENSMUSG00000014813 Stc1 protein_coding 1.304 14.34 1.028 92.793
0.747 4.963 0.881 83.724
ENSMUSG00000014837 4931428F04Rik protein_coding 0.704 1.017
1.143 82.904 1.244 6.506 1.033 93.132
ENSMUSG00000014846 Tppp3 protein_coding 0.868 3.448 1.222 76.086
0.94 68.584 1.086 42.736
ENSMUSG00000014850 Msh3 protein_coding 1.068 48.288 1.006
88.551 0.931 38.434 0.968 87.218
ENSMUSG00000014856 Tmem208 protein_coding 0.826 4.176 1.097
78.629 1.105 30.292 1.033 92.748
ENSMUSG00000014859 E2f4 protein_coding 0.97 51.447 1.075
80.277 0.959 33.689 1.011 96.183
ENSMUSG00000014867 Surf4 protein_coding 1.011 83.169 1.073
71.617 0.923 16.949 1.108 36.988
ENSMUSG00000014873 Surf2 protein_coding 0.953 48.939 0.988
86.277 1.064 31.4 1.08 68.696
ENSMUSG00000014905 Dnajb9 protein_coding 1.171 2.998 1.013
88.823 0.839 0.72 0.955 86.991
ENSMUSG00000014907 Naf1 protein_coding 1.125 1.009 1.001 87.998
0.886 16.129 0.976 95.273
ENSMUSG00000014932 Yes1 protein_coding 1.098 35.024 0.96
79.807 0.946 82.903 0.886 84.29
ENSMUSG00000014956 Ppp1cb protein_coding 1.122 0.461 0.976
84.594 0.911 4.759 0.967 83.724
ENSMUSG00000014959 Gorasp2 protein_coding 0.914 33.599

1.092 71.98 1.003 69.134 1.128 44.882
ENSMUSG00000014980 Tsen15 protein_coding 1.093 9.695 0.96
79.475 0.955 52.972 1.071 90.177
ENSMUSG00000015001 Oc90 protein_coding 1 78.054 1
95.708 1 73.598 1 91.372
ENSMUSG00000015002 Efr3a protein_coding 1.182 0.2 0.983 96.948
0.862 0.72 0.931 83.724
ENSMUSG00000015013 Trappc2l protein_coding 0.988 81.76 0.988
93.654 1.022 70.328 0.979 93.316
ENSMUSG00000015016 Acsf3 protein_coding 0.817 1.965 1.019 96.407
1.198 13.259 1.03 95.74
ENSMUSG00000015023 Ddx19a protein_coding 1.085 7.332 0.981
95.708 0.936 6.381 0.971 89.892
ENSMUSG00000015027 Galns protein_coding 0.973 58.264 1.088
79.579 0.94 59.726 1.033 90.125
ENSMUSG00000015053 Gata2 protein_coding 1.044 74.745 0.918
72.399 1.045 43.185 0.98 94.654
ENSMUSG00000015083 C8g protein_coding 1.079 66.272 0.92
89.277 NA NA 0.966 92.181
ENSMUSG00000015085 Entpd2 protein_coding 1.175 6.281 0.952
95.211 0.894 11.314 1.019 94.153
ENSMUSG00000015087 Rabl6 protein_coding 0.911 16.472 1.048
82.904 1.045 53.882 1.108 31.133
ENSMUSG00000015090 Ptgds protein_coding 0.567 6.484 0.989 94.48
1.778 17.998 1.176 91.872
ENSMUSG00000015092 Edf1 protein_coding 0.82 1.15 1.055 81.127
1.152 18.208 1.025 96.192
ENSMUSG00000015093 Clic3 protein_coding 0.995 54.043 0.999
93.529 1.004 84.961 0.896 95.74
ENSMUSG00000015094 Npdc1 protein_coding 0.901 2.009 1.04 80.678
1.071 22.517 0.964 94.468
ENSMUSG00000015095 Fbxw5 protein_coding 0.914 10.143 1.087
72.546 1.009 67.823 1 93.316
ENSMUSG00000015112 Slc25a13 protein_coding 1.262 16.539
0.884 80.463 0.896 72.269 0.842 96.358
ENSMUSG00000015120 Ube2i protein_coding 1.007 82.705 1.007
87.553 0.984 78.155 1.032 92.018
ENSMUSG00000015126 Tsr3 protein_coding 0.968 49.621 1.043
94.848 0.993 55.81 1.031 59.394
ENSMUSG00000015127 Unkl protein_coding 0.948 49.047 0.974
88.691 1.081 44.125 0.996 91.65
ENSMUSG00000015133 Lrrk1 protein_coding 0.963 23.923 1.024
97.049 1.009 53.587 1.011 91.872
ENSMUSG00000015134 Aldh1a3 protein_coding 1.039 72.85 0.786
64.123 1.22 23.454 0.983 88.552
ENSMUSG00000015143 Actn1 protein_coding 0.936 29.725 1.044
93.574 1.023 53.134 0.975 85.493
ENSMUSG00000015149 Sirt2 protein_coding 0.966 52.9 1.015 83.464
1.016 83.925 0.937 83.724
ENSMUSG00000015165 Hnrnpl protein_coding 0.996 65.869
0.993 96.216 1.008 63.801 0.943 83.724
ENSMUSG00000015176 Nolc1 protein_coding 0.979 86.819 1.073
96.407 0.948 86.727 1.056 91.525
ENSMUSG00000015189 Casd1 protein_coding 1.026 32.873 1.002

88.704 0.972 74.535 0.972 85.493
ENSMUSG00000015202 Cnksr3 protein_coding 0.932 10.955
1.106 61.032 0.972 54.25 0.894 84.29
ENSMUSG00000015214 Mtmr1 protein_coding 1.023 66.272 1.015
94.887 0.963 85.58 1.019 96.139
ENSMUSG00000015217 Hmgb3 protein_coding 0.929 52.258 0.957
90.744 1.126 42.522 1.014 87.624
ENSMUSG00000015222 Map2 protein_coding 1.133 53.228 1.04
96.378 0.85 58.775 0.94 94.174
ENSMUSG00000015224 Cyp2j9 protein_coding 1.114 48.462
0.988 95.708 0.911 52.087 0.894 86.699
ENSMUSG00000015242 Nipsnap3a protein_coding 1 80.251 NA
NA 1 84.896 1 94.99
ENSMUSG00000015243 Abca1 protein_coding 1.065 43.344 0.824
15.364 1.139 5.864 0.96 92.675
ENSMUSG00000015247 Nipsnap3b protein_coding 0.995 76.827 1.06
82.904 0.948 45.093 0.99 92.762
ENSMUSG00000015289 Lage3 protein_coding 0.943 28.804 NA NA
1.061 36.839 1.058 69.404
ENSMUSG00000015290 Ubl4 protein_coding 1.008 66.129 1.016
93.899 0.972 87.686 1.006 93.156
ENSMUSG00000015291 Gdi1 protein_coding 0.917 3.623 1.008 95.431
1.078 27.431 1.043 67.169
ENSMUSG00000015305 Sash1 protein_coding 1.04 28.693 0.92
55.265 1.041 58.616 0.927 83.724
ENSMUSG00000015312 Gadd45b protein_coding 1.146 64.956
0.985 95.504 0.884 88.161 0.955 88.505
ENSMUSG00000015314 Slamf6 protein_coding 1.207 36.124
1.926 80.806 0.431 3.969 0.799 86.699
ENSMUSG00000015316 Slamf1 protein_coding 1.035 85.499
1.924 83.104 0.504 46.32 0.756 86.699
ENSMUSG00000015335 Zdhhc12 protein_coding 0.886 22.431 NA
NA 1.128 40.955 1.075 93.964
ENSMUSG00000015337 Endog protein_coding 0.886 20.116 1.224
79.411 0.924 78.713 0.901 83.724
ENSMUSG00000015340 Cybb protein_coding 1.866 0 0.826 51.159
0.648 5.336 1.012 95.593
ENSMUSG00000015341 Golga7 protein_coding 0.983 59.674 NA
NA 0.979 82.74 0.992 89.152
ENSMUSG00000015342 Xk protein_coding 1.063 34.439 0.977
93.094 0.96 70.791 0.941 84.29
ENSMUSG00000015354 Pcolce2 protein_coding 1.185 16.124
0.992 93.908 0.848 64.778 0.957 92.181
ENSMUSG00000015355 Cd48 protein_coding 1.566 0 0.961 94.102
0.666 1.898 1.02 94.384
ENSMUSG00000015357 Clpx protein_coding 1.051 46.533 0.936
96.847 1.019 54.964 1.016 94.016
ENSMUSG00000015363 Trabd protein_coding 0.947 36.945 1.039
89.014 1.016 79.897 1.111 62.594
ENSMUSG00000015365 Mov10l1 protein_coding 1 80.608 1
80.052 1 67.163 1 85.786
ENSMUSG00000015377 Dennd6b protein_coding 0.892 4.115 1.012
93.994 1.111 13.047 0.978 91.105
ENSMUSG00000015396 Cd83 protein_coding 1.316 40.635 0.796

86.604	0.953	75.353	0.649	85.493
ENSMUSG00000015405	Ace2	protein_coding	0.906	86.464	0.955
87.998	1.151	81.857	1.025	86.699
ENSMUSG00000015437	Gzmb	protein_coding	1.456	27.731	1.019
83.464	0.675	6.858	0.641	83.724
ENSMUSG00000015441	Gzmf	protein_coding	1	83.726	NA	NA
1	83.857	1	91.011
ENSMUSG00000015443	Gzmn	protein_coding	1	81.178	NA	NA
1	81.525	1	95.877
ENSMUSG00000015451	C4a	processed_transcript	0.805	17.839
1.006	95.708	1.229	25.808	1.136	93.261
ENSMUSG00000015452	Ager	protein_coding	0.742	7.004	1.007	96.378
1.332	30.107	0.905	88.136
ENSMUSG00000015461	Atf6b	protein_coding	0.926	5.778	1.048	79.261
1.03	51.851	1.048	70.624
ENSMUSG00000015467	Egfl8	protein_coding	1.061	68.16	1.111	89.494
0.849	18.965	0.939	91.985
ENSMUSG00000015468	Notch4	protein_coding	0.632	0.987	1.174
91.456	1.351	5.864	0.984	93.964
ENSMUSG00000015474	Ppt2	protein_coding	0.893	5.544	1.075	79.411
1.042	70.328	0.981	85.493
ENSMUSG00000015476	Prrt1	protein_coding	0.766	9.752	1.113	87.282
1.172	36.578	0.951	93.612
ENSMUSG00000015478	Rnf5	protein_coding	0.928	27.836	1.099
82.904	NA	NA	0.985	90.289
ENSMUSG00000015484	Fam163a	protein_coding	0.843	70.121
0.488	93.046	2.427	88.693	2.897	92.748
ENSMUSG00000015488	Cacfd1	protein_coding	0.985	84.324	NA
NA	NA	NA	1.046	78.948
ENSMUSG00000015501	Hivep2	protein_coding	1.122	36.438
1.007	95.735	0.886	16.643	1.001	90.455
ENSMUSG00000015519	2310057J18Rik	protein_coding	1	83.658
NA	NA	1	83.925	1	96.183
ENSMUSG00000015522	Arnt	protein_coding	1.027	86.658	0.978
94.262	0.992	86.666	1.068	48.857
ENSMUSG00000015533	Itga2	protein_coding	0.915	45.229	1.181
83.104	0.925	71.999	0.743	83.724
ENSMUSG00000015536	Mocs2	protein_coding	0.942	8.365	0.869	86.071
1.219	19.877	1.157	31.133
ENSMUSG00000015542	Nat9	protein_coding	0.937	14.403	0.963
91.532	1.104	10.079	0.982	94.468
ENSMUSG00000015568	Lpl	protein_coding	0.922	26.483	1.023
88.91	1.062	64.624	1.003	93.156
ENSMUSG00000015575	Atp6v0e	protein_coding	0.96	24.577
0.995	95.708	1.05	38.152	1.006	92.018
ENSMUSG00000015579	Nkx2-5	protein_coding	1	85.728	1
96.847	1	79.776	1	96.358
ENSMUSG00000015597	Zfp318	protein_coding	1.04	65.537
0.986	95.504	0.975	73.531	1.028	76.046
ENSMUSG00000015599	Ttbk1	protein_coding	0.705	43.816	1.558
95.809	0.906	74.082	0.303	85.493
ENSMUSG00000015605	Srf	protein_coding	1.031	77.407	1.063
83.104	0.913	39.079	0.873	86.699
ENSMUSG00000015619	Gata3	protein_coding	0.888	64.893	1.084

85.756 1.036 72.269 1.099 90.289
ENSMUSG00000015647 Lama5 protein_coding 1.092 84.977 0.93
94.848 0.985 79.776 1.076 89.052
ENSMUSG00000015652 Steap1 protein_coding 1.217 58.896
1.013 90.378 0.813 88.657 1.199 78.948
ENSMUSG00000015653 Steap2 protein_coding 0.943 58.077
1.089 82.904 0.973 85.58 1.068 96.192
ENSMUSG00000015656 Hspa8 protein_coding 0.845 34.572 1.286
97.263 0.917 84.961 1.047 49.806
ENSMUSG00000015659 Serac1 protein_coding 1.048 75.196
0.947 91.361 1.007 79.426 0.958 92.675
ENSMUSG00000015665 Awat1 protein_coding 1 73.734 NA NA
NA NA 1 95.273
ENSMUSG00000015668 Pdzd11 protein_coding 0.959 56.768
0.963 91.275 1.087 47.31 1.078 50.452
ENSMUSG00000015671 Psma2 protein_coding 1.032 74.745 0.977
90.378 0.993 84.626 1.058 60.968
ENSMUSG00000015672 Mrpl32 protein_coding 1.019 74.482
0.963 96.001 1.022 77.17 1.02 67.691
ENSMUSG00000015697 Setdb1 protein_coding 1.069 0.999 1.038
96.68 0.901 2.361 1.044 66.944
ENSMUSG00000015702 Anxa9 protein_coding 0.918 41.33 1.064 87.609
1.02 69.002 1.073 92.564
ENSMUSG00000015709 Arnt2 protein_coding 0.941 81.465 1.007
91.955 1.055 30.561 1.006 90.463
ENSMUSG00000015711 NA NA NA NA 1.029 83.104 0.967
26.369 1.013 90.177
ENSMUSG00000015714 Cers2 protein_coding 1.01 86.006 1.138
60.309 0.872 4.389 0.942 88.955
ENSMUSG00000015721 Nlrp5 protein_coding 1 83.658 1
95.504 1 84.504 1 95.92
ENSMUSG00000015733 Capza2 protein_coding 1.181 0 0.942
67.839 0.9 6.524 1.087 49.806
ENSMUSG00000015745 Plekho1 protein_coding 1.081 29.057
0.996 93.654 0.932 62.533 0.979 93.722
ENSMUSG00000015747 Vps45 protein_coding 1.004 86.199 0.949
74.851 1.047 57.637 1.032 79.713
ENSMUSG00000015748 Prpf3 protein_coding 0.985 80.251 1.057
79.055 0.963 48.577 1.057 71.653
ENSMUSG00000015749 Anp32e protein_coding 0.983 52.9 0.993
87.857 1.02 31.317 1.087 0
ENSMUSG00000015750 Aph1a protein_coding 1.019 60.435 NA NA
0.975 75.353 1.022 81.901
ENSMUSG00000015755 Tab2 protein_coding 1.123 3.393 0.991 96.335
0.9 0.72 0.984 88.387
ENSMUSG00000015757 Ppil4 protein_coding 1.061 77.291 1.027
91.456 0.917 60.233 0.976 92.762
ENSMUSG00000015759 Cnih1 protein_coding 1.009 71.06 1.04 96.407
0.949 41.154 1.081 44.679
ENSMUSG00000015766 Eps8 protein_coding 1.135 0.944 0.913 63.912
0.962 37.617 1.072 48.857
ENSMUSG00000015776 Med22 protein_coding 0.926 67.477 1.042
82.904 1.033 84.724 1.047 94.153
ENSMUSG00000015790 Surf1 protein_coding 0.982 73.585 0.997

92.137 1.021 56.708 1.018 96.192
ENSMUSG00000015804 Med28 protein_coding 0.986 58.56 0.991 92.631
1.022 52.8 1.005 96.183
ENSMUSG00000015806 Qdpr protein_coding 0.968 38.195 0.997
94.887 1.034 45.208 0.963 87.651
ENSMUSG00000015812 Gnrh1 protein_coding 1.049 40.333 1
90.576 0.951 83.538 1.118 83.067
ENSMUSG00000015829 Tnr protein_coding 1.094 86.843 0.864
82.848 1.059 60.5 1.057 92.762
ENSMUSG00000015837 Sqstm1 protein_coding 0.993 82.092
0.975 89.713 1.03 58.915 1.002 90.865
ENSMUSG00000015839 Nfe2l2 protein_coding 1.054 12.985
1.023 83.104 0.928 4.935 1.124 44.882
ENSMUSG00000015843 Rxrg protein_coding 1.251 4.675 1.16 82.904
0.687 0.945 0.881 84.29
ENSMUSG00000015846 Rxra protein_coding 1.087 26.338 1.113
87.609 0.825 7.742 1.02 95.676
ENSMUSG00000015850 Adamtsl4 protein_coding 0.835 1.15 1.075
90.952 1.115 25.144 0.983 93.413
ENSMUSG00000015852 Fcrls protein_coding 1.47 0.664 0.789 64.123
0.86 47.641 1.02 92.263
ENSMUSG00000015854 Cd5l protein_coding 2.217 0 0.557 40.053
0.806 44.211 1.37 40.024
ENSMUSG00000015869 Prpsap1 protein_coding 1.046 27.731 NA
NA 0.964 21.666 1.003 96.192
ENSMUSG00000015880 Ncapg protein_coding 1.409 32.873 0.94
95.941 0.757 50.184 1.688 67.94
ENSMUSG00000015882 Lcorl protein_coding 1.141 25.421 0.982
90.256 0.894 48.851 0.867 83.724
ENSMUSG00000015889 Lta4h protein_coding 1.137 0 0.96 72.399
0.917 6.858 0.962 83.724
ENSMUSG00000015890 Amdhd1 protein_coding 2.1 57.133
0.995 95.708 0.476 47.12 0.517 91.625
ENSMUSG00000015932 Dstn protein_coding 1.097 20.799 0.927
81.284 0.986 57.819 0.979 91.807
ENSMUSG00000015937 H2afy protein_coding 0.913 36.259 1.029
95.211 1.06 37.741 1.035 83.067
ENSMUSG00000015942 Gtf2ird2 protein_coding 1.037 43.939
0.903 37.426 1.069 42.75 0.948 90.157
ENSMUSG00000015943 Bola1 protein_coding 0.876 10.955 1.011
93.899 1.124 29.819 1.016 94.904
ENSMUSG00000015944 Gatsl2 protein_coding 0.985 66.652
1.089 71.617 0.933 33.971 0.979 93.584
ENSMUSG00000015947 Fcgr1 protein_coding 1.546 0.976 1.193 96.763
0.543 11.314 1.785 57.075
ENSMUSG00000015950 Ncf1 protein_coding 1.429 0 0.72 40.463
0.971 35.264 1.276 62.918
ENSMUSG00000015957 Wnt11 protein_coding 1.08 29.596 0.963
90.576 0.959 62.533 0.772 78.025
ENSMUSG00000015961 Adss protein_coding 1.027 85.203 0.916
44.268 1.066 7.635 0.971 92.12
ENSMUSG00000015962 1700016C15Rik protein_coding 0.935 84.819
1.956 91.776 0.544 56.365 0.5 86.445
ENSMUSG00000015966 Il17rb protein_coding 0.612 3.07 1.083

79.411	1.503	11.329	0.928	87.218
ENSMUSG00000015968	Cacna1d	protein_coding	1.021	52.429
1.137	92.111	0.859	32.963	0.952	84.19
ENSMUSG00000015970	Chdh	protein_coding	0.58	1.031	1.254	80.678
1.376	9.334	1.05	90.95
ENSMUSG00000015971	Actr8	protein_coding	1.028	43.654	0.966
73.166	1.005	88.601	0.948	86.699
ENSMUSG00000015980	Lrrc27	protein_coding	0.716	13.062	1.13
77.085	1.231	72.148	1.203	95.74
ENSMUSG00000015981	Stk32c	protein_coding	1.173	58.709
0.865	96.216	0.983	70.214	1.052	94.82
ENSMUSG00000015994	Fnta	protein_coding	1.032	20.942	1.05
86.793	0.923	5.336	1.005	90.125
ENSMUSG00000016018	Skiv2l2	protein_coding	0.962	60.707
0.988	95.504	1.054	49.601	1.061	68.354
ENSMUSG00000016024	Lbp	protein_coding	0.823	48.646	1.003
96.843	1.213	67.163	1.881	44.882
ENSMUSG00000016028	Celsr1	protein_coding	0.953	69.715
0.928	85.116	1.131	34.285	0.87	83.724
ENSMUSG00000016087	Fli1	protein_coding	1.231	2.651	0.958	94.96
0.845	2.687	0.893	84.19
ENSMUSG00000016128	Stard13	protein_coding	1.017	86.333
1.032	88.923	0.95	54.037	0.988	87.088
ENSMUSG00000016150	Tenm1	protein_coding	0.818	62.437	NA	NA
1.335	63.801	1.135	89.294
ENSMUSG00000016179	Camk1g	protein_coding	0.808	37.531
0.994	97.097	1.248	51.596	0.837	94.825
ENSMUSG00000016181	Diexf	protein_coding	1.005	44.061	1.056
95.211	0.941	52.972	1.041	89.498
ENSMUSG00000016194	Hsd11b1	protein_coding	0.538	0.987	1.061
96.407	1.755	7.635	1.033	90.674
ENSMUSG00000016206	H2-M3	protein_coding	1.235	0.577	0.956	96.216
0.845	1.973	1.043	85.577
ENSMUSG00000016239	Lonrf3	protein_coding	0.77	33.906	NA
NA	1.541	30.107	1.124	77.854
ENSMUSG00000016252	Atp5e	protein_coding	0.799	2.142	1.073	96.68
1.167	7.964	1.109	50.452
ENSMUSG00000016253	Nelfcd	protein_coding	0.884	0.987	1.046
83.104	1.083	29.302	1.018	83.067
ENSMUSG00000016255	Tubb1	protein_coding	0.573	52.9	0.724	88.705
2.41	39.439	1.455	93.156
ENSMUSG00000016256	Ctsz	protein_coding	1.162	0.2	0.917	59.396
0.939	23.607	1.149	58.557
ENSMUSG00000016257	Slmo2	protein_coding	0.905	10.014	0.971
95.735	1.139	23.334	1.084	79.268
ENSMUSG00000016262	Sertad4	protein_coding	1.011	73.401	1
96.407	0.991	64.778	0.884	86.699
ENSMUSG00000016283	H2-M2	protein_coding	2.219	3.434	1.254	85.985
0.358	1.061	0.551	83.724
ENSMUSG00000016308	Ube2a	protein_coding	0.876	0.987	1.057	78.629
1.079	35.13	1.028	86.133
ENSMUSG00000016319	Slc25a5	protein_coding	0.959	65.869	1.1
74.663	0.947	42.621	1.013	95.273
ENSMUSG00000016344	Ppdpf	protein_coding	0.829	4.941	1.154	83.104

1.046 48.935 1.005 96.358
ENSMUSG00000016349 Eef1a2 protein_coding 5.49 10.105
1.314 82.904 0.139 0.72 0.892 96.248
ENSMUSG00000016356 Col20a1 protein_coding 0.738 1.239 1.003
95.504 1.35 6.202 0.947 86.991
ENSMUSG00000016382 Pls3 protein_coding 1.001 77.182 1.033
87.037 0.969 25.688 1.048 90.674
ENSMUSG00000016386 Mpped2 protein_coding 1.237 17.582
1.026 95.211 0.79 31.135 0.851 83.724
ENSMUSG00000016409 Nkap protein_coding 1.006 77.291 0.974
93.817 1.021 84.504 1.067 62.918
ENSMUSG00000016427 Ndufa1 protein_coding 0.846 5.962 NA NA
1.263 17.662 1.001 96.086
ENSMUSG00000016458 Wt1 protein_coding 1 68.485 1
93.469 1 86.346 1 96.248
ENSMUSG00000016477 E2f3 protein_coding 0.989 51.056 0.93
72.399 1.088 19.609 1.007 91.872
ENSMUSG00000016481 Cr1l protein_coding 1.056 34.439 1.048
90.454 0.903 15.465 0.984 96.287
ENSMUSG00000016487 Ppfibp1 protein_coding 1.111 15.972
0.893 24.911 1.009 59.274 0.952 87.735
ENSMUSG00000016493 Cd46 protein_coding 0.995 86.006 0.986
85.418 1.019 62.307 0.852 83.724
ENSMUSG00000016494 Cd34 protein_coding 1.121 1.441 1.008 92.062
0.884 15.024 1.176 31.133
ENSMUSG00000016495 Plgrkt protein_coding 1.148 1.712 0.952
68.115 0.916 14.262 0.935 83.724
ENSMUSG00000016496 Cd274 protein_coding 1.02 86.74 1.366 68.96
0.717 7.809 0.844 83.724
ENSMUSG00000016503 Gtf3a protein_coding 0.972 31.333 1.034
88.923 0.994 74.082 1.03 90.674
ENSMUSG00000016510 Mtif3 protein_coding 1 77.763 1.039
82.904 0.962 35.815 1.009 92.181
ENSMUSG00000016520 Lnx2 protein_coding 0.98 79.068 1.055
92.111 0.965 46.575 1.215 93.016
ENSMUSG00000016526 Dyrk3 protein_coding 1.037 84.977 0.818
51.322 1.174 30.213 1.33 40.894
ENSMUSG00000016528 Mapkapk2 protein_coding 1.114 0.577 1.079
65.165 0.829 0.72 1.082 73.368
ENSMUSG00000016529 Il10 protein_coding 1.681 30.788 0.81
88.705 0.734 54.964 1.698 44.882
ENSMUSG00000016534 Lamp2 protein_coding 1.123 0 0.962 77.598
0.926 0.72 1.038 95.877
ENSMUSG00000016541 Atxn10 protein_coding 0.971 81.01 0.992
96.268 1.04 84.896 1.044 83.067
ENSMUSG00000016552 Foxred2 protein_coding 1.253 7.397 0.783
28.822 1.019 78.954 1.106 96.183
ENSMUSG00000016554 Eif3d protein_coding 0.939 22.142 0.968
75.493 1.099 10.819 1.058 83.581
ENSMUSG00000016559 H3f3b protein_coding 0.978 68.846 0.924
65.165 1.109 9.429 0.925 86.699
ENSMUSG00000016619 Nup50 protein_coding 1.066 7.492 1.09 96.216
0.857 2.563 0.991 96.358
ENSMUSG00000016624 Phf21b protein_coding 0.813 13.581

1.134 79.411 1.081 83.857 0.945 93.612
ENSMUSG00000016626 Nlrp14 protein_coding 1 86.658 1
95.809 1 85.352 1 95.877
ENSMUSG00000016637 Ift27 protein_coding 0.965 85.391 1.006
93.574 1.032 77.704 1.14 67.691
ENSMUSG00000016664 Pacsin2 protein_coding 1.009 83.523
0.971 90.053 1.017 69.196 1.072 31.133
ENSMUSG00000016756 Cmah protein_coding 1.204 25.207 0.978
97.263 0.85 51.715 0.984 94.215
ENSMUSG00000016757 Ttll12 protein_coding 1.005 38.682
1.038 96.407 0.953 26.809 1.048 78.042
ENSMUSG00000016758 Bik protein_coding 0.705 1.991 1.042 83.104
1.362 22.366 0.929 92.748
ENSMUSG00000016763 Scube1 protein_coding 1.01 74.147
1.052 83.104 0.94 39.245 0.999 93.685
ENSMUSG00000016831 Tox4 protein_coding 1.031 49.868 0.98
82.947 0.992 80.163 1.027 59.441
ENSMUSG00000016833 Mrps18c protein_coding 1.027 50.913
0.993 91.532 0.979 73.874 1.035 86.445
ENSMUSG00000016918 Sulf1 protein_coding 0.902 40.422 0.979
93.654 1.133 22.444 1.11 49.514
ENSMUSG00000016921 Srsf6 protein_coding 0.963 23.13 1.007 95.504
1.033 45.449 1.006 92.748
ENSMUSG00000016933 Plcg1 protein_coding 1.049 62.007 0.972
92.137 0.978 87.744 1.002 93.612
ENSMUSG00000016940 Kctd2 protein_coding 0.915 15.912 1.033
90.68 1.061 43.842 0.92 77.245
ENSMUSG00000016942 Tmprss6 protein_coding 1.719 3.932 0.735
71.889 0.792 22.864 0.726 85.493
ENSMUSG00000016946 Kctd5 protein_coding 1.036 63.804 0.986
90.744 0.975 86.727 0.99 92.564
ENSMUSG00000016984 Etaa1 protein_coding 1.016 69.777 0.946
85.075 1.037 80.899 1.103 83.067
ENSMUSG00000016995 Matn4 protein_coding 0.884 27.664 0.967
83.876 1.171 28.979 0.897 85.493
ENSMUSG00000017000 Svs6 protein_coding 1 72.985 NA NA
1 83.066 1 96.192
ENSMUSG00000017002 Slpi protein_coding 0.806 66.406 0.953
84.594 1.295 26.663 0.752 93.964
ENSMUSG00000017003 Svs3a protein_coding 1 76.135 1
96.847 1 81.492 1 94.904
ENSMUSG00000017004 Svs5 protein_coding 1 68.353 1
96.68 NA NA 1 95.92
ENSMUSG00000017007 Rbpjl protein_coding 0.709 44.922 0.602
74.851 2.344 23.334 0.64 86.445
ENSMUSG00000017009 Sdc4 protein_coding 1.181 0 0.878 44.566
0.961 44.019 1.035 82.85
ENSMUSG00000017057 Il13ra1 protein_coding 1.129 68.485
0.804 81.284 1.103 62.307 0.748 83.724
ENSMUSG00000017064 Prl5a1 protein_coding 1 83.658 1
95.504 1 88.461 1 95.74
ENSMUSG00000017119 Nbr1 protein_coding 0.966 11.128 1.002
96.652 1.035 15.049 0.988 93.964
ENSMUSG00000017132 Cyth1 protein_coding 0.998 69.089 0.938

						32.358	1.065	25.808	1.025	94.904
ENSMUSG00000017144	Rnd3	protein_coding	0.931	83.333	1.156	89.434	0.93	70.44	1.363	83.067
ENSMUSG00000017146	Brca1	protein_coding	1.552	22.871	0.768	56.027	0.836	84.552	1.053	74.193
ENSMUSG00000017165	Gast	protein_coding	1	81.657	1	95.504	1	80.634	1	94.215
ENSMUSG00000017167	Cntnap1	protein_coding	0.849	19.595	1.162	67.843	NA	NA	1.083	78.042
ENSMUSG00000017176	Nt5c3b	protein_coding	1.101	5.313	1.055	83.104	0.862	3.073	0.954	83.724
ENSMUSG00000017188	Coa3	protein_coding	0.884	7.118	1.084	79.579	1.043	56.931	1.022	82.85
ENSMUSG00000017195	Zpbp2	protein_coding	1.098	77.291	0.989	96.216	0.917	75.353	0.699	85.493
ENSMUSG00000017204	Gsdma	protein_coding	1.821	25.944	NA	NA	0.478	27.675	1.277	91.807
ENSMUSG00000017210	Med24	protein_coding	0.962	81.52	1.064	88.551	0.977	60.614	1.08	82.85
ENSMUSG00000017211	NA	NA	NA	NA	1	93.908	1	57.637	1	96.183
ENSMUSG00000017221	Psmd3	protein_coding	0.874	9.863	1.059	83.104	1.083	35.894	1.068	86.445
ENSMUSG00000017264	Exosc10	protein_coding	1.003	79.93	1.005	96.847	0.989	86.831	1.026	86.445
ENSMUSG00000017286	Glod4	protein_coding	1.04	43.939	0.979	79.475	0.982	87.744	1.028	67.169
ENSMUSG00000017288	Vps53	protein_coding	0.972	71.763	1.024	90.138	1.007	58.051	1.035	90.674
ENSMUSG00000017291	Taok1	protein_coding	1.007	58.802	0.955	96.68	1.04	56.708	0.991	95.92
ENSMUSG00000017299	Dnttip1	protein_coding	1.01	81.657	0.981	60.678	1.005	40.511	0.992	90.463
ENSMUSG00000017300	Tnnc2	protein_coding	7.724	7.501	1.086	83.104	0.119	0.72	0.889	96.183
ENSMUSG00000017307	Acot8	protein_coding	0.888	4.385	0.976	90.138	1.151	7.024	0.985	94.99
ENSMUSG00000017309	Cd300lg	protein_coding	0.982	70.597	0.868	86.604	1.17	55.352	0.927	83.724
ENSMUSG00000017310	Spint4	protein_coding	1	86.006	1	95.735	1	85.352	1	95.92
ENSMUSG00000017311	Pyy	protein_coding	1	71.674	1	96.001	1	74.325	1	96.248
ENSMUSG00000017314	Mpp2	protein_coding	0.724	2.174	0.924	96.68	1.492	15.049	1.202	51.238
ENSMUSG00000017316	NA	NA	NA	NA	1	85.116	1	84.724	1	95.273
ENSMUSG00000017344	Vtn	protein_coding	1.127	45.307	1.112	79.261	0.8	2.722	0.91	85.493
ENSMUSG00000017376	Nlk	protein_coding	1.039	36.686	1.033	83.104	0.934	14.206	0.955	83.724
ENSMUSG00000017386	Traf4	protein_coding	0.926	32.557	1.062	83.104	1.011	84.504	0.982	92.561
ENSMUSG00000017390	Aldoc	protein_coding	1.144	44.061	0.875					

					93.908	1.001	79.524	0.933	88.387
ENSMUSG00000017400	Stac2	protein_coding	0.873	29.725	0.869	81.731	1.318 16.446	1.223	44.679
ENSMUSG00000017404	Rpl19	protein_coding	0.894	11.769	1.041	96.138	1.07 34.403	1.09	42.736
ENSMUSG00000017405	Nek8	protein_coding	0.969	64.284	1.061	87.185	0.97 73.027	1.036	75.81
ENSMUSG00000017412	Cacnb4	protein_coding	1	63.126	0.868	95.504	1.152 39.439	0.932	86.991
ENSMUSG00000017417	Plxdc1	protein_coding	1.481	29.811	1.038	91.456	0.651 9.131	0.669	83.724
ENSMUSG00000017418	Arl5b	protein_coding	0.942	34.162	1.007	97.155	1.055 49.384	0.949	84.29
ENSMUSG00000017421	Zfp207	protein_coding	0.996	85.499	0.999	90.256	1.008 75.155	1.058	59.464
ENSMUSG00000017428	Psmd11	protein_coding	0.97	50.519	1.055	78.705	0.975 55.9	1.032	65.558
ENSMUSG00000017446	C1qtnf1	protein_coding	1.401	1.009	0.929	76.431	0.765 18.122	1.006	83.067
ENSMUSG00000017453	Pipox	protein_coding	1.188	52.429	NA	NA	0.83 55.9	0.869	92.181
ENSMUSG00000017466	Timp2	protein_coding	1.122	9.695	1.117	97.097	0.797 24.163	1.002	90.805
ENSMUSG00000017478	Zc3h18	protein_coding	1	44.922	0.984	93.146	1.017 70.328	0.96	95.877
ENSMUSG00000017485	Top2b	protein_coding	1.069	51.826	0.965	83.733	0.97 85.464	0.986	96.071
ENSMUSG00000017491	Rarb	protein_coding	1.116	65.537	1.028	92.253	0.869 32.703	1.041	92.018
ENSMUSG00000017493	Igfbp4	protein_coding	0.971	41.33	0.906	91.897	1.133 28.997	1.03	79.268
ENSMUSG00000017499	Cdc6	protein_coding	1.156	42.555	0.958	85.418	0.904 84.071	1.571	32.045
ENSMUSG00000017548	Suz12	protein_coding	1.137	4.925	0.903	44.268	0.97 54.037	0.931	83.724
ENSMUSG00000017550	Atad5	protein_coding	0.965	85.391	0.959	75.052	1.077 45.035	1.088	78.948
ENSMUSG00000017561	Crlf3	protein_coding	1.133	1.828	0.953	85.921	0.925 19.552	1.047	92.389
ENSMUSG00000017588	Krt27	protein_coding	1	85.832	1	95.329	1 69.728	1	94.752
ENSMUSG00000017607	Tns4	protein_coding	1.119	1.216	NA	NA	0.893 8.634	0.834	84.29
ENSMUSG00000017615	Tnfaip1	protein_coding	1.052	42.134	1.078	80.277	0.878 2.361	0.981	96.183
ENSMUSG00000017631	Abr	protein_coding	1.022	37.702	1.002	93.654	0.977 59.922	0.955	83.724
ENSMUSG00000017639	Rab11fip4	protein_coding	0.739	15.76	0.892	84.017	1.523 11.348	1.038	91.807
ENSMUSG00000017652	Cd40	protein_coding	1.571	0.767	0.838	92.631	0.757 2.189	1.08	76.831
ENSMUSG00000017664	Slc35c2	protein_coding	1	82.352	1.065	92.111	0.939 21.852	0.967	87.331
ENSMUSG00000017667	Zfp334	protein_coding	0.897	13.952	NA				

NA 1.138 25.808 1.086 83.067
ENSMUSG00000017670 Elmo2 protein_coding 1.113 10.687 1.052
95.211 0.857 9.746 0.995 93.685
ENSMUSG00000017677 Wsb1 protein_coding 1.054 36.195 0.863
38.699 1.101 22.93 0.997 90.611
ENSMUSG00000017679 Ttpal protein_coding 1.082 37.414 1.034
85.756 0.895 23.212 1.028 83.276
ENSMUSG00000017686 Rhot1 protein_coding 0.994 86.145 1.007
95.735 0.996 75.967 0.99 95.273
ENSMUSG00000017692 Rhbdl3 protein_coding 0.995 71.763
1.197 79.411 0.839 20.751 0.839 85.493
ENSMUSG00000017697 Ada protein_coding 1.507 1.815 1.054 87.609
0.63 7.399 0.997 90.674
ENSMUSG00000017707 Serinc3 protein_coding 1.028 39.295
0.871 51.277 1.115 55.512 1.041 91.217
ENSMUSG00000017713 Tha1 protein_coding 0.845 8.181 1.028 91.83
1.152 15.185 0.957 86.699
ENSMUSG00000017715 Pgs1 protein_coding 0.876 15.76 1.038 85.756
1.101 35.441 1.165 88.512
ENSMUSG00000017716 Birc5 protein_coding 1.312 29.057 0.774
51.277 0.984 88.729 1.349 78.948
ENSMUSG00000017718 Afmid protein_coding 0.59 0.987 0.999 88.345
1.696 5.864 1.143 89.525
ENSMUSG00000017720 Trp53tg5 protein_coding 0.724 81.01 2.117
95.088 0.652 85.675 0.589 83.724
ENSMUSG00000017721 Pigt protein_coding 0.923 9.341 1.139 71.617
0.952 65.688 1.027 84.499
ENSMUSG00000017723 Wfdc2 protein_coding 1.073 62.007 0.884
66.09 1.058 67.823 1.146 73.597
ENSMUSG00000017724 Etv4 protein_coding 0.84 32.557 NA NA
1.246 49.601 1.355 57.952
ENSMUSG00000017733 Eppin protein_coding 1 71.516 1
93.654 1 78.864 1 94.904
ENSMUSG00000017734 Dbndd2 protein_coding 1.054 27.731
0.919 80.463 1.031 81.976 1.035 91.525
ENSMUSG00000017737 Mmp9 protein_coding 1.302 11.316 1.1
96.138 0.7 11.116 0.73 83.724
ENSMUSG00000017740 Slc12a5 protein_coding 1.027 69.715 NA
NA 0.989 72.853 0.841 84.29
ENSMUSG00000017747 Ghdc protein_coding 0.945 5.686 1.081 83.104
0.976 74.686 1.03 64.513
ENSMUSG00000017754 Pltp protein_coding 1.286 0.824 0.866 53.434
0.899 39.079 0.925 84.29
ENSMUSG00000017756 Slc12a7 protein_coding 0.925 37.248
1.081 83.104 1 86.258 1.108 90.674
ENSMUSG00000017760 Ctsa protein_coding 1.139 5.848 0.905 55.265
0.971 62.533 0.998 95.877
ENSMUSG00000017764 Zswim1 protein_coding 0.875 16.472
1.082 82.904 1.056 48.772 1.08 79.713
ENSMUSG00000017765 Slc12a4 protein_coding 0.963 23.556
1.025 79.367 1.014 82.74 1.128 61.984
ENSMUSG00000017767 Spata25 protein_coding 1.181 86.74 0.698
74.851 1.215 29.302 0.977 83.067
ENSMUSG00000017774 Myo1c protein_coding 0.893 8.181 1.18 40.99 0.95

87.898 1.184 52.366
ENSMUSG00000017776 Crk protein_coding 1.101 0.664 1.021 83.104
0.89 0.72 1.014 85.786
ENSMUSG00000017778 Cox7c protein_coding 0.854 11.519 1.052
97.097 1.112 27.89 1.08 87.886
ENSMUSG00000017781 Pitpna protein_coding 1.104 9.695 1.033
84.126 0.877 5.336 1.143 61.753
ENSMUSG00000017801 Mlx protein_coding 0.96 83.726 1.195
67.462 0.872 6.202 1.028 95.273
ENSMUSG00000017802 Fam134c protein_coding 1.064 8.996 1.034
95.35 0.905 36.499 1.069 61.316
ENSMUSG00000017817 Jph2 protein_coding 1.533 7.397 1.428 64.304
0.458 0.72 0.992 96.183
ENSMUSG00000017830 Dhx58 protein_coding 1.39 1.129 0.985 90.256
0.731 16.409 1.328 44.882
ENSMUSG00000017831 Rab5a protein_coding 1.048 63.534 1.015
83.104 0.939 28.669 0.956 91.65
ENSMUSG00000017832 Hspb9 protein_coding 1.214 86.145 0.571
76.978 1.437 43.481 1.448 83.067
ENSMUSG00000017837 Nkiras2 protein_coding 1.033 54.44 0.987
96.268 0.979 57.091 0.983 93.584
ENSMUSG00000017843 Ppp2r5c protein_coding 1.123 0 0.978
90.378 0.91 0.72 0.991 96.248
ENSMUSG00000017858 Ift52 protein_coding 1.099 10.391 0.95
64.123 0.957 76.64 0.956 86.445
ENSMUSG00000017861 Mybl2 protein_coding 0.867 84.819 0.974
90.378 1.187 63.665 1.246 73.368
ENSMUSG00000017897 Eya2 protein_coding 1.151 16.249 0.857
41.951 1.014 72.695 1.086 96.184
ENSMUSG00000017914 BC034902 protein_coding 1 74.745 NA
NA 1 87.744 1 94.82
ENSMUSG00000017922 NA NA NA NA 1 95.809 1
87.744 1 94.297
ENSMUSG00000017929 B4galt5 protein_coding 1.43 0.664 1.06
82.904 0.661 0.72 1.401 78.042
ENSMUSG00000017943 Gdap1l1 protein_coding 0.408 50.768
1.066 95.708 2.301 69.825 1.737 78.948
ENSMUSG00000017969 Ptgis protein_coding 1.21 5.109 NA NA
0.836 15.904 1.274 40.894
ENSMUSG00000017978 Cadps2 protein_coding 1.083 43.939
0.878 62.322 NA NA 0.954 86.699
ENSMUSG00000017999 Ddx27 protein_coding 0.84 0.987 1.018 85.756
1.173 8.339 1.112 66.468
ENSMUSG00000018001 Cyth3 protein_coding 1.272 5.253 0.93 59.946
0.847 37.906 1.07 62.594
ENSMUSG00000018008 Cyth4 protein_coding 1.332 0.461 0.946 87.322
0.791 6.858 1.109 70.539
ENSMUSG00000018012 Rac3 protein_coding 0.991 65.869 1.215
76.065 0.829 25.345 0.957 87.182
ENSMUSG00000018040 Rrp7a protein_coding 0.903 10.739 1.018
91.776 1.083 24.383 1.101 77.331
ENSMUSG00000018042 Cyb5r3 protein_coding 0.915 8.122 1.068
85.756 1.024 77.17 1.13 57.1
ENSMUSG00000018068 Ints2 protein_coding 0.869 14.849 1.027

87.282 1.118 67.163 1.068 71.305
ENSMUSG00000018076 Med13l protein_coding 0.971 79.93 0.945
74.483 1.086 50.636 0.98 92.181
ENSMUSG00000018102 Hist1h2bc protein_coding 0.811 7.218 1.132
87.609 1.092 24.383 1.06 93.722
ENSMUSG00000018126 Baiap2l2 protein_coding 0.998 69.408
1.685 67.843 0.594 8.027 0.951 89.892
ENSMUSG00000018143 Mafk protein_coding 0.821 1.15 0.929 60.678
1.31 5.864 1.008 94.825
ENSMUSG00000018160 Med1 protein_coding 1.026 54.44 1.06 79.579
0.919 6.381 0.983 94.99
ENSMUSG00000018166 Erbb3 protein_coding 0.902 20.116 0.827
38.699 1.344 5.864 0.881 86.699
ENSMUSG00000018167 Stard3 protein_coding 0.963 84.263
0.928 60.678 1.117 48.772 1.039 74.896
ENSMUSG00000018168 Ikzf3 protein_coding 1.205 66.58 1.511 81.039
0.55 7.329 0.73 83.724
ENSMUSG00000018169 Mfng protein_coding 0.921 31.614 0.956
88.705 1.132 41.806 0.967 91.291
ENSMUSG00000018171 Vmp1 protein_coding 0.92 35.894 0.886
86.071 1.22 7.024 1.081 69.918
ENSMUSG00000018189 Uchl5 protein_coding 1.132 7.501 0.933 77.598
0.948 31.887 1.103 62.918
ENSMUSG00000018196 Glrx2 protein_coding 0.979 73.585 1.013
93.899 1.004 81.976 0.921 83.724
ENSMUSG00000018199 Trove2 protein_coding 1.028 38.386
0.923 71.889 1.055 83.719 0.941 83.724
ENSMUSG00000018209 Stk4 protein_coding 1.072 6.281 0.994 90.053
0.941 21.852 1.047 74.047
ENSMUSG00000018211 Wfdc15b protein_coding 1 81.125 NA
NA 1 82.31 1 95.92
ENSMUSG00000018217 Pmp22 protein_coding 0.939 28.584 1.149
60.309 0.928 33.402 1.008 93.132
ENSMUSG00000018238 Gdf9 protein_coding 0.924 72.139 1.06
96.216 1.02 86.258 0.923 92.181
ENSMUSG00000018239 Zcchc10 protein_coding 0.995 84.865
0.987 96.68 1.017 73.096 1.038 78.379
ENSMUSG00000018259 Prl8a2 protein_coding 1 78.054 1
96.407 1 87.744 1 94.016
ENSMUSG00000018263 Tbx5 protein_coding 1.023 72.372 1.098
88.092 0.888 39.079 1.126 83.067
ENSMUSG00000018286 Psmb6 protein_coding 0.848 0.987 1.07 67.843
1.1 23.454 1.043 83.067
ENSMUSG00000018287 Spag7 protein_coding 0.88 3.47 1.058 87.282
1.076 7.024 1.088 31.133
ENSMUSG00000018293 Pfn1 protein_coding 1.018 76.135 1.024
82.904 0.958 20.511 1.015 85.786
ENSMUSG00000018322 Tomm34 protein_coding 1.046 38.682
0.967 74.483 0.983 88.601 1 91.372
ENSMUSG00000018326 Ywhab protein_coding 0.962 46.289 0.997
95.329 1.044 50.091 1.026 91.783
ENSMUSG00000018334 Ksr1 protein_coding 1.191 3.644 1.102 78.235
0.762 0.72 1.171 72.116
ENSMUSG00000018339 Gpx3 protein_coding 1.528 0.461 1.068 76.065

0.613 1.342 1.213 85.577
ENSMUSG00000018340 Anxa6 protein_coding 1.025 85.283 1.075
83.104 0.909 55.9 1.243 62.594
ENSMUSG00000018341 Il12rb2 protein_coding 0.815 40.786
1.278 84.768 0.961 82.685 0.891 86.699
ENSMUSG00000018347 Zkscan6 protein_coding 1.076 33.854
0.868 52.266 1.069 54.363 1.009 96.248
ENSMUSG00000018362 Kpna2 protein_coding 1.16 21.146 NA NA
0.863 19.236 1.148 71.008
ENSMUSG00000018363 Smurf2 protein_coding 1.041 24.481
1.006 93.654 0.953 70.214 0.949 83.724
ENSMUSG00000018372 Cep95 protein_coding 0.91 8.938 1.015 82.904
1.086 69.196 1.062 81.901
ENSMUSG00000018377 Vezf1 protein_coding 1.125 1.395 1.055 82.904
0.84 0.72 1.005 94.608
ENSMUSG00000018378 Cuedc1 protein_coding 0.915 57.014
1.147 79.411 0.953 75.637 1.065 74.193
ENSMUSG00000018379 Srsf1 protein_coding 1.007 75.399 1
80.463 0.994 65.8 1.018 72.116
ENSMUSG00000018381 Abi3 protein_coding 0.916 51.089 0.964
96.138 1.133 63.665 0.974 87.651
ENSMUSG00000018387 Shroom1 protein_coding 0.854 10.014
1.024 95.941 1.144 13.259 0.983 86.699
ENSMUSG00000018395 Kif3a protein_coding 1.014 65.284 1.059
93.899 0.93 71.999 0.964 85.493
ENSMUSG00000018398 8-Sep protein_coding 1.027 34.439 1.039
96.335 0.938 48.851 1.065 83.067
ENSMUSG00000018401 Mtmr4 protein_coding 1.006 80.688 0.997
97.263 0.993 83.397 1.048 91.525
ENSMUSG00000018405 Mrm1 protein_coding 0.988 61.043 0.95
87.147 1.067 30.292 1.008 91.335
ENSMUSG00000018411 Mapt protein_coding 1.024 61.633 1.002
89.434 0.973 79.255 1.119 77.245
ENSMUSG00000018412 Kansl1 protein_coding 1.039 17.336
1.016 96.68 0.943 24.163 0.948 85.493
ENSMUSG00000018415 Gid4 protein_coding 0.956 43.495 1.049
66.695 0.998 76.768 1.026 94.654
ENSMUSG00000018417 Myo1b protein_coding 0.899 8.614 0.963 47.727
1.155 5.864 1.007 95.74
ENSMUSG00000018425 Dhx40 protein_coding 1.161 13.633 0.94
76.431 0.916 55.214 0.935 95.74
ENSMUSG00000018427 Ypel2 protein_coding 1.022 86.351 1.004
96.847 0.977 78.045 0.936 86.699
ENSMUSG00000018428 Akap1 protein_coding 1.174 1.493 1.057 94.367
0.807 0.945 0.954 83.952
ENSMUSG00000018433 Nol11 protein_coding 1.134 7.049 NA NA
0.884 8.634 1.006 89.052
ENSMUSG00000018442 Derl2 protein_coding 0.965 51.447 0.991
94.848 1.048 40.875 1.046 60.31
ENSMUSG00000018446 C1qbp protein_coding 1.018 79.068 1.024
86.156 0.959 34.21 1.057 79.268
ENSMUSG00000018449 Rpain protein_coding 1.069 51.297 0.924
61.883 1.008 80.381 1.023 85.786
ENSMUSG00000018451 6330403K07Rik lincRNA 0.999 81.01 1.065

92.931 0.937 80.634 0.572 83.724
ENSMUSG00000018459 Slc13a3 protein_coding 1.63 0.2 0.818
52.266 0.746 19.765 1.328 72.826
ENSMUSG00000018470 Kcnab3 protein_coding 0.861 57.544
0.841 91.532 1.378 45.94 1.393 52.982
ENSMUSG00000018474 Chd3 protein_coding 0.899 38.555 0.971
66.796 1.147 23.334 0.916 91.372
ENSMUSG00000018476 Kdm6b protein_coding 0.968 82.231 1.086
83.104 0.952 59.383 0.943 84.29
ENSMUSG00000018481 Appbp2 protein_coding 1.042 42.134
0.921 51.159 1.039 74.686 1.038 83.067
ENSMUSG00000018486 Wnt9b protein_coding 0.941 59.527 0.744
66.09 1.423 66.928 1.891 52.804
ENSMUSG00000018500 Adora2b protein_coding 1.457 8.477 0.835
90.378 0.819 15.376 1.067 56.339
ENSMUSG00000018501 Ncor1 protein_coding 1.049 66.944 0.983
85.921 0.971 84.83 0.929 83.724
ENSMUSG00000018507 Trpv2 protein_coding 1.173 10.595 1.024
96.216 0.829 22.864 1.001 94.608
ENSMUSG00000018509 Cenpv protein_coding 0.99 73.292 0.958
87.313 1.057 45.035 0.878 83.724
ENSMUSG00000018537 Pcgf2 protein_coding 0.843 8.405 NA NA
1.177 10.76 0.929 85.493
ENSMUSG00000018541 Cwc25 protein_coding 0.949 18.602 1.033
95.735 1.016 41.806 1.027 92.018
ENSMUSG00000018543 1700001P01Rik protein_coding 1.639 68.727
0.992 97.049 0.615 73.096 0.821 92.564
ENSMUSG00000018547 Pip4k2b protein_coding 0.978 83.169
1.113 74.663 0.914 4.759 1.046 83.067
ENSMUSG00000018548 Trim37 protein_coding 0.991 85.655 1
85.756 1.009 66.425 1.065 75.198
ENSMUSG00000018554 Ybx2 protein_coding 0.938 82.092 1.014
87.313 1.05 55.512 0.87 83.724
ENSMUSG00000018559 Ctdnep1 protein_coding 1.037 37.414
1.041 75.62 0.926 1.973 0.995 87.134
ENSMUSG00000018565 Elp5 protein_coding 0.904 10.955 1.052
78.629 1.055 47.512 1.103 50.452
ENSMUSG00000018566 Slc2a4 protein_coding 0.756 0.987 1.08
60.309 1.219 19.286 0.951 90.691
ENSMUSG00000018567 Gabarap protein_coding 1.039 36.124
1.057 71.617 0.906 7.809 1 93.964
ENSMUSG00000018569 Cldn7 protein_coding 1.077 81.657 0.871
75.493 1.067 84.626 0.854 90.674
ENSMUSG00000018570 2810408A11Rik protein_coding 0.807 22.481
0.994 92.355 1.248 13.356 0.939 87.735
ENSMUSG00000018572 Phf23 protein_coding 0.962 71.142 1.086
79.29 0.958 16.719 0.997 91.65
ENSMUSG00000018574 Acadvl protein_coding 0.942 58.077
0.982 96.001 1.082 54.163 1.012 94.608
ENSMUSG00000018581 Dnah11 protein_coding 1.275 40.88 1.085
82.904 0.723 15.63 1.141 90.177
ENSMUSG00000018583 G3bp1 protein_coding 1.014 68.485 NA NA
0.923 69.134 1.002 93.964
ENSMUSG00000018585 Atox1 protein_coding 0.87 8.405 0.999 91.643

1.144 36.839 0.987 92.748
ENSMUSG00000018589 Glra2 protein_coding 1 82.732 1
97.155 1 84.308 1 95.74
ENSMUSG00000018593 Sparc protein_coding 0.989 48.939 0.97
85.116 1.044 53.587 1.104 44.213
ENSMUSG00000018599 Mief2 protein_coding 0.823 18.731 1.081
89.434 1.127 31.761 1.05 79.442
ENSMUSG00000018604 Tbx3 protein_coding 1.112 35.499 0.981
74.483 0.92 86.112 0.924 86.699
ENSMUSG00000018620 Mmp20 protein_coding 1 73.292 1
95.504 1 81.525 1 93.612
ENSMUSG00000018634 Crhr1 protein_coding 1 82.479 1
96.984 1 88.274 1 96.183
ENSMUSG00000018648 Dusp14 protein_coding 0.804 53.679
1.162 86.071 1.07 47.512 1.303 63.708
ENSMUSG00000018651 Tada2a protein_coding 0.985 77.615
1.035 97.155 0.98 85.675 1.042 60.31
ENSMUSG00000018654 Ikzf1 protein_coding 1.246 0 0.989 96.216
0.812 1.342 1.104 72.116
ENSMUSG00000018656 Fam115e protein_coding 1 52.018 1
94.367 1 82.74 1 94.608
ENSMUSG00000018659 Pnpo protein_coding 0.977 86.333 1.094
88.092 0.938 37.103 1.051 78.379
ENSMUSG00000018661 Cog1 protein_coding 0.945 21.025 1.051
92.062 1.008 55.352 0.975 93.964
ENSMUSG00000018666 Cbx1 protein_coding 1.084 7.397 1.019 93.654
0.905 16.051 0.988 95.74
ENSMUSG00000018669 Cdk5rap3 protein_coding 0.94 3.623 0.956
75.618 1.111 8.209 1.058 38.598
ENSMUSG00000018672 Copz2 protein_coding 0.864 1.175 1.051 74.663
1.099 26.978 1.071 78.379
ENSMUSG00000018677 Slc25a39 protein_coding 0.913 4.115 1.045
71.98 1.048 65.235 1.104 78.042
ENSMUSG00000018678 Sp2 protein_coding 0.959 45.728 1.019
85.985 1.02 78.369 0.997 93.045
ENSMUSG00000018697 Aatf protein_coding 1.075 34.696 0.926
65.819 1.001 76.225 1.029 74.193
ENSMUSG00000018698 Lhx1 protein_coding 1 86.112 1
95.329 1 84.724 1 91.783
ENSMUSG00000018707 Dync1h1 protein_coding 0.938 11.232
0.922 61.883 1.155 5.864 0.983 91.372
ENSMUSG00000018727 Cpsf4l protein_coding 0.704 6.952 0.859
85.418 1.652 10.76 0.87 83.724
ENSMUSG00000018733 Pex12 protein_coding 0.973 72.985 1.021
96.847 1.008 82.436 1.031 88.197
ENSMUSG00000018736 Ndel1 protein_coding 1.033 68.727 1.015
84.126 0.952 25.052 1.006 92.381
ENSMUSG00000018740 Slc25a35 protein_coding 0.717 1.175 1.32
60.309 1.053 55.109 1.005 92.181
ENSMUSG00000018750 Zbtb4 protein_coding 0.993 52.134 1.029
82.904 0.974 11.751 1.01 92.851
ENSMUSG00000018752 BC096441 protein_coding 1 73.487 1
96.216 NA NA 1 95.273
ENSMUSG00000018761 Mpdu1 protein_coding 0.927 17.276 1.081

71.617 1.001 81.525 1.009 94.654
ENSMUSG00000018765 Fxr2 protein_coding 0.977 18.379 1.036
85.756 0.983 72.378 0.961 85.493
ENSMUSG00000018770 Atp5g3 protein_coding 0.883 2.536 1.043
77.598 1.088 35.441 1.056 71.653
ENSMUSG00000018774 Cd68 protein_coding 2.207 0 0.713 53.434
0.636 2.144 1.121 62.594
ENSMUSG00000018776 Slc35g3 protein_coding 0.726 75.567 NA
NA 1.772 48.304 1.185 94.904
ENSMUSG00000018796 Acsl1 protein_coding 0.782 7.574 1.03 95.708
1.233 10.76 1.016 94.904
ENSMUSG00000018800 Abca5 protein_coding 1.051 58.896 0.913
56.781 1.042 38.683 0.902 96.358
ENSMUSG00000018809 Smyd4 protein_coding 1.228 15.662 0.952
92.111 0.853 8.907 1.005 89.052
ENSMUSG00000018819 Lsp1 protein_coding 1.143 6.026 0.952 89.713
0.916 52.213 1.221 31.133
ENSMUSG00000018820 Zfyve27 protein_coding 0.939 18.138
0.981 84.594 1.087 14.131 0.948 88.505
ENSMUSG00000018821 Avpi1 protein_coding 1.052 37.091 0.923
59.946 1.029 75.809 1.137 50.452
ENSMUSG00000018822 Sfrp5 protein_coding 1.07 54.44 1.095 93.707
0.851 40.794 1.123 83.067
ENSMUSG00000018830 Myh11 protein_coding 0.989 84.534 1.13
80.678 0.897 41.928 0.97 92.12
ENSMUSG00000018841 Rad51d protein_coding 1.071 74.745
0.831 86.071 1.124 75.009 1.058 87.624
ENSMUSG00000018844 Fndc8 protein_coding 1.254 68.16 1.311 88.136
0.608 30.024 0.719 83.724
ENSMUSG00000018845 Unc45b protein_coding 1.022 55.462
0.998 94.597 0.981 59.726 0.976 94.99
ENSMUSG00000018846 Pank3 protein_coding 0.828 27.836 1.09
83.104 1.109 50.375 1.244 48.119
ENSMUSG00000018848 Rars protein_coding 1.094 14.746 0.995
75.052 0.917 81.388 1.134 51.901
ENSMUSG00000018849 Wwc1 protein_coding 0.956 23.319 0.939
75.493 1.112 12.16 0.868 90.674
ENSMUSG00000018858 Ict1 protein_coding 0.89 2.405 1.101 82.809
1.015 53.587 1.046 86.118
ENSMUSG00000018861 Fdxr protein_coding 0.955 60.339 0.941
83.58 1.115 27.047 1.066 74.047
ENSMUSG00000018862 Otop3 protein_coding 1 86.112 1
90.378 1 87.898 1 87.088
ENSMUSG00000018865 Sult4a1 protein_coding 0.892 62.437
1.511 83.104 0.742 73.027 0.737 94.904
ENSMUSG00000018868 Pnpla5 protein_coding 0.298 7.887 1.089
84.999 3.083 31.4 1.341 95.273
ENSMUSG00000018882 Mrpl45 protein_coding 1.027 62.689
0.989 96.216 0.98 65.688 1.008 95.832
ENSMUSG00000018893 Mb protein_coding 0.899 46.413 1.02
95.211 1.087 70.1 1.097 57.075
ENSMUSG00000018899 Irf1 protein_coding 0.909 11.128 1.035
93.146 1.061 47.31 0.947 84.19
ENSMUSG00000018900 Slc22a5 protein_coding 1.07 24.481

0.911 78.877 1.026 86.475 0.956 91.291
ENSMUSG00000018906 P4ha2 protein_coding 1.402 16.124 0.903
94.597 0.786 18.965 1.114 67.691
ENSMUSG00000018907 Alox12e protein_coding 0.296 65.199
1.792 83.104 1.878 78.864 1.89 95.92
ENSMUSG00000018909 Arrb1 protein_coding 1.085 0 1.043 79.411
0.886 0.72 0.945 83.952
ENSMUSG00000018914 Il3 protein_coding 1 84.865 1
95.809 1 84.504 1 94.82
ENSMUSG00000018919 NA NA NA NA 1.199 90.712 0.779
79.625 0.418 84.29
ENSMUSG00000018920 Cxcl16 protein_coding 1.15 20.296 1.02
85.756 0.849 5.564 1.035 87.182
ENSMUSG00000018921 Pelp1 protein_coding 0.903 12.084 1.01
96.138 1.091 20.315 1.123 31.133
ENSMUSG00000018923 Med11 protein_coding 0.906 30.035 1.121
72.642 0.98 66.201 0.969 92.12
ENSMUSG00000018924 Alox15 protein_coding 1.307 60.435
0.974 87.857 0.782 88.693 1.019 86.445
ENSMUSG00000018925 Gm11435 protein_coding 0.803 83.523
1.096 92.931 1.138 58.516 0.886 86.254
ENSMUSG00000018927 Ccl6 protein_coding 1.153 2.569 0.818 33.183
1.057 69.315 1.583 31.133
ENSMUSG00000018930 Ccl4 protein_coding 0.708 60.003 2.662
72.642 0.531 33.971 0.827 95.72
ENSMUSG00000018931 Gm16515 protein_coding 0.992 83.169
1.105 60.309 0.91 5.087 0.999 85.493
ENSMUSG00000018932 Map2k3 protein_coding 0.88 37.248
1.107 71.617 1.024 81.525 1.169 42.736
ENSMUSG00000018965 NA NA NA NA 1.042 82.809 0.955
27.321 1.03 83.067
ENSMUSG00000018973 Hoxb1 protein_coding 1 86.351 1
88.345 1 74.082 1 88.197
ENSMUSG00000018974 Sart3 protein_coding 0.958 25.3 1.003 85.075
1.045 15.411 1.023 86.209
ENSMUSG00000018983 E2f2 protein_coding 0.938 81.925 1.027
94.367 1.035 85.675 1.179 90.674
ENSMUSG00000018986 Slfn3 protein_coding 1.016 73.841 1.088
82.904 0.901 27.321 1.221 83.067
ENSMUSG00000018995 Nars2 protein_coding 0.979 80.751 1.054
87.604 0.969 59.125 0.941 86.699
ENSMUSG00000018999 Slc35b4 protein_coding 0.971 84.661
1.074 71.617 0.96 17.258 1.036 95.273
ENSMUSG00000019027 Dnah1 protein_coding 0.817 59.358 1.243
91.065 0.987 85.58 1.16 94.654
ENSMUSG00000019039 Dalrd3 protein_coding 0.862 1.343 0.992
91.643 1.169 7.635 0.956 86.991
ENSMUSG00000019054 Fis1 protein_coding 0.84 0.987 1.035 93.574
1.151 5.864 1.095 56.079
ENSMUSG00000019055 Plod1 protein_coding 0.956 79.165 1.025
96.843 NA NA 1.132 56.863
ENSMUSG00000019066 Rab3d protein_coding 0.992 81.925 0.973
96.763 NA NA 1.001 83.865
ENSMUSG00000019080 Mfsd3 protein_coding 0.952 61.334 1.05

83.104 1.001 54.964 0.952 84.29
ENSMUSG00000019082 Slc25a22 protein_coding 1.004 69.943
1.075 75.612 0.925 52.972 0.955 83.724
ENSMUSG00000019087 Atp6ap1 protein_coding 0.996 82.092
1.023 83.104 0.985 42.621 1.034 85.577
ENSMUSG00000019088 Dnase1l1 protein_coding 1.181 6.853 1.026
96.984 0.826 7.339 0.94 92.748
ENSMUSG00000019102 Aldh3a1 protein_coding 2.252 20.942
0.576 38.699 0.774 86.258 1.502 91.872
ENSMUSG00000019122 Ccl9 protein_coding 1.242 0.2 0.868 52.266
0.923 67.339 1.444 33.269
ENSMUSG00000019124 Scrn1 protein_coding 1.205 13.557 NA NA
0.787 26.369 1.028 95.35
ENSMUSG00000019132 BC005537 protein_coding 1.072 0.461 0.985
93.817 0.943 7.509 1.06 40.024
ENSMUSG00000019139 Isyna1 protein_coding 0.731 8.405 0.978
96.407 1.392 25.425 1.171 48.857
ENSMUSG00000019143 Hars2 protein_coding 0.943 18.731 0.994
90.053 1.069 18.208 0.988 93.264
ENSMUSG00000019146 NA NA NA NA 1 95.504 1
86.258 1 93.016
ENSMUSG00000019158 Tmem160 protein_coding 0.83 9.341 1.125
72.642 1.073 55.512 0.9 94.654
ENSMUSG00000019173 Rab5c protein_coding 0.985 55.939 1.04
71.98 0.977 55.214 1.035 83.067
ENSMUSG00000019178 Styxl1 protein_coding 0.848 45.728
1.105 85.378 1.069 81.032 0.944 93.132
ENSMUSG00000019179 Mdh2 protein_coding 0.856 0.987 1.095 77.085
1.068 34.727 1.093 82.85
ENSMUSG00000019188 H13 protein_coding 0.985 81.178 1.106
79.864 0.915 43.426 0.963 94.82
ENSMUSG00000019189 Rnf145 protein_coding 1.08 3.061 1.044
79.29 0.884 0.945 1.022 93.316
ENSMUSG00000019194 Scn1b protein_coding 1.032 69.24 1.115 83.104
0.871 56.365 1.028 71.855
ENSMUSG00000019210 Atp6v1e1 protein_coding 0.95 24.577 0.98
94.102 1.072 25.425 1.051 55.841
ENSMUSG00000019214 Chtf18 protein_coding 1.056 39.416
0.916 59.946 1.032 88.729 1.217 80.77
ENSMUSG00000019235 Rps6kl1 protein_coding 1.066 81.52 0.971
88.551 0.968 56.625 2.232 87.447
ENSMUSG00000019254 Ppp1r12c protein_coding 0.973 56.768
0.971 87.857 1.06 65.034 1.04 54.265
ENSMUSG00000019256 Ahr protein_coding 1.08 6.683 1.043 91.584
0.883 6.858 0.924 83.724
ENSMUSG00000019261 Map1s protein_coding 1.031 85.728 1.022
88.551 0.95 60.614 1.085 50.452
ENSMUSG00000019278 Dpep1 protein_coding 1.103 67.163 0.751
57.613 1.207 61.841 1.051 95.35
ENSMUSG00000019295 Tmem129 protein_coding 0.934 40.786
1.061 82.904 NA NA 1.021 93.457
ENSMUSG00000019297 Nop9 protein_coding 1.032 47.847 0.958
83.733 NA NA 1.018 86.209
ENSMUSG00000019301 Hsd17b1 protein_coding 0.74 34.305

1.343 75.62 1.008 65.902 0.974 92.675
ENSMUSG00000019302 Atp6v0a1 protein_coding 0.992 65.622 0.968 96.652 1.043 82.74 1.167 48.119
ENSMUSG00000019303 Psmc3ip protein_coding 0.769 25.492 1.192 71.617 NA NA 1.088 83.067
ENSMUSG00000019312 Grb7 protein_coding 0.775 5.946 1.029 93.654 1.256 10.76 0.842 87.542
ENSMUSG00000019320 Noxo1 protein_coding 1.06 66.272 0.822 32.358 1.143 16.666 0.844 83.724
ENSMUSG00000019326 Aoc3 protein_coding 0.957 63.126 1.17 71.98 0.891 39.528 0.917 92.31
ENSMUSG00000019338 Zfp687 protein_coding 1.001 79.433 1.058 83.104 0.944 52.658 0.957 85.493
ENSMUSG00000019359 Gdpd2 protein_coding 1.206 80.751 0.771 87.553 1.076 62.648 0.814 93.316
ENSMUSG00000019362 D8Ertd738e protein_coding 0.91 3.848 1.018 89.494 1.082 21.705 1.024 92.762
ENSMUSG00000019368 Sec14l4 protein_coding 0.897 16.02 0.873 56.027 1.28 6.506 0.868 87.496
ENSMUSG00000019370 Calm3 protein_coding 0.93 41.617 1.069 71.222 1.007 60.614 1.104 49.806
ENSMUSG00000019373 Cops3 protein_coding 1.012 86.57 0.968 90.256 1.022 71.839 1.025 80.187
ENSMUSG00000019428 Fkbp8 protein_coding 0.857 0.987 1.044 83.104 1.119 5.864 1.047 60.968
ENSMUSG00000019429 Ffar3 protein_coding 1 75.671 1 94.96 1 74.853 1 94.904
ENSMUSG00000019432 Ddx39b protein_coding 0.925 7.916 1.02 95.809 1.058 9.429 1.022 76.467
ENSMUSG00000019433 Gipc1 protein_coding 0.945 64.589 1.008 95.708 1.045 61.418 1.065 76.046
ENSMUSG00000019437 Tlcd1 protein_coding 0.851 5.962 1.054 83.104 1.115 24.375 0.948 84.29
ENSMUSG00000019461 Plscr3 protein_coding 0.954 61.887 1.029 90.092 NA NA 1.004 94.001
ENSMUSG00000019464 Ptger1 protein_coding 0.792 30.136 1.038 96.216 1.217 42.199 0.991 88.505
ENSMUSG00000019467 Arhgef25 protein_coding 0.909 2.405 0.982 86.277 1.116 5.864 1.06 76.831
ENSMUSG00000019470 Xab2 protein_coding 0.974 30.352 0.94 67.941 1.092 7.635 0.971 96.183
ENSMUSG00000019471 Cdc37 protein_coding 0.874 0.987 0.974 96.138 1.173 5.864 1.087 31.133
ENSMUSG00000019478 Rab4a protein_coding 0.991 83.263 0.991 96.847 1.015 85.921 0.925 85.493
ENSMUSG00000019487 Trip10 protein_coding 0.848 1.15 1.022 89.996 1.157 10.76 1.109 52.804
ENSMUSG00000019489 Cd70 protein_coding 0.567 57.544 1.102 91.661 1.601 41.806 1.656 82.66
ENSMUSG00000019494 Cops6 protein_coding 0.929 3.688 1.006 94.767 1.072 35.673 0.991 94.904
ENSMUSG00000019505 Ubb protein_coding 0.815 0.987 0.94 86.728 1.306 5.864 1.043 59.394
ENSMUSG00000019518 Ap4m1 protein_coding 0.915 15.76 0.978 82.848

1.118 7.635 0.983 95.777
ENSMUSG00000019528 Gyg protein_coding 1.315 0 0.939 87.998
0.808 3.654 0.911 84.29
ENSMUSG00000019539 Rcn3 protein_coding 1.143 10.687 1.035
97.155 0.845 14.788 1.189 35.053
ENSMUSG00000019558 Slc6a8 protein_coding 1.027 53.809
1.109 83.104 0.879 7.329 1.019 96.287
ENSMUSG00000019564 Arid3a protein_coding 1.185 3.598 0.954
88.705 0.884 13.848 0.923 86.991
ENSMUSG00000019577 Pdk4 protein_coding 1.488 11.638 0.662
87.147 1.017 85.58 1.28 92.12
ENSMUSG00000019578 Ubxn6 protein_coding 0.925 5.967 1.008 93.817
1.074 14.449 0.986 95.877
ENSMUSG00000019579 D17Wsu104e protein_coding 0.908 5.967 1.056
75.62 1.045 42.522 1.162 37.634
ENSMUSG00000019590 Cyb561 protein_coding 0.959 54.176
0.851 21.916 1.225 5.864 1.001 95.877
ENSMUSG00000019647 Sema6a protein_coding 1.106 57.133
1.057 83.104 0.853 20.258 0.802 83.724
ENSMUSG00000019659 Ccdc12 protein_coding 0.862 1.017 1.021
96.763 1.136 5.864 1.039 50.452
ENSMUSG00000019689 1110001J03Rik protein_coding 0.81 22.92
1.043 84.768 1.185 62.237 1.082 95.877
ENSMUSG00000019699 Akt3 protein_coding 1.253 0 0.905 53.434
0.884 8.634 0.953 83.724
ENSMUSG00000019710 Mrpl24 protein_coding 0.993 28.804
0.952 72.576 1.059 7.143 0.997 94.153
ENSMUSG00000019715 Gle1 protein_coding 0.833 30.352 NA NA
1.22 37.741 1.19 56.769
ENSMUSG00000019718 L3hypdh protein_coding 0.923 81.925 1.06
83.104 1.023 70.791 1.02 92.018
ENSMUSG00000019726 Lyst protein_coding 0.943 28.514 0.913
96.138 1.163 20.886 1.008 93.132
ENSMUSG00000019731 Slc35e1 protein_coding 1.117 4.925 1.04
68.96 0.858 0.72 1.029 73.401
ENSMUSG00000019732 Calr3 protein_coding 1.226 13.363 0.941
79.261 0.866 52.213 0.81 83.724
ENSMUSG00000019734 Tmc4 protein_coding 0.847 24.817 0.993
87.998 1.189 26.663 0.87 86.991
ENSMUSG00000019737 Syne4 protein_coding 0.732 2.405 0.925 93.046
1.479 7.024 0.961 84.19
ENSMUSG00000019738 Polr2i protein_coding 1.056 74.482 1.04
91.643 0.909 63.075 0.892 83.724
ENSMUSG00000019756 Prl8a1 protein_coding 1 72.655 NA
NA 1 88.693 1 93.964
ENSMUSG00000019761 Krt10 protein_coding 1.095 86.112 1.102
90.116 0.826 62.791 0.797 86.991
ENSMUSG00000019762 Iyd protein_coding 0.607 85.355 1.006
96.216 1.633 85.921 1.001 95.35
ENSMUSG00000019763 Rmnd1 protein_coding 0.88 18.487 1.035
82.904 1.1 31.4 1.013 94.904
ENSMUSG00000019767 Ccdc170 protein_coding 0.939 63.283
0.982 90.256 1.085 46.956 1.045 87.886
ENSMUSG00000019768 Esr1 protein_coding 1.072 69.089 0.978

96.138 0.952 83.397 0.969 93.685
ENSMUSG00000019769 Syne1 protein_coding 1.161 9.206 1.081 82.904
0.798 1.061 0.917 86.699
ENSMUSG00000019773 Fbxo5 protein_coding 1.022 32.676 0.88
74.483 1.108 78.045 1.196 80.77
ENSMUSG00000019774 Mtrf1l protein_coding 1.098 34.439
0.961 86.277 0.948 82.31 0.974 93.685
ENSMUSG00000019775 Rgs17 protein_coding 1.928 22.349 0.587
81.731 0.882 73.598 0.951 95.74
ENSMUSG00000019777 Hdac2 protein_coding 1.02 20.942 1.008
95.735 0.971 13.202 1.037 91.525
ENSMUSG00000019779 Frk protein_coding 1.232 19.132 0.898
93.469 0.905 25.688 1.028 83.581
ENSMUSG00000019782 Rwdd1 protein_coding 0.95 27.968 1.045
82.904 1.009 77.532 1.127 32.698
ENSMUSG00000019785 Clvs2 protein_coding 0.953 72 2.042 71.617
0.512 10.003 2.353 65.845
ENSMUSG00000019787 Trdn protein_coding 6.217 8.102 1.288 86.34
0.125 0.72 0.904 96.139
ENSMUSG00000019789 Hey2 protein_coding 0.915 79.709 1.08
91.584 1.014 87.431 0.913 90.674
ENSMUSG00000019790 Stxbp5 protein_coding 1.025 44.061
0.973 91.361 1.001 71.734 0.987 86.991
ENSMUSG00000019791 Hint3 protein_coding 0.965 39.953 1.014
93.574 1.022 64.024 0.956 94.825
ENSMUSG00000019792 Trmt11 protein_coding 1.12 35.748
0.987 95.329 0.902 25.625 1.041 94.016
ENSMUSG00000019794 Katna1 protein_coding 0.934 36.034
1.019 95.211 1.05 26.179 1.058 40.894
ENSMUSG00000019795 Pcmt1 protein_coding 1.02 84.294 1.067
71.98 0.914 25.512 1.057 40.024
ENSMUSG00000019796 Lrp11 protein_coding 0.767 1.274 1.203 74.663
1.079 40.052 0.88 93.316
ENSMUSG00000019797 1700021F05Rik protein_coding 0.982 85.637
1.018 95.211 0.997 84.434 1.103 57.075
ENSMUSG00000019802 Sec63 protein_coding 1.078 16.959 0.935
60.806 0.989 84.669 1.022 60.968
ENSMUSG00000019803 Nr2e1 protein_coding 1 84.534 1
96.138 1 88.693 1 95.593
ENSMUSG00000019804 Snx3 protein_coding 1.086 7.179 1.037 79.411
0.889 2.563 1.075 73.939
ENSMUSG00000019806 Aig1 protein_coding 1.129 13.492 0.895
66.651 0.993 86.144 0.94 84.29
ENSMUSG00000019808 Adat2 protein_coding 1.064 52.799 0.881
69.175 1.066 75.809 1.01 92.675
ENSMUSG00000019809 Pex3 protein_coding 1.018 57.949 0.968
90.378 1.009 85.921 0.935 83.724
ENSMUSG00000019810 Fuca2 protein_coding 1.229 0.461 0.832 12.804
0.974 56.822 0.992 94.215
ENSMUSG00000019813 Cep57l1 protein_coding 1.059 68.727
1.011 95.735 0.933 71.004 0.927 87.992
ENSMUSG00000019814 Ltv1 protein_coding 0.938 53.679 1.046
97.155 1.018 57.907 1.142 37.634
ENSMUSG00000019815 Zc2hc1b protein_coding 1 56.311 1

93.654 1 88.729 1 90.289
ENSMUSG00000019817 Plagl1 protein_coding 1.189 39.416
0.929 75.4 0.906 68.266 0.839 83.724
ENSMUSG00000019818 Cd164 protein_coding 1.005 74.482 0.964
82.848 1.026 81.857 0.938 92.31
ENSMUSG00000019820 Utrn protein_coding 0.901 20.659 0.948
85.671 1.165 14.376 1.043 95.273
ENSMUSG00000019822 Smpd2 protein_coding 0.886 5.962 0.997 88.705
1.131 5.864 0.984 92.675
ENSMUSG00000019823 Mical1 protein_coding 0.942 26.77 0.996
94.48 1.062 38.975 1.071 52.982
ENSMUSG00000019826 Zbtb24 protein_coding 0.979 65.388
0.941 83.58 1.084 32.173 0.958 86.991
ENSMUSG00000019828 Grm1 protein_coding 1 66.944 1
85.95 1 81.29 1 93.156
ENSMUSG00000019831 Wasf1 protein_coding 1.131 26.136 1.221
75.21 0.724 6.858 0.876 95.832
ENSMUSG00000019832 Rab32 protein_coding 1.081 24.958 1.1
89.434 0.843 19.552 1.028 81.061
ENSMUSG00000019834 Slc22a16 protein_coding 1 82.231 1
96.984 1 88.161 1 96.192
ENSMUSG00000019837 Gtf3c6 protein_coding 0.948 28.584
1.028 96.843 1.028 37.764 0.958 91.291
ENSMUSG00000019838 Slc16a10 protein_coding 1.042 74.958
1.033 93.994 0.931 67.029 1.065 83.276
ENSMUSG00000019841 Rev3l protein_coding 0.761 1.343 1.327 60.309
0.994 54.163 0.95 92.12
ENSMUSG00000019842 Traf3ip2 protein_coding 0.937 38.555
1.071 71.617 0.993 86.985 1.047 92.762
ENSMUSG00000019843 Fyn protein_coding 1.078 24.689 0.991
83.733 0.935 86.084 0.986 95.877
ENSMUSG00000019845 Tube1 protein_coding 1.033 38.682 1.085
92.137 0.894 83.811 0.954 94.654
ENSMUSG00000019846 Lama4 protein_coding 1 76.396 NA NA
NA NA 1.045 85.786
ENSMUSG00000019848 Popdc3 protein_coding 4.778 16.436
1.351 92.561 0.154 2.361 1.284 93.457
ENSMUSG00000019849 Prep protein_coding 0.967 52.258 0.981
96.68 1.054 76.754 1.098 46.846
ENSMUSG00000019850 Tnfaip3 protein_coding 1.043 84.452
1.007 96.847 0.952 80.749 0.985 92.31
ENSMUSG00000019851 Perp protein_coding 1.14 52.429 0.857
75.345 1.024 86.985 1.008 91.928
ENSMUSG00000019852 D10Bwg1379e protein_coding 0.601 23.923
0.844 81.134 1.971 23.41 0.992 95.72
ENSMUSG00000019853 Hebp2 protein_coding 0.932 51.089 0.975
87.553 1.101 30.213 0.982 93.132
ENSMUSG00000019854 Reps1 protein_coding 1.047 15.85 0.962 83.392
0.988 84.552 0.985 92.561
ENSMUSG00000019856 Fam184a protein_coding 1.164 20.032
0.847 89.996 1.014 73.027 0.815 83.724
ENSMUSG00000019857 Asf1a protein_coding 1.047 54.44 1.012 96.897
0.944 48.851 0.999 96.192
ENSMUSG00000019861 Gopc protein_coding 1.028 50.377 0.958

83.733 1.012 84.961 1.032 93.156
ENSMUSG00000019863 Qrsl1 protein_coding 1.02 83.658 1.087
83.104 0.902 57.091 1.055 87.134
ENSMUSG00000019864 Rtn4ip1 protein_coding 0.893 4.468 0.993
92.931 1.124 23.668 1.01 86.445
ENSMUSG00000019866 Aim1 protein_coding 0.991 75.567 1.001
96.138 1.01 84.308 0.931 85.493
ENSMUSG00000019867 NA NA NA NA 1 95.088 1
78.864 1 95.559
ENSMUSG00000019868 Vta1 protein_coding 0.981 85.946 0.922
79.261 1.106 45.449 1.055 83.067
ENSMUSG00000019872 Smpdl3a protein_coding 1.256 0 0.888
39.547 0.891 16.409 0.937 83.724
ENSMUSG00000019873 Reep3 protein_coding 1.076 10.595 1.053
91.584 0.883 4.521 0.948 83.724
ENSMUSG00000019874 Fabp7 protein_coding 1.487 46.097 1.758
94.597 0.382 50.523 1.239 78.379
ENSMUSG00000019876 Pkib protein_coding 1.695 0.2 1 93.899
0.591 0.72 0.997 91.291
ENSMUSG00000019877 Serinc1 protein_coding 1.088 63.126
0.987 95.504 0.931 47.641 0.978 92.181
ENSMUSG00000019878 Hsf2 protein_coding 1.096 2.446 1.103 80.775
0.825 0.879 1.013 94.001
ENSMUSG00000019880 Rspo3 protein_coding 1.764 39.851 0.575
74.633 0.981 87.898 0.559 85.493
ENSMUSG00000019883 Echdc1 protein_coding 1.055 78.224
0.886 90.138 1.07 66.269 0.764 84.19
ENSMUSG00000019889 Ptprk protein_coding 0.944 47.266 0.984
82.848 1.076 28.979 0.944 86.991
ENSMUSG00000019891 Dcbld1 protein_coding 1.098 57.133
0.949 83.392 0.955 88.693 0.913 92.181
ENSMUSG00000019892 Lrriq1 protein_coding 1.333 71.915
0.697 97.263 1.08 80.634 0.877 94.825
ENSMUSG00000019893 Ros1 protein_coding 1 84.819 1
95.211 1 85.935 1 95.35
ENSMUSG00000019894 Slc6a15 protein_coding 0.911 82.092 NA
NA 0.999 76.498 1.053 93.734
ENSMUSG00000019897 Ccdc59 protein_coding 0.981 66.897
0.961 65.165 1.064 22.994 1.031 72.116
ENSMUSG00000019899 Lama2 protein_coding 0.965 79.577 0.925
82.947 1.12 78.155 1.117 90.455
ENSMUSG00000019900 Rfx6 protein_coding 1 77.291 1
95.708 1 86.258 1 96.358
ENSMUSG00000019905 Gprc6a protein_coding 1 83.523 NA
NA 1 78.954 1 94.904
ENSMUSG00000019906 Lin7a protein_coding 1.102 39.416 0.876
87.998 1.036 75.353 1.173 74.607
ENSMUSG00000019907 Ppp1r12a protein_coding 1.023 81.647 NA
NA 0.974 84.961 0.945 88.955
ENSMUSG00000019909 Fam162b protein_coding 1 70.213 NA
NA 1 81.492 1 93.156
ENSMUSG00000019913 Sim1 protein_coding 1.082 50.377 1.223
96.138 0.753 30.827 2.286 87.088
ENSMUSG00000019916 P4ha1 protein_coding 0.94 51.447 1.081

92.561 0.988 88.203 1.202 31.133
ENSMUSG00000019917 10-Sep protein_coding 1.026 30.282
0.987 91.361 0.985 67.962 1.049 92.389
ENSMUSG00000019920 Lims1 protein_coding 1.134 0 0.968 73.094
0.91 1.898 0.994 92.181
ENSMUSG00000019923 Zwint protein_coding 0.912 8.614 1.064 87.185
1.029 36.011 1.082 55.054
ENSMUSG00000019927 Ube2d1 protein_coding 1.06 3.713 1.047
82.904 0.898 0.879 0.986 93.132
ENSMUSG00000019929 Dcn protein_coding 1.203 6.61 1.061 91.361
0.784 10.23 1.051 94.19
ENSMUSG00000019932 Kera protein_coding 1.631 44.694 0.732
91.532 0.837 83.397 0.762 96.071
ENSMUSG00000019933 2310015B20Rik protein_coding 1.101 51.189
1.284 96.948 0.708 50.523 0.862 94.904
ENSMUSG00000019935 Slc17a8 protein_coding 1.399 66.58 0.706
86.277 1.012 84.896 1.021 96.358
ENSMUSG00000019936 Epyc protein_coding 2.124 22.025 0.827
93.574 0.569 15.169 1.494 59.464
ENSMUSG00000019942 Cdk1 protein_coding 1.137 45.307 1.069
79.807 0.819 87.744 1.746 68.354
ENSMUSG00000019943 Atp2b1 protein_coding 1.106 5.109 0.959
87.452 0.943 20.009 0.962 87.735
ENSMUSG00000019944 Rhobtb1 protein_coding 1.12 28.927
0.967 76.978 0.923 50.523 0.988 92.381
ENSMUSG00000019945 1700040L02Rik protein_coding 3.919 3.152
0.431 78.471 0.592 42.895 0.537 83.724
ENSMUSG00000019947 Arid5b protein_coding 1.097 62.354
1.015 95.088 0.899 57.819 0.871 83.724
ENSMUSG00000019948 Actr6 protein_coding 1 77.497 0.973
84.017 1.024 53.587 0.939 86.445
ENSMUSG00000019951 Uhrf1bp1l protein_coding 1.024 70.121
1.078 96.843 0.903 44.865 0.919 87.542
ENSMUSG00000019952 Poc1b protein_coding 1.08 12.985 0.963
82.947 0.962 45.093 0.991 90.157
ENSMUSG00000019960 Dusp6 protein_coding 0.941 44.787 1.003
96.216 1.057 65.034 1.121 40.024
ENSMUSG00000019961 Tmpo protein_coding 1.073 7.501 0.989 96.268
0.945 9.288 0.976 86.445
ENSMUSG00000019966 Kitl protein_coding 0.995 86.843 0.924
84.858 1.085 58.915 0.975 92.181
ENSMUSG00000019969 Psen1 protein_coding 1.003 50.913 1.043
86.387 0.957 16.051 1.037 76.697
ENSMUSG00000019970 Sgk1 protein_coding 1.376 7.799 0.676 40.053
1.076 84.308 0.948 87.651
ENSMUSG00000019971 Cep290 protein_coding 0.898 63.126
0.858 70.693 1.301 27.047 1 94.82
ENSMUSG00000019975 Ikbip protein_coding 1.247 0.976 1.034 87.029
0.776 0.879 1.114 44.882
ENSMUSG00000019977 Hbs1l protein_coding 1.031 43.939 0.959
78.471 1.013 84.308 0.996 94.904
ENSMUSG00000019978 Epb4.1l2 protein_coding 1.045 73.841
1.095 83.104 0.872 20.511 1.026 89.428
ENSMUSG00000019979 Apaf1 protein_coding 1.284 0 0.86 37.426

0.902 32.082 0.957 95.273
ENSMUSG00000019982 Myb protein_coding 1.239 23.254 0.739
78.471 1.091 86.144 1.487 35.053
ENSMUSG00000019984 Med23 protein_coding 0.994 82.352 0.981
79.807 1.027 65.567 0.984 84.29
ENSMUSG00000019986 Ahi1 protein_coding 1.01 81.52 NA NA 0.99
76.388 1.029 83.276
ENSMUSG00000019987 Arg1 protein_coding 0.349 29.454 0.906
95.504 3.165 28.74 1.276 96.071
ENSMUSG00000019988 Nedd1 protein_coding 1.013 39.075 0.964
64.555 1.023 88.41 1.077 72.369
ENSMUSG00000019989 Enpp3 protein_coding 1.24 1.009 0.924 85.95
0.868 6.381 0.782 83.724
ENSMUSG00000019990 Pde7b protein_coding 1.137 11.197 0.956
81.443 0.918 68.266 1.099 53.923
ENSMUSG00000019992 Mtfr2 protein_coding 1.016 48.857 1.017
96.216 0.964 35.559 1.298 83.067
ENSMUSG00000019996 NA NA NA NA 0.87 82.503 1.16
47.776 0.967 86.699
ENSMUSG00000019997 Ctgf protein_coding 1.195 74.482 0.907
93.654 NA NA 0.726 85.493
ENSMUSG00000019998 Stx7 protein_coding 1.139 0 0.969 90.378
0.906 4.759 1.03 76.831
ENSMUSG00000020000 Moxd1 protein_coding 1.07 80.688 1.052
92.253 0.885 67.648 1.045 92.748
ENSMUSG00000020003 Pex7 protein_coding 0.993 81.657 0.993
87.609 1.011 59.125 0.981 86.254
ENSMUSG00000020009 Ifngr1 protein_coding 0.879 30.637
0.878 67.513 1.29 6.202 1.018 94.904
ENSMUSG00000020010 Vnn3 protein_coding 1.189 50.627 0.99
85.075 0.846 86.346 0.855 83.724
ENSMUSG00000020014 Gm872 protein_coding 1.005 65.622 1.074
83.104 0.928 66.201 0.863 92.181
ENSMUSG00000020015 Cdk17 protein_coding 0.948 57.818 1.087
74.663 0.973 66.753 0.909 84.19
ENSMUSG00000020017 Hal protein_coding 1.166 71.516 0.981
93.994 0.872 44.678 1.489 76.347
ENSMUSG00000020018 Snrpf protein_coding 0.959 46.693 1.027
96.216 1.014 58.915 1.084 57.1
ENSMUSG00000020019 Ntn4 protein_coding 1.036 41.211 0.896
71.889 1.077 71.232 0.896 83.724
ENSMUSG00000020021 Fgd6 protein_coding 1.212 0.2 1.108 95.735
0.743 4.23 1.043 91.65
ENSMUSG00000020022 Ndufa12 protein_coding 0.8 3.623 1.042
88.823 1.201 36.839 1.025 91.928
ENSMUSG00000020023 Tmcc3 protein_coding 1.125 26.863 0.868
34.058 1.021 54.163 0.994 95.92
ENSMUSG00000020024 Ccdc41 protein_coding 1.085 36.124 0.99
87.452 0.928 66.425 1.12 48.119
ENSMUSG00000020027 Socs2 protein_coding 1.081 66.76 1.424 75.62 0.65
60.96 1.111 86.118
ENSMUSG00000020029 Nudt4 protein_coding 0.996 83.658 1.125
90.454 0.895 21.852 0.983 91.372
ENSMUSG00000020032 Nuak1 protein_coding 1.092 57.133 0.927

94.48 0.989 82.903 0.973 87.331
ENSMUSG00000020033 4930463O16Rik lincRNA 1 86.74 1
95.211 1 88.203 1 83.067
ENSMUSG00000020034 Tcp11l2 protein_coding 1.023 75.399
0.871 59.396 1.118 51.243 0.924 84.19
ENSMUSG00000020038 Cry1 protein_coding 0.638 8.45 0.917 97.049
1.708 21.201 1.249 40.894
ENSMUSG00000020042 Btbd11 protein_coding 1.12 28.693
1.009 95.735 0.882 7.329 0.797 83.724
ENSMUSG00000020044 Timp3 protein_coding 1.075 81.24 0.961 90.046
0.966 87.431 1.02 90.397
ENSMUSG00000020048 Hsp90b1 protein_coding 0.957 74.021
0.988 91.532 1.06 47.512 1.127 31.133
ENSMUSG00000020052 Ascl1 protein_coding 0.678 27.664 1.608
67.23 0.918 52.087 0.76 83.724
ENSMUSG00000020053 Igf1 protein_coding 1.104 15.85 1.335 72.546
0.68 0.945 0.843 87.735
ENSMUSG00000020056 Ccdc53 protein_coding 1.031 86.145
0.957 87.857 1.015 66.647 1.015 96.287
ENSMUSG00000020057 Dram1 protein_coding 1.036 43.654 1.122
71.98 0.859 2.889 0.846 83.724
ENSMUSG00000020059 Sycp3 protein_coding 0.626 1.698 0.961 97.288
1.661 10.819 1.133 91.807
ENSMUSG00000020061 Mybpc1 protein_coding 2.033 29.536
1.094 89.996 0.451 14.206 1.486 81.251
ENSMUSG00000020062 Slc5a8 protein_coding 0.843 23.923
0.917 85.671 1.297 18.565 1.063 83.276
ENSMUSG00000020063 Sirt1 protein_coding 1.028 51.297 0.944
78.51 1.032 84.961 1.014 91.078
ENSMUSG00000020064 Herc4 protein_coding 1.108 3.393 0.95 83.876
0.948 16.643 0.974 87.651
ENSMUSG00000020067 Mypn protein_coding 5.545 7.553 1.259 83.104
0.143 0.72 0.904 96.192
ENSMUSG00000020068 Pbld2 protein_coding 0.874 37.248 0.936
95.504 1.224 28.997 1.001 96.086
ENSMUSG00000020069 Hnrnph3 protein_coding 0.997 67.334
0.928 47.716 1.083 7.24 0.971 86.699
ENSMUSG00000020070 Rufy2 protein_coding 1.035 68.727 0.97
90.138 0.996 85.464 0.932 86.991
ENSMUSG00000020072 Pbld1 protein_coding 1.117 58.38 0.669 75.993
1.339 54.673 1.015 95.273
ENSMUSG00000020074 Ccar1 protein_coding 1.066 85.283 0.962
90.378 0.973 65.8 1.101 33.269
ENSMUSG00000020075 Ddx21 protein_coding 0.962 77.407 1.018
96.847 1.018 80.089 1.134 63.114
ENSMUSG00000020076 Ddx50 protein_coding 1.067 34.077 0.947
71.889 0.988 76.225 1.004 83.067
ENSMUSG00000020077 Srgn protein_coding 1.19 0.664 0.86 70.971
0.98 50.184 1.078 68.696
ENSMUSG00000020078 Vps26a protein_coding 0.974 80.751
1.087 93.707 0.945 52.213 1.022 73.823
ENSMUSG00000020079 Supv3l1 protein_coding 0.926 44.922
1.062 85.756 1.018 83.857 1.068 74.193
ENSMUSG00000020083 2010107G23Rik protein_coding 0.935 66.944

0.928 68.848 1.152 41.235 0.741 83.724
ENSMUSG00000020085 Aifm2 protein_coding 0.858 6.34 0.937 89.996
1.239 13.174 1.005 90.455
ENSMUSG00000020086 H2afy2 protein_coding 0.957 69.943
0.923 71.889 1.128 18.208 0.976 86.991
ENSMUSG00000020087 Tysnd1 protein_coding 0.944 8.139 1.01
90.68 1.047 42.199 0.91 85.493
ENSMUSG00000020088 Sar1a protein_coding 1.097 0 1.014 97.288
0.899 1.58 1.051 70.539
ENSMUSG00000020089 Ppa1 protein_coding 1.015 83.823 0.881
95.708 1.114 86.666 0.868 83.724
ENSMUSG00000020091 Eif4ebp2 protein_coding 1.003 66.58 1.082
62.898 0.919 1.764 0.952 83.724
ENSMUSG00000020092 Pald1 protein_coding 0.854 9.841 1.152 79.261
1.018 55.81 1.019 95.877
ENSMUSG00000020096 Tbata protein_coding 0.846 79.577 1.365
96.847 0.864 87.898 0.587 84.19
ENSMUSG00000020097 Sgpl1 protein_coding 1.218 1.009 0.877 72.399
0.934 11.64 0.896 83.724
ENSMUSG00000020098 Pcbd1 protein_coding 0.965 78.538 1.03
93.574 NA NA 1.379 71.653
ENSMUSG00000020099 Unc5b protein_coding 0.89 19.83 1.284 71.617
0.877 54.521 0.979 96.183
ENSMUSG00000020100 Slc29a3 protein_coding 0.96 78.224
1.001 96.68 1.036 86.258 1.013 91.291
ENSMUSG00000020101 4632428N05Rik protein_coding 1.154 7.083
0.964 82.503 0.899 26.809 0.956 92.181
ENSMUSG00000020102 Slc16a7 protein_coding 0.875 31.921
1.085 87.609 1.049 61.171 1.134 74.047
ENSMUSG00000020105 Lrig3 protein_coding 0.899 38.555 0.976
96.407 1.138 54.809 0.963 91.078
ENSMUSG00000020107 Anapc16 protein_coding 0.994 73.734
0.964 75.345 1.045 62.995 0.986 94.99
ENSMUSG00000020108 Ddit4 protein_coding 1.168 54.44 0.802 79.807
1.069 84.961 0.97 96.183
ENSMUSG00000020109 Dnajb12 protein_coding 0.987 70.213
0.997 95.708 1.018 82.778 1.038 63.708
ENSMUSG00000020111 Micu1 protein_coding 1.113 4.228 0.971 84.594
0.92 28.237 1.021 60.31
ENSMUSG00000020114 Cand1 protein_coding 1.08 10.464 0.996
94.48 0.93 16.409 0.988 95.74
ENSMUSG00000020115 Tbk1 protein_coding 1.071 13.734 0.941
35.072 0.991 73.598 1.003 96.183
ENSMUSG00000020116 Pno1 protein_coding 0.992 84.324 1.162
83.104 0.865 41.506 1.06 83.581
ENSMUSG00000020120 Plek protein_coding 1.289 0.2 1.059 95.301
0.731 0.72 1.373 31.133
ENSMUSG00000020121 Srgap1 protein_coding 1.428 1.395 0.871
68.115 0.803 4.389 0.994 93.584
ENSMUSG00000020122 Egfr protein_coding 1.525 3.932 0.796 47.831
0.82 50.184 1.204 59.464
ENSMUSG00000020123 Avpr1a protein_coding 0.852 35.894
0.945 93.094 1.244 28.74 1.037 92.389
ENSMUSG00000020124 Usp15 protein_coding 0.987 82.479 0.986

93.654 1.027 38.038 1.036 54.153
ENSMUSG00000020125 Elane protein_coding 0.932 75.858 1.078
93.654 0.997 62.648 0.946 95.74
ENSMUSG00000020128 Vps54 protein_coding 1.109 20.659 0.92
67.839 0.98 88.657 1.005 94.215
ENSMUSG00000020130 Tbc1d15 protein_coding 1.139 0.2 0.958
83.392 0.916 2.563 0.957 84.19
ENSMUSG00000020131 Pcsk4 protein_coding 0.967 63.126 NA NA
1.074 76.225 1.037 92.018
ENSMUSG00000020132 Rab21 protein_coding 1.114 10.595 1.068
64.304 0.841 1.48 0.961 89.892
ENSMUSG00000020133 2310011J03Rik protein_coding 0.943 22.431
1.065 80.775 0.998 79.325 0.971 95.35
ENSMUSG00000020134 Peli1 protein_coding 1.072 40.065 0.934
73.766 0.994 88.729 0.92 84.29
ENSMUSG00000020135 Apc2 protein_coding 1.036 85.499 1.037
89.494 0.931 54.037 0.951 86.991
ENSMUSG00000020137 Thap2 protein_coding 1.109 8.102 0.994 92.561
0.907 7.339 0.964 91.372
ENSMUSG00000020140 Lgr5 protein_coding 0.897 84.865 1.248
96.68 0.893 63.919 0.869 83.724
ENSMUSG00000020142 Slc1a4 protein_coding 1.335 5.313 1.114
79.055 0.673 0.945 1.139 83.865
ENSMUSG00000020143 Dock2 protein_coding 1.537 0.2 0.741 0
0.875 69.134 0.978 88.197
ENSMUSG00000020149 Rab1 protein_coding 1.112 0.355 1.033 79.864
0.871 0.72 1.024 83.067
ENSMUSG00000020150 Gamt protein_coding 0.744 1.031 1.2 74.663
1.122 46.206 1.213 49.514
ENSMUSG00000020151 Ptprr protein_coding 1.221 40.065 0.759
67.165 1.081 78.155 0.799 87.651
ENSMUSG00000020152 Actr2 protein_coding 1.153 0 0.977 73.766
0.887 3.428 1.04 80.324
ENSMUSG00000020153 Ndufs7 protein_coding 0.869 1.031 1.093
88.551 1.054 21.522 1.025 93.156
ENSMUSG00000020154 Ptprb protein_coding 0.952 44.467 0.896
24.911 1.175 5.964 1.036 90.691
ENSMUSG00000020155 Kcnmb1 protein_coding 0.974 62.158
1.287 71.617 0.794 21.431 0.853 84.29
ENSMUSG00000020156 Mum1 protein_coding 0.989 63.283 1.02
72.546 0.988 25.052 1.006 95.74
ENSMUSG00000020159 Gabrp protein_coding 0.809 31.333 1.258
83.104 NA NA 1.019 93.685
ENSMUSG00000020160 Meis1 protein_coding 0.939 41.021 1.039
86.277 1.026 79.325 0.947 88.245
ENSMUSG00000020163 Uqcr11 protein_coding 0.773 1.031 1.063
89.434 1.214 13.356 1.023 96.183
ENSMUSG00000020164 1700058G18Rik antisense 0.331 17.664
1.627 82.904 1.861 63.801 0.754 95.74
ENSMUSG00000020166 Cnot2 protein_coding 1.059 21.599 0.978
88.705 0.966 55.701 1.021 87.447
ENSMUSG00000020167 Tcf3 protein_coding 0.907 11.519 1.044
90.092 1.056 45.449 0.906 83.724
ENSMUSG00000020168 Olfr299 protein_coding 1 86.918 NA

NA	1	88.161	1	96.287
ENSMUSG00000020169	Best3	protein_coding	0.75	44.467	1.122
89.996	1.187	61.841	1.417	86.133
ENSMUSG00000020170	Frs2	protein_coding	1.045	32.058	1.006
95.088	0.951	32.082	0.982	86.991
ENSMUSG00000020171	Yeats4	protein_coding	1.009	85.728
0.998	94.597	0.991	88.693	1.059	77.245
ENSMUSG00000020173	Cobl	protein_coding	1.136	64.401	0.858
74.483	1.022	80.089	0.824	86.699
ENSMUSG00000020175	Rab36	protein_coding	1.093	56.085	1.04
95.735	0.878	57.091	0.78	83.952
ENSMUSG00000020176	Grb10	protein_coding	1.086	19.132	0.969
84.858	0.952	71.388	0.99	96.358
ENSMUSG00000020177	9530003J23Rik	protein_coding	1	63.804
1	96.216	1	88.161	1	94.904
ENSMUSG00000020178	Adora2a	protein_coding	0.807	8.302	1.049
96.843	1.18	13.047	0.772	83.724
ENSMUSG00000020180	Snrpd3	protein_coding	0.946	27	0.999
83.464	1.06	57.637	0.984	87.447
ENSMUSG00000020181	Nav3	protein_coding	1.138	45.307	0.911
93.654	0.967	73.598	0.902	85.493
ENSMUSG00000020182	Ddc	protein_coding	1.019	55.462	1.122
96.013	0.877	58.271	1.083	92.181
ENSMUSG00000020183	Cpm	protein_coding	0.781	17.705	1.042
94.48	1.231	16.666	1.053	94.904
ENSMUSG00000020184	Mdm2	protein_coding	1.059	36.438	0.972
96.68	0.975	25.688	1.068	49.806
ENSMUSG00000020185	E2f7	protein_coding	0.766	14.645	1.001
94.96	1.308	17.412	1.165	60.31
ENSMUSG00000020186	Csrp2	protein_coding	1.381	0.2	0.938	95.088
0.772	1.061	1.148	64.513
ENSMUSG00000020189	Osbpl8	protein_coding	1.022	34.861
0.916	71.331	1.068	76.498	1.012	90.157
ENSMUSG00000020190	Mknk2	protein_coding	0.945	33.745	0.949
94.89	1.114	72.695	1.007	92.181
ENSMUSG00000020191	4930415F15Rik	protein_coding	1.9	81.76
0.291	75.052	1.807	50.636	1.391	82.328
ENSMUSG00000020193	Zpbp	protein_coding	1.266	47.847	0.686
72.399	NA	NA	0.732	85.493
ENSMUSG00000020196	Cabin1	protein_coding	0.944	13.581	1.06
83.104	0.997	55.352	0.975	95.877
ENSMUSG00000020198	Ap3d1	protein_coding	0.959	30.136	1.011
85.756	1.027	68.643	0.969	93.316
ENSMUSG00000020205	Phlda1	protein_coding	0.852	5.204	1.101
72.546	1.068	51.596	0.906	84.29
ENSMUSG00000020211	Sf3a2	protein_coding	1.019	78.299	1.037
79.864	0.948	38.534	1.028	93.964
ENSMUSG00000020212	Mdm1	protein_coding	1.085	59.211	1.066
96.378	0.865	48.387	1.032	74.047
ENSMUSG00000020213	Glipr1l1	protein_coding	1	84.661	1
95.708	1	83.857	1	95.832
ENSMUSG00000020214	Glipr1l2	protein_coding	1	84.294	1
96.68	1	88.274	1	94.297
ENSMUSG00000020216	Jsrp1	protein_coding	4.434	8.847	1.161	90.952

0.193 0.879 0.969 96.042
ENSMUSG00000020218 Wif1 protein_coding 1.355 6.438 0.868 74.633
0.851 44.211 0.917 87.331
ENSMUSG00000020219 Timm13 protein_coding 0.78 1.274 1.059
79.055 1.205 11.518 1.023 94.99
ENSMUSG00000020220 Vps13d protein_coding 1.145 72.372
0.871 68.115 1.004 84.229 1.017 88.136
ENSMUSG00000020224 Llph protein_coding 0.94 34.162 0.967
45.262 1.1 10.615 1.047 61.984
ENSMUSG00000020225 Tmbim4 protein_coding 0.997 76.827
1.033 96.897 0.969 65.902 0.972 84.29
ENSMUSG00000020226 Slc5a4b protein_coding 1 63.385 1
96.001 1 86.112 1 91.807
ENSMUSG00000020227 Irak3 protein_coding 1.249 1.216 0.959 81.731
0.835 11.116 1.06 89.052
ENSMUSG00000020228 Helb protein_coding 0.957 39.174 0.94
93.817 1.111 38.975 1.017 85.577
ENSMUSG00000020229 Slc5a4a protein_coding 1 84.865 1
91.661 1 61.171 1 91.291
ENSMUSG00000020230 Prmt2 protein_coding 1.027 77.182 1.055
87.185 0.923 35.264 1.017 91.217
ENSMUSG00000020231 Dip2a protein_coding 1.076 26.136 0.96
75.345 0.964 60.614 0.998 96.248
ENSMUSG00000020232 Hmg20b protein_coding 0.822 1.803 1.1
82.904 1.106 10.615 0.969 92.12
ENSMUSG00000020234 4930404N11Rik protein_coding 0.744 35.158
1.291 82.904 1.044 79.426 1.03 91.335
ENSMUSG00000020235 Fzr1 protein_coding 1.004 65.537 1.027
96.216 0.971 46.575 0.952 83.724
ENSMUSG00000020238 Ncln protein_coding 0.916 8.45 1.018 86.387
1.071 33.802 1.089 44.679
ENSMUSG00000020241 Col6a2 protein_coding 1.073 68.727
0.953 94.48 0.977 54.25 1.106 49.239
ENSMUSG00000020246 Hcfc2 protein_coding 1.047 16.959 1.039
83.104 0.921 5.628 0.98 96.183
ENSMUSG00000020248 Nfyb protein_coding 1.001 83.523 1.003
93.654 0.994 87.103 1.047 52.324
ENSMUSG00000020250 Txnrd1 protein_coding 1.032 25.026
1.078 87.185 0.897 19.765 1.043 93.612
ENSMUSG00000020251 Glt8d2 protein_coding 1.155 32.873 1.06
93.574 0.815 21.431 1.01 91.525
ENSMUSG00000020253 Ppm1m protein_coding 0.941 36.541 1.142
82.904 0.932 82.685 0.906 84.19
ENSMUSG00000020255 D10Wsu102e protein_coding 1.121 1.292 0.944
43.505 0.942 27.86 1.014 79.545
ENSMUSG00000020256 Aldh1l2 protein_coding 1.375 32.676
1.113 82.904 0.654 6.858 1.152 55.485
ENSMUSG00000020257 Wdr82 protein_coding 1.135 4.465 1.062 71.617
0.83 0.72 1.034 52.982
ENSMUSG00000020258 Glyctk protein_coding 1.099 65.537
1.096 95.735 0.83 59.726 1.05 78.379
ENSMUSG00000020260 Pofut2 protein_coding 0.959 86.918 1.09
82.904 0.953 27.86 1.082 54.265
ENSMUSG00000020261 Slc36a1 protein_coding 1.13 0.664 0.97

97.155 0.908 6.858 1.064 82.66
ENSMUSG00000020262 Adarb1 protein_coding 0.912 17.705
0.905 76.431 1.213 12.703 0.954 92.691
ENSMUSG00000020263 Appl2 protein_coding 1.083 14.59 0.898 50.664
1.031 81.492 0.853 83.724
ENSMUSG00000020264 Slc36a2 protein_coding 1.096 30.497
1.049 96.847 0.867 28.669 0.787 83.724
ENSMUSG00000020265 Sumo3 protein_coding 1.043 13.292 0.962
93.094 0.999 49.967 1.068 44.679
ENSMUSG00000020267 Hint1 protein_coding 0.997 81.196 0.983
89.277 1.016 71.839 1.063 51.967
ENSMUSG00000020268 Lyrm7 protein_coding 0.873 23.13 1.056 83.104
1.087 49.601 0.946 95.273
ENSMUSG00000020270 1700008A04Rik protein_coding 1 85.946
NA NA 1 74.325 1 96.287
ENSMUSG00000020271 Fbxw11 protein_coding 0.954 29.21 1.061
75.62 0.985 86.475 0.909 83.724
ENSMUSG00000020272 Stk10 protein_coding 1.069 41.211 1.104
80.277 0.848 3.943 0.924 83.724
ENSMUSG00000020273 Papolg protein_coding 1.034 58.38 1
96.216 0.965 55.701 1.063 89.108
ENSMUSG00000020275 Rel protein_coding 0.91 49.047 1.171
71.98 0.941 27.194 1.011 95.559
ENSMUSG00000020277 Pfkl protein_coding 0.662 0.987 1.13 87.185
1.34 7.24 1.011 92.018
ENSMUSG00000020279 Il9r protein_coding 2.079 12.985 1.422
96.847 0.338 14.262 0.662 83.724
ENSMUSG00000020280 Pus10 protein_coding 0.9 16.652 0.991
91.955 1.116 8.647 1.001 83.581
ENSMUSG00000020282 Rhbdf1 protein_coding 0.883 1.422 1.093
71.617 1.037 40.875 0.995 87.624
ENSMUSG00000020283 Pex13 protein_coding 0.902 4.351 NA NA
1.111 20.315 0.981 93.722
ENSMUSG00000020284 1810043G02Rik protein_coding 0.924 21.176
NA NA 1.067 42.522 0.99 92.466
ENSMUSG00000020286 1700093K21Rik protein_coding 0.805 71.915
0.685 91.361 1.804 46.206 0.898 90.805
ENSMUSG00000020287 Mpg protein_coding 0.952 45.894 0.888
68.115 1.178 14.169 1.029 71.653
ENSMUSG00000020288 Ahsa2 protein_coding 0.919 42.922 1.114
96.68 0.975 81.976 0.999 81.901
ENSMUSG00000020289 Nprl3 protein_coding 0.889 2.5 1.002 95.211
1.124 5.977 0.932 87.218
ENSMUSG00000020290 Xpo1 protein_coding 1.171 2.784 0.948 92.137
0.899 15.904 1.065 69.965
ENSMUSG00000020297 Nsg2 protein_coding 0.977 64.193 1.262
82.904 0.808 21.431 0.875 84.29
ENSMUSG00000020299 4930524B15Rik protein_coding 1 65.284
1 95.504 1 84.626 1 95.273
ENSMUSG00000020300 Cpeb4 protein_coding 1.021 82.231 0.974
95.211 1.002 83.719 0.885 83.724
ENSMUSG00000020303 Stc2 protein_coding 0.677 1.031 0.975 96.68
1.508 6.202 1.091 82.328
ENSMUSG00000020305 Asb3 protein_coding 1.14 5.932 0.973 93.765

0.904 12.41 0.923 84.29
ENSMUSG00000020307 Cdc34 protein_coding 0.732 1.031 1.218 78.494
1.122 36.011 1.023 87.088
ENSMUSG00000020308 Tpgs1 protein_coding 0.884 20.369 0.961
91.897 1.175 18.411 0.997 90.95
ENSMUSG00000020309 Chac2 protein_coding 1.022 50.913 0.94
95.708 1.04 87.519 1.032 96.183
ENSMUSG00000020310 Madcam1 protein_coding 0.714 58.802
0.981 93.654 1.428 42.75 0.61 83.724
ENSMUSG00000020311 Erlec1 protein_coding 1.066 16.436
1.051 71.617 0.892 0.879 1.061 48.857
ENSMUSG00000020312 Shc2 protein_coding 1.203 4.675 1.017 96.652
0.816 19.552 1.126 84.31
ENSMUSG00000020315 Sptbn1 protein_coding 1.016 66.129 0.87
62.322 1.13 13.047 0.999 93.734
ENSMUSG00000020317 Theg protein_coding 1 73.292 1
95.504 1 84.552 1 92.381
ENSMUSG00000020319 Wdpcp protein_coding 1.19 9.805 0.99 91.83
0.851 20.751 0.93 90.289
ENSMUSG00000020321 Mdh1 protein_coding 0.905 33.415 1.053
93.27 1.05 65.8 0.955 90.854
ENSMUSG00000020323 Prss57 protein_coding 0.74 27.431
1.019 97.097 1.33 25.564 1.171 86.118
ENSMUSG00000020325 Fstl3 protein_coding 1.032 82.092 0.952
91.532 1.018 61.841 1.049 65.558
ENSMUSG00000020326 Ccng1 protein_coding 1.04 49.868 1.055
82.904 0.908 6.858 1.158 50.74
ENSMUSG00000020328 Nudcd2 protein_coding 1.054 21.703
0.892 66.651 1.062 68.82 1.159 65.845
ENSMUSG00000020329 Polrmt protein_coding 0.884 0.987 1.061
72.546 1.067 26.498 1.042 70.85
ENSMUSG00000020330 Hmmr protein_coding 2.334 17.336 0.493
35.072 0.87 86.258 1.486 69.708
ENSMUSG00000020331 Hcn2 protein_coding 0.916 77.182 0.986
96.407 1.106 76.64 1.039 96.183
ENSMUSG00000020332 4930404A10Rik protein_coding 1 85.129
1 95.708 1 86.258 1 95.273
ENSMUSG00000020333 Acsl6 protein_coding 1.836 1.712 1.519 87.609
0.359 1.061 1.156 83.067
ENSMUSG00000020334 Slc22a4 protein_coding 1.004 86.351
0.939 87.041 1.064 53.134 1.048 60.968
ENSMUSG00000020335 Zfp354b protein_coding 0.857 37.531
1.045 88.91 1.115 63.801 1.241 71.653
ENSMUSG00000020340 Cyfip2 protein_coding 1.171 11.879
1.331 64.123 0.643 0.879 0.914 84.29
ENSMUSG00000020346 Mgat1 protein_coding 1.029 73.401 1.004
94.367 0.97 46.956 1.005 83.067
ENSMUSG00000020349 Ppp2ca protein_coding 0.982 39.174
0.979 95.504 1.035 48.451 1.042 82.328
ENSMUSG00000020354 Sgcd protein_coding 0.711 5.686 NA NA
1.381 15.049 1.204 78.948
ENSMUSG00000020357 Flt4 protein_coding 0.874 21.897 1.09
81.127 1.048 70.1 0.906 83.724
ENSMUSG00000020358 Hnrnpab protein_coding 0.996 85.499

1.017 89.434 0.987 86.112 0.976 79.268
ENSMUSG00000020359 Phykpl protein_coding 0.933 76.135
1.039 96.948 1.033 79.119 0.98 85.493
ENSMUSG00000020361 Hspa4 protein_coding 1.003 78.909 1.049
96.763 0.947 86.144 1.036 46.412
ENSMUSG00000020362 Cnot6 protein_coding 1.104 7.255 0.938 87.313
0.966 36.499 1.028 84.246
ENSMUSG00000020363 Gfpt2 protein_coding 0.83 2.536 0.929 84.017
1.292 14.449 1.137 71.653
ENSMUSG00000020364 Zfp354a protein_coding 1.097 48.288
0.896 90.287 1.017 87.274 0.874 84.29
ENSMUSG00000020366 Mapk9 protein_coding 0.977 85.946 1.052
88.551 0.975 73.874 1.019 93.722
ENSMUSG00000020368 Canx protein_coding 1.025 71.896 0.995
97.129 0.981 55.214 1.042 66.468
ENSMUSG00000020372 Gnb2l1 protein_coding 1.116 4.874 0.963
55.71 0.929 48.577 1.018 95.273
ENSMUSG00000020374 Rasgef1c protein_coding 0.795 3.559 1.199
90.895 1.05 39.471 0.864 86.991
ENSMUSG00000020375 Rufy1 protein_coding 1.042 29.596 0.987
94.96 0.971 59.922 1.029 86.133
ENSMUSG00000020376 Rnf130 protein_coding 1.165 0 0.966
46.764 0.884 0.945 1.048 91.872
ENSMUSG00000020377 Ltc4s protein_coding 1.135 29.596 1.127
82.219 0.782 9.563 0.927 93.964
ENSMUSG00000020380 Rad50 protein_coding 1.154 4.628 0.985 86.538
0.881 17.258 1.04 83.067
ENSMUSG00000020381 3010026O09Rik protein_coding 0.898 14.086
0.912 87.553 1.217 10.079 1.094 50.74
ENSMUSG00000020385 Clk4 protein_coding 0.886 8.181 1.033 84.594
1.09 5.964 1.037 82.091
ENSMUSG00000020386 Sar1b protein_coding 1.028 43.654 1.002
95.504 0.972 57.446 1.13 57.075
ENSMUSG00000020387 Phf15 protein_coding 1.158 29.536 0.899
55.71 0.96 88.274 1.062 96.183
ENSMUSG00000020388 Pdlim4 protein_coding 1.159 2.085 0.936
86.861 0.922 11.751 1.237 49.239
ENSMUSG00000020389 Cdkl3 protein_coding 0.903 54.604 1.049
97.155 1.059 56.931 1.112 62.594
ENSMUSG00000020390 Ube2b protein_coding 1.029 52.134 0.964
72.576 1.009 78.155 0.97 88.136
ENSMUSG00000020392 Cdkn2aipnl protein_coding 1.104 28.395
1.021 90.389 0.885 43.426 1.002 78.546
ENSMUSG00000020393 Kremen1 protein_coding 1.076 18.067
0.987 95.809 0.939 6.858 0.95 84.29
ENSMUSG00000020395 Itk protein_coding 0.969 86.112 1.285
82.904 0.804 52.213 0.928 83.952
ENSMUSG00000020396 Nefh protein_coding 0.496 39.174 0.967
90.138 2.08 29.819 1.56 85.629
ENSMUSG00000020397 Med7 protein_coding 1.141 3.061 1.009 96.216
0.867 6.202 0.947 96.287
ENSMUSG00000020399 Havcr2 protein_coding 1.236 51.737 0.88
96.216 0.92 62.533 0.937 95.593
ENSMUSG00000020400 Tnip1 protein_coding 0.965 67.334 1.034

95.088 0.999 76.923 1.001 96.287
ENSMUSG00000020401 Fam71b protein_coding 1 83.968 1
92.253 1 58.775 1 96.183
ENSMUSG00000020402 Vdac1 protein_coding 0.977 60.339 0.994
89.713 1.033 41.235 1.098 44.213
ENSMUSG00000020405 Fabp6 protein_coding 1 85.728 NA NA
1 67.648 1 93.261
ENSMUSG00000020407 Upp1 protein_coding 0.779 17.031 NA NA
1.216 20.886 0.988 96.358
ENSMUSG00000020409 Slu7 protein_coding 1.005 62.354 0.958
79.475 1.042 72.695 1.067 50.452
ENSMUSG00000020411 Nipal4 protein_coding 1.011 82.891
1.195 80.806 0.829 39.528 0.924 96.183
ENSMUSG00000020412 Ascc2 protein_coding 0.961 10.739 1.034
83.819 1.003 62.648 1.027 73.939
ENSMUSG00000020413 Hus1 protein_coding 1.034 67.608 0.987
96.68 0.978 78.045 0.982 88.955
ENSMUSG00000020415 Pttg1 protein_coding 0.976 63.126 1.039
90.952 0.982 86.831 1.035 79.268
ENSMUSG00000020419 Hormad2 protein_coding 1.395 82.982
0.878 96.268 0.816 86.985 0.844 87.088
ENSMUSG00000020420 Zfp607 protein_coding 0.951 60.003
0.898 87.452 1.166 49.384 1.028 96.086
ENSMUSG00000020422 Tns3 protein_coding 1.003 86.351 0.994
95.735 1.003 70.214 0.98 92.12
ENSMUSG00000020423 Btg2 protein_coding 1.057 32.873 0.889
12.804 1.059 73.428 1.056 78.379
ENSMUSG00000020424 Gatsl3 protein_coding 1.02 53.809
0.921 75.4 1.067 65.034 1.023 95.74
ENSMUSG00000020427 Igfbp3 protein_coding 0.781 1.15 0.871
82.503 1.467 6.449 0.841 85.493
ENSMUSG00000020428 Gabra6 protein_coding 1 85.092 1
96.001 1 79.524 1 95.877
ENSMUSG00000020429 Igfbp1 protein_coding 1 82.231 1
85.671 1 57.227 1 95.877
ENSMUSG00000020430 Pes1 protein_coding 1.02 85.203 0.96
68.115 1.02 58.414 1.035 50.452
ENSMUSG00000020431 Adcy1 protein_coding 0.886 55.641 1.006
91.361 1.121 19.452 0.951 96.287
ENSMUSG00000020432 Tcn2 protein_coding 1.094 7.083 0.869 59.946
1.049 71.839 0.907 85.493
ENSMUSG00000020434 4921536K21Rik protein_coding 0.682 5.544
1.497 66.01 0.978 88.203 0.921 86.699
ENSMUSG00000020435 Osbp2 protein_coding 1.205 11.316 0.891
82.671 0.928 52.087 0.931 94.608
ENSMUSG00000020436 Gabrg2 protein_coding 1 84.049 1
92.793 1 88.657 1 96.071
ENSMUSG00000020437 Myo1g protein_coding 1.457 0 0.88 82.671
0.777 4.759 1.105 68.696
ENSMUSG00000020439 Smtn protein_coding 0.843 2.142 1.12 83.104
1.056 84.724 1.013 92.018
ENSMUSG00000020440 Arf5 protein_coding 0.988 80.751 0.981
95.088 1.029 67.562 1.057 69.918
ENSMUSG00000020441 2310033P09Rik protein_coding 0.977 70.862

					1.062	89.014	0.965	63.075	0.935	83.724
ENSMUSG00000020444	Guk1	protein_coding	1.001	64.401	0.918	37.187	1.09	27.533	1.045	92.691
ENSMUSG00000020447	Npc1l1	protein_coding	0.999	85.203	0.881	92.355	1.137	70.9	0.665	85.493
ENSMUSG00000020448	Rnf185	protein_coding	1.106	0.355	NA	NA	0.882	6.858	1.04	93.734
ENSMUSG00000020451	Limk2	protein_coding	0.898	11.519	0.922	82.671	1.203	10.76	1.026	93.964
ENSMUSG00000020453	Patz1	protein_coding	0.985	40.217	0.983	95.735	1.034	69.728	0.948	91.372
ENSMUSG00000020454	Eif4enif1	protein_coding	1.044	16.436	0.996	93.654	0.961	30.024	1.03	69.708
ENSMUSG00000020455	Trim11	protein_coding	0.956	21.658	0.969	82.503	1.081	10.819	1.028	75.81
ENSMUSG00000020456	Ogdh	protein_coding	0.957	26.483	1.016	89.434	1.028	66.647	1.008	86.118
ENSMUSG00000020457	Drg1	protein_coding	1.04	31.732	0.955	24.911	1.005	85.792	1.054	73.823
ENSMUSG00000020458	Rtn4	protein_coding	1.028	67.374	NA	NA	0.936	59.383	0.987	90.628
ENSMUSG00000020459	Mtif2	protein_coding	1.015	78.428	1.011	96.843	0.971	69.974	0.999	93.612
ENSMUSG00000020460	Rps27a	protein_coding	1.093	11.197	0.925	44.268	NA	NA	1.157	31.133
ENSMUSG00000020461	Clhc1	protein_coding	1.097	82.352	0.822	96.013	1.107	87.37	0.815	88.505
ENSMUSG00000020462	Ccdc104	protein_coding	0.998	75.567	1.006	96.407	NA	NA	1.1	40.729
ENSMUSG00000020463	Smek2	protein_coding	1.067	18.314	NA	NA	0.956	24.163	0.969	86.991
ENSMUSG00000020464	Pnpt1	protein_coding	1.001	84.452	1.017	85.756	0.979	60.614	1.05	70.539
ENSMUSG00000020467	Efemp1	protein_coding	1.282	0	0.866	54.06	0.898	14.583	1.245	86.133
ENSMUSG00000020469	Myl7	protein_coding	1	55.789	1	95.504	1	87.971	1	95.74
ENSMUSG00000020471	Pold2	protein_coding	0.912	23.923	1.118	67.843	0.978	70.214	1.053	72.204
ENSMUSG00000020472	Zkscan17	protein_coding	0.994	85.092	0.879	71.98	1.14	34.07	1	86.699
ENSMUSG00000020473	Aebp1	protein_coding	0.933	56.242	1.114	77.598	0.959	52.087	1.023	93.457
ENSMUSG00000020474	Polm	protein_coding	0.987	66.897	0.958	87.857	1.059	51.851	0.879	86.699
ENSMUSG00000020475	Pgam2	protein_coding	5.843	7.179	0.902	91.584	0.19	0.879	1.09	94.904
ENSMUSG00000020476	Dbnl	protein_coding	0.959	51.056	1.007	96.843	1.037	68.643	1.151	55.485
ENSMUSG00000020477	Mrps24	protein_coding	0.959	45.229	1.051	95.735	0.99	73.309	1.04	91.335
ENSMUSG00000020481	Ankrd36	protein_coding	1	82.982	1	93.707	1	60.233	1	96.358
ENSMUSG00000020482	Ccdc117	protein_coding	1.076	20.942						

1.092 75.612 0.851 0.72 1.025 92.903
ENSMUSG00000020483 Dynll2 protein_coding 0.922 21.957
1.062 82.904 1.02 72.148 0.974 93.264
ENSMUSG00000020484 Xbp1 protein_coding 1.036 57.38 1.06 79.29
0.911 7.155 1.013 95.273
ENSMUSG00000020485 Supt4a protein_coding 0.962 65.106
0.964 84.594 1.08 28.603 0.962 90.289
ENSMUSG00000020486 4-Sep protein_coding 0.854 19.023 0.906
95.301 1.289 25.966 0.968 84.29
ENSMUSG00000020490 Btnl10 protein_coding 1 83.658 1
94.597 1 84.308 1 96.287
ENSMUSG00000020491 2810021J22Rik protein_coding 1.02 65.284
0.958 77.598 1.022 65.8 1.015 85.786
ENSMUSG00000020492 Ska2 protein_coding 1.09 13.492 0.906
84.594 1.013 88.461 1.059 67.691
ENSMUSG00000020493 Prr11 protein_coding 1.313 31.24 NA NA
0.706 35.022 1.278 80.324
ENSMUSG00000020495 Smg8 protein_coding 1.087 9.983 1.033 95.088
0.892 3.123 1.005 95.35
ENSMUSG00000020496 Rnf187 protein_coding 0.901 4.099 1.063
91.456 1.041 24.07 1.044 92.381
ENSMUSG00000020513 Tubd1 protein_coding 0.873 49.944 1.01
95.301 1.132 25.564 1.103 49.806
ENSMUSG00000020514 Mrpl22 protein_coding 0.976 58.802
0.966 86.277 1.06 43.481 1.038 75.81
ENSMUSG00000020515 Cnot8 protein_coding 1.036 11.928 NA NA
0.965 23.938 1.065 80.77
ENSMUSG00000020516 Rps6kb1 protein_coding 1.001 83.333 NA
NA 1.01 84.961 1.036 79.268
ENSMUSG00000020519 Sap30l protein_coding 0.914 13.74 0.956
90.712 1.147 8.647 0.96 86.699
ENSMUSG00000020520 Galnt10 protein_coding 1.207 1.913 0.89
85.95 0.927 4.23 1.019 90.463
ENSMUSG00000020521 Rnft1 protein_coding 1.134 0.2 0.961 75.618
0.915 9.456 1.007 96.183
ENSMUSG00000020522 Mfap3 protein_coding 1.109 0 1.018 83.104
0.885 0.72 0.991 93.264
ENSMUSG00000020523 Fam114a2 protein_coding 0.99 85.655
0.989 94.597 1.018 88.693 1.065 72.369
ENSMUSG00000020524 Gria1 protein_coding 0.996 64.193 1.426
79.055 0.706 18.797 1.114 96.071
ENSMUSG00000020525 Ppm1d protein_coding 0.957 38.983 0.963
90.256 1.086 40.875 1.033 86.301
ENSMUSG00000020526 Znhit3 protein_coding 0.919 62.888 NA
NA 0.998 55.352 1.043 82.85
ENSMUSG00000020527 Myo19 protein_coding 0.871 8.139 1.016 85.378
1.129 36.578 1.141 57.1
ENSMUSG00000020528 Prpsap2 protein_coding 1.005 86.918
0.961 85.418 1.037 55.352 0.986 95.832
ENSMUSG00000020530 Ggnbp2 protein_coding 1.028 63.534
0.926 59.946 1.051 23.41 1.035 74.193
ENSMUSG00000020532 Acaca protein_coding 0.354 1.031 1.136 84.768
2.496 6.202 0.832 94.001
ENSMUSG00000020534 Shmt1 protein_coding 0.987 85.655 0.901

79.475 1.126 51.596 0.972 91.625
ENSMUSG00000020536 Llgl1 protein_coding 0.955 62.158 1.033
95.211 1.012 53.134 1.041 92.381
ENSMUSG00000020537 Drg2 protein_coding 0.948 11.519 1.018
92.111 1.038 43.307 1.021 79.713
ENSMUSG00000020538 Srebf1 protein_coding 0.816 7.004 1.129
71.617 1.085 69.728 1.041 92.381
ENSMUSG00000020541 Tom1l1 protein_coding 0.986 69.715
0.968 95.211 1.046 58.414 0.942 91.65
ENSMUSG00000020542 Myocd protein_coding 1.324 35.787 1.225
83.104 0.616 13.202 0.765 83.724
ENSMUSG00000020544 Cox11 protein_coding 0.95 85.391 1.093
88.923 0.964 78.045 0.936 87.705
ENSMUSG00000020545 Lrrc72 protein_coding 1 75.671 1
91.361 1 68.643 1 94.904
ENSMUSG00000020546 Stxbp4 protein_coding 0.947 74.8 1.022
95.735 1.035 86.831 1.07 67.691
ENSMUSG00000020547 Bzw2 protein_coding 1.046 74.147 0.914
82.848 1.048 63.301 1.11 48.119
ENSMUSG00000020548 1700086D15Rik protein_coding 1 83.523
1 95.431 1 88.601 1 94.904
ENSMUSG00000020549 Elac2 protein_coding 1.004 71.36 0.975 86.861
1.019 49.601 0.931 85.493
ENSMUSG00000020553 Pctp protein_coding 1.012 52.799 0.96
75.993 1.03 85.935 1.036 95.74
ENSMUSG00000020561 Twistnb protein_coding 1.027 43.939
0.997 85.793 0.973 86.112 1.117 76.046
ENSMUSG00000020562 Efcab10 protein_coding 1 76.686 1
96.407 1 84.669 1 95.35
ENSMUSG00000020564 Atxn7l1 protein_coding 1.088 12.072
1.027 81.039 0.894 1.764 0.972 91.372
ENSMUSG00000020566 Atp6v1c2 protein_coding 1.224 23.493
1.017 81.284 0.804 87.431 0.906 95.35
ENSMUSG00000020570 Sypl protein_coding 1.029 81.52 1.025 93.994
0.949 55.9 0.981 85.493
ENSMUSG00000020571 Pdia6 protein_coding 0.877 26.483 1.051
92.561 1.084 31.4 1.121 42.736
ENSMUSG00000020572 Nampt protein_coding 0.816 52.726 0.917
91.661 1.338 32.776 1.113 59.394
ENSMUSG00000020573 Pik3cg protein_coding 1.138 60.435
0.803 70.971 1.094 48.935 0.951 86.991
ENSMUSG00000020576 Nbas protein_coding 0.969 49.944 1.014
90.454 1.015 76.754 1.036 61.984
ENSMUSG00000020577 Tspan13 protein_coding 1.142 1.161 1.033
92.062 0.846 1.342 1.046 93.457
ENSMUSG00000020580 Rock2 protein_coding 0.944 41.33 0.985 96.68
1.074 66.647 1.133 72.826
ENSMUSG00000020583 Matn3 protein_coding 1 80.146 1
95.504 1 76.225 1 89.72
ENSMUSG00000020585 Laptm4a protein_coding 1.017 41.211 0.95
90.287 1.033 58.775 1.001 96.183
ENSMUSG00000020589 Fam49a protein_coding 1.012 82.705
1.074 92.111 0.921 73.874 1.085 95.877
ENSMUSG00000020590 Snx13 protein_coding 1.053 41.454 0.981

79.807 0.967 87.744 0.994 91.807
ENSMUSG00000020591 Ntsr2 protein_coding 0.353 0.987 1.114 84.007
2.55 6.449 1.439 60.31
ENSMUSG00000020592 Sdc1 protein_coding 0.982 86.918 0.97
86.728 1.052 62.648 1.239 52.324
ENSMUSG00000020593 Lpin1 protein_coding 0.835 45.894 0.878
70.29 1.367 20.886 1.143 86.209
ENSMUSG00000020594 Pum2 protein_coding 1.039 31.24 0.998 87.313
0.964 80.163 0.96 86.699
ENSMUSG00000020598 Nrcam protein_coding 1.358 17.492 0.915
81.443 0.806 46.956 0.981 96.192
ENSMUSG00000020599 Rgs9 protein_coding 1.32 24.253 0.91
91.897 0.833 52.972 0.704 83.724
ENSMUSG00000020601 Trib2 protein_coding 0.915 31.333 0.906
55.71 1.203 10.523 0.907 83.724
ENSMUSG00000020604 Arsg protein_coding 1.281 0.999 0.937 87.919
0.835 1.764 0.998 95.877
ENSMUSG00000020605 Hs1bp3 protein_coding 1.15 4.825 0.998
93.654 0.869 6.858 0.912 84.29
ENSMUSG00000020607 Fam84a protein_coding 0.81 28.514
0.851 94.262 1.447 30.292 1.138 85.493
ENSMUSG00000020608 Smc6 protein_coding 1.138 0.976 0.937 68.115
0.932 12.571 1.089 72.826
ENSMUSG00000020610 Amz2 protein_coding 1.021 51.826 1.025
84.126 0.95 28.489 0.971 92.31
ENSMUSG00000020611 Gna13 protein_coding 1.076 0.976 0.995 93.908
0.934 4.389 0.99 85.493
ENSMUSG00000020612 Prkar1a protein_coding 1.053 32.238
0.945 73.766 NA NA 1.015 57.952
ENSMUSG00000020614 Fam20a protein_coding 1.3 3.598 0.848
60.678 0.907 45.799 1.063 87.088
ENSMUSG00000020617 1700012B07Rik protein_coding 1 83.523
1 95.708 1 74.535 1 86.699
ENSMUSG00000020620 Abca8b protein_coding 1.061 72.372
0.842 60.678 1.122 26.663 1.002 92.748
ENSMUSG00000020621 Rdh14 protein_coding 1.013 80.815 0.977
85.116 1.012 86.602 0.974 95.405
ENSMUSG00000020623 Map2k6 protein_coding 1.252 30.788
0.989 90.454 0.808 10.003 0.964 83.067
ENSMUSG00000020624 4933434M16Rik lincRNA 1 86.333 1
96.407 1 85.757 1 93.156
ENSMUSG00000020627 Klhl29 protein_coding 1.047 69.408
0.929 81.02 1.025 80.381 0.926 88.245
ENSMUSG00000020628 Trappc12 protein_coding 1.008 70.862 0.98
91.361 1.01 61.418 1.01 74.193
ENSMUSG00000020629 Adi1 protein_coding 0.93 9.841 1.075 71.617
0.997 85.935 1.005 90.865
ENSMUSG00000020630 Rnaseh1 protein_coding 0.92 12.436 0.95
86.604 1.14 6.916 1.11 64.513
ENSMUSG00000020633 Dcdc2c protein_coding 0.863 84.324
0.979 92.355 1.178 76.923 1.047 94.654
ENSMUSG00000020634 Ubxn2a protein_coding 1.062 72.655
1.021 86.34 0.923 29.451 1.013 89.498
ENSMUSG00000020635 Fkbp1b protein_coding 1.822 0 0.884

87.452 0.618 0.72 0.928 84.29
ENSMUSG00000020636 Allc protein_coding 0.561 69.715 NA NA
1.495 69.196 0.891 88.387
ENSMUSG00000020638 Cmpk2 protein_coding 0.976 63.385 1.162
82.904 0.878 15.376 1.155 83.276
ENSMUSG00000020639 Pfn4 protein_coding 1.293 24.689 0.808
88.705 0.958 66.201 0.55 83.724
ENSMUSG00000020640 Itsn2 protein_coding 1.025 66.944 0.941
65.165 1.031 51.851 0.996 89.892
ENSMUSG00000020641 Rsad2 protein_coding 1.469 0.355 0.866 92.631
0.783 3.718 1.065 88.197
ENSMUSG00000020642 Rnf144a protein_coding 1.072 50.913
0.995 96.843 0.934 41.928 0.909 85.636
ENSMUSG00000020644 Id2 protein_coding 1.018 50.111 1.031
92.931 0.954 33.971 0.98 90.95
ENSMUSG00000020646 Mboat2 protein_coding 1.078 67.163
0.948 92.355 0.975 82.31 1.123 86.445
ENSMUSG00000020647 Ncoa1 protein_coding 1.051 30.788 0.989
96.216 0.963 22.279 1.057 93.016
ENSMUSG00000020648 Dus4l protein_coding 0.982 66.897 NA NA
1.118 77.17 0.964 95.74
ENSMUSG00000020649 Rrm2 protein_coding 1.162 67.163 1.031
94.887 0.837 85.675 1.702 57.1
ENSMUSG00000020650 Bcap29 protein_coding 1.129 1.949 0.969
83.58 0.909 5.884 0.98 88.505
ENSMUSG00000020651 Slc26a4 protein_coding 1 80.251 1
93.908 1 80.634 1 95.559
ENSMUSG00000020652 Cenpo protein_coding 0.902 38.286 1.022
93.908 1.083 38.926 0.973 84.29
ENSMUSG00000020653 Klf11 protein_coding 1.071 25.026 0.847
15.938 1.101 28.979 0.989 85.493
ENSMUSG00000020654 Adcy3 protein_coding 1.083 77.497 0.922
94.96 1.001 88.274 0.911 88.245
ENSMUSG00000020656 Grhl1 protein_coding 0.853 18.795 0.908
74.633 1.286 9.334 1.013 90.611
ENSMUSG00000020657 Dnajc27 protein_coding 1.119 10.464
1.064 85.378 0.837 6.515 1.067 91.492
ENSMUSG00000020658 Efr3b protein_coding 1.076 39.075 1.219
83.104 0.76 11.116 0.794 83.724
ENSMUSG00000020659 Cbll1 protein_coding 1.06 8.847 1.001 91.955
0.939 15.904 0.997 95.72
ENSMUSG00000020660 Pomc protein_coding 0.771 37.82 1.163 89.434
1.114 84.83 1.188 92.381
ENSMUSG00000020661 Dnmt3a protein_coding 1.005 74.689
1.018 83.104 0.974 66.753 0.914 88.505
ENSMUSG00000020664 Dld protein_coding 0.903 27.968 0.946
92.631 1.171 26.978 1.088 92.762
ENSMUSG00000020668 Kif3c protein_coding 0.891 7.004 1.1 71.617
NA NA 1.012 87.735
ENSMUSG00000020669 Sh3yl1 protein_coding 0.989 58.802
0.965 87.452 1.044 21.705 0.839 83.724
ENSMUSG00000020671 Rab10 protein_coding 1.049 17.191 1.013
83.104 0.938 6.515 1.004 79.268
ENSMUSG00000020672 Sntg2 protein_coding 1.348 3.152 0.766 87.857

0.967 39.245 0.964 96.183
ENSMUSG00000020674 Pxdn protein_coding 0.996 72 0.925 87.041
1.083 80.381 0.978 85.493
ENSMUSG00000020676 Ccl11 protein_coding 1.263 3.498 1.007 94.102
0.785 7.155 1.003 95.676
ENSMUSG00000020677 Ddx52 protein_coding 1.039 25.421 0.977
74.483 0.981 78.642 0.925 93.685
ENSMUSG00000020680 Taf15 protein_coding 0.888 21.176 0.901
21.916 1.25 5.864 0.989 96.192
ENSMUSG00000020681 Ace protein_coding 0.905 28.514 0.97
89.509 1.135 27.732 1.004 93.045
ENSMUSG00000020682 Mmp28 protein_coding 0.872 12.181 0.965
92.404 1.187 44.125 0.954 93.584
ENSMUSG00000020684 Rasl10b protein_coding 1.151 57.133 NA
NA 0.963 85.58 0.797 85.493
ENSMUSG00000020686 Gas2l2 protein_coding 0.576 45.502
2.059 81.127 0.844 52.972 0.687 86.254
ENSMUSG00000020687 Cdc27 protein_coding 0.988 86.351 1.024
97.129 0.99 79.776 1.024 91.492
ENSMUSG00000020689 Itgb3 protein_coding 1.035 40.88 1.063 97.049
0.909 27.321 1.289 42.736
ENSMUSG00000020690 Efcab3 protein_coding 0.905 82.092
1.457 96.308 0.76 73.096 0.923 96.192
ENSMUSG00000020691 Mettl2 protein_coding 0.963 32.749
0.935 96.216 1.109 58.051 0.992 93.734
ENSMUSG00000020692 Nle1 protein_coding 0.958 83.09 NA NA
1.053 86.258 1.012 94.99
ENSMUSG00000020694 Tlk2 protein_coding 1.018 63.804 0.984
91.275 0.998 88.601 1.004 93.457
ENSMUSG00000020695 Mrc2 protein_coding 1.197 1.242 1.008 96.763
0.826 4.759 1.247 38.598
ENSMUSG00000020696 Rffl protein_coding 0.978 47.677 1.069
75.62 0.957 66.753 0.983 90.611
ENSMUSG00000020697 Lig3 protein_coding 1.02 51.297 0.975
83.58 1.007 86.475 0.989 92.561
ENSMUSG00000020698 Cct6b protein_coding 1.101 69.089 0.706
90.256 1.286 78.864 1.261 95.273
ENSMUSG00000020700 Map3k3 protein_coding 1.039 30.282
0.993 93.654 0.967 53.7 0.963 84.19
ENSMUSG00000020701 Tmem132e protein_coding 1.136 69.777
1.061 84.832 0.831 23.212 1.229 72.204
ENSMUSG00000020703 5530401A14Rik protein_coding 1 84.661
1 96.013 1 74.535 1 93.964
ENSMUSG00000020704 Asic2 protein_coding 0.447 37.248 1.384
93.863 1.614 57.227 1.541 60.466
ENSMUSG00000020705 Ddx42 protein_coding 1.013 69.489 0.945
15.364 1.046 8.209 1.01 88.197
ENSMUSG00000020706 Ftsj3 protein_coding 1.011 77.113 0.954
65.165 1.038 45.449 1.109 42.736
ENSMUSG00000020707 Rnf135 protein_coding 0.999 52.799
1.004 87.452 NA NA 1.012 92.018
ENSMUSG00000020708 Psmc5 protein_coding 0.864 0.987 1.023 94.767
1.132 5.864 1.127 0
ENSMUSG00000020709 Adap2 protein_coding 1.096 35.748 0.82

39.547 1.107 65.235 1.032 96.287
ENSMUSG00000020712 Tcam1 protein_coding 1.3 29.596 0.573
59.396 1.346 55.512 1.065 90.157
ENSMUSG00000020713 Gh protein_coding 1 80.751 1
97.129 1 76.498 1 93.627
ENSMUSG00000020715 Ern1 protein_coding 1.472 8.365 0.909 81.284
0.748 37.617 1.19 59.394
ENSMUSG00000020716 Nf1 protein_coding 0.973 75.761 0.953
85.671 1.074 48.935 1.026 94.99
ENSMUSG00000020717 Pecam1 protein_coding 0.962 65.388 0.97
90.256 1.068 44.125 1.006 89.587
ENSMUSG00000020718 Polg2 protein_coding 0.954 47.174 0.98
93.046 1.064 30.292 0.919 84.29
ENSMUSG00000020719 Ddx5 protein_coding 0.979 52.258 0.977
95.329 1.043 44.787 1.003 83.581
ENSMUSG00000020720 Psmd12 protein_coding 0.992 83.09 0.967
91.532 1.046 69.315 1.05 53.923
ENSMUSG00000020721 Helz protein_coding 0.93 3.401 0.964 92.474
1.114 15.049 0.94 83.724
ENSMUSG00000020722 Cacng1 protein_coding 8.133 3.393 3.106
91.584 0.04 0.72 0.881 93.045
ENSMUSG00000020723 Cacng4 protein_coding 0.955 86.57 1.248
96.843 0.841 86.985 1.146 67.169
ENSMUSG00000020728 Cep112 protein_coding 1.119 2.512 0.917
61.883 0.976 50.523 1.154 91.872
ENSMUSG00000020732 Rab37 protein_coding 1.994 0.355 0.631 50.664
0.796 14.788 0.919 96.183
ENSMUSG00000020733 Slc9a3r1 protein_coding 1.027 70.121
0.955 73.766 1.022 79.625 0.963 93.264
ENSMUSG00000020736 Nt5c protein_coding 0.882 4.895 1.051 96.897
1.075 20.556 1.087 36.174
ENSMUSG00000020737 Hn1 protein_coding 0.966 54.176 0.967
86.538 1.072 36.578 1.085 56.079
ENSMUSG00000020738 Sumo2 protein_coding 1.024 41.211 1.007
93.899 0.971 28.669 1.067 40.426
ENSMUSG00000020739 Nup85 protein_coding 1.011 85.832 1.007
96.843 0.981 87.431 1.1 38.624
ENSMUSG00000020740 Gga3 protein_coding 1.012 72.85 1.11 79.367
0.888 8.313 0.998 94.904
ENSMUSG00000020741 Cluh protein_coding 0.957 85.655 1.048
82.904 0.994 47.641 0.894 83.724
ENSMUSG00000020743 Mif4gd protein_coding 0.965 72.546
0.993 96.68 1.044 80.634 1.01 92.748
ENSMUSG00000020744 Slc25a19 protein_coding 0.912 28.998
1.153 82.904 0.953 87.173 0.985 93.413
ENSMUSG00000020745 Pafah1b1 protein_coding 0.989 83.09 1.044
91.065 0.968 71.999 1.006 79.545
ENSMUSG00000020747 2310067B10Rik protein_coding 0.946 84.397
1.098 75.62 0.96 32.963 0.956 83.724
ENSMUSG00000020752 Recql5 protein_coding 0.929 2.009 1.026
94.848 1.05 8.93 0.978 94.016
ENSMUSG00000020755 Sap30bp protein_coding 1 82.732
1.003 97.155 1 87.744 0.956 87.542
ENSMUSG00000020758 Itgb4 protein_coding 0.951 55.641 0.961

91.661 1.093 44.361 0.953 90.289
ENSMUSG00000020766 Galk1 protein_coding 0.996 49.368 1.02
96.68 0.984 73.428 1.051 61.753
ENSMUSG00000020770 Unk protein_coding 0.931 19.985 1.02
93.899 1.055 30.561 0.995 92.742
ENSMUSG00000020773 Trim47 protein_coding 0.98 84.168
1.052 87.029 0.97 48.387 1.01 90.674
ENSMUSG00000020774 Aspa protein_coding 1.011 73.841 1.201
60.309 0.823 4.963 0.941 85.493
ENSMUSG00000020775 Mrpl38 protein_coding 0.912 10.014
1.059 85.501 1.037 40.875 0.987 87.705
ENSMUSG00000020776 Fbf1 protein_coding 0.813 0.987 0.977 95.301
1.261 6.202 0.983 89.827
ENSMUSG00000020777 Acox1 protein_coding 0.955 86.474 0.977
96.138 1.068 87.898 0.97 92.181
ENSMUSG00000020778 Ten1 protein_coding 1.028 75.858 0.931
84.017 1.046 43.307 0.95 93.316
ENSMUSG00000020780 Srp68 protein_coding 0.993 86.351 0.998
88.691 1.01 69.196 1.03 59.464
ENSMUSG00000020781 Tsen54 protein_coding 1.003 76.827
0.949 95.504 NA NA 1.006 95.273
ENSMUSG00000020782 Llgl2 protein_coding 0.888 18.907 1.088
84.126 1.036 57.907 0.946 83.724
ENSMUSG00000020783 1200014J11Rik protein_coding 1.057 4.228
0.957 53.434 0.989 38.534 1.029 92.181
ENSMUSG00000020785 Camkk1 protein_coding 1.159 32.411
1.082 96.68 0.8 21.117 1.198 65.401
ENSMUSG00000020787 P2rx1 protein_coding 1.001 42.255 1.007
83.876 NA NA 0.855 83.724
ENSMUSG00000020788 Atp2a3 protein_coding 1.255 0.461 0.781
34.058 1.023 81.525 1.007 96.184
ENSMUSG00000020790 Ankfy1 protein_coding 1.068 11.044
0.992 96.948 0.943 15.169 1.045 57.1
ENSMUSG00000020792 Exoc7 protein_coding 1.07 18.985 0.979
94.102 0.952 23.48 0.976 92.903
ENSMUSG00000020793 Galr2 protein_coding 0.58 9.508 1.259 72.546
1.37 86.831 1.424 81.901
ENSMUSG00000020794 Ube2g1 protein_coding 1.084 5.848 0.969
91.661 0.953 31.135 0.975 91.105
ENSMUSG00000020798 Spns3 protein_coding 0.727 7.58 1.315 61.943
1.048 85.464 0.763 83.724
ENSMUSG00000020799 Tekt1 protein_coding 0.743 54.176 NA NA
0.952 51.851 0.651 88.505
ENSMUSG00000020801 Med31 protein_coding 1.007 69.408 0.977
92.545 1.018 88.601 1.038 95.273
ENSMUSG00000020802 Ube2o protein_coding 0.955 65.199 1.02
92.931 1.029 84.896 1.007 87.886
ENSMUSG00000020803 Txndc17 protein_coding 1.062 74.147 NA
NA 0.97 72.464 1.002 83.067
ENSMUSG00000020804 Aanat protein_coding 0.364 76.686 2.4
95.431 NA NA 2.083 94.752
ENSMUSG00000020806 Rhbdf2 protein_coding 1.051 76.319
1.026 83.104 0.929 32.963 1.034 73.368
ENSMUSG00000020807 4933427D14Rik protein_coding 0.974 51.089

1.005 89.014 1.018 84.961 0.989 92.181
ENSMUSG00000020808 Fam64a protein_coding 1.043 81.76 0.948
88.881 NA NA 1.525 72.826
ENSMUSG00000020810 Cygb protein_coding 1.298 2.784 NA NA
0.785 6.858 0.874 86.699
ENSMUSG00000020811 Wscd1 protein_coding 1.324 20.799 NA NA
0.956 34.21 0.835 83.724
ENSMUSG00000020812 1810032O08Rik protein_coding 0.993 75.761
0.966 88.897 1.04 50.851 0.97 91.372
ENSMUSG00000020814 Mxra7 protein_coding 1.108 5.109 1.016 87.604
0.886 1.898 1.007 95.676
ENSMUSG00000020817 Rabep1 protein_coding 1.085 15.662
0.959 90.378 0.957 27.86 1.017 85.577
ENSMUSG00000020818 Mfsd11 protein_coding 1.104 6.76 0.977
90.287 0.928 12.948 0.994 92.181
ENSMUSG00000020821 Kif1c protein_coding 0.879 1.274 1.086 81.039
1.049 32.524 1.025 75.81
ENSMUSG00000020823 Sec14l1 protein_coding 1.061 41.951
0.878 28.822 1.076 17.412 1.031 83.067
ENSMUSG00000020826 Nos2 protein_coding 0.782 39.953 1.421
60.309 0.898 5.701 0.873 83.724
ENSMUSG00000020827 Mink1 protein_coding 0.969 75.567 1.046
91.643 0.986 69.445 0.974 83.724
ENSMUSG00000020828 Pld2 protein_coding 0.986 63.736 1
97.263 1.011 77.532 1.018 90.463
ENSMUSG00000020829 Slc46a1 protein_coding 0.941 17.705
1.121 60.309 0.947 38.434 0.895 83.724
ENSMUSG00000020830 Vmo1 protein_coding 0.627 62.437 1.28
95.088 1.242 48.772 1.186 94.904
ENSMUSG00000020831 0610010K14Rik protein_coding 0.908 76.396
1.045 93.994 NA NA 1.09 92.381
ENSMUSG00000020832 Eral1 protein_coding 0.975 28.584 NA NA
1.008 63.665 1.031 84.246
ENSMUSG00000020834 Dhrs13 protein_coding 0.896 37.531
1.038 96.216 1.071 53.882 1.068 87.134
ENSMUSG00000020836 Coro6 protein_coding 1.049 44.694 0.856
68.115 1.114 66.647 1.007 89.827
ENSMUSG00000020838 Slc6a4 protein_coding 1.387 3.152 1.038
94.102 0.696 19.449 1.156 96.248
ENSMUSG00000020839 Tmigd1 protein_coding 1.706 3.536 0.687
66.651 0.851 54.037 0.834 83.724
ENSMUSG00000020840 Blmh protein_coding 0.913 3.448 1.055 92.561
1.032 20.315 1.041 65.558
ENSMUSG00000020841 Cpd protein_coding 1.071 24.958 0.993
93.817 0.94 46.956 1.027 94.82
ENSMUSG00000020843 Timm22 protein_coding 0.961 45.728
1.017 93.707 1.021 61.743 1.002 96.192
ENSMUSG00000020844 Nxn protein_coding 1.087 22.025 0.963
74.633 0.956 84.434 1.199 32.698
ENSMUSG00000020846 Fam101b protein_coding 0.859 36.034
1.144 82.904 1.018 86.475 0.892 83.724
ENSMUSG00000020847 Rph3al protein_coding 1.055 84.294 0.92
82.073 1.032 64.305 0.933 94.608
ENSMUSG00000020848 Doc2b protein_coding 1.097 67.991 0.753

33.865	1.211 41.235	1.133 86.118
ENSMUSG00000020849	Ywhae protein_coding	1.051 82.732	0.977
89.713	0.976 85.352	1.051 32.045
ENSMUSG00000020850	Prpf8 protein_coding	0.98	69.985	1.053
66.695	0.967 19.449	0.959 84.29
ENSMUSG00000020857	Nme2	protein_coding	0.752 34.954	0.967
96.407	1.37	45.449	1.447 65.558
ENSMUSG00000020859	Spag9 protein_coding	0.979 51.56 0.972 84.594
1.05	18.208	1.017 87.7
ENSMUSG00000020863	Luc7l3	protein_coding	0.953 7.916 0.968
78.51 1.085 7.365 0.973 92.675
ENSMUSG00000020864	Ankrd40	protein_coding	0.876 3.623 1.147
71.617	NA	NA	1.119 83.067
ENSMUSG00000020865	Abcc3 protein_coding	1.102 58.38 0.886 81.443
1.019 81.976	1.027 76.347
ENSMUSG00000020866	Cacna1g	protein_coding	1.138 63.126
0.948 96.847	0.928 40.794	1.332 44.882
ENSMUSG00000020868	Xylt2 protein_coding	1.006 85.203	1.003
94.367	0.989 72.464	0.93	83.724
ENSMUSG00000020869	Lrrc59	protein_coding	0.924 35.449
1.094 73.092	0.99	68.82 1.206 69.965
ENSMUSG00000020871	Dlx4	protein_coding	1.124 55.124	0.876
90.053	1.016 78.498	1.039 90.125
ENSMUSG00000020873	Slc35b1	protein_coding	0.875 28.584
1.166 60.309	0.983 66.753	1.144 83.581
ENSMUSG00000020875	Hoxb9 protein_coding	1.046 35.748	0.988
89.277	0.969 87.744	1.174 83.067
ENSMUSG00000020876	Snx11 protein_coding	0.912 6.409 1.064 71.98
1.029 70.1	0.953 84.19
ENSMUSG00000020877	Scrn2 protein_coding	0.875 25.551	0.962
91.361	1.19	20.315	1.053 95.35
ENSMUSG00000020878	Lrrc46	protein_coding	0.856 66.406
0.884 95.708	1.327 65.235	1.194 83.067
ENSMUSG00000020882	Cacnb1	protein_coding	1.077 64.193
1.006 95.809	0.92	26.369	0.922 92.181
ENSMUSG00000020883	Fbxl20	protein_coding	0.933 7.118 0.933
79.475	1.143 8.339 0.953 83.724
ENSMUSG00000020884	Asgr1 protein_coding	0.689 76.135	0.499
87.313	2.919 30.948	4.608 56.863
ENSMUSG00000020886	Dlg4	protein_coding	0.873 32.557	0.983
94.887	1.162 38.099	1.123 72.992
ENSMUSG00000020888	Dvl2	protein_coding	0.805 23.923	1.088
90.454	1.14	38.926	0.968 86.699
ENSMUSG00000020889	Nr1d1 protein_coding	1.036 67.163	1.316
96.763	0.734 8.142 0.787 83.724
ENSMUSG00000020890	Gucy2e	protein_coding	0.626 30.136
1.511 79.411	1.056 87.103	1.092 84.499
ENSMUSG00000020891	Alox8 protein_coding	0.819 47.677	1.078
97.129	1.129 64.024	0.724 83.724
ENSMUSG00000020892	Aloxe3	protein_coding	0.893 74.299
1.183 97.263	0.949 86.144	0.907 85.493
ENSMUSG00000020893	Per1	protein_coding	1.054 84.738	0.894
85.921	1.062 63.665	0.963 96.086
ENSMUSG00000020894	Vamp2 protein_coding	1.004 63.804	1.074

64.304 0.928 5.453 0.944 84.29
ENSMUSG00000020895 Tmem107 protein_coding 0.967 84.661
1.036 95.708 NA NA 1.124 80.573
ENSMUSG00000020897 Aurkb protein_coding 2.058 11.044 0.623
45.961 0.781 75.353 1.2 81.061
ENSMUSG00000020898 Ctc1 protein_coding 0.918 23.923 1.039
97.288 1.046 21.522 0.98 96.183
ENSMUSG00000020899 Pfas protein_coding 0.985 64.014 0.982
88.881 1.032 65.034 1.006 96.183
ENSMUSG00000020900 Myh10 protein_coding 0.995 83.169 NA NA
1 88.657 0.97 89.475
ENSMUSG00000020901 Pik3r5 protein_coding 1.098 8.253 0.79 22
1.148 57.286 1.266 67.691
ENSMUSG00000020902 Ntn1 protein_coding 0.955 80.608 1.007
93.654 1.036 71.839 0.943 85.493
ENSMUSG00000020903 Stx8 protein_coding 1 82.732 1.009
94.848 0.992 85.58 1.103 42.736
ENSMUSG00000020905 Usp43 protein_coding 0.859 8.302 0.838 87.998
1.385 27.431 0.872 86.991
ENSMUSG00000020907 Rcvrn protein_coding 1.528 67.163 0.527
94.887 1.24 68.82 1.864 84.246
ENSMUSG00000020910 Adprm protein_coding 1.002 74.482 0.997
74.483 1 60.5 1.001 96.183
ENSMUSG00000020911 Krt19 protein_coding 0.921 55.641 0.978
85.242 1.108 36.95 0.988 93.734
ENSMUSG00000020912 Krt12 protein_coding 1.278 80.688 1.422
83.104 0.547 46.575 0.319 85.493
ENSMUSG00000020913 Krt24 protein_coding 1 81.01 1 97.263
1 88.693 1 95.877
ENSMUSG00000020914 Top2a protein_coding 1.243 61.725 0.875
71.889 0.918 85.464 1.321 60.968
ENSMUSG00000020916 Krt36 protein_coding 1.25 21.278 0.982
86.277 0.811 76.388 1.089 92.691
ENSMUSG00000020917 Acly protein_coding 0.39 0.987 1.253 83.104
2.042 6.693 0.778 96.358
ENSMUSG00000020918 Kat2a protein_coding 0.943 55.939 1.047
82.76 1.014 87.898 1.064 70.85
ENSMUSG00000020919 Stat5b protein_coding 1.107 3.176 1.012
90.29 0.891 1.898 1.06 92.748
ENSMUSG00000020921 Tmem101 protein_coding 0.976 55.641
0.983 90.053 1.04 50.091 0.989 91.625
ENSMUSG00000020922 Lsm12 protein_coding 1.047 15.662 1.075
71.617 0.886 0.945 0.931 84.19
ENSMUSG00000020923 Ubtf protein_coding 0.944 26.483 1.029
92.253 1.023 48.451 0.998 94.752
ENSMUSG00000020925 Ccdc43 protein_coding 1.035 72.372
1.034 91.643 0.936 49.453 1.02 85.786
ENSMUSG00000020926 Adam11 protein_coding 0.891 3.848 0.838
60.678 1.333 5.864 0.936 94.904
ENSMUSG00000020928 Higd1b protein_coding 0.812 48.036
1.029 84.594 1.2 10.079 0.978 96.086
ENSMUSG00000020929 Eftud2 protein_coding 0.984 64.284
0.999 93.817 1.016 51.596 1.058 44.679
ENSMUSG00000020930 Ccdc103 protein_coding 0.819 38.555

1.068 96.335 1.145 46.206 0.74 96.287
ENSMUSG00000020932 Gfap protein_coding 0.613 49.621 0.803
70.29 2.034 18.797 1.159 93.264
ENSMUSG00000020935 Dcakd protein_coding 1.047 49.517 0.977
90.378 0.974 76.64 1.035 79.268
ENSMUSG00000020936 Nmt1 protein_coding 0.955 9.752 0.984 88.705
1.061 21.522 1.053 44.679
ENSMUSG00000020937 Plcd3 protein_coding 0.83 12.181 1.15
72.642 1.045 65.8 0.92 83.724
ENSMUSG00000020940 1700023F06Rik protein_coding 0.921 48.354
0.887 96.763 1.228 61.743 0.802 85.493
ENSMUSG00000020941 Map3k14 protein_coding 1.006 58.896
1.119 81.127 0.885 14.409 0.92 84.29
ENSMUSG00000020946 Gosr2 protein_coding 1.006 66 NA NA
1.003 77.704 1.093 51.238
ENSMUSG00000020948 Klhl28 protein_coding 0.885 14.221
1.006 96.652 1.124 36.95 1.027 95.273
ENSMUSG00000020949 Fkbp3 protein_coding 1.043 20.296 1.002
95.329 0.959 23.1 1.066 75.81
ENSMUSG00000020950 Foxg1 protein_coding 1 85.655 1
88.705 1 79.554 1 95.273
ENSMUSG00000020952 Scfd1 protein_coding 1.01 81.125 1.039
90.092 0.955 53.224 1.036 74.193
ENSMUSG00000020953 Coch protein_coding 1.049 66 0.82 75.052
1.165 59.612 0.703 84.19
ENSMUSG00000020954 Strn3 protein_coding 1.114 0.2 0.96 96.897
0.935 0.72 1.007 96.248
ENSMUSG00000020955 NA NA NA NA NA NA 1.045 85.757
1.008 95.273
ENSMUSG00000020956 NA NA NA NA 1.042 83.104 0.982
61.288 1.032 71.653
ENSMUSG00000020961 Ston2 protein_coding 0.979 60.707 0.94
94.48 1.083 65.8 1.149 95.74
ENSMUSG00000020962 Gtf2a1 protein_coding 0.966 40.786
1.021 95.211 1.015 78.369 0.957 90.157
ENSMUSG00000020963 Tshr protein_coding 1.358 4.052 1.158 95.735
0.633 11.64 0.902 83.724
ENSMUSG00000020964 Sel1l protein_coding 0.997 45.229 0.941
60.678 1.064 12.831 0.992 93.457
ENSMUSG00000020973 Dnaaf2 protein_coding 1.014 82.732
1.004 94.102 0.984 86.346 0.936 84.29
ENSMUSG00000020974 Pole2 protein_coding 1.08 55.789 0.94
92.111 0.986 42.895 1.455 37.634
ENSMUSG00000020978 Klhdc2 protein_coding 1.058 13.8 0.933
59.396 1.012 78.369 0.95 83.952
ENSMUSG00000020982 Nemf protein_coding 1.002 83.333 NA NA
0.972 81.976 1.064 60.466
ENSMUSG00000020986 Sec23a protein_coding 1.039 25.735
1.049 79.367 0.919 6.858 1.02 60.31
ENSMUSG00000020988 L2hgdh protein_coding 0.988 85.637
1.087 90.895 0.933 41.928 0.962 85.493
ENSMUSG00000020990 Cdkl1 protein_coding 0.834 86.333 0.819
96.216 1.458 86.602 0.771 83.724
ENSMUSG00000020993 Trappc6b protein_coding 0.974 68.846

1.036 80.277 0.994 68.266 0.977 92.31
ENSMUSG00000020994 Pnn protein_coding 1.018 77.615 0.966
75.345 1.014 31.4 0.986 96.139
ENSMUSG00000021000 Ctage5 protein_coding 1.008 59.211
1.038 93.574 0.958 46.32 1.02 94.016
ENSMUSG00000021003 Galc protein_coding 1.105 11.044 0.951
81.731 0.952 33.402 1.013 92.181
ENSMUSG00000021007 Spata7 protein_coding 1.072 77.291
0.813 63.773 1.144 30.292 1.011 83.067
ENSMUSG00000021009 Ptpn21 protein_coding 0.978 82.092
0.992 91.776 1.033 82.575 1.038 95.273
ENSMUSG00000021010 Npas3 protein_coding 0.78 18.731 1.156
84.126 1.106 54.363 1.03 92.851
ENSMUSG00000021012 Zc3h14 protein_coding 1.088 2.352 0.979
69.611 0.934 37.103 1.013 92.748
ENSMUSG00000021013 Ttc8 protein_coding 1.001 86.172 1.007
96.407 0.994 73.874 1.026 90.397
ENSMUSG00000021018 Polr2h protein_coding 1.034 81.24 NA NA
NA NA 0.94 85.493
ENSMUSG00000021020 Srp54c protein_coding 0.819 14.403
1.049 97.129 1.16 54.363 1.35 40.426
ENSMUSG00000021022 Ppp2r3c protein_coding 1.082 12.549
1.018 92.545 0.904 17.662 0.96 86.445
ENSMUSG00000021023 1110008L16Rik protein_coding 1.002 74.482
0.975 95.211 1.025 80.089 0.984 96.358
ENSMUSG00000021024 Psma6 protein_coding 0.946 33.224 0.954
74.483 1.108 23.334 1.023 55.841
ENSMUSG00000021025 Nfkbia protein_coding 1.175 19.893
0.862 85.075 0.987 75.637 0.912 92.12
ENSMUSG00000021027 Ralgapa1 protein_coding 0.922 11.103
1.018 96.763 1.062 13.047 0.972 93.734
ENSMUSG00000021028 Mbip protein_coding 0.992 70.741 1.016
94.262 0.991 88.729 0.998 95.72
ENSMUSG00000021032 Ngb protein_coding 0.805 36.945 1.301
82.904 0.953 84.434 0.717 84.29
ENSMUSG00000021033 Gstz1 protein_coding 0.833 14.221 1.021
95.809 1.17 37.741 0.997 95.832
ENSMUSG00000021036 Sptlc2 protein_coding 1.135 2.897 0.965
64.123 0.911 57.091 1.048 38.624
ENSMUSG00000021037 Ahsa1 protein_coding 0.795 16.652 1.21
92.253 1.037 57.286 1.134 31.133
ENSMUSG00000021038 Vipas39 protein_coding 1.084 0.664 0.958
64.555 0.962 41.154 0.963 85.636
ENSMUSG00000021039 Snw1 protein_coding 1.022 64.956 0.959
64.123 1.024 54.363 1.036 50.74
ENSMUSG00000021040 Slirp protein_coding 0.925 45.229 1.005
95.35 1.071 48.304 1.061 83.067
ENSMUSG00000021041 2700073G19Rik protein_coding 0.763 28.242
1.191 91.776 1.1 62.648 1.002 96.086
ENSMUSG00000021044 Adck1 protein_coding 0.955 8.267 0.955 92.631
1.091 17.412 0.968 96.358
ENSMUSG00000021047 Nova1 protein_coding 1.022 84.865 0.926
76.431 1.056 61.743 0.936 86.991
ENSMUSG00000021048 Mthfd1 protein_coding 0.927 23.641

1.036 82.904 1.043 87.971 1.137 40.024
ENSMUSG00000021051 Ppp2r5e protein_coding 0.997 68.727
1.041 83.104 0.962 18.965 1.03 77.245
ENSMUSG00000021054 Sgpp1 protein_coding 1.238 0 0.932 50.715
0.867 1.48 1.059 78.948
ENSMUSG00000021055 Esr2 protein_coding 0.429 38.555 1.422
88.092 1.633 83.066 1.758 91.807
ENSMUSG00000021057 Akap5 protein_coding 1.34 47.466 1.216
83.464 0.612 4.759 0.712 83.724
ENSMUSG00000021061 Sptb protein_coding 1.243 12.549 0.987
96.407 0.811 7.742 0.851 88.505
ENSMUSG00000021062 Rab15 protein_coding 0.989 86.57 0.806 27.163
1.256 25.808 1.075 95.92
ENSMUSG00000021065 Fut8 protein_coding 1.19 24.253 0.976
91.361 0.861 19.765 1.056 54.153
ENSMUSG00000021066 Atl1 protein_coding 1.085 81.125 0.96
87.919 0.961 88.349 0.902 86.991
ENSMUSG00000021067 Sav1 protein_coding 0.934 59.674 NA NA
1.068 78.155 0.96 83.724
ENSMUSG00000021068 Nin protein_coding 1.066 74.689 NA NA
0.998 87.971 1.047 63.708
ENSMUSG00000021069 Pygl protein_coding 0.554 0.987 1.249 88.092
1.442 5.864 0.845 90.95
ENSMUSG00000021070 Bdkrb2 protein_coding 1.247 40.333 NA
NA 0.682 49.967 0.825 88.053
ENSMUSG00000021071 Trim9 protein_coding 1.422 22.025 1.028
86.538 0.685 65.902 1.601 0
ENSMUSG00000021072 Tmx1 protein_coding 1.096 8.281 0.958 85.671
0.954 27.321 1.005 92.381
ENSMUSG00000021076 Actr10 protein_coding 1.062 37.091
1.011 97.155 0.933 48.387 1.011 86.301
ENSMUSG00000021079 Timm9 protein_coding 0.967 49.749 1.008
95.504 1.023 45.208 1.036 85.577
ENSMUSG00000021081 Serpina1f protein_coding 1 84.865 NA
NA 1 86.666 1 95.74
ENSMUSG00000021087 Rtn1 protein_coding 1.147 23.019 0.993
95.211 0.875 18.336 0.891 83.724
ENSMUSG00000021090 Lrrc9 protein_coding 1.141 71.06 1.103 84.832
0.797 22.279 1.165 95.273
ENSMUSG00000021091 Serpina3n protein_coding 0.926 37.531 1.03
95.708 1.046 47.512 1.432 49.806
ENSMUSG00000021094 Dhrs7 protein_coding 0.75 1.422 1.104 85.756
1.212 32.524 1.009 93.156
ENSMUSG00000021095 Gsc protein_coding 1.637 2.651 0.795 83.392
0.769 6.858 0.899 86.991
ENSMUSG00000021096 Ppm1a protein_coding 1.008 67.991 0.984
93.654 1.004 80.749 0.982 91.625
ENSMUSG00000021097 Clmn protein_coding 0.867 19.023 0.904
88.345 1.274 38.152 0.94 83.724
ENSMUSG00000021098 4930447C04Rik protein_coding 1.523 10.464
0.796 68.115 0.827 52.213 0.795 85.493
ENSMUSG00000021099 Six6 protein_coding 1 81.52 1 97.155
1 84.308 1 96.183
ENSMUSG00000021102 Glrx5 protein_coding 0.885 24 1.066 92.111

1.058 63.301 0.957 85.493
ENSMUSG00000021103 Mnat1 protein_coding 1.123 14.59 0.865 42.923
1.032 61.418 0.984 93.964
ENSMUSG00000021108 Prkch protein_coding 1.127 2.569 0.973 87.919
0.914 6.68 0.997 85.493
ENSMUSG00000021109 Hif1a protein_coding 1.341 0 0.954 64.555
0.78 0.945 1.209 46.412
ENSMUSG00000021111 Papola protein_coding 1.015 85.283
0.996 96.897 0.987 80.163 0.99 93.316
ENSMUSG00000021112 Mpp5 protein_coding 1.004 73.734 0.966
84.594 1.034 80.089 1.017 83.276
ENSMUSG00000021113 Snapc1 protein_coding 1.097 13.492
1.047 92.561 0.872 6.858 0.932 84.29
ENSMUSG00000021114 Atp6v1d protein_coding 0.995 62.888
0.982 96.268 1.022 75.527 1 79.268
ENSMUSG00000021115 Vrk1 protein_coding 1.052 32.873 0.965
82.848 0.984 77.294 1.049 89.428
ENSMUSG00000021116 Eif2s1 protein_coding 1.089 48.288
0.948 88.705 0.967 78.045 1.044 48.119
ENSMUSG00000021118 Plek2 protein_coding 0.843 55.939 0.967
91.83 1.228 38.26 1.088 92.018
ENSMUSG00000021120 Pigh protein_coding 1.092 10.391 0.931
55.265 0.985 85.921 0.951 85.493
ENSMUSG00000021123 Rdh12 protein_coding 0.707 29.21 1.053 96.847
1.336 33.042 1.193 83.067
ENSMUSG00000021124 Vti1b protein_coding 0.963 58.077 1.053
89.434 0.988 87.431 1.019 95.676
ENSMUSG00000021125 Arg2 protein_coding 0.851 58.56 0.867 72.576
1.35 45.667 1.187 73.401
ENSMUSG00000021127 Zfp36l1 protein_coding 1.034 85.129 NA
NA 0.939 74.325 0.915 86.699
ENSMUSG00000021130 Galnt16 protein_coding 0.984 56.477
0.986 86.538 1.031 40.575 1.086 31.133
ENSMUSG00000021131 Erh protein_coding 1.056 74.745 0.938
68.848 1.011 55.352 1.136 31.133
ENSMUSG00000021133 4933426M11Rik protein_coding 1.158 4.916
0.914 73.461 0.945 28.901 0.939 83.724
ENSMUSG00000021134 Srsf5 protein_coding 0.977 84.452 0.959
55.71 1.062 17.662 1.023 92.742
ENSMUSG00000021136 Smoc1 protein_coding 0.946 37.531 1.014
95.504 1.044 49.384 0.954 93.316
ENSMUSG00000021139 Gm20498 protein_coding 1.214 75.671
0.984 95.708 0.839 71.388 0.549 85.493
ENSMUSG00000021140 Pcnx protein_coding 0.988 61.334 0.971
96.897 1.045 79.325 0.962 83.724
ENSMUSG00000021143 Pacs2 protein_coding 1.036 73.124 1.031
86.277 0.933 45.799 1.005 78.546
ENSMUSG00000021144 Mta1 protein_coding 1.05 15.85 0.972 95.708
0.98 14.262 1.013 95.72
ENSMUSG00000021147 Wdr37 protein_coding 1.05 7.501 0.98 91.532
0.969 28.901 1.026 86.133
ENSMUSG00000021148 Gm9745 protein_coding 1 84.168 1
95.088 1 78.369 1 94.825
ENSMUSG00000021149 Gtpbp4 protein_coding 1.128 31.477

0.932 61.883 0.948 87.431 1.043 74.193
ENSMUSG00000021156 Zmynd11 protein_coding 0.996 60.003
0.991 85.418 1.012 42.065 0.991 95.273
ENSMUSG00000021171 Esyt2 protein_coding 1.077 13.292 0.966
82.848 0.962 37.906 0.984 93.132
ENSMUSG00000021175 Cdca7l protein_coding 0.948 74.482
0.963 85.075 1.099 46.956 0.787 86.991
ENSMUSG00000021176 Efcab11 protein_coding 1.603 10.858
0.582 79.475 1.071 65.902 1.617 92.748
ENSMUSG00000021177 Tdp1 protein_coding 1.079 25.026 0.978
81.731 0.947 86.552 1.042 92.389
ENSMUSG00000021178 Psmc1 protein_coding 0.92 3.009 0.994 94.597
1.091 10.76 1.056 49.514
ENSMUSG00000021179 Nrde2 protein_coding 0.958 17.705 0.93
86.538 1.124 27.533 1 96.071
ENSMUSG00000021180 Rps6ka5 protein_coding 1.124 15.972
0.979 83.104 0.907 6.858 0.987 96.086
ENSMUSG00000021182 Ccdc88c protein_coding 0.855 17.705
1.094 85.756 1.065 49.384 0.889 83.724
ENSMUSG00000021185 9030617O03Rik protein_coding 1.118 20.942
1.143 84.832 0.779 6.858 0.985 79.268
ENSMUSG00000021186 Fbln5 protein_coding 1.266 0 0.909 52.085
0.866 20.751 0.921 90.805
ENSMUSG00000021187 Tc2n protein_coding 1.268 28.098 0.778
74.633 1.013 78.155 0.821 83.724
ENSMUSG00000021188 Trip11 protein_coding 0.959 48.036
0.966 91.661 1.082 44.515 1.011 91.783
ENSMUSG00000021189 Atxn3 protein_coding 1.017 59.806 1.011
87.919 0.971 86.258 1.025 85.577
ENSMUSG00000021190 Lgmn protein_coding 1.665 0 0.88 37.426
0.682 4.521 1.057 74.047
ENSMUSG00000021192 Golga5 protein_coding 0.932 31.11 1.011
96.013 1.063 27.732 1.032 57.711
ENSMUSG00000021193 Pitrm1 protein_coding 0.954 20.799
1.038 85.378 1.006 61.743 0.996 89.428
ENSMUSG00000021194 Chga protein_coding 0.894 65.199 0.791
91.955 1.412 41.331 1.85 76.467
ENSMUSG00000021196 Pfkp protein_coding 0.88 16.472 1.16
72.642 0.982 72.269 0.921 86.991
ENSMUSG00000021198 Unc79 protein_coding 1.263 29.372 0.968
95.504 0.818 30.024 0.969 89.892
ENSMUSG00000021200 Asb2 protein_coding 1.721 29.596 1.353
60.309 0.429 1.342 0.648 90.289
ENSMUSG00000021203 Otub2 protein_coding 1.125 23.254 1.159
84.007 0.768 14.788 0.928 85.493
ENSMUSG00000021206 D830046C22Rik protein_coding 1 76.968
NA NA NA NA 1 95.273
ENSMUSG00000021209 Ppp4r4 protein_coding 1.182 26.136
1.048 91.584 0.804 15.778 0.863 91.985
ENSMUSG00000021210 Akr1c6 protein_coding 1 86.819 1
96.138 1 86.985 1 94.904
ENSMUSG00000021211 Akr1c12 protein_coding 1.238 60.168
0.897 91.361 0.899 70.44 1.293 71.305
ENSMUSG00000021213 Akr1c13 protein_coding 1.373 0 0.737

21.315 0.99 83.925 1.026 81.251
ENSMUSG00000021214 Akr1c18 protein_coding 0.971 69.985
1.205 72.642 0.855 19.236 1.597 56.339
ENSMUSG00000021215 Net1 protein_coding 0.937 72.743 0.976
82.073 1.087 41.235 0.904 86.991
ENSMUSG00000021216 Tubal3 protein_coding 1 84.738 1
96.216 1 57.286 1 96.183
ENSMUSG00000021217 Tshz3 protein_coding 1.1 66.58 0.997 90.092
0.913 32.082 1.107 58.721
ENSMUSG00000021218 Gdi2 protein_coding 1.077 19.746 0.974
82.073 0.955 61.288 1.007 87.447
ENSMUSG00000021219 Rgs6 protein_coding 0.976 80.408 1.105
91.584 0.928 45.093 1.191 78.379
ENSMUSG00000021221 Dpf3 protein_coding 0.947 48.036 0.935
90.053 1.13 33.802 1.018 92.018
ENSMUSG00000021222 Dcaf4 protein_coding 1.076 56.311 NA NA
0.935 57.819 0.925 86.445
ENSMUSG00000021223 Papln protein_coding 0.866 22.237 1.041
96.216 1.108 33.042 0.97 86.991
ENSMUSG00000021224 Numb protein_coding 0.967 43.495 1.023
96.268 1.011 35.13 0.985 87.651
ENSMUSG00000021226 Acot2 protein_coding 1.092 61.725 0.931
96.652 0.983 69.134 1.027 83.581
ENSMUSG00000021228 Acot3 protein_coding 0.776 84.324 0.803
94.887 1.6 84.961 0.68 86.445
ENSMUSG00000021234 Fam161b protein_coding 0.992 61.334
1.127 78.705 0.895 38.534 0.976 94.654
ENSMUSG00000021235 Coq6 protein_coding 0.974 51.056 1.051
90.092 NA NA 0.954 84.29
ENSMUSG00000021236 Entpd5 protein_coding 0.931 33.002
1.029 90.116 1.046 48.451 1.014 93.457
ENSMUSG00000021238 Aldh6a1 protein_coding 1.155 12.855 0.95
95.708 0.912 28.237 0.832 83.724
ENSMUSG00000021239 Vsx2 protein_coding 1 82.092 1
87.452 1 67.163 1 86.301
ENSMUSG00000021240 Abcd4 protein_coding 0.986 61.334 1.007
95.211 1.009 81.492 0.992 92.903
ENSMUSG00000021241 Isca2 protein_coding 0.856 3.009 1.079 71.617
1.084 38.038 1.051 83.067
ENSMUSG00000021242 Npc2 protein_coding 1.043 72 0.932 67.513
1.024 54.363 0.987 89.525
ENSMUSG00000021243 Fcf1 protein_coding 1.036 75.196 0.987
95.211 0.977 88.601 0.95 94.654
ENSMUSG00000021244 Ylpm1 protein_coding 1.058 57.38 1.012 96.407
0.933 59.383 1.018 87.886
ENSMUSG00000021245 Mlh3 protein_coding 1.117 6.958 1.01 92.404
0.888 29.451 1.044 91.335
ENSMUSG00000021248 Tmed10 protein_coding 1.076 1.188 1
91.897 0.926 5.323 1.036 74.607
ENSMUSG00000021250 Fos protein_coding 0.704 25.3 1.183 96.843
1.203 36.95 1.162 78.042
ENSMUSG00000021252 0610007P14Rik protein_coding 1.054 37.941
1.049 82.904 0.904 7.329 1.036 68.502
ENSMUSG00000021253 Tgfb3 protein_coding 0.961 84.049 1.12

71.98 0.927 15.024 0.958 92.675
ENSMUSG00000021254 Gpatch2l protein_coding 1.059 37.702 NA
NA 0.935 33.137 1.022 79.268
ENSMUSG00000021255 Esrrb protein_coding 1.343 24.147 0.765
95.088 0.974 41.506 1.36 87.7
ENSMUSG00000021256 Vash1 protein_coding 0.838 31.333 1.204
64.123 0.992 86.084 1.587 57.1
ENSMUSG00000021257 Angel1 protein_coding 1.034 53.228
1.039 81.127 0.933 8.382 0.951 84.29
ENSMUSG00000021258 Ccnk protein_coding 1.118 0.461 0.975 91.361
0.92 4.338 0.978 91.372
ENSMUSG00000021259 Cyp46a1 protein_coding 1.031 30.984
0.848 63.619 1.139 58.414 1.108 93.264
ENSMUSG00000021260 Hhipl1 protein_coding 1.23 42.255
0.822 56.781 0.989 86.985 1.288 71.653
ENSMUSG00000021262 Evl protein_coding 1.135 6.399 0.986 88.705
0.894 20.009 1.11 50.74
ENSMUSG00000021263 Degs2 protein_coding 1.088 82.352 0.942
95.211 0.978 86.475 0.898 86.699
ENSMUSG00000021264 Yy1 protein_coding 1.086 13.292 1.021
97.155 0.9 8.142 0.93 83.724
ENSMUSG00000021265 Slc25a29 protein_coding 0.934 47.266 1.07
85.756 1.002 88.461 1.038 86.445
ENSMUSG00000021266 Wars protein_coding 0.992 71.674 1.034
93.707 0.976 86.084 1.001 96.358
ENSMUSG00000021268 Meg3 lincRNA 0.767 3.763 1.087 93.529 1.2
9.176 1.353 31.133
ENSMUSG00000021270 Hsp90aa1 protein_coding 0.925 76.968 1.17
97.263 0.922 82.778 1.098 57.839
ENSMUSG00000021271 Zfp839 protein_coding 1.014 85.655
0.954 73.766 1.033 40.875 0.945 88.552
ENSMUSG00000021273 Fdft1 protein_coding 1.049 18.388 1
94.597 0.95 44.678 1.014 93.016
ENSMUSG00000021275 Tecpr2 protein_coding 0.931 18.138
1.031 83.104 1.045 55.352 1.043 83.067
ENSMUSG00000021276 Cinp protein_coding 0.977 59.028 1.011
97.155 1.016 69.315 1.101 44.679
ENSMUSG00000021277 Traf3 protein_coding 1.062 35.748 1.073
71.617 0.874 4.723 0.981 95.676
ENSMUSG00000021278 Amn protein_coding 0.696 44.562 1.29
88.533 1.109 53.134 1.299 78.948
ENSMUSG00000021279 Cdc42bpb protein_coding 0.956 66.652
1.007 96.763 1.041 64.624 1.022 95.74
ENSMUSG00000021280 Exoc3l4 protein_coding 0.867 42.034 NA
NA 1.139 60.789 0.797 90.157
ENSMUSG00000021281 Tnfaip2 protein_coding 1.028 78.054
0.919 79.475 1.057 40.955 0.949 91.625
ENSMUSG00000021282 Eif5 protein_coding 0.974 86.74 0.993 85.075
1.034 45.667 1.056 70.539
ENSMUSG00000021285 Ppp1r13b protein_coding 0.999 59.527
1.006 89.996 0.995 84.071 1.017 93.316
ENSMUSG00000021286 Zfyve21 protein_coding 1.055 85.203
1.073 82.904 0.885 29.765 0.906 83.724
ENSMUSG00000021287 Xrcc3 protein_coding 1.052 83.263 0.935

95.504 1.017 72.148 0.772 83.724
ENSMUSG00000021288 Klc1 protein_coding 0.96 33.745 1.012
90.744 1.028 21.522 0.992 93.156
ENSMUSG00000021290 2010107E04Rik protein_coding 0.976 43.816
1.006 92.793 1.019 70.9 1.109 61.984
ENSMUSG00000021294 Kif26a protein_coding 0.739 13.239
1.075 95.431 1.254 30.107 0.914 90.157
ENSMUSG00000021298 Gpr132 protein_coding 1.109 19.315 NA
NA 0.771 41.662 0.813 83.724
ENSMUSG00000021301 Hecw1 protein_coding 1 85.728 1
95.708 1 78.293 1 92.748
ENSMUSG00000021302 Ggps1 protein_coding 1.061 19.477 1.14
60.309 0.828 0.72 0.964 86.991
ENSMUSG00000021303 Gng4 protein_coding 1.098 52.429 0.906
82.073 1.008 82.778 1.148 92.762
ENSMUSG00000021306 Gpr137b protein_coding 1.176 1.079 1.023
95.431 0.829 1.898 0.916 83.724
ENSMUSG00000021311 Mtr protein_coding 1.055 26.338 0.897
54.427 1.054 69.002 1.003 92.564
ENSMUSG00000021313 Ryr2 protein_coding 1.151 58.38 0.869 97.288
1.003 69.974 1.216 79.545
ENSMUSG00000021314 Amph protein_coding 1.125 75.671 1.301
86.34 0.68 27.675 0.808 85.493
ENSMUSG00000021318 Gli3 protein_coding 1.179 12.549 0.957
90.378 0.887 17.381 1.137 40.894
ENSMUSG00000021319 Sfrp4 protein_coding 0.995 71.06 1.267 82.904
0.792 13.643 1.13 51.238
ENSMUSG00000021322 Aoah protein_coding 1.434 1.967 0.868 80.052
0.8 28.901 1.177 83.276
ENSMUSG00000021326 Trim27 protein_coding 1.001 86.464
0.975 85.075 1.025 71.232 1.026 93.156
ENSMUSG00000021327 Zkscan3 protein_coding 1.041 29.596
1.049 93.899 0.915 33.137 0.965 90.289
ENSMUSG00000021336 Slc17a4 protein_coding 1 76.686 1
95.504 1 80.089 1 94.153
ENSMUSG00000021337 Scgn protein_coding 1 86.145 1
96.948 1 85.134 1 95.273
ENSMUSG00000021338 Lrrc16a protein_coding 1.1 30.788
0.928 79.807 0.981 73.874 1.027 93.964
ENSMUSG00000021339 Mrs2 protein_coding 0.962 52.9 1.02 93.574
1.017 84.896 0.947 86.991
ENSMUSG00000021340 Gpld1 protein_coding 0.889 14.849 1.023
83.104 1.095 61.743 0.971 87.182
ENSMUSG00000021342 Prl protein_coding 1 84.977 1
96.843 1 84.669 1 96.192
ENSMUSG00000021345 Prl8a6 protein_coding 1 84.819 1
95.301 1 84.724 1 94.904
ENSMUSG00000021346 Prl8a8 protein_coding 1 63.385 1
96.216 1 84.896 1 92.561
ENSMUSG00000021347 Prl7b1 protein_coding 1 86.351 1
91.955 1 83.066 1 91.985
ENSMUSG00000021348 Prl7d1 protein_coding 1 84.738 NA
NA 1 88.349 1 92.748
ENSMUSG00000021356 Irf4 protein_coding 1.087 74.745 0.926

79.807 0.99 68.129 1.192 83.067
ENSMUSG00000021357 Exoc2 protein_coding 0.982 77.113 NA NA
1.028 81.492 1.092 82.328
ENSMUSG00000021359 Tfap2a protein_coding 0.79 27.836
1.069 83.104 1.179 50.091 0.9 84.19
ENSMUSG00000021360 Gcnt2 protein_coding 1.209 2.512 0.934 61.883
0.887 27.321 0.951 91.372
ENSMUSG00000021361 Tmem14c protein_coding 0.942 23.13 0.987
93.146 1.073 61.841 1.028 83.581
ENSMUSG00000021362 Gcm2 protein_coding 1 82.732 1
96.216 1 84.626 1 95.92
ENSMUSG00000021363 Mak protein_coding 0.979 72.372 1.361
97.155 0.748 80.015 1.117 95.273
ENSMUSG00000021364 Elovl2 protein_coding 1.099 71.896 NA
NA 0.64 71.004 0.606 86.699
ENSMUSG00000021365 Nedd9 protein_coding 1.146 35.499 0.929
92.474 0.938 25.345 0.897 83.724
ENSMUSG00000021366 Hivep1 protein_coding 1.054 57.655
1.077 81.127 0.883 9.288 0.893 83.724
ENSMUSG00000021367 Edn1 protein_coding 1.018 72.27 0.817 20.272
1.203 5.977 1.116 88.797
ENSMUSG00000021368 Tbc1d7 protein_coding 0.957 47.266
1.083 85.756 0.967 87.898 1.06 70.539
ENSMUSG00000021371 Mcur1 protein_coding 0.948 54.176 1.112
60.309 0.945 6.858 1.012 94.654
ENSMUSG00000021373 Cap2 protein_coding 1.658 5.253 1.05 83.104
0.575 1.48 1.015 78.948
ENSMUSG00000021374 Nup153 protein_coding 1.063 5.109 1.017
83.104 0.925 6.202 1.008 96.071
ENSMUSG00000021375 Kif13a protein_coding 0.96 28.998
1.016 93.146 1.023 60.789 1.011 91.011
ENSMUSG00000021376 Tpmt protein_coding 0.834 21.658 0.938
95.088 1.274 27.047 1.037 87.134
ENSMUSG00000021377 Dek protein_coding 1.221 0 0.941 70.864
0.868 0.72 1.006 93.264
ENSMUSG00000021379 Id4 protein_coding 1.203 45.746 1.023
96.335 0.81 29.765 1.014 96.086
ENSMUSG00000021381 Barx1 protein_coding 1 75.761 1
96.216 1 87.786 1 83.276
ENSMUSG00000021384 Susd3 protein_coding 1.143 29.372 0.851
55.71 1.025 74.325 0.916 93.584
ENSMUSG00000021385 Ippk protein_coding 1.097 56.907 1.015
95.088 0.899 42.376 1.053 74.193
ENSMUSG00000021388 Aspn protein_coding 1.051 57.133 0.916
79.894 1.037 87.898 1.037 83.865
ENSMUSG00000021390 Ogn protein_coding 1.202 0 1.025 95.211
0.812 1.898 1.195 44.882
ENSMUSG00000021391 Cenpp protein_coding 1.479 7.799 0.871 81.284
0.776 37.617 1.163 95.35
ENSMUSG00000021392 Nol8 protein_coding 1.09 39.851 1.001
90.712 0.915 85.134 1.004 87.447
ENSMUSG00000021395 Spin1 protein_coding 1.12 0 0.925 79.807
0.965 5.323 0.963 83.724
ENSMUSG00000021396 Nxnl2 protein_coding 1.255 41.177 1.058

87.609 0.755 28.237 1.186 80.77
ENSMUSG00000021400 Wrnip1 protein_coding 0.997 83.418
1.015 91.643 0.99 64.427 0.908 83.724
ENSMUSG00000021403 Serpinb9b protein_coding 1.22 50.377 1.14
95.504 0.717 49.967 1.963 68.502
ENSMUSG00000021404 Serpinb9c protein_coding 1 85.655 NA
NA 1 85.675 1 94.82
ENSMUSG00000021408 Ripk1 protein_coding 1.183 7.083 0.973 88.881
0.869 8.472 0.983 93.156
ENSMUSG00000021411 Pxdc1 protein_coding 0.855 33.906 0.949
78.51 1.232 16.26 1.041 95.74
ENSMUSG00000021413 Prpf4b protein_coding 0.93 7.574 0.992
93.908 1.084 11.518 1.022 84.246
ENSMUSG00000021414 Fam217a protein_coding 1.014 69.089 NA
NA 0.91 76.64 1.08 92.389
ENSMUSG00000021415 4933417A18Rik protein_coding 0.61 11.128
0.993 93.046 1.648 7.635 0.87 92.52
ENSMUSG00000021416 Eci3 protein_coding 0.844 11.128 NA NA
1.189 28.979 0.864 92.31
ENSMUSG00000021417 Eci2 protein_coding 0.949 59.028 0.823
67.941 1.28 22.517 1.013 89.108
ENSMUSG00000021418 Rpp40 protein_coding 1.001 84.564 1.006
97.155 0.992 58.271 0.974 86.445
ENSMUSG00000021420 Fars2 protein_coding 1.051 72.85 0.955 94.48
0.995 86.666 0.98 90.611
ENSMUSG00000021423 Ly86 protein_coding 1.683 0 0.862 68.848
0.687 5.336 1.074 87.447
ENSMUSG00000021427 Ssr1 protein_coding 1.074 35.499 1.05
60.309 0.887 1.408 1.102 51.967
ENSMUSG00000021428 Riok1 protein_coding 1.111 22.349 0.874
12.804 1.027 30.213 1.058 46.846
ENSMUSG00000021431 Snrnp48 protein_coding 1.058 33.325
0.926 21.315 1.019 37.741 0.992 95.74
ENSMUSG00000021432 Slc35b3 protein_coding 0.944 33.599
1.007 93.654 1.054 37.764 1.057 91.872
ENSMUSG00000021439 Ctsq protein_coding 1 75.399 1
93.817 1 79.255 1 95.877
ENSMUSG00000021440 Cts7 protein_coding 1 86.199 NA NA
1 83.275 1 96.183
ENSMUSG00000021441 Cts6 protein_coding 1 81.01 1 97.263
1 85.675 1 77.245
ENSMUSG00000021448 Shc3 protein_coding 1.599 53.809 NA NA
0.567 42.621 0.825 92.181
ENSMUSG00000021451 Sema4d protein_coding 1.39 0.984 0.846
65.819 0.849 8.634 1.073 81.061
ENSMUSG00000021453 Gadd45g protein_coding 1.129 43.939 1.14
79.261 0.778 5.323 1.547 93.413
ENSMUSG00000021456 Fbp2 protein_coding 1.838 15.602 1.22
83.104 0.445 1.58 1.3 73.401
ENSMUSG00000021457 Syk protein_coding 1.329 0.461 0.874 58.64
0.862 58.271 1.042 93.964
ENSMUSG00000021458 2010111I01Rik protein_coding 0.962 27.968
0.987 96.984 1.056 36.95 0.984 96.358
ENSMUSG00000021460 Auh protein_coding 0.957 43.167 1.079

83.464	NA	NA	1.015 94.608
ENSMUSG00000021461	Fancc protein_coding	1.163 6.541 0.941 74.483
0.911 21.331	0.952 90.177
ENSMUSG00000021464	Ror2 protein_coding	1.265 18.985	0.942
90.378	0.839 25.688	1.16 44.882
ENSMUSG00000021466	Ptch1 protein_coding	0.854 19.023	1.072
76.065	1.088 55.512	0.945 83.724
ENSMUSG00000021468	Sptlc1	protein_coding	0.978 57.014
1.003 94.848	1.02 42.75 0.996 92.389
ENSMUSG00000021469	Msx2 protein_coding	0.565 8.302 1.104 82.904
1.606 25.425	1.043 95.832
ENSMUSG00000021470	Ercc6l2	protein_coding	1.081 18.556	NA
NA	0.901 16.129	0.983 92.748
ENSMUSG00000021474	Sfxn1 protein_coding	1.09 50.627	0.978
94.597	0.938 88.076	0.913 85.493
ENSMUSG00000021476	Habp4 protein_coding	0.999 76.135	0.985
95.708	1.013 81.032	0.976 94.468
ENSMUSG00000021477	Ctsl protein_coding	1.007 62.354	0.941
65.165	1.055 52.8 1.115 46.846
ENSMUSG00000021478	Drd1a protein_coding	1.806 18.635	0.47
39.056	1.172 57.286	0.6 83.724
ENSMUSG00000021481	Zfp346	protein_coding	1.043 10.687
0.955 73.766	1.007 88.076	0.976 94.654
ENSMUSG00000021482	Aaed1 protein_coding	1.091 21.703	0.97
97.049	0.947 17.381	0.987 85.493
ENSMUSG00000021483	Cdk20 protein_coding	1.032 59.806	1.026
95.088	0.946 81.62 0.978 92.851
ENSMUSG00000021484	Lman2 protein_coding	0.956 30.136	1.067
71.617	0.982 53.224	0.991 86.699
ENSMUSG00000021485	Mxd3 protein_coding	0.884 75.115	1.001
95.504	1.131 59.612	1.143 73.368
ENSMUSG00000021486	Prelid1	protein_coding	0.958 25.85 1.022
76.065	1.019 81.492	1.151 49.806
ENSMUSG00000021488	Nsd1 protein_coding	1.057 52.986	0.962
95.504	0.985 66.425	0.998 92.12
ENSMUSG00000021492	F12 protein_coding	0.816 86.199	3.104
79.411	0.394 25.625	0.43 83.724
ENSMUSG00000021493	Pdlim7	protein_coding	1.059 49.868
1.107 87.609	0.854 6.858 1.12 40.729
ENSMUSG00000021494	Ddx41 protein_coding	1	76.546	0.995
96.897	1.007 77.704	1.012 95.92
ENSMUSG00000021495	Fam193b	protein_coding	0.876 4.874 0.955
89.277	1.192 10.76 0.987 83.724
ENSMUSG00000021496	Pcbd2 protein_coding	0.97 47.266	NA	NA
1.077 71.232	0.967 91.625
ENSMUSG00000021497	Txndc15	protein_coding	1.049 29.536
0.953 87.857	1.001 64.427	0.999 88.387
ENSMUSG00000021499	Catsper3	protein_coding	0.934 75.115
0.967 96.843	1.104 86.831	0.878 84.29
ENSMUSG00000021500	NA	NA	NA	NA	0.973 95.504	0.995
80.749	1.012 80.324
ENSMUSG00000021501	Caml protein_coding	0.995 76.546	NA	NA
0.968 83.607	0.966 85.493
ENSMUSG00000021504	B4galt7	protein_coding	0.959 54.043

1.041 95.809 1.003 79.897 0.977 92.181
ENSMUSG00000021506 Pitx1 protein_coding 1.024 79.165 1.069
83.104 0.915 64.778 0.938 96.287
ENSMUSG00000021508 Cxcl14 protein_coding 0.869 11.973
0.934 96.652 1.233 38.926 0.921 83.724
ENSMUSG00000021509 Slc25a48 protein_coding 0.775 20.799
1.005 94.367 1.285 62.648 1.331 32.045
ENSMUSG00000021510 A530054K11Rik protein_coding 1.028 45.307
0.97 87.452 1.004 87.786 1.019 93.722
ENSMUSG00000021514 Zfp369 protein_coding 0.811 17.031
0.949 92.631 1.294 10.76 0.863 86.254
ENSMUSG00000021518 Ptdss1 protein_coding 1.012 67.374
1.004 95.211 0.986 48.577 0.999 94.19
ENSMUSG00000021519 Mterfd1 protein_coding 1.013 86.843
0.938 74.851 1.048 30.645 0.967 86.699
ENSMUSG00000021520 Uqcrb protein_coding 0.976 76.968 0.988
92.545 1.032 74.082 0.987 96.086
ENSMUSG00000021532 Fastkd3 protein_coding 1.099 23.493
0.977 95.504 0.931 56.822 1.004 96.287
ENSMUSG00000021534 1700001L19Rik protein_coding 0.844 62.158
0.49 92.914 2.414 57.227 1.651 94.752
ENSMUSG00000021536 Adcy2 protein_coding 1.15 74.516 0.963
95.431 0.899 67.962 1.052 82.328
ENSMUSG00000021537 Cetn3 protein_coding 0.978 80.51 0.954 82.947
1.073 50.091 1.102 48.119
ENSMUSG00000021538 NA NA NA NA 1 97.288 1
87.786 1 94.215
ENSMUSG00000021539 Lect2 protein_coding 1 68.353 1
94.767 1 72.695 1 79.545
ENSMUSG00000021540 Smad5 protein_coding 0.996 86.199 NA NA
NA NA 0.962 83.724
ENSMUSG00000021541 Trpc7 protein_coding 1 78.054 1
96.763 1 86.084 1 94.825
ENSMUSG00000021546 Hnrnpk protein_coding 1.106 0.2 0.983
79.807 0.919 4.963 1.018 83.067
ENSMUSG00000021548 Ccnh protein_coding 1.063 42.255 1.05
91.065 0.896 29.217 1.092 50.452
ENSMUSG00000021549 Rasa1 protein_coding 1.01 85.129 1.003
86.387 0.986 64.427 1.013 88.111
ENSMUSG00000021550 2210016F16Rik protein_coding 0.965 16.02 NA
NA 1.06 39.471 0.977 96.183
ENSMUSG00000021552 Gkap1 protein_coding 1.016 49.868 0.937
62.322 1.05 36.95 1.016 92.762
ENSMUSG00000021553 Slc28a3 protein_coding 0.86 60.569
0.697 63.619 1.665 22.366 1.095 94.904
ENSMUSG00000021555 Naa35 protein_coding 1.091 7.501 0.944 26.053
0.97 86.666 0.98 95.92
ENSMUSG00000021556 Golm1 protein_coding 1.05 72 0.88 28.822
1.08 43.586 1.067 60.31
ENSMUSG00000021557 Agtpbp1 protein_coding 1.05 63.126
1.033 88.551 0.924 15.778 1.011 91.872
ENSMUSG00000021559 Dapk1 protein_coding 1.123 52.134 0.879
69.175 1.011 74.325 0.896 83.724
ENSMUSG00000021565 Slc6a19 protein_coding 0.746 39.612

1.169 79.864 1.148 84.434 0.867 91.625
ENSMUSG00000021566 5430425J12Rik antisense 1 82.479 1
91.661 1 81.032 1 94.567
ENSMUSG00000021567 Nkd2 protein_coding 0.949 74.299 1.046
87.029 1.005 82.575 1.065 96.287
ENSMUSG00000021569 Trip13 protein_coding 1.261 21.836
0.766 67.941 1.036 84.434 1.284 91.783
ENSMUSG00000021572 Cep72 protein_coding 1.182 82.352 0.854
86.861 0.988 75.009 0.833 84.29
ENSMUSG00000021573 Tppp protein_coding 0.995 71.516 1.085
97.129 0.929 78.293 0.792 83.724
ENSMUSG00000021575 Ahrr protein_coding 1.976 0.976 0.53 20.174
0.956 73.027 0.922 87.705
ENSMUSG00000021576 Pdcd6 protein_coding 0.996 82.231 0.992
95.211 1.013 78.045 0.981 95.74
ENSMUSG00000021577 Sdha protein_coding 0.944 15.76 0.99 96.138
1.069 35.441 0.966 93.612
ENSMUSG00000021578 Ccdc127 protein_coding 1.073 9.435 0.992
94.48 0.94 20.009 1.007 77.854
ENSMUSG00000021579 Lrrc14b protein_coding 1.037 85.728
1.137 79.579 0.844 35.022 0.957 94.016
ENSMUSG00000021583 Erap1 protein_coding 1.203 11.316 0.898
75.345 0.927 40.19 0.971 96.287
ENSMUSG00000021585 Cast protein_coding 0.967 41.759 NA NA
1.055 64.305 1.043 74.607
ENSMUSG00000021587 Pcsk1 protein_coding 1.866 77.916 0.852
91.955 0.626 84.229 0.451 83.724
ENSMUSG00000021589 Rhobtb3 protein_coding 1.105 18.388
1.019 93.529 0.888 17.381 0.963 88.136
ENSMUSG00000021590 Spata9 protein_coding 0.799 61.633 NA
NA NA NA 1.196 96.287
ENSMUSG00000021591 Glrx protein_coding 1.201 2.784 0.991 93.654
0.841 7.948 1.105 60.466
ENSMUSG00000021592 Arsk protein_coding 1.162 5.481 0.95 78.877
0.907 46.575 0.932 84.29
ENSMUSG00000021594 Srd5a1 protein_coding 0.926 42.722
1.176 84.768 0.92 88.657 1.086 67.169
ENSMUSG00000021595 Nsun2 protein_coding 1.015 81.178 0.928
60.678 1.062 44.361 1.061 60.31
ENSMUSG00000021596 Mctp1 protein_coding 1.275 0.2 1.006 92.137
0.78 9.288 0.923 88.505
ENSMUSG00000021597 Ankrd32 protein_coding 1.001 83.523
0.928 68.848 1.08 33.802 1.101 62.594
ENSMUSG00000021598 Med10 protein_coding 1.055 47.466 1.003
91.661 0.943 69.583 1.048 73.401
ENSMUSG00000021600 8030423J24Rik protein_coding 1 77.291
1 97.155 1 82.74 1 96.248
ENSMUSG00000021604 Irx4 protein_coding 0.792 37.82 1.294 90.092
0.978 78.498 0.975 93.612
ENSMUSG00000021606 Ndufs6 protein_coding 0.803 3.325 1.034
92.062 1.203 22.366 1.071 87.447
ENSMUSG00000021607 Mrpl36 protein_coding 0.939 44.787
1.079 83.104 NA NA 0.993 94.904
ENSMUSG00000021608 Lpcat1 protein_coding 1.165 3.932 NA NA

0.854 3.123 1.005 92.381
ENSMUSG00000021610 Clptm1l protein_coding 0.969 53.679
1.039 72.642 0.994 50.523 1.073 83.067
ENSMUSG00000021611 Tert protein_coding 0.997 59.358 0.923
95.211 1.089 59.612 0.851 93.261
ENSMUSG00000021612 NA NA NA NA 0.682 81.731 1.309
47.512 0.777 93.132
ENSMUSG00000021613 Hapln1 protein_coding 1.619 51.297
0.509 75.618 1.215 72.853 0.645 96.183
ENSMUSG00000021614 Vcan protein_coding 1.224 56.907 0.899
84.594 0.906 83.607 1.449 40.426
ENSMUSG00000021615 Xrcc4 protein_coding 0.924 41.617 1.113
86.156 0.973 88.274 0.994 96.287
ENSMUSG00000021619 Atg10 protein_coding 0.989 49.944 1.015
97.097 0.991 59.274 0.941 95.273
ENSMUSG00000021620 Acot12 protein_coding 1.224 85.203
0.756 91.661 NA NA 1.042 95.92
ENSMUSG00000021621 Zcchc9 protein_coding 1.054 22.349
0.936 72.712 NA NA 0.982 86.699
ENSMUSG00000021622 Ckmt2 protein_coding 3.341 10.105 1.355
85.378 0.221 1.898 0.949 92.691
ENSMUSG00000021624 Cd180 protein_coding 2.053 0 1.131 95.088
0.429 0.72 1.158 59.208
ENSMUSG00000021629 Slc30a5 protein_coding 1.073 13.363
1.001 96.216 0.933 17.258 1.021 90.455
ENSMUSG00000021631 Mrps36-ps1 protein_coding 1 77.113 1
95.431 1 83.397 1 95.74
ENSMUSG00000021635 Rad17 protein_coding 1.058 21.599 1.027
97.155 0.921 37.617 1.033 95.72
ENSMUSG00000021636 Marveld2 protein_coding 0.732 8.365 0.935
84.594 1.462 9.334 0.886 86.991
ENSMUSG00000021638 Ocln protein_coding 1.024 64.193 0.904
74.633 1.081 79.897 0.924 85.636
ENSMUSG00000021639 Gtf2h2 protein_coding 1.011 84.294 0.9
85.418 1.099 55.352 1.063 62.594
ENSMUSG00000021640 Naip1 protein_coding 1.982 31.24 1.023 94.848
0.494 32.297 0.715 86.991
ENSMUSG00000021643 Serf1 protein_coding 0.953 60.569 1.007
96.847 1.04 69.002 0.936 90.854
ENSMUSG00000021645 Smn1 protein_coding 1 80.608 1.025
94.367 0.972 78.954 0.985 93.457
ENSMUSG00000021646 Mccc2 protein_coding 0.969 57.014 0.988
96.984 1.045 54.163 0.936 83.952
ENSMUSG00000021650 Ptcd2 protein_coding 1.12 5.253 0.949 96.268
0.938 3.943 1.017 90.674
ENSMUSG00000021660 Btf3 protein_coding 1.017 53.562 0.903
24.911 1.09 30.948 0.998 91.372
ENSMUSG00000021661 Ankra2 protein_coding 1.05 58.198
0.947 81.284 1.003 72.695 0.987 85.493
ENSMUSG00000021662 Arhgef28 protein_coding 0.94 84.397
0.949 71.98 1.119 43.129 0.999 91.625
ENSMUSG00000021665 Hexb protein_coding 1.144 1.967 1.06 87.609
0.826 7.083 1.01 96.192
ENSMUSG00000021666 Gfm2 protein_coding 0.865 1.815 1.058 78.235

1.09 5.964 0.997 92.466
ENSMUSG00000021668 Polk protein_coding 1.104 10.391 0.994
93.654 0.912 33.137 0.982 95.72
ENSMUSG00000021669 Col4a3bp protein_coding 1.003 67.867 NA
NA 1.01 81.29 0.957 85.636
ENSMUSG00000021670 Hmgcr protein_coding 1.115 27.731 0.981
96.897 0.913 20.009 1.086 80.77
ENSMUSG00000021671 Poc5 protein_coding 1.031 77.497 0.962
85.116 1.009 84.229 1.063 74.193
ENSMUSG00000021675 F2rl2 protein_coding 1.751 24.958 0.891
84.594 0.639 77.704 0.819 95.92
ENSMUSG00000021676 Iqgap2 protein_coding 1.108 51.297
0.804 15.364 1.125 5.964 1.062 84.31
ENSMUSG00000021678 F2rl1 protein_coding 0.813 15.06 1.081 83.104
1.132 50.375 0.937 91.625
ENSMUSG00000021679 S100z protein_coding 1 84.738 NA NA
1 73.531 1 95.877
ENSMUSG00000021681 Aggf1 protein_coding 1.115 0.2 0.999 96.984
0.898 2.563 0.979 88.505
ENSMUSG00000021684 Pde8b protein_coding 1.108 26.338 0.885
72.576 1.021 86.144 0.976 86.991
ENSMUSG00000021685 Otp protein_coding 1 76.042 NA NA
1 87.786 1 96.086
ENSMUSG00000021686 Ap3b1 protein_coding 0.97 72.546 0.973
58.64 1.061 10.079 1.034 75.81
ENSMUSG00000021687 Scamp1 protein_coding 1.114 0 0.914
24.911 0.979 37.103 0.945 83.724
ENSMUSG00000021690 Jmy protein_coding 1.045 66.58 0.931 74.483
1.028 59.274 0.937 83.724
ENSMUSG00000021692 Dimt1 protein_coding 0.965 74.299 1.055
92.931 0.984 87.336 0.995 93.457
ENSMUSG00000021693 Kif2a protein_coding 1.021 29.372 0.943
68.848 1.038 76.498 0.974 91.372
ENSMUSG00000021694 Ercc8 protein_coding 1.028 57.818 1.126
74.663 0.865 64.427 0.98 92.748
ENSMUSG00000021696 Elovl7 protein_coding 0.915 82.231
0.914 79.261 1.196 47.512 0.957 87.218
ENSMUSG00000021697 Depdc1b protein_coding 1.722 25.026
0.855 74.633 0.681 73.027 1.344 86.209
ENSMUSG00000021699 Pde4d protein_coding 0.929 85.728 1.006
94.48 1.067 64.624 1.296 87.134
ENSMUSG00000021701 Plk2 protein_coding 0.903 36.823 0.914
81.02 1.207 27.89 0.995 96.183
ENSMUSG00000021702 Thbs4 protein_coding 0.923 30.637 1.048
86.156 1.037 80.634 1.041 89.108
ENSMUSG00000021703 Serinc5 protein_coding 1.206 1.643 0.923
79.807 0.897 1.898 0.818 83.724
ENSMUSG00000021704 Mtx3 protein_coding 0.961 41.617 0.972
73.094 1.07 9.301 1 94.654
ENSMUSG00000021706 Zfyve16 protein_coding 1.139 5.527 0.911
90.053 0.965 27.86 0.976 92.748
ENSMUSG00000021707 Dhfr protein_coding 1.051 59.211 1.081
90.092 0.883 46.32 1.116 83.067
ENSMUSG00000021708 Rasgrf2 protein_coding 1.288 7.174 0.983

94.48 0.791 17.662 1.005 95.273
ENSMUSG00000021709 Erbb2ip protein_coding 0.937 72.546
0.996 85.075 1.073 32.776 0.953 85.493
ENSMUSG00000021710 Nln protein_coding 0.936 34.305 1.098
72.642 0.971 74.179 1.147 57.075
ENSMUSG00000021711 Trappc13 protein_coding 1.103 20.444 1
95.196 0.909 16.949 1.007 95.273
ENSMUSG00000021712 Trim23 protein_coding 1.025 25.944
0.926 40.463 1.054 53.134 1.074 67.691
ENSMUSG00000021713 Ppwd1 protein_coding 0.927 75.761 NA NA
1.052 77.532 1.018 96.086
ENSMUSG00000021714 Cenpk protein_coding 0.92 85.59 1.037 90.378
1.049 75.009 1.122 83.067
ENSMUSG00000021715 Cwc27 protein_coding 0.93 74.482 1.009
95.504 1.059 57.227 1.104 64.513
ENSMUSG00000021716 Srek1ip1 protein_coding 0.927 14.427
0.996 90.053 1.083 8.339 1.029 84.246
ENSMUSG00000021719 Rgs7bp protein_coding 1.063 75.399 1.02
88.092 0.918 37.103 1.156 48.177
ENSMUSG00000021720 Rnf180 protein_coding 1.134 33.099
0.969 87.041 0.91 60.233 0.91 85.493
ENSMUSG00000021721 Htr1a protein_coding 1 81.647 1
95.708 1 88.601 1 94.297
ENSMUSG00000021725 Parp8 protein_coding 1.188 28.395 0.978
94.848 0.858 32.082 0.895 93.584
ENSMUSG00000021728 Emb protein_coding 1.546 0 0.88 70.693
0.732 6.858 1.114 67.691
ENSMUSG00000021730 Hcn1 protein_coding 0.809 53.138 0.769
95.504 1.609 33.508 1.221 75.81
ENSMUSG00000021731 Mrps30 protein_coding 1.004 68.727
1.017 95.708 0.978 88.657 0.97 90.691
ENSMUSG00000021732 Fgf10 protein_coding 1.016 82.352 0.972
96.843 1.015 78.642 1.01 85.577
ENSMUSG00000021733 Slc4a7 protein_coding 0.946 42.722
0.934 87.041 1.134 31.975 0.873 83.724
ENSMUSG00000021737 Psmd6 protein_coding 0.964 57.014 0.987
96.138 1.046 57.227 1.126 43.377
ENSMUSG00000021738 Atxn7 protein_coding 1.021 52.986 0.965
68.115 1.012 72.853 1.012 93.964
ENSMUSG00000021741 Gm5457 pseudogene 1 82.092 1
94.102 1 84.229 1 96.184
ENSMUSG00000021743 Fezf2 protein_coding 1 69.943 NA NA
1 87.686 1 86.133
ENSMUSG00000021745 Ptprg protein_coding 0.977 74.482 0.998
91.361 1.023 51.596 0.929 83.724
ENSMUSG00000021747 4930452B06Rik protein_coding 1.965 18.556
0.594 73.766 0.855 87.898 0.788 95.593
ENSMUSG00000021748 Pdhb protein_coding 0.638 0.987 1.105 87.029
1.421 13.259 1.042 92.748
ENSMUSG00000021750 Fam107a protein_coding 1.02 70.862 0.73
62.71 1.348 24.029 1.147 58.333
ENSMUSG00000021751 Acox2 protein_coding 0.645 24.748 1.076
97.288 1.435 34.807 1.008 94.215
ENSMUSG00000021752 Kctd6 protein_coding 0.932 28.998 0.982

95.301 1.094 64.624 1.002 95.35
ENSMUSG00000021754 Map3k1 protein_coding 1.105 44.694
1.024 93.899 0.885 40.19 0.875 84.29
ENSMUSG00000021756 Il6st protein_coding 1.225 0 0.969 92.137
0.842 0.72 1.006 89.827
ENSMUSG00000021759 Ppap2a protein_coding 1.046 29.536
1.049 96.68 0.909 27.321 0.936 83.724
ENSMUSG00000021760 Gpx8 protein_coding 0.862 39.174 1.093
82.809 1.059 84.724 0.983 90.95
ENSMUSG00000021763 BC067074 protein_coding 0.892 65.388
1.111 82.809 1.005 70.44 1.002 92.31
ENSMUSG00000021764 Ndufs4 protein_coding 0.877 9.841 NA NA
1.105 31.479 1.106 74.047
ENSMUSG00000021765 Fst protein_coding 1.318 3.151 0.991 90.712
0.765 9.347 0.951 94.19
ENSMUSG00000021767 Kat6b protein_coding 0.959 49.944 1.029
95.504 1.013 47.776 0.971 93.584
ENSMUSG00000021768 Dusp13 protein_coding 1.865 11.044 NA
NA 0.432 3.428 1.089 94.567
ENSMUSG00000021770 Samd8 protein_coding 0.984 71.217 1.068
86.387 0.947 42.997 1.041 86.133
ENSMUSG00000021771 Vdac2 protein_coding 1.02 69.089 1.044
90.389 0.939 33.402 1.073 59.394
ENSMUSG00000021772 Nkiras1 protein_coding 0.988 74.482
1.006 95.708 1.006 73.428 1.021 90.177
ENSMUSG00000021773 Comtd1 protein_coding 0.828 64.014
1.239 72.546 0.974 59.922 1.139 95.273
ENSMUSG00000021774 Ube2e1 protein_coding 1.047 21.599
0.951 78.51 1.005 85.21 1.039 85.577
ENSMUSG00000021775 Nr1d2 protein_coding 1.172 17.582 1.002
93.654 0.853 20.712 1.058 83.067
ENSMUSG00000021779 Thrb protein_coding 1.072 61.171 1.084
97.155 0.858 46.32 0.943 85.493
ENSMUSG00000021782 Dlg5 protein_coding 0.993 53.407 0.924
75.993 1.086 10.523 1.021 95.74
ENSMUSG00000021785 Ngly1 protein_coding 1.042 34.696 0.949
70.29 1.005 83.925 1.029 71.305
ENSMUSG00000021786 Oxsm protein_coding 0.966 85.355 NA NA
1.047 87.744 1.026 93.156
ENSMUSG00000021789 Sftpa1 protein_coding 1.033 85.637
1.145 96.216 0.846 78.369 0.674 96.086
ENSMUSG00000021790 Dydc1 protein_coding 1 86.474 1
96.843 1 86.112 1 88.136
ENSMUSG00000021791 Dydc2 protein_coding 1 78.054 1
95.35 1 67.029 1 89.892
ENSMUSG00000021792 Fam213a protein_coding 0.908 18.907
0.913 76.431 1.209 10.76 0.963 86.254
ENSMUSG00000021794 Glud1 protein_coding 1.189 7.049 0.943 96.001
0.892 22.279 0.972 94.904
ENSMUSG00000021795 Sftpd protein_coding 5.973 7.501 0.567 40.463
0.295 44.211 1.082 92.564
ENSMUSG00000021796 Bmpr1a protein_coding 1.065 21.599
1.057 92.793 0.889 13.848 1.035 90.865
ENSMUSG00000021798 Ldb3 protein_coding 1.741 1.712 1.79 72.546

0.321 0.72 2.118 83.067
ENSMUSG00000021799 Opn4 protein_coding 1 73.841 1
85.075 1 75.009 1 95.877
ENSMUSG00000021803 Cdhr1 protein_coding 0.564 1.703 1.439 64.304
1.232 54.673 0.866 83.724
ENSMUSG00000021806 Nid2 protein_coding 0.81 52.018 1.287
72.642 0.961 67.339 1.027 96.358
ENSMUSG00000021807 2700060E02Rik protein_coding 1.128 10.595
0.899 54.427 0.985 85.675 1.051 76.467
ENSMUSG00000021809 Nudt13 protein_coding 0.936 34.795
0.958 83.58 1.11 27.89 1.001 94.654
ENSMUSG00000021810 NA NA NA NA 0.995 96.68 NA NA
1.021 63.708
ENSMUSG00000021811 Dnajc9 protein_coding 1.015 63.804
0.986 96.843 0.995 61.743 1.113 55.841
ENSMUSG00000021814 Anxa7 protein_coding 1.003 83.169 0.994
92.355 1.005 80.634 1.1 44.679
ENSMUSG00000021815 Mss51 protein_coding 1.043 37.414 1.119
94.597 0.858 71.004 1.166 81.901
ENSMUSG00000021816 Ppp3cb protein_coding 1.115 1.967 0.985
94.367 0.912 1.705 1.016 94.016
ENSMUSG00000021819 Zswim8 protein_coding 0.887 0.987 1.04
83.104 1.082 5.864 0.996 95.559
ENSMUSG00000021820 Camk2g protein_coding 1.027 51.297
1.011 91.456 0.959 35.559 0.961 95.877
ENSMUSG00000021822 Plau protein_coding 1.453 0 0.936 95.088
0.735 0.945 1.069 59.464
ENSMUSG00000021823 Vcl protein_coding 1.01 67.374 0.997
93.899 0.987 59.383 0.961 88.387
ENSMUSG00000021824 Ap3m1 protein_coding 0.933 29.972 1.07
72.546 1.001 87.103 1.041 85.786
ENSMUSG00000021830 Txndc16 protein_coding 1.178 5.313 0.949
80.463 0.892 18.122 0.863 83.724
ENSMUSG00000021831 Ero1l protein_coding 1.016 48.288 1
96.847 0.987 44.211 0.993 95.777
ENSMUSG00000021832 Psmc6 protein_coding 1.096 40.065 0.933
79.475 0.978 87.173 1.009 83.067
ENSMUSG00000021835 Bmp4 protein_coding 0.859 28.242 0.957
92.404 1.215 28.997 0.94 92.742
ENSMUSG00000021838 Samd4 protein_coding 1.296 0.2 0.947 85.671
0.816 10.362 1.11 93.734
ENSMUSG00000021840 Mapk1ip1l protein_coding 1.048 35.371 1.02
91.661 0.936 64.427 1.019 77.854
ENSMUSG00000021843 Ktn1 protein_coding 1.237 17.582 0.864
61.883 0.938 65.688 1.068 63.708
ENSMUSG00000021846 Peli2 protein_coding 1.008 85.832 0.917
72.576 1.085 56.708 0.953 94.001
ENSMUSG00000021848 Otx2 protein_coding 1 69.777 1
97.155 1 88.693 1 94.904
ENSMUSG00000021850 1700011H14Rik protein_coding 1 78.538
1 95.504 1 79.426 1 95.273
ENSMUSG00000021852 Slc35f4 protein_coding 1 78.538 1
93.654 1 80.089 1 95.273
ENSMUSG00000021866 Anxa11 protein_coding 0.944 22.237

1.044 80.806 1.012 88.274 1.04 71.855
ENSMUSG00000021867 Tmem254b protein_coding 1 82.092 1
96.984 1 83.275 1 91.335
ENSMUSG00000021868 Ppif protein_coding 1.073 23.254 1.083
80.277 0.859 7.339 0.963 96.287
ENSMUSG00000021870 Slmap protein_coding 0.924 32.168 1.03
95.735 1.046 35.441 1.032 69.918
ENSMUSG00000021871 Pnp protein_coding 0.909 85.283 1.182
82.76 0.927 33.402 1.372 49.514
ENSMUSG00000021872 Rnase10 protein_coding 0.678 69.24 1.087
94.48 1.353 58.915 2.089 38.624
ENSMUSG00000021874 4933413J09Rik lincRNA 0.892 30.136
0.633 96.68 1.766 53.587 1.182 87.088
ENSMUSG00000021876 Rnase4 protein_coding 1.02 28.395
0.912 65.165 1.073 82.685 1.247 52.324
ENSMUSG00000021877 Arf4 protein_coding 1.036 80.408 1.015
92.062 0.951 46.575 1.037 57.1
ENSMUSG00000021879 Dnah12 protein_coding 1.372 30.788
1.001 96.763 0.726 25.512 0.989 91.492
ENSMUSG00000021880 Rnase6 protein_coding 1.681 1.828 0.921
79.261 0.647 21.431 0.956 96.248
ENSMUSG00000021884 Hacl1 protein_coding 1.023 61.725 1.014
96.138 0.963 69.583 0.947 95.593
ENSMUSG00000021886 Gpr65 protein_coding 1.269 10.19 0.925 82.947
0.851 34.21 0.977 88.505
ENSMUSG00000021890 Eaf1 protein_coding 1.061 25.207 1.047
85.756 0.899 11.902 1.015 86.133
ENSMUSG00000021891 Mettl6 protein_coding 1.051 30.984 NA
NA 0.952 54.964 0.973 93.132
ENSMUSG00000021892 Sh3bp5 protein_coding 0.994 85.728
0.922 81.134 1.088 43.586 0.955 83.724
ENSMUSG00000021893 Capn7 protein_coding 1.07 6.958 0.944 79.475
0.988 58.516 0.996 94.608
ENSMUSG00000021895 Arhgef3 protein_coding 1.073 5.109 0.869
8.462 1.068 44.515 0.919 90.157
ENSMUSG00000021898 Asb14 protein_coding 3.208 5.016 2.104 84.126
0.149 0.72 0.874 96.183
ENSMUSG00000021900 Btd protein_coding 0.909 16.325 1.083
79.261 1.016 84.669 1.012 91.928
ENSMUSG00000021901 Bap1 protein_coding 0.95 3.853 NA NA
1.045 14.873 0.979 93.612
ENSMUSG00000021902 Phf7 protein_coding 1.123 32.058 0.826
68.818 1.075 74.325 0.879 86.699
ENSMUSG00000021903 Galnt15 protein_coding 0.788 22.92 0.809
64.555 1.572 7.635 0.949 92.181
ENSMUSG00000021904 Sema3g protein_coding 0.964 57.818
0.935 65.819 1.109 24.691 1.048 76.046
ENSMUSG00000021905 Dph3 protein_coding 0.959 57.818 1.018
95.301 1.023 55.512 1.054 74.896
ENSMUSG00000021906 Oxnad1 protein_coding 0.929 24.577 NA
NA 1.059 24.691 1.059 71.653
ENSMUSG00000021907 Msmb protein_coding 1 62.158 1
96.335 1 68.643 1 95.273
ENSMUSG00000021910 Nisch protein_coding 0.945 1.017 1.012 87.609

1.045 16.666 0.988 88.797
ENSMUSG00000021911 Parg protein_coding 0.982 81.01 1.031 95.211
0.989 83.719 0.981 96.184
ENSMUSG00000021913 Ogdhl protein_coding 1.074 83.726 0.837
91.361 1.108 76.754 1.104 53.923
ENSMUSG00000021916 Glt8d1 protein_coding 1.103 25.026 0.93
93.654 0.977 69.974 1.052 44.882
ENSMUSG00000021917 Spcs1 protein_coding 0.922 33.224 1.084
71.98 0.997 67.648 1.095 31.133
ENSMUSG00000021918 Nek4 protein_coding 1.074 28.395 NA NA
0.922 55.214 0.901 83.724
ENSMUSG00000021919 Chat protein_coding 1 67.608 1
96.407 1 86.144 1 93.132
ENSMUSG00000021922 Itih4 protein_coding 0.854 40.568 0.864
96.407 1.349 77.17 NA NA
ENSMUSG00000021927 Gm5803 pseudogene 1 86.006 NA NA
1 84.724 1 91.372
ENSMUSG00000021928 Ebpl protein_coding 1.095 9.435 0.884 53.79
1.036 87.786 1.058 86.133
ENSMUSG00000021929 Kpna3 protein_coding 1.116 1.161 0.998 96.013
0.895 4.338 1.065 59.441
ENSMUSG00000021930 Spryd7 protein_coding 0.952 77.113
1.013 97.263 1.031 85.352 0.954 83.952
ENSMUSG00000021932 Rnaseh2b protein_coding 1.124 46.097 0.89
90.053 1 79.524 1.09 62.594
ENSMUSG00000021933 Gucy1b2 protein_coding 1 82.891 NA
NA 1 78.498 1 96.086
ENSMUSG00000021936 Mapk8 protein_coding 1.019 53.228 1.023
95.504 0.96 75.967 1.004 95.832
ENSMUSG00000021938 Pspc1 protein_coding 1.168 2.361 1.041 96.984
0.824 0.72 1.071 46.846
ENSMUSG00000021939 Ctsb protein_coding 1.124 6.026 0.9 51.159
0.99 59.726 1.033 82.091
ENSMUSG00000021940 Ptpn20 protein_coding 1 82.891 1
95.735 1 85.935 1 88.053
ENSMUSG00000021943 Gdf10 protein_coding 1.091 36.686 NA NA
0.92 71.388 0.999 94.904
ENSMUSG00000021944 Gata4 protein_coding 1 62.574 1
90.576 1 69.196 1 92.181
ENSMUSG00000021945 Zmym2 protein_coding 1.044 15.85 0.979 97.155
0.979 26.369 0.954 88.387
ENSMUSG00000021947 Cryl1 protein_coding 1.27 0.767 0.88 77.598
0.892 16.409 0.966 86.991
ENSMUSG00000021948 Prkcd protein_coding 1.108 12.396 0.959
92.137 0.936 38.434 0.978 92.31
ENSMUSG00000021950 Anxa8 protein_coding 1.246 18.985 1.147
89.014 0.7 21.431 0.651 83.724
ENSMUSG00000021951 N6amt2 protein_coding 0.944 82.092
1.094 87.029 0.966 64.155 1.134 59.441
ENSMUSG00000021952 Xpo4 protein_coding 0.938 56.768 0.934
92.404 1.14 54.163 1.068 95.676
ENSMUSG00000021957 Tkt protein_coding 0.327 0.987 1.144 82.904
2.686 5.864 1.009 95.273
ENSMUSG00000021958 Pinx1 protein_coding 0.939 60.707 1.173

81.127 0.909 71.388 1.05 82.328
ENSMUSG00000021959 Lats2 protein_coding 1.044 16.249 1.094
80.775 0.873 0.879 0.923 83.952
ENSMUSG00000021961 4930578I06Rik protein_coding 1 72.655
1 95.708 1 86.666 1 95.35
ENSMUSG00000021962 Dcp1a protein_coding 0.993 74.299 1.048
82.904 0.963 63.075 1.003 90.125
ENSMUSG00000021963 Sap18 protein_coding 0.948 5.491 1.073 71.98
0.985 84.83 1.028 70.36
ENSMUSG00000021965 Ska3 protein_coding 1.377 44.061 0.759
78.877 0.955 85.464 1.457 57.711
ENSMUSG00000021967 Mrp63 protein_coding 0.821 2.174 1.053 90.952
1.16 8.007 0.992 96.248
ENSMUSG00000021969 Zdhhc20 protein_coding 1.142 1.216 0.94
65.165 0.933 66.201 0.91 83.724
ENSMUSG00000021972 Hmbox1 protein_coding 1.005 80.51 0.997
95.735 0.999 87.173 0.933 83.724
ENSMUSG00000021973 Micu2 protein_coding 1.065 24.147 0.926
38.699 1.016 84.896 1.085 50.452
ENSMUSG00000021974 Fgf9 protein_coding 1.133 36.438 1.116
95.431 0.792 28.489 1.254 60.968
ENSMUSG00000021975 Ints9 protein_coding 1.073 42.134 1.033
86.34 0.898 21.331 1.004 91.492
ENSMUSG00000021977 1700129C05Rik protein_coding 1 86.918
1 94.597 NA NA 1 94.99
ENSMUSG00000021978 Extl3 protein_coding 1.003 71.915 1.074
71.617 0.93 33.402 1.07 91.525
ENSMUSG00000021981 Cab39l protein_coding 0.968 79.433
0.943 68.818 1.098 19.452 0.983 86.699
ENSMUSG00000021982 Cdadc1 protein_coding 1.026 70.36 0.888
47.831 1.093 21.522 0.983 96.183
ENSMUSG00000021983 Atp8a2 protein_coding 1.459 26.338 1.09
96.216 0.629 19.552 0.861 90.674
ENSMUSG00000021986 Amer2 protein_coding 0.606 15.227 0.871
94.48 1.892 20.315 0.859 87.705
ENSMUSG00000021987 Mtmr6 protein_coding 1.069 3.893 0.948 68.818
0.984 47.641 1.068 58.557
ENSMUSG00000021990 Spata13 protein_coding 0.942 57.014
1.074 83.104 0.99 76.64 0.892 83.724
ENSMUSG00000021991 Cacna2d3 protein_coding 1.659 64.956
0.828 88.704 0.73 78.045 0.892 96.248
ENSMUSG00000021993 Mipep protein_coding 0.964 23.556 NA NA
1.053 43.842 1.008 91.011
ENSMUSG00000021994 Wnt5a protein_coding 0.987 86.819 1.15
80.14 0.884 41.154 0.89 88.505
ENSMUSG00000021996 Esd protein_coding 0.965 51.447 0.912
39.547 1.134 11.329 1.043 56.863
ENSMUSG00000021998 Lcp1 protein_coding 1.507 0 0.94 88.705
0.703 0.72 1.004 89.108
ENSMUSG00000022000 Zc3h13 protein_coding 1.039 85.203
0.989 96.847 0.973 73.027 0.986 94.608
ENSMUSG00000022002 4930564B18Rik protein_coding 1 79.165
1 93.899 1 84.229 1 94.016
ENSMUSG00000022003 Slc25a30 protein_coding 1.05 79.068

0.865 64.624 1.099 28.979 1.03 82.66
ENSMUSG00000022008 Gpalpp1 protein_coding 1.15 0 0.902
21.315 0.961 43.426 0.968 86.991
ENSMUSG00000022009 Nufip1 protein_coding 1.211 2.651 0.889
56.027 0.927 48.851 0.982 95.74
ENSMUSG00000022010 Tsc22d1 protein_coding 1.049 42.255
1.009 94.767 0.94 35.559 0.975 94.904
ENSMUSG00000022012 Enox1 protein_coding 1.316 12.396 0.927
85.921 0.822 13.202 1.077 87.886
ENSMUSG00000022013 Dnajc15 protein_coding 0.936 26.217
1.036 87.609 1.029 84.961 1.019 81.56
ENSMUSG00000022014 Epsti1 protein_coding 1.727 0.2 0.806
55.265 0.718 17.898 1.105 73.597
ENSMUSG00000022015 Tnfsf11 protein_coding 0.477 52.9 0.933
95.504 2.238 57.637 0.436 86.991
ENSMUSG00000022016 Akap11 protein_coding 0.972 73.292
0.966 88.897 1.065 62.995 1.034 94.752
ENSMUSG00000022018 Rgcc protein_coding 1.004 65.106 0.924
96.652 1.076 41.331 0.903 91.372
ENSMUSG00000022019 Tdrd3 protein_coding 1.035 26.338 0.939
62.692 1.031 73.428 0.992 94.904
ENSMUSG00000022020 Naa16 protein_coding 1.105 14.59 1.003 90.712
0.901 30.827 1.026 83.067
ENSMUSG00000022021 Diap3 protein_coding 1.314 29.057 0.836
56.955 0.91 85.675 1.329 68.696
ENSMUSG00000022022 Mtrf1 protein_coding 1.098 26.136 1.003
93.574 0.908 12.41 1.014 94.215
ENSMUSG00000022023 Wbp4 protein_coding 0.968 46.693 0.994
95.301 1.04 38.975 1.014 76.831
ENSMUSG00000022024 Sugt1 protein_coding 1.053 3.536 0.986 95.504
0.962 6.858 1.014 93.117
ENSMUSG00000022025 Lect1 protein_coding 1.049 64.956 0.984
90.053 0.964 84.308 0.836 93.316
ENSMUSG00000022026 Olfm4 protein_coding 0.438 86.145 0.909
95.088 2.52 50.636 8.747 31.133
ENSMUSG00000022031 Elp3 protein_coding 1.045 36.438 1.019
92.931 0.936 15.169 1.069 37.634
ENSMUSG00000022032 Scara5 protein_coding 1.013 74.299
0.836 54.06 1.176 10.819 1.039 96.086
ENSMUSG00000022033 Pbk protein_coding 1.421 30.497 0.695
69.175 1.011 88.601 1.607 68.696
ENSMUSG00000022034 Esco2 protein_coding 1.25 53.562 0.791
79.475 1.01 86.112 1.699 70.539
ENSMUSG00000022035 Ccdc25 protein_coding 1.045 58.198
0.975 94.887 0.977 76.64 1.028 69.404
ENSMUSG00000022037 Clu protein_coding 0.935 39.612 0.945
65.165 1.128 18.411 0.896 88.387
ENSMUSG00000022039 Adam2 protein_coding 2.342 7.492 0.906 95.211
0.469 20.751 0.713 87.992
ENSMUSG00000022040 Ephx2 protein_coding 1.23 4.916 0.948 97.288
0.856 20.511 0.933 88.245
ENSMUSG00000022041 Chrna2 protein_coding 0.992 83.263
1.209 85.756 0.831 54.25 0.901 87.735
ENSMUSG00000022043 Trim35 protein_coding 1.097 1.949 0.979

96.138 0.931 0.879 1.087 51.908
ENSMUSG00000022044 NA NA NA NA 1.113 94.597 0.967
84.504 1.293 57.952
ENSMUSG00000022048 NA NA NA NA 1.084 68.96 0.921 3.718
1.059 38.598
ENSMUSG00000022051 Bnip3l protein_coding 0.965 65.388
0.946 85.418 1.096 36.95 0.994 92.31
ENSMUSG00000022052 Ppp2r2a protein_coding 1.112 5.109 0.97
87.919 0.928 4.23 1.026 67.94
ENSMUSG00000022053 Ebf2 protein_coding 0.924 79.433 1.001
84.594 1.075 43.586 1.038 92.018
ENSMUSG00000022054 Nefm protein_coding 0.998 62.158 0.798
95.708 1.256 59.612 0.842 78.379
ENSMUSG00000022057 Adamdec1 protein_coding 0.968 57.949
1.793 79.411 0.576 9.494 0.77 85.493
ENSMUSG00000022064 Pibf1 protein_coding 1.006 82.231 0.968
95.211 1.024 81.857 1.057 71.653
ENSMUSG00000022070 Bora protein_coding 1.035 74.482 NA NA
0.905 67.029 1.208 60.968
ENSMUSG00000022074 Tnfrsf10b protein_coding 1.03 73.124
1.128 79.367 0.863 21.852 0.912 88.136
ENSMUSG00000022075 Rhobtb2 protein_coding 1.021 51.737
1.161 71.222 0.844 4.521 1.077 44.679
ENSMUSG00000022076 Klhl1 protein_coding 1 85.857 NA NA
1 88.657 1 93.045
ENSMUSG00000022089 Bin3 protein_coding 0.979 77.407 0.97
72.399 1.049 27.732 0.987 91.783
ENSMUSG00000022090 Pdlim2 protein_coding 1.144 1.323 0.797
11.228 1.096 42.199 1.147 82.85
ENSMUSG00000022091 Sorbs3 protein_coding 1.028 74.8 0.927
86.538 1.049 48.772 1.013 96.358
ENSMUSG00000022092 Ppp3cc protein_coding 1.02 83.658
0.982 92.355 0.996 79.625 1.031 82.328
ENSMUSG00000022094 Slc39a14 protein_coding 1.132 50.627
0.825 64.123 1.072 70.9 1.041 75.198
ENSMUSG00000022095 Fam160b2 protein_coding 0.935 18.731
1.036 94.767 1.035 36.388 1.027 94.904
ENSMUSG00000022096 Hr protein_coding 1.171 20.799 NA NA
0.946 28.901 1.047 91.872
ENSMUSG00000022098 Bmp1 protein_coding 0.962 51.089 1.031
90.116 1.005 86.666 1.158 31.133
ENSMUSG00000022099 Dmtn protein_coding 0.689 0.987 1.1 72.642
1.315 7.635 0.986 96.183
ENSMUSG00000022100 Xpo7 protein_coding 1.084 0.976 0.945 65.165
0.98 9.288 0.987 87.705
ENSMUSG00000022101 Fgf17 protein_coding 1.343 80.03 0.741 92.474
1.003 82.436 0.826 91.979
ENSMUSG00000022102 Dok2 protein_coding 1.234 2.723 0.871 67.513
0.928 57.819 1.312 46.412
ENSMUSG00000022103 Gfra2 protein_coding 1.173 8.102 1.121 96.378
0.757 19.236 1.075 70.624
ENSMUSG00000022105 Rb1 protein_coding 0.953 32.557 0.947
95.329 1.106 24.029 0.95 83.724
ENSMUSG00000022106 Rcbtb2 protein_coding 1.085 8.477 1.052

83.104 0.873 6.524 0.944 83.724
ENSMUSG00000022108 Itm2b protein_coding 1.114 3.598 0.936 52.266
0.955 57.819 0.948 84.19
ENSMUSG00000022109 Med4 protein_coding 1.008 69.408 1.003
95.211 0.991 82.903 1.028 96.287
ENSMUSG00000022110 Sucla2 protein_coding 1.042 85.637
0.932 96.138 1.024 85.921 1.039 95.273
ENSMUSG00000022111 Uchl3 protein_coding 1.144 30.282 1.058
88.533 0.824 23.607 0.954 96.183
ENSMUSG00000022113 NA NA NA NA 1 94.48 1 78.864
1 96.287
ENSMUSG00000022114 Spry2 protein_coding 0.86 2.668 0.938 84.594
1.24 8.339 1.059 67.691
ENSMUSG00000022116 4930449E01Rik protein_coding 1 69.489
1 87.313 1 69.825 1 94.752
ENSMUSG00000022119 Rbm26 protein_coding 0.92 16.895 0.989
81.731 1.096 18.797 1.065 83.067
ENSMUSG00000022120 Rnf219 protein_coding 1.141 4.925 0.934
88.704 0.939 25.688 1.018 96.287
ENSMUSG00000022122 Ednrb protein_coding 1.454 0 0.881 81.02
0.778 0.72 1.071 74.193
ENSMUSG00000022123 Scel protein_coding 1.427 27.113 0.788
95.504 0.891 26.809 0.953 94.19
ENSMUSG00000022124 Fbxl3 protein_coding 0.986 69.715 0.967
90.287 1.051 49.601 0.931 83.724
ENSMUSG00000022125 Cln5 protein_coding 1.102 4.675 1.023 96.378
0.888 7.339 1.009 76.831
ENSMUSG00000022129 Dct protein_coding 0.404 3.009 1.515 72.546
1.63 16.808 1.495 70.85
ENSMUSG00000022130 Tgds protein_coding 0.931 21.025 1.085
80.775 0.987 77.17 0.976 94.001
ENSMUSG00000022131 Gpr180 protein_coding 0.951 19.224 1.14
62.898 0.92 59.125 0.994 96.248
ENSMUSG00000022132 Cldn10 protein_coding 0.8 16.02 0.811
37.426 1.533 5.864 0.881 92.742
ENSMUSG00000022136 Dnajc3 protein_coding 1.015 84.865
1.034 93.707 0.95 78.642 1.019 54.153
ENSMUSG00000022139 Mbnl2 protein_coding 1.009 82.231 1.033
90.29 0.963 86.346 1.066 60.968
ENSMUSG00000022141 Nipbl protein_coding 0.924 3.47 0.933 81.731
1.158 7.482 1.001 91.928
ENSMUSG00000022142 Nup155 protein_coding 1.041 82.705 0.98
96.138 0.983 67.962 1.046 78.042
ENSMUSG00000022144 Gdnf protein_coding 0.937 82.732 2.166
91.584 0.494 61.288 1.577 79.268
ENSMUSG00000022146 Osmr protein_coding 0.897 48.146 NA NA
1.089 60.789 1.152 72.204
ENSMUSG00000022148 Fyb protein_coding 1.268 12.396 1.056
92.111 0.746 22.279 1.005 94.904
ENSMUSG00000022149 C9 protein_coding 1.973 51.826 1.346
91.065 0.376 23.1 0.709 96.287
ENSMUSG00000022150 Dab2 protein_coding 1.372 0.461 1.01 95.196
0.72 0.72 1.114 55.054
ENSMUSG00000022151 Ttc33 protein_coding 0.979 70.862 0.978

84.594 1.047 53.882 1.031 70.539
ENSMUSG00000022155 Mroh2b protein_coding 1 73.401 1
96.652 1 75.637 1 93.612
ENSMUSG00000022156 Gzme protein_coding 1 66.944 1
96.138 1 78.642 1 96.183
ENSMUSG00000022157 Mcpt8 protein_coding 1 84.452 1
96.216 1 88.274 1 90.397
ENSMUSG00000022159 Rab2b protein_coding 1.027 61.171 NA NA
0.985 78.293 0.937 83.724
ENSMUSG00000022160 Mettl3 protein_coding 0.945 14.526
1.034 93.899 1.021 55.81 1.05 83.581
ENSMUSG00000022174 Dad1 protein_coding 0.928 36.823 1.131
67.843 0.954 59.922 0.972 93.964
ENSMUSG00000022175 Lrp10 protein_coding 1.003 82.891 0.982
93.654 1.017 80.381 0.99 92.742
ENSMUSG00000022176 Rem2 protein_coding 0.625 38.555 1.268
96.216 1.263 60.104 0.842 89.587
ENSMUSG00000022177 Haus4 protein_coding 0.916 61.633 0.957
79.475 1.138 39.471 0.989 91.105
ENSMUSG00000022178 Ajuba protein_coding 0.902 21.957 1.08
83.104 1.024 58.616 0.909 86.699
ENSMUSG00000022179 4931414P19Rik protein_coding 0.983 41.021
0.976 96.847 1.042 80.089 0.956 92.31
ENSMUSG00000022180 Slc7a8 protein_coding 1.712 0.2 0.776
34.058 0.753 6.935 1.117 94.19
ENSMUSG00000022181 C6 protein_coding 1.313 12.985 0.808
68.115 0.939 78.954 0.947 95.832
ENSMUSG00000022184 Fbxo4 protein_coding 1.02 80.51 0.98 95.708
1.003 88.601 0.95 95.832
ENSMUSG00000022185 Acin1 protein_coding 0.923 1.343 1.042 96.407
1.04 23.454 0.956 85.493
ENSMUSG00000022186 Oxct1 protein_coding 1.116 1.395 1.092 75.62
0.822 0.72 0.979 90.805
ENSMUSG00000022191 Drosha protein_coding 0.974 39.712
1.009 85.756 1.015 69.728 1.025 84.31
ENSMUSG00000022193 Psmb5 protein_coding 0.767 0.987 1.024 84.007
1.274 5.864 1.04 60.31
ENSMUSG00000022194 Pabpn1 protein_coding 0.704 1.031 1.121
83.104 1.272 8.466 1.077 92.52
ENSMUSG00000022195 6030458C11Rik protein_coding 1.069 5.109
1.014 97.155 0.925 8.626 0.986 91.291
ENSMUSG00000022197 Pdzd2 protein_coding 1.062 82.732 0.973
97.263 0.965 84.071 1.034 95.74
ENSMUSG00000022199 Slc22a17 protein_coding 0.859 4.176 1.142
79.055 1.021 42.065 0.971 85.493
ENSMUSG00000022200 Golph3 protein_coding 1.097 1.815 0.985
94.887 0.921 7.509 0.96 87.992
ENSMUSG00000022201 Zfr protein_coding 1.017 74.958 1.001
90.256 0.981 87.103 0.997 89.892
ENSMUSG00000022203 Efs protein_coding 1.019 82.231 1.005
97.155 0.972 77.704 0.849 84.29
ENSMUSG00000022204 Ngdn protein_coding 1.01 79.709 0.931
73.166 1.063 36.578 0.955 88.505
ENSMUSG00000022205 Sub1 protein_coding 0.992 81.76 0.944 53.79

1.063 38.038 1.039 80.497
ENSMUSG00000022206 Npr3 protein_coding 0.841 60.339 1.058
90.454 1.123 80.015 1.443 92.381
ENSMUSG00000022208 Jph4 protein_coding 0.774 47.174 1.28
79.864 1.009 88.657 1.252 89.498
ENSMUSG00000022209 Dhrs2 protein_coding 1.519 75.196 NA NA
0.761 63.504 1.023 92.381
ENSMUSG00000022210 Dhrs4 protein_coding 0.952 71.915 1.055
92.253 0.996 88.601 0.995 95.593
ENSMUSG00000022211 Lrrc16b protein_coding 0.961 57.227
1.038 89.996 1.003 78.864 0.808 85.493
ENSMUSG00000022214 Dcaf11 protein_coding 0.919 8.751 1.042
87.604 1.047 40.052 0.979 86.699
ENSMUSG00000022215 Fitm1 protein_coding 4.817 7.959 3.759 87.609
0.055 0.72 0.857 96.183
ENSMUSG00000022216 Psme1 protein_coding 1.044 59.211 1.067
79.055 0.9 14.262 1.032 48.119
ENSMUSG00000022217 Emc9 protein_coding 0.889 35.158 1.036
84.126 1.088 61.418 0.954 84.29
ENSMUSG00000022218 Tgm1 protein_coding 2.164 0 0.694 59.946
0.665 19.552 1.164 83.067
ENSMUSG00000022219 Cideb protein_coding 0.628 18.731 0.798
96.268 2.001 16.205 1.192 80.187
ENSMUSG00000022220 Adcy4 protein_coding 0.879 26.217 1.065
94.367 1.062 51.243 0.975 90.95
ENSMUSG00000022221 Ripk3 protein_coding 1.203 12.549 1.177
91.584 0.705 9.563 1.222 42.736
ENSMUSG00000022223 Sdr39u1 protein_coding 0.816 9.863 1.167
85.756 1.05 39.439 0.978 96.183
ENSMUSG00000022225 Cma1 protein_coding 1.847 0 0.79 68.818
0.687 6.751 0.983 92.466
ENSMUSG00000022226 Mcpt2 protein_coding 1 83.169 1
95.504 NA NA 1 94.153
ENSMUSG00000022227 Mcpt1 protein_coding 1 82.231 1
94.89 1 85.464 1 96.248
ENSMUSG00000022228 Zscan26 protein_coding 1.123 0.664 0.988
94.887 0.902 2.333 0.953 83.724
ENSMUSG00000022229 Atp12a protein_coding 1.716 76.319
0.759 92.404 0.764 81.29 0.455 87.705
ENSMUSG00000022231 Sema5a protein_coding 1.061 22.633
1.022 95.708 0.924 35.264 0.946 86.991
ENSMUSG00000022234 Cct5 protein_coding 1.004 85.092 0.953
68.115 1.043 22.93 1.04 32.045
ENSMUSG00000022235 Cmbl protein_coding 1.005 70.213 1.045
93.27 0.954 87.336 0.914 87.735
ENSMUSG00000022236 Ropn1l protein_coding 0.996 73.841
0.853 71.41 1.173 40.052 0.904 86.699
ENSMUSG00000022237 Ankrd33b protein_coding 1.292 13.105
0.787 50.664 0.982 86.144 0.95 96.287
ENSMUSG00000022240 Ctnnd2 protein_coding 1.035 85.092
1.087 85.756 0.89 65.688 1.003 94.904
ENSMUSG00000022241 Tars protein_coding 1.014 86.658 0.941
69.611 1.044 68.425 1.062 69.404
ENSMUSG00000022244 Amacr protein_coding 0.899 44.922 0.925

85.116 1.198 32.776 1.052 83.276
ENSMUSG00000022245 Skor1 protein_coding 1.609 69.489 0.464
84.594 1.338 77.532 0.288 83.724
ENSMUSG00000022246 Rai14 protein_coding 0.92 51.089 0.959
74.851 1.129 32.173 1.038 91.525
ENSMUSG00000022247 Brix1 protein_coding 1.185 7.179 0.932 53.434
0.902 35.264 0.939 91.269
ENSMUSG00000022248 Rad1 protein_coding 1.138 6.541 0.923 68.115
0.954 40.794 0.981 95.74
ENSMUSG00000022253 Nadk2 protein_coding 1.114 1.949 0.998 97.263
0.901 4.935 0.882 83.724
ENSMUSG00000022255 Mtdh protein_coding 1.034 41.211 0.994
95.708 0.974 52.213 1.013 90.177
ENSMUSG00000022257 Laptm4b protein_coding 0.979 71.36 1.168
66.695 0.875 10.726 1.051 95.273
ENSMUSG00000022261 Sdc2 protein_coding 1.066 32.873 0.97
86.604 0.965 54.964 0.995 89.587
ENSMUSG00000022262 Dnah5 protein_coding 1 71.142 1
95.708 1 38.26 1 95.273
ENSMUSG00000022263 Trio protein_coding 0.996 75.115 0.9
83.58 1.118 34.285 0.981 94.82
ENSMUSG00000022265 Ank protein_coding 0.892 9.752 1.068 83.104
1.048 61.841 1.096 91.335
ENSMUSG00000022269 11-Mar protein_coding 1 83.726 NA
NA 1 77.294 1 92.018
ENSMUSG00000022270 Fam134b protein_coding 1.121 35.787
0.866 53.434 1.032 52.574 0.848 86.991
ENSMUSG00000022272 Myo10 protein_coding 0.949 77.763 0.958
93.817 1.102 43.481 0.93 83.724
ENSMUSG00000022280 Rnf19a protein_coding 0.935 43.495
1.071 67.843 0.997 84.669 1.112 79.268
ENSMUSG00000022283 Pabpc1 protein_coding 1.166 0 0.909
8.462 0.941 8.192 1.044 68.696
ENSMUSG00000022285 Ywhaz protein_coding 1.133 2.352 0.951 90.046
0.93 6.858 1.013 91.492
ENSMUSG00000022286 Grhl2 protein_coding 0.807 24.344 0.927
93.654 1.34 29.568 0.922 92.181
ENSMUSG00000022288 4930447A16Rik protein_coding 1 86.145
1 95.504 1 83.158 1 92.748
ENSMUSG00000022292 Rrm2b protein_coding 1.095 29.596 0.966
84.858 0.944 69.974 0.916 83.724
ENSMUSG00000022295 Atp6v1c1 protein_coding 0.992 81.125
0.972 88.705 1.033 60.5 1.07 85.577
ENSMUSG00000022296 Baalc protein_coding 0.778 74.299 1.294
93.899 0.994 84.669 1.212 96.192
ENSMUSG00000022297 Fzd6 protein_coding 1.035 83.263 0.784
46.764 1.23 10.281 0.942 83.724
ENSMUSG00000022299 Slc25a32 protein_coding 1.082 32.676
0.839 38.699 1.096 28.603 0.965 87.735
ENSMUSG00000022300 Dcaf13 protein_coding 1.038 43.344
0.944 66.796 1.022 80.015 1.088 52.324
ENSMUSG00000022303 Dcstamp protein_coding 0.933 43.816 0.72
90.744 1.484 20.572 1.193 78.948
ENSMUSG00000022305 Lrp12 protein_coding 0.996 69.985 0.655

83.58 1.526 37.259 1.046 60.31
ENSMUSG00000022306 Zfpm2 protein_coding 1.279 13.8 0.884 73.166
0.883 62.533 1.008 80.187
ENSMUSG00000022307 Oxr1 protein_coding 1.041 50.627 0.965
90.046 0.998 87.898 0.973 84.19
ENSMUSG00000022309 Angpt1 protein_coding 1.037 64.956
1.136 90.952 0.846 45.799 0.942 85.493
ENSMUSG00000022311 Csmd3 protein_coding 1 80.51 1 85.671
1 71.839 1 92.381
ENSMUSG00000022312 Eif3h protein_coding 1.12 0.976 0.916 42.923
0.975 39.528 0.97 92.31
ENSMUSG00000022313 Utp23 protein_coding 1.01 76.396 1.035
83.104 0.957 57.091 1.042 79.268
ENSMUSG00000022314 Rad21 protein_coding 0.96 55.939 0.876
60.678 1.187 11.151 1.043 83.276
ENSMUSG00000022315 Slc30a8 protein_coding 1 78.744 1
96.763 1 86.475 1 96.183
ENSMUSG00000022321 Cdh10 protein_coding 0.703 55.641 1.949
64.304 0.731 28.489 1.334 78.948
ENSMUSG00000022322 Shcbp1 protein_coding 1.531 22.633
0.814 73.094 0.801 70.791 1.615 69.404
ENSMUSG00000022323 Hrsp12 protein_coding 0.806 3.641 1
88.881 1.24 7.635 1.029 94.608
ENSMUSG00000022324 Matn2 protein_coding 0.674 0.987 1.27 71.617
1.172 16.666 1.115 46.846
ENSMUSG00000022325 Pop1 protein_coding 1.025 55.789 1.088
97.097 0.893 50.184 1.113 43.605
ENSMUSG00000022329 Stk3 protein_coding 1.078 36.124 0.952
86.861 0.975 75.353 1.047 60.466
ENSMUSG00000022330 Osr2 protein_coding 0.939 71.915 1.108
83.104 0.959 69.974 1.158 72.369
ENSMUSG00000022332 Khdrbs3 protein_coding 1.013 80.251
0.971 97.288 1.012 88.693 0.992 89.108
ENSMUSG00000022335 Zfat protein_coding 1.114 35.787 1.017
95.504 0.885 42.621 0.985 89.108
ENSMUSG00000022336 Eif3e protein_coding 1.126 0 0.892 48.565
0.997 32.082 0.988 87.218
ENSMUSG00000022337 Emc2 protein_coding 0.961 56.768 0.979
95.088 1.062 47.31 1.059 57.1
ENSMUSG00000022338 Eny2 protein_coding 0.999 77.497 1
63.773 1.003 37.764 0.983 96.248
ENSMUSG00000022339 Ebag9 protein_coding 1.087 11.316 0.965
90.256 0.949 36.786 1.019 85.786
ENSMUSG00000022340 Sybu protein_coding 0.853 68.66 1.016 97.155
1.15 64.024 1.087 93.261
ENSMUSG00000022342 Kcnv1 protein_coding 1 80.51 1 93.27 1
87.173 1 96.248
ENSMUSG00000022346 Myc protein_coding 0.915 52.726 0.946
81.731 1.158 19.609 1.174 91.492
ENSMUSG00000022350 E430025E21Rik protein_coding 1.123 0.824
1.055 83.104 0.845 0.72 0.99 95.593
ENSMUSG00000022351 Sqle protein_coding 1.075 81.52 1.027 92.793
0.903 71.999 1.038 85.577
ENSMUSG00000022353 Mtss1 protein_coding 0.972 47.677 1.066

83.104 0.967 88.274 0.929 83.724
ENSMUSG00000022354 Ndufb9 protein_coding 0.927 15.486
0.991 95.708 1.087 31.479 1.023 94.904
ENSMUSG00000022357 Klhl38 protein_coding 1.072 78.538
1.093 92.793 0.855 85.21 1.194 68.696
ENSMUSG00000022358 Fbxo32 protein_coding 0.855 4.268 1.027
83.104 1.142 28.301 1.037 90.455
ENSMUSG00000022359 Wdyhv1 protein_coding 1.072 23.82 1.021
96.843 0.914 23.1 0.898 83.724
ENSMUSG00000022360 Atad2 protein_coding 1.079 50.627 0.979
95.504 0.95 76.388 1.18 51.967
ENSMUSG00000022361 Zhx1 protein_coding 1.094 54.44 1.019 85.756
0.894 19.449 0.979 96.071
ENSMUSG00000022362 9130401M01Rik protein_coding 0.939 13.952
1.056 79.367 1.01 79.426 1.096 60.968
ENSMUSG00000022364 Tbc1d31 protein_coding 1.05 59.806
1.031 89.434 0.921 59.383 0.93 87.651
ENSMUSG00000022365 Derl1 protein_coding 0.907 1.15 1.062 83.104
1.039 32.524 0.929 84.19
ENSMUSG00000022367 Has2 protein_coding 1.254 28.098 1.19
95.211 0.669 21.852 1.667 59.441
ENSMUSG00000022369 NA NA NA NA 0.95 92.545 1.085
62.307 1.146 56.863
ENSMUSG00000022370 Mrpl13 protein_coding 1.005 59.211
1.029 96.407 0.963 67.029 1.087 42.736
ENSMUSG00000022371 Col14a1 protein_coding 0.943 65.622 NA
NA 1.021 84.308 0.861 92.181
ENSMUSG00000022372 Sla protein_coding 1.607 2.651 0.71 38.699
0.873 57.819 1.106 86.301
ENSMUSG00000022376 Adcy8 protein_coding 1.413 22.025 1.087
93.765 0.65 31.887 0.993 96.086
ENSMUSG00000022377 Asap1 protein_coding 1.292 0.2 0.95 72.576
0.816 2.563 1.023 72.616
ENSMUSG00000022378 Fam49b protein_coding 1.044 18.314
0.962 88.691 0.998 44.865 1.033 82.328
ENSMUSG00000022382 Wnt7b protein_coding 0.807 9.599 0.911 96.68
1.362 19.877 0.924 83.724
ENSMUSG00000022383 Ppara protein_coding 1.336 14.59 1.009 95.088
0.743 23.212 1.026 93.612
ENSMUSG00000022385 Gtse1 protein_coding 1.275 11.197 0.673
53.79 1.163 85.21 1.567 72.204
ENSMUSG00000022386 Trmu protein_coding 0.896 8.002 0.957 68.818
1.169 6.202 1.064 75.198
ENSMUSG00000022387 Brd1 protein_coding 1.015 66.76 0.97 82.848
1.013 80.381 0.985 95.92
ENSMUSG00000022389 Tef protein_coding 1.037 67.163 0.989
96.68 0.976 67.648 1.071 86.445
ENSMUSG00000022390 Zc3h7b protein_coding 0.972 37.531
1.039 79.055 0.992 84.552 0.994 94.016
ENSMUSG00000022391 Rangap1 protein_coding 0.953 33.906
0.992 95.504 1.053 34.807 1.129 33.269
ENSMUSG00000022394 L3mbtl2 protein_coding 0.967 49.749
1.017 93.574 1.02 76.923 1.02 86.445
ENSMUSG00000022400 Rbx1 protein_coding 0.922 11.924 0.961

93.094 1.126 15.688 1.033 71.653
ENSMUSG00000022401 Xpnpep3 protein_coding 0.882 7.43 1.038
83.104 1.09 19.025 1.073 80.77
ENSMUSG00000022403 St13 protein_coding 1.011 81.01 1.029 94.89
0.958 77.037 1.022 83.067
ENSMUSG00000022404 Slc25a17 protein_coding 0.914 44.467
0.938 42.923 1.17 14.449 1.068 90.455
ENSMUSG00000022407 Adsl protein_coding 1.025 45.746 0.993
90.378 0.983 83.158 0.988 79.268
ENSMUSG00000022408 Fam83f protein_coding 0.97 65.622
0.938 93.817 1.1 55.81 1.006 95.35
ENSMUSG00000022412 Mief1 protein_coding 1.044 64.956 0.993
96.407 0.966 28.489 0.966 83.724
ENSMUSG00000022414 Tab1 protein_coding 0.912 10.739 0.914
51.159 1.198 5.864 1.027 92.691
ENSMUSG00000022415 Syngr1 protein_coding 1.001 86.74 0.988
94.48 1.012 82.778 0.869 83.724
ENSMUSG00000022419 Deptor protein_coding 0.902 22.481
1.283 71.617 0.861 78.642 1.082 93.734
ENSMUSG00000022420 Dnal4 protein_coding 1.072 35.787 0.986
89.996 0.945 48.577 0.959 83.952
ENSMUSG00000022421 Nptxr protein_coding 1.037 74.958 0.887
85.242 1.085 75.527 0.922 84.29
ENSMUSG00000022422 Dscc1 protein_coding 1.477 21.146 0.723
63.773 0.935 78.954 1.191 69.404
ENSMUSG00000022425 Enpp2 protein_coding 1.187 23.019 NA NA
0.807 24.523 0.885 85.493
ENSMUSG00000022426 Josd1 protein_coding 1.017 16.74 1.023 89.713
0.959 32.703 0.976 84.29
ENSMUSG00000022427 Tomm22 protein_coding 1.048 38.682 NA
NA 0.949 48.387 1.048 72.369
ENSMUSG00000022428 Cby1 protein_coding 1.008 67.477 1.15
71.617 0.863 15.63 0.998 83.067
ENSMUSG00000022429 Dmc1 protein_coding 1.407 28.395 1.161
96.216 0.613 39.079 0.732 86.254
ENSMUSG00000022432 Smc1b protein_coding 2.144 43.654 0.502
96.138 0.933 84.434 2.149 87.7
ENSMUSG00000022433 Csnk1e protein_coding 0.932 26.217
0.998 95.708 1.076 36.011 0.974 87.651
ENSMUSG00000022434 Fam118a protein_coding 1.165 0.577 0.911
70.971 0.94 22.279 1.086 76.467
ENSMUSG00000022435 Upk3a protein_coding 0.351 22.92 0.454 47.831
6.277 5.864 1.953 60.31
ENSMUSG00000022436 Sh3bp1 protein_coding 0.967 74.689
1.074 84.768 0.965 63.504 1.097 71.305
ENSMUSG00000022437 Samm50 protein_coding 1.01 84.564
1.052 89.494 0.939 42.895 1.008 96.071
ENSMUSG00000022438 Parvb protein_coding 0.945 41.021 0.904
82.848 1.172 37.259 1.013 73.597
ENSMUSG00000022439 Parvg protein_coding 1.247 16.539 0.978
96.68 0.823 23.212 0.853 86.699
ENSMUSG00000022440 C1qtnf6 protein_coding 1.208 7.501 0.963
95.708 0.861 8.634 1.272 0
ENSMUSG00000022442 Ttll1 protein_coding 1.126 14.34 0.989 88.91

0.898 1.871 1.041 92.748
ENSMUSG00000022443 Myh9 protein_coding 0.92 5.311 1.021 97.263
1.066 10.76 0.931 90.289
ENSMUSG00000022445 Cyp2d26 protein_coding 0.344 22.92 1.075
96.984 2.697 27.047 1.699 96.287
ENSMUSG00000022449 Adamts20 protein_coding 0.784 22.92 1.079
91.584 1.179 47.935 1.255 72.616
ENSMUSG00000022450 Ndufa6 protein_coding 0.947 43.495
0.921 73.766 1.143 24.029 1.018 95.559
ENSMUSG00000022451 Twf1 protein_coding 1.04 35.787 0.965
90.378 0.995 59.922 1.067 74.193
ENSMUSG00000022452 Smdt1 protein_coding 0.775 3.07 1.14 78.494
1.135 31.317 1.083 95.74
ENSMUSG00000022453 Naga protein_coding 1.075 9.805 0.916 21.315
1.016 61.171 1.024 85.577
ENSMUSG00000022454 Nell2 protein_coding 1.92 13.363 0.874
96.013 0.595 23.938 0.823 90.611
ENSMUSG00000022455 Wbp2nl protein_coding 1.337 72 0.408
82.947 1.842 81.29 1.387 94.904
ENSMUSG00000022456 3-Sep protein_coding 0.902 43.167 1.069
84.007 1.037 86.475 0.941 96.183
ENSMUSG00000022462 Slc38a2 protein_coding 1.013 77.291
1.111 79.261 0.89 11.314 1.021 95.273
ENSMUSG00000022463 Srebf2 protein_coding 1.048 82.231
1.086 82.904 0.878 17.258 1.078 67.691
ENSMUSG00000022464 Slc38a4 protein_coding 1.626 2.233 0.96
96.68 0.641 3.123 1.114 83.067
ENSMUSG00000022466 Rpap3 protein_coding 1.074 37.091 0.99
74.483 NA NA 0.996 78.546
ENSMUSG00000022468 Endou protein_coding 1.215 38.682 0.708
79.261 1.164 85.792 1.345 67.691
ENSMUSG00000022469 Rapgef3 protein_coding 1.011 66.129
0.849 48.565 1.167 21.201 0.954 92.381
ENSMUSG00000022471 Xrcc6 protein_coding 0.937 53.138 0.957
84.594 1.111 41.235 1.006 96.192
ENSMUSG00000022472 Desi1 protein_coding 0.924 25.551 0.978
90.092 1.103 59.274 0.969 86.699
ENSMUSG00000022474 Pmm1 protein_coding 0.844 4.941 1.023 95.196
1.158 31.4 1.005 91.979
ENSMUSG00000022475 Hdac7 protein_coding 0.874 6.952 1.045 96.013
1.095 12.831 1.019 80.573
ENSMUSG00000022476 Polr3h protein_coding 0.933 21.658
0.987 93.899 1.085 43.586 1.027 72.616
ENSMUSG00000022477 Aco2 protein_coding 0.903 19.224 NA NA
1.116 31.761 1.047 95.273
ENSMUSG00000022479 Vdr protein_coding 0.879 51.056 0.977
87.322 1.167 29.302 0.992 85.493
ENSMUSG00000022483 Col2a1 protein_coding 0.507 20.116
0.997 93.27 1.98 26.912 1.134 96.071
ENSMUSG00000022484 Hoxc10 protein_coding 1.053 71.217
1.134 75.62 0.837 11.902 1.033 83.067
ENSMUSG00000022485 Hoxc5 protein_coding 0.981 83.09 1.2 71.617
0.851 6.858 1.115 87.447
ENSMUSG00000022487 Gtsf1 protein_coding 1 74.8 1 96.948

1 84.626 1 86.445
ENSMUSG00000022488 Nckap1l protein_coding 1.434 0 0.869
85.075 0.8 4.723 1.161 60.968
ENSMUSG00000022489 Pde1b protein_coding 1.265 8.365 0.946 95.708
0.837 15.169 0.9 84.29
ENSMUSG00000022490 Ppp1r1a protein_coding 0.869 5.311 1.188
63.504 0.97 81.388 0.828 83.724
ENSMUSG00000022494 Shisa9 protein_coding 0.431 68.846 0.56
93.654 4.162 56.708 1.749 81.061
ENSMUSG00000022496 Tnfrsf17 protein_coding 2.023 15.456
0.771 88.691 0.639 35.559 0.755 91.985
ENSMUSG00000022498 Txndc11 protein_coding 1.049 61.171 1.06
72.642 0.9 39.079 1.017 93.964
ENSMUSG00000022500 Litaf protein_coding 1.242 0.577 0.944 65.819
0.852 0.879 1.014 94.608
ENSMUSG00000022501 Prm1 protein_coding 1 72.27 1 85.756
1 54.25 1 95.92
ENSMUSG00000022503 Nubp1 protein_coding 0.924 36.823 1.063
71.617 1.018 83.607 1.057 74.047
ENSMUSG00000022504 Ciita protein_coding 1.23 21.703 1.276
90.116 0.635 62.791 0.827 83.724
ENSMUSG00000022505 Emp2 protein_coding 1.144 20.799 0.96
88.704 0.912 46.575 0.93 88.387
ENSMUSG00000022507 1810013L24Rik protein_coding 1.051 38.682
1.058 87.037 0.897 9.901 0.896 83.724
ENSMUSG00000022508 Bcl6 protein_coding 0.823 18.602 1.162
79.29 1.045 75.527 1.36 64.513
ENSMUSG00000022510 Trp63 protein_coding 0.768 22.237 1.224
80.775 1.059 70.623 0.997 93.316
ENSMUSG00000022512 Cldn1 protein_coding 0.799 9.599 1.115 83.104
1.116 31.975 0.94 91.078
ENSMUSG00000022514 Il1rap protein_coding 1.021 85.499 NA
NA 1.021 85.58 1.071 94.99
ENSMUSG00000022515 Anks3 protein_coding 0.948 4.268 0.97 96.216
1.092 33.787 1.006 88.755
ENSMUSG00000022516 Nudt16l1 protein_coding 0.982 49.621
1.013 85.756 NA NA 1.063 63.708
ENSMUSG00000022517 Mgrn1 protein_coding 1.033 30.497 1.028
83.104 0.937 16.409 0.978 83.952
ENSMUSG00000022518 4930562C15Rik protein_coding 0.6 9.599
0.907 82.947 1.842 7.024 0.789 92.181
ENSMUSG00000022519 Srl protein_coding 2.165 18.635 1.113
80.678 0.416 2.453 0.825 96.183
ENSMUSG00000022521 Crebbp protein_coding 0.944 21.176
1.076 64.304 0.986 49.217 0.955 83.724
ENSMUSG00000022523 Fgf12 protein_coding 1.073 64.956 0.861
90.256 1.078 69.728 0.805 94.174
ENSMUSG00000022525 Hrasls protein_coding 1.624 52.134
1.898 82.904 0.324 6.858 1.303 87.447
ENSMUSG00000022526 Zfp251 protein_coding 1.091 13.633
1.013 96.407 0.906 13.643 0.994 95.877
ENSMUSG00000022528 Hes1 protein_coding 0.956 57.014 0.967
95.504 1.08 51.977 0.78 96.287
ENSMUSG00000022529 Zfp263 protein_coding 1.067 33.099

0.951 73.166 0.986 80.015 0.96 93.964
ENSMUSG00000022533 Atp13a3 protein_coding 1.149 28.927
1.061 96.216 0.819 18.539 1.129 90.674
ENSMUSG00000022534 Mefv protein_coding 1.256 49.517 1.02
91.661 0.781 51.715 0.943 82.091
ENSMUSG00000022536 Glyr1 protein_coding 0.977 51.56 1.008 96.843
1.017 57.227 1.019 80.497
ENSMUSG00000022537 Tmem44 protein_coding 1.06 46.875
0.861 42.923 1.091 44.515 0.932 88.552
ENSMUSG00000022538 Lsg1 protein_coding 1.035 68.727 0.955
73.766 1.014 67.562 1.045 73.939
ENSMUSG00000022540 Rogdi protein_coding 1.037 57.38 1.029 97.263
0.934 61.743 0.963 91.65
ENSMUSG00000022542 12-Sep protein_coding 1 24.147 1
94.102 1 69.583 1 92.466
ENSMUSG00000022543 4930451G09Rik protein_coding 1.031 85.092
1.032 96.843 0.94 70.791 0.907 90.611
ENSMUSG00000022544 Fam86 protein_coding 0.942 30.637 1.021
87.604 1.041 50.636 1.018 78.379
ENSMUSG00000022545 Ercc4 protein_coding 1.01 85.77 0.924 53.434
1.07 10.281 0.998 96.183
ENSMUSG00000022546 Gpt protein_coding 0.683 0.987 1.104 71.98
1.326 20.085 0.906 92.31
ENSMUSG00000022548 Apod protein_coding 1.059 85.283 0.847
73.766 1.118 67.823 0.884 86.699
ENSMUSG00000022550 Adck5 protein_coding 0.92 51.447 1.031
97.155 1.057 62.307 0.942 84.29
ENSMUSG00000022551 Cyc1 protein_coding 0.859 3.623 1.112 79.29
1.049 43.586 NA NA
ENSMUSG00000022552 Sharpin protein_coding 0.96 63.126
0.992 96.407 1.047 74.082 1.016 96.086
ENSMUSG00000022553 Maf1 protein_coding 0.952 28.242 NA NA
1.058 43.481 0.963 83.724
ENSMUSG00000022554 Fam203a protein_coding 0.949 77.615 NA
NA 1.063 72.853 1.068 96.358
ENSMUSG00000022555 Dgat1 protein_coding 0.833 4.941 1.042 92.111
1.153 28.74 0.961 83.724
ENSMUSG00000022556 Hsf1 protein_coding 1.027 77.182 1.001
83.104 0.969 24.309 0.972 90.611
ENSMUSG00000022557 Bop1 protein_coding 0.897 26.483 1.068
79.579 1.039 70.1 1.064 74.193
ENSMUSG00000022558 Mroh1 protein_coding 0.993 71.06 0.96 65.819
1.051 59.612 0.951 83.724
ENSMUSG00000022559 Fbxl6 protein_coding 0.979 61.887 0.963
83.392 1.062 50.636 1.014 96.358
ENSMUSG00000022560 Slc52a2 protein_coding 0.99 57.014 1.05
73.092 0.964 56.365 1.019 90.177
ENSMUSG00000022561 Gpaa1 protein_coding 0.885 3.009 1.102 40.99
1.024 75.155 1.064 57.075
ENSMUSG00000022562 Oplah protein_coding 0.937 42.922 1.014
95.431 1.051 80.899 0.984 91.985
ENSMUSG00000022564 Grina protein_coding 1.04 84.977 0.954
96.268 1.007 81.388 0.968 88.955
ENSMUSG00000022565 Plec protein_coding 0.881 44.252 1.161

79.055 0.979 81.525 1.012 95.74
ENSMUSG00000022568 Scrib protein_coding 0.891 1.343 0.991 95.088
1.133 7.855 0.992 95.35
ENSMUSG00000022570 Tsta3 protein_coding 0.96 46.413 0.976
75.493 1.067 19.106 1.027 79.713
ENSMUSG00000022571 Pycrl protein_coding 0.857 12.181 1.054
92.561 1.11 25.808 1.029 82.85
ENSMUSG00000022574 Naprt1 protein_coding 0.923 24 1.068
82.904 1.015 84.896 1.033 92.018
ENSMUSG00000022575 Gsdmd protein_coding 1.12 6.683 0.935 73.166
0.955 57.446 1.048 57.075
ENSMUSG00000022577 Ly6h protein_coding 0.579 1.422 1.291 71.617
1.341 24.383 1.062 94.297
ENSMUSG00000022579 Gpihbp1 protein_coding 0.699 0.987 NA NA
1.397 5.864 0.971 88.387
ENSMUSG00000022580 Rhpn1 protein_coding 0.844 48.354 NA NA
1.28 54.363 1.139 96.192
ENSMUSG00000022582 Ly6g protein_coding 1 76.042 1
82.073 1 75.009 1 92.31
ENSMUSG00000022583 Ly6f protein_coding 1 72.985 1
95.708 1 83.857 1 93.156
ENSMUSG00000022584 Ly6c2 protein_coding 3.924 0.2 0.931 91.361
0.273 2.722 0.673 93.734
ENSMUSG00000022586 Ly6i protein_coding 0.823 60.569 0.796
85.671 1.527 25.564 1.023 93.156
ENSMUSG00000022587 Ly6e protein_coding 1.048 20.659 0.985
91.955 0.966 56.625 0.964 86.991
ENSMUSG00000022589 Cyp11b2 protein_coding 1 78.744 NA
NA 1 83.538 1 91.492
ENSMUSG00000022591 Gm9747 protein_coding 0.79 28.514
1.378 64.304 0.92 80.5 0.782 90.674
ENSMUSG00000022594 Lynx1 protein_coding 1.147 3.536 1.063 89.434
0.817 2.189 1.092 44.075
ENSMUSG00000022595 Lypd2 protein_coding 1.423 68.727 1.429
82.904 0.492 8.626 1.087 88.197
ENSMUSG00000022596 Slurp1 protein_coding 1 84.324 1
95.211 1 84.504 1 96.358
ENSMUSG00000022601 Rpl24 protein_coding 1.02 57.949 1.046
82.904 0.935 10.726 1.01 94.82
ENSMUSG00000022602 Arc protein_coding 1.09 86.172 1.15
89.996 0.796 54.037 0.862 96.071
ENSMUSG00000022604 Cep97 protein_coding 0.848 8.45 1.049 94.367
1.125 27.89 0.972 86.991
ENSMUSG00000022607 Ptk2 protein_coding 0.999 83.09 0.987 95.329
1.012 80.381 1.061 31.133
ENSMUSG00000022610 Mapk12 protein_coding 1.031 68.727
1.048 88.823 0.925 35.559 1.01 96.042
ENSMUSG00000022613 Miox protein_coding 1 85.857 1
92.137 1 83.925 1 94.153
ENSMUSG00000022614 Lmf2 protein_coding 0.955 33.906 1.07
71.617 0.981 65.409 1.021 88.755
ENSMUSG00000022615 Tymp protein_coding 1.034 72.372 1.414
87.604 0.683 37.103 0.689 84.29
ENSMUSG00000022617 Chkb protein_coding 0.914 9.863 1.059 82.904

1.032 45.035 0.997 96.192
ENSMUSG00000022619 Mapk8ip2 protein_coding 0.896 80.251
1.048 96.216 1.062 76.923 0.871 96.287
ENSMUSG00000022620 Arsa protein_coding 0.943 60.569 1.007
96.001 1.055 67.562 1.053 83.276
ENSMUSG00000022621 Rabl2 protein_coding 0.945 43.435 1.062
83.104 0.997 85.757 1.022 91.872
ENSMUSG00000022622 Acr protein_coding 0.516 6.221 0.707 62.692
2.745 5.864 1.125 92.691
ENSMUSG00000022623 Shank3 protein_coding 0.803 3.448 1.088
86.387 1.148 16.808 0.92 83.724
ENSMUSG00000022629 Kif21a protein_coding 0.88 1.991 1.092
82.809 1.042 38.099 1.068 66.068
ENSMUSG00000022634 Yaf2 protein_coding 0.966 43.495 0.915
95.504 1.127 43.307 1.021 92.903
ENSMUSG00000022635 Zcrb1 protein_coding 0.949 10.014 0.931
72.576 1.129 6.202 1.022 85.629
ENSMUSG00000022636 Alcam protein_coding 0.942 46.289 0.933
62.322 1.132 25.808 0.724 86.445
ENSMUSG00000022637 Cblb protein_coding 0.945 67.477 0.773
47.831 1.366 9.429 1.162 86.445
ENSMUSG00000022639 5330426P16Rik protein_coding 0.938 86.474
0.986 84.594 1.078 78.155 1.015 93.264
ENSMUSG00000022641 Bbx protein_coding 0.962 75.761 1.114
89.014 0.932 65.688 0.916 83.952
ENSMUSG00000022650 Retnlb protein_coding 1 68.66 1
95.35 1 80.381 1 93.156
ENSMUSG00000022651 Retnlg protein_coding 1 62.689
0.532 74.851 1.878 79.625 1.36 96.287
ENSMUSG00000022652 Morc1 protein_coding 0.556 63.126 NA NA
1.458 57.907 1.3 93.722
ENSMUSG00000022656 Pvrl3 protein_coding 1.133 20.659 0.956
91.955 0.922 39.528 0.956 95.273
ENSMUSG00000022657 Cd96 protein_coding 1.276 53.228 1.695
66.01 0.464 5.564 0.618 83.952
ENSMUSG00000022658 Tagln3 protein_coding 1.02 86.843
1.048 83.104 0.935 49.967 0.877 91.217
ENSMUSG00000022659 Gcsam protein_coding 1.106 24.689 0.988
95.088 0.918 31.887 1.002 88.505
ENSMUSG00000022661 Cd200 protein_coding 1.035 86.199 1.234
60.309 0.784 11.314 1.016 87.088
ENSMUSG00000022663 Atg3 protein_coding 1.015 62.689 1.042
73.092 0.948 10.362 1.101 65.558
ENSMUSG00000022664 Slc35a5 protein_coding 1.134 3.176 0.968
87.041 0.906 3.943 0.913 83.724
ENSMUSG00000022665 Ccdc80 protein_coding 0.832 17.664
1.054 93.899 1.137 42.522 1.187 61.984
ENSMUSG00000022667 Cd200r1 protein_coding 1.821 0.577 0.866
81.731 0.635 15.169 1.015 94.904
ENSMUSG00000022668 Gtpbp8 protein_coding 1.02 85.832
0.908 73.766 1.08 49.384 1.031 92.018
ENSMUSG00000022671 Mzt2 protein_coding 0.947 49.621 1.138
71.617 0.929 44.865 0.954 89.475
ENSMUSG00000022672 Prkdc protein_coding 0.943 65.622 1.036

95.301 1.025 50.851 1 96.358
ENSMUSG00000022673 Mcm4 protein_coding 1.217 14.966 0.917
74.851 0.895 50.523 1.082 42.736
ENSMUSG00000022674 Ube2v2 protein_coding 1.164 5.253 0.919
87.553 0.937 9.573 1.009 83.581
ENSMUSG00000022676 Snai2 protein_coding 1.046 45.096 1.167
72.546 0.818 4.723 1.136 63.708
ENSMUSG00000022677 Fopnl protein_coding 0.986 84.534 0.952
87.998 1.068 72.378 1.048 73.823
ENSMUSG00000022678 Nde1 protein_coding 0.992 67.867 1.039
89.494 0.972 82.903 1.032 49.806
ENSMUSG00000022679 Mpv17l protein_coding 1.006 78.538
1.029 94.597 0.965 74.325 0.984 87.496
ENSMUSG00000022680 Pdxdc1 protein_coding 1.024 78.639
0.972 94.102 1.005 82.161 1.013 60.466
ENSMUSG00000022681 Ntan1 protein_coding 1.04 44.334 1.018
93.654 0.943 73.531 0.975 92.31
ENSMUSG00000022682 Rrn3 protein_coding 1.132 2.352 1.026 90.092
0.859 1.342 1 87.7
ENSMUSG00000022684 Bfar protein_coding 1.03 86.199 0.99
96.68 0.982 60.233 1.049 43.377
ENSMUSG00000022685 Parn protein_coding 1.045 26.863 0.979
90.287 0.973 41.928 1.012 91.625
ENSMUSG00000022686 B3gnt5 protein_coding 1.072 84.865
1.172 82.904 0.796 29.765 0.803 85.493
ENSMUSG00000022687 Boc protein_coding 0.952 50.229 1.041
83.464 1.007 79.625 0.912 83.724
ENSMUSG00000022696 Sidt1 protein_coding 1.065 54.44 0.896 92.137
1.042 81.388 0.644 83.724
ENSMUSG00000022698 Naa50 protein_coding 1.073 45.096 NA NA
0.93 19.765 1.111 52.324
ENSMUSG00000022701 2610015P09Rik protein_coding 0.962 43.816
0.847 55.71 1.229 14.169 1.005 94.82
ENSMUSG00000022702 Hira protein_coding 1 74.021 1.039
86.156 0.958 73.598 0.957 92.675
ENSMUSG00000022704 Qtrtd1 protein_coding 1.027 68.727 NA
NA 0.957 86.112 1.001 93.156
ENSMUSG00000022705 Drd3 protein_coding 1 85.637 1
96.68 1 51.055 1 96.192
ENSMUSG00000022706 Mrpl40 protein_coding 0.913 2.009 1.048
83.104 1.048 34.727 1.061 59.394
ENSMUSG00000022707 Gbe1 protein_coding 0.548 0.987 0.995 92.561
1.831 5.864 1.092 92.018
ENSMUSG00000022708 Zbtb20 protein_coding 0.97 47.266
1.004 93.27 1.028 69.315 0.999 95.559
ENSMUSG00000022710 Usp7 protein_coding 1.073 10.595 0.972
81.731 0.959 22.864 1.019 77.245
ENSMUSG00000022711 Pmm2 protein_coding 1.034 62.574 0.978
82.671 0.99 80.089 1.024 92.018
ENSMUSG00000022718 Dgcr8 protein_coding 1.022 80.751 0.98
95.35 0.996 78.864 1.044 49.806
ENSMUSG00000022721 Trmt2a protein_coding 1.024 82.092 0.98
77.598 0.992 69.002 1.067 67.169
ENSMUSG00000022722 Arl6 protein_coding 1.045 55.462 1.008

90.454 0.951 45.799 1.042 94.215
ENSMUSG00000022723 Crybg3 protein_coding 1.025 18.388
0.995 81.731 0.981 62.058 0.893 83.724
ENSMUSG00000022724 Mina protein_coding 1.003 82.891 0.985
96.216 1.01 84.83 1.033 91.335
ENSMUSG00000022742 Cpox protein_coding 1.044 67.163 1.024
82.904 0.931 16.409 1.045 71.653
ENSMUSG00000022744 Cldn25 protein_coding 1.028 64.193
0.995 95.504 0.974 64.155 0.933 89.525
ENSMUSG00000022747 St3gal6 protein_coding 0.966 75.761
0.885 90.576 1.166 43.307 0.886 84.29
ENSMUSG00000022748 Cmss1 protein_coding 1.036 69.408 0.994
91.361 0.968 87.686 1.083 74.047
ENSMUSG00000022749 Tbc1d23 protein_coding 1.057 12.855
1.002 86.604 0.943 25.688 0.945 84.29
ENSMUSG00000022750 Klhl22 protein_coding 1.103 4.874 0.954
71.41 0.953 21.331 0.945 84.29
ENSMUSG00000022751 Nit2 protein_coding 0.944 17.898 0.996
95.301 1.061 29.819 0.985 85.636
ENSMUSG00000022752 Tomm70a protein_coding 1.152 2.569 0.972
88.691 0.893 2.453 0.957 94.608
ENSMUSG00000022753 Tmem30c protein_coding 0.537 63.126
1.311 96.843 1.412 87.971 1.071 90.611
ENSMUSG00000022754 Tmem45a protein_coding 1.222 13.492 1.02
87.609 0.803 21.431 1.24 48.119
ENSMUSG00000022755 Gpr128 protein_coding 2.353 56.907
0.251 66.651 1.699 49.898 1.274 78.546
ENSMUSG00000022756 Slc7a4 protein_coding 0.859 20.116 0.89
82.073 1.306 20.315 1.027 90.95
ENSMUSG00000022757 Tfg protein_coding 1.106 6.958 1.049 82.809
0.865 1.061 1.007 94.016
ENSMUSG00000022758 P2rx6 protein_coding 0.674 1.15 1.244 72.546
1.194 18.411 1.075 83.067
ENSMUSG00000022759 4930451C15Rik protein_coding 1.55 40.635
0.428 73.166 1.501 48.935 1.006 87.496
ENSMUSG00000022760 Thap7 protein_coding 0.956 40.786 1.035
84.999 NA NA 0.964 91.291
ENSMUSG00000022761 Lztr1 protein_coding 1.08 7.049 0.992 85.116
0.929 64.778 0.994 95.273
ENSMUSG00000022762 Ncam2 protein_coding 0.992 83.169 1.107
84.007 0.912 47.12 1.021 95.273
ENSMUSG00000022763 Aifm3 protein_coding 0.986 86.333 0.735
39.547 1.384 27.533 1.023 96.183
ENSMUSG00000022765 Snap29 protein_coding 1.079 0.461 0.993
93.765 0.933 11.751 1.052 63.708
ENSMUSG00000022766 Serpind1 protein_coding 0.833 79.93 0.961
90.256 1.25 67.823 1.455 61.753
ENSMUSG00000022768 Ccdc116 protein_coding 0.846 58.802
1.636 82.904 0.72 52.213 0.946 95.877
ENSMUSG00000022769 Sdf2l1 protein_coding 1.028 55.124 NA
NA 0.864 61.288 1.101 56.769
ENSMUSG00000022770 Dlg1 protein_coding 1.067 5.016 0.938 34.058
0.994 88.41 1.012 89.498
ENSMUSG00000022771 Ppil2 protein_coding 0.96 11.973 0.974

63.773 1.068 7.24 1.036 68.696
ENSMUSG00000022772 Senp5 protein_coding 0.929 37.248 1.023
83.819 1.05 81.29 0.997 92.748
ENSMUSG00000022773 Ypel1 protein_coding 0.736 11.232 1.146
96.407 1.179 13.174 0.809 83.724
ENSMUSG00000022774 Ncbp2 protein_coding 1.019 83.823 1.043
96.216 0.943 84.724 0.979 94.904
ENSMUSG00000022779 Top3b protein_coding 0.967 11.232 0.955
88.704 1.081 20.085 0.978 86.991
ENSMUSG00000022780 Mfi2 protein_coding 0.988 78.909 0.735
87.452 1.371 31.479 0.908 85.493
ENSMUSG00000022781 Pak2 protein_coding 1.042 7.397 0.996 95.431
0.965 5.628 1.031 85.786
ENSMUSG00000022783 Spag6 protein_coding 0.866 72.743 1.222
96.216 0.946 78.155 1.187 87.134
ENSMUSG00000022787 Wdr53 protein_coding 0.988 75.399 0.899
45.262 1.129 11.932 1.032 93.156
ENSMUSG00000022788 Fgd4 protein_coding 1.155 6.438 0.936 71.889
0.926 46.071 0.95 83.724
ENSMUSG00000022789 Dnm1l protein_coding 0.98 48.354 1.028
93.994 0.996 72.148 0.995 77.854
ENSMUSG00000022790 Igsf11 protein_coding 0.823 61.633
1.073 95.708 1.132 73.309 1.386 66.944
ENSMUSG00000022791 Tnk2 protein_coding 1.188 13.734 0.991
95.211 0.852 7.339 0.943 94.654
ENSMUSG00000022792 Yars2 protein_coding 1.042 83.658 NA NA
0.964 85.134 0.952 85.493
ENSMUSG00000022793 B4galt4 protein_coding 1.046 61.49 0.94
82.503 1.018 71.615 1.009 91.872
ENSMUSG00000022797 Tfrc protein_coding 0.974 57.014 1.003
96.847 1.024 58.051 0.793 95.593
ENSMUSG00000022798 4930435E12Rik protein_coding 1 81.01 1
95.941 NA NA 1 92.466
ENSMUSG00000022799 Arhgap31 protein_coding 1.064 10.391 1
95.735 0.942 5.701 0.998 91.217
ENSMUSG00000022800 Fyttd1 protein_coding 1.157 0 0.929
35.072 0.931 20.258 0.966 83.724
ENSMUSG00000022801 Lrch3 protein_coding 0.97 77.615 1.069
85.756 0.965 73.598 1.002 90.289
ENSMUSG00000022802 Lmln protein_coding 1.038 59.211 0.918
71.98 1.044 55.352 0.911 84.29
ENSMUSG00000022803 Popdc2 protein_coding 0.899 22.431
1.141 81.039 0.976 85.675 1.165 76.831
ENSMUSG00000022805 Maats1 protein_coding 0.852 57.544
0.827 90.053 1.412 49.384 1.095 94.904
ENSMUSG00000022807 Osbpl11 protein_coding 1.065 72.655
0.887 61.883 1.062 45.035 0.982 95.877
ENSMUSG00000022808 Snx4 protein_coding 1.107 0.461 NA NA
0.899 1.061 0.984 95.273
ENSMUSG00000022809 Nr1i2 protein_coding 1 62.158 1
90.712 1 55.352 1 87.447
ENSMUSG00000022811 Zfp148 protein_coding 0.987 75.567
0.952 82.503 1.065 22.131 1.051 90.674
ENSMUSG00000022812 Gsk3b protein_coding 1.025 36.438 1.068

60.309 0.914 2.986 1.014 91.525
ENSMUSG00000022814 Umps protein_coding 1.145 2.651 0.938 77.944
0.929 31.676 1.041 49.806
ENSMUSG00000022816 Fstl1 protein_coding 1.36 0.355 0.869 73.766
0.843 9.494 1.205 42.736
ENSMUSG00000022817 Itgb5 protein_coding 1.046 52.799 1.001
91.661 0.951 87.686 0.986 92.181
ENSMUSG00000022818 Cyp2ab1 protein_coding 1.282 21.146
0.653 21.315 1.19 43.129 0.7 86.991
ENSMUSG00000022820 Ndufb4 protein_coding 0.871 3.47 1.076
74.663 1.064 34.807 0.932 86.699
ENSMUSG00000022821 Hgd protein_coding 0.958 54.87 0.635 93.908
1.642 46.575 1.318 91.525
ENSMUSG00000022822 Abcc5 protein_coding 0.915 19.224 1.028
97.263 1.061 70.623 1.019 96.086
ENSMUSG00000022824 Muc13 protein_coding 1.179 44.334 0.732
60.678 1.162 79.625 1.886 54.265
ENSMUSG00000022827 Rabl3 protein_coding 1.063 84.738 0.916
86.861 1.031 66.269 1.038 88.111
ENSMUSG00000022828 Gtf2e1 protein_coding 1.072 20.296
1.097 88.533 0.851 28.901 1.011 94.297
ENSMUSG00000022831 Hcls1 protein_coding 1.144 1.643 0.901 70.693
0.969 39.755 1.197 71.305
ENSMUSG00000022832 Ropn1 protein_coding 1 83.169 1
96.68 1 87.37 1 95.676
ENSMUSG00000022833 Ccdc14 protein_coding 0.874 28.804
0.926 73.766 1.232 7.365 1.053 94.82
ENSMUSG00000022836 Mylk protein_coding 0.971 27.836 1.049
83.464 0.984 81.032 0.942 91.65
ENSMUSG00000022837 Iqcb1 protein_coding 1.057 60.168 0.928
78.877 1.017 83.538 0.953 86.991
ENSMUSG00000022838 Eaf2 protein_coding 0.793 14.427 0.918
87.147 1.376 14.449 0.846 85.493
ENSMUSG00000022840 Adcy5 protein_coding 0.984 85.283 1.259
78.629 0.807 23.938 1.567 95.273
ENSMUSG00000022841 Ap2m1 protein_coding 1.077 3.893 1.026 92.931
0.904 7.742 1.135 33.269
ENSMUSG00000022842 Ece2 protein_coding 1.142 21.146 0.97
94.367 0.905 18.336 1.073 83.067
ENSMUSG00000022843 Clcn2 protein_coding 0.764 3.009 1.026 82.904
1.272 28.603 1.117 91.783
ENSMUSG00000022844 Pdia5 protein_coding 1.236 11.879 0.984
95.708 0.823 19.765 1.121 36.988
ENSMUSG00000022847 Thpo protein_coding 0.87 30.352 1.104
96.407 1.044 61.418 0.973 96.071
ENSMUSG00000022848 Dirc2 protein_coding 1.045 45.307 0.925
55.265 1.032 41.331 0.936 84.29
ENSMUSG00000022849 Hspbap1 protein_coding 0.979 86.199
0.929 70.29 1.096 32.173 1.079 79.442
ENSMUSG00000022853 Ehhadh protein_coding 1.013 72.655
1.111 87.029 0.886 9.456 0.861 83.724
ENSMUSG00000022855 Senp2 protein_coding 1.023 63.804 0.952
62.71 1.025 43.185 0.966 90.854
ENSMUSG00000022856 NA NA NA NA 0.987 96.847 0.98

87.519 0.9 90.157
ENSMUSG00000022857 Tmprss15 protein_coding 1 74.482 1
90.092 1 86.666 1 95.92
ENSMUSG00000022858 Tra2b protein_coding 1.124 2.047 1.038 88.923
0.857 0.72 0.992 96.071
ENSMUSG00000022860 Chodl protein_coding 1.126 61.725 0.504
56.781 1.759 43.307 0.415 87.992
ENSMUSG00000022861 Dgkg protein_coding 0.811 64.014 1.491
95.196 0.827 87.744 1.332 60.968
ENSMUSG00000022863 Btg3 protein_coding 1.177 5.527 0.934 87.857
0.909 17.898 1.003 96.358
ENSMUSG00000022864 D16Ertd472e protein_coding 1.042 79.433
1.056 94.767 0.906 78.954 0.999 91.372
ENSMUSG00000022865 Cxadr protein_coding 1.109 38.682 1.044
96.138 0.863 60.96 0.773 83.724
ENSMUSG00000022867 Usp25 protein_coding 1.08 15.662 1.004
91.643 0.922 2.889 0.993 94.904
ENSMUSG00000022868 Ahsg protein_coding 1.785 35.371 0.352
75.493 1.587 75.009 0.849 96.287
ENSMUSG00000022871 Fetub protein_coding 1.288 62.689 NA NA
0.574 49.217 0.862 96.183
ENSMUSG00000022875 Kng1 protein_coding 1 81.196 1
95.809 1 87.786 1 96.183
ENSMUSG00000022876 Samsn1 protein_coding 1.387 1.712 0.853
59.946 0.841 60.233 1.145 74.607
ENSMUSG00000022877 Hrg protein_coding 1 79.709 1
95.504 1 79.897 1 96.248
ENSMUSG00000022878 Adipoq protein_coding 1.098 13.633
0.986 96.843 0.92 25.688 0.926 83.724
ENSMUSG00000022881 Rfc4 protein_coding 1.305 0.355 0.87 72.399
0.88 15.904 1.125 64.513
ENSMUSG00000022883 Robo1 protein_coding 1.007 81.01 0.928 85.671
1.073 49.601 NA NA
ENSMUSG00000022884 Eif4a2 protein_coding 1.068 17.336
0.836 46.547 1.122 44.515 1.074 95.676
ENSMUSG00000022885 St6gal1 protein_coding 1.215 2.723 0.99
85.671 0.828 28.669 0.963 92.466
ENSMUSG00000022886 Prl2a1 protein_coding 1 84.324 1
96.843 1 86.985 1 96.071
ENSMUSG00000022887 Masp1 protein_coding 0.815 26.217 1.317
75.612 0.933 73.874 0.989 84.29
ENSMUSG00000022889 Mrpl39 protein_coding 0.956 45.229 1
96.68 1.043 57.907 1.001 83.067
ENSMUSG00000022890 Atp5j protein_coding 0.908 4.176 1.058 79.367
1.042 56.497 1.007 93.316
ENSMUSG00000022892 App protein_coding 1.054 23.019 0.968
93.046 0.977 64.155 0.956 84.29
ENSMUSG00000022893 Adamts1 protein_coding 1.171 29.057
1.092 79.055 0.78 1.898 1.185 87.134
ENSMUSG00000022894 Adamts5 protein_coding 1.013 81.657 0.99
94.262 0.996 84.229 0.973 90.289
ENSMUSG00000022895 Ets2 protein_coding 1.152 35.024 1.107
82.904 0.784 7.647 0.903 86.991
ENSMUSG00000022897 Dyrk1a protein_coding 1.052 5.481 1.036

80.14 0.915 0.72 0.99 83.724
ENSMUSG00000022898 Dscr3 protein_coding 1.147 0.2 1.005 95.504
0.868 1.973 0.952 84.29
ENSMUSG00000022899 Slc15a2 protein_coding 1.399 43.344
0.483 83.392 1.48 81.032 1.027 95.74
ENSMUSG00000022900 Ildr1 protein_coding 0.891 25.85 0.974 93.469
1.15 24.985 0.832 85.493
ENSMUSG00000022901 Cd86 protein_coding 1.431 1.188 NA NA
0.642 7.339 1.319 64.513
ENSMUSG00000022902 Stfa2 protein_coding 1 80.608 1
96.335 1 84.896 1 96.071
ENSMUSG00000022905 Kpna1 protein_coding 0.997 75.761 1.071
71.617 0.932 30.827 1.069 63.018
ENSMUSG00000022906 Parp9 protein_coding 1.256 0.2 0.961 82.947
0.831 6.858 1.029 86.445
ENSMUSG00000022911 Arl13b protein_coding 0.982 56.477 1.12
80.277 0.904 42.376 0.998 95.405
ENSMUSG00000022912 Pros1 protein_coding 1.238 0 0.902 70.29
0.894 4.389 1.097 83.581
ENSMUSG00000022913 Psmg1 protein_coding 0.959 57.014 1.005
90.29 1.039 75.527 1.04 90.463
ENSMUSG00000022914 Brwd1 protein_coding 0.972 24.817 0.936
85.242 1.098 19.025 0.978 91.105
ENSMUSG00000022915 1700093J21Rik antisense 1.292 69.489
1.038 95.708 NA NA 0.842 93.264
ENSMUSG00000022931 4930553J12Rik protein_coding 1 65.622
1 95.809 1 84.724 1 93.132
ENSMUSG00000022935 Grik1 protein_coding 1 84.977 1
93.899 1 85.921 1 96.358
ENSMUSG00000022938 Fam3b protein_coding 0.872 86.333 0.546
38.699 2.096 23.604 1.449 86.301
ENSMUSG00000022940 Pigp protein_coding 1.094 16.124 0.963
90.576 0.942 46.32 1.01 93.117
ENSMUSG00000022941 Ripply3 protein_coding 0.91 80.146
0.908 85.793 1.209 56.708 0.868 86.445
ENSMUSG00000022945 Chaf1b protein_coding 0.731 61.043
0.986 95.504 1.38 57.907 1.548 65.401
ENSMUSG00000022946 Dopey2 protein_coding 1.104 35.499
0.974 91.361 0.932 8.313 0.971 95.559
ENSMUSG00000022947 Cbr3 protein_coding 1.146 18.635 0.964
93.654 0.902 50.184 1.042 74.047
ENSMUSG00000022948 Setd4 protein_coding 0.905 38.195 0.772
68.818 1.426 16.26 0.928 87.182
ENSMUSG00000022949 Clic6 protein_coding 0.761 5.204 1.012 97.097
1.304 28.979 0.828 83.724
ENSMUSG00000022951 Rcan1 protein_coding 1.069 24.147 0.952
66.09 0.983 77.294 1.042 83.581
ENSMUSG00000022952 Runx1 protein_coding 0.862 21.957 0.998
95.196 1.162 50.375 1.008 95.559
ENSMUSG00000022956 Atp5o protein_coding 0.884 8.405 1.106 76.065
1.025 66.043 1.129 64.513
ENSMUSG00000022957 Itsn1 protein_coding 1.256 0.2 0.987 97.155
0.809 0.879 1.152 44.679
ENSMUSG00000022960 Donson protein_coding 0.966 75.858

1.041 83.104 0.991 73.598 1.007 94.174
ENSMUSG00000022961 Son protein_coding 0.974 16.325 0.951
82.947 1.081 10.819 0.991 95.559
ENSMUSG00000022962 Gart protein_coding 0.973 83.823 1.042
85.756 0.981 63.504 1.06 83.581
ENSMUSG00000022964 Tmem50b protein_coding 0.848 1.488 1.087
71.98 1.082 54.809 0.999 92.12
ENSMUSG00000022965 Ifngr2 protein_coding 1.165 0.355 0.999
96.847 0.862 0.72 1.116 43.377
ENSMUSG00000022967 Ifnar1 protein_coding 1.115 1.441 1.016
95.735 0.882 1.58 0.988 96.192
ENSMUSG00000022969 Il10rb protein_coding 1.15 0.999 1.018
92.931 0.852 0.72 0.994 96.287
ENSMUSG00000022971 Ifnar2 protein_coding 0.996 86.74 1.044
83.104 0.96 44.211 0.975 85.493
ENSMUSG00000022972 1110004E09Rik protein_coding 0.995 81.465
1.024 94.102 0.983 80.015 NA NA
ENSMUSG00000022973 Synj1 protein_coding 1.143 0 0.937 58.64
0.933 11.314 1.079 83.276
ENSMUSG00000022974 Paxbp1 protein_coding 0.876 8.122 1.019
94.48 1.121 5.964 0.956 83.724
ENSMUSG00000022978 Mis18a protein_coding 1.097 26.77 0.88
64.123 1.034 67.823 1.11 87.134
ENSMUSG00000022982 Sod1 protein_coding 0.963 27.431 1.008
90.895 1.032 63.665 0.928 83.724
ENSMUSG00000022983 Scaf4 protein_coding 0.909 5.544 1.084 72.546
1.012 57.286 1.008 95.405
ENSMUSG00000022987 Zfp641 protein_coding 0.888 26.483
0.982 91.361 1.144 26.498 0.978 94.904
ENSMUSG00000022991 Lalba protein_coding 0.222 0.987 1.724 85.756
2.617 9.176 0.52 92.31
ENSMUSG00000022992 Kansl2 protein_coding 0.99 68.846
0.982 97.155 1.024 78.864 1.018 95.273
ENSMUSG00000022993 4930415O20Rik protein_coding 0.688 53.407
NA NA 1.27 54.163 1.031 94.752
ENSMUSG00000022994 Adcy6 protein_coding 0.872 1.017 1.078 60.309
1.065 25.455 0.982 90.611
ENSMUSG00000022995 Enah protein_coding 1.155 9.435 0.991 94.597
0.875 26.057 1.171 55.485
ENSMUSG00000022996 Wnt10b protein_coding 0.882 49.368
1.266 87.029 0.897 78.155 1.134 78.948
ENSMUSG00000022997 Wnt1 protein_coding 0.836 84.819 2.192
87.282 0.543 57.446 0.979 86.991
ENSMUSG00000022999 Lmbr1l protein_coding 0.859 2.009 0.921
85.95 1.259 7.24 0.964 91.372
ENSMUSG00000023000 NA NA NA NA NA NA 0.986 86.258
0.974 95.74
ENSMUSG00000023004 Tuba1b protein_coding 0.82 3.47 1.109
60.309 1.099 56.497 1.371 43.377
ENSMUSG00000023007 Prpf40b protein_coding 0.862 2.009 0.985
96.138 1.174 6.506 0.929 83.724
ENSMUSG00000023008 Fmnl3 protein_coding 0.926 52.258 1.14
71.617 0.949 21.431 0.985 83.724
ENSMUSG00000023009 Nckap5l protein_coding 0.876 17.898

1.028 96.68 1.105 23.454 1.016 92.018
ENSMUSG00000023010 Tmbim6 protein_coding 0.972 45.894
1.022 94.48 1.009 40.052 0.996 96.086
ENSMUSG00000023011 Faim2 protein_coding 0.958 86.658 1.289
80.277 0.81 55.9 2.252 61.753
ENSMUSG00000023013 Aqp2 protein_coding 1.143 86.74 NA NA
0.737 69.974 0.735 88.552
ENSMUSG00000023015 Racgap1 protein_coding 1.168 35.024
0.927 74.483 0.927 80.163 1.25 71.305
ENSMUSG00000023017 Asic1 protein_coding 0.718 8.938 1.055 86.156
1.322 28.055 1.112 96.358
ENSMUSG00000023018 Smarcd1 protein_coding 0.951 33.906
0.962 96.216 1.096 51.596 1 95.35
ENSMUSG00000023019 Gpd1 protein_coding 0.53 0.987 1.177 61.943
1.605 7.855 1.015 96.183
ENSMUSG00000023020 Cox14 protein_coding 0.896 16.652 1.121
61.943 0.997 74.535 0.994 90.611
ENSMUSG00000023021 Cers5 protein_coding 1.002 86.199 NA NA
0.996 88.161 0.944 83.724
ENSMUSG00000023022 Lima1 protein_coding 1.093 5.932 1.009 87.609
0.908 0.72 1.041 71.855
ENSMUSG00000023025 Larp4 protein_coding 1.051 59.527 0.966
87.452 0.985 82.903 0.968 92.181
ENSMUSG00000023026 Dip2b protein_coding 1.102 35.499 0.947
83.392 0.955 33.402 0.941 83.724
ENSMUSG00000023027 Atf1 protein_coding 1.114 0.461 1.008 96.843
0.891 1.408 0.979 87.735
ENSMUSG00000023030 Slc11a2 protein_coding 1.033 46.533
1.034 83.104 0.938 14.079 0.99 86.991
ENSMUSG00000023031 Cela1 protein_coding 0.793 10.29 1.243 83.104
1.011 49.898 1.43 44.882
ENSMUSG00000023032 Slc4a8 protein_coding 0.881 68.66 0.879
52.266 1.29 28.74 1.191 83.067
ENSMUSG00000023033 Scn8a protein_coding 1.306 14.746 0.942
85.95 0.809 39.528 1.083 60.968
ENSMUSG00000023034 Nr4a1 protein_coding 1.066 65.537 NA NA
0.913 44.865 1.134 87.088
ENSMUSG00000023036 Pcdhga9 protein_coding 1.261 0.984 1.061
76.065 0.747 0.72 0.977 96.086
ENSMUSG00000023039 Krt7 protein_coding 0.909 46.289 0.899
49.266 1.219 40.511 1.211 64.513
ENSMUSG00000023041 Krt6b protein_coding 1 83.726 1
96.68 1 84.896 1 92.748
ENSMUSG00000023043 Krt18 protein_coding 1.114 82.732 0.869
52.266 1.031 56.708 1.012 95.877
ENSMUSG00000023044 Csad protein_coding 1.067 16.436 0.941
71.41 0.992 60.96 0.781 83.724
ENSMUSG00000023045 Soat2 protein_coding 0.893 73.292 2.014
69.228 0.556 10.003 1.012 94.215
ENSMUSG00000023046 Igfbp6 protein_coding 1.139 25.421
0.854 94.48 1.027 83.158 1.404 49.514
ENSMUSG00000023047 Amhr2 protein_coding 0.799 27.968 0.979
92.561 1.273 47.935 0.923 94.99
ENSMUSG00000023048 Prr13 protein_coding 1.001 78.428 0.942

87.313 1.059 62.648 0.997 94.99
ENSMUSG00000023050 Map3k12 protein_coding 0.89 9.863 1.113
76.634 1.01 56.931 1 95.72
ENSMUSG00000023051 Tarbp2 protein_coding 0.818 0.987 1.129
62.898 1.079 16.666 1.038 94.384
ENSMUSG00000023052 Npff protein_coding 1.044 70.862 0.863
75.993 1.109 37.741 0.936 87.992
ENSMUSG00000023055 Calcoco1 protein_coding 0.824 1.15 0.914
82.073 1.323 5.864 0.97 84.29
ENSMUSG00000023064 Sncg protein_coding 0.856 54.983 1.023
90.116 1.14 57.637 1.185 76.831
ENSMUSG00000023066 Rttn protein_coding 1.021 74.482 0.984
93.654 0.996 79.776 1.059 93.156
ENSMUSG00000023067 Cdkn1a protein_coding 0.9 79.709
0.955 75.4 1.164 38.26 1.441 31.133
ENSMUSG00000023068 Nus1 protein_coding 1.028 23.254 1.006
90.053 0.965 47.641 0.957 84.19
ENSMUSG00000023070 Rgn protein_coding 0.544 56.768 1.145
96.68 1.607 77.17 0.853 93.132
ENSMUSG00000023072 Cep89 protein_coding 0.938 8.122 0.91 59.946
1.175 5.864 1.024 75.198
ENSMUSG00000023073 Slc10a2 protein_coding 1 74.958 1
95.088 1 87.686 1 95.877
ENSMUSG00000023074 Mospd1 protein_coding 1.031 78.428
1.103 89.434 0.881 16.129 0.965 90.95
ENSMUSG00000023075 Akirin1 protein_coding 1.048 27.113
1.065 71.617 0.894 0.72 1.032 83.067
ENSMUSG00000023078 Cxcl13 protein_coding 1.419 46.097
0.569 74.851 1.236 83.857 1.138 93.457
ENSMUSG00000023079 Gtf2ird1 protein_coding 1.258 41.951
1.015 95.735 0.782 9.347 0.788 83.724
ENSMUSG00000023083 H2-M10.2 protein_coding 1 82.732 1
96.407 1 84.626 1 94.174
ENSMUSG00000023084 Lrrc71 protein_coding 0.726 3.895 1.026
92.561 1.344 13.082 0.921 89.525
ENSMUSG00000023087 Ccrn4l protein_coding 1.127 29.536
1.025 96.216 0.867 19.765 1.098 93.964
ENSMUSG00000023088 Abcc1 protein_coding 1.052 3.393 1.088 83.104
0.875 0.72 1.02 94.752
ENSMUSG00000023089 Ndufa5 protein_coding 0.879 5.946 1.004
92.561 1.133 32.776 1.007 81.061
ENSMUSG00000023092 Fhl1 protein_coding 0.817 4.468 0.966 86.538
1.268 5.864 1.231 49.514
ENSMUSG00000023093 Gm7257 protein_coding 1 60.853 1
95.196 1 62.791 1 96.183
ENSMUSG00000023094 Msrb2 protein_coding 0.945 74.689 1.038
90.29 1.019 87.173 0.994 87.992
ENSMUSG00000023104 NA NA NA NA 1.026 95.809 1.011
86.552 1.125 60.466
ENSMUSG00000023106 Denr protein_coding 0.95 39.953 0.984
91.661 1.07 35.673 1.075 46.846
ENSMUSG00000023110 Prmt5 protein_coding 0.962 36.945 1.051
79.261 0.984 85.675 1.053 59.464
ENSMUSG00000023118 Sympk protein_coding 0.937 14.221 1.006

95.196 1.063 28.997 1.047 78.379
ENSMUSG00000023122 Sult1c2 protein_coding 1 85.129 1
95.211 1 84.434 1 94.825
ENSMUSG00000023132 Gzma protein_coding 0.98 68.66 1.403 71.617
0.726 18.797 0.704 84.29
ENSMUSG00000023140 Reg2 protein_coding 1 78.299 1
96.68 1 87.274 1 92.748
ENSMUSG00000023143 Nagpa protein_coding 0.973 60.339 1.027
96.897 1.003 83.925 1.056 90.865
ENSMUSG00000023147 Wrb protein_coding 1.067 7.908 1.041 91.776
0.897 12.907 1.025 95.877
ENSMUSG00000023150 Ivns1abp protein_coding 1.16 16.124
0.885 68.115 0.976 87.686 1.06 92.263
ENSMUSG00000023151 Lrrc69 protein_coding 0.452 30.136 NA
NA 2.664 49.384 0.881 92.12
ENSMUSG00000023153 Tmem52 protein_coding 0.881 85.092 1.76
82.103 0.647 51.443 1.132 83.067
ENSMUSG00000023156 Rpp14 protein_coding 0.993 86.843 1.032
95.735 0.973 80.163 1.007 92.762
ENSMUSG00000023159 Psg29 protein_coding 1 79.709 NA NA
1 83.066 1 93.584
ENSMUSG00000023165 Ssxb2 protein_coding 1 80.251 NA NA
1 88.657 1 96.086
ENSMUSG00000023169 Slc38a1 protein_coding 0.908 23.556
0.904 66.651 1.216 6.506 0.976 96.287
ENSMUSG00000023170 Gps2 protein_coding 0.962 24.344 0.96
95.504 1.083 38.683 1.041 86.445
ENSMUSG00000023175 Bsg protein_coding 1.038 66.58 1.002 95.941
0.958 78.713 1.012 93.627
ENSMUSG00000023176 Cpn2 protein_coding 0.797 86.819 1.003
96.216 1.249 84.896 0.935 95.273
ENSMUSG00000023185 Ceacam14 protein_coding 1 85.391 NA
NA 1 87.173 1 96.358
ENSMUSG00000023186 Vwa5a protein_coding 1.049 7.049 1.029 95.431
0.925 5.323 1.047 71.855
ENSMUSG00000023191 Leprel2 protein_coding 1.107 11.638 NA
NA 0.949 32.082 1.109 42.736
ENSMUSG00000023192 Grm2 protein_coding 1 54.87 NA NA 1
79.625 1 92.762
ENSMUSG00000023206 Il15ra protein_coding 1.188 21.703
0.911 90.576 0.92 36.499 1.061 66.944
ENSMUSG00000023210 Lcn9 protein_coding 1 84.819 1
97.155 1 86.475 1 94.904
ENSMUSG00000023224 Serping1 protein_coding 1.193 12.855
0.996 96.216 0.839 24.523 0.916 93.261
ENSMUSG00000023232 Serinc2 protein_coding 0.9 54.176
0.843 78.471 1.32 38.975 0.86 86.699
ENSMUSG00000023235 Ccl25 protein_coding 0.859 42.034 1.103
87.609 1.055 76.923 1.073 94.904
ENSMUSG00000023236 Scg5 protein_coding 1.314 76.546 0.838
95.504 0.905 83.607 1.476 80.77
ENSMUSG00000023243 Kcnk5 protein_coding 1.115 84.865 1.04
82.904 0.863 42.376 0.835 85.493
ENSMUSG00000023249 Parp3 protein_coding 0.959 48.036 1.145

71.98 0.912 55.701 1.177 67.691
ENSMUSG00000023257 Cypt1 protein_coding 1 81.76 1 96.652
1 88.657 1 96.287
ENSMUSG00000023259 Slc26a6 protein_coding 1.178 35.499
1.116 79.864 0.758 0.72 1.107 48.177
ENSMUSG00000023262 Acy1 protein_coding 0.938 45.229 0.958
82.671 1.113 29.819 1.111 83.067
ENSMUSG00000023263 9530002B09Rik protein_coding 1 86.006
NA NA 1 88.657 1 95.676
ENSMUSG00000023266 Frs3 protein_coding 0.832 18.907 1.027
94.848 1.166 29.568 0.944 83.724
ENSMUSG00000023267 Gabrr2 protein_coding 0.695 17.062
1.212 94.367 1.18 40.052 1.003 95.273
ENSMUSG00000023272 Creld2 protein_coding 0.936 42.922
1.019 92.561 1.044 61.743 1.109 48.119
ENSMUSG00000023274 Cd4 protein_coding 1.438 61.725 0.962
95.809 0.724 25.345 0.711 83.724
ENSMUSG00000023277 Twf2 protein_coding 1.038 63.126 1.038
90.952 0.93 29.451 1.042 85.577
ENSMUSG00000023279 Bmp15 protein_coding 0.501 13.74 1.31 82.904
1.518 68.129 1.352 88.797
ENSMUSG00000023284 Zfp605 protein_coding 1.03 71.06 1.088
96.843 0.895 57.091 0.888 84.19
ENSMUSG00000023286 Ube2j2 protein_coding 0.985 72.139
1.031 82.904 0.986 62.058 0.97 95.72
ENSMUSG00000023289 Sva protein_coding 1 68.485 1
95.504 NA NA 1 95.273
ENSMUSG00000023307 5-Mar protein_coding 1.014 86.464 NA NA
0.998 87.173 1.072 87.7
ENSMUSG00000023328 Ache protein_coding 0.699 28.242 0.805
74.483 1.77 21.462 0.744 84.19
ENSMUSG00000023330 Dtwd1 protein_coding 1.041 75.399 1.085
82.904 0.886 32.297 1.005 86.133
ENSMUSG00000023333 Gcm1 protein_coding 1 74.021 1
95.35 1 86.144 1 95.559
ENSMUSG00000023336 Wfdc1 protein_coding 1.155 42.134 1.144
85.756 0.757 13.848 0.968 90.397
ENSMUSG00000023341 Mx2 protein_coding 1.204 12.855 0.989
96.847 0.84 17.662 1.182 61.316
ENSMUSG00000023345 Poc1a protein_coding 1.027 84.564 0.95
96.268 1.025 86.552 1.092 79.442
ENSMUSG00000023348 Trip6 protein_coding 0.888 7.207 1.089 79.411
1.033 37.259 0.999 87.447
ENSMUSG00000023349 Clec4n protein_coding 1.708 11.928
0.975 95.088 0.6 20.511 1.378 78.042
ENSMUSG00000023350 4921501E09Rik protein_coding 1 84.397
1 96.843 1 82.575 1 95.777
ENSMUSG00000023353 Agap3 protein_coding 0.966 11.726 1.011
92.253 1.026 45.94 0.976 96.248
ENSMUSG00000023367 Tmem176a protein_coding 1.104 24.253
0.958 83.733 0.946 45.093 1.164 90.455
ENSMUSG00000023387 Kcnk16 protein_coding 0.784 85.655
0.404 52.266 3.144 19.286 0.83 92.742
ENSMUSG00000023393 Slc17a9 protein_coding 1.102 29.596

0.844 66.09 1.078 88.461 1.144 64.513
ENSMUSG00000023411 Nfatc4 protein_coding 1.05 54.717
1.047 84.007 0.912 32.703 1.094 79.268
ENSMUSG00000023433 Cela3b protein_coding 1 82.982 1
95.504 NA NA 1 96.287
ENSMUSG00000023439 Gnb3 protein_coding 0.877 54.87 0.782 95.708
1.451 38.975 1.102 91.872
ENSMUSG00000023443 Esx1 protein_coding 1 83.968 1
94.96 1 81.388 1 91.625
ENSMUSG00000023452 Pisd protein_coding 1.03 55.124 1.03
83.104 0.942 11.902 1.016 82.328
ENSMUSG00000023456 Tpi1 protein_coding 0.839 0.987 1.115 71.617
1.065 39.471 1.125 31.133
ENSMUSG00000023460 Rab12 protein_coding 1.117 0 0.964 74.483
0.926 0.879 1.002 90.611
ENSMUSG00000023467 Tulp2 protein_coding 0.901 52.258 0.756
63.912 1.471 11.518 0.701 83.724
ENSMUSG00000023473 Celsr3 protein_coding 1.019 84.534 NA
NA 1.091 72.148 1.294 83.067
ENSMUSG00000023484 Prph protein_coding 1.32 75.671 0.611
85.116 1.241 57.286 0.576 87.218
ENSMUSG00000023495 Pcbp4 protein_coding 0.989 50.768 1.092
71.98 0.927 30.827 1.003 93.132
ENSMUSG00000023505 Cdca3 protein_coding 1.342 15.972 0.794
69.611 0.941 71.999 1.121 83.067
ENSMUSG00000023571 Fam132a protein_coding 0.853 6.677 1.075
92.561 1.087 37.369 0.974 90.611
ENSMUSG00000023572 Ccndbp1 protein_coding 0.991 67.477 NA
NA 1.004 78.498 0.925 84.29
ENSMUSG00000023577 Iqcf3 protein_coding 1 72.655 1
93.046 1 84.961 1 96.358
ENSMUSG00000023707 Ogfod2 protein_coding 0.821 1.031 1.002
95.196 1.217 6.202 1.037 92.018
ENSMUSG00000023723 Mrps23 protein_coding 1.007 78.538
1.003 96.843 0.991 87.898 1.023 82.091
ENSMUSG00000023755 Rhebl1 protein_coding 0.914 15.227
0.908 93.654 1.201 27.047 0.816 85.493
ENSMUSG00000023764 Sfi1 protein_coding 0.881 2.174 0.946 83.58
1.201 6.506 0.984 96.248
ENSMUSG00000023781 Hes7 protein_coding 1.106 79.277 0.844
91.83 1.071 84.724 1.185 89.108
ENSMUSG00000023791 Pigx protein_coding 1.119 5.932 0.886 28.832
NA NA 1.004 94.82
ENSMUSG00000023795 Pisd-ps2 pseudogene 0.899 30.035 1.113
83.104 0.999 83.857 0.976 86.699
ENSMUSG00000023800 Tiam2 protein_coding 1.046 64.401 1.151
79.411 0.829 12.41 0.863 83.724
ENSMUSG00000023802 Nox3 protein_coding 1 69.943 1
95.504 1 88.076 1 93.584
ENSMUSG00000023805 Synj2 protein_coding 1.043 72.85 0.921 79.475
1.04 69.002 0.957 92.181
ENSMUSG00000023806 Rsph3b protein_coding 0.863 4.055 1.103
83.104 1.053 32.173 0.958 84.29
ENSMUSG00000023809 Rps6ka2 protein_coding 1.095 36.686

1.059 87.282 0.86 8.142 0.974 95.273
ENSMUSG00000023826 Park2 protein_coding 1.244 29.372 1.009
97.155 0.798 34.592 0.955 88.505
ENSMUSG00000023827 Agpat4 protein_coding 1.128 7.65 0.989
85.242 0.893 40.794 1.085 42.736
ENSMUSG00000023828 Slc22a3 protein_coding 1.072 45.307
1.169 75.62 0.798 5.564 1.061 89.108
ENSMUSG00000023829 Slc22a1 protein_coding 1.408 31.24 0.525
67.941 1.354 73.759 1.079 95.273
ENSMUSG00000023830 Igf2r protein_coding 1.102 3.393 0.998 94.96
0.911 6.858 0.904 83.724
ENSMUSG00000023832 Acat2 protein_coding 0.837 3.763 1.097 72.546
1.085 31.761 1.031 90.674
ENSMUSG00000023845 Lnpep protein_coding 0.709 3.559 0.964 96.68
1.456 6.449 1.001 90.628
ENSMUSG00000023846 Riok2 protein_coding 0.811 24.817 0.975
90.053 1.264 12.35 1.17 43.377
ENSMUSG00000023852 Chd1 protein_coding 1.039 45.307 0.953
86.604 1.01 87.686 1.022 96.183
ENSMUSG00000023861 Mpc1 protein_coding 0.638 0.987 1.055 82.904
1.487 10.76 1.036 96.358
ENSMUSG00000023868 Pde10a protein_coding 1.145 12.549
0.877 68.818 0.993 83.811 1.138 55.841
ENSMUSG00000023873 1700010I14Rik protein_coding 1.055 80.751
0.832 91.83 1.14 64.024 0.783 84.29
ENSMUSG00000023882 Zfp54 protein_coding 1.068 45.307 0.986
95.088 0.949 51.443 1.039 94.215
ENSMUSG00000023883 NA NA NA NA 0.991 87.857 1.013
64.624 1.011 93.132
ENSMUSG00000023885 Thbs2 protein_coding 1.382 1.828 0.704 37.376
1.025 86.552 1.363 60.466
ENSMUSG00000023886 Smoc2 protein_coding 1.014 85.391 1.157
67.843 0.85 50.523 0.907 96.183
ENSMUSG00000023892 Zfp51 protein_coding 1.022 56.907 1.004
84.594 NA NA 1.108 83.067
ENSMUSG00000023903 Mmp25 protein_coding 0.973 69.985 1.136
96.897 NA NA 0.977 90.805
ENSMUSG00000023904 Hcfc1r1 protein_coding 0.861 2.009 1.118
60.309 1.041 80.015 1.006 91.525
ENSMUSG00000023905 Tnfrsf12a protein_coding 0.82 59.028
1.272 66.695 0.954 88.076 1.231 60.466
ENSMUSG00000023906 Cldn6 protein_coding 0.774 66.897 NA NA
1.238 80.899 0.941 92.181
ENSMUSG00000023908 Pkmyt1 protein_coding 0.834 11.924
1.159 74.663 1.033 60.104 1.085 55.841
ENSMUSG00000023909 Paqr4 protein_coding 0.828 1.623 1.275 40.99
0.949 77.037 1.016 90.463
ENSMUSG00000023911 Flywch2 protein_coding 0.97 78.639
0.871 73.461 1.183 37.369 0.936 86.699
ENSMUSG00000023912 Slc25a27 protein_coding 0.836 9.508 0.832
68.818 1.432 10.079 0.826 91.078
ENSMUSG00000023913 Pla2g7 protein_coding 1.23 13.363
0.826 49.859 0.986 77.037 1.029 94.99
ENSMUSG00000023915 Tnfrsf21 protein_coding 1.201 16.249

0.902 75.345	0.923 76.64 1.012 95.273
ENSMUSG00000023918	Gpr115	protein_coding	0.828 62.888
1.011 93.574	1.191 84.896	0.649 83.724
ENSMUSG00000023919	Cenpq protein_coding	0.794 9.508 1.222 83.104
1.027 57.286	1.091 86.445
ENSMUSG00000023921	Mut	protein_coding	0.891 44.787	0.998
93.899	1.124 82.436	1.042 92.12
ENSMUSG00000023923	Tbc1d5	protein_coding	1.103 7.255 0.95
71.41 0.955 23.86 0.982 96.183
ENSMUSG00000023926	Rhag	protein_coding	1	58.264	NA	NA
1	64.024	1	93.627
ENSMUSG00000023927	Satb1 protein_coding	1.162 1.43	1.015 92.062
0.848 0.945 0.892 83.724
ENSMUSG00000023931	Efhb	protein_coding	0.976 75.298	1.126
91.776	0.911 88.601	0.87	86.699
ENSMUSG00000023932	Cdc5l protein_coding	1.092 17.492	0.967
89.713	0.949 36.499	1.013 83.276
ENSMUSG00000023938	Aars2 protein_coding	0.831 1.017 1.092 85.756
1.105 10.76 1.012 96.287
ENSMUSG00000023939	Mrpl14	protein_coding	0.945 34.572
1.014 96.763	1.045 44.361	0.957 84.29
ENSMUSG00000023940	Sgol1 protein_coding	1.415 23.82 0.578 50.715
1.225 81.29 1.257 74.193
ENSMUSG00000023942	Slc29a1	protein_coding	0.947 44.562
0.986 88.345	1.071 38.152	1.067 91.928
ENSMUSG00000023943	Sult1c1	protein_coding	1	86.145	1
90.389	1	62.791	1	91.979
ENSMUSG00000023944	Hsp90ab1	protein_coding	0.886 21.658
1.027 86.728	1.096 10.281	1.069 31.133
ENSMUSG00000023947	Nfkbie	protein_coding	0.942 65.199
1.313 60.309	0.806 3.718 0.862 83.724
ENSMUSG00000023951	Vegfa protein_coding	0.869 27.836	0.924
73.766	1.241 15.602	0.937 83.724
ENSMUSG00000023952	Gtpbp2	protein_coding	0.871 0.987 1.048
79.411	1.091 10.819	0.987 85.493
ENSMUSG00000023953	Polh	protein_coding	1.11	5.313 1.039 95.809
0.867 6.858 1.046 91.872
ENSMUSG00000023959	Clic5 protein_coding	1.02	71.516	1.155
71.617	0.85	4.935 0.966 92.748
ENSMUSG00000023960	Enpp5 protein_coding	1.023 78.639	0.926
87.322	1.053 64.024	0.956 83.724
ENSMUSG00000023961	Enpp4 protein_coding	1.061 34.439	1.067
93.529	0.885 39.528	0.924 83.952
ENSMUSG00000023963	Cyp39a1	protein_coding	1.083 45.307
0.979 90.046	0.94	66.425	0.882 92.181
ENSMUSG00000023965	Fbxl17	protein_coding	1.001 75.196
1.017 94.89 0.982 43.842	0.977 93.685
ENSMUSG00000023966	Rsph9 protein_coding	1.316 1.161 0.842 79.475
0.901 16.719	0.991 90.289
ENSMUSG00000023967	Mrps18a	protein_coding	0.977 42.361
1.031 86.34 0.995 84.552	1.027 93.964
ENSMUSG00000023968	Crip3 protein_coding	0.947 60.339	1.164
83.104	0.905 86.475	1.095 79.268
ENSMUSG00000023971	Rrp36 protein_coding	0.901 17.401	1.009

93.863	1.097	43.586	0.988	92.12
ENSMUSG00000023972	Ptk7	protein_coding	0.937	31.333	0.892
82.947	1.199	19.106	0.893	83.724
ENSMUSG00000023973	Cnpy3	protein_coding	0.934	11.924	1.015
95.196	1.055	50.636	1.065	56.863
ENSMUSG00000023977	Ubr2	protein_coding	0.923	18.731	0.965
81.443	1.124	8.007	1.05	83.067
ENSMUSG00000023978	Prph2	protein_coding	1	83.263	1
93.654	1	60.789	1	95.877
ENSMUSG00000023980	Taf8	protein_coding	0.945	18.907	0.928
68.848	1.139	7.635	1.018	93.457
ENSMUSG00000023982	Guca1a	protein_coding	1.109	13.633
0.886	87.857	1.013	70.214	1.397	72.369
ENSMUSG00000023984	Gm20517	protein_coding	1.167	59.806
0.958	95.301	0.895	68.266	0.832	91.65
ENSMUSG00000023987	Pgc	protein_coding	1	80.751	1
95.504	1	57.286	1	95.74
ENSMUSG00000023988	Bysl	protein_coding	0.854	8.365	1.115	71.98
1.049	71.839	1.043	83.067
ENSMUSG00000023990	Tfeb	protein_coding	0.913	15.06	1.156	88.823
0.945	69.315	1.032	67.94
ENSMUSG00000023991	Foxp4	protein_coding	0.844	12.084	1.057
92.793	1.122	32.524	0.918	83.724
ENSMUSG00000023992	Trem2	protein_coding	1.333	8.102	0.872	87.553
0.862	37.103	1.505	0
ENSMUSG00000023993	Treml1	protein_coding	0.694	10.29	1.015
90.256	1.419	31.761	1.133	91.928
ENSMUSG00000023994	Nfya	protein_coding	1.075	15.662	1.027
87.282	0.905	14.409	1.021	96.287
ENSMUSG00000023995	Tspo2	protein_coding	0.782	76.135	1.29
95.735	0.992	83.811	1.229	94.904
ENSMUSG00000023999	Kif6	protein_coding	0.678	13.062	1.307
82.809	1.129	84.896	0.947	96.183
ENSMUSG00000024002	Brd4	protein_coding	0.991	78.054	1.065
72.642	0.949	14.262	0.994	87.735
ENSMUSG00000024006	Stk38	protein_coding	0.969	47.266	0.942
82.503	1.095	15.411	1.054	50.74
ENSMUSG00000024007	Ppil1	protein_coding	0.987	81.125	0.942
92.914	1.071	84.724	1.127	60.466
ENSMUSG00000024008	Cpne5	protein_coding	1.242	59.806	0.783
96.001	1.029	88.076	1.269	94.904
ENSMUSG00000024011	Pi16	protein_coding	0.877	14.526	0.889
87.553	1.276	29.777	1.025	78.546
ENSMUSG00000024012	Mtch1	protein_coding	0.895	6.221	1.015	92.062
1.105	19.609	1.013	93.156
ENSMUSG00000024013	Fgd2	protein_coding	1.361	0.461	0.978	81.731
0.749	3.718	1.005	90.455
ENSMUSG00000024014	Pim1	protein_coding	0.833	18.487	1.009
96.308	1.193	39.471	0.951	94.654
ENSMUSG00000024018	Ccdc167	protein_coding	0.861	3.07	1.066
83.104	1.086	23.41	0.99	90.157
ENSMUSG00000024019	Cmtr1	protein_coding	0.867	5.962	1.071	82.809
1.079	41.235	0.976	94.99
ENSMUSG00000024026	Glo1	protein_coding	0.984	45.894	0.958

93.908	1.059	38.099	1.015	92.762
ENSMUSG00000024028	Tff2	protein_coding	1	84.049	1
94.96	1	84.724	1	93.964
ENSMUSG00000024029	Tff3	protein_coding	1	81.01	1	94.597
1	80.015	1	94.567
ENSMUSG00000024030	Abcg1	protein_coding	1.362	0	0.815	0	0.9
19.236	1.071	94.19
ENSMUSG00000024032	Tff1	protein_coding	1	79.433	1
93.707	1	84.626	1	92.018
ENSMUSG00000024033	Rsph1	protein_coding	0.888	58.38	1.233	97.155
0.916	60.96	1.091	94.016
ENSMUSG00000024036	Slc37a1	protein_coding	0.912	61.633
0.932	70.864	1.178	19.877	0.874	83.724
ENSMUSG00000024037	Wdr4	protein_coding	0.994	69.24	NA	NA
1.039	64.024	0.961	85.493
ENSMUSG00000024038	Ndufv3	protein_coding	0.966	55.939
1.057	90.29	0.979	86.727	0.944	83.724
ENSMUSG00000024039	Cbs	protein_coding	1.397	0	0.949	78.471
0.751	0.72	0.802	83.724
ENSMUSG00000024041	Cryaa	protein_coding	1	85.728	1
95.504	1	88.461	1	94.153
ENSMUSG00000024042	Sik1	protein_coding	1.055	55.789	0.958
85.116	0.986	82.74	0.816	83.724
ENSMUSG00000024043	Arhgap28	protein_coding	1.273	0.577	0.919
89.277	0.851	33.689	1.117	94.904
ENSMUSG00000024044	Epb4.1l3	protein_coding	1.206	15.85	1.056
96.216	0.782	15.63	1.036	83.581
ENSMUSG00000024045	Akap8	protein_coding	0.999	86.333	1.015
97.155	0.984	84.669	0.987	93.612
ENSMUSG00000024048	Myl12a	protein_coding	0.89	10.29	1.065
82.904	1.057	43.842	1.114	63.708
ENSMUSG00000024049	Myom1	protein_coding	2.412	9.206	1.213	91.776
0.341	0.72	0.962	96.071
ENSMUSG00000024050	Wiz	protein_coding	0.975	48.354	1.072
85.378	0.96	78.954	0.965	83.724
ENSMUSG00000024052	Lpin2	protein_coding	1.194	17.582	0.838
37.426	1	83.925	1.042	86.133
ENSMUSG00000024053	Emilin2	protein_coding	1.136	7.049	0.963
90.378	0.911	46.071	1.166	37.634
ENSMUSG00000024054	Smchd1	protein_coding	1.071	28.098
0.923	52.085	1.011	70.328	0.986	96.287
ENSMUSG00000024055	Cyp4f13	protein_coding	0.884	0.987	0.964
79.807	1.175	5.864	0.939	83.724
ENSMUSG00000024056	Ndc80	protein_coding	1.771	15.602	0.714
28.822	0.793	88.693	1.685	69.404
ENSMUSG00000024059	Clip4	protein_coding	1.069	42.134	0.949
89.713	NA	NA	0.895	91.372
ENSMUSG00000024063	Lbh	protein_coding	1.372	0.577	0.962	94.48
0.755	1.342	0.954	92.181
ENSMUSG00000024064	Galnt14	protein_coding	2.034	13.8	0.437
55.71	1.118	79.554	1.74	68.696
ENSMUSG00000024065	Ehd3	protein_coding	1.071	62.574	1.094
83.104	0.855	16.719	1.009	96.287
ENSMUSG00000024066	Xdh	protein_coding	0.959	49.944	0.875

60.678	1.194	8.339	1.007	79.268
ENSMUSG00000024067	Dpy30	protein_coding	1.022	86.006	0.959
97.263	1.022	88.693	1.017	72.826
ENSMUSG00000024068	Spast	protein_coding	1.09	10.105	0.984
90.053	0.928	25.688	0.989	93.734
ENSMUSG00000024069	Slc30a6	protein_coding	0.965	39.712
1.009	84.768	1.029	71.839	0.995	96.287
ENSMUSG00000024070	Prkd3	protein_coding	1.088	0.577	0.977	92.404
0.937	6.858	1.004	95.74
ENSMUSG00000024072	Yipf4	protein_coding	1.118	0	0.97	63.912
0.921	6.515	0.975	87.651
ENSMUSG00000024073	Birc6	protein_coding	0.973	13.883	0.968
96.335	1.061	39.471	0.987	92.181
ENSMUSG00000024074	Crim1	protein_coding	1.049	66.58	0.96	85.793
0.995	87.898	0.991	92.675
ENSMUSG00000024076	Vit	protein_coding	1.306	0	0.876	65.165
0.873	11.314	0.997	93.316
ENSMUSG00000024077	Strn	protein_coding	0.969	37.531	0.98
84.594	1.049	18.797	1.002	93.457
ENSMUSG00000024078	Ttc27	protein_coding	1.032	74.482	0.98
95.504	0.986	87.786	1.039	66.068
ENSMUSG00000024079	Eif2ak2	protein_coding	1.093	51.826	NA
NA	0.934	60.233	1.124	53.923
ENSMUSG00000024081	Cebpz	protein_coding	1.058	39.295	0.949
74.851	0.998	87.744	1.014	87.134
ENSMUSG00000024082	Ndufaf7	protein_coding	1.009	82.982
1.049	92.561	0.946	67.029	1.023	78.042
ENSMUSG00000024083	Pja2	protein_coding	1.098	3.152	0.98	95.211
0.925	6.858	1.044	79.268
ENSMUSG00000024084	Qpct	protein_coding	1.319	0	0.956	89.996
0.795	15.778	0.989	95.877
ENSMUSG00000024085	Man2a1	protein_coding	0.98	67.477
0.976	90.053	1.045	44.125	1.02	76.046
ENSMUSG00000024087	Cyp1b1	protein_coding	1.072	58.38	0.842
37.426	1.107	38.683	1.012	96.183
ENSMUSG00000024088	4930583I09Rik	protein_coding	1	85.77	1
95.708	1	88.41	1	92.31
ENSMUSG00000024091	Vapa	protein_coding	1.226	0	NA	NA
0.817	0.72	0.988	94.904
ENSMUSG00000024095	Hnrnpll	protein_coding	1.019	71.06	NA	NA
1.002	73.096	0.99	86.254
ENSMUSG00000024096	Ralbp1	protein_coding	1.017	23.493	NA
NA	0.987	62.533	0.966	83.724
ENSMUSG00000024097	Srsf7	protein_coding	1.106	19.132	0.953
82.671	0.947	74.853	0.998	95.273
ENSMUSG00000024098	Twsg1	protein_coding	1.098	0.355	1.035	96.138
0.88	3.943	0.982	86.699
ENSMUSG00000024099	Ndufv2	protein_coding	0.966	44.562
1.081	71.98	0.955	59.922	1.038	83.581
ENSMUSG00000024101	Wash	protein_coding	0.994	85.59	1	90.378
1.009	78.369	1.017	93.685
ENSMUSG00000024104	Fam21	protein_coding	0.994	84.865	0.96
90.378	1.045	62.648	1.026	61.753
ENSMUSG00000024105	Themis3	protein_coding	0.977	74.745

1.259 95.088 0.81 86.552 0.707 83.724
ENSMUSG00000024107 Lhcgr protein_coding 0.868 66.406 NA NA
1.232 83.538 1.366 80.187
ENSMUSG00000024109 Nrxn1 protein_coding 1.08 85.129 0.946
93.908 0.977 80.089 0.825 92.181
ENSMUSG00000024112 Cacna1h protein_coding 1.113 32.676
0.904 86.277 0.994 85.675 0.968 86.991
ENSMUSG00000024114 Prss41 protein_coding 0.634 86.843 NA
NA 1.762 79.554 1.383 94.904
ENSMUSG00000024116 Prss21 protein_coding 1 85.59 1
97.263 1 84.724 1 93.964
ENSMUSG00000024118 1600002H07Rik protein_coding 0.914 34.162
1.124 79.411 0.971 70.214 0.995 96.192
ENSMUSG00000024120 Lrpprc protein_coding 1.005 82.543 NA
NA 0.991 84.504 1.005 73.401
ENSMUSG00000024121 Atp6v0c protein_coding 1.006 76.319
1.048 82.76 0.95 39.245 1.083 70.36
ENSMUSG00000024122 Pdpk1 protein_coding 0.997 84.294 1.007
96.138 1 84.071 0.955 83.724
ENSMUSG00000024124 Prss30 protein_coding 1.24 68.485
0.789 77.598 1.02 71.232 0.9 96.287
ENSMUSG00000024125 Sbpl protein_coding 1 83.09 1 90.053
1 88.41 1 96.183
ENSMUSG00000024127 Prepl protein_coding 1.023 75.671 0.988
88.705 0.988 81.29 0.963 84.29
ENSMUSG00000024128 Sbp protein_coding 0.339 5.946 1.045 96.847
2.817 28.997 0.504 85.493
ENSMUSG00000024130 Abca3 protein_coding 1.057 14.205 0.968
79.475 0.975 48.577 0.901 83.724
ENSMUSG00000024131 Slc3a1 protein_coding 0.776 4.825 1.107
87.604 1.163 27.431 0.986 95.405
ENSMUSG00000024132 Eci1 protein_coding 1.027 42.255 1.006
93.27 0.964 39.079 0.906 83.724
ENSMUSG00000024134 Six2 protein_coding 3.793 42.255 1.055
92.931 0.25 23.212 0.729 96.183
ENSMUSG00000024135 Srbd1 protein_coding 0.92 20.116 1.002
96.948 1.082 32.524 1.041 80.324
ENSMUSG00000024136 Dnase1l2 protein_coding 0.939 49.749
1.102 80.806 0.967 68.584 1 94.904
ENSMUSG00000024137 E4f1 protein_coding 0.847 1.488 1.056 96.897
1.121 7.635 0.967 86.699
ENSMUSG00000024140 Epas1 protein_coding 1.017 82.092 NA NA
1.034 82.161 0.995 92.181
ENSMUSG00000024142 Mlst8 protein_coding 0.953 25.3 1.017 91.643
1.034 50.636 1.032 86.445
ENSMUSG00000024143 Rhoq protein_coding 1.073 7.501 1.138 64.304
0.82 0.72 0.979 94.608
ENSMUSG00000024145 Pigf protein_coding 0.903 33.906 1.048
95.809 1.054 75.527 1.036 88.197
ENSMUSG00000024146 Cript protein_coding 0.988 77.113 1.032
83.104 0.983 66.201 1.023 88.755
ENSMUSG00000024150 Mcfd2 protein_coding 1.014 78.639 0.982
95.35 1.002 88.601 1.052 46.846
ENSMUSG00000024151 Msh2 protein_coding 1 82.092 0.919

69.611 1.087 40.955 1.059 68.696
ENSMUSG00000024155 Meiob protein_coding 1.483 2.784 0.807 51.277
0.833 75.967 0.841 90.805
ENSMUSG00000024158 Hagh protein_coding 0.95 13.581 1.08
71.617 0.975 86.112 0.96 87.735
ENSMUSG00000024160 Spsb3 protein_coding 0.934 37.82 1.047 84.832
1.026 47.776 0.966 86.991
ENSMUSG00000024163 Mapk8ip3 protein_coding 0.917 2.009 0.988
90.712 1.101 6.506 0.984 86.699
ENSMUSG00000024164 C3 protein_coding 1.084 46.875 0.979
94.48 0.939 72.464 0.905 88.505
ENSMUSG00000024165 Hn1l protein_coding 1.005 65.733 1.097
82.904 0.909 27.321 1.202 32.698
ENSMUSG00000024168 Tmem204 protein_coding 0.983 83.263
1.018 97.263 1.001 87.744 0.9 83.724
ENSMUSG00000024169 Ift140 protein_coding 0.941 11.769
1.039 97.049 1.025 35.673 1.04 88.197
ENSMUSG00000024170 Telo2 protein_coding 0.983 82.479 0.959
94.887 1.06 69.315 1.019 96.248
ENSMUSG00000024171 Prss28 protein_coding 1 72.743 1
97.155 1 77.888 1 95.273
ENSMUSG00000024172 St6gal2 protein_coding 3.327 33.099
0.481 91.661 0.624 84.434 0.676 96.192
ENSMUSG00000024173 Tpsab1 protein_coding 1.886 23.493
0.789 68.115 0.673 85.134 0.882 96.192
ENSMUSG00000024174 Pot1b protein_coding 1.069 60.168 0.945
97.155 0.988 67.029 0.982 92.675
ENSMUSG00000024175 Tekt4 protein_coding 1 86.464 1
87.322 1 83.538 1 92.748
ENSMUSG00000024176 Sox8 protein_coding 1.193 52.429 1.333
83.104 0.627 15.778 1.209 67.94
ENSMUSG00000024177 Nme4 protein_coding 1.069 39.075 NA NA
0.905 32.703 1.011 95.559
ENSMUSG00000024180 Tmem8 protein_coding 1.179 8.477 0.941 90.378
0.898 33.137 0.938 94.215
ENSMUSG00000024181 Mrpl28 protein_coding 0.872 1.698 1.032
92.561 1.106 9.86 1.027 93.156
ENSMUSG00000024182 Axin1 protein_coding 1.045 18.388 0.998
94.102 0.96 67.339 1.011 83.067
ENSMUSG00000024184 Pdia2 protein_coding 1 82.705 NA NA
1 85.935 1 95.72
ENSMUSG00000024186 Rgs11 protein_coding 0.909 47.677 0.914
70.29 1.203 18.411 0.958 86.445
ENSMUSG00000024187 Itfg3 protein_coding 1.145 6.76 0.953 49.859
0.918 44.865 0.954 86.991
ENSMUSG00000024188 Luc7l protein_coding 0.906 9.863 0.96 76.431
1.147 8.339 1.011 94.904
ENSMUSG00000024190 Dusp1 protein_coding 1.009 84.049 0.859
68.115 1.153 31.761 0.914 92.742
ENSMUSG00000024191 Bnip1 protein_coding 0.963 38.195 0.875
64.123 1.183 5.864 1.058 77.245
ENSMUSG00000024193 Phf1 protein_coding 0.988 65.199 1.019
96.407 0.993 88.461 0.918 84.19
ENSMUSG00000024194 Cuta protein_coding 0.903 8.181 1.135 71.222

0.971 68.266 1.008 94.904
ENSMUSG00000024197 Plin3 protein_coding 1.05 24.481 0.987
87.553 0.965 63.075 1.025 90.865
ENSMUSG00000024201 Kdm4b protein_coding 0.894 0.987 0.999 85.985
1.116 16.808 0.967 87.651
ENSMUSG00000024206 Rfx2 protein_coding 0.823 36.945 1.4
40.99 0.867 7.329 1.115 76.831
ENSMUSG00000024208 Uqcc2 protein_coding 0.816 10.514 1.089
71.617 1.119 64.024 1.096 76.347
ENSMUSG00000024209 1700061G19Rik protein_coding 0.569 21.897
0.99 95.211 1.777 19.609 1.169 65.558
ENSMUSG00000024210 Ip6k3 protein_coding 2.232 7.842 1.15 95.431
0.387 2.361 0.87 96.358
ENSMUSG00000024211 Grm8 protein_coding 0.906 49.368 0.437
60.678 2.515 10.281 2.377 31.133
ENSMUSG00000024212 Mllt1 protein_coding 0.98 55.303 1.128
60.309 0.906 6.858 1.016 88.657
ENSMUSG00000024213 Nudt3 protein_coding 0.941 42.034 1.088
71.617 0.972 72.269 0.98 92.018
ENSMUSG00000024215 Spdef protein_coding 0.626 8.614 0.795 24.911
2.008 5.864 1.222 93.457
ENSMUSG00000024217 Snrpc protein_coding 0.986 69.24 1.059 78.705
0.955 44.865 1.04 61.984
ENSMUSG00000024218 Taf11 protein_coding 0.969 58.264 1.019
96.138 1.01 64.305 0.97 93.612
ENSMUSG00000024219 Anks1 protein_coding 1.004 68.727 0.929
54.06 1.071 40.511 1.037 85.786
ENSMUSG00000024220 Zfp523 protein_coding 0.954 32.557
1.017 96.897 1.032 47.512 0.97 86.699
ENSMUSG00000024222 Fkbp5 protein_coding 0.73 56.242 0.777
79.475 1.761 25.144 1.712 69.965
ENSMUSG00000024224 Clpsl2 protein_coding 1 84.738 1
94.89 1 81.976 1 95.74
ENSMUSG00000024225 Clps protein_coding 1 59.674 1
96.984 1 82.161 1 95.877
ENSMUSG00000024227 2610034M16Rik protein_coding 0.921 58.802
0.854 88.691 1.27 28.603 0.992 78.042
ENSMUSG00000024228 Nudt12 protein_coding 0.827 19.023
1.016 96.652 1.184 16.205 1.114 73.939
ENSMUSG00000024231 Cul2 protein_coding 1.065 32.238 0.986
91.83 0.951 45.093 1.032 82.85
ENSMUSG00000024232 Bambi protein_coding 1.137 21.703 1.024
96.847 0.856 25.052 0.899 85.493
ENSMUSG00000024233 Lyzl1 protein_coding 1 81.647 1
97.263 1 88.161 1 92.263
ENSMUSG00000024234 Mtpap protein_coding 1.003 28.514 0.985
91.532 1.015 30.107 0.94 83.724
ENSMUSG00000024235 Map3k8 protein_coding 1.131 11.638
0.736 49.266 1.202 51.977 1.096 87.624
ENSMUSG00000024236 Svil protein_coding 0.757 3.401 1.026 92.561
1.283 43.307 1.032 92.675
ENSMUSG00000024238 Zeb1 protein_coding 1.131 7.842 0.964 84.594
0.916 35.815 1.032 60.968
ENSMUSG00000024240 Epc1 protein_coding 1.012 48.462 1.008

90.053	0.981	65.688	0.953	93.261
ENSMUSG00000024241	Sos1	protein_coding	1.084	20.032	1.01
93.863	0.913	15.904	0.943	84.19
ENSMUSG00000024242	Map4k3	protein_coding	1.035	55.124	NA
NA	0.95	73.096	0.952	87.735
ENSMUSG00000024245	Tmem178	protein_coding	1.172	53.562
0.922	94.102	0.926	86.666	1.18	93.964
ENSMUSG00000024246	Thumpd2	protein_coding	1.056	41.211
0.958	88.704	0.989	85.464	0.911	84.29
ENSMUSG00000024247	Pkdcc	protein_coding	1.156	37.091	0.853
72.576	NA	NA	0.953	96.358
ENSMUSG00000024248	Cox7a2l	protein_coding	1.183	1.712	0.879
15.938	0.957	74.179	0.95	92.31
ENSMUSG00000024251	Thada	protein_coding	0.95	59.028	1.014
89.996	1.041	39.925	1.007	96.183
ENSMUSG00000024253	Dync2li1	protein_coding	1.142	27.545
0.922	87.322	0.948	67.029	0.91	92.181
ENSMUSG00000024254	Abcg8	protein_coding	0.784	67.334	1.378
89.434	0.927	75.353	1.13	94.016
ENSMUSG00000024256	Adcyap1	protein_coding	1	48.288	1
92.931	1	51.715	1	92.31
ENSMUSG00000024258	Polr2d	protein_coding	1.061	19.893
1.062	79.411	0.885	3.943	0.969	96.139
ENSMUSG00000024259	Slc25a46	protein_coding	1.054	50.627
0.938	82.947	1.012	86.258	0.957	83.724
ENSMUSG00000024260	Sap130	protein_coding	0.994	65.106
1.069	89.014	0.939	78.713	0.99	91.625
ENSMUSG00000024261	Syt4	protein_coding	1.486	26.863	0.636
49.859	1.055	72.853	1.197	87.447
ENSMUSG00000024266	Adad2	protein_coding	1	67.991	1
97.155	1	86.084	1	95.877
ENSMUSG00000024268	Celf4	protein_coding	1.281	62.574	0.788
83.733	0.991	88.601	1.065	94.904
ENSMUSG00000024269	Tpgs2	protein_coding	0.958	60.707	1.175
60.309	0.891	17.381	1.043	83.067
ENSMUSG00000024270	Slc39a6	protein_coding	1.196	0	0.961
68.818	0.872	2.563	1.015	87.447
ENSMUSG00000024271	Elp2	protein_coding	1.045	36.686	0.956
72.399	1.003	81.857	0.992	86.133
ENSMUSG00000024273	2700062C07Rik	protein_coding	1.004	86.819
0.953	90.053	1.044	69.728	0.964	88.245
ENSMUSG00000024274	Zscan30	protein_coding	0.673	19.985
1.369	88.533	1.081	78.864	1.006	95.273
ENSMUSG00000024276	Zfp397	protein_coding	1.107	15.178
0.972	93.654	0.931	40.794	0.955	85.493
ENSMUSG00000024277	Mapre2	protein_coding	0.98	46.289
0.993	94.597	1.03	45.94	1.111	42.736
ENSMUSG00000024283	Wac	protein_coding	1.109	0.355	1.018	83.104
0.883	0.72	0.947	86.699
ENSMUSG00000024286	Ccny	protein_coding	1.014	68.485	0.962
86.538	1.022	66.269	1.022	89.108
ENSMUSG00000024287	Thoc1	protein_coding	1.072	52.429	0.978
90.138	0.952	80.749	0.962	96.183
ENSMUSG00000024290	Rock1	protein_coding	1.18	11.197	0.945

82.947 0.899 28.901 0.921 96.183
ENSMUSG00000024292 Cyp4f14 protein_coding 0.568 39.174
1.101 90.092 1.593 62.307 1.245 95.35
ENSMUSG00000024293 Esco1 protein_coding 1.083 9.983 1.022 89.014
0.9 9.746 1.062 90.177
ENSMUSG00000024294 Mib1 protein_coding 0.946 29.725 0.974
79.475 1.087 15.185 0.956 83.724
ENSMUSG00000024295 Cyp4f41-ps pseudogene 0.854 73.292 0.985
83.733 1.186 38.038 0.807 91.105
ENSMUSG00000024298 Zfp871 protein_coding 0.518 2.009 NA NA
2.007 5.864 0.955 92.12
ENSMUSG00000024299 Adamts10 protein_coding 0.899 49.944
1.104 90.454 1.003 83.857 1.017 95.273
ENSMUSG00000024300 Myo1f protein_coding 1.356 0.355 1.066 91.361
0.69 8.907 1.078 93.156
ENSMUSG00000024301 Kifc5b protein_coding 0.923 86.006
0.825 79.475 1.31 48.451 1.471 59.464
ENSMUSG00000024302 Dtna protein_coding 1.114 16.436 1.139
79.29 0.785 1.973 0.976 93.016
ENSMUSG00000024304 Cdh2 protein_coding 1.029 59.806 1.122
83.104 0.867 14.788 0.976 96.248
ENSMUSG00000024306 Ccdc178 protein_coding 1 83.823 1
95.211 NA NA 1 96.358
ENSMUSG00000024308 Tapbp protein_coding 1.024 52.134 1.055
91.584 0.926 39.528 1.026 71.305
ENSMUSG00000024309 H2-Ke2 protein_coding 0.9 4.73 1.039
85.756 1.07 28.301 0.996 96.358
ENSMUSG00000024312 Wdr46 protein_coding 0.888 16.652 1.043
84.007 1.073 43.185 1.1 59.464
ENSMUSG00000024313 Mep1b protein_coding 1 66.652 1
96.407 1 77.17 1 96.183
ENSMUSG00000024317 Rnf138 protein_coding 1.117 1.712 0.942
72.399 0.952 26.809 0.903 77.012
ENSMUSG00000024319 Vps52 protein_coding 0.942 1.379 0.964 93.654
1.097 10.76 1.02 70.624
ENSMUSG00000024325 Ring1 protein_coding 0.757 0.987 0.996 92.137
1.324 5.864 1.007 86.991
ENSMUSG00000024327 Slc39a7 protein_coding 0.843 7.71 1.093
87.282 1.086 45.035 1.097 62.918
ENSMUSG00000024330 Col11a2 protein_coding 0.632 0.987 NA NA
1.616 6.506 0.89 91.372
ENSMUSG00000024331 Dsc2 protein_coding 1.161 39.416 1.055
93.654 0.818 64.155 1.097 95.273
ENSMUSG00000024334 H2-Oa protein_coding 1.164 55.789 1.361
93.146 0.632 33.137 0.902 95.877
ENSMUSG00000024335 Brd2 protein_coding 0.963 54.983 1.048
81.127 0.989 59.726 0.974 83.724
ENSMUSG00000024338 Psmb8 protein_coding 1.065 27.731 1.018
96.013 0.925 29.451 1.055 79.713
ENSMUSG00000024339 Tap2 protein_coding 0.917 56.477 1.152
71.617 0.946 53.7 0.965 91.372
ENSMUSG00000024340 Btnl2 protein_coding 2.747 1.967 0.468 64.555
0.778 25.625 0.985 88.245
ENSMUSG00000024346 Pfdn1 protein_coding 0.946 47.677 0.982

95.211	1.079	24.383	1.082	67.169
ENSMUSG00000024347	Psd2	protein_coding	0.95	66.652	1.003	87.857	1.052	34.727	0.915	88.136
ENSMUSG00000024349	Tmem173	protein_coding	1.198	1.441	1.097	93.899	0.76	8.907	1.133	68.696
ENSMUSG00000024350	Dnajc18	protein_coding	1.082	19.893	1	96.68	0.923	15.904	1.083	36.988
ENSMUSG00000024352	Spata24	protein_coding	1.13	27.545	0.955	94.102	0.925	42.895	0.997	86.991
ENSMUSG00000024353	Mzb1	protein_coding	1.235	58.896	1.305	79.29	0.621	15.465	0.896	94.608
ENSMUSG00000024354	Slc23a1	protein_coding	1.239	68.16	0.578	70.29	1.398	59.274	1.01	92.12
ENSMUSG00000024357	Sil1	protein_coding	1.043	43.019	0.924	67.513	1.032	54.363	0.989	92.381
ENSMUSG00000024359	Hspa9	protein_coding	0.96	48.146	NA	NA	1.082	53.882	1.07	88.512
ENSMUSG00000024360	Etf1	protein_coding	1.051	13.557	0.972	65.165	0.973	69.134	1.069	40.894
ENSMUSG00000024365	Cyp21a1	protein_coding	1.045	83.726	0.742	83.321	1.29	66.647	1.034	90.177
ENSMUSG00000024366	Gfra3	protein_coding	1.087	64.714	0.998	96.763	0.923	57.819	0.935	86.699
ENSMUSG00000024369	Nelfe	protein_coding	0.886	0.987	1.056	83.104	1.069	6.506	0.961	96.192
ENSMUSG00000024370	Cdc23	protein_coding	1.077	6.76	0.931	59.946	1	74.853	0.99	93.612
ENSMUSG00000024371	C2	protein_coding	1.023	84.738	1.204	71.617	0.812	9.456	1.044	78.948
ENSMUSG00000024376	Epb4.1l4a	protein_coding	1.058	27.545	NA	NA	0.946	69.134	0.834	83.724
ENSMUSG00000024378	Stard4	protein_coding	1.014	57.38	1.144	74.663	0.859	11.314	0.999	96.248
ENSMUSG00000024379	Tslp	protein_coding	1.233	36.195	0.794	60.678	1.02	83.275	0.68	94.752
ENSMUSG00000024381	Bin1	protein_coding	1.27	3.598	NA	NA	0.784	0.945	1.227	57.1
ENSMUSG00000024382	Ercc3	protein_coding	0.935	30.136	1.028	82.904	1.036	71.232	1.08	44.679
ENSMUSG00000024383	Map3k2	protein_coding	0.987	79.709	0.945	91.361	1.068	53.587	0.905	83.724
ENSMUSG00000024384	Iws1	protein_coding	0.924	2.436	0.976	88.691	1.11	5.864	0.951	87.331
ENSMUSG00000024386	Proc	protein_coding	1.054	63.385	0.804	47.727	1.183	41.235	0.774	88.505
ENSMUSG00000024387	Csnk2b	protein_coding	0.965	24.748	1.032	87.609	1.006	74.325	0.989	92.018
ENSMUSG00000024388	Myo7b	protein_coding	0.654	31.921	0.786	72.576	1.947	12.703	1.315	72.204
ENSMUSG00000024391	Apom	protein_coding	0.95	80.751	0.384	55.71	2.744	10.819	0.922	86.699
ENSMUSG00000024392	Bag6	protein_coding	0.9	0.987	1.038	80.277	1.073	6.693	0.982	88.505
ENSMUSG00000024393	Prrc2a	protein_coding	0.94	1.175	1.01

82.904	1.056	32.524	0.981	92.181
ENSMUSG00000024395	Lims2	protein_coding	1.038	84.661	0.793
43.505	1.216	15.688	1.008	94.904
ENSMUSG00000024397	Aif1	protein_coding	1.713	2.361	NA	NA
0.504	1.342	1.132	88.512
ENSMUSG00000024399	Ltb	protein_coding	0.812	48.939	1.202
82.904	NA	NA	0.804	84.29
ENSMUSG00000024400	Wdr33	protein_coding	1.009	59.806	0.981
79.475	1.01	88.461	1.012	88.387
ENSMUSG00000024401	Tnf	protein_coding	1.071	74.8	1.029	97.263
0.908	85.21	0.848	92.561
ENSMUSG00000024402	Lta	protein_coding	1.247	35.748	0.813
79.475	0.989	88.349	1.721	74.193
ENSMUSG00000024403	Atp6v1g2	protein_coding	0.911	35.646
0.985	95.088	1.117	30.292	0.888	84.29
ENSMUSG00000024404	Riok3	protein_coding	1.041	44.334	1.015
88.691	0.949	45.799	1.014	79.268
ENSMUSG00000024406	Pou5f1	protein_coding	1	74.482	1
91.361	1	58.051	1	85.786
ENSMUSG00000024409	Psors1c2	protein_coding	0.677	40.786
0.977	95.088	1.512	52.8	2.408	71.653
ENSMUSG00000024410	3110002H16Rik	protein_coding	0.949	39.174
1.017	96.335	1.038	60.789	0.97	85.493
ENSMUSG00000024411	Aqp4	protein_coding	2.693	16.539	0.983
91.643	0.376	4.48	1.245	87.447
ENSMUSG00000024413	Npc1	protein_coding	1.221	7.799	0.826	72.576
0.989	69.583	0.944	85.493
ENSMUSG00000024414	Mrpl27	protein_coding	0.897	4.73	1.024
90.895	1.092	42.75	1.046	93.156
ENSMUSG00000024420	Zfp521	protein_coding	1.08	25.026
0.952	80.052	0.97	74.853	1.016	94.608
ENSMUSG00000024421	Lama3	protein_coding	0.862	10.143	1.124
71.98	1.029	84.896	1.022	86.699
ENSMUSG00000024422	Dhx16	protein_coding	0.952	31.333	0.973
94.597	1.08	33.802	1.017	95.35
ENSMUSG00000024423	Impact	protein_coding	1.097	1.292	1.042
92.111	0.876	5.564	1.002	94.904
ENSMUSG00000024424	Ttc39c	protein_coding	0.827	13.239
0.952	88.345	1.27	10.76	1.102	74.607
ENSMUSG00000024425	Ndfip1	protein_coding	1.089	1.689	0.947
50.664	0.971	46.956	1.032	46.846
ENSMUSG00000024426	Atat1	protein_coding	0.795	2.142	1.165	66.01
1.077	64.305	0.958	83.724
ENSMUSG00000024427	Spry4	protein_coding	1.003	44.787	0.963
87.037	1.037	83.066	1.056	85.786
ENSMUSG00000024429	Gnl1	protein_coding	0.968	43.816	1.012
93.654	1.02	43.307	1.055	78.379
ENSMUSG00000024430	Cabyr	protein_coding	0.699	20.369	1.114
96.216	1.285	51.596	1.308	57.1
ENSMUSG00000024431	Nr3c1	protein_coding	1.015	69.408	1.12
71.617	0.877	8.907	0.953	86.699
ENSMUSG00000024436	Mrps18b	protein_coding	0.983	65.199
1.039	83.104	0.979	79.255	0.992	86.209
ENSMUSG00000024437	Gm8615	pseudogene	0.849	71.763	1.787

82.904 0.66 20.511 0.848 94.654
ENSMUSG00000024440 Pcdh12 protein_coding 0.878 60.569
1.608 71.617 0.705 14.583 0.874 84.29
ENSMUSG00000024442 0610009O20Rik protein_coding 0.981 32.749
1.066 67.23 0.957 71.999 0.98 96.287
ENSMUSG00000024446 Rpp21 protein_coding 0.87 13.581 1.057
86.387 1.085 38.152 1 96.183
ENSMUSG00000024448 H2-M10.1 protein_coding 1 84.397 1
95.211 1 84.83 1 95.273
ENSMUSG00000024451 Arap3 protein_coding 0.851 5.962 1.019 93.899
1.152 10.819 0.983 84.29
ENSMUSG00000024454 Hdac3 protein_coding 0.995 47.677 0.983
96.001 1.02 55.352 0.975 85.636
ENSMUSG00000024456 Diap1 protein_coding 0.991 73.841 0.915
8.462 1.102 13.356 1.072 54.265
ENSMUSG00000024457 Trim26 protein_coding 1.007 68.16 0.937
72.399 1.055 55.109 1.026 73.939
ENSMUSG00000024459 H2-M5 protein_coding 0.883 84.049 1.13
97.155 1.006 83.275 1.048 94.904
ENSMUSG00000024462 Gabbr1 protein_coding 0.93 7.58 0.975
94.102 1.103 16.808 0.941 88.505
ENSMUSG00000024471 Myot protein_coding 6.706 6.61 1.168 88.533
0.128 0.72 0.943 95.273
ENSMUSG00000024472 Dcp2 protein_coding 1.018 53.228 0.97
75.993 1.01 88.729 1.037 82.85
ENSMUSG00000024474 Ik protein_coding 0.978 47.266 1.02
97.288 1.005 51.977 1.036 40.024
ENSMUSG00000024477 NA NA NA NA 0.885 42.923 1.123
13.356 0.998 91.625
ENSMUSG00000024479 Mal2 protein_coding 0.853 21.957 1.174
83.104 0.999 71.839 0.944 86.254
ENSMUSG00000024480 Ap3s1 protein_coding 0.961 75.761 0.967
94.848 1.073 62.648 0.964 85.493
ENSMUSG00000024481 4833403I15Rik protein_coding 1 69.777
0.671 21.426 1.488 8.647 0.948 86.991
ENSMUSG00000024483 Ankhd1 protein_coding 1.042 37.091
0.948 78.877 1.011 87.274 1.018 95.832
ENSMUSG00000024486 Hbegf protein_coding 1.131 46.097 0.975
95.088 0.908 32.963 1.096 96.287
ENSMUSG00000024487 Yipf5 protein_coding 1.088 23.493 1.014
89.494 0.907 9.288 1.052 44.075
ENSMUSG00000024491 Rbm27 protein_coding 1.022 58.896 1.027
89.996 0.951 29.217 1.02 73.939
ENSMUSG00000024493 Lars protein_coding 0.977 66.897 NA NA
1.032 54.809 1.155 31.133
ENSMUSG00000024497 Pou4f3 protein_coding 1 82.352 1
95.088 1 84.626 1 95.593
ENSMUSG00000024498 Tcerg1 protein_coding 1.01 75.196
1.014 96.847 0.972 81.169 1.131 43.605
ENSMUSG00000024500 Ppp2r2b protein_coding 1.294 11.638
0.949 90.744 0.816 31.887 1.078 87.7
ENSMUSG00000024501 Dpysl3 protein_coding 1.316 6.853 0.91
95.211 0.836 11.751 1.105 69.965
ENSMUSG00000024502 Jakmip2 protein_coding 1.366 21.539 NA

NA	0.694	51.715	0.797	85.493
ENSMUSG00000024505	Dtwd2	protein_coding	1.145	22.349	0.986
95.196	0.882	7.155	1.027	95.877
ENSMUSG00000024507	Hsd17b4	protein_coding	0.853	3.009	1.021
96.68	1.148	7.635	1.068	67.691
ENSMUSG00000024510	Ftmt	protein_coding	1	83.823	1
94.848	1	83.158	1	96.287
ENSMUSG00000024511	Rab27b	protein_coding	0.939	78.744
0.916	84.017	1.166	50.636	1.017	86.991
ENSMUSG00000024512	Dynap	protein_coding	1	82.092	1
96.216	1	73.759	1	93.261
ENSMUSG00000024513	Mbd2	protein_coding	1.064	54.717	1.104
82.219	0.852	18.336	1.043	93.016
ENSMUSG00000024515	Smad4	protein_coding	1.067	5.313	0.975	45.262
0.958	70.791	0.968	86.991
ENSMUSG00000024516	Sec11c	protein_coding	1.026	51.189
0.948	86.538	NA	NA	1.077	66.068
ENSMUSG00000024517	Grp	protein_coding	1	73.734	1
96.013	1	86.346	1	91.492
ENSMUSG00000024521	Pmaip1	protein_coding	1.353	7.492	0.776
42.923	0.948	77.294	1.106	94.825
ENSMUSG00000024524	Gnal	protein_coding	0.907	33.224	0.908
95.708	1.209	57.907	0.941	87.735
ENSMUSG00000024525	Impa2	protein_coding	1.077	84.397	0.962
97.155	0.962	84.83	0.8	83.724
ENSMUSG00000024526	Cidea	protein_coding	0.626	30.877	0.959
89.996	1.666	29.302	1.598	94.99
ENSMUSG00000024527	Afg3l2	protein_coding	1.024	41.454
0.939	75.052	1.042	69.002	1.012	96.358
ENSMUSG00000024528	Srfbp1	protein_coding	0.974	51.56	0.967
85.116	1.06	25.144	1.006	82.85
ENSMUSG00000024529	Lox	protein_coding	0.939	77.291	0.826
46.764	1.284	32.524	1.184	93.627
ENSMUSG00000024530	Slmo1	protein_coding	0.873	80.146	0.952
84.594	1.198	30.561	0.966	93.016
ENSMUSG00000024532	1700034E13Rik	protein_coding	1	80.751
1	97.288	1	78.954	1	96.139
ENSMUSG00000024533	Spire1	protein_coding	1.008	79.277
0.924	37.426	1.071	36.011	1.056	76.831
ENSMUSG00000024534	Sncaip	protein_coding	1.061	67.163
1.027	95.735	0.916	45.093	1.042	76.697
ENSMUSG00000024535	Snx24	protein_coding	1.212	7.908	0.922	85.671
0.892	25.625	1.077	82.328
ENSMUSG00000024537	Psmg2	protein_coding	1.101	21.599	1.085
85.756	0.836	25.052	1.001	95.92
ENSMUSG00000024538	Ppic	protein_coding	1.15	8.365	0.951	92.545
0.912	32.703	1.126	62.918
ENSMUSG00000024539	Ptpn2	protein_coding	1.191	3.932	0.883	72.399
0.948	46.071	0.999	86.991
ENSMUSG00000024542	Cep192	protein_coding	1.052	68.16	0.964
84.594	0.988	81.976	1.052	84.31
ENSMUSG00000024544	Ldlrad4	protein_coding	1.117	24.689
0.877	63.773	1.022	86.144	1.019	93.964
ENSMUSG00000024546	4930546C10Rik	protein_coding	1	81.178

1	95.329	1	84.308	1	96.358
ENSMUSG00000024548	Setbp1	protein_coding	0.987	52.726
0.972	93.654	1.043	30.292	0.928	92.742
ENSMUSG00000024556	Me2	protein_coding	1.147	6.61	0.914	90.053
0.954	40.19	1.225	57.1
ENSMUSG00000024558	Mapk4	protein_coding	0.791	58.264	1.232
96.407	1.025	64.624	1.074	91.525
ENSMUSG00000024560	Cxxc1	protein_coding	0.945	34.572	1.029
88.136	1.033	80.634	0.983	95.74
ENSMUSG00000024561	Mbd1	protein_coding	0.969	72.985	0.914
74.483	1.13	28.997	1.08	71.855
ENSMUSG00000024563	Smad2	protein_coding	1.037	32.676	1.045
85.756	0.918	11.902	0.96	84.29
ENSMUSG00000024566	Atp9b	protein_coding	1.022	48.857	1.001
94.597	0.975	58.271	1.045	37.634
ENSMUSG00000024570	Rbfa	protein_coding	0.936	41.33	1.005	95.941
1.062	54.673	0.995	93.156
ENSMUSG00000024571	Gm16286	protein_coding	1.039	29.596
1.015	97.263	0.946	33.971	1.051	53.923
ENSMUSG00000024575	Pde6a	protein_coding	1.674	60.435	0.587
94.262	1.02	75.353	0.811	90.95
ENSMUSG00000024576	Csnk1a1	protein_coding	1.058	1.949	0.978
70.29	0.966	42.621	0.961	84.29
ENSMUSG00000024578	Il17b	protein_coding	0.602	17.062	1.105
93.863	1.506	15.945	1.087	88.197
ENSMUSG00000024579	Pcyox1l	protein_coding	0.734	4.176	1.207
87.609	1.128	27.475	1.052	91.783
ENSMUSG00000024580	Grpel2	protein_coding	0.981	59.358
1.027	93.27	0.988	86.258	0.961	85.493
ENSMUSG00000024581	Napg	protein_coding	0.957	22.431	0.984
96.138	1.061	33.787	0.966	91.625
ENSMUSG00000024583	Txnl1	protein_coding	1.017	75.761	0.952
95.211	1.031	69.196	1.027	86.209
ENSMUSG00000024587	Nars	protein_coding	1.023	77.291	0.97
68.818	1.011	49.384	1.101	38.624
ENSMUSG00000024588	Fech	protein_coding	0.96	71.36	1.066	79.411
0.974	39.755	1.037	86.445
ENSMUSG00000024589	Nedd4l	protein_coding	0.991	67.991
0.983	85.418	1.028	87.103	1.099	75.198
ENSMUSG00000024590	Lmnb1	protein_coding	1.082	50.913	1.001
96.216	0.927	73.531	1.281	55.841
ENSMUSG00000024592	C330018D20Rik	protein_coding	0.992	81.196
1.063	96.335	0.948	71.999	0.967	96.248
ENSMUSG00000024593	Megf10	protein_coding	1.206	46.875	NA
NA	0.796	61.743	0.782	90.157
ENSMUSG00000024594	Prrc1	protein_coding	1.039	32.411	1.056
78.705	0.914	3.234	0.948	84.29
ENSMUSG00000024597	Slc12a2	protein_coding	0.997	80.251
0.889	83.876	1.125	67.163	0.954	88.505
ENSMUSG00000024598	Fbn2	protein_coding	0.883	48.146	0.934
95.196	1.215	84.83	1.295	77.854
ENSMUSG00000024600	Slc27a6	protein_coding	1.81	11.459
1.047	96.763	0.526	23.86	1.32	44.882
ENSMUSG00000024601	Isoc1	protein_coding	0.976	81.465	1.093

87.037 0.937 44.211 0.98 91.372
ENSMUSG00000024603 Dctn4 protein_coding 1.032 29.057 NA NA
0.95 15.376 0.972 83.724
ENSMUSG00000024604 Rbm22 protein_coding 1.019 56.907 0.974
75.493 1.009 73.428 1.002 91.217
ENSMUSG00000024608 Rps14 protein_coding 1.016 80.03 0.883 11.228
1.112 18.797 1.034 95.877
ENSMUSG00000024610 Cd74 protein_coding 1.002 78.538 1.187
89.996 0.838 83.607 0.834 84.19
ENSMUSG00000024613 Tcof1 protein_coding 1.075 25.207 NA NA
0.92 30.024 1.081 83.067
ENSMUSG00000024614 Tmx3 protein_coding 1.14 2.085 0.95 90.046
0.92 10.726 0.97 95.593
ENSMUSG00000024617 Camk2a protein_coding 2.594 11.879
1.682 82.809 0.229 0.879 0.83 95.877
ENSMUSG00000024619 Cdx1 protein_coding 1 85.728 1
95.708 1 73.759 1 83.067
ENSMUSG00000024620 Pdgfrb protein_coding 1.104 46.013 1.04
87.029 0.87 24.918 0.988 90.674
ENSMUSG00000024621 Csf1r protein_coding 1.392 2.352 0.828 65.165
0.864 45.093 0.96 90.611
ENSMUSG00000024622 Hmgxb3 protein_coding 1.012 52.9 1.02
84.832 0.966 81.857 0.974 96.358
ENSMUSG00000024639 Gnaq protein_coding 1.002 80.03 1.013 94.367
0.98 60.233 0.977 84.29
ENSMUSG00000024640 Psat1 protein_coding 0.937 31.333 1.086
81.127 0.98 67.029 0.987 90.157
ENSMUSG00000024642 Tle4 protein_coding 1.105 5.527 1.064 82.76
0.848 0.72 1.018 93.734
ENSMUSG00000024644 Cndp2 protein_coding 1.104 0.999 0.964 90.256
0.94 28.901 1.185 0
ENSMUSG00000024645 Timm21 protein_coding 0.948 70.213
1.044 95.431 1.013 85.757 1.026 96.287
ENSMUSG00000024646 Cyb5 protein_coding 0.839 7.58 0.92 85.671
1.292 11.151 1.134 94.825
ENSMUSG00000024647 Cbln2 protein_coding 1.318 80.408 0.881
94.848 NA NA 0.866 95.593
ENSMUSG00000024650 Slc22a6 protein_coding 1 86.474 1
96.001 1 83.925 1 95.74
ENSMUSG00000024654 Asrgl1 protein_coding 1.118 30.282
0.927 79.261 0.963 86.985 1.011 91.928
ENSMUSG00000024659 Anxa1 protein_coding 0.986 86.006 0.999
92.355 1.015 83.066 1.254 31.133
ENSMUSG00000024660 Incenp protein_coding 1.045 71.516
1.034 97.049 0.923 71.734 1.203 77.621
ENSMUSG00000024661 Fth1 protein_coding 0.964 35.646 1.043
71.98 0.995 78.045 1.033 79.545
ENSMUSG00000024663 Rab3il1 protein_coding 1.412 0.999 1.044
96.847 0.678 0.72 1.106 31.133
ENSMUSG00000024664 Fads3 protein_coding 0.749 2.524 1.165 71.617
1.146 26.978 1.083 83.067
ENSMUSG00000024665 Fads2 protein_coding 0.944 57.014 1.154
87.609 0.92 77.037 0.937 84.29
ENSMUSG00000024666 Tmem138 protein_coding 0.926 76.135

1.081 83.464 0.996 87.686 1.058 92.018
ENSMUSG00000024667 Tmem216 protein_coding 0.953 30.877
0.946 89.509 1.108 27.732 0.962 95.273
ENSMUSG00000024668 Sdhaf2 protein_coding 1.021 82.231
0.993 95.504 0.988 83.607 1.025 63.708
ENSMUSG00000024669 Cd5 protein_coding 1.243 72.655 1.322
80.775 0.609 33.137 0.793 91.625
ENSMUSG00000024670 Cd6 protein_coding 1.064 84.452 1.239
96.216 0.76 83.811 0.581 83.724
ENSMUSG00000024672 Ms4a7 protein_coding 1.509 3.825 0.874 68.115
0.759 41.662 0.908 94.174
ENSMUSG00000024673 Ms4a1 protein_coding 1.076 86.112 2.137
91.643 0.433 61.743 0.868 96.183
ENSMUSG00000024675 Ms4a4c protein_coding 2.475 0 1.027
66.796 0.393 17.258 1.556 51.967
ENSMUSG00000024677 Ms4a6b protein_coding 1.482 0.767 0.861
52.266 0.783 62.791 1.107 63.708
ENSMUSG00000024678 Ms4a4d protein_coding 1.189 11.928
1.074 95.708 0.781 20.511 1.122 53.923
ENSMUSG00000024679 Ms4a6d protein_coding 1.55 0.435 0.857
71.41 0.753 28.237 1.655 46.412
ENSMUSG00000024680 Ms4a2 protein_coding 1.133 36.686 1.284
83.104 0.69 9.456 1.031 87.735
ENSMUSG00000024681 Ms4a3 protein_coding 1 81.01 1 97.288
1 87.744 1 93.612
ENSMUSG00000024682 Gif protein_coding 1 72.372 1
95.504 1 74.179 1 92.181
ENSMUSG00000024683 Mrpl16 protein_coding 0.931 45.229
1.089 75.62 0.983 76.64 1.114 90.397
ENSMUSG00000024687 Osbp protein_coding 1.002 79.433 1.022
71.617 0.978 33.137 1.02 88.197
ENSMUSG00000024691 Fam111a protein_coding 1.403 0.767 1.026
95.088 0.692 1.342 1.21 43.377
ENSMUSG00000024694 Keg1 protein_coding 1 85.203 1
97.049 1 80.163 1 94.215
ENSMUSG00000024695 Zfp91 protein_coding 0.985 58.077 NA NA
1.012 61.743 1.004 86.301
ENSMUSG00000024696 Lpxn protein_coding 1.341 0.2 0.84 60.678
0.884 25.345 1.106 93.132
ENSMUSG00000024697 Gna14 protein_coding 1.07 37.414 0.991
88.691 0.939 84.552 0.842 83.724
ENSMUSG00000024712 Rfk protein_coding 1.028 50.913 NA NA
0.972 35.815 0.988 86.991
ENSMUSG00000024713 Pcsk5 protein_coding 1.206 2.569 0.817 59.946
1.012 80.749 1.302 57.1
ENSMUSG00000024725 Ostf1 protein_coding 0.99 61.043 0.995
92.793 1.012 81.492 1.02 67.169
ENSMUSG00000024726 2410127L17Rik protein_coding 1.078 10.595
1.071 91.643 0.867 6.858 1.028 93.734
ENSMUSG00000024727 Trpm6 protein_coding 0.912 33.599 0.73
62.692 1.507 12.16 0.852 83.724
ENSMUSG00000024728 1700025F22Rik protein_coding 1 82.352
1 96.68 1 88.161 1 95.74
ENSMUSG00000024729 1700017D01Rik protein_coding 1 56.311

1 94.262 1 87.686 1 91.269
ENSMUSG00000024730 Ms4a8a protein_coding 1.63 0 0.796
72.576 0.771 47.12 1.095 79.268
ENSMUSG00000024731 Ms4a10 protein_coding 1 81.196 NA
NA 1 84.434 1 95.593
ENSMUSG00000024732 Ccdc86 protein_coding 0.874 51.056
1.063 80.678 1.076 86.727 1.159 72.116
ENSMUSG00000024735 Prpf19 protein_coding 0.931 9.863 1.028
83.104 1.042 31.975 1.045 67.691
ENSMUSG00000024736 Tmem132a protein_coding 0.758 0.987 1.032
92.561 1.277 6.506 0.963 96.192
ENSMUSG00000024737 Slc15a3 protein_coding 0.868 19.023
1.079 95.088 1.066 38.926 0.884 85.493
ENSMUSG00000024738 Pga5 protein_coding 1 71.915 1
95.504 1 56.497 1 95.877
ENSMUSG00000024740 Ddb1 protein_coding 0.966 54.176 0.975
71.889 1.064 26.912 1.104 31.133
ENSMUSG00000024742 Fen1 protein_coding 1.109 39.416 0.976
81.02 0.926 87.744 1.28 31.133
ENSMUSG00000024743 Syt7 protein_coding 1.11 29.596 0.891
49.859 1.005 47.31 0.893 83.724
ENSMUSG00000024747 Aldh1a7 protein_coding 0.578 0.987 1.001
93.707 1.729 5.864 1.033 82.85
ENSMUSG00000024750 Zfand5 protein_coding 1.045 35.748
0.955 71.889 NA NA 1.033 80.187
ENSMUSG00000024754 Tmem2 protein_coding 0.902 58.264 0.838
82.947 1.325 26.498 1.039 95.74
ENSMUSG00000024757 Slc22a19 protein_coding 1 85.129 NA
NA 1 88.601 NA NA
ENSMUSG00000024758 Rtn3 protein_coding 1.045 34.077 1.046
89.014 0.915 7.742 0.975 83.724
ENSMUSG00000024759 Atl3 protein_coding 0.964 38.195 1.025
87.185 1.011 52.8 1.009 96.287
ENSMUSG00000024761 Gm16437 pseudogene 1 74.482 NA NA
1 86.084 1 93.964
ENSMUSG00000024764 Naa40 protein_coding 1.094 12.72 0.998 97.263
0.919 5.323 0.992 90.157
ENSMUSG00000024766 Lipo1 protein_coding 0.851 22.142 1.083
83.464 1.084 48.935 1.131 76.046
ENSMUSG00000024767 Otub1 protein_coding 1.004 85.832 1.028
91.584 0.97 57.446 1.052 81.56
ENSMUSG00000024769 Cdc42bpg protein_coding 0.911 12.436
1.026 95.301 1.073 15.241 0.962 92.851
ENSMUSG00000024770 Lipn protein_coding 1 85.728 1
96.763 1 86.144 1 83.276
ENSMUSG00000024772 Ehd1 protein_coding 0.881 6.221 1.026 90.389
1.109 17.412 1.194 57.952
ENSMUSG00000024773 Atg2a protein_coding 0.895 0.987 1.024 84.832
1.093 11.782 0.963 87.651
ENSMUSG00000024774 Ankrd22 protein_coding 1.03 81.465
1.269 97.288 0.761 87.686 2.437 49.806
ENSMUSG00000024776 Stambpl1 protein_coding 0.997 70.121
1.007 95.431 0.996 51.055 0.991 91.65
ENSMUSG00000024777 Ppp2r5b protein_coding 0.827 0.987 1.15

72.546	1.047	30.107	1.053	52.324
ENSMUSG00000024778	Fas	protein_coding	1.081	47.466	0.901
59.946	1.029	52.432	1.008	90.865
ENSMUSG00000024780	Cdc37l1	protein_coding	1.018	85.946
0.939	90.287	1.049	74.082	1.013	96.086
ENSMUSG00000024781	Lipa	protein_coding	1.345	0	0.99	93.046
0.75	0.72	0.948	85.493
ENSMUSG00000024782	Ak3	protein_coding	0.884	4.268	1.092	71.98
1.036	43.842	0.977	96.183
ENSMUSG00000024785	Rcl1	protein_coding	1.106	14.746	0.933
74.483	0.966	85.675	0.971	90.691
ENSMUSG00000024786	1700123I01Rik	protein_coding	0.967	81.01	1.25
88.551	0.827	60.614	0.821	96.358
ENSMUSG00000024787	Snx15	protein_coding	0.934	14.086	1.072
82.904	0.999	72.148	0.986	94.001
ENSMUSG00000024789	Jak2	protein_coding	1.152	25.207	NA	NA
0.859	32.703	1.025	82.328
ENSMUSG00000024790	Sac3d1	protein_coding	0.925	25.551
1.057	81.039	1.019	80.015	0.96	94.016
ENSMUSG00000024791	Cdca5	protein_coding	1.045	66.272	1.004
91.532	0.951	85.58	1.751	46.412
ENSMUSG00000024792	Zfpl1	protein_coding	0.968	41.33	1.011	87.609
1.023	61.418	0.927	83.724
ENSMUSG00000024793	Tnfrsf25	protein_coding	0.804	26.013
1.093	92.111	1.137	58.051	1.026	93.132
ENSMUSG00000024795	Kif20b	protein_coding	1.316	16.539
0.912	70.971	0.833	68.82	1.079	91.492
ENSMUSG00000024797	Vps51	protein_coding	0.933	24.748	0.999
75.052	1.074	10.523	1.009	94.904
ENSMUSG00000024798	Htr7	protein_coding	1.287	32.238	1.344
82.904	0.575	7.809	1.041	94.904
ENSMUSG00000024799	Tm7sf2	protein_coding	0.65	0.987	1.078
75.612	1.427	7.24	1.065	89.108
ENSMUSG00000024800	Rpp30	protein_coding	1.047	57.949	0.972
95.431	0.982	37.906	1.03	69.918
ENSMUSG00000024803	Ankrd1	protein_coding	1.695	22.633
1.758	81.039	0.334	6.68	1.167	91.335
ENSMUSG00000024805	Pcgf5	protein_coding	1.037	58.38	1.014	95.431
0.948	35.264	0.999	94.297
ENSMUSG00000024806	Mlana	protein_coding	1.512	85.728	0.564
85.418	1.169	82.778	0.628	84.29
ENSMUSG00000024807	Syvn1	protein_coding	0.9	6.484	1.117	60.309
0.995	71.734	NA	NA
ENSMUSG00000024810	Il33	protein_coding	0.865	49.621	1.219
82.904	0.95	88.693	1.342	64.905
ENSMUSG00000024811	Tnks2	protein_coding	1.083	17.761	0.949
78.51	0.971	66.425	0.981	96.139
ENSMUSG00000024812	Tjp2	protein_coding	0.912	5.084	1.015	96.216
1.084	22.517	0.942	83.724
ENSMUSG00000024815	Trpd52l3	protein_coding	1	80.751	1
95.088	1	86.552	1	94.904
ENSMUSG00000024816	Frmd8	protein_coding	1.007	80.608	1.058
92.062	0.939	73.096	0.959	90.611
ENSMUSG00000024817	Uhrf2	protein_coding	0.987	80.03	1.052	95.504

0.962 87.744 0.964 85.493
ENSMUSG00000024818 Slc25a45 protein_coding 1.092 13.363
0.937 72.576 0.979 81.62 1.102 61.984
ENSMUSG00000024824 Rad9a protein_coding 0.969 34.572 1.122
75.62 0.92 61.743 0.98 91.985
ENSMUSG00000024826 Dpf2 protein_coding 0.977 54.043 1.059
81.039 0.963 56.822 0.949 83.724
ENSMUSG00000024827 Gldc protein_coding 0.853 34.305 1.344
82.76 0.872 88.461 0.922 95.35
ENSMUSG00000024829 Mrpl21 protein_coding 0.901 5.962 0.998
91.532 1.112 16.808 1.052 80.77
ENSMUSG00000024830 Rps6kb2 protein_coding 0.931 7.916 1.021
83.104 1.049 44.787 0.99 95.877
ENSMUSG00000024831 Ighmbp2 protein_coding 0.935 16.183
0.911 71.41 1.17 12.16 1.085 63.708
ENSMUSG00000024833 Pola2 protein_coding 0.945 58.264 0.968
88.705 1.089 40.955 0.984 95.676
ENSMUSG00000024835 Coro1b protein_coding 0.93 22.237
0.969 81.443 1.107 24.791 1.013 91.525
ENSMUSG00000024837 Dmrt1 protein_coding 1 81.76 1 95.708
1 88.203 1 96.358
ENSMUSG00000024841 Eif1ad protein_coding 1.007 58.38 1.044
92.931 0.947 31.676 0.99 94.297
ENSMUSG00000024842 Cabp4 protein_coding 0.915 54.176 0.78
78.471 1.404 24.985 0.81 91.625
ENSMUSG00000024843 Chka protein_coding 1.131 51.737 0.803
66.09 1.096 84.229 1.128 71.008
ENSMUSG00000024844 Banf1 protein_coding 0.857 9.863 1.043 91.065
1.115 40.575 1.059 72.116
ENSMUSG00000024845 Tmem134 processed_transcript 0.879 20.116
0.964 91.361 1.178 41.235 0.961 83.724
ENSMUSG00000024846 Cst6 protein_coding 0.899 79.277 0.816
95.708 1.357 81.492 0.783 91.372
ENSMUSG00000024847 Aip protein_coding 0.844 0.987 NA NA
1.195 7.24 1.063 71.305
ENSMUSG00000024851 Pitpnm1 protein_coding 0.823 0.987 1.01
92.561 1.2 8.466 0.983 84.29
ENSMUSG00000024853 Sf3b2 protein_coding 0.948 0.987 0.997 94.597
1.061 6.069 1.029 52.324
ENSMUSG00000024854 Pold4 protein_coding 0.936 54.043 0.986
88.705 1.084 48.451 0.964 89.892
ENSMUSG00000024855 Pacs1 protein_coding 1.116 13.734 0.979
96.847 0.916 1.342 0.912 83.724
ENSMUSG00000024856 Cdk2ap2 protein_coding 0.872 6.155 1.111
79.29 1.033 43.185 0.984 85.493
ENSMUSG00000024857 Cabp2 protein_coding 1 71.915 NA NA
1 80.634 1 93.156
ENSMUSG00000024858 Adrbk1 protein_coding 1.017 72.85 0.995
97.155 0.984 80.749 0.992 89.525
ENSMUSG00000024862 Klc2 protein_coding 0.79 0.987 1.126 60.309
1.125 12.831 1.071 79.442
ENSMUSG00000024863 Mbl2 protein_coding 1 81.925 1
96.68 1 87.686 1 92.381
ENSMUSG00000024866 Acy3 protein_coding 0.936 35.646 1.036

96.897 1.032 63.665 0.888 83.724
ENSMUSG00000024867 Pip5k1b protein_coding 1.26 47.174
0.966 96.335 0.822 16.949 0.866 84.29
ENSMUSG00000024869 Nudt8 protein_coding 0.829 1.815 1.035 96.68
1.165 7.024 0.969 87.651
ENSMUSG00000024870 Rab1b protein_coding 0.88 0.987 1.067 63.504
1.066 43.481 1.035 93.261
ENSMUSG00000024871 Doc2g protein_coding 0.854 30.035 1.03
84.858 1.134 23.668 0.963 95.273
ENSMUSG00000024873 Cnih2 protein_coding 1.067 66.897 0.898
95.431 1.046 81.388 0.972 96.183
ENSMUSG00000024875 Yif1a protein_coding 0.965 61.633 1.018
88.923 1.019 78.713 1.049 67.169
ENSMUSG00000024878 Cbwd1 protein_coding 1.045 49.202 1.037
92.561 0.922 48.387 0.941 86.699
ENSMUSG00000024883 Rin1 protein_coding 0.763 5.491 1.167 79.864
1.124 18.797 1.045 93.261
ENSMUSG00000024885 Aldh3b1 protein_coding 1.283 0.767 0.842
13.578 0.927 67.962 1.11 66.068
ENSMUSG00000024887 Asah2 protein_coding 1.262 0 0.963 78.471
0.824 0.879 0.924 83.724
ENSMUSG00000024889 Rce1 protein_coding 0.903 10.739 1.012
96.335 1.096 44.515 1.029 94.297
ENSMUSG00000024891 Slc29a2 protein_coding 0.935 78.538 1.07
84.832 0.997 80.015 1.153 60.31
ENSMUSG00000024892 Pcx protein_coding 0.506 0.987 1.113 92.062
1.77 5.864 0.922 95.559
ENSMUSG00000024896 Minpp1 protein_coding 1.085 10.19 1.043
95.431 0.885 10.23 1.041 46.412
ENSMUSG00000024897 Apba1 protein_coding 1.346 0.664 0.905 72.712
0.82 30.827 0.954 96.071
ENSMUSG00000024899 Papss2 protein_coding 1.165 34.861
0.827 90.378 1.033 72.269 0.94 95.92
ENSMUSG00000024900 Cpt1a protein_coding 1.121 7.842 0.861 37.376
1.036 86.144 0.989 90.289
ENSMUSG00000024901 Peli3 protein_coding 1.19 1.913 NA NA
0.848 3.654 0.898 84.29
ENSMUSG00000024902 Mrpl11 protein_coding 0.949 14.526
0.968 95.088 1.087 21.462 0.99 95.676
ENSMUSG00000024903 Lao1 protein_coding 0.988 56.242 NA NA
1.093 49.601 1.161 78.948
ENSMUSG00000024905 Mtl5 protein_coding 1.454 14.966 0.631
79.475 1.089 69.445 1.075 95.74
ENSMUSG00000024906 Mus81 protein_coding 0.914 14.645 0.99
97.263 1.102 15.945 0.997 88.136
ENSMUSG00000024908 Ppp6r3 protein_coding 1.049 10.105
0.974 75.052 0.979 33.137 1.016 83.067
ENSMUSG00000024909 Efemp2 protein_coding 1.237 4.362 1.056
92.253 0.768 1.48 1.098 35.053
ENSMUSG00000024910 Ctsw protein_coding 1.046 85.499 1.574
60.309 0.608 19.449 0.601 84.29
ENSMUSG00000024911 Fibp protein_coding 0.988 79.433 0.969
91.361 1.045 55.81 0.988 96.183
ENSMUSG00000024912 Fosl1 protein_coding 0.783 40.217 1.098

96.013 1.16 54.673 0.797 85.493
ENSMUSG00000024913 Lrp5 protein_coding 0.931 28.804 1.072
85.756 1.003 73.428 0.996 92.018
ENSMUSG00000024914 Drap1 protein_coding 0.823 2.668 1.112 79.055
1.095 53.882 0.995 96.183
ENSMUSG00000024921 Smarca2 protein_coding 0.964 52.9 0.967
96.013 1.072 50.851 1.116 49.806
ENSMUSG00000024922 Ovol1 protein_coding 0.923 45.894 1.071
96.843 1.011 70.1 0.954 96.192
ENSMUSG00000024924 Vldlr protein_coding 1.11 18.388 1.097
96.407 0.824 9.288 0.989 95.777
ENSMUSG00000024925 Rnaseh2c protein_coding 0.85 57.544
1.129 91.776 1.038 71.839 1.036 94.904
ENSMUSG00000024926 Kat5 protein_coding 0.995 82.891 0.961
85.116 1.046 42.065 0.991 90.611
ENSMUSG00000024927 Rela protein_coding 0.991 43.816 1
96.68 1.009 55.512 0.966 91.105
ENSMUSG00000024935 Slc1a1 protein_coding 1.023 51.737
1.016 91.532 0.964 73.874 0.899 92.466
ENSMUSG00000024936 Kcnk7 protein_coding 1.562 56.907 0.665
81.284 0.962 87.103 0.486 83.724
ENSMUSG00000024937 Ehbp1l1 protein_coding 1.13 33.854
1.101 85.501 0.801 7.339 1.033 83.067
ENSMUSG00000024939 Fam89b protein_coding 1 62.689 1
92.404 1 75.967 1 94.016
ENSMUSG00000024940 Ltbp3 protein_coding 0.932 34.305 1.036
87.604 1.032 63.665 1.045 88.512
ENSMUSG00000024941 Scyl1 protein_coding 0.943 7.574 1.058 81.127
1.004 78.864 1.02 87.134
ENSMUSG00000024942 Capn1 protein_coding 0.964 38.195 0.986
90.138 1.053 28.055 1.118 60.31
ENSMUSG00000024943 Smc5 protein_coding 1.053 21.539 0.94
33.865 1.008 56.497 1.031 81.061
ENSMUSG00000024944 Arl2 protein_coding 0.858 3.07 1.108 82.904
1.053 55.109 0.995 95.273
ENSMUSG00000024947 Men1 protein_coding 0.952 14.849 1.014
81.039 1.036 64.305 1.017 91.872
ENSMUSG00000024948 Map4k2 protein_coding 0.859 0.987 1.084
83.104 1.071 15.241 0.946 92.181
ENSMUSG00000024949 Sf1 protein_coding 0.955 12.309 NA NA
1.035 16.808 0.986 86.699
ENSMUSG00000024952 Rps6ka4 protein_coding 1.002 81.823
1.055 71.617 0.947 23.1 1.03 58.557
ENSMUSG00000024953 Prdx5 protein_coding 0.818 1.698 1.051 96.216
1.166 8.007 1.028 94.654
ENSMUSG00000024955 Esrra protein_coding 0.838 7.887 1.227 71.617
0.973 86.985 1.087 78.379
ENSMUSG00000024957 Kcnk4 protein_coding 0.711 37.82 1.013 95.708
1.39 34.807 1.052 88.797
ENSMUSG00000024958 Gpr137 protein_coding 0.821 1.175 1.062
72.546 1.149 22.517 0.956 85.493
ENSMUSG00000024959 Bad protein_coding 0.886 11.519 1.034
81.127 1.092 30.292 1.002 95.72
ENSMUSG00000024960 Plcb3 protein_coding 0.938 13.952 1.028

90.116 1.038 33.042 1 88.505
ENSMUSG00000024962 Vegfb protein_coding 0.842 1.239 1.148 63.504
1.035 42.065 0.948 88.387
ENSMUSG00000024963 Dnajc4 protein_coding 0.942 10.704
1.022 90.116 1.037 62.307 0.972 95.74
ENSMUSG00000024965 Fermt3 protein_coding 1.463 0 0.855
58.277 0.8 21.431 1.058 74.193
ENSMUSG00000024966 Stip1 protein_coding 0.818 14.427 1.363
82.904 0.896 72.853 1.071 48.119
ENSMUSG00000024968 Rcor2 protein_coding 1.135 58.896 1.144
82.904 0.773 4.23 1.085 57.075
ENSMUSG00000024969 Mark2 protein_coding 0.925 4.115 1.087 60.309
0.996 88.729 1.018 89.108
ENSMUSG00000024970 AI846148 protein_coding 0.909 2.174 1.074
86.277 1.026 39.471 0.973 91.625
ENSMUSG00000024972 Lgals12 protein_coding 0.866 3.009 1.09
64.304 1.058 58.616 0.912 83.724
ENSMUSG00000024973 Hrasls5 protein_coding 0.889 61.043
1.102 96.407 1.02 73.428 0.955 96.192
ENSMUSG00000024974 Smc3 protein_coding 1.008 71.06 0.968 94.597
1.025 85.134 1.07 35.053
ENSMUSG00000024975 Pdcd4 protein_coding 1.219 0.355 0.953 84.017
0.863 2.563 1.06 73.823
ENSMUSG00000024976 Shoc2 protein_coding 1.086 6.76 1.016 90.952
0.904 6.858 1.026 90.455
ENSMUSG00000024978 Gpam protein_coding 0.978 66.652 1.197
80.14 0.852 67.962 0.922 96.183
ENSMUSG00000024979 Tectb protein_coding 1.304 20.799 1.09
95.809 0.703 18.539 0.839 84.29
ENSMUSG00000024981 Acsl5 protein_coding 1.102 0.355 0.91 63.912
0.996 27.994 1.046 54.265
ENSMUSG00000024982 Zdhhc6 protein_coding 1.011 62.354
0.995 91.361 0.993 67.339 0.996 90.611
ENSMUSG00000024983 Vti1a protein_coding 0.981 65.388 1.026
92.111 NA NA 1.04 83.067
ENSMUSG00000024985 Tcf7l2 protein_coding 1.094 14.205 NA
NA 0.877 10.362 0.889 83.724
ENSMUSG00000024986 Hhex protein_coding 0.877 71.674 1.124
87.282 1.015 69.974 0.972 93.132
ENSMUSG00000024987 Cyp26a1 protein_coding 0.856 84.534
0.782 95.708 1.492 84.961 0.967 86.991
ENSMUSG00000024989 Cep55 protein_coding 1.391 12.549 0.69
40.463 1.045 85.921 1.452 63.708
ENSMUSG00000024990 Rbp4 protein_coding 0.498 0.987 1.007 90.895
1.984 5.864 1.128 94.654
ENSMUSG00000024991 Eif3a protein_coding 1.009 76.827 0.926
27.551 1.066 24.383 1.045 72.826
ENSMUSG00000024993 Fam45a protein_coding 0.988 83.169
1.057 86.156 0.961 58.516 1.033 87.088
ENSMUSG00000024997 Prdx3 protein_coding 0.959 51.056 1.05
95.211 0.99 84.626 0.989 87.218
ENSMUSG00000024998 Plce1 protein_coding 0.834 8.45 1.144 77.598
1.046 57.227 1.107 53.923
ENSMUSG00000024999 Noc3l protein_coding 1.054 33.325 NA NA

0.872 44.019 1.071 83.067
ENSMUSG00000025001 Hells protein_coding 1.006 79.577 0.961
87.452 1.032 84.308 1.587 31.133
ENSMUSG00000025003 Cyp2c39 protein_coding 1 86.74 NA NA
1 88.693 1 93.457
ENSMUSG00000025004 Cyp2c40 protein_coding 1 72.27 NA NA
1 86.985 1 95.273
ENSMUSG00000025006 Sorbs1 protein_coding 0.997 81.823
1.021 89.434 0.983 85.58 0.979 94.904
ENSMUSG00000025007 Aldh18a1 protein_coding 1.058 52.429
0.989 96.216 0.957 63.919 1.119 50.452
ENSMUSG00000025008 Tctn3 protein_coding 1.037 75.196 1.049
92.561 0.916 35.264 0.977 95.676
ENSMUSG00000025010 Ccnj protein_coding 0.955 45.728 0.996
95.35 1.048 51.851 1.001 83.067
ENSMUSG00000025016 Tm9sf3 protein_coding 1.114 2.723 0.942
78.471 0.955 8.142 0.994 94.99
ENSMUSG00000025017 Pik3ap1 protein_coding 1.548 0 0.954
91.361 0.677 2.563 1.085 90.177
ENSMUSG00000025019 Lcor protein_coding 0.554 1.815 0.962 95.708
1.878 7.24 1.051 94.654
ENSMUSG00000025020 Slit1 protein_coding 1.103 80.251 0.596
43.505 1.514 15.945 0.916 91.625
ENSMUSG00000025024 Smndc1 protein_coding 1.054 27.731 0.98
93.654 0.965 57.446 1.063 52.366
ENSMUSG00000025025 Mxi1 protein_coding 1.028 56.907 0.921
68.115 1.057 49.898 1.003 90.611
ENSMUSG00000025026 Add3 protein_coding 1.071 0.577 0.995 86.538
0.934 20.751 1.024 65.401
ENSMUSG00000025027 Xpnpep1 protein_coding 0.924 3.641 NA NA
1.051 12.831 1.114 31.133
ENSMUSG00000025034 Trim8 protein_coding 1.06 84.564 1.076
74.663 0.879 44.019 0.958 95.273
ENSMUSG00000025035 Arl3 protein_coding 0.985 61.334 1.058
71.617 0.957 39.079 1.063 57.711
ENSMUSG00000025036 Sfxn2 protein_coding 0.978 77.182 1.249
79.055 0.816 10.23 0.968 87.735
ENSMUSG00000025037 Maoa protein_coding 1.041 79.853 1.031
82.904 0.931 15.376 1.072 36.988
ENSMUSG00000025038 Efhc2 protein_coding 0.897 85.499 1.222
94.597 0.911 64.305 0.879 94.608
ENSMUSG00000025040 Fundc1 protein_coding 1.135 2.085 0.988
96.847 0.892 1.342 0.942 93.584
ENSMUSG00000025041 Nt5c2 protein_coding 1.093 17.191 0.993
91.897 0.919 21.331 1.034 74.193
ENSMUSG00000025043 Dusp21 protein_coding 1 81.657 1
96.268 1 86.727 1 95.832
ENSMUSG00000025044 Msr1 protein_coding 1.37 1.846 0.945 75.493
0.771 27.194 1.568 56.769
ENSMUSG00000025047 Pdcd11 protein_coding 0.952 52.9 0.982
79.894 1.065 43.586 1.061 64.513
ENSMUSG00000025049 Taf5 protein_coding 1 80.251 1.028
96.407 NA NA 1.021 96.248
ENSMUSG00000025050 Pcgf6 protein_coding 0.937 34.795 0.937

87.857 1.142 10.281 0.95 89.892
ENSMUSG00000025051 Samt4 protein_coding 1 78.428 1
96.407 1 85.757 1 95.92
ENSMUSG00000025056 Nr0b1 protein_coding 1 84.564 1
91.065 1 64.778 1 94.001
ENSMUSG00000025058 5430427O19Rik protein_coding 1.272 37.702
0.963 95.088 0.814 49.217 1.115 93.261
ENSMUSG00000025059 Gyk protein_coding 1.105 33.555 1.074
93.529 0.839 16.129 1.126 95.92
ENSMUSG00000025060 Slk protein_coding 1.037 69.489 0.945
56.781 1.023 62.307 0.975 94.468
ENSMUSG00000025064 Col17a1 protein_coding 0.88 3.07 1.164
66.01 0.976 77.704 0.946 83.724
ENSMUSG00000025066 Sfr1 protein_coding 1.031 18.635 1.013
95.504 0.96 27.994 1.074 32.698
ENSMUSG00000025068 Gsto1 protein_coding 1.071 46.097 0.93
89.277 1.002 84.552 0.883 83.952
ENSMUSG00000025069 Gsto2 protein_coding 1.196 19.477 0.857
87.147 0.976 62.058 0.903 86.991
ENSMUSG00000025075 Habp2 protein_coding 2.772 29.057 1.135
94.767 0.316 9.456 0.633 96.183
ENSMUSG00000025076 Casp7 protein_coding 1.117 2.897 NA NA
0.867 3.432 1.055 92.748
ENSMUSG00000025077 Dclre1a protein_coding 0.91 16.325
1.049 89.996 1.049 44.361 1.052 65.558
ENSMUSG00000025078 Nhlrc2 protein_coding 1.01 76.827
0.954 65.165 1.039 51.596 1.04 95.273
ENSMUSG00000025082 Vwa2 protein_coding 0.884 81.24 1.01 93.654
1.117 82.436 1.368 90.674
ENSMUSG00000025083 Afap1l2 protein_coding 1.09 84.049 1.07
85.378 0.855 67.029 0.971 96.071
ENSMUSG00000025085 Ablim1 protein_coding 1.018 74.147
0.969 68.818 1.01 68.425 0.956 94.19
ENSMUSG00000025086 Trub1 protein_coding 0.952 65.199 0.995
79.475 1.056 42.75 0.96 83.724
ENSMUSG00000025089 Gfra1 protein_coding 1.06 41.951 1.23
75.21 0.763 8.382 1.011 94.904
ENSMUSG00000025090 Ccdc172 protein_coding 1 74.958 NA
NA 1 85.792 1 95.273
ENSMUSG00000025091 Pnliprp2 protein_coding 1 76.968 1
89.434 1 88.161 1 93.156
ENSMUSG00000025092 Hspa12a protein_coding 0.878 36.945
1.218 60.309 0.935 58.271 1.102 71.305
ENSMUSG00000025094 Slc18a2 protein_coding 1.7 0.2 0.766
81.284 0.769 10.003 0.899 90.157
ENSMUSG00000025102 3110040N11Rik protein_coding 0.925 37.531
1.01 85.671 1.073 27.475 0.976 87.705
ENSMUSG00000025103 Btbd1 protein_coding 1.103 0.2 0.975 85.116
0.929 0.879 1.036 93.734
ENSMUSG00000025104 Hdgfrp3 protein_coding 1.146 1.161 0.884
75.345 0.983 24.523 0.991 90.611
ENSMUSG00000025105 Bnc1 protein_coding 1.799 13.492 0.701
62.025 0.795 68.266 0.885 86.991
ENSMUSG00000025127 Gcgr protein_coding 0.854 50.229 NA NA

1.044 71.839 1.001 94.384
ENSMUSG00000025128 Bhlhe22 protein_coding 1 76.827
1.434 94.767 0.695 58.516 1.31 75.198
ENSMUSG00000025129 Ppp1r27 protein_coding 3.793 3.932 2.008
91.643 0.131 6.858 0.918 89.498
ENSMUSG00000025130 P4hb protein_coding 1.129 8.365 0.979 92.631
0.907 13.643 1.056 31.133
ENSMUSG00000025132 Arhgdia protein_coding 0.953 5.946 1.038
79.29 1.005 83.857 1.078 65.558
ENSMUSG00000025133 Ints4 protein_coding 1.006 85.77 0.965 87.857
1.033 58.051 0.983 95.92
ENSMUSG00000025134 Alyref protein_coding 0.975 84.865
1.023 85.378 0.999 47.12 0.992 96.358
ENSMUSG00000025135 Anapc11 protein_coding 0.987 84.865
0.987 87.452 1.025 81.032 0.976 90.854
ENSMUSG00000025137 Pcyt2 protein_coding 0.827 2.436 1.147 82.809
1.055 24.985 0.949 93.584
ENSMUSG00000025138 Sirt7 protein_coding 0.895 1.343 1.041 95.211
1.067 9.567 0.971 91.105
ENSMUSG00000025139 Tollip protein_coding 1.077 20.032
0.995 96.897 0.937 13.202 0.983 87.735
ENSMUSG00000025140 Pycr1 protein_coding 1.171 51.737 0.973
96.843 0.879 48.133 1.358 37.634
ENSMUSG00000025141 Myadml2 protein_coding 6.952 2.998 3.377
79.411 0.043 0.72 0.734 96.086
ENSMUSG00000025142 Aspscr1 protein_coding 0.861 1.239 1.028
85.756 1.131 7.024 1.02 90.177
ENSMUSG00000025144 Stra13 protein_coding 0.948 38.286
1.006 97.263 1.05 55.109 1.074 76.831
ENSMUSG00000025145 Lrrc45 protein_coding 0.809 0.987 1.004
95.708 1.228 5.864 0.983 90.289
ENSMUSG00000025147 Mob2 protein_coding 0.954 14.221 NA NA
1.068 47.776 0.963 84.29
ENSMUSG00000025150 Cbr2 protein_coding 1.565 0 0.9 83.58
0.705 6.935 1.296 40.894
ENSMUSG00000025151 Maged1 protein_coding 0.901 30.035
1.146 68.96 0.97 59.726 1.213 46.846
ENSMUSG00000025153 Fasn protein_coding 0.306 0.987 1.187 83.819
2.76 6.202 0.886 95.832
ENSMUSG00000025154 Arhgap19 protein_coding 1.109 40.065
1.003 90.138 0.899 71.999 0.832 92.12
ENSMUSG00000025155 Dus1l protein_coding 0.975 41.617 1.037
72.642 0.984 87.744 1.018 94.904
ENSMUSG00000025156 Gps1 protein_coding 0.934 8.181 1.081 79.055
0.991 85.352 1.059 72.369
ENSMUSG00000025157 Zdhhc16 protein_coding 0.957 54.983
1.053 81.127 0.994 60.233 1.025 83.067
ENSMUSG00000025158 Rfng protein_coding 0.968 35.894 1.059
63.504 0.976 22.864 0.968 86.699
ENSMUSG00000025159 Mms19 protein_coding 0.983 81.925 NA NA
1.046 82.74 1.006 86.991
ENSMUSG00000025161 Slc16a3 protein_coding 2.285 2.655 0.86
97.155 0.507 0.72 1.557 86.209
ENSMUSG00000025162 Csnk1d protein_coding 0.945 2.009 1.003

94.102 1.056 6.202 0.975 94.001
ENSMUSG00000025163 Cd7 protein_coding 0.848 68.846 1.776
60.309 0.665 11.997 0.779 83.724
ENSMUSG00000025165 Sectm1a protein_coding 1.647 16.74 1.234
96.948 0.493 12.907 1.597 42.736
ENSMUSG00000025169 Ogfod3 protein_coding 0.963 60.339
1.064 95.431 0.977 87.173 0.991 95.877
ENSMUSG00000025170 Rab40b protein_coding 1.233 3.644 0.834
90.053 0.974 61.743 1.004 92.018
ENSMUSG00000025171 Ubtd1 protein_coding 0.888 18.795 1.187
71.617 0.949 79.524 1.102 83.067
ENSMUSG00000025172 Ankrd2 protein_coding 1.028 72.655
1.563 97.288 0.622 59.383 1.11 87.134
ENSMUSG00000025173 Wdr45b protein_coding 1.146 2.361 0.946
86.604 0.926 8.313 0.996 94.904
ENSMUSG00000025175 Fn3k protein_coding 1.007 86.843 1.117
90.454 0.887 69.445 0.921 85.493
ENSMUSG00000025176 Hoga1 protein_coding 0.996 86.199 0.9
90.576 1.117 55.512 1.22 50.74
ENSMUSG00000025178 Pi4k2a protein_coding 1.004 85.355
1.007 97.263 0.986 84.229 0.977 91.372
ENSMUSG00000025184 R3hcc1l protein_coding 1.023 58.198
1.009 97.263 0.969 63.504 1.06 71.653
ENSMUSG00000025185 Loxl4 protein_coding 1.332 8.281 0.996 92.631
0.751 22.009 0.978 96.183
ENSMUSG00000025188 Hps1 protein_coding 1.004 75.858 0.954
80.052 1.042 38.683 0.982 91.078
ENSMUSG00000025189 Cnnm1 protein_coding 0.704 41.021 0.857
85.075 1.653 26.978 0.621 90.611
ENSMUSG00000025190 Got1 protein_coding 1.534 0 1.018 87.322
0.638 0.72 1.026 71.008
ENSMUSG00000025192 Entpd7 protein_coding 0.999 80.03 1.201
71.617 0.831 6.858 1.109 60.968
ENSMUSG00000025193 Cutc protein_coding 1.05 71.516 0.849
65.165 1.12 40.875 0.962 96.183
ENSMUSG00000025194 Abcc2 protein_coding 0.465 0.987 0.705 95.504
3.059 16.808 0.861 96.086
ENSMUSG00000025195 Dnmbp protein_coding 1.052 8.281 0.93 54.427
1.02 81.492 0.956 86.991
ENSMUSG00000025196 Cpn1 protein_coding 1.779 47.174 0.49
75.493 1.146 84.504 1.056 94.654
ENSMUSG00000025197 Cyp2c44 protein_coding 1.327 44.694
0.676 75.345 1.113 75.527 0.797 84.29
ENSMUSG00000025198 Erlin1 protein_coding 1.162 14.34 0.909
56.955 NA NA 1.014 60.31
ENSMUSG00000025199 Chuk protein_coding 0.979 70.862 NA NA
1.003 78.498 0.989 93.261
ENSMUSG00000025200 Cwf19l1 protein_coding 0.94 13.199
1.069 71.617 0.992 88.729 1.009 85.577
ENSMUSG00000025201 Bloc1s2-ps pseudogene 1 85.637 1
93.469 1 84.83 1 83.067
ENSMUSG00000025202 Scd3 protein_coding 0.483 18.907 0.931
91.361 2.219 12.831 1.212 95.74
ENSMUSG00000025203 Scd2 protein_coding 0.707 9.863 1.467 86.156

0.966 56.497 1.165 83.067
ENSMUSG00000025204 Ndufb8 protein_coding 0.785 2.009 1.072
86.277 1.189 20.315 1.024 90.674
ENSMUSG00000025207 Sema4g protein_coding 0.874 35.894
0.957 88.345 1.192 21.201 0.936 86.991
ENSMUSG00000025208 Mrpl43 protein_coding 0.953 19.595 1.01
97.263 1.041 38.099 1.036 75.81
ENSMUSG00000025209 Peo1 protein_coding 0.909 58.56 1 95.35
1.099 43.185 1.087 60.466
ENSMUSG00000025212 Sfxn3 protein_coding 1.01 85.728 0.989
90.287 1.003 80.089 0.951 84.29
ENSMUSG00000025213 Kazald1 protein_coding 1.047 64.401
0.846 85.116 1.127 86.602 0.889 85.493
ENSMUSG00000025215 Tlx1 protein_coding 1 79.93 1 89.014
1 52.087 1 83.724
ENSMUSG00000025216 Lbx1 protein_coding 3.473 2.569 1.322 83.104
0.217 0.72 0.515 92.742
ENSMUSG00000025217 Btrc protein_coding 1.076 33.854 0.922
72.399 1.005 85.352 1.025 89.498
ENSMUSG00000025218 Poll protein_coding 0.97 58.264 0.94
91.83 1.094 33.787 1.024 90.463
ENSMUSG00000025219 Fgf8 protein_coding 1 79.93 1 92.062
1 88.41 1 93.132
ENSMUSG00000025220 Mgea5 protein_coding 0.939 30.136 0.995
79.807 1.071 15.602 1.012 96.358
ENSMUSG00000025221 Kcnip2 protein_coding 0.883 45.502
0.904 70.693 1.253 18.797 0.698 83.952
ENSMUSG00000025223 Ldb1 protein_coding 0.973 58.802 1.036
97.155 0.991 80.381 0.882 83.724
ENSMUSG00000025224 Gbf1 protein_coding 0.981 30.035 1.056
79.864 0.96 85.21 1.041 53.923
ENSMUSG00000025225 Nfkb2 protein_coding 0.954 44.252 1.08
79.367 0.973 86.084 0.916 83.724
ENSMUSG00000025226 Fbxl15 protein_coding 0.986 86.145
1.008 97.155 1.003 83.857 0.938 92.31
ENSMUSG00000025227 Tmem180 protein_coding 0.874 40.422
1.048 95.708 1.092 40.875 0.827 83.724
ENSMUSG00000025228 Actr1a protein_coding 0.92 0.987 1.021
93.146 1.063 7.482 1.047 31.133
ENSMUSG00000025229 Pitx3 protein_coding 1.498 66.944 0.724
95.211 NA NA 0.901 90.865
ENSMUSG00000025231 Sufu protein_coding 0.982 65.869 1.048
84.999 0.969 87.336 1 96.183
ENSMUSG00000025232 Hexa protein_coding 1.206 9.695 0.903 30.872
0.916 52.087 0.944 92.675
ENSMUSG00000025234 Arih1 protein_coding 1.056 0.976 0.974 75.052
0.973 14.262 0.968 88.245
ENSMUSG00000025235 Bbs4 protein_coding 0.984 85.728 1.097
83.104 0.929 15.63 1.068 83.067
ENSMUSG00000025236 Adpgk protein_coding 0.966 59.028 NA NA
0.987 73.309 1.052 46.846
ENSMUSG00000025237 Parp6 protein_coding 1.005 83.169 0.95
74.633 1.051 52.432 0.915 83.724
ENSMUSG00000025239 Limd1 protein_coding 1.151 0 1.041 85.756

0.832 0.72 1.013 93.117
ENSMUSG00000025240 Sacm1l protein_coding 1.051 17.492
1.014 93.899 0.941 5.628 0.983 85.493
ENSMUSG00000025241 Fyco1 protein_coding 0.969 60.003 1.058
83.104 0.977 80.5 1.007 84.31
ENSMUSG00000025243 Slc6a20b protein_coding 1 31.921 1
91.643 1 70.9 1 95.74
ENSMUSG00000025245 Lztfl1 protein_coding 0.873 7.574 0.957
95.088 1.2 10.76 1.018 78.379
ENSMUSG00000025246 Tbl1x protein_coding 1.064 20.032 0.99
88.705 0.951 49.733 0.985 92.181
ENSMUSG00000025255 Zfhx4 protein_coding 0.96 68.353 1.083
92.561 0.961 86.985 1.007 96.192
ENSMUSG00000025257 Ribc1 protein_coding 0.842 15.76 0.935 83.733
1.268 12.703 0.788 83.724
ENSMUSG00000025260 Hsd17b10 protein_coding 0.963 27.836
0.831 85.116 1.25 30.213 0.899 83.724
ENSMUSG00000025261 Huwe1 protein_coding 0.993 32.168 0.93
90.046 1.078 20.968 0.974 85.493
ENSMUSG00000025262 Fam120c protein_coding 0.871 0.987 0.926
92.355 1.243 7.635 0.925 84.29
ENSMUSG00000025264 Tsr2 protein_coding 1.062 75.196 0.977
90.053 0.966 88.161 1.018 86.445
ENSMUSG00000025265 Fgd1 protein_coding 1.007 54.344 0.979
94.48 1.015 65.688 1.007 86.991
ENSMUSG00000025266 Gnl3l protein_coding 1.024 51.737 1.029
94.848 0.946 80.749 1.045 52.982
ENSMUSG00000025268 Maged2 protein_coding 1.091 67.374
0.918 94.597 0.996 62.533 1.284 31.133
ENSMUSG00000025269 Apex2 protein_coding 1.017 74.482 1.1
82.904 0.895 13.848 1.033 91.105
ENSMUSG00000025270 Alas2 protein_coding 1.115 76.546 0.781
59.946 1.143 38.152 1.27 86.209
ENSMUSG00000025271 Pfkfb1 protein_coding 0.869 5.591 1.083
60.309 1.059 84.229 0.908 86.699
ENSMUSG00000025272 Tro protein_coding 0.977 83.418 0.977
87.313 1.05 44.125 1.058 83.067
ENSMUSG00000025277 Abhd6 protein_coding 1.181 15.662 0.947
92.793 0.89 6.858 0.895 83.724
ENSMUSG00000025278 Flnb protein_coding 0.9 10.014 1.006
96.216 1.1 16.26 1.037 86.133
ENSMUSG00000025279 Dnase1l3 protein_coding 1.986 9.695 1.146
88.923 0.439 4.935 0.723 83.724
ENSMUSG00000025280 Polr3a protein_coding 0.98 49.944
0.966 95.329 1.05 45.208 1.051 83.067
ENSMUSG00000025283 Sat1 protein_coding 1.231 0.435 0.874 26.151
0.927 80.015 1.03 90.125
ENSMUSG00000025287 Acot9 protein_coding 1.032 76.827 0.996
94.102 0.973 88.203 1.022 92.564
ENSMUSG00000025288 4933436I01Rik protein_coding 1 75.115
1 95.504 1 87.971 1 96.086
ENSMUSG00000025289 Prdx4 protein_coding 1.104 6.958 0.993 85.921
0.914 18.965 1.179 46.412
ENSMUSG00000025290 Rps24 protein_coding 1.077 33.325 0.91

56.955	1.022	80.899	1.085	72.616
ENSMUSG00000025314	Ptprj	protein_coding	1.318	3.598	0.901	62.692
0.843	37.103	0.931	94.468
ENSMUSG00000025316	Banp	protein_coding	1.283	19.315	1.272
87.609	0.609	15.904	1.154	83.067
ENSMUSG00000025318	Jph3	protein_coding	0.814	82.231	2.704
82.904	0.453	42.621	1.823	73.823
ENSMUSG00000025321	Itgb8	protein_coding	1.028	69.089	0.955
93.046	1.019	86.258	0.901	83.724
ENSMUSG00000025323	Sp4	protein_coding	1.038	51.737	1.01
97.263	0.95	63.075	0.953	84.19
ENSMUSG00000025324	Atp10a	protein_coding	0.864	54.983
1.036	92.793	1.12	88.349	1.287	52.324
ENSMUSG00000025326	Ube3a	protein_coding	0.945	24.817	0.956
86.728	1.108	23.41	1.003	96.183
ENSMUSG00000025328	Padi3	protein_coding	1.088	51.826	0.691
38.699	1.334	34.403	1.349	71.305
ENSMUSG00000025329	Padi1	protein_coding	0.766	29.725	0.868
90.256	1.508	25.425	0.776	84.19
ENSMUSG00000025330	Padi4	protein_coding	2.112	0	0.819	53.434
0.577	15.376	1.926	54.265
ENSMUSG00000025332	Kdm5c	protein_coding	0.956	29.972	1.009
85.756	1.037	83.719	1.057	31.133
ENSMUSG00000025337	Sbds	protein_coding	1.012	81.52	0.978	78.877
1.012	50.851	0.983	96.183
ENSMUSG00000025340	Rabgef1	protein_coding	1.032	36.195
0.995	90.895	0.974	14.079	1.034	86.133
ENSMUSG00000025347	Mettl7b	protein_coding	0.498	1.031	1.552
63.504	1.291	15.602	1.078	79.268
ENSMUSG00000025348	Itga7	protein_coding	0.825	4.115	1.118	75.62
1.081	73.428	1.027	93.964
ENSMUSG00000025350	Rdh5	protein_coding	0.955	74.299	1.1
93.899	NA	NA	0.902	91.105
ENSMUSG00000025351	Cd63	protein_coding	1.034	26.136	NA	NA
0.926	15.465	1.05	68.696
ENSMUSG00000025352	Gdf11	protein_coding	1.232	21.703	1.036
87.609	0.781	3.718	1.024	83.276
ENSMUSG00000025353	Ormdl2	protein_coding	0.992	65.106
1.064	90.29	0.946	87.519	0.944	86.699
ENSMUSG00000025354	Dnajc14	protein_coding	0.984	61.334
1.015	93.529	1.003	87.686	1.038	43.605
ENSMUSG00000025355	Mmp19	protein_coding	1.033	85.499	1.064
94.367	0.908	76.768	1.115	31.133
ENSMUSG00000025357	Dgka	protein_coding	1.079	27.32	0.895	65.819
1.037	78.498	0.95	84.29
ENSMUSG00000025358	Cdk2	protein_coding	0.943	35.646	0.955
96.013	1.109	59.612	1.017	94.99
ENSMUSG00000025359	Pmel	protein_coding	0.925	53.407	0.91
89.996	1.183	32.433	0.952	91.078
ENSMUSG00000025362	Rps26	protein_coding	1.164	2.233	0.895	8.462
0.956	41.154	0.971	93.612
ENSMUSG00000025364	Pa2g4	protein_coding	1.006	81.925	0.935
34.058	1.061	25.144	1.065	46.412
ENSMUSG00000025366	Esyt1	protein_coding	1.083	14.34	1.058	78.629

0.875 1.898 1.118 87.134
ENSMUSG00000025369 Smarcc2 protein_coding 0.957 65.106
1.018 93.529 1.026 78.498 0.935 85.493
ENSMUSG00000025370 Cdh9 protein_coding 1.241 6.541 1.035 90.952
0.778 3.863 0.96 95.593
ENSMUSG00000025371 Chmp6 protein_coding 0.915 17.839 0.996
97.049 1.096 32.173 1.025 86.445
ENSMUSG00000025372 Baiap2 protein_coding 1.088 66.58 0.901
78.471 NA NA 0.974 86.699
ENSMUSG00000025373 Rnf41 protein_coding 0.928 56.768 1.05
83.104 1.022 86.831 1.016 95.676
ENSMUSG00000025374 Nabp2 protein_coding 0.887 0.987 1.147 77.618
0.984 72.378 0.981 91.625
ENSMUSG00000025375 Aatk protein_coding 0.785 14.645 1.095
84.007 1.165 42.065 1.007 86.991
ENSMUSG00000025377 Enthd2 protein_coding 0.977 45.502
1.045 83.104 0.976 72.269 1.031 88.111
ENSMUSG00000025380 Fscn2 protein_coding 1.713 58.896 0.895
91.275 0.653 79.776 1.186 93.734
ENSMUSG00000025381 Cnpy2 protein_coding 0.951 45.502 1.018
95.196 1.03 77.17 1.031 95.74
ENSMUSG00000025383 Il23a protein_coding 0.707 85.946 1.48
92.793 0.952 78.642 1.133 95.35
ENSMUSG00000025384 2310003H01Rik protein_coding 1.198 4.874
1.029 95.211 0.808 0.945 0.926 84.29
ENSMUSG00000025386 Pde6g protein_coding 0.938 71.763 0.94
96.216 1.135 72.148 0.943 92.52
ENSMUSG00000025393 Atp5b protein_coding 0.885 0.987 1.09 72.546
1.034 45.667 1.059 79.442
ENSMUSG00000025395 Prim1 protein_coding 1.02 57.38 NA NA
0.908 59.922 1.229 57.075
ENSMUSG00000025396 Hsd17b6 protein_coding 1 72.139 1
97.155 1 75.527 1 91.525
ENSMUSG00000025400 Tac2 protein_coding 1 77.113 1
93.765 1 85.464 1 95.273
ENSMUSG00000025401 Myo1a protein_coding 1.111 84.661 0.639
73.461 1.4 38.038 1.224 88.197
ENSMUSG00000025402 Nab2 protein_coding 1.063 55.124 1.126
82.904 0.834 7.329 0.992 87.134
ENSMUSG00000025403 Shmt2 protein_coding 0.934 26.77 1 96.308
1.071 48.304 1.079 60.968
ENSMUSG00000025404 R3hdm2 protein_coding 0.956 44.787
1.017 95.431 1.025 67.823 0.996 91.291
ENSMUSG00000025405 Inhbc protein_coding 1 83.418 1
97.129 1 87.786 1 94.654
ENSMUSG00000025407 Gli1 protein_coding 0.979 85.857 1.328
64.304 0.771 6.858 0.976 93.156
ENSMUSG00000025408 Ddit3 protein_coding 1.041 78.054 1.119
89.494 0.861 23.212 1.012 91.269
ENSMUSG00000025409 Mbd6 protein_coding 0.9 10.514 1.09
71.617 1.017 62.648 1.018 96.183
ENSMUSG00000025410 Dctn2 protein_coding 0.905 0.987 0.993 95.504
1.112 5.864 1.021 83.581
ENSMUSG00000025413 Ttc4 protein_coding 0.984 58.802 0.997

96.948 1.021 78.864 0.997 92.762
ENSMUSG00000025417 Pip4k2c protein_coding 0.988 83.968
0.989 79.894 1.024 54.809 0.967 83.724
ENSMUSG00000025418 Bsnd protein_coding 1 85.283 1
92.545 1 69.728 1 95.593
ENSMUSG00000025420 Katnal2 protein_coding 0.862 33.906
0.995 85.116 1.168 18.208 0.974 86.991
ENSMUSG00000025421 Hdhd2 protein_coding 0.985 72.743 1.015
93.863 NA NA 0.973 85.493
ENSMUSG00000025422 Agap2 protein_coding 1.024 84.168 1.11
95.735 0.877 73.096 0.942 92.564
ENSMUSG00000025423 Pias2 protein_coding 1.042 24.147 1.024
88.91 0.933 15.778 0.95 83.724
ENSMUSG00000025425 St8sia5 protein_coding 1.449 30.282
1.698 78.235 0.406 3.718 0.855 96.358
ENSMUSG00000025427 Rnf165 protein_coding 1.113 46.097
1.118 84.832 0.802 17.258 1.253 57.075
ENSMUSG00000025428 Atp5a1 protein_coding 0.93 9.508 NA NA
1.079 35.894 1.067 71.305
ENSMUSG00000025429 Pstpip2 protein_coding 1.285 0.999 1.057
96.216 0.733 5.564 1.195 78.546
ENSMUSG00000025431 Crisp1 protein_coding 1 81.925 1
97.263 1 88.693 1 95.74
ENSMUSG00000025432 Avil protein_coding 1.134 53.228 1.096
96.138 0.803 81.388 1.04 95.877
ENSMUSG00000025436 Xrcc6bp1 protein_coding 1.022 70.741 NA
NA 0.917 87.519 0.972 96.358
ENSMUSG00000025437 Usp33 protein_coding 0.921 55.303 0.949
80.463 1.14 24.383 1.093 59.394
ENSMUSG00000025439 Clns1a protein_coding 1.046 85.728
1.005 97.288 0.951 84.896 1.152 32.045
ENSMUSG00000025450 Gm9752 processed_transcript 0.694 4.351
0.912 61.883 1.573 5.864 0.873 83.724
ENSMUSG00000025451 Paip1 protein_coding 1.007 47.466 1.062
91.643 0.935 16.409 1.023 95.273
ENSMUSG00000025453 Nnt protein_coding 1.014 67.163 1.072
92.062 0.918 56.625 1.058 56.079
ENSMUSG00000025461 Cd163l1 protein_coding 2.272 4.675 0.863
87.452 0.508 23.48 0.585 84.29
ENSMUSG00000025464 Paox protein_coding 1.072 44.061 1.01
96.216 0.922 38.534 0.958 92.466
ENSMUSG00000025465 Echs1 protein_coding 0.988 65.388 1.01
95.708 1.005 72.378 0.97 90.611
ENSMUSG00000025466 Fuom protein_coding 0.958 84.049 1.098
86.34 0.951 68.82 1.046 93.016
ENSMUSG00000025469 Msx3 protein_coding 1 68.846 1
93.899 1 83.857 1 96.086
ENSMUSG00000025470 Zfp511 protein_coding 0.951 31.333
1.082 93.899 0.969 61.171 0.974 96.248
ENSMUSG00000025473 Adam8 protein_coding 1.22 7.332 0.911 64.555
0.898 68.82 1.113 46.412
ENSMUSG00000025474 Tubgcp2 protein_coding 1.128 7.501 1.017
96.948 0.869 0.945 1.022 72.992
ENSMUSG00000025477 Inpp5a protein_coding 0.969 60.339

1.051 72.642	0.979 44.019	1.029 92.389
ENSMUSG00000025478	Dpysl4	protein_coding	1.212 57.655	0.79
68.818	1.045 78.498	0.862 96.183
ENSMUSG00000025479	Cyp2e1	protein_coding	0.998 49.047
0.879 95.211	1.135 38.975	0.697 85.493
ENSMUSG00000025481	Urah	protein_coding	0.998 47.847	0.861
62.025	1.165 43.481	0.757 84.19
ENSMUSG00000025484	Bet1l	protein_coding	1.011 77.291	1.036
83.104	0.952 23.212	1.003 93.734
ENSMUSG00000025485	Ric8	protein_coding	1.035 43.939	NA	NA
0.966 38.534	0.972 83.724
ENSMUSG00000025486	Sirt3	protein_coding	0.923 28.998	0.977
91.361	1.107 28.603	0.946 86.699
ENSMUSG00000025487	Psmd13	protein_coding	0.892 6.409 0.98
95.211	1.139 15.241	1.155 44.882
ENSMUSG00000025488	Cox8b	protein_coding	1.05	82.092	1.184
85.756	0.805 38.534	1.017 95.72
ENSMUSG00000025489	Ifitm5	protein_coding	0.948 52.018
1.258 83.104	0.838 87.786	1.017 91.011
ENSMUSG00000025491	Ifitm1	protein_coding	1.289 12.396
0.911 78.51 0.852 40.338	1.049 93.734
ENSMUSG00000025492	Ifitm3	protein_coding	1.296 0	1.092
96.68 0.706 6.858 1.152 55.841
ENSMUSG00000025494	Sigirr	protein_coding	0.9	8.267 0.918
85.075	1.207 10.76 0.886 83.724
ENSMUSG00000025495	Ptdss2	protein_coding	0.938 65.106	NA
NA	1.037 80.015	0.957 83.724
ENSMUSG00000025497	Cdhr5	protein_coding	0.506 23.923	1.093
93.863	1.805 27.431	1.498 76.831
ENSMUSG00000025498	Irf7	protein_coding	1.456 5.848 0.999 96.847
0.686 59.922	1.208 37.634
ENSMUSG00000025499	Hras	protein_coding	0.868 2.277 1.077 75.612
1.071 35.673	1.033 79.268
ENSMUSG00000025500	1600016N20Rik	protein_coding	0.92	25.551
NA	NA	1.146 40.955	0.784 83.724
ENSMUSG00000025503	Taldo1	protein_coding	0.675 1.031 1.04
91.643	1.425 5.864 1.045 92.018
ENSMUSG00000025504	Eps8l2	protein_coding	0.875 14.427
0.963 94.102	1.184 16.666	0.814 83.724
ENSMUSG00000025505	Tmem80	protein_coding	0.864 5.084 1.047
87.282	1.103 9.807 0.939 85.493
ENSMUSG00000025507	Lrdd	protein_coding	1.001 82.092	1.094
85.985	0.912 54.521	1.106 83.067
ENSMUSG00000025508	Rplp2	protein_coding	1.035 49.202	0.927
37.187	1.046 52.574	0.979 94.654
ENSMUSG00000025509	Pnpla2	protein_coding	0.864 8.938 1.001
96.843	1.152 33.287	1.011 96.183
ENSMUSG00000025510	Cd151	protein_coding	0.846 0.987 1.071 71.617
1.105 19.025	0.999 91.525
ENSMUSG00000025511	Tspan4	protein_coding	0.855 56.768
1.151 71.222	NA	NA	1.105 70.539
ENSMUSG00000025512	Chid1	protein_coding	0.935 4.468 0.986 92.914
1.087 8.339 0.994 86.133
ENSMUSG00000025515	Muc2	protein_coding	0.957 54.604	0.771

73.166	1.357	20.051	0.956	86.133
ENSMUSG00000025519	Tktl2	protein_coding	1	48.857	1
96.652	1	75.637	1	90.95
ENSMUSG00000025521	Tmem192	protein_coding	1.024	62.007
1.086	87.604	0.901	28.901	1.012	94.567
ENSMUSG00000025525	Apool	protein_coding	0.958	51.089	1.02
90.092	1.024	84.626	1.096	54.153
ENSMUSG00000025527	Satl1	protein_coding	1	85.857	1
95.35	1	79.554	1	96.248
ENSMUSG00000025528	2010106E10Rik	protein_coding	1	86.351
1	84.594	1	61.418	1	94.567
ENSMUSG00000025529	Zfp711	protein_coding	0.879	43.495
0.948	96.138	1.197	47.31	0.854	86.991
ENSMUSG00000025531	Chm	protein_coding	1.126	6.399	0.972	93.654
0.914	25.512	1.003	92.762
ENSMUSG00000025532	Crcp	protein_coding	1.044	49.868	1.038
90.29	0.924	39.245	1.044	92.762
ENSMUSG00000025533	Asl	protein_coding	1.08	27.545	0.941
71.98	0.984	86.552	0.952	89.892
ENSMUSG00000025534	Gusb	protein_coding	1.152	0.461	0.922	86.861
0.943	6.858	1.098	60.466
ENSMUSG00000025537	Phkg1	protein_coding	1.248	2.567	1.311	60.309
0.612	0.72	1.048	94.904
ENSMUSG00000025538	Sumf2	protein_coding	0.935	17.839	1.149
64.123	0.933	48.577	1.019	85.629
ENSMUSG00000025544	Tm9sf2	protein_coding	1.04	15.972	1
94.597	0.963	28.669	0.985	92.181
ENSMUSG00000025545	Clybl	protein_coding	0.853	3.688	1.116	83.104
1.05	38.152	0.955	96.248
ENSMUSG00000025551	Fgf14	protein_coding	1.408	32.873	0.924
90.046	0.766	37.906	0.702	83.724
ENSMUSG00000025555	Farp1	protein_coding	1.064	32.873	0.918
82.073	1.023	83.066	1.047	58.333
ENSMUSG00000025558	Dock9	protein_coding	1.076	72.27	0.77	85.418
1.205	70.328	0.89	83.724
ENSMUSG00000025571	Tnrc6c	protein_coding	1.029	62.574	NA
NA	0.995	57.091	0.92	83.724
ENSMUSG00000025572	Tmc6	protein_coding	0.848	2.009	0.997	72.576
1.18	5.864	0.977	95.593
ENSMUSG00000025574	Tk1	protein_coding	0.776	68.846	0.994
88.705	1.301	62.995	1.547	62.918
ENSMUSG00000025575	Cant1	protein_coding	1.025	32.411	1.006
96.216	0.966	23.86	1.053	91.872
ENSMUSG00000025576	Rbfox3	protein_coding	0.607	7.574	1.18
96.843	1.399	9.429	1.314	76.347
ENSMUSG00000025577	Cbx2	protein_coding	0.884	48.036	1.13
79.411	1	75.353	0.945	95.877
ENSMUSG00000025578	Cbx8	protein_coding	0.87	15.486	1.128
73.092	1.02	82.575	0.966	96.358
ENSMUSG00000025579	Gaa	protein_coding	1.058	42.555	0.96
91.532	0.986	41.662	0.955	88.505
ENSMUSG00000025580	Eif4a3	protein_coding	0.978	82.092
0.998	86.538	1.018	66.928	1.054	75.81
ENSMUSG00000025582	Nptx1	protein_coding	1.428	57.38	0.764	93.654

0.913 67.339 1.601 80.324
ENSMUSG00000025583 Rptor protein_coding 0.949 14.086 1.011
88.823 1.045 53.449 1.035 81.56
ENSMUSG00000025584 Pde8a protein_coding 1.056 30.984 1.012
96.216 0.932 16.051 0.979 93.132
ENSMUSG00000025586 Cpeb1 protein_coding 0.91 73.487 1.071
96.335 1.026 81.976 1.21 52.324
ENSMUSG00000025588 Nat1 protein_coding 0.98 56.311 0.966
91.897 1.053 86.985 0.833 83.724
ENSMUSG00000025591 Tma16 protein_coding 1.012 61.334 0.986
96.216 1.002 74.686 1.055 70.624
ENSMUSG00000025592 Dach2 protein_coding 1.435 37.941 0.751
72.399 NA NA 1.234 93.734
ENSMUSG00000025597 Klhl4 protein_coding 1.127 14.746 1.009
95.504 0.882 32.297 0.958 83.724
ENSMUSG00000025602 Zfp202 protein_coding 0.999 83.263
0.885 41.951 1.132 19.609 1.065 59.394
ENSMUSG00000025607 Copg2 protein_coding 1.037 21.278 1.001
86.861 0.962 56.365 1 95.273
ENSMUSG00000025608 Podxl protein_coding 0.843 47.174 0.996
92.474 1.186 38.975 1.099 90.674
ENSMUSG00000025609 Mkln1 protein_coding 0.995 77.113 0.965
61.883 1.038 14.131 0.988 93.722
ENSMUSG00000025610 Map3k7cl protein_coding 1.149 33.325
1.021 83.104 0.853 13.848 1.44 55.054
ENSMUSG00000025612 Bach1 protein_coding 1.068 34.696 0.98
78.471 0.954 87.898 0.994 95.559
ENSMUSG00000025613 Cct8 protein_coding 1.02 84.294 0.963
90.378 1.019 82.436 1.105 40.024
ENSMUSG00000025616 Usp16 protein_coding 1.052 82.352 1.004
95.504 0.948 88.693 1.017 72.992
ENSMUSG00000025626 Phf6 protein_coding 1.006 69.777 0.998
92.561 0.998 49.453 1.015 94.904
ENSMUSG00000025630 Hprt protein_coding 1.022 41.177 0.984
87.553 0.996 85.675 1.061 56.769
ENSMUSG00000025644 Gm7628 processed_transcript 0.549 20.369
1.131 97.129 1.61 25.966 1.118 95.405
ENSMUSG00000025645 Ccdc51 protein_coding 0.997 74.299
1.013 97.155 0.987 87.786 0.956 95.559
ENSMUSG00000025646 Atrip protein_coding 1.011 86.199 0.993
90.744 0.997 78.369 1.02 84.31
ENSMUSG00000025647 Shisa5 protein_coding 1.055 8.685 0.974
94.848 0.967 36.248 1.068 68.502
ENSMUSG00000025648 Pfkfb4 protein_coding 1.054 51.826
1.032 93.899 0.917 27.321 1.074 80.324
ENSMUSG00000025650 Col7a1 protein_coding 0.622 2.822 1.003
94.89 1.605 10.76 1.061 91.928
ENSMUSG00000025651 Uqcrc1 protein_coding 0.91 8.405 1.065
83.104 1.035 55.109 1.061 91.011
ENSMUSG00000025656 Arhgef9 protein_coding 1.16 36.438
0.933 96.138 0.92 41.662 0.93 87.496
ENSMUSG00000025658 Cnksr2 protein_coding 1.12 64.193
1.101 92.137 0.813 76.64 0.828 95.273
ENSMUSG00000025665 NA NA NA NA 0.791 74.851 1.426

46.206	0.988 94.468
ENSMUSG00000025666	Tmem47	protein_coding	1.194 0	0.956
85.242	0.876 1.898 0.966 84.29
ENSMUSG00000025701	Alox5 protein_coding	1.275 3.932 0.946 95.504
0.828 11.997	1.632 48.119
ENSMUSG00000025702	8-Mar protein_coding	1.071 9.805 1.004 95.504
0.929 12.948	1.021 92.748
ENSMUSG00000025716	Myo3a protein_coding	1	78.744	1
91.584	1	80.015	NA	NA
ENSMUSG00000025722	Wdr73 protein_coding	0.958 26.013	0.882
65.165	1.181 7.24	NA	NA
ENSMUSG00000025723	Nmb	protein_coding	0.999 85.391	0.731
85.242	1.363 62.648	1.15	83.276
ENSMUSG00000025724	Sec11a	protein_coding	1.124 22.205
0.953 87.452	0.934 41.928	1.024 71.008
ENSMUSG00000025726	Slc28a1	protein_coding	1.359 76.546
0.857 96.013	0.86	84.434	1.299 90.865
ENSMUSG00000025728	Pigq	protein_coding	0.928 9.841 1.053 82.904
1.025 75.155	0.966 90.611
ENSMUSG00000025730	Rab40c	protein_coding	0.956 30.035
1.084 75.21 0.961 86.084	0.971 87.992
ENSMUSG00000025731	0610011F06Rik	protein_coding	0.985 85.728
0.955 73.461	1.065 43.842	0.988 86.991
ENSMUSG00000025732	Fam195a	protein_coding	0.75	5.686 1.055
93.994	1.265 38.099	0.993 95.832
ENSMUSG00000025733	Rhot2 protein_coding	0.937 25.111	1.044
82.76 1.022 83.857	1.047 93.132
ENSMUSG00000025735	Rhbdl1	protein_coding	0.846 51.56 0.978
93.094	1.21	37.764	0.924 88.505
ENSMUSG00000025736	Jmjd8 protein_coding	0.876 1.274 1.127 70.816
1.011 79.119	1.013 94.904
ENSMUSG00000025737	Wdr24 protein_coding	1.021 65.284	0.957
81.731	1.02	79.325	1.022 95.74
ENSMUSG00000025738	Fbxl16	protein_coding	1.117 48.462
0.972 92.793	0.92	27.194	0.96	93.685
ENSMUSG00000025739	Gng13 protein_coding	1.19	50.377	0.904
79.475	0.931 87.37 0.6	83.724
ENSMUSG00000025742	Prps2 protein_coding	1.192 1.395 0.932 68.818
0.898 30.024	0.959 89.892
ENSMUSG00000025743	Sdc3	protein_coding	1.505 0	0.842 84.858
0.785 6.858 1.208 59.441
ENSMUSG00000025745	Hadha protein_coding	0.918 36.945	NA	NA
1.093 54.673	1.025 95.273
ENSMUSG00000025747	Tyms	protein_coding	1.075 47.174	0.922
87.147	1.007 87.971	1.064 78.948
ENSMUSG00000025757	Hspa4l	protein_coding	0.824 9.599 1.067
93.899	1.137 28.997	1.077 67.94
ENSMUSG00000025758	Plk4	protein_coding	0.98	84.661	0.993
96.407	1.025 76.768	1.313 36.988
ENSMUSG00000025759	Mfsd8 protein_coding	0.876 8.002 1.133 78.235
1.008 80.015	0.988 90.611
ENSMUSG00000025762	Larp1b	protein_coding	0.899 15.76 0.882
87.041	1.263 21.705	0.961 90.691
ENSMUSG00000025764	Phf17 protein_coding	1.047 37.941	1.026

96.407 0.931 6.858 0.927 83.724
ENSMUSG00000025766 D3Ertd751e protein_coding 1.18 2.969 0.928
80.052 0.915 23.938 1.022 96.287
ENSMUSG00000025774 Crisp4 protein_coding 1 86.006 1
96.268 1 84.724 1 95.273
ENSMUSG00000025776 Crispld1 protein_coding 1.17 3.498 1.04
97.129 0.823 9.494 1.19 55.841
ENSMUSG00000025777 Gdap1 protein_coding 2.031 11.879 1.019
95.708 0.483 22.279 0.989 93.964
ENSMUSG00000025779 Ly96 protein_coding 1.453 2.723 0.885 87.919
0.778 16.949 1.061 96.287
ENSMUSG00000025780 Itih5 protein_coding 1.176 4.675 0.977 84.017
0.868 19.552 1.003 87.7
ENSMUSG00000025781 Atp5c1 protein_coding 0.954 19.985
1.023 95.211 1.025 58.616 0.991 95.832
ENSMUSG00000025782 Taf3 protein_coding 0.948 52.258 1.081
71.222 0.976 36.248 0.986 86.699
ENSMUSG00000025783 4930412O13Rik protein_coding 0.649 8.002
1.082 87.604 1.421 32.433 0.819 86.445
ENSMUSG00000025784 Clec3b protein_coding 1.028 80.688
1.038 96.847 0.937 79.776 1 92.52
ENSMUSG00000025785 Exosc7 protein_coding 0.999 82.231 0.87
50.664 1.149 9.502 1.086 54.153
ENSMUSG00000025786 Zdhhc3 protein_coding 1.056 13.633
0.989 90.053 0.952 11.314 1.06 46.412
ENSMUSG00000025787 Tgm4 protein_coding 0.602 3.101 1.102 84.126
1.507 6.506 0.875 96.183
ENSMUSG00000025789 St8sia2 protein_coding 2.374 4.874 0.991
83.104 0.426 1.764 0.876 92.748
ENSMUSG00000025790 Slco3a1 protein_coding 0.785 9.081 0.959
95.301 1.329 27.047 1.174 48.119
ENSMUSG00000025791 Pgm2 protein_coding 0.922 34.162 1.068
71.617 1.012 82.903 1.009 86.445
ENSMUSG00000025792 Slc25a10 protein_coding 0.586 1.031 1.287
71.222 1.328 31.761 0.954 90.289
ENSMUSG00000025793 Hgs protein_coding 0.959 11.519 1.041
78.235 1.001 78.155 1.01 87.447
ENSMUSG00000025794 Rpl14 protein_coding 1.067 18.388 0.92
35.072 1.015 80.089 1.054 91.928
ENSMUSG00000025795 Rassf3 protein_coding 0.849 18.907
1.054 83.104 1.113 41.331 0.974 83.724
ENSMUSG00000025804 Ccr1 protein_coding 1.256 44.694 0.824
52.266 0.963 70.9 1.475 0
ENSMUSG00000025808 Ccdc7 protein_coding 1 83.169 1
93.094 1 73.874 1 96.287
ENSMUSG00000025809 Itgb1 protein_coding 1.026 53.228 1.021
96.216 0.956 44.865 1.064 50.452
ENSMUSG00000025810 Nrp1 protein_coding 1.194 5.313 0.962 96.68 0.87
6.524 0.902 88.505
ENSMUSG00000025812 Pard3 protein_coding 0.966 13.45 0.957 88.704
1.082 13.259 1.013 95.593
ENSMUSG00000025813 Homer2 protein_coding 1.136 33.325
0.939 83.733 0.938 82.685 0.956 94.016
ENSMUSG00000025815 Dhtkd1 protein_coding 1.058 83.658

0.996 90.29 0.95 71.004 0.868 85.493
ENSMUSG00000025816 Sec61a2 protein_coding 0.885 4.055 0.988 84.858 1.147 5.964 1.046 60.968
ENSMUSG00000025817 Nudt5 protein_coding 0.925 22.731 NA NA 1.113 30.292 0.998 93.132
ENSMUSG00000025821 Zfp282 protein_coding 0.927 30.035 0.997 90.256 1.078 15.602 0.989 91.979
ENSMUSG00000025823 Pdia4 protein_coding 1.037 73.124 NA NA 0.949 75.637 1.114 44.679
ENSMUSG00000025825 Iscu protein_coding 0.858 0.987 1.053 76.065 1.106 23.604 0.983 88.245
ENSMUSG00000025838 Pramel6 protein_coding 1 85.946 NA NA 1 88.693 1 94.654
ENSMUSG00000025839 Pramel7 protein_coding 1 67.991 1 95.211 1 38.434 1 92.12
ENSMUSG00000025854 Fam20c protein_coding 1.155 26.77 1.017 95.708 0.853 51.055 1.208 50.74
ENSMUSG00000025855 Prkar1b protein_coding 0.931 61.334 1.015 92.474 1.055 51.243 0.999 86.699
ENSMUSG00000025856 Pdgfa protein_coding 1.02 72 1.171 79.411 0.837 3.943 0.964 86.445
ENSMUSG00000025857 Heatr2 protein_coding 0.842 1.239 0.969 85.418 1.227 5.964 1.008 91.928
ENSMUSG00000025858 Get4 protein_coding 0.942 24.344 0.997 95.708 1.065 33.802 1.008 94.904
ENSMUSG00000025860 Xiap protein_coding 0.958 32.749 0.954 85.242 1.092 7.855 0.924 83.724
ENSMUSG00000025862 Stag2 protein_coding 1.055 22.205 0.942 67.839 1.005 84.724 0.957 86.699
ENSMUSG00000025867 Cplx2 protein_coding 1.104 31.732 0.89 82.848 1.016 86.727 0.839 83.724
ENSMUSG00000025868 Higd2a protein_coding 0.908 2.559 1.047 79.411 1.049 52.8 0.986 86.991
ENSMUSG00000025869 Nop16 protein_coding 0.964 60.569 1.015 92.137 1.018 53.587 1.085 60.466
ENSMUSG00000025870 Arl10 protein_coding 1.059 51.826 1.065 96.763 0.885 55.701 0.956 84.29
ENSMUSG00000025871 4833439L19Rik protein_coding 0.987 74.958 1.105 63.504 0.917 6.858 1.063 62.594
ENSMUSG00000025872 Thoc3 protein_coding 1.037 22.025 0.994 93.094 0.972 63.919 1.016 78.042
ENSMUSG00000025873 Faf2 protein_coding 1.07 1.493 0.984 90.138 0.947 12.571 0.997 95.593
ENSMUSG00000025875 Tspan17 protein_coding 0.809 1.803 1.058 80.806 1.169 16.666 0.966 88.245
ENSMUSG00000025876 Unc5a protein_coding 0.917 28.514 1.06 82.904 NA NA 1.12 57.1
ENSMUSG00000025877 Hk3 protein_coding 1.696 0 0.767 56.781 0.769 14.079 1.045 72.826
ENSMUSG00000025878 Uimc1 protein_coding 1.01 71.217 0.944 56.781 1.046 38.152 1.014 77.854
ENSMUSG00000025880 Smad7 protein_coding 0.974 79.433 0.939 95.504 1.097 79.325 1.052 96.248
ENSMUSG00000025885 Myo5b protein_coding 1.126 21.836 0.905

68.115	0.983	87.786	0.975	94.654
ENSMUSG00000025887	Casp12	protein_coding	1.112	12.072
1.056	96.407	0.85	13.848	1.224	52.324
ENSMUSG00000025888	Casp1	protein_coding	1.369	0.999	0.922	90.712
0.793	7.742	1.026	86.445
ENSMUSG00000025889	Snca	protein_coding	1.291	44.334	0.732
68.818	1.058	84.724	0.961	94.904
ENSMUSG00000025892	Gria4	protein_coding	1.241	31.24	0.975	91.361
0.828	67.648	1.022	84.31
ENSMUSG00000025893	Kbtbd3	protein_coding	1.153	18.556	NA
NA	0.807	16.129	0.929	90.691
ENSMUSG00000025894	Aasdhppt	protein_coding	1.023	76.042
0.965	87.452	1.015	80.089	1.003	96.248
ENSMUSG00000025898	Cwf19l2	protein_coding	0.992	74.745	0.95
54.06	1.056	19.106	1.032	78.379
ENSMUSG00000025899	Alkbh8	protein_coding	1.024	85.355
0.946	73.461	1.034	45.94	0.952	96.042
ENSMUSG00000025900	Rp1	protein_coding	0.843	78.909	0.408
83.321	2.919	47.776	2.377	83.067
ENSMUSG00000025902	Sox17	protein_coding	0.885	57.014	0.887
55.71	1.272	13.082	1.058	91.807
ENSMUSG00000025903	Lypla1	protein_coding	0.989	81.01	1.032
90.389	0.982	69.583	0.992	83.067
ENSMUSG00000025905	Oprk1	protein_coding	1	84.452	1
93.994	1	87.336	1	86.301
ENSMUSG00000025907	Rb1cc1	protein_coding	0.97	71.674
0.995	87.857	1.037	38.152	1.027	86.118
ENSMUSG00000025909	Sntg1	protein_coding	1.236	52.429	1.395
90.116	0.581	31.135	1.409	72.116
ENSMUSG00000025911	Adhfe1	protein_coding	1.047	79.577
1.047	84.768	0.915	32.297	0.955	85.493
ENSMUSG00000025912	Mybl1	protein_coding	1.126	61.171	1.133
93.994	0.784	46.071	1.155	73.597
ENSMUSG00000025915	Sgk3	protein_coding	1.039	37.414	1.11
96.847	0.869	32.082	1.08	54.153
ENSMUSG00000025917	Cops5	protein_coding	0.94	18.907	1.021
84.126	1.039	52.8	0.998	78.948
ENSMUSG00000025920	Stau2	protein_coding	1.096	12.549	1.029
97.263	0.885	12.948	1.014	94.153
ENSMUSG00000025921	Rdh10	protein_coding	1.105	28.693	1.087
82.219	0.831	3.718	0.928	85.493
ENSMUSG00000025922	Gm5045	pseudogene	1	81.24	1	90.712
1	82.778	1	96.086
ENSMUSG00000025925	Terf1	protein_coding	1	86.112	0.898
75.052	1.117	37.259	1.047	79.713
ENSMUSG00000025927	Tfap2b	protein_coding	0.715	12.624
0.745	60.806	1.883	5.864	0.689	86.445
ENSMUSG00000025929	Il17a	protein_coding	1	86.57	1	95.088
1	82.161	1	96.358
ENSMUSG00000025930	Msc	protein_coding	1.016	81.125	0.961
95.708	1.027	87.971	0.731	83.724
ENSMUSG00000025931	Paqr8	protein_coding	1.018	81.24	0.942	73.461
1.038	67.562	0.994	93.261
ENSMUSG00000025932	Eya1	protein_coding	0.957	77.763	1.648

60.309 0.632 6.858 0.905 90.691
ENSMUSG00000025933 Tmem14a protein_coding 0.979 77.113
0.885 70.864 1.156 36.839 1.014 93.734
ENSMUSG00000025934 Gsta3 protein_coding 0.46 1.274 0.718 81.731
3.016 5.964 1.18 91.525
ENSMUSG00000025935 Tram1 protein_coding 1.143 0.355 1.069 83.104
0.82 0.72 1.037 83.581
ENSMUSG00000025936 Gm4956 pseudogene 1.094 66.944 0.789
88.704 1.157 70.623 0.832 94.19
ENSMUSG00000025937 Lactb2 protein_coding 0.931 14.849
1.043 82.904 1.026 64.624 0.904 86.991
ENSMUSG00000025938 Slco5a1 protein_coding 1.527 39.416
1.071 82.904 0.61 4.48 1.988 31.133
ENSMUSG00000025939 Ube2w protein_coding 1.117 15.178 1.05
96.843 0.854 33.137 1.091 83.581
ENSMUSG00000025940 Tmem70 protein_coding 0.967 28.584 1.08
64.304 0.954 25.512 0.985 95.72
ENSMUSG00000025945 Crygf protein_coding 1 62.007 NA NA
1 87.519 1 95.273
ENSMUSG00000025949 Pikfyve protein_coding 0.9 27.968 NA
NA 1.16 39.925 1.021 94.904
ENSMUSG00000025950 Idh1 protein_coding 1.144 2.512 1.215 64.123
0.719 0.72 1.087 83.276
ENSMUSG00000025952 Crygc protein_coding 1 78.428 1
95.708 1 81.29 1 95.777
ENSMUSG00000025955 Akr1cl protein_coding 3.811 2.047 0.675
95.35 0.389 5.884 3.617 78.042
ENSMUSG00000025956 Mettl21a protein_coding 1.177 1.292 0.833
28.822 1.022 77.888 0.929 83.724
ENSMUSG00000025958 Creb1 protein_coding 1.011 75.196 1.019
96.407 0.971 71.388 0.94 85.493
ENSMUSG00000025959 Klf7 protein_coding 0.952 66.406 1.049
83.104 0.995 82.575 0.944 83.724
ENSMUSG00000025961 NA NA NA NA 1 67.839 1
74.325 1 93.413
ENSMUSG00000025962 Fastkd2 protein_coding 1.19 7.842 0.919
91.83 0.916 34.21 0.967 92.31
ENSMUSG00000025963 Mdh1b protein_coding 0.73 63.283 1.248
93.908 1.095 52.432 0.821 96.287
ENSMUSG00000025964 Adam23 protein_coding 1.338 2.352 1.029
94.262 0.726 10.23 1.23 43.377
ENSMUSG00000025967 Eef1b2 protein_coding 1.074 29.372
0.907 28.822 1.028 66.043 1.153 66.944
ENSMUSG00000025968 Ndufs1 protein_coding 0.93 24.748
0.997 96.843 1.078 43.129 1.06 88.797
ENSMUSG00000025969 Nrp2 protein_coding 1.021 36.686 1.073
83.819 0.914 9.131 1.025 95.273
ENSMUSG00000025971 9430016H08Rik protein_coding 0.967 64.589
1.055 93.899 0.98 71.734 0.943 85.493
ENSMUSG00000025978 Rftn2 protein_coding 1.16 6.399 NA NA
0.854 6.858 0.924 84.19
ENSMUSG00000025979 Mob4 protein_coding 1.132 0.767 0.997 95.809
0.887 0.72 0.941 83.724
ENSMUSG00000025980 Hspd1 protein_coding 1.005 83.823 0.922

96.216 1.078 82.778 1.048 73.939
ENSMUSG00000025981 Coq10b protein_coding 0.972 74.299
1.053 83.464 0.972 62.058 1.18 49.769
ENSMUSG00000025982 Sf3b1 protein_coding 1.039 44.694 0.948
44.566 1.013 44.361 1.007 68.696
ENSMUSG00000025983 Ccdc150 protein_coding 1.852 52.134
1.251 83.104 0.432 14.262 0.545 88.955
ENSMUSG00000025986 Slc39a10 protein_coding 1.04 54.44 1.032
96.68 0.929 33.689 1.04 64.513
ENSMUSG00000025993 Slc40a1 protein_coding 1.46 6.683 0.886
75.493 0.774 48.851 0.803 90.289
ENSMUSG00000025995 Wdr75 protein_coding 1.116 11.638 0.945
94.48 0.944 36.499 0.973 93.612
ENSMUSG00000025997 Ikzf2 protein_coding 1.023 69.985 1.056
93.529 0.926 87.37 0.835 83.724
ENSMUSG00000026000 Lancl1 protein_coding 0.933 19.985
1.003 97.049 1.069 26.179 1.003 94.608
ENSMUSG00000026003 Acadl protein_coding 1.036 53.562 0.941
93.094 1.026 78.642 0.933 83.724
ENSMUSG00000026004 Kansl1l protein_coding 1.12 4.825 1.044
82.904 0.854 1.764 0.976 86.699
ENSMUSG00000026005 Rpe protein_coding 0.867 13.952 1.023
88.136 1.128 43.185 1.05 91.492
ENSMUSG00000026009 Icos protein_coding 1.292 49.517 0.895
88.345 0.864 77.037 1.169 91.291
ENSMUSG00000026011 Ctla4 protein_coding 0.899 82.352 3.372
82.904 0.328 33.971 0.739 84.29
ENSMUSG00000026012 Cd28 protein_coding 1.884 11.879 0.821
91.83 0.647 23.938 0.515 83.724
ENSMUSG00000026014 Raph1 protein_coding 0.965 67.334 1.09
79.29 0.946 46.575 0.984 88.505
ENSMUSG00000026017 Carf protein_coding 0.908 12.624 0.946
91.361 1.166 20.886 0.991 96.192
ENSMUSG00000026018 Ica1l protein_coding 0.818 72.139 0.846
75.618 1.438 54.363 1.161 95.877
ENSMUSG00000026019 Wdr12 protein_coding 1.044 56.311 0.968
84.594 0.991 79.426 1.066 72.204
ENSMUSG00000026020 Nop58 protein_coding 1.224 7.179 0.867 72.712
0.942 51.055 1.126 55.841
ENSMUSG00000026021 Sumo1 protein_coding 1.022 72.85 0.928 81.731
1.055 50.091 1.139 36.174
ENSMUSG00000026023 Cdk15 protein_coding 1.409 2.446 1.105 96.847
0.64 4.23 1.065 94.608
ENSMUSG00000026024 Als2 protein_coding 1.096 13.492 1.007
83.104 0.906 5.564 0.903 83.724
ENSMUSG00000026027 Stradb protein_coding 0.997 73.487
1.107 71.617 0.905 11.902 1.007 95.74
ENSMUSG00000026028 Trak2 protein_coding 1.08 14.34 1.029 89.434
0.9 8.027 1.065 44.882
ENSMUSG00000026029 Casp8 protein_coding 1.154 0.461 0.919 82.947
0.945 10.602 0.986 95.72
ENSMUSG00000026031 Cflar protein_coding 1.004 60.853 1.052
81.039 0.942 11.751 0.976 86.991
ENSMUSG00000026032 Ndufb3 protein_coding 0.885 1.214 1.006

91.643	1.117	18.208	1.012	96.183
ENSMUSG00000026034	Clk1	protein_coding	1.016	40.88	0.941	74.851
1.047	69.825	1.003	91.217
ENSMUSG00000026035	Ppil3	protein_coding	1.111	1.815	1.01	96.68
0.892	1.58	1.003	95.74
ENSMUSG00000026036	Nif3l1	protein_coding	0.881	13.45	1.074
93.899	1.054	45.667	1.03	87.134
ENSMUSG00000026037	Orc2	protein_coding	1.113	12.72	1.019	93.529
0.882	8.192	1.039	83.276
ENSMUSG00000026039	Sgol2	protein_coding	1.036	64.956	0.819
79.475	1.182	85.464	1.192	90.177
ENSMUSG00000026042	Col5a2	protein_coding	1.093	36.195
0.789	42.923	1.158	88.693	1.302	44.882
ENSMUSG00000026043	Col3a1	protein_coding	1.193	39.416
0.785	54.427	1.067	86.831	1.456	42.736
ENSMUSG00000026047	Kdelc1	protein_coding	1.016	85.637
0.997	96.407	0.985	75.967	1.116	42.736
ENSMUSG00000026048	Ercc5	protein_coding	1.02	39.295	0.988
85.242	0.991	88.41	1.005	96.287
ENSMUSG00000026049	Tex30	protein_coding	1.027	64.401	0.917
86.071	1.057	79.119	1.116	64.513
ENSMUSG00000026051	1500015O10Rik	protein_coding	1.422	3.151
1.251	93.707	0.561	3.943	1.08	91.011
ENSMUSG00000026058	Khdrbs2	protein_coding	1	66.652	1
94.848	1	63.301	1	83.067
ENSMUSG00000026062	Slc9a2	protein_coding	1.413	20.799
1.286	87.604	0.551	3.123	0.878	93.261
ENSMUSG00000026063	Pih1d3	protein_coding	1	74.689	1
97.155	1	84.626	1	93.734
ENSMUSG00000026064	Ptp4a1	protein_coding	0.927	49.047
0.896	95.211	1.206	48.304	1.091	83.067
ENSMUSG00000026065	Slc9a4	protein_coding	1.637	3.536	0.868
81.134	0.7	32.082	1.18	67.691
ENSMUSG00000026068	Il18rap	protein_coding	0.827	46.413
1.274	79.367	0.946	78.045	1.073	92.748
ENSMUSG00000026069	Il1rl1	protein_coding	1.181	5.313	0.873
73.166	0.972	78.954	1.175	90.674
ENSMUSG00000026070	Il18r1	protein_coding	1.186	15.456
0.867	73.766	0.969	75.967	1.138	56.863
ENSMUSG00000026072	Il1r1	protein_coding	1.361	0	0.914	41.141
0.804	1.764	1.1	67.94
ENSMUSG00000026073	Il1r2	protein_coding	0.896	14.849	NA	NA
1.124	46.956	1.188	37.634
ENSMUSG00000026074	Map4k4	protein_coding	1.105	11.197
0.913	74.851	0.99	54.521	0.975	86.991
ENSMUSG00000026077	Npas2	protein_coding	1.247	72.372	0.889
95.329	0.901	86.112	0.67	84.29
ENSMUSG00000026078	Pdcl3	protein_coding	1.053	30.497	0.965
81.731	0.982	69.445	0.993	94.001
ENSMUSG00000026080	Chst10	protein_coding	1.448	3.498	1.169
83.104	0.589	3.718	0.994	96.183
ENSMUSG00000026082	Rev1	protein_coding	1.12	49.517	0.976
96.897	0.911	25.688	1.041	69.404
ENSMUSG00000026083	Eif5b	protein_coding	0.968	54.983	0.989

81.02 1.044 22.517 1.046 57.1
ENSMUSG00000026087 Mrpl30 protein_coding 0.973 38.555 NA
NA 1.058 22.444 1.075 76.046
ENSMUSG00000026088 Mitd1 protein_coding 1.04 77.182 0.953
95.088 1.011 83.397 1.017 94.82
ENSMUSG00000026090 2010300C02Rik protein_coding 1.011 71.142
0.805 79.807 1.227 31.761 0.811 83.724
ENSMUSG00000026094 Stk17b protein_coding 1.14 1.913 0.886
49.859 0.991 80.163 0.958 83.724
ENSMUSG00000026095 Asnsd1 protein_coding 1.085 6.958 0.912
65.165 1.01 55.214 1.068 83.067
ENSMUSG00000026096 Osgepl1 protein_coding 0.994 78.428 1.01
95.941 0.998 85.58 1.029 84.499
ENSMUSG00000026097 Ormdl1 protein_coding 1.14 5.657 0.953
87.857 0.921 22.279 0.956 87.705
ENSMUSG00000026098 Pms1 protein_coding 1.15 29.536 0.957
94.848 0.905 68.584 0.992 96.183
ENSMUSG00000026100 Mstn protein_coding 3.23 64.956 3.746
88.092 0.082 8.192 0.864 96.358
ENSMUSG00000026102 Inpp1 protein_coding 1.015 67.477 0.909
96.268 1.084 63.801 1.002 95.777
ENSMUSG00000026103 Gls protein_coding 1.099 0.664 0.948 80.052
0.961 42.895 1.033 93.156
ENSMUSG00000026104 Stat1 protein_coding 1.035 83.968 1.115
71.617 0.864 36.499 1.084 70.85
ENSMUSG00000026107 Nabp1 protein_coding 1.129 65.733 1.269
79.261 0.696 16.643 0.835 86.991
ENSMUSG00000026109 Tmeff2 protein_coding 1.056 48.462
1.029 96.763 0.918 43.426 1.048 83.067
ENSMUSG00000026110 Mgat4a protein_coding 1.109 2.352 0.905
8.462 0.997 72.853 0.943 84.29
ENSMUSG00000026111 Unc50 protein_coding 1.001 77.763 0.979
93.654 1.016 82.161 NA NA
ENSMUSG00000026112 Coa5 protein_coding 0.956 42.922 1.099
87.609 0.953 86.475 0.927 86.445
ENSMUSG00000026113 Inpp4a protein_coding 1.207 0.355 0.968
92.404 0.853 6.381 0.979 96.183
ENSMUSG00000026115 Vwa3b protein_coding 1.224 75.671 0.707
95.504 1.15 88.729 0.891 92.675
ENSMUSG00000026116 Tmem131 protein_coding 1.064 34.696 0.96
85.418 0.976 50.184 0.923 84.19
ENSMUSG00000026117 Zap70 protein_coding 0.877 73.292 1.076
88.136 1.06 69.445 1.119 96.086
ENSMUSG00000026121 Sema4c protein_coding 0.831 7.218 1.062
96.407 1.134 10.523 1.027 90.177
ENSMUSG00000026123 Plekhb2 protein_coding 1.383 0 0.901
20.174 0.801 0.879 0.941 96.248
ENSMUSG00000026124 Cfc1 protein_coding 1 86.351 1
90.712 1 86.727 1 88.755
ENSMUSG00000026126 Ptpn18 protein_coding 1.026 82.982 0.97
91.897 1.008 71.615 0.966 93.734
ENSMUSG00000026127 Imp4 protein_coding 1.01 83.169 1.02
86.156 0.974 55.214 0.998 96.248
ENSMUSG00000026131 Dst protein_coding 1.034 85.946 0.946

66.651 1.025 52.574 1 87.331
ENSMUSG00000026134 Prim2 protein_coding 1.211 6.958 0.885 79.807
0.935 23.938 1.178 44.679
ENSMUSG00000026135 Zfp142 protein_coding 1.054 53.228
1.076 79.29 0.883 1.764 0.983 92.466
ENSMUSG00000026141 Col19a1 protein_coding 2.195 6.399 1.049
82.503 0.434 23.607 0.431 83.724
ENSMUSG00000026142 Rhbdd1 protein_coding 1.067 1.323 1.019
93.574 0.915 3.432 0.978 83.724
ENSMUSG00000026147 Col9a1 protein_coding 0.678 61.887
1.134 91.361 1.302 40.511 1.279 77.331
ENSMUSG00000026149 Tm4sf20 protein_coding 1 85.637 1
96.847 1 76.498 1 96.183
ENSMUSG00000026150 Mff protein_coding 0.958 55.641 1.002
97.263 1.045 53.587 1.062 51.908
ENSMUSG00000026153 Fam135a protein_coding 1.107 12.855
0.973 89.509 0.925 48.387 0.9 83.724
ENSMUSG00000026154 1110058L19Rik protein_coding 0.957 66.129
NA NA 1.066 64.024 1.017 93.964
ENSMUSG00000026155 Smap1 protein_coding 1.075 7.501 0.966 63.619
0.964 48.577 1.021 76.697
ENSMUSG00000026156 B3gat2 protein_coding 0.839 16.652 0.96
97.263 1.245 30.645 0.871 91.372
ENSMUSG00000026158 Ogfrl1 protein_coding 1.015 77.916
0.962 85.95 1.025 69.825 0.989 86.991
ENSMUSG00000026159 Agfg1 protein_coding 0.988 67.163 1.036
95.431 0.974 66.753 1.137 56.863
ENSMUSG00000026162 Nhej1 protein_coding 0.969 64.589 0.94
91.955 1.092 47.776 1.049 86.209
ENSMUSG00000026163 Sphkap protein_coding 1.76 36.195
0.903 93.765 0.628 47.12 1.353 48.857
ENSMUSG00000026166 Ccl20 protein_coding 1 86.172 1
97.263 1 84.552 1 93.457
ENSMUSG00000026167 Wnt10a protein_coding 0.812 43.167
1.472 60.309 0.838 39.245 0.849 86.699
ENSMUSG00000026170 Cyp27a1 protein_coding 1.112 33.854
0.972 79.261 0.921 88.203 0.943 86.991
ENSMUSG00000026171 Rnf25 protein_coding 0.965 53.138 1.025
94.767 1.012 57.637 0.981 86.991
ENSMUSG00000026172 Bcs1l protein_coding 0.981 64.589 1.058
71.617 0.96 31.676 0.992 89.587
ENSMUSG00000026173 Plcd4 protein_coding 1.55 32.058 1.232
90.29 0.524 6.202 0.639 90.674
ENSMUSG00000026174 Rqcd1 protein_coding 1.093 11.197 0.891
55.71 1.029 86.552 1.071 50.74
ENSMUSG00000026175 Vil1 protein_coding 0.906 76.968 0.779
85.418 1.411 52.432 0.917 90.854
ENSMUSG00000026176 Ctdsp1 protein_coding 0.969 76.135
1.061 71.617 0.971 29.451 1.031 96.184
ENSMUSG00000026177 Slc11a1 protein_coding 1.453 0 0.809
55.71 0.847 8.313 1.099 76.831
ENSMUSG00000026179 Pnkd protein_coding 0.885 22.481 1.127
78.494 1.001 84.434 0.96 83.724
ENSMUSG00000026180 Cxcr2 protein_coding 1.363 31.24 1.219 94.367

0.602 39.755 0.625 86.991
ENSMUSG00000026181 Ppm1f protein_coding 0.892 4.993 1.057 71.617
1.062 45.449 1.021 83.581
ENSMUSG00000026182 Tnp1 protein_coding 1 85.728 1
95.504 1 87.971 1 92.181
ENSMUSG00000026185 Igfbp5 protein_coding 0.951 38.827
1.108 82.904 0.945 60.614 0.929 87.331
ENSMUSG00000026187 Xrcc5 protein_coding 0.964 85.946 0.914
88.704 1.137 61.743 1.071 77.331
ENSMUSG00000026188 Tmem169 protein_coding 0.702 62.888
0.783 96.68 1.821 82.161 2.249 49.239
ENSMUSG00000026189 Pecr protein_coding 1.074 39.851 NA NA
0.919 51.715 0.833 83.724
ENSMUSG00000026192 Atic protein_coding 0.913 48.646 NA NA
1.096 57.637 1.285 31.133
ENSMUSG00000026193 Fn1 protein_coding 1.152 49.868 0.854
79.261 1.017 86.258 0.988 93.132
ENSMUSG00000026196 Bard1 protein_coding 1.51 19.746 1.024
90.378 0.648 64.778 0.995 89.052
ENSMUSG00000026197 Zfand2b protein_coding 0.993 72.985
0.998 91.897 1.007 66.269 0.966 90.157
ENSMUSG00000026198 Abcb6 protein_coding 1.007 85.129 1.032
92.111 0.962 77.294 1.055 66.468
ENSMUSG00000026199 Ankzf1 protein_coding 0.944 30.352
0.944 75.4 1.121 11.329 0.969 89.892
ENSMUSG00000026200 Glb1l protein_coding 1.001 80.815 0.972
64.555 1.027 39.439 1.039 83.276
ENSMUSG00000026201 Stk16 protein_coding 0.897 1.422 1.072 62.898
1.04 26.179 1.023 87.447
ENSMUSG00000026202 Tuba4a protein_coding 0.999 74.745
0.941 71.41 1.064 48.304 1.173 96.139
ENSMUSG00000026203 Dnajb2 protein_coding 0.9 18.602
1.143 84.126 0.974 76.923 0.923 83.724
ENSMUSG00000026204 Ptprn protein_coding 0.712 27 0.941 95.196
1.495 31.317 0.977 96.358
ENSMUSG00000026205 Slc23a3 protein_coding 0.913 86.843
0.995 91.532 1.101 72.378 0.911 86.991
ENSMUSG00000026207 Speg protein_coding 1.022 81.925 1.225
60.309 0.799 10.23 0.945 93.734
ENSMUSG00000026208 Des protein_coding 1.834 15.662 1.127
79.411 0.482 1.898 0.914 96.248
ENSMUSG00000026209 Dnpep protein_coding 0.89 3.895 1.058 72.642
1.064 34.403 1.009 93.457
ENSMUSG00000026211 Obsl1 protein_coding 0.876 8.709 1.065 92.931
1.073 50.091 1.137 33.269
ENSMUSG00000026213 Stk11ip protein_coding 0.88 3.895 NA NA
1.147 5.964 1.003 96.071
ENSMUSG00000026219 Trip12 protein_coding 1.008 81.196
0.965 65.165 1.028 52.574 1.063 67.94
ENSMUSG00000026220 Slc16a14 protein_coding 0.991 84.977
0.704 75.345 1.43 72.853 1.432 92.018
ENSMUSG00000026222 Sp100 protein_coding 1.19 0.2 0.991 87.919
0.847 27.675 1.15 35.053
ENSMUSG00000026223 Itm2c protein_coding 1.055 6.23 0.958 84.594

0.989 49.453 1.019 96.183
ENSMUSG00000026226 Spata3 protein_coding 0.913 86.333
1.929 82.904 0.568 15.904 0.702 86.699
ENSMUSG00000026227 2810459M11Rik protein_coding 1.087 35.787
0.881 93.817 1.041 85.21 0.96 86.445
ENSMUSG00000026228 Htr2b protein_coding 1.905 10.19 1.011 96.763
0.517 14.079 0.981 89.827
ENSMUSG00000026229 Psmd1 protein_coding 0.925 15.486 1.03
87.029 1.046 38.975 1.056 33.269
ENSMUSG00000026234 Ncl protein_coding 1.053 61.49 0.895 35.072
1.063 30.561 1.114 0
ENSMUSG00000026235 Epha4 protein_coding 1.034 68.16 1.175 71.222
0.823 6.515 0.879 85.493
ENSMUSG00000026238 Ptma protein_coding 0.958 84.263 0.983
96.138 1.063 72.148 0.99 87.218
ENSMUSG00000026239 Pde6d protein_coding 1.014 85.283 1.021
83.104 0.962 52.972 0.998 89.892
ENSMUSG00000026240 Cops7b protein_coding 0.975 54.176
1.024 92.931 1.001 83.719 0.973 86.991
ENSMUSG00000026245 Farsb protein_coding 0.994 60.168 1.006
91.661 1.002 88.274 1.109 68.696
ENSMUSG00000026246 Alppl2 protein_coding 1 84.738 1
92.545 1 82.31 NA NA
ENSMUSG00000026247 Ecel1 protein_coding 2.559 41.454 0.927
96.216 0.421 69.583 0.695 86.699
ENSMUSG00000026248 Mrpl44 protein_coding 0.991 57.227
0.952 93.908 1.057 68.129 1.041 95.832
ENSMUSG00000026249 Serpine2 protein_coding 1.307 2.569 0.898
82.947 0.852 14.409 1.053 85.786
ENSMUSG00000026251 Chrnd protein_coding 1 83.823 NA NA
1 84.896 1 82.66
ENSMUSG00000026253 NA NA NA NA 1 96.216 1
79.625 1 91.928
ENSMUSG00000026254 Eif4e2 protein_coding 0.98 39.712
1.036 83.464 0.986 78.293 1.035 48.119
ENSMUSG00000026255 Efhd1 protein_coding 1.194 26.77 1.011 92.561
0.824 33.971 1.094 92.381
ENSMUSG00000026258 3110079O15Rik protein_coding 0.881 66.129
0.985 94.597 1.155 52.574 0.828 83.724
ENSMUSG00000026259 Ngef protein_coding 0.991 68.353 0.991
94.262 1.019 65.034 0.992 89.587
ENSMUSG00000026260 Ndufa10 protein_coding 0.891 0.987 1.069
79.411 1.049 35.441 1.039 93.734
ENSMUSG00000026269 Rnpepl1 protein_coding 0.905 5.544 1.094
78.235 1.01 50.091 0.922 84.29
ENSMUSG00000026270 Capn10 protein_coding 0.945 25.85 0.957
68.818 1.105 5.964 1.041 83.067
ENSMUSG00000026271 Gpr35 protein_coding 1.623 0.767 0.757 36.617
0.81 38.534 0.964 83.067
ENSMUSG00000026272 Agxt protein_coding 1 81.823 1
96.652 1 85.352 1 94.904
ENSMUSG00000026273 Mterfd2 protein_coding 0.974 55.303
1.027 87.029 0.998 88.203 1.021 87.7
ENSMUSG00000026274 Pask protein_coding 0.996 74.482 1.008

93.094 0.994 83.719 1.268 72.616
ENSMUSG00000026275 Ppp1r7 protein_coding 1.045 27.32 0.946
68.818 1.01 84.724 1.053 69.965
ENSMUSG00000026276 2-Sep protein_coding 1.092 2.085 1.017 96.847
0.898 4.759 1.041 57.075
ENSMUSG00000026277 Stk25 protein_coding 0.939 3.623 0.986 96.68
1.077 7.635 0.988 95.273
ENSMUSG00000026278 Bok protein_coding 0.957 64.284 0.952
93.469 1.094 66.269 0.937 85.493
ENSMUSG00000026279 Thap4 protein_coding 1.065 30.984 0.906
23.703 1.038 32.173 0.97 92.466
ENSMUSG00000026280 Atg4b protein_coding 0.975 67.334 1.041
93.899 0.988 83.719 1.004 81.251
ENSMUSG00000026281 Dtymk protein_coding 1.014 80.815 0.983
95.211 1 87.898 1.028 76.697
ENSMUSG00000026283 Ing5 protein_coding 1.055 15.972 0.984
95.504 0.961 46.575 0.98 94.654
ENSMUSG00000026285 Pdcd1 protein_coding 1.097 66.58 0.836 95.211
1.093 67.339 1.267 81.251
ENSMUSG00000026288 Inpp5d protein_coding 1.223 1.323 0.888
37.426 0.919 59.726 1.189 69.404
ENSMUSG00000026289 Atg16l1 protein_coding 0.999 86.172
0.994 92.631 1.003 79.426 1.009 93.964
ENSMUSG00000026301 Iqca protein_coding 1 85.203 1
96.216 1 85.21 1 92.52
ENSMUSG00000026303 Mlph protein_coding 0.887 28.584 0.851
55.71 1.316 10.76 0.946 93.132
ENSMUSG00000026304 Rab17 protein_coding 0.881 41.759 0.863
90.046 1.317 35.894 0.943 85.493
ENSMUSG00000026305 Lrrfip1 protein_coding 1.032 53.562
0.992 96.652 0.981 48.387 1.082 52.982
ENSMUSG00000026307 Scly protein_coding 0.986 82.231 1.07
97.263 0.945 87.744 0.952 95.593
ENSMUSG00000026308 Klhl30 protein_coding 2.733 27.113
1.778 76.065 0.205 4.338 0.679 95.74
ENSMUSG00000026309 Ilkap protein_coding 1.015 72.85 0.942 52.266
1.041 27.047 1.019 78.948
ENSMUSG00000026311 Asb1 protein_coding 0.997 84.397 1.06
75.108 0.947 54.25 1.005 94.904
ENSMUSG00000026312 Cdh7 protein_coding 1 85.355 1
97.129 1 70.44 1 91.335
ENSMUSG00000026313 Hdac4 protein_coding 0.883 45.229 1.073
96.407 1.058 58.414 1.079 72.369
ENSMUSG00000026315 Serpinb8 protein_coding 1.249 0.999 0.917
90.378 0.875 13.848 1.121 60.968
ENSMUSG00000026317 Cln8 protein_coding 1.133 0.355 1.012 94.262
0.873 6.858 1.003 91.872
ENSMUSG00000026319 2310035C23Rik protein_coding 1.034 54.344
0.934 63.912 1.036 48.772 1.013 91.979
ENSMUSG00000026321 Tnfrsf11a protein_coding 1.12 4.825 1.073
88.923 0.832 3.428 1.012 76.697
ENSMUSG00000026322 Htr4 protein_coding 0.215 10.704 0.747
76.431 6.205 5.864 1.37 94.82
ENSMUSG00000026327 Serpinb11 protein_coding 1.006 72.372

0.629 85.116 1.577 84.896 2.565 77.331
ENSMUSG00000026331 Slco6c1 protein_coding 1 86.333 1
94.262 1 55.352 1 94.82
ENSMUSG00000026333 Gin1 protein_coding 0.861 10.955 1.075
76.065 1.078 54.809 1.072 83.067
ENSMUSG00000026335 Pam protein_coding 1.144 0 0.977 74.483
0.891 16.719 0.993 92.762
ENSMUSG00000026336 Slco6d1 protein_coding 1 85.946 NA
NA NA NA 1 92.52
ENSMUSG00000026339 Ccdc93 protein_coding 1.056 22.205
0.963 93.094 0.985 69.974 1.04 83.067
ENSMUSG00000026341 Actr3 protein_coding 1.173 0 0.977 83.733
0.872 0.72 1.07 65.558
ENSMUSG00000026342 Slc35f5 protein_coding 1.104 13.633
0.936 74.483 0.967 68.584 0.923 83.724
ENSMUSG00000026343 Gpr39 protein_coding 1.875 2.998 0.91 91.897
0.585 5.453 1.185 71.305
ENSMUSG00000026344 Lypd1 protein_coding 1.855 0.2 1.22 88.823
0.442 0.945 1.265 74.193
ENSMUSG00000026347 Tmem163 protein_coding 1.413 39.851
0.843 88.345 0.84 60.233 0.733 92.181
ENSMUSG00000026349 Ccnt2 protein_coding 0.937 6.677 0.95 56.781
1.126 5.864 0.978 93.156
ENSMUSG00000026353 Ubxn4 protein_coding 1.016 67.374 1.008
96.68 0.972 63.075 1.002 96.287
ENSMUSG00000026354 Lct protein_coding 0.919 55.303 0.792
91.532 1.377 56.931 1.04 93.964
ENSMUSG00000026355 Mcm6 protein_coding 1.231 4.675 0.821 56.781
0.988 60.96 1.142 81.56
ENSMUSG00000026356 Dars protein_coding 1.073 21.703 0.948
65.819 0.985 81.492 1.027 64.513
ENSMUSG00000026357 Rgs18 protein_coding 2.061 0 0.741 71.889
0.652 1.898 1.014 92.903
ENSMUSG00000026358 Rgs1 protein_coding 1.063 63.534 0.862
87.919 1.089 84.961 0.925 89.525
ENSMUSG00000026360 Rgs2 protein_coding 1.086 28.098 0.753
52.266 1.216 55.81 0.834 85.493
ENSMUSG00000026361 Cdc73 protein_coding 1.071 12.295 0.944
53.79 0.991 85.675 0.955 85.493
ENSMUSG00000026365 Cfh protein_coding 1.072 9.983 0.958 85.242
0.974 36.248 1.176 95.273
ENSMUSG00000026368 NA NA NA NA 1 88.691 1
86.985 1 94.297
ENSMUSG00000026374 Tsn protein_coding 1.148 0.664 0.99 96.407
0.879 2.563 0.992 95.273
ENSMUSG00000026377 Mki67ip protein_coding 1.18 15.178
0.988 91.661 0.859 54.521 1.066 57.1
ENSMUSG00000026380 Tfcp2l1 protein_coding 1.006 63.385
0.801 56.781 1.243 54.363 1.132 92.018
ENSMUSG00000026383 Epb4.1l5 protein_coding 1.054 82.231
0.891 79.894 1.064 67.823 0.964 92.466
ENSMUSG00000026384 Ptpn4 protein_coding 0.828 20.799 0.916
95.211 1.317 38.152 0.981 89.892
ENSMUSG00000026385 Dbi protein_coding 0.756 0.987 1.176 64.304

1.127 18.208	0.961 96.248
ENSMUSG00000026387	Sctr	protein_coding	0.976 83.418	1.154
91.065	0.884 64.427	0.723 83.724
ENSMUSG00000026388	3110009E18Rik	protein_coding	1.141 65.537
0.959 95.431	0.915 57.819	1.147 71.855
ENSMUSG00000026389	Steap3	protein_coding	1.444 0.2	1.039
96.216	0.668 0.72	1.036 40.426
ENSMUSG00000026390	Marco protein_coding	1.415 10.595	0.816
84.858	0.865 41.154	0.824 85.493
ENSMUSG00000026393	Nek7	protein_coding	0.838 34.305	0.846
37.187	1.407 10.76 1.138 87.447
ENSMUSG00000026394	Atp6v1g3	protein_coding	1	84.294	1
92.931	1	81.169	1	90.289
ENSMUSG00000026395	Ptprc protein_coding	1.37	0.2	0.89	86.071
0.818 14.788	1.024 83.581
ENSMUSG00000026398	Nr5a2 protein_coding	1.127 40.635	1.297
71.617	0.684 6.858 0.832 83.724
ENSMUSG00000026399	Cd55	protein_coding	1.028 45.61 1.072 96.407
0.907 19.552	1.065 86.301
ENSMUSG00000026404	Ddx59 protein_coding	1.11	86.199	0.93
96.216	NA	NA	0.913 85.493
ENSMUSG00000026405	C4bp	protein_coding	1	83.418	1
97.263	1	88.161	1	96.183
ENSMUSG00000026407	Cacna1s	protein_coding	7.76	5.313 1.197
83.104	0.108 0.72	0.95	95.832
ENSMUSG00000026409	Pfkfb2	protein_coding	0.948 36.945
0.965 61.625	1.093 11.518	0.94	96.183
ENSMUSG00000026411	Tmem9 protein_coding	1.07	25.026	1.001
96.001	0.931 23.212	0.932 84.29
ENSMUSG00000026413	Pkp1	protein_coding	0.751 3.009 1.064 96.843
1.254 5.864 0.91	88.053
ENSMUSG00000026414	Tnnt2 protein_coding	0.917 53.407	0.953
95.088	1.14	48.935	1.052 96.071
ENSMUSG00000026415	Fcamr protein_coding	0.557 2.644 1.006 93.146
1.779 14.376	0.65	84.29
ENSMUSG00000026416	NA	NA	NA	NA	1	94.102	NA	NA
1	95.74
ENSMUSG00000026417	Pigr	protein_coding	0.767 39.953	0.866
95.088	1.501 35.673	0.805 83.724
ENSMUSG00000026418	Tnni1 protein_coding	1.103 77.113	0.777
93.654	1.167 74.325	1.242 92.564
ENSMUSG00000026420	Il24	protein_coding	1	56.085	1
94.848	NA	NA	1	96.183
ENSMUSG00000026421	Csrp1 protein_coding	0.953 33.745	1.06
81.54 0.987 82.161	1	96.248
ENSMUSG00000026424	Gpr37l1	protein_coding	0.972 69.24 1.162
72.642	0.882 34.592	0.9	89.587
ENSMUSG00000026425	Srgap2	protein_coding	1.073 6.853 0.942
66.796	0.987 72.269	1.079 79.713
ENSMUSG00000026426	Arl8a protein_coding	0.99	79.93 1.043 90.29
0.966 81.62 0.974 94.99
ENSMUSG00000026427	Eif2d protein_coding	1.041 39.295	0.973
88.691	0.982 87.971	0.981 95.405
ENSMUSG00000026429	Ube2t protein_coding	1.043 52.134	0.832

54.427 1.149 61.841 1.445 57.839
ENSMUSG00000026430 Rassf5 protein_coding 1.451 0 0.899
60.678 0.767 0.879 1.047 93.457
ENSMUSG00000026432 Avpr1b protein_coding 1 83.523 1
97.263 1 87.103 1 95.92
ENSMUSG00000026433 Rab7l1 protein_coding 1.114 0.767 0.957
74.483 0.934 13.551 1.062 79.268
ENSMUSG00000026434 Nucks1 protein_coding 1.012 63.126
0.961 88.345 NA NA 1.047 86.118
ENSMUSG00000026435 Slc45a3 protein_coding 1.286 7.553 0.924
73.094 0.838 43.842 1.451 86.133
ENSMUSG00000026436 Elk4 protein_coding 1.063 8.685 1.021 96.984
0.921 7.742 0.955 83.724
ENSMUSG00000026437 Cdk18 protein_coding 0.759 14.427 1.033
91.643 1.275 30.561 0.96 94.215
ENSMUSG00000026439 Rbbp5 protein_coding 1.022 79.277 NA NA
1.02 86.552 1.023 77.621
ENSMUSG00000026442 Nfasc protein_coding 0.891 22.237 0.998
92.111 1.128 32.524 1.02 93.964
ENSMUSG00000026443 Lrrn2 protein_coding 1.033 52.799 1.062
97.288 0.909 33.971 0.922 94.99
ENSMUSG00000026447 Pik3c2b protein_coding 0.819 26.77 1.143
61.032 1.069 66.269 0.916 83.724
ENSMUSG00000026452 Syt2 protein_coding 0.884 82.352 1.041
97.288 1.086 86.666 1.884 83.067
ENSMUSG00000026455 Klhl12 protein_coding 1.076 43.654
0.934 68.818 0.996 86.666 0.944 85.493
ENSMUSG00000026456 Cyb5r1 protein_coding 1.058 35.748
1.028 79.411 0.921 16.949 1.041 76.831
ENSMUSG00000026457 Adipor1 protein_coding 0.992 85.283
0.957 63.912 1.055 55.81 0.965 83.952
ENSMUSG00000026458 Ppfia4 protein_coding 1.119 38.386
1.107 96.138 0.805 42.997 0.873 86.991
ENSMUSG00000026463 Atp2b4 protein_coding 1.001 65.199
1.096 96.68 0.908 84.308 0.927 84.29
ENSMUSG00000026464 Zc3h11a protein_coding 1 48.939 1
83.104 1 87.898 1 83.581
ENSMUSG00000026466 Tor1aip1 protein_coding 1.103 6.853 1.039
84.832 0.87 3.123 1.046 78.379
ENSMUSG00000026468 Lhx4 protein_coding 1 85.728 1
95.708 1 64.427 1 96.192
ENSMUSG00000026469 Xpr1 protein_coding 1.09 12.072 NA NA
0.916 4.538 0.961 96.086
ENSMUSG00000026470 Stx6 protein_coding 1.07 13.633 1.05
92.561 0.886 36.786 0.948 83.724
ENSMUSG00000026471 Mr1 protein_coding 1.068 48.462 NA NA
0.97 46.071 0.954 86.991
ENSMUSG00000026473 Glul protein_coding 0.842 75.298 0.968
78.877 1.229 24.383 1.185 55.841
ENSMUSG00000026475 Rgs16 protein_coding 0.87 21.176 1.029
71.617 1.112 78.954 0.95 91.105
ENSMUSG00000026478 Lamc1 protein_coding 0.9 16.02 0.974 92.474
1.143 33.508 1.084 32.045
ENSMUSG00000026479 Lamc2 protein_coding 1.043 45.61 1.045 85.95

0.914 83.397 0.947 95.832
ENSMUSG00000026480 Ncf2 protein_coding 1.491 0 0.862 61.883
0.78 24.163 1.079 60.968
ENSMUSG00000026482 Rgl1 protein_coding 1.049 15.972 1.072
85.756 0.89 3.428 1.205 85.577
ENSMUSG00000026483 Fam129a protein_coding 1.096 13.492
0.975 96.763 0.93 14.206 1.041 94.608
ENSMUSG00000026484 Rnf2 protein_coding 1.112 7.492 0.888 65.165
1.009 64.778 0.993 94.99
ENSMUSG00000026489 Adck3 protein_coding 1.026 78.054 0.995
95.088 0.981 88.161 0.908 90.289
ENSMUSG00000026490 Cdc42bpa protein_coding 0.876 0.987 1.025
96.843 1.11 5.864 1.037 83.067
ENSMUSG00000026491 Ahctf1 protein_coding 0.986 68.66 1.002
93.908 1.008 67.163 1.008 87.447
ENSMUSG00000026492 Tfb2m protein_coding 1.038 57.38 0.977 73.166
0.988 71.839 1.017 94.904
ENSMUSG00000026494 Kif26b protein_coding 0.98 68.66 1.037
86.387 0.983 81.388 1.274 64.905
ENSMUSG00000026495 Efcab2 protein_coding 1.201 13.734
0.946 87.452 0.875 53.7 0.776 83.724
ENSMUSG00000026496 Parp1 protein_coding 0.987 36.259 NA NA
1.011 43.842 1.014 74.047
ENSMUSG00000026497 Mixl1 protein_coding 1 85.499 1
93.654 1 84.308 1 93.734
ENSMUSG00000026499 Acbd3 protein_coding 1.011 46.875 1.006
96.843 0.985 39.755 1.081 74.193
ENSMUSG00000026500 Cox20 protein_coding 0.908 54.87 1.033 95.088
1.065 87.744 1.026 91.625
ENSMUSG00000026502 Desi2 protein_coding 1.16 0 1.017 93.994
0.847 0.72 1.041 71.653
ENSMUSG00000026504 Sdccag8 protein_coding 1.013 68.485
0.971 87.313 1.015 62.237 1.032 66.944
ENSMUSG00000026509 Capn2 protein_coding 0.95 46.413 1.056
88.092 NA NA 1.013 91.783
ENSMUSG00000026510 Trp53bp2 protein_coding 0.915 15.76 1.061
79.864 1.034 81.976 0.994 86.699
ENSMUSG00000026511 Srp9 protein_coding 1.021 63.804 0.96
72.712 1.021 83.925 1.048 64.905
ENSMUSG00000026514 Cnih3 protein_coding 0.952 83.263 0.609
95.088 1.729 71.232 1.4 93.132
ENSMUSG00000026516 Nvl protein_coding 0.974 64.893 0.979
92.137 1.045 40.511 1.039 78.948
ENSMUSG00000026519 Tmem63a protein_coding 1.141 7.959 0.998
93.27 0.881 1.764 0.998 91.105
ENSMUSG00000026520 Pycr2 protein_coding 0.943 31.11 0.983 96.68
1.074 56.708 1.047 71.008
ENSMUSG00000026523 Wdr64 protein_coding 1 69.489 1
90.138 1 76.225 1 93.156
ENSMUSG00000026525 Opn3 protein_coding 0.807 7.004 1.156 83.104
1.073 35.13 0.945 83.724
ENSMUSG00000026526 Fh1 protein_coding 0.969 54.043 1.05
85.378 0.98 87.898 1.043 82.091
ENSMUSG00000026527 Rgs7 protein_coding 0.967 85.499 1.139

80.678 0.908 30.024 NA NA
ENSMUSG00000026531 Mptx1 protein_coding 1 76.827 NA NA
1 86.346 1 90.854
ENSMUSG00000026532 Spta1 protein_coding 0.6 61.887 1.861
90.952 0.897 88.203 3.339 31.133
ENSMUSG00000026536 Mnda protein_coding 1.21 7.332 0.896 87.041
0.919 46.071 1.291 50.452
ENSMUSG00000026542 Apcs protein_coding 1 83.523 1
96.216 1 82.161 1 95.877
ENSMUSG00000026544 Dusp23 protein_coding 1.049 66.272
1.045 83.104 0.91 25.625 0.923 86.699
ENSMUSG00000026546 Ccdc19 protein_coding 0.841 37.531
0.936 93.654 1.267 36.388 1.085 95.777
ENSMUSG00000026547 Tagln2 protein_coding 0.962 69.715
0.992 95.088 1.05 77.532 1.159 48.119
ENSMUSG00000026548 Slamf9 protein_coding 1.516 0 0.894
85.075 0.737 1.871 1.184 50.74
ENSMUSG00000026553 Copa protein_coding 0.968 52.9 1.008 97.288
1.027 57.286 1.099 31.133
ENSMUSG00000026554 Dcaf8 protein_coding 0.94 13.74 0.969 75.493
1.095 7.635 0.992 91.625
ENSMUSG00000026556 Vangl2 protein_coding 0.886 16.02 1.117
79.261 1.012 85.921 0.929 86.445
ENSMUSG00000026558 Uck2 protein_coding 1.086 80.815 0.899
85.671 1.02 79.426 0.948 95.877
ENSMUSG00000026563 Tada1 protein_coding 1.031 38.195 1.014
96.216 0.957 71.388 0.995 86.209
ENSMUSG00000026564 Dusp27 protein_coding 3.921 13.363
1.615 83.104 0.158 1.061 0.868 96.248
ENSMUSG00000026565 Pou2f1 protein_coding 0.938 42.922
1.004 91.532 1.064 25.564 0.874 83.724
ENSMUSG00000026566 Mpzl1 protein_coding 0.996 68.846 0.993
95.431 1.008 88.203 0.91 83.724
ENSMUSG00000026567 Adcy10 protein_coding 0.476 4.825 1.426
82.219 1.475 23.41 1.027 96.358
ENSMUSG00000026568 Mpc2 protein_coding 0.721 1.449 1.05 91.584
1.319 14.449 1.004 94.904
ENSMUSG00000026571 Dcaf6 protein_coding 1.22 0 0.983 96.268
0.833 0.72 0.967 95.74
ENSMUSG00000026572 Tbx19 protein_coding 1 82.352 1
96.948 1 87.103 1 83.581
ENSMUSG00000026573 Xcl1 protein_coding 0.863 69.985 1.77
79.367 0.653 33.689 0.677 83.724
ENSMUSG00000026574 Dpt protein_coding 0.965 82.705 1.049
96.268 0.989 83.925 1.028 70.36
ENSMUSG00000026575 Nme7 protein_coding 1.111 25.026 0.941
92.914 0.96 32.703 1.017 96.183
ENSMUSG00000026576 Atp1b1 protein_coding 1.199 25.944
0.687 21.315 1.209 14.169 1.102 96.287
ENSMUSG00000026577 Blzf1 protein_coding 1.078 7.332 0.936 71.331
0.993 64.778 0.966 85.493
ENSMUSG00000026578 Ccdc181 protein_coding 0.912 54.87 1.021
94.887 1.071 27.732 1.031 90.455
ENSMUSG00000026579 F5 protein_coding 1.223 21.703 0.721

47.831 1.132 59.612 1.284 91.525
ENSMUSG00000026580 Selp protein_coding 1.636 0.355 1.016 93.908
0.601 0.72 NA NA
ENSMUSG00000026581 Sell protein_coding 1.213 14.966 1.292
91.643 0.635 18.965 0.851 88.505
ENSMUSG00000026582 Sele protein_coding 0.97 61.725 NA NA
1.192 86.346 0.999 95.74
ENSMUSG00000026584 Scyl3 protein_coding 0.98 70.213 0.966
74.483 1.056 14.597 1.027 95.676
ENSMUSG00000026585 Kifap3 protein_coding 1.106 2.352 0.96
79.261 0.942 17.258 1.013 92.748
ENSMUSG00000026586 Prrx1 protein_coding 1.228 12.549 1.055
88.136 0.77 2.361 1.028 82.091
ENSMUSG00000026587 Astn1 protein_coding 0.987 84.661 0.965
90.952 1.046 85.58 1.038 94.016
ENSMUSG00000026589 Sec16b protein_coding 1.239 7.397 0.931
90.046 0.866 12.41 0.938 90.805
ENSMUSG00000026594 Ralgps2 protein_coding 1.073 52.799
0.924 74.851 1.012 88.601 0.898 83.952
ENSMUSG00000026596 Abl2 protein_coding 0.998 65.869 0.942
68.818 1.063 34.07 1.009 94.608
ENSMUSG00000026600 Soat1 protein_coding 1.494 0 0.994 87.452
0.675 1.898 1.01 86.133
ENSMUSG00000026601 Axdnd1 protein_coding 1.008 69.777
0.985 96.268 1.003 70.214 0.626 83.724
ENSMUSG00000026602 Nphs2 protein_coding 0.827 83.333 0.591
79.807 2.048 49.898 1.713 71.653
ENSMUSG00000026603 Smyd2 protein_coding 0.927 11.232 1.046
88.923 1.03 51.851 1.076 37.634
ENSMUSG00000026604 Ptpn14 protein_coding 0.93 37.531
1.111 71.617 0.964 70.44 0.92 83.724
ENSMUSG00000026605 Cenpf protein_coding 1.507 32.676 0.572
20.272 1.158 72.695 1.247 75.81
ENSMUSG00000026608 Kctd3 protein_coding 0.871 1.867 1.038 96.847
1.101 11.665 NA NA
ENSMUSG00000026609 Ush2a protein_coding 1 84.819 1
94.262 1 79.426 1 83.067
ENSMUSG00000026610 Esrrg protein_coding 0.988 77.497 1.024
90.744 0.989 85.464 0.754 83.724
ENSMUSG00000026611 Spata17 protein_coding 0.846 85.283
0.802 93.654 1.468 72.148 0.975 92.675
ENSMUSG00000026615 Eprs protein_coding 1.009 82.479 0.968
96.268 1.023 82.436 1.057 38.2
ENSMUSG00000026616 Cr2 protein_coding 0.472 39.712 2.61
79.261 0.813 45.799 0.47 91.372
ENSMUSG00000026617 Bpnt1 protein_coding 0.788 0.987 1.106 79.29
1.143 17.751 1.094 78.948
ENSMUSG00000026618 Iars2 protein_coding 1.031 83.523 0.985
95.329 0.984 87.744 0.992 91.492
ENSMUSG00000026620 Mark1 protein_coding 1.103 27.113 1.004
93.817 0.904 44.678 1.038 88.111
ENSMUSG00000026621 1-Mar protein_coding 0.291 0.987 1.649 76.086
2.088 5.864 1.313 88.197
ENSMUSG00000026622 Nek2 protein_coding 1.628 14.008 0.755

55.265	0.813 81.62 1.26	85.629
ENSMUSG00000026623	Lpgat1	protein_coding	1.056 75.196
1.176 82.904	0.807 30.468	1.192 73.368
ENSMUSG00000026626	Ppp2r5a	protein_coding	0.956 21.393
1.005 85.378	1.038 62.307	1.082 72.369
ENSMUSG00000026627	Tmem206	protein_coding	1.021 84.738
0.988 96.948	0.987 72.464	1.021 91.291
ENSMUSG00000026628	Atf3	protein_coding	1.207 27.731	1.222
87.609	0.676 16.719	0.951 94.608
ENSMUSG00000026630	Batf3 protein_coding	1.114 40.88 1.071 94.96
0.837 79.255	0.908 91.372
ENSMUSG00000026632	Tatdn3	protein_coding	0.987 83.726
0.963 74.851	1.054 34.727	0.931 86.991
ENSMUSG00000026634	Angel2	protein_coding	1.069 18.067	NA
NA	0.959 20.751	1.009 82.328
ENSMUSG00000026637	Traf5 protein_coding	1.117 21.703	0.923
84.017	0.97	55.9	1.015 93.316
ENSMUSG00000026638	Irf6	protein_coding	1.019 52.134	1.032
97.263	0.948 68.584	0.956 86.991
ENSMUSG00000026639	Lamb3 protein_coding	0.801 2.142 1.1	71.617
1.135 22.517	0.918 88.053
ENSMUSG00000026640	Plxna2	protein_coding	0.982 55.641
0.918 73.166	1.113 16.666	0.914 96.086
ENSMUSG00000026641	Usf1	protein_coding	0.927 39.953	1.06
96.843	1.013 48.935	0.952 83.724
ENSMUSG00000026643	Nmt2	protein_coding	1.057 36.124	0.957
72.399	0.989 79.897	1.041 87.7
ENSMUSG00000026646	Suv39h2	protein_coding	1.112 41.211
0.989 86.861	0.909 74.535	1.24	83.067
ENSMUSG00000026648	Dclre1c	protein_coding	1.185 16.74 0.84
61.883	1.006 87.431	0.922 86.991
ENSMUSG00000026649	1700009P17Rik	protein_coding	1.214 24.481
0.837 68.115	NA	NA	0.905 84.29
ENSMUSG00000026650	NA	NA	NA	NA	0.612 87.452	1.512
72.378	0.895 89.475
ENSMUSG00000026655	Fam107b	protein_coding	1.079 78.428
1.013 85.501	0.916 27.194	0.989 92.018
ENSMUSG00000026656	Fcgr2b	protein_coding	1.48	0	0.797
35.072	0.845 32.703	1.251 57.075
ENSMUSG00000026657	Frmd4a	protein_coding	1.162 6.399 0.944
75.052	0.912 21.117	1.045 77.854
ENSMUSG00000026659	Dusp12	protein_coding	0.955 43.495
0.975 91.361	1.071 27.533	1.026 85.786
ENSMUSG00000026662	Sephs1	protein_coding	1.035 45.307
0.979 81.134	0.985 86.258	0.962 86.699
ENSMUSG00000026663	Atf6	protein_coding	1.05	29.536	1.02
95.196	0.935 15.904	1.035 81.251
ENSMUSG00000026664	Phyh	protein_coding	1.13	18.067	0.89
51.277	0.994 84.229	0.993 94.82
ENSMUSG00000026667	Uhmk1 protein_coding	1.005 86.112	0.998
95.35 NA	NA	0.979 92.181
ENSMUSG00000026669	Mcm10 protein_coding	1.184 31.477	0.974
95.504	0.87	44.211	1.993 49.514
ENSMUSG00000026670	Uap1	protein_coding	0.888 23.13 0.991 92.404

1.135 34.807 1.338 31.133
ENSMUSG00000026672 Optn protein_coding 0.831 23.556 0.992
91.83 1.207 28.74 1.15 83.276
ENSMUSG00000026674 Ddr2 protein_coding 1.087 2.723 0.953 86.604
0.961 52.087 1.013 93.612
ENSMUSG00000026675 Hsd17b7 protein_coding 0.996 66.76 1.185
71.617 0.846 7.647 1.071 93.156
ENSMUSG00000026676 Ccdc3 protein_coding 1.014 84.452 1.062
79.411 0.926 15.63 0.996 95.273
ENSMUSG00000026678 Rgs5 protein_coding 1.042 82.092 1.033
93.707 0.926 75.353 0.944 94.99
ENSMUSG00000026679 Enkur protein_coding 0.996 53.679 1.157
90.29 NA NA 1.019 90.691
ENSMUSG00000026683 Nuf2 protein_coding 1.714 34.439 0.792
79.261 0.738 65.902 1.531 63.708
ENSMUSG00000026686 Lmx1a protein_coding 0.889 83.333 NA NA
0.954 88.601 1.092 94.904
ENSMUSG00000026687 Aldh9a1 protein_coding 1.029 46.097
0.952 91.361 1.022 82.903 1.025 95.777
ENSMUSG00000026688 Mgst3 protein_coding 1.124 61.725 0.926
94.597 0.956 85.21 0.928 90.157
ENSMUSG00000026691 Fmo3 protein_coding 0.715 54.604 0.729
90.256 1.912 27.431 1.173 77.854
ENSMUSG00000026692 Fmo4 protein_coding 0.864 50.519 0.684
85.242 1.691 20.315 1.301 58.557
ENSMUSG00000026694 Mettl13 protein_coding 1.067 53.562
0.964 87.452 0.974 88.693 0.976 95.273
ENSMUSG00000026696 Vamp4 protein_coding 1.056 14.59 0.931 72.576
1.013 73.531 1.008 95.273
ENSMUSG00000026697 Myoc protein_coding 0.692 8.938 1.287 82.904
1.125 80.089 1.021 91.985
ENSMUSG00000026698 Pigc protein_coding 0.948 44.562 0.993
88.881 1.06 53.882 1.056 81.56
ENSMUSG00000026701 Prdx6 protein_coding 0.943 16.652 0.975
87.452 1.09 15.688 1.038 93.132
ENSMUSG00000026705 Klhl20 protein_coding 1.005 84.324
0.986 86.277 1.006 70.9 1.05 48.857
ENSMUSG00000026707 Nsun6 protein_coding 1.04 78.538 0.881
72.399 1.086 16.808 0.974 86.445
ENSMUSG00000026708 Cenpl protein_coding 1.126 11.928 0.949
85.242 0.933 58.271 1.095 79.713
ENSMUSG00000026709 Dars2 protein_coding 0.951 59.028 1.031
87.604 1.016 82.903 1.024 92.381
ENSMUSG00000026712 Mrc1 protein_coding 1.34 0 0.926 85.793
0.806 2.563 1.243 46.846
ENSMUSG00000026715 Serpinc1 protein_coding 0.859 43.435 0.79
87.313 1.472 27.533 1.003 93.722
ENSMUSG00000026718 Stam protein_coding 1.084 6.853 1.028 96.897
0.894 7.083 0.929 84.29
ENSMUSG00000026721 Rabgap1l protein_coding 1.004 77.916
0.963 73.766 1.03 49.601 1.004 95.832
ENSMUSG00000026723 Trdmt1 protein_coding 1.144 30.984 0.93
84.858 0.937 86.552 1.016 92.018
ENSMUSG00000026726 Cubn protein_coding 1.151 41.177 1.012

96.984 0.858 49.733 1.207 90.865
ENSMUSG00000026727 Rsu1 protein_coding 0.956 8.122 1.101 60.309
0.949 65.409 0.971 91.928
ENSMUSG00000026728 Vim protein_coding 0.947 65.869 NA NA
1.077 88.601 1.239 35.053
ENSMUSG00000026729 4930562F07Rik protein_coding 0.748 14.427
1.074 95.301 1.248 17.412 0.858 85.493
ENSMUSG00000026730 Pter protein_coding 1.066 46.013 0.942
61.883 0.997 79.426 1.005 95.92
ENSMUSG00000026734 4921504E06Rik protein_coding 1 81.657
1 94.848 1 85.675 1 95.273
ENSMUSG00000026735 Ptf1a protein_coding 1 86.006 1
93.654 NA NA 1 95.74
ENSMUSG00000026736 4930426L09Rik protein_coding 0.955 74.021
0.937 84.594 1.119 68.129 1.115 95.74
ENSMUSG00000026737 Pip4k2a protein_coding 1.336 0 0.932
68.115 0.799 1.705 1.037 95.74
ENSMUSG00000026739 Bmi1 protein_coding 1.176 0 1.056 81.039
0.806 0.72 0.902 83.724
ENSMUSG00000026740 Dnajc1 protein_coding 1.014 53.228
0.981 95.708 1.001 73.598 1.026 96.086
ENSMUSG00000026743 Mllt10 protein_coding 0.964 16.02 1.021
83.104 1.017 66.043 0.945 83.724
ENSMUSG00000026748 Plxdc2 protein_coding 1.134 7.255 0.944
82.503 0.931 66.425 0.967 96.086
ENSMUSG00000026749 Nek6 protein_coding 1.02 73.841 1.101
83.104 0.887 36.499 1.568 73.368
ENSMUSG00000026750 Psmb7 protein_coding 0.856 0.987 1.05 90.092
1.109 10.523 1.126 63.708
ENSMUSG00000026753 Ppp6c protein_coding 1.08 0.664 1.043 96.763
0.891 1.061 0.97 88.387
ENSMUSG00000026754 Golga1 protein_coding 1.014 73.124
1.017 95.301 0.968 87.971 1.001 95.877
ENSMUSG00000026755 Arpc5l protein_coding 0.968 57.818
1.071 86.156 0.968 88.41 1.042 69.708
ENSMUSG00000026761 Orc4 protein_coding 1.07 18.388 1.005
91.661 0.932 33.971 0.968 90.854
ENSMUSG00000026764 Kif5c protein_coding 1.166 21.836 1.04
95.431 0.824 25.625 1.045 70.539
ENSMUSG00000026765 Lypd6b protein_coding 0.817 32.557
1.196 83.104 NA NA 1.126 96.358
ENSMUSG00000026766 Mmadhc protein_coding 1.004 85.77 1.02
96.407 0.973 74.179 1.081 51.901
ENSMUSG00000026767 Fam188a protein_coding 0.948 24.748
0.975 96.652 1.079 45.208 1.021 85.786
ENSMUSG00000026768 Itga8 protein_coding 1.233 1.395 0.997 90.046
0.816 9.456 0.835 83.724
ENSMUSG00000026770 Il2ra protein_coding 1.254 23.019 1.048
95.708 0.757 31.135 1.033 96.358
ENSMUSG00000026771 Spopl protein_coding 1.046 52.986 0.957
87.452 1 88.601 1.003 95.74
ENSMUSG00000026773 Pfkfb3 protein_coding 1.358 0.461 1.05
97.263 0.702 3.969 0.907 90.157
ENSMUSG00000026775 Yme1l1 protein_coding 1.112 2.085 0.987

95.708	0.909 2.563 1.005 83.581
ENSMUSG00000026778	Prkcq protein_coding	1.458 3.152 1.157 79.411
0.591 0.72	0.863 90.805
ENSMUSG00000026779	Mastl protein_coding	1.578 23.493	0.761
66.651	0.831 78.954	1.429 68.696
ENSMUSG00000026781	Acbd5 protein_coding	1.024 39.075	1.003
88.533	0.974 14.409	0.983 91.078
ENSMUSG00000026782	Abi2	protein_coding	1.17	0.461 0.941 79.807
0.909 10.362	1.08	64.513
ENSMUSG00000026784	Pdss1 protein_coding	0.862 26.217	1.12
88.923	1.032 76.923	1.201 50.452
ENSMUSG00000026785	Pkn3	protein_coding	0.921 8.139 0.915 90.046
1.183 16.808	0.997 94.384
ENSMUSG00000026786	Apbb1ip	protein_coding	1.147 10.464
1.052 96.138	0.831 14.409	1.251 32.045
ENSMUSG00000026787	Gad2	protein_coding	1	85.832	1
95.211	1	83.719	NA	NA
ENSMUSG00000026788	Zbtb43	protein_coding	1.004 86.351
0.887 24.911	1.123 10.76 1.019 88.197
ENSMUSG00000026790	Odf2	protein_coding	1.006 84.263	0.947
66.796	1.046 25.966	0.984 95.593
ENSMUSG00000026791	NA	NA	NA	NA	1.056 83.104	0.974
36.499	0.952 85.493
ENSMUSG00000026792	Lrsam1	protein_coding	1.004 80.251
0.981 97.155	1.012 79.554	0.986 92.31
ENSMUSG00000026796	Fam129b	protein_coding	0.995 81.01 0.908
24.911	1.107 8.209 1.029 84.31
ENSMUSG00000026797	Stxbp1	protein_coding	1.115 14.205	1.08
66.01 0.832 0.879 1.008 94.016
ENSMUSG00000026798	Coq4	protein_coding	0.903 16.183	1.083
82.904	1.018 66.043	1.01	83.276
ENSMUSG00000026799	Med27 protein_coding	0.987 67.163	1.052
94.367	0.966 82.74 1.039 76.697
ENSMUSG00000026803	Ttf1	protein_coding	0.996 85.728	0.987
95.211	1.016 84.626	1.003 95.593
ENSMUSG00000026806	Ddx31 protein_coding	1.119 20.659	0.944
85.671	0.947 67.648	1.076 78.948
ENSMUSG00000026807	Ak8	protein_coding	1.727 1.493 0.767 68.115
0.753 59.922	0.976 93.117
ENSMUSG00000026809	1700026L06Rik	protein_coding	2.274 7.501 0.62
78.877	0.709 24.163	1.156 89.052
ENSMUSG00000026810	Dpm2	protein_coding	0.9	10.29 1.033 93.574
1.072 48.451	1.061 69.404
ENSMUSG00000026811	St6galnac6 protein_coding	1.205 0.664 0.898
85.418	0.921 8.192 1.042 68.696
ENSMUSG00000026812	Tsc1	protein_coding	1.014 56.66 0.97	82.848
1.013 80.749	1	88.245
ENSMUSG00000026814	Eng	protein_coding	0.898 9.341 0.946 91.532
1.175 48.451	0.98	92.181
ENSMUSG00000026815	Gfi1b protein_coding	1.644 5.657 0.56	39.056
1.085 78.498	1.079 77.621
ENSMUSG00000026816	Gtf3c5	protein_coding	0.955 29.21 0.993
83.104	1.057 64.305	1.005 96.358
ENSMUSG00000026817	Ak1	protein_coding	1.728 14.966	1.209

82.904 0.48 0.72 1.073 90.455
ENSMUSG00000026818 Cel protein_coding 1.046 83.968 0.77
88.881 1.24 69.315 1.575 95.273
ENSMUSG00000026819 Slc25a25 protein_coding 1.046 64.714
1.011 97.155 0.945 69.974 1.122 95.74
ENSMUSG00000026820 Ptges2 protein_coding 0.855 1.031 1.07
82.103 1.096 38.099 0.946 86.991
ENSMUSG00000026821 Ralgds protein_coding 1.033 51.737
0.994 94.597 0.972 66.425 0.953 90.691
ENSMUSG00000026822 Lcn2 protein_coding 0.787 11.726 0.845
69.175 1.506 5.864 0.913 87.705
ENSMUSG00000026825 Dnm1 protein_coding 1.245 0.577 1.018 96.847
0.786 2.563 1.188 31.133
ENSMUSG00000026826 Nr4a2 protein_coding 1.005 79.277 0.98
92.062 1.015 86.727 1.077 88.797
ENSMUSG00000026827 Gpd2 protein_coding 0.765 1.031 1.038 90.29
1.256 27.431 0.919 96.192
ENSMUSG00000026828 Galnt5 protein_coding 1.727 14.34 1.25
90.092 0.464 3.718 1.244 67.691
ENSMUSG00000026829 Gbgt1 protein_coding 1.433 0.461 0.906 88.704
0.77 6.858 1.165 76.697
ENSMUSG00000026830 Ermn protein_coding 1 83.726 1
96.68 1 76.64 1 92.748
ENSMUSG00000026831 1700007K13Rik protein_coding 0.85 66.406
0.705 95.708 1.672 59.612 0.842 90.289
ENSMUSG00000026832 Cytip protein_coding 1.014 86.57 0.896 72.576
1.095 38.152 0.85 84.29
ENSMUSG00000026833 Olfm1 protein_coding 0.881 13.883 1.082
80.277 1.046 72.853 1.091 57.075
ENSMUSG00000026834 Acvr1c protein_coding 1.123 55.124
0.911 93.654 0.979 88.601 0.802 83.724
ENSMUSG00000026835 Fcnb protein_coding 1 28.584 1
88.551 1 80.089 1 94.904
ENSMUSG00000026836 Acvr1 protein_coding 1.17 3.498 0.942 81.02
0.905 19.449 0.998 93.964
ENSMUSG00000026837 NA NA NA NA 0.972 97.263 1.059
67.648 1.348 31.133
ENSMUSG00000026839 Upp2 protein_coding 0.819 17.898 0.954
71.41 1.282 10.523 0.992 95.74
ENSMUSG00000026840 Lamc3 protein_coding 1.041 60.853 1.054
87.609 0.912 34.21 1.34 92.018
ENSMUSG00000026842 Abl1 protein_coding 0.975 54.604 1.011
95.211 1.017 87.786 1.017 94.904
ENSMUSG00000026843 Fubp3 protein_coding 1.033 71.896 0.966
70.29 0.998 38.683 1.007 92.748
ENSMUSG00000026848 Tor1b protein_coding 1.074 11.928 0.996
95.504 0.931 3.943 0.998 85.577
ENSMUSG00000026849 Tor1a protein_coding 1.003 72.85 1.044 82.904
0.952 22.279 0.931 83.724
ENSMUSG00000026851 BC005624 protein_coding 0.882 0.987 0.95
71.889 1.189 5.864 1.029 60.968
ENSMUSG00000026853 Crat protein_coding 1.091 66.272 0.968
95.504 0.948 82.31 0.874 84.29
ENSMUSG00000026854 Usp20 protein_coding 0.945 4.825 1.045 83.104

1.016 29.777 0.97 96.192
ENSMUSG00000026856 Dolpp1 protein_coding 0.947 27.968
1.144 63.504 0.919 35.559 0.961 86.699
ENSMUSG00000026857 Ntmt1 protein_coding 0.98 69.985 0.93
79.475 1.091 29.819 1.014 92.903
ENSMUSG00000026858 Fam73b protein_coding 0.83 0.987 0.998
91.643 1.204 6.506 0.986 90.157
ENSMUSG00000026860 Sh3glb2 protein_coding 0.861 1.017 1.021
83.104 1.139 5.964 0.917 83.724
ENSMUSG00000026864 Hspa5 protein_coding 0.932 76.968 0.984
94.89 1.094 85.921 1.186 40.127
ENSMUSG00000026866 Kynu protein_coding 1.032 79.433 0.341
54.427 2.837 20.556 0.607 87.496
ENSMUSG00000026867 Gapvd1 protein_coding 1.048 29.057
0.991 87.452 0.959 88.274 0.976 87.331
ENSMUSG00000026869 Psmd5 protein_coding 1.045 48.462 1.021
96.216 0.935 55.214 0.998 93.722
ENSMUSG00000026870 Cutal protein_coding 0.719 30.035 0.832
63.619 1.678 10.281 1.064 93.132
ENSMUSG00000026872 Zeb2 protein_coding 0.928 64.284 1.138
83.104 0.946 69.583 0.918 85.493
ENSMUSG00000026873 Phf19 protein_coding 0.88 60.339 1.074
95.211 1.056 76.498 1.378 31.133
ENSMUSG00000026874 Hc protein_coding 1.052 85.59 0.808 96.216
1.18 86.985 0.94 88.136
ENSMUSG00000026875 Traf1 protein_coding 1.072 51.189 1.04
92.137 0.896 73.027 0.781 83.724
ENSMUSG00000026878 Rab14 protein_coding 1.074 5.253 1.024 96.68
0.907 6.202 1.008 85.786
ENSMUSG00000026879 Gsn protein_coding 0.986 84.049 1.04
95.809 0.977 67.339 0.878 88.387
ENSMUSG00000026880 Stom protein_coding 0.945 69.985 1.103
82.904 0.957 74.853 0.994 90.854
ENSMUSG00000026882 4930568D16Rik protein_coding 1 74.482
1 93.654 1 82.778 1 95.877
ENSMUSG00000026883 Dab2ip protein_coding 0.901 5.084 1.02
88.551 1.085 50.851 1.025 92.018
ENSMUSG00000026885 Ttll11 protein_coding 0.898 9.863 1.241
79.411 0.894 82.74 0.834 91.625
ENSMUSG00000026887 Mrrf protein_coding 0.92 2.174 1.041 74.663
1.043 60.104 1.029 83.067
ENSMUSG00000026888 Grb14 protein_coding 0.993 57.014 0.774
73.166 1.305 22.444 1.226 78.546
ENSMUSG00000026889 Rbm18 protein_coding 1.011 69.24 0.956 75.052
1.036 29.819 0.981 89.108
ENSMUSG00000026890 Lhx6 protein_coding 0.907 22.481 1.058
93.707 1.039 44.787 0.981 93.685
ENSMUSG00000026893 Gca protein_coding 1.002 71.896 1.02
96.407 0.979 66.201 0.952 84.29
ENSMUSG00000026894 Morn5 protein_coding 0.631 40.422 1.831
86.793 0.867 80.163 0.676 91.105
ENSMUSG00000026895 Ndufa8 protein_coding 0.921 11.726
0.981 96.68 1.108 26.498 1.073 94.297
ENSMUSG00000026896 Ifih1 protein_coding 1.099 0.2 1.091 90.389

0.834 3.969 1.074 87.134
ENSMUSG00000026904 Slc4a10 protein_coding 1.2 51.826
1.082 95.431 0.769 52.658 0.908 96.358
ENSMUSG00000026914 Psmd14 protein_coding 0.884 3.763 1.07
87.037 1.056 25.93 1.256 32.698
ENSMUSG00000026915 Strbp protein_coding 0.938 56.768 0.974
62.692 1.097 25.564 0.954 94.654
ENSMUSG00000026917 Wdr5 protein_coding 1.031 50.377 0.981
90.256 0.984 68.266 1.005 92.181
ENSMUSG00000026918 Brd3 protein_coding 0.945 8.122 0.998 89.996
1.056 32.173 0.96 84.19
ENSMUSG00000026921 Egfl7 protein_coding 0.785 4.468 1.023 96.68
1.245 5.964 0.951 91.979
ENSMUSG00000026922 Agpat2 protein_coding 0.867 33.002
1.196 79.261 0.963 78.045 0.965 90.854
ENSMUSG00000026923 Notch1 protein_coding 0.914 12.084
1.043 94.367 1.05 41.331 0.917 84.19
ENSMUSG00000026924 Sec16a protein_coding 0.946 33.224 1.04
72.546 1.018 79.426 1.046 86.301
ENSMUSG00000026925 Inpp5e protein_coding 0.868 0.987 1.067
75.108 1.076 30.107 0.974 86.254
ENSMUSG00000026926 Pmpca protein_coding 0.986 68.955 0.996
95.431 1.015 83.538 1.028 83.067
ENSMUSG00000026927 Sdccag3 protein_coding 0.928 30.136
1.077 82.76 1.001 87.744 1.051 60.31
ENSMUSG00000026928 Card9 protein_coding 1.305 0.976 0.967 87.147
0.788 30.024 1.171 32.045
ENSMUSG00000026930 Gpsm1 protein_coding 0.888 30.352 0.987
97.155 1.142 49.384 1.02 94.19
ENSMUSG00000026931 1700019N19Rik protein_coding 0.673 57.544
2.117 92.561 0.698 87.274 2.008 91.011
ENSMUSG00000026932 Nacc2 protein_coding 1.062 21.836 1.162
63.504 0.811 0.72 0.972 83.952
ENSMUSG00000026933 Camsap1 protein_coding 1.003 51.297
1.067 89.494 0.937 20.751 0.99 95.593
ENSMUSG00000026936 Lcn3 protein_coding 1 77.407 1
96.216 1 88.076 1 96.287
ENSMUSG00000026937 Lcn5 protein_coding 1 62.007 1
93.817 NA NA 1 95.273
ENSMUSG00000026938 Fcna protein_coding 1.77 0 0.574 12.804
0.985 68.266 1.134 44.213
ENSMUSG00000026939 Tmem141 protein_coding 0.97 86.819
1.059 79.29 0.974 48.133 1.047 77.621
ENSMUSG00000026940 4921530D09Rik protein_coding 0.553 6.221
1.139 97.155 1.584 13.174 1.326 64.513
ENSMUSG00000026941 Mamdc4 protein_coding 0.857 14.075
1.122 83.104 1.041 30.292 0.84 85.493
ENSMUSG00000026942 Traf2 protein_coding 0.923 20.116 1.048
71.617 1.034 85.935 0.99 86.699
ENSMUSG00000026944 Abca2 protein_coding 0.877 0.987 1.096 74.663
1.041 37.369 0.985 96.358
ENSMUSG00000026946 Nmi protein_coding 1.148 2.361 0.959 79.807
0.908 24.523 1.042 83.067
ENSMUSG00000026950 Neb protein_coding 7.093 5.481 0.842 87.029

0.168 0.72 0.925 95.777
ENSMUSG00000026955 Sapcd2 protein_coding 1.146 77.291
0.669 72.399 1.301 74.686 1.87 70.539
ENSMUSG00000026956 Uap1l1 protein_coding 1.496 0 0.84
61.883 0.797 1.871 0.889 90.95
ENSMUSG00000026958 Dpp7 protein_coding 1.048 19.746 0.989
91.532 0.96 33.402 1.061 92.018
ENSMUSG00000026959 Grin1 protein_coding 0.714 44.252 0.935
92.914 1.493 38.038 1.162 92.564
ENSMUSG00000026960 Arl6ip6 protein_coding 1.055 17.582
0.993 96.68 0.953 21.331 0.977 84.29
ENSMUSG00000026961 Lrrc26 protein_coding 0.996 63.385
0.596 24.911 1.681 32.173 0.919 87.651
ENSMUSG00000026963 Tmem210 protein_coding 1 71.674 1
91.275 1 62.237 1 87.447
ENSMUSG00000026965 Anapc2 protein_coding 0.999 78.299
1.026 82.904 0.978 66.753 0.988 93.261
ENSMUSG00000026966 Ssna1 protein_coding 0.893 7.218 0.986 95.504
1.139 8.586 1.109 31.133
ENSMUSG00000026969 Fam166a protein_coding 0.492 0.987 1.419
40.99 1.433 21.873 1.075 95.74
ENSMUSG00000026970 Rbms1 protein_coding 1.071 2.998 1.005 88.691
0.924 11.314 1.07 77.854
ENSMUSG00000026971 Itgb6 protein_coding 0.991 86.006 0.861
95.504 1.168 84.961 1.019 96.086
ENSMUSG00000026972 Arrdc1 protein_coding 0.918 45.502 0.96
93.654 1.136 33.508 0.994 91.078
ENSMUSG00000026974 Zmynd19 protein_coding 0.985 86.351
1.053 97.155 0.967 87.37 1.054 80.573
ENSMUSG00000026975 Dph7 protein_coding 1.006 72 0.92 64.555
1.076 46.206 0.987 95.273
ENSMUSG00000026976 Pax8 protein_coding 1 58.077 1
96.407 1 70.623 1 94.904
ENSMUSG00000026977 7-Mar protein_coding 0.99 85.857 0.974
85.242 1.039 55.81 0.894 83.724
ENSMUSG00000026979 Psd4 protein_coding 1.021 80.408 0.956
81.731 1.028 65.8 0.978 95.273
ENSMUSG00000026980 Ly75 protein_coding 1.006 70.121 0.86
75.052 1.157 67.823 0.813 86.991
ENSMUSG00000026981 Il1rn protein_coding 1.96 13.734 0.697
80.463 0.73 60.233 1.653 57.075
ENSMUSG00000026984 Il1f6 protein_coding 1 84.294 1
96.378 NA NA 1 95.877
ENSMUSG00000026985 Il1f8 protein_coding 1 81.01 NA NA 1
84.229 1 96.183
ENSMUSG00000026986 Hnmt protein_coding 0.966 81.01 1.112 79.864
0.933 30.827 0.976 88.387
ENSMUSG00000026987 Baz2b protein_coding 0.957 39.612 0.988
92.137 1.059 9.176 0.908 87.735
ENSMUSG00000026988 Wdsub1 protein_coding 1.057 19.315
1.067 72.642 0.883 2.333 1.001 93.584
ENSMUSG00000026991 Pkp4 protein_coding 0.882 20.369 0.956
79.475 1.189 22.994 0.934 85.493
ENSMUSG00000026994 Galnt3 protein_coding 0.948 72.985

1.084 83.104 0.974 75.967 0.903 87.705
ENSMUSG00000026999 Nup35 protein_coding 1.036 52.986 1.102
71.617 0.875 3.123 1.016 90.865
ENSMUSG00000027001 Dusp19 protein_coding 0.996 78.054
1.167 72.642 0.862 27.675 1.078 88.512
ENSMUSG00000027002 Nckap1 protein_coding 0.967 80.608
1.043 80.678 0.988 54.964 0.991 95.72
ENSMUSG00000027004 Frzb protein_coding 1.349 20.032 1.044
96.843 0.71 18.797 1.043 96.248
ENSMUSG00000027006 Dnajc10 protein_coding 1.022 39.075
0.976 67.839 1.003 84.308 1.014 62.918
ENSMUSG00000027007 Ssfa2 protein_coding 1.053 26.136 0.993
96.68 0.952 12.948 0.976 90.611
ENSMUSG00000027009 Itga4 protein_coding 1.381 0 1.057 95.708
0.686 7.809 1.083 91.525
ENSMUSG00000027010 Slc25a12 protein_coding 1.217 1.712 0.947
90.287 0.869 0.72 1.102 48.857
ENSMUSG00000027011 Ube2e3 protein_coding 1.156 0 1.016
95.735 0.849 0.72 0.978 93.612
ENSMUSG00000027012 Dync1i2 protein_coding 1.021 80.815
0.951 90.256 1.027 81.032 1.014 63.708
ENSMUSG00000027014 Cwc22 protein_coding 0.968 65.733 0.926
15.938 1.118 7.635 1.045 91.492
ENSMUSG00000027015 Cybrd1 protein_coding 1.282 22.025
0.825 84.858 0.946 69.134 0.83 84.29
ENSMUSG00000027016 Zfp385b protein_coding 1.497 25.735
0.974 95.088 0.687 25.625 1.17 86.133
ENSMUSG00000027018 Hat1 protein_coding 1.14 11.316 0.967
86.538 0.905 18.539 1.178 43.377
ENSMUSG00000027022 Xirp2 protein_coding 6.553 4.362 1.004 87.185
0.152 0.72 1.035 96.248
ENSMUSG00000027030 Stk39 protein_coding 0.964 15.227 1.055
72.546 0.979 83.397 1.118 57.1
ENSMUSG00000027035 Cers6 protein_coding 1.102 31.477 1
95.941 0.908 46.32 1.051 86.445
ENSMUSG00000027048 Abcb11 protein_coding 0.868 65.869
0.943 87.452 1.217 59.274 1.347 67.691
ENSMUSG00000027067 Ssrp1 protein_coding 0.968 35.646 0.988
83.392 1.044 24.029 1.057 31.133
ENSMUSG00000027068 Dhrs9 protein_coding 1.028 84.564 1.203
80.678 0.809 47.641 1.341 89.052
ENSMUSG00000027070 Lrp2 protein_coding 0.992 67.334 0.836
91.532 1.202 70.534 0.903 95.92
ENSMUSG00000027071 P2rx3 protein_coding 0.933 84.049 1.085
95.431 0.988 88.203 0.68 77.331
ENSMUSG00000027073 Prg2 protein_coding 0.695 48.646 1.446
83.819 0.992 85.792 0.986 96.287
ENSMUSG00000027074 Slc43a3 protein_coding 0.841 34.795
0.794 85.793 1.497 15.185 1.039 72.826
ENSMUSG00000027075 Slc43a1 protein_coding 0.926 76.396
0.954 88.705 1.135 33.787 0.87 83.724
ENSMUSG00000027076 Timm10 protein_coding 0.915 37.82 0.981
92.545 1.107 38.152 1.017 83.067
ENSMUSG00000027078 Ube2l6 protein_coding 1.18 3.598 1.12

91.456 0.753 16.949 1.356 60.968
ENSMUSG00000027079 Clp1 protein_coding 0.953 67.867 0.935
84.858 1.125 46.956 1.037 83.067
ENSMUSG00000027080 Med19 protein_coding 1.009 69.489 1.091
71.222 0.91 6.381 1.05 70.85
ENSMUSG00000027082 Tfpi protein_coding 1.219 7.553 1.027 91.643
0.799 10.23 1.056 93.132
ENSMUSG00000027086 Fastkd1 protein_coding 1.004 60.569
1.034 90.895 0.961 80.015 0.934 87.218
ENSMUSG00000027087 Itgav protein_coding 1.18 7.799 0.99 97.263
0.854 14.788 1.003 90.674
ENSMUSG00000027088 Phospho2 protein_coding 1.071 26.863 0.98
95.708 0.949 20.751 0.983 96.086
ENSMUSG00000027091 Zc3h15 protein_coding 1.144 2.569 0.898
20.272 0.974 74.853 0.982 90.397
ENSMUSG00000027099 Mtx2 protein_coding 0.983 83.263 0.965
85.116 1.056 38.099 1.036 88.197
ENSMUSG00000027102 Hoxd8 protein_coding 0.974 44.467 1.082
89.434 0.951 84.896 0.873 85.493
ENSMUSG00000027104 Atf2 protein_coding 1.114 1.43 1.053 86.277
0.854 0.72 0.968 91.625
ENSMUSG00000027107 Chrna1 protein_coding 1.561 55.462
1.358 91.643 0.472 32.082 1.516 90.865
ENSMUSG00000027108 Ola1 protein_coding 0.998 52.726 0.948
85.116 1.055 36.011 1.044 56.079
ENSMUSG00000027109 Sp3 protein_coding 1.101 0.577 1.041 83.104
0.87 0.879 0.98 93.316
ENSMUSG00000027111 Itga6 protein_coding 1.102 6.541 NA NA
0.919 17.898 0.965 85.493
ENSMUSG00000027115 Kif18a protein_coding 1.454 26.338
0.727 60.678 0.942 87.519 1.017 92.381
ENSMUSG00000027120 Fshb protein_coding 1 83.169 1
95.329 1 82.685 1 94.567
ENSMUSG00000027122 Arl14ep protein_coding 1.002 43.019
1.034 94.887 0.968 79.255 1.091 88.111
ENSMUSG00000027130 Slc12a6 protein_coding 0.989 70.36 1.015
92.914 0.992 87.786 0.932 83.724
ENSMUSG00000027131 Emc4 protein_coding 0.857 0.987 1.04 76.634
1.123 16.666 1.006 94.384
ENSMUSG00000027132 Katnbl1 protein_coding 1.127 12.295
1.014 95.431 0.876 14.409 1.024 83.067
ENSMUSG00000027133 Nop10 protein_coding 1.003 44.922 1.066
80.775 0.932 81.169 0.905 86.991
ENSMUSG00000027134 Lpcat4 protein_coding 0.941 35.894 0.92
83.392 1.157 22.93 1 87.134
ENSMUSG00000027157 4930430A15Rik protein_coding 1 85.728
1 95.301 1 85.792 1 91.65
ENSMUSG00000027160 Ccdc34 protein_coding 1.013 69.489
1.031 94.102 0.959 88.161 0.998 91.525
ENSMUSG00000027162 Lin7c protein_coding 1.059 24.253 0.911
53.79 1.039 79.426 1.042 82.328
ENSMUSG00000027163 Commd9 protein_coding 0.881 7.58 1.045
79.261 1.088 58.616 1.074 74.896
ENSMUSG00000027164 Traf6 protein_coding 1.033 43.654 1.023

89.494 0.944 21.331 0.995 88.387
ENSMUSG00000027165 B230118H07Rik protein_coding 0.971 85.77
1.033 95.211 1 84.896 1.107 44.882
ENSMUSG00000027166 Dnajc24 protein_coding 1.103 32.238
1.074 92.111 0.841 29.451 1.077 73.939
ENSMUSG00000027167 Elp4 protein_coding 1.046 29.372 1
96.216 0.956 54.964 0.902 83.724
ENSMUSG00000027170 Eif3m protein_coding 1.168 1.292 0.878 25.341
0.977 54.037 0.972 95.877
ENSMUSG00000027171 Prrg4 protein_coding 1.057 81.24 0.862 79.261
1.095 69.315 0.898 92.851
ENSMUSG00000027173 Depdc7 protein_coding 1.062 21.836
0.825 62.025 1.14 70.1 1.003 93.964
ENSMUSG00000027175 Tcp11l1 protein_coding 1.067 69.408
1.014 95.735 0.925 63.504 0.882 85.493
ENSMUSG00000027176 Cstf3 protein_coding 0.996 52.018 0.917
68.115 1.096 14.597 0.998 95.877
ENSMUSG00000027177 Hipk3 protein_coding 1.091 2.352 1.021 96.897
0.895 6.858 0.945 87.496
ENSMUSG00000027180 Fbxo3 protein_coding 1.002 64.714 0.931
87.857 1.068 88.729 1.033 86.118
ENSMUSG00000027184 Caprin1 protein_coding 1.019 63.736
1.027 88.823 0.952 84.308 1.027 46.412
ENSMUSG00000027185 Nat10 protein_coding 0.946 43.435 0.996
94.96 1.059 40.875 1.09 42.736
ENSMUSG00000027186 Elf5 protein_coding 0.879 41.021 1.003
91.661 1.133 36.011 0.92 90.611
ENSMUSG00000027187 Cat protein_coding 1.116 56.66 0.92 88.897
NA NA 0.972 95.72
ENSMUSG00000027188 Pamr1 protein_coding 1.133 61.49 0.74 55.265
1.187 50.091 0.714 83.724
ENSMUSG00000027189 Trim44 protein_coding 1.012 45.746
1.006 95.735 0.978 33.137 0.977 90.805
ENSMUSG00000027193 Api5 protein_coding 1.032 32.676 1.04
92.062 0.933 8.027 1.031 83.067
ENSMUSG00000027194 Ttc17 protein_coding 1.037 51.297 0.978
95.301 0.981 54.037 0.982 96.183
ENSMUSG00000027195 Hsd17b12 protein_coding 0.842 14.221
1.074 81.54 1.104 48.304 1.027 93.264
ENSMUSG00000027196 4921507L20Rik processed_transcript 1.236
85.391 0.836 93.469 0.969 73.428 NA NA
ENSMUSG00000027198 Ext2 protein_coding 1.103 0.984 0.994 93.469
0.909 5.336 1.01 91.928
ENSMUSG00000027199 Gatm protein_coding 1.421 0 0.974 97.129
0.721 0.72 0.925 86.699
ENSMUSG00000027200 Sema6d protein_coding 0.935 23.319
1.033 72.546 1.034 82.575 0.964 96.183
ENSMUSG00000027201 Myef2 protein_coding 0.914 22.481 0.948
88.691 1.152 20.572 1.136 44.882
ENSMUSG00000027202 Slc12a1 protein_coding 1 53.679 NA
NA 1 84.229 1 94.19
ENSMUSG00000027203 Dut protein_coding 1.022 43.019 0.951
78.877 1.028 83.925 1.179 42.736
ENSMUSG00000027204 Fbn1 protein_coding 1.276 25.735 0.919

75.345	0.853	62.791	1.014	92.018
ENSMUSG00000027206	Cops2	protein_coding	1.155	2.361	0.937	64.123	0.926	34.592	0.945	90.95
ENSMUSG00000027207	Galk2	protein_coding	0.999	79.068	0.977	89.509	1.02	54.363	1.012	86.445
ENSMUSG00000027208	Fgf7	protein_coding	1.179	6.958	1.085	79.411	0.779	1.898	1.169	43.377
ENSMUSG00000027209	Fam227b	protein_coding	1.58	6.853	0.745	74.483	0.847	23.86	0.804	83.724
ENSMUSG00000027210	Meis2	protein_coding	1.16	19.746	1.018	95.211	0.844	4.23	1.053	89.052
ENSMUSG00000027215	Cd82	protein_coding	1.071	33.854	0.987	92.545	0.945	59.383	0.961	92.181
ENSMUSG00000027217	Tspan18	protein_coding	1.222	26.338	1.011	82.904	0.807	2.563	0.918	91.105
ENSMUSG00000027219	Slc28a2	protein_coding	1.226	2.651	1.026	96.847	0.796	2.563	0.931	89.475
ENSMUSG00000027220	Syt13	protein_coding	2.998	76.546	0.131	60.678	2.545	30.213	2.219	74.193
ENSMUSG00000027221	Chst1	protein_coding	0.752	1.698	0.889	86.728	1.493	5.864	0.953	90.611
ENSMUSG00000027222	Pex16	protein_coding	0.831	1.274	1.078	90.116	1.117	19.286	1.052	93.627
ENSMUSG00000027223	Mapk8ip1	protein_coding	0.567	0.987	1.356	83.104	1.305	6.202	0.929	92.561
ENSMUSG00000027224	Duoxa1	protein_coding	0.997	35.158	0.865	94.48	1.162	43.185	0.765	84.29
ENSMUSG00000027225	Duoxa2	protein_coding	1.244	80.815	1.067	97.288	0.755	82.903	0.899	93.964
ENSMUSG00000027227	Sord	protein_coding	1.114	10.687	0.98	91.361	0.917	21.431	0.97	95.74
ENSMUSG00000027229	4933406J08Rik	protein_coding	1	75.858	1	97.288	1	86.985	1	93.457
ENSMUSG00000027230	Creb3l1	protein_coding	1.163	29.057	0.973	97.155	0.886	35.815	1.102	59.464
ENSMUSG00000027236	Eif3j1	protein_coding	1.055	7.492	0.982	94.597	0.966	11.314	1.062	52.804
ENSMUSG00000027238	Frmd5	protein_coding	1.023	84.564	1.415	88.533	0.69	58.516	1.02	94.215
ENSMUSG00000027239	Mdk	protein_coding	0.679	7.58	1.083	85.756	1.364	40.955	1.119	92.018
ENSMUSG00000027242	Wdr76	protein_coding	1.102	17.582	0.902	73.766	1.003	86.985	1.138	72.992
ENSMUSG00000027243	Harbi1	protein_coding	0.967	86.918	0.982	78.51	1.054	40.875	0.915	88.505
ENSMUSG00000027244	Atg13	protein_coding	0.969	42.034	0.979	93.817	1.049	33.508	0.982	86.445
ENSMUSG00000027245	Hypk	protein_coding	0.89	40.422	0.966	96.138	1.16	50.091	1.06	94.752
ENSMUSG00000027247	Arhgap1	protein_coding	1.049	6.541	1.055	64.304	0.901	0.72	0.977	83.724
ENSMUSG00000027248	Pdia3	protein_coding	1.073	36.195	0.963	94.848	0.971	42.895	1.148	35.053
ENSMUSG00000027249	F2	protein_coding	1.02	75.115	0.736

82.848	1.335	54.163	1.114	87.447
ENSMUSG00000027253	Lrp4	protein_coding	1.081	43.654	1.04
96.268	0.889	49.967	0.857	87.182
ENSMUSG00000027254	Map1a	protein_coding	1.081	7.049	0.992	96.335
0.934	10.726	1.533	31.133
ENSMUSG00000027255	Arfgap2	protein_coding	0.986	75.567
0.992	90.378	1.018	53.134	1.031	92.52
ENSMUSG00000027257	Pacsin3	protein_coding	0.964	55.641
0.988	87.041	1.051	28.301	0.945	93.264
ENSMUSG00000027259	Adal	protein_coding	0.952	36.541	0.995
83.104	1.056	80.089	1.064	69.918
ENSMUSG00000027261	Hao1	protein_coding	1.11	78.909	1.021
95.301	0.883	84.724	1.38	87.447
ENSMUSG00000027263	Tubgcp4	protein_coding	0.973	42.922
0.995	96.984	1.03	56.125	1.03	82.091
ENSMUSG00000027270	Lamp5	protein_coding	0.315	84.977	0.552
90.046	5.763	62.648	5.565	94.904
ENSMUSG00000027272	Ubr1	protein_coding	1.003	76.135	0.988
90.256	1.011	64.624	0.982	85.493
ENSMUSG00000027274	Mkks	protein_coding	0.952	60.569	1.142
79.411	0.92	34.592	1.055	69.965
ENSMUSG00000027276	Jag1	protein_coding	1.077	8.685	1.043	89.014
0.89	3.943	0.861	83.724
ENSMUSG00000027281	Slx4ip	protein_coding	1.009	79.277
1.007	95.211	0.984	85.58	0.977	90.691
ENSMUSG00000027282	Mtch2	protein_coding	0.901	1.965	1.036	83.104
1.074	31.479	0.942	85.493
ENSMUSG00000027284	Cdan1	protein_coding	0.959	29.454	1.038
91.776	1.002	75.009	0.987	95.593
ENSMUSG00000027285	Haus2	protein_coding	0.967	83.823	1.023
96.001	1.011	86.258	1.049	95.273
ENSMUSG00000027286	Lrrc57	protein_coding	1.109	20.942	NA
NA	0.879	9.573	1.094	36.988
ENSMUSG00000027287	Snap23	protein_coding	1.046	38.682
0.941	88.691	1.017	87.786	1.025	79.268
ENSMUSG00000027288	Zfp106	protein_coding	1.034	32.238
1.005	94.597	0.961	36.499	1.024	83.067
ENSMUSG00000027291	Vps39	protein_coding	1.013	54.44	0.967	87.919
1.019	81.976	0.991	94.608
ENSMUSG00000027293	Ehd4	protein_coding	1.249	0	0.895	91.661
0.896	3.432	1.062	66.468
ENSMUSG00000027296	Itpka	protein_coding	1.169	7.174	1.001	96.013
0.854	15.169	0.949	85.493
ENSMUSG00000027297	Ltk	protein_coding	0.553	7.43	1.045	95.504
1.735	10.523	0.815	84.29
ENSMUSG00000027298	Tyro3	protein_coding	1.032	82.352	0.864
64.123	1.123	35.441	0.934	85.493
ENSMUSG00000027300	Ubox5	protein_coding	0.996	83.09	0.955	86.277
1.048	53.587	0.949	94.654
ENSMUSG00000027303	Ptpra	protein_coding	1.082	24.253	0.998
88.823	0.923	9.573	1.016	88.111
ENSMUSG00000027304	Rtf1	protein_coding	1.046	47.466	0.95
78.471	1.007	62.237	0.961	92.31
ENSMUSG00000027305	Ndufaf1	protein_coding	1.008	57.014

0.934 75.052 1.063 27.732 1.085 72.826
ENSMUSG00000027306 Nusap1 protein_coding 1.478 4.925 0.813
70.29 0.834 56.822 1.278 71.305
ENSMUSG00000027309 4930402H24Rik protein_coding 0.872 0.987
1.093 82.904 1.047 58.915 1.015 94.904
ENSMUSG00000027312 Atrn protein_coding 0.936 3.853 1.019 83.104
1.044 26.498 0.986 93.734
ENSMUSG00000027313 Chac1 protein_coding 1.108 84.324 0.82
79.475 1.102 51.851 1.522 44.882
ENSMUSG00000027314 Dll4 protein_coding 0.66 32.378 1.3
80.277 1.162 69.002 1.032 96.287
ENSMUSG00000027315 Spint1 protein_coding 1.046 29.596 0.98
75.345 0.974 83.811 0.898 88.505
ENSMUSG00000027316 Gfra4 protein_coding 0.982 76.686 1.099
84.832 0.929 60.96 0.976 90.157
ENSMUSG00000027317 Ppp1r14d protein_coding 0.796 30.035
0.978 96.268 1.279 40.052 0.856 86.991
ENSMUSG00000027318 Adam33 protein_coding 0.829 7.857 1.1
82.904 1.093 50.375 1.065 93.685
ENSMUSG00000027322 Siglec1 protein_coding 1.865 0 0.922
90.138 0.578 8.626 1.246 55.485
ENSMUSG00000027323 Rad51 protein_coding 0.966 84.534 NA NA
1.024 85.935 1.057 87.088
ENSMUSG00000027324 Rpusd2 protein_coding 1.136 25.207
0.901 90.046 0.976 78.713 0.912 84.29
ENSMUSG00000027326 Casc5 protein_coding 1.707 13.633 0.701
38.699 0.832 88.601 1.227 74.193
ENSMUSG00000027327 1700037H04Rik protein_coding 0.718 0.987 1.31
71.617 1.064 30.292 0.921 83.724
ENSMUSG00000027329 Spef1 protein_coding 0.719 0.987 1.073 94.767
1.3 5.864 1.078 44.882
ENSMUSG00000027330 Cdc25b protein_coding 1.123 29.536
1.124 71.98 0.795 2.986 0.983 94.215
ENSMUSG00000027331 Knstrn protein_coding 1.433 24.958
0.841 37.426 0.828 87.173 0.987 87.886
ENSMUSG00000027332 Ivd protein_coding 0.858 52.9 1.109 82.904
NA NA 0.969 86.991
ENSMUSG00000027333 Smox protein_coding 1.085 5.481 1.061 96.897
0.865 7.339 0.995 94.904
ENSMUSG00000027335 Adra1d protein_coding 0.879 52.258
1.121 84.768 1.012 84.961 0.898 90.289
ENSMUSG00000027339 Rassf2 protein_coding 1.253 1.493 0.875
53.434 0.908 33.402 1.045 94.608
ENSMUSG00000027340 Slc23a2 protein_coding 1.2 0.2 0.99
82.503 0.843 1.764 0.936 85.493
ENSMUSG00000027341 Tmem230 protein_coding 1.045 56.311 0.99
96.268 0.964 49.733 0.958 83.724
ENSMUSG00000027342 Pcna protein_coding 1.085 7.397 1.071 84.832
0.862 3.718 1.139 43.377
ENSMUSG00000027345 4921508D12Rik lincRNA 1 76.319 1
97.263 1 86.258 1 95.559
ENSMUSG00000027346 Gpcpd1 protein_coding 1.219 1.323 0.883
57.613 0.925 46.575 0.96 93.457
ENSMUSG00000027347 Rasgrp1 protein_coding 1.65 2.969 0.843

83.321 0.721 11.902 1.004 94.384
ENSMUSG00000027349 Fam98b protein_coding 1.05 40.065
0.991 79.807 0.956 73.874 1.109 31.133
ENSMUSG00000027350 Chgb protein_coding 1.197 86.172 0.847
90.256 0.987 76.754 0.952 96.139
ENSMUSG00000027351 Spred1 protein_coding 1.069 14.205
0.961 81.284 0.971 63.075 0.994 94.904
ENSMUSG00000027353 Mcm8 protein_coding 1.159 10.105 0.883
90.287 0.975 46.071 1.091 86.133
ENSMUSG00000027355 Tmco5 protein_coding 1 81.76 NA NA 1
74.535 1 96.287
ENSMUSG00000027356 Fermt1 protein_coding 0.866 67.867
0.989 96.984 1.172 64.305 1.094 84.31
ENSMUSG00000027357 Crls1 protein_coding 1.11 51.189 0.942
78.471 0.956 84.669 0.865 85.493
ENSMUSG00000027358 Bmp2 protein_coding 1.134 62.007 NA NA
0.979 63.504 0.964 95.273
ENSMUSG00000027359 Slc27a2 protein_coding 0.928 68.485
0.782 95.088 1.382 88.41 0.904 95.877
ENSMUSG00000027360 Hdc protein_coding 1.99 0.976 0.755 51.159
0.662 8.634 0.968 86.991
ENSMUSG00000027361 Gabpb1 protein_coding 1.029 72.372
0.921 92.137 1.057 84.83 1.02 91.928
ENSMUSG00000027363 Usp8 protein_coding 1.011 66.76 0.985 71.889
1.005 69.196 0.992 94.904
ENSMUSG00000027364 Usp50 protein_coding 1.347 52.134 0.593
56.781 1.249 50.851 0.842 85.493
ENSMUSG00000027365 Trpm7 protein_coding 0.986 84.168 1.003
85.793 1.012 65.8 0.939 85.493
ENSMUSG00000027366 Sppl2a protein_coding 1.011 53.228
0.944 53.434 1.044 27.89 1.007 93.156
ENSMUSG00000027367 Stard7 protein_coding 1.004 66.76 1.027
93.707 0.967 36.499 0.985 95.273
ENSMUSG00000027368 Dusp2 protein_coding 0.941 85.655 1.416
79.261 0.75 19.552 0.869 90.611
ENSMUSG00000027371 Fahd2a protein_coding 0.88 10.014
1.002 88.705 1.133 20.315 1.032 92.748
ENSMUSG00000027374 Mrps5 protein_coding 0.905 8.45 1.061 83.104
1.038 44.125 0.987 87.331
ENSMUSG00000027375 Mal protein_coding 1.038 63.804 1.026
97.263 0.941 59.726 1.084 88.797
ENSMUSG00000027376 Prom2 protein_coding 0.678 20.799 0.988
96.843 1.488 28.997 1.118 78.379
ENSMUSG00000027377 Mall protein_coding 0.746 13.74 1.103 83.104
1.218 27.431 1.01 94.904
ENSMUSG00000027378 Nphp1 protein_coding 0.998 56.085 1.062
81.127 0.941 6.524 1.03 96.192
ENSMUSG00000027379 Bub1 protein_coding 1.499 13.492 0.886
68.818 0.754 70.214 1.605 68.696
ENSMUSG00000027380 Acoxl protein_coding 0.724 55.641 1.175
92.931 1.172 62.307 0.939 89.587
ENSMUSG00000027381 Bcl2l11 protein_coding 0.859 21.025
1.159 96.407 1.006 48.935 0.803 84.29
ENSMUSG00000027384 Ndufaf5 protein_coding 0.9 17.705

1.088 83.104 1.023 74.325 1.075 71.305
ENSMUSG00000027386 Fbln7 protein_coding 1.097 86.199 NA NA
0.875 88.274 1.019 94.82
ENSMUSG00000027387 Zc3h8 protein_coding 1.056 68.485 1.06
96.407 0.895 35.559 0.979 95.832
ENSMUSG00000027394 Ttl protein_coding 1.057 18.556 1.116
82.904 0.849 2.453 1.024 93.964
ENSMUSG00000027395 Polr1b protein_coding 0.984 85.946
0.921 37.187 1.107 24.985 1.015 92.903
ENSMUSG00000027397 Slc20a1 protein_coding 1.074 86.464
1.027 79.367 0.908 37.906 0.881 94.174
ENSMUSG00000027398 Il1b protein_coding 0.959 84.324 1.881
85.756 0.553 41.662 0.656 84.19
ENSMUSG00000027399 Il1a protein_coding 0.921 84.564 NA NA
0.893 81.388 0.48 83.952
ENSMUSG00000027400 Pdyn protein_coding 1 74.8 1 95.504
1 66.269 1 94.567
ENSMUSG00000027401 Tgm3 protein_coding 0.851 83.333 1.082
96.407 1.088 88.657 1.473 95.273
ENSMUSG00000027403 Tgm6 protein_coding 1 52.799 1
95.708 1 57.091 1 86.991
ENSMUSG00000027404 Snrpb protein_coding 0.891 2.436 1.136 71.617
0.989 68.129 1.068 44.882
ENSMUSG00000027405 Nop56 protein_coding 1.006 83.658 1.01
96.216 NA NA 1.118 51.238
ENSMUSG00000027406 Idh3b protein_coding 0.97 40.217 0.979
95.708 1.054 45.449 1.021 94.904
ENSMUSG00000027408 Cpxm1 protein_coding 1.318 2.361 1.119 96.847
0.679 6.751 1.156 38.624
ENSMUSG00000027411 Vps16 protein_coding 0.998 61.043 0.957
75.618 1.047 29.302 1.044 63.018
ENSMUSG00000027412 Lpin3 protein_coding 0.93 51.056 0.865
31.138 1.246 7.635 1.017 90.157
ENSMUSG00000027416 Otor protein_coding 1 80.51 1 96.407
1 80.015 1 95.273
ENSMUSG00000027419 Pcsk2 protein_coding 1.454 62.354 0.714
90.053 0.965 86.258 1.446 69.404
ENSMUSG00000027420 Bfsp1 protein_coding 1.061 64.401 1.04
93.654 0.904 66.753 1.177 71.653
ENSMUSG00000027422 Rrbp1 protein_coding 0.973 33.599 0.975
96.001 1.049 70.9 1.096 44.213
ENSMUSG00000027423 Snx5 protein_coding 1.401 0 0.833 8.462
0.856 22.864 1.014 93.457
ENSMUSG00000027424 Mgme1 protein_coding 1.235 3.151 0.948 93.094
0.854 6.858 0.955 86.699
ENSMUSG00000027425 Csrp2bp protein_coding 1.116 2.784 1.01
84.999 0.889 2.189 1.043 83.067
ENSMUSG00000027427 NA NA NA NA 1.018 93.863 0.956
84.434 0.975 95.877
ENSMUSG00000027428 Rbbp9 protein_coding 1.042 39.075 1.002
96.216 0.96 46.32 0.993 95.877
ENSMUSG00000027429 Sec23b protein_coding 0.95 75.298
1.027 80.775 1.024 85.58 1.226 61.316
ENSMUSG00000027430 Dtd1 protein_coding 1.143 7.179 0.95 90.138

0.922 22.864 0.946 85.493
ENSMUSG00000027431 Scp2d1 protein_coding 1 86.658 NA
NA 1 78.369 1 94.752
ENSMUSG00000027433 Xrn2 protein_coding 0.964 84.738 0.939
68.115 1.106 33.802 1.113 31.133
ENSMUSG00000027434 Nkx2-2 protein_coding 1 79.165 1
97.097 1 88.461 1 93.734
ENSMUSG00000027435 Cd93 protein_coding 0.946 75.115 1.148
75.62 0.92 23.938 0.947 84.19
ENSMUSG00000027438 Napb protein_coding 1.165 28.098 0.967
95.708 0.889 51.055 0.964 88.755
ENSMUSG00000027439 Gzf1 protein_coding 0.988 81.657 0.955
55.71 1.054 52.8 1.046 94.19
ENSMUSG00000027442 Cst8 protein_coding 1 74.745 1
95.329 1 85.352 1 96.287
ENSMUSG00000027443 Cst12 protein_coding 1 82.479 1
96.847 1 87.431 1 95.273
ENSMUSG00000027444 8030411F24Rik protein_coding 1 67.163
NA NA 1 86.727 1 93.964
ENSMUSG00000027445 Cst9 protein_coding 1 85.728 1
92.545 1 84.961 1 94.752
ENSMUSG00000027446 9230104L09Rik protein_coding 0.911 71.674
1.955 66.695 0.563 9.347 0.986 92.748
ENSMUSG00000027447 Cst3 protein_coding 1.07 74.958 0.947
91.361 0.984 87.744 0.906 83.724
ENSMUSG00000027452 Acss1 protein_coding 1.128 17.761 0.983
84.594 0.904 47.12 0.942 84.29
ENSMUSG00000027454 Gins1 protein_coding 0.977 84.263 0.995
95.708 1.031 87.37 1.097 91.011
ENSMUSG00000027455 Nsfl1c protein_coding 0.932 5.544 0.984
87.147 1.094 7.635 1.044 74.047
ENSMUSG00000027456 Sdcbp2 protein_coding 0.931 66.897
0.872 63.912 1.235 25.564 1.006 94.19
ENSMUSG00000027457 Snph protein_coding 0.913 53.407 1.267
81.127 0.863 76.768 0.853 91.105
ENSMUSG00000027459 Fam110a protein_coding 1.019 85.655
1.136 79.261 0.86 37.617 1.054 83.067
ENSMUSG00000027460 Angpt4 protein_coding 0.606 38.555
1.429 79.367 NA NA 2.445 72.204
ENSMUSG00000027463 Slc52a3 protein_coding 0.851 71.915 NA
NA 1.153 63.665 1.092 83.067
ENSMUSG00000027465 Tbc1d20 protein_coding 1.106 6.026 0.955
95.504 0.944 13.643 0.984 86.991
ENSMUSG00000027466 Rbck1 protein_coding 0.975 53.679 1.012
88.923 1.012 80.015 0.979 92.31
ENSMUSG00000027468 Defb22 protein_coding 1 85.637 1
88.897 1 78.369 1 95.273
ENSMUSG00000027469 Tpx2 protein_coding 1.308 30.788 0.751
67.941 1.022 86.666 1.468 70.36
ENSMUSG00000027470 Mylk2 protein_coding 5.106 11.928 0.931
85.756 0.211 0.945 0.663 95.832
ENSMUSG00000027472 Pdrg1 protein_coding 0.978 77.407 0.994
95.504 1.03 75.155 1.033 87.447
ENSMUSG00000027474 Ccm2l protein_coding 0.889 33.415 1.123

91.776 0.997 68.643 0.949 83.724
ENSMUSG00000027475 Kif3b protein_coding 1.143 0.461 1.002 87.029
0.871 0.72 0.997 94.567
ENSMUSG00000027478 Dnmt3b protein_coding 1.047 85.946
1.003 90.712 0.952 82.436 0.942 86.699
ENSMUSG00000027479 Mapre1 protein_coding 1.041 24.481
0.993 94.89 0.967 18.336 1.037 50.452
ENSMUSG00000027480 Sun5 protein_coding 1 85.637 1
85.95 1 63.301 1 95.593
ENSMUSG00000027481 Bpifb2 protein_coding 1 81.465 1
88.345 1 78.155 1 93.964
ENSMUSG00000027482 Bpifa3 protein_coding 1 85.203 1
93.899 1 79.897 1 95.74
ENSMUSG00000027483 Bpifa1 protein_coding 1 84.819 NA
NA 1 86.258 1 95.559
ENSMUSG00000027484 Bpifa5 protein_coding 1 83.09 1
96.216 1 87.744 1 95.273
ENSMUSG00000027487 Cdk5rap1 protein_coding 0.988 82.231 1.02
91.065 0.993 59.125 1.064 92.762
ENSMUSG00000027488 Snta1 protein_coding 0.853 28.242 1.054
81.039 1.111 49.898 1.083 69.965
ENSMUSG00000027489 Necab3 protein_coding 0.847 27.836
1.369 82.904 0.861 87.686 0.986 94.19
ENSMUSG00000027490 E2f1 protein_coding 1.02 51.826 0.936
87.452 1.048 86.552 1.219 51.901
ENSMUSG00000027495 Fam210b protein_coding 0.985 71.674
0.885 35.072 1.15 10.76 1.004 90.854
ENSMUSG00000027496 Aurka protein_coding 1.042 61.725 0.875
71.889 1.092 77.888 1.362 83.067
ENSMUSG00000027498 Cstf1 protein_coding 1.005 81.125 1.06
95.196 0.94 55.214 0.958 83.724
ENSMUSG00000027499 Pkia protein_coding 1.496 0.767 1.191 63.504
0.562 0.72 1.067 90.177
ENSMUSG00000027500 Stmn2 protein_coding 1.237 28.098 0.809
65.165 0.997 75.155 1.003 86.209
ENSMUSG00000027502 Rtfdc1 protein_coding 0.927 9.081 1.006
94.848 1.074 25.455 1.006 85.786
ENSMUSG00000027505 Fam209 protein_coding 1.089 77.291
0.681 82.503 1.343 43.185 1.25 95.676
ENSMUSG00000027506 Tpd52 protein_coding 1.122 1.967 0.903 59.946
0.983 50.184 1.01 96.287
ENSMUSG00000027508 Pag1 protein_coding 1.082 61.725 1.032
83.104 0.896 11.902 0.911 83.724
ENSMUSG00000027509 Rae1 protein_coding 1.022 78.744 0.975
95.35 1.003 68.643 0.987 96.183
ENSMUSG00000027510 Rbm38 protein_coding 1.16 56.311 1.163
86.277 0.743 26.057 0.668 83.724
ENSMUSG00000027513 Pck1 protein_coding 0.729 33.415 0.85
67.165 1.609 9.176 1.229 74.047
ENSMUSG00000027514 Zbp1 protein_coding 1.507 5.559 1.228 96.407
0.537 15.024 1.727 38.624
ENSMUSG00000027518 1700021F07Rik protein_coding 1 82.543
1 95.708 1 88.161 1 88.512
ENSMUSG00000027519 Rab22a protein_coding 1.094 0.2 0.954

84.858 0.958 9.494 1.009 93.261
ENSMUSG00000027520 Zdbf2 protein_coding 0.859 74.021 0.631
52.266 1.845 22.444 1.031 95.72
ENSMUSG00000027522 Stx16 protein_coding 0.971 68.353 0.945
65.819 1.087 18.208 1.011 94.19
ENSMUSG00000027523 Gnas protein_coding 0.967 52.9 1.092 40.99
0.948 12.41 1.05 83.067
ENSMUSG00000027524 Edn3 protein_coding 1.19 69.408 0.836
93.574 1.002 46.32 0.895 96.358
ENSMUSG00000027525 Phactr3 protein_coding 1.775 16.249 0.44
73.461 1.282 79.524 1.362 95.92
ENSMUSG00000027528 Fabp9 protein_coding 0.235 17.839 2.271
92.793 1.87 47.31 1.86 88.657
ENSMUSG00000027530 Fabp12 protein_coding 0.854 73.585
1.414 91.456 0.827 78.155 1.022 96.042
ENSMUSG00000027531 Impa1 protein_coding 1.057 26.136 1.017
94.848 0.934 32.082 1.005 96.248
ENSMUSG00000027533 Fabp5 protein_coding 0.678 0.987 1.036 95.735
1.425 9.176 0.835 87.705
ENSMUSG00000027534 Snx16 protein_coding 1.122 2.651 1.069 85.756
0.836 1.764 1.024 96.183
ENSMUSG00000027536 Chmp4c protein_coding 0.883 30.035
1.101 83.104 1.03 62.237 0.869 83.724
ENSMUSG00000027540 Ptpn1 protein_coding 0.981 64.014 0.912
33.183 1.117 10.523 1.077 73.597
ENSMUSG00000027544 Nfatc2 protein_coding 1.593 0 0.823
90.378 0.758 1.408 1.181 63.708
ENSMUSG00000027546 Atp9a protein_coding 0.92 41.759 1.002
94.367 1.085 60.5 1.122 83.067
ENSMUSG00000027550 Lrrcc1 protein_coding 1.023 38.386
0.914 45.262 1.072 58.616 1.02 96.086
ENSMUSG00000027551 Zfp64 protein_coding 0.967 45.229 0.983
90.053 1.051 34.807 0.926 85.493
ENSMUSG00000027552 E2f5 protein_coding 1.047 18.388 0.964
85.075 0.987 62.058 0.958 84.29
ENSMUSG00000027555 Car13 protein_coding 1.094 19.132 1.043
90.092 0.875 6.935 1.077 93.964
ENSMUSG00000027556 Car1 protein_coding 1.004 86.333 0.873
85.671 1.141 55.109 0.907 85.493
ENSMUSG00000027559 Car3 protein_coding 0.962 83.263 NA NA
1.072 85.58 0.979 86.699
ENSMUSG00000027560 Dok5 protein_coding 1.328 63.385 1.054
96.843 0.712 54.964 1.336 85.786
ENSMUSG00000027562 Car2 protein_coding 0.943 75.115 1.114
95.431 NA NA 0.949 95.92
ENSMUSG00000027564 NA NA NA NA 1 96.763 1
87.173 1 93.964
ENSMUSG00000027566 Psma7 protein_coding 0.965 39.712 1.008
97.263 1.029 44.361 1.039 54.265
ENSMUSG00000027568 Ntsr1 protein_coding 1 78.224 1
96.216 1 88.601 1 96.086
ENSMUSG00000027569 Mrgbp protein_coding 1.015 86.112 0.996
93.899 0.991 75.353 0.967 88.053
ENSMUSG00000027570 Col9a3 protein_coding 0.782 24.577

1.162 87.029 1.097 50.636 1.011 95.777
ENSMUSG00000027573 Gid8 protein_coding 1.03 46.097 1.004
96.847 0.968 38.534 1.011 87.7
ENSMUSG00000027574 Nkain4 protein_coding 1.096 78.428
1.174 92.062 0.778 52.658 0.893 83.067
ENSMUSG00000027575 Arfgap1 protein_coding 0.927 11.384 NA
NA 1.069 17.662 1.048 43.377
ENSMUSG00000027577 Chrna4 protein_coding 1.279 72 0.75
88.897 1.042 86.666 0.883 96.183
ENSMUSG00000027579 Srms protein_coding 0.787 27.202 1.158
95.211 1.096 49.384 1.075 87.134
ENSMUSG00000027580 Helz2 protein_coding 0.966 57.014 1.038
85.756 0.999 76.768 1.129 59.464
ENSMUSG00000027581 Stmn3 protein_coding 0.853 75.671 1.232
92.561 0.954 50.523 1.227 92.52
ENSMUSG00000027582 Zgpat protein_coding 0.896 1.343 1.018 88.91
1.093 28.74 0.976 86.991
ENSMUSG00000027583 Zbtb46 protein_coding 0.85 10.903
1.069 83.104 1.101 31.975 0.984 95.273
ENSMUSG00000027584 Oprl1 protein_coding 1.158 74.516 0.511
85.793 1.684 76.225 0.583 83.724
ENSMUSG00000027589 Pcmtd2 protein_coding 1.082 19.477
0.923 12.804 0.999 52.574 0.878 83.724
ENSMUSG00000027593 Raly protein_coding 0.957 41.759 1.024
86.277 1.016 79.426 1.128 78.948
ENSMUSG00000027596 a protein_coding 0.741 84.661 NA NA
0.936 88.41 1.57 84.246
ENSMUSG00000027597 Ahcy protein_coding 0.955 32.378 1.007
90.287 1.044 34.07 1.1 40.894
ENSMUSG00000027598 Itch protein_coding 1.08 2.567 0.978 72.576
0.948 39.755 0.986 86.991
ENSMUSG00000027599 Armc1 protein_coding 1.049 22.633 1.019
95.735 0.935 2.687 1.031 92.564
ENSMUSG00000027601 Mtfr1 protein_coding 0.977 86.333 1.054
83.104 0.968 38.799 1.025 96.248
ENSMUSG00000027602 Map1lc3a protein_coding 0.851 7.887 1.104
82.904 1.067 38.099 0.971 95.832
ENSMUSG00000027603 Ggt7 protein_coding 0.374 0.987 1.492 83.104
1.785 6.506 0.911 93.045
ENSMUSG00000027605 Acss2 protein_coding 0.281 0.987 1.434 83.104
2.478 6.916 0.911 94.904
ENSMUSG00000027606 Dnajc5b protein_coding 1 76.686 1
97.288 1 87.744 1 94.904
ENSMUSG00000027610 Gss protein_coding 0.984 85.77 0.954 88.691
1.064 58.414 0.951 87.331
ENSMUSG00000027611 Procr protein_coding 1.057 36.124 0.806
54.427 1.168 37.259 1.096 83.067
ENSMUSG00000027612 Mmp24 protein_coding 0.738 14.645 1.164
93.707 1.167 30.292 0.796 83.724
ENSMUSG00000027613 Eif6 protein_coding 0.951 55.939 0.987
96.216 1.062 42.199 1.015 93.016
ENSMUSG00000027615 Hps3 protein_coding 0.954 83.523 1.086
97.155 0.961 83.811 0.992 94.904
ENSMUSG00000027618 Nfs1 protein_coding 0.919 10.143 1.024

90.116 1.062 28.979 1.026 83.067
ENSMUSG00000027620 Rbm39 protein_coding 1.004 76.319 0.959 41.951 1.039 25.455 1.023 94.215
ENSMUSG00000027624 Epb4.1l1 protein_coding 0.907 25.3 1.107 72.642 0.992 71.734 1.038 96.183
ENSMUSG00000027628 Aar2 protein_coding 1.014 84.738 1.035 83.104 0.949 39.528 0.974 86.445
ENSMUSG00000027630 Tbl1xr1 protein_coding 1.19 1.323 0.947 95.708 0.884 0.72 0.98 93.612
ENSMUSG00000027634 Ndrg3 protein_coding 1.014 83.263 0.999 93.574 0.984 83.607 0.985 92.018
ENSMUSG00000027635 Dsn1 protein_coding 1.053 71.896 0.942 85.671 1.005 82.74 1.264 46.412
ENSMUSG00000027636 Sla2 protein_coding 0.766 3.895 1.077 94.367 1.21 16.205 0.904 83.724
ENSMUSG00000027637 1110008F13Rik protein_coding 0.949 40.217 1.053 90.952 0.999 71.232 0.959 86.991
ENSMUSG00000027639 Samhd1 protein_coding 1.135 0.664 0.933 41.951 0.94 83.811 1.098 52.366
ENSMUSG00000027641 Rbl1 protein_coding 1.061 24.958 NA NA 0.937 42.997 1.115 73.368
ENSMUSG00000027642 Rpn2 protein_coding 1.065 13.8 1.047 89.996 0.897 20.009 1.074 50.452
ENSMUSG00000027643 Ghrh protein_coding 1 85.655 NA NA 1 75.155 1 86.991
ENSMUSG00000027646 Src protein_coding 1.015 81.76 1.049 90.389 0.94 48.387 0.973 84.29
ENSMUSG00000027649 Ctnnbl1 protein_coding 0.824 0.987 0.903 88.704 1.342 6.202 0.952 91.625
ENSMUSG00000027650 Tti1 protein_coding 1.021 58.896 1.047 87.185 0.933 36.248 1.003 90.125
ENSMUSG00000027651 Rprd1b protein_coding 0.96 54.87 1.056 79.367 0.988 87.744 1.177 60.968
ENSMUSG00000027652 Ralgapb protein_coding 0.999 64.714 1.025 97.155 0.975 85.58 0.998 96.086
ENSMUSG00000027654 Fam83d protein_coding 0.923 83.09 0.966 90.256 1.123 70.328 1.327 86.118
ENSMUSG00000027655 Dhx35 protein_coding 0.914 27 0.969 87.998 1.123 18.208 0.953 91.269
ENSMUSG00000027656 Wisp2 protein_coding 0.935 84.661 1.13 78.705 0.949 49.733 1.508 78.379
ENSMUSG00000027660 Skil protein_coding 1 71.36 0.944 85.418 1.057 54.809 1.012 91.985
ENSMUSG00000027661 Slc2a10 protein_coding 1.177 37.941 1.005 95.504 0.844 43.426 1.059 56.079
ENSMUSG00000027663 Zmat3 protein_coding 0.978 81.465 1.136 60.309 0.9 6.858 1.038 89.498
ENSMUSG00000027665 Pik3ca protein_coding 1.071 2.651 0.997 97.288 0.939 2.333 0.994 95.877
ENSMUSG00000027667 Zfp639 protein_coding 1.111 4.628 0.965 88.881 0.932 39.755 1.006 95.559
ENSMUSG00000027668 Mfn1 protein_coding 1.04 9.206 0.949 37.376 1.014 84.504 1.017 93.261
ENSMUSG00000027669 Gnb4 protein_coding 0.884 21.176 1.048

87.037 1.077 65.8 1.014 94.19
ENSMUSG00000027670 Ocstamp protein_coding 1.091 81.196 1.55
82.904 0.59 19.236 0.923 96.248
ENSMUSG00000027671 Actl6a protein_coding 1.005 68.353
1.066 83.464 0.935 39.245 1.016 83.067
ENSMUSG00000027673 Ndufb5 protein_coding 0.941 38.195
1.035 83.104 1.023 74.082 1.013 95.273
ENSMUSG00000027674 Pex5l protein_coding 0.945 56.768 1.042
96.138 1.013 51.851 0.958 94.654
ENSMUSG00000027676 Ccdc39 protein_coding 0.927 25.3 0.961
96.68 1.12 49.058 0.885 87.735
ENSMUSG00000027677 Ttc14 protein_coding 0.885 6.34 1.012 91.532
1.118 10.76 0.996 92.561
ENSMUSG00000027678 Ncoa3 protein_coding 1.17 0 0.983 96.847
0.866 0.72 0.995 89.892
ENSMUSG00000027679 Dnajc19 protein_coding 1.045 38.386
1.038 77.598 0.922 5.884 0.977 95.273
ENSMUSG00000027680 Fxr1 protein_coding 1.131 17.582 0.91
55.265 0.975 73.096 1.002 72.369
ENSMUSG00000027684 Mecom protein_coding 1.165 38.195 1.009
96.407 0.846 49.733 0.898 85.493
ENSMUSG00000027692 Tnik protein_coding 0.981 72 1.131 90.116
0.899 15.465 1.002 90.289
ENSMUSG00000027694 Gm8325 protein_coding 1.231 41.454
0.824 89.996 0.982 70.44 0.92 86.699
ENSMUSG00000027695 Pld1 protein_coding 1.106 17.336 0.936
74.483 0.966 69.974 1.032 91.928
ENSMUSG00000027698 Nceh1 protein_coding 1.311 0.767 0.836 53.434
0.911 18.122 0.872 83.724
ENSMUSG00000027699 Ect2 protein_coding 1.092 62.354 0.852
79.261 1.076 88.693 1.604 66.468
ENSMUSG00000027703 Lrriq4 protein_coding 1 84.865 1
94.102 1 81.857 1 96.086
ENSMUSG00000027706 Sec62 protein_coding 1.061 33.555 0.958
75.618 0.979 87.898 1.038 59.464
ENSMUSG00000027708 Dcun1d1 protein_coding 1.088 1.626 NA NA
0.925 6.858 0.985 86.699
ENSMUSG00000027709 Mccc1 protein_coding 0.863 11.384 1.043
83.104 1.107 50.636 0.997 96.183
ENSMUSG00000027710 Acad9 protein_coding 0.917 3.853 1.092 82.809
0.994 72.378 1.005 96.358
ENSMUSG00000027712 Anxa5 protein_coding 1.089 34.696 0.955
84.858 0.962 63.075 1.032 87.447
ENSMUSG00000027713 1810062G17Rik protein_coding 1.037 84.397
0.759 81.134 1.266 46.956 1.003 92.748
ENSMUSG00000027714 Exosc9 protein_coding 1.012 58.709
0.913 38.699 1.084 33.802 1.059 48.119
ENSMUSG00000027715 Ccna2 protein_coding 1.023 71.06 0.958 90.053
1.025 88.657 1.447 63.114
ENSMUSG00000027716 Trpc3 protein_coding 1.371 17.582 0.903
96.216 0.808 1.48 1.209 91.335
ENSMUSG00000027718 Il21 protein_coding 1 86.333 1
97.263 1 84.308 1 93.734
ENSMUSG00000027719 Adad1 protein_coding 1 72.546 1

92.931	1	82.31	1	90.674
ENSMUSG00000027722	Spata5	protein_coding	1.18	38.195	0.77
8.462	1.098	43.185	1.08	67.691
ENSMUSG00000027737	Slc7a11	protein_coding	0.967	82.732	NA
NA	0.975	88.203	0.86	88.552
ENSMUSG00000027739	Rab33b	protein_coding	1.048	23.019
0.973	84.017	0.982	85.675	0.955	86.991
ENSMUSG00000027742	Cog6	protein_coding	1.044	14.205	1.035
97.155	0.925	22.864	1.017	79.268
ENSMUSG00000027746	Ufm1	protein_coding	0.968	66.129	0.998
95.708	1.035	70.623	1.136	50.452
ENSMUSG00000027748	Trpc4	protein_coding	1.697	85.728	0.249
88.897	2.361	56.125	2.341	73.597
ENSMUSG00000027750	Postn	protein_coding	1.245	38.682	0.815
66.09	0.987	88.076	1.135	87.7
ENSMUSG00000027751	Supt20	protein_coding	0.976	84.049
1.021	92.561	1.003	87.686	1.035	80.324
ENSMUSG00000027752	Exosc8	protein_coding	1.086	16.124
0.939	91.897	0.984	33.137	1.088	72.369
ENSMUSG00000027761	Aadac	protein_coding	1	66.58	1	93.654
1	84.896	1	94.174
ENSMUSG00000027762	Sucnr1	protein_coding	1	82.732
1.008	96.68	0.99	74.179	0.964	83.724
ENSMUSG00000027763	Mbnl1	protein_coding	1.161	0	1.068	83.104
0.808	0.72	1.009	92.675
ENSMUSG00000027765	P2ry1	protein_coding	0.931	75.298	1.017
96.001	1.058	77.17	1.194	82.328
ENSMUSG00000027770	Dhx36	protein_coding	1.022	58.709	0.989
88.705	0.989	87.336	1.02	96.358
ENSMUSG00000027774	Gfm1	protein_coding	0.959	30.637	1.002
97.129	1.037	44.125	1.098	82.328
ENSMUSG00000027775	Mfsd1	protein_coding	1.251	0	0.935	74.483
0.855	0.72	0.947	84.29
ENSMUSG00000027777	Schip1	protein_coding	0.988	75.298
1.134	60.309	0.894	9.494	1.055	86.133
ENSMUSG00000027778	Ift80	protein_coding	1.025	56.311	1.066
95.504	0.917	80.015	0.915	90.674
ENSMUSG00000027782	Kpna4	protein_coding	1.037	82.543	1.036
85.756	0.928	47.641	1.04	54.265
ENSMUSG00000027783	Arl14	pseudogene	1	72	1	94.48	1
87.744	1	96.183
ENSMUSG00000027784	Ppm1l	protein_coding	0.954	14.849	NA	NA
1.09	40.052	0.903	85.493
ENSMUSG00000027787	Nmd3	protein_coding	1.081	4.675	0.952	70.29
0.969	39.079	1.058	91.335
ENSMUSG00000027790	Sis	protein_coding	1	81.01	1	97.263
1	81.388	1	92.748
ENSMUSG00000027792	Bche	protein_coding	0.805	4.268	1.046	83.104
1.189	21.873	1.098	81.901
ENSMUSG00000027794	Sohlh2	protein_coding	1	82.231	1
93.654	1	64.305	1	95.273
ENSMUSG00000027796	Smad9	protein_coding	0.931	48.146	1.118
83.104	0.957	80.163	0.882	83.724
ENSMUSG00000027797	Dclk1	protein_coding	1.64	1.967	0.896	95.708

0.678 0.72 1.181 43.377
ENSMUSG00000027799 Nbea protein_coding 1.082 41.454 0.963
95.088 0.96 55.214 0.897 85.493
ENSMUSG00000027800 Tm4sf1 protein_coding 1.088 24.481
1.022 87.029 0.898 10.23 0.982 90.628
ENSMUSG00000027801 Tm4sf4 protein_coding 1 84.977 1
92.931 1 82.575 1 95.92
ENSMUSG00000027803 Wwtr1 protein_coding 1.078 48.288 0.932
95.211 0.996 65.902 0.895 83.724
ENSMUSG00000027804 Ppid protein_coding 1.074 72 0.893 75.345
1.042 57.227 0.998 75.81
ENSMUSG00000027805 Pfn2 protein_coding 1.205 4.628 1.006 93.529
0.822 0.72 0.976 94.215
ENSMUSG00000027806 Tsc22d2 protein_coding 0.991 81.647
1.072 72.546 0.942 11.504 0.977 86.699
ENSMUSG00000027808 Serp1 protein_coding 1.052 54.44 1.027 95.809
0.926 27.86 1.12 60.31
ENSMUSG00000027809 Etfdh protein_coding 0.968 76.319 NA NA
NA NA 1.055 94.752
ENSMUSG00000027810 Eif2a protein_coding 1.122 3.536 0.892 51.322
0.995 85.675 1.045 79.268
ENSMUSG00000027811 4930579G24Rik protein_coding 1.078 26.863
1.201 86.387 0.774 6.935 1.079 95.74
ENSMUSG00000027820 Mme protein_coding 0.952 34.954 1.074
72.546 0.98 46.071 0.962 88.505
ENSMUSG00000027822 Slc33a1 protein_coding 1.019 81.24 NA NA
1.003 67.962 1.032 93.156
ENSMUSG00000027823 Gmps protein_coding 1.113 15.456 0.957
87.553 0.939 39.079 0.976 94.654
ENSMUSG00000027824 Vmn2r1 protein_coding 0.871 85.355
0.939 92.137 1.218 78.369 0.934 95.72
ENSMUSG00000027827 Kcnab1 protein_coding 1.423 1.545 0.922
79.475 0.76 3.943 0.893 86.991
ENSMUSG00000027828 Ssr3 protein_coding 1.16 0 0.97 93.654
0.891 3.654 1.08 50.74
ENSMUSG00000027829 Ccnl1 protein_coding 1.021 74.482 0.989
81.731 0.989 77.888 0.958 83.724
ENSMUSG00000027831 Veph1 protein_coding 1.373 67.991 0.49
70.29 1.482 58.414 1.299 78.948
ENSMUSG00000027832 Ptx3 protein_coding 1.1 69.777 0.887
78.471 1.024 76.754 0.963 93.132
ENSMUSG00000027833 Shox2 protein_coding 0.868 24.817 1.013
95.809 1.138 29.777 1.044 91.335
ENSMUSG00000027834 Serpini1 protein_coding 1.49 21.599 NA
NA 0.742 25.052 0.982 86.133
ENSMUSG00000027835 Pdcd10 protein_coding 1.029 35.748 NA
NA 0.961 54.037 1.022 69.918
ENSMUSG00000027840 Wnt2b protein_coding 0.914 75.858 0.698
35.072 1.563 11.782 1.077 94.904
ENSMUSG00000027843 Ptpn22 protein_coding 1.568 0.2 0.842
69.175 0.756 9.456 1.079 96.183
ENSMUSG00000027845 Dclre1b protein_coding 1.078 64.956
0.967 87.553 0.96 88.601 1 87.7
ENSMUSG00000027848 Olfml3 protein_coding 1.447 0 1.107

82.904 0.623 0.72 1.188 57.839
ENSMUSG00000027849 Syt6 protein_coding 1.064 74.745 1.031
95.708 0.912 85.464 0.609 83.724
ENSMUSG00000027852 Nras protein_coding 1.073 1.99 0.995 93.707
0.933 0.72 1.094 73.401
ENSMUSG00000027854 Sike1 protein_coding 1.012 52.429 1.076
82.809 0.918 7.742 0.995 90.691
ENSMUSG00000027855 Sycp1 protein_coding 1 84.294 NA NA
1 86.666 1 83.276
ENSMUSG00000027858 Tspan2 protein_coding 1.152 0.767 0.967
85.116 0.897 12.948 0.949 83.724
ENSMUSG00000027859 Ngf protein_coding 0.898 83.09 1.012 96.843
1.1 86.144 1.105 92.851
ENSMUSG00000027860 Vangl1 protein_coding 1.092 8.102 1.015
95.301 0.902 19.236 0.986 96.358
ENSMUSG00000027861 Casq2 protein_coding 1.051 56.66 1.082 90.116
0.881 13.643 1.066 73.597
ENSMUSG00000027863 Cd2 protein_coding 1.159 44.334 0.938
91.661 0.92 72.464 0.836 83.724
ENSMUSG00000027864 Ptgfrn protein_coding 1.115 24.253 0.96
85.116 0.936 49.733 1.053 74.607
ENSMUSG00000027865 Gdap2 protein_coding 1.092 10.595 0.969
86.538 0.946 33.971 1.045 79.442
ENSMUSG00000027867 Spag17 protein_coding 0.904 22.237
1.178 81.127 0.935 74.686 0.871 96.358
ENSMUSG00000027868 Tbx15 protein_coding 1.017 73.124 1.118
79.411 0.881 19.765 1.214 31.133
ENSMUSG00000027869 NA NA NA NA 1 93.469 1
85.935 1 93.156
ENSMUSG00000027870 Hao2 protein_coding 1 65.869 1
97.155 1 85.921 1 95.676
ENSMUSG00000027871 Hsd3b1 protein_coding 1 85.655 1
95.196 1 86.258 1 95.273
ENSMUSG00000027875 Hmgcs2 protein_coding 1.556 4.675 0.488 0
1.312 67.562 1.171 93.612
ENSMUSG00000027876 Reg4 protein_coding 1 67.867 1
96.68 1 71.615 1 95.273
ENSMUSG00000027878 Notch2 protein_coding 1.141 3.152 0.999
89.996 0.875 17.258 1.009 96.192
ENSMUSG00000027879 Sec22b protein_coding 1.024 84.324
1.067 71.617 0.913 17.381 1.066 57.952
ENSMUSG00000027880 NA NA NA NA 1 96.843 NA NA
1 92.748
ENSMUSG00000027881 Prpf38b protein_coding 0.916 1.703 0.99
92.631 1.104 6.449 0.988 84.29
ENSMUSG00000027882 Stxbp3a protein_coding 1.079 4.675 1.039
95.088 0.89 16.129 0.977 91.372
ENSMUSG00000027883 Gpsm2 protein_coding 1.239 5.313 1.013 96.843
0.799 8.907 1.076 61.984
ENSMUSG00000027884 Clcc1 protein_coding 1.081 30.984 0.954
79.807 NA NA 1.056 72.992
ENSMUSG00000027886 1700013F07Rik protein_coding 2.848 29.536
0.209 61.883 1.676 70.328 0.673 87.735
ENSMUSG00000027887 Sypl2 protein_coding 5.293 7.65 1.037 82.904

0.182 0.72 1.1 93.627
ENSMUSG00000027889 Ampd2 protein_coding 1.039 86.474 1.036
85.756 0.932 21.431 1.036 72.992
ENSMUSG00000027890 Gstm4 protein_coding 0.939 35.449 1.007
92.062 1.054 60.5 1 85.493
ENSMUSG00000027893 Ahcyl1 protein_coding 0.961 72.139
0.944 82.848 1.102 31.4 1.001 91.011
ENSMUSG00000027894 Slc6a17 protein_coding 1.356 10.595
0.924 85.921 0.797 32.082 0.882 91.625
ENSMUSG00000027895 Kcnc4 protein_coding 4.541 7.255 0.691 90.576
0.317 9.494 0.725 93.264
ENSMUSG00000027896 Slc16a4 protein_coding 1.012 77.407
0.948 86.071 1.041 70.1 0.941 94.608
ENSMUSG00000027900 Dram2 protein_coding 0.954 29.454 0.98
95.211 1.062 25.966 0.941 85.493
ENSMUSG00000027901 Dennd2d protein_coding 0.862 3.325 0.991
94.597 1.171 10.615 0.934 90.611
ENSMUSG00000027902 BC051070 protein_coding 0.743 85.857
1.143 93.908 1.181 67.823 0.826 92.181
ENSMUSG00000027905 Ddx20 protein_coding 1.107 2.655 0.981 96.843
0.919 5.453 0.975 92.181
ENSMUSG00000027907 S100a11 protein_coding 0.892 7.887 1.055
90.389 1.066 48.451 1.055 75.198
ENSMUSG00000027908 Tchhl1 protein_coding 1 85.857 1
96.013 1 85.21 1 95.877
ENSMUSG00000027912 Lce1m protein_coding 1 85.499 1
95.504 1 82.436 1 93.964
ENSMUSG00000027913 Crct1 protein_coding 1 86.006 1
90.053 1 62.307 1 91.525
ENSMUSG00000027919 Lce1g protein_coding 1 83.263 1
97.155 NA NA 1 91.269
ENSMUSG00000027923 Lce1b protein_coding 1 61.171 1
95.211 1 78.954 1 95.273
ENSMUSG00000027925 Sprr2j-ps protein_coding 1 81.657 1
96.897 NA NA 1 94.016
ENSMUSG00000027927 Lelp1 protein_coding 1 74.482 1
95.735 1 81.976 1 93.316
ENSMUSG00000027931 Npr1 protein_coding 1.048 25.944 0.949
79.807 1.007 86.144 1.007 94.904
ENSMUSG00000027932 Slc27a3 protein_coding 0.998 76.135
1.019 95.504 0.98 75.527 0.979 94.016
ENSMUSG00000027933 Ints3 protein_coding 0.971 36.945 1.078
87.029 0.954 83.607 1.059 40.894
ENSMUSG00000027935 Rab13 protein_coding 1.013 73.124 0.968
66.09 1.02 70.9 1.07 92.691
ENSMUSG00000027936 Crtc2 protein_coding 0.925 6.952 1.082 72.546
0.999 80.381 0.962 84.29
ENSMUSG00000027937 Jtb protein_coding 1.054 59.806 0.954
94.848 0.997 70.791 1.007 95.273
ENSMUSG00000027938 Creb3l4 protein_coding 0.811 47.266
0.829 41.268 1.489 10.76 1.033 89.892
ENSMUSG00000027939 Nup210l protein_coding 0.826 36.259
1.057 90.68 1.148 69.315 1.061 93.156
ENSMUSG00000027940 Tpm3 protein_coding 1.049 13.8 0.984 87.322

0.969 60.614 1.114 31.133
ENSMUSG00000027942 4933434E20Rik protein_coding 1.133 0.2 NA NA 0.861 3.234 0.998 95.593
ENSMUSG00000027944 Hax1 protein_coding 0.881 8.751 1.043 90.68 1.086 26.498 1.027 83.276
ENSMUSG00000027947 Il6ra protein_coding 1.128 55.789 0.841 44.268 1.056 54.673 1.112 95.72
ENSMUSG00000027950 Chrnb2 protein_coding 0.924 35.449 1.035 86.793 1.045 62.307 0.798 83.724
ENSMUSG00000027951 Adar protein_coding 1.094 20.296 1.008 92.545 0.907 52.087 1.126 55.841
ENSMUSG00000027952 Pmvk protein_coding 0.678 0.987 0.969 96.407 1.524 6.202 1.009 94.016
ENSMUSG00000027953 Slc50a1 protein_coding 1.081 14.966 0.985 91.661 0.935 45.573 0.991 96.071
ENSMUSG00000027954 Efna1 protein_coding 0.836 2.559 0.981 88.705 1.216 7.635 0.945 92.181
ENSMUSG00000027955 Fam198b protein_coding 1.227 0 0.997 94.89 0.816 1.58 0.93 86.991
ENSMUSG00000027956 Tmem144 protein_coding 1.171 54.344 0.706 55.71 1.211 54.363 1.074 91.335
ENSMUSG00000027957 Slc35a3 protein_coding 1.02 70.121 0.967 90.378 1.016 85.921 0.976 88.387
ENSMUSG00000027959 Sass6 protein_coding 1.266 13.557 0.914 73.094 0.862 49.733 1.077 57.075
ENSMUSG00000027961 Lrrc39 protein_coding 0.935 42.361 1.421 71.617 0.751 42.997 1.163 82.091
ENSMUSG00000027962 Vcam1 protein_coding 1.321 41.177 0.908 79.475 0.832 69.134 0.923 95.92
ENSMUSG00000027963 Extl2 protein_coding 1.106 14.008 1.02 97.263 0.883 25.052 1.049 73.401
ENSMUSG00000027965 Olfm3 protein_coding 1 81.657 1 95.431 1 81.976 1 95.273
ENSMUSG00000027966 Col11a1 protein_coding 0.885 12.309 0.869 90.046 1.303 8.93 1.023 83.865
ENSMUSG00000027967 Neurog2 protein_coding 1 82.479 NA NA 1 87.173 1 96.183
ENSMUSG00000027968 Larp7 protein_coding 1.102 73.734 0.908 88.691 1.001 87.431 1.068 50.74
ENSMUSG00000027971 Ndst4 protein_coding 0.915 74.8 0.371 68.115 2.944 22.517 0.782 93.734
ENSMUSG00000027973 1700006A11Rik protein_coding 1 64.893 1 97.155 1 83.275 1 95.593
ENSMUSG00000027977 Ndst3 protein_coding 1.647 1.242 1.097 87.147 0.551 5.701 1.241 82.85
ENSMUSG00000027978 Prss12 protein_coding 1.243 7.332 0.982 96.138 0.816 14.583 1.071 95.92
ENSMUSG00000027981 Rnpc3 protein_coding 1.092 45.307 0.93 82.848 NA NA 0.955 91.625
ENSMUSG00000027983 Cyp2u1 protein_coding 0.934 80.815 1.211 82.904 0.884 27.321 0.967 95.92
ENSMUSG00000027984 Hadh protein_coding 0.896 2.822 1.039 83.464 1.073 37.764 0.988 88.245
ENSMUSG00000027985 Lef1 protein_coding 1.112 79.277 1.24

96.68 0.724 59.726 0.791 85.493
ENSMUSG00000027993 Trim2 protein_coding 0.923 34.162 NA NA
1.045 49.601 0.992 94.608
ENSMUSG00000027994 Ccdc109b protein_coding 1.588 0 0.852
61.883 0.739 14.262 1.204 61.984
ENSMUSG00000027995 Tlr2 protein_coding 1.084 24.147 1.038
92.931 0.884 15.465 0.968 86.991
ENSMUSG00000027996 Sfrp2 protein_coding 1.441 23.82 1.045 87.029
0.661 13.848 0.728 83.724
ENSMUSG00000027997 Casp6 protein_coding 0.93 42.722 1.013
96.847 1.058 39.471 0.949 85.493
ENSMUSG00000027998 Plrg1 protein_coding 0.964 57.227 0.955
59.946 1.089 23.334 1.043 93.156
ENSMUSG00000027999 Pla2g12a protein_coding 0.911 14.086
0.945 95.504 1.164 37.741 0.999 96.358
ENSMUSG00000028001 Fga protein_coding 1 85.203 1
95.708 1 86.144 1 95.676
ENSMUSG00000028003 Lrat protein_coding 1.192 22.871 1.072
90.053 0.781 57.091 0.972 92.181
ENSMUSG00000028004 Npy2r protein_coding 1.974 43.939 0.791
95.211 0.642 42.621 1.489 52.982
ENSMUSG00000028005 Gucy1b3 protein_coding 1.162 24.481 1.1
94.367 0.779 20.258 0.938 93.964
ENSMUSG00000028007 Snx7 protein_coding 0.988 72.139 0.972
90.116 1.042 78.954 1.136 64.905
ENSMUSG00000028008 Asic5 protein_coding 1 74.958 1
95.708 1 87.431 1 96.183
ENSMUSG00000028009 1700061I17Rik protein_coding 1 81.125
1 95.504 1 81.857 1 95.92
ENSMUSG00000028010 Gar1 protein_coding 1.016 70.36 1.007 95.708
0.979 86.346 0.947 94.016
ENSMUSG00000028011 Tdo2 protein_coding 0.968 81.925 1.009
94.597 1.019 84.83 1.04 91.525
ENSMUSG00000028013 Ppa2 protein_coding 0.977 76.546 0.985
90.378 NA NA 1.063 86.301
ENSMUSG00000028015 Ctso protein_coding 1.134 9.983 0.916 93.654
0.962 23.48 0.985 90.805
ENSMUSG00000028016 Ints12 protein_coding 1.119 34.861
0.868 68.818 1.029 71.232 1.029 83.067
ENSMUSG00000028017 Egf protein_coding 1.368 17.761 0.909
84.594 0.804 39.528 1.044 90.177
ENSMUSG00000028018 Gstcd protein_coding 1.02 79.577 0.907
83.58 1.083 59.274 1.046 91.872
ENSMUSG00000028019 Pdgfc protein_coding 1.01 63.385 0.932
66.651 1.062 76.754 1.118 94.567
ENSMUSG00000028020 Glrb protein_coding 1.075 40.065 0.836
83.58 1.112 88.601 1.003 84.19
ENSMUSG00000028024 Enpep protein_coding 0.821 5.962 1.147 60.309
1.064 57.907 1.037 92.762
ENSMUSG00000028028 Alpk1 protein_coding 1.104 9.983 0.941 82.947
0.962 55.214 1.003 93.156
ENSMUSG00000028029 Aimp1 protein_coding 1.062 12.295 0.916
59.946 1.023 83.275 1.021 92.018
ENSMUSG00000028030 Tbck protein_coding 1.036 61.725 1.054

82.904 0.915 10.362 0.95 86.991
ENSMUSG00000028031 Dkk2 protein_coding 1.466 7.553 1.066 95.941
0.637 7.647 0.757 86.991
ENSMUSG00000028032 Papss1 protein_coding 1.015 82.982 1
95.431 0.985 83.607 0.942 84.29
ENSMUSG00000028033 Kcnq5 protein_coding 1.184 4.159 1.178 76.065
0.718 0.72 0.987 96.287
ENSMUSG00000028034 Fubp1 protein_coding 0.972 86.006 1.031
96.847 0.998 87.898 1.083 79.545
ENSMUSG00000028035 Dnajb4 protein_coding 1.076 24.958
0.973 96.847 0.954 27.675 1.146 43.377
ENSMUSG00000028036 Ptgfr protein_coding 1.049 85.655 0.846
79.261 1.126 43.129 0.958 95.877
ENSMUSG00000028037 Ifi44 protein_coding 1.274 17.336 1.086
96.68 0.722 41.662 1.244 55.485
ENSMUSG00000028039 Efna3 protein_coding 0.871 81.925 0.976
95.301 1.177 56.708 1.121 90.674
ENSMUSG00000028040 Efna4 protein_coding 0.925 65.106 0.92
75.345 1.17 23.334 0.888 84.29
ENSMUSG00000028041 Adam15 protein_coding 0.975 51.447
0.966 88.691 1.062 67.562 1.073 33.269
ENSMUSG00000028042 Zbtb7b protein_coding 0.882 3.401 1.062
84.007 1.069 23.41 0.945 85.493
ENSMUSG00000028044 Cks1b protein_coding 1.087 51.826 0.979
95.708 0.938 59.726 1.091 57.711
ENSMUSG00000028047 Thbs3 protein_coding 0.959 65.199 1.122
87.037 0.931 73.027 1.133 32.698
ENSMUSG00000028048 Gba protein_coding 1.081 38.195 NA NA
0.94 54.037 1.105 0
ENSMUSG00000028049 Scamp3 protein_coding 0.869 0.987 1.108
61.032 1.04 33.787 0.941 92.31
ENSMUSG00000028051 Hcn3 protein_coding 1.097 85.832 0.769
93.094 1.183 52.8 0.842 84.29
ENSMUSG00000028053 Ash1l protein_coding 1.051 44.694 0.953
71.41 1.001 79.554 0.974 85.493
ENSMUSG00000028057 Rit1 protein_coding 1.019 57.949 0.988
96.407 0.994 40.19 1.053 72.826
ENSMUSG00000028059 Arhgef2 protein_coding 0.928 8.938 0.984
96.847 1.094 24.375 1.005 94.904
ENSMUSG00000028060 2810403A07Rik protein_coding 0.955 18.602
0.974 91.361 1.078 21.705 0.995 87.447
ENSMUSG00000028062 Lamtor2 protein_coding 0.854 1.274 1.1
71.617 1.065 25.564 1.059 72.369
ENSMUSG00000028063 Lmna protein_coding 0.97 48.354 1.053
72.546 0.977 68.584 1.045 73.939
ENSMUSG00000028064 Sema4a protein_coding 1.123 3.825 0.864
54.06 1.026 87.519 1.003 94.654
ENSMUSG00000028066 Pmf1 protein_coding 0.995 82.231 1.063
89.014 0.943 62.791 1.121 70.624
ENSMUSG00000028068 Iqgap3 protein_coding 1.285 39.075
0.739 62.025 1.058 85.792 1.25 77.245
ENSMUSG00000028069 Gpatch4 protein_coding 0.961 49.749
0.964 87.322 1.077 42.522 1.181 50.452
ENSMUSG00000028070 Apoa1bp protein_coding 0.942 15.76 0.994

94.887 1.07 20.051 0.983 93.316
ENSMUSG00000028071 Sh2d2a protein_coding 0.849 59.358 1.25
82.904 0.945 57.819 0.881 88.505
ENSMUSG00000028072 Ntrk1 protein_coding 1.026 72.372 1.085
96.268 0.895 78.713 1.409 87.134
ENSMUSG00000028073 Pear1 protein_coding 0.984 62.888 0.904
92.355 1.124 49.601 1.032 89.052
ENSMUSG00000028076 Cd1d1 protein_coding 0.888 4.634 1.083 87.609
1.039 54.673 0.98 94.99
ENSMUSG00000028078 Dclk2 protein_coding 1.083 82.231 0.942
92.355 0.982 62.995 0.91 86.991
ENSMUSG00000028080 Lrba protein_coding 1 81.647 0.97
81.284 1.031 47.31 0.979 94.654
ENSMUSG00000028081 Rps3a1 protein_coding 1.155 0.461 0.902
34.058 0.96 27.675 1.014 95.273
ENSMUSG00000028082 Sh3d19 protein_coding 1.029 78.428
1.075 79.261 0.906 32.297 1.03 87.088
ENSMUSG00000028085 Pet112 protein_coding 0.974 77.615
1.168 63.504 0.879 42.376 1.001 95.74
ENSMUSG00000028086 Fbxw7 protein_coding 1.082 15.85 0.952 71.41
0.969 46.32 1.004 90.854
ENSMUSG00000028088 Fmo5 protein_coding 1.118 52.134 0.958
75.493 0.935 76.923 0.937 96.248
ENSMUSG00000028089 Chd1l protein_coding 1.013 80.146 0.982
91.897 1.006 87.898 1.01 80.497
ENSMUSG00000028093 Acp6 protein_coding 0.92 25.492 1.059
83.104 1.022 78.369 0.937 84.29
ENSMUSG00000028096 Gpr89 protein_coding 0.989 84.865 1.07
76.086 0.947 16.949 1.031 87.886
ENSMUSG00000028098 Rnf115 protein_coding 1.094 0.2 NA NA
0.879 1.061 1.036 94.825
ENSMUSG00000028099 Polr3c protein_coding 0.982 72.743
0.934 60.678 1.091 27.732 1.023 94.904
ENSMUSG00000028100 Nudt17 protein_coding 0.718 1.017 1.052
95.809 1.326 7.24 0.943 85.493
ENSMUSG00000028101 Pias3 protein_coding 0.995 72.372 1.078
71.98 0.93 2.563 1.035 96.248
ENSMUSG00000028102 Pex11b protein_coding 0.967 59.358
1.005 96.652 1.029 57.637 1.03 83.276
ENSMUSG00000028104 Polr3gl protein_coding 0.934 9.508 0.935
79.475 1.141 16.808 0.986 89.587
ENSMUSG00000028106 Rprd2 protein_coding 1.093 2.085 1.044 85.756
0.877 1.92 0.939 83.724
ENSMUSG00000028107 Tars2 protein_coding 0.923 2.142 1.002 92.793
1.076 22.994 0.986 93.132
ENSMUSG00000028108 Ecm1 protein_coding 1.141 7.553 0.986 90.287
0.889 39.528 1 93.964
ENSMUSG00000028109 Hormad1 protein_coding 1 78.744 1
96.847 NA NA 1 93.264
ENSMUSG00000028111 Ctsk protein_coding 1.159 77.497 1.1
82.904 0.781 30.468 0.965 95.35
ENSMUSG00000028114 NA NA NA NA 1.008 96.268 1.012
88.203 1.009 96.192
ENSMUSG00000028115 Bnipl protein_coding 0.83 58.56 0.908 74.851

1.326 28.301 1.018 92.466
ENSMUSG00000028121 Bcar3 protein_coding 1.116 85.092 1.115
90.116 0.8 43.842 0.932 94.904
ENSMUSG00000028124 Gclm protein_coding 0.659 1.703 1.075 90.256
1.406 6.506 1 92.675
ENSMUSG00000028125 Abca4 protein_coding 0.743 23.13 0.934 76.431
1.438 12.703 0.902 95.273
ENSMUSG00000028126 Pip5k1a protein_coding 1.003 79.068
1.043 82.904 0.951 35.559 0.935 83.724
ENSMUSG00000028127 Abcd3 protein_coding 0.987 71.36 0.971 89.509
1.039 40.052 0.978 95.74
ENSMUSG00000028128 F3 protein_coding 0.693 27.836 1.142
79.29 1.259 39.925 1.058 70.85
ENSMUSG00000028132 Tmem56 protein_coding 0.985 60.003
0.854 68.818 1.191 50.851 0.927 85.493
ENSMUSG00000028133 Rwdd3 protein_coding 0.911 58.802 0.876
68.115 1.253 16.808 0.829 84.19
ENSMUSG00000028134 Ptbp2 protein_coding 0.968 57.227 0.868
20.174 1.188 5.964 0.98 95.72
ENSMUSG00000028136 Snx27 protein_coding 1.101 2.047 NA NA
0.912 7.329 1.012 71.855
ENSMUSG00000028137 Celf3 protein_coding 0.646 7.857 1.011 91.532
1.534 9.502 0.658 85.493
ENSMUSG00000028138 Adh5 protein_coding 0.898 2.174 0.986 79.475
1.13 7.635 1.055 80.77
ENSMUSG00000028139 Riiad1 protein_coding 1.123 62.689
0.736 72.576 1.211 55.512 0.968 88.387
ENSMUSG00000028140 Mrpl9 protein_coding 0.995 81.178 0.945
80.463 1.064 56.708 0.968 84.29
ENSMUSG00000028141 Oaz3 protein_coding 0.785 52.258 0.876
92.111 1.457 78.498 NA NA
ENSMUSG00000028145 Them4 protein_coding 0.939 24.817 1.082
72.642 0.987 65.409 1.078 71.305
ENSMUSG00000028148 Them5 protein_coding 1.271 71.763 0.61
85.116 1.294 45.94 0.786 96.071
ENSMUSG00000028149 Rap1gds1 protein_coding 1.048 17.582
1.041 91.643 0.918 20.511 1.117 31.133
ENSMUSG00000028150 Rorc protein_coding 0.755 6.677 0.797 95.504
1.663 15.688 1.011 94.608
ENSMUSG00000028152 Tspan5 protein_coding 1.022 86.474
0.932 73.166 1.05 53.882 1.087 58.721
ENSMUSG00000028156 Eif4e protein_coding 1.056 43.939 1.027
91.584 0.923 27.86 1.114 40.024
ENSMUSG00000028158 Mttp protein_coding 0.905 58.56 0.979 84.594
1.125 43.586 0.954 85.947
ENSMUSG00000028159 Dapp1 protein_coding 1.015 77.113 1.047
83.104 0.938 77.294 0.924 86.699
ENSMUSG00000028161 Ppp3ca protein_coding 1.213 0 1.011
95.504 0.814 0.72 1.019 74.607
ENSMUSG00000028163 Nfkb1 protein_coding 1.031 26.136 0.976
85.242 0.995 88.274 0.948 83.724
ENSMUSG00000028164 Manba protein_coding 1.234 0 0.904 37.376
0.897 4.759 0.942 86.991
ENSMUSG00000028165 Cisd2 protein_coding 1.016 82.982 NA NA

0.981 84.552 1.06 48.177
ENSMUSG00000028167 Bdh2 protein_coding 1.524 17.336 0.878
92.355 0.748 34.21 0.812 85.493
ENSMUSG00000028172 Tacr3 protein_coding 1.118 57.949 0.936
95.088 0.954 72.269 0.911 91.291
ENSMUSG00000028173 Wls protein_coding 1.127 0 0.967 90.046
0.917 9.494 0.944 86.699
ENSMUSG00000028174 Rpe65 protein_coding 1 85.203 1
93.654 1 86.602 1 95.877
ENSMUSG00000028175 Depdc1a protein_coding 0.877 77.916
0.837 83.58 1.361 84.504 2.078 60.31
ENSMUSG00000028179 Cth protein_coding 0.838 31.921 1.166
77.598 1.02 78.293 0.674 83.724
ENSMUSG00000028180 Zranb2 protein_coding 1.008 66.76 0.958
38.923 1.031 24.383 0.998 94.654
ENSMUSG00000028182 Lrriq3 protein_coding 1.243 13.734
0.885 85.95 0.912 36.499 0.841 83.724
ENSMUSG00000028184 Lphn2 protein_coding 1.033 39.075 1.086
79.411 0.888 9.456 0.896 83.724
ENSMUSG00000028185 Dnase2b protein_coding 1 83.09 1
95.708 1 84.626 1 86.445
ENSMUSG00000028186 Uox protein_coding 1 79.277 1
96.843 1 88.461 1 92.181
ENSMUSG00000028187 Rpf1 protein_coding 1.009 73.585 0.977
89.713 1.014 65.567 1.007 93.612
ENSMUSG00000028188 Spata1 protein_coding 0.862 13.581
1.046 94.102 1.11 17.412 0.992 93.316
ENSMUSG00000028189 Ctbs protein_coding 1.1 18.985 0.906
73.166 1.005 87.786 1.031 75.198
ENSMUSG00000028191 Bcl10 protein_coding 1.122 6.541 0.963 93.654
0.925 63.075 0.946 85.493
ENSMUSG00000028194 Ddah1 protein_coding 0.94 69.943 NA NA
1.197 83.275 1.136 44.882
ENSMUSG00000028195 Cyr61 protein_coding 1.352 16.74 0.678 65.819
1.093 85.352 1.568 67.169
ENSMUSG00000028197 Col24a1 protein_coding 0.772 55.303
1.208 82.904 1.076 88.161 1.118 85.577
ENSMUSG00000028199 Cryz protein_coding 1.018 49.202 1.097
94.767 0.899 43.426 0.965 87.182
ENSMUSG00000028207 Asph protein_coding 0.857 27 0.842 88.691
1.388 16.666 1.092 69.708
ENSMUSG00000028211 Trp53inp1 protein_coding 1.162 67.991
0.636 39.056 1.357 9.334 0.923 92.31
ENSMUSG00000028212 Ccne2 protein_coding 0.881 79.165 1.014
96.216 1.119 83.719 1.31 65.558
ENSMUSG00000028214 Gem protein_coding 1.725 0 0.877 79.894
0.661 0.72 0.908 83.724
ENSMUSG00000028218 Fam92a protein_coding 0.998 85.391
0.928 85.418 1.082 51.596 1.053 90.177
ENSMUSG00000028221 Tmem55a protein_coding 1.146 0.577 1.049
88.923 0.829 0.72 0.953 87.331
ENSMUSG00000028222 Calb1 protein_coding 1 84.534 1
96.216 1 88.461 1 95.74
ENSMUSG00000028223 NA NA NA NA NA NA 1.007 86.084

0.974 94.654
ENSMUSG00000028224 Nbn protein_coding 1.066 36.686 1.042
82.904 0.896 18.539 0.985 94.297
ENSMUSG00000028226 Mmp16 protein_coding 0.885 53.138 1.022
88.691 1.103 58.051 1.368 64.513
ENSMUSG00000028228 Cpne3 protein_coding 1.038 26.77 1.041 92.561
0.926 25.625 0.939 84.19
ENSMUSG00000028229 Rmdn1 protein_coding 1.046 84.865 1.018
96.216 0.937 80.163 0.951 88.387
ENSMUSG00000028232 Tmem68 protein_coding 1.144 9.983 0.904
72.576 0.97 69.445 0.975 95.593
ENSMUSG00000028233 Tgs1 protein_coding 1.039 43.019 1.023
88.092 0.938 33.137 1.006 95.593
ENSMUSG00000028234 Rps20 protein_coding 1.089 19.477 0.904
60.678 1.019 87.744 0.995 96.183
ENSMUSG00000028236 Sdr16c5 protein_coding 1 83.333 1
95.301 1 68.425 1 95.832
ENSMUSG00000028238 Atp6v0d2 protein_coding 1.533 2.567 0.844
79.807 0.773 27.994 0.971 92.181
ENSMUSG00000028240 Cyp7a1 protein_coding 1 85.283 NA
NA 1 80.089 1 95.35
ENSMUSG00000028243 Ubxn2b protein_coding 0.945 48.036
1.008 96.378 1.051 66.928 1.096 53.923
ENSMUSG00000028244 1700025O08Rik lincRNA 1 81.24 1
97.049 1 75.967 1 94.904
ENSMUSG00000028245 Nsmaf protein_coding 0.935 62.888 0.93
56.027 1.146 10.523 1.008 94.904
ENSMUSG00000028246 Faxc protein_coding 1.02 83.263 0.948
94.597 1.035 80.015 0.941 87.496
ENSMUSG00000028247 Coq3 protein_coding 0.882 25.551 NA NA
1.078 30.213 1.06 92.903
ENSMUSG00000028248 Sfrs18 protein_coding 0.875 1.698 0.979
42.923 1.166 5.864 0.983 94.19
ENSMUSG00000028249 Sdcbp protein_coding 1.11 6.135 0.988 91.532
0.909 8.382 1.009 88.797
ENSMUSG00000028251 Tstd3 protein_coding 1.013 75.399 1.091
87.609 0.905 39.528 1.223 44.679
ENSMUSG00000028252 Ccnc protein_coding 1.014 73.124 0.998
94.887 0.985 78.045 1.12 69.404
ENSMUSG00000028255 Clca3 protein_coding 2.021 12.396 0.643
75.345 0.766 88.349 1.022 85.629
ENSMUSG00000028256 Odf2l protein_coding 1.022 58.198 0.999
95.329 0.981 77.294 1.106 94.82
ENSMUSG00000028259 Fhl5 protein_coding 0.954 74.299 0.782
34.058 1.345 7.482 0.97 93.261
ENSMUSG00000028261 Ndufaf4 protein_coding 0.92 43.435
1.035 92.253 1.051 65.034 1.091 44.882
ENSMUSG00000028262 Clca2 protein_coding 1.267 9.983 0.988 92.631
0.797 11.314 0.811 85.493
ENSMUSG00000028264 Spaca1 protein_coding 1.013 60.168
0.957 92.931 1.028 40.19 0.939 85.493
ENSMUSG00000028266 Lmo4 protein_coding 1.05 38.682 0.962
96.268 0.989 55.9 0.947 84.19
ENSMUSG00000028268 Gbp3 protein_coding 1.066 47.174 1.124

82.904 0.833 22.864 1.299 36.988
ENSMUSG00000028269 Gbp2b protein_coding 2.155 19.477 0.489
56.027 0.946 86.084 0.609 84.29
ENSMUSG00000028270 Gbp2 protein_coding 1.075 59.806 1.117
83.104 0.833 19.449 1.043 80.573
ENSMUSG00000028271 Gtf2b protein_coding 1.157 7.842 0.937 85.921
0.922 23.938 0.957 95.593
ENSMUSG00000028273 Pdlim5 protein_coding 1.076 16.124
0.956 74.483 0.974 59.125 0.93 86.699
ENSMUSG00000028274 Rngtt protein_coding 0.936 59.674 1.074
93.574 0.995 86.475 1.09 38.598
ENSMUSG00000028277 Ube2j1 protein_coding 1.042 49.517
1.012 96.68 0.95 71.004 1.037 67.691
ENSMUSG00000028278 Rragd protein_coding 1.956 15.456 1.48
78.494 0.345 0.879 0.887 95.832
ENSMUSG00000028282 Casp8ap2 protein_coding 0.923 48.646
1.085 83.104 1 67.648 1.05 87.447
ENSMUSG00000028284 Map3k7 protein_coding 1.004 78.639
1.024 96.847 0.971 59.125 1.016 78.379
ENSMUSG00000028287 1700009N14Rik protein_coding 1 74.689
1 93.654 1 87.336 1 94.654
ENSMUSG00000028289 Epha7 protein_coding 0.99 69.715 0.963
96.843 1.051 75.155 0.958 85.636
ENSMUSG00000028291 Akirin2 protein_coding 1.125 2.723 1.004
97.263 0.887 3.234 0.991 94.608
ENSMUSG00000028292 Rars2 protein_coding 1.016 84.294 NA NA
0.976 76.768 1.013 84.246
ENSMUSG00000028293 Slc35a1 protein_coding 1.062 28.395
0.957 88.345 0.98 73.096 1.001 86.301
ENSMUSG00000028295 Smim8 protein_coding 0.891 24.748 1.034
91.643 1.085 38.683 1.058 59.464
ENSMUSG00000028298 Cga protein_coding 1 81.125 1
83.392 1 86.144 1 92.851
ENSMUSG00000028300 3110043O21Rik protein_coding 1.071 48.288
1.047 90.29 0.892 28.489 0.91 84.29
ENSMUSG00000028309 Rnf20 protein_coding 1.041 65.733 0.952
85.418 1.005 79.554 1.064 46.846
ENSMUSG00000028312 Smc2 protein_coding 1.118 24.253 1.02
86.728 0.879 71.004 1.218 57.1
ENSMUSG00000028314 4930547C10Rik protein_coding 1 62.574
1 88.345 1 83.538 1 92.389
ENSMUSG00000028318 Polr1e protein_coding 0.993 84.263 NA
NA 0.99 81.525 1.082 81.251
ENSMUSG00000028322 Exosc3 protein_coding 1.085 5.527 1
85.075 0.918 27.321 1.027 87.886
ENSMUSG00000028327 1300002K09Rik protein_coding 1.369 36.438
1.378 96.948 0.529 23.607 0.462 83.724
ENSMUSG00000028328 Tmod1 protein_coding 1.478 3.151 0.994 96.843
0.679 4.23 1.193 44.882
ENSMUSG00000028329 Xpa protein_coding 1.095 47.174 0.836
35.072 1.094 15.241 0.903 84.29
ENSMUSG00000028330 Ncbp1 protein_coding 0.931 24.577 1.019
95.809 1.057 43.586 1.078 78.042
ENSMUSG00000028331 5830415F09Rik protein_coding 1.008 76.319

1.097 82.904 0.902 31.887 1.012 91.625
ENSMUSG00000028333 Anp32b protein_coding 1.038 58.198
0.925 15.938 1.043 41.235 1.15 0
ENSMUSG00000028334 Nans protein_coding 1.063 41.177 0.96
96.407 0.976 52.087 1.153 31.133
ENSMUSG00000028337 Coro2a protein_coding 0.865 18.379 0.79
65.165 1.461 12.16 0.947 88.136
ENSMUSG00000028339 Col15a1 protein_coding 0.759 3.895 1.17
81.127 1.126 75.009 1.207 70.624
ENSMUSG00000028341 Nr4a3 protein_coding 0.927 75.567 0.909
92.137 1.19 66.043 0.815 88.245
ENSMUSG00000028343 Erp44 protein_coding 1.115 12.396 0.915
66.796 0.978 45.093 1.095 31.133
ENSMUSG00000028344 Invs protein_coding 0.966 67.477 0.971
95.196 1.063 84.961 1.088 63.018
ENSMUSG00000028345 Tex10 protein_coding 1.027 36.124 0.984
91.275 0.988 88.349 1.039 85.786
ENSMUSG00000028347 Tmeff1 protein_coding 1.064 61.171
1.024 91.456 0.916 54.521 0.864 83.724
ENSMUSG00000028348 Murc protein_coding 1.739 11.044 0.851
91.661 0.674 6.858 1.173 92.691
ENSMUSG00000028351 Brinp1 protein_coding 1.057 86.351
1.254 96.216 0.753 82.685 0.75 86.699
ENSMUSG00000028354 Fmn2 protein_coding 1.213 17.191 1.069
96.843 0.772 6.858 0.895 96.248
ENSMUSG00000028358 Zfp618 protein_coding 1.217 20.032
0.991 94.89 0.826 20.751 0.902 86.254
ENSMUSG00000028359 Orm3 protein_coding 0.67 16.02 1.403 82.809
1.063 64.024 0.856 86.445
ENSMUSG00000028360 Slc44a5 protein_coding 0.907 80.608
0.882 95.708 NA NA 1.009 78.948
ENSMUSG00000028362 Tnfsf8 protein_coding 1.008 81.125
1.483 86.793 0.67 32.297 1.179 67.169
ENSMUSG00000028364 Tnc protein_coding 1.071 69.408 1.082
92.062 0.86 48.851 0.948 92.742
ENSMUSG00000028367 Txn1 protein_coding 1.017 83.418 1.062
83.104 0.923 83.607 1.056 86.301
ENSMUSG00000028369 Svep1 protein_coding 1.031 53.809 1.03
97.263 0.945 53.224 1.047 91.928
ENSMUSG00000028370 Pappa protein_coding 0.733 1.488 NA NA
1.414 10.819 1.096 93.734
ENSMUSG00000028373 Astn2 protein_coding 1.05 55.789 1.121
96.216 0.85 25.625 1.066 95.593
ENSMUSG00000028378 Ptgr1 protein_coding 1.18 61.171 0.891
61.883 0.954 84.626 1.093 72.992
ENSMUSG00000028381 Ugcg protein_coding 1.177 0 0.986 96.216
0.863 1.898 0.932 91.372
ENSMUSG00000028382 Ptbp3 protein_coding 1.037 22.871 0.952
79.261 1.011 86.727 0.986 84.29
ENSMUSG00000028383 Hsdl2 protein_coding 0.967 56.242 NA NA
1.012 69.002 0.94 92.675
ENSMUSG00000028385 Snx30 protein_coding 1.178 0.435 0.924 59.946
0.921 16.409 1.027 94.99
ENSMUSG00000028386 Slc46a2 protein_coding 0.481 51.56 1.564

83.104 1.327 66.425 0.707 83.724
ENSMUSG00000028389 Zfp37 protein_coding 1.077 39.851 0.984
95.301 0.944 67.029 0.96 93.264
ENSMUSG00000028391 Wdr31 protein_coding 0.888 61.633 0.885
87.998 1.277 20.886 0.817 90.805
ENSMUSG00000028392 Bspry protein_coding 0.946 84.819 0.822
67.513 1.285 65.034 0.977 94.001
ENSMUSG00000028393 Alad protein_coding 0.878 8.614 1.165 76.086
0.978 88.076 1.041 80.573
ENSMUSG00000028394 Pole3 protein_coding 1.034 36.124 1.023
92.253 0.947 24.523 1.067 73.368
ENSMUSG00000028397 Kdm4c protein_coding 1.029 35.499 0.977
85.242 0.998 77.037 1.009 93.132
ENSMUSG00000028398 Tmem261 protein_coding 0.928 16.895
1.055 82.904 1.023 81.032 1.008 96.139
ENSMUSG00000028399 Ptprd protein_coding 0.912 8.302 1.042 90.29
1.047 44.361 1.119 57.1
ENSMUSG00000028402 Mpdz protein_coding 0.916 5.778 1.062 71.98 1.03
81.29 1.148 46.846
ENSMUSG00000028403 Zdhhc21 protein_coding 1.016 68.16 0.967
75.493 1.017 79.554 0.976 95.74
ENSMUSG00000028405 Aco1 protein_coding 0.951 10.143 1.068
60.309 0.979 86.666 0.912 93.045
ENSMUSG00000028407 2010003O02Rik antisense 0.875 35.449
1.091 93.574 1.049 79.625 0.839 83.724
ENSMUSG00000028409 Smu1 protein_coding 1.055 44.061 0.968
75.345 0.975 85.757 1.035 55.841
ENSMUSG00000028410 Dnaja1 protein_coding 0.942 51.56 1.153
91.456 NA NA 1.071 67.691
ENSMUSG00000028411 Aptx protein_coding 0.963 49.749 0.938
69.175 1.105 18.411 0.973 87.735
ENSMUSG00000028412 Slc44a1 protein_coding 1.135 0.2 1.01
95.708 0.873 0.72 0.953 84.29
ENSMUSG00000028413 B4galt1 protein_coding 1.195 7.65 1.049
93.529 0.796 5.336 1.048 83.581
ENSMUSG00000028414 Fktn protein_coding 1.052 57.133 1.102
83.104 0.864 22.735 0.941 83.724
ENSMUSG00000028415 Spink4 protein_coding 1 66.58 1
83.321 1 77.888 1 91.625
ENSMUSG00000028416 Bag1 protein_coding 0.89 3.325 1.078 85.756
1.041 38.152 1.025 93.156
ENSMUSG00000028417 Tal2 protein_coding 0.802 63.126 1.527
83.104 0.818 81.169 0.996 95.273
ENSMUSG00000028419 Chmp5 protein_coding 0.947 42.034 1.006
94.887 1.05 35.673 1.094 31.133
ENSMUSG00000028420 Tmem38b protein_coding 1.041 77.291 1.04
86.387 0.926 39.528 0.981 94.654
ENSMUSG00000028423 Nfx1 protein_coding 1.091 9.206 0.921 26.053
0.992 78.498 0.961 90.674
ENSMUSG00000028426 Rad23b protein_coding 1.007 52.799
0.995 91.361 0.994 78.045 1.083 31.133
ENSMUSG00000028427 Aqp7 protein_coding 0.886 51.056 1.053
95.708 1.07 58.915 0.964 87.331
ENSMUSG00000028430 Nol6 protein_coding 0.881 3.956 1.064 62.898

1.065 71.839 1.046 76.046
ENSMUSG00000028431 Ikbkap protein_coding 1.044 13.105
0.906 28.822 1.052 51.977 1.045 63.114
ENSMUSG00000028433 Ubap2 protein_coding 0.981 49.047 1.053
82.904 0.97 70.44 1.032 54.265
ENSMUSG00000028434 Epb4.1l4b protein_coding 1.188 5.313 0.929
62.71 0.904 73.598 0.937 90.157
ENSMUSG00000028435 Aqp3 protein_coding 1.741 52.986 1.821
96.308 0.314 38.799 0.561 92.263
ENSMUSG00000028436 Dcaf12 protein_coding 1.004 62.354
1.081 82.904 0.92 11.504 0.978 89.525
ENSMUSG00000028437 Ubap1 protein_coding 1.009 67.163 0.97
85.242 1.018 71.839 1.02 73.401
ENSMUSG00000028438 Kif24 protein_coding 0.828 43.495 1.227
83.104 0.983 82.575 1.109 95.35
ENSMUSG00000028439 Fam219a protein_coding 0.927 26.483
0.983 97.288 1.099 57.907 1.057 86.209
ENSMUSG00000028441 1110017D15Rik protein_coding 0.412 7.916
1.432 80.775 1.693 27.89 1.632 61.753
ENSMUSG00000028443 Nudt2 protein_coding 0.965 74.299 1.014
93.899 1.021 86.552 1.028 93.156
ENSMUSG00000028444 Cntfr protein_coding 0.69 7.218 1.06 87.037
1.369 23.668 1.083 83.581
ENSMUSG00000028445 Enho protein_coding 0.573 0.987 NA NA
1.685 5.864 0.999 96.086
ENSMUSG00000028447 Dctn3 protein_coding 0.816 0.987 0.979 91.275
1.253 5.864 1.111 40.024
ENSMUSG00000028451 1700022I11Rik protein_coding 0.668 1.239
1.095 79.411 1.371 7.635 0.874 85.493
ENSMUSG00000028452 Vcp protein_coding 0.938 6.822 1.04 88.533
1.023 38.152 1.077 31.133
ENSMUSG00000028453 Fancg protein_coding 0.971 85.637 1.004
95.211 1.024 81.032 1.029 96.358
ENSMUSG00000028454 Pigo protein_coding 0.826 1.175 1.046 83.104
1.161 11.151 1.012 93.264
ENSMUSG00000028455 Stoml2 protein_coding 0.916 3.956 1.007
95.211 1.08 20.572 1.118 62.918
ENSMUSG00000028456 Unc13b protein_coding 1.018 82.352
0.912 51.277 1.073 51.596 0.881 95.74
ENSMUSG00000028457 Atp8b5 protein_coding 1.447 21.703
0.951 90.576 0.727 31.887 0.911 95.273
ENSMUSG00000028458 Tesk1 protein_coding 0.956 73.585 1.062
82.904 0.988 46.32 0.986 84.29
ENSMUSG00000028459 Cd72 protein_coding 1.064 79.853 1.021
83.104 0.915 16.719 1.118 69.918
ENSMUSG00000028460 Sit1 protein_coding 1.111 65.537 1.401
92.931 0.644 25.625 0.642 87.705
ENSMUSG00000028461 Ccdc107 protein_coding 0.936 25.551 NA
NA 1.084 28.979 0.948 86.991
ENSMUSG00000028463 Car9 protein_coding 0.983 77.113 1.171
96.847 0.865 86.666 1.037 96.358
ENSMUSG00000028464 Tpm2 protein_coding 1.385 17.761 1.265
71.222 0.568 0.72 0.899 94.19
ENSMUSG00000028465 Tln1 protein_coding 0.956 38.983 1.053

83.104	0.99	84.724	1.024	72.826
ENSMUSG00000028466	Creb3	protein_coding	0.985	84.397	0.996
97.263	1.021	88.601	1.041	72.992
ENSMUSG00000028467	Gba2	protein_coding	0.876	6.822	0.977	95.35
1.168	11.518	1.014	95.273
ENSMUSG00000028468	Rgp1	protein_coding	0.907	5.962	1.016	90.116
1.088	23.454	0.989	96.086
ENSMUSG00000028469	Npr2	protein_coding	0.964	60.707	1.061
82.809	0.978	83.158	1.049	83.067
ENSMUSG00000028470	Hint2	protein_coding	0.914	11.128	1.024
92.111	1.069	33.042	0.923	85.493
ENSMUSG00000028475	5430416O09Rik	lincRNA	0.986	61.043
0.864	38.699	1.169	7.024	0.956	96.248
ENSMUSG00000028476	Reck	protein_coding	1.113	32.676	0.96
90.378	0.935	63.504	1.049	71.653
ENSMUSG00000028478	Clta	protein_coding	1.007	56.311	0.972
70.971	1.018	80.899	1.081	79.268
ENSMUSG00000028479	Gne	protein_coding	0.959	8.938	0.975	93.817
1.072	23.41	1.002	77.331
ENSMUSG00000028480	Glipr2	protein_coding	1.14	21.836
0.919	90.256	0.956	41.154	1.249	44.882
ENSMUSG00000028483	Snapc3	protein_coding	0.923	15.76	NA	NA
1.041	54.363	0.983	87.992
ENSMUSG00000028484	Psip1	protein_coding	1.008	73.124	0.958
79.475	1.038	84.308	1.108	55.054
ENSMUSG00000028487	Bnc2	protein_coding	0.8	31.11	1.291	64.304
0.965	87.519	1.536	79.442
ENSMUSG00000028488	Sh3gl2	protein_coding	1.22	30.984
0.836	81.731	0.976	61.743	0.879	86.991
ENSMUSG00000028492	Fam154a	protein_coding	1	86.351	1
96.216	1	86.985	1	90.674
ENSMUSG00000028494	Plin2	protein_coding	0.918	3.559	NA	NA	1.09
30.292	1.098	70.539
ENSMUSG00000028495	Rps6	protein_coding	1.077	15.178	0.906
35.072	1.027	65.235	1.062	53.923
ENSMUSG00000028496	Mllt3	protein_coding	1.103	4.628	1.002	93.654
0.902	13.848	1.045	94.215
ENSMUSG00000028497	Ptplad2	protein_coding	1.583	0	0.863
47.727	0.732	6.858	0.96	96.358
ENSMUSG00000028514	Usp24	protein_coding	1.009	84.564	0.944
86.728	1.051	60.789	1.024	96.042
ENSMUSG00000028517	Ppap2b	protein_coding	1.199	0.461	0.981
72.399	0.847	17.662	1	92.181
ENSMUSG00000028518	Prkaa2	protein_coding	1.21	24.253
1.067	79.864	0.772	2.563	0.962	96.192
ENSMUSG00000028520	4921539E11Rik	protein_coding	1	86.464
1	96.843	1	85.935	1	93.722
ENSMUSG00000028521	Slc35d1	protein_coding	1.009	86.199
1.075	71.617	0.92	35.559	0.982	96.183
ENSMUSG00000028522	Mier1	protein_coding	1.094	1.242	0.99	95.088
0.925	13.848	0.985	89.475
ENSMUSG00000028523	Tctex1d1	protein_coding	1	42.722	1
88.823	1	84.83	1	96.287
ENSMUSG00000028524	Sgip1	protein_coding	1.263	6.438	0.925	74.483

0.856 42.997 0.842 83.724
ENSMUSG00000028525 Pde4b protein_coding 0.852 61.633 1.065
93.899 1.101 69.196 0.927 90.289
ENSMUSG00000028527 Ak4 protein_coding 1.187 9.435 0.937 88.691
0.9 26.809 1.045 72.616
ENSMUSG00000028528 Dnajc6 protein_coding 1.078 33.325
0.894 75.345 NA NA 0.907 89.827
ENSMUSG00000028530 Jak1 protein_coding 1.059 4.052 1.01 96.268
0.936 19.449 1.08 48.857
ENSMUSG00000028532 Cachd1 protein_coding 1.127 78.639
1.136 93.574 0.781 78.713 0.823 83.724
ENSMUSG00000028533 Izumo3 protein_coding 1 85.092 1
96.013 1 88.601 1 94.82
ENSMUSG00000028536 2610528J11Rik protein_coding 0.823 17.276
1.104 96.407 1.104 46.575 0.82 92.12
ENSMUSG00000028538 St3gal3 protein_coding 0.975 49.944
1.021 76.086 1.001 71.004 0.945 84.29
ENSMUSG00000028539 Artn protein_coding 1.177 37.091 0.867
93.654 0.981 85.675 0.736 83.724
ENSMUSG00000028540 Dph2 protein_coding 0.986 81.01 0.981 91.776
1.03 75.353 1.111 65.558
ENSMUSG00000028541 B4galt2 protein_coding 1.08 49.868 1.03
90.952 0.9 23.607 1.256 31.133
ENSMUSG00000028542 Slc6a9 protein_coding 1.112 11.459
0.968 96.847 0.924 7.509 0.916 86.699
ENSMUSG00000028544 Slc5a9 protein_coding 0.602 3.641 1.061
96.68 1.565 32.524 0.694 83.724
ENSMUSG00000028545 Bend5 protein_coding 0.901 70.597 0.972
94.102 1.14 70.9 0.914 88.505
ENSMUSG00000028549 Itgb3bp protein_coding 0.839 15.227
1.039 94.89 1.146 36.95 0.949 92.181
ENSMUSG00000028550 Atg4c protein_coding 1.012 66.272 1.093
64.304 0.906 2.563 0.928 83.724
ENSMUSG00000028551 Cdkn2c protein_coding 1.006 80.51 1.21
62.898 0.819 14.206 0.889 86.699
ENSMUSG00000028552 Eps15 protein_coding 1.11 11.879 0.9
52.266 0.997 84.229 1.082 92.691
ENSMUSG00000028553 Angptl3 protein_coding 0.988 75.858
0.937 89.713 1.08 43.185 1.136 82.328
ENSMUSG00000028555 Ttc39a protein_coding 0.922 59.674
0.719 11.228 1.508 6.202 1.194 95.273
ENSMUSG00000028556 Dock7 protein_coding 1.175 0.2 1.002 93.765
0.848 24.523 0.897 83.724
ENSMUSG00000028557 NA NA NA NA 1.057 82.904 0.935
43.426 0.972 91.372
ENSMUSG00000028558 Calr4 protein_coding 1.055 66.272 0.978
96.216 0.972 84.552 0.942 94.016
ENSMUSG00000028559 Osbpl9 protein_coding 0.973 52.429 0.95
51.115 1.076 50.091 1.166 70.539
ENSMUSG00000028560 Usp1 protein_coding 1.096 32.411 0.978
92.137 0.934 49.733 1.077 50.452
ENSMUSG00000028563 Tm2d1 protein_coding 1.008 81.24 0.984 82.947
1.009 83.857 0.972 84.29
ENSMUSG00000028565 Nfia protein_coding 1.156 17.582 0.962

97.263	0.9	27.86	0.926	85.493
ENSMUSG00000028567	Txndc12	protein_coding	1.01	67.163
0.992	92.404	0.998	86.985	1.03	92.018
ENSMUSG00000028568	Btf3l4	protein_coding	0.999	85.946
0.962	59.946	1.04	25.455	NA	NA
ENSMUSG00000028571	Cyp2j13	protein_coding	1	84.865	1
96.216	NA	NA	1	93.584
ENSMUSG00000028572	Hook1	protein_coding	0.959	74.482	0.846
59.396	1.235	38.099	0.984	93.734
ENSMUSG00000028573	Fggy	protein_coding	1.071	54.717	0.952
90.138	0.978	86.727	1.043	85.786
ENSMUSG00000028575	Eqtn	protein_coding	1.817	21.146	0.579
51.322	0.954	83.719	0.616	89.587
ENSMUSG00000028576	Ift74	protein_coding	1.197	13.363	0.924
83.876	0.906	52.087	1.028	84.499
ENSMUSG00000028577	Plaa	protein_coding	1.094	10.687	0.919
79.475	0.995	54.25	1.07	68.354
ENSMUSG00000028578	Caap1	protein_coding	1.084	19.132	0.967
90.053	0.952	53.224	0.949	85.493
ENSMUSG00000028580	Pum1	protein_coding	0.988	86.474	1.008
93.654	1.001	77.532	1.008	87.735
ENSMUSG00000028581	Laptm5	protein_coding	1.512	0	0.848
68.115	0.779	5.628	1.115	67.94
ENSMUSG00000028582	Cc2d1b	protein_coding	0.943	9.599	0.985
79.807	1.078	6.449	1.055	48.857
ENSMUSG00000028583	Pdpn	protein_coding	1.196	0.355	1.009	93.654
0.827	8.634	1.162	31.133
ENSMUSG00000028587	Orc1	protein_coding	2.154	34.439	0.549
79.894	0.847	83.811	1.474	57.1
ENSMUSG00000028591	Pramef12	protein_coding	0.762	49.368
0.996	91.955	1.32	25.564	1.194	83.067
ENSMUSG00000028593	9430007A20Rik	protein_coding	1	82.092
1	96.216	1	84.896	1	93.627
ENSMUSG00000028597	Gpx7	protein_coding	1.146	41.211	0.961
96.68	0.906	25.688	1.159	37.634
ENSMUSG00000028599	Tnfrsf1b	protein_coding	1.357	0	0.899
66.651	0.815	5.628	1.054	80.324
ENSMUSG00000028600	Podn	protein_coding	0.914	79.433	0.913
95.941	NA	NA	1.058	92.748
ENSMUSG00000028601	Echdc2	protein_coding	0.917	7.574	0.992
86.861	1.102	7.365	0.9	83.724
ENSMUSG00000028602	Tnfrsf8	protein_coding	0.687	15.06	1.354
78.494	1.075	85.352	0.889	94.608
ENSMUSG00000028603	Scp2	protein_coding	1.054	31.477	0.987
93.765	0.957	59.726	0.97	85.493
ENSMUSG00000028607	Cpt2	protein_coding	0.862	22.92	1.086	82.904
1.066	85.464	1.023	90.157
ENSMUSG00000028608	0610037L13Rik	protein_coding	0.945	15.76
1.043	96.763	1.013	65.567	1.057	50.452
ENSMUSG00000028609	Magoh	protein_coding	1.136	13.633	0.967
84.858	0.91	34.21	0.982	95.273
ENSMUSG00000028612	4930430J20Rik	antisense	1	67.334	1
94.48	1	76.923	1	80.573
ENSMUSG00000028613	Lrp8	protein_coding	1.009	83.658	1.103

92.253	0.901 59.922	0.861 84.29
ENSMUSG00000028614	Ndc1	protein_coding	1.034 56.085	1.097
93.27 0.884 29.765	0.984 93.264
ENSMUSG00000028617	Lrrc42	protein_coding	1.038 41.951
1.027 97.263	0.936 36.499	0.992 95.35
ENSMUSG00000028618	Tmem59	protein_coding	1.093 1.323 0.968
78.877	0.946 19.765	0.961 92.31
ENSMUSG00000028619	Tceanc2	protein_coding	0.928 7.574 0.966
91.897	1.116 12.831	0.971 87.542
ENSMUSG00000028621	Cyb5rl	protein_coding	1.049 75.671
1.012 83.104	0.941 37.906	1.114 49.806
ENSMUSG00000028622	Mrpl37	protein_coding	0.953 40.568
1.048 94.367	1.004 69.825	1.029 83.067
ENSMUSG00000028626	Col9a2	protein_coding	0.639 3.325 1.019
95.504	1.541 6.506 1.14	92.748
ENSMUSG00000028629	Exo5	protein_coding	1.042 70.36 0.888 69.175
1.08	39.471	1.07	90.125
ENSMUSG00000028630	Dyrk2	protein_coding	0.967 84.738	1.072
83.104	0.965 48.387	0.935 83.724
ENSMUSG00000028631	Kcnq4	protein_coding	1.084 23.493	1.047
71.617	0.881 0.879 1.001 96.184
ENSMUSG00000028633	NA	NA	NA	NA	0.967 85.921	1.021
79.325	1.075 87.134
ENSMUSG00000028634	Hivep3	protein_coding	0.997 73.585
1.068 83.104	0.936 61.288	0.907 84.19
ENSMUSG00000028636	Ppcs	protein_coding	0.908 55.641	1.022
91.584	1.073 83.538	0.995 85.493
ENSMUSG00000028637	Ccdc30	protein_coding	1.206 84.977
0.994 96.138	0.835 87.686	0.833 90.805
ENSMUSG00000028639	Ybx1	protein_coding	1.005 85.832	1.012
88.136	0.981 70.44 1.035 83.067
ENSMUSG00000028640	Tfap2c	protein_coding	0.931 58.802
1.058 83.104	NA	NA	0.893 89.892
ENSMUSG00000028641	Lepre1	protein_coding	0.998 83.823
0.991 89.494	1.012 74.853	1.171 38.2
ENSMUSG00000028642	4930538K18Rik	protein_coding	1.34	21.278
0.762 68.115	0.976 88.076	1.002 84.29
ENSMUSG00000028643	Ccdc23	protein_coding	0.959 51.447
1.005 88.881	1.034 38.26 1.024 91.872
ENSMUSG00000028644	Ermap	protein_coding	1.533 37.414	0.572
70.29 1.141 71.615	0.551 83.724
ENSMUSG00000028645	Slc2a1	protein_coding	0.964 63.126
1.051 96.847	0.987 56.931	0.941 93.132
ENSMUSG00000028646	Rragc	protein_coding	1.212 0	0.999 97.155
0.824 0.72	0.951 88.955
ENSMUSG00000028647	Mycbp	protein_coding	0.974 40.422	0.969
95.708	1.061 58.616	1.04	71.855
ENSMUSG00000028648	Ndufs5	protein_coding	0.78	1.031 1.011
95.735	1.268 6.449 0.948 94.904
ENSMUSG00000028649	Macf1	protein_coding	0.98	55.939	0.99
95.708	1.028 54.163	0.98	91.291
ENSMUSG00000028651	Ppie	protein_coding	0.941 37.248	0.963
71.41 1.1	8.586 0.999 83.276
ENSMUSG00000028653	Trit1	protein_coding	0.816 19.224	0.922

62.692 1.33 5.864 1.029 90.125
ENSMUSG00000028654 Mycl protein_coding 1.045 71.516 0.993
93.094 0.965 84.552 1.075 86.133
ENSMUSG00000028655 Mfsd2a protein_coding 3.78 0.2 0.428
73.461 0.621 18.122 1.121 92.762
ENSMUSG00000028656 Cap1 protein_coding 1.123 0.355 0.991 95.431
0.899 1.705 1.04 78.546
ENSMUSG00000028657 Ppt1 protein_coding 1.167 0.355 NA NA
0.869 0.879 0.974 96.287
ENSMUSG00000028664 Ephb2 protein_coding 1.007 74.745 1.37
66.01 0.723 5.628 1.012 85.786
ENSMUSG00000028668 Tceb3 protein_coding 1.035 37.414 0.989
96.138 0.973 39.528 1.023 49.514
ENSMUSG00000028669 Pithd1 protein_coding 1.045 25.421
0.981 97.155 0.977 50.184 0.948 85.493
ENSMUSG00000028670 Lypla2 protein_coding 0.977 42.034 1.02
82.904 1.004 87.786 1.009 93.264
ENSMUSG00000028671 Gale protein_coding 0.848 15.76 1.009 83.104
1.172 45.667 1.062 81.061
ENSMUSG00000028672 Hmgcl protein_coding 0.993 82.231 0.932
37.426 1.082 15.049 1.006 96.183
ENSMUSG00000028673 Fuca1 protein_coding 0.963 58.077 1.059
83.104 0.983 73.027 1.022 89.498
ENSMUSG00000028675 Pnrc2 protein_coding 1.057 14.966 1.002
81.443 0.942 70.214 1.043 60.31
ENSMUSG00000028676 Srsf10 protein_coding 1.058 36.438
1.022 93.817 0.926 57.819 0.999 92.748
ENSMUSG00000028677 Rnf220 protein_coding 0.897 0.987 1.013
95.735 1.101 5.977 0.99 92.381
ENSMUSG00000028678 Kif2c protein_coding 1.589 35.787 0.686
59.946 NA NA 1.224 71.305
ENSMUSG00000028680 Plk3 protein_coding 0.869 38.983 1.124
82.904 1.021 88.693 0.976 91.783
ENSMUSG00000028681 Ptch2 protein_coding 1.416 40.065 1.702
67.843 0.414 0.72 1.083 85.577
ENSMUSG00000028683 Eif2b3 protein_coding 1.096 14.59 0.936
74.633 0.975 54.25 0.989 90.125
ENSMUSG00000028684 Urod protein_coding 0.923 27.968 1.018
96.897 1.064 61.743 1.008 96.248
ENSMUSG00000028687 Mutyh protein_coding 0.943 61.633 1.102
83.819 0.964 63.075 0.981 87.134
ENSMUSG00000028688 Toe1 protein_coding 0.951 53.407 0.986
87.041 1.063 30.107 1.053 50.74
ENSMUSG00000028689 Ccdc163 protein_coding 0.932 48.354
0.979 75.345 1.098 17.412 0.933 86.991
ENSMUSG00000028690 Mmachc protein_coding 0.892 4.634 1.12
71.617 NA NA 0.944 91.078
ENSMUSG00000028691 Prdx1 protein_coding 0.963 65.106 1.09
93.707 NA NA 1.029 88.755
ENSMUSG00000028692 Akr1a1 protein_coding 0.953 29.725 1.03
83.464 1.019 65.8 1.044 77.331
ENSMUSG00000028693 Nasp protein_coding 1.019 75.399 0.853
69.611 1.154 48.451 1.211 31.133
ENSMUSG00000028696 Ipp protein_coding 0.961 38.286 0.902

56.781	1.153 9.176 0.941 90.691
ENSMUSG00000028698	Pik3r3	protein_coding	1.135 40.065
1.011 96.407	0.873 39.079	0.902 84.29
ENSMUSG00000028699	Tspan1	protein_coding	1.198 19.315
0.768 28.822	1.09 68.129	0.574 86.991
ENSMUSG00000028700	Pomgnt1	protein_coding	0.953 17.839	NA
NA	1.042 35.673	1.034 73.597
ENSMUSG00000028701	Lurap1	protein_coding	0.988 82.543
1.033 97.097	0.982 87.971	0.889 84.29
ENSMUSG00000028702	Rad54l	protein_coding	0.962 84.324
1.024 91.361	1.012 82.161	1.055 82.328
ENSMUSG00000028703	Lrrc41	protein_coding	1.019 41.454
1.073 71.617	0.914 4.389 1.088 79.442
ENSMUSG00000028706	Nsun4 protein_coding	0.929 50.519	1.093
97.049	NA	NA	1.079 53.923
ENSMUSG00000028707	Dmbx1 protein_coding	1	77.497	1
96.216	1	80.015	1	95.877
ENSMUSG00000028708	Mknk1 protein_coding	1.042 13.492	0.976
94.848	0.978 36.786	1.019 94.654
ENSMUSG00000028709	Mob3c protein_coding	0.984 84.263	1.064
82.904	0.957 42.895	0.992 96.042
ENSMUSG00000028710	Atpaf1	protein_coding	1.045 28.098
1.031 83.104	0.925 5.884 1.019 95.877
ENSMUSG00000028712	Cyp4a31	protein_coding	1	85.355	1
96.216	1	80.163	1	96.139
ENSMUSG00000028713	Cyp4b1	protein_coding	0.916 63.736
0.864 90.712	1.259 35.13 1.035 90.463
ENSMUSG00000028716	Pdzk1ip1	protein_coding	0.904 64.284	0.84
87.857	1.32	50.851	0.919 86.991
ENSMUSG00000028717	Tal1	protein_coding	1.101 31.732	0.883
69.611	1.027 85.134	0.947 88.505
ENSMUSG00000028718	Stil	protein_coding	1.709 17.582	0.59
58.277	NA	NA	1.177 76.347
ENSMUSG00000028719	Cmpk1 protein_coding	1.006 83.823	0.979
91.532	1.018 68.129	0.999 94.384
ENSMUSG00000028729	Ebna1bp2	protein_coding	0.877 10.739
1.031 96.335	1.102 20.556	1.076 51.901
ENSMUSG00000028730	Wdr65 protein_coding	1.124 85.637	1.201
82.904	0.743 32.297	0.385 83.724
ENSMUSG00000028737	Aldh4a1	protein_coding	0.867 23.923
1.137 79.367	1.017 86.666	0.819 83.952
ENSMUSG00000028741	Mrto4 protein_coding	0.883 33.745	1.063
96.652	1.069 46.575	1.232 32.698
ENSMUSG00000028743	Akr7a5	protein_coding	0.886 5.491 1.088
77.085	1.037 38.683	0.891 83.724
ENSMUSG00000028744	Pqlc2 protein_coding	0.99	58.264	1.031
92.111	0.98	73.598	0.996 93.964
ENSMUSG00000028745	Capzb protein_coding	1.023 29.596	0.973
87.041	1.004 68.266	1.097 31.133
ENSMUSG00000028749	Pla2g2f	protein_coding	1	86.819	1
90.256	1	82.74 1	92.263
ENSMUSG00000028751	Pla2g2e	protein_coding	0.606 18.138
1.227 82.904	1.35	66.269	1.223 87.134
ENSMUSG00000028753	Vwa5b1	protein_coding	1	83.823	NA

NA 1 77.532 1 93.413
ENSMUSG00000028755 Cda protein_coding 1.345 27.113 1.036
96.216 0.716 22.864 0.885 91.105
ENSMUSG00000028756 Pink1 protein_coding 1.038 66.944 0.886
71.331 1.083 70.534 1.031 93.132
ENSMUSG00000028757 Ddost protein_coding 1.065 36.438 1.017
93.899 0.924 25.688 1.067 48.119
ENSMUSG00000028758 Kif17 protein_coding 1.611 15.178 NA NA
0.659 15.465 0.749 83.724
ENSMUSG00000028759 Hp1bp3 protein_coding 1.008 86.172
0.994 93.654 0.996 73.759 0.971 87.735
ENSMUSG00000028760 Eif4g3 protein_coding 0.95 1.031 1.035
82.904 1.02 35.13 1.042 56.339
ENSMUSG00000028763 Hspg2 protein_coding 0.817 4.468 1.14 72.546
1.074 50.375 1.034 91.525
ENSMUSG00000028766 Alpl protein_coding 0.845 5.084 1.12 91.776
1.059 29.819 1.026 93.132
ENSMUSG00000028771 Ptpn12 protein_coding 1.08 10.595
1.027 82.904 0.905 1.871 1.003 92.12
ENSMUSG00000028772 Zcchc17 protein_coding 0.966 36.945
1.088 80.806 0.953 83.811 1.06 31.133
ENSMUSG00000028773 Fabp3 protein_coding 1.585 35.748 1.149
96.216 0.549 40.338 2.215 90.674
ENSMUSG00000028776 Tinagl1 protein_coding 0.759 1.175 0.946
86.728 1.394 8.647 1.027 86.445
ENSMUSG00000028777 Gnat3 protein_coding 0.658 48.036 1.024
95.211 1.485 22.994 1.271 84.246
ENSMUSG00000028778 Hcrtr1 protein_coding 1.037 80.146
0.637 78.877 1.514 53.134 1.162 96.248
ENSMUSG00000028779 Pef1 protein_coding 0.893 2.174 1.1 81.54
1.018 67.163 0.991 96.248
ENSMUSG00000028780 Sema3c protein_coding 1.155 86.464
1.136 96.847 0.759 86.985 0.756 91.65
ENSMUSG00000028782 Bai2 protein_coding 0.813 46.413 0.991
95.504 1.244 38.099 1.148 93.264
ENSMUSG00000028784 Spocd1 protein_coding 1 84.564 1
96.984 1 46.32 1 86.699
ENSMUSG00000028785 Hpca protein_coding 1.429 5.559 1.291 82.904
0.541 8.634 1.417 0
ENSMUSG00000028786 NA NA NA NA 0.915 70.29 1.203 41.235
0.901 86.991
ENSMUSG00000028788 Ptp4a2 protein_coding 1.169 0 0.956
87.553 0.893 0.879 1.054 65.845
ENSMUSG00000028789 Adc protein_coding 0.871 8.122 1.039 93.574
1.101 35.673 1.092 78.042
ENSMUSG00000028790 Khdrbs1 protein_coding 1.155 0.2 0.984
91.532 0.877 1.898 1.017 82.66
ENSMUSG00000028792 Ak2 protein_coding 0.881 0.987 1.033 79.367
1.1 17.751 0.991 92.903
ENSMUSG00000028793 Rnf19b protein_coding 1.136 12.396
1.021 93.574 0.861 13.848 1.055 94.384
ENSMUSG00000028794 A3galt2 protein_coding 0.722 5.204 0.957
90.053 1.442 19.106 0.977 96.248
ENSMUSG00000028795 Ccdc28b protein_coding 0.971 70.862

0.925 93.654 1.11 64.305 1.072 93.627
ENSMUSG00000028796 Phc2 protein_coding 0.944 7.71 0.952 87.322
1.115 15.185 0.978 86.991
ENSMUSG00000028797 Tmem234 protein_coding 0.972 58.077
1.009 97.155 1.017 73.428 0.998 94.654
ENSMUSG00000028798 Eif3i protein_coding 0.981 82.231 0.968
68.818 1.052 38.26 1.085 31.133
ENSMUSG00000028799 Zfp362 protein_coding 0.902 16.325
1.032 90.29 1.077 38.26 1.011 89.892
ENSMUSG00000028800 Hdac1 protein_coding 1.011 81.657 1.019
88.823 0.966 48.577 1.031 40.426
ENSMUSG00000028801 Stpg1 protein_coding 0.827 59.028 1.082
95.431 1.116 76.498 0.821 84.29
ENSMUSG00000028803 Nipal3 protein_coding 0.966 45.894
0.956 78.877 1.083 20.315 0.892 85.493
ENSMUSG00000028807 Zbtb8a protein_coding 1.018 85.77 1.159
91.456 0.845 36.499 1.239 86.445
ENSMUSG00000028809 Srrm1 protein_coding 0.995 36.259 0.984
74.633 1.024 11.518 0.951 83.724
ENSMUSG00000028811 Yars protein_coding 0.873 9.599 1.06 83.104
1.08 28.979 1.137 31.133
ENSMUSG00000028813 CK137956 protein_coding 1 81.465 1
93.899 1 76.498 1 91.011
ENSMUSG00000028820 Sfpq protein_coding 1.147 11.928 1.125
91.643 0.775 13.848 1.081 60.968
ENSMUSG00000028821 Syf2 protein_coding 0.922 15.227 NA NA
1.099 28.979 1.031 96.071
ENSMUSG00000028822 Tmem50a protein_coding 1.098 2.361 0.908
50.715 1 57.819 0.987 95.832
ENSMUSG00000028826 Tmem57 protein_coding 0.916 39.953
0.974 91.361 1.121 33.508 1.015 89.152
ENSMUSG00000028830 AU040320 protein_coding 0.959 14.221
0.983 97.155 1.058 26.663 0.948 83.724
ENSMUSG00000028832 Stmn1 protein_coding 1.061 74.147 0.819
60.678 1.153 70.328 1.144 77.331
ENSMUSG00000028833 Ncdn protein_coding 1.006 86.57 1.103 77.618
0.902 16.051 1.031 92.018
ENSMUSG00000028834 Trim63 protein_coding 14.543 6.853
0.837 97.155 0.082 1.58 0.564 94.654
ENSMUSG00000028836 Slc30a2 protein_coding 1.495 4.916 0.953
96.68 0.703 1.764 1.061 92.561
ENSMUSG00000028837 Psmb2 protein_coding 0.952 57.014 1.057
79.864 0.992 84.071 0.992 94.297
ENSMUSG00000028838 Extl1 protein_coding 1.046 77.182 0.927
84.594 1.033 74.686 1.039 94.904
ENSMUSG00000028840 Zfp593 protein_coding 0.931 76.686 1.06
97.288 1.012 79.625 1.062 83.067
ENSMUSG00000028841 Cnksr1 protein_coding 0.784 3.848 1.14
80.775 1.115 36.388 0.95 95.593
ENSMUSG00000028842 Ago3 protein_coding 0.96 35.894 NA NA
1 37.764 1.034 64.513
ENSMUSG00000028843 Sh3bgrl3 protein_coding 1.279 0.461 0.927
94.102 0.846 7.742 1.172 38.624
ENSMUSG00000028845 Tekt2 protein_coding 0.78 6.822 1.185 71.98

1.085 73.759 1.097 76.831
ENSMUSG00000028847 Trappc3 protein_coding 0.933 1.15 0.994
92.111 1.078 38.099 1.038 73.597
ENSMUSG00000028848 Gpn2 protein_coding 0.93 18.907 1.062
90.116 1.013 58.051 0.998 90.397
ENSMUSG00000028849 Map7d1 protein_coding 0.984 58.264 1.11
71.222 0.917 41.154 0.959 93.722
ENSMUSG00000028850 Gpatch3 protein_coding 0.852 13.952
0.939 86.728 1.245 12.831 1.058 94.904
ENSMUSG00000028851 Nudc protein_coding 0.93 1.031 0.984 82.947
1.094 7.365 1.08 50.74
ENSMUSG00000028854 Slc9a1 protein_coding 1.061 35.024
0.966 87.313 0.975 59.726 0.923 77.331
ENSMUSG00000028857 Tmem222 protein_coding 0.924 27.202 1
96.138 1.085 32.776 0.991 89.827
ENSMUSG00000028859 Csf3r protein_coding 1.521 3.825 1.169 96.216
0.561 9.288 0.906 92.181
ENSMUSG00000028860 Sytl1 protein_coding 0.874 10.739 0.922
79.261 1.242 9.567 0.841 87.182
ENSMUSG00000028861 Mrps15 protein_coding 0.799 1.15 1.121
80.14 1.113 15.241 0.987 83.067
ENSMUSG00000028862 Map3k6 protein_coding 0.768 9.841 0.913
78.471 1.425 5.977 1.087 64.513
ENSMUSG00000028863 Meaf6 protein_coding 0.969 37.248 0.975
73.166 1.059 23.604 0.962 87.182
ENSMUSG00000028864 Hgf protein_coding 1.177 14.008 1.075
89.996 0.79 3.147 0.961 86.991
ENSMUSG00000028865 Cd164l2 protein_coding 0.843 13.199 0.81
62.71 1.466 8.339 0.81 89.587
ENSMUSG00000028868 Wasf2 protein_coding 1.058 32.873 0.968
94.848 0.977 55.9 0.951 88.136
ENSMUSG00000028869 Gnl2 protein_coding 1.003 86.006 0.943
74.633 1.061 38.038 1.024 64.513
ENSMUSG00000028871 Rspo1 protein_coding 1.237 37.091 1.41
85.756 0.571 16.409 0.804 87.496
ENSMUSG00000028873 Cdca8 protein_coding 0.946 81.178 0.923
62.692 1.142 66.043 1.546 68.354
ENSMUSG00000028874 Fgr protein_coding 1.247 4.052 0.927 91.361
0.864 10.726 1.128 72.826
ENSMUSG00000028878 Fam76a protein_coding 1.025 73.401
0.968 89.996 1.006 87.898 0.993 93.117
ENSMUSG00000028879 Stx12 protein_coding 1.004 81.647 0.999
95.211 0.998 86.346 1.016 59.464
ENSMUSG00000028882 Ppp1r8 protein_coding 0.989 85.283
1.014 96.843 0.995 85.58 1.08 50.74
ENSMUSG00000028883 Sema3a protein_coding 1.169 29.536
1.337 83.104 0.64 17.898 1.024 92.018
ENSMUSG00000028884 Rpa2 protein_coding 0.988 86.819 0.987
97.155 1.022 88.729 1.229 32.698
ENSMUSG00000028885 Smpdl3b protein_coding 1.169 31.732
0.946 80.463 0.902 79.776 1.13 53.923
ENSMUSG00000028886 Eya3 protein_coding 1.066 19.132 0.972
90.053 0.965 63.504 NA NA
ENSMUSG00000028889 Yrdc protein_coding 0.944 42.922 1.006

96.843 1.048 69.825 0.965 95.74
ENSMUSG00000028890 Mtf1 protein_coding 0.99 83.333 1.087
80.806 0.927 27.86 0.997 95.74
ENSMUSG00000028893 Sesn2 protein_coding 1.173 25.026 0.981
85.671 0.866 46.956 1.086 66.468
ENSMUSG00000028894 Inpp5b protein_coding 1.045 61.725
0.995 83.104 0.963 18.797 1.013 93.457
ENSMUSG00000028896 Rcc1 protein_coding 1.048 75.399 NA NA
0.956 87.336 1.069 40.729
ENSMUSG00000028898 Trnau1ap protein_coding 1.058 26.77 0.933
75.345 1.013 88.161 0.997 95.273
ENSMUSG00000028899 Taf12 protein_coding 1.004 82.352 1.1
93.574 0.904 39.528 1.028 83.067
ENSMUSG00000028901 Gmeb1 protein_coding 1.037 60.853 0.981
89.713 0.983 86.346 1.036 78.948
ENSMUSG00000028902 Sf3a3 protein_coding 1.042 43.939 NA NA
0.958 56.625 1.059 70.85
ENSMUSG00000028906 Epb4.1 protein_coding 0.934 73.487
0.936 85.671 1.147 50.375 1.078 94.904
ENSMUSG00000028907 Utp11l protein_coding 1.04 56.085 0.95
67.739 1.013 42.199 1.04 64.513
ENSMUSG00000028909 Ptpru protein_coding 0.905 68.955 1.022
95.504 1.083 70.623 0.976 93.964
ENSMUSG00000028910 Mecr protein_coding 0.985 67.163 0.995
93.27 1.02 88.461 1.242 87.134
ENSMUSG00000028911 Srsf4 protein_coding 0.923 7.881 1.016 90.29
1.062 47.935 0.989 95.74
ENSMUSG00000028914 Casp9 protein_coding 1.09 18.556 0.996
92.561 0.918 11.314 0.932 83.724
ENSMUSG00000028917 Plekhm2 protein_coding 1.006 75.399
0.942 71.41 1.057 45.94 0.98 85.493
ENSMUSG00000028919 Arhgef19 protein_coding 0.82 11.707
0.973 96.216 1.254 20.315 0.907 91.291
ENSMUSG00000028920 Fbxo42 protein_coding 0.985 71.217
1.059 72.546 0.959 7.339 0.964 83.724
ENSMUSG00000028923 Necap2 protein_coding 0.988 74.147
1.065 92.561 0.953 39.755 1.049 61.984
ENSMUSG00000028926 Cdk14 protein_coding 1.054 11.044 1.143
83.104 0.829 9.131 1.028 88.245
ENSMUSG00000028927 Padi2 protein_coding 1.179 1.441 0.834 68.818
1.014 65.902 1.3 44.882
ENSMUSG00000028931 Kcnab2 protein_coding 1.749 0 0.895
75.052 0.638 2.563 1.519 40.894
ENSMUSG00000028932 Psmc2 protein_coding 0.986 84.534 0.996
91.83 1.016 70.328 1.122 60.968
ENSMUSG00000028933 Xrcc2 protein_coding 1.265 9.259 1.121 85.756
0.706 8.634 1.023 76.697
ENSMUSG00000028936 Rpl22 protein_coding 1.06 8.281 0.925 20.174
1.018 40.575 1.01 88.797
ENSMUSG00000028937 Acot7 protein_coding 0.82 5.967 1.107 91.643
1.101 25.455 1.213 52.804
ENSMUSG00000028938 Galntl5 protein_coding 1 82.705 1
96.308 1 85.792 1 93.627
ENSMUSG00000028940 Hes2 protein_coding 3.059 0.664 0.497 51.159

0.656 42.621 0.99 88.245
ENSMUSG00000028943 Espn protein_coding 1.127 35.024 0.849
75.345 1.047 88.657 0.788 85.493
ENSMUSG00000028944 Prkag2 protein_coding 1.141 21.836
1.029 92.253 0.852 5.087 1.263 71.653
ENSMUSG00000028945 Rheb protein_coding 1.014 74.958 1.066
71.617 0.922 9.901 1.014 60.466
ENSMUSG00000028948 Nol9 protein_coding 1.104 12.855 0.936
96.013 0.968 26.057 0.992 93.734
ENSMUSG00000028949 Smarcd3 protein_coding 0.942 34.572
1.075 82.904 0.988 83.066 1.002 83.276
ENSMUSG00000028950 Tas1r1 protein_coding 0.95 70.862
1.096 88.136 0.958 86.727 0.628 83.724
ENSMUSG00000028952 Zbtb48 protein_coding 0.938 48.146 1.09
87.185 0.98 83.925 0.962 96.183
ENSMUSG00000028953 Abcf2 protein_coding 0.907 24.748 1.059
72.642 1.042 68.643 1.154 32.045
ENSMUSG00000028954 Nub1 protein_coding 0.993 79.577 0.953
65.819 1.054 31.761 1.037 87.447
ENSMUSG00000028955 Vamp3 protein_coding 1.044 30.282 NA NA
0.943 43.842 1.082 46.412
ENSMUSG00000028957 Per3 protein_coding 0.9 68.846 1.015
93.529 1.091 83.158 1.138 71.855
ENSMUSG00000028958 Tmub1 protein_coding 0.918 20.369 1.083
87.029 1.002 67.163 0.95 84.29
ENSMUSG00000028959 Fastk protein_coding 0.916 13.45 1.052 72.546
1.039 88.161 1.025 93.964
ENSMUSG00000028960 Ube4b protein_coding 1.038 23.254 0.988
91.361 0.976 40.19 0.969 83.724
ENSMUSG00000028961 Pgd protein_coding 0.655 0.987 1.008 87.037
1.51 27.732 1.196 74.047
ENSMUSG00000028962 Slc4a2 protein_coding 0.934 10.143
1.078 71.617 0.995 79.625 0.992 96.183
ENSMUSG00000028964 Park7 protein_coding 0.851 0.987 1.042 83.104
1.129 9.429 1.017 72.992
ENSMUSG00000028965 Tnfrsf9 protein_coding 1.058 44.694
0.821 72.399 1.154 84.504 1.293 93.156
ENSMUSG00000028967 Errfi1 protein_coding 1.051 58.38 0.938
91.897 1.016 55.701 1.001 93.734
ENSMUSG00000028969 Cdk5 protein_coding 0.925 5.946 1.049 88.823
1.031 31.4 1.067 40.127
ENSMUSG00000028970 Abcb1b protein_coding 1.128 52.799
0.971 89.277 0.909 69.134 0.999 93.045
ENSMUSG00000028972 Car6 protein_coding 0.839 67.334 0.453
82.503 2.635 30.213 3.439 66.068
ENSMUSG00000028973 Abcb8 protein_coding 0.911 6.221 1.032 83.104
1.066 49.058 1.038 92.691
ENSMUSG00000028974 Dffa protein_coding 0.971 48.939 0.934
83.58 1.099 18.411 1.077 64.513
ENSMUSG00000028975 Pex14 protein_coding 0.87 2.644 1.088 83.104
1.054 48.935 0.994 91.269
ENSMUSG00000028976 Slc2a5 protein_coding 0.095 0.987 1.504
85.756 7.028 6.449 0.772 93.457
ENSMUSG00000028977 Casz1 protein_coding 0.82 1.15 1.121 87.282

1.088 16.808 0.903 84.29
ENSMUSG00000028978 Nos3 protein_coding 0.851 3.101 1.11 71.222
1.053 70.1 0.961 94.174
ENSMUSG00000028979 Masp2 protein_coding 0.759 3.325 0.991 96.216
1.328 6.693 0.82 87.496
ENSMUSG00000028980 H6pd protein_coding 0.932 57.544 0.94
94.597 1.139 51.851 1.046 82.85
ENSMUSG00000028982 Slc25a33 protein_coding 0.899 83.333
0.924 74.483 1.205 43.481 1.058 95.92
ENSMUSG00000028986 Klhl7 protein_coding 1 84.049 0.943
83.392 1.058 33.802 1.051 74.193
ENSMUSG00000028988 Ctnnbip1 protein_coding 0.977 44.467
1.003 87.609 1.017 71.232 0.949 86.991
ENSMUSG00000028989 Angptl7 protein_coding 0.581 11.769
0.778 87.553 2.204 10.523 1.39 86.301
ENSMUSG00000028990 Lzic protein_coding 1.032 63.534 NA NA
0.982 79.524 0.99 92.389
ENSMUSG00000028991 Mtor protein_coding 0.941 12.181 1
95.708 1.061 16.446 1 92.748
ENSMUSG00000028992 Nmnat1 protein_coding 1.002 76.319
1.008 88.691 0.987 74.082 0.893 84.29
ENSMUSG00000028995 Fam126a protein_coding 1.199 0 NA NA
0.825 0.72 1.06 79.713
ENSMUSG00000028996 Rbp7 protein_coding 0.545 0.987 0.874 92.474
2.1 5.864 0.921 87.447
ENSMUSG00000028998 Tomm7 protein_coding 0.889 27 0.96 90.378
1.168 27.047 0.984 93.132
ENSMUSG00000028999 Rint1 protein_coding 0.966 58.077 1.105
61.032 0.933 24.918 1.097 49.806
ENSMUSG00000029001 Fbxo44 protein_coding 1.066 81.178
1.001 90.053 0.939 83.925 0.781 83.724
ENSMUSG00000029003 Mad2l2 protein_coding 0.888 28.514
1.021 95.941 1.104 34.727 1.012 94.654
ENSMUSG00000029004 Kmt2e protein_coding 0.985 86.112 1.121
72.642 0.908 13.643 0.958 87.651
ENSMUSG00000029005 Draxin protein_coding 1.075 85.391 0.88
94.597 1.058 67.823 0.921 94.19
ENSMUSG00000029007 Agtrap protein_coding 1.221 0 0.796
13.578 1.025 73.096 1.003 95.92
ENSMUSG00000029009 Mthfr protein_coding 1.085 35.024 0.974
92.137 0.944 49.733 1.053 51.967
ENSMUSG00000029012 Orc5 protein_coding 1.072 15.602 0.974
91.361 0.953 48.851 1.013 94.904
ENSMUSG00000029014 Dnajc2 protein_coding 1.174 9.983 0.889
54.06 0.958 77.037 1.018 57.711
ENSMUSG00000029016 Clcn6 protein_coding 0.891 28.242 1.047
80.806 1.075 72.148 1.041 95.273
ENSMUSG00000029017 Pmpcb protein_coding 0.938 41.33 0.967 94.848
1.104 37.764 1.031 94.825
ENSMUSG00000029019 Nppb protein_coding 1 85.129 1
93.654 1 80.163 1 95.273
ENSMUSG00000029020 Mfn2 protein_coding 0.932 11.384 1.068
83.104 1.004 72.695 1.039 78.379
ENSMUSG00000029022 Miip protein_coding 0.894 1.031 1.025 96.335

1.092 6.506 1.064 59.394
ENSMUSG00000029026 Trp73 protein_coding 0.609 7.916 1.119 82.904
1.463 45.667 0.667 85.493
ENSMUSG00000029027 Dffb protein_coding 0.904 24 0.94 93.654
1.179 29.302 1.109 82.85
ENSMUSG00000029028 Lrrc47 protein_coding 0.89 3.231 0.982
88.705 1.145 7.855 0.999 93.734
ENSMUSG00000029029 Wrap73 protein_coding 0.987 70.862
1.046 79.055 0.97 43.426 1.022 96.192
ENSMUSG00000029030 Tprgl protein_coding 0.942 32.749 1.021
93.899 1.036 55.109 0.946 83.724
ENSMUSG00000029032 Arhgef16 protein_coding 0.888 67.334 0.9
67.513 1.248 28.603 1.069 93.612
ENSMUSG00000029033 Acap3 protein_coding 1.153 3.644 1.007 94.89
0.863 1.705 0.984 85.577
ENSMUSG00000029034 Cpsf3l protein_coding 1.045 40.333
0.987 91.661 0.968 77.294 1.011 79.545
ENSMUSG00000029036 Atad3a protein_coding 0.981 49.944
1.001 96.407 1.018 72.853 0.983 85.493
ENSMUSG00000029038 Ssu72 protein_coding 1.013 62.354 NA NA
0.99 72.464 1.066 56.863
ENSMUSG00000029044 4930584F24Rik protein_coding 1 84.324
1 96.013 1 88.601 1 93.117
ENSMUSG00000029047 Pex10 protein_coding 1.002 48.146 1.055
88.092 NA NA 0.954 94.904
ENSMUSG00000029048 Rer1 protein_coding 0.987 85.391 0.981
87.919 1.029 81.032 1.075 31.133
ENSMUSG00000029049 Morn1 protein_coding 0.849 19.365 1.049
93.765 1.119 10.76 0.974 95.405
ENSMUSG00000029050 Ski protein_coding 1.057 29.057 1.056
79.055 0.892 1.342 0.98 91.625
ENSMUSG00000029053 Prkcz protein_coding 0.983 71.06 0.877 62.322
1.164 51.977 0.844 83.724
ENSMUSG00000029054 Gabrd protein_coding 1.192 83.968 1.364
80.678 0.616 9.347 0.706 85.493
ENSMUSG00000029055 Plch2 protein_coding 0.685 7.004 1.027 96.378
1.42 10.819 1.131 94.82
ENSMUSG00000029056 Pank4 protein_coding 0.991 55.641 1.005
82.904 0.998 84.434 1 96.248
ENSMUSG00000029059 Fam213b protein_coding 0.829 0.987 1.045
83.819 1.151 14.376 1.009 86.209
ENSMUSG00000029060 Mib2 protein_coding 0.912 18.379 NA NA
1.067 27.533 0.986 84.29
ENSMUSG00000029061 Mmp23 protein_coding 1.082 10.595 0.975
96.843 0.944 47.12 1.196 0
ENSMUSG00000029062 Cdk11b protein_coding 0.886 0.987 1.018
93.146 1.108 5.864 1.082 40.894
ENSMUSG00000029063 Nadk protein_coding 0.91 27.836 NA NA
1.091 63.801 1.011 95.74
ENSMUSG00000029064 Gnb1 protein_coding 1.003 81.178 1.04
60.309 0.96 3.123 0.979 96.287
ENSMUSG00000029066 Mrpl20 protein_coding 0.976 38.555 NA
NA 1.072 52.8 0.985 95.273
ENSMUSG00000029068 Ccnl2 protein_coding 0.923 19.595 1.003

90.378 1.081 7.365 0.94 83.724
ENSMUSG00000029070 Mxra8 protein_coding 1.307 0.461 0.948 87.452
0.804 6.381 1.047 95.273
ENSMUSG00000029071 Dvl1 protein_coding 0.962 37.531 0.995
87.609 1.046 66.043 0.99 92.181
ENSMUSG00000029072 Tas1r3 protein_coding 0.856 52.9 NA NA
1.025 68.129 0.766 83.724
ENSMUSG00000029073 Gltpd1 protein_coding 1.109 11.044
1.032 79.367 0.869 6.007 1.041 95.92
ENSMUSG00000029074 Ttll10 protein_coding 0.606 1.274 0.845
72.712 1.955 5.864 0.91 85.493
ENSMUSG00000029075 Tnfrsf4 protein_coding 0.718 27.836
1.024 95.708 1.352 27.533 1.102 69.708
ENSMUSG00000029076 Sdf4 protein_coding 1.014 85.391 0.96
90.378 1.023 72.853 0.994 90.289
ENSMUSG00000029082 Bst1 protein_coding 1.797 0 0.809 79.475
0.685 13.848 1.519 33.269
ENSMUSG00000029084 Cd38 protein_coding 1.224 17.582 0.946
94.597 0.865 39.079 1.094 57.075
ENSMUSG00000029086 Prom1 protein_coding 1.593 0 1.029 96.843
0.607 0.72 0.893 84.29
ENSMUSG00000029088 Kcnip4 protein_coding 0.939 85.129
1.268 96.138 0.835 87.971 1.369 84.31
ENSMUSG00000029089 Pacrgl protein_coding 0.988 86.112
0.983 95.301 1.025 85.792 1.036 95.273
ENSMUSG00000029090 Gpr125 protein_coding 0.956 39.612
1.029 84.007 1.015 83.719 0.939 83.724
ENSMUSG00000029092 4930435H24Rik protein_coding 1 86.333
1 96.763 NA NA 1 96.287
ENSMUSG00000029093 Sorcs2 protein_coding 0.447 0.987 1.388
83.104 1.617 5.864 0.983 95.74
ENSMUSG00000029094 Afap1 protein_coding 1.046 71.516 1.094
79.411 0.871 22.009 0.984 90.628
ENSMUSG00000029095 Ablim2 protein_coding 1.061 40.88 1.152
71.617 0.819 2.563 1.069 84.499
ENSMUSG00000029096 Htra3 protein_coding 1.001 84.977 0.948
94.848 1.049 69.728 1.057 86.118
ENSMUSG00000029097 Trmt44 protein_coding 1.058 63.804
1.106 90.29 0.856 41.928 0.943 92.31
ENSMUSG00000029098 Acox3 protein_coding 1.076 17.191 0.981
96.652 0.951 8.027 0.934 83.724
ENSMUSG00000029101 Rgs12 protein_coding 0.93 18.138 0.913
37.426 1.176 9.502 1.08 93.734
ENSMUSG00000029102 Hgfac protein_coding 1.357 63.534 0.68
67.941 1.081 54.163 0.757 83.724
ENSMUSG00000029103 Lrpap1 protein_coding 0.923 14.086
0.955 94.48 1.134 19.609 1.039 82.091
ENSMUSG00000029104 Htt protein_coding 0.968 51.056 0.974
82.947 1.056 38.683 1.086 49.514
ENSMUSG00000029106 Add1 protein_coding 1.005 85.355 0.994
97.049 0.995 87.744 1.035 52.982
ENSMUSG00000029108 Pcdh7 protein_coding 0.924 50.768 1.107
71.617 0.976 52.213 1.119 46.846
ENSMUSG00000029110 Rnf4 protein_coding 1.022 65.537 0.941

73.766 1.041 45.94 1.037 66.468
ENSMUSG00000029111 Nelfa protein_coding 1.014 47.174 1.096
79.055 0.896 2.087 1.05 96.139
ENSMUSG00000029112 Nkx1-1 protein_coding 1 81.125 1
95.504 1 88.729 1 95.832
ENSMUSG00000029119 Man2b2 protein_coding 1.071 8.477 0.881
63.912 1.054 88.076 0.996 93.734
ENSMUSG00000029120 Ppp2r2c protein_coding 0.771 84.049
1.468 82.904 0.88 49.967 1.179 93.612
ENSMUSG00000029121 Crmp1 protein_coding 2.368 13.105 0.744
95.708 0.569 18.965 0.462 83.724
ENSMUSG00000029122 Evc protein_coding 1.248 0.984 0.976 83.58
0.818 12.907 1.012 96.086
ENSMUSG00000029123 Stk32b protein_coding 1.207 45.61 1.04
95.301 0.795 69.134 1.076 91.928
ENSMUSG00000029125 Stx18 protein_coding 0.906 3.325 0.992 90.046
1.113 6.693 1.048 72.116
ENSMUSG00000029126 Nsg1 protein_coding 1.153 27.113 0.913
72.399 0.951 86.144 0.974 93.964
ENSMUSG00000029127 Zbtb49 protein_coding 1.009 73.585
0.902 83.58 1.102 36.95 0.992 95.74
ENSMUSG00000029128 Rab28 protein_coding 1.042 33.854 1.017
92.253 0.945 11.751 1.015 83.067
ENSMUSG00000029130 Rnf32 protein_coding 0.776 48.646 1.171
93.27 1.102 53.134 0.729 91.625
ENSMUSG00000029131 Dnajb6 protein_coding 0.973 60.339
0.915 79.475 1.125 11.518 1.101 31.133
ENSMUSG00000029134 Plb1 protein_coding 0.996 84.397 1.043
92.793 0.963 77.037 0.935 85.493
ENSMUSG00000029135 Fosl2 protein_coding 1.033 82.352 0.948
95.708 1.02 88.657 0.937 94.904
ENSMUSG00000029136 Rbks protein_coding 0.916 44.562 NA NA
1.129 54.673 1.074 95.273
ENSMUSG00000029141 Slc4a1ap protein_coding 0.993 58.077 1.01
97.155 0.995 79.554 1.014 80.497
ENSMUSG00000029142 Mrpl33 protein_coding 0.98 66.129 NA
NA 1.008 77.888 1.053 54.153
ENSMUSG00000029144 Gtf3c2 protein_coding 0.922 0.987 1.033
90.29 1.049 6.506 0.988 88.505
ENSMUSG00000029145 Eif2b4 protein_coding 0.889 7.71 1.031
96.68 1.091 10.281 1.039 86.133
ENSMUSG00000029146 Snx17 protein_coding 0.969 78.744 0.981
71.331 1.052 38.975 1.062 78.042
ENSMUSG00000029147 Ppm1g protein_coding 0.951 32.168 0.995
89.509 1.058 27.89 1.091 44.679
ENSMUSG00000029148 Nrbp1 protein_coding 0.947 5.778 1.027 79.411
1.023 52.8 0.995 96.192
ENSMUSG00000029149 Krtcap3 protein_coding 0.856 33.745
0.916 69.611 1.276 13.259 0.987 93.316
ENSMUSG00000029151 Slc30a3 protein_coding 1 61.334 1
95.211 1 88.461 1 83.276
ENSMUSG00000029152 Ociad1 protein_coding 0.931 17.664
0.977 85.793 1.098 15.185 1.058 57.1
ENSMUSG00000029153 Ociad2 protein_coding 1.385 1.188 0.884

65.819	0.819	29.217	0.709	84.29
ENSMUSG00000029154	Cwh43	protein_coding	2.121	16.249	0.56
91.532	0.843	39.079	0.576	85.493
ENSMUSG00000029156	Sgcb	protein_coding	1.116	46.097	1.065
79.367	0.843	14.262	1.026	69.708
ENSMUSG00000029158	Yipf7	protein_coding	8.895	1.188	1.594	96.763
0.07	0.879	0.748	95.832
ENSMUSG00000029161	Cgref1	protein_coding	0.924	49.749	NA
NA	1.037	61.418	0.939	83.276
ENSMUSG00000029162	Khk	protein_coding	1.068	56.311	0.997
90.378	0.939	87.336	0.887	84.29
ENSMUSG00000029163	Emilin1	protein_coding	1.141	7.179	1.098
89.494	0.8	6.858	1.437	42.736
ENSMUSG00000029165	Agbl5	protein_coding	0.951	38.286	1.026
97.155	1.028	54.673	0.923	83.724
ENSMUSG00000029166	Mapre3	protein_coding	0.923	24	0.982
96.652	1.102	43.129	1.041	83.067
ENSMUSG00000029167	Ppargc1a	protein_coding	1.465	28.395
0.833	90.053	0.816	54.521	0.792	84.29
ENSMUSG00000029168	Dpysl5	protein_coding	0.724	15.06	0.803
81.731	1.71	17.998	0.667	86.699
ENSMUSG00000029169	Dhx15	protein_coding	1.159	21.146	0.921
79.475	0.94	41.662	1.033	59.464
ENSMUSG00000029171	Pgm1	protein_coding	1.14	23.82	0.951	64.123
0.92	74.179	1.05	67.691
ENSMUSG00000029173	Sepsecs	protein_coding	0.943	65.622
0.951	86.071	1.118	21.462	1.041	76.697
ENSMUSG00000029174	Tbc1d1	protein_coding	1.075	24.253
0.948	74.483	0.978	75.353	1.035	79.545
ENSMUSG00000029175	Slc35f6	protein_coding	1.122	6.23	0.901
52.266	0.985	68.266	0.98	95.273
ENSMUSG00000029176	Anapc4	protein_coding	1.019	85.499
0.905	38.923	1.08	8.586	1.004	78.546
ENSMUSG00000029177	Cenpa	protein_coding	1.473	50.111	0.799
56.027	0.849	87.744	0.945	94.904
ENSMUSG00000029178	Klf3	protein_coding	0.969	38.983	1.086
75.612	0.951	51.055	1.021	83.067
ENSMUSG00000029179	Zcchc4	protein_coding	1.024	85.283
0.899	82.848	1.086	41.6	0.958	85.493
ENSMUSG00000029182	1700001C02Rik	protein_coding	1	72.743
1	92.111	1	79.255	1	88.657
ENSMUSG00000029184	Olfr109	protein_coding	1	74.299	NA
NA	1	85.134	1	96.248
ENSMUSG00000029185	Fam114a1	protein_coding	1.149	29.057	NA
NA	0.889	11.902	1.14	44.882
ENSMUSG00000029186	Pi4k2b	protein_coding	1.011	78.744
0.863	73.166	1.145	23.41	0.931	86.699
ENSMUSG00000029188	Slc34a2	protein_coding	0.657	35.894
1.122	90.389	1.359	60.789	1.13	92.31
ENSMUSG00000029189	Sel1l3	protein_coding	1.793	3.061	0.954
79.894	0.585	9.456	0.691	94.608
ENSMUSG00000029190	D5Ertd579e	protein_coding	0.907	23.13	1.132
82.904	0.969	84.071	1.023	95.877
ENSMUSG00000029191	Rfc1	protein_coding	1.11	7.959	0.988	95.708

0.912 11.116 0.965 90.289
ENSMUSG00000029192 Tbc1d14 protein_coding 1.253 0.2 0.958
87.041 0.83 11.314 0.988 87.886
ENSMUSG00000029195 Klb protein_coding 0.876 86.464 1.29
93.707 0.887 46.32 0.676 83.724
ENSMUSG00000029196 Tada2b protein_coding 1.115 0.435 1.06
83.104 0.843 0.72 0.961 85.493
ENSMUSG00000029198 Grpel1 protein_coding 0.931 8.181 0.96
97.263 1.117 24.985 1.034 92.762
ENSMUSG00000029199 Lias protein_coding 0.95 29.21 0.935 71.41 1.12
12.16 1.076 71.855
ENSMUSG00000029201 Ugdh protein_coding 1.056 13.557 1.087
82.904 0.868 9.563 1.097 61.753
ENSMUSG00000029202 Pds5a protein_coding 0.967 36.034 0.988
87.322 1.048 26.663 0.973 96.248
ENSMUSG00000029203 Ube2k protein_coding 0.898 8.139 1.014 90.138
1.097 5.964 1.049 74.047
ENSMUSG00000029204 Rhoh protein_coding 1.078 24.689 1.084
92.561 0.851 25.345 1.051 83.067
ENSMUSG00000029205 Chrna9 protein_coding 1 79.709 1
96.407 NA NA 1 94.904
ENSMUSG00000029206 Nsun7 protein_coding 1.031 72.655 1.138
97.049 0.852 68.266 0.99 93.584
ENSMUSG00000029207 Apbb2 protein_coding 0.967 42.034 0.972
90.712 1.065 40.052 1.039 96.287
ENSMUSG00000029208 Guf1 protein_coding 0.969 79.93 0.977 73.094
1.056 14.597 1.007 89.498
ENSMUSG00000029209 Gnpda2 protein_coding 1.198 0.664 0.918
61.883 0.907 22.279 0.97 84.29
ENSMUSG00000029213 Commd8 protein_coding 1.114 4.825 0.893
70.971 1.006 67.962 1.07 78.379
ENSMUSG00000029217 Tec protein_coding 1.397 0 0.87 64.123
0.821 2.563 1.059 96.358
ENSMUSG00000029221 Slc30a9 protein_coding 0.963 29.454
1.006 91.643 1.031 60.5 1.021 71.653
ENSMUSG00000029223 Uchl1 protein_coding 0.97 82.231 0.97
83.58 1.06 52.432 0.969 95.273
ENSMUSG00000029227 Fip1l1 protein_coding 0.974 35.449
0.959 90.287 1.064 44.787 1.024 94.215
ENSMUSG00000029228 Lnx1 protein_coding 0.949 33.906 0.938
68.818 1.125 10.819 0.915 83.724
ENSMUSG00000029229 Chic2 protein_coding 0.925 59.028 1.033
93.469 1.049 41.806 0.965 84.29
ENSMUSG00000029231 Pdgfra protein_coding 1.195 7.842 0.917
75.052 0.91 32.963 1.231 57.1
ENSMUSG00000029233 Srd5a3 protein_coding 1.157 0 1.058
83.104 0.817 0.72 1.036 91.807
ENSMUSG00000029234 Tmem165 protein_coding 1.143 1.079 0.986
95.301 0.888 3.943 1.053 58.333
ENSMUSG00000029235 Pdcl2 protein_coding 1 79.93 1 96.216
1 87.274 1 96.086
ENSMUSG00000029238 Clock protein_coding 1.067 56.311 0.981
90.712 0.956 87.173 0.884 86.991
ENSMUSG00000029245 Epha5 protein_coding 0.985 75.671 1.026

88.691 0.988 88.729 1.355 78.948
ENSMUSG00000029246 Ppat protein_coding 1.087 34.696 0.994
92.545 0.927 52.087 1.067 76.831
ENSMUSG00000029247 Paics protein_coding 0.994 76.968 0.966
56.027 1.038 16.26 1.02 60.31
ENSMUSG00000029248 1700023E05Rik protein_coding 1 74.516
1 79.807 1 83.538 1 90.674
ENSMUSG00000029249 Rest protein_coding 0.931 40.786 0.984
94.102 1.09 34.285 0.956 83.724
ENSMUSG00000029250 Polr2b protein_coding 1.121 9.435 0.979
93.765 0.911 14.079 0.98 93.722
ENSMUSG00000029253 Cenpc1 protein_coding 1.007 80.688
1.082 88.533 0.914 27.994 0.992 90.289
ENSMUSG00000029254 Stap1 protein_coding 1.28 14.59 0.861 88.704
0.908 15.63 0.826 83.724
ENSMUSG00000029255 Gnrhr protein_coding 0.745 72.985 0.844
85.242 1.592 45.94 1.214 93.964
ENSMUSG00000029260 Ugt2b34 protein_coding 1 83.333 1
91.532 1 87.686 1 94.567
ENSMUSG00000029263 Pigg protein_coding 0.948 36.034 0.979
97.155 1.076 32.524 1.012 91.525
ENSMUSG00000029265 Dr1 protein_coding 1.075 0.944 0.967 85.418
0.959 21.852 1.013 95.74
ENSMUSG00000029267 Mtf2 protein_coding 0.997 85.655 0.946
85.921 1.057 78.864 0.993 95.273
ENSMUSG00000029268 Ugt2a1 protein_coding 1 81.01 1
94.96 1 80.089 1 94.608
ENSMUSG00000029269 Sult1b1 protein_coding 1 49.368 1
96.216 1 80.163 1 94.608
ENSMUSG00000029270 Fam69a protein_coding 1.223 0 0.998
72.712 0.817 17.381 0.932 88.505
ENSMUSG00000029275 Gfi1 protein_coding 1.467 28.395 0.721
91.897 0.948 70.44 1.1 83.067
ENSMUSG00000029276 Glmn protein_coding 1.007 84.324 0.917
75.052 1.085 36.95 1.058 86.445
ENSMUSG00000029279 Brdt protein_coding 1.066 82.705 0.98
93.654 0.959 83.719 1.08 83.067
ENSMUSG00000029280 Smr3a protein_coding 1 77.291 1
96.948 1 85.134 1 94.82
ENSMUSG00000029281 Smr2 protein_coding 1 85.499 1
95.301 1 88.274 1 95.92
ENSMUSG00000029282 Amtn protein_coding 2.765 32.411 0.927
95.708 0.389 46.575 0.423 88.245
ENSMUSG00000029283 Cdc7 protein_coding 0.926 36.541 0.979
97.129 1.1 58.414 1.231 0
ENSMUSG00000029286 Enam protein_coding 1 83.169 1
96.843 1 88.601 1 95.877
ENSMUSG00000029287 Tgfbr3 protein_coding 1.059 41.211
1.033 95.196 0.915 29.217 1.056 79.268
ENSMUSG00000029288 Ambn protein_coding 1 86.474 1
92.561 NA NA 1 94.654
ENSMUSG00000029290 Zfp326 protein_coding 0.985 24.817
0.941 71.41 1.08 14.131 0.978 96.086
ENSMUSG00000029291 Rufy3 protein_coding 1.162 1.395 0.936 78.471

0.918 9.901 0.914 84.29
ENSMUSG00000029298 Gbp9 protein_coding 1.248 11.316 0.939
97.263 0.855 16.409 1.033 96.358
ENSMUSG00000029299 Abcg3 protein_coding 1.424 0.355 0.985 72.399
0.714 18.336 0.965 90.674
ENSMUSG00000029304 Spp1 protein_coding 0.863 42.034 1.13
81.127 1.028 87.37 1.045 95.593
ENSMUSG00000029306 Ibsp protein_coding 1 81.01 1 95.504
1 85.792 1 94.904
ENSMUSG00000029307 Dmp1 protein_coding 1.105 60.853 1.601
93.529 0.567 32.082 0.569 83.724
ENSMUSG00000029309 Sparcl1 protein_coding 1.093 37.941
1.081 88.551 0.848 45.093 0.957 84.29
ENSMUSG00000029310 Nudt9 protein_coding 0.935 8.181 1.012 96.378
1.055 30.561 1.025 86.301
ENSMUSG00000029311 Hsd17b11 protein_coding 1.042 7.959 0.905
31.966 1.063 65.034 0.966 83.724
ENSMUSG00000029312 Klhl8 protein_coding 1.161 5.932 1.058 91.065
0.814 2.986 0.976 92.181
ENSMUSG00000029313 Aff1 protein_coding 1.039 85.77 0.856 60.678
1.123 45.208 1.112 82.328
ENSMUSG00000029314 Agpat9 protein_coding 1.039 77.291 1.05
95.431 0.916 66.201 0.908 86.699
ENSMUSG00000029319 Coq2 protein_coding 0.956 8.45 NA NA
1.049 15.185 0.981 96.248
ENSMUSG00000029321 Slc10a6 protein_coding 1.159 77.497
0.775 70.971 1.115 80.899 1.132 83.067
ENSMUSG00000029322 Plac8 protein_coding 1.48 0.664 1.036 91.897
0.649 42.376 1.421 31.133
ENSMUSG00000029326 Enoph1 protein_coding 1.052 57.38 NA NA
0.931 78.293 1.011 94.904
ENSMUSG00000029328 Hnrnpdl protein_coding 0.982 36.945
0.962 84.594 1.053 30.645 1.076 0
ENSMUSG00000029330 Cds1 protein_coding 1.005 78.639 1.106
83.104 0.902 10.726 1 93.261
ENSMUSG00000029331 4930522N08Rik antisense 1 85.637 1
96.216 1 71.999 1 93.964
ENSMUSG00000029333 Rasgef1b protein_coding 1.407 50.913 0.81
59.946 0.875 87.898 0.976 94.904
ENSMUSG00000029334 Prkg2 protein_coding 0.572 3.07 0.895 95.211
1.949 6.202 1.354 31.133
ENSMUSG00000029335 Bmp3 protein_coding 0.859 64.893 NA NA
1.174 84.308 1.272 94.904
ENSMUSG00000029338 Antxr2 protein_coding 1.302 4.675 0.908
60.678 0.842 39.079 0.992 91.979
ENSMUSG00000029343 Crybb1 protein_coding 1.403 86.145 0.61
79.807 1.165 43.307 0.975 93.132
ENSMUSG00000029344 Tpst2 protein_coding 0.995 58.38 1.112 79.29
0.905 18.797 1.189 83.067
ENSMUSG00000029345 Tfip11 protein_coding 0.952 18.379
1.042 87.029 1.005 49.898 0.979 91.291
ENSMUSG00000029346 Srrd protein_coding 1.035 72.85 0.891 54.245
1.085 49.058 0.954 96.183
ENSMUSG00000029348 Asphd2 protein_coding 0.987 85.391

0.872 82.848 1.158 62.307 1.414 43.377
ENSMUSG00000029352 Crybb3 protein_coding 0.77 5.962 0.875
83.876 1.483 7.024 0.903 87.331
ENSMUSG00000029359 Tesc protein_coding 1.007 85.728 NA NA
0.935 77.294 0.879 94.608
ENSMUSG00000029360 Gm9754 protein_coding 0.768 2.009 1.051
94.767 1.242 8.339 0.915 89.827
ENSMUSG00000029361 Nos1 protein_coding 1.05 62.354 1.715
71.617 0.554 6.858 1.266 60.466
ENSMUSG00000029363 Rfc5 protein_coding 0.982 83.523 0.959
94.48 1.064 74.082 1.239 37.634
ENSMUSG00000029364 Wsb2 protein_coding 1.041 19.132 1.064
61.943 0.903 0.72 1.056 86.118
ENSMUSG00000029366 Dck protein_coding 1.312 0.577 1.135 92.793
0.672 2.563 1.406 46.846
ENSMUSG00000029368 Alb protein_coding 0.529 0.987 1.08 90.116
1.746 7.24 1.031 93.413
ENSMUSG00000029369 Afm protein_coding 1.271 68.727 0.669
79.807 1.18 66.043 0.478 86.991
ENSMUSG00000029370 Rassf6 protein_coding 0.766 8.45 1.039
83.104 1.257 57.907 0.977 96.183
ENSMUSG00000029372 Ppbp protein_coding 1.133 56.311 0.758
82.848 1.159 84.308 0.947 95.877
ENSMUSG00000029373 Pf4 protein_coding 1.88 0 1.057 95.211
0.503 1.342 1.213 50.452
ENSMUSG00000029375 Cxcl15 protein_coding 1.459 1.292 1.597
60.309 0.429 0.72 1.287 83.067
ENSMUSG00000029376 Mthfd2l protein_coding 0.963 79.165
0.974 95.504 1.063 65.034 0.98 86.991
ENSMUSG00000029378 Areg protein_coding 0.943 77.291 0.783
92.545 1.356 84.504 1.614 90.177
ENSMUSG00000029379 Cxcl3 protein_coding 1 59.674 NA NA
1 67.823 1 93.612
ENSMUSG00000029380 Cxcl1 protein_coding 0.979 56.477 0.907
90.287 1.122 43.185 0.927 96.358
ENSMUSG00000029381 Shroom3 protein_coding 1.069 37.702
1.006 96.268 0.932 42.895 0.913 84.29
ENSMUSG00000029384 2010109A12Rik protein_coding 1.41 20.444
0.857 79.807 0.825 67.962 0.545 83.724
ENSMUSG00000029385 Ccng2 protein_coding 1.078 21.703 0.929
70.693 0.995 85.464 0.975 84.29
ENSMUSG00000029386 Tctn2 protein_coding 1.044 77.182 0.95
60.678 1.006 53.449 0.957 94.82
ENSMUSG00000029387 Gtf2h3 protein_coding 1.009 84.865
0.968 96.948 1.022 78.864 0.976 85.493
ENSMUSG00000029388 Eif2b1 protein_coding 0.962 35.646
0.971 77.944 1.072 11.348 1.018 83.067
ENSMUSG00000029389 Ddx55 protein_coding 0.921 8.45 1.031 82.904
1.054 70.328 1.084 42.736
ENSMUSG00000029390 Tmed2 protein_coding 1.009 81.178 1.059
72.642 0.933 14.262 1.034 85.629
ENSMUSG00000029392 Rilpl1 protein_coding 0.945 38.555
1.097 72.642 0.966 88.693 1.059 72.992
ENSMUSG00000029394 Cdk2ap1 protein_coding 0.912 8.365 1.063

71.98 1.027 56.931 1.017 83.067
ENSMUSG00000029397 Rchy1 protein_coding 1.006 85.728 0.977
94.96 1.015 84.83 1.008 96.248
ENSMUSG00000029401 Rilpl2 protein_coding 1.116 47.847 1.03
82.904 0.867 8.313 0.842 83.724
ENSMUSG00000029402 Snrnp35 protein_coding 0.958 18.138
0.976 90.046 1.069 24.375 1.11 44.882
ENSMUSG00000029403 Cdkl2 protein_coding 0.891 18.602 1.094
79.864 1.024 85.352 1.053 96.358
ENSMUSG00000029404 Arl6ip4 protein_coding 0.918 17.705
1.035 91.584 1.052 27.732 1.021 90.397
ENSMUSG00000029405 G3bp2 protein_coding 1.045 10.391 1.07
83.104 0.895 4.23 1.002 91.291
ENSMUSG00000029406 Pitpnm2 protein_coding 0.801 0.987 1.108
72.642 1.129 13.356 0.975 92.018
ENSMUSG00000029407 Uso1 protein_coding 1.007 74.689 0.97
94.262 1.021 67.163 1.045 61.316
ENSMUSG00000029408 Abcb9 protein_coding 0.851 21.897 1.16
60.309 1.013 51.715 0.991 96.183
ENSMUSG00000029410 Ppef2 protein_coding 0.986 84.168 1.509
71.98 0.67 11.314 0.953 94.825
ENSMUSG00000029413 Naaa protein_coding 1.189 1.009 0.983 95.504
0.856 3.234 1.226 46.846
ENSMUSG00000029414 Kntc1 protein_coding 1.531 36.195 1.02
90.744 0.642 78.642 1.709 67.691
ENSMUSG00000029415 Sdad1 protein_coding 0.942 54.983 0.978
95.504 1.089 65.567 1.137 44.679
ENSMUSG00000029416 Slc15a4 protein_coding 1.065 32.676 NA
NA 0.929 28.489 1.072 80.324
ENSMUSG00000029417 Cxcl9 protein_coding 1.074 57.655 1.054
83.104 0.88 22.009 0.79 83.724
ENSMUSG00000029419 Gm996 protein_coding 1.111 71.516 0.885
91.955 1.014 83.719 0.81 86.699
ENSMUSG00000029420 Rimbp2 protein_coding 0.745 70.213
1.364 83.104 0.986 64.778 0.74 96.183
ENSMUSG00000029422 Rsrc2 protein_coding 0.934 8.365 0.966 70.693
1.109 6.916 1.026 83.276
ENSMUSG00000029426 Scarb2 protein_coding 1.009 48.288
0.931 85.95 1.063 83.275 1.047 68.696
ENSMUSG00000029427 Zcchc8 protein_coding 1.007 75.196 1.01
90.116 0.985 41.928 0.995 93.964
ENSMUSG00000029428 Stx2 protein_coding 1.054 72.85 1.091 72.642
0.87 12.41 1.074 42.736
ENSMUSG00000029430 Ran protein_coding 0.95 15.456 1.088
72.546 0.969 88.693 1.067 66.068
ENSMUSG00000029431 B3gnt4 protein_coding 1.06 86.351
1.088 82.904 0.869 41.662 0.93 91.625
ENSMUSG00000029432 Gbas protein_coding 1.03 75.196 1.063
77.618 0.913 17.662 0.99 95.35
ENSMUSG00000029433 Diablo protein_coding 0.962 34.572
1.035 82.219 1.004 82.685 1.038 71.305
ENSMUSG00000029434 Vps33a protein_coding 1.06 52.134
0.956 79.475 0.989 88.601 1.031 71.305
ENSMUSG00000029436 Mmp17 protein_coding 1.147 16.124 0.955

95.088	0.914	35.022	1.02	94.82
ENSMUSG00000029437	Il31	protein_coding	1	86.918	1
96.843	1	85.757	1	95.676
ENSMUSG00000029438	Bcl7a	protein_coding	0.98	67.163	1.016
96.216	1.007	70.9	0.871	83.724
ENSMUSG00000029439	Sfswap	protein_coding	0.884	0.987	1.004
96.216	1.128	5.864	0.963	88.387
ENSMUSG00000029440	Psmd9	protein_coding	0.888	1.623	0.993	88.881
1.135	7.143	1.07	57.075
ENSMUSG00000029442	Wdr66	protein_coding	1.115	41.454	0.906
71.889	NA	NA	0.933	91.217
ENSMUSG00000029445	Hpd	protein_coding	1.2	70.741	1.09
95.504	0.761	85.935	0.327	85.493
ENSMUSG00000029446	Psph	protein_coding	0.76	11.924	1.274
82.76	1.03	65.034	0.976	96.248
ENSMUSG00000029447	Cct6a	protein_coding	0.981	54.043	1.005
95.504	1.012	64.624	1.058	44.882
ENSMUSG00000029449	Rhof	protein_coding	0.588	1.15	1.089	90.116
1.562	6.202	0.807	83.724
ENSMUSG00000029451	Gm13103	protein_coding	1	84.977	1
96.268	1	87.971	1	93.261
ENSMUSG00000029452	Tmem116	protein_coding	0.768	11.769
1.041	96.843	1.245	24.383	1.028	91.525
ENSMUSG00000029454	Mapkapk5	protein_coding	0.881	0.987	1.076
65.165	1.056	5.964	1.024	56.339
ENSMUSG00000029455	Aldh2	protein_coding	0.842	1.343	1.051	90.092
1.132	9.176	0.963	89.892
ENSMUSG00000029456	Acad10	protein_coding	1.163	22.205
0.903	73.766	0.953	73.096	0.857	83.724
ENSMUSG00000029458	Brap	protein_coding	1.019	53.562	1.037
85.985	0.944	21.666	0.99	92.675
ENSMUSG00000029461	Fam168a	protein_coding	1.073	6.853	0.932
48.565	0.999	67.962	1.037	93.734
ENSMUSG00000029462	Vps29	protein_coding	0.927	9.841	1.085	64.304
0.99	85.352	1.107	49.514
ENSMUSG00000029463	Fam216a	protein_coding	0.928	29.454
1.057	72.546	NA	NA	0.993	95.72
ENSMUSG00000029464	Gpn3	protein_coding	0.953	45.229	0.992
89.509	1.056	41.235	0.976	86.254
ENSMUSG00000029465	Arpc3	protein_coding	1.009	76.135	0.962
79.894	1.031	27.431	1.099	52.982
ENSMUSG00000029466	Anapc7	protein_coding	0.982	79.709	0.99
73.766	1.026	45.449	0.997	87.992
ENSMUSG00000029467	Atp2a2	protein_coding	1.043	28.927
1.029	72.642	0.928	3.234	1.052	83.067
ENSMUSG00000029468	P2rx7	protein_coding	1.212	13.292	0.894
82.503	0.918	36.786	0.904	84.29
ENSMUSG00000029469	Ift81	protein_coding	1.106	25.421	0.92
82.848	0.983	76.768	0.983	92.675
ENSMUSG00000029470	P2rx4	protein_coding	1.209	0.664	0.897	40.463
0.922	25.345	0.954	84.19
ENSMUSG00000029471	Camkk2	protein_coding	1.022	56.311
1.118	79.055	0.872	6.935	1.045	86.445
ENSMUSG00000029472	Anapc5	protein_coding	0.976	54.604

1.032 79.261 0.994 86.346 1.06 31.133
ENSMUSG00000029474 Rnf34 protein_coding 1.092 14.194 0.997
93.654 0.919 23.607 1.024 89.72
ENSMUSG00000029475 Kdm2b protein_coding 1.062 16.959 1.014
93.046 0.925 31.676 0.955 83.724
ENSMUSG00000029477 Morn3 protein_coding 0.803 76.968 0.813
86.604 1.537 49.601 0.86 87.542
ENSMUSG00000029478 Ncor2 protein_coding 0.992 85.655 1.047
87.029 0.963 66.425 1.027 95.273
ENSMUSG00000029480 Dhx37 protein_coding 0.973 56.477 1.002
82.904 1.028 87.898 1.052 92.691
ENSMUSG00000029482 Aacs protein_coding 0.624 1.175 1.185 77.598
1.355 20.085 1.184 91.525
ENSMUSG00000029484 Anxa3 protein_coding 1.136 17.492 0.964
93.654 0.916 52.213 1.236 48.119
ENSMUSG00000029486 Mrpl1 protein_coding 1.012 66.944 0.988
96.138 1.003 83.811 1.06 68.696
ENSMUSG00000029490 Mfsd7a protein_coding 1.212 0.767 0.851
53.434 0.97 69.583 1.097 36.174
ENSMUSG00000029499 Pxmp2 protein_coding 0.834 4.634 NA NA
1.287 32.524 0.933 86.699
ENSMUSG00000029500 Pgam5 protein_coding 0.997 86.74 1.053 87.185
0.956 46.956 1.029 83.067
ENSMUSG00000029501 Ankle2 protein_coding 1.077 3.176 0.98
71.41 0.949 15.376 1.024 85.786
ENSMUSG00000029502 Golga3 protein_coding 1.001 74.689
0.993 96.138 1.008 78.369 1.053 40.894
ENSMUSG00000029503 P2rx2 protein_coding 0.922 81.178 0.72
74.483 1.51 37.369 1.251 92.748
ENSMUSG00000029504 Ddx51 protein_coding 0.921 42.361 1.015
90.712 1.071 36.388 1.014 94.904
ENSMUSG00000029505 Ep400 protein_coding 0.953 23.319 0.966
75.052 1.088 12.467 1.032 87.447
ENSMUSG00000029507 Pus1 protein_coding 0.962 42.034 0.972
91.661 1.07 38.975 1.012 86.301
ENSMUSG00000029510 Gpc2 protein_coding 0.718 1.422 1.108 95.196
1.251 9.567 0.792 85.493
ENSMUSG00000029512 Ulk1 protein_coding 0.984 76.546 1.025
95.708 0.991 82.31 1.03 92.31
ENSMUSG00000029513 Prkab1 protein_coding 1.108 38.195
0.964 97.155 0.933 34.21 0.942 83.952
ENSMUSG00000029516 Cit protein_coding 1.184 27.32 0.867 75.052
0.972 77.037 1.219 87.447
ENSMUSG00000029518 Rab35 protein_coding 1.062 14.59 0.996 96.984
0.945 3.943 1.036 79.268
ENSMUSG00000029521 Chek2 protein_coding 1.001 83.333 1.035
95.088 0.965 81.525 0.981 92.12
ENSMUSG00000029524 Sirt4 protein_coding 0.887 0.987 1.007 84.999
1.113 6.202 0.923 83.724
ENSMUSG00000029528 Pxn protein_coding 1.03 55.462 1.019
95.504 0.954 56.822 1.085 31.133
ENSMUSG00000029530 Ccr9 protein_coding 2.546 58.709 0.491
59.946 0.802 76.498 0.775 95.35
ENSMUSG00000029534 St7 protein_coding 1.04 71.516 0.961

90.256	1.003	81.29	1.038	79.545
ENSMUSG00000029535	Triap1	protein_coding	0.987	81.925
1.064	88.533	0.954	67.029	1.009	83.067
ENSMUSG00000029536	Gatc	protein_coding	0.996	72.85	1.101	72.546
0.911	5.453	1.007	95.35
ENSMUSG00000029538	Srsf9	protein_coding	1.058	44.061	0.984
85.671	0.963	69.134	0.965	93.261
ENSMUSG00000029541	Cyp2w1	protein_coding	1	81.01	1
91.643	1	66.201	1	91.269
ENSMUSG00000029544	Cabp1	protein_coding	0.909	32.557	0.983
94.48	1.124	31.317	0.98	92.181
ENSMUSG00000029545	Acads	protein_coding	0.844	0.987	1.047	79.411
1.133	16.808	0.986	91.372
ENSMUSG00000029546	Uncx	protein_coding	1	84.564	1
97.097	1	87.786	1	95.92
ENSMUSG00000029547	Ints1	protein_coding	0.846	1.031	1.081	71.98
1.092	21.462	1.024	73.823
ENSMUSG00000029550	Sppl3	protein_coding	1.069	4.675	0.994	94.767
0.944	4.759	0.938	83.724
ENSMUSG00000029551	Psmg3	protein_coding	0.946	81.465	1.005
96.013	1.053	66.928	1.021	93.722
ENSMUSG00000029552	Tes	protein_coding	1.116	12.72	0.98	83.58
0.912	40.19	1.028	93.685
ENSMUSG00000029553	Tfec	protein_coding	1.451	20.032	0.706
82.848	0.974	72.269	1.429	59.394
ENSMUSG00000029554	Mad1l1	protein_coding	0.901	34.954
0.953	71.98	1.165	7.365	1.173	31.133
ENSMUSG00000029556	Hnf1a	protein_coding	0.838	67.867	0.919
88.897	1.297	43.307	1.172	95.72
ENSMUSG00000029557	Ftsj2	protein_coding	0.994	86.819	1.001
93.654	1	82.436	1.032	92.018
ENSMUSG00000029559	2210016L21Rik	protein_coding	1.001	70.862
1.015	92.931	NA	NA	1.028	89.428
ENSMUSG00000029560	Snx8	protein_coding	1.136	0	0.942	61.883
0.931	33.971	1.123	57.1
ENSMUSG00000029561	Oasl2	protein_coding	1.383	0.999	1.146	96.897
0.63	21.117	1.496	38.598
ENSMUSG00000029563	Foxp2	protein_coding	1.242	27.731	0.958
96.216	0.836	34.592	1.224	83.067
ENSMUSG00000029564	4930519G04Rik	protein_coding	1	77.407
1	91.361	1	72.853	1	89.152
ENSMUSG00000029569	Tmem168	protein_coding	1.112	2.651	0.92
85.671	0.979	15.465	1.045	94.82
ENSMUSG00000029570	Lfng	protein_coding	1.075	68.727	1.01
94.367	0.922	41.662	1.015	91.928
ENSMUSG00000029571	Tmem106b	protein_coding	1.043	66.76	0.974
88.897	0.982	81.525	0.981	86.699
ENSMUSG00000029575	Mmab	protein_coding	0.921	22.481	1.241
60.309	0.871	13.848	1.018	83.276
ENSMUSG00000029576	Radil	protein_coding	0.93	53.407	1.082
83.104	0.988	87.686	0.99	95.35
ENSMUSG00000029577	Ube3b	protein_coding	1.008	86.819	1.056
71.617	0.935	23.1	0.991	93.734
ENSMUSG00000029578	Wipi2	protein_coding	0.913	4.176	1.049	92.561

1.041 8.339 0.973 91.985
ENSMUSG00000029580 Actb protein_coding 1.077 8.996 1.098 82.904
0.845 1.061 1.058 92.762
ENSMUSG00000029581 Fscn1 protein_coding 0.8 17.401 1.119
80.806 1.116 70.328 1.076 91.492
ENSMUSG00000029586 4933411G11Rik protein_coding 1 81.465
1 89.996 1 53.587 1 96.358
ENSMUSG00000029587 Zfp12 protein_coding 1.032 70.741 1.012
96.652 0.954 64.427 0.989 95.593
ENSMUSG00000029591 Ung protein_coding 0.986 73.487 NA NA
0.922 79.426 0.871 92.748
ENSMUSG00000029592 Usp30 protein_coding 1.09 15.85 0.939 71.889
0.979 77.704 0.955 83.724
ENSMUSG00000029594 Rbm19 protein_coding 0.926 34.795 0.998
96.68 1.083 55.109 1.11 56.079
ENSMUSG00000029596 Sdsl protein_coding 0.65 0.987 1.102 79.055
1.39 7.855 1.105 87.624
ENSMUSG00000029597 Sds protein_coding 1.169 73.734 0.865
90.053 0.987 87.686 0.49 83.724
ENSMUSG00000029598 Plbd2 protein_coding 0.965 30.877 0.994
96.407 1.039 64.024 0.983 93.734
ENSMUSG00000029599 Ddx54 protein_coding 0.85 4.634 0.981 91.361
1.201 9.807 1.063 75.81
ENSMUSG00000029600 1110008J03Rik protein_coding 0.892 21.658
1.099 71.617 1.022 88.693 0.973 86.699
ENSMUSG00000029601 Iqcd protein_coding 0.808 66.652 NA NA
1.054 79.897 1.094 84.246
ENSMUSG00000029602 Rasal1 protein_coding 0.885 38.983
0.939 83.733 1.205 16.26 0.826 84.29
ENSMUSG00000029603 Dtx1 protein_coding 0.984 65.199 1.079
91.776 0.94 79.325 0.838 83.724
ENSMUSG00000029605 Oas1b polymorphic_pseudogene 1.047 76.546 0.99
96.843 0.958 56.822 1.138 54.153
ENSMUSG00000029607 Ankrd61 protein_coding 0.929 77.615 1.15
91.643 0.938 88.729 0.788 83.724
ENSMUSG00000029608 Rph3a protein_coding 1.166 86.006 0.803
90.378 1.068 83.925 1.26 94.904
ENSMUSG00000029610 Aimp2 protein_coding 0.942 29.454 1.145
64.304 0.927 42.895 1.106 68.354
ENSMUSG00000029613 Eif2ak1 protein_coding 1.004 79.853
1.049 83.104 0.952 30.468 1.022 87.447
ENSMUSG00000029614 Rpl6 protein_coding 1.022 67.608 0.92
31.637 1.066 35.894 1.026 86.301
ENSMUSG00000029616 Erp29 protein_coding 1.026 64.956 0.961
82.671 1.013 83.925 1.057 56.863
ENSMUSG00000029617 Ccz1 protein_coding 1.051 15.602 0.959
67.739 0.997 76.768 1.019 96.183
ENSMUSG00000029618 Ocm protein_coding 1 70.213 1
95.708 1 88.274 1 95.74
ENSMUSG00000029620 1700018F24Rik protein_coding 1 80.251
1 97.155 1 86.144 1 94.468
ENSMUSG00000029621 Arpc1a protein_coding 1.068 20.942 0.97
72.399 0.968 77.704 0.962 95.832
ENSMUSG00000029622 Arpc1b protein_coding 1.087 16.249

0.956 94.597 0.966 52.087 1.138 44.075
ENSMUSG00000029623 Pdap1 protein_coding 0.851 0.987 NA NA
1.158 5.864 1.091 46.412
ENSMUSG00000029624 NA NA NA NA 1.004 79.475 1.023
46.206 1.002 95.273
ENSMUSG00000029625 Cpsf4 protein_coding 0.941 44.922 0.972
93.046 1.089 34.285 1.058 89.428
ENSMUSG00000029627 Zkscan14 protein_coding 0.953 42.034
0.917 47.727 1.146 5.864 1.012 93.722
ENSMUSG00000029629 Phf14 protein_coding 1.018 24.689 0.965
71.41 1.015 84.434 1.097 71.305
ENSMUSG00000029630 Cyp3a25 protein_coding 1 84.049 1
97.129 1 86.084 1 96.358
ENSMUSG00000029632 Ndufa4 protein_coding 0.849 4.825 1.045
80.277 1.125 41.6 0.958 96.248
ENSMUSG00000029633 Gm5578 pseudogene 1.169 76.319 1.128
96.948 0.76 77.704 1.221 91.872
ENSMUSG00000029634 Rnf6 protein_coding 1.132 4.228 0.982 97.263
0.9 1.764 1.014 94.752
ENSMUSG00000029635 Cdk8 protein_coding 1.056 35.787 0.898
74.851 1.051 81.29 0.985 85.493
ENSMUSG00000029636 Wasf3 protein_coding 1.011 72.139 0.925
97.288 1.067 88.203 1.164 59.464
ENSMUSG00000029638 Glcci1 protein_coding 0.838 33.599
1.206 86.34 0.987 74.082 0.779 85.493
ENSMUSG00000029640 Usp12 protein_coding 1.069 8.477 0.997 96.763
0.938 1.871 1.009 91.872
ENSMUSG00000029641 Rasl11a protein_coding 0.975 81.465
0.931 97.263 1.097 83.275 0.835 83.724
ENSMUSG00000029642 Polr1d protein_coding 0.953 17.062
0.995 80.052 1.051 10.819 1.051 84.499
ENSMUSG00000029644 Pdx1 protein_coding 1 82.891 1
93.654 1 86.831 1 96.183
ENSMUSG00000029646 Cdx2 protein_coding 1 81.125 NA NA
1 83.719 1 95.92
ENSMUSG00000029647 Pan3 protein_coding 1.022 26.338 0.974
94.102 1.007 54.521 0.965 83.724
ENSMUSG00000029648 Flt1 protein_coding 1.035 84.977 1.028
97.049 0.94 71.734 0.954 92.31
ENSMUSG00000029649 Pomp protein_coding 0.968 48.646 1.067
93.899 0.965 87.37 1.029 87.7
ENSMUSG00000029650 Slc46a3 protein_coding 1.129 34.696
0.877 87.452 1.007 81.169 0.933 83.724
ENSMUSG00000029651 Mtus2 protein_coding 0.948 86.658 1.16
95.088 0.906 70.9 1.17 83.067
ENSMUSG00000029655 N4bp2l2 protein_coding 1.062 2.723 0.951
68.115 0.99 63.919 0.996 94.153
ENSMUSG00000029656 C8b protein_coding 1 84.324 1
86.071 1 84.504 NA NA
ENSMUSG00000029657 Hsph1 protein_coding 0.811 44.787 1.837
89.434 0.669 78.713 1.14 67.691
ENSMUSG00000029658 Wdr95 protein_coding 0.688 83.169 2.134
92.111 0.677 66.753 0.523 84.19
ENSMUSG00000029659 Medag protein_coding 1.271 0.2 0.971 82.503

0.812 7.155 1.176 31.133
ENSMUSG00000029660 Tex26 protein_coding 0.581 57.227 2.012
82.809 0.853 82.31 0.682 92.742
ENSMUSG00000029661 Col1a2 protein_coding 1.151 10.391
0.877 59.946 0.992 76.388 1.338 31.133
ENSMUSG00000029663 Gngt1 protein_coding 1 86.112 NA NA
1 88.693 1 94.904
ENSMUSG00000029664 Tfpi2 protein_coding 1.497 3.176 0.961 88.691
0.696 15.169 1.126 78.948
ENSMUSG00000029669 Tspan12 protein_coding 1.046 82.231
1.038 82.904 0.919 22.009 0.908 88.053
ENSMUSG00000029670 Ing3 protein_coding 1.072 19.893 0.987
90.287 0.948 56.365 0.968 85.493
ENSMUSG00000029671 Wnt16 protein_coding 1.183 67.163 0.655
90.053 1.293 88.349 0.801 89.587
ENSMUSG00000029672 Fam3c protein_coding 0.993 83.968 1.087
95.504 0.922 87.898 1.002 94.153
ENSMUSG00000029673 Auts2 protein_coding 0.945 73.585 1.115
77.618 0.949 29.765 0.921 83.724
ENSMUSG00000029674 Limk1 protein_coding 1.023 33.099 1.001
95.504 0.976 44.865 1.21 31.133
ENSMUSG00000029675 Eln protein_coding 0.676 0.987 0.904 74.483
1.637 5.864 1.061 80.187
ENSMUSG00000029676 Pot1a protein_coding 0.992 76.135 0.931
94.96 1.079 75.527 1.042 78.948
ENSMUSG00000029678 Hyal5 protein_coding 1 85.499 1
97.155 1 86.831 1 93.964
ENSMUSG00000029679 Hyal6 protein_coding 1 53.138 1
90.576 1 86.602 1 96.358
ENSMUSG00000029680 Hyal4 protein_coding 1 81.01 1 95.211
1 87.274 1 93.264
ENSMUSG00000029681 Bcl7b protein_coding 0.872 2.009 1.033 81.127
1.11 38.926 1.017 91.872
ENSMUSG00000029682 Spam1 protein_coding 1 85.391 NA NA
1 88.461 1 89.72
ENSMUSG00000029683 Lmod2 protein_coding 2.406 38.195 1.19
88.91 0.348 20.511 1.401 78.042
ENSMUSG00000029684 Wasl protein_coding 1.073 8.281 NA NA
0.934 8.382 0.966 85.493
ENSMUSG00000029685 Asb15 protein_coding 4.145 7.174 1.511 95.735
0.159 0.879 1.321 94.016
ENSMUSG00000029686 Cul1 protein_coding 1.084 16.539 0.934
79.807 0.987 56.822 0.985 95.273
ENSMUSG00000029687 Ezh2 protein_coding 1.039 34.861 0.868
62.322 1.107 76.923 1.052 94.016
ENSMUSG00000029695 Aass protein_coding 1.107 70.36 0.872 82.848
1.04 66.928 1.146 91.783
ENSMUSG00000029699 Srcrb4d protein_coding 0.755 24 1.05
95.329 1.261 43.586 1.194 87.134
ENSMUSG00000029701 Rbm28 protein_coding 0.873 7.857 1.142 83.104
0.999 49.898 1.079 50.452
ENSMUSG00000029703 Lrwd1 protein_coding 0.908 11.726 1.038
91.584 1.062 45.035 1.024 92.691
ENSMUSG00000029705 Cux1 protein_coding 1.007 80.146 1.024

82.904	0.971	56.625	0.948	85.493
ENSMUSG00000029706	Pax4	protein_coding	1	85.946	1
96.216	1	88.729	1	87.624
ENSMUSG00000029708	Gcc1	protein_coding	1.034	25.026	0.968
94.597	0.997	60.233	0.923	85.493
ENSMUSG00000029710	Ephb4	protein_coding	0.916	16.652	0.993
95.088	1.095	48.451	1.025	96.287
ENSMUSG00000029711	Epo	protein_coding	1	78.299	1
95.504	1	87.431	1	96.287
ENSMUSG00000029713	Gnb2	protein_coding	0.96	46.413	0.995
93.899	1.046	69.002	1.053	71.305
ENSMUSG00000029714	Gigyf1	protein_coding	0.86	11.707	NA
NA	1.161	25.808	0.999	84.29
ENSMUSG00000029715	Pop7	protein_coding	0.843	8.002	1.069	72.546
1.112	54.163	1.114	71.855
ENSMUSG00000029716	Tfr2	protein_coding	0.961	43.495	0.84
97.097	1.242	55.109	1.124	87.447
ENSMUSG00000029718	Pcolce	protein_coding	1.249	0.664	0.934
97.155	0.857	3.428	1.142	60.31
ENSMUSG00000029720	Gm20605	protein_coding	0.601	2.5	1.156
83.104	1.437	31.4	0.821	91.372
ENSMUSG00000029722	Agfg2	protein_coding	0.973	39.712	1.078
82.103	0.955	87.37	1.011	95.74
ENSMUSG00000029723	Tsc22d4	protein_coding	1.006	67.991
1.014	96.407	0.98	32.963	1.003	91.105
ENSMUSG00000029725	Ppp1r35	protein_coding	0.905	17.898
1.102	82.103	NA	NA	1.071	88.111
ENSMUSG00000029726	Mepce	protein_coding	1.04	41.211	0.994
96.68	0.967	30.468	0.945	83.952
ENSMUSG00000029727	Cyp3a13	protein_coding	1.135	77.291
0.739	82.073	1.187	54.163	1.136	96.183
ENSMUSG00000029729	Zkscan1	protein_coding	0.979	49.621
0.995	87.322	1.021	32.173	0.979	92.181
ENSMUSG00000029730	Mcm7	protein_coding	0.905	76.827	0.912
21.315	1.206	27.533	1.162	76.046
ENSMUSG00000029735	Tpk1	protein_coding	1.134	20.296	0.996
96.897	0.881	6.935	0.938	86.991
ENSMUSG00000029752	Asns	protein_coding	1.04	70.741	1.066
96.948	0.899	75.967	1.012	95.273
ENSMUSG00000029755	Dlx5	protein_coding	1.124	76.319	0.84
91.532	1.057	72.378	0.833	86.699
ENSMUSG00000029757	Dync1i1	protein_coding	1.752	24.481
0.583	96.984	0.976	41.506	1.661	88.111
ENSMUSG00000029759	Pon3	protein_coding	0.974	55.303	0.966
87.041	1.059	33.042	0.971	88.552
ENSMUSG00000029761	Cald1	protein_coding	0.991	40.786	1.046
89.434	0.968	84.724	1.042	50.452
ENSMUSG00000029762	Akr1b8	protein_coding	1.003	82.982
1.005	91.361	0.986	75.155	1.198	33.269
ENSMUSG00000029763	Exoc4	protein_coding	1.046	20.659	0.983
96.847	0.973	30.468	1.1	31.133
ENSMUSG00000029765	Plxna4	protein_coding	0.88	51.056
0.755	42.923	1.505	7.635	0.998	93.964
ENSMUSG00000029766	1700012A03Rik	protein_coding	1	85.203

1	96.897	1	79.524	1	96.086
ENSMUSG00000029767	Calu	protein_coding	1.206	4.465	0.904	96.843	0.917	7.399	1.157	31.133
ENSMUSG00000029769	Ccdc136	protein_coding	0.83	39.953	NA	NA	1.174	52.8	0.837	88.505
ENSMUSG00000029771	Irf5	protein_coding	1.266	0	0.86	65.819	0.915	27.994	1.088	70.85
ENSMUSG00000029772	Ahcyl2	protein_coding	0.854	8.365	1.066	82.904	1.102	39.439	1.052	73.368
ENSMUSG00000029775	Klhdc10	protein_coding	0.963	23.556	1.01	96.68	1.028	38.926	1	83.276
ENSMUSG00000029776	Hibadh	protein_coding	1.05	85.283	0.937	95.088	1.016	87.744	0.93	83.952
ENSMUSG00000029777	Gars	protein_coding	1.04	38.195	0.997	97.155	0.964	42.997	1.026	86.133
ENSMUSG00000029778	Adcyap1r1	protein_coding	0.97	64.014	0.852	93.654	1.212	50.636	1.134	86.133
ENSMUSG00000029780	Nt5c3	protein_coding	1.086	22.871	0.992	85.793	0.929	74.535	1.095	65.175
ENSMUSG00000029781	Fkbp9	protein_coding	1.136	9.695	0.962	81.02	0.914	48.577	1.027	68.696
ENSMUSG00000029782	Tmem209	protein_coding	1.094	26.136	0.898	76.431	1.021	72.148	1.034	68.696
ENSMUSG00000029787	Avl9	protein_coding	0.957	31.333	0.974	83.58	1.073	16.808	0.914	83.724
ENSMUSG00000029790	Cep41	protein_coding	1.142	41.211	1.062	83.464	0.826	22.009	1.045	74.193
ENSMUSG00000029797	Sspo	protein_coding	0.71	5.962	1.076	92.793	1.307	11.151	0.835	83.724
ENSMUSG00000029798	Herc6	protein_coding	1.012	84.168	1.102	89.434	0.897	40.794	1.151	60.968
ENSMUSG00000029802	Abcg2	protein_coding	1.124	19.315	0.859	81.731	1.037	86.985	1.013	90.177
ENSMUSG00000029804	Herc3	protein_coding	1.029	57.133	1.158	87.029	0.841	2.817	0.929	83.724
ENSMUSG00000029810	Tmem176b	protein_coding	1.036	72.85	1.018	92.404	0.944	74.325	1.034	88.111
ENSMUSG00000029811	Aoc1	protein_coding	0.86	85.832	5.105	81.127	0.228	24.918	6.8	57.075
ENSMUSG00000029813	1600015I10Rik	protein_coding	1.239	86.351	NA	NA	0.751	82.31	0.757	94.904
ENSMUSG00000029814	Igf2bp3	protein_coding	0.903	67.867	0.642	79.475	1.729	38.152	0.992	87.088
ENSMUSG00000029815	Malsu1	protein_coding	1.022	70.741	0.952	87.041	1.027	64.305	1.02	89.052
ENSMUSG00000029816	Gpnmb	protein_coding	1.216	58.38	0.906	93.994	0.904	42.997	0.784	83.724
ENSMUSG00000029817	Tra2a	protein_coding	0.965	77.615	1.098	93.27	0.945	74.179	1.04	88.797
ENSMUSG00000029819	Npy	protein_coding	1	71.915	1	89.014	1	88.601	1	94.904
ENSMUSG00000029821	Dfna5	protein_coding	0.899	8.365	0.985	97.129	1.132	20.968	0.983	92.018
ENSMUSG00000029822	Osbpl3	protein_coding	0.993	84.661

1.042 96.68 0.964 81.62 1.048 93.156
ENSMUSG00000029823 Luc7l2 protein_coding 1.036 10.858
0.924 57.613 1.045 62.237 0.94 83.724
ENSMUSG00000029826 Zc3hav1 protein_coding 1.169 0 NA NA
0.834 0.879 1.03 63.708
ENSMUSG00000029828 4921507P07Rik protein_coding 0.795 25.85
1.063 96.216 1.185 25.966 1.053 83.276
ENSMUSG00000029829 Tmem213 protein_coding 1 81.647 1
92.545 1 80.899 1 92.181
ENSMUSG00000029830 Svopl protein_coding 1.311 76.546 0.928
87.998 NA NA 0.846 95.72
ENSMUSG00000029831 Npvf protein_coding 1 76.319 1
92.631 1 76.498 1 96.287
ENSMUSG00000029832 Nfe2l3 protein_coding 1.344 9.206 0.816
71.889 0.912 59.125 0.928 90.611
ENSMUSG00000029833 Trim24 protein_coding 0.869 10.143
0.902 65.165 1.279 5.864 1.066 66.068
ENSMUSG00000029836 Cbx3 protein_coding 1.183 0 0.912 41.951
0.928 6.858 1.017 87.7
ENSMUSG00000029837 1700111E14Rik protein_coding 0.203 5.544
1.209 92.253 4.072 13.956 0.932 96.183
ENSMUSG00000029838 Ptn protein_coding 0.622 0.987 0.978 96.843
1.639 11.782 0.895 86.254
ENSMUSG00000029840 Mtpn protein_coding 1.023 43.344 1.033
90.68 0.947 21.666 1.012 84.499
ENSMUSG00000029843 Slc13a4 protein_coding 0.863 24.817
1.128 91.584 1.028 72.695 0.966 87.735
ENSMUSG00000029844 Hoxa1 protein_coding 0.959 80.688 1.062
95.735 0.977 54.037 1.128 93.132
ENSMUSG00000029847 2010107G12Rik protein_coding 0.751 38.555
0.892 95.504 1.497 44.515 0.96 95.273
ENSMUSG00000029848 Stra8 protein_coding 1 73.585 1
92.545 1 79.426 1 92.564
ENSMUSG00000029851 Fam115c protein_coding 0.969 48.036
1.073 71.617 0.958 71.999 0.943 94.654
ENSMUSG00000029859 Epha1 protein_coding 0.916 59.674 0.905
64.123 1.202 36.578 0.954 85.493
ENSMUSG00000029860 Zyx protein_coding 1.206 1.441 0.981 92.474
0.845 10.003 1.057 49.514
ENSMUSG00000029861 Fam131b protein_coding 1.14 34.696
0.921 64.123 0.949 82.161 1.065 89.108
ENSMUSG00000029862 Clcn1 protein_coding 2.509 7.397 2.885 76.086
0.138 0.72 0.894 96.192
ENSMUSG00000029863 Casp2 protein_coding 1.028 70.741 0.974
92.137 1.001 87.336 0.855 84.29
ENSMUSG00000029864 Gstk1 protein_coding 0.936 65.388 1.023
96.843 1.046 79.554 1 95.676
ENSMUSG00000029865 Sval1 protein_coding 1 85.59 1 95.088
1 85.757 1 96.071
ENSMUSG00000029866 NA NA NA NA 1 91.776 1
76.64 1 95.593
ENSMUSG00000029867 1700034O15Rik protein_coding 1 68.66 1
90.29 1 84.308 1 95.72
ENSMUSG00000029868 Trpv6 protein_coding 0.769 36.541 1.023

95.35 1.275 35.894 0.942 94.001
ENSMUSG00000029869 Ephb6 protein_coding 0.955 49.621 1.006
96.268 1.042 58.915 0.906 86.699
ENSMUSG00000029878 Dbpht2 protein_coding 1 66 1
93.469 1 77.704 1 85.577
ENSMUSG00000029882 2210010C04Rik protein_coding 1 68.16 1
95.211 1 73.096 1 87.7
ENSMUSG00000029883 1700074P13Rik protein_coding 1 85.857
1 95.708 1 87.274 1 95.74
ENSMUSG00000029885 Moxd2 protein_coding 1 84.397 1
96.216 1 88.601 1 95.273
ENSMUSG00000029909 Prss37 protein_coding 1 75.567 1
95.504 1 84.724 1 96.086
ENSMUSG00000029910 Mad2l1 protein_coding 1.068 45.307 1
95.301 0.939 51.055 1.28 52.324
ENSMUSG00000029911 Ssbp1 protein_coding 0.998 77.615 0.942
81.731 1.06 24.985 1.046 46.412
ENSMUSG00000029913 Prdm5 protein_coding 1.144 13.557 0.995
92.474 0.881 16.949 1.132 70.36
ENSMUSG00000029915 Clec5a protein_coding 1.527 14.205
1.216 82.904 0.54 1.871 1.404 46.846
ENSMUSG00000029916 Agk protein_coding 0.956 49.944 1.067
82.904 0.981 67.962 1.061 53.923
ENSMUSG00000029917 C130060K24Rik protein_coding 1 85.77 1
95.708 1 87.744 1 92.748
ENSMUSG00000029918 Mrps33 protein_coding 0.994 79.165
0.991 96.897 1.009 82.436 0.918 91.291
ENSMUSG00000029919 Hpgds protein_coding 1.682 0 NA NA
0.603 0.879 0.926 93.264
ENSMUSG00000029920 Smarcad1 protein_coding 1.045 70.36 0.958
88.705 0.999 87.686 0.992 94.19
ENSMUSG00000029922 Mkrn1 protein_coding 1.039 59.527 0.89
13.578 1.078 17.662 0.99 91.985
ENSMUSG00000029923 Rab19 protein_coding 1.073 37.091 0.957
72.576 0.971 83.275 0.921 92.561
ENSMUSG00000029924 Slc37a3 protein_coding 1.103 0.461 1.008
90.29 0.901 3.428 1.011 94.904
ENSMUSG00000029925 Tbxas1 protein_coding 1.457 0 0.848
60.678 0.807 5.884 1.01 90.177
ENSMUSG00000029992 Gfpt1 protein_coding 1.103 5.313 0.959 85.075
0.944 6.858 1.018 92.018
ENSMUSG00000029993 Nfu1 protein_coding 0.928 1.379 1.072 72.546
1.008 82.161 1.063 63.114
ENSMUSG00000029994 Anxa4 protein_coding 1.039 83.263 0.942
68.115 1.019 30.107 1.077 31.133
ENSMUSG00000029998 Pcyox1 protein_coding 1.067 11.044
0.952 84.017 0.985 39.245 1.039 93.016
ENSMUSG00000029999 Tgfa protein_coding 1.013 78.054 0.943
94.48 1.047 81.525 1.066 96.183
ENSMUSG00000030001 Figla protein_coding 1 86.474 1
95.301 1 74.179 1 94.904
ENSMUSG00000030002 Dusp11 protein_coding 0.906 8.267 1.082
90.952 1.021 61.418 0.979 84.29
ENSMUSG00000030004 Nat8 protein_coding 0.825 71.674 2.206

79.579 0.548 22.864 0.734 85.493
ENSMUSG00000030007 Cct7 protein_coding 0.926 18.907 1.017
95.088 1.059 17.751 1.091 50.452
ENSMUSG00000030008 Pradc1 protein_coding 1.076 29.596
1.105 86.793 0.843 22.009 0.929 86.991
ENSMUSG00000030016 Zfml protein_coding 1.06 62.689 NA NA
0.894 88.349 0.97 92.018
ENSMUSG00000030017 Reg3g protein_coding 3.575 6.541 0.348 67.513
0.802 58.775 0.8 96.287
ENSMUSG00000030019 Fbxl14 protein_coding 1.01 81.178
1.117 72.546 0.888 8.626 0.978 86.991
ENSMUSG00000030020 Prickle2 protein_coding 1.068 34.861 1.02
93.654 0.919 46.575 0.978 93.612
ENSMUSG00000030022 Adamts9 protein_coding 0.915 23.319
0.923 95.504 1.184 53.134 1.035 87.088
ENSMUSG00000030029 Lrig1 protein_coding 1.052 54.44 0.93 85.671
NA NA 1.014 93.045
ENSMUSG00000030030 1700003E16Rik protein_coding 0.999 66.406
1.158 91.584 0.865 82.685 0.718 85.493
ENSMUSG00000030031 Kbtbd8 protein_coding 0.992 62.888
1.126 85.756 0.891 68.584 0.982 68.502
ENSMUSG00000030032 Wdr54 protein_coding 0.713 5.204 1.158 83.104
1.212 15.241 0.854 84.29
ENSMUSG00000030034 Ino80b protein_coding 0.856 30.136
1.087 83.104 1.072 74.082 0.945 84.29
ENSMUSG00000030035 Wbp1 protein_coding 0.874 13.45 1.026 96.847
1.116 21.522 0.972 87.992
ENSMUSG00000030036 Mogs protein_coding 0.971 68.66 0.989 95.088
NA NA 1.036 92.389
ENSMUSG00000030037 Mrpl53 protein_coding 1 83.09 1
96.138 1 86.112 1 96.287
ENSMUSG00000030041 M1ap protein_coding 1.156 30.497 1.126
93.654 0.768 42.621 0.939 87.705
ENSMUSG00000030042 Pole4 protein_coding 0.894 0.987 1.038 83.104
1.079 13.956 1.028 93.261
ENSMUSG00000030043 Tacr1 protein_coding 1.233 20.799 1.296
82.904 0.627 8.634 1.194 89.498
ENSMUSG00000030045 Mrpl19 protein_coding 0.988 69.943 0.98
79.475 1.033 27.533 1.068 55.485
ENSMUSG00000030046 Bmp10 protein_coding 1 86.145 1
96.216 1 87.336 1 95.72
ENSMUSG00000030047 Arhgap25 protein_coding 1.068 39.416
0.916 70.29 1.019 85.935 1.006 96.086
ENSMUSG00000030048 Gkn3 protein_coding 1.691 6.23 1.057 90.092
0.56 3.718 1.04 95.74
ENSMUSG00000030049 Gkn2 protein_coding 1 80.608 1
86.728 1 59.612 1 95.72
ENSMUSG00000030050 Gkn1 protein_coding 1 81.178 NA NA
1 87.786 1 96.287
ENSMUSG00000030051 Aplf protein_coding 1.108 60.168 0.881
70.693 1.026 70.328 0.934 93.722
ENSMUSG00000030055 Rab43 protein_coding 1.153 1.079 0.963 71.41
0.901 23.938 0.967 86.445
ENSMUSG00000030056 Isy1 protein_coding 1.102 10.595 0.944

65.165 0.963 60.96 1.09 31.133
ENSMUSG00000030057 Cnbp protein_coding 1.041 35.499 0.944
53.434 1.019 53.882 1.008 91.928
ENSMUSG00000030058 Copg1 protein_coding 0.956 20.116 1.055
60.309 0.993 50.523 1.139 46.846
ENSMUSG00000030059 Tmf1 protein_coding 0.983 85.391 0.992
96.68 1.026 77.294 1.052 76.697
ENSMUSG00000030060 Hmces protein_coding 0.961 86.918 1.142
60.309 0.907 1.92 1.061 85.629
ENSMUSG00000030061 Uba3 protein_coding 0.979 71.674 1.028
90.29 NA NA 1.007 80.187
ENSMUSG00000030062 Rpn1 protein_coding 1.027 77.291 1.025
85.985 0.951 50.523 1.144 63.708
ENSMUSG00000030064 Frmd4b protein_coding 1.259 6.958 0.85
62.025 0.935 73.096 1.18 59.208
ENSMUSG00000030067 Foxp1 protein_coding 0.941 79.165 0.992
92.137 1.068 49.601 0.949 83.724
ENSMUSG00000030068 Gm20696 protein_coding 0.231 27.836
0.896 97.288 4.841 28.979 5.886 55.054
ENSMUSG00000030069 Prok2 protein_coding 1 66.76 1 97.155
1 67.339 1 93.045
ENSMUSG00000030074 Gxylt2 protein_coding 1.841 7.397 0.735
66.796 0.741 33.971 0.899 91.078
ENSMUSG00000030077 Chl1 protein_coding 1.136 20.444 0.886
89.996 0.988 49.967 0.934 86.991
ENSMUSG00000030079 Ruvbl1 protein_coding 0.968 86.918
0.974 81.731 1.062 55.109 1.058 72.116
ENSMUSG00000030082 Sec61a1 protein_coding 1.087 7.501 1.038
79.261 0.887 1.58 1.052 76.467
ENSMUSG00000030083 Abtb1 protein_coding 0.856 3.848 0.957 87.322
1.217 7.024 0.943 88.245
ENSMUSG00000030084 Plxna1 protein_coding 1.069 19.132
1.101 90.116 0.85 30.024 1.043 82.66
ENSMUSG00000030086 Chchd6 protein_coding 0.846 0.987 1.003
90.092 1.175 7.365 1.027 95.72
ENSMUSG00000030087 Klf15 protein_coding 0.974 82.092 0.956
91.897 1.076 79.554 0.931 85.493
ENSMUSG00000030088 Aldh1l1 protein_coding 0.65 0.987 1.116
89.996 1.383 10.76 0.899 92.561
ENSMUSG00000030089 Slc41a3 protein_coding 1.452 7.397 0.96
94.48 0.717 7.329 1.131 83.067
ENSMUSG00000030091 Nup210 protein_coding 0.885 57.014 0.83
78.877 1.362 24.791 1.088 94.99
ENSMUSG00000030092 Cntn6 protein_coding 1.028 86.843 1.959
83.104 0.495 32.082 0.577 86.991
ENSMUSG00000030094 Xpc protein_coding 0.973 71.915 0.971
75.493 1.055 29.302 0.909 83.724
ENSMUSG00000030095 Tmem43 protein_coding 0.888 1.867 1.075
92.561 1.044 33.802 1.003 94.016
ENSMUSG00000030096 Slc6a6 protein_coding 1.243 0.461 0.846
88.897 0.949 45.799 0.984 87.218
ENSMUSG00000030098 Grip2 protein_coding 1.095 58.38 1.105 96.897
0.826 48.133 0.88 85.493
ENSMUSG00000030101 Sumf1 protein_coding 0.992 82.891 0.997

85.921 1.011 67.163 1.023 71.653
ENSMUSG00000030102 Itpr1 protein_coding 1.123 14.59 0.912 45.262
0.974 69.445 0.987 91.985
ENSMUSG00000030103 Bhlhe40 protein_coding 0.897 55.303 1.09
82.904 1.024 81.388 0.945 95.559
ENSMUSG00000030104 Edem1 protein_coding 1.031 22.633 1.023
83.104 0.95 9.573 1.011 94.468
ENSMUSG00000030105 Arl8b protein_coding 1.061 5.253 1.032 90.952
0.912 1.58 0.996 95.593
ENSMUSG00000030107 Usp18 protein_coding 1.047 44.334 1.144
96.378 0.834 33.402 1.355 62.594
ENSMUSG00000030108 Slc6a13 protein_coding 0.872 64.589
1.314 61.032 0.874 68.266 1.158 80.324
ENSMUSG00000030109 Slc6a12 protein_coding 0.892 44.562
1.082 82.904 1.036 87.744 0.927 95.72
ENSMUSG00000030110 Ret protein_coding 0.813 23.13 1.129 88.092
1.09 67.163 1.084 95.273
ENSMUSG00000030111 A2m protein_coding 3.912 29.057 0.23
79.475 1.114 81.388 1.156 96.287
ENSMUSG00000030114 Klrg1 protein_coding 0.344 12.624 1.671
71.617 1.734 76.923 1.512 92.018
ENSMUSG00000030116 Mfap5 protein_coding 1.002 81.24 0.979 72.576
1.015 38.975 1.097 66.944
ENSMUSG00000030117 Gdf3 protein_coding 1.876 8.281 1.175 89.996
0.453 5.884 0.971 94.904
ENSMUSG00000030120 Mlf2 protein_coding 0.857 2.009 1.142 72.642
1.023 52.432 0.971 95.559
ENSMUSG00000030122 Ptms protein_coding 0.875 30.637 1.102
79.411 1.034 71.615 0.992 92.12
ENSMUSG00000030123 Plxnd1 protein_coding 1.073 77.497 1.09
87.029 0.85 22.735 0.925 84.19
ENSMUSG00000030124 Lag3 protein_coding 0.945 61.043 1.103
87.609 0.956 84.961 0.919 91.372
ENSMUSG00000030125 Lrrc23 protein_coding 1.065 68.727
0.859 67.165 1.094 57.907 0.892 96.183
ENSMUSG00000030126 Tmcc1 protein_coding 1.123 14.194 0.986
94.96 0.9 14.583 0.981 92.181
ENSMUSG00000030127 Cops7a protein_coding 0.984 21.393 0.98
93.994 1.039 55.352 1.053 42.736
ENSMUSG00000030131 Mug2 protein_coding 1 70.862 NA NA
1 85.352 1 94.654
ENSMUSG00000030134 Rasgef1a protein_coding 1.019 70.36 0.953
90.053 1.027 84.626 0.866 84.29
ENSMUSG00000030137 Tuba8 protein_coding 1.174 47.174 1.016
96.652 0.835 51.443 0.79 94.99
ENSMUSG00000030138 Bms1 protein_coding 0.945 23.641 0.996
95.088 1.058 41.331 1.095 31.133
ENSMUSG00000030142 Clec4e protein_coding 1.759 8.685 0.703
62.692 0.807 54.037 0.394 83.724
ENSMUSG00000030143 Gm8882 protein_coding 1 71.36 1
96.216 1 88.693 1 95.559
ENSMUSG00000030144 Clec4d protein_coding 1.15 54.44 0.832
94.887 1.047 78.642 0.979 96.184
ENSMUSG00000030145 Zfp248 protein_coding 0.944 85.355

1.139 90.116 0.93 64.155 1.009 87.447
ENSMUSG00000030147 Clec4b1 protein_coding 1.469 8.477 1.021
95.196 0.667 8.313 0.989 92.762
ENSMUSG00000030148 Clec4a2 protein_coding 1.506 0 0.835
67.739 0.793 8.192 1.127 91.525
ENSMUSG00000030149 Klrk1 protein_coding 0.87 74.021 1.378
60.309 0.837 6.858 0.907 84.29
ENSMUSG00000030154 Klrb1f protein_coding 1.142 73.841
1.171 96.138 0.749 72.464 0.651 83.724
ENSMUSG00000030155 Clec2e protein_coding 0.775 27.431
0.601 60.678 2.139 11.329 1.601 49.514
ENSMUSG00000030156 Cd69 protein_coding 1.86 14.205 0.765
91.955 0.705 42.376 0.691 84.19
ENSMUSG00000030157 Clec2d protein_coding 1.061 66.58 0.959
93.654 0.983 81.169 0.96 95.593
ENSMUSG00000030158 Clec12b protein_coding 0.922 82.092
1.026 90.744 1.058 76.225 1.093 96.086
ENSMUSG00000030159 Clec1b protein_coding 1.973 21.836
0.378 59.946 1.334 81.492 1.035 96.358
ENSMUSG00000030160 Tmem52b protein_coding 1 82.231 1
95.708 1 86.258 1 92.181
ENSMUSG00000030161 Gabarapl1 protein_coding 0.961 75.115
0.988 88.881 1.056 60.789 0.927 83.724
ENSMUSG00000030162 Olr1 protein_coding 1.683 70.36 0.464 90.712
1.282 87.786 1.14 93.627
ENSMUSG00000030165 Klrd1 protein_coding 1.08 67.991 1.152
86.387 0.804 35.264 0.802 83.724
ENSMUSG00000030166 Rad52 protein_coding 0.99 83.263 0.993
91.361 1.014 56.708 0.97 95.877
ENSMUSG00000030167 Klrc1 protein_coding 1.504 39.295 0.973
96.013 0.682 29.765 0.768 85.493
ENSMUSG00000030168 Adipor2 protein_coding 0.948 80.608
0.999 93.654 1.054 86.258 1.027 95.74
ENSMUSG00000030170 Wnt5b protein_coding 1.006 76.827 0.97
95.708 1.02 87.786 0.847 83.724
ENSMUSG00000030172 Erc1 protein_coding 1.008 80.688 1.022
83.104 0.973 47.641 1.074 31.133
ENSMUSG00000030177 Ccdc77 protein_coding 0.903 4.115 0.979
96.407 1.133 9.176 0.979 94.904
ENSMUSG00000030180 Kdm5a protein_coding 0.923 8.122 0.964 95.504
1.125 10.76 0.964 85.493
ENSMUSG00000030187 Klra2 protein_coding 1.118 51.826 0.917
79.475 0.972 80.163 1.205 95.273
ENSMUSG00000030188 Magohb protein_coding 1.065 39.295
0.914 75.052 1.029 86.084 1.142 91.783
ENSMUSG00000030189 Ybx3 protein_coding 0.965 79.433 1.017
97.129 1.016 84.308 0.965 95.74
ENSMUSG00000030194 Tas2r116 protein_coding 1 84.263 1
95.504 1 85.134 1 93.156
ENSMUSG00000030196 Tas2r103 protein_coding 1 84.324 1
95.431 1 85.757 1 96.358
ENSMUSG00000030199 Etv6 protein_coding 1.032 45.096 0.997
97.155 0.967 49.967 1.057 87.7
ENSMUSG00000030200 Bcl2l14 protein_coding 1.618 0.976 0.85

62.322 0.728 9.456 0.925 86.699
ENSMUSG00000030201 Lrp6 protein_coding 1.035 58.896 0.918
73.766 1.051 50.636 0.993 86.699
ENSMUSG00000030203 Dusp16 protein_coding 0.955 40.217
0.997 97.049 1.046 65.567 0.977 94.654
ENSMUSG00000030204 Ddx47 protein_coding 1.044 13.8 0.95 62.025
1.005 62.237 0.976 86.254
ENSMUSG00000030205 Gprc5d protein_coding 1.016 83.169
0.597 84.594 1.645 77.17 1.464 93.156
ENSMUSG00000030206 Gsg1 protein_coding 0.755 76.135 1.294
93.765 1.023 79.119 2.031 69.918
ENSMUSG00000030207 8430419L09Rik protein_coding 1.078 20.799
NA NA 0.967 20.511 0.929 91.217
ENSMUSG00000030208 Emp1 protein_coding 1.48 2.723 0.889 94.597
0.758 15.904 1.431 40.426
ENSMUSG00000030209 Grin2b protein_coding 0.837 51.447
1.102 95.196 1.084 69.002 0.756 83.724
ENSMUSG00000030213 Atf7ip protein_coding 1.081 29.596
1.039 82.904 0.886 7.742 0.981 84.29
ENSMUSG00000030214 Plbd1 protein_coding 1.1 59.806 0.997
97.155 0.912 44.211 0.985 93.156
ENSMUSG00000030216 Wbp11 protein_coding 1.048 8.537 1.011 95.431
0.941 4.23 1.011 93.413
ENSMUSG00000030217 Art4 protein_coding 1.008 83.263 0.897
88.345 1.108 43.842 NA NA
ENSMUSG00000030218 Mgp protein_coding 1.038 81.76 0.934 61.883
1.031 34.403 0.914 93.964
ENSMUSG00000030219 Erp27 protein_coding 1.662 64.714 0.906
88.345 0.661 83.925 0.831 96.358
ENSMUSG00000030220 Arhgdib protein_coding 1.408 0 0.875
81.443 0.812 1.705 1.241 54.265
ENSMUSG00000030222 Rerg protein_coding 1.132 36.438 0.962
88.704 0.92 82.685 0.982 91.078
ENSMUSG00000030223 Ptpro protein_coding 1.556 0 1.023 93.469
0.627 7.339 0.876 95.593
ENSMUSG00000030224 Strap protein_coding 1.041 20.296 1.014
91.275 0.948 51.055 0.986 92.691
ENSMUSG00000030225 Dera protein_coding 0.855 10.739 NA NA
1.147 13.259 1.116 67.169
ENSMUSG00000030226 Lmo3 protein_coding 0.854 84.397 3.017
83.104 0.387 58.271 1.665 83.067
ENSMUSG00000030228 Pik3c2g protein_coding 1.206 72.27 0.976
96.68 0.849 66.425 0.673 83.724
ENSMUSG00000030230 Plcz1 protein_coding 1.322 80.408 1.316
88.136 0.575 59.726 0.743 96.139
ENSMUSG00000030231 Plekha5 protein_coding 0.94 58.077
0.988 94.96 1.076 39.439 1.009 93.685
ENSMUSG00000030232 Aebp2 protein_coding 1.109 6.399 0.989 93.817
0.91 12.41 0.997 87.331
ENSMUSG00000030235 Slco1c1 protein_coding 0.731 49.368
2.342 79.411 0.585 36.499 0.68 84.29
ENSMUSG00000030243 Recql protein_coding 0.913 21.957 1.104
71.98 0.989 76.768 1.107 60.466
ENSMUSG00000030244 Gys2 protein_coding 0.514 0.987 1.246 75.108

1.557 18.208 1.235 76.467
ENSMUSG00000030245 Golt1b protein_coding 1.102 5.559 0.988
95.504 0.917 9.901 1.149 49.514
ENSMUSG00000030246 Ldhb protein_coding 0.954 50.229 1.038
82.904 1.009 88.349 1.05 93.627
ENSMUSG00000030247 Kcnj8 protein_coding 0.872 58.264 0.943
96.843 1.21 88.601 0.951 84.29
ENSMUSG00000030249 Abcc9 protein_coding 1.1 22.349 1.224
82.904 0.743 7.742 1.064 90.125
ENSMUSG00000030254 Rad18 protein_coding 1.092 20.032 NA NA
0.849 20.009 0.935 86.699
ENSMUSG00000030255 Sspn protein_coding 1.053 28.395 1.058
79.055 0.899 8.907 1.069 78.379
ENSMUSG00000030256 Bhlhe41 protein_coding 1.005 57.949 1.05
94.848 0.949 57.446 0.906 83.724
ENSMUSG00000030257 Srgap3 protein_coding 1.498 1.323 0.921
93.908 0.726 1.898 0.759 83.724
ENSMUSG00000030259 Rassf8 protein_coding 1.034 73.124
1.144 91.584 0.846 25.345 0.956 93.685
ENSMUSG00000030263 Lrmp protein_coding 1.375 0.2 0.888 87.553
0.815 23.938 1.132 53.923
ENSMUSG00000030264 Thumpd3 protein_coding 0.999 83.169
1.009 96.847 0.992 86.666 1.041 76.697
ENSMUSG00000030265 Kras protein_coding 1.145 13.734 0.964
82.671 0.904 36.786 1.045 72.204
ENSMUSG00000030268 Bcat1 protein_coding 1.187 33.325 1.029
94.96 0.817 44.019 1.257 31.133
ENSMUSG00000030269 Mtmr14 protein_coding 0.91 16.02 0.982
95.35 1.12 40.575 1.011 87.447
ENSMUSG00000030270 Cpne9 protein_coding 0.543 48.939 1.066
96.843 1.722 45.449 1.282 93.016
ENSMUSG00000030271 Ogg1 protein_coding 0.941 58.802 1.022
96.847 1.04 66.043 1.008 95.676
ENSMUSG00000030272 Camk1 protein_coding 1.166 6.61 1.023 92.561
0.84 1.58 1.075 52.366
ENSMUSG00000030275 Etnk1 protein_coding 1.033 43.654 0.965
95.504 1.004 55.214 1.021 79.545
ENSMUSG00000030276 Ttll3 protein_coding 0.879 12.181 1.118
96.407 1.015 57.227 0.951 96.183
ENSMUSG00000030278 Cidec protein_coding 0.867 15.456 0.921
94.848 1.25 19.609 0.986 96.183
ENSMUSG00000030279 C2cd5 protein_coding 1.02 57.133 1.005
92.404 0.979 82.685 1.002 93.316
ENSMUSG00000030281 Il17rc protein_coding 0.872 4.099 1.019
96.308 1.122 20.051 1.022 81.251
ENSMUSG00000030282 Cmas protein_coding 0.983 59.674 0.939
40.463 1.082 9.567 1.017 79.545
ENSMUSG00000030283 St8sia1 protein_coding 1.73 13.8 0.581
82.848 0.99 49.967 1.29 94.904
ENSMUSG00000030284 Creld1 protein_coding 0.761 0.987 1.011
96.268 1.301 5.864 1.058 72.616
ENSMUSG00000030286 Emc3 protein_coding 1.008 86.145 0.995
85.921 0.998 48.772 1.01 87.7
ENSMUSG00000030287 Itpr2 protein_coding 0.967 44.467 0.981

95.708 1.055 47.935 0.918 83.724
ENSMUSG00000030291 Med21 protein_coding 1.073 52.799 0.995
95.196 0.939 30.468 1.039 79.442
ENSMUSG00000030292 Smco2 protein_coding 1 83.968 1
95.809 1 86.602 1 93.156
ENSMUSG00000030298 Sec13 protein_coding 0.881 4.468 1.077 78.705
1.049 34.807 1.214 49.769
ENSMUSG00000030301 Ccdc91 protein_coding 0.933 39.953
0.995 91.361 1.075 24.375 1.042 61.316
ENSMUSG00000030302 Atp2b2 protein_coding 0.878 35.449
1.136 96.335 1.005 69.728 1.292 57.075
ENSMUSG00000030303 Far2 protein_coding 1.199 25.026 NA NA
0.775 35.264 1.283 72.369
ENSMUSG00000030304 Ergic2 protein_coding 1.046 28.927
0.975 93.654 0.975 59.726 1.05 72.116
ENSMUSG00000030306 Tmtc1 protein_coding 1.036 44.334 0.832
82.848 1.163 80.381 1.011 93.734
ENSMUSG00000030309 Caprin2 protein_coding 1.215 12.295
0.935 96.013 0.881 8.626 0.917 86.991
ENSMUSG00000030310 Slc6a1 protein_coding 1.236 85.832
0.899 92.137 NA NA 0.833 91.807
ENSMUSG00000030313 Dennd5b protein_coding 1.077 35.499
0.939 68.115 NA NA 0.974 86.991
ENSMUSG00000030314 Atg7 protein_coding 1.017 47.466 1.011
86.793 0.974 21.117 1.029 79.713
ENSMUSG00000030315 Vgll4 protein_coding 1.154 19.132 0.99
95.431 0.875 7.509 0.888 83.724
ENSMUSG00000030316 Tamm41 protein_coding 1.026 35.499
0.989 97.263 0.984 56.625 1.048 93.734
ENSMUSG00000030317 Timp4 protein_coding 0.775 18.379 1.119
95.735 1.154 33.508 0.965 96.287
ENSMUSG00000030319 Cand2 protein_coding 1.31 1.323 0.924 88.691
0.823 1.48 0.857 92.31
ENSMUSG00000030321 Efcab12 protein_coding 0.874 29.725
0.877 91.532 1.306 30.948 0.792 86.699
ENSMUSG00000030322 Mbd4 protein_coding 1.118 16.74 1.09 96.216
0.823 24.163 1.021 96.183
ENSMUSG00000030323 Ift122 protein_coding 1.041 57.949
1.004 97.263 0.959 65.409 0.86 83.724
ENSMUSG00000030324 Rho protein_coding 0.701 62.437 0.93
92.355 1.526 51.243 1.461 93.016
ENSMUSG00000030325 Klrb1c protein_coding 1.04 59.806
0.997 92.111 0.96 44.019 0.927 87.331
ENSMUSG00000030327 Necap1 protein_coding 1.058 16.539
0.961 63.619 0.987 80.5 1.025 86.133
ENSMUSG00000030329 Pianp protein_coding 1.286 21.703 0.929
90.256 0.834 71.999 0.752 88.505
ENSMUSG00000030330 Ing4 protein_coding 0.92 5.686 0.98 96.216
1.111 13.082 0.909 83.724
ENSMUSG00000030335 Mrpl51 protein_coding 0.929 32.557
1.037 96.308 1.041 63.801 0.992 96.183
ENSMUSG00000030336 Cd27 protein_coding 0.797 25.111 1.093
90.389 1.146 41.806 0.728 84.29
ENSMUSG00000030337 Vamp1 protein_coding 0.974 40.422 1.038

87.609 0.988 84.308 0.939 83.724
ENSMUSG00000030339 Ltbr protein_coding 0.944 15.76 1.08 79.579
0.979 88.203 0.987 96.071
ENSMUSG00000030340 Scnn1a protein_coding 0.76 10.514
0.949 94.48 1.378 11.518 0.85 83.724
ENSMUSG00000030341 Tnfrsf1a protein_coding 1.046 61.49 1.054
67.843 0.908 7.809 1.1 64.513
ENSMUSG00000030342 Cd9 protein_coding 1.09 35.748 0.929
60.806 NA NA 0.994 91.928
ENSMUSG00000030344 Akap3 protein_coding 0.75 81.01 1.143 96.847
1.166 84.724 1.212 95.676
ENSMUSG00000030346 Rad51ap1 protein_coding 1.592 19.893
0.819 82.671 0.766 67.962 1.371 60.968
ENSMUSG00000030347 D6Wsu163e protein_coding 1.211 1.292 0.901
65.819 0.917 13.643 1.079 60.31
ENSMUSG00000030351 Tspan11 protein_coding 1.436 1.441 1.147
83.104 0.608 0.72 0.927 95.72
ENSMUSG00000030352 Tspan9 protein_coding 1.03 86.199
0.915 53.434 1.061 34.807 0.947 92.181
ENSMUSG00000030353 Tead4 protein_coding 0.853 11.128 0.952
95.211 1.229 30.292 1.006 91.625
ENSMUSG00000030357 Fkbp4 protein_coding 0.781 6.484 1.126 94.89
1.133 15.049 1.045 68.696
ENSMUSG00000030361 Klrb1a protein_coding 0.944 67.163
1.243 93.27 NA NA 1.301 80.324
ENSMUSG00000030363 Clec2j protein_coding 1 85.655 1
91.955 1 86.112 1 95.832
ENSMUSG00000030365 Clec2i protein_coding 1.872 6.958 0.645
35.072 0.827 74.853 0.79 91.217
ENSMUSG00000030368 Ceacam11 protein_coding 1 84.324 1
95.708 NA NA 1 96.183
ENSMUSG00000030373 Psg28 protein_coding 1 77.916 1
97.263 1 84.724 1 95.559
ENSMUSG00000030374 Strn4 protein_coding 0.959 11.232 1.012
88.551 1.032 38.26 1.085 31.133
ENSMUSG00000030376 Slc8a2 protein_coding 1.081 86.351
1.132 82.904 0.818 25.625 0.861 86.991
ENSMUSG00000030378 2810007J24Rik protein_coding 1 73.124
1 91.661 1 88.729 1 95.676
ENSMUSG00000030380 Mzf1 protein_coding 0.931 56.477 0.965
96.843 1.109 60.104 0.906 94.468
ENSMUSG00000030382 Slc27a5 protein_coding 0.853 84.263 0.92
91.955 1.274 69.825 0.999 94.904
ENSMUSG00000030385 2900092C05Rik protein_coding 1 65.622
NA NA 1 76.754 1 94.99
ENSMUSG00000030386 Zfp606 protein_coding 1.016 74.147
1.008 95.708 0.976 85.675 0.9 83.724
ENSMUSG00000030393 Zik1 protein_coding 1.088 60.435 0.872
92.474 NA NA 1.144 80.187
ENSMUSG00000030397 Mark4 protein_coding 1.005 84.263 1.05
83.819 0.948 66.753 0.966 86.699
ENSMUSG00000030399 Ckm protein_coding 8.911 5.481 1.006 83.104
0.112 0.72 0.866 94.654
ENSMUSG00000030400 Ercc2 protein_coding 0.892 4.115 1.028 82.904

							1.091	25.966	0.953	86.699
ENSMUSG00000030401	Rtn2	protein_coding	1.306	12.396	1.086	90.29	0.703	3.428	1.005	95.74
ENSMUSG00000030403	Vasp	protein_coding	0.994	78.299	0.979	95.211	1.026	86.831	0.985	95.405
ENSMUSG00000030406	Gipr	protein_coding	0.847	12.309	1.149	82.809	1.026	74.082	0.898	89.892
ENSMUSG00000030407	Qpctl	protein_coding	1.014	84.534	1.068	79.29	0.922	42.376	1.009	81.061
ENSMUSG00000030409	Dmpk	protein_coding	0.677	0.987	1.288	60.309	1.149	16.666	1.119	38.598
ENSMUSG00000030410	Dmwd	protein_coding	0.858	3.231	1.127	74.663	1.036	42.199	0.975	93.612
ENSMUSG00000030411	Nova2	protein_coding	0.775	1.422	1.131	79.29	1.142	34.285	0.94	88.552
ENSMUSG00000030413	Pglyrp1	protein_coding	0.845	66.652	1.207	95.196	0.979	86.144	0.845	84.29
ENSMUSG00000030417	Pdcd5	protein_coding	0.951	19.023	0.961	82.503	1.096	23.41	1.062	50.452
ENSMUSG00000030421	Uri1	protein_coding	1.042	30.282	0.969	85.418	0.989	63.919	0.971	86.991
ENSMUSG00000030423	Pop4	protein_coding	0.957	41.33	0.913	90.053	1.14	27.475	1.102	65.401
ENSMUSG00000030424	Zfp939	protein_coding	1.103	40.333	0.887	72.576	1.021	81.62	1.053	93.132
ENSMUSG00000030427	Lilra6	protein_coding	1.257	47.466	1.04	79.807	0.764	84.896	1.066	91.807
ENSMUSG00000030428	Ttyh1	protein_coding	1.083	42.134	1.182	82.904	0.777	15.63	0.915	92.561
ENSMUSG00000030431	Tmem238	protein_coding	0.788	13.239	1.312	77.085	0.971	81.032	0.931	86.699
ENSMUSG00000030432	Rpl28	protein_coding	0.986	76.396	1.065	97.155	0.952	87.431	0.976	96.358
ENSMUSG00000030433	Sbk2	protein_coding	1.071	63.126	1.053	95.708	0.889	59.383	0.873	90.691
ENSMUSG00000030435	U2af2	protein_coding	0.944	19.83	0.998	97.155	1.061	32.524	1.041	84.31
ENSMUSG00000030443	Zfp583	protein_coding	1.354	2.361	0.827	75.493	0.895	26.057	0.937	95.877
ENSMUSG00000030446	Zfp273	protein_coding	1.153	43.654	0.932	92.631	0.927	52.972	1.04	91.492
ENSMUSG00000030447	Cyfip1	protein_coding	0.986	66.406	1.008	94.848	1.007	63.801	1.106	31.133
ENSMUSG00000030450	Oca2	protein_coding	1	80.688	1	97.097	1	86.666	1	90.157
ENSMUSG00000030451	Herc2	protein_coding	0.938	19.595	0.877	50.664	1.218	5.864	1.026	93.413
ENSMUSG00000030452	Nipa2	protein_coding	1.104	3.893	0.987	83.58	0.915	9.573	0.986	95.74
ENSMUSG00000030463	4933421I07Rik	protein_coding	1	75.399	1	96.847	1	87.971	1	95.273
ENSMUSG00000030465	Psd3	protein_coding	1.051	55.124	0.95	84.017	NA	NA	1.007	87.7
ENSMUSG00000030468	Siglecg	protein_coding	0.982	63.804						

1.067 95.088 0.952 80.5 1.194 90.177
ENSMUSG00000030469 Zfp719 protein_coding 1.078 51.297
0.896 95.211 1.032 85.134 1.01 83.581
ENSMUSG00000030471 Zdhhc13 protein_coding 0.896 62.158
1.044 83.104 1.069 84.552 0.861 83.952
ENSMUSG00000030472 Ceacam18 protein_coding 1 73.292 1
96.378 NA NA 1 92.762
ENSMUSG00000030474 Siglece protein_coding 1.052 59.527
1.299 79.261 0.73 17.662 0.834 91.105
ENSMUSG00000030483 Cyp2b10 protein_coding 0.867 76.396
1.099 95.211 1.051 80.634 0.975 92.851
ENSMUSG00000030484 Lypd5 protein_coding 1 85.728 1
93.27 1 81.976 1 94.215
ENSMUSG00000030486 Zfp108 protein_coding 0.827 23.13 1.159
87.029 1.038 75.155 1.129 78.042
ENSMUSG00000030492 Slc7a9 protein_coding 1 85.728 NA
NA 1 83.275 1 94.016
ENSMUSG00000030493 C230052I12Rik protein_coding 1.138 8.477
0.934 73.766 0.943 71.999 1.063 90.865
ENSMUSG00000030494 Rhpn2 protein_coding 0.867 49.047 0.984
90.712 1.167 34.403 1.091 93.685
ENSMUSG00000030495 Slc7a10 protein_coding 0.928 64.284
1.056 94.597 1.023 63.801 0.902 83.724
ENSMUSG00000030498 Gas2 protein_coding 1.073 32.873 1
96.407 0.928 28.669 0.929 84.29
ENSMUSG00000030499 Kctd15 protein_coding 1.074 25.421
0.944 91.361 0.986 56.625 0.989 90.611
ENSMUSG00000030500 Slc17a6 protein_coding 1 80.751 1
88.897 1 87.37 1 95.777
ENSMUSG00000030505 Prmt3 protein_coding 0.998 65.733 0.903
78.471 1.107 61.171 1.089 86.301
ENSMUSG00000030507 Dbx1 protein_coding 1 82.092 1
96.216 1 88.693 1 95.35
ENSMUSG00000030509 Asb7 protein_coding 0.967 15.227 0.981
95.735 1.049 36.578 1.023 86.133
ENSMUSG00000030510 Cers3 protein_coding 0.781 72.985 0.544
74.851 2.359 35.894 0.89 91.078
ENSMUSG00000030512 Snrpa1 protein_coding 0.992 57.014
0.932 72.399 1.081 18.208 1.087 37.634
ENSMUSG00000030513 Pcsk6 protein_coding 0.872 20.369 0.998
95.708 1.153 31.761 1.025 89.152
ENSMUSG00000030515 Tarsl2 protein_coding 1.202 20.799
1.016 92.111 0.814 6.858 0.903 93.612
ENSMUSG00000030516 Tjp1 protein_coding 1.096 1.009 NA NA
0.907 2.294 0.964 83.724
ENSMUSG00000030518 Fam189a1 protein_coding 1.172 52.134
0.624 84.594 1.363 76.923 0.99 96.248
ENSMUSG00000030519 Apba2 protein_coding 1.233 74.958 1.524
96.216 0.531 42.997 1.07 92.762
ENSMUSG00000030521 Mphosph10 protein_coding 0.947 75.298
0.978 85.418 1.081 49.601 1.204 49.514
ENSMUSG00000030522 Mtmr10 protein_coding 1.042 54.44 0.919
64.123 1.043 65.567 0.979 86.699
ENSMUSG00000030523 Trpm1 protein_coding 1.819 25.735 1.367

91.83 0.403 48.851 0.932 95.92
ENSMUSG00000030525 Chrna7 protein_coding 1.541 82.732
1.534 94.887 0.424 86.985 0.666 92.181
ENSMUSG00000030527 Crtc3 protein_coding 1.027 65.284 1.075
80.14 0.906 9.456 0.927 84.29
ENSMUSG00000030528 Blm protein_coding 1.126 21.599 1.038
95.735 0.859 23.212 1.02 79.713
ENSMUSG00000030530 Furin protein_coding 1.039 58.896 1.047
90.092 0.921 27.86 0.999 91.372
ENSMUSG00000030532 Hddc3 protein_coding 1.087 69.777 1.014
86.156 0.909 32.297 0.91 92.31
ENSMUSG00000030533 Unc45a protein_coding 0.854 1.422 0.998
95.708 1.169 7.365 1.016 93.413
ENSMUSG00000030534 Vps33b protein_coding 0.92 17.276
1.006 96.948 1.077 17.662 1.024 87.886
ENSMUSG00000030536 Iqgap1 protein_coding 1.107 0.355 0.957
38.699 0.94 15.024 1.062 46.412
ENSMUSG00000030538 Cib1 protein_coding 0.869 24 1.056 83.104
1.085 64.024 1.078 70.624
ENSMUSG00000030539 Sema4b protein_coding 1.113 16.74 1.151
60.309 0.783 0.72 1.021 77.245
ENSMUSG00000030541 Idh2 protein_coding 1.024 48.288 1.027
85.378 0.949 6.858 1.058 61.984
ENSMUSG00000030543 Mesp2 protein_coding 1.004 84.263 1.283
95.504 0.775 85.464 0.843 95.273
ENSMUSG00000030545 Pex11a protein_coding 0.948 42.922 1.07
83.104 0.984 83.607 0.968 91.105
ENSMUSG00000030546 Plin1 protein_coding 0.883 5.491 1.091 64.304
1.034 80.015 0.962 90.611
ENSMUSG00000030549 Rhcg protein_coding 1 82.231 NA NA
1 87.103 1 95.832
ENSMUSG00000030551 Nr2f2 protein_coding 1.008 37.702 1.07
82.809 0.924 6.68 1.028 89.052
ENSMUSG00000030553 Pgpep1l protein_coding 0.91 60.168
0.722 73.766 1.518 62.995 1.648 88.657
ENSMUSG00000030554 Synm protein_coding 1.247 11.197 1.028
89.434 0.782 3.234 0.834 92.181
ENSMUSG00000030555 Ttc23 protein_coding 1.003 85.655 0.988
92.474 1.004 63.665 1.023 95.74
ENSMUSG00000030556 Lrrc28 protein_coding 1.02 77.916
1.043 95.211 0.939 63.919 0.932 84.29
ENSMUSG00000030557 Mef2a protein_coding 1.151 0 1 96.763
0.87 0.72 1.018 90.177
ENSMUSG00000030559 Rab38 protein_coding 1.328 44.694 0.807
72.399 0.936 86.258 0.896 95.593
ENSMUSG00000030560 Ctsc protein_coding 1.203 5.313 0.927 94.102
0.894 36.499 1.102 83.276
ENSMUSG00000030562 Nox4 protein_coding 1.135 38.195 0.737
28.822 1.192 12.831 0.681 83.724
ENSMUSG00000030577 Cd22 protein_coding 1.403 78.054 1.054
86.277 0.674 46.956 0.98 90.611
ENSMUSG00000030579 Tyrobp protein_coding 1.421 0 0.813
61.883 0.862 13.848 1.053 86.301
ENSMUSG00000030583 Sipa1l3 protein_coding 0.909 10.014

1.044 83.104 1.054 51.243 0.857 83.724
ENSMUSG00000030584 Dpf1 protein_coding 1.291 52.986 1.127 90.116 0.687 11.314 0.883 93.612
ENSMUSG00000030587 2200002D01Rik protein_coding 0.851 18.138 NA NA 1.152 33.802 0.953 85.493
ENSMUSG00000030588 Yif1b protein_coding 0.869 3.623 1.108 80.277 1.039 55.81 1.003 85.577
ENSMUSG00000030589 Rasgrp4 protein_coding 1.173 20.032 1.107 95.431 0.772 24.163 1.039 74.193
ENSMUSG00000030590 Fam98c protein_coding 0.923 31.333 0.987 96.216 1.101 40.955 0.97 86.991
ENSMUSG00000030591 Psmd8 protein_coding 0.946 14.526 1.066 74.663 0.993 80.089 1.019 77.245
ENSMUSG00000030592 Ryr1 protein_coding 5.545 7.255 1.105 89.434 0.163 0.72 0.879 94.608
ENSMUSG00000030595 Nfkbib protein_coding 0.826 1.727 1.092 81.039 1.107 31.479 1.027 92.564
ENSMUSG00000030598 Fbxo17 protein_coding 1.062 72.27 0.983 96.268 0.956 86.727 0.976 92.851
ENSMUSG00000030600 Lrfn1 protein_coding 0.989 83.263 1.08 96.763 0.933 86.144 0.863 88.387
ENSMUSG00000030602 Pak4 protein_coding 1.192 14.34 0.913 82.503 0.92 58.271 0.899 86.699
ENSMUSG00000030603 Psmc4 protein_coding 0.864 0.987 1.015 90.952 1.135 6.202 1.173 33.269
ENSMUSG00000030604 Zfp626 protein_coding 1.012 83.169 1.001 92.631 0.989 86.144 1.016 93.722
ENSMUSG00000030605 Mfge8 protein_coding 0.968 67.334 1.032 96.407 0.999 75.155 0.898 83.724
ENSMUSG00000030606 Hapln3 protein_coding 0.976 65.199 1.208 82.904 0.849 71.004 0.924 94.174
ENSMUSG00000030609 Aen protein_coding 0.927 85.092 0.973 93.765 1.102 86.475 1.247 57.839
ENSMUSG00000030610 Det1 protein_coding 1.039 53.562 NA NA 0.92 55.701 0.971 93.316
ENSMUSG00000030611 Mrps11 protein_coding 0.893 13.239 1.058 82.904 1.06 67.163 0.998 95.777
ENSMUSG00000030612 Mrpl46 protein_coding 0.95 20.659 0.938 94.102 1.118 22.994 0.999 94.904
ENSMUSG00000030613 Ccdc90b protein_coding 1.167 6.958 1.029 95.211 0.83 6.751 0.976 93.612
ENSMUSG00000030614 Tmem126b protein_coding 1.119 16.74 0.858 81.02 1.038 88.693 0.996 76.347
ENSMUSG00000030615 Tmem126a protein_coding 1.026 81.925 1.126 77.085 0.868 25.625 0.927 86.699
ENSMUSG00000030616 Sytl2 protein_coding 1.347 35.748 0.943 91.361 0.788 52.213 0.787 85.493
ENSMUSG00000030619 Eed protein_coding 1.031 47.847 NA NA 0.958 26.809 0.99 92.181
ENSMUSG00000030621 Me3 protein_coding 1.585 6.853 0.898 95.504 0.702 7.155 0.758 90.157
ENSMUSG00000030623 1700019G06Rik antisense 1.009 86.658 1.033 95.211 0.961 79.426 0.838 85.493
ENSMUSG00000030629 Zfand6 protein_coding 1.092 21.539

1.002 96.013 0.914 9.563 0.876 77.012
ENSMUSG00000030630 Fah protein_coding 0.812 1.175 1.043 83.104
1.178 18.208 0.903 83.724
ENSMUSG00000030636 1700010L04Rik protein_coding 1 84.294
1 97.263 1 79.524 1 96.183
ENSMUSG00000030638 Sh3gl3 protein_coding 0.945 82.982
1.683 82.904 0.629 36.786 1.131 74.193
ENSMUSG00000030641 4632434I11Rik protein_coding 1.296 25.944
1.093 96.843 0.705 20.511 1.409 51.967
ENSMUSG00000030643 Rab30 protein_coding 1.082 36.686 1.158
71.617 0.8 3.428 1.036 83.067
ENSMUSG00000030647 Ndufc2 protein_coding 0.926 3.623 0.948
96.948 1.137 41.806 1.001 90.289
ENSMUSG00000030649 Anapc15 protein_coding 0.99 56.477
1.017 96.763 0.991 72.695 1.022 85.629
ENSMUSG00000030650 Tmc5 protein_coding 1.106 45.61 1.202 96.948
0.752 34.592 1.017 94.19
ENSMUSG00000030652 Coq7 protein_coding 0.688 0.987 1.152 67.843
1.257 25.455 1.187 87.886
ENSMUSG00000030653 Pde2a protein_coding 0.913 43.816 0.896
72.399 1.219 13.082 0.957 85.493
ENSMUSG00000030654 Arl6ip1 protein_coding 1.113 0.577 0.973
85.921 0.927 7.742 0.946 83.724
ENSMUSG00000030655 Smg1 protein_coding 0.982 27.968 NA NA
1.035 52.574 0.991 94.904
ENSMUSG00000030657 Xylt1 protein_coding 0.934 54.176 0.887
83.733 1.208 35.13 1.041 95.273
ENSMUSG00000030659 Nucb2 protein_coding 1.262 1.493 0.893 45.262
0.886 26.057 1.147 33.269
ENSMUSG00000030660 Pik3c2a protein_coding 1.1 28.693
0.914 20.174 0.992 61.841 1.079 80.573
ENSMUSG00000030662 Ipo5 protein_coding 1.024 35.748 0.959
75.345 1.021 88.601 1.059 32.698
ENSMUSG00000030663 1110004F10Rik protein_coding 1.061 2.567
0.944 61.625 0.996 48.387 1.018 94.654
ENSMUSG00000030664 1110006G14Rik antisense 0.923 60.707
1.062 90.952 1.018 88.461 0.836 85.493
ENSMUSG00000030669 Calca protein_coding 0.962 82.231 0.625
53.79 1.66 22.517 1.005 92.31
ENSMUSG00000030670 Cyp2r1 protein_coding 0.635 8.267 1.19
82.904 1.321 42.75 0.958 92.12
ENSMUSG00000030671 Pde3b protein_coding 0.836 7.857 0.83 90.744
1.438 6.693 0.978 96.071
ENSMUSG00000030672 Mylpf protein_coding 7.344 8.685 0.875 89.494
0.156 1.061 0.862 96.086
ENSMUSG00000030674 Qprt protein_coding 1.242 14.59 1.229 85.501
0.653 5.336 1.12 89.72
ENSMUSG00000030677 Kif22 protein_coding 1.074 66.76 0.857 81.02
1.085 88.349 1.61 61.984
ENSMUSG00000030678 Maz protein_coding 0.946 20.799 0.994
88.923 1.064 73.428 1.028 86.445
ENSMUSG00000030680 Pagr1a protein_coding 0.858 17.705
1.011 90.29 1.154 29.819 1.062 94.215
ENSMUSG00000030681 Mvp protein_coding 0.932 7.916 1.001 92.631

1.073 13.709 1.174 62.594
ENSMUSG00000030682 Cdipt protein_coding 0.924 6.952 1.084 79.055
0.999 50.091 0.973 92.52
ENSMUSG00000030683 Sez6l2 protein_coding 0.942 61.043
1.076 93.529 0.989 79.554 0.883 85.493
ENSMUSG00000030685 Kctd13 protein_coding 1.079 84.564
0.933 93.817 0.995 87.274 0.894 85.493
ENSMUSG00000030688 Stard10 protein_coding 0.873 30.035
0.902 53.79 1.267 9.807 1.001 93.264
ENSMUSG00000030689 Ino80e protein_coding 1.017 73.585
0.945 97.263 1.041 81.032 1.009 90.865
ENSMUSG00000030691 Fchsd2 protein_coding 1.241 7.179 1.02
95.708 0.786 3.428 0.924 92.561
ENSMUSG00000030693 Klk10 protein_coding 0.95 75.298 0.748
91.361 1.4 60.104 1.081 95.92
ENSMUSG00000030695 Aldoa protein_coding 0.646 0.987 1.292 71.617
1.202 21.201 1.252 57.075
ENSMUSG00000030697 Ppp4c protein_coding 1.023 81.657 0.998
96.763 0.981 84.669 1.007 95.273
ENSMUSG00000030699 Tbx6 protein_coding 0.818 25.3 1.207 89.434
1.015 60.789 0.867 93.316
ENSMUSG00000030701 Plekhb1 protein_coding 0.897 28.242
0.998 96.216 1.116 30.561 1.022 91.291
ENSMUSG00000030703 Gdpd3 protein_coding 0.815 19.985 1.109
83.104 1.109 72.853 1.053 78.042
ENSMUSG00000030704 Rab6a protein_coding 1.07 8.685 1.026 72.642
0.909 2.687 0.999 93.261
ENSMUSG00000030706 Mrpl48 protein_coding 0.963 65.388
0.979 93.654 1.057 60.789 1.076 54.265
ENSMUSG00000030707 Coro1a protein_coding 1.337 0 0.869
73.166 0.862 22.009 1.096 52.982
ENSMUSG00000030708 Dnajb13 protein_coding 1.575 2.567 0.592
28.832 1.069 65.235 0.837 86.991
ENSMUSG00000030711 Sult1a1 protein_coding 0.704 3.07 0.9
70.29 1.57 5.864 1.137 84.499
ENSMUSG00000030714 Ccdc101 protein_coding 0.882 8.751 1.084
82.904 1.05 49.384 1.034 82.66
ENSMUSG00000030717 Nupr1 protein_coding 0.967 37.248 0.971
96.763 1.064 52.432 0.951 84.29
ENSMUSG00000030718 Ppme1 protein_coding 0.916 8.139 1.063 79.367
1.03 51.977 1.08 44.213
ENSMUSG00000030720 Cln3 protein_coding 1.023 49.868 1.013
85.075 0.962 88.693 0.948 83.724
ENSMUSG00000030722 Nfatc2ip protein_coding 0.987 86.474
1.052 91.584 0.965 45.573 0.872 83.724
ENSMUSG00000030724 Cd19 protein_coding 0.967 83.658 1.495
81.127 0.693 27.194 1.152 93.685
ENSMUSG00000030725 Lipt2 protein_coding 0.955 27 0.979 93.046
1.074 35.894 0.877 93.685
ENSMUSG00000030726 Pold3 protein_coding 1.096 5.253 0.934 75.052
0.973 25.345 0.968 92.181
ENSMUSG00000030727 Rabep2 protein_coding 1.16 64.956
1.127 82.904 0.764 21.331 0.945 93.685
ENSMUSG00000030729 Pgm2l1 protein_coding 0.929 39.712

1.025 91.532 1.053 34.807 1.027 94.567
ENSMUSG00000030730 Atp2a1 protein_coding 7.642 7.174 0.979
85.756 0.134 0.72 0.886 95.92
ENSMUSG00000030731 Syt3 protein_coding 1.01 80.408 1.139
83.104 0.867 55.701 0.825 84.29
ENSMUSG00000030733 Sh2b1 protein_coding 0.952 22.731 1.041
79.055 1.009 85.675 1.004 96.184
ENSMUSG00000030737 Slco2b1 protein_coding 1.18 8.847 0.901
76.431 0.936 33.402 0.968 88.245
ENSMUSG00000030738 Eif3c protein_coding 0.967 63.283 1.016
91.361 1.015 45.94 1.025 83.067
ENSMUSG00000030739 Myh14 protein_coding 0.769 34.162 0.973
82.073 1.336 39.471 1.218 74.047
ENSMUSG00000030741 Spns1 protein_coding 0.94 19.224 0.959
88.881 1.104 25.93 0.969 91.291
ENSMUSG00000030742 Lat protein_coding 0.831 60.569 1.205
71.617 0.994 54.521 1.038 86.991
ENSMUSG00000030744 Rps3 protein_coding 0.99 81.24 0.9 0
1.124 25.425 1.052 64.513
ENSMUSG00000030745 Il21r protein_coding 1.098 64.401 1.38
82.904 0.661 12.41 1.034 93.156
ENSMUSG00000030747 Dgat2 protein_coding 0.597 0.987 1.213 82.809
1.376 9.176 1.057 76.697
ENSMUSG00000030748 Il4ra protein_coding 0.844 62.888 1.534
60.309 0.768 14.583 2.095 72.826
ENSMUSG00000030750 Nsmce1 protein_coding 1.072 6.281 0.966
85.116 0.963 35.264 1.109 82.328
ENSMUSG00000030751 Psma1 protein_coding 0.941 13.239 1.045
83.104 1.012 48.935 1.004 85.577
ENSMUSG00000030752 Kdm8 protein_coding 0.965 82.231 0.966
92.137 1.074 61.171 1.01 96.086
ENSMUSG00000030753 Prkrir protein_coding 1.076 7.492 1.052
71.617 0.883 1.061 1.021 85.629
ENSMUSG00000030754 Copb1 protein_coding 1.086 43.654 0.95
91.361 0.967 48.851 1.029 78.948
ENSMUSG00000030757 Zkscan2 protein_coding 2.123 37.414
1.145 82.904 0.412 11.116 1.24 91.872
ENSMUSG00000030759 Far1 protein_coding 1.138 44.061 0.934
74.483 0.939 83.607 1.013 76.831
ENSMUSG00000030760 Acer3 protein_coding 1.193 2.352 0.98 93.654
0.853 11.314 1.032 83.581
ENSMUSG00000030761 Myo7a protein_coding 1.11 21.539 0.964
81.731 0.933 51.443 1.154 40.894
ENSMUSG00000030763 Lcmt1 protein_coding 1.04 18.388 1.011
96.897 0.952 13.848 1.079 44.679
ENSMUSG00000030766 Arhgap17 protein_coding 1.151 0.2 0.968
78.877 0.899 1.764 0.998 96.358
ENSMUSG00000030768 Disp1 protein_coding 1.059 55.124 1.055
92.111 0.893 35.559 1.009 91.372
ENSMUSG00000030769 Slc5a11 protein_coding 0.778 82.092
2.114 93.574 0.609 56.625 1.48 91.492
ENSMUSG00000030770 Parva protein_coding 0.904 4.099 1.031 86.277
1.071 26.912 1.018 65.401
ENSMUSG00000030771 Micalcl protein_coding 0.782 36.823 NA

NA 1.303 62.995 0.977 93.685
ENSMUSG00000030772 Dkk3 protein_coding 1.074 10.19 0.932 89.509
0.996 63.504 0.822 78.025
ENSMUSG00000030774 Pak1 protein_coding 0.993 78.538 0.883
73.166 1.135 41.331 1.073 79.545
ENSMUSG00000030779 Rbbp6 protein_coding 0.988 70.862 0.984
96.216 1.03 61.418 1.001 95.676
ENSMUSG00000030780 BC017158 protein_coding 0.911 9.863 1.109
71.617 0.988 88.729 1.06 40.894
ENSMUSG00000030781 Slc5a2 protein_coding 0.798 8.139 1.257
79.579 0.994 68.643 0.928 86.699
ENSMUSG00000030782 Tgfb1i1 protein_coding 1.002 81.823
1.101 72.546 0.909 13.202 0.997 93.734
ENSMUSG00000030785 Cox6a2 protein_coding 4.985 8.996 1.635
83.104 0.123 0.72 1.078 95.273
ENSMUSG00000030786 Itgam protein_coding 2.019 0 0.82 62.322
0.603 6.858 1.188 58.557
ENSMUSG00000030787 Lyve1 protein_coding 1.386 45.61 0.744 81.731
0.965 76.388 2.27 35.053
ENSMUSG00000030788 Rnf141 protein_coding 1.073 7.959 0.966
90.712 0.963 35.022 1.059 68.696
ENSMUSG00000030789 Itgax protein_coding 0.996 83.523 0.957
92.561 1.053 57.446 0.97 85.493
ENSMUSG00000030790 Adm protein_coding 1.279 42.255 0.785
70.864 0.997 78.155 1.097 83.067
ENSMUSG00000030792 Dkkl1 protein_coding 1.156 25.421 0.944
95.708 0.919 20.009 1.149 96.358
ENSMUSG00000030793 Pycard protein_coding 1.371 0.664 0.891
84.594 0.814 11.997 1.169 40.426
ENSMUSG00000030795 Fus protein_coding 1.143 13.105 0.921
79.807 0.946 24.918 0.968 92.31
ENSMUSG00000030796 Tead2 protein_coding 0.707 5.962 1.029 92.561
1.371 26.912 1.044 91.625
ENSMUSG00000030798 Cd37 protein_coding 1.523 0.984 0.706 60.678
0.927 58.775 1.029 95.92
ENSMUSG00000030800 Prss8 protein_coding 0.917 61.334 0.917
87.857 1.188 39.471 0.857 90.628
ENSMUSG00000030801 Kat8 protein_coding 0.974 16.325 0.996
96.948 1.032 43.307 0.991 94.016
ENSMUSG00000030802 Bckdk protein_coding 0.975 67.867 1.004
96.843 1.02 83.857 1.028 85.786
ENSMUSG00000030804 Gm21974 protein_coding 1 81.196 1
93.654 1 81.976 1 92.748
ENSMUSG00000030805 Stx4a protein_coding 1.016 74.516 0.951
87.452 1.038 72.148 1.051 56.339
ENSMUSG00000030806 Stx1b protein_coding 0.865 78.744 1.052
95.196 1.095 78.498 1.005 96.248
ENSMUSG00000030811 Fbxl19 protein_coding 0.883 38.286
0.989 94.597 1.147 48.304 1.001 95.593
ENSMUSG00000030814 Bcl7c protein_coding 0.988 81.01 1.06 83.104
0.957 56.822 1.023 95.676
ENSMUSG00000030815 Phkg2 protein_coding 0.926 36.034 1.001
93.994 1.075 43.307 1.039 92.018
ENSMUSG00000030816 Rnf40 protein_coding 0.977 85.857 1.041

96.216	0.985 85.935	1.013 87.7
ENSMUSG00000030822	Prr14 protein_coding	0.912 8.181 1.098 67.462
0.996 81.62 1.003 86.991
ENSMUSG00000030823	9130019O22Rik	protein_coding	1.018 74.516
1.065 82.904	0.921 30.468	0.962 88.053
ENSMUSG00000030824	Nucb1 protein_coding	1.021 69.408	0.947
85.921	1.035 84.896	1.034 76.697
ENSMUSG00000030825	Hsd17b14	protein_coding	0.753 16.02 1.196
93.899	1.112 58.051	0.841 86.699
ENSMUSG00000030826	Bcat2 protein_coding	0.943 80.251	0.998
96.138	1.064 79.426	0.782 83.952
ENSMUSG00000030830	Itgal protein_coding	1.238 19.315	1.185
72.642	0.679 6.202 0.754 83.724
ENSMUSG00000030834	Abcc6 protein_coding	0.625 18.379	1.087
95.196	1.476 44.361	1.026 94.82
ENSMUSG00000030835	Nomo1 protein_coding	0.874 1.698 1.071 78.629
1.068 32.433	1.086 85.577
ENSMUSG00000030839	Sergef	protein_coding	1.151 9.259 0.919
51.277	0.941 88.693	1	93.734
ENSMUSG00000030842	Lamtor1	protein_coding	0.845 0.987 1.087
79.29 1.091 34.285	1.016 90.455
ENSMUSG00000030844	Rgs10 protein_coding	1.483 2.233 0.849 66.796
0.795 21.666	0.922 91.372
ENSMUSG00000030846	Tial1 protein_coding	0.986 85.129	0.998
82.947	1.017 54.809	1.005 95.273
ENSMUSG00000030847	Bag3	protein_coding	1.042 25.207	1.023
96.847	0.936 18.797	1.012 91.525
ENSMUSG00000030849	Fgfr2 protein_coding	0.82	5.204 0.933 87.452
1.309 19.877	0.709 86.445
ENSMUSG00000030850	Ate1	protein_coding	1.032 25.026	0.952
66.651	1.019 85.352	0.948 84.29
ENSMUSG00000030851	Ldhc	protein_coding	1	72	NA	NA	1
87.744	1	95.405
ENSMUSG00000030852	Tacc2 protein_coding	1.072 52.799	0.986
92.404	0.944 82.575	1.155 31.133
ENSMUSG00000030854	Ptpn5 protein_coding	0.551 61.633	2.896
71.98 0.627 43.842	17.622	52.324
ENSMUSG00000030859	Fam24a	protein_coding	1	86.819	1
97.288	1	84.961	1	87.447
ENSMUSG00000030861	Acadsb	protein_coding	1.025 56.085
1.023 92.253	0.956 36.248	0.969 84.29
ENSMUSG00000030862	Cpxm2 protein_coding	1.093 16.249	1.128
82.904	0.809 8.907 0.932 84.29
ENSMUSG00000030865	Chp2	protein_coding	1.617 1.643 0.869 55.265
0.71	20.009	0.696 83.724
ENSMUSG00000030866	Ern2	protein_coding	1	73.124	1
95.504	1	52.972	1	92.181
ENSMUSG00000030867	Plk1	protein_coding	1.197 50.913	0.829
53.79 1.004 82.161	1.35	74.193
ENSMUSG00000030868	Dctn5 protein_coding	1.015 41.211	1.061
96.407	0.93	32.297	1.036 79.545
ENSMUSG00000030869	Ndufab1	protein_coding	0.924 1.175 1.01
87.185	1.071 27.732	0.968 90.691
ENSMUSG00000030870	Ubfd1 protein_coding	1.026 54.344	1.042

88.91 0.936 24.523 0.97 92.31
ENSMUSG00000030871 Ears2 protein_coding 0.912 42.361 NA NA
1.087 60.789 1.038 93.156
ENSMUSG00000030872 Gga2 protein_coding 0.969 53.679 1.082
60.309 0.95 14.206 0.989 92.018
ENSMUSG00000030873 Scnn1b protein_coding 1.229 39.851
1.038 93.146 0.783 17.381 0.777 83.724
ENSMUSG00000030876 Mettl9 protein_coding 1.046 13.557
1.073 82.904 0.887 7.329 0.983 86.991
ENSMUSG00000030877 4933427G17Rik protein_coding 0.752 35.449
1.095 97.263 1.213 46.956 0.862 85.493
ENSMUSG00000030878 Cdr2 protein_coding 0.822 2.536 1.367 72.642
0.889 69.134 1.214 76.467
ENSMUSG00000030879 Mrpl17 protein_coding 0.955 28.998
1.071 79.411 0.978 87.173 0.968 87.651
ENSMUSG00000030880 Polr3e protein_coding 0.865 11.128
0.997 95.708 1.161 23.454 1.125 67.169
ENSMUSG00000030881 Arfip2 protein_coding 0.884 0.987 1.001
91.456 1.126 6.449 1.033 96.248
ENSMUSG00000030882 Dnhd1 protein_coding 0.964 32.749 0.917
86.071 1.134 18.411 0.874 83.724
ENSMUSG00000030884 Uqcrc2 protein_coding 0.965 41.021 1.01
92.111 1.023 73.309 0.997 92.31
ENSMUSG00000030887 Pdzd9 protein_coding 0.892 79.709 1.276
91.584 0.879 78.954 0.993 95.273
ENSMUSG00000030888 Rrp8 protein_coding 0.985 78.299 1.046
78.705 0.968 63.504 1.043 67.691
ENSMUSG00000030889 Vwa3a protein_coding 0.797 9.752 0.747 39.547
1.67 5.864 0.747 83.724
ENSMUSG00000030890 Ilk protein_coding 0.947 6.677 1.093 71.617
0.969 77.037 1.014 76.697
ENSMUSG00000030894 Tpp1 protein_coding 1.074 38.386 1.013
87.609 0.917 17.662 0.98 94.608
ENSMUSG00000030895 Hpx protein_coding 0.848 80.51 0.78 92.137
1.504 40.875 0.836 90.805
ENSMUSG00000030897 Cnga4 protein_coding 1.074 77.113 0.877
92.137 1.062 62.307 0.532 84.19
ENSMUSG00000030905 Crym protein_coding 1.107 84.452 0.797
70.29 1.137 71.615 1.391 89.498
ENSMUSG00000030911 Zp2 protein_coding 1 86.474 NA NA
1 80.015 1 94.153
ENSMUSG00000030917 Tmem159 protein_coding 1.047 44.061
1.037 96.335 0.919 36.499 0.905 85.493
ENSMUSG00000030921 Trim30a protein_coding 1.255 0 1.027
96.216 0.776 12.41 1.355 42.736
ENSMUSG00000030922 Lyrm1 protein_coding 0.937 44.252 0.927
83.321 1.155 23.334 0.995 96.042
ENSMUSG00000030924 2610020H08Rik protein_coding 0.877 19.595
0.79 45.262 1.444 5.864 0.97 92.181
ENSMUSG00000030929 Eri2 protein_coding 1.004 74.8 0.958 76.978
1.036 34.285 1.059 83.067
ENSMUSG00000030930 Chst15 protein_coding 1.202 39.416
0.901 76.431 0.924 59.726 0.994 95.273
ENSMUSG00000030934 Oat protein_coding 1.222 0.355 1.01 96.68

0.813 0.72 1.03 76.347
ENSMUSG00000030935 Acsm3 protein_coding 1.354 81.52 0.571 82.848
1.295 55.81 0.694 84.29
ENSMUSG00000030942 Thumpd1 protein_coding 0.978 62.437 0.96
68.818 1.068 10.281 1.069 40.024
ENSMUSG00000030945 Acsm2 protein_coding 1 81.01 NA NA 1
88.461 1 96.192
ENSMUSG00000030946 Lhpp protein_coding 0.964 56.242 0.945
96.763 1.097 84.724 1.11 54.265
ENSMUSG00000030954 Gp2 protein_coding 1 86.57 NA NA 1
87.744 1 96.287
ENSMUSG00000030956 Fam53b protein_coding 1.028 54.717
1.115 71.98 0.872 7.083 1.09 83.276
ENSMUSG00000030960 Mettl10 protein_coding 0.99 84.564
1.013 96.013 0.993 87.686 1.096 49.806
ENSMUSG00000030963 Umod protein_coding 1 81.01 1 95.504
1 87.519 1 92.748
ENSMUSG00000030965 Fam175b protein_coding 1.085 1.913 0.987
88.881 0.936 9.288 1.023 95.405
ENSMUSG00000030966 Trim21 protein_coding 1.143 1.643 1.086
90.389 0.804 0.72 0.957 89.892
ENSMUSG00000030967 Zranb1 protein_coding 1.01 62.574
0.994 85.671 0.991 76.754 1.015 73.823
ENSMUSG00000030970 Ctbp2 protein_coding 1.102 5.109 0.985 75.993
0.922 35.022 1.055 87.7
ENSMUSG00000030972 Acsm5 protein_coding 0.785 39.174 0.743
89.277 1.717 30.213 1.112 68.502
ENSMUSG00000030976 Tex36 protein_coding 1.149 84.865 0.851
94.848 1.018 84.504 1.192 92.762
ENSMUSG00000030978 Rrm1 protein_coding 1.09 38.195 1
96.268 0.916 48.577 1.144 56.863
ENSMUSG00000030979 Uros protein_coding 0.858 1.031 1.062 67.843
1.094 27.533 0.983 96.192
ENSMUSG00000030980 Knop1 protein_coding 1.02 67.991 NA NA
0.974 50.184 1.002 85.786
ENSMUSG00000030982 9030624J02Rik protein_coding 1.044 20.659
NA NA 0.974 24.309 1.022 73.368
ENSMUSG00000030983 Bccip protein_coding 0.981 79.433 1.039
82.904 0.978 26.809 1.009 89.052
ENSMUSG00000030986 Dhx32 protein_coding 1.047 37.941 1.139
83.104 0.84 11.997 1.041 87.447
ENSMUSG00000030987 Stim1 protein_coding 0.997 82.705 0.994
95.088 1.012 88.601 0.988 90.611
ENSMUSG00000030990 Pgap2 protein_coding 1.087 0.2 1.001 94.48
0.917 3.969 0.995 92.181
ENSMUSG00000030994 D7Ertd443e protein_coding 0.856 29.972
1.097 96.407 1.064 51.596 0.877 90.95
ENSMUSG00000030996 Art1 protein_coding 3.443 14.205 2.019
80.775 0.144 0.72 0.802 95.273
ENSMUSG00000031004 Mki67 protein_coding 1.398 46.533 0.797
71.331 0.897 88.729 1.246 70.85
ENSMUSG00000031007 Atp6ap2 protein_coding 1.296 0 0.901
53.434 0.852 2.817 1.007 89.152
ENSMUSG00000031010 Usp9x protein_coding 0.977 36.945 NA NA

1.046 35.673 0.956 95.832
ENSMUSG00000031012 Cask protein_coding 0.974 69.985 0.985
72.399 1.044 27.533 1.029 90.455
ENSMUSG00000031015 Swap70 protein_coding 1.107 0.976 0.962
72.399 0.937 56.822 0.979 88.053
ENSMUSG00000031016 Wee1 protein_coding 1.082 69.777 0.905
91.955 1.019 82.31 1.177 77.854
ENSMUSG00000031021 Tmem9b protein_coding 0.995 84.324 1.01
88.092 0.991 67.962 1.06 65.401
ENSMUSG00000031023 Akip1 protein_coding 1.014 66.58 0.952 71.889
1.036 84.724 0.975 89.827
ENSMUSG00000031024 St5 protein_coding 1.157 13.292 0.971
97.263 0.887 4.935 0.944 83.952
ENSMUSG00000031026 Trim66 protein_coding 0.861 80.608
0.657 75.618 1.77 40.052 1.309 95.35
ENSMUSG00000031027 Stk33 protein_coding 1.362 84.819 0.482
90.378 1.524 82.74 1.573 92.691
ENSMUSG00000031028 Tub protein_coding 1.076 63.385 1.036
95.809 0.9 39.528 1.166 73.939
ENSMUSG00000031029 Eif3f protein_coding 1.148 0.577 0.933 35.895
0.933 32.963 0.964 85.493
ENSMUSG00000031059 Ndufb11 protein_coding 0.88 5.686 1.037
83.104 1.095 34.07 0.962 86.699
ENSMUSG00000031060 Rbm10 protein_coding 0.952 19.595 1.028
96.68 1.016 33.802 0.994 92.742
ENSMUSG00000031065 Cdk16 protein_coding 0.911 2.009 1.082 60.309
1.014 85.921 1.021 81.56
ENSMUSG00000031066 Usp11 protein_coding 0.97 79.165 0.999
93.654 1.029 57.227 1.05 92.691
ENSMUSG00000031068 Glrx3 protein_coding 1.009 83.823 0.945
95.708 1.044 79.897 1.108 52.324
ENSMUSG00000031070 Mrgprf protein_coding 1.676 0.984 1.145
96.407 0.522 0.72 0.989 90.397
ENSMUSG00000031072 Oraov1 protein_coding 0.947 38.286
1.006 96.407 1.047 40.875 1.092 44.679
ENSMUSG00000031073 Fgf15 protein_coding 1 75.196 1
85.95 NA NA 1 90.691
ENSMUSG00000031075 Ano1 protein_coding 0.819 48.146 1.027
91.361 1.193 38.683 1.079 95.74
ENSMUSG00000031077 Fadd protein_coding 0.921 14.849 NA NA
1.107 45.035 1.014 94.384
ENSMUSG00000031078 Cttn protein_coding 0.889 1.031 0.957 65.165
1.172 5.864 1.008 95.273
ENSMUSG00000031079 Zfp300 protein_coding 0.993 77.916
1.387 82.904 0.722 21.117 1.153 74.047
ENSMUSG00000031085 Gm498 protein_coding 1 67.334 1
95.708 1 88.161 1 96.183
ENSMUSG00000031090 Nadsyn1 protein_coding 1.004 76.686 NA
NA 1.061 74.082 0.965 93.156
ENSMUSG00000031093 Dock11 protein_coding 1.105 57.133
0.824 75.4 1.099 50.636 0.965 96.192
ENSMUSG00000031095 Cul4b protein_coding 1.077 21.146 0.963
90.138 0.96 27.194 0.973 86.699
ENSMUSG00000031097 Tnni2 protein_coding 7.103 7.179 1.244 87.037

0.113 0.72 0.948 95.877
ENSMUSG00000031098 Syt8 protein_coding 0.874 29.972 0.711
66.796 1.612 13.709 1.472 81.901
ENSMUSG00000031099 Smarca1 protein_coding 1.4 1.441 1
94.262 0.714 6.858 1.227 72.616
ENSMUSG00000031101 Sash3 protein_coding 1.604 0.767 1.017 96.216
0.614 6.858 0.965 96.248
ENSMUSG00000031103 Elf4 protein_coding 1.221 1.913 1.009 90.68
0.811 3.943 1.038 85.577
ENSMUSG00000031104 Rab33a protein_coding 0.701 29.972
1.177 82.904 1.213 84.961 1.236 91.872
ENSMUSG00000031105 Slc25a14 protein_coding 1.187 23.019
1.019 90.389 0.826 30.024 0.921 93.584
ENSMUSG00000031107 Rbmx2 protein_coding 1.227 32.238 0.825
76.978 0.986 87.336 0.948 94.608
ENSMUSG00000031109 Enox2 protein_coding 1.092 4.628 0.913 23.928
1.006 86.084 1.008 93.964
ENSMUSG00000031111 Igsf1 protein_coding 0.984 76.968 NA NA
0.902 78.155 1.019 94.153
ENSMUSG00000031112 2610018G03Rik protein_coding 1.048 33.099
0.997 91.661 0.953 57.091 0.896 84.29
ENSMUSG00000031118 1700080O16Rik protein_coding 1 81.52 1
90.046 1 84.896 1 96.042
ENSMUSG00000031119 Gpc4 protein_coding 0.985 85.655 1.066
83.104 0.95 52.087 1.021 94.19
ENSMUSG00000031125 3830403N18Rik protein_coding 0.751 85.355
2.189 82.904 0.606 38.434 1.781 89.108
ENSMUSG00000031129 Slc9a9 protein_coding 1.651 0.2 0.925
78.471 0.652 8.192 1.083 85.629
ENSMUSG00000031130 Brs3 protein_coding 1 70.213 1
95.708 1 86.552 1 93.722
ENSMUSG00000031131 Vgll1 protein_coding 1 83.169 1
96.68 1 59.383 1 91.525
ENSMUSG00000031132 Cd40lg protein_coding 1.787 45.746
0.522 86.861 1.071 88.41 0.884 93.316
ENSMUSG00000031133 Arhgef6 protein_coding 1.229 0 0.993
85.075 0.821 0.72 0.918 83.724
ENSMUSG00000031134 Rbmx protein_coding 1.094 0 1.017 96.013
0.899 6.858 0.996 88.053
ENSMUSG00000031137 Fgf13 protein_coding 0.986 40.422 1.076
83.104 0.942 87.686 1.075 92.381
ENSMUSG00000031142 Cacna1f protein_coding 1.144 77.291
1.319 96.847 0.662 74.853 0.906 93.734
ENSMUSG00000031143 Ccdc22 protein_coding 0.96 45.894
1.031 92.793 1.006 78.864 1.066 72.116
ENSMUSG00000031144 Syp protein_coding 1.148 35.748 1.058
91.776 0.824 39.755 1.001 91.525
ENSMUSG00000031145 Prickle3 protein_coding 0.917 14.849
0.997 93.899 1.095 43.842 0.96 86.991
ENSMUSG00000031146 Plp2 protein_coding 1.076 27.545 0.963
88.691 0.964 65.409 1.042 69.965
ENSMUSG00000031147 Magix protein_coding 1.226 5.559 0.978 95.088
0.831 11.997 0.915 87.218
ENSMUSG00000031148 Gpkow protein_coding 1.007 84.168 0.991

93.654 1.004 84.724 1.039 82.328
ENSMUSG00000031149 Praf2 protein_coding 1.092 34.861 1.015
86.34 0.903 13.551 1.146 42.736
ENSMUSG00000031150 Ccdc120 protein_coding 0.774 5.084 1.019
94.48 1.273 13.174 1.036 87.735
ENSMUSG00000031153 Gripap1 protein_coding 0.978 30.877
0.975 81.443 1.044 10.615 1.001 88.657
ENSMUSG00000031154 Otud5 protein_coding 0.961 19.365 1.021
93.899 1.019 48.304 1.008 77.854
ENSMUSG00000031155 Pim2 protein_coding 0.919 56.477 1.167
79.579 0.932 81.525 1.028 91.928
ENSMUSG00000031156 Slc35a2 protein_coding 0.958 37.82 0.975
93.094 1.073 50.091 1.021 74.193
ENSMUSG00000031157 Pqbp1 protein_coding 0.885 5.686 1.076 89.996
1.052 29.819 1.069 63.708
ENSMUSG00000031158 Timm17b protein_coding 0.897 3.325 1.025
83.464 1.081 24.691 1.043 83.276
ENSMUSG00000031160 Eras protein_coding 1 70.213 1
96.843 1 85.757 1 94.297
ENSMUSG00000031161 Hdac6 protein_coding 0.762 1.6 0.964 91.532
1.366 5.864 0.963 85.493
ENSMUSG00000031162 Gata1 protein_coding 2.775 10.687 0.352
54.06 1.025 86.084 1.081 95.273
ENSMUSG00000031165 Was protein_coding 1.307 1.828 0.938 94.96
0.818 6.381 1.028 95.273
ENSMUSG00000031166 Wdr13 protein_coding 0.951 4.895 0.987 94.102
1.06 12.703 1.051 49.806
ENSMUSG00000031167 Rbm3 protein_coding 1.109 1.129 1.028 90.092
0.877 3.943 1.177 43.377
ENSMUSG00000031168 Ebp protein_coding 0.985 51.089 1.052
83.104 0.966 84.961 1.009 78.948
ENSMUSG00000031169 Porcn protein_coding 1.032 86.57 0.847 81.731
1.139 21.522 0.959 85.636
ENSMUSG00000031170 Slc38a5 protein_coding 0.567 73.124
0.598 92.474 2.95 82.161 4.32 90.463
ENSMUSG00000031171 Ftsj1 protein_coding 0.929 36.541 1.091
67.462 0.984 72.269 1.035 73.401
ENSMUSG00000031173 Otc protein_coding 1 60.707 1
88.91 1 86.258 1 93.261
ENSMUSG00000031174 Rpgr protein_coding 1.077 51.297 0.886
82.503 1.042 61.743 0.896 83.952
ENSMUSG00000031176 Dynlt3 protein_coding 0.969 76.686
1.017 95.941 1.017 77.532 1.021 93.264
ENSMUSG00000031179 3830417A13Rik protein_coding 2.149 0.461 1.43
82.219 0.326 0.72 1.154 96.248
ENSMUSG00000031181 Ctag2 protein_coding 1 85.832 1
94.848 NA NA 1 95.877
ENSMUSG00000031182 NA NA NA NA NA NA 1 87.173
1 95.877
ENSMUSG00000031189 Aff2 protein_coding 0.659 15.76 1.002 86.387
1.508 54.163 1.452 48.857
ENSMUSG00000031194 4931400O07Rik protein_coding 1 81.925
1 90.744 1 86.084 1 96.248
ENSMUSG00000031196 F8 protein_coding 1.056 71.217 0.942

87.919	1	81.857	1.112 49.514
ENSMUSG00000031197	Vbp1	protein_coding	1.122 4.228 1.001 95.708
0.888 9.901 1.05	75.81
ENSMUSG00000031198	Fundc2	protein_coding	1.061 8.847 0.96
73.166	0.979 54.25 1.099 57.1
ENSMUSG00000031200	Mtcp1 protein_coding	0.836 33.415	NA	NA
1.098 33.287	1.104 75.198
ENSMUSG00000031201	Brcc3 protein_coding	1.048 52.429	0.937
86.071	1.017 86.831	1.033 83.067
ENSMUSG00000031202	Rab39b	protein_coding	1.295 29.811
1.097 96.68 0.701 25.625	0.724 85.493
ENSMUSG00000031204	Asb12 protein_coding	1.792 50.627	2.044
72.642	0.273 4.23	0.695 96.287
ENSMUSG00000031207	Msn	protein_coding	1.034 65.537	1.05
82.809	0.922 22.864	1.155 64.513
ENSMUSG00000031209	Heph	protein_coding	1.056 74.958	0.996
96.001	0.951 68.266	1.061 78.379
ENSMUSG00000031210	Gpr165	protein_coding	1.008 82.891
0.767 93.908	1.293 62.995	1.036 94.82
ENSMUSG00000031212	Pgr15l	protein_coding	1	85.129	1
95.708	1	80.634	1	95.559
ENSMUSG00000031214	Ophn1 protein_coding	1.265 2.897 0.848 68.848
0.932 29.765	0.899 83.724
ENSMUSG00000031216	Stard8	protein_coding	1.241 0	0.87
64.624	0.924 17.898	1.145 64.513
ENSMUSG00000031217	Efnb1 protein_coding	1.018 82.231	1.022
91.897	0.959 70.534	0.935 84.29
ENSMUSG00000031220	Awat2 protein_coding	1.187 45.096	0.95
96.847	0.882 44.865	1.063 81.56
ENSMUSG00000031221	Igbp1 protein_coding	1.077 13.8	0.896 13.578
1.035 32.433	0.952 88.955
ENSMUSG00000031224	Magee2	protein_coding	0.905 69.777
2.277 96.984	0.484 62.533	1.825 83.067
ENSMUSG00000031226	Pbdc1 protein_coding	0.989 82.891	0.997
97.155	1.012 84.83 1.106 52.982
ENSMUSG00000031227	Magee1	protein_coding	1.012 68.353
1.057 71.98 0.935 37.617	1.016 79.268
ENSMUSG00000031229	Atrx	protein_coding	0.999 46.413	0.939
72.399	1.066 15.411	0.96	92.181
ENSMUSG00000031230	Fgf16 protein_coding	1.196 46.533	0.893
81.731	0.937 88.729	1.127 92.748
ENSMUSG00000031231	Cox7b protein_coding	0.9	14.403	1.127
60.309	0.985 87.744	0.984 94.654
ENSMUSG00000031232	Magt1 protein_coding	0.964 84.661	1.075
85.756	0.964 57.819	1.039 76.831
ENSMUSG00000031233	Pgk2	protein_coding	1	76.042	1
95.211	NA	NA	1	96.183
ENSMUSG00000031239	Itm2a protein_coding	1.162 23.254	0.865
62.322	0.995 79.255	1.091 93.457
ENSMUSG00000031241	Tbx22 protein_coding	1	82.352	1
90.378	1	75.527	1	93.734
ENSMUSG00000031242	2610002M06Rik	protein_coding	1.11	3.498
0.993 92.111	0.908 6.858 1.024 96.248
ENSMUSG00000031245	Hmgn5 protein_coding	0.853 4.115 0.933 90.256

1.252 7.635 1.029 91.872
ENSMUSG00000031246 Sh3bgrl protein_coding 1.202 0.664 0.948
86.861 0.881 0.72 1.024 93.413
ENSMUSG00000031250 Tnmd protein_coding 1.536 54.344 NA NA
0.475 71.004 1.746 83.067
ENSMUSG00000031253 Srpx2 protein_coding 1.117 11.044 1.088
94.367 0.824 17.258 1.263 40.894
ENSMUSG00000031255 Sytl4 protein_coding 1.14 37.091 NA NA
0.906 35.022 1.08 83.067
ENSMUSG00000031256 Cstf2 protein_coding 0.939 9.841 0.993 96.407
1.071 30.948 1.045 50.74
ENSMUSG00000031257 Nox1 protein_coding 1.602 62.689 0.603
85.242 1.03 66.269 0.705 91.625
ENSMUSG00000031258 Xkrx protein_coding 0.972 76.396 NA NA
0.893 82.436 0.795 86.991
ENSMUSG00000031262 Cenpi protein_coding 1.227 38.195 0.856
92.137 0.949 67.339 1.438 70.539
ENSMUSG00000031264 Btk protein_coding 1.545 0 0.957 84.594
0.677 4.759 1.243 52.804
ENSMUSG00000031266 Gla protein_coding 1.148 5.313 0.964 86.071
0.904 46.32 1.202 44.679
ENSMUSG00000031270 4930513O06Rik protein_coding 1 85.391
1 96.68 1 86.258 1 92.748
ENSMUSG00000031273 Col4a6 protein_coding 1.086 81.823
0.877 90.378 1.047 62.307 0.79 86.445
ENSMUSG00000031274 Col4a5 protein_coding 0.969 51.089
0.926 68.818 1.113 24.691 0.802 91.372
ENSMUSG00000031278 Acsl4 protein_coding 1.431 0.2 0.832 61.883
0.838 8.192 1.114 80.497
ENSMUSG00000031283 Chrdl1 protein_coding 1.443 0.2 1.045
94.848 0.661 4.759 0.946 92.466
ENSMUSG00000031284 Pak3 protein_coding 1.13 39.416 0.978
96.68 0.905 38.799 0.873 83.724
ENSMUSG00000031286 Glt28d2 protein_coding 1.049 86.474 1.33
40.99 0.717 31.887 1.035 93.156
ENSMUSG00000031289 Il13ra2 protein_coding 1.054 72.27 0.457
66.651 2.071 38.26 1.244 91.217
ENSMUSG00000031290 Lrch2 protein_coding 1.094 58.38 1.131 87.609
0.809 33.971 0.922 85.493
ENSMUSG00000031292 Cdkl5 protein_coding 0.873 47.266 0.967
88.881 1.178 31.4 1.024 96.358
ENSMUSG00000031294 D630029K05Rik lincRNA 0.629 10.955
0.696 91.361 2.293 22.994 0.493 85.493
ENSMUSG00000031295 Phka2 protein_coding 0.869 13.239 1.01
95.708 1.134 41.806 0.957 84.29
ENSMUSG00000031297 Slc7a3 protein_coding 0.949 62.437 0.9
85.116 1.166 30.107 0.835 86.699
ENSMUSG00000031298 Gpr64 protein_coding 0.872 35.449 1.016
93.765 1.13 37.764 1.049 79.442
ENSMUSG00000031299 Pdha1 protein_coding 0.611 1.274 1.034 91.643
1.582 7.24 1.009 93.132
ENSMUSG00000031302 Nlgn3 protein_coding 0.915 51.056 1.446
85.756 0.755 85.134 1.003 93.261
ENSMUSG00000031303 Map3k15 protein_coding 0.614 3.623 1.151

87.609	1.413 7.482 0.846 83.724
ENSMUSG00000031304	Il2rg protein_coding	1.297 7.179 1.058 92.561
0.728 0.945 1.01	88.387
ENSMUSG00000031309	Rps6ka3	protein_coding	1.114 24.689
1.105 91.643	0.813 23.212	0.994 95.676
ENSMUSG00000031310	Zmym3 protein_coding	0.938 36.034	1.007
96.847	1.059 47.31 0.971 86.445
ENSMUSG00000031311	Nono	protein_coding	1.006 85.283	0.943
42.923	1.05	9.502 1.029 46.412
ENSMUSG00000031312	Itgb1bp2	protein_coding	2.487 23.493
1.319 93.899	0.305 2.889 1.216 83.581
ENSMUSG00000031314	Taf1	protein_coding	1.018 74.8	0.938 85.242
1.051 42.199	0.994 87.886
ENSMUSG00000031320	Rps4x protein_coding	1.249 0	0.836 24.911
0.959 36.248	1.005 91.928
ENSMUSG00000031323	Dmrtc1a	protein_coding	0.388 27.431
0.763 92.474	3.375 23.41 1.127 92.31
ENSMUSG00000031327	Chic1 protein_coding	1.086 49.868	1.004
95.196	0.919 43.842	0.922 83.724
ENSMUSG00000031328	Flna	protein_coding	0.963 48.939	1.066
79.411	0.974 78.642	1.06	40.024
ENSMUSG00000031329	Tsx	protein_coding	1.127 86.658	NA	NA
0.577 60.614	0.715 87.735
ENSMUSG00000031333	Abcb7 protein_coding	1.05	66.58 0.994 95.301
0.958 77.704	1.035 59.464
ENSMUSG00000031337	Mtm1	protein_coding	1.151 7.842 0.948 86.604
0.918 18.797	0.988 87.735
ENSMUSG00000031340	Gabre protein_coding	1.212 65.733	0.643
84.017	1.284 36.578	1.256 31.133
ENSMUSG00000031342	Gpm6b protein_coding	1.058 52.799	0.942
87.147	1.003 75.637	1.111 78.379
ENSMUSG00000031343	Gabra3	protein_coding	1.17	13.292
0.858 86.604	0.992 35.815	1.104 67.169
ENSMUSG00000031344	Gabrq protein_coding	1.012 80.751	0.803
88.705	1.23	40.052	0.967 93.584
ENSMUSG00000031347	Cetn2 protein_coding	0.937 44.467	0.935
87.919	1.144 25.564	1.161 31.133
ENSMUSG00000031349	Nsdhl protein_coding	0.647 0.987 1.175 79.29
1.316 17.662	1.028 77.854
ENSMUSG00000031351	Zfp185	protein_coding	0.981 67.334	1.18
79.055	0.865 56.822	0.984 94.174
ENSMUSG00000031352	Hccs	protein_coding	1.083 29.596	1.054
82.904	0.877 1.92	0.958 96.183
ENSMUSG00000031353	Rbbp7 protein_coding	1.046 26.863	0.982
93.654	0.977 52.213	1.08	86.133
ENSMUSG00000031354	Amelx protein_coding	1	60.707	NA	NA
1	69.315	1	65.845
ENSMUSG00000031355	Arhgap6	protein_coding	1.271 0.355 0.88
78.51 0.895 3.654 1.075 95.676
ENSMUSG00000031357	Syap1 protein_coding	0.948 32.557	0.949
97.263	1.114 56.497	0.995 94.016
ENSMUSG00000031358	Msl3	protein_coding	1.017 86.819	1.007
96.68 0.976 82.575	1.002 89.108
ENSMUSG00000031360	Ctps2 protein_coding	1.036 32.411	1.014

94.848	0.952	39.528	0.952	88.053
ENSMUSG00000031362	Xlr4c	protein_coding	1.5	43.019	0.737
87.553	0.902	87.336	0.745	94.654
ENSMUSG00000031364	Grpr	protein_coding	1	74.958	1
97.263	1	87.744	1	96.071
ENSMUSG00000031365	Zfp275	protein_coding	0.931	29.972
1.154	60.309	0.931	38.434	1.096	79.545
ENSMUSG00000031367	Ap1s2	protein_coding	1.352	0.824	0.814	71.889
0.908	21.431	1.061	88.197
ENSMUSG00000031370	Zrsr2	protein_coding	0.929	27.431	0.963
82.073	1.121	13.259	1.079	83.865
ENSMUSG00000031371	Haus7	protein_coding	0.932	40.786	1.252
60.309	0.853	20.258	1.248	83.276
ENSMUSG00000031373	Car5b	protein_coding	0.736	1.343	0.979	89.277
1.387	6.449	0.942	85.493
ENSMUSG00000031374	Zfp92	protein_coding	0.697	77.615	1.437
84.126	1	85.21	1.305	86.133
ENSMUSG00000031375	Bgn	protein_coding	1.191	2.352	0.922	74.483
0.907	9.347	1.069	92.762
ENSMUSG00000031377	Bmx	protein_coding	1.471	0.2	0.804	44.566
0.844	9.901	0.967	88.552
ENSMUSG00000031378	Abcd1	protein_coding	0.989	84.534	0.965
81.284	1.046	52.574	1.061	60.968
ENSMUSG00000031379	Pir	protein_coding	1.003	64.014	0.81
61.883	1.229	18.411	0.912	96.192
ENSMUSG00000031380	Figf	protein_coding	1.051	29.372	0.915
85.95	1.036	78.642	1.193	59.464
ENSMUSG00000031381	Piga	protein_coding	0.884	30.136	1.081
97.155	1.045	36.578	1.122	44.882
ENSMUSG00000031382	Asb11	protein_coding	8.958	1.216	1.856	94.367
0.06	0.72	0.757	94.99
ENSMUSG00000031383	Dusp9	protein_coding	0.902	82.479	0.967
91.361	1.147	53.134	1.023	86.133
ENSMUSG00000031384	Asb9	protein_coding	1	72.985	1
95.504	1	84.83	1	91.335
ENSMUSG00000031385	Plxnb3	protein_coding	1.043	85.728
0.846	79.261	1.134	26.179	1.062	76.467
ENSMUSG00000031386	Hcfc1	protein_coding	1.022	74.147	1.056
71.617	0.928	3.147	NA	NA
ENSMUSG00000031387	Renbp	protein_coding	1.254	0	0.962	78.51
0.826	6.515	1.101	86.445
ENSMUSG00000031388	Naa10	protein_coding	0.898	23.641	1.036
96.216	1.077	29.568	1.021	94.153
ENSMUSG00000031389	Arhgap4	protein_coding	1.126	13.8	0.88
89.996	1.005	44.019	1.184	43.377
ENSMUSG00000031390	Avpr2	protein_coding	0.595	29.725	1.156
95.708	1.447	30.645	1.409	78.379
ENSMUSG00000031391	L1cam	protein_coding	0.992	85.59	0.978	87.041
1.03	69.825	0.96	88.387
ENSMUSG00000031392	Irak1	protein_coding	0.989	47.174	1.052
60.309	0.959	33.137	1.031	74.193
ENSMUSG00000031393	Mecp2	protein_coding	0.91	1.274	0.954	72.399
1.151	5.864	0.962	95.92
ENSMUSG00000031398	Plxna3	protein_coding	1.013	51.297

1.042 93.899 0.944 32.297 1.001 86.991
ENSMUSG00000031399 Fam3a protein_coding 0.96 61.633 1.031
92.793 1.012 70.9 0.998 95.593
ENSMUSG00000031400 G6pdx protein_coding 0.733 5.491 1.256 72.546
1.083 47.31 1.43 65.401
ENSMUSG00000031402 Mpp1 protein_coding 0.906 51.056 1.315
71.617 0.836 23.1 1.039 94.904
ENSMUSG00000031403 Dkc1 protein_coding 1.143 9.805 0.959 96.013
0.913 6.858 0.932 85.493
ENSMUSG00000031410 Nxf7 protein_coding 1.321 0.664 0.782 66.796
0.971 20.712 0.814 86.699
ENSMUSG00000031411 Prame protein_coding 1 77.615 1
95.941 1 79.554 1 96.192
ENSMUSG00000031422 Morf4l2 protein_coding 1.005 71.763
1.003 92.062 NA NA 1.173 31.133
ENSMUSG00000031424 Kir3dl1 protein_coding 1 83.263 1
90.378 1 64.024 1 94.904
ENSMUSG00000031425 Plp1 protein_coding 1.003 57.544 0.912
71.889 1.092 16.26 1.003 96.192
ENSMUSG00000031428 Zcchc18 protein_coding 1.392 43.939
0.729 70.693 0.988 86.475 0.815 86.991
ENSMUSG00000031429 Psmd10 protein_coding 0.986 68.955
0.999 97.155 1.017 57.286 1.029 77.621
ENSMUSG00000031430 Vsig1 protein_coding 1 75.858 NA NA
1 84.724 1 62.918
ENSMUSG00000031431 Tsc22d3 protein_coding 1.015 70.862
0.907 90.256 1.087 59.612 1.036 91.492
ENSMUSG00000031432 Prps1 protein_coding 1.376 0.461 0.999 92.931
0.73 0.879 1.068 73.939
ENSMUSG00000031433 Rbm41 protein_coding 1.085 57.655 NA NA
0.976 71.004 1.02 95.559
ENSMUSG00000031434 Morc4 protein_coding 1.028 64.956 1.111
84.832 0.876 31.887 0.905 83.724
ENSMUSG00000031438 Rnf128 protein_coding 1.229 32.411
1.051 94.102 0.772 44.678 0.913 94.001
ENSMUSG00000031441 Atp11a protein_coding 1.045 47.174
1.053 93.146 0.907 36.499 0.98 94.384
ENSMUSG00000031442 Mcf2l protein_coding 0.819 26.013 0.914
42.923 1.338 5.964 1.076 88.797
ENSMUSG00000031443 F7 protein_coding 0.791 63.126 0.873
94.848 1.442 56.931 1.472 65.401
ENSMUSG00000031444 F10 protein_coding 1.088 54.344 0.933
83.876 0.986 80.899 1.004 83.067
ENSMUSG00000031445 Proz protein_coding 1.327 49.868 0.61
65.165 1.236 59.612 0.788 92.561
ENSMUSG00000031446 Cul4a protein_coding 1.016 65.284 0.975
82.947 NA NA 0.992 95.273
ENSMUSG00000031447 Lamp1 protein_coding 1.019 68.727 1.021
83.104 0.963 42.997 1.032 83.276
ENSMUSG00000031448 Adprhl1 protein_coding 1.282 58.709
0.826 94.597 0.941 86.985 1.476 78.379
ENSMUSG00000031450 Grk1 protein_coding 0.855 71.142 0.812
64.123 1.443 29.819 1.315 72.369
ENSMUSG00000031451 Gas6 protein_coding 1.04 41.211 1.255

72.642	0.766 2.563 2.054 49.514
ENSMUSG00000031452	1700029H14Rik	protein_coding	0.988 85.355
1.725 83.464	0.587 32.963	1.128 87.088
ENSMUSG00000031453	Rasa3 protein_coding	1.057 25.207	0.972
97.263	0.972 26.369	1.075 91.783
ENSMUSG00000031458	Coprs protein_coding	0.998 86.464	1.114
82.904	0.903 39.079	1.136 79.713
ENSMUSG00000031461	Myom2 protein_coding	6.791 7.492 0.912 96.407
0.162 0.879 0.999 95.74
ENSMUSG00000031465	Angpt2	protein_coding	1.015 71.06 1.234
90.092	0.796 36.248	0.996 88.505
ENSMUSG00000031467	Agpat5	protein_coding	1.038 23.019
0.994 93.654	0.969 31.887	1.069 77.854
ENSMUSG00000031471	Defb8 protein_coding	1	86.145	1
95.35 1	80.089	1	93.612
ENSMUSG00000031478	Nek3	protein_coding	0.905 42.722	0.992
95.211	1.116 36.011	1.033 95.777
ENSMUSG00000031479	Vps36 protein_coding	1.001 75.115	0.917
55.265	1.088 15.049	1.044 53.923
ENSMUSG00000031480	Thsd1 protein_coding	0.942 67.163	1.04
95.735	1.017 77.17 1.068 85.577
ENSMUSG00000031481	Tpte	protein_coding	1.348 54.44 0.777 94.102
0.952 79.255	0.946 92.691
ENSMUSG00000031482	Slc25a15	protein_coding	1.054 37.091
1.057 75.108	0.897 3.147 1.038 75.198
ENSMUSG00000031483	Erlin2	protein_coding	1.028 15.178
1.027 93.27 0.943 15.63 1.009 94.567
ENSMUSG00000031485	Prosc protein_coding	1.036 35.787	0.995
96.216	0.969 23.938	0.993 95.559
ENSMUSG00000031486	Gpr124	protein_coding	1.365 1.216 0.947
90.68 0.774 1.705 1.207 50.74
ENSMUSG00000031487	Brf2	protein_coding	0.951 66.897	0.96
74.633	1.097 23.454	1.061 91.492
ENSMUSG00000031488	Rab11fip1	protein_coding	0.982 43.495
0.992 85.95 1.027 27.732	0.895 87.542
ENSMUSG00000031489	Adrb3 protein_coding	0.98	86.351	1.392
71.617	0.733 12.41 0.802 83.724
ENSMUSG00000031490	Eif4ebp1	protein_coding	0.908 16.895
1.049 79.261	1.049 85.134	0.925 83.724
ENSMUSG00000031491	Chrna6	protein_coding	1	81.178	1
95.735	1	72.378	1	95.74
ENSMUSG00000031492	Chrnb3	protein_coding	1	82.732	1
94.597	1	65.8	1	93.612
ENSMUSG00000031493	Ggn	protein_coding	0.963 82.231	0.831
79.261	1.25	54.163	0.882 90.157
ENSMUSG00000031494	Cd209a	protein_coding	0.844 81.465
1.276 95.708	0.929 79.625	1.072 96.248
ENSMUSG00000031495	Cd209d	protein_coding	1.261 2.567 0.801
54.427	0.991 75.637	1.173 79.268
ENSMUSG00000031497	Tnfsf13b	protein_coding	1.444 1.949 1.266
82.904	0.547 0.879 0.93	85.493
ENSMUSG00000031502	Col4a1	protein_coding	0.885 29.972
0.994 95.504	1.139 44.515	1.06	64.513
ENSMUSG00000031503	Col4a2	protein_coding	0.88	17.664	1.04

83.104 1.095 46.206 0.985 85.577
ENSMUSG00000031504 Rab20 protein_coding 0.855 32.168 1.009
94.89 1.155 55.352 0.98 96.358
ENSMUSG00000031505 Carkd protein_coding 0.897 3.895 1.004 96.013
1.112 7.635 1.008 91.011
ENSMUSG00000031506 Ptpn7 protein_coding 1.501 0 0.895 82.073
0.743 1.764 1.15 60.31
ENSMUSG00000031508 Ankrd10 protein_coding 0.972 80.51 0.993
95.329 1.036 83.275 1.023 94.825
ENSMUSG00000031509 1700016D06Rik protein_coding 1 71.763
1 96.843 1 83.158 1 93.734
ENSMUSG00000031510 1700128E19Rik lincRNA 1 84.564 1
95.504 NA NA 1 95.273
ENSMUSG00000031511 Arhgef7 protein_coding 1.003 67.991
1.046 72.642 0.95 4.759 1.03 94.904
ENSMUSG00000031512 Tex29 protein_coding 0.671 65.388 0.576
84.017 2.589 53.587 2.054 91.525
ENSMUSG00000031513 Leprotl1 protein_coding 1.006 43.019 NA
NA 0.97 54.25 1.039 79.545
ENSMUSG00000031516 Dctn6 protein_coding 1.025 48.288 0.938
53.79 1.036 66.928 1.045 70.36
ENSMUSG00000031517 Gpm6a protein_coding 1.494 0.2 0.734 39.547
0.91 27.321 0.996 91.525
ENSMUSG00000031518 Spata4 protein_coding 1 85.832 1
95.504 1 82.903 1 95.74
ENSMUSG00000031519 Asb5 protein_coding 3.258 16.436 1.906
83.104 0.161 3.432 0.767 95.676
ENSMUSG00000031520 Vegfc protein_coding 1.079 47.466 1.216
80.678 0.761 6.68 1.007 96.183
ENSMUSG00000031521 Aga protein_coding 0.972 85.728 1.008
88.691 1.016 63.665 1.032 80.497
ENSMUSG00000031523 Dlc1 protein_coding 0.886 14.645 1.011
97.155 1.117 45.208 0.967 93.612
ENSMUSG00000031527 Eri1 protein_coding 1.086 17.582 0.932
88.691 0.99 50.523 1.071 83.067
ENSMUSG00000031529 Tnks protein_coding 0.91 21.393 0.934
85.671 1.176 22.366 1.046 96.287
ENSMUSG00000031530 Dusp4 protein_coding 0.914 65.869 1.17
65.165 0.934 64.427 1.04 92.748
ENSMUSG00000031532 Tmem66 protein_coding 1 84.564
0.975 91.661 1.027 77.532 1.048 79.268
ENSMUSG00000031533 Mrps31 protein_coding 0.916 34.795
1.022 95.504 1.067 24.383 1.003 83.067
ENSMUSG00000031534 Smim19 protein_coding 0.922 45.229
1.044 90.116 1.041 69.002 1.005 92.742
ENSMUSG00000031535 Dkk4 protein_coding 1 70.597 NA NA
NA NA 1 94.904
ENSMUSG00000031536 Polb protein_coding 0.986 75.761 0.928
79.475 1.09 28.055 1.092 48.119
ENSMUSG00000031537 Ikbkb protein_coding 1.02 83.726 1.008
92.793 0.967 65.902 1.031 77.854
ENSMUSG00000031538 Plat protein_coding 1.32 8.365 NA NA
0.762 26.369 0.96 96.192
ENSMUSG00000031539 Ap3m2 protein_coding 1.078 20.659 0.986

95.35 0.943 22.735 1.015 94.82
ENSMUSG00000031540 Kat6a protein_coding 1.043 38.386 1.029
72.546 0.933 7.083 0.948 83.724
ENSMUSG00000031543 Ank1 protein_coding 3.199 3.152 1.396 87.029
0.223 0.72 1.132 91.783
ENSMUSG00000031545 Agpat6 protein_coding 0.986 72.743
0.882 79.807 1.148 25.808 0.997 88.505
ENSMUSG00000031546 Gins4 protein_coding 1 85.203 1.052
88.923 0.951 41.154 0.971 90.611
ENSMUSG00000031548 Sfrp1 protein_coding 1.209 23.019 0.994
95.196 0.83 15.63 1.152 72.369
ENSMUSG00000031549 Ido2 protein_coding 1.364 54.717 0.836
95.504 0.878 86.346 1.425 76.467
ENSMUSG00000031551 Ido1 protein_coding 1.609 13.105 0.808
95.708 0.767 22.864 1.078 92.748
ENSMUSG00000031553 Adam3 protein_coding 2.32 32.676 0.495
71.41 0.871 87.173 0.786 94.608
ENSMUSG00000031554 Adam5 protein_coding 2.273 1.43 0.516 51.159
0.854 77.037 0.912 95.273
ENSMUSG00000031555 Adam9 protein_coding 1.132 4.052 0.902 86.538
0.981 23.938 1.102 49.514
ENSMUSG00000031556 Tm2d2 protein_coding 0.943 21.658 1.109
60.309 0.956 42.376 0.973 91.269
ENSMUSG00000031557 Plekha2 protein_coding 1.184 0 0.961
64.123 0.879 1.764 1.074 48.119
ENSMUSG00000031558 Slit2 protein_coding 1.338 33.555 0.853
80.463 0.876 63.919 0.996 94.016
ENSMUSG00000031559 4930555F03Rik protein_coding 1 72.85 1
95.708 1 83.275 1 95.273
ENSMUSG00000031561 Tenm3 protein_coding 1.203 2.569 1.037 89.494
0.8 0.72 0.957 85.493
ENSMUSG00000031562 Dctd protein_coding 0.775 12.436 0.846
75.052 1.52 7.635 1.288 44.679
ENSMUSG00000031563 Wwc2 protein_coding 0.969 50.229 1.018
82.76 1.014 85.675 1.002 94.174
ENSMUSG00000031565 Fgfr1 protein_coding 0.988 61.633 0.983
96.216 1.028 54.673 0.953 83.724
ENSMUSG00000031568 Rwdd4a protein_coding 0.938 33.745
1.001 91.361 1.066 18.208 1.103 55.485
ENSMUSG00000031570 Ppapdc1b protein_coding 0.992 85.499
1.045 83.104 0.961 49.453 1.029 84.31
ENSMUSG00000031574 Star protein_coding 0.993 74.299 1.018
92.474 0.99 76.923 0.997 94.904
ENSMUSG00000031575 Ash2l protein_coding 1.008 80.751 0.948
79.261 1.044 23.41 1.041 44.882
ENSMUSG00000031576 Kcnu1 protein_coding 1.188 35.371 0.936
93.654 0.898 74.179 1.014 96.086
ENSMUSG00000031577 Tti2 protein_coding 0.993 84.738 1.001
94.367 1.008 73.598 0.983 87.542
ENSMUSG00000031578 Mak16 protein_coding 1.14 21.703 0.968
90.378 0.903 41.506 0.974 90.674
ENSMUSG00000031583 Wrn protein_coding 1.099 0.2 NA NA
0.917 1.061 0.925 83.724
ENSMUSG00000031584 Gsr protein_coding 0.912 72.985 0.882

82.848 1.244 27.475 1.128 56.863
ENSMUSG00000031585 Gtf2e2 protein_coding 1.135 4.675 0.94
86.728 0.938 12.907 1.032 86.301
ENSMUSG00000031586 Rbpms protein_coding 1.003 84.661 1.138
82.809 0.878 42.895 1.035 83.581
ENSMUSG00000031590 Frg1 protein_coding 0.945 60.707 0.987
97.263 1.07 79.119 1.098 63.114
ENSMUSG00000031591 Asah1 protein_coding 1.238 0 0.902 53.434
0.894 6.935 1.044 92.018
ENSMUSG00000031592 Pcm1 protein_coding 0.964 53.679 0.963
85.793 1.078 49.058 1.01 91.807
ENSMUSG00000031595 Pdgfrl protein_coding 0.845 20.799 0.99
93.707 1.196 42.75 0.895 88.552
ENSMUSG00000031596 Slc7a2 protein_coding 1.118 80.688
0.936 79.261 0.952 82.778 0.972 96.183
ENSMUSG00000031600 Vps37a protein_coding 0.964 63.283
0.946 70.29 1.1 24.383 1.022 95.74
ENSMUSG00000031601 Cnot7 protein_coding 0.978 81.01 1.067 87.282
0.956 80.749 0.971 90.95
ENSMUSG00000031603 Fgf20 protein_coding 0.776 65.869 1.089
96.984 1.181 65.235 0.639 83.724
ENSMUSG00000031604 Sc4mol protein_coding 1.078 64.401
1.089 79.411 0.852 13.643 1.114 69.708
ENSMUSG00000031605 Klhl2 protein_coding 1.002 77.497 1.084
83.104 0.923 10.602 0.868 83.724
ENSMUSG00000031608 Galnt7 protein_coding 1.197 0.664 0.886
20.272 0.945 50.523 1.015 91.783
ENSMUSG00000031609 Sap30 protein_coding 0.953 46.289 NA NA
1.068 61.171 1.073 77.331
ENSMUSG00000031613 Hpgd protein_coding 1.1 62.689 0.864
73.094 1.053 84.724 0.986 94.654
ENSMUSG00000031616 Ednra protein_coding 1.139 40.88 1.115 83.104
0.786 2.563 1.002 93.457
ENSMUSG00000031617 Tmem184c protein_coding 1.009 81.01 1.058
72.642 0.937 15.024 0.961 86.699
ENSMUSG00000031618 Nr3c2 protein_coding 1.13 22.349 0.945
95.708 0.935 11.116 0.962 87.992
ENSMUSG00000031621 Isx protein_coding 1 83.169 1
94.848 1 84.308 1 93.734
ENSMUSG00000031622 Sin3b protein_coding 0.917 2.142 1.017 76.634
1.069 47.776 1.027 91.525
ENSMUSG00000031626 Sorbs2 protein_coding 1.057 44.334
0.956 85.921 0.99 73.598 0.892 83.724
ENSMUSG00000031627 Irf2 protein_coding 0.933 77.763 1.107
96.216 0.969 85.757 0.952 83.724
ENSMUSG00000031628 Casp3 protein_coding 1.129 18.388 1.031
94.367 0.858 10.362 0.971 92.181
ENSMUSG00000031629 Mlf1ip protein_coding 1.17 26.77 0.77
71.41 1.111 77.037 0.94 88.505
ENSMUSG00000031631 4933411K20Rik protein_coding 1.02 53.562
1.029 97.263 0.955 64.778 1.014 95.273
ENSMUSG00000031633 Slc25a4 protein_coding 1.029 61.49 1.037
93.529 0.939 46.32 1.023 60.466
ENSMUSG00000031634 Ufsp2 protein_coding 1.008 67.608 0.983

95.504 1.009 88.693 1.039 61.316
ENSMUSG00000031635 Anxa10 protein_coding 1 81.01 1
95.504 1 85.935 1 96.192
ENSMUSG00000031636 Pdlim3 protein_coding 1.462 7.908 1.083
83.104 0.631 0.72 0.757 84.29
ENSMUSG00000031637 Lrp2bp protein_coding 1.748 17.582
1.576 88.136 0.364 3.943 0.859 90.805
ENSMUSG00000031639 Tlr3 protein_coding 1.07 32.411 1.044
75.21 0.893 5.087 0.996 96.139
ENSMUSG00000031641 Cbr4 protein_coding 1.051 84.977 0.893
67.739 1.066 26.978 0.972 92.12
ENSMUSG00000031642 Sh3rf1 protein_coding 1.114 7.908 NA NA
0.841 19.449 0.902 83.724
ENSMUSG00000031644 Nek1 protein_coding 1.087 15.602 0.923
79.894 0.995 80.015 0.984 93.132
ENSMUSG00000031647 Mfap3l protein_coding 0.993 85.092
1.139 71.98 0.885 12.41 0.99 95.74
ENSMUSG00000031651 NA NA NA NA 1 96.847 1
86.831 1 90.177
ENSMUSG00000031652 N4bp1 protein_coding 1.102 22.871 1.059
81.127 0.857 6.202 0.947 83.724
ENSMUSG00000031657 Heatr3 protein_coding 1.034 32.676
0.993 92.355 0.975 62.791 1.062 78.948
ENSMUSG00000031659 Adcy7 protein_coding 1.465 0 0.929 81.731
0.737 15.169 1.038 84.246
ENSMUSG00000031660 Brd7 protein_coding 0.969 19.985 0.964
95.708 1.066 33.042 1.082 37.634
ENSMUSG00000031661 Nkd1 protein_coding 1.064 39.075 0.993
96.763 0.95 57.446 0.945 90.611
ENSMUSG00000031662 Snx20 protein_coding 1.441 0 0.965 85.116
0.717 2.333 0.959 92.12
ENSMUSG00000031665 Sall1 protein_coding 1.545 57.133 0.674
86.071 0.958 75.009 1.234 93.457
ENSMUSG00000031666 Rbl2 protein_coding 1.067 54.344 0.971
95.329 0.964 57.446 0.996 94.608
ENSMUSG00000031667 Aktip protein_coding 1.044 7.083 1 92.561
0.96 8.382 1.076 59.441
ENSMUSG00000031668 Eif2ak3 protein_coding 0.998 63.385
1.014 89.014 0.984 37.103 1.124 40.729
ENSMUSG00000031669 Gins3 protein_coding 1.03 69.777 1.081
89.996 0.896 44.865 1.108 92.748
ENSMUSG00000031671 Setd6 protein_coding 0.983 73.487 NA NA
1.014 79.554 0.982 91.525
ENSMUSG00000031672 Got2 protein_coding 1.077 33.325 0.96
75.993 0.967 87.686 1.062 40.894
ENSMUSG00000031673 Cdh11 protein_coding 1.672 2.651 1.063 93.146
0.561 1.898 1.079 71.855
ENSMUSG00000031681 Smad1 protein_coding 1.001 74.147 1.016
95.088 0.979 68.584 0.955 84.29
ENSMUSG00000031683 Lsm6 protein_coding 1.064 35.748 0.972
95.504 0.965 69.134 1.007 84.499
ENSMUSG00000031684 Slc10a7 protein_coding 0.941 50.229
1.017 96.335 1.046 58.915 1.065 79.713
ENSMUSG00000031688 Pou4f2 protein_coding 1 86.74 1

91.661 1 68.129 1 94.904
ENSMUSG00000031691 Tnpo2 protein_coding 0.936 44.562 1.069
79.261 0.996 71.004 0.995 94.654
ENSMUSG00000031696 Vps35 protein_coding 1.084 29.596 0.977
92.474 0.943 18.122 1.017 68.502
ENSMUSG00000031697 Orc6 protein_coding 1.061 36.195 0.963
89.996 0.975 67.339 1.013 85.786
ENSMUSG00000031698 Mylk3 protein_coding 1.26 55.789 1.036
95.708 0.763 60.233 0.72 87.331
ENSMUSG00000031700 Gpt2 protein_coding 0.926 75.858 0.851
70.864 1.266 30.292 0.95 87.651
ENSMUSG00000031701 Dnaja2 protein_coding 0.991 81.52 0.997
88.345 1.006 69.002 1.114 31.133
ENSMUSG00000031703 Itfg1 protein_coding 0.962 46.693 1.003
93.654 1.037 41.235 1.012 91.269
ENSMUSG00000031706 Rfx1 protein_coding 0.875 2.822 1.064 78.705
1.076 20.556 1.034 81.251
ENSMUSG00000031708 Tecr protein_coding 0.671 2.142 1.198 82.904
1.247 10.615 1.012 93.722
ENSMUSG00000031709 Tbc1d9 protein_coding 1.083 37.414
0.928 78.877 0.993 86.475 0.98 86.699
ENSMUSG00000031710 Ucp1 protein_coding 3.323 22.025 0.826
95.504 0.365 30.024 1.727 95.877
ENSMUSG00000031711 Zfp330 protein_coding 1.072 12.985 0.9
8.462 1.033 61.418 1.041 75.81
ENSMUSG00000031712 Il15 protein_coding 1.179 8.365 0.914 86.277
0.932 40.19 1.072 90.397
ENSMUSG00000031714 Gab1 protein_coding 0.969 86.74 1.112 83.104
0.929 29.451 0.964 84.29
ENSMUSG00000031715 Smarca5 protein_coding 1.052 25.026
0.976 85.075 0.976 65.902 1.077 69.918
ENSMUSG00000031722 Hp protein_coding 0.867 40.786 0.823
60.678 1.399 22.366 1.375 49.239
ENSMUSG00000031723 Txnl4b protein_coding 0.911 4.385 1.063
88.91 1.034 41.806 1.05 81.56
ENSMUSG00000031725 Ces1f protein_coding 0.983 86.74 0.959 95.301
1.061 86.831 0.916 85.493
ENSMUSG00000031727 Pmfbp1 protein_coding 1.179 86.351
1.433 96.407 0.593 71.734 0.886 96.183
ENSMUSG00000031728 Zfp821 protein_coding 0.897 16.652
0.978 93.817 1.136 11.348 1.07 49.769
ENSMUSG00000031729 Ist1 protein_coding 0.97 80.751 1.025
93.908 1.006 75.155 1.043 50.452
ENSMUSG00000031730 Dhodh protein_coding 0.87 5.962 1.148 64.304
1.002 80.089 1.042 88.797
ENSMUSG00000031731 Ap1g1 protein_coding 1.094 6.399 1.016 96.001
0.899 13.202 1.001 91.525
ENSMUSG00000031732 Phlpp2 protein_coding 1.002 76.135
1.076 79.367 0.926 36.786 1.018 88.797
ENSMUSG00000031734 Irx3 protein_coding 1.295 35.499 0.949
85.075 0.813 64.778 0.806 83.724
ENSMUSG00000031736 4933436C20Rik lincRNA 0.966 85.655
0.734 70.864 1.405 37.741 0.989 92.181
ENSMUSG00000031737 Irx5 protein_coding 0.972 86.74 1.044 97.049

0.987 88.657 0.925 83.724
ENSMUSG00000031740 Mmp2 protein_coding 1.194 14.205 1.035
95.504 0.805 17.381 1.179 88.657
ENSMUSG00000031748 Gnao1 protein_coding 0.9 66.897 0.917
87.147 1.211 40.575 1.079 86.445
ENSMUSG00000031749 St3gal2 protein_coding 1.115 8.537 1.112
83.104 0.808 12.288 1.096 94.904
ENSMUSG00000031750 Il34 protein_coding 0.983 61.887 0.913
87.857 1.111 42.75 1.11 71.305
ENSMUSG00000031751 Amfr protein_coding 1.04 32.238 1.004
92.137 0.954 40.19 1.026 76.831
ENSMUSG00000031753 Cog4 protein_coding 0.965 75.298 0.998
90.256 1.037 56.931 1.037 80.324
ENSMUSG00000031754 Nudt21 protein_coding 1.035 54.344
0.997 95.211 0.971 62.533 0.982 91.625
ENSMUSG00000031755 Bbs2 protein_coding 1.093 35.371 0.876
61.883 1.041 63.801 0.912 83.724
ENSMUSG00000031756 Cenpn protein_coding 0.952 86.112 1.13
93.529 0.924 80.749 1.325 63.708
ENSMUSG00000031757 Mt4 protein_coding 1 84.452 1
96.378 1 86.985 1 93.316
ENSMUSG00000031758 Cdyl2 protein_coding 1.017 82.732 1.046
83.104 0.94 28.901 0.908 83.952
ENSMUSG00000031760 Mt3 protein_coding 0.666 17.898 1.193
91.275 1.256 19.286 0.783 84.29
ENSMUSG00000031762 Mt2 protein_coding 1.05 84.452 0.58
84.017 1.637 31.4 1.175 53.923
ENSMUSG00000031765 Mt1 protein_coding 0.922 51.089 0.915
88.897 1.182 38.038 0.986 94.153
ENSMUSG00000031766 Slc12a3 protein_coding 0.712 77.113 0.36
95.088 3.882 56.125 1.052 94.608
ENSMUSG00000031767 Nudt7 protein_coding 0.905 18.731 1.045
93.994 1.057 49.058 0.978 93.612
ENSMUSG00000031770 Herpud1 protein_coding 1.166 34.696 0.98
93.046 0.876 54.25 1.089 86.133
ENSMUSG00000031774 Fam192a protein_coding 0.977 59.674
0.993 96.948 1.025 79.325 1.034 86.209
ENSMUSG00000031775 Pllp protein_coding 1.09 29.536 0.978
95.708 0.94 51.055 0.97 87.496
ENSMUSG00000031776 Arl2bp protein_coding 0.97 63.283
1.032 95.809 0.996 85.792 1.034 87.7
ENSMUSG00000031778 Cx3cl1 protein_coding 0.979 83.726
0.996 89.434 1.022 66.201 0.828 83.724
ENSMUSG00000031779 Ccl22 protein_coding 1.274 54.717 0.819
91.83 0.961 84.669 0.633 86.445
ENSMUSG00000031780 Ccl17 protein_coding 0.974 66.129 1.528
96.984 NA NA 0.536 86.699
ENSMUSG00000031781 Ciapin1 protein_coding 0.909 3.009 1.059
86.793 1.037 45.449 1.015 87.886
ENSMUSG00000031782 Coq9 protein_coding 0.973 54.604 1.029
96.407 1 79.426 0.97 84.29
ENSMUSG00000031783 Polr2c protein_coding 1.012 79.277
1.005 94.597 0.986 86.985 1.01 96.192
ENSMUSG00000031785 Gpr56 protein_coding 0.873 5.778 NA NA

1.128 8.586 0.969 84.29
ENSMUSG00000031787 Katnb1 protein_coding 0.945 39.953
1.066 82.904 0.995 82.685 0.984 95.273
ENSMUSG00000031788 Kifc3 protein_coding 1 86.199 0.932
83.392 1.074 53.449 0.965 92.851
ENSMUSG00000031789 Cngb1 protein_coding 1.026 86.57 0.88 79.894
1.103 56.931 0.812 84.29
ENSMUSG00000031790 Mmp15 protein_coding 1.003 86.333 0.939
82.947 1.06 49.898 0.886 83.724
ENSMUSG00000031791 Tmem38a protein_coding 2.026 10.19 1.169
87.282 0.422 1.342 0.837 95.92
ENSMUSG00000031792 Usb1 protein_coding 1 77.497 0.952
81.731 1.054 70.9 1.07 93.016
ENSMUSG00000031796 Gtl3 protein_coding 1.079 37.702 1.036
92.561 0.897 40.19 0.856 83.724
ENSMUSG00000031799 Tpm4 protein_coding 0.977 82.092 1.076
87.609 0.95 45.093 1.178 40.729
ENSMUSG00000031802 Phxr4 protein_coding 0.875 61.043 0.867
95.504 1.316 55.512 0.993 93.316
ENSMUSG00000031803 B3gnt3 protein_coding 1.068 35.748
0.954 83.876 0.983 79.776 1.027 94.19
ENSMUSG00000031805 Jak3 protein_coding 0.869 7.887 1.148 83.104
0.999 58.616 1.052 94.153
ENSMUSG00000031807 Pgls protein_coding 0.9 5.946 1.027 96.216
1.084 30.107 1.005 94.904
ENSMUSG00000031808 Slc27a1 protein_coding 0.743 11.726
0.912 82.073 1.473 9.176 0.851 83.952
ENSMUSG00000031812 Map1lc3b protein_coding 1.056 9.695 0.954
73.766 0.994 63.919 1.025 95.832
ENSMUSG00000031813 Mvb12a protein_coding 1.004 71.142
0.981 79.261 1.011 54.163 1.048 44.882
ENSMUSG00000031816 Mthfsd protein_coding 0.921 4.055 0.986
90.576 1.105 7.635 1.053 71.305
ENSMUSG00000031818 Cox4i1 protein_coding 0.937 7.574 1.07
82.904 0.998 70.1 0.94 84.29
ENSMUSG00000031819 Emc8 protein_coding 0.859 14.221 1.035
95.809 1.126 30.561 0.991 85.493
ENSMUSG00000031820 Babam1 protein_coding 0.893 0.987 1.057
80.678 1.063 21.522 1.009 72.616
ENSMUSG00000031821 Gins2 protein_coding 0.985 59.028 1.045
94.262 0.973 69.196 0.952 94.654
ENSMUSG00000031822 Gse1 protein_coding 0.78 4.176 1.129 82.904
1.138 22.131 1.016 95.832
ENSMUSG00000031823 Zdhhc7 protein_coding 1.064 34.861
1.005 92.111 0.937 10.602 0.97 90.157
ENSMUSG00000031824 6430548M08Rik protein_coding 1.232 3.151 NA
NA 0.802 10.602 1.134 32.698
ENSMUSG00000031825 Crispld2 protein_coding 1.2 7.799 0.971
90.576 0.86 6.858 0.976 91.372
ENSMUSG00000031826 Usp10 protein_coding 0.885 4.825 1.086 75.62
1.035 39.471 1.044 83.067
ENSMUSG00000031827 Cotl1 protein_coding 1.385 0.767 0.918 81.731
0.786 6.68 1.182 38.598
ENSMUSG00000031828 Klhl36 protein_coding 1.061 45.096

1.046 96.216 0.9 30.468 0.957 85.493
ENSMUSG00000031831 Dnaaf1 protein_coding 1.224 70.597
0.594 83.58 1.37 40.511 1.277 85.629
ENSMUSG00000031832 Taf1c protein_coding 0.961 49.749 1.081
71.98 0.959 42.895 0.965 96.358
ENSMUSG00000031833 Mast3 protein_coding 0.944 38.983 0.945
87.452 1.118 27.475 1.037 95.35
ENSMUSG00000031834 Pik3r2 protein_coding 1.135 10.105
1.009 96.843 0.876 3.073 0.974 86.699
ENSMUSG00000031835 Mbtps1 protein_coding 1.007 76.546
0.996 92.355 1 82.436 0.977 96.192
ENSMUSG00000031837 Necab2 protein_coding 2.013 17.336
1.201 97.263 0.415 14.079 0.262 83.724
ENSMUSG00000031838 Ifi30 protein_coding 1.35 14.59 0.893 79.261
0.826 43.426 1.038 83.067
ENSMUSG00000031839 Hsbp1 protein_coding 0.926 9.863 1.037 79.864
1.042 55.512 1.089 73.823
ENSMUSG00000031840 Rab3a protein_coding 1.029 67.374 1.051
82.904 0.922 24.523 1.039 92.748
ENSMUSG00000031841 Cdh13 protein_coding 0.94 20.799 1.116
75.62 0.954 82.903 0.937 95.832
ENSMUSG00000031842 Pde4c protein_coding 0.998 51.056 0.862
68.848 1.163 17.662 0.879 92.31
ENSMUSG00000031843 Mphosph6 protein_coding 0.993 86.918
0.949 85.418 1.057 64.305 1.194 31.133
ENSMUSG00000031844 Hsd17b2 protein_coding 1 64.714 1
97.155 1 86.144 1 91.783
ENSMUSG00000031845 Bcmo1 protein_coding 1.156 81.657 1.145
93.27 0.753 64.155 1.039 72.204
ENSMUSG00000031847 1700030J22Rik protein_coding 1.094 84.168
1.128 79.29 0.808 12.948 0.792 83.952
ENSMUSG00000031848 Lsm4 protein_coding 0.912 18.602 1.002
95.211 1.095 56.708 1.083 90.455
ENSMUSG00000031849 Comp protein_coding 0.986 81.178 0.788
66.651 1.291 33.787 0.935 88.053
ENSMUSG00000031851 Ntpcr protein_coding 0.982 84.865 0.973
94.597 1.043 72.148 1.099 74.193
ENSMUSG00000031853 BC021891 protein_coding 1.041 57.949 1.15
89.494 0.836 22.735 0.845 83.724
ENSMUSG00000031858 Mau2 protein_coding 0.992 46.693 1.006
92.561 1.004 84.504 0.974 93.316
ENSMUSG00000031860 Pbx4 protein_coding 0.976 72.139 NA NA
0.98 83.275 1.003 95.832
ENSMUSG00000031861 Lpar2 protein_coding 0.928 80.251 0.898
85.418 1.198 33.287 0.998 92.181
ENSMUSG00000031862 Atp13a1 protein_coding 0.927 27.202
1.039 83.104 1.039 58.414 1.059 72.826
ENSMUSG00000031864 Ints10 protein_coding 0.917 24 1.062
83.464 1.028 84.626 1.047 43.377
ENSMUSG00000031865 Dctn1 protein_coding 0.915 5.778 1.032 96.847
1.055 16.446 1.005 94.904
ENSMUSG00000031870 Pgr protein_coding 0.872 81.647 1.218
79.864 0.938 54.964 1.615 73.823
ENSMUSG00000031871 Cdh5 protein_coding 0.928 61.887 1.085

95.431 0.996 82.161 0.929 83.724
ENSMUSG00000031872 Bean1 protein_coding 0.899 58.802 0.739
87.322 1.509 30.645 1.136 82.328
ENSMUSG00000031875 Cmtm3 protein_coding 1.316 0.2 1.065 95.735
0.713 1.898 0.978 96.358
ENSMUSG00000031876 Cmtm1 protein_coding 1 72.743 1
97.263 1 86.727 1 96.183
ENSMUSG00000031877 Ces2g protein_coding 1.127 11.879 1.044
88.823 0.849 10.726 1.136 44.679
ENSMUSG00000031878 Nae1 protein_coding 1.079 13.8 0.961 79.475
0.966 73.027 0.957 89.892
ENSMUSG00000031879 Fam96b protein_coding 0.901 21.658
1.071 81.127 1.04 84.308 1.096 94.825
ENSMUSG00000031880 Rrad protein_coding 1.543 15.178 1.067
83.104 0.605 0.72 0.739 83.724
ENSMUSG00000031881 Cdh16 protein_coding 1 81.01 1 97.129
1 85.792 1 93.627
ENSMUSG00000031883 Car7 protein_coding 1.072 59.527 1.065
83.104 0.874 10.726 0.906 86.991
ENSMUSG00000031885 Cbfb protein_coding 1.127 0 1.058 96.763
0.838 0.72 1.047 71.305
ENSMUSG00000031886 Ces2e protein_coding 0.803 36.945 1.374
81.039 0.907 82.575 0.933 92.018
ENSMUSG00000031887 Tradd protein_coding 0.963 59.674 0.961
95.301 1.083 72.148 1.073 89.152
ENSMUSG00000031889 D230025D16Rik protein_coding 0.919 77.615
0.865 67.513 1.254 30.213 1.068 95.273
ENSMUSG00000031891 Hsd11b2 protein_coding 1.021 84.738
1.933 72.642 0.508 29.451 1.454 44.679
ENSMUSG00000031893 Tsnaxip1 protein_coding 0.625 45.728 NA
NA 0.746 86.666 1.121 94.99
ENSMUSG00000031896 Ctrl protein_coding 1.221 50.627 1.26
87.609 0.648 28.669 0.89 91.372
ENSMUSG00000031897 Psmb10 protein_coding 1.078 37.941
1.026 86.277 0.9 3.718 1.012 81.061
ENSMUSG00000031898 NA NA NA NA 1.696 71.617 0.67
19.765 1.443 93.264
ENSMUSG00000031901 Dus2l protein_coding 0.799 21.957 1.106
71.222 1.127 78.155 1.006 91.928
ENSMUSG00000031902 Nfatc3 protein_coding 1.056 22.871 1
87.037 0.948 6.858 0.958 86.991
ENSMUSG00000031903 Pla2g15 protein_coding 1.608 0 0.919
90.378 0.676 0.72 0.863 83.952
ENSMUSG00000031904 Slc7a6 protein_coding 1.126 10.464
0.955 95.211 0.931 21.431 0.949 83.724
ENSMUSG00000031906 Smpd3 protein_coding 0.969 61.633 0.896
91.955 1.148 19.877 0.966 96.071
ENSMUSG00000031907 Zfp90 protein_coding 1.114 16.74 0.914 75.345
0.979 79.255 1.009 96.086
ENSMUSG00000031910 Has3 protein_coding 0.868 30.637 1.235
82.904 0.933 82.685 0.854 83.724
ENSMUSG00000031913 Vps4a protein_coding 0.905 0.987 1.034 83.464
1.065 13.259 1.031 69.404
ENSMUSG00000031916 Cog8 protein_coding 0.999 58.077 1.035

71.617 0.965 82.31 0.943 91.65
ENSMUSG00000031917 Nip7 protein_coding 1.144 11.316 0.945
75.345 0.927 33.137 1.082 94.904
ENSMUSG00000031918 Mtmr2 protein_coding 1.089 9.435 1.019 95.504
0.902 24.309 1.013 60.968
ENSMUSG00000031921 Terf2 protein_coding 0.974 71.36 1.01 94.887
1.014 74.082 0.974 86.699
ENSMUSG00000031922 Cep57 protein_coding 0.995 62.007 0.976
81.02 1.033 56.497 1.01 85.577
ENSMUSG00000031924 Cyb5b protein_coding 0.88 14.221 1.082
82.76 NA NA 0.932 85.493
ENSMUSG00000031925 Maml2 protein_coding 1.188 0.577 0.973 87.147
0.866 4.521 0.951 90.854
ENSMUSG00000031927 1700012B09Rik protein_coding 1 84.738
1 95.708 1 85.792 1 94.153
ENSMUSG00000031928 Mre11a protein_coding 1.074 25.026
0.914 60.678 1.018 76.225 1.1 67.691
ENSMUSG00000031930 Wwp2 protein_coding 1.109 5.253 0.975 79.475
0.923 26.369 0.994 93.734
ENSMUSG00000031931 Ankrd49 protein_coding 1.074 9.206 0.979
81.731 0.948 28.237 1.084 68.696
ENSMUSG00000031933 Folr4 protein_coding 0.62 80.688 1.289
94.597 1.247 84.724 2.356 88.197
ENSMUSG00000031934 Panx1 protein_coding 1.164 18.314 0.967
92.137 0.886 42.895 1.178 72.116
ENSMUSG00000031935 Med17 protein_coding 1.067 1.99 1.031 96.68
0.911 1.898 0.964 84.29
ENSMUSG00000031936 Hephl1 protein_coding 0.241 1.15 2.048
82.904 2.021 8.586 0.799 94.468
ENSMUSG00000031937 Vstm5 protein_coding 0.941 66.897 NA NA
1.104 78.155 0.96 95.273
ENSMUSG00000031938 4931406C07Rik protein_coding 0.927 46.693
0.993 96.897 1.087 32.433 1.023 91.525
ENSMUSG00000031939 Taf1d protein_coding 1.111 13.105 0.916
55.71 0.979 82.161 1.134 73.823
ENSMUSG00000031948 Kars protein_coding 0.983 50.768 1.001
92.062 1.013 81.032 1.036 60.968
ENSMUSG00000031949 Adat1 protein_coding 0.913 27 0.996 93.817
1.099 35.673 0.94 94.608
ENSMUSG00000031950 Gabarapl2 protein_coding 0.96 43.816
0.986 91.955 1.055 31.479 1.053 66.944
ENSMUSG00000031951 Tmem231 protein_coding 1.033 78.639
0.991 96.984 0.978 79.524 0.914 86.991
ENSMUSG00000031953 Tmem170 protein_coding 1.056 67.374
1.002 96.335 0.941 27.86 0.869 83.724
ENSMUSG00000031954 Cfdp1 protein_coding 0.991 68.727 0.957
68.115 1.051 70.9 1.134 31.133
ENSMUSG00000031955 Bcar1 protein_coding 0.989 63.283 1.087
72.642 0.93 24.163 0.973 85.493
ENSMUSG00000031958 Ldhd protein_coding 0.798 5.778 0.997 94.597
1.257 6.449 0.952 87.331
ENSMUSG00000031959 Wdr59 protein_coding 0.905 9.841 1.022 86.387
1.082 22.994 1.006 94.153
ENSMUSG00000031960 Aars protein_coding 0.872 2.5 1.066 80.775

1.071 40.575 1.131 57.075
ENSMUSG00000031962 Cdh15 protein_coding 2.271 25.421 NA NA
0.47 15.376 1.575 90.463
ENSMUSG00000031963 Bmper protein_coding 1.384 8.281 0.982 89.996
0.733 33.971 1.187 52.324
ENSMUSG00000031965 Tbx20 protein_coding 1 85.092 1
96.407 1 86.602 1 93.413
ENSMUSG00000031966 Glb1l3 protein_coding 1.506 60.435
0.491 79.475 1.353 77.532 1.374 90.674
ENSMUSG00000031967 Afg3l1 protein_coding 0.957 2.436 1.032
90.29 1.015 50.091 1.024 66.944
ENSMUSG00000031969 Acad8 protein_coding 0.992 70.36 1.051 92.062
0.961 45.573 0.974 86.445
ENSMUSG00000031970 Dbndd1 protein_coding 0.61 5.967 0.979
88.691 1.669 5.864 0.955 93.734
ENSMUSG00000031971 Ccsap protein_coding 0.872 82.092 0.976
87.919 1.17 60.104 1.038 96.287
ENSMUSG00000031972 Acta1 protein_coding 7.091 7.174 0.966 83.104
0.146 0.72 0.913 96.358
ENSMUSG00000031974 Abcb10 protein_coding 0.905 8.709 1.091
71.617 1.013 87.971 0.978 94.654
ENSMUSG00000031976 Urb2 protein_coding 1.061 62.007 1.011
88.881 0.929 87.744 0.988 94.904
ENSMUSG00000031979 Cog2 protein_coding 0.996 43.495 0.974
91.955 1.027 37.741 0.97 93.612
ENSMUSG00000031980 Agt protein_coding 1.31 2.446 0.901 82.503
0.844 10.003 0.964 92.748
ENSMUSG00000031981 Capn9 protein_coding 0.587 11.707 1.682
89.434 1.015 60.104 1.082 94.904
ENSMUSG00000031982 Arv1 protein_coding 0.997 72.985 1.039
89.494 0.966 86.346 0.902 85.493
ENSMUSG00000031983 2310022B05Rik protein_coding 1.132 2.352
0.935 62.322 0.947 11.997 0.993 95.273
ENSMUSG00000031984 2810004N23Rik protein_coding 0.87 8.45
0.976 94.48 1.17 10.819 1.135 46.412
ENSMUSG00000031985 Gnpat protein_coding 1.037 18.067 1.072
75.612 0.899 5.087 0.879 83.724
ENSMUSG00000031986 Sprtn protein_coding 0.974 49.749 1.107
82.904 0.929 46.071 0.954 85.636
ENSMUSG00000031987 Egln1 protein_coding 0.996 73.841 1.095
72.642 0.919 6.858 1.008 93.612
ENSMUSG00000031988 Vps26b protein_coding 1.012 41.211 1.03
79.29 0.955 5.564 0.96 83.724
ENSMUSG00000031990 Jam3 protein_coding 1.056 47.174 1.161
71.98 0.813 6.935 1.111 71.305
ENSMUSG00000031991 Spata19 protein_coding 1 79.165 1
89.014 1 88.693 1 96.248
ENSMUSG00000031993 Snx19 protein_coding 1.011 83.523 1.021
96.948 0.966 85.21 1.049 52.324
ENSMUSG00000031994 Adamts8 protein_coding 1.162 33.325
1.062 90.389 0.812 14.409 1.096 73.823
ENSMUSG00000031995 St14 protein_coding 0.937 54.983 0.969
90.378 1.1 45.035 0.951 91.291
ENSMUSG00000031996 Aplp2 protein_coding 0.949 25.492 1.007

95.504 1.047 45.208 0.998 96.358
ENSMUSG00000031997 Trpc6 protein_coding 0.884 68.955 1.215
86.387 0.93 73.096 1.089 72.369
ENSMUSG00000031998 1700128F08Rik protein_coding 1 77.291
1 96.984 NA NA 1 93.685
ENSMUSG00000032000 Birc3 protein_coding 1.122 9.206 0.908 52.085
0.98 78.954 0.959 84.19
ENSMUSG00000032002 Dcun1d5 protein_coding 1.017 57.949
1.014 90.046 0.965 87.431 1.023 87.7
ENSMUSG00000032006 Pdgfd protein_coding 1.021 27.731 1.018
82.947 0.961 85.21 0.988 94.608
ENSMUSG00000032009 Sesn3 protein_coding 0.999 69.24 0.956 96.843
1.049 83.719 0.996 93.734
ENSMUSG00000032010 Usp2 protein_coding 0.661 66.406 1.216
96.216 1.246 69.196 1.524 59.208
ENSMUSG00000032011 Thy1 protein_coding 1.373 1.441 1.038 92.793
0.702 1.764 1.236 31.133
ENSMUSG00000032012 Pvrl1 protein_coding 1.072 58.709 NA NA
0.966 48.133 1.02 92.018
ENSMUSG00000032013 Trim29 protein_coding 0.927 27.202 NA
NA 1.061 39.925 0.749 83.724
ENSMUSG00000032014 Oaf protein_coding 1.076 61.171 0.949
87.857 0.981 86.602 1.205 31.133
ENSMUSG00000032015 Pou2f3 protein_coding 1.148 52.134 NA
NA 0.927 87.898 0.732 95.72
ENSMUSG00000032018 Sc5d protein_coding 1.096 37.414 1.165
79.411 0.782 1.898 0.944 90.157
ENSMUSG00000032020 Ubash3b protein_coding 1.327 18.388
0.998 96.308 0.756 13.848 1.02 75.198
ENSMUSG00000032021 Crtam protein_coding 0.955 82.092 1.54
92.062 0.679 59.125 0.313 83.724
ENSMUSG00000032023 4931429I11Rik protein_coding 1 85.129
1 82.503 1 47.935 1 84.499
ENSMUSG00000032024 Clmp protein_coding 1.143 58.709 1.16
71.617 0.754 39.245 0.839 85.493
ENSMUSG00000032026 Rexo2 protein_coding 0.85 0.987 1.051 80.678
1.114 27.431 1.099 90.397
ENSMUSG00000032028 Nxpe2 protein_coding 1.117 52.134 1.084
79.579 0.824 6.858 1.012 95.72
ENSMUSG00000032030 Cul5 protein_coding 1.097 24.147 0.965
96.138 0.947 17.381 1.042 87.134
ENSMUSG00000032033 Barx2 protein_coding 1.005 80.751 1.036
96.407 0.958 84.961 0.894 83.724
ENSMUSG00000032035 Ets1 protein_coding 1.111 16.249 1.086
77.618 0.831 4.723 0.838 83.724
ENSMUSG00000032036 Kirrel3 protein_coding 1.122 61.49 0.916
94.48 NA NA 1.033 90.463
ENSMUSG00000032038 St3gal4 protein_coding 0.875 33.599
1.019 96.138 1.124 27.89 1.121 76.831
ENSMUSG00000032040 Dcps protein_coding 0.963 51.56 0.962 86.071
1.073 20.085 1.008 95.676
ENSMUSG00000032041 Tirap protein_coding 1.167 1.412 0.981 88.691
0.875 9.456 1.026 87.447
ENSMUSG00000032042 Srpr protein_coding 0.977 82.092 1.034

90.092 0.984 47.12 1.124 35.053
ENSMUSG00000032044 Rpusd4 protein_coding 1.14 0.461 0.92
54.427 0.955 40.338 1.043 58.557
ENSMUSG00000032046 Abhd12 protein_coding 1.08 2.998 0.97
82.073 0.954 18.965 0.984 88.505
ENSMUSG00000032047 Acat1 protein_coding 1.073 37.702 0.997
96.216 0.938 28.237 0.999 94.567
ENSMUSG00000032048 4930510E17Rik protein_coding 1 84.819
1 94.96 1 88.693 1 94.654
ENSMUSG00000032050 Rdx protein_coding 1.057 13.633 0.974
85.075 0.972 46.32 1.065 65.845
ENSMUSG00000032051 Fdx1 protein_coding 0.911 42.361 1.024
94.102 1.073 35.673 0.952 86.699
ENSMUSG00000032053 Pou2af1 protein_coding 1 73.487
1.239 82.904 0.807 59.125 1.107 86.209
ENSMUSG00000032056 Btg4 protein_coding 1 68.846 1
93.654 1 65.567 1 93.964
ENSMUSG00000032058 Ppp2r1b protein_coding 1.124 29.596
1.062 78.705 0.834 6.858 0.828 83.724
ENSMUSG00000032059 Alg9 protein_coding 0.986 56.768 1.082
79.055 0.939 46.575 1.009 92.561
ENSMUSG00000032060 Cryab protein_coding 0.644 0.987 1.288 60.309
1.208 24.383 1.128 63.708
ENSMUSG00000032062 2310030G06Rik protein_coding 0.849 34.162
0.992 96.407 1.19 39.471 1.028 93.132
ENSMUSG00000032064 Dixdc1 protein_coding 0.813 6.822 1.063
79.055 1.155 74.686 0.922 83.724
ENSMUSG00000032066 Bco2 protein_coding 1.025 58.709 0.849
80.463 1.146 65.8 1.136 79.442
ENSMUSG00000032067 Pts protein_coding 0.987 86.145 0.946
61.883 1.07 43.307 0.967 84.29
ENSMUSG00000032068 1600029D21Rik protein_coding 0.847 58.56
0.943 88.691 1.247 33.802 1.01 95.74
ENSMUSG00000032076 Cadm1 protein_coding 1.163 6.541 0.993 87.147
0.867 16.643 0.921 85.493
ENSMUSG00000032077 Bud13 protein_coding 0.989 65.869 0.996
97.049 1.017 88.076 1.003 95.273
ENSMUSG00000032078 Zfp259 protein_coding 0.949 43.816
0.953 75.345 1.103 14.597 1.058 63.114
ENSMUSG00000032079 Apoa5 protein_coding 1 71.142 NA NA
1 85.134 1 95.35
ENSMUSG00000032080 Apoa4 protein_coding 1 66.897 1
85.756 1 85.134 1 90.455
ENSMUSG00000032081 Apoc3 protein_coding 0.648 5.084 1.241 75.108
1.242 43.842 0.768 91.269
ENSMUSG00000032085 Tagln protein_coding 0.936 49.621 1.044
88.91 1.026 84.626 1.04 93.132
ENSMUSG00000032086 Bace1 protein_coding 1.04 65.537 1.058
71.617 0.907 5.323 1.011 95.273
ENSMUSG00000032089 Il10ra protein_coding 1.45 7.255 0.957
89.277 0.722 27.86 0.898 88.387
ENSMUSG00000032091 Tmprss4 protein_coding 1.369 4.675 0.674
34.058 1.087 83.066 1.111 74.896
ENSMUSG00000032092 Mpzl2 protein_coding 0.935 41.021 1.003

83.876 1.067 22.517 0.891 86.991
ENSMUSG00000032093 Cd3e protein_coding 0.896 76.686 1.207
84.832 0.925 64.155 0.961 90.674
ENSMUSG00000032094 Cd3d protein_coding 1.018 84.263 1.199
83.104 0.817 40.338 0.753 83.724
ENSMUSG00000032096 Arcn1 protein_coding 1.094 2.998 0.989 97.155
0.924 0.72 1.079 72.116
ENSMUSG00000032097 Ddx6 protein_coding 1.075 0.355 0.963 56.955
0.964 25.052 0.985 93.612
ENSMUSG00000032099 Pate4 protein_coding 1 77.763 1
96.843 1 77.17 1 92.748
ENSMUSG00000032101 Ddx25 protein_coding 0.491 27.664 1.811
80.277 1.122 63.801 1.014 91.783
ENSMUSG00000032103 Pus3 protein_coding 1.101 38.195 0.902
65.165 1.006 72.853 0.993 93.261
ENSMUSG00000032105 Pdzd3 protein_coding 1.035 76.827 1.121
92.931 0.863 38.534 0.852 83.724
ENSMUSG00000032109 Nlrx1 protein_coding 0.909 22.431 NA NA
1.047 28.74 0.944 93.612
ENSMUSG00000032112 Trappc4 protein_coding 0.868 3.07 1.087
84.832 1.057 25.966 1.002 92.52
ENSMUSG00000032113 Chek1 protein_coding 1.494 28.693 0.798
76.431 0.84 74.535 1.301 44.882
ENSMUSG00000032114 Slc37a4 protein_coding 0.891 9.599 1.116
82.904 1.003 69.315 1.057 83.067
ENSMUSG00000032115 Hyou1 protein_coding 0.782 17.062 1.046
90.116 1.223 25.455 1.247 44.882
ENSMUSG00000032116 Stt3a protein_coding 1.068 6.61 1.029 96.984
0.907 5.564 1.117 44.679
ENSMUSG00000032118 Fez1 protein_coding 1.266 0.461 0.908 77.598
0.869 17.898 1.052 90.177
ENSMUSG00000032119 Hinfp protein_coding 1.022 81.925 0.983
91.532 0.993 63.665 0.96 92.181
ENSMUSG00000032120 C2cd2l protein_coding 0.643 1.701 1.125
82.904 1.385 7.635 1.024 93.132
ENSMUSG00000032121 Tmem218 protein_coding 1.016 79.068
1.024 96.308 0.958 52.213 0.917 83.724
ENSMUSG00000032122 Slc37a2 protein_coding 1.626 0.461 0.924
52.085 0.667 15.904 1.032 96.183
ENSMUSG00000032123 Dpagt1 protein_coding 0.986 85.59 1.1
83.104 0.92 33.971 1.025 90.674
ENSMUSG00000032125 Robo4 protein_coding 0.801 4.115 1.004 93.654
1.247 6.506 0.966 92.181
ENSMUSG00000032126 Hmbs protein_coding 0.853 0.987 1.131 75.62
1.038 30.213 1.116 44.882
ENSMUSG00000032127 Vps11 protein_coding 1.009 68.485 0.969
87.857 1.023 69.315 1.031 68.502
ENSMUSG00000032128 Robo3 protein_coding 1.497 53.228 0.938
94.597 0.711 65.409 1.022 92.181
ENSMUSG00000032131 Abcg4 protein_coding 0.702 1.214 1.29 60.309
1.106 44.515 1.015 95.877
ENSMUSG00000032134 Muc16 protein_coding 0.799 55.641 1.152
86.156 1.084 87.898 0.569 83.724
ENSMUSG00000032135 Mcam protein_coding 0.892 55.641 1.151

83.104 0.97 84.669 0.929 91.492
ENSMUSG00000032171 Pin1 protein_coding 0.962 27.431 0.99
83.104 1.051 53.134 1.008 92.903
ENSMUSG00000032172 Olfm2 protein_coding 1.419 41.211 1.127
84.007 0.627 23.938 1.33 83.276
ENSMUSG00000032174 Icam5 protein_coding 0.951 62.007 0.947
90.256 1.105 88.601 1.309 73.368
ENSMUSG00000032175 Tyk2 protein_coding 0.986 77.182 NA NA
0.975 81.62 0.947 88.505
ENSMUSG00000032177 Pde4a protein_coding 0.994 67.163 1.023
79.864 0.98 75.637 0.985 89.892
ENSMUSG00000032178 Ilf3 protein_coding 0.99 86.333 1.016
97.049 0.99 70.44 1.017 83.067
ENSMUSG00000032179 Bmp5 protein_coding 1.589 7.799 0.862 90.378
0.731 6.858 0.989 88.136
ENSMUSG00000032180 Tmed1 protein_coding 0.976 74.8 1.083 76.086
0.947 37.617 1.067 57.1
ENSMUSG00000032181 Scg3 protein_coding 1.877 1.161 0.565 64.555
0.944 38.534 0.975 94.19
ENSMUSG00000032182 Yipf2 protein_coding 0.95 17.401 1.027
86.387 1.028 62.307 0.984 91.625
ENSMUSG00000032184 Lysmd2 protein_coding 0.992 85.283
1.001 94.848 1.006 88.076 1.027 90.674
ENSMUSG00000032185 Carm1 protein_coding 1.001 82.092 1.022
95.431 0.978 79.255 1.007 87.134
ENSMUSG00000032186 NA NA NA NA 0.895 80.052 1.126
65.567 1.111 70.624
ENSMUSG00000032187 Smarca4 protein_coding 0.93 16.472
1.035 94.367 1.04 43.842 1 87.331
ENSMUSG00000032191 Bcl2l10 protein_coding 0.964 68.955
0.877 85.242 1.184 45.94 0.666 90.157
ENSMUSG00000032192 Gnb5 protein_coding 1.06 23.82 1.069 93.146
0.88 6.858 0.995 92.181
ENSMUSG00000032193 Ldlr protein_coding 0.982 82.891 1.321
72.546 0.771 20.009 1.113 93.722
ENSMUSG00000032194 Kank2 protein_coding 1.178 19.893 1.072
67.843 0.79 0.72 1.046 60.466
ENSMUSG00000032198 Dock6 protein_coding 0.956 46.693 0.973
95.301 1.073 47.512 0.956 86.699
ENSMUSG00000032199 Polr2m protein_coding 1.026 50.111
0.987 96.001 0.986 48.851 0.999 96.248
ENSMUSG00000032202 Rab27a protein_coding 0.948 86.843
0.929 55.265 1.136 28.74 1.121 87.7
ENSMUSG00000032203 Pigb protein_coding 1 80.251 1
97.263 1 86.346 1 96.139
ENSMUSG00000032204 Aqp9 protein_coding 1.293 51.826 0.727
27.163 1.064 45.667 0.665 83.952
ENSMUSG00000032207 Lipc protein_coding 0.798 13.45 0.894 92.404
1.405 24.07 0.832 87.735
ENSMUSG00000032212 Sltm protein_coding 1.053 35.371 0.936
73.094 NA NA 0.971 91.291
ENSMUSG00000032215 Rsl24d1 protein_coding 1.113 21.703 0.95
96.001 0.948 26.057 0.985 95.777
ENSMUSG00000032216 Nedd4 protein_coding 1.128 0.664 0.958 84.594

0.926 8.027 1.015 94.904
ENSMUSG00000032217 Rnf111 protein_coding 1.052 4.874 1.019
96.407 0.931 6.858 0.996 92.181
ENSMUSG00000032218 Ccnb2 protein_coding 1.635 48.288 0.604
20.174 1.015 84.83 1.216 82.85
ENSMUSG00000032220 Myo1e protein_coding 0.966 77.763 0.943
90.138 1.093 68.129 0.947 83.724
ENSMUSG00000032221 Mns1 protein_coding 0.829 86.843 0.9
93.654 1.334 78.155 1.748 90.865
ENSMUSG00000032224 Fam81a protein_coding 1.448 2.784 0.919
94.597 0.75 6.858 0.955 93.685
ENSMUSG00000032228 Tcf12 protein_coding 1.051 36.195 1.018
92.931 0.931 38.799 1.005 95.676
ENSMUSG00000032231 Anxa2 protein_coding 0.855 1.422 1.066 86.156
1.097 36.95 1.185 40.426
ENSMUSG00000032232 Cgnl1 protein_coding 0.964 65.622 1
95.211 1.034 76.498 0.957 83.724
ENSMUSG00000032235 Narg2 protein_coding 1.008 52.134 0.948
75.345 1.049 57.286 1.011 91.872
ENSMUSG00000032238 Rora protein_coding 0.932 44.922 0.758
73.461 1.414 10.76 1.002 92.675
ENSMUSG00000032239 Rp9 protein_coding 0.99 86.145 0.979
75.493 1.033 45.208 0.953 87.218
ENSMUSG00000032243 Itga11 protein_coding 0.963 73.585
0.918 79.475 1.125 38.26 0.94 83.952
ENSMUSG00000032244 Fem1b protein_coding 1.067 39.416 1.015
90.116 0.922 32.703 0.972 95.877
ENSMUSG00000032245 Cln6 protein_coding 1.26 0.664 0.849 52.266
0.938 9.563 1.019 95.877
ENSMUSG00000032246 Calml4 protein_coding 1.88 2.047 0.83
96.216 0.641 8.472 1.329 57.075
ENSMUSG00000032249 Anp32a protein_coding 0.96 34.954
0.885 26.151 1.175 6.506 1.05 78.042
ENSMUSG00000032251 Irak1bp1 protein_coding 0.988 71.915 1.04
93.654 0.975 64.024 1.011 96.287
ENSMUSG00000032252 Glce protein_coding 1.02 50.377 0.872
67.839 1.124 46.575 1.008 83.865
ENSMUSG00000032253 Phip protein_coding 1.007 62.888 0.93
89.713 1.07 43.586 0.971 96.183
ENSMUSG00000032254 Kif23 protein_coding 1.638 14.59 0.848 71.889
0.719 70.791 1.201 78.042
ENSMUSG00000032258 Lca5 protein_coding 0.963 60.003 1.181
71.617 0.879 33.402 1.085 63.708
ENSMUSG00000032259 Drd2 protein_coding 1 75.567 1
95.708 1 86.258 1 88.797
ENSMUSG00000032261 Sh3bgrl2 protein_coding 1.095 37.702
0.834 61.883 1.097 57.286 0.942 85.493
ENSMUSG00000032262 Elovl4 protein_coding 1.013 73.292 NA
NA 0.958 74.686 0.963 96.183
ENSMUSG00000032263 Bckdhb protein_coding 0.969 45.502 NA
NA 1.016 72.148 0.958 85.493
ENSMUSG00000032264 Zw10 protein_coding 1.045 65.733 0.966
91.275 0.989 85.757 1.046 55.841
ENSMUSG00000032265 Fam46a protein_coding 0.851 16.325

1.089 82.904 1.073 70.328 0.939 95.559
ENSMUSG00000032267 Usp28 protein_coding 1.052 32.058 NA NA
0.916 40.19 0.981 91.872
ENSMUSG00000032268 Tmprss5 protein_coding 1.025 76.546
1.153 96.897 0.844 35.815 0.841 85.493
ENSMUSG00000032269 Htr3a protein_coding 1 74.958 1
96.68 1 65.688 1 95.877
ENSMUSG00000032271 Nnmt protein_coding 1.018 83.823 1.112
82.904 0.882 46.32 0.907 84.29
ENSMUSG00000032274 Cyp19a1 protein_coding 1 86.464 1
93.899 1 86.727 1 93.261
ENSMUSG00000032278 Paqr5 protein_coding 1.45 21.599 0.488
38.699 1.412 45.208 0.678 87.182
ENSMUSG00000032279 Idh3a protein_coding 1.009 78.299 0.988
96.68 1.006 84.071 1.017 95.72
ENSMUSG00000032280 Tle3 protein_coding 1.079 25.207 0.986
86.604 0.937 72.464 1.087 40.024
ENSMUSG00000032281 Acsbg1 protein_coding 0.939 80.251
0.741 46.764 1.436 27.431 1.173 87.7
ENSMUSG00000032285 Dnaja4 protein_coding 0.963 63.126 NA
NA 1.014 57.286 1.078 86.133
ENSMUSG00000032288 Imp3 protein_coding 0.996 71.763 1.035
94.367 0.971 88.601 0.965 92.181
ENSMUSG00000032289 Thsd4 protein_coding 0.967 83.823 1.056
96.68 NA NA 1.641 69.404
ENSMUSG00000032290 Ptpn9 protein_coding 1.036 23.82 1.001 86.277
0.966 49.217 1.058 79.713
ENSMUSG00000032291 Crabp1 protein_coding 2.939 3.893 0.857
93.654 0.398 3.073 0.886 96.086
ENSMUSG00000032292 Nr2e3 protein_coding 1 74.745 1
82.503 1 69.825 1 95.74
ENSMUSG00000032293 Ireb2 protein_coding 1.005 86.112 0.933
59.946 1.069 28.979 1.01 93.117
ENSMUSG00000032294 Pkm protein_coding 0.668 1.15 1.155 91.065
1.298 6.202 1.207 49.806
ENSMUSG00000032295 Man2c1 protein_coding 0.922 5.544 NA NA
1.071 17.412 0.996 94.654
ENSMUSG00000032298 Neil1 protein_coding 0.887 8.751 0.971 95.329
1.161 17.279 0.973 92.675
ENSMUSG00000032299 Commd4 protein_coding 0.966 38.983 1.02
93.574 1.017 68.129 1.054 55.485
ENSMUSG00000032300 1700017B05Rik protein_coding 1.088 58.709
0.92 79.807 0.998 81.976 0.961 89.827
ENSMUSG00000032301 Psma4 protein_coding 1.011 85.283 0.992
96.68 0.994 82.31 1.105 44.679
ENSMUSG00000032305 Fam219b protein_coding 1.044 10.687 NA
NA 0.961 20.258 1.025 81.061
ENSMUSG00000032306 Mpi protein_coding 1.217 2.651 0.915 73.094
0.899 72.464 1.084 36.988
ENSMUSG00000032307 Ube2q2 protein_coding 1.019 80.688
0.819 55.265 1.198 33.508 0.983 92.181
ENSMUSG00000032308 Ulk3 protein_coding 0.831 6.221 1.027 97.263
1.168 9.301 0.932 84.29
ENSMUSG00000032309 Fbxo22 protein_coding 0.997 81.24 0.993

95.504	1.012	88.203	1.015	93.722
ENSMUSG00000032310	Cyp1a2	protein_coding	1	86.199	1
96.68	1	87.173	1	84.499
ENSMUSG00000032311	Nrg4	protein_coding	0.582	0.987	1.118	79.864
1.537	6.202	1.092	77.331
ENSMUSG00000032312	Csk	protein_coding	0.945	44.787	1.02
90.389	1.038	79.554	1.079	33.269
ENSMUSG00000032313	AI118078	protein_coding	1.168	84.452
0.954	92.111	0.895	63.075	0.684	91.078
ENSMUSG00000032314	Etfa	protein_coding	1.075	43.019	0.994
96.843	0.934	23.607	0.99	89.475
ENSMUSG00000032315	Cyp1a1	protein_coding	1.244	57.655
1.097	96.847	0.734	66.753	0.998	93.316
ENSMUSG00000032316	Clk3	protein_coding	0.875	0.987	0.977	70.29
1.167	5.864	0.98	85.493
ENSMUSG00000032318	Isl2	protein_coding	0.471	71.763	0.961
95.504	2.203	55.81	3.838	80.324
ENSMUSG00000032320	Rcn2	protein_coding	1.105	1.323	0.931	60.678
0.968	21.666	1.031	92.018
ENSMUSG00000032322	Pstpip1	protein_coding	1.246	23.82	0.889
64.555	0.904	83.397	NA	NA
ENSMUSG00000032323	Cyp11a1	protein_coding	1.468	17.191
0.991	96.138	0.689	24.918	0.891	93.612
ENSMUSG00000032324	Tspan3	protein_coding	1.017	84.865
0.989	95.35	0.99	87.173	0.982	94.608
ENSMUSG00000032327	Stra6	protein_coding	1.169	37.414	0.986
96.68	0.87	41.506	1.093	91.525
ENSMUSG00000032328	Tmem30a	protein_coding	1.11	1.828	0.973
90.053	0.926	0.72	0.994	89.892
ENSMUSG00000032329	Hmg20a	protein_coding	1.046	4.159	0.966
86.277	0.987	39.079	1.001	95.273
ENSMUSG00000032330	Cox7a2	protein_coding	0.969	67.163
1.076	85.756	0.955	75.353	1.03	92.903
ENSMUSG00000032332	Col12a1	protein_coding	0.845	16.652
0.927	93.094	1.281	12.467	1.277	0
ENSMUSG00000032333	Stoml1	protein_coding	0.994	73.841
0.985	89.996	1.021	84.896	1.049	82.85
ENSMUSG00000032334	Loxl1	protein_coding	1.269	1.161	1.02	93.146
0.77	0.72	1.269	43.377
ENSMUSG00000032336	Nptn	protein_coding	1.038	28.098	1.039
90.68	0.923	8.027	1.05	31.133
ENSMUSG00000032338	Hcn4	protein_coding	0.914	59.028	1.044
95.431	1.047	84.229	1.182	71.653
ENSMUSG00000032340	Neo1	protein_coding	1.036	81.125	1.022
94.89	0.945	60.614	0.958	95.72
ENSMUSG00000032342	Mto1	protein_coding	0.973	47.174	0.964
92.931	1.069	53.449	1.013	95.877
ENSMUSG00000032343	Impg1	protein_coding	1	69.777	1
85.116	1	77.888	1	86.991
ENSMUSG00000032344	Mb21d1	protein_coding	1.311	2.512	NA	NA
0.849	10.602	1.277	49.514
ENSMUSG00000032348	Gsta4	protein_coding	0.997	83.263	0.998
94.848	1.003	82.778	0.902	96.086
ENSMUSG00000032349	Elovl5	protein_coding	1.248	2.651	1.023

97.263 0.781 0.879 0.873 83.724
ENSMUSG00000032350 Gclc protein_coding 1.255 9.805 0.844 85.075
0.942 15.465 1.031 94.904
ENSMUSG00000032352 Lrrc1 protein_coding 1.072 29.596 0.942
86.861 0.984 62.791 0.955 86.254
ENSMUSG00000032353 Tmed3 protein_coding 0.993 85.655 1.213
79.411 0.829 28.669 1.513 55.054
ENSMUSG00000032355 Mlip protein_coding 1.248 38.682 1.078
95.431 0.74 24.523 0.953 95.273
ENSMUSG00000032356 Rasgrf1 protein_coding 1.028 78.909
0.999 92.793 0.974 87.37 1.078 94.904
ENSMUSG00000032357 Tinag protein_coding 0.61 2.5 1.315 78.494
1.242 28.055 0.99 96.183
ENSMUSG00000032358 Fam83b protein_coding 0.784 12.785
0.946 83.58 1.352 13.356 0.991 94.904
ENSMUSG00000032359 Ctsh protein_coding 1.404 0.355 0.8 34.058
0.889 39.528 1.114 83.067
ENSMUSG00000032363 Adamts7 protein_coding 0.865 36.259
1.139 83.104 1.015 72.853 1.037 92.466
ENSMUSG00000032366 Tpm1 protein_coding 1.567 7.65 1.074 83.104
0.594 0.72 0.902 96.183
ENSMUSG00000032368 Zic1 protein_coding 0.955 71.142 0.431
95.211 2.427 66.647 4.918 49.514
ENSMUSG00000032369 Plscr1 protein_coding 1.101 20.942
0.813 74.851 1.119 76.768 1.175 78.948
ENSMUSG00000032370 Lactb protein_coding 0.954 74.482 0.982
95.708 1.068 77.888 1.026 86.301
ENSMUSG00000032372 Plscr2 protein_coding 1.206 7.842 0.966
96.216 0.856 16.051 1.125 93.016
ENSMUSG00000032373 Car12 protein_coding 1.238 68.16 0.833 93.654
0.971 86.084 1.708 57.1
ENSMUSG00000032374 Plod2 protein_coding 0.992 72.655 1.26
72.546 0.801 11.751 2.085 52.324
ENSMUSG00000032375 Aph1b protein_coding 1.028 67.374 0.984
83.392 0.991 85.352 0.968 91.078
ENSMUSG00000032376 Usp3 protein_coding 1.154 25.421 0.815
29.448 1.066 19.609 0.886 96.086
ENSMUSG00000032377 Plscr4 protein_coding 1.121 6.135 1.025
90.092 0.871 1.061 1.05 83.067
ENSMUSG00000032380 Dapk2 protein_coding 0.985 62.888 0.963
97.155 1.049 73.428 0.891 84.29
ENSMUSG00000032381 Fam96a protein_coding 1.087 9.259 1.011
83.392 0.911 58.516 1.061 60.968
ENSMUSG00000032382 Snx1 protein_coding 1.11 4.159 0.991 95.708
0.912 12.948 1.094 46.412
ENSMUSG00000032383 Ppib protein_coding 1.115 9.805 0.972 79.475
0.922 27.675 1.027 48.119
ENSMUSG00000032384 Csnk1g1 protein_coding 1.103 11.638
0.978 91.361 0.929 24.309 0.942 88.053
ENSMUSG00000032386 Trip4 protein_coding 0.955 45.502 1.019
96.138 1.028 31.975 1.027 86.209
ENSMUSG00000032387 Rbpms2 protein_coding 0.878 15.76 1.062
83.464 1.073 70.328 0.991 90.628
ENSMUSG00000032388 Spg21 protein_coding 1.119 0.2 1.04 85.756

0.858 0.72 0.954 83.724
ENSMUSG00000032392 Parp16 protein_coding 0.917 67.477
1.244 72.546 0.874 2.817 0.986 96.287
ENSMUSG00000032393 Dpp8 protein_coding 1.034 52.134 0.983
87.452 0.986 82.31 0.999 93.156
ENSMUSG00000032394 Igdcc3 protein_coding 0.925 82.231
1.047 97.263 1.033 83.607 0.984 90.611
ENSMUSG00000032396 Dis3l protein_coding 0.999 84.534 1.035
95.431 0.969 68.82 1.013 93.156
ENSMUSG00000032397 Tipin protein_coding 1.209 13.557 0.889
82.503 0.934 30.827 1.086 49.239
ENSMUSG00000032398 Snapc5 protein_coding 0.943 71.142
1.136 80.806 0.934 39.079 1.092 52.804
ENSMUSG00000032399 Rpl4 protein_coding 1.018 66.76 0.951 75.345
1.033 48.772 1.06 68.354
ENSMUSG00000032400 Zwilch protein_coding 1.134 76.319
0.947 92.137 0.934 85.134 1.358 64.905
ENSMUSG00000032401 Lctl protein_coding 0.529 0.987 1.133 83.104
1.661 6.202 1.363 72.116
ENSMUSG00000032402 Smad3 protein_coding 0.992 81.925 1.081
82.904 0.933 61.288 1.009 90.177
ENSMUSG00000032403 2300009A05Rik protein_coding 0.929 57.818
1.006 88.923 1.068 81.388 0.877 75.345
ENSMUSG00000032405 Pias1 protein_coding 1.179 0 0.925 56.781
0.915 16.129 0.954 83.724
ENSMUSG00000032407 U2surp protein_coding 1.009 84.865
0.962 79.475 1.029 47.31 0.996 95.74
ENSMUSG00000032409 Atr protein_coding 0.88 10.29 1.014 96.407
1.123 33.042 1.02 95.74
ENSMUSG00000032410 Xrn1 protein_coding 0.867 19.83 0.92 85.418
1.255 9.86 0.997 95.72
ENSMUSG00000032411 Tfdp2 protein_coding 1.007 57.227 0.983
95.708 1.008 66.928 0.882 83.724
ENSMUSG00000032412 Atp1b3 protein_coding 1.054 20.444
1.044 79.29 0.911 3.147 1.051 74.047
ENSMUSG00000032413 Rasa2 protein_coding 1.033 83.263 1.06
82.904 0.913 59.125 0.927 88.245
ENSMUSG00000032415 Ube2cbp protein_coding 1.071 69.408
0.871 73.766 1.069 56.497 1.038 81.251
ENSMUSG00000032417 Rwdd2a protein_coding 1.321 35.748
0.937 95.35 0.805 58.271 0.881 85.493
ENSMUSG00000032418 Me1 protein_coding 0.326 1.031 1.172 90.092
2.607 5.864 0.881 92.181
ENSMUSG00000032419 Tbx18 protein_coding 1.093 74.147 0.934
75.618 0.982 84.896 0.927 96.358
ENSMUSG00000032420 Nt5e protein_coding 1.099 20.942 0.988
96.68 0.921 28.489 0.859 90.674
ENSMUSG00000032422 Snx14 protein_coding 1.004 86.464 0.962
87.452 1.032 70.9 1.051 96.287
ENSMUSG00000032423 Syncrip protein_coding 1.107 1.689 0.986
92.474 0.918 14.079 0.975 89.587
ENSMUSG00000032425 Zfp949 protein_coding 0.997 80.608
0.877 46.764 1.142 17.412 1.03 95.405
ENSMUSG00000032431 Crtap protein_coding 1.046 34.077 1.062

82.76 0.9 12.571 1.135 50.452
ENSMUSG00000032434 Cmtm6 protein_coding 1.206 0.461 1.013 91.584
0.82 0.72 0.911 83.724
ENSMUSG00000032435 Dync1li1 protein_coding 1.123 0.2 0.974
86.861 0.912 0.879 1.009 92.762
ENSMUSG00000032436 Cmtm7 protein_coding 1.196 12.72 NA NA
0.873 6.858 0.925 83.724
ENSMUSG00000032437 Stt3b protein_coding 1.003 76.686 1.006
93.899 0.991 77.037 0.966 86.699
ENSMUSG00000032440 Tgfbr2 protein_coding 1.075 10.391
0.979 95.504 0.946 19.449 1.176 69.708
ENSMUSG00000032443 Zcwpw2 protein_coding 0.81 50.519 0.88
90.256 1.403 56.708 0.978 93.964
ENSMUSG00000032446 Eomes protein_coding 1.102 77.182 1.724
66.01 0.528 9.288 0.74 90.157
ENSMUSG00000032449 Slc25a36 protein_coding 0.98 69.943
0.984 96.138 1.04 79.897 0.933 83.724
ENSMUSG00000032451 Trim42 protein_coding 1 80.608 1
96.652 1 86.258 1 96.183
ENSMUSG00000032452 Clstn2 protein_coding 0.957 74.482
1.075 95.708 0.969 84.308 1.052 86.445
ENSMUSG00000032456 Nmnat3 protein_coding 1.046 65.284
1.011 94.767 0.948 57.091 0.917 83.724
ENSMUSG00000032458 Copb2 protein_coding 1.028 69.489 0.954
84.594 1.021 84.961 1.094 31.133
ENSMUSG00000032459 Mrps22 protein_coding 0.877 49.944
1.158 90.952 0.981 87.336 1.064 69.918
ENSMUSG00000032462 Pik3cb protein_coding 0.929 54.983
1.139 62.898 0.947 18.539 1 90.289
ENSMUSG00000032463 Faim protein_coding 1.037 24.481 1.009
90.389 0.952 17.898 1.081 67.691
ENSMUSG00000032468 Armc8 protein_coding 1.092 1.216 0.998 94.48
0.917 6.202 1.037 83.865
ENSMUSG00000032469 Dbr1 protein_coding 0.999 82.092 1.048
83.104 0.953 33.402 0.992 94.608
ENSMUSG00000032470 Mras protein_coding 1.002 84.452 NA NA
0.977 81.525 1.064 55.841
ENSMUSG00000032475 Nck1 protein_coding 1.122 0.824 0.951 93.654
0.941 6.524 1.054 83.067
ENSMUSG00000032477 Cdc25a protein_coding 0.894 26.217
0.996 96.843 1.119 23.41 1.028 91.525
ENSMUSG00000032478 Nme6 protein_coding 0.998 63.736 1.125
79.367 0.892 45.799 1.07 79.442
ENSMUSG00000032479 Map4 protein_coding 1.009 77.916 0.991
96.216 1.002 69.445 1.129 32.045
ENSMUSG00000032480 Dhx30 protein_coding 0.951 10.955 NA NA
1.06 21.873 0.994 95.877
ENSMUSG00000032481 Smarcc1 protein_coding 0.985 78.224
1.045 96.652 0.972 72.378 1.01 96.248
ENSMUSG00000032482 Cspg5 protein_coding 0.613 7.574 NA NA
1.723 24.691 1.056 95.74
ENSMUSG00000032484 Ngp protein_coding 1 86.006 1
85.116 1 53.449 1 96.287
ENSMUSG00000032485 Scap protein_coding 0.94 11.769 1.043

88.823 1.021 34.07 0.903 83.724
ENSMUSG00000032487 Ptgs2 protein_coding 0.672 13.062 1.103
92.931 1.349 57.286 1.671 31.133
ENSMUSG00000032489 Kif9 protein_coding 1.172 45.61 0.901 85.116
0.949 86.144 0.773 89.892
ENSMUSG00000032491 Nradd protein_coding 0.86 15.76 1.068 92.062
1.089 42.199 1.122 72.992
ENSMUSG00000032492 Pth1r protein_coding 0.758 3.956 1.17 82.904
1.129 38.152 1.098 80.187
ENSMUSG00000032493 Prss44 protein_coding 1 76.135 1
96.763 1 84.504 1 96.358
ENSMUSG00000032495 Lrrc2 protein_coding 5.633 10.105 0.966
93.707 0.183 1.061 0.864 95.273
ENSMUSG00000032496 Ltf protein_coding 0.934 85.637 0.755
69.611 1.421 51.977 1.031 96.358
ENSMUSG00000032497 Lrrfip2 protein_coding 0.951 14.221
1.004 95.35 1.047 25.93 1.075 31.133
ENSMUSG00000032498 Mlh1 protein_coding 1.017 84.534 NA NA
1.003 84.83 0.991 94.016
ENSMUSG00000032500 Dclk3 protein_coding 1.068 65.733 1.145
88.092 0.82 18.965 1.256 62.918
ENSMUSG00000032501 Trib1 protein_coding 1.06 37.091 1.067
84.832 0.886 25.512 1.051 90.455
ENSMUSG00000032502 Stac protein_coding 1.202 16.436 0.882
79.894 0.945 64.778 1.063 79.268
ENSMUSG00000032504 Pdcd6ip protein_coding 0.957 8.709 0.993
96.847 1.053 25.93 1.046 66.944
ENSMUSG00000032507 Fbxl2 protein_coding 0.957 59.358 0.983
83.464 1.062 87.898 0.84 83.724
ENSMUSG00000032508 Myd88 protein_coding 1.055 31.24 1.012 95.735
0.939 7.339 1.055 71.855
ENSMUSG00000032511 Scn5a protein_coding 1.27 17.336 0.863
68.115 0.915 87.173 1.008 91.872
ENSMUSG00000032512 Wdr48 protein_coding 1.018 66.272 0.96
68.848 1.018 41.6 0.962 84.29
ENSMUSG00000032513 Gorasp1 protein_coding 0.982 72.139
0.985 95.301 1.03 70.1 1.016 93.734
ENSMUSG00000032514 Ttc21a protein_coding 1.314 20.032
0.751 68.115 1.013 85.921 0.878 92.748
ENSMUSG00000032515 Csrnp1 protein_coding 1.112 25.026
0.966 83.321 0.931 68.584 0.924 83.724
ENSMUSG00000032517 Mobp protein_coding 1.236 71.896 0.72
94.102 1.126 80.899 0.711 93.734
ENSMUSG00000032518 Rpsa protein_coding 1.045 46.013 0.945
56.027 1.014 84.724 1.09 60.968
ENSMUSG00000032519 Slc25a38 protein_coding 0.881 3.07 1.069
81.039 1.062 31.317 1.023 95.74
ENSMUSG00000032523 Hhatl protein_coding 3.724 20.296 1.731
95.431 0.156 6.858 0.824 95.273
ENSMUSG00000032525 Nktr protein_coding 0.938 14.526 0.947
79.894 1.122 13.356 0.996 96.183
ENSMUSG00000032526 Deb1 protein_coding 0.798 1.343 1.096 71.617
1.142 28.979 1.049 90.125
ENSMUSG00000032527 Pccb protein_coding 0.839 1.031 1.063 88.823

1.122 20.556 1.019 94.608
ENSMUSG00000032528 Vipr1 protein_coding 0.655 21.393 0.932
87.041 1.634 24.029 1.182 87.134
ENSMUSG00000032530 Lyzl4 protein_coding 0.607 2.822 0.876 74.851
1.884 7.482 0.989 88.552
ENSMUSG00000032531 Amotl2 protein_coding 0.988 70.597
0.879 93.654 1.15 35.441 0.946 88.552
ENSMUSG00000032532 Cck protein_coding 1.232 32.058 1.074
92.931 0.751 22.279 0.879 88.136
ENSMUSG00000032534 Cep63 protein_coding 0.915 2.009 0.981 79.807
1.11 5.864 0.949 83.724
ENSMUSG00000032536 Trak1 protein_coding 0.894 1.15 1.093 71.617
1.021 38.26 0.928 94.654
ENSMUSG00000032537 Ephb1 protein_coding 0.902 46.413 1.189
82.904 0.931 88.657 1.14 83.581
ENSMUSG00000032540 Abhd5 protein_coding 0.855 9.841 1.163 72.546
1.004 77.888 0.897 83.724
ENSMUSG00000032547 Ryk protein_coding 1.065 22.349 0.96
85.418 0.979 52.087 0.975 95.72
ENSMUSG00000032548 Slco2a1 protein_coding 1.029 50.111
0.934 91.361 1.042 76.388 1.216 62.594
ENSMUSG00000032549 Rab6b protein_coding 0.981 84.324 0.994
94.96 1.021 83.719 0.966 90.628
ENSMUSG00000032551 1110059G10Rik protein_coding 1.05 67.991
1.071 92.062 0.887 31.887 1.066 60.31
ENSMUSG00000032553 Srprb protein_coding 0.914 56.768 1.045
93.146 1.049 77.17 1.013 85.786
ENSMUSG00000032554 Trf protein_coding 0.934 23.556 0.99
96.407 1.078 67.562 0.89 85.493
ENSMUSG00000032555 Topbp1 protein_coding 0.984 77.615
0.993 87.919 1.023 54.673 1.074 54.265
ENSMUSG00000032556 Bfsp2 protein_coding 1.452 59.211 1.084
96.847 0.637 51.715 0.806 93.264
ENSMUSG00000032557 Uba5 protein_coding 0.958 83.726 1.125
86.387 0.928 46.071 1.067 74.193
ENSMUSG00000032558 Nphp3 protein_coding 1.065 74.958 0.929
89.509 1.009 70.534 0.997 95.74
ENSMUSG00000032560 Dnajc13 protein_coding 1.114 0 0.946
83.733 0.948 11.997 1.048 71.855
ENSMUSG00000032561 Acpp protein_coding 1.281 8.365 0.93 88.691
0.841 52.658 0.917 83.724
ENSMUSG00000032562 Gnai2 protein_coding 1.021 58.198 1.049
92.253 0.936 28.237 0.996 83.067
ENSMUSG00000032563 Mrpl3 protein_coding 1.032 56.66 0.997 96.68
0.973 53.7 1.016 79.545
ENSMUSG00000032565 Nudt16 protein_coding 1.165 7.492 0.905
60.806 0.947 68.82 0.889 83.724
ENSMUSG00000032566 1700080E11Rik protein_coding 1 84.049
1 96.847 1 82.31 1 95.92
ENSMUSG00000032567 Aste1 protein_coding 1.044 77.916 1.075
96.216 0.894 58.271 0.967 95.273
ENSMUSG00000032570 Atp2c1 protein_coding 1.178 8.685 0.948
79.894 0.894 32.297 0.973 94.19
ENSMUSG00000032571 Pik3r4 protein_coding 1.106 5.253 0.917

42.923	0.989	88.657	0.96	83.952
ENSMUSG00000032572	Col6a4	protein_coding	0.724	15.76	0.993
95.708	1.384	24.375	1.041	94.608
ENSMUSG00000032575	Manf	protein_coding	0.867	51.447	1.05
86.387	1.095	88.657	1.222	46.846
ENSMUSG00000032577	Mapkapk3	protein_coding	1.111	46.533
1.154	67.462	0.777	2.087	0.928	84.19
ENSMUSG00000032578	Cish	protein_coding	0.717	48.354	2.409
71.617	0.579	24.918	0.987	92.748
ENSMUSG00000032579	Hemk1	protein_coding	0.885	8.122	1.064	92.793
1.059	31.479	0.968	91.269
ENSMUSG00000032580	Rbm5	protein_coding	0.956	4.115	0.997	95.708
1.05	36.578	0.983	94.752
ENSMUSG00000032582	Rbm6	protein_coding	0.955	57.227	0.953
84.594	1.098	33.287	1.025	92.018
ENSMUSG00000032583	Mon1a	protein_coding	0.917	26.013	1.006
97.049	1.085	35.13	0.992	96.287
ENSMUSG00000032584	Mst1r	protein_coding	0.664	2.277	1.36	73.092
1.102	66.928	1.145	59.394
ENSMUSG00000032586	Traip	protein_coding	0.86	49.944	1.036
92.631	1.125	46.575	1.346	31.133
ENSMUSG00000032589	Bsn	protein_coding	1.113	60.435	NA	NA
0.889	87.744	0.955	86.991
ENSMUSG00000032590	Apeh	protein_coding	0.888	1.031	1.003	90.378
1.123	5.864	1.053	65.558
ENSMUSG00000032591	Mst1	protein_coding	0.618	33.745	0.984
92.793	1.645	57.637	0.609	91.979
ENSMUSG00000032593	Amigo3	protein_coding	1.06	81.925
1.155	91.643	0.818	54.25	1.102	55.485
ENSMUSG00000032594	Ip6k1	protein_coding	1.058	56.085	0.981
94.848	0.965	86.346	0.916	83.724
ENSMUSG00000032595	Cdhr4	protein_coding	0.999	55.939	0.785
92.631	1.278	65.235	0.965	95.273
ENSMUSG00000032596	Uba7	protein_coding	1.033	51.826	1.051
91.584	0.923	50.523	1.043	62.594
ENSMUSG00000032598	Nckipsd	protein_coding	1.101	15.85	1.052
83.104	0.864	6.202	0.995	94.904
ENSMUSG00000032599	Ip6k2	protein_coding	0.9	17.664	0.873
40.463	1.268	5.864	1.037	91.525
ENSMUSG00000032601	Prkar2a	protein_coding	1.018	44.334
1.048	82.904	0.936	5.564	1.092	40.426
ENSMUSG00000032602	Slc25a20	protein_coding	0.983	85.283
0.878	93.765	1.16	88.657	1.014	93.685
ENSMUSG00000032604	Qars	protein_coding	1.076	12.855	0.945
62.692	0.984	72.269	0.986	94.904
ENSMUSG00000032606	Nicn1	protein_coding	1.067	29.811	1.033
95.735	0.907	11.314	0.966	88.505
ENSMUSG00000032607	Amt	protein_coding	0.835	6.822	0.937	78.471
1.279	6.506	0.946	90.611
ENSMUSG00000032609	Klhdc8b	protein_coding	0.805	11.769	0.92
53.434	1.346	7.024	1.027	94.608
ENSMUSG00000032611	1700102P08Rik	protein_coding	1.324	26.863
1.083	96.843	0.7	22.009	0.813	85.493
ENSMUSG00000032612	Usp4	protein_coding	1	80.146	0.987

91.361 1.014 68.425 1.05 75.198
ENSMUSG00000032615 Nt5m protein_coding 0.985 40.568 0.986
85.985 1.027 85.792 1 91.525
ENSMUSG00000032621 Srek1 protein_coding 0.91 0.987 0.964 64.624
1.143 5.864 1.014 52.804
ENSMUSG00000032624 Eml4 protein_coding 1.034 32.411 0.979
86.071 0.987 81.525 1.026 57.839
ENSMUSG00000032625 Thsd7a protein_coding 1.022 52.799
0.883 66.651 1.107 55.81 1.006 93.612
ENSMUSG00000032633 Flcn protein_coding 1.068 29.596 0.96
84.594 0.975 69.583 0.908 83.724
ENSMUSG00000032637 Atxn2l protein_coding 0.902 1.343 1.014
80.775 1.094 17.412 0.963 87.496
ENSMUSG00000032640 Chsy1 protein_coding 1.124 10.464 1.074
91.584 0.827 10.23 1.044 88.755
ENSMUSG00000032641 Gpr19 protein_coding 0.839 21.897 1.002
94.597 1.191 30.213 1.047 94.297
ENSMUSG00000032643 Fhl3 protein_coding 1.142 32.873 1.303
80.14 0.671 0.72 1.263 83.276
ENSMUSG00000032648 Pygm protein_coding 5.718 5.481 1.19 82.904
0.147 0.72 0.998 96.248
ENSMUSG00000032649 Colgalt2 protein_coding 1.176 43.654 1.03
90.952 0.825 10.602 0.825 83.724
ENSMUSG00000032652 Crebl2 protein_coding 1.138 0.2 0.98
93.094 0.898 1.48 0.962 83.724
ENSMUSG00000032656 3-Mar protein_coding 0.714 20.659 1.047
96.216 1.338 26.498 1.174 88.797
ENSMUSG00000032657 Fam189b protein_coding 1.054 56.311
1.029 91.584 0.919 32.082 1.049 91.807
ENSMUSG00000032661 Oas3 protein_coding 1.629 4.825 1.128 95.708
0.542 53.7 1.791 52.324
ENSMUSG00000032666 1700025G04Rik protein_coding 1.105 12.396
0.969 87.998 0.935 25.688 0.998 92.762
ENSMUSG00000032667 Pon2 protein_coding 0.965 34.305 0.972
91.955 1.063 14.449 1.032 59.394
ENSMUSG00000032671 A930018P22Rik protein_coding 1.059 62.689
1.561 87.029 0.607 65.688 1.125 92.181
ENSMUSG00000032673 Prorsd1 protein_coding 0.981 72.985
0.972 94.848 1.051 51.243 1.039 75.81
ENSMUSG00000032679 Cd59a protein_coding 1.019 72.85 0.995 96.013
0.985 81.169 1.02 94.752
ENSMUSG00000032680 6820408C15Rik protein_coding 0.686 18.731
1.103 85.756 1.317 40.575 1.033 93.016
ENSMUSG00000032688 Malt1 protein_coding 1.002 84.865 0.996
93.654 1.003 83.066 0.97 93.685
ENSMUSG00000032690 Oas2 protein_coding 1.235 6.61 NA NA
0.815 20.511 1.302 32.698
ENSMUSG00000032691 Nlrp3 protein_coding 1.218 16.249 NA NA
0.623 27.994 1.22 90.455
ENSMUSG00000032698 Lmo2 protein_coding 1.328 0.355 0.895 68.818
0.839 16.719 1.014 95.877
ENSMUSG00000032702 Kank1 protein_coding 0.839 23.641 1.01
91.361 1.182 20.085 1.062 82.091
ENSMUSG00000032705 Exd2 protein_coding 0.998 50.111 1.043

91.776 0.963 22.864 0.975 91.372
ENSMUSG00000032712 2810474O19Rik protein_coding 1.129 32.873
0.95 94.597 0.928 62.058 1.105 65.401
ENSMUSG00000032714 Syde1 protein_coding 0.841 4.73 1.128 40.99
1.055 66.647 1.106 90.177
ENSMUSG00000032715 Trib3 protein_coding 1.425 0 0.97 74.483
0.722 1.871 1.288 83.067
ENSMUSG00000032717 Mdfi protein_coding 0.729 3.101 0.921 84.017
1.49 12.831 0.783 86.699
ENSMUSG00000032718 Mansc1 protein_coding 0.979 84.534
0.878 63.619 1.159 46.575 0.991 86.991
ENSMUSG00000032719 Sbspon protein_coding 1.013 76.135
1.003 94.89 0.985 85.134 0.937 92.31
ENSMUSG00000032724 Abtb2 protein_coding 1.376 5.313 0.877 75.345
0.824 44.865 1.082 94.297
ENSMUSG00000032725 Folr2 protein_coding 1.415 0 0.761 61.883
0.925 41.154 1.2 78.948
ENSMUSG00000032726 Bmp8a protein_coding 0.571 28.242 1.07
85.756 1.635 69.196 2.564 60.968
ENSMUSG00000032727 Mier3 protein_coding 1.114 4.874 1.025 85.756
0.873 5.628 0.999 96.358
ENSMUSG00000032733 Snx33 protein_coding 1.034 46.013 1.088
90.389 0.886 28.237 0.997 88.053
ENSMUSG00000032735 Ablim3 protein_coding 0.88 27.664
0.989 95.088 1.151 25.93 1.012 93.457
ENSMUSG00000032737 Inppl1 protein_coding 0.901 8.122 1.056
79.29 1.048 43.481 NA NA
ENSMUSG00000032739 Pram1 protein_coding 0.368 35.894 1.15
92.914 2.362 24.375 1.913 71.008
ENSMUSG00000032740 Ccdc88a protein_coding 1.031 74.745
0.964 90.256 1.008 84.83 1.088 67.691
ENSMUSG00000032741 Tpcn1 protein_coding 1.11 1.009 1.071 75.21 0.84
0.72 0.994 92.12
ENSMUSG00000032743 D430042O09Rik protein_coding 0.913 1.701
0.953 85.671 1.15 9.301 0.959 84.29
ENSMUSG00000032744 Heyl protein_coding 0.729 6.677 1.134 93.899
1.211 29.777 1.095 94.19
ENSMUSG00000032745 Gpbp1 protein_coding 1.107 1.689 0.966 86.071
0.935 15.024 1.034 81.251
ENSMUSG00000032750 Gab3 protein_coding 1.479 0.577 0.85 82.073
0.795 7.647 1.13 81.251
ENSMUSG00000032754 Slc8b1 protein_coding 0.994 83.726
0.897 41.268 1.122 55.352 0.994 86.991
ENSMUSG00000032757 Bet1 protein_coding 0.988 54.043 0.934
86.071 1.085 32.433 1.048 40.426
ENSMUSG00000032758 Kap protein_coding 1 80.03 1 95.708
1 83.158 1 94.654
ENSMUSG00000032763 Ilvbl protein_coding 0.897 4.993 1.002 93.899
1.114 22.93 0.996 86.991
ENSMUSG00000032766 Gng11 protein_coding 0.929 52.258 1.049
84.007 1.025 82.74 1.062 85.577
ENSMUSG00000032773 Chrm1 protein_coding 0.965 59.358 0.989
94.48 1.046 68.643 0.873 84.29
ENSMUSG00000032776 Mctp2 protein_coding 0.877 27.836 0.97

95.211 1.173 28.997 1.038 83.067
ENSMUSG00000032777 Gtf3c1 protein_coding 0.971 13.062
0.959 85.116 1.076 14.131 0.981 91.525
ENSMUSG00000032782 Cntrob protein_coding 0.8 2.009 1.045
90.092 1.198 7.635 1.063 83.067
ENSMUSG00000032783 Troap protein_coding 1.133 50.111 0.731
56.027 1.208 82.74 2.09 68.696
ENSMUSG00000032786 Alas1 protein_coding 0.663 27.664 1.149
82.904 1.309 47.512 1.292 67.691
ENSMUSG00000032788 Pdxk protein_coding 1.048 35.499 1.064
72.546 0.899 2.563 1.104 79.545
ENSMUSG00000032796 Lama1 protein_coding 1.017 75.567 1.015
94.48 0.969 63.801 1.065 83.067
ENSMUSG00000032802 Srxn1 protein_coding 1.046 30.984 1.053
85.756 0.909 8.382 1.131 46.846
ENSMUSG00000032803 Cdv3 protein_coding 1.069 1.99 NA NA
0.923 4.963 1.05 69.708
ENSMUSG00000032806 Slc10a3 protein_coding 0.998 69.089 1.04
86.793 0.963 35.264 0.971 95.74
ENSMUSG00000032807 Alox12b protein_coding 1 64.714 1
89.713 1 82.436 1 96.248
ENSMUSG00000032808 Cyp2c38 protein_coding 1 84.865 1
96.268 1 86.727 1 95.35
ENSMUSG00000032812 Arap1 protein_coding 1.052 33.099 0.987
90.287 0.959 70.791 1.047 87.7
ENSMUSG00000032815 Fanca protein_coding 0.999 86.333 NA NA
1.04 86.985 1.253 73.368
ENSMUSG00000032816 Igdcc4 protein_coding 0.972 85.655 NA
NA 0.963 85.675 1.152 83.581
ENSMUSG00000032826 Ank2 protein_coding 1.312 7.553 1.139 80.806
0.671 0.945 0.963 96.184
ENSMUSG00000032827 Ppp1r9a protein_coding 0.973 69.985
0.921 72.576 1.118 39.439 0.956 90.289
ENSMUSG00000032834 Pwp2 protein_coding 0.969 86.333 0.998
87.857 1.036 82.161 1.05 64.513
ENSMUSG00000032839 Trpc1 protein_coding 0.939 63.126 1.022
93.654 1.043 72.378 1.211 57.1
ENSMUSG00000032840 2410131K14Rik protein_coding 0.944 5.967
1.036 82.904 1.024 55.352 0.971 96.192
ENSMUSG00000032841 Prr5l protein_coding 1.141 41.177 0.905
79.807 0.969 62.791 0.873 85.493
ENSMUSG00000032842 Abcc10 protein_coding 0.905 21.897
0.985 93.654 1.118 21.462 0.904 83.724
ENSMUSG00000032845 Alpk2 protein_coding 2.425 30.497 1.147
90.68 0.36 11.314 0.463 91.372
ENSMUSG00000032846 Zswim6 protein_coding 1.092 6.23 0.99
95.708 0.926 6.524 0.959 85.493
ENSMUSG00000032849 Abcc4 protein_coding 1.117 10.105 1.032
94.848 0.869 39.245 1.072 71.305
ENSMUSG00000032850 Rnft2 protein_coding 0.723 1.031 1.121 75.62
1.231 30.107 1.05 59.208
ENSMUSG00000032852 Rspo4 protein_coding 1.148 83.968 0.929
91.532 0.937 77.532 2.622 36.174
ENSMUSG00000032854 Ugt8a protein_coding 0.951 51.089 1.056

85.985 NA NA 0.981 93.264
ENSMUSG00000032855 Pkd1 protein_coding 0.957 19.83 1.007 82.904
1.034 76.225 0.972 87.735
ENSMUSG00000032860 P2ry2 protein_coding 1.112 33.555 0.884
85.671 1.018 82.575 1.078 74.193
ENSMUSG00000032864 Rag2 protein_coding 1 81.52 1 95.088
1 86.144 1 95.74
ENSMUSG00000032867 Fbxw8 protein_coding 0.894 0.987 1.008 94.102
1.107 5.864 1.062 59.208
ENSMUSG00000032869 Psmf1 protein_coding 1.04 53.228 0.977
86.277 0.985 73.531 1.042 85.786
ENSMUSG00000032870 Smap2 protein_coding 1.114 2.897 0.971 74.483
0.926 48.577 1.051 67.169
ENSMUSG00000032872 Cyb5r4 protein_coding 1.085 7.65 0.97
89.713 0.948 26.809 1.06 83.067
ENSMUSG00000032875 Arhgef17 protein_coding 0.961 16.325 1.07
65.165 0.971 71.004 0.933 83.724
ENSMUSG00000032878 Ccdc85a protein_coding 1.05 81.178
1.093 93.654 0.872 86.258 1.007 96.183
ENSMUSG00000032883 Acsl3 protein_coding 1.225 0 0.916 73.461
0.888 8.626 1.011 94.19
ENSMUSG00000032889 Gm6685 protein_coding 1 43.816 1
91.065 1 79.554 1 93.734
ENSMUSG00000032890 Rims3 protein_coding 1.451 14.008 0.875
74.851 0.788 54.964 0.911 95.273
ENSMUSG00000032892 Rangrf protein_coding 0.897 24 1.097
83.104 1.016 71.232 0.963 96.192
ENSMUSG00000032894 1700031F05Rik protein_coding 1 83.169
1 92.793 1 88.693 1 94.904
ENSMUSG00000032897 Nfyc protein_coding 1.007 80.251 1.067
72.642 0.929 32.963 1.002 93.156
ENSMUSG00000032898 Fbxo21 protein_coding 1.115 81.52 0.578
65.165 1.547 42.065 1.017 92.564
ENSMUSG00000032899 Styk1 protein_coding 0.889 86.74 0.932 90.712
1.207 68.643 1.422 49.769
ENSMUSG00000032900 1700065I17Rik protein_coding 1 77.916
1 95.708 1 79.897 1 95.273
ENSMUSG00000032902 Slc16a1 protein_coding 1.273 5.481 1.044
92.111 0.753 0.72 0.894 90.805
ENSMUSG00000032905 Atg12 protein_coding 1.021 76.546 0.966
88.705 1.01 84.308 0.964 86.699
ENSMUSG00000032908 Sgpp2 protein_coding 1.469 29.536 1.442
95.809 0.471 20.009 0.68 87.651
ENSMUSG00000032911 Cspg4 protein_coding 0.661 0.987 1.215 65.165
1.249 11.348 1.22 57.075
ENSMUSG00000032913 Lrig2 protein_coding 1.035 58.38 0.969 94.102
0.999 65.902 1.012 90.691
ENSMUSG00000032915 Emr4 protein_coding 1.222 44.334 0.804
34.058 1.012 58.915 0.827 85.493
ENSMUSG00000032921 Odf4 protein_coding 1.164 84.294 0.591
90.712 1.455 72.695 0.906 93.964
ENSMUSG00000032925 Itgbl1 protein_coding 1.069 61.725
0.855 75.618 1.094 81.032 0.894 91.078
ENSMUSG00000032932 Hspa13 protein_coding 1.045 60.853 1

91.456 0.959 39.079 1.119 54.153
ENSMUSG00000032936 Camkv protein_coding 0.882 81.823 1.207
96.407 0.937 88.693 1.771 83.276
ENSMUSG00000032937 Fshr protein_coding 1 83.418 1
94.597 1 84.724 1 95.35
ENSMUSG00000032939 Nup93 protein_coding 0.964 49.368 1.039
93.574 1 82.161 1.202 0
ENSMUSG00000032940 Rbm11 protein_coding 1.111 82.092 0.696
94.48 1.289 71.615 0.712 85.493
ENSMUSG00000032942 Ucp3 protein_coding 0.924 57.014 1.196
80.775 0.907 76.768 0.84 84.19
ENSMUSG00000032946 Rasgrp2 protein_coding 0.788 1.6 1.031
96.216 1.232 5.864 1.001 91.985
ENSMUSG00000032948 Lipi protein_coding 1 86.474 1
95.809 1 86.666 1 95.877
ENSMUSG00000032952 Ap4b1 protein_coding 1.012 64.956 0.921
54.06 1.072 18.565 1.014 83.067
ENSMUSG00000032959 Pebp1 protein_coding 0.894 2.142 NA NA
1.131 7.635 1.027 79.713
ENSMUSG00000032965 NA NA NA NA 1.114 86.387 0.932 55.9
1.029 95.273
ENSMUSG00000032966 Fkbp1a protein_coding 0.968 59.674
1.058 81.127 0.977 69.134 1.082 71.653
ENSMUSG00000032968 Inha protein_coding 0.801 18.602 0.892
84.594 1.402 7.482 0.834 85.493
ENSMUSG00000032977 Fam207a protein_coding 1.032 39.075
0.979 93.654 0.986 48.387 0.995 96.183
ENSMUSG00000032985 5730522E02Rik protein_coding 0.834 45.502
1.75 83.464 0.685 88.41 1.181 74.193
ENSMUSG00000032987 Olfr281 protein_coding 1 85.832 1
95.301 1 57.446 1 95.92
ENSMUSG00000032988 Slc16a8 protein_coding 0.819 58.264
1.497 81.54 NA NA 0.759 92.466
ENSMUSG00000032997 Chpf protein_coding 0.831 0.987 NA NA
1.162 5.864 1.056 74.193
ENSMUSG00000032998 Foxj3 protein_coding 1.122 11.316 0.903
66.09 0.988 63.919 0.998 92.748
ENSMUSG00000033004 Mycbp2 protein_coding 1.169 10.687 0.97
95.301 0.884 29.765 1.109 91.525
ENSMUSG00000033006 Sox10 protein_coding 0.886 33.745 1.121
82.904 NA NA 0.887 83.724
ENSMUSG00000033007 Asic4 protein_coding 1.253 82.732 NA NA
0.675 55.214 0.718 86.991
ENSMUSG00000033009 Ogfod1 protein_coding 0.996 72.372
0.976 87.452 1.03 78.864 1.084 54.265
ENSMUSG00000033014 Trim33 protein_coding 1.106 2.361 0.951
67.739 0.948 36.786 0.953 85.493
ENSMUSG00000033015 4933427E13Rik lincRNA 1 82.705 1
94.89 1 86.985 1 95.676
ENSMUSG00000033016 Nfatc1 protein_coding 1.046 48.288
1.083 66.695 0.88 3.432 0.969 85.493
ENSMUSG00000033020 Polr2f protein_coding 0.869 0.987 0.988
89.509 1.168 5.964 0.989 90.463
ENSMUSG00000033021 Gmppa protein_coding 0.938 8.405 1.05 74.663

1.011 66.269 1.044 72.826
ENSMUSG00000033022 Cdo1 protein_coding 0.856 1.343 0.939 96.138
1.24 16.446 0.847 83.724
ENSMUSG00000033024 Klra9 protein_coding 1.135 75.196 1.47
80.678 0.599 38.434 0.865 91.105
ENSMUSG00000033027 Klrc3 protein_coding 0.638 66.129 0.945
95.431 1.663 81.492 0.856 95.559
ENSMUSG00000033029 1700088E04Rik protein_coding 0.861 12.624
NA NA 1.197 17.751 0.87 86.699
ENSMUSG00000033031 C330027C09Rik protein_coding 1.089 72.372
0.898 83.392 1.026 87.898 1.222 73.401
ENSMUSG00000033032 Afap1l1 protein_coding 0.967 74.689
1.084 83.104 0.956 62.533 0.984 87.496
ENSMUSG00000033033 Calhm2 protein_coding 1.109 23.254 NA
NA 0.886 34.21 1.067 84.499
ENSMUSG00000033036 Gm7879 pseudogene 1 83.169 1
95.504 1 80.749 1 92.12
ENSMUSG00000033039 Micall1 protein_coding 0.919 22.92 1.043
82.904 1.041 83.925 1.027 95.273
ENSMUSG00000033044 Dhrs7c protein_coding 4.46 8.102 2.968
72.546 0.075 0.72 1.189 93.156
ENSMUSG00000033047 Eif3l protein_coding 1.063 7.501 0.991 96.68
0.947 14.409 1.024 95.273
ENSMUSG00000033053 1700028P14Rik protein_coding 1 80.608
1 96.216 1 83.607 1 95.273
ENSMUSG00000033054 Npat protein_coding 0.943 49.749 0.999
91.661 1.063 42.199 1.032 80.187
ENSMUSG00000033055 Ankrd54 protein_coding 0.972 80.751
1.053 89.996 0.98 81.62 0.975 90.289
ENSMUSG00000033059 Pygb protein_coding 0.859 78.299 1.088
94.367 1.065 79.426 1.005 91.625
ENSMUSG00000033060 NA NA NA NA 0.967 93.654 1
78.498 0.983 90.611
ENSMUSG00000033061 Resp18 protein_coding 1 82.092 1
96.847 1 73.759 1 92.742
ENSMUSG00000033065 Pfkm protein_coding 1.696 18.556 0.997
96.68 0.59 6.858 0.943 94.825
ENSMUSG00000033066 Gas7 protein_coding 1.19 0.355 1.115 84.832
0.752 20.511 1.131 31.133
ENSMUSG00000033068 Entpd6 protein_coding 0.833 1.274 1.037
83.464 1.153 19.106 0.926 86.991
ENSMUSG00000033075 Senp1 protein_coding 1.036 46.013 1.01
85.756 0.958 29.217 1.042 86.118
ENSMUSG00000033080 Vsx1 protein_coding 1 62.574 1
95.301 1 78.713 1 92.762
ENSMUSG00000033082 Clec1a protein_coding 1.28 13.105
0.914 95.211 0.851 33.689 0.968 95.593
ENSMUSG00000033083 Tbc1d4 protein_coding 1.094 20.296
1.089 83.104 0.839 11.116 0.923 83.724
ENSMUSG00000033088 Triobp protein_coding 0.944 57.227
1.124 71.617 0.941 10.726 1.205 87.7
ENSMUSG00000033096 Apmap protein_coding 1.01 81.24 0.903 79.261
1.098 55.81 0.874 83.724
ENSMUSG00000033099 Nol12 protein_coding 0.934 13.239 1.007

97.263	1.058	22.994	1.086	65.558
ENSMUSG00000033102	Cdc14b	protein_coding	1.122	38.195
0.922	84.858	0.968	78.954	0.993	95.74
ENSMUSG00000033105	Lss	protein_coding	0.845	1.274	1.011	82.904
1.173	41.806	1.086	60.968
ENSMUSG00000033106	Slc7a6os	protein_coding	1.068	49.202
0.973	88.704	0.96	64.155	1.005	92.52
ENSMUSG00000033107	Rnf125	protein_coding	1.203	4.874	0.847
81.134	0.977	59.726	0.979	86.445
ENSMUSG00000033111	3830406C13Rik	protein_coding	1.052	41.951
1.001	82.671	0.949	87.971	0.994	96.071
ENSMUSG00000033114	Slc35d2	protein_coding	1.071	48.857
0.839	69.175	1.109	63.301	1.048	92.691
ENSMUSG00000033122	Hsd17b3	protein_coding	1	86.57	NA	NA
1	86.346	1	95.832
ENSMUSG00000033124	Atg9a	protein_coding	0.897	2.009	1.135	71.617
0.985	81.976	1.011	94.82
ENSMUSG00000033126	Ybey	protein_coding	0.979	85.355	0.967
77.598	1.058	53.134	0.915	85.493
ENSMUSG00000033128	Gga1	protein_coding	0.93	3.07	1.025	82.904
1.053	30.645	1.007	92.018
ENSMUSG00000033147	Slc22a15	protein_coding	1.173	13.8	0.824
69.611	1.031	88.693	0.861	83.724
ENSMUSG00000033149	Phldb2	protein_coding	0.991	49.047
1.043	78.629	0.967	53.7	0.99	96.183
ENSMUSG00000033152	Podxl2	protein_coding	1.054	50.377	1.01
94.597	0.94	78.713	1.013	96.358
ENSMUSG00000033156	Cst10	protein_coding	1	80.815	1
96.68	1	81.388	1	95.877
ENSMUSG00000033157	Abhd10	protein_coding	0.954	45.502
1.014	93.899	1.032	60.789	1.023	87.624
ENSMUSG00000033159	Cnppd1	protein_coding	0.982	68.353
0.917	72.576	1.104	38.926	0.98	85.493
ENSMUSG00000033161	Atp1a1	protein_coding	0.978	65.622
0.938	43.505	1.089	8.007	0.98	96.287
ENSMUSG00000033166	Dis3	protein_coding	1.152	22.205	0.98
96.216	0.886	30.827	1.024	71.855
ENSMUSG00000033170	Card10	protein_coding	0.905	21.025
1.026	90.29	1.075	59.274	0.961	88.505
ENSMUSG00000033174	Mgll	protein_coding	0.955	40.786	1.077
60.309	0.973	52.658	0.942	83.724
ENSMUSG00000033177	Tmprss7	protein_coding	0.509	18.731
1.761	96.407	1.119	63.301	0.891	95.273
ENSMUSG00000033182	Kbtbd12	protein_coding	0.981	83.658	NA
NA	1.088	88.41	1.166	74.193
ENSMUSG00000033184	Tmed7	protein_coding	1.088	10.595	0.996
90.712	0.923	17.258	0.968	93.156
ENSMUSG00000033186	Mzt1	protein_coding	1.042	8.996	1.014	93.817
0.95	15.778	0.99	88.387
ENSMUSG00000033187	BC016579	protein_coding	1.363	80.608
0.709	92.137	1.038	58.915	0.501	86.699
ENSMUSG00000033191	Tie1	protein_coding	0.89	72.743	1.093
88.551	1.027	83.397	0.976	84.29
ENSMUSG00000033192	Lpcat2	protein_coding	1.156	9.259	1.043

90.389 0.831 6.858 0.859 83.724
ENSMUSG00000033196 Myh2 protein_coding 0.542 80.251 1.638
88.136 1.127 84.071 1.444 71.653
ENSMUSG00000033200 Tpsg1 protein_coding 1.467 16.124 0.806
95.708 0.845 32.703 1.136 93.584
ENSMUSG00000033207 Mamdc2 protein_coding 1.355 2.361 0.807
31.966 0.91 77.294 1.007 91.372
ENSMUSG00000033208 S100b protein_coding 1.478 0.461 1.008 91.776
0.672 0.72 1.174 68.354
ENSMUSG00000033209 Ttc28 protein_coding 1.147 27.731 0.976
95.809 0.892 8.634 0.96 87.735
ENSMUSG00000033210 Slc9c1 protein_coding 1 74.482 1
89.014 1 86.666 1 87.134
ENSMUSG00000033213 AA467197 protein_coding 0.567 24.344
1.213 97.155 1.45 44.787 0.836 92.31
ENSMUSG00000033214 Slitrk5 protein_coding 1.245 75.399
1.407 90.895 0.571 26.369 0.874 87.651
ENSMUSG00000033216 Eefsec protein_coding 1.017 76.396
1.022 92.111 0.96 83.397 1.01 86.118
ENSMUSG00000033219 Gm9758 protein_coding 0.461 46.413 0.99
85.116 2.191 28.301 1.501 94.752
ENSMUSG00000033220 Rac2 protein_coding 1.577 0 0.863 83.733
0.734 5.453 1.136 74.193
ENSMUSG00000033222 Ttf2 protein_coding 0.981 75.761 0.975
88.691 1.048 61.418 1.013 79.268
ENSMUSG00000033227 Wnt6 protein_coding 0.8 38.827 1.276
71.98 0.976 60.233 1.172 87.447
ENSMUSG00000033228 Scaf11 protein_coding 1.083 0.999 0.944
55.71 0.979 49.217 0.968 83.724
ENSMUSG00000033233 Trim45 protein_coding 0.959 45.502
0.996 97.155 1.047 74.686 1.007 89.827
ENSMUSG00000033237 Arid2 protein_coding 0.979 53.407 0.993
95.708 1.026 68.129 0.954 84.29
ENSMUSG00000033249 Hsf4 protein_coding 0.978 86.006 1.029
94.48 0.995 84.308 1.033 94.608
ENSMUSG00000033253 Szt2 protein_coding 0.844 0.987 0.999 95.196
1.189 5.864 1.007 94.904
ENSMUSG00000033255 Gm5134 protein_coding 0.374 64.284
0.966 93.994 2.774 84.552 1.605 93.132
ENSMUSG00000033256 Shf protein_coding 0.923 28.514 1.021
81.127 1.063 80.381 1.008 95.559
ENSMUSG00000033257 Ttll4 protein_coding 0.944 36.259 NA NA
1.059 60.5 1.011 94.153
ENSMUSG00000033268 Duox1 protein_coding 0.87 44.787 0.907
79.475 1.269 20.886 0.904 87.496
ENSMUSG00000033272 Slc35a4 protein_coding 0.954 28.584
1.105 60.309 0.949 23.1 1.034 78.379
ENSMUSG00000033276 Stk36 protein_coding 0.834 8.302 1.034 96.68
1.158 16.666 0.962 95.92
ENSMUSG00000033278 Ptprm protein_coding 0.975 78.224 1.012
97.155 1.011 86.552 1.04 96.287
ENSMUSG00000033282 NA NA NA NA 0.99 93.654 0.985
76.225 0.85 86.991
ENSMUSG00000033285 Wdr3 protein_coding 1.095 2.998 0.931 44.566

0.981 78.642 1 90.865
ENSMUSG00000033287 Kctd17 protein_coding 0.86 2.009 1.086
62.898 1.065 74.082 1.033 92.748
ENSMUSG00000033294 Noc4l protein_coding 1.033 64.714 0.978
87.452 0.986 86.985 1.069 94.153
ENSMUSG00000033295 Ptprf protein_coding 0.87 44.467 0.989
94.48 1.158 40.875 1.055 95.273
ENSMUSG00000033306 Lpp protein_coding 1.016 68.16 1.008 96.138
0.975 58.775 1.037 91.335
ENSMUSG00000033307 Mif protein_coding 1.072 52.799 0.976
87.857 0.96 77.037 1.083 89.152
ENSMUSG00000033308 Dpyd protein_coding 1.104 25.944 0.949
81.731 0.956 84.434 0.903 83.724
ENSMUSG00000033313 Fbxl8 protein_coding 1.004 57.544 1.005
96.216 0.992 75.155 1.064 63.708
ENSMUSG00000033316 Galnt9 protein_coding 1.128 67.374
0.978 96.68 0.906 59.726 0.921 94.654
ENSMUSG00000033318 Gstt2 protein_coding 1.173 1.161 0.85 71.331
1 52.213 0.934 85.493
ENSMUSG00000033319 Fem1c protein_coding 1.085 3.498 0.983 84.017
0.937 22.735 0.983 85.493
ENSMUSG00000033323 Ctdp1 protein_coding 0.996 85.728 1.015
93.899 0.991 69.445 1.027 95.273
ENSMUSG00000033326 Kdm4a protein_coding 0.938 27.836 1.012
95.088 1.055 28.979 1.011 94.904
ENSMUSG00000033327 Tnxb protein_coding 0.877 27.968 NA NA
1.123 44.361 0.92 90.805
ENSMUSG00000033335 Dnm2 protein_coding 0.927 7.887 NA NA
1.073 17.662 1.007 72.204
ENSMUSG00000033343 Magea4 protein_coding 1 74.516 1
91.83 1 83.607 1 92.181
ENSMUSG00000033350 Chst2 protein_coding 0.997 52.799 1.208
85.985 0.831 22.864 1.183 79.268
ENSMUSG00000033352 Map2k4 protein_coding 1.123 1.545 0.956
81.134 0.928 11.64 1.043 72.826
ENSMUSG00000033355 Rtp4 protein_coding 1.216 25.207 1.129
83.104 0.728 13.643 1.115 57.839
ENSMUSG00000033356 Pus7l protein_coding 1.062 83.823 0.994
93.574 0.946 87.744 1.008 93.156
ENSMUSG00000033361 Prrg3 protein_coding 0.816 8.139 0.942 87.313
1.302 6.202 0.956 95.877
ENSMUSG00000033364 Usp37 protein_coding 1.065 7.553 0.973 91.661
0.965 17.898 1.076 87.134
ENSMUSG00000033365 Ipo13 protein_coding 0.887 21.637 1.138
60.309 0.987 68.266 1.103 59.208
ENSMUSG00000033368 Trim69 protein_coding 0.69 45.229
1.557 92.111 0.933 70.1 0.892 94.904
ENSMUSG00000033373 Fntb protein_coding 0.885 7.71 1.072 81.039
1.05 32.524 1.069 46.412
ENSMUSG00000033377 Palmd protein_coding 0.87 27.664 0.984
88.136 1.168 58.051 0.922 93.612
ENSMUSG00000033379 Atp6v0b protein_coding 0.958 33.745
1.013 92.253 1.025 83.857 0.986 86.699
ENSMUSG00000033382 Trappc8 protein_coding 1.071 26.136

1.039 79.261 0.898 12.571 0.964 88.387
ENSMUSG00000033383 Rtp1 protein_coding 1 46.875 NA NA
1 70.44 1 90.611
ENSMUSG00000033386 Frrs1 protein_coding 1.165 0.999 0.94 90.053
0.914 21.431 0.971 86.991
ENSMUSG00000033389 Arhgap44 protein_coding 0.959 76.396
1.033 87.609 1.011 80.749 0.899 86.699
ENSMUSG00000033392 Clasp2 protein_coding 1.155 1.323 1.022
95.301 0.844 6.68 0.993 96.358
ENSMUSG00000033396 Spg11 protein_coding 0.953 33.415 0.956
66.09 1.096 8.93 1.029 93.734
ENSMUSG00000033400 Agl protein_coding 1.12 44.694 0.965
95.708 0.923 24.163 1.024 87.088
ENSMUSG00000033405 Nudt15 protein_coding 1.064 86.658
1.017 80.775 0.927 36.248 1.021 92.762
ENSMUSG00000033409 Syce1l protein_coding 1.019 79.277
0.703 94.262 1.392 87.103 1.337 90.157
ENSMUSG00000033411 Ctdspl2 protein_coding 1.072 26.77 NA NA
0.915 33.689 1.004 92.31
ENSMUSG00000033416 Gucd1 protein_coding 0.897 16.472 1.044
94.848 1.069 16.666 0.942 96.139
ENSMUSG00000033417 Cacul1 protein_coding 1.062 1.129 0.981
89.713 0.957 8.382 0.959 85.493
ENSMUSG00000033419 Snap91 protein_coding 1.063 69.777
0.711 81.443 1.326 73.428 0.539 96.248
ENSMUSG00000033420 Antxr1 protein_coding 1.21 7.501 1.082
96.847 0.764 22.735 0.969 91.372
ENSMUSG00000033423 Eri3 protein_coding 0.916 3.07 1.056 63.504
1.031 50.636 1.062 85.577
ENSMUSG00000033427 Upb1 protein_coding 0.872 51.56 1.026 90.378
1.12 42.75 0.842 85.493
ENSMUSG00000033429 Mcee protein_coding 0.999 56.477 0.952
89.996 1.051 43.129 0.963 88.387
ENSMUSG00000033430 Terf2ip protein_coding 1.046 12.396 0.98
71.41 0.978 67.339 0.957 84.19
ENSMUSG00000033434 Gtpbp6 protein_coding 0.932 17.839
0.984 96.843 1.086 35.441 1.001 92.12
ENSMUSG00000033436 Armcx2 protein_coding 1.065 37.414
1.024 93.574 0.915 51.715 1.054 88.512
ENSMUSG00000033439 Trmt13 protein_coding 1.015 56.907
0.915 35.072 1.075 23.604 1.026 90.177
ENSMUSG00000033444 Specc1l protein_coding 1.105 16.436
0.979 82.671 0.921 52.658 0.942 87.182
ENSMUSG00000033446 Lpar6 protein_coding 0.894 24.344 1.107
87.029 NA NA 0.951 88.505
ENSMUSG00000033450 Tagap protein_coding 1.205 35.024 0.878
94.848 0.943 66.201 0.738 83.724
ENSMUSG00000033453 Adamts15 protein_coding 0.913 82.092
0.958 87.553 1.139 53.587 1.218 81.251
ENSMUSG00000033454 Zbtb1 protein_coding 0.978 53.138 0.941
69.611 1.09 22.366 1.057 87.134
ENSMUSG00000033458 Fan1 protein_coding 0.986 74.689 0.944
81.731 1.075 43.586 1.01 95.832
ENSMUSG00000033460 Armcx1 protein_coding 1.036 51.826

0.973 92.914 0.988 86.985 0.982 96.086
ENSMUSG00000033467 Crlf2 protein_coding 0.93 65.199 1.017
93.817 1.058 68.643 1.065 71.855
ENSMUSG00000033470 Cysltr2 protein_coding 1.499 12.855
0.904 95.941 0.734 13.551 1.094 96.183
ENSMUSG00000033478 Fam160b1 protein_coding 1.03 33.099 0.97
68.115 1 78.864 0.995 92.181
ENSMUSG00000033486 Catsper2 protein_coding 0.966 54.176
1.045 82.904 NA NA 0.903 84.29
ENSMUSG00000033487 Fndc3a protein_coding 1.044 65.284 NA
NA 0.961 81.169 0.996 95.74
ENSMUSG00000033488 BC026585 protein_coding 0.897 3.231 1.001
94.767 1.11 18.411 0.955 96.248
ENSMUSG00000033491 Prss35 protein_coding 2.407 29.057
0.676 93.469 0.616 39.245 1.357 78.948
ENSMUSG00000033499 Larp4b protein_coding 1.078 0.577 0.968
45.262 0.957 14.079 1.026 94.654
ENSMUSG00000033502 Cdc14a protein_coding 0.833 8.751 1.04
87.185 1.155 30.292 0.828 83.724
ENSMUSG00000033508 Asprv1 protein_coding 0.857 52.9 0.899
92.404 1.297 42.199 0.891 91.625
ENSMUSG00000033510 Otud7a protein_coding 0.891 76.968 0.6
91.532 1.877 72.148 0.837 96.248
ENSMUSG00000033520 Idi2 protein_coding 1 75.671 1
91.897 1 71.388 1 84.29
ENSMUSG00000033526 Ppip5k1 protein_coding 0.915 4.268 1.097
74.663 NA NA 1.04 76.831
ENSMUSG00000033530 Ttc7b protein_coding 0.793 3.848 1.26 72.546
0.998 84.724 1.01 93.261
ENSMUSG00000033533 Acsm1 protein_coding 1 82.092 NA NA
NA NA 1 93.964
ENSMUSG00000033538 Casp4 protein_coding 1.12 35.371 0.989
96.308 0.903 19.552 1.058 83.067
ENSMUSG00000033540 Idua protein_coding 1.053 65.537 0.944
87.147 0.999 85.792 0.927 84.29
ENSMUSG00000033542 Arhgef5 protein_coding 1.056 30.497
1.037 82.904 0.912 9.901 0.887 83.724
ENSMUSG00000033543 Gtf2a2 protein_coding 1.075 13.492
0.955 81.443 0.974 44.865 0.971 91.625
ENSMUSG00000033544 Angptl1 protein_coding 0.979 61.043
0.985 95.196 1.037 84.83 1.177 50.452
ENSMUSG00000033545 Znrf1 protein_coding 0.895 7.43 0.962 81.134
1.164 7.635 0.948 85.493
ENSMUSG00000033554 Dph5 protein_coding 0.978 62.158 0.933
68.848 1.096 32.776 1.034 72.992
ENSMUSG00000033557 Fam20b protein_coding 1.049 11.316
0.994 96.763 0.958 20.258 0.985 93.264
ENSMUSG00000033565 Rbfox2 protein_coding 1.009 45.307 1.05
88.551 0.945 10.934 1.124 0
ENSMUSG00000033569 Bai3 protein_coding 2.558 20.942 0.446
74.483 0.875 77.037 1.023 92.762
ENSMUSG00000033576 Apol6 protein_coding 1.004 84.294 1.035
95.504 0.958 86.985 1.138 61.984
ENSMUSG00000033577 Myo6 protein_coding 0.976 54.87 1.033 95.504

0.988 56.125	0.903 83.724
ENSMUSG00000033578	Tmem35	protein_coding	1.334 4.052 0.829
60.678	0.902 84.308	0.836 83.724
ENSMUSG00000033579	Fa2h	protein_coding	0.819 9.599 1.027 92.931
1.19	29.819	1.101 78.379
ENSMUSG00000033581	Igf2bp2	protein_coding	1.075 13.492
1.043 95.735	0.893 6.858 0.868 83.724
ENSMUSG00000033585	Ndn	protein_coding	0.993 83.333	1.058
87.037	0.954 65.902	1.218 0
ENSMUSG00000033589	Reep4	protein_coding	1.021 86.006	0.955
84.594	1.022 52.574	1.017 93.722
ENSMUSG00000033590	Myo5c	protein_coding	1.196 34.861	0.768
61.883	1.086 88.203	0.9	87.218
ENSMUSG00000033594	Spata2l	protein_coding	0.935 80.146	1.19
72.546	0.894 16.643	1.099 83.067
ENSMUSG00000033595	Lgi3	protein_coding	1.157 34.077	0.84
87.041	1.027 86.112	1.437 44.882
ENSMUSG00000033596	Rfwd3	protein_coding	1.102 3.536 0.983 85.95
0.926 20.009	0.939 85.493
ENSMUSG00000033597	Caskin1	protein_coding	0.781 26.217	1.12
95.431	1.144 39.471	1.024 84.29
ENSMUSG00000033610	Pank1	protein_coding	1.024 80.03 0.89	96.984
1.097 82.685	1.017 94.19
ENSMUSG00000033615	Cplx1	protein_coding	0.717 43.816	1.187
93.899	1.173 79.554	0.808 86.699
ENSMUSG00000033618	Map3k13	protein_coding	0.803 2.536 0.933
91.532	1.329 5.864 0.915 90.289
ENSMUSG00000033623	Pcgf3	protein_coding	1.088 0.435 1.018 96.68
0.903 2.333 0.977 86.991
ENSMUSG00000033624	Pdpr	protein_coding	1.012 84.564	1.06
95.431	0.933 60.96 1.013 96.248
ENSMUSG00000033628	Pik3c3	protein_coding	1.047 39.851
0.914 34.058	1.046 37.764	1.007 90.463
ENSMUSG00000033629	Ptplad1	protein_coding	1.079 32.411
0.978 95.211	0.95	37.103	0.96	91.105
ENSMUSG00000033632	AW554918	protein_coding	1.034 12.72 0.994
74.851	0.973 79.255	0.977 93.316
ENSMUSG00000033633	Clec18a	protein_coding	0.958 66.652
1.043 96.984	1	82.778	0.895 95.74
ENSMUSG00000033634	Cml2	protein_coding	1.418 32.058	0.983
95.35 0.717 51.443	0.568 83.724
ENSMUSG00000033644	Piwil2	protein_coding	1.126 81.52 0.963
95.504	0.919 87.686	0.915 94.904
ENSMUSG00000033653	Vps8	protein_coding	1.084 40.88 0.958 97.155
0.961 37.617	0.977 82.85
ENSMUSG00000033658	Ddx19b	protein_coding	1.049 80.03 0.872
62.322	1.09	26.978	1.005 92.381
ENSMUSG00000033669	Zfp7	protein_coding	0.936 68.66 0.921 68.115
1.154 26.978	0.997 92.31
ENSMUSG00000033671	Cep350	protein_coding	1.005 65.388
0.968 95.735	NA	NA	1.038 90.674
ENSMUSG00000033676	Gabrb3	protein_coding	1.106 62.007
1.524 88.533	0.592 27.994	1.401 78.042
ENSMUSG00000033684	Qsox1	protein_coding	0.896 44.787	1.056

84.007 1.06 69.315 1.044 83.067
ENSMUSG00000033685 Ucp2 protein_coding 1.096 39.416 0.737 22
1.234 19.106 1.042 93.964
ENSMUSG00000033688 1300017J02Rik protein_coding 0.723 18.907
0.992 93.146 1.389 49.058 1.029 95.676
ENSMUSG00000033697 Arhgap39 protein_coding 1.007 82.352
0.928 88.881 1.071 62.307 1.015 96.358
ENSMUSG00000033701 Acbd6 protein_coding 1.038 45.61 0.93 84.017
1.038 83.719 1.011 93.413
ENSMUSG00000033703 Fuk protein_coding 0.945 30.352 1.079
82.904 0.982 83.066 0.951 86.991
ENSMUSG00000033705 Stard9 protein_coding 1.312 0.577 0.907
90.053 0.84 4.723 0.897 85.493
ENSMUSG00000033706 Smyd5 protein_coding 1.023 43.019 0.991
97.097 0.985 49.967 1.095 84.246
ENSMUSG00000033707 Lrrc24 protein_coding 0.642 8.267 1.078
83.104 1.445 30.645 1.057 95.273
ENSMUSG00000033712 Ccar2 protein_coding 0.977 60.339 1.006
93.899 1.019 75.155 1.008 95.273
ENSMUSG00000033713 Foxn3 protein_coding 1.091 37.702 1.021
86.793 0.897 8.192 0.949 83.724
ENSMUSG00000033715 Akr1c14 protein_coding 0.899 53.138
1.324 79.261 0.836 67.962 1.49 54.265
ENSMUSG00000033717 Adra2a protein_coding 0.512 0.987 0.929
97.288 2.103 6.693 0.783 84.29
ENSMUSG00000033720 Sfxn5 protein_coding 1.135 9.206 1.075 71.222
0.817 0.72 1.009 92.561
ENSMUSG00000033721 Vav3 protein_coding 1.174 9.805 0.963 81.02
0.883 41.928 0.949 92.12
ENSMUSG00000033722 BC034090 protein_coding 0.997 79.93 1.015
97.129 NA NA 1.185 40.024
ENSMUSG00000033726 Emx1 protein_coding 1 82.705 1
95.329 NA NA 1 96.183
ENSMUSG00000033728 Lrrc14 protein_coding 0.942 28.584
1.059 71.617 1.003 52.972 0.996 95.559
ENSMUSG00000033730 Egr3 protein_coding 0.916 77.763 1.174
95.211 0.93 87.786 1.11 89.498
ENSMUSG00000033731 3300002A11Rik antisense 1 75.196 1
85.418 1 79.426 1 95.273
ENSMUSG00000033732 Sf3b3 protein_coding 1.018 81.647 0.976
94.48 1.008 80.089 1.063 49.514
ENSMUSG00000033735 Spr protein_coding 0.951 62.158 1.046
91.584 1.004 79.776 1.056 83.067
ENSMUSG00000033739 Fkbpl protein_coding 0.873 2.822 NA NA
1.128 12.467 0.985 93.261
ENSMUSG00000033751 Gadd45gip1 protein_coding 0.938 63.283 NA
NA 1.056 23.604 0.963 91.105
ENSMUSG00000033752 Mnd1 protein_coding 0.869 21.393 1.021
87.037 1.125 69.196 1.088 86.209
ENSMUSG00000033760 Rbm4b protein_coding 1.034 86.57 0.991 97.155
0.978 75.637 0.931 83.724
ENSMUSG00000033762 Recql4 protein_coding 0.643 27.202
1.163 93.994 1.333 34.07 1.242 80.497
ENSMUSG00000033763 Mtss1l protein_coding 1.06 84.661 1.1

60.309	0.86	6.007	1.069	86.209
ENSMUSG00000033767	D930015E06Rik	protein_coding	1.057	70.36
0.964	85.116	0.981	75.155	0.86	83.724
ENSMUSG00000033768	Nrxn2	protein_coding	0.965	72.139	0.689
79.475	1.502	35.441	0.964	89.525
ENSMUSG00000033769	Exoc6b	protein_coding	1.068	46.533
0.974	90.053	0.96	70.214	0.945	88.053
ENSMUSG00000033773	Rpap2	protein_coding	0.957	79.709	0.933
74.851	1.119	28.055	1.019	96.071
ENSMUSG00000033777	Tlr13	protein_coding	1.996	0.461	0.812	41.268
0.615	16.051	1.44	36.988
ENSMUSG00000033781	Asb13	protein_coding	1.06	15.456	0.967
91.361	0.977	44.678	0.947	83.724
ENSMUSG00000033788	Dysf	protein_coding	0.781	0.987	1.11	79.261
1.157	13.259	0.978	86.991
ENSMUSG00000033790	Tubgcp5	protein_coding	0.942	72.985
1.078	86.793	0.986	73.027	0.987	92.389
ENSMUSG00000033792	Atp7a	protein_coding	1.221	50.377	0.96
84.017	0.851	86.258	1.066	85.786
ENSMUSG00000033793	Atp6v1h	protein_coding	0.895	8.267	1.077
93.574	1.031	37.741	1.108	72.204
ENSMUSG00000033794	Lpcat2b	protein_coding	1	74.299	1
96.847	1	81.857	1	95.74
ENSMUSG00000033799	BC016423	protein_coding	1.02	74.689
0.911	86.604	1.078	48.772	0.95	91.291
ENSMUSG00000033805	Ephx4	protein_coding	0.989	81.925	1.169
82.904	0.862	41.928	0.987	88.387
ENSMUSG00000033808	Tmem87a	protein_coding	1.089	0.435	0.904
23.928	1.012	56.708	0.988	93.722
ENSMUSG00000033809	Alg3	protein_coding	0.964	52.726	1.13
83.104	0.915	72.464	0.971	96.287
ENSMUSG00000033813	Tcea1	protein_coding	1.026	64.193	1.022
96.843	0.955	53.7	1.007	94.297
ENSMUSG00000033819	Ppp1r16a	protein_coding	0.867	0.987	1.087
64.304	1.061	20.085	0.994	91.65
ENSMUSG00000033825	Tpsb2	protein_coding	1.7	0	0.84	62.322
0.698	8.907	0.954	95.72
ENSMUSG00000033826	Dnah8	protein_coding	1.297	21.278	0.854
92.474	0.9	60.233	0.803	84.29
ENSMUSG00000033831	Fgb	protein_coding	0.352	15.227	0.714
49.859	3.968	6.069	1.941	82.091
ENSMUSG00000033834	Tpbpa	protein_coding	1	83.726	NA	NA
NA	NA	1	95.74
ENSMUSG00000033845	Mrpl15	protein_coding	0.972	52.9	1.012
95.431	1.012	56.125	1.04	68.502
ENSMUSG00000033847	Pla2g4c	protein_coding	1.244	26.863
0.805	73.166	0.996	83.607	1.006	95.676
ENSMUSG00000033849	B3galt2	protein_coding	1.171	78.054
0.925	94.96	0.921	86.258	0.701	84.29
ENSMUSG00000033850	Olfr1015	protein_coding	1	84.738	1
96.138	1	86.084	1	96.358
ENSMUSG00000033852	Pla2g4b	protein_coding	1	85.77	1
97.263	1	86.258	1	94.752
ENSMUSG00000033855	Ston1	protein_coding	0.832	3.07	1.003	90.378

1.199 6.693 0.893 85.636
ENSMUSG00000033857 Engase protein_coding 1.116 11.928
0.992 91.361 0.904 39.079 1.021 95.74
ENSMUSG00000033860 Fgg protein_coding 0.764 43.435 0.617
81.731 2.122 21.462 0.842 88.505
ENSMUSG00000033862 Cdk10 protein_coding 0.945 15.76 0.998 95.504
1.06 31.317 1.014 92.691
ENSMUSG00000033863 Klf9 protein_coding 1.124 41.951 0.9
68.818 0.99 85.935 1.046 93.612
ENSMUSG00000033871 Ppargc1b protein_coding 0.98 43.435
0.921 88.691 1.105 26.498 0.685 83.724
ENSMUSG00000033872 Best4-ps pseudogene 1 80.51 1 90.895
1 74.853 1 95.676
ENSMUSG00000033880 Lgals3bp protein_coding 1.154 13.492
1.131 83.104 0.767 49.453 1.162 40.894
ENSMUSG00000033882 Rbm46 protein_coding 1.17 56.66 0.906 91.361
0.945 80.163 0.825 91.269
ENSMUSG00000033883 D3Ertd254e protein_coding 1.004 79.577
1.097 80.277 0.906 6.858 0.976 91.985
ENSMUSG00000033885 Pxk protein_coding 1.032 69.089 0.986
96.948 0.979 60.96 1.086 71.653
ENSMUSG00000033898 Cfhr2 protein_coding 1.186 86.351 0.567
87.857 1.488 56.497 0.795 93.964
ENSMUSG00000033900 Map9 protein_coding 0.814 22.481 1.099
82.904 1.12 85.464 1.057 85.786
ENSMUSG00000033902 Mapkbp1 protein_coding 1.165 31.24 0.908
55.71 0.948 87.173 0.912 87.992
ENSMUSG00000033904 Ccp110 protein_coding 0.873 14.075
1.085 92.111 1.055 31.761 1 92.748
ENSMUSG00000033906 Zdhhc15 protein_coding 1.345 3.498 0.87
78.51 0.853 22.735 0.871 84.29
ENSMUSG00000033909 Usp36 protein_coding 0.939 36.259 1.028
90.116 1.037 61.743 1.013 91.928
ENSMUSG00000033910 Gucy1a3 protein_coding 1.168 11.044
0.978 97.288 0.872 10.602 0.979 89.525
ENSMUSG00000033916 Chmp2a protein_coding 0.922 4.73 1.056
91.065 1.028 20.315 1.044 31.133
ENSMUSG00000033917 Gde1 protein_coding 0.944 24.817 NA NA
1.065 37.369 0.938 83.724
ENSMUSG00000033918 Parl protein_coding 1.013 85.728 0.998
89.509 0.987 84.724 1.026 74.193
ENSMUSG00000033931 Rbm34 protein_coding 1.034 56.085 0.937
44.268 1.029 25.455 0.985 93.964
ENSMUSG00000033933 Vhl protein_coding 1.005 78.428 1.09
71.617 0.914 15.465 0.981 95.273
ENSMUSG00000033938 Ndufb7 protein_coding 0.734 1.031 1.186
80.775 1.15 6.506 1.018 85.786
ENSMUSG00000033940 Brk1 protein_coding 1.02 40.065 0.967
49.859 1.013 55.109 1.037 69.918
ENSMUSG00000033943 Mga protein_coding 1.007 68.66 0.875 81.02
1.137 35.673 0.969 87.735
ENSMUSG00000033948 Zswim5 protein_coding 1.012 71.36 0.771
70.971 1.278 24.383 0.659 83.724
ENSMUSG00000033949 Trim36 protein_coding 1.18 26.863 0.98

82.073 0.863 77.294 1.027 94.82
ENSMUSG00000033952 Aspm protein_coding 1.445 35.371 0.659
73.766 1.052 86.985 1.258 90.125
ENSMUSG00000033953 Ppp3r1 protein_coding 1.082 37.091
0.979 93.707 0.942 12.948 1.063 46.846
ENSMUSG00000033955 Tnks1bp1 protein_coding 0.876 5.544 1.056
64.304 1.082 53.882 1.043 83.067
ENSMUSG00000033960 9430020K01Rik protein_coding 0.931 45.229
1.075 92.062 0.997 76.498 1.022 94.016
ENSMUSG00000033961 Zfp446 protein_coding 0.93 25.551
1.065 83.104 1.005 80.015 0.949 90.95
ENSMUSG00000033963 Fancd2os protein_coding 1 84.263 1
96.843 1 76.388 1 95.877
ENSMUSG00000033964 Zbtb41 protein_coding 0.922 19.985
0.988 87.553 1.098 15.411 0.913 85.493
ENSMUSG00000033965 Slc16a2 protein_coding 1.404 0.2 1.089
79.367 0.651 0.72 0.998 90.805
ENSMUSG00000033966 Cdkl4 protein_coding 2.14 2.723 0.788 85.793
0.595 9.131 2.132 80.573
ENSMUSG00000033967 2310014L17Rik protein_coding 1.241 18.388
0.968 95.708 0.831 12.948 0.963 85.493
ENSMUSG00000033970 Rfc3 protein_coding 1.091 27.113 0.955
82.848 0.962 66.425 1.187 64.513
ENSMUSG00000033972 Zfp944 protein_coding 1.103 53.809
0.947 77.598 0.959 88.693 1.044 87.886
ENSMUSG00000033981 Gria2 protein_coding 1 83.418 1
90.287 1 75.527 1 92.181
ENSMUSG00000033983 Coil protein_coding 0.987 80.51 0.971 85.116
1.047 70.328 1.034 92.903
ENSMUSG00000033985 Tesk2 protein_coding 0.844 8.002 1.129 96.268
1.046 30.645 1.003 93.964
ENSMUSG00000033987 Dnah17 protein_coding 0.753 3.956 1.067
96.652 1.248 10.819 0.865 83.952
ENSMUSG00000033991 Ttc37 protein_coding 0.931 50.229 0.95
82.503 1.132 18.208 1.071 50.452
ENSMUSG00000033993 1700018A14Rik protein_coding 1 83.09 1
96.843 1 87.898 1 95.74
ENSMUSG00000033998 Kcnk1 protein_coding 1.041 81.24 1.003 88.691
0.953 83.925 0.968 93.132
ENSMUSG00000034000 Neu4 protein_coding 1 69.985 1
97.049 NA NA 1 94.82
ENSMUSG00000034006 Pqlc1 protein_coding 1.145 1.913 0.955 84.594
0.917 8.626 0.958 83.724
ENSMUSG00000034007 Scaper protein_coding 1.116 15.662
0.939 75.345 0.952 25.345 1.004 93.045
ENSMUSG00000034021 Pds5b protein_coding 1.027 40.333 1.04
93.707 0.939 28.237 1.006 71.653
ENSMUSG00000034022 Cpsf1 protein_coding 0.928 4.099 0.975 34.058
1.102 5.864 1.011 93.457
ENSMUSG00000034023 Fancd2 protein_coding 1.251 17.761
0.841 37.426 0.946 86.258 1.124 93.413
ENSMUSG00000034024 Cct2 protein_coding 1.024 85.832 0.961
77.944 1.017 62.237 1.053 42.736
ENSMUSG00000034028 Cd226 protein_coding 1.476 0.2 NA NA

0.716 8.626 1.301 72.369
ENSMUSG00000034031 1700106J16Rik protein_coding 1 75.298
1 95.708 1 87.173 1 95.273
ENSMUSG00000034032 Rpap1 protein_coding 0.951 17.031 0.956
87.452 1.101 14.449 1.034 83.067
ENSMUSG00000034035 Ccdc17 protein_coding 1.073 56.311
0.905 86.071 1.028 87.519 0.92 87.651
ENSMUSG00000034037 Fgd5 protein_coding 0.85 34.572 1.184
83.104 0.993 85.935 1.019 89.294
ENSMUSG00000034039 NA NA NA NA 1 95.504 1
78.713 1 93.722
ENSMUSG00000034040 Wbscr17 protein_coding 1.075 53.228
1.004 93.654 0.929 72.269 1.045 92.389
ENSMUSG00000034041 Lyl1 protein_coding 1.225 5.527 0.826 62.322
0.987 81.525 1.021 96.248
ENSMUSG00000034042 Gpbp1l1 protein_coding 0.948 8.709 0.989
96.216 1.063 6.202 0.976 93.612
ENSMUSG00000034055 Phka1 protein_coding 1.313 4.874 1.057 82.103
0.718 0.72 0.996 95.877
ENSMUSG00000034057 Myrfl protein_coding 1.526 82.092 0.814
96.216 0.808 80.089 0.902 95.72
ENSMUSG00000034059 Ypel4 protein_coding 0.867 17.898 0.905
87.041 1.272 10.819 0.996 91.372
ENSMUSG00000034063 4930590J08Rik protein_coding 0.706 52.018
0.979 96.268 1.44 37.764 0.441 87.735
ENSMUSG00000034064 Poglut1 protein_coding 1.021 27.113
1.007 92.545 0.971 65.902 1.014 92.018
ENSMUSG00000034066 Farp2 protein_coding 0.95 25.551 0.98
95.211 1.075 30.107 1.005 95.74
ENSMUSG00000034071 Zfp551 protein_coding 0.966 76.968
0.943 90.046 1.096 30.213 0.915 94.654
ENSMUSG00000034075 Zdhhc5 protein_coding 1.02 51.826
1.052 61.943 0.933 3.428 1.035 51.238
ENSMUSG00000034083 Ccdc174 protein_coding 1.017 83.169
0.955 63.619 1.026 33.042 0.939 88.387
ENSMUSG00000034087 Nlrp4b protein_coding 1 76.135 1
96.897 1 78.642 1 91.979
ENSMUSG00000034088 Hdlbp protein_coding 0.97 35.894 NA NA
1.008 45.449 1.087 0
ENSMUSG00000034098 Fstl5 protein_coding 1 81.24 1 93.899
1 86.831 1 91.928
ENSMUSG00000034101 Ctnnd1 protein_coding 1.018 58.709
1.024 92.561 0.96 20.258 0.978 83.724
ENSMUSG00000034105 Tldc1 protein_coding 0.86 52.258 1.018
82.073 1.143 32.173 0.918 85.636
ENSMUSG00000034107 Ano7 protein_coding 1.761 38.682 0.984
96.138 0.579 36.786 1.082 93.156
ENSMUSG00000034108 Ccs protein_coding 0.943 58.56 0.908 62.692
1.166 11.348 1.013 90.674
ENSMUSG00000034109 Golim4 protein_coding 0.96 43.435
0.972 94.48 1.07 27.047 0.975 94.904
ENSMUSG00000034110 Kctd7 protein_coding 1.343 0.2 1.117 89.494
0.664 0.72 0.969 92.31
ENSMUSG00000034111 Tmed8 protein_coding 1.081 30.984 1.08

81.039	0.854 7.809 0.962 86.991
ENSMUSG00000034112	Atp2c2	protein_coding	0.777 33.002
0.759 82.503	1.696 7.365 0.957 92.181
ENSMUSG00000034115	Scn11a	protein_coding	1.318 80.815
0.957 95.708	0.791 64.155	1.122 95.74
ENSMUSG00000034116	Vav1 protein_coding	1.261 0.2	0.975 92.545
0.813 9.746 1.128 67.169
ENSMUSG00000034117	Gpr44 protein_coding	1.086 58.198	0.636
83.733	1.453 69.315	0.811 90.628
ENSMUSG00000034118	Tpst1 protein_coding	1.139 7.332 1.054 82.904
0.836 3.428 0.911 85.493
ENSMUSG00000034120	Srsf2 protein_coding	1.011 50.627	1.018
96.68 0.973 28.237	1.068 56.863
ENSMUSG00000034121	Mks1 protein_coding	0.884 4.351 0.951 88.691
1.189 7.482 0.991 92.466
ENSMUSG00000034122	Nlrp4d	pseudogene 1	86.112	NA	NA
1	86.831	1	95.273
ENSMUSG00000034126	Pomt2 protein_coding	0.95	35.646	1.089
71.617	0.964 64.427	1.005 94.752
ENSMUSG00000034127	Tspan8	protein_coding	1.343 1.99	1.022
91.955	0.725 8.192 0.87	84.29
ENSMUSG00000034135	Sik3 protein_coding	1.006 81.125	0.991
94.102	NA	NA	0.983 86.699
ENSMUSG00000034139	Serpini2	protein_coding	1	82.705	1
97.263	NA	NA	1	93.156
ENSMUSG00000034151	Zbbx protein_coding	1	79.068	1
96.68 NA	NA	1	94.016
ENSMUSG00000034152	Exoc3 protein_coding	1.075 21.278	0.991
91.361	0.938 53.7	1.021 72.116
ENSMUSG00000034154	Ino80 protein_coding	1.018 51.297	0.955
97.263	1.025 76.768	1.002 94.654
ENSMUSG00000034156	Bzrap1	protein_coding	1.108 39.851	NA
NA	0.845 55.9	0.919 83.724
ENSMUSG00000034157	2310044G17Rik	protein_coding	0.987 71.142
0.995 95.708	1.021 66.269	1.093 87.088
ENSMUSG00000034158	Lrrc58	protein_coding	0.863 28.804	0.99
95.504	1.172 24.791	0.963 93.316
ENSMUSG00000034159	2310007B03Rik	protein_coding	1.635 55.789
0.576 89.509	1.062 87.744	0.647 93.316
ENSMUSG00000034160	Ogt	protein_coding	0.97	40.422	0.988
85.793	1.046 36.011	0.903 86.699
ENSMUSG00000034161	Scx	protein_coding	1.522 11.928	1.029
92.062	0.639 3.943 0.774 83.724
ENSMUSG00000034163	Zfc3h1	protein_coding	0.864 2.644 0.99
85.075	1.165 7.143 0.969 90.95
ENSMUSG00000034164	Emid1 protein_coding	0.827 21.957	1.101
91.643	1.096 46.206	1.017 81.56
ENSMUSG00000034165	Ccnd3 protein_coding	0.867 1.6	1.095 91.643
1.052 8.586 1.048 38.2
ENSMUSG00000034168	Irf2bpl	protein_coding	0.947 24.577
1.105 82.904	0.954 81.525	0.967 95.593
ENSMUSG00000034171	Faah protein_coding	0.814 65.199	0.864
62.025	1.417 18.797	1.147 94.567
ENSMUSG00000034173	2410018M08Rik	protein_coding	1.051 86.474

0.724 27.163 1.316 9.176 0.86 84.19
ENSMUSG00000034175 Rhbdd3 protein_coding 0.824 0.987 1.08
83.104 1.126 7.365 0.937 83.724
ENSMUSG00000034177 Rnf43 protein_coding 0.819 14.403 1.058
83.104 1.157 42.065 0.812 84.29
ENSMUSG00000034185 6430628N08Rik antisense 1 80.03 1
90.053 1 86.258 1 91.078
ENSMUSG00000034187 Nsf protein_coding 1.02 71.516 0.99
90.744 0.988 86.985 1.001 90.674
ENSMUSG00000034189 Hsdl1 protein_coding 1.187 0 0.995 96.138
0.845 0.72 0.919 83.724
ENSMUSG00000034190 Chmp7 protein_coding 0.949 16.325 1.042
87.609 1.007 55.352 1.002 91.492
ENSMUSG00000034192 Lsm3 protein_coding 1.112 9.435 0.941 95.211
0.957 24.163 1.11 50.452
ENSMUSG00000034194 R3hcc1 protein_coding 0.977 57.818
0.982 95.211 1.038 50.851 0.921 83.724
ENSMUSG00000034201 Gas2l1 protein_coding 0.963 15.227
1.076 64.123 0.966 73.096 0.99 93.132
ENSMUSG00000034203 Chchd4 protein_coding 0.907 12.785
1.045 93.899 1.058 31.4 1.089 72.826
ENSMUSG00000034205 Loxl2 protein_coding 1.212 4.628 0.894 74.851
0.924 49.733 1.181 57.1
ENSMUSG00000034206 Polq protein_coding 1.14 60.853 0.879
85.242 0.996 88.461 1.314 60.466
ENSMUSG00000034209 Rasl10a protein_coding 0.921 66.406
1.241 85.756 0.877 68.82 0.98 90.805
ENSMUSG00000034210 Efcab14 protein_coding 1.021 71.896
1.079 60.309 0.906 9.746 0.99 92.748
ENSMUSG00000034211 Mrps17 protein_coding 0.997 78.538 NA
NA 0.952 86.666 1.044 67.691
ENSMUSG00000034212 Ankmy1 protein_coding 0.97 64.956 0.76
70.864 1.36 43.481 0.978 95.676
ENSMUSG00000034216 Vps18 protein_coding 1.071 16.539 0.971
82.848 0.962 54.964 0.953 83.952
ENSMUSG00000034218 Atm protein_coding 0.919 42.034 0.907
91.361 1.2 24.791 1.013 87.651
ENSMUSG00000034219 14-Sep protein_coding 0.941 61.043
1.404 83.104 0.754 64.427 1.009 95.832
ENSMUSG00000034220 Gpc1 protein_coding 1.184 25.735 0.902
79.894 0.934 41.928 1.057 82.328
ENSMUSG00000034224 Slc38a8 protein_coding 1 83.418 1
95.504 1 84.83 1 94.904
ENSMUSG00000034226 Rhov protein_coding 0.831 25.85 1.106 88.91 1.09
51.596 0.898 96.183
ENSMUSG00000034227 Foxj1 protein_coding 1.329 17.191 1.194
83.104 0.631 1.764 0.805 83.724
ENSMUSG00000034235 Usp54 protein_coding 0.971 57.818 0.906
71.331 1.133 13.259 1.016 80.77
ENSMUSG00000034239 Gm884 protein_coding 1 76.135 1
96.216 1 87.173 1 96.139
ENSMUSG00000034243 Golgb1 protein_coding 0.986 62.158 0.99
71.889 1.025 27.533 1.028 93.156
ENSMUSG00000034245 Hdac11 protein_coding 0.966 79.93 0.923

73.166 1.123 28.997 0.799 83.724
ENSMUSG00000034247 Plekhm1 protein_coding 0.994 81.52 1.104
71.222 0.912 7.647 1.02 92.389
ENSMUSG00000034248 Slc25a37 protein_coding 1.179 0 0.931
61.883 0.908 3.432 1.002 92.675
ENSMUSG00000034252 Senp6 protein_coding 1.043 23.254 0.987
96.216 0.971 54.037 1.036 96.183
ENSMUSG00000034254 Agpat1 protein_coding 0.936 40.786
1.183 70.816 0.903 46.071 1.033 92.748
ENSMUSG00000034255 Arhgap27 protein_coding 0.819 0.987 1.081
92.111 1.131 6.202 0.908 83.724
ENSMUSG00000034258 Mfsd7c protein_coding 1.072 38.195
0.964 85.242 0.971 81.62 0.867 88.552
ENSMUSG00000034259 Exosc4 protein_coding 1.037 46.533
1.025 96.948 0.937 49.453 0.975 95.72
ENSMUSG00000034263 Vwa9 protein_coding 0.949 61.043 1.012
97.155 1.041 73.428 1.094 46.412
ENSMUSG00000034265 Zdhhc14 protein_coding 1.099 31.24 1.011
91.532 0.895 67.962 0.891 83.724
ENSMUSG00000034266 Batf protein_coding 1.109 53.228 0.857
85.671 1.051 84.896 1.027 87.447
ENSMUSG00000034269 Setd5 protein_coding 0.95 16.472 0.989
88.691 1.06 11.518 0.982 85.493
ENSMUSG00000034271 Jdp2 protein_coding 1.039 58.38 0.9 88.345
1.067 65.235 1.103 83.067
ENSMUSG00000034274 Thoc5 protein_coding 0.947 21.637 0.987
96.216 1.064 30.645 1.046 56.339
ENSMUSG00000034275 Igsf9b protein_coding 0.876 64.893 NA
NA 1.19 79.426 1.147 93.132
ENSMUSG00000034278 Dnajc17 protein_coding 0.963 60.003 0.89
44.268 1.161 10.079 0.988 94.608
ENSMUSG00000034282 Evpl protein_coding 0.73 3.47 1.096 92.931
1.251 7.635 0.603 84.29
ENSMUSG00000034285 Nipsnap1 protein_coding 0.924 45.502
0.856 86.728 1.266 14.131 0.878 83.724
ENSMUSG00000034290 Nek9 protein_coding 1.039 7.553 0.979 72.399
0.979 37.103 1.028 76.697
ENSMUSG00000034292 Traf3ip1 protein_coding 1.076 13.363
0.944 90.378 0.986 27.321 0.968 83.724
ENSMUSG00000034295 Fhod3 protein_coding 0.938 38.286 1.03
84.007 1.033 88.349 0.915 84.29
ENSMUSG00000034297 Med13 protein_coding 0.995 68.353 0.949
92.137 1.061 50.375 0.926 83.724
ENSMUSG00000034300 Fam53c protein_coding 1.015 24.958 1.04
96.216 0.946 26.057 0.999 86.991
ENSMUSG00000034303 Ccdc15 protein_coding 0.94 73.292
0.983 90.053 1.077 46.575 0.88 86.699
ENSMUSG00000034308 Sdr42e1 protein_coding 1.133 4.228 0.961
86.277 0.915 32.963 1.023 93.264
ENSMUSG00000034311 Kif4 protein_coding 0.857 74.958 1.195
96.216 0.975 85.675 1.456 68.502
ENSMUSG00000034312 Iqsec1 protein_coding 1.045 58.709
1.038 82.904 0.922 21.117 0.985 93.964
ENSMUSG00000034317 Trim59 protein_coding 1.01 85.499 0.85

86.861 1.163 53.587 0.948 82.328
ENSMUSG00000034320 Slc26a2 protein_coding 1.194 0.664 1.02
95.329 0.819 1.58 0.993 83.865
ENSMUSG00000034321 Exosc1 protein_coding 1.036 77.916
1.026 94.89 0.942 45.093 1.02 90.463
ENSMUSG00000034324 Tmem132c protein_coding 1.766 0.2 1.006
91.361 0.56 3.969 1.062 91.65
ENSMUSG00000034327 Kctd9 protein_coding 0.995 63.385 0.978
81.731 1.023 58.414 1.074 57.1
ENSMUSG00000034329 Brip1 protein_coding 1.202 33.325 0.89
90.378 0.937 59.726 1.203 92.748
ENSMUSG00000034330 Plcg2 protein_coding 1.205 0.355 0.856 12.804
0.965 49.217 0.986 96.086
ENSMUSG00000034333 Zbed4 protein_coding 1.048 41.951 1.071
85.378 0.891 16.051 1.076 70.624
ENSMUSG00000034334 Fam151b protein_coding 1.186 15.178
0.946 81.284 0.891 36.499 0.921 83.724
ENSMUSG00000034336 Ina protein_coding 2.602 20.444 0.47
92.914 0.821 56.625 1.025 88.755
ENSMUSG00000034339 Gm5689 protein_coding 1 62.888 1
85.756 1 84.504 1 93.016
ENSMUSG00000034341 Wbp2 protein_coding 0.894 1.274 1.062 82.904
1.053 20.051 1.024 78.948
ENSMUSG00000034342 Cbl protein_coding 1.021 56.477 0.873
91.661 1.123 33.042 0.845 85.947
ENSMUSG00000034343 Ube2f protein_coding 0.98 48.939 1.008
95.431 1.009 84.961 1.019 83.067
ENSMUSG00000034345 Gtf2h5 protein_coding 0.958 39.953
0.971 96.68 1.077 63.665 1.038 82.328
ENSMUSG00000034349 Smc4 protein_coding 1.066 49.517 0.919
73.766 1.023 84.626 1.139 57.1
ENSMUSG00000034353 Ramp1 protein_coding 1.394 1.323 0.767 68.115
0.933 26.057 0.821 88.245
ENSMUSG00000034354 Mtmr3 protein_coding 1.047 13.492 0.978
79.807 0.975 37.103 0.989 86.699
ENSMUSG00000034359 Skint2 protein_coding 1 81.925 1
95.504 1 81.388 1 94.904
ENSMUSG00000034361 Cpne2 protein_coding 1.053 35.024 1.144
72.642 0.831 4.521 0.957 85.493
ENSMUSG00000034362 Csta protein_coding 1 77.113 1
96.68 1 87.744 1 70.539
ENSMUSG00000034371 Dak protein_coding 0.903 18.795 1.034
96.847 1.072 39.471 1.004 96.287
ENSMUSG00000034377 Tulp4 protein_coding 1.003 61.334 0.967
92.474 1.035 53.882 NA NA
ENSMUSG00000034379 Wdr5b protein_coding 0.99 69.24 1.108 72.642
0.913 38.434 1.004 96.183
ENSMUSG00000034382 AI661453 protein_coding 0.855 30.352 1.09
84.768 1.069 64.024 0.951 92.851
ENSMUSG00000034384 Barhl2 protein_coding 1 82.352 1
96.268 1 86.258 1 95.559
ENSMUSG00000034387 Ssu2 protein_coding 0.91 86.74 0.94 72.399
1.17 34.285 1.062 96.192
ENSMUSG00000034390 Cmip protein_coding 1.039 24.481 1.03

97.263 0.934 16.719 0.996 96.358
ENSMUSG00000034394 Lif protein_coding 0.882 40.568 1.113
96.68 1.021 79.325 1.134 52.982
ENSMUSG00000034401 Spata6 protein_coding 1.106 7.049 0.912
68.818 0.986 70.214 1.041 60.968
ENSMUSG00000034402 Kcnh5 protein_coding 1 66.944 1
87.041 1 86.552 1 92.762
ENSMUSG00000034403 Pja1 protein_coding 1.009 83.169 1.005
94.367 0.989 64.155 0.993 94.904
ENSMUSG00000034412 Tbc1d10a protein_coding 1.165 3.061 0.935
87.919 0.914 17.662 0.943 92.31
ENSMUSG00000034413 Neurl1b protein_coding 0.945 50.768
1.194 82.904 0.888 57.819 0.996 89.475
ENSMUSG00000034416 Pkd1l2 protein_coding 0.624 30.352
1.325 88.533 1.21 47.512 1.462 83.067
ENSMUSG00000034422 Parp14 protein_coding 1.157 6.61 1.045
95.809 0.828 6.858 0.985 92.748
ENSMUSG00000034424 Gcsh protein_coding 1.045 32.058 1.073
82.809 0.892 13.643 1.041 88.111
ENSMUSG00000034427 Myo15b protein_coding 0.767 74.482
0.983 95.809 NA NA 1.463 92.389
ENSMUSG00000034429 Zfp707 protein_coding 0.881 1.274 0.965
95.35 1.173 10.523 0.997 95.273
ENSMUSG00000034430 Zxdc protein_coding 1.115 8.685 0.963 84.858
0.933 8.192 1.166 89.052
ENSMUSG00000034432 Cops8 protein_coding 0.991 51.447 0.999
95.431 1.007 78.155 1.136 31.133
ENSMUSG00000034435 Tmem30b protein_coding 1.08 51.297
0.855 68.818 1.085 81.857 1.022 88.552
ENSMUSG00000034437 Gm9761 pseudogene 1 53.138 1
84.007 1 82.74 1 90.177
ENSMUSG00000034438 Gbp8 protein_coding 1.643 2.567 1.108 89.494
0.549 1.705 0.836 93.264
ENSMUSG00000034442 Trmt5 protein_coding 1.096 29.057 0.986
96.013 0.927 48.851 1.058 80.77
ENSMUSG00000034445 Cyb561a3 protein_coding 1.081 17.582
0.942 92.914 0.977 46.071 0.937 84.29
ENSMUSG00000034449 Dhrs11 protein_coding 0.856 12.436 1.1
86.793 1.059 53.882 1.026 93.584
ENSMUSG00000034450 Gulo protein_coding 1.647 23.019 1.116
91.361 0.544 63.504 0.769 91.625
ENSMUSG00000034452 Slc24a1 protein_coding 0.652 32.557
1.088 93.899 1.413 65.034 0.629 84.29
ENSMUSG00000034453 Polr3b protein_coding 0.969 81.01 0.988
93.654 1.043 75.155 1.054 59.464
ENSMUSG00000034457 Eda2r protein_coding 1.279 13.633 1.222
88.91 0.639 6.515 1.006 74.193
ENSMUSG00000034459 Ifit1 protein_coding 1.309 1.949 1.027 95.735
0.744 10.362 2.022 44.213
ENSMUSG00000034460 Six4 protein_coding 0.91 18.602 1.116
71.617 0.98 86.258 0.986 96.287
ENSMUSG00000034462 Pkd2 protein_coding 0.975 49.368 0.987
74.851 1.034 20.886 0.999 94.99
ENSMUSG00000034463 Scara3 protein_coding 1.378 0.664 0.971

97.263 0.745 3.654 1.033 93.156
ENSMUSG00000034467 Dynlrb2 protein_coding 1.762 59.527
0.546 91.955 1.034 78.155 0.401 85.493
ENSMUSG00000034471 Caskin2 protein_coding 0.864 5.311 1.106
83.104 1.046 44.361 1.044 94.001
ENSMUSG00000034472 Rasd2 protein_coding 2.559 0 1.009 92.355
0.387 0.72 0.739 86.991
ENSMUSG00000034473 Sec22a protein_coding 1.014 54.344
1.084 80.775 0.911 12.948 0.989 96.042
ENSMUSG00000034480 Diap2 protein_coding 1.051 30.282 0.975
90.046 0.97 64.778 0.993 95.74
ENSMUSG00000034482 Ly6g5c protein_coding 1 77.763 1
95.708 NA NA 1 94.608
ENSMUSG00000034484 Snx2 protein_coding 1.245 0 0.952 77.944
0.841 0.879 1.241 40.894
ENSMUSG00000034485 Uaca protein_coding 0.918 21.658 0.917
94.262 1.183 26.912 1.014 90.674
ENSMUSG00000034486 Gbx2 protein_coding 1 84.168 1
96.984 1 82.31 1 90.455
ENSMUSG00000034487 Kdelc2 protein_coding 1.087 8.996 1.033
93.654 0.891 32.297 1.1 38.598
ENSMUSG00000034488 Edil3 protein_coding 1.194 35.748 1.037
96.897 0.806 39.079 1.253 94.016
ENSMUSG00000034493 4930556J24Rik protein_coding 0.894 28.804
0.937 83.58 1.196 19.106 0.938 89.587
ENSMUSG00000034501 Pcnxl4 protein_coding 1.002 84.661
0.945 84.017 1.052 53.449 1.005 92.748
ENSMUSG00000034509 Mad2l1bp protein_coding 1.065 54.717
1.016 92.062 0.926 23.48 1.076 86.445
ENSMUSG00000034518 Hmgxb4 protein_coding 1.033 65.537
0.994 90.046 0.975 73.598 1.011 95.405
ENSMUSG00000034520 Gjc1 protein_coding 1.088 29.372 0.939
87.553 0.982 75.637 0.961 92.466
ENSMUSG00000034522 Zfp395 protein_coding 0.931 40.786 NA
NA 1.113 51.977 0.963 84.29
ENSMUSG00000034525 BC018507 protein_coding 1.005 67.163
0.939 41.951 1.061 37.369 0.996 86.699
ENSMUSG00000034528 Hsd17b13 protein_coding 2.535 4.228 0.651
92.137 0.605 22.735 1.321 71.855
ENSMUSG00000034532 Fbxo16 protein_coding 0.82 80.146
1.211 96.013 1.001 85.464 0.932 96.192
ENSMUSG00000034533 Scn10a protein_coding 1 67.374 1
85.075 1 88.076 1 95.273
ENSMUSG00000034538 Zfp418 protein_coding 0.967 79.709 NA
NA NA NA 1.153 63.708
ENSMUSG00000034543 Morc2a protein_coding 1.019 69.24 1.004
94.848 0.976 76.754 0.989 92.389
ENSMUSG00000034544 Rsrc1 protein_coding 0.948 16.472 NA NA
1.083 27.89 1.014 82.85
ENSMUSG00000034551 Hdx protein_coding 1.119 75.196 1.147
83.104 0.778 22.864 0.837 90.805
ENSMUSG00000034555 Tex16 protein_coding 1 78.224 1
94.89 1 78.369 1 96.287
ENSMUSG00000034557 Zfyve9 protein_coding 1.002 83.523

1.004 96.216 0.994 80.749 1.028 70.85
ENSMUSG00000034560 A230046K03Rik protein_coding 1.052 10.595
0.97 74.633 0.983 82.778 1.011 93.016
ENSMUSG00000034563 Ccpg1 protein_coding 0.959 85.832 0.962
60.678 1.084 33.787 1.091 83.067
ENSMUSG00000034566 Atp5h protein_coding 0.921 46.413 1.013
92.137 1.074 39.925 1.047 80.497
ENSMUSG00000034570 Inpp5j protein_coding 0.956 71.36 0.891
84.594 1.174 45.94 1.085 93.722
ENSMUSG00000034573 Ptpn13 protein_coding 1.056 60.435
1.013 97.263 0.935 66.753 1.041 80.324
ENSMUSG00000034574 Daam1 protein_coding 1.039 23.82 1.02 90.116
0.943 24.309 0.991 92.675
ENSMUSG00000034575 Papd7 protein_coding 1.139 0.355 1.042 82.904
0.842 0.72 1.067 67.94
ENSMUSG00000034579 Pla2g3 protein_coding 0.844 27.664
1.327 72.642 0.895 88.657 0.893 95.92
ENSMUSG00000034583 Olfr1347 protein_coding 1 83.658 1
96.001 1 71.839 1 94.904
ENSMUSG00000034584 Exph5 protein_coding 0.837 44.562 0.898
95.088 1.327 32.776 0.769 86.991
ENSMUSG00000034586 Hid1 protein_coding 0.964 57.544 1.027
93.863 1.012 70.328 0.928 86.699
ENSMUSG00000034587 8430429K09Rik processed_transcript 0.85
12.181 1.069 82.904 1.099 44.787 1.128 80.77
ENSMUSG00000034591 Slc41a2 protein_coding 1.319 1.412 0.965
91.661 0.785 6.007 1.274 57.711
ENSMUSG00000034593 Myo5a protein_coding 0.983 84.452 0.911
64.555 1.109 31.479 1.017 93.156
ENSMUSG00000034595 Ppp1r18 protein_coding 1.035 59.806
1.007 83.104 0.96 16.643 1.026 60.968
ENSMUSG00000034601 2700049A03Rik protein_coding 0.968 69.24
1.058 83.104 0.973 76.388 1.025 93.457
ENSMUSG00000034602 Mon2 protein_coding 0.994 85.129 0.986
85.793 1.015 65.235 1.029 69.918
ENSMUSG00000034607 Pof1b protein_coding 0.999 86.74 0.952 94.96 1.05
84.724 1.034 91.105
ENSMUSG00000034610 Zcchc11 protein_coding 1.073 20.444
1.032 84.858 0.902 34.592 0.972 84.29
ENSMUSG00000034612 Chst11 protein_coding 1.149 24.481 1.11
88.923 0.785 18.797 0.932 84.29
ENSMUSG00000034613 Ppm1h protein_coding 1.157 5.527 0.986 82.671
0.876 16.949 0.94 84.29
ENSMUSG00000034614 Pik3ip1 protein_coding 1.046 74.021
0.717 53.434 1.328 10.819 0.771 94.654
ENSMUSG00000034616 Ssh3 protein_coding 0.827 0.987 0.984 95.211
1.231 5.864 0.93 83.724
ENSMUSG00000034617 Mtrr protein_coding 1.024 54.176 0.961
96.013 1.013 56.708 1.051 57.1
ENSMUSG00000034620 Tmem5 protein_coding 1.087 3.152 0.996 92.631
0.922 3.428 1.004 91.335
ENSMUSG00000034621 Gpatch8 protein_coding 0.979 22.431
1.024 82.904 0.999 83.538 0.928 83.724
ENSMUSG00000034634 Ly6d protein_coding 1.279 6.23 0.822 41.951

0.949 68.584 1.047 90.805
ENSMUSG00000034636 Zyg11b protein_coding 1.052 58.896
0.974 90.053 0.975 85.352 1.005 94.904
ENSMUSG00000034639 Setmar protein_coding 1.057 69.489
0.907 85.921 1.04 84.896 0.925 83.724
ENSMUSG00000034640 Tiparp protein_coding 1.155 7.799 NA NA
0.785 9.288 1.162 71.305
ENSMUSG00000034641 Cd300ld protein_coding 1.815 0 0.854
75.345 0.642 3.654 1.34 68.696
ENSMUSG00000034645 Zyg11a protein_coding 0.647 20.369
1.293 82.904 1.193 73.428 1.011 96.183
ENSMUSG00000034647 Ankrd12 protein_coding 1.165 1.323 0.952
65.819 0.899 13.848 1.002 87.624
ENSMUSG00000034648 Lrrn1 protein_coding 0.972 74.689 0.998
94.102 1.028 45.035 0.997 94.567
ENSMUSG00000034652 Cd300a protein_coding 1.402 0.2 0.811
35.072 0.878 26.057 1.118 93.734
ENSMUSG00000034653 Ythdc2 protein_coding 0.996 85.637
0.948 91.361 1.056 79.119 0.992 96.086
ENSMUSG00000034656 Cacna1a protein_coding 1.009 74.482
1.084 91.456 0.91 32.297 1.233 61.316
ENSMUSG00000034659 Tmem109 protein_coding 0.915 14.526
1.101 83.104 0.991 79.325 1.01 83.067
ENSMUSG00000034660 Galp protein_coding 1 70.741 1
96.948 1 78.954 1 96.183
ENSMUSG00000034663 Bmp2k protein_coding 1.121 24.147 1.013
92.561 0.882 7.742 1.046 83.067
ENSMUSG00000034664 Itga2b protein_coding 1.251 21.278 NA
NA 0.862 23.48 1.28 31.133
ENSMUSG00000034667 Xpot protein_coding 0.986 83.523 1.042
91.643 0.972 49.217 1.003 92.389
ENSMUSG00000034673 Pbx2 protein_coding 0.929 63.283 1.117
82.76 0.963 76.64 0.986 92.181
ENSMUSG00000034674 Tdg protein_coding 0.984 69.985 0.928
60.806 1.091 17.998 1.043 79.268
ENSMUSG00000034675 Dbn1 protein_coding 0.948 44.922 1.04
96.216 1.013 53.882 1.391 31.133
ENSMUSG00000034677 Gpr142 protein_coding 1 84.819 1
96.378 1 88.729 1 94.904
ENSMUSG00000034681 Rnps1 protein_coding 1.012 86.658 0.947
72.399 1.046 48.304 0.992 96.183
ENSMUSG00000034684 Sema3f protein_coding 0.859 7.916 1.125
71.617 1.036 68.425 0.957 84.29
ENSMUSG00000034685 Fam171a2 protein_coding 1.017 86.658 NA
NA 1.005 88.349 1.01 83.581
ENSMUSG00000034686 Prr7 protein_coding 0.784 17.401 1.398
71.617 0.914 54.964 0.904 92.181
ENSMUSG00000034687 Fras1 protein_coding 1.505 12.855 0.745
82.503 0.89 58.775 0.839 90.611
ENSMUSG00000034689 4921530L21Rik protein_coding 1 80.51 1
94.887 1 84.229 1 96.183
ENSMUSG00000034690 Nlrp4c protein_coding 1 51.089 1
95.211 1 85.757 1 93.457
ENSMUSG00000034701 Neurod1 protein_coding 1 79.93 1

93.654	1	84.896	1	95.405
ENSMUSG00000034706	Dnaic2	protein_coding	1.342	22.349
0.868	97.129	0.86	18.539	1.088	75.198
ENSMUSG00000034707	Gns	protein_coding	1.112	13.363	0.944
57.613	0.951	49.967	0.979	87.331
ENSMUSG00000034708	Grn	protein_coding	1.334	0	0.927	60.678
0.805	6.381	1.001	92.381
ENSMUSG00000034709	Ppp1r21	protein_coding	1.075	16.249
0.878	15.364	1.06	42.199	1.071	83.067
ENSMUSG00000034714	Ttyh2	protein_coding	0.946	81.196	1.046
89.434	1.011	58.775	1.224	58.557
ENSMUSG00000034723	Tmx4	protein_coding	1.072	26.338	0.988
96.268	0.947	30.468	0.937	83.724
ENSMUSG00000034724	Cnot6l	protein_coding	1.028	72	0.913
56.955	1.061	26.663	0.965	92.181
ENSMUSG00000034729	Mrps10	protein_coding	0.943	8.709	0.999
97.155	1.063	10.819	1.005	83.067
ENSMUSG00000034730	Bai1	protein_coding	1.175	60.435	1.115
85.985	0.763	24.918	1.054	83.276
ENSMUSG00000034731	Dgkh	protein_coding	0.829	24.817	0.996
93.908	1.213	9.176	0.814	84.19
ENSMUSG00000034732	Pabpc5	protein_coding	0.719	84.397
0.709	75.052	1.965	26.663	1.484	75.81
ENSMUSG00000034738	Nostrin	protein_coding	1.486	1.846	0.856
90.138	0.784	13.551	1.102	63.708
ENSMUSG00000034739	Mfrp	protein_coding	0.644	44.922	0.988
95.504	1.572	45.035	0.722	87.331
ENSMUSG00000034744	Nagk	protein_coding	1.146	1.292	0.911	58.64	0.96
27.994	0.99	94.608
ENSMUSG00000034748	Sirt6	protein_coding	0.933	19.365	1.042
96.897	1.029	25.144	1.052	79.268
ENSMUSG00000034751	Mast4	protein_coding	1.176	7.908	0.921	66.651
0.922	21.666	0.993	96.192
ENSMUSG00000034755	Pcdh11x	protein_coding	1.464	62.354
0.992	95.431	0.689	66.753	1.058	91.525
ENSMUSG00000034757	Tmub2	protein_coding	0.992	80.03	1.031	83.104
0.974	72.269	0.997	92.675
ENSMUSG00000034758	Tle6	protein_coding	1.114	36.124	0.76
28.822	1.182	28.74	0.696	94.468
ENSMUSG00000034761	Map4k5	protein_coding	1.035	25.207
1.011	96.308	0.956	38.799	0.911	75.345
ENSMUSG00000034762	Glis1	protein_coding	1.159	42.134	0.875
95.301	0.985	86.727	0.985	92.12
ENSMUSG00000034764	1700006J14Rik	processed_transcript	1.222
41.951	1.11	96.68	0.735	24.523	0.926	86.991
ENSMUSG00000034765	Dusp5	protein_coding	0.831	54.604	0.961
95.708	1.247	38.26	1.001	96.139
ENSMUSG00000034768	Asb16	protein_coding	3.19	10.464	1.499
83.104	0.208	1.898	0.782	96.183
ENSMUSG00000034771	Tle2	protein_coding	1.042	74.958	0.904
79.475	1.065	52.8	0.907	91.625
ENSMUSG00000034773	BC030867	protein_coding	1.079	34.861
0.706	79.807	NA	NA	2.298	44.679
ENSMUSG00000034780	B3galt1	protein_coding	1.322	25.735	NA

NA 0.71 28.489 0.856 85.636
ENSMUSG00000034781 Gna11 protein_coding 0.938 19.224 1.045
87.029 1.021 56.125 0.995 90.289
ENSMUSG00000034785 Dio1 protein_coding 1 84.661 1
83.321 1 62.995 1 96.086
ENSMUSG00000034786 Gpsm3 protein_coding 1.002 65.869 0.969
95.809 1.029 86.666 0.98 89.525
ENSMUSG00000034789 Rab24 protein_coding 0.932 6.409 0.93 65.819
1.151 6.506 1.055 49.806
ENSMUSG00000034792 Gna15 protein_coding 1.475 4.916 0.854 83.876
0.796 15.024 1.182 59.394
ENSMUSG00000034793 G6pc3 protein_coding 0.962 46.693 1.063
72.546 0.976 64.155 0.95 83.724
ENSMUSG00000034795 Ccdc122 protein_coding 1.292 2.233 0.925
97.263 0.833 0.945 1.008 90.611
ENSMUSG00000034796 Cpne7 protein_coding 0.856 35.158 1.248
83.104 0.931 86.985 0.906 94.99
ENSMUSG00000034799 Unc13a protein_coding 0.875 30.035
0.942 91.83 1.209 29.568 0.904 88.053
ENSMUSG00000034800 Zfp661 protein_coding 0.903 17.062
0.956 83.733 1.156 8.339 0.971 94.608
ENSMUSG00000034801 Sos2 protein_coding 1.041 28.098 1.05
95.735 0.91 23.48 0.979 83.952
ENSMUSG00000034807 Colgalt1 protein_coding 1.068 8.685 0.962
73.766 0.975 54.25 1.081 56.769
ENSMUSG00000034810 Scn7a protein_coding 0.914 7.58 1.037 83.104
1.055 54.163 0.949 84.29
ENSMUSG00000034813 Grip1 protein_coding 0.79 46.693 1.254
82.904 1.004 80.381 1.15 91.011
ENSMUSG00000034818 Celf5 protein_coding 0.896 86.464 1.144
96.407 0.977 87.37 0.955 86.699
ENSMUSG00000034820 Cpsf7 protein_coding 0.992 33.906 0.955
90.378 1.052 25.455 0.998 95.74
ENSMUSG00000034825 Nrip3 protein_coding 1.191 67.867 0.991
95.088 0.847 74.082 1.265 70.36
ENSMUSG00000034826 Nup54 protein_coding 1.043 58.709 0.983
86.728 0.973 88.693 1.031 79.442
ENSMUSG00000034829 Nxnl1 protein_coding 0.655 4.176 1.069 93.27
1.423 7.635 1.018 96.042
ENSMUSG00000034832 Tet3 protein_coding 0.969 64.284 1.131
71.98 0.908 30.024 0.963 84.29
ENSMUSG00000034833 Tespa1 protein_coding 1.195 59.806
1.229 96.847 0.68 41.506 1.172 79.268
ENSMUSG00000034839 Larp6 protein_coding 1.038 58.896 0.935
79.894 1.027 78.864 1.076 78.948
ENSMUSG00000034842 Art3 protein_coding 1.054 74.482 1.031
96.897 0.917 69.583 0.974 96.192
ENSMUSG00000034845 Plvap protein_coding 1.053 29.811 0.95
91.361 1.002 73.874 0.981 95.777
ENSMUSG00000034848 Ttc21b protein_coding 0.996 82.732
0.938 95.504 1.072 85.757 1.017 91.372
ENSMUSG00000034850 Tmem127 protein_coding 1.068 0.664 1.016
83.104 0.924 0.72 1.006 96.139
ENSMUSG00000034853 Acot11 protein_coding 0.964 84.168

0.781 83.733 1.324 66.269 1.283 80.497
ENSMUSG00000034854 Mfsd12 protein_coding 0.864 69.943
1.203 82.904 0.961 45.799 1.096 42.736
ENSMUSG00000034855 Cxcl10 protein_coding 1.005 65.388
1.494 60.309 0.664 7.509 0.809 85.493
ENSMUSG00000034858 Fam214a protein_coding 0.907 49.621
0.711 38.699 1.555 5.864 1.232 57.1
ENSMUSG00000034863 Ano8 protein_coding 0.918 28.804 1.01
89.434 1.08 57.907 0.988 94.99
ENSMUSG00000034867 Ankrd27 protein_coding 1.06 14.746
1.013 94.367 0.932 12.41 1.011 95.92
ENSMUSG00000034868 Myl12b protein_coding 0.924 1.274 1.026
88.092 1.051 11.348 1.006 84.246
ENSMUSG00000034871 Fam151a protein_coding 0.572 51.056 NA
NA 1.324 58.915 1.346 95.92
ENSMUSG00000034872 Gipc3 protein_coding 0.786 44.467 1.172
83.104 NA NA 0.996 91.625
ENSMUSG00000034875 Nudt19 protein_coding 0.988 68.846
1.089 79.261 0.929 36.499 0.968 93.156
ENSMUSG00000034880 Mrpl34 protein_coding 0.801 3.325 1.08
83.464 1.159 11.518 0.972 90.95
ENSMUSG00000034881 Tbxa2r protein_coding 1.022 46.013
0.932 94.48 1.045 77.294 1.055 96.287
ENSMUSG00000034883 Lrr1 protein_coding 1.411 41.454 0.535
88.897 1.321 77.037 2.149 64.513
ENSMUSG00000034889 Cactin protein_coding 0.912 34.795
1.151 83.819 0.954 86.727 0.927 85.493
ENSMUSG00000034892 Rps29 protein_coding 1.034 66.944 0.93
94.597 1.04 70.9 1.141 44.679
ENSMUSG00000034893 Cog3 protein_coding 1.024 65.284 0.96
85.793 1.021 85.352 1.02 90.674
ENSMUSG00000034898 Filip1 protein_coding 1.049 45.746
0.941 85.95 1.011 73.027 0.917 90.289
ENSMUSG00000034902 Pip5k1c protein_coding 0.989 68.727
1.003 93.765 1.009 88.203 1.002 88.505
ENSMUSG00000034903 Cobll1 protein_coding 0.981 70.862
0.993 95.211 1.022 66.043 0.98 96.183
ENSMUSG00000034906 Ncaph protein_coding 1.034 83.169 NA NA
0.933 86.475 1.511 50.74
ENSMUSG00000034908 Sidt2 protein_coding 1.023 59.527 1.041
82.904 0.94 14.079 0.957 85.493
ENSMUSG00000034910 Pygo1 protein_coding 1.2 11.928 0.915
94.848 0.911 8.142 0.938 83.724
ENSMUSG00000034911 Ushbp1 protein_coding 0.888 21.025
0.929 85.671 1.213 11.782 0.939 83.724
ENSMUSG00000034912 Mdga2 protein_coding 1.799 21.278 0.609
88.705 0.913 87.431 1.091 96.086
ENSMUSG00000034913 Spert protein_coding 1 77.497 NA NA
1 73.531 1 94.567
ENSMUSG00000034917 Tjp3 protein_coding 0.717 7.43 1.088 94.767
1.282 17.998 0.775 83.724
ENSMUSG00000034919 Ttc22 protein_coding 0.847 9.863 0.931 90.378
1.271 7.635 0.964 93.685
ENSMUSG00000034923 Ly6g6f protein_coding 2.524 61.49 0.542

95.504 0.734 80.163 0.709 86.991
ENSMUSG00000034926 Dhcr24 protein_coding 0.817 19.83 1.026
92.062 1.193 42.065 1.12 73.368
ENSMUSG00000034928 Rnf44 protein_coding 0.938 1.803 1.028 80.806
1.035 30.292 0.902 83.724
ENSMUSG00000034930 Rtkn protein_coding 0.796 4.385 1.006 88.705
1.25 6.202 0.979 91.078
ENSMUSG00000034931 Dhx8 protein_coding 0.993 73.585 1.009
96.843 0.995 85.921 1.033 90.177
ENSMUSG00000034932 Mrpl54 protein_coding 0.894 3.853 1.044
83.104 1.075 22.517 1.093 83.067
ENSMUSG00000034936 Arl4d protein_coding 0.895 30.352 0.917
90.576 1.214 21.873 0.872 83.724
ENSMUSG00000034940 Synrg protein_coding 1.013 66.58 1.025 96.68
0.962 47.641 0.982 91.269
ENSMUSG00000034947 Tmem106a protein_coding 1.334 0 0.952
94.848 0.788 2.294 1.166 40.894
ENSMUSG00000034949 Zfr2 protein_coding 0.811 2.142 0.961 95.088
1.28 21.462 0.982 90.611
ENSMUSG00000034951 Cog7 protein_coding 1.044 32.238 0.955
82.848 1.003 73.598 1.024 89.152
ENSMUSG00000034957 Cebpa protein_coding 0.886 14.086 1.006
96.847 1.12 38.099 0.98 86.991
ENSMUSG00000034958 Atcay protein_coding 1.028 77.615 1.75
81.127 0.554 63.075 1.024 79.268
ENSMUSG00000034959 5031414D18Rik protein_coding 1.146 52.134
0.665 54.06 1.31 38.152 1.353 56.769
ENSMUSG00000034968 Lbx2 protein_coding 0.95 80.251 0.917
96.68 1.146 86.258 0.818 89.587
ENSMUSG00000034973 Dopey1 protein_coding 0.881 26.483
0.925 71.98 1.228 5.864 1.041 90.397
ENSMUSG00000034974 Dapk3 protein_coding 0.901 3.623 0.97 95.211
1.143 7.635 0.967 93.261
ENSMUSG00000034981 Parm1 protein_coding 0.605 0.987 1.269 71.98 1.3
12.35 0.94 95.273
ENSMUSG00000034987 Hrh2 protein_coding 1.275 10.391 1.077
95.735 0.728 7.329 0.937 96.086
ENSMUSG00000034990 Otoa protein_coding 0.853 82.982 1.363
93.654 0.861 84.961 0.451 83.724
ENSMUSG00000034993 Vat1 protein_coding 1.182 1.323 1.02 96.847
0.832 4.48 1.013 96.358
ENSMUSG00000034994 Eef2 protein_coding 1.058 18.985 0.874 0
1.079 19.286 0.992 91.291
ENSMUSG00000034997 Htr2a protein_coding 1.358 29.057 0.814
96.268 0.905 37.103 0.872 91.217
ENSMUSG00000034998 Foxn2 protein_coding 1.062 0.999 1.025 83.104
0.917 1.342 1.015 84.29
ENSMUSG00000035000 Dpp4 protein_coding 1.165 6.541 1.053 87.919
0.811 44.211 0.976 96.192
ENSMUSG00000035004 Igsf6 protein_coding 1.562 0.577 0.976 94.96
0.657 13.643 0.987 96.184
ENSMUSG00000035007 Rundc1 protein_coding 0.961 66.129
1.052 89.996 0.989 80.163 1.041 81.061
ENSMUSG00000035011 Zbtb7a protein_coding 1.009 84.324

1.042 82.904 0.949 22.009 0.989 93.964
ENSMUSG00000035020 Epgn protein_coding 0.827 53.138 2.364
77.085 0.512 56.625 1.751 59.394
ENSMUSG00000035021 Baz1a protein_coding 1.017 86.145 0.999
96.897 0.984 87.744 1.018 83.067
ENSMUSG00000035024 Ncapd3 protein_coding 0.998 63.736
0.959 86.728 1.048 57.227 1.018 93.156
ENSMUSG00000035027 Map2k2 protein_coding 0.892 2.559 1.059
82.904 1.06 45.035 1.036 83.581
ENSMUSG00000035032 Nek11 protein_coding 1.203 38.195 0.773
88.691 1.077 79.119 1.019 95.877
ENSMUSG00000035041 Creb3l3 protein_coding 1.128 74.958 0.88
90.895 1.009 57.091 1.547 50.452
ENSMUSG00000035042 Ccl5 protein_coding 0.786 42.034 1.391
63.504 0.91 80.015 0.837 88.387
ENSMUSG00000035045 Zc3h12b protein_coding 1.09 49.517
0.888 89.277 NA NA 0.998 90.611
ENSMUSG00000035047 Kri1 protein_coding 0.936 2.277 0.959 87.322
1.109 9.301 1.028 61.984
ENSMUSG00000035048 Anapc13 protein_coding 0.853 5.591 1.027
84.126 1.143 37.741 1.052 93.964
ENSMUSG00000035049 Rrp12 protein_coding 0.877 49.749 1.053
90.454 1.078 69.002 1.106 68.696
ENSMUSG00000035051 Dhx57 protein_coding 0.994 81.196 0.999
88.705 1.005 72.148 1.027 91.011
ENSMUSG00000035057 4933434I20Rik protein_coding 1 79.853
1 85.671 1 84.896 1 88.505
ENSMUSG00000035062 Zc4h2 protein_coding 1.129 10.19 1.001 95.301
0.886 28.669 1.009 92.381
ENSMUSG00000035064 Eef2k protein_coding 0.979 77.916 0.952
79.475 1.07 58.414 1.057 96.183
ENSMUSG00000035067 Xkr6 protein_coding 0.916 69.24 1.125 82.904
0.972 52.658 1.047 93.156
ENSMUSG00000035069 Oma1 protein_coding 0.932 58.264 1.053
96.652 1.014 58.616 1.056 90.397
ENSMUSG00000035078 Mtmr9 protein_coding 0.98 55.303 1.044
83.464 0.977 78.293 1.032 90.463
ENSMUSG00000035085 1700020L24Rik protein_coding 0.7 1.422
0.923 81.731 1.541 5.864 1.013 95.877
ENSMUSG00000035086 Becn1 protein_coding 0.925 15.76 0.99 77.944
1.089 7.635 1.047 57.952
ENSMUSG00000035093 Secisbp2l protein_coding 0.95 16.652
1.008 95.431 1.04 52.8 1.047 74.896
ENSMUSG00000035095 Fam167a protein_coding 1.235 26.863
1.358 90.454 0.596 23.607 1.117 91.928
ENSMUSG00000035104 Eva1a protein_coding 1.192 34.861 0.966
85.116 0.87 80.089 0.93 95.405
ENSMUSG00000035105 Egln3 protein_coding 0.811 7.58 0.969 94.848
1.278 10.819 0.907 83.724
ENSMUSG00000035107 Dcbld2 protein_coding 0.942 63.283 1.1
88.092 0.962 71.004 1.193 48.119
ENSMUSG00000035109 Shc4 protein_coding 1.043 84.564 0.947
91.661 1.012 79.325 1.012 95.676
ENSMUSG00000035112 Wnk4 protein_coding 1.144 86.351 0.714 75.4

1.224 37.369 1.097 90.397
ENSMUSG00000035121 Neil2 protein_coding 1.138 24.958 0.887
71.41 0.991 81.62 0.832 84.29
ENSMUSG00000035125 Gcfc2 protein_coding 1.023 67.374 0.838
55.71 1.166 30.948 0.961 91.217
ENSMUSG00000035126 Wdr78 protein_coding 0.794 10.955 1.003
95.088 1.251 11.348 0.946 95.273
ENSMUSG00000035129 Gm6781 pseudogene 1 85.728 1
96.138 1 78.864 1 92.52
ENSMUSG00000035131 Brinp3 protein_coding 1.4 16.74 NA NA
0.703 16.643 0.953 88.505
ENSMUSG00000035133 Arhgap5 protein_coding 1.021 57.227
0.946 93.469 1.032 45.94 0.968 92.742
ENSMUSG00000035139 Secisbp2 protein_coding 0.927 18.907
0.976 75.493 1.105 7.635 1.119 40.024
ENSMUSG00000035142 Nubpl protein_coding 1.055 57.949 1.026
96.68 0.926 65.688 0.955 93.964
ENSMUSG00000035150 Eif2s3x protein_coding 1.046 22.349
0.965 78.51 0.994 69.583 0.991 96.287
ENSMUSG00000035151 Elmod2 protein_coding 1.012 85.499
0.996 92.404 0.995 74.325 0.873 83.724
ENSMUSG00000035152 Ap2b1 protein_coding 1.052 41.211 0.974
95.708 0.972 57.446 1.065 0
ENSMUSG00000035158 Mitf protein_coding 1.176 0.2 0.934 24.911
0.907 31.135 0.923 88.505
ENSMUSG00000035161 Ints6 protein_coding 1.153 0.577 1.054 82.809
0.822 0.72 0.948 84.19
ENSMUSG00000035164 Zc3h12c protein_coding 1.027 62.689
0.885 75.052 1.097 65.235 0.988 86.991
ENSMUSG00000035165 Kcne3 protein_coding 1.038 82.982 0.862
82.848 1.119 54.363 0.902 86.991
ENSMUSG00000035168 Tanc1 protein_coding 0.948 32.749 1.004
95.708 1.054 28.997 1.012 92.181
ENSMUSG00000035171 1110059E24Rik protein_coding 1.081 23.82
0.932 67.839 0.994 80.899 1.026 83.067
ENSMUSG00000035172 Plekhh3 protein_coding 1.037 81.657
1.285 71.222 0.752 5.042 0.937 86.991
ENSMUSG00000035173 A630007B06Rik protein_coding 0.992 63.126
0.995 96.216 1.014 63.801 1.031 83.067
ENSMUSG00000035177 Nlrp2 protein_coding 1 80.608 1
90.116 1 87.786 1 83.067
ENSMUSG00000035179 Ppp1r32 protein_coding 0.546 12.084
1.806 85.985 1.013 58.051 0.997 95.676
ENSMUSG00000035181 Heatr5a protein_coding 1.057 9.259 1.001
95.431 0.943 17.898 1.046 80.497
ENSMUSG00000035183 Slc24a5 protein_coding 0.442 30.877
0.847 96.216 2.67 38.26 1.057 92.12
ENSMUSG00000035184 Fam124a protein_coding 1.209 14.008
0.898 55.71 0.923 86.144 0.956 93.734
ENSMUSG00000035186 Ubd protein_coding 1.07 50.111 1.017
93.899 0.921 48.851 0.998 96.042
ENSMUSG00000035189 Ano4 protein_coding 0.853 78.224 1.175
83.104 0.992 67.029 1.181 91.807
ENSMUSG00000035191 Rfpl4 protein_coding 1 83.169 1

96.001 1 82.161 1 96.358
ENSMUSG00000035198 Tubg1 protein_coding 0.888 24.817 1.119
78.494 1.007 86.084 1.127 69.918
ENSMUSG00000035199 Arl6ip5 protein_coding 1.054 35.787
0.995 93.817 0.952 39.755 1.029 91.492
ENSMUSG00000035200 Chrnb4 protein_coding 1.028 72.85 0.928
90.712 NA NA 0.841 86.991
ENSMUSG00000035201 B020004J07Rik protein_coding 1 86.464
1 95.708 1 81.492 1 94.752
ENSMUSG00000035202 Lars2 protein_coding 1 80.608 0.899
67.739 1.116 20.556 1.146 0
ENSMUSG00000035203 Epn1 protein_coding 0.871 2.009 1.057 83.104
1.089 15.241 1.036 83.067
ENSMUSG00000035206 Sppl2b protein_coding 0.928 10.514
1.059 72.642 1.017 68.129 1.007 95.74
ENSMUSG00000035208 Slfn8 protein_coding 1.256 7.959 1.069 96.843
0.744 25.052 1.345 48.119
ENSMUSG00000035211 Xrra1 protein_coding 0.753 44.562 0.861
95.708 1.546 46.206 0.856 85.493
ENSMUSG00000035212 Leprot protein_coding 1.124 0.461 0.993
68.818 0.895 10.362 1.006 94.904
ENSMUSG00000035215 Lsm7 protein_coding 0.985 59.674 0.97
95.708 1.049 64.624 1.02 92.018
ENSMUSG00000035226 Rims4 protein_coding 0.869 35.894 1.044
96.652 1.1 42.522 0.903 85.493
ENSMUSG00000035227 Spcs2 protein_coding 0.996 72.655 1.007
90.092 0.997 49.967 1.05 72.616
ENSMUSG00000035228 Ccdc106 protein_coding 0.874 49.047 0.97
94.262 1.18 36.578 1.036 96.183
ENSMUSG00000035232 Pdk3 protein_coding 1.03 64.714 0.953
90.378 1.02 84.071 1.085 69.404
ENSMUSG00000035234 Fam175a protein_coding 1.043 57.38 0.948
89.277 1.012 87.274 1.004 93.016
ENSMUSG00000035235 Trim13 protein_coding 1.039 84.263 1.02
96.407 0.944 60.614 0.953 92.31
ENSMUSG00000035236 Scai protein_coding 0.906 32.749 0.95
86.538 1.164 25.93 0.928 90.157
ENSMUSG00000035237 Lcat protein_coding 0.913 45.894 1.118
92.062 0.975 79.524 1.108 82.85
ENSMUSG00000035239 Neu3 protein_coding 0.93 64.014 0.979
91.361 1.099 34.285 0.988 92.762
ENSMUSG00000035242 Oaz1 protein_coding 0.867 1.727 1.066 82.904
1.078 15.602 1.007 90.674
ENSMUSG00000035245 Eogt protein_coding 0.967 31.11 1.019 92.931
1.018 57.907 1.019 91.872
ENSMUSG00000035246 Pcyt1b protein_coding 1.354 18.388
0.971 93.094 0.762 14.262 0.836 86.991
ENSMUSG00000035247 Hectd1 protein_coding 0.973 65.199
0.954 72.576 1.079 26.978 0.956 91.291
ENSMUSG00000035248 Zcchc6 protein_coding 1.073 1.689 1.001
95.504 0.931 4.935 1.006 96.183
ENSMUSG00000035258 Abi3bp protein_coding 1.221 18.314
1.028 94.96 0.797 19.765 1.001 92.181
ENSMUSG00000035262 Amh protein_coding 1.038 77.916 1.212

96.335 0.792 66.425 1.028 94.82
ENSMUSG00000035266 Helq protein_coding 0.969 78.909 0.924
72.399 1.113 33.787 1.077 76.347
ENSMUSG00000035268 Pkig protein_coding 0.997 81.465 0.953
81.134 1.049 36.839 1.016 93.132
ENSMUSG00000035270 Impg2 protein_coding 0.805 30.136 1.122
93.529 1.109 79.119 0.967 95.74
ENSMUSG00000035273 Hpse protein_coding 1.448 0 0.871 86.277
0.792 7.329 1.156 82.66
ENSMUSG00000035274 Tpbg protein_coding 1.298 11.638 0.992
97.263 0.774 11.504 1.132 31.133
ENSMUSG00000035275 Raver2 protein_coding 0.878 20.369
1.086 82.904 1.05 73.759 1.013 91.525
ENSMUSG00000035278 Plekhj1 protein_coding 0.929 21.025
1.083 87.604 0.995 76.498 1.046 91.525
ENSMUSG00000035279 Ssc5d protein_coding 1.178 6.23 0.925 82.848
0.918 52.658 1.283 31.133
ENSMUSG00000035283 Adrb1 protein_coding 1.102 69.408 1.116
85.501 0.811 11.504 0.732 83.724
ENSMUSG00000035284 Vps13c protein_coding 0.87 5.686 1.039
89.494 1.102 30.107 0.989 93.264
ENSMUSG00000035285 Nat14 protein_coding 1.008 81.76 1.106 95.735
0.893 77.704 0.833 83.724
ENSMUSG00000035293 G2e3 protein_coding 1.015 71.142 0.883
80.052 1.118 38.26 0.987 95.877
ENSMUSG00000035295 Wdr38 protein_coding 0.846 12.436 0.962
94.597 1.227 14.169 0.878 83.724
ENSMUSG00000035296 Sgcg protein_coding 5.469 7.959 1.199 92.253
0.153 1.061 0.963 93.132
ENSMUSG00000035297 Cops4 protein_coding 1.106 1.626 0.979 74.633
0.924 7.339 1.024 83.067
ENSMUSG00000035298 Klhl35 protein_coding 1.135 54.344
1.009 83.104 0.874 29.217 1.042 88.053
ENSMUSG00000035299 Mid1 protein_coding 0.891 14.645 1.009
97.263 1.113 24.07 0.894 84.19
ENSMUSG00000035305 Ror1 protein_coding 1.162 60.435 0.943
94.48 0.914 73.598 1.024 96.183
ENSMUSG00000035310 Lin54 protein_coding 1.073 21.703 1.109
71.617 0.839 0.879 1.004 95.74
ENSMUSG00000035311 Gnptab protein_coding 0.875 0.987 1.055
62.898 1.08 43.185 0.979 91.011
ENSMUSG00000035314 Gdpd5 protein_coding 1.02 81.125 0.973
91.361 1.01 87.103 1.051 96.086
ENSMUSG00000035325 Sec31a protein_coding 0.998 86.57 1.068
72.642 0.941 46.32 1.042 62.594
ENSMUSG00000035329 Fbxo33 protein_coding 1.181 1.079 1.027
83.819 0.825 0.945 0.988 95.593
ENSMUSG00000035337 Uchl4 protein_coding 1 79.93 NA NA 1
73.309 1 94.99
ENSMUSG00000035342 Lzts2 protein_coding 1.02 85.728 1.007
92.062 0.975 75.637 1.03 89.108
ENSMUSG00000035349 Mia2 protein_coding 0.808 52.018 0.644
58.277 1.928 15.049 0.844 91.372
ENSMUSG00000035351 Nup37 protein_coding 0.98 81.125 0.976

95.301 1.042 84.724 1.137 83.067
ENSMUSG00000035352 Ccl12 protein_coding 0.79 70.862 1.014
96.68 NA NA 3.017 48.857
ENSMUSG00000035354 Uvrag protein_coding 1.051 7.397 0.961 69.175
0.991 86.727 1.011 91.011
ENSMUSG00000035355 Kcnh4 protein_coding 0.394 1.422 NA NA
2.452 6.916 1.143 93.627
ENSMUSG00000035356 Nfkbiz protein_coding 0.956 75.298
0.947 79.475 1.102 31.975 1.017 79.268
ENSMUSG00000035357 Pdzrn3 protein_coding 1.139 2.651 0.965
90.744 0.907 30.468 1.095 73.823
ENSMUSG00000035364 4930524J08Rik protein_coding 0.949 31.614
1.17 86.387 0.901 88.657 1.015 95.273
ENSMUSG00000035365 Parpbp protein_coding 1.056 82.705
0.978 95.211 NA NA 1.156 75.198
ENSMUSG00000035367 Rmi1 protein_coding 1.103 1.323 1.023 97.263
0.888 4.759 0.976 92.181
ENSMUSG00000035370 Adat3 protein_coding 0.997 80.146 1.204
71.617 0.833 21.666 0.975 95.72
ENSMUSG00000035371 Ssx9 protein_coding 1 77.916 1
96.984 1 69.583 1 95.72
ENSMUSG00000035372 1810055G02Rik protein_coding 0.967 85.59 1.16
63.504 0.892 11.314 1.292 56.863
ENSMUSG00000035373 Ccl7 protein_coding 1.011 66.272 1.306
83.104 0.759 42.997 1.951 60.31
ENSMUSG00000035376 Ptplb protein_coding 0.724 5.544 0.936 96.268
1.478 14.131 0.96 91.928
ENSMUSG00000035378 Shq1 protein_coding 1.061 69.489 0.917
61.883 1.03 76.754 1.018 92.018
ENSMUSG00000035382 Pcsk7 protein_coding 0.946 8.181 1.008 96.013
1.045 34.807 1.06 50.452
ENSMUSG00000035385 Ccl2 protein_coding 1.199 45.61 0.791 87.147
1.053 88.657 1.24 79.268
ENSMUSG00000035387 4930415L06Rik protein_coding 1 81.196
1 96.897 1 75.155 1 95.72
ENSMUSG00000035390 Brsk1 protein_coding 0.937 55.303 1.075
83.104 0.989 75.967 1.015 86.991
ENSMUSG00000035392 Dennd1a protein_coding 1.018 79.277
0.969 87.313 1.014 87.519 1.002 93.457
ENSMUSG00000035394 Ccdc11 protein_coding 0.877 61.633
0.971 75.345 1.177 24.383 0.993 91.783
ENSMUSG00000035395 Pet2 protein_coding 1 85.946 1
95.088 1 87.686 1 96.183
ENSMUSG00000035397 Klf16 protein_coding 1.042 85.391 1.045
85.756 0.919 16.949 0.929 86.991
ENSMUSG00000035399 Oser1 protein_coding 1.07 8.477 1.003 83.392
0.932 35.559 0.997 92.181
ENSMUSG00000035401 2210018M11Rik protein_coding 1.078 4.628 NA
NA 0.922 25.625 0.963 86.991
ENSMUSG00000035407 Kank4 protein_coding 1.026 84.865 1.157
83.104 0.841 23.1 0.922 83.724
ENSMUSG00000035413 Tmem98 protein_coding 0.924 14.645
1.023 86.277 1.056 54.673 1.014 94.99
ENSMUSG00000035420 Fam170a protein_coding 1 80.51 1

95.088 1 69.728 1 96.183
ENSMUSG00000035427 Mageb4 protein_coding 1 84.661 NA
NA 1 84.308 1 95.273
ENSMUSG00000035431 Sstr1 protein_coding 1.678 78.538 0.895
92.561 0.667 53.224 1.681 83.067
ENSMUSG00000035437 Rabgap1 protein_coding 1.011 64.714
0.952 85.418 1.038 78.498 0.959 85.493
ENSMUSG00000035439 Haus8 protein_coding 1.108 36.124 0.932
92.137 0.964 47.12 1.072 81.901
ENSMUSG00000035441 Myo1d protein_coding 0.961 44.922 1.074
79.864 0.969 74.179 0.941 92.181
ENSMUSG00000035443 Thyn1 protein_coding 0.857 17.839 1.11
82.904 1.055 62.648 1.243 31.133
ENSMUSG00000035448 Ccr3 protein_coding 1.282 74.147 0.986
91.361 0.79 84.626 0.934 94.654
ENSMUSG00000035451 Foxa1 protein_coding 0.664 1.343 1.098 83.104
1.367 16.446 0.703 90.95
ENSMUSG00000035454 Samt3 protein_coding 1 83.523 1
96.138 1 88.729 1 96.183
ENSMUSG00000035455 Fignl1 protein_coding 1.741 28.098
0.984 91.643 0.582 29.451 1.289 50.74
ENSMUSG00000035456 Prdm8 protein_coding 0.69 17.401 0.88
87.452 1.645 18.797 0.861 90.289
ENSMUSG00000035458 Tnni3 protein_coding 1.297 34.861 0.897
93.654 0.859 46.071 0.985 93.964
ENSMUSG00000035459 Stab2 protein_coding 1.452 32.058 0.974
96.843 0.706 26.057 0.958 94.654
ENSMUSG00000035469 Rcbtb1 protein_coding 1.056 28.693
0.972 91.661 0.977 52.213 0.959 85.493
ENSMUSG00000035472 Slc25a21 protein_coding 0.605 9.081 1.403
87.029 1.174 43.586 1.063 92.748
ENSMUSG00000035473 Galm protein_coding 1.038 50.111 1.056
73.092 0.913 6.858 0.985 96.086
ENSMUSG00000035476 Tab3 protein_coding 1.104 23.82 0.929 66.651
0.972 85.464 0.898 83.724
ENSMUSG00000035478 Mbd3 protein_coding 0.858 2.436 1.083 71.222
1.073 65.235 0.972 96.287
ENSMUSG00000035486 Plk5 protein_coding 1.032 76.686 1.027
92.111 0.944 85.792 0.943 93.132
ENSMUSG00000035491 1600014K23Rik protein_coding 1 78.744
1 94.48 1 58.414 1 95.273
ENSMUSG00000035493 Tgfbi protein_coding 1.34 0.461 0.88 61.883
0.844 25.052 1.025 88.111
ENSMUSG00000035495 Tstd2 protein_coding 1.082 0.2 NA NA
0.945 11.751 0.978 85.493
ENSMUSG00000035498 Cdcp1 protein_coding 1.089 55.124 0.936
82.073 0.979 86.112 0.971 96.183
ENSMUSG00000035504 Reep6 protein_coding 0.867 4.115 1.117 83.104
1.033 47.776 0.824 83.724
ENSMUSG00000035505 Cox18 protein_coding 0.975 49.047 1.045
90.092 0.981 87.173 1.008 94.904
ENSMUSG00000035506 Slc12a8 protein_coding 1.048 78.744
0.975 79.475 0.981 37.741 0.895 91.291
ENSMUSG00000035509 Fbxl21 protein_coding 1.097 57.655

1.106 83.104 0.826 13.848 0.842 83.724
ENSMUSG00000035513 Ntng2 protein_coding 1.111 28.395 0.996
96.68 0.901 12.571 1.028 93.734
ENSMUSG00000035517 Tdrd7 protein_coding 1.02 86.474 NA NA
0.998 78.954 1.105 75.198
ENSMUSG00000035521 Gnptg protein_coding 0.976 74.8 1.07 83.104
0.958 59.726 0.959 87.735
ENSMUSG00000035522 Tsga8 protein_coding 1 84.661 1
93.046 1 86.084 1 93.413
ENSMUSG00000035523 Vmn1r172 protein_coding 1 81.125 1
97.263 NA NA 1 95.72
ENSMUSG00000035528 Npffr2 protein_coding 1 77.113 1
95.708 1 80.163 1 93.685
ENSMUSG00000035529 Prdm4 protein_coding 1.024 64.956 1.039
85.756 0.943 35.264 1.032 85.629
ENSMUSG00000035530 Eif1 protein_coding 1.101 0.577 0.973 87.041
0.929 1.061 1.02 76.046
ENSMUSG00000035539 E230008N13Rik protein_coding 0.806 84.865
0.863 94.48 NA NA 1.554 93.156
ENSMUSG00000035540 Gc protein_coding 1 84.168 NA NA
1 83.857 1 93.964
ENSMUSG00000035545 Leng8 protein_coding 0.873 2.009 1.064 83.104
1.074 21.873 0.962 86.445
ENSMUSG00000035547 Capn5 protein_coding 1.078 16.436 0.841
60.678 1.104 84.504 0.873 86.699
ENSMUSG00000035557 Krt17 protein_coding 0.689 8.302 1.386 71.617
1.046 66.043 1.048 91.928
ENSMUSG00000035559 Mpv17l2 protein_coding 0.777 0.987 1.135
71.98 1.136 25.564 1.062 90.397
ENSMUSG00000035560 Wdr20b protein_coding 1 80.751 1
96.268 1 87.274 1 92.748
ENSMUSG00000035561 Aldh1b1 protein_coding 1.148 63.804
0.674 38.699 1.29 36.95 1.117 89.152
ENSMUSG00000035566 Pcdh17 protein_coding 0.944 58.802
1.048 92.931 NA NA 0.79 83.724
ENSMUSG00000035569 Ankrd11 protein_coding 0.948 33.599
1.018 96.847 1.039 44.361 0.981 85.493
ENSMUSG00000035572 Dcaf10 protein_coding 0.983 51.56 0.996
84.999 1.018 88.349 0.945 83.724
ENSMUSG00000035575 Utp6 protein_coding 1.061 44.334 1.013
96.378 0.929 48.387 1.058 31.133
ENSMUSG00000035576 L3mbtl1 protein_coding 1.394 66.76 0.551
72.576 1.298 51.851 0.784 83.724
ENSMUSG00000035578 Iqcg protein_coding 1.055 68.485 0.992
95.735 0.957 70.791 1.012 93.722
ENSMUSG00000035580 Kcnh8 protein_coding 0.647 61.043 0.825
96.68 1.866 43.185 1.618 86.445
ENSMUSG00000035582 Gdpd4 protein_coding 1 84.324 1
95.708 1 84.724 1 96.139
ENSMUSG00000035585 Tsen34 protein_coding 0.919 15.06 1.06
79.29 1.022 64.624 1.008 90.455
ENSMUSG00000035594 Chrna5 protein_coding 2.054 2.569 1.005
95.504 0.484 6.858 1.452 61.984
ENSMUSG00000035595 1600002K03Rik protein_coding 0.809 7.118

1.181 74.663 1.05 85.352 1.096 78.379
ENSMUSG00000035596 Mboat7 protein_coding 0.975 67.477
1.087 71.617 0.945 27.194 0.987 83.724
ENSMUSG00000035597 Prpf39 protein_coding 1.064 7.65 0.972
96.138 0.968 28.489 0.973 84.29
ENSMUSG00000035601 Trmt10b protein_coding 1.073 50.111
0.957 87.998 0.976 85.21 0.927 90.805
ENSMUSG00000035606 Ky protein_coding 5.648 2.723 2.115 82.904
0.084 0.72 0.769 94.608
ENSMUSG00000035614 Fam179b protein_coding 1.032 59.806
0.967 90.053 1.003 86.258 0.947 85.493
ENSMUSG00000035615 Frmpd1 protein_coding 0.979 70.597
1.114 92.111 0.918 75.967 0.707 83.724
ENSMUSG00000035620 Ric8b protein_coding 1.023 20.032 1.178
63.504 0.828 0.945 0.996 93.685
ENSMUSG00000035621 Midn protein_coding 0.844 7.43 1.155 83.104
1.028 35.894 1.006 95.74
ENSMUSG00000035623 Rsf1 protein_coding 0.992 84.263 0.929
62.025 1.082 12.831 0.952 86.445
ENSMUSG00000035626 Olfr1509 protein_coding 1 81.178 1
96.847 1 86.985 1 93.261
ENSMUSG00000035629 1700021K19Rik protein_coding 1.075 7.049
1.049 82.904 0.888 6.68 0.939 83.724
ENSMUSG00000035632 Cnot3 protein_coding 0.919 5.686 1.102 60.309
0.99 68.266 1 94.904
ENSMUSG00000035637 Grhpr protein_coding 1.026 86.74 0.899 87.452
1.081 54.363 1.018 94.904
ENSMUSG00000035638 Muc20 protein_coding 0.794 22.237 1.242
60.309 1.018 66.425 0.86 83.724
ENSMUSG00000035640 Dos protein_coding 0.669 1.031 1.136 79.367
1.317 10.819 0.885 84.29
ENSMUSG00000035642 Aamdc protein_coding 0.786 0.987 1.005 96.948
1.259 13.356 1.077 60.466
ENSMUSG00000035649 Zcchc7 protein_coding 0.938 48.354
0.931 79.261 1.146 30.645 0.962 84.29
ENSMUSG00000035651 4930480E11Rik protein_coding 1 82.231
NA NA 1 86.985 1 95.559
ENSMUSG00000035653 Lrfn5 protein_coding 1.15 69.408 1.316
88.533 0.659 37.906 0.358 87.705
ENSMUSG00000035666 Gtf3c4 protein_coding 1.011 81.52 1.036
87.609 0.955 49.453 1.01 96.287
ENSMUSG00000035671 Zswim4 protein_coding 0.806 1.239 1.047
90.29 1.182 12.467 0.951 90.805
ENSMUSG00000035673 Sbno2 protein_coding 0.844 3.07 1.01 96.216
1.174 7.365 0.936 94.99
ENSMUSG00000035674 Ndufa3 protein_coding 0.799 2.009 1.071
71.98 1.166 43.481 1.073 72.369
ENSMUSG00000035678 Tnfsf9 protein_coding 1.151 65.733 NA
NA 0.801 66.201 1.034 78.948
ENSMUSG00000035681 Kcnc2 protein_coding 1.124 55.124 0.851
83.392 1.047 80.899 0.998 91.625
ENSMUSG00000035683 Melk protein_coding 1.567 25.735 0.83
60.678 0.769 81.62 1.295 72.616
ENSMUSG00000035686 Thrsp protein_coding 0.506 0.987 0.964 91.661

2.052 5.864 1.027 95.74
ENSMUSG00000035692 Isg15 protein_coding 1.184 30.282 1.14
83.104 0.742 20.511 1.92 56.769
ENSMUSG00000035694 Caps2 protein_coding 2.448 85.832 0.22
70.29 1.861 39.925 1.461 93.156
ENSMUSG00000035696 Rnf38 protein_coding 0.994 71.142 1.006
86.861 1.001 45.94 0.884 83.724
ENSMUSG00000035697 Hmha1 protein_coding 1.264 18.985 1.002
85.921 0.788 83.811 0.918 92.31
ENSMUSG00000035699 Slc51a protein_coding 1 85.499 1
96.216 1 65.902 1 92.851
ENSMUSG00000035704 Alg8 protein_coding 1.105 53.228 0.924
79.807 NA NA 1.062 79.268
ENSMUSG00000035711 Dok3 protein_coding 1.156 19.893 1.076
93.899 0.805 20.258 0.978 83.276
ENSMUSG00000035713 Usp35 protein_coding 0.816 27.664 0.842 0
1.452 5.864 1.055 91.372
ENSMUSG00000035722 Abca7 protein_coding 0.944 70.213 1.142
71.98 0.927 24.309 1.022 86.118
ENSMUSG00000035725 Prkx protein_coding 1.114 4.362 0.996 96.948
0.897 6.524 0.981 95.273
ENSMUSG00000035726 Supt16 protein_coding 1.061 15.178
0.938 52.266 1.004 87.744 1.012 95.273
ENSMUSG00000035735 Dagla protein_coding 0.789 3.641 1.111 97.155
1.143 15.049 0.983 95.273
ENSMUSG00000035745 Grin3b protein_coding 0.75 37.82 1.143
95.088 1.168 60.789 1.033 93.261
ENSMUSG00000035754 Wdr18 protein_coding 0.913 14.221 1.064
86.793 1.025 61.171 1.023 95.676
ENSMUSG00000035757 Selo protein_coding 0.898 3.231 1.057 87.029
1.053 38.26 0.994 95.676
ENSMUSG00000035759 Bbs10 protein_coding 0.879 22.142 1.192
82.904 0.954 78.498 0.966 94.001
ENSMUSG00000035762 Tmem161b protein_coding 1.052 24.253
1.003 94.597 0.951 52.213 1.045 92.748
ENSMUSG00000035764 Fbxo45 protein_coding 0.968 33.415
1.102 72.642 0.938 66.201 0.924 85.493
ENSMUSG00000035765 Dym protein_coding 1.091 22.349 0.978
95.088 0.937 29.451 0.988 93.045
ENSMUSG00000035769 Xylb protein_coding 1.008 84.294 0.936
89.996 1.063 71.839 0.958 96.183
ENSMUSG00000035770 Dync1li2 protein_coding 1.026 35.024
0.974 87.553 1.003 85.134 0.971 83.724
ENSMUSG00000035772 Mrps2 protein_coding 1.066 35.748 0.992
97.155 0.942 22.279 1.023 94.904
ENSMUSG00000035773 Kiss1r protein_coding 1.335 12.072
0.793 64.123 0.945 77.704 0.862 86.991
ENSMUSG00000035775 Krt20 protein_coding 1.198 52.799 1.12
95.708 0.744 70.214 1.014 96.042
ENSMUSG00000035776 Cd99l2 protein_coding 1.044 35.371
1.009 96.013 0.95 42.895 0.992 87.331
ENSMUSG00000035778 Ggta1 protein_coding 1.041 42.555 0.979
90.744 0.984 84.552 0.981 84.19
ENSMUSG00000035780 Ugt2a3 protein_coding 1 71.06 1

93.707	1	87.519	1	95.74
ENSMUSG00000035781	R3hdm4	protein_coding	1.039	82.352	1.025	82.904	0.938	25.625	1.006	80.324
ENSMUSG00000035783	Acta2	protein_coding	1.003	72.27	1.135	60.309	0.875	4.935	1.016	96.139
ENSMUSG00000035785	Cmtm2b	protein_coding	1	65.106	NA	NA	1	88.274	1	91.783
ENSMUSG00000035790	Cep19	protein_coding	1.089	32.676	1.049	90.116	0.878	27.321	1.022	95.74
ENSMUSG00000035798	Zdhhc17	protein_coding	1.002	85.59	1.044	97.097	0.954	73.874	0.971	86.991
ENSMUSG00000035799	Twist1	protein_coding	1.079	14.59	1.068	87.185	0.866	6.858	1.013	95.74
ENSMUSG00000035804	Ins1	protein_coding	1	86.145	1	97.263	1	86.602	1	94.825
ENSMUSG00000035811	Ugt2b35	protein_coding	1	79.577	1	95.211	NA	NA	1	92.561
ENSMUSG00000035818	Plekhs1	protein_coding	0.622	30.352	0.918	85.075	1.745	10.281	1.366	48.177
ENSMUSG00000035824	Tk2	protein_coding	1.073	8.365	0.839	21.916	1.109	53.587	0.961	84.29
ENSMUSG00000035828	Pim3	protein_coding	0.915	49.047	0.881	56.027	1.239	21.522	1.001	84.29
ENSMUSG00000035829	Ppp1r26	protein_coding	0.99	61.887	0.904	93.654	1.116	48.451	0.817	83.952
ENSMUSG00000035831	Krt25	protein_coding	1	78.639	1	86.277	1	83.275	1	90.628
ENSMUSG00000035834	Polr3g	protein_coding	0.902	40.568	1.124	73.092	0.987	86.552	1.058	76.697
ENSMUSG00000035835	BC005764	protein_coding	1.47	0	1.103	88.136	0.618	0.72	1.178	83.067
ENSMUSG00000035836	Ugt2b1	protein_coding	2.998	33.854	0.359	91.661	0.93	76.64	1.134	95.273
ENSMUSG00000035840	Lysmd3	protein_coding	0.964	86.145	1.106	71.617	0.933	7.329	1.056	89.72
ENSMUSG00000035842	Ddx11	protein_coding	0.848	41.33	0.986	92.137	1.198	37.764	1.089	93.457
ENSMUSG00000035845	Alg12	protein_coding	0.991	75.858	0.978	91.643	NA	NA	1.045	83.067
ENSMUSG00000035847	Ids	protein_coding	1.082	24.958	0.99	95.735	0.933	18.122	0.945	92.12
ENSMUSG00000035849	Krt222	protein_coding	0.985	65.388	1.033	83.464	0.982	79.524	1.01	94.654
ENSMUSG00000035851	Ythdc1	protein_coding	1.097	1.441	0.947	90.378	0.962	21.431	0.96	83.724
ENSMUSG00000035852	Misp	protein_coding	0.877	12.785	0.942	91.361	1.206	15.241	0.865	94.001
ENSMUSG00000035860	Cdhr3	protein_coding	0.459	13.239	1.874	71.617	1.164	79.897	0.709	93.584
ENSMUSG00000035861	Tmprss11bnl	protein_coding	1	75.858	1	95.088	1	78.713	1	94.904
ENSMUSG00000035863	Palm	protein_coding	0.922	24	0.998	95.301	1.084	43.185	1.035	86.118
ENSMUSG00000035868	3110052M02Rik	protein_coding	1.217	16.124

0.969 85.242 0.845 37.906 0.925 85.493
ENSMUSG00000035873 Pawr protein_coding 1.041 52.986 NA NA
0.904 58.775 0.978 89.892
ENSMUSG00000035875 AI182371 protein_coding 0.656 6.952 1.015
94.48 1.497 10.615 0.9 90.854
ENSMUSG00000035877 Zhx3 protein_coding 1.067 40.333 0.949
81.731 0.992 71.999 1.063 96.184
ENSMUSG00000035878 Hykk protein_coding 1.377 0.2 1.019 96.308
0.714 0.72 0.941 85.493
ENSMUSG00000035885 Cox8a protein_coding 0.762 2.524 1.105 75.612
1.189 16.808 0.92 86.699
ENSMUSG00000035890 Rnf126 protein_coding 0.941 17.705
1.057 80.14 1.006 86.666 0.952 95.35
ENSMUSG00000035891 Cerk protein_coding 1.277 0 0.911 56.781
0.861 17.381 0.971 93.584
ENSMUSG00000035896 Rnase1 protein_coding 4.945 14.34 0.249
73.766 0.81 80.015 1.441 80.573
ENSMUSG00000035898 Uba6 protein_coding 1.036 44.061 0.981
91.955 0.983 83.158 0.979 94.654
ENSMUSG00000035900 Gramd4 protein_coding 0.976 83.09 1.073
90.092 0.954 54.964 0.996 92.12
ENSMUSG00000035901 Dennd5a protein_coding 1.112 3.713 0.993
95.301 0.905 7.509 0.989 96.183
ENSMUSG00000035910 Dcdc2a protein_coding 0.936 85.728
0.526 40.463 2.029 28.997 2.597 46.412
ENSMUSG00000035914 Cd276 protein_coding 0.983 86.74 NA NA
1.061 83.397 1.167 77.621
ENSMUSG00000035919 Bbs9 protein_coding 0.893 22.142 0.851
61.883 1.318 5.864 0.886 83.724
ENSMUSG00000035929 H2-Q4 protein_coding 1.127 52.429 1.03
90.116 0.86 11.751 0.92 83.724
ENSMUSG00000035932 Olfr750 protein_coding 1 85.59 NA NA
1 82.778 1 96.042
ENSMUSG00000035933 Cog5 protein_coding 0.99 86.658 1.019
92.931 0.99 80.163 0.956 93.264
ENSMUSG00000035934 Pknox2 protein_coding 1.345 11.638
1.228 71.617 0.604 2.333 1.29 62.594
ENSMUSG00000035936 Aldh5a1 protein_coding 1.016 72.27 1.024
97.155 0.958 59.383 0.882 83.724
ENSMUSG00000035941 Ibtk protein_coding 1.017 81.125 0.923
86.604 1.066 59.612 1.028 81.061
ENSMUSG00000035944 Ttc38 protein_coding 0.999 74.745 0.912
88.881 1.093 84.626 0.985 93.316
ENSMUSG00000035946 Gsx2 protein_coding 1 81.823 1
96.138 1 79.776 1 95.74
ENSMUSG00000035948 Acss3 protein_coding 0.703 2.5 0.937 89.509
1.511 5.864 0.93 86.991
ENSMUSG00000035949 Fbxw2 protein_coding 0.934 60.003 0.943
68.848 1.135 11.348 0.995 91.078
ENSMUSG00000035951 Ascl3 protein_coding 0.405 15.486 1.692
83.104 1.461 61.841 1.61 95.877
ENSMUSG00000035953 Tmem55b protein_coding 1.02 66.58 1.068
90.116 0.916 24.163 0.916 83.724
ENSMUSG00000035954 Dock4 protein_coding 1.082 59.806 0.96

97.263	0.964 42.997	0.954 93.261
ENSMUSG00000035958	Tdp2	protein_coding	0.93	17.898	0.898
41.951	1.194 5.964 1.029 94.016
ENSMUSG00000035960	Apex1 protein_coding	0.935 38.983	1.041
92.111	1.029 56.931	1.07	68.696
ENSMUSG00000035964	NA	NA	NA	NA	NA	NA	1.171 88.693
0.932 95.273
ENSMUSG00000035967	Ddx26b	protein_coding	1.039 75.671
0.863 83.321	1.108 65.8	0.949 86.991
ENSMUSG00000035969	Rusc2 protein_coding	0.95	44.562	0.935
68.818	1.127 16.808	0.979 94.904
ENSMUSG00000035983	Gm7008	protein_coding	0.629 7.004 1.031
92.404	1.539 7.365 0.782 84.19
ENSMUSG00000035984	Nme5	protein_coding	1.138 81.52 1.211 83.104
0.727 53.7	0.926 93.584
ENSMUSG00000035992	Fnip1 protein_coding	1.081 7.397 0.981 85.418
0.939 37.617	0.961 83.952
ENSMUSG00000036002	Fam214b	protein_coding	0.891 2.009 1.03
89.494	1.092 28.979	0.97	85.493
ENSMUSG00000036006	Fam65b	protein_coding	1.092 25.944
1.078 82.904	0.851 2.722 0.966 96.183
ENSMUSG00000036009	Mettl25	protein_coding	1.195 13.8	0.927
73.461	0.903 76.768	0.874 83.724
ENSMUSG00000036013	Fam122c	protein_coding	1	85.129	1
96.138	1	83.275	1	96.042
ENSMUSG00000036019	Tmtc2 protein_coding	1.04	32.411	1.055
92.561	0.908 26.369	0.987 86.991
ENSMUSG00000036022	Fam122b	protein_coding	1.104 24.147	0.98
96.268	0.921 44.211	0.893 83.952
ENSMUSG00000036023	Parp2 protein_coding	1.05	20.799	0.924
81.02 NA	NA	1.065 60.968
ENSMUSG00000036026	Tmem63b	protein_coding	0.949 47.266
1.074 87.609	0.985 84.504	0.981 92.12
ENSMUSG00000036030	Prtg	protein_coding	1.092 20.032	0.867
70.29 1.06	76.225	1.132 83.067
ENSMUSG00000036036	Zfp57 protein_coding	0.828 17.839	1.02
90.053	1.181 13.259	0.985 93.734
ENSMUSG00000036040	Adamtsl2	protein_coding	1.404 56.66 0.777
67.165	0.916 84.896	1.23	76.697
ENSMUSG00000036045	Gm12221	pseudogene	1	86.918	1
96.013	1	87.744	1	93.457
ENSMUSG00000036046	5031439G07Rik	protein_coding	1.111 0.2
1.047 83.819	0.861 1.342 1.053 61.984
ENSMUSG00000036052	Dnajb5	protein_coding	0.989 57.818
1.042 82.904	0.97	50.523	1.152 51.901
ENSMUSG00000036053	Fmnl2 protein_coding	1.006 58.38 NA	NA
0.975 72.464	1.113 63.708
ENSMUSG00000036054	Sugp2 protein_coding	0.907 4.115 1.071 71.222
1.026 63.665	1.092 31.133
ENSMUSG00000036057	Ptpn23	protein_coding	0.98	57.227
1.013 83.464	1.001 87.786	1.002 96.183
ENSMUSG00000036061	Smug1 protein_coding	1.051 41.454	1.011
95.809	0.943 27.321	1	96.248
ENSMUSG00000036062	N28178	protein_coding	1.584 0.2	0.812

90.378 0.774 3.123 1.105 44.882
ENSMUSG00000036067 Slc2a6 protein_coding 1.479 13.105
0.781 73.461 0.869 76.768 1.138 95.877
ENSMUSG00000036073 Galt protein_coding 0.935 65.199 1.09
82.809 0.983 83.811 0.991 88.505
ENSMUSG00000036078 Sigmar1 protein_coding 0.914 10.704
1.081 72.642 1.008 88.274 0.988 91.65
ENSMUSG00000036086 Zranb3 protein_coding 1.174 23.019
0.957 86.604 0.892 46.575 0.932 90.805
ENSMUSG00000036087 Slain2 protein_coding 1.085 6.683 1.019
82.809 0.907 1.898 0.981 95.877
ENSMUSG00000036091 Hyal3 protein_coding 0.819 50.519 NA NA
1.119 54.809 0.989 95.92
ENSMUSG00000036093 Arl5a protein_coding 1.006 83.726 0.971
95.941 1.026 75.155 0.978 95.832
ENSMUSG00000036095 Dgkb protein_coding 1.583 0.664 0.736 21.315
0.857 45.093 0.983 88.552
ENSMUSG00000036097 Fam178a protein_coding 0.972 36.259
1.022 87.185 1.008 69.196 0.998 91.078
ENSMUSG00000036098 Myrf protein_coding 0.843 84.452 1.057
96.216 1.122 86.258 1.125 93.734
ENSMUSG00000036099 Vezt protein_coding 1.044 33.555 1.016
95.504 0.942 56.822 1.013 86.209
ENSMUSG00000036103 Colec12 protein_coding 1.051 31.477
0.977 84.594 0.972 67.029 1.132 91.525
ENSMUSG00000036104 Rab3gap1 protein_coding 1.04 7.501 0.956
35.072 1.007 86.258 1.027 93.457
ENSMUSG00000036106 Prr5 protein_coding 0.974 50.229 1.001
96.68 1.025 51.977 0.959 95.559
ENSMUSG00000036109 Mbnl3 protein_coding 0.67 11.924 1.087
97.288 1.375 21.873 1.007 95.72
ENSMUSG00000036111 Lmo1 protein_coding 1.086 74.147 0.919
88.691 1.003 82.161 0.911 84.29
ENSMUSG00000036112 Metap2 protein_coding 1.036 45.746
0.973 60.678 0.994 72.378 1.02 81.56
ENSMUSG00000036114 Rpp25l protein_coding 0.948 46.693
0.965 88.881 1.09 36.578 0.983 95.593
ENSMUSG00000036117 Il5 protein_coding 0.639 68.955 0.962
95.504 1.618 67.163 0.881 95.35
ENSMUSG00000036120 Rfxank protein_coding 0.958 60.569
1.066 87.282 0.981 88.349 0.976 90.628
ENSMUSG00000036123 Slc9a3 protein_coding 0.631 24.344
0.958 93.908 1.656 25.425 1.683 57.1
ENSMUSG00000036131 Frmd7 protein_coding 1.192 3.536 1.289 96.843
0.651 3.718 0.968 96.287
ENSMUSG00000036136 Fam110c protein_coding 0.69 25.551
1.275 92.793 1.136 58.616 0.997 96.287
ENSMUSG00000036138 Acaa1a protein_coding 0.908 11.769
1.061 92.793 1.037 39.439 0.924 83.724
ENSMUSG00000036139 Hoxc9 protein_coding 0.849 22.237 1.169
71.98 1.006 76.64 1.054 74.047
ENSMUSG00000036144 Meox2 protein_coding 1.028 82.352 1.378
60.309 0.702 1.705 0.849 84.29
ENSMUSG00000036151 Tm6sf2 protein_coding 1.034 82.092

1.044 95.941 0.922 81.62 1.126 78.379
ENSMUSG00000036155 Mgat5 protein_coding 1.075 19.132 1.064
97.155 0.874 15.63 1.091 74.193
ENSMUSG00000036158 Prickle1 protein_coding 1.086 13.363
0.937 94.262 0.981 28.669 1.09 95.74
ENSMUSG00000036160 Surf6 protein_coding 0.94 52.9 0.969 72.399
1.095 23.668 1.051 86.118
ENSMUSG00000036162 1700066J24Rik antisense 0.736 8.365 1.087
96.001 1.251 12.703 0.844 86.991
ENSMUSG00000036167 Pphln1 protein_coding 1.022 38.195
1.002 91.532 0.976 48.851 0.989 94.82
ENSMUSG00000036168 Ccdc38 protein_coding 0.78 24.817
1.185 82.103 1.079 70.623 0.842 95.559
ENSMUSG00000036169 Sostdc1 protein_coding 0.775 24.748
1.067 83.104 1.21 47.512 1.116 95.74
ENSMUSG00000036172 Cd200r3 protein_coding 1.902 12.072
1.049 94.262 0.5 26.057 0.816 90.805
ENSMUSG00000036180 Gatad2a protein_coding 0.985 79.433
1.081 60.309 0.936 5.453 1.094 92.762
ENSMUSG00000036181 Hist1h1c protein_coding 0.921 43.816 0.92
88.705 1.177 36.95 0.873 83.724
ENSMUSG00000036185 Sapcd1 protein_coding 0.868 45.229
1.097 92.561 1.052 80.015 0.864 93.612
ENSMUSG00000036186 Fam69b protein_coding 1.009 63.534
1.271 80.806 0.782 9.288 0.904 85.493
ENSMUSG00000036188 Ankmy2 protein_coding 1.052 62.007
0.948 73.166 1.004 88.461 0.98 92.181
ENSMUSG00000036192 Rorb protein_coding 1.209 7.553 0.924 85.075
0.896 75.967 0.931 88.505
ENSMUSG00000036196 NA NA NA NA 0.98 85.793 1.035
79.625 0.777 83.724
ENSMUSG00000036197 Gxylt1 protein_coding 1.048 25.207
0.984 95.504 0.965 49.733 1.015 78.546
ENSMUSG00000036198 Arhgap36 protein_coding 1.394 54.344 0.8
93.654 0.895 75.353 0.977 96.086
ENSMUSG00000036199 Ndufa13 protein_coding 0.849 3.009 1.068
86.34 1.102 38.099 1.037 91.807
ENSMUSG00000036202 Rif1 protein_coding 0.907 28.242 1.023
95.431 1.079 45.667 1.031 96.248
ENSMUSG00000036206 Sh3bp4 protein_coding 1.002 48.036
0.929 74.851 1.075 19.452 0.88 83.724
ENSMUSG00000036208 BC027231 protein_coding 1.036 83.968
1.027 96.763 0.943 66.201 0.989 96.183
ENSMUSG00000036211 Hist1h1t protein_coding 1 82.982 1
96.843 1 83.397 1 93.261
ENSMUSG00000036214 NA NA NA NA 0.951 91.275 1.023
83.275 0.958 91.105
ENSMUSG00000036223 Ska1 protein_coding 1.609 61.49 0.57 62.322
1.088 82.161 1.769 63.114
ENSMUSG00000036225 NA NA NA NA 0.875 69.611 1.141
47.776 1.003 88.053
ENSMUSG00000036231 Agr3 protein_coding 1 81.657 1
96.68 1 84.669 1 94.904
ENSMUSG00000036241 Ube2r2 protein_coding 1.099 0.664 1.038

87.185	0.877 8.142 1.081 63.708
ENSMUSG00000036242	3632451O06Rik	protein_coding	1.169 7.332
0.942 89.277	0.91 24.309	1.149 83.276
ENSMUSG00000036244	Tbc1d21	protein_coding	0.965 74.516
1.132 96.68 0.911 77.294	1.057 93.045
ENSMUSG00000036246	Gmip protein_coding	1.026 73.841	0.98
94.102	0.995 86.985	1.037 78.546
ENSMUSG00000036249	Rbm43 protein_coding	1.051 47.466	0.989
96.407	0.959 31.135	1.012 82.328
ENSMUSG00000036256	Igfbp7	protein_coding	0.881 3.895 1.014
90.116	1.123 30.645	1.039 96.086
ENSMUSG00000036257	Pnpla8	protein_coding	1.025 58.896
0.999 94.597	0.976 75.967	0.944 84.29
ENSMUSG00000036270	Edc4 protein_coding	1.006 70.121	0.965
72.576	1.027 61.171	1.038 81.061
ENSMUSG00000036273	Lrrk2 protein_coding	0.965 81.01 1.081 89.996
0.956 67.029	1.043 93.685
ENSMUSG00000036275	9530068E07Rik	protein_coding	1.052 1.967
0.994 86.604	0.955 17.898	1.043 76.467
ENSMUSG00000036278	Macrod1	protein_coding	0.968 84.865
1.069 90.116	0.967 82.31 0.882 84.19
ENSMUSG00000036279	Zbtb20	protein_coding	1	85.499	1
95.504	1	86.258	1	95.877
ENSMUSG00000036281	Snapc4	protein_coding	0.919 13.952
1.062 96.897	1.024 22.994	1.003 76.831
ENSMUSG00000036282	Naa30 protein_coding	0.973 70.121	0.978
91.361	1.047 76.498	0.992 83.724
ENSMUSG00000036285	Noa1 protein_coding	0.992 70.862	1.061
86.277	0.947 78.293	0.984 96.192
ENSMUSG00000036291	Ap5m1 protein_coding	1.146 0.461 0.966 83.392
0.899 26.809	0.902 83.724
ENSMUSG00000036292	Gramd1c	protein_coding	0.886 38.195
1.086 96.013	1.034 54.809	0.878 86.699
ENSMUSG00000036295	Lrrn3 protein_coding	1.036 80.688	0.968
95.708	0.994 81.388	0.987 93.132
ENSMUSG00000036298	Slc2a13	protein_coding	0.882 63.126	1.03
96.843	1.102 86.602	0.992 86.699
ENSMUSG00000036299	BC031181	protein_coding	0.855 0.987 1.025
82.904	1.141 10.281	0.983 96.358
ENSMUSG00000036304	Zdhhc23	protein_coding	1.198 62.689
1.143 93.146	0.731 42.621	0.998 93.964
ENSMUSG00000036306	Lzts1 protein_coding	1.081 58.709	1.29
60.309	0.717 1.061 1.495 72.369
ENSMUSG00000036309	Skp1a protein_coding	1.041 69.408	0.99
90.744	0.97 86.144	1.036 73.939
ENSMUSG00000036315	Znrd1 protein_coding	1.08 10.464	1.093
82.904	0.844 3.073 0.988 93.156
ENSMUSG00000036323	Srp72 protein_coding	1.075 35.024	0.995
85.793	0.937 73.874	1.022 43.377
ENSMUSG00000036327	Qsox2 protein_coding	1.011 84.049	0.976
96.013	1.014 85.757	1.018 90.177
ENSMUSG00000036330	Slc18a1	protein_coding	1.033 63.804
0.788 79.807	1.232 70.9 1.236 90.397
ENSMUSG00000036333	Kidins220	protein_coding	1.097 0.2	0.952

75.993 0.958 14.079 0.959 83.724
ENSMUSG00000036334 Igsf10 protein_coding 1.461 9.206 0.841
90.046 0.815 27.321 1.089 93.156
ENSMUSG00000036339 Tmem260 protein_coding 1.026 65.284
0.959 65.819 1.019 50.091 0.977 88.245
ENSMUSG00000036352 Ubac1 protein_coding 0.972 30.352 1.034
82.904 0.995 82.436 0.973 96.183
ENSMUSG00000036353 P2ry12 protein_coding 1.893 0 0.831
65.165 0.634 1.764 1.325 57.1
ENSMUSG00000036356 Csgalnact1 protein_coding 1.34 14.59 0.924
93.654 0.81 12.41 0.928 92.181
ENSMUSG00000036357 Gpr101 protein_coding 1 82.092 1
94.848 1 84.071 1 96.183
ENSMUSG00000036362 P2ry13 protein_coding 1.343 12.855
1.147 95.735 0.651 3.428 1.08 94.82
ENSMUSG00000036368 Rmdn2 protein_coding 0.87 21.897 0.996
90.744 1.155 14.873 0.942 90.854
ENSMUSG00000036371 Serbp1 protein_coding 1.125 0 0.98
62.322 0.904 2.563 1.044 46.412
ENSMUSG00000036372 Tmem258 protein_coding 0.988 46.413
1.063 84.007 0.95 84.724 1.09 46.846
ENSMUSG00000036376 Abt1 protein_coding 1.027 85.129 0.944
76.431 1.032 58.414 0.938 84.29
ENSMUSG00000036377 C530008M17Rik protein_coding 1.157 39.851
0.75 70.864 1.153 76.754 1.09 86.301
ENSMUSG00000036381 P2ry14 protein_coding 1.048 37.941
1.028 96.847 0.926 52.213 1.151 87.447
ENSMUSG00000036390 Gadd45a protein_coding 0.946 83.169 1.15
84.007 0.914 49.967 0.986 95.273
ENSMUSG00000036391 Sec24a protein_coding 1.001 84.564
1.085 61.032 0.923 7.155 1.003 91.65
ENSMUSG00000036395 Glb1l2 protein_coding 1.217 3.893 1.039
96.001 0.791 27.194 0.872 87.735
ENSMUSG00000036398 Ppp1r11 protein_coding 0.863 13.952
1.061 82.904 1.09 58.414 1.001 92.381
ENSMUSG00000036401 Glt6d1 protein_coding 1 75.196 1
95.301 1 87.519 1 95.273
ENSMUSG00000036402 Gng12 protein_coding 0.988 81.196 1.153
70.816 0.879 12.571 1.393 68.696
ENSMUSG00000036403 Cep135 protein_coding 0.896 19.595 NA
NA 1.15 20.315 1.082 57.1
ENSMUSG00000036411 9530077C05Rik protein_coding 0.881 58.264
1.051 90.389 1.078 70.1 0.894 96.086
ENSMUSG00000036412 Arsi protein_coding 1.313 2.446 1.204 82.904
0.634 1.342 1.286 53.923
ENSMUSG00000036422 Pcdh8 protein_coding 1.214 80.251 0.453
85.95 1.809 61.743 0.687 89.294
ENSMUSG00000036427 Gpi1 protein_coding 0.754 1.815 1.102 95.708
1.201 6.202 1.144 49.239
ENSMUSG00000036430 Tbcc protein_coding 1.003 44.562 0.994
93.707 1 70.534 0.957 86.699
ENSMUSG00000036432 Siah2 protein_coding 1.136 23.493 1.025
82.904 0.859 5.701 0.934 90.805
ENSMUSG00000036435 Exoc1 protein_coding 0.978 47.677 0.954

45.262 1.076 8.93 1.073 44.882
ENSMUSG00000036437 Npy1r protein_coding 1.472 14.205 1.131
92.931 0.601 7.809 1.086 81.251
ENSMUSG00000036438 Calm2 protein_coding 1.07 4.628 0.988 81.731
0.948 15.63 1.072 31.133
ENSMUSG00000036442 Thap11 protein_coding 1.021 50.913
1.011 95.809 0.97 61.288 0.995 86.445
ENSMUSG00000036446 Lum protein_coding 1.752 0 0.921 85.116
0.617 0.72 1.099 67.691
ENSMUSG00000036450 Hif1an protein_coding 1.039 70.121
1.008 95.211 0.951 40.19 0.964 94.99
ENSMUSG00000036452 Arhgap26 protein_coding 0.883 73.487
1.023 91.643 1.102 76.498 0.944 84.29
ENSMUSG00000036459 Wtip protein_coding 1.048 70.741 0.912
93.654 1.041 84.961 1.012 87.886
ENSMUSG00000036461 Elf1 protein_coding 1.083 3.825 0.984 95.088
0.94 22.864 0.928 83.724
ENSMUSG00000036463 4930544G11Rik protein_coding 1 84.263
1 97.155 1 85.134 1 96.358
ENSMUSG00000036466 Megf11 protein_coding 1.561 38.386
0.584 77.944 1.099 79.897 1.537 57.1
ENSMUSG00000036469 1-Mar protein_coding 1.409 1.188 0.69 8.462 NA
NA 1.208 78.546
ENSMUSG00000036473 Tbc1d24 protein_coding 1.198 5.527 1.108
82.904 0.753 2.087 0.851 83.724
ENSMUSG00000036478 Btg1 protein_coding 1.176 0.2 0.871 53.434
0.973 39.755 0.984 90.674
ENSMUSG00000036480 Prss56 protein_coding 1 71.142 NA
NA 1 88.693 1 96.139
ENSMUSG00000036492 Rnf39 protein_coding 0.796 20.659 1.026
96.68 1.223 35.441 0.947 83.724
ENSMUSG00000036499 Eea1 protein_coding 1.01 66.76 1.011 93.908
0.978 87.744 1 91.928
ENSMUSG00000036500 Akp3 protein_coding 1 84.564 1
93.469 NA NA 1 96.287
ENSMUSG00000036501 Fam13b protein_coding 0.995 72.546
1.044 91.776 0.964 80.015 0.981 93.612
ENSMUSG00000036502 Tmem255a protein_coding 0.99 72 1.079
93.994 0.938 43.426 1.233 94.016
ENSMUSG00000036503 Rnf13 protein_coding 1.081 8.537 0.988 89.713
0.939 21.117 0.954 84.29
ENSMUSG00000036504 Phpt1 protein_coding 0.79 21.658 1.034
96.847 1.22 30.948 0.925 88.505
ENSMUSG00000036513 Commd2 protein_coding 1.086 65.284
1.029 83.104 0.892 13.551 0.985 94.99
ENSMUSG00000036521 Scgb2b2 protein_coding 1 85.637 1
96.216 1 85.675 1 92.381
ENSMUSG00000036523 Greb1 protein_coding 0.725 69.985 1.611
82.904 0.855 55.214 4.914 67.691
ENSMUSG00000036526 Card11 protein_coding 1.342 11.638
1.075 90.116 0.692 2.563 0.837 83.724
ENSMUSG00000036528 Ppfibp2 protein_coding 0.848 22.237 0.96
88.704 1.222 14.169 1.066 92.748
ENSMUSG00000036529 Sbf1 protein_coding 0.976 35.158 1.021

87.029 1 80.899 0.991 95.74
ENSMUSG00000036533 Cdc42ep3 protein_coding 0.998 74.745
1.036 83.104 0.968 30.827 0.933 83.724
ENSMUSG00000036534 Slc38a7 protein_coding 1.006 59.806
1.034 96.763 0.959 38.534 0.985 85.493
ENSMUSG00000036537 Rnf113a1 protein_coding 0.937 47.677
1.033 92.111 1.035 84.308 0.95 95.273
ENSMUSG00000036545 Adamts2 protein_coding 1.223 4.675 0.856
71.889 0.953 53.7 1.3 46.846
ENSMUSG00000036550 Cnot1 protein_coding 1.062 23.493 0.965
87.998 0.976 49.217 0.973 88.136
ENSMUSG00000036552 Ermard protein_coding 1.137 12.72 0.9
64.624 0.976 82.575 1.044 82.328
ENSMUSG00000036553 Sh3tc1 protein_coding 1.038 82.891
0.982 95.504 0.984 82.74 0.969 83.581
ENSMUSG00000036555 Iqce protein_coding 0.949 25.3 0.979 91.275
1.074 14.597 1.011 94.752
ENSMUSG00000036560 Lgi4 protein_coding 1.032 82.231 0.99
96.138 0.98 87.336 1.086 60.466
ENSMUSG00000036561 Ppp6r2 protein_coding 1.012 77.182
0.982 94.767 1.003 60.233 0.985 94.654
ENSMUSG00000036564 Ndrg4 protein_coding 0.996 78.744 1.163
87.029 0.865 64.778 1.074 61.316
ENSMUSG00000036565 Ttyh3 protein_coding 1.049 56.085 1.087
82.904 0.875 4.935 1.041 83.067
ENSMUSG00000036568 Gltscr1l protein_coding 0.999 83.658
1.023 81.127 0.975 73.874 0.953 84.29
ENSMUSG00000036570 Fxyd1 protein_coding 0.921 7.881 1.07 79.411
1.016 65.8 0.964 92.181
ENSMUSG00000036572 Upf3b protein_coding 0.89 28.804 0.97
86.861 1.162 10.819 1.01 95.877
ENSMUSG00000036574 1700019O17Rik protein_coding 1 85.728
1 92.137 1 82.161 1 91.625
ENSMUSG00000036578 Fxyd7 protein_coding 0.945 71.674 0.802
68.848 1.317 37.741 1.006 92.561
ENSMUSG00000036580 Spg20 protein_coding 1.103 11.197 1.026
92.561 0.885 6.858 0.965 91.372
ENSMUSG00000036585 Fgf1 protein_coding 0.902 43.495 1.076
83.104 1.027 75.155 0.962 88.245
ENSMUSG00000036587 Fut7 protein_coding 1.26 38.386 0.747
81.134 1.064 87.744 0.84 91.291
ENSMUSG00000036591 Arhgap21 protein_coding 1.053 16.249 1.06
71.222 0.898 1.342 1.024 96.287
ENSMUSG00000036594 H2-Aa protein_coding 1.071 85.283 1.196
83.104 0.783 87.744 0.786 84.29
ENSMUSG00000036596 Cpz protein_coding 0.735 13.952 0.969
95.211 1.405 31.4 1.193 83.276
ENSMUSG00000036598 Ccdc113 protein_coding 1.972 39.295
0.597 71.889 0.847 71.839 0.422 88.053
ENSMUSG00000036599 Chst12 protein_coding 1.012 47.174
0.996 95.708 0.989 48.133 0.963 83.952
ENSMUSG00000036602 NA NA NA NA 1 95.088 1
79.426 1 94.904
ENSMUSG00000036606 Plxnb2 protein_coding 1.136 0.2 1.002

75.345	0.879 9.563 0.967 89.475
ENSMUSG00000036611	Eepd1 protein_coding	0.963 75.298	0.989
96.843	NA	NA	1.106 93.117
ENSMUSG00000036613	Tssc1 protein_coding	0.798 1.422 1.047 72.642
1.196 22.366	1.086 92.381
ENSMUSG00000036615	Rfxap protein_coding	1.03	75.399	0.992
85.921	0.976 80.634	0.914 83.724
ENSMUSG00000036617	Etl4	protein_coding	0.956 44.922	0.937
15.938	1.114 5.864 0.863 83.724
ENSMUSG00000036620	Mgat4b	protein_coding	1.072 75.399
1.138 72.642	0.822 6.515 0.96	89.152
ENSMUSG00000036622	Atp13a2	protein_coding	1.125 22.633
0.934 67.513	0.951 77.294	0.99	91.372
ENSMUSG00000036632	Alg5	protein_coding	1.011 85.59 NA	NA
1.004 88.41 1.055 67.691
ENSMUSG00000036634	Mag	protein_coding	1.087 82.982	1.128
84.007	0.814 25.345	1.051 68.696
ENSMUSG00000036636	Clcn7 protein_coding	1.053 20.296	0.997
90.256	0.955 21.431	0.946 90.289
ENSMUSG00000036639	Nudt1 protein_coding	0.975 69.985	0.894
85.242	1.145 31.761	1.058 92.762
ENSMUSG00000036641	Ccdc148	protein_coding	1.342 82.231
0.689 91.955	1.078 87.173	0.914 92.675
ENSMUSG00000036644	Tbc1d9b	protein_coding	1.025 42.255	0.96
73.766	1.013 88.601	1.028 87.447
ENSMUSG00000036646	Man1b1	protein_coding	1.015 55.789
1.005 95.088	0.978 67.029	1.004 92.52
ENSMUSG00000036647	NA	NA	NA	NA	1	94.102	1
76.754	1	89.152
ENSMUSG00000036648	Gm3417	protein_coding	0.845 81.125
1.314 91.361	0.904 63.801	1.436 85.577
ENSMUSG00000036655	Colec11	protein_coding	1.201 14.205
0.891 75.052	0.93	86.475	1.056 95.74
ENSMUSG00000036658	Olfr11	protein_coding	1	83.823	1
93.529	1	71.004	1	89.475
ENSMUSG00000036661	Dennd3	protein_coding	1	82.231
0.949 68.818	1.055 38.683	0.924 85.947
ENSMUSG00000036667	Fam115a	protein_coding	1.029 86.57 1.018
83.104	0.955 45.093	1.01	93.722
ENSMUSG00000036672	Cenpt protein_coding	0.987 51.056	0.985
96.407	1.028 69.315	1.083 42.736
ENSMUSG00000036676	Tmtc3 protein_coding	1.038 67.608	1.042
83.104	0.928 32.082	0.962 85.493
ENSMUSG00000036678	Aaas	protein_coding	1.078 13.557	1.01
96.216	0.918 16.949	1.051 69.918
ENSMUSG00000036686	Cc2d1a	protein_coding	0.962 32.168	0.98
91.532	1.062 12.16 1	90.95
ENSMUSG00000036687	Tmem184a	protein_coding	0.876 28.242	NA
NA	1.112 41.6	0.834 83.724
ENSMUSG00000036693	Nop14 protein_coding	1.011 84.168	0.947
59.396	1.046 38.975	1.043 55.054
ENSMUSG00000036698	Ago2	protein_coding	1.011 84.168	1.008
95.809	0.978 85.134	0.956 85.493
ENSMUSG00000036699	Zcchc12	protein_coding	1.192 82.732

0.927 96.138 0.905 87.336 0.62 90.854
ENSMUSG00000036707 Cab39 protein_coding 1.031 26.136 0.975
90.378 0.995 55.214 1.013 92.263
ENSMUSG00000036712 Cyld protein_coding 1.026 63.385 0.951
83.58 1.027 71.615 0.97 90.157
ENSMUSG00000036718 Micall2 protein_coding 0.767 4.73 1.038
96.847 1.251 24.383 0.995 91.65
ENSMUSG00000036721 Zscan12 protein_coding 1.13 4.925 0.859
24.911 1.032 71.232 1.021 95.593
ENSMUSG00000036731 2310002J15Rik protein_coding 0.738 12.084
0.921 85.116 1.466 7.635 1.289 57.075
ENSMUSG00000036733 Rbm42 protein_coding 0.811 69.985 1.06
95.35 1.162 51.596 0.847 96.086
ENSMUSG00000036737 Oxsr1 protein_coding 0.993 81.465 1.007
96.847 1.003 83.719 1.004 92.564
ENSMUSG00000036743 Psma8 protein_coding 0.786 78.744 1.207
96.216 1.05 77.532 1.378 64.513
ENSMUSG00000036744 Olfr701 protein_coding 1 85.59 1
96.216 1 80.5 1 95.559
ENSMUSG00000036745 Ttll7 protein_coding 0.915 56.242 0.943
94.48 1.155 56.497 1.036 88.111
ENSMUSG00000036748 Cuedc2 protein_coding 0.906 22.481 1.04
93.899 1.065 31.761 1.084 57.711
ENSMUSG00000036749 Pramel5 protein_coding 1 77.763 1
97.155 1 88.076 1 96.358
ENSMUSG00000036751 Cox6b1 protein_coding 0.846 1.031 1.183
60.309 0.996 63.665 0.984 93.612
ENSMUSG00000036752 Tubb4b protein_coding 0.733 2.644 1.205
71.98 1.133 36.95 1.185 31.133
ENSMUSG00000036760 Kcnk9 protein_coding 1 86.145 1
97.155 1 87.173 1 95.593
ENSMUSG00000036764 Dnajc12 protein_coding 1.013 74.482
0.859 62.71 1.149 37.369 0.699 83.952
ENSMUSG00000036766 Dner protein_coding 1.761 81.24 2.104 83.104
0.271 20.712 0.677 95.72
ENSMUSG00000036768 Kif15 protein_coding 1.325 49.868 0.907
75.993 0.831 81.388 1.717 61.984
ENSMUSG00000036769 Wdr44 protein_coding 1.127 5.109 NA NA
0.907 12.571 1.057 71.008
ENSMUSG00000036770 4933433C11Rik protein_coding 0.151 17.031
0.771 96.138 8.645 21.873 3.21 66.944
ENSMUSG00000036775 Decr2 protein_coding 1.021 40.635 1.029
96.847 0.947 33.402 0.977 91.625
ENSMUSG00000036777 Anln protein_coding 1.111 38.195 0.857
51.322 1.048 81.857 1.257 73.368
ENSMUSG00000036779 Papd5 protein_coding 0.962 68.955 1.006
94.848 1.028 57.637 0.98 83.724
ENSMUSG00000036781 Rps27l protein_coding 1.056 68.727
0.992 88.881 0.956 88.729 1.032 85.786
ENSMUSG00000036782 Klhl13 protein_coding 1.265 5.527 1.119
96.216 0.707 33.402 0.902 84.19
ENSMUSG00000036790 Slitrk2 protein_coding 0.797 69.985
1.355 83.104 0.927 58.271 0.99 92.12
ENSMUSG00000036792 Mbd5 protein_coding 1.059 18.635 1.072

96.216 0.881 11.997 0.956 87.182
ENSMUSG00000036800 Fam135b protein_coding 1 85.655 1
95.301 1 88.461 1 94.904
ENSMUSG00000036810 Cnep1r1 protein_coding 1.101 1.292 1.035
90.29 0.876 0.72 1.026 80.77
ENSMUSG00000036813 Entpd8 protein_coding 0.442 26.217 1.3
97.288 1.737 57.637 0.828 96.287
ENSMUSG00000036814 Slc6a20a protein_coding 2.634 52.799
0.498 82.671 0.761 86.084 1.256 88.657
ENSMUSG00000036817 Sun1 protein_coding 1.044 50.111 0.941
71.98 1.018 61.841 0.98 88.053
ENSMUSG00000036819 Jmjd4 protein_coding 0.96 49.368 1.017
93.574 1.023 86.985 1.052 83.067
ENSMUSG00000036820 Amdhd2 protein_coding 1.121 2.567 0.958
79.261 0.928 9.288 1.054 95.273
ENSMUSG00000036822 Topors protein_coding 1.099 3.893 1.002
95.196 0.905 4.759 0.956 84.29
ENSMUSG00000036825 Ssx2ip protein_coding 1.204 1.949 NA NA
0.746 3.654 1.127 96.287
ENSMUSG00000036826 Igflr1 protein_coding 1 82.092 1
94.102 1 87.274 1 96.192
ENSMUSG00000036832 Lpar3 protein_coding 1.197 66.944 1.476
88.136 0.568 48.851 0.644 95.593
ENSMUSG00000036833 Pnpla7 protein_coding 1.013 66.58 0.843
51.322 1.172 42.065 1.022 90.611
ENSMUSG00000036834 Plch1 protein_coding 1.101 84.738 0.668
65.819 1.36 43.586 1.148 74.047
ENSMUSG00000036835 Psenen protein_coding 1.021 49.868
0.997 96.216 0.981 39.528 0.937 83.724
ENSMUSG00000036840 Siah1a protein_coding 1.013 82.231
0.965 94.848 1.022 77.532 0.947 84.19
ENSMUSG00000036845 Lin37 protein_coding 0.837 3.401 1.108 82.904
1.078 19.025 0.999 95.877
ENSMUSG00000036850 Mrpl41 protein_coding 0.908 35.158 NA
NA 1.118 36.388 1.023 82.85
ENSMUSG00000036854 Hspb6 protein_coding 1.449 23.493 1.08
84.007 0.638 5.323 0.994 92.018
ENSMUSG00000036855 Gjd4 protein_coding 1 75.761 1
96.216 1 88.693 1 87.447
ENSMUSG00000036856 Wnt4 protein_coding 0.736 24.577 0.826
59.946 1.643 15.411 1.01 89.892
ENSMUSG00000036860 Mrpl55 protein_coding 0.94 34.305
1.142 71.617 0.927 68.266 0.978 85.493
ENSMUSG00000036862 Dchs1 protein_coding 1.116 29.372 NA NA
0.931 31.676 1.008 92.12
ENSMUSG00000036863 Syde2 protein_coding 1.344 35.787 0.838
79.807 0.889 73.531 1.052 95.74
ENSMUSG00000036864 BC053749 protein_coding 0.887 2.009 1.095
71.617 1.026 40.511 0.929 84.29
ENSMUSG00000036867 Smad6 protein_coding 0.861 54.983 0.979
97.155 1.182 69.002 1.126 96.183
ENSMUSG00000036872 Abcc12 protein_coding 2.383 12.072
0.805 94.597 0.52 39.528 1.129 95.35
ENSMUSG00000036873 2410004B18Rik protein_coding 0.952 48.939

1.032 84.999 1.018 85.757 1.051 92.018
ENSMUSG00000036875 Dna2 protein_coding 1.181 15.85 0.845 65.165
1.004 86.346 1.121 69.708
ENSMUSG00000036879 Phkb protein_coding 0.875 1.031 1.037 89.494
1.101 11.782 1.043 76.467
ENSMUSG00000036880 Acaa2 protein_coding 0.884 68.846 1.018
95.941 1.111 43.842 1.09 92.691
ENSMUSG00000036882 Arhgap33 protein_coding 0.82 25.3 1.234
83.104 0.986 84.308 1.007 95.676
ENSMUSG00000036885 Arhgef26 protein_coding 0.869 35.894
0.925 93.654 1.24 30.292 1.073 96.071
ENSMUSG00000036887 C1qa protein_coding 1.224 1.079 0.862 73.766
0.944 51.715 1.179 69.918
ENSMUSG00000036890 Gtdc1 protein_coding 1.206 0.577 NA NA
0.848 0.879 1.033 92.748
ENSMUSG00000036892 Prodh2 protein_coding 1 85.77 1
96.843 1 87.336 1 95.593
ENSMUSG00000036893 Ehmt1 protein_coding 0.971 44.562 0.981
91.83 1.051 42.522 1.048 78.546
ENSMUSG00000036894 Rap2b protein_coding 1.05 24.253 1.109
40.99 0.859 0.72 1.029 80.497
ENSMUSG00000036896 C1qc protein_coding 1.221 0.2 0.894 82.671
0.916 21.117 1.108 80.497
ENSMUSG00000036898 Zfp157 protein_coding 1.057 25.207
0.862 56.781 1.099 54.163 0.999 96.358
ENSMUSG00000036899 Trpv5 protein_coding 1 80.815 1
96.68 1 88.203 1 95.676
ENSMUSG00000036902 Neto2 protein_coding 0.878 67.163 0.975
93.654 1.169 54.673 1.265 66.468
ENSMUSG00000036904 Fzd8 protein_coding 0.802 13.45 1.131 79.29
1.098 48.304 0.936 85.493
ENSMUSG00000036905 C1qb protein_coding 1.386 0 0.929 74.851
0.775 8.907 1.188 64.905
ENSMUSG00000036908 Unc93b1 protein_coding 1.187 1.81 0.906
68.818 0.929 39.245 1.022 96.086
ENSMUSG00000036912 Piwil4 protein_coding 1.49 19.477
0.963 97.049 0.698 31.135 0.807 89.892
ENSMUSG00000036913 Trim67 protein_coding 1.218 46.013
0.937 91.776 0.875 23.607 0.923 83.724
ENSMUSG00000036915 Kirrel2 protein_coding 1 65.388 1
95.735 1 86.602 1 90.125
ENSMUSG00000036916 Zfp280c protein_coding 1.016 84.819
0.916 83.876 1.071 53.882 0.94 83.724
ENSMUSG00000036918 Ttc7 protein_coding 1.112 54.717 0.992
95.735 0.903 25.052 0.965 96.287
ENSMUSG00000036921 4930549C01Rik protein_coding 1 78.639
NA NA 1 83.607 1 95.777
ENSMUSG00000036923 Stox1 protein_coding 1.407 6.135 0.896 85.793
0.792 28.901 0.77 83.724
ENSMUSG00000036924 Cst13 protein_coding 1 77.763 1
93.899 1 86.144 1 96.086
ENSMUSG00000036925 Spt1 protein_coding 1 81.465 1
95.211 1 71.232 1 96.183
ENSMUSG00000036928 Stag3 protein_coding 1.208 45.307 0.751

82.503 1.101 87.744 0.972 93.261
ENSMUSG00000036931 Nfkbid protein_coding 1.1 70.36 0.792
87.147 1.148 61.171 0.966 93.612
ENSMUSG00000036932 Aifm1 protein_coding 0.921 22.431 1.061
90.454 1.023 56.708 1.049 89.498
ENSMUSG00000036934 4921524J17Rik protein_coding 1.079 6.135
0.953 91.897 0.975 28.669 0.973 87.651
ENSMUSG00000036938 Try5 protein_coding 1 76.042 1
94.848 1 87.786 1 95.273
ENSMUSG00000036940 Kdm1a protein_coding 1.019 54.717 0.954
76.431 1.031 79.426 0.956 84.29
ENSMUSG00000036941 Elac1 protein_coding 1.123 10.464 0.907
53.434 0.984 82.31 0.97 90.157
ENSMUSG00000036943 Rab8b protein_coding 1.433 0 0.97 92.545
0.717 0.879 1.018 93.156
ENSMUSG00000036944 Tmem71 protein_coding 1.378 2.651 0.909
60.678 0.795 24.309 0.857 85.493
ENSMUSG00000036948 BC037034 protein_coding 1 77.615 1
96.308 1 84.896 1 93.457
ENSMUSG00000036949 Slc39a12 protein_coding 1.076 62.574
0.619 79.261 1.501 69.315 0.995 96.248
ENSMUSG00000036951 NA NA NA NA 1 93.574 1
86.552 1 95.74
ENSMUSG00000036955 2510003E04Rik protein_coding 1.019 81.52
1.027 83.104 0.957 55.9 1.015 92.389
ENSMUSG00000036957 Lrfn3 protein_coding 1.165 8.281 0.993 95.431
0.864 16.643 1.036 77.331
ENSMUSG00000036959 Bcorl1 protein_coding 0.98 85.092
1.227 71.617 0.832 1.342 0.97 83.724
ENSMUSG00000036960 Clca5 protein_coding 0.765 7.574 1.601 60.309
0.817 25.625 0.938 91.372
ENSMUSG00000036962 Gm101 protein_coding 0.858 48.939 1.715
82.904 0.677 68.266 0.824 86.699
ENSMUSG00000036964 Trim17 protein_coding 0.606 11.707
1.013 83.464 1.623 51.977 0.941 85.493
ENSMUSG00000036966 Spryd3 protein_coding 1.027 63.126
0.999 95.708 0.976 50.523 0.996 92.018
ENSMUSG00000036968 Cnpy4 protein_coding 1.001 73.124 0.942
91.83 1.061 84.896 1.098 42.736
ENSMUSG00000036975 Tmem177 protein_coding 0.76 10.29 1.347
75.21 0.974 69.196 0.908 83.724
ENSMUSG00000036977 Anapc10 protein_coding 1.108 6.958 1.021
87.313 0.886 52.658 1.08 67.691
ENSMUSG00000036980 Taf6 protein_coding 0.908 8.181 1.043 93.146
1.057 9.176 0.989 91.65
ENSMUSG00000036983 Tfb1m protein_coding 1.281 5.016 1.015 95.329
0.769 8.634 0.951 94.99
ENSMUSG00000036985 Zdhhc9 protein_coding 1.1 5.559 1.092
82.904 0.83 1.705 1.042 86.209
ENSMUSG00000036986 Pml protein_coding 1.09 6.026 0.948 95.708
0.969 36.786 1.155 38.598
ENSMUSG00000036989 Trim3 protein_coding 0.913 8.938 NA NA
1.108 15.688 0.991 85.493
ENSMUSG00000036990 Otud4 protein_coding 0.975 84.661 1.027

92.062	0.998 84.552	0.947 84.29
ENSMUSG00000036992	Nxt1	protein_coding	1.007 80.688	1.071
85.756	0.924 44.211	0.996 95.72
ENSMUSG00000036995	Asap3 protein_coding	1.123 9.983 1.027 91.456
0.864 1.764 0.96	83.724
ENSMUSG00000037001	Zfp39 protein_coding	1.015 73.841	0.925
71.41 1.066 71.839	1.166 68.696
ENSMUSG00000037003	Tenc1 protein_coding	1.049 50.627	0.977
91.897	0.971 76.768	0.999 93.413
ENSMUSG00000037005	Xpnpep2	protein_coding	1.451 0	0.887
72.576	0.773 6.858 1.116 63.114
ENSMUSG00000037007	Zfp113	protein_coding	0.985 85.637
0.951 81.443	1.065 34.07 0.978 92.466
ENSMUSG00000037010	Apln	protein_coding	0.524 2.009 1.676 64.304
1.134 50.091	0.843 86.699
ENSMUSG00000037012	Hk1	protein_coding	0.987 61.633	0.933
34.058	1.084 17.412	1.008 92.263
ENSMUSG00000037013	Ss18	protein_coding	0.998 43.816	0.955
92.545	1.049 33.287	1.034 56.079
ENSMUSG00000037014	Sstr4 protein_coding	1.273 21.278	0.883
85.116	0.89	68.266	0.956 93.261
ENSMUSG00000037015	Tmem185b	pseudogene	1	77.763	1
94.848	1	81.62 1	95.74
ENSMUSG00000037016	Frem2 protein_coding	0.884 48.646	1.004
96.407	1.12	61.418	0.982 96.086
ENSMUSG00000037017	Zscan21	protein_coding	1.008 86.474
1.013 90.454	0.976 55.214	1.036 91.872
ENSMUSG00000037020	Wdr62 protein_coding	0.926 30.035	NA	NA
1.153 54.363	1.08	78.379
ENSMUSG00000037022	Mmaa	protein_coding	0.914 21.658	0.931
94.96 1.172 7.482 1.063 44.679
ENSMUSG00000037028	Gm12800	protein_coding	1	81.196	1
96.216	NA	NA	1	92.564
ENSMUSG00000037029	Zfp146	protein_coding	1.044 14.746
0.949 55.265	1.01	86.258	1.006 94.654
ENSMUSG00000037031	Tspan15	protein_coding	0.922 54.604	0.96
93.654	1.132 69.196	0.901 83.724
ENSMUSG00000037032	Apbb1 protein_coding	1.031 86.199	0.972
96.138	0.995 84.434	0.933 84.29
ENSMUSG00000037034	Pax1	protein_coding	0.557 37.248	1.022
96.308	1.752 38.975	1.638 68.696
ENSMUSG00000037035	Inhbb protein_coding	0.717 3.101 0.98	96.843
1.42	10.76 0.984 86.699
ENSMUSG00000037049	Smpd1 protein_coding	0.9	1.274 1.073 79.411
1.038 46.206	0.983 93.584
ENSMUSG00000037053	Azgp1 protein_coding	2.162 10.595	1.077
96.68 0.429 23.1	0.406 83.724
ENSMUSG00000037058	Paip2 protein_coding	1.002 80.815	0.974
94.597	NA	NA	1.001 95.72
ENSMUSG00000037060	Prkcdbp	protein_coding	0.838 43.435
1.146 87.609	1.038 87.274	1.169 88.797
ENSMUSG00000037062	Sh3glb1	protein_coding	0.908 21.658	NA
NA	1.054 30.948	0.997 83.067
ENSMUSG00000037064	Gm5229	pseudogene	1	76.135	1

94.96 1 86.666 1 96.139
ENSMUSG00000037070 Rbmxl1 protein_coding 1.067 45.746
0.954 91.361 0.982 63.919 1.049 72.204
ENSMUSG00000037071 Scd1 protein_coding 0.383 0.987 0.918 86.861
2.837 5.864 1.162 92.381
ENSMUSG00000037072 15-Sep protein_coding 0.978 49.749
1.063 80.806 0.958 63.075 1.037 61.753
ENSMUSG00000037075 Rnf139 protein_coding 1.079 1.292 1.041
92.561 0.891 5.042 1.036 91.335
ENSMUSG00000037085 Trmt12 protein_coding 1.002 57.949
1.039 96.897 0.962 66.201 1.006 86.991
ENSMUSG00000037086 1110059M19Rik protein_coding 1.328 41.454
0.926 92.137 0.812 65.688 2.114 71.305
ENSMUSG00000037089 Slc35b2 protein_coding 1.098 8.685 0.957
79.475 0.954 33.971 0.984 91.625
ENSMUSG00000037095 Lrg1 protein_coding 1.029 65.537 1.168
79.411 0.829 15.024 2.242 71.653
ENSMUSG00000037096 Gm9762 pseudogene 1 75.196 1
97.129 1 84.669 1 90.289
ENSMUSG00000037098 Rab11fip3 protein_coding 0.996 85.203
0.996 97.097 1.002 86.346 0.983 96.358
ENSMUSG00000037101 Ttc29 protein_coding 1 86.199 NA NA
1 85.21 1 96.086
ENSMUSG00000037103 Dcaf15 protein_coding 0.852 8.709 1.058
89.996 1.11 27.533 1.005 95.72
ENSMUSG00000037104 Socs5 protein_coding 0.967 40.568 1.091
60.309 0.949 22.864 1.002 93.132
ENSMUSG00000037106 Fer1l6 protein_coding 0.962 58.264
0.919 88.923 NA NA 1.779 63.018
ENSMUSG00000037108 Zcwpw1 protein_coding 0.912 54.043
0.992 96.652 1.099 58.915 0.949 96.139
ENSMUSG00000037110 Ralgapa2 protein_coding 0.833 0.987 1.006
93.899 1.187 8.586 0.889 95.92
ENSMUSG00000037111 Setd7 protein_coding 1.113 6.853 0.998 92.137
0.898 4.759 0.972 85.493
ENSMUSG00000037112 Sik2 protein_coding 1.041 67.608 0.969
92.137 0.989 86.727 0.885 90.628
ENSMUSG00000037119 D15Ertd621e protein_coding 1.132 11.879
0.984 87.041 0.895 23.212 0.974 87.705
ENSMUSG00000037126 Psd protein_coding 0.87 38.195 1.064
86.277 1.081 64.624 0.874 83.724
ENSMUSG00000037129 Tmprss13 protein_coding 1.518 0.984 0.889
70.971 0.742 7.083 0.892 90.611
ENSMUSG00000037130 H2-M10.6 protein_coding 1 82.543 1
95.708 1 80.634 1 95.877
ENSMUSG00000037134 Prmt10 protein_coding 1.072 50.627
0.945 79.475 NA NA 0.972 91.492
ENSMUSG00000037138 Aff3 protein_coding 1.207 56.311 1.022
93.574 0.81 41.662 1.452 60.31
ENSMUSG00000037139 Myom3 protein_coding 1.142 70.121 1.157
80.678 0.759 18.797 1.291 93.964
ENSMUSG00000037145 2210407C18Rik protein_coding 1 76.546
1 95.504 1 76.768 1 92.018
ENSMUSG00000037148 Arhgap10 protein_coding 1.058 14.59 0.91

47.831	1.036 82.161	1.107 44.679
ENSMUSG00000037149	Ddx1	protein_coding	1.034 82.352	0.988
96.268	NA	NA	1.096 31.133
ENSMUSG00000037151	Lrrc20	protein_coding	1.337 4.465 1.112
97.155	0.671 0.72	1.078 88.197
ENSMUSG00000037152	Ndufc1	protein_coding	0.985 40.568
0.997 95.431	1.014 68.425	0.97	88.245
ENSMUSG00000037157	Il22ra1	protein_coding	0.92	76.968
0.906 95.35 1.195 73.428	1.219 93.734
ENSMUSG00000037159	Wee2	protein_coding	1	82.352	1
88.881	1	56.125	1	95.273
ENSMUSG00000037161	Mgarp protein_coding	1.111 57.949	0.806
90.053	1.116 87.274	1.703 80.77
ENSMUSG00000037166	Ppp1r14a	protein_coding	0.984 84.049
1.004 93.046	1.015 66.928	0.883 84.29
ENSMUSG00000037167	Spaca5	protein_coding	1	57.818	1
96.68 1	83.857	1	93.734
ENSMUSG00000037169	Mycn	protein_coding	0.92	62.437	1.422
83.104	0.766 71.734	1.057 95.74
ENSMUSG00000037171	Nodal protein_coding	0.672 23.923	1.509
96.216	0.984 81.492	0.876 90.805
ENSMUSG00000037172	E330009J07Rik	protein_coding	1.162 2.969 0.89
61.883	0.97	45.799	0.976 92.12
ENSMUSG00000037174	Elf2	protein_coding	1.014 61.49 0.986 95.708
0.999 75.353	1.037 51.967
ENSMUSG00000037185	Krt80 protein_coding	1.193 13.557	0.882
82.848	0.948 54.964	1.201 83.067
ENSMUSG00000037188	Grhl3 protein_coding	0.732 48.939	1.524
84.999	NA	NA	1.094 93.132
ENSMUSG00000037190	Cyb561d2	protein_coding	0.948 83.263
1.104 83.104	0.957 55.9	0.987 96.183
ENSMUSG00000037196	Pacrg protein_coding	1.258 32.676	1.188
97.263	0.671 22.864	0.825 86.991
ENSMUSG00000037197	Rbm17 protein_coding	1.051 17.492	0.944
31.966	1.007 78.864	1.019 76.831
ENSMUSG00000037202	Prf1	protein_coding	0.935 74.689	1.519
62.898	0.702 7.647 0.945 87.735
ENSMUSG00000037204	9430023L20Rik	protein_coding	0.868 4.115
0.958 66.651	1.202 7.024 1.033 81.56
ENSMUSG00000037206	Islr	protein_coding	1.125 34.439	1.005
97.263	0.881 60.233	1.011 90.177
ENSMUSG00000037210	Fam193a	protein_coding	1.027 53.809
0.961 80.052	1.017 80.899	1.003 91.872
ENSMUSG00000037211	Spry1 protein_coding	0.963 61.334	1.012
96.138	1.028 69.728	1.038 94.904
ENSMUSG00000037214	Thap1 protein_coding	0.954 65.106	0.983
97.263	1.068 64.024	0.97	84.29
ENSMUSG00000037216	Lipt1 protein_coding	0.887 59.674	NA	NA
1.034 81.492	0.998 92.31
ENSMUSG00000037217	Syn1	protein_coding	1.379 1.323 0.749 65.819
0.966 67.029	1.193 72.616
ENSMUSG00000037221	Mospd3	protein_coding	0.855 3.325 1.077
71.98 1.085 60.104	1.042 94.904
ENSMUSG00000037224	Zfyve28	protein_coding	1.073 65.284

0.899 82.503 1.035 71.615 1.222 50.452
ENSMUSG00000037225 Fgf2 protein_coding 0.922 73.585 1.151
79.864 0.94 35.815 1.184 70.624
ENSMUSG00000037234 Hook3 protein_coding 0.936 11.103 0.973
96.216 1.101 8.93 0.991 94.297
ENSMUSG00000037235 Mxd4 protein_coding 1.005 86.474 1.041
87.037 0.957 58.775 1.01 95.273
ENSMUSG00000037236 Matr3 protein_coding 1.165 0 0.92 47.716
0.936 3.428 0.989 95.72
ENSMUSG00000037239 Spred3 protein_coding 0.954 43.167
0.912 93.046 1.151 42.065 0.957 90.289
ENSMUSG00000037242 Clic4 protein_coding 1.046 25.735 NA NA
0.962 42.997 1.029 89.498
ENSMUSG00000037243 Zfp692 protein_coding 0.905 1.031 0.925
74.483 1.195 5.864 1.012 89.72
ENSMUSG00000037246 H2-M10.5 protein_coding 1 81.657 1
95.504 1 87.37 1 93.457
ENSMUSG00000037247 Pldi lincRNA 1 85.728 NA NA 1
87.898 1 95.72
ENSMUSG00000037251 Pomk protein_coding 1.059 7.799 0.986 95.708
0.958 7.742 1.079 74.607
ENSMUSG00000037253 Mex3c protein_coding 1.033 24.147 1.11
71.98 0.869 5.884 0.938 84.29
ENSMUSG00000037254 Itih2 protein_coding 0.904 86.918 0.519
27.551 2.127 20.556 0.715 95.832
ENSMUSG00000037257 Aagab protein_coding 0.978 77.615 1.002
95.708 1.018 76.498 0.999 92.263
ENSMUSG00000037259 Dzank1 protein_coding 1.304 3.825 0.897
86.071 0.855 13.848 0.855 91.078
ENSMUSG00000037260 Hgsnat protein_coding 1.086 7.397 1.022
88.923 0.901 2.453 1.008 83.067
ENSMUSG00000037262 Kin protein_coding 0.981 35.449 0.933
82.848 1.096 14.131 0.975 95.273
ENSMUSG00000037263 1700055N04Rik protein_coding 0.532 8.751 NA
NA 1.806 11.932 1.008 83.067
ENSMUSG00000037266 D4Wsu53e protein_coding 0.967 27.968
0.947 69.611 1.087 9.334 0.905 86.699
ENSMUSG00000037270 4932438A13Rik protein_coding 0.989 58.56
1.006 94.48 1.005 50.375 0.993 94.19
ENSMUSG00000037275 Gemin5 protein_coding 1.046 32.058
0.983 91.532 0.975 59.726 1.051 69.918
ENSMUSG00000037278 Tmem97 protein_coding 1.212 2.569 0.932
81.284 0.882 11.64 1.106 86.118
ENSMUSG00000037279 Ovol2 protein_coding 0.771 18.379 1.151
83.104 1.127 74.686 1.039 96.086
ENSMUSG00000037280 Galnt6 protein_coding 1.374 10.858
0.946 90.378 0.769 34.592 1.103 50.452
ENSMUSG00000037286 Stag1 protein_coding 1.072 24.958 0.928
81.731 1.003 71.388 1.03 86.445
ENSMUSG00000037287 Tbcel protein_coding 1.07 11.638 0.956
71.889 0.975 61.288 0.985 91.291
ENSMUSG00000037295 Ldlrap1 protein_coding 0.916 10.143 1.04
82.904 1.047 64.024 1.016 91.372
ENSMUSG00000037296 Lsm1 protein_coding 1.089 20.444 0.93

69.611	0.99	85.21	0.974	88.552
ENSMUSG00000037300	Ttc13	protein_coding	1.003	83.333	0.928
79.475	1.076	30.561	0.992	91.269
ENSMUSG00000037306	Man1c1	protein_coding	1.298	0	0.893	75.4
0.859	8.382	0.922	84.29
ENSMUSG00000037307	Banf2	protein_coding	1	85.655	1
95.211	1	65.235	1	92.381
ENSMUSG00000037313	Tacc3	protein_coding	1.172	46.875	0.816
53.79	1.044	84.626	1.351	60.31
ENSMUSG00000037315	Phf16	protein_coding	0.989	84.263	1.007
96.652	1.006	82.575	0.945	86.699
ENSMUSG00000037316	Bag4	protein_coding	1.017	74.147	1.037
97.097	0.945	78.642	0.829	77.245
ENSMUSG00000037318	Traf3ip3	protein_coding	1.048	47.174
1.161	84.007	0.824	11.116	0.938	90.674
ENSMUSG00000037321	Tap1	protein_coding	1.065	24.689	1.099
79.367	0.854	9.563	0.976	95.777
ENSMUSG00000037325	Bbs7	protein_coding	1.056	26.136	0.985
92.137	0.958	33.689	1.084	93.132
ENSMUSG00000037326	Capn15	protein_coding	0.967	50.768
1.046	90.68	0.988	81.032	0.998	92.675
ENSMUSG00000037331	Larp1	protein_coding	1.02	36.195	1.008
96.407	0.97	42.376	1.048	91.492
ENSMUSG00000037335	Hand1	protein_coding	1	84.534	1
96.268	NA	NA	1	96.287
ENSMUSG00000037336	Mfsd2b	protein_coding	1.043	77.113
0.868	67.165	1.102	23.334	0.775	83.724
ENSMUSG00000037337	Map4k1	protein_coding	1.317	39.075
0.726	30.872	1.041	73.759	1.034	87.088
ENSMUSG00000037339	Fam53a	protein_coding	0.956	30.877
0.933	59.946	1.119	6.693	0.988	96.183
ENSMUSG00000037341	Slc9a7	protein_coding	1.116	39.295
0.684	74.633	1.312	78.155	0.829	85.493
ENSMUSG00000037343	Taf2	protein_coding	1.042	43.019	1.023
97.129	0.935	18.122	0.989	96.071
ENSMUSG00000037344	Slc12a9	protein_coding	0.824	8.122	1.064
90.092	1.138	29.819	0.975	85.493
ENSMUSG00000037346	Hrh4	protein_coding	1.319	53.228	1.566
97.097	0.485	57.446	0.761	89.475
ENSMUSG00000037347	Chst7	protein_coding	0.97	73.292	1.038
85.756	0.989	61.743	0.938	85.636
ENSMUSG00000037348	Paqr7	protein_coding	0.962	60.003	1.098
79.367	0.949	29.451	1.227	31.133
ENSMUSG00000037349	Nudt22	protein_coding	0.918	48.939
1.153	83.104	0.944	63.919	1.028	78.948
ENSMUSG00000037351	Actr1b	protein_coding	0.937	11.924
1.031	87.037	1.032	45.449	1.078	50.74
ENSMUSG00000037353	Letmd1	protein_coding	0.891	25.551
1.088	83.104	1.031	75.009	1.044	95.273
ENSMUSG00000037355	Uvssa	protein_coding	1.002	83.658	0.97
83.876	1.026	61.841	1.025	96.183
ENSMUSG00000037358	4930578C19Rik	protein_coding	1.165	6.61
0.869	84.594	0.984	63.075	0.943	83.724
ENSMUSG00000037361	0610009D07Rik	protein_coding	1.084	57.38

0.979 83.321 0.945 78.642 1.006 82.85
ENSMUSG00000037362 Nov protein_coding 1.388 32.676 0.813
72.399 0.888 85.134 0.592 87.651
ENSMUSG00000037363 Letm2 protein_coding 0.859 36.823 0.963
91.661 1.204 27.475 1.002 95.273
ENSMUSG00000037364 Srrt protein_coding 0.85 0.987 NA NA
1.172 5.864 1.062 42.736
ENSMUSG00000037366 Pafah2 protein_coding 0.958 21.393 0.93
65.819 1.124 10.079 1.016 95.74
ENSMUSG00000037369 Kdm6a protein_coding 1.027 84.168 1.002
94.89 0.972 70.791 0.953 83.724
ENSMUSG00000037370 Enpp1 protein_coding 1.011 85.77 NA NA
1.011 86.144 1.018 96.183
ENSMUSG00000037373 Ctbp1 protein_coding 0.951 9.341 1.043 83.104
1.009 57.286 1.04 64.513
ENSMUSG00000037375 Hhat protein_coding 1.403 0.664 1.013 93.654
0.703 0.879 0.991 96.248
ENSMUSG00000037376 Trmt6 protein_coding 1.001 73.734 0.99
94.48 1.011 85.464 1.144 31.133
ENSMUSG00000037379 Spon2 protein_coding 1.315 14.205 1.072
83.104 0.708 6.68 1.187 46.412
ENSMUSG00000037386 Rims2 protein_coding 0.971 84.865 0.304
37.187 3.389 8.586 1.091 92.12
ENSMUSG00000037393 Nmur2 protein_coding 1 86.658 NA NA
1 84.724 1 95.559
ENSMUSG00000037395 Rcor3 protein_coding 1.039 43.654 0.983
94.89 0.98 41.662 0.972 83.724
ENSMUSG00000037400 Atp11b protein_coding 1.057 2.651 0.977
68.818 0.969 40.794 0.99 95.92
ENSMUSG00000037405 Icam1 protein_coding 1.036 51.189 0.954
92.137 1.014 71.004 0.934 85.493
ENSMUSG00000037406 Htra4 protein_coding 1.493 0.577 0.855 81.284
0.782 14.262 1.914 43.377
ENSMUSG00000037408 Cnnm4 protein_coding 1.065 39.295 1.083
88.533 0.864 0.879 0.97 87.218
ENSMUSG00000037410 Tbc1d2b protein_coding 1.311 0 0.984
85.793 0.773 0.72 0.986 96.071
ENSMUSG00000037411 Serpine1 protein_coding 3.322 2.651 0.57
56.781 0.529 23.607 1.145 78.379
ENSMUSG00000037415 Ranbp10 protein_coding 0.989 74.689
1.018 90.116 0.995 87.898 0.997 91.979
ENSMUSG00000037416 Dmxl1 protein_coding 0.954 52.258 0.911
85.116 1.153 37.369 1.044 94.654
ENSMUSG00000037419 Endod1 protein_coding 1.118 0.461 0.982
93.654 0.913 2.563 0.961 88.053
ENSMUSG00000037424 NA NA NA NA 1 95.088 1
87.103 1 86.991
ENSMUSG00000037426 Depdc5 protein_coding 1.038 14.746
1.092 82.904 0.88 4.338 0.997 95.832
ENSMUSG00000037432 Fer1l5 pseudogene 0.835 15.76 0.954 96.843
1.259 32.173 0.848 83.724
ENSMUSG00000037434 Slc30a1 protein_coding 0.943 43.816
0.936 82.073 1.13 58.915 1.015 94.654
ENSMUSG00000037437 Adam32 protein_coding 0.678 34.795

0.916 95.504 1.606 47.512 0.685 85.493
ENSMUSG00000037438 Gm9763 pseudogene 1 78.224 1
94.367 1 86.144 1 93.612
ENSMUSG00000037440 Vnn1 protein_coding 1.965 1.216 0.634 60.678
0.803 56.822 1.223 84.246
ENSMUSG00000037443 Cep85 protein_coding 1.011 86.57 0.973 91.532
1.014 74.082 0.933 86.699
ENSMUSG00000037446 Tulp1 protein_coding 0.985 84.977 0.908
90.378 1.118 55.81 0.778 84.29
ENSMUSG00000037447 Arid5a protein_coding 0.864 27.968
1.049 83.104 1.102 55.512 1.151 93.016
ENSMUSG00000037451 Slc22a20 protein_coding 1 69.777 1
96.013 1 84.308 1 96.287
ENSMUSG00000037455 Slc18b1 protein_coding 0.834 1.031 1.124
61.032 1.069 55.81 0.973 92.31
ENSMUSG00000037458 Azin1 protein_coding 0.99 80.51 1.087 61.032
0.931 6.515 1.094 71.305
ENSMUSG00000037461 Ints7 protein_coding 0.998 80.251 1.077
82.904 0.926 39.755 0.997 96.183
ENSMUSG00000037463 Fbxo27 protein_coding 1.175 20.659 0.99
97.263 0.86 35.264 0.95 96.184
ENSMUSG00000037465 Klf10 protein_coding 0.979 71.763 0.887
72.399 1.151 24.07 1.032 96.183
ENSMUSG00000037466 4930427A07Rik protein_coding 1.032 83.968
1.039 94.848 0.934 81.62 1.524 83.067
ENSMUSG00000037470 Uggt1 protein_coding 1.04 81.125 0.968
94.96 0.991 86.346 1.041 46.412
ENSMUSG00000037474 Dtl protein_coding 1.216 63.126 0.867
94.96 0.947 82.685 1.407 55.054
ENSMUSG00000037475 Thoc2 protein_coding 0.899 17.031 0.918
66.09 1.209 8.339 0.988 94.19
ENSMUSG00000037477 Tbx10 protein_coding 1 85.283 1
96.843 1 73.598 1 91.625
ENSMUSG00000037482 Erv3 protein_coding 1 75.298 1
97.263 1 84.896 1 93.734
ENSMUSG00000037486 Asxl2 protein_coding 0.951 30.877 NA NA
1.045 52.574 1.031 95.273
ENSMUSG00000037487 Ubr5 protein_coding 0.931 57.014 0.941
60.678 1.142 17.662 1.087 88.197
ENSMUSG00000037490 Slc2a12 protein_coding 1.344 5.932 1.216
82.904 0.613 2.889 0.857 85.493
ENSMUSG00000037492 Zmat4 protein_coding 1.718 15.972 1.74
83.104 0.334 6.202 0.794 92.181
ENSMUSG00000037493 Cib2 protein_coding 0.884 14.403 0.999
93.899 1.129 38.038 0.827 83.724
ENSMUSG00000037499 Nenf protein_coding 0.954 20.116 0.998
91.584 1.052 62.648 1.006 96.358
ENSMUSG00000037503 Fam168b protein_coding 1.099 0 1.009
90.378 0.899 4.759 0.949 83.724
ENSMUSG00000037509 Arhgef4 protein_coding 0.948 78.538
1.102 96.843 0.954 84.724 0.981 96.183
ENSMUSG00000037513 Samd4b protein_coding 1.182 1.412 1.008
91.776 0.841 0.72 1.004 92.31
ENSMUSG00000037514 Pank2 protein_coding 1.024 46.533 1.035

86.387 0.945 15.904 0.954 83.952
ENSMUSG00000037519 Ppfia1 protein_coding 0.95 39.174 0.95
62.692 1.11 25.93 1.038 86.445
ENSMUSG00000037523 Mavs protein_coding 0.995 59.674 1.082
71.617 0.925 23.607 1.046 82.85
ENSMUSG00000037525 Bcdin3d protein_coding 1.007 85.655
0.883 69.611 1.125 24.791 1.041 78.948
ENSMUSG00000037526 Atg14 protein_coding 1.032 31.732 0.933
44.268 1.038 43.129 0.942 85.493
ENSMUSG00000037531 Mrpl47 protein_coding 0.892 11.384
1.128 69.228 0.993 88.657 0.993 93.722
ENSMUSG00000037533 Rapgef6 protein_coding 1.01 83.523
0.954 81.02 1.041 46.206 0.96 84.29
ENSMUSG00000037535 1700021A07Rik protein_coding 1 83.968
1 91.83 1 86.084 1 94.904
ENSMUSG00000037536 Fbxo34 protein_coding 1.056 46.097 0.94
68.818 1.007 70.623 0.987 95.273
ENSMUSG00000037537 H2-M11 protein_coding 1.156 78.428
1.113 93.146 0.776 37.906 1.072 90.397
ENSMUSG00000037541 Shank2 protein_coding 0.733 1.701 1.049
96.216 1.302 5.864 0.991 91.372
ENSMUSG00000037542 Aldh8a1 protein_coding 0.533 86.918 NA
NA 1.425 86.258 1.293 93.132
ENSMUSG00000037544 Dlgap5 protein_coding 1.566 17.336
0.759 65.165 0.84 60.233 1.162 87.7
ENSMUSG00000037548 H2-DMb2 protein_coding 1.283 16.959
0.892 85.95 0.875 84.434 0.984 96.183
ENSMUSG00000037552 Plekhg2 protein_coding 1.034 85.728
0.999 92.931 0.964 53.224 1.058 57.075
ENSMUSG00000037553 Zdhhc18 protein_coding 0.985 53.679
1.072 60.309 0.947 18.965 0.928 83.724
ENSMUSG00000037563 Rps16 protein_coding 0.976 63.126 1.029
93.654 0.998 84.434 1.033 95.74
ENSMUSG00000037568 Vash2 protein_coding 1.211 39.075 1.05
90.29 0.787 30.024 1.029 86.445
ENSMUSG00000037570 Mcrs1 protein_coding 0.92 14.403 1.068
82.904 1.02 44.361 1.029 59.208
ENSMUSG00000037572 Wdhd1 protein_coding 1.154 27.32 0.982 88.704
0.88 64.778 1.152 61.984
ENSMUSG00000037573 Tob1 protein_coding 0.922 39.174 1.086
90.29 0.997 86.666 1.008 96.192
ENSMUSG00000037577 Ephx3 protein_coding 0.803 32.168 1.151
82.76 1.086 84.83 1.05 74.047
ENSMUSG00000037578 Pkd2l1 protein_coding 2.52 20.799 NA
NA 0.36 14.262 1.003 96.086
ENSMUSG00000037579 Kcnh3 protein_coding 1.048 86.112 1.241
97.263 0.765 77.704 0.634 86.699
ENSMUSG00000037580 Gch1 protein_coding 1.135 12.855 0.867
70.29 1.014 68.82 1.118 78.948
ENSMUSG00000037583 Nr0b2 protein_coding 1 85.092 1
82.671 1 86.666 1 95.676
ENSMUSG00000037593 BC030499 protein_coding 0.775 44.787
0.908 94.597 1.422 36.388 1.115 94.904
ENSMUSG00000037594 BC022687 protein_coding 0.854 17.031

1.074 93.529 1.085 45.208 1.041 96.183
ENSMUSG00000037600 1810019J16Rik protein_coding 0.82 26.77
0.999 96.138 1.224 32.776 1.008 95.92
ENSMUSG00000037601 Nme1 protein_coding 0.964 33.745 NA NA
1.118 36.388 1.136 37.634
ENSMUSG00000037603 Gm5519 protein_coding 1 79.709 1
96.138 1 82.31 1 95.74
ENSMUSG00000037605 Lphn3 protein_coding 1.082 50.627 1.064
91.776 0.868 23.607 0.871 83.724
ENSMUSG00000037606 Osbpl5 protein_coding 0.903 0.987 1.067
71.617 1.039 23.454 1.028 78.948
ENSMUSG00000037608 Bclaf1 protein_coding 1.066 35.024
0.965 52.266 0.973 88.693 1.021 72.369
ENSMUSG00000037610 Kcnmb2 protein_coding 1 69.715 1
91.361 1 69.825 1 92.381
ENSMUSG00000037613 Tnfrsf23 protein_coding 1.234 26.136
1.023 96.216 0.794 18.539 1.159 74.047
ENSMUSG00000037617 Spag1 protein_coding 1.001 72.743 1.062
96.948 0.937 81.492 1.082 40.894
ENSMUSG00000037621 Atoh8 protein_coding 0.999 54.176 0.952
82.671 1.05 31.479 0.88 93.045
ENSMUSG00000037622 Wdtc1 protein_coding 0.959 83.09 1.022 96.984
1.021 85.757 0.971 83.724
ENSMUSG00000037624 Kcnk2 protein_coding 1.164 44.334 1.076
81.127 0.8 19.765 0.874 84.29
ENSMUSG00000037625 Cldn11 protein_coding 1.44 44.061
1.378 71.98 0.504 7.742 3.257 71.305
ENSMUSG00000037628 Cdkn3 protein_coding 2.296 1.846 0.96 96.138
0.455 9.494 1.287 93.156
ENSMUSG00000037636 Slc25a43 protein_coding 0.811 27.664
1.191 93.994 1.033 55.512 1.062 83.067
ENSMUSG00000037638 Zbtb42 protein_coding 0.887 18.795 0.98
95.196 1.151 63.801 0.964 85.493
ENSMUSG00000037640 Zfp60 protein_coding 1.035 27.32 0.938 68.818
1.032 69.002 0.969 92.181
ENSMUSG00000037643 Prkci protein_coding 0.948 14.427 1.017
93.899 1.041 29.777 0.922 83.724
ENSMUSG00000037646 Vps13b protein_coding 0.989 79.93 0.941
86.604 1.075 26.179 0.996 86.699
ENSMUSG00000037649 H2-DMa protein_coding 1.17 7.842 1.09
93.654 0.782 72.269 0.837 85.493
ENSMUSG00000037652 Phc3 protein_coding 0.938 42.361 0.885
63.912 1.199 15.049 0.893 83.952
ENSMUSG00000037653 Kctd8 protein_coding 0.788 42.034 0.906
96.847 1.394 62.237 1.659 86.118
ENSMUSG00000037656 Slc20a2 protein_coding 0.852 2.009 0.97
97.155 1.213 8.466 0.998 83.067
ENSMUSG00000037660 Gdf7 protein_coding 0.553 7.887 2.012 80.775
0.901 65.235 1.258 74.047
ENSMUSG00000037661 Gpr160 protein_coding 1.107 29.536
0.892 87.313 1.009 88.601 0.958 85.493
ENSMUSG00000037664 Cdkn1c protein_coding 0.841 5.946 0.934
66.09 1.267 8.007 1.078 96.192
ENSMUSG00000037669 1110057K04Rik protein_coding 1.082 57.949

1.098 67.843 0.843 6.751 0.973 93.627
ENSMUSG00000037674 Rfx7 protein_coding 0.95 37.531 1.015
95.504 1.038 50.091 0.931 85.493
ENSMUSG00000037679 Inf2 protein_coding 0.813 3.07 0.933 85.418
1.316 7.24 1.004 95.273
ENSMUSG00000037681 Esyt3 protein_coding 0.974 79.853 NA NA
NA NA 1.05 95.273
ENSMUSG00000037683 Armc3 protein_coding 0.981 65.622 1.06
96.407 0.962 84.961 0.996 95.74
ENSMUSG00000037685 Atp8a1 protein_coding 1.02 63.385
0.884 49.266 1.109 39.471 1.02 93.685
ENSMUSG00000037686 Aspg protein_coding 0.652 18.138 NA NA
1.373 34.807 0.716 86.991
ENSMUSG00000037689 Tmem247 protein_coding 1 67.991 1
96.407 1 84.071 1 96.192
ENSMUSG00000037692 Ahdc1 protein_coding 1.019 85.129 1.056
82.103 0.93 23.86 0.99 89.827
ENSMUSG00000037697 Ddhd1 protein_coding 1.227 0.664 0.973 91.83 0.84
5.453 0.921 83.724
ENSMUSG00000037703 Lzts3 protein_coding 1.011 85.77 1.031 82.904
0.956 52.972 1.068 80.497
ENSMUSG00000037705 Tecta protein_coding 1.724 66 0.257 58.64
2.259 35.441 0.654 91.625
ENSMUSG00000037706 Cd81 protein_coding 1.01 75.196 1.036
82.809 0.953 30.827 1.002 95.273
ENSMUSG00000037708 BC061194 protein_coding 0.604 60.707
0.744 92.474 2.213 43.307 0.799 93.612
ENSMUSG00000037709 Fam13a protein_coding 0.916 54.176
0.803 71.889 1.361 9.86 1 96.183
ENSMUSG00000037710 Cisd1 protein_coding 0.978 80.51 0.958 72.399
1.07 37.764 0.896 83.724
ENSMUSG00000037712 Fermt2 protein_coding 1.106 24.147
0.997 96.138 0.905 30.024 0.968 96.139
ENSMUSG00000037716 Ccdc33 protein_coding 1.128 46.533
0.856 96.001 1.032 88.693 0.561 94.174
ENSMUSG00000037720 Tmem33 protein_coding 0.974 67.477 NA
NA 1.049 76.225 0.971 94.19
ENSMUSG00000037722 Gnpnat1 protein_coding 1.05 64.956
0.989 95.504 0.962 52.658 1.057 95.74
ENSMUSG00000037725 Ckap2 protein_coding 1.427 20.659 0.744
55.265 0.94 82.903 1.395 68.696
ENSMUSG00000037727 Avp protein_coding 1 78.054 NA NA
1 87.744 1 94.468
ENSMUSG00000037730 Mynn protein_coding 1.075 14.746 0.975
87.147 0.954 56.365 0.996 87.331
ENSMUSG00000037731 Themis2 protein_coding 1.383 0 0.898
79.475 0.805 9.347 1.084 59.394
ENSMUSG00000037735 2810032G03Rik protein_coding 1.081 80.03
1.129 88.092 0.817 45.093 0.987 92.903
ENSMUSG00000037736 Limch1 protein_coding 1.095 18.635
0.905 42.923 1.007 68.643 0.966 87.542
ENSMUSG00000037738 Nek5 protein_coding 1.412 48.462 0.797
89.996 0.89 69.974 0.723 84.29
ENSMUSG00000037740 Mrps26 protein_coding 0.965 11.232

1.058 90.895 0.977 76.923 0.927 83.724
ENSMUSG00000037742 Eef1a1 protein_coding 1.186 0.461 0.885
8.462 0.954 39.079 0.969 95.405
ENSMUSG00000037747 Phyhipl protein_coding 1.228 46.875
1.155 87.609 0.705 33.402 1.059 95.559
ENSMUSG00000037750 Fam222b protein_coding 1.032 77.615
1.068 71.98 0.909 31.887 0.866 83.724
ENSMUSG00000037752 Xkr8 protein_coding 1.025 35.499 1.113
71.617 0.871 3.432 1.006 94.904
ENSMUSG00000037754 Ppp1r16b protein_coding 0.852 38.827
1.195 76.634 0.984 87.744 0.902 83.724
ENSMUSG00000037759 Ptger2 protein_coding 0.973 79.165
0.838 67.739 1.223 21.522 0.868 84.29
ENSMUSG00000037761 Actr5 protein_coding 0.921 12.084 1.033
93.27 1.052 38.099 1.004 94.19
ENSMUSG00000037762 Slc16a9 protein_coding 1.081 54.717
0.803 62.692 1.147 41.6 1.004 91.872
ENSMUSG00000037771 Slc32a1 protein_coding 1 79.93 1
96.68 1 84.961 1 95.832
ENSMUSG00000037772 Mrpl23 protein_coding 0.986 84.661
1.014 85.756 0.997 71.999 1.062 90.177
ENSMUSG00000037773 Pced1a protein_coding 1.008 66.272
0.989 74.483 1.003 71.615 0.98 93.261
ENSMUSG00000037780 Mbl1 protein_coding 1 86.006 1
89.713 1 84.961 1 93.457
ENSMUSG00000037784 Dzip1l protein_coding 0.945 36.259 1.01
96.001 1.042 52.8 1.005 96.086
ENSMUSG00000037787 Apopt1 protein_coding 0.885 3.07 0.989
92.062 1.14 18.797 1.012 96.086
ENSMUSG00000037788 Vopp1 protein_coding 1.134 29.596 NA NA
0.885 31.135 1.061 83.276
ENSMUSG00000037790 Defb7 protein_coding 1 84.865 1
94.48 1 81.29 1 94.99
ENSMUSG00000037791 Phf12 protein_coding 0.957 10.143 1.038
83.104 1.008 53.449 0.984 83.724
ENSMUSG00000037795 N4bp2 protein_coding 0.951 21.957 0.921
95.329 1.143 32.524 1.041 95.74
ENSMUSG00000037798 Mat1a protein_coding 1.506 59.806 0.733
85.418 0.908 84.229 0.761 91.105
ENSMUSG00000037801 Iqch protein_coding 0.204 7.218 0.882 94.767
5.549 27.431 2.667 69.404
ENSMUSG00000037805 Rpl10a protein_coding 1.008 42.134
0.903 20.174 1.094 34.727 1.048 76.831
ENSMUSG00000037808 Fam76b protein_coding 1.033 67.991
0.868 54.245 1.114 24.985 0.989 86.699
ENSMUSG00000037813 D630003M21Rik protein_coding 1.133 80.688
0.814 75.345 1.085 51.243 1.086 82.85
ENSMUSG00000037814 Larp1b protein_coding 0.882 6.822 1.116
61.943 1.017 85.464 1.045 87.134
ENSMUSG00000037815 Ctnna1 protein_coding 0.994 47.266
0.966 64.123 1.045 8.209 1.026 70.539
ENSMUSG00000037816 Fbxw17 protein_coding 0.989 76.686
1.145 69.228 0.885 41.154 1.494 67.691
ENSMUSG00000037818 3110057O12Rik protein_coding 0.969 54.983

0.972 91.661 1.057 42.199 0.962 92.675
ENSMUSG00000037820 Tgm2 protein_coding 0.834 62.888 1.289
82.904 0.93 59.922 1.817 40.024
ENSMUSG00000037822 Smim14 protein_coding 1.048 28.098
0.928 71.889 1.029 84.896 0.966 83.724
ENSMUSG00000037824 Tspan14 protein_coding 0.98 78.744
1.014 97.155 1.005 86.258 0.956 85.493
ENSMUSG00000037826 Ppm1k protein_coding 1.007 86.333 0.959
87.857 1.039 64.305 0.843 83.724
ENSMUSG00000037827 Gm5884 protein_coding 1 67.608 1
95.088 1 59.383 1 96.358
ENSMUSG00000037833 Sh2d4b protein_coding 1.388 36.124 0.71
73.766 1.01 84.071 1.106 91.492
ENSMUSG00000037845 Fdxacb1 protein_coding 0.993 83.823
1.047 96.847 0.963 88.349 1.023 96.183
ENSMUSG00000037846 Rtkn2 protein_coding 1.499 3.893 0.98 92.631
0.677 13.551 0.921 91.291
ENSMUSG00000037847 Nmrk1 protein_coding 1.05 22.871 1.05
95.329 0.902 64.427 0.938 95.877
ENSMUSG00000037851 Iars protein_coding 1.016 79.853 1.079
79.261 0.91 22.009 1.116 57.075
ENSMUSG00000037852 Cpe protein_coding 1.101 20.032 0.948
89.277 0.954 33.402 0.938 88.053
ENSMUSG00000037855 Zfp365 protein_coding 1.092 58.709
1.219 67.843 0.748 2.817 1.093 81.901
ENSMUSG00000037857 Nufip2 protein_coding 1.039 55.462
1.013 83.104 0.946 11.64 0.965 84.19
ENSMUSG00000037860 Aim2 protein_coding 1.107 39.416 0.749
55.71 1.203 57.907 1.004 93.132
ENSMUSG00000037868 Egr2 protein_coding 1.002 85.655 1.01
96.216 0.985 84.83 1.747 31.133
ENSMUSG00000037872 Darc protein_coding 1.276 3.498 0.965 96.216
0.812 10.934 1.044 80.324
ENSMUSG00000037876 Jmjd1c protein_coding 0.879 47.677
0.987 81.731 1.15 15.411 1.092 83.067
ENSMUSG00000037884 1700017G19Rik lincRNA 1 83.823 1
94.887 1 70.534 1 96.183
ENSMUSG00000037885 Stk35 protein_coding 0.857 19.365 1.031
96.843 1.13 55.81 1.085 71.305
ENSMUSG00000037887 Dusp8 protein_coding 1.05 51.189 1.184
83.104 0.805 11.314 1.128 92.389
ENSMUSG00000037890 Wdr19 protein_coding 0.951 54.87 1.06 90.053
0.993 44.515 0.864 84.29
ENSMUSG00000037892 Pcdh18 protein_coding 1.033 70.121
1.216 81.039 0.797 10.003 0.865 86.699
ENSMUSG00000037894 H2afz protein_coding 1.018 63.534 0.967
86.728 1.019 81.62 1.078 88.755
ENSMUSG00000037896 Rcor1 protein_coding 1.06 2.723 NA NA
0.933 16.949 0.968 83.724
ENSMUSG00000037902 Sirpa protein_coding 1.506 0 0.98 93.574
0.679 0.72 1.092 44.882
ENSMUSG00000037904 Ankrd9 protein_coding 1.013 85.832 NA
NA 1.034 86.727 1.095 79.713
ENSMUSG00000037905 Bri3bp protein_coding 1.054 50.377

1.012 95.088 0.935 65.409 0.881 84.29
ENSMUSG00000037907 Ankrd13b protein_coding 0.751 18.379
0.945 87.322 1.407 12.16 1.166 93.734
ENSMUSG00000037910 1700018B24Rik protein_coding 1 83.333
1 95.211 1 80.899 1 96.358
ENSMUSG00000037913 Tmem156 protein_coding 1.968 48.462
0.705 95.35 0.72 52.972 0.688 96.086
ENSMUSG00000037916 Ndufv1 protein_coding 0.873 0.987 1.037
96.378 1.102 11.518 1.052 87.134
ENSMUSG00000037921 Ddx60 protein_coding 1.34 1.079 1.058 97.155
0.702 14.583 1.082 67.691
ENSMUSG00000037922 Bank1 protein_coding 1.821 0 0.872 72.576
0.631 11.64 1.221 50.452
ENSMUSG00000037924 Olfr16 protein_coding 1 79.277 1
97.288 1 67.339 1 92.389
ENSMUSG00000037926 Ssh2 protein_coding 1.019 78.299 0.85
65.819 1.148 17.412 1.026 93.156
ENSMUSG00000037933 Bicd2 protein_coding 0.908 16.02 1.1 85.378
0.997 84.626 0.969 92.748
ENSMUSG00000037935 Smarce1 protein_coding 1.001 81.823
0.954 90.744 1.048 63.301 1.022 60.968
ENSMUSG00000037936 Scarb1 protein_coding 1.134 33.099
1.076 83.104 0.819 5.628 0.922 83.724
ENSMUSG00000037938 Chchd5 protein_coding 1.095 27.545
0.888 67.513 1.028 84.308 0.934 83.724
ENSMUSG00000037940 Inpp4b protein_coding 1.152 58.896
0.835 87.452 1.039 84.229 0.931 93.584
ENSMUSG00000037942 Crp protein_coding 2.104 51.826 0.403
70.29 1.176 73.428 0.925 90.865
ENSMUSG00000037944 Ccr7 protein_coding 0.582 22.92 NA NA
1.464 19.877 0.693 83.724
ENSMUSG00000037946 Fgd3 protein_coding 1.204 2.569 1.027 96.138
0.809 19.236 1.126 90.177
ENSMUSG00000037949 Ano10 protein_coding 1.126 6.76 1.083 89.014
0.822 5.701 0.968 84.29
ENSMUSG00000037953 A4gnt protein_coding 1 86.333 NA NA
1 81.857 1 86.133
ENSMUSG00000037957 Wdr20a protein_coding 1.022 42.555
0.979 93.817 0.997 69.445 0.994 93.117
ENSMUSG00000037958 Ccdc55 protein_coding 1.056 14.59 0.904
24.911 1.046 41.6 1.017 92.389
ENSMUSG00000037960 1110007C09Rik protein_coding 1.009 51.297
1.121 95.196 0.887 25.688 1.004 93.132
ENSMUSG00000037962 Fam101a protein_coding 1.09 84.397
1.035 93.863 0.883 65.902 1.105 85.577
ENSMUSG00000037965 Zc3h7a protein_coding 1.109 27.113
0.955 72.399 0.941 51.715 1.043 63.114
ENSMUSG00000037966 Ninj1 protein_coding 1.184 1.712 0.849 53.79
0.992 88.349 1.075 61.753
ENSMUSG00000037971 1110032A03Rik protein_coding 0.992 60.339
0.981 95.088 1.027 50.636 0.96 88.245
ENSMUSG00000037972 Snn protein_coding 1.07 61.725 1.082
74.663 0.86 7.509 0.953 86.991
ENSMUSG00000037973 Ccdc129 protein_coding 1.199 56.311

1.246 40.99 0.67 0.72 0.789 83.724
ENSMUSG00000037974 Muc5ac protein_coding 1 79.068 1
92.355 1 88.657 1 94.567
ENSMUSG00000037977 6430571L13Rik protein_coding 1.592 22.349
1.176 82.76 0.532 3.863 0.757 95.74
ENSMUSG00000037979 Ccdc92 protein_coding 0.695 23.556
0.817 40.463 1.763 8.586 1.187 92.263
ENSMUSG00000037982 Gm9725 lincRNA 0.887 50.519 1.142
88.136 0.983 86.602 0.719 84.29
ENSMUSG00000037984 Neurod6 protein_coding 1 84.263 1
97.155 1 87.519 1 96.042
ENSMUSG00000037989 Wnk2 protein_coding 0.824 19.023 0.946
91.361 1.282 40.575 0.974 86.991
ENSMUSG00000037990 Sh3rf3 protein_coding 1.135 53.809
1.178 91.584 0.745 26.369 0.977 93.261
ENSMUSG00000037991 Rmi2 protein_coding 1.375 69.489 0.997
95.088 0.73 80.015 1.914 31.133
ENSMUSG00000037992 Rara protein_coding 1.044 84.324 1.071
82.76 0.892 13.643 1.011 92.748
ENSMUSG00000037993 Dhx38 protein_coding 0.93 4.941 1.007 93.707
1.062 19.609 0.976 94.904
ENSMUSG00000037994 Slc9b2 protein_coding 0.867 43.495
1.265 86.793 NA NA 1.124 87.134
ENSMUSG00000037995 Igsf9 protein_coding 0.712 5.778 1.164 96.763
1.205 23.454 0.87 91.291
ENSMUSG00000037996 Slc24a2 protein_coding 1.088 71.217
1.235 96.216 0.746 28.237 0.91 91.928
ENSMUSG00000037997 Parp11 protein_coding 1.079 7.083 NA NA
0.918 19.449 1.065 87.624
ENSMUSG00000037999 Arap2 protein_coding 1.077 86.918 1.079
87.037 0.862 59.383 0.892 93.685
ENSMUSG00000038000 Acd protein_coding 1.005 85.129 0.963
96.216 1.032 88.693 0.976 95.877
ENSMUSG00000038002 Cramp1l protein_coding 1.002 77.763
1.004 95.329 0.995 76.754 0.952 83.724
ENSMUSG00000038005 2700029M09Rik protein_coding 1.083 15.178
0.963 70.29 0.961 77.037 1.117 59.394
ENSMUSG00000038007 Acer2 protein_coding 0.969 84.819 0.873
54.245 1.186 20.315 1.055 91.525
ENSMUSG00000038009 Dnajc22 protein_coding 0.605 12.181
0.921 96.013 1.799 35.673 0.789 84.29
ENSMUSG00000038010 Ccdc138 protein_coding 1.136 51.297
0.949 91.361 0.927 69.583 1.093 92.691
ENSMUSG00000038011 Dnah10 protein_coding 0.763 23.641 NA
NA 1.246 36.388 0.935 96.183
ENSMUSG00000038013 Wipf2 protein_coding 0.963 61.043 1.099
77.085 0.942 60.614 0.947 83.724
ENSMUSG00000038014 Fam120a protein_coding 1.023 27.545
0.998 97.155 0.982 27.321 1.043 43.605
ENSMUSG00000038015 Prm2 protein_coding 1 82.732 1
97.263 1 85.352 1 94.904
ENSMUSG00000038020 Rapgefl1 protein_coding 0.756 10.955
1.054 95.088 1.256 25.144 1.094 91.105
ENSMUSG00000038022 Fam188b protein_coding 1.135 54.717

0.878 91.532 0.998 88.601 1.11 86.301
ENSMUSG00000038023 Atp6v0a2 protein_coding 0.979 86.464
1.031 95.088 0.989 81.62 0.994 95.92
ENSMUSG00000038024 Dennd4c protein_coding 1.134 8.537 0.96
87.147 0.915 25.512 0.937 95.74
ENSMUSG00000038025 Phf2 protein_coding 1.016 69.089 1.001
96.847 0.982 68.584 0.981 85.493
ENSMUSG00000038026 Kcnj9 protein_coding 2.274 44.694 0.63
92.137 0.7 73.874 0.52 83.724
ENSMUSG00000038028 9630033F20Rik protein_coding 0.968 81.465
1.098 74.663 0.937 26.057 1.094 81.901
ENSMUSG00000038034 Igsf8 protein_coding 0.911 43.435 NA NA
1.015 53.134 1.028 94.904
ENSMUSG00000038037 Socs1 protein_coding 1.225 41.951 1.165
82.809 0.702 7.742 0.988 92.018
ENSMUSG00000038039 Gcc2 protein_coding 0.926 26.217 1.024
95.708 1.056 30.292 0.961 96.183
ENSMUSG00000038042 Ptpdc1 protein_coding 1.067 52.799
1.032 95.504 0.911 40.19 0.965 92.675
ENSMUSG00000038044 Cct8l1 protein_coding 2.582 5.932 0.63
75.993 0.614 22.009 0.529 85.493
ENSMUSG00000038045 Sult6b1 protein_coding 1.123 83.333 NA
NA 0.868 88.274 0.891 96.183
ENSMUSG00000038046 Rnmtl1 protein_coding 1.064 85.857 NA
NA 0.948 85.464 0.959 92.381
ENSMUSG00000038047 Haus6 protein_coding 1.07 62.354 1
90.138 0.932 64.155 1.068 72.369
ENSMUSG00000038048 Cntnap5c protein_coding 1 73.841 NA
NA 1 88.076 1 92.181
ENSMUSG00000038055 Dexi protein_coding 0.959 69.715 1.011
90.895 1.028 80.015 0.98 86.699
ENSMUSG00000038056 Kmt2c protein_coding 0.988 86.74 1.033 97.263
0.981 87.519 1.006 90.805
ENSMUSG00000038057 Dbil5 protein_coding 0.547 17.031 1.072
90.895 1.706 36.839 1.467 71.305
ENSMUSG00000038058 Nod1 protein_coding 1.003 81.125 1.134
60.309 0.88 4.963 0.951 88.505
ENSMUSG00000038059 Smim3 protein_coding 0.986 71.915 0.78
73.766 1.298 25.93 0.831 83.724
ENSMUSG00000038060 Dlec1 protein_coding 0.879 50.768 1.051
94.48 1.084 49.384 0.736 83.724
ENSMUSG00000038064 Cldn22 protein_coding 0.431 14.075
1.029 93.817 2.249 22.366 0.625 86.445
ENSMUSG00000038065 2410066E13Rik protein_coding 0.92 45.502
1.059 96.407 1.028 64.024 0.973 94.215
ENSMUSG00000038067 Csf3 protein_coding 1.194 59.806 1.641
82.904 0.511 31.135 0.864 96.183
ENSMUSG00000038068 Rnf144b protein_coding 1.209 70.121
0.781 54.06 1.06 43.842 0.938 92.851
ENSMUSG00000038069 Cdkn2aip protein_coding 1.072 13.492
0.983 97.263 0.949 3.234 0.921 83.724
ENSMUSG00000038070 Cntln protein_coding 1.161 0.355 0.993 85.95
0.868 3.432 1.036 82.091
ENSMUSG00000038072 Galnt11 protein_coding 0.942 34.572

0.959 79.475 1.108 20.556 1.049 92.52
ENSMUSG00000038074 Fkbp14 protein_coding 1.075 27.32 1.083 79.411 0.86 3.943 1.328 31.133
ENSMUSG00000038077 Kcna6 protein_coding 1.182 24.958 0.929 86.728 0.908 36.248 0.92 86.699
ENSMUSG00000038079 Tmem237 protein_coding 0.948 61.334 1.043 89.014 1.009 70.1 1.029 83.067
ENSMUSG00000038080 Kdm1b protein_coding 1.015 86.843 1.049 71.98 0.937 28.669 1.015 95.74
ENSMUSG00000038084 Opa1 protein_coding 0.984 62.158 NA NA 1.034 66.043 1.019 60.31
ENSMUSG00000038085 Cnbd2 protein_coding 0.79 12.084 1.181 83.104 1.072 35.13 0.986 96.183
ENSMUSG00000038086 Hspb2 protein_coding 0.737 8.365 1.255 72.546 1.079 73.309 1.28 68.696
ENSMUSG00000038092 Hsd3b5 protein_coding 1 80.51 NA NA 1 88.203 1 94.752
ENSMUSG00000038095 Sbno1 protein_coding 0.991 62.437 0.998 93.817 1.01 44.787 1.01 95.273
ENSMUSG00000038102 Trappc11 protein_coding 1.067 19.132 0.983 92.355 0.953 42.895 1.003 91.928
ENSMUSG00000038112 AW551984 protein_coding 1.54 1.079 1.14 95.809 0.568 5.336 0.944 84.31
ENSMUSG00000038115 Ano2 protein_coding 1.764 39.295 0.855 95.708 0.664 49.453 0.763 86.699
ENSMUSG00000038116 Phf20 protein_coding 1.012 49.517 0.924 63.619 1.071 45.035 1.027 69.708
ENSMUSG00000038119 Cdon protein_coding 1.212 3.498 0.965 90.092 0.854 1.342 0.962 83.724
ENSMUSG00000038121 Fam210a protein_coding 1.019 62.354 1.031 95.708 0.953 66.201 0.988 89.892
ENSMUSG00000038122 Tbc1d32 protein_coding 1.008 66.76 0.96 67.165 1.035 70.623 0.96 86.699
ENSMUSG00000038126 Mphosph9 protein_coding 1.063 58.38 1.043 82.904 0.901 20.751 0.945 83.724
ENSMUSG00000038127 Ccdc50 protein_coding 0.986 86.112 0.994 86.728 1.022 69.728 0.988 95.74
ENSMUSG00000038128 Camk4 protein_coding 1.066 86.112 1.038 96.68 0.9 83.607 0.933 90.289
ENSMUSG00000038132 Rbm24 protein_coding 1.397 46.097 1.228 92.931 0.58 17.381 0.869 93.261
ENSMUSG00000038135 Crygn protein_coding 0.771 78.224 1.078 95.211 1.204 86.602 1.074 90.157
ENSMUSG00000038141 Tmem181a protein_coding 1.087 27.32 0.935 77.944 0.986 76.768 0.977 96.183
ENSMUSG00000038143 Stox2 protein_coding 0.958 18.487 1.01 72.642 1.03 82.778 0.94 84.19
ENSMUSG00000038145 Snrk protein_coding 1.046 55.462 0.785 35.072 1.218 19.452 1.009 95.74
ENSMUSG00000038146 Notch3 protein_coding 1.055 44.061 0.997 96.897 0.951 49.453 0.964 86.699
ENSMUSG00000038147 Cd84 protein_coding 1.714 0 0.755 33.301 0.77 10.934 1.264 66.944
ENSMUSG00000038148 NA NA NA NA 1 93.654 NA NA

1 95.35
ENSMUSG00000038150 Ormdl3 protein_coding 0.994 84.977
0.909 90.256 NA NA 0.976 94.19
ENSMUSG00000038151 Prdm1 protein_coding 0.918 81.76 1.398 79.367
0.776 15.465 1.031 96.183
ENSMUSG00000038152 5033430I15Rik protein_coding 0.608 14.427
1.605 71.617 1.026 84.83 1.098 94.904
ENSMUSG00000038155 Gstp2 protein_coding 1.269 3.713 1.01 92.561
0.779 4.935 1.011 92.181
ENSMUSG00000038156 Spon1 protein_coding 0.98 69.408 1.402
75.612 0.73 20.712 1.947 72.992
ENSMUSG00000038160 Atg5 protein_coding 1.033 60.853 0.994
96.763 0.972 54.037 1.088 75.198
ENSMUSG00000038165 1700022A21Rik pseudogene 1 82.231 1
96.216 1 85.352 1 93.722
ENSMUSG00000038167 Plekhg6 protein_coding 0.866 21.637
1.081 86.156 1.068 33.042 0.937 85.493
ENSMUSG00000038168 Leprel1 protein_coding 1.031 60.435
0.933 66.796 1.036 80.089 0.974 92.12
ENSMUSG00000038170 Pde4dip protein_coding 1.536 10.105
1.091 73.092 0.595 0.72 0.922 95.72
ENSMUSG00000038172 Ttc39b protein_coding 1.02 79.068
0.903 55.265 1.084 32.524 0.943 86.699
ENSMUSG00000038173 Enpp6 protein_coding 1.004 81.647 1.171
83.104 0.851 60.233 1.284 60.968
ENSMUSG00000038174 Fam126b protein_coding 0.919 29.972
1.162 85.756 0.933 86.727 0.982 96.287
ENSMUSG00000038175 Mylip protein_coding 1.052 50.377 0.866
68.115 1.096 70.328 0.843 83.724
ENSMUSG00000038178 Slc43a2 protein_coding 1.231 0.577 0.913
73.094 0.886 12.41 0.998 94.654
ENSMUSG00000038179 Slamf7 protein_coding 1.021 44.334
1.255 96.843 0.779 21.431 0.842 83.724
ENSMUSG00000038180 Spag4 protein_coding 0.721 2.174 1.023 96.763
1.351 8.339 0.883 86.991
ENSMUSG00000038181 Chpf2 protein_coding 0.942 33.224 1.1
83.104 0.968 69.196 1.092 57.839
ENSMUSG00000038187 Btbd10 protein_coding 1.007 57.38 1.061
90.092 0.937 18.797 1.006 91.525
ENSMUSG00000038188 Scarf1 protein_coding 0.935 36.034
1.101 83.104 0.972 86.831 0.971 93.156
ENSMUSG00000038194 Lhb protein_coding 0.418 4.115 1.438 83.104
1.669 24.691 1.075 92.018
ENSMUSG00000038195 Rilp protein_coding 0.819 4.115 1.128 83.104
1.084 54.809 1.141 73.401
ENSMUSG00000038201 Kcna7 protein_coding 1.918 28.098 2.155
82.904 0.243 2.333 1.063 92.263
ENSMUSG00000038203 Hoxa13 protein_coding 1 73.401 NA
NA 1 78.642 1 92.466
ENSMUSG00000038204 Asb10 protein_coding 2.359 9.259 1.065 96.216
0.396 1.871 1.096 92.018
ENSMUSG00000038205 Prkab2 protein_coding 1.185 1.815 1.135
90.952 0.743 1.342 1.057 85.786
ENSMUSG00000038206 Fbxo8 protein_coding 1 75.858 0.993

96.68 1.005 85.921 0.979 83.724
ENSMUSG00000038208 Pgap3 protein_coding 1 74.147 0.953
86.728 1.05 83.925 0.965 84.29
ENSMUSG00000038212 Hiatl1 protein_coding 1.076 7.492 0.991
87.919 0.932 45.799 0.969 84.19
ENSMUSG00000038213 Tapbpl protein_coding 1.065 24.253
1.059 71.617 0.888 4.759 1.019 83.067
ENSMUSG00000038214 Bend3 protein_coding 0.842 6.952 0.961 78.877
1.233 9.567 0.994 93.685
ENSMUSG00000038215 Cep44 protein_coding 0.897 18.602 0.966
86.277 1.156 8.209 1.043 64.513
ENSMUSG00000038216 Pnmt protein_coding 1 75.671 1
95.211 1 85.675 1 86.209
ENSMUSG00000038217 Tlcd2 protein_coding 0.697 1.031 1.311 72.546
1.095 40.875 0.996 79.442
ENSMUSG00000038218 Gm9765 protein_coding 1 84.324 1
96.984 1 78.155 1 93.016
ENSMUSG00000038224 Serpinf2 protein_coding 0.84 77.497
0.929 95.708 1.282 80.015 1.328 93.627
ENSMUSG00000038225 Ccdc111 protein_coding 1.012 86.112 0.96
92.545 1.024 85.792 0.972 94.001
ENSMUSG00000038227 Hoxa9 protein_coding 0.779 2.009 1.149 77.085
1.112 25.425 0.949 90.805
ENSMUSG00000038233 Fam198a protein_coding 1.268 26.136
1.148 92.561 0.684 16.051 1.042 94.608
ENSMUSG00000038235 F11r protein_coding 0.876 3.325 1.006 96.216
1.132 8.647 0.935 84.29
ENSMUSG00000038236 Hoxa7 protein_coding 0.884 22.431 1.058
83.104 1.066 84.308 0.958 86.699
ENSMUSG00000038239 Hrc protein_coding 4.289 9.206 1.375 83.104
0.17 0.72 0.946 96.248
ENSMUSG00000038240 Pdss2 protein_coding 0.971 49.621 1.108
72.642 0.93 45.093 0.979 91.372
ENSMUSG00000038241 Cep250 protein_coding 0.887 2.822 1.005
94.102 1.124 5.964 1.039 87.886
ENSMUSG00000038244 Mical2 protein_coding 0.981 78.299
1.063 89.494 0.955 76.64 1.1 44.882
ENSMUSG00000038246 Fam50b protein_coding 1 60.339 1
86.387 1 86.084 1 93.132
ENSMUSG00000038248 Sobp protein_coding 1.154 30.282 1.037
86.387 0.832 7.809 0.956 90.177
ENSMUSG00000038250 Usp38 protein_coding 1.027 43.654 1.038
88.533 0.939 16.719 1 94.654
ENSMUSG00000038252 Ncapd2 protein_coding 0.986 84.324 0.98
91.532 1.033 86.985 1.091 78.948
ENSMUSG00000038253 Hoxa5 protein_coding 1.138 67.163 0.985
82.904 0.891 19.552 0.992 88.245
ENSMUSG00000038255 Neurod2 protein_coding 1 81.647 NA
NA 1 83.607 1 96.248
ENSMUSG00000038256 Bcl9 protein_coding 1.047 61.725 1.081
79.055 0.882 6.858 0.944 87.992
ENSMUSG00000038257 Glra3 protein_coding 1 79.709 1
91.776 1 87.519 1 96.287
ENSMUSG00000038259 Gdf5 protein_coding 0.763 18.379 1.373

72.546	0.95	87.971	0.98	86.133
ENSMUSG00000038260	Trpm4	protein_coding	0.963	57.014	0.936	74.483	1.111	25.93	0.844	84.29
ENSMUSG00000038264	Sema7a	protein_coding	0.901	23.319	1.057	95.735	1.052	52.574	0.938	95.405
ENSMUSG00000038267	Slc22a23	protein_coding	1.071	52.134	1.094	79.579	0.851	5.087	0.83	75.345
ENSMUSG00000038268	Ovca2	protein_coding	1.011	82.479	1.179	88.923	0.836	56.822	0.962	88.111
ENSMUSG00000038271	NA	NA	NA	NA	NA	NA	1.024	84.896	1.024	92.018
ENSMUSG00000038274	Fau	protein_coding	0.944	36.034	0.946	78.877	1.118	14.597	0.984	96.248
ENSMUSG00000038276	Asic3	protein_coding	0.763	58.802	0.905	85.418	1.442	56.708	0.91	85.493
ENSMUSG00000038279	Nop2	protein_coding	0.999	76.396	0.955	87.313	1.045	53.587	1.104	51.901
ENSMUSG00000038280	Ostm1	protein_coding	1.046	29.811	0.94	52.266	1.012	65.8	1.046	78.042
ENSMUSG00000038286	Bphl	protein_coding	0.969	29.21	1.047	91.643	0.988	67.823	0.911	84.19
ENSMUSG00000038290	Smg6	protein_coding	1.034	32.411	0.986	94.96	0.979	52.213	1.012	96.086
ENSMUSG00000038291	Snx25	protein_coding	1.067	66.76	0.938	87.857	0.996	85.58	1.009	96.183
ENSMUSG00000038292	Ccdc155	protein_coding	1.015	78.428	0.949	95.301	1.035	81.62	0.702	92.181
ENSMUSG00000038295	Atg9b	protein_coding	1.031	56.477	0.943	93.529	1.03	87.103	0.974	93.722
ENSMUSG00000038296	Galnt18	protein_coding	0.919	45.229	1.068	82.904	1.02	87.686	0.96	85.493
ENSMUSG00000038298	Pdzk1	protein_coding	0.569	9.863	0.933	96.407	1.884	27.047	1.033	87.134
ENSMUSG00000038299	Wdr36	protein_coding	1.135	3.434	0.998	86.277	0.882	10.934	1.017	83.067
ENSMUSG00000038300	Pth2	protein_coding	1	51.56	1	95.809	1	85.134	1	94.752
ENSMUSG00000038301	Snx10	protein_coding	0.875	30.877	1.082	94.367	1.055	52.8	1.044	96.086
ENSMUSG00000038302	Lace1	protein_coding	0.921	36.945	1.015	96.407	1.065	49.058	1.053	72.369
ENSMUSG00000038304	Cd160	protein_coding	0.675	65.869	2.435	60.309	0.61	32.703	0.577	83.724
ENSMUSG00000038305	Spats2l	protein_coding	1.076	46.097	1.022	95.735	0.909	51.443	1.004	73.597
ENSMUSG00000038311	2410017I17Rik	protein_coding	0.585	45.894	1.054	96.68	1.625	58.915	1.221	96.287
ENSMUSG00000038312	Edem2	protein_coding	1.004	75.567	0.965	84.017	1.029	48.935	0.976	94.904
ENSMUSG00000038319	Kcnh2	protein_coding	0.711	36.823	1.343	72.642	1.045	81.525	1.123	94.904
ENSMUSG00000038323	1700066M21Rik	protein_coding	1.177	2.784	1.084	85.985	0.784	0.72	0.91	83.724
ENSMUSG00000038324	Trpc4ap	protein_coding	1.02	28.927

0.992 88.704 0.989 67.962 0.993 83.724
ENSMUSG00000038327 NA NA NA NA 1 95.211 1
87.898 1 93.612
ENSMUSG00000038330 C87499 protein_coding 1 85.59 1
97.263 NA NA 1 94.608
ENSMUSG00000038331 Satb2 protein_coding 1.011 72.655 1.079
93.817 NA NA 1.06 94.752
ENSMUSG00000038332 Sesn1 protein_coding 1.145 7.083 0.835 21.315
1.047 76.498 1.013 94.904
ENSMUSG00000038335 Tsr1 protein_coding 1.093 32.238 0.918
75.993 0.997 84.669 1.081 82.091
ENSMUSG00000038342 Mlxip protein_coding 1.085 22.633 0.906
53.434 1.015 80.015 1.017 96.287
ENSMUSG00000038344 Txlng protein_coding 0.832 15.76 0.914 73.166
1.313 5.964 1.005 94.904
ENSMUSG00000038346 Zfp384 protein_coding 0.946 5.591 1.031
82.904 1.025 36.388 1.014 72.826
ENSMUSG00000038347 Tcte2 protein_coding 0.969 59.358 1.17
82.904 0.883 61.743 0.839 83.724
ENSMUSG00000038349 Plcl1 protein_coding 0.825 48.354 0.936
97.155 1.299 49.058 1.22 81.061
ENSMUSG00000038351 Sgsm2 protein_coding 0.977 64.401 1.106
83.464 0.926 7.329 1.052 93.132
ENSMUSG00000038352 Arl5c protein_coding 1.545 2.651 0.978 79.807
0.663 16.129 0.723 83.724
ENSMUSG00000038354 Ankrd35 protein_coding 0.895 34.572
1.151 75.108 0.974 84.552 1.015 96.071
ENSMUSG00000038365 Fbxo25 protein_coding 1.041 34.077
1.071 91.776 0.898 16.949 0.954 84.29
ENSMUSG00000038366 Lasp1 protein_coding 0.968 66.652 1.127
72.642 0.914 24.309 0.967 95.74
ENSMUSG00000038368 Focad protein_coding 0.965 80.146 1.047
88.533 0.99 73.874 1.095 67.169
ENSMUSG00000038369 Ncoa6 protein_coding 1.048 30.497 1.024
96.68 0.933 35.815 0.99 94.016
ENSMUSG00000038370 Pcp4l1 protein_coding 1.462 12.985
1.087 85.756 0.631 6.007 0.909 85.493
ENSMUSG00000038371 Sbf2 protein_coding 1.043 19.477 1.014
90.116 0.944 9.494 1 91.335
ENSMUSG00000038372 Gmds protein_coding 1.075 51.826 1.062
83.104 0.873 32.703 0.958 90.805
ENSMUSG00000038374 Rbm8a protein_coding 0.948 25.3 1.043 95.431
1.009 57.227 1.028 62.918
ENSMUSG00000038375 Trp53inp2 protein_coding 0.936 38.286
1.115 79.864 0.957 78.954 1.201 31.133
ENSMUSG00000038379 Ttk protein_coding 1.108 65.733 1.239
91.361 0.726 87.898 1.766 69.965
ENSMUSG00000038383 Pigu protein_coding 1.046 56.311 1.035
79.864 0.926 28.669 1.012 93.685
ENSMUSG00000038384 Setd1b protein_coding 1.031 81.657
1.078 71.222 0.902 3.428 0.91 83.724
ENSMUSG00000038387 Rras protein_coding 0.963 79.165 1.05
95.088 NA NA 0.968 84.29
ENSMUSG00000038388 Mpp6 protein_coding 1.144 14.59 0.962 85.075

0.906 40.794 1.214 0
ENSMUSG00000038390 Gpr162 protein_coding 0.951 80.688
1.009 92.474 1.041 83.719 0.908 85.493
ENSMUSG00000038393 Txnip protein_coding 0.955 83.263 0.709
23.703 1.474 5.864 0.825 83.724
ENSMUSG00000038398 Upf3a protein_coding 0.986 72.985 1.012
96.268 0.999 70.1 1.008 95.273
ENSMUSG00000038400 Pmepa1 protein_coding 1.171 7.332 1.031
95.211 0.825 9.347 1.212 52.366
ENSMUSG00000038402 Foxf2 protein_coding 0.748 78.909 1.087
96.843 1.229 85.134 1.338 92.263
ENSMUSG00000038403 Hfe2 protein_coding 4.659 7.908 1.87 71.617
0.114 0.72 0.955 96.248
ENSMUSG00000038406 Scaf1 protein_coding 0.917 16.183 0.998
92.931 1.095 33.508 0.945 83.724
ENSMUSG00000038408 1700018A04Rik lincRNA 1 84.977 1
95.301 1 86.666 1 90.289
ENSMUSG00000038412 Higd1a protein_coding 0.856 2.524 1.012
85.985 1.149 24.029 1.026 79.268
ENSMUSG00000038415 Foxq1 protein_coding 2.181 14.194 0.846
95.504 0.544 20.258 0.936 91.65
ENSMUSG00000038416 Cdc16 protein_coding 1.014 86.658 1.007
96.68 0.981 88.601 1.09 44.882
ENSMUSG00000038417 Fig4 protein_coding 1.06 30.282 1.001
95.196 0.944 10.362 NA NA
ENSMUSG00000038418 Egr1 protein_coding 0.746 22.92 1.069 92.793
1.249 30.645 0.946 92.762
ENSMUSG00000038421 Fcrla protein_coding 1.241 85.946 1.052
92.561 0.765 57.819 0.786 85.493
ENSMUSG00000038422 Hdhd3 protein_coding 0.883 32.168 1.503
60.309 0.752 9.347 1.048 96.287
ENSMUSG00000038425 Poli protein_coding 1.01 80.815 0.956
87.998 1.038 80.089 0.941 85.493
ENSMUSG00000038429 Usp5 protein_coding 0.894 0.987 1.037 72.642
1.073 12.703 1.066 46.412
ENSMUSG00000038437 Mllt6 protein_coding 0.866 4.176 1.085 71.617
1.062 45.449 0.96 83.724
ENSMUSG00000038446 Cdc40 protein_coding 0.994 85.832 0.914
59.946 1.096 29.777 0.993 96.183
ENSMUSG00000038451 Spsb2 protein_coding 0.902 53.407 1.093
84.832 1.015 75.527 1.047 92.381
ENSMUSG00000038453 Srcin1 protein_coding 0.789 23.319
1.106 96.138 1.148 48.304 0.795 84.29
ENSMUSG00000038456 Dennd2a protein_coding 1.071 43.939
0.895 55.265 1.041 61.171 1.08 59.441
ENSMUSG00000038457 Tmem255b protein_coding 1.193 25.026
0.997 96.407 0.841 8.634 0.958 93.264
ENSMUSG00000038459 Abhd17c protein_coding 1.027 62.689
1.008 96.843 0.962 59.383 0.911 83.724
ENSMUSG00000038462 Uqcrfs1 protein_coding 0.989 64.589
0.981 97.155 1.033 74.082 1.009 94.904
ENSMUSG00000038463 Olfml2b protein_coding 1.278 0.461 0.948
93.654 0.823 10.726 1.01 72.826
ENSMUSG00000038467 Chmp4b protein_coding 0.976 79.93 1.007

88.705 1.019 56.708 1.073 95.777
ENSMUSG00000038473 Nos1ap protein_coding 1.158 42.134
1.029 96.216 0.839 50.523 0.985 96.086
ENSMUSG00000038481 Cdk19 protein_coding 0.84 2.009 0.916 96.216
1.301 7.482 0.862 83.724
ENSMUSG00000038482 Tfdp1 protein_coding 1.017 76.968 0.984
97.263 NA NA 1.091 32.698
ENSMUSG00000038485 Socs7 protein_coding 1.022 18.635 1.076
82.76 0.906 1.58 0.963 84.29
ENSMUSG00000038486 Sv2a protein_coding 1.035 60.435 1.029
95.708 0.938 78.642 1.078 88.111
ENSMUSG00000038489 Polr2l protein_coding 0.808 1.175 1.101
60.309 1.119 43.842 1.019 90.177
ENSMUSG00000038491 4930520K10Rik antisense 0.755 30.877
1.299 82.904 1.014 79.554 0.809 83.724
ENSMUSG00000038495 Otud7b protein_coding 0.995 80.251
0.938 79.475 1.074 44.361 1.038 82.328
ENSMUSG00000038496 Slc19a3 protein_coding 0.94 74.299
1.348 75.62 0.792 28.669 0.68 83.724
ENSMUSG00000038497 Tmco3 protein_coding 0.894 1.15 0.964 85.793
1.159 6.069 0.964 94.174
ENSMUSG00000038498 Catsper1 protein_coding 1 59.028 1
96.216 1 79.897 1 96.183
ENSMUSG00000038500 Prr3 protein_coding 0.88 5.686 1.016 95.809
1.113 13.047 0.993 95.877
ENSMUSG00000038502 Ptov1 protein_coding 0.916 14.221 1.045
85.378 1.043 45.94 1.017 93.016
ENSMUSG00000038503 Mesdc2 protein_coding 1.006 73.124
1.055 79.411 0.943 21.117 1.025 92.018
ENSMUSG00000038506 Dcun1d2 protein_coding 0.997 82.705
1.101 71.222 0.913 3.969 1.041 48.857
ENSMUSG00000038507 Parp12 protein_coding 1.098 4.628 1.038
96.216 0.875 22.009 1.087 49.514
ENSMUSG00000038508 Gdf15 protein_coding 1.251 43.939 0.898
94.848 0.888 64.155 1.134 74.607
ENSMUSG00000038510 Rpf2 protein_coding 1.18 10.858 0.925 75.4
0.918 83.607 1.074 51.967
ENSMUSG00000038515 Grtp1 protein_coding 1.031 74.516 1.029
94.767 0.943 59.383 0.981 90.157
ENSMUSG00000038517 Tbkbp1 protein_coding 0.944 63.283
1.092 79.411 0.97 82.685 1.02 92.381
ENSMUSG00000038518 Jarid2 protein_coding 1.071 16.436
1.018 88.923 0.914 5.884 0.954 84.29
ENSMUSG00000038520 Tbc1d17 protein_coding 0.928 51.56 0.943
66.09 1.143 30.292 1.043 90.611
ENSMUSG00000038521 C1s protein_coding 1.201 10.464 0.942
86.277 0.882 47.12 1.04 83.276
ENSMUSG00000038522 NA NA NA NA NA NA 0.867 84.552
0.982 96.183
ENSMUSG00000038523 1700003F12Rik protein_coding 0.593 24.817
1.857 76.634 0.905 85.792 1.043 96.139
ENSMUSG00000038524 Fchsd1 protein_coding 0.898 17.062 1
95.708 1.114 19.609 0.985 96.183
ENSMUSG00000038525 Armc10 protein_coding 1.107 5.313 0.894

38.699	NA	NA	0.944	83.724
ENSMUSG00000038526	Car14	protein_coding	1.279	73.401	1.088
87.282	0.717	42.621	1.1	96.358
ENSMUSG00000038527	C1rl	protein_coding	1.162	27.545	1.019
97.263	0.845	27.86	0.898	85.493
ENSMUSG00000038528	BC021785	protein_coding	0.812	77.763
1.087	90.116	1.127	86.666	1.036	95.74
ENSMUSG00000038530	Rgs4	protein_coding	1.074	66.58	1.064	83.104
0.874	55.214	0.932	91.217
ENSMUSG00000038533	Cbfa2t2	protein_coding	1.033	44.334
0.976	86.538	0.988	82.575	1.016	94.016
ENSMUSG00000038534	Osbpl7	protein_coding	0.908	26.013	1.01
93.908	1.088	24.375	1.031	90.455
ENSMUSG00000038535	Zfp280d	protein_coding	1.084	13.734
0.931	62.692	0.986	88.657	1.001	94.19
ENSMUSG00000038537	Mc3r	protein_coding	1	85.946	1
92.474	1	86.985	1	96.287
ENSMUSG00000038538	Ubn2	protein_coding	0.932	18.138	0.97
90.378	1.105	17.412	0.997	95.593
ENSMUSG00000038539	Atf5	protein_coding	0.82	3.009	1.112	79.367
1.1	37.741	1.046	89.052
ENSMUSG00000038540	Tmc3	protein_coding	0.991	84.324	1.155
91.643	0.874	36.499	1.138	93.132
ENSMUSG00000038541	Srd5a2	protein_coding	1	76.396	1
91.643	1	76.64	1	93.612
ENSMUSG00000038542	Pcid2	protein_coding	1.085	7.959	0.953	71.889
0.964	55.701	0.925	83.724
ENSMUSG00000038543	BC028528	protein_coding	1.209	1.712	1.006
86.861	0.824	9.347	1.082	84.31
ENSMUSG00000038544	Inip	protein_coding	1.031	69.089	0.979
91.955	0.99	88.729	1.038	93.016
ENSMUSG00000038545	Cul7	protein_coding	0.947	54.176	0.995
95.301	1.062	47.935	1.05	84.499
ENSMUSG00000038546	Ranbp9	protein_coding	0.944	75.761
0.898	57.613	1.178	18.411	1.029	94.904
ENSMUSG00000038550	Gm129	protein_coding	0.746	85.203	1.198
97.049	1.121	77.17	1.268	74.047
ENSMUSG00000038552	Fndc4	protein_coding	1.119	44.334	1.083
96.68	0.827	29.217	1.023	89.152
ENSMUSG00000038555	Reep2	protein_coding	1.109	52.134	1.112
89.434	0.812	41.506	1.165	64.513
ENSMUSG00000038560	Sp6	protein_coding	1.167	62.007	NA	NA
0.861	78.954	0.884	87.331
ENSMUSG00000038563	Eftud1	protein_coding	0.988	65.388
0.999	87.998	1.009	44.515	0.984	95.273
ENSMUSG00000038564	Ift172	protein_coding	0.91	5.544	1.002
95.708	1.093	5.964	1.042	86.301
ENSMUSG00000038567	Cyp24a1	protein_coding	1	63.126	1
96.308	1	62.237	1	91.011
ENSMUSG00000038569	Rad9b	protein_coding	0.91	39.953	1.021
97.129	1.078	59.274	0.937	83.724
ENSMUSG00000038570	Fam154b	protein_coding	1.066	85.355
1.003	96.378	0.937	84.669	0.833	83.724
ENSMUSG00000038576	Susd4	protein_coding	0.862	44.787	1.026

95.708 1.127 54.163 0.911 96.183
ENSMUSG00000038578 Susd1 protein_coding 1.215 14.205 0.792
20.174 1.041 72.378 1.016 96.183
ENSMUSG00000038580 Sct protein_coding 0.869 28.514 0.721
95.504 1.589 44.515 1.324 59.464
ENSMUSG00000038582 Pptc7 protein_coding 1.065 18.067 1.027
96.407 0.915 20.712 1.014 93.964
ENSMUSG00000038583 Pln protein_coding 2.023 1.323 0.963 91.361
0.513 6.858 0.895 95.273
ENSMUSG00000038587 Akap12 protein_coding 1.064 67.163
0.905 72.399 1.041 73.309 1.078 91.625
ENSMUSG00000038591 Colec10 protein_coding 4.054 16.539
0.185 44.268 1.334 70.534 0.621 86.991
ENSMUSG00000038593 Tctn1 protein_coding 0.933 30.136 0.983
95.809 1.09 48.772 1.007 90.289
ENSMUSG00000038594 Cep85l protein_coding 1.101 82.543
0.953 95.708 0.954 87.173 0.937 92.466
ENSMUSG00000038599 Capn8 protein_coding 0.641 1.175 0.88 83.58
1.777 5.864 0.922 90.611
ENSMUSG00000038600 Atp6v0a4 protein_coding 1.088 85.728
0.502 72.576 1.826 38.038 1.511 85.629
ENSMUSG00000038602 Slc35f1 protein_coding 1.07 71.896 1
95.504 0.933 77.704 0.976 91.291
ENSMUSG00000038604 Fam65a protein_coding 0.895 12.181
1.059 83.104 1.057 68.643 1.038 83.067
ENSMUSG00000038605 Samd10 protein_coding 0.855 18.795
0.809 67.839 1.443 6.916 0.879 86.991
ENSMUSG00000038607 Gng10 protein_coding 0.758 74.482 0.835
91.532 1.577 47.512 0.947 94.904
ENSMUSG00000038608 Dock10 protein_coding 1.1 15.662 0.99
95.211 0.919 7.329 1.13 64.513
ENSMUSG00000038611 Phrf1 protein_coding 0.983 43.816 1.003
89.494 1.016 70.534 0.973 96.287
ENSMUSG00000038612 Mcl1 protein_coding 1.094 1.441 1.005 96.216
0.91 17.381 1.041 79.442
ENSMUSG00000038615 Nfe2l1 protein_coding 0.94 33.599
1.056 82.904 1.008 84.896 1.068 76.831
ENSMUSG00000038618 Rassf7 protein_coding 0.88 23.556
0.926 87.452 1.23 17.662 0.926 85.493
ENSMUSG00000038619 Ensa protein_coding 1.109 1.129 0.931 80.463
0.968 18.965 1.054 44.679
ENSMUSG00000038622 Med30 protein_coding 1.006 66.406 1.021
96.407 0.972 84.896 1.006 92.762
ENSMUSG00000038623 Tm6sf1 protein_coding 1.275 0 0.812
40.463 0.965 53.224 1.061 83.067
ENSMUSG00000038624 Nepn protein_coding 1.263 15.602 1.061
92.062 0.748 12.571 0.793 83.724
ENSMUSG00000038628 Polr3k protein_coding 1.021 81.178
0.962 93.469 1.018 87.37 1.013 96.358
ENSMUSG00000038630 Zkscan16 protein_coding 0.703 69.985
1.047 96.984 1.352 73.309 1.617 82.85
ENSMUSG00000038633 Degs1 protein_coding 1.046 82.732 1.112
60.309 0.86 10.934 1.045 91.872
ENSMUSG00000038637 Lrrc56 protein_coding 0.928 12.309

0.914 94.262 1.181 22.994 0.959 90.95
ENSMUSG00000038642 Ctss protein_coding 1.746 0 0.748 45.262
0.765 8.626 1.097 91.525
ENSMUSG00000038644 Pold1 protein_coding 0.937 48.146 1.042
96.948 1.022 70.9 1.098 50.452
ENSMUSG00000038646 Fam103a1 protein_coding 0.997 86.57 1.07
79.579 0.937 22.009 0.95 83.724
ENSMUSG00000038648 Creb3l2 protein_coding 1.014 82.231
1.092 61.943 0.905 17.381 0.976 95.273
ENSMUSG00000038650 Rnh1 protein_coding 0.988 63.283 0.969
84.858 1.041 40.575 1.058 43.377
ENSMUSG00000038656 Cyp3a16 protein_coding 1 80.408 1
97.263 1 74.082 1 94.567
ENSMUSG00000038658 C030046E11Rik protein_coding 1.014 60.168
1.057 82.904 0.933 16.949 0.946 83.724
ENSMUSG00000038663 Fsd2 protein_coding 1.066 74.516 1.17
88.092 0.803 40.338 1.07 44.213
ENSMUSG00000038664 Herc1 protein_coding 1.074 35.787 0.893
53.79 1.045 50.375 1.039 92.762
ENSMUSG00000038665 Dgki protein_coding 0.757 25.111 1.183
83.104 1.115 57.637 1.055 95.559
ENSMUSG00000038668 Lpar1 protein_coding 1.238 0.2 0.944 82.503
0.853 14.079 1.187 38.2
ENSMUSG00000038670 Mybpc2 protein_coding 6.519 5.481 0.854
92.111 0.179 0.72 0.967 95.273
ENSMUSG00000038671 Arfrp1 protein_coding 0.913 3.47 0.983
96.763 1.108 11.348 1.033 91.525
ENSMUSG00000038677 Scube3 protein_coding 1.137 17.191
1.122 82.904 0.781 0.72 0.874 83.724
ENSMUSG00000038679 Trps1 protein_coding 0.992 77.615 1.028
95.196 0.978 82.778 0.885 88.053
ENSMUSG00000038683 Pak1ip1 protein_coding 0.905 3.559 1.043
95.196 1.056 15.411 1 86.133
ENSMUSG00000038685 Rtel1 protein_coding 0.974 60.003 0.999
95.504 1.023 51.851 1.064 44.679
ENSMUSG00000038690 Atp5j2 protein_coding 0.881 12.181 1.06
82.904 1.071 53.587 1.097 83.276
ENSMUSG00000038691 Mbd3l1 protein_coding 1 76.042 NA
NA 1 80.5 1 94.174
ENSMUSG00000038692 Hoxb4 protein_coding 0.899 12.785 1.053
86.156 1.057 42.065 0.98 93.964
ENSMUSG00000038695 Josd2 protein_coding 0.85 0.987 1.051 82.904
1.121 13.174 1.094 71.855
ENSMUSG00000038696 Mapkap1 protein_coding 0.959 8.405 1.088
79.411 0.955 86.727 1.048 72.369
ENSMUSG00000038697 Taf5l protein_coding 1.019 45.746 0.99
70.29 0.993 77.17 0.991 88.505
ENSMUSG00000038700 Hoxb5 protein_coding 0.798 11.128 1.183
74.663 1.059 76.498 0.943 93.316
ENSMUSG00000038702 Dsel protein_coding 1.18 1.129 NA NA
0.803 7.948 1.071 83.067
ENSMUSG00000038704 Aspdh protein_coding 0.511 35.449 1.156
95.735 1.698 67.562 0.652 85.493
ENSMUSG00000038705 Gmeb2 protein_coding 1.042 60.435 0.908

62.025	1.059 34.07 0.994 93.156
ENSMUSG00000038708	Golga4	protein_coding	0.97	53.407
0.964 84.858	1.066 52.574	1.066 78.042
ENSMUSG00000038709	Txndc8	protein_coding	1	65.199	1
93.707	NA	NA	1	95.74
ENSMUSG00000038712	Fam63a	protein_coding	0.969 61.043
0.961 93.654	1.076 13.356	1.007 96.183
ENSMUSG00000038717	Atp5l protein_coding	0.965 46.413	1.006
92.561	1.03	81.857	1.015 93.261
ENSMUSG00000038718	Pbx3	protein_coding	1.128 10.105	0.994
96.847	0.894 11.116	0.948 92.31
ENSMUSG00000038721	Hoxb7 protein_coding	0.922 41.021	1.049
93.529	NA	NA	0.944 95.72
ENSMUSG00000038722	Bud31 protein_coding	0.968 50.519	1.057
83.104	0.975 73.598	1.048 71.855
ENSMUSG00000038725	Pkhd1l1	protein_coding	1.275 25.207
0.994 94.597	0.79	59.125	0.938 92.181
ENSMUSG00000038729	Akap2 protein_coding	1.133 76.319	1.145
88.551	0.772 47.12 0.902 90.628
ENSMUSG00000038732	Mboat1	protein_coding	1.24	49.868
0.799 68.818	1.005 73.759	1.04	86.118
ENSMUSG00000038733	Wdr26 protein_coding	1.023 33.099	0.931
41.951	1.05	29.819	1.011 96.358
ENSMUSG00000038736	Nudcd1	protein_coding	1.054 49.868
0.907 88.881	1.047 83.607	1.036 70.36
ENSMUSG00000038738	Shank1	protein_coding	0.918 56.242
1.083 83.104	1.008 85.935	1.06	83.067
ENSMUSG00000038740	Mvb12b	protein_coding	1.091 15.602
1.071 81.127	0.857 6.68	0.966 93.612
ENSMUSG00000038742	Angptl6	protein_coding	0.808 18.602
0.872 76.431	1.421 12.831	0.89	84.29
ENSMUSG00000038750	Omt2b protein_coding	1	72.27 1	95.941
1	87.744	1	96.192
ENSMUSG00000038751	Ptk6	protein_coding	0.913 39.712	1.311
86.277	0.836 79.119	0.865 95.832
ENSMUSG00000038754	Elovl3	protein_coding	2.44	11.197
0.591 73.766	0.692 40.338	1.154 94.001
ENSMUSG00000038756	Ttll6 protein_coding	0.804 60.707	0.635
79.475	1.957 24.791	2.711 36.174
ENSMUSG00000038759	Nup205	protein_coding	1.057 38.682
0.958 87.452	0.989 76.388	1.03	60.968
ENSMUSG00000038760	Trhr	protein_coding	1	67.374	1
96.216	1	86.346	1	96.287
ENSMUSG00000038762	Abcf1 protein_coding	0.924 7.71	0.988 93.046
1.097 9.176 1.047 49.239
ENSMUSG00000038763	Alpk3 protein_coding	2.189 15.662	1.187
89.434	0.386 4.521 1.005 91.011
ENSMUSG00000038764	Ptpn3 protein_coding	0.922 69.985	0.933
95.35 1.161 58.414	1.056 95.72
ENSMUSG00000038765	Lmx1b protein_coding	0.772 3.641 1.07	97.263
1.213 14.873	1.016 91.625
ENSMUSG00000038766	Gabpb2	protein_coding	1.091 10.464	0.91
68.818	1.009 58.775	0.951 86.445
ENSMUSG00000038768	9130409I23Rik	protein_coding	1	81.01 1

96.68 1 88.349 1 95.593
ENSMUSG00000038770 Kpna7 protein_coding 0.706 26.013 0.924
90.576 1.532 22.994 0.978 87.542
ENSMUSG00000038773 Kdm3b protein_coding 0.978 47.266 0.98
78.471 1.043 23.454 0.986 87.735
ENSMUSG00000038774 Ascc3 protein_coding 1.018 71.36 0.943 96.847
1.042 70.9 1.101 50.452
ENSMUSG00000038775 Vill protein_coding 0.831 3.623 0.845 59.946
1.424 5.864 0.836 86.699
ENSMUSG00000038776 Ephx1 protein_coding 1.137 1.412 0.887 62.692
0.993 47.12 1.148 84.246
ENSMUSG00000038777 Sema6c protein_coding 1.053 72.372
1.039 94.767 0.915 48.851 0.955 92.181
ENSMUSG00000038780 Smurf1 protein_coding 1.153 22.349 NA
NA 0.881 8.634 0.901 83.724
ENSMUSG00000038781 Stap2 protein_coding 0.784 3.623 NA NA
1.222 10.281 0.88 85.493
ENSMUSG00000038782 1700028J19Rik protein_coding 1.044 71.36
0.736 78.51 1.302 28.301 0.96 91.928
ENSMUSG00000038784 Cnot4 protein_coding 1.122 0.2 1.003 92.793
0.888 0.72 1.004 93.316
ENSMUSG00000038791 Scgb3a2 protein_coding 1 78.639 1
97.129 1 84.724 1 93.734
ENSMUSG00000038792 4931403M11Rik lincRNA 1 71.217 1
97.049 1 73.598 1 92.181
ENSMUSG00000038793 Lefty1 protein_coding 1.326 11.044
0.989 97.263 0.761 14.262 1.14 48.857
ENSMUSG00000038797 Zscan2 protein_coding 0.906 21.176
0.853 41.951 1.291 6.202 1.002 96.287
ENSMUSG00000038803 Ost4 protein_coding 1.013 85.391 1.048
72.642 0.94 26.369 1.04 59.464
ENSMUSG00000038805 Six3 protein_coding 1 74.516 1
96.216 1 80.089 1 95.559
ENSMUSG00000038806 NA NA NA NA 0.963 74.483 1.02
78.369 1.006 95.92
ENSMUSG00000038807 Rap1gap2 protein_coding 1.427 18.067 NA
NA 0.689 16.051 0.963 92.742
ENSMUSG00000038811 Gngt2 protein_coding 0.942 74.482 0.955
85.95 1.109 43.586 0.923 83.724
ENSMUSG00000038812 Trmt112 protein_coding 0.972 65.388
1.032 86.277 0.997 80.015 0.998 93.964
ENSMUSG00000038816 Ctnnal1 protein_coding 1.205 10.391 1.03
96.652 0.802 6.858 0.904 85.493
ENSMUSG00000038822 Hace1 protein_coding 0.982 85.857 0.923
67.739 1.106 27.475 1.022 95.676
ENSMUSG00000038827 BC026590 protein_coding 0.994 69.24 1.092
88.092 0.923 72.269 1.037 83.067
ENSMUSG00000038828 Tmem214 protein_coding 1.051 7.799 1.04
75.62 0.911 0.945 0.994 93.117
ENSMUSG00000038831 Ralgps1 protein_coding 0.916 23.319
0.927 93.654 1.179 11.932 1.098 73.401
ENSMUSG00000038836 Agbl3 protein_coding 0.939 67.477 1.062
97.263 1.004 80.089 0.991 86.991
ENSMUSG00000038838 Vars2 protein_coding 0.891 1.031 1.033 86.387

1.087 11.518 0.993 93.964
ENSMUSG00000038840 Birc7 protein_coding 1 82.732 1
90.256 1 51.977 1 95.676
ENSMUSG00000038843 Gcnt1 protein_coding 1.608 0 0.748 31.966
0.828 23.938 1.314 71.305
ENSMUSG00000038844 Kif16b protein_coding 1.08 1.242 0.943
70.29 0.981 35.559 0.997 93.964
ENSMUSG00000038845 Phb protein_coding 0.847 0.987 1.081 88.092
1.095 10.76 1.064 70.85
ENSMUSG00000038848 Ythdf1 protein_coding 1.031 54.44 0.998
93.654 0.967 67.339 1.038 65.558
ENSMUSG00000038855 Itpkb protein_coding 1.057 42.134 1.091
82.904 0.869 3.863 0.883 83.724
ENSMUSG00000038859 Baiap2l1 protein_coding 0.956 84.263
0.919 73.166 1.139 42.75 0.902 88.387
ENSMUSG00000038860 Garnl3 protein_coding 1.018 85.59 1.072
90.68 0.914 35.815 0.937 86.991
ENSMUSG00000038861 Pi4kb protein_coding 1.006 77.291 1.021
95.196 0.973 56.822 1.027 72.616
ENSMUSG00000038866 Zcchc2 protein_coding 1.076 7.553 NA NA
0.931 5.042 0.984 95.832
ENSMUSG00000038871 Bpgm protein_coding 1.085 2.085 0.928 20.272
0.99 72.269 1.102 72.116
ENSMUSG00000038872 Zfhx3 protein_coding 0.894 18.138 1.045
93.707 1.069 25.425 0.964 86.699
ENSMUSG00000038876 Rnf146 protein_coding 0.875 15.486
1.073 86.34 1.065 45.667 0.886 83.724
ENSMUSG00000038879 Nipal2 protein_coding 1.049 48.857
0.987 85.242 0.964 88.076 1.024 93.132
ENSMUSG00000038880 Mrps34 protein_coding 1.075 40.88 1.095
91.584 0.847 38.534 0.951 94.654
ENSMUSG00000038883 Prl3a1 protein_coding 1 81.178 1
94.767 1 78.155 1 94.567
ENSMUSG00000038884 A230050P20Rik protein_coding 1 75.115
0.942 83.876 1.063 42.75 1.005 86.699
ENSMUSG00000038886 Man2a2 protein_coding 0.962 41.021
1.035 83.104 NA NA 1.005 90.177
ENSMUSG00000038888 Ctu1 protein_coding 0.897 47.677 0.995
91.83 1.12 38.099 1.037 95.273
ENSMUSG00000038891 Prl3b1 protein_coding 1 75.671 1
95.301 1 85.935 1 96.071
ENSMUSG00000038893 Fam117a protein_coding 0.949 58.56 1.115
71.617 0.941 32.297 1.065 90.397
ENSMUSG00000038894 Irs2 protein_coding 0.792 81.465 1.008
83.733 1.247 50.091 0.998 95.593
ENSMUSG00000038895 Zfp653 protein_coding 0.934 6.34 1.001
84.126 1.07 50.375 1.008 94.654
ENSMUSG00000038900 Rpl12 protein_coding 0.986 86.74 0.941 73.766
1.076 51.596 1.044 92.018
ENSMUSG00000038902 Pogz protein_coding 1.026 61.725 1.027
71.617 0.95 6.007 1.002 94.904
ENSMUSG00000038903 Ccdc68 protein_coding 1.052 45.746 0.79
35.072 1.202 38.099 0.904 85.493
ENSMUSG00000038909 Kat7 protein_coding 1.011 69.408 0.989

83.321 1.002 78.155 0.984 93.457
ENSMUSG00000038910 Plcl2 protein_coding 1.311 0 0.942 75.052
0.81 7.809 0.98 92.12
ENSMUSG00000038914 Dido1 protein_coding 0.912 7.207 1.023 96.407
1.069 10.523 0.974 90.289
ENSMUSG00000038916 Soga3 protein_coding 0.654 58.802 NA NA
1.355 44.125 0.585 94.174
ENSMUSG00000038917 NA NA NA NA 0.865 68.818 1.189
56.125 1.231 68.696
ENSMUSG00000038925 E330034G19Rik protein_coding 1 79.853
1 90.712 1 72.148 1 92.762
ENSMUSG00000038930 Rccd1 protein_coding 0.791 1.15 1.15 64.304
1.101 17.412 1.044 83.067
ENSMUSG00000038932 Tcfl5 protein_coding 0.911 54.604 0.75
97.155 1.46 60.5 1.105 82.66
ENSMUSG00000038936 Sccpdh protein_coding 0.811 8.181 1.008
94.767 1.222 17.998 1.047 90.674
ENSMUSG00000038943 Prc1 protein_coding 1.238 69.777 0.683
67.739 1.179 82.74 1.511 68.502
ENSMUSG00000038949 Cnst protein_coding 0.985 42.722 0.807
90.138 1.258 36.839 0.971 88.505
ENSMUSG00000038954 Supt3 protein_coding 0.851 12.624 1.074
92.111 1.09 32.776 1.034 95.35
ENSMUSG00000038957 Edc3 protein_coding 0.926 30.877 0.956
53.79 1.132 5.864 0.976 85.493
ENSMUSG00000038963 Slco4a1 protein_coding 1.5 74.958
0.787 96.652 0.848 80.5 1.057 95.273
ENSMUSG00000038965 Ube2l3 protein_coding 0.979 50.229
1.041 79.261 0.984 72.269 1.111 31.133
ENSMUSG00000038967 Pdk2 protein_coding 0.904 33.002 0.837
84.594 1.324 52.8 1.02 96.287
ENSMUSG00000038968 Klk1b16 protein_coding 1 80.608 1
97.097 1 86.666 1 96.287
ENSMUSG00000038970 Lmtk2 protein_coding 1.02 56.907 1.068
71.617 0.915 3.943 0.966 84.29
ENSMUSG00000038973 Cldnd2 protein_coding 0.876 78.909
1.225 97.288 0.928 87.519 0.658 84.29
ENSMUSG00000038975 Rabggtb protein_coding 1.086 19.477
0.912 54.06 1.013 74.325 1.018 79.268
ENSMUSG00000038976 Ppp1r9b protein_coding 1.037 47.466
1.088 88.923 0.889 11.504 0.89 83.724
ENSMUSG00000038980 Rbbp8nl protein_coding 1.084 79.068
0.915 95.211 NA NA 0.905 88.505
ENSMUSG00000038982 Bloc1s5 protein_coding 1.026 49.517 1.02
87.609 0.953 15.63 0.959 89.475
ENSMUSG00000038984 Tspyl5 protein_coding 1.102 84.324
0.936 92.404 NA NA 0.843 84.29
ENSMUSG00000038987 1700019L03Rik protein_coding 0.802 61.043
1.295 93.707 NA NA 0.817 85.493
ENSMUSG00000038990 Cables2 protein_coding 0.955 29.972
1.039 93.707 1.003 55.109 1.008 96.358
ENSMUSG00000038991 Txndc5 protein_coding 1.046 22.871
1.068 85.756 0.893 27.994 1.076 0
ENSMUSG00000038997 Asb17 protein_coding 1 86.918 1

95.708 1 88.601 1 96.183
ENSMUSG00000039000 Ube3c protein_coding 1.033 43.939 0.993
89.494 0.972 12.948 1.022 87.088
ENSMUSG00000039001 Rps21 protein_coding 1.01 85.203 0.928
60.678 1.07 41.6 0.983 91.807
ENSMUSG00000039004 Bmp6 protein_coding 1.031 73.401 0.835
78.877 1.158 51.851 0.999 96.248
ENSMUSG00000039005 Tlr4 protein_coding 1.096 47.174 0.95
95.301 0.961 56.822 1.02 92.762
ENSMUSG00000039007 Cpq protein_coding 1.025 77.615 0.919
86.861 1.063 64.624 1.05 91.525
ENSMUSG00000039013 Siglec5 protein_coding 1.292 32.676 0.63
48.565 1.227 63.665 1.07 92.564
ENSMUSG00000039016 Timm8b protein_coding 1.04 46.097
0.989 93.654 0.97 67.339 1.005 91.872
ENSMUSG00000039018 Mtg1 protein_coding 0.955 28.804 1
92.914 1.049 28.997 1.031 93.964
ENSMUSG00000039021 Ttc16 protein_coding 0.751 8.45 1.101 84.007
1.211 44.361 1.134 84.499
ENSMUSG00000039031 Arhgap18 protein_coding 1.183 20.942
1.057 96.407 0.801 15.169 0.862 83.724
ENSMUSG00000039032 Tsga13 protein_coding 2.04 58.198
0.815 93.094 0.599 78.713 0.7 91.269
ENSMUSG00000039033 Tasp1 protein_coding 1.145 17.761 0.92
94.96 0.946 44.211 1.022 92.691
ENSMUSG00000039037 St6galnac5 protein_coding 1.58 1.712 0.747
71.41 0.847 19.765 0.849 86.991
ENSMUSG00000039041 Adrm1 protein_coding 0.877 0.987 1.084 72.642
1.052 26.978 1.001 90.177
ENSMUSG00000039043 2610034B18Rik protein_coding 0.925 19.985
1.01 93.817 1.065 15.688 1.012 93.722
ENSMUSG00000039046 Usp6nl protein_coding 1.006 78.224 0.96
95.504 1.032 77.532 0.961 86.991
ENSMUSG00000039047 Pigk protein_coding 1.01 85.832 1.003
96.268 NA NA 1.04 57.075
ENSMUSG00000039048 Foxred1 protein_coding 0.941 43.816
1.022 92.253 1.037 72.853 1.016 94.215
ENSMUSG00000039050 Osbpl2 protein_coding 1.002 84.168
1.034 96.843 0.962 67.648 0.999 93.612
ENSMUSG00000039055 Eme1 protein_coding 0.834 35.449 0.971
93.146 1.239 74.686 0.958 85.493
ENSMUSG00000039057 Myo16 protein_coding 0.896 72.372 1.431
96.268 0.778 58.775 0.783 90.289
ENSMUSG00000039058 Ak5 protein_coding 0.97 63.736 1.004
87.185 1.023 85.675 0.912 86.991
ENSMUSG00000039062 Anpep protein_coding 1.11 25.735 0.961
95.329 0.934 14.583 0.969 83.724
ENSMUSG00000039063 Echdc3 protein_coding 0.874 14.075
1.049 78.705 1.086 74.082 0.906 83.724
ENSMUSG00000039065 Fam173b protein_coding 0.948 47.677
0.991 88.691 1.061 34.07 1.016 86.301
ENSMUSG00000039067 Psmd7 protein_coding 0.956 15.76 1.016 91.065
1.03 38.26 1.073 31.133
ENSMUSG00000039068 Zzz3 protein_coding 1.068 12.72 0.971 90.744

0.965 25.345 0.954 86.991
ENSMUSG00000039069 Mtg2 protein_coding 0.988 84.534 0.958
95.708 1.058 84.308 0.969 87.735
ENSMUSG00000039079 Trhr2 protein_coding 1 68.66 1 93.899
1 85.921 1 95.273
ENSMUSG00000039081 Zfp503 protein_coding 0.959 39.712
1.181 71.617 0.883 40.338 0.933 91.105
ENSMUSG00000039084 Chad protein_coding 0.626 38.555 1.111
96.68 1.442 32.433 0.936 84.29
ENSMUSG00000039086 Ss18l1 protein_coding 0.963 48.354 NA
NA 1.066 70.328 0.953 83.952
ENSMUSG00000039087 Rreb1 protein_coding 1.027 80.51 1.005 96.68
0.967 87.431 0.944 92.675
ENSMUSG00000039089 L3mbtl3 protein_coding 0.983 44.562
1.002 96.335 1.014 70.534 0.981 94.904
ENSMUSG00000039092 Sptlc3 protein_coding 0.949 86.57 0.998
72.399 1.058 47.935 1.088 96.287
ENSMUSG00000039095 En2 protein_coding 3.528 61.171 0.271
87.553 NA NA 0.541 94.904
ENSMUSG00000039096 Rsad1 protein_coding 1.037 36.124 1.031
97.263 0.939 25.688 0.98 95.593
ENSMUSG00000039098 Gm9767 protein_coding 0.646 49.621
1.196 90.895 1.291 82.778 1.234 94.99
ENSMUSG00000039099 Wdr93 protein_coding 1.013 74.689 0.986
87.041 1.004 71.232 0.897 84.19
ENSMUSG00000039100 6-Mar protein_coding 1.065 8.253 0.959 79.261
0.976 52.658 0.933 83.724
ENSMUSG00000039103 Nexn protein_coding 1.426 5.481 0.979 87.609
0.718 0.72 0.924 96.086
ENSMUSG00000039105 Atp6v1g1 protein_coding 0.854 0.987 1.04
83.104 1.124 6.202 1.05 60.31
ENSMUSG00000039106 Htr5a protein_coding 1 52.018 1
97.263 1 88.349 1 93.156
ENSMUSG00000039108 Lsm14b protein_coding 0.963 23.923
1.015 95.196 1.023 51.977 1.032 60.466
ENSMUSG00000039109 F13a1 protein_coding 1.321 0 0.896 64.123
0.845 7.083 1.234 32.045
ENSMUSG00000039110 Mycbpap protein_coding 0.826 23.641
0.789 85.075 1.531 34.07 0.842 83.724
ENSMUSG00000039114 Nrn1 protein_coding 0.756 7.118 1.278 72.546
1.034 81.976 0.88 83.724
ENSMUSG00000039115 Itga9 protein_coding 0.92 14.086 1.006
93.899 1.076 53.449 1.031 79.268
ENSMUSG00000039116 Gpr126 protein_coding 1.035 45.746
0.965 66.796 0.995 78.155 1.005 76.697
ENSMUSG00000039117 Taf4a protein_coding 1.082 3.713 0.996 95.504
0.927 7.155 0.933 83.724
ENSMUSG00000039126 Prune2 protein_coding 1.105 59.527
0.873 81.134 1.031 72.695 1.131 53.923
ENSMUSG00000039128 Cdc123 protein_coding 0.948 36.034
0.953 59.946 1.106 6.202 1.031 83.276
ENSMUSG00000039130 Zc3hc1 protein_coding 0.973 63.283
0.985 85.671 1.041 42.522 1.061 67.691
ENSMUSG00000039131 Gipc2 protein_coding 0.939 68.955 1.011

93.654	1.052	50.851	1.035	96.183
ENSMUSG00000039133	9330171B17Rik	protein_coding	0.652	44.467
1.413	89.434	1.082	69.825	0.92	87.496
ENSMUSG00000039137	Whrn	protein_coding	0.967	35.449	1.023
90.895	1.012	88.601	0.992	96.192
ENSMUSG00000039138	4930591A17Rik	lincRNA	1	72.985	1
96.216	1	83.811	1	96.248
ENSMUSG00000039145	Camk1d	protein_coding	1.256	0.461	1.029
96.68	0.772	13.848	1.074	85.577
ENSMUSG00000039146	Ifi44l	protein_coding	1	80.146	1
93.899	1	80.015	1	92.675
ENSMUSG00000039148	Sart1	protein_coding	0.92	11.232	0.991
94.597	1.1	15.688	0.969	86.991
ENSMUSG00000039153	Runx2	protein_coding	1.179	7.553	1.087	95.735
0.782	3.654	0.959	84.29
ENSMUSG00000039154	Shd	protein_coding	0.948	74.689	0.874
97.288	1.211	69.728	0.706	83.724
ENSMUSG00000039155	Cdh26	protein_coding	0.928	85.832	1.567
96.843	0.69	88.693	1.277	93.413
ENSMUSG00000039156	Stim2	protein_coding	1.121	0.2	NA	NA	0.91
0.72	1.055	91.525
ENSMUSG00000039157	Fam102a	protein_coding	0.96	57.544
1.139	93.529	0.914	85.935	0.925	84.29
ENSMUSG00000039158	Akna	protein_coding	1.161	11.879	0.985
93.654	0.872	23.86	0.991	95.593
ENSMUSG00000039159	Ube2h	protein_coding	1.046	21.146	0.987
91.897	0.968	44.865	0.983	84.29
ENSMUSG00000039163	NA	NA	NA	NA	1.105	71.98	0.904	15.169
0.999	94.19
ENSMUSG00000039164	Naif1	protein_coding	0.958	50.519	1.103
79.055	0.947	56.822	0.987	94.016
ENSMUSG00000039166	Akap7	protein_coding	1.03	56.66	1.005	95.301
0.966	74.853	1.007	92.018
ENSMUSG00000039167	NA	NA	NA	NA	0.961	95.504	1.02
84.961	0.872	77.331
ENSMUSG00000039168	Dap	protein_coding	0.938	10.29	1.064	79.261
1.005	70.534	1.027	72.992
ENSMUSG00000039174	1700003H04Rik	protein_coding	1	84.263
NA	NA	1	87.431	1	93.685
ENSMUSG00000039176	Polg	protein_coding	0.871	0.987	1.039	79.261
1.106	5.864	1.023	62.594
ENSMUSG00000039178	Tbc1d19	protein_coding	0.968	60.003
1.034	93.529	0.996	82.74	1.028	64.513
ENSMUSG00000039182	AW209491	protein_coding	1.054	43.939
0.994	91.661	0.95	60.233	0.938	89.525
ENSMUSG00000039183	Nubp2	protein_coding	1.059	86.145	0.976
91.361	0.97	79.119	0.927	90.805
ENSMUSG00000039187	Fanci	protein_coding	1.067	62.689	1.027
90.256	NA	NA	1.092	93.734
ENSMUSG00000039191	Rbpj	protein_coding	1.214	0.2	0.972	72.712
0.845	9.746	0.994	96.183
ENSMUSG00000039193	Nlrc4	protein_coding	1.331	10.687	0.855
82.947	0.88	17.381	0.917	84.29
ENSMUSG00000039195	1110008P14Rik	protein_coding	0.711	2.009

1.049 80.14 1.344 11.518 0.968 94.384
ENSMUSG00000039196 Orm1 protein_coding 0.58 0.987 0.995 95.504
1.728 5.864 0.89 86.301
ENSMUSG00000039197 Adk protein_coding 0.93 51.447 1.077
97.155 0.998 85.464 1.005 78.948
ENSMUSG00000039198 Ptchd3 protein_coding 1 50.913 1
95.211 1 64.427 1 86.699
ENSMUSG00000039199 Zdhhc1 protein_coding 0.927 5.544 1.008
95.809 1.071 26.498 1.019 92.181
ENSMUSG00000039201 Tbc1d25 protein_coding 1.036 81.24 1.068
78.705 0.904 14.206 0.948 84.29
ENSMUSG00000039202 Abhd2 protein_coding 1.31 13.734 0.859
56.781 0.886 69.583 1.175 71.305
ENSMUSG00000039205 Ciz1 protein_coding 0.864 0.987 1.063 88.923
1.09 7.635 1.005 95.877
ENSMUSG00000039206 Daglb protein_coding 1.097 33.325 0.856
62.71 1.063 45.208 1.066 77.331
ENSMUSG00000039208 Metrnl protein_coding 1.311 1.712 0.985
92.137 0.772 10.362 1.08 59.464
ENSMUSG00000039209 Rpl39l protein_coding 0.891 67.163
1.213 87.609 0.926 84.434 0.982 96.139
ENSMUSG00000039210 Gpatch2 protein_coding 0.971 64.893
1.037 83.104 0.99 86.258 0.938 91.625
ENSMUSG00000039215 NA NA NA NA NA NA 1 87.786
1 94.904
ENSMUSG00000039217 Il18 protein_coding 1.545 0.355 0.942 90.744
0.689 15.465 1.087 64.513
ENSMUSG00000039218 Srrm2 protein_coding 0.874 0.987 NA NA
1.139 6.202 0.952 83.724
ENSMUSG00000039219 Arid4b protein_coding 0.963 61.633
0.984 84.594 1.056 38.975 0.978 91.625
ENSMUSG00000039220 Ppp1r10 protein_coding 0.884 15.912
1.213 60.309 0.929 70.214 1.003 91.525
ENSMUSG00000039221 Rpl22l1 protein_coding 1.167 6.853 0.884
48.565 0.971 77.037 1.093 86.118
ENSMUSG00000039224 D1Pas1 protein_coding 1 73.841 NA
NA 1 86.666 1 93.413
ENSMUSG00000039230 Tbcd protein_coding 0.979 56.768 0.973
93.654 1.049 57.227 1.087 40.894
ENSMUSG00000039231 Suv39h1 protein_coding 1.033 41.177
0.984 96.68 0.98 44.019 1.004 83.067
ENSMUSG00000039232 Stx11 protein_coding 1.082 55.124 1.103
93.899 0.834 20.009 0.88 85.493
ENSMUSG00000039233 Tbce protein_coding 0.99 58.077 0.98
90.712 1.03 53.134 1.002 95.877
ENSMUSG00000039234 Sec24d protein_coding 1.141 7.049 1.057
85.756 0.83 2.333 1.075 64.513
ENSMUSG00000039236 Isg20 protein_coding 1.133 17.336 0.919
55.265 NA NA 1.094 60.466
ENSMUSG00000039238 Zfp750 protein_coding 0.77 28.242
0.879 82.671 1.471 24.07 0.955 91.105
ENSMUSG00000039239 Tgfb2 protein_coding 1.073 53.809 1.241
72.642 0.751 6.858 1.228 81.901
ENSMUSG00000039242 B3galnt2 protein_coding 1.155 5.313 0.961

96.216 0.901 11.64 1.008 90.125
ENSMUSG00000039244 E130309D02Rik protein_coding 0.945 26.013
1.127 79.055 0.942 60.233 0.957 91.291
ENSMUSG00000039246 Lyplal1 protein_coding 1.237 3.152 0.887
63.619 0.91 22.864 0.838 83.724
ENSMUSG00000039252 Lgi2 protein_coding 1.26 15.456 1.159
79.411 0.684 2.087 1.199 73.597
ENSMUSG00000039253 Fn3krp protein_coding 1.002 58.077
1.057 83.104 0.941 61.288 1.033 79.268
ENSMUSG00000039254 Pomt1 protein_coding 0.964 65.106 1.147
80.806 0.906 76.768 0.924 86.699
ENSMUSG00000039257 Vstm2b protein_coding 1.086 67.991
1.053 96.216 0.875 66.201 1.137 86.301
ENSMUSG00000039262 Prrc2b protein_coding 1.014 84.738
1.006 89.494 0.983 44.019 0.928 83.724
ENSMUSG00000039263 Npepl1 protein_coding 0.908 5.946 1.08
79.29 1.018 41.331 0.942 83.724
ENSMUSG00000039264 Gimap3 protein_coding 1.087 69.089 1
95.088 0.921 54.521 1.029 90.674
ENSMUSG00000039269 2300002M23Rik protein_coding 1 86.918
1 95.088 NA NA 1 96.192
ENSMUSG00000039270 Megf9 protein_coding 1.059 69.777 1.087
71.617 0.87 1.898 0.97 94.99
ENSMUSG00000039275 Foxk2 protein_coding 1.002 75.399 0.993
94.96 1.006 86.346 1.019 83.067
ENSMUSG00000039278 Pcsk1n protein_coding 0.91 86.843
1.116 90.952 0.986 64.778 0.715 85.493
ENSMUSG00000039285 Azi2 protein_coding 0.996 83.726 0.976
95.504 1.025 83.857 1.037 61.984
ENSMUSG00000039286 Fndc3b protein_coding 1.006 85.203 NA
NA NA NA 1.159 86.301
ENSMUSG00000039294 BC017643 protein_coding 0.954 67.163 NA
NA 1.036 79.897 1.119 38.2
ENSMUSG00000039296 Spdyb protein_coding 1 61.49 1 95.431
1 66.425 1 96.184
ENSMUSG00000039298 Cdk5rap2 protein_coding 1.102 9.983 0.968
92.355 0.934 31.887 0.984 95.777
ENSMUSG00000039304 Tnfsf10 protein_coding 1.123 23.493
1.244 83.104 0.713 4.338 0.98 84.29
ENSMUSG00000039307 Hexdc protein_coding 1.016 35.499 0.995
86.728 0.992 67.339 0.956 83.724
ENSMUSG00000039308 Ndst2 protein_coding 1.115 0.577 0.942 83.876
0.954 15.465 1.087 65.845
ENSMUSG00000039313 AF529169 protein_coding 1.056 83.658
0.818 95.504 1.154 87.37 1.217 91.928
ENSMUSG00000039315 Clnk protein_coding 0.576 76.396 0.897
95.431 1.939 88.203 1.208 91.372
ENSMUSG00000039316 Rftn1 protein_coding 1.047 41.211 1.04
93.529 0.916 52.213 0.888 83.724
ENSMUSG00000039318 Rab3gap2 protein_coding 1.042 4.916 0.966
62.692 0.994 66.753 1.02 83.276
ENSMUSG00000039321 Uts2r protein_coding 0.45 36.945 1.999
85.501 1.114 65.235 1.196 85.577
ENSMUSG00000039323 Igfbp2 protein_coding 0.719 25.551

1.069 92.561 1.303 49.601 1.345 79.268
ENSMUSG00000039328 Rnf122 protein_coding 0.97 43.167
1.004 94.48 1.029 52.574 0.93 86.699
ENSMUSG00000039335 Spata16 protein_coding 1 84.738 1
94.262 1 79.524 1 92.564
ENSMUSG00000039337 Tex19.2 protein_coding 1 84.819 1
96.216 1 80.5 1 95.35
ENSMUSG00000039339 2010001E11Rik protein_coding 1.187 71.217
NA NA 0.913 87.744 0.77 88.387
ENSMUSG00000039342 Ankar protein_coding 1 83.09 1 97.155
1 86.985 1 96.139
ENSMUSG00000039345 Mettl22 protein_coding 0.912 38.827
1.126 64.304 0.973 56.625 1.028 83.067
ENSMUSG00000039347 Atp6v0e2 protein_coding 0.9 8.751 0.842
37.426 1.316 5.864 1.05 90.177
ENSMUSG00000039349 C130074G19Rik protein_coding 0.746 0.987
1.066 82.904 1.253 9.567 0.928 85.493
ENSMUSG00000039354 Smarcal1 protein_coding 1.044 69.489
0.976 79.475 0.978 80.015 0.952 86.991
ENSMUSG00000039356 Exosc2 protein_coding 1.01 32.873
0.974 87.452 1.014 86.666 1.123 74.193
ENSMUSG00000039357 Fut11 protein_coding 1.056 6.76 1.032 92.111
0.915 9.494 0.995 91.625
ENSMUSG00000039358 Drd5 protein_coding 1 85.499 1
95.329 1 84.552 1 96.183
ENSMUSG00000039361 Picalm protein_coding 1.126 17.492
0.935 87.147 0.95 53.224 0.98 93.734
ENSMUSG00000039364 Sectm1b protein_coding 1.044 82.092
1.352 83.104 0.71 38.799 0.776 83.724
ENSMUSG00000039367 Sec24c protein_coding 1.018 37.414
0.977 96.68 1.003 45.573 1.051 62.594
ENSMUSG00000039375 Wdr17 protein_coding 1.266 66.272 1.145
97.097 0.69 66.425 0.717 93.264
ENSMUSG00000039376 Synpo2l protein_coding 4.563 7.179 1.503
82.904 0.145 0.72 0.947 95.74
ENSMUSG00000039377 Hlx protein_coding 0.946 77.113 1.177
74.663 0.895 33.137 0.936 90.805
ENSMUSG00000039382 Wdr45 protein_coding 0.916 15.456 1.014
94.102 1.075 25.455 0.992 86.699
ENSMUSG00000039384 Dusp10 protein_coding 1.286 39.416 0.95
83.104 0.815 11.314 1.044 94.016
ENSMUSG00000039385 Cdh6 protein_coding 1.108 37.091 0.954
95.708 0.947 71.004 0.859 90.611
ENSMUSG00000039391 Ccdc81 protein_coding 0.856 76.968
1.378 90.895 0.849 84.071 0.687 85.493
ENSMUSG00000039395 Mreg protein_coding 1.551 3.498 1.273 77.598
0.506 0.72 1.671 63.708
ENSMUSG00000039396 Neil3 protein_coding 1.555 24.689 0.718
78.877 0.898 64.427 1.344 82.85
ENSMUSG00000039405 Prss23 protein_coding 1.184 12.396
0.932 93.654 0.903 39.755 1.021 88.657
ENSMUSG00000039410 Prdm16 protein_coding 1.031 84.534
1.032 72.642 0.937 24.918 1.09 83.067
ENSMUSG00000039414 Heatr5b protein_coding 0.978 36.823

0.984 96.847 1.041 49.058 1.011 77.621
ENSMUSG00000039419 Cntnap2 protein_coding 1.437 47.174
0.889 90.378 0.781 58.775 0.911 92.762
ENSMUSG00000039427 Alg1 protein_coding 0.938 33.224 NA NA
1.096 29.302 1.037 87.886
ENSMUSG00000039428 Tmem135 protein_coding 0.935 14.427
1.045 85.501 1.02 61.418 0.971 84.29
ENSMUSG00000039431 Mtmr7 protein_coding 1.095 46.533 1.038
95.708 0.876 79.255 1.037 96.287
ENSMUSG00000039438 Ttc36 protein_coding 1.262 33.854 1.026
83.58 0.772 88.657 0.813 83.724
ENSMUSG00000039449 Prpf18 protein_coding 0.96 33.906 0.99
97.049 1.053 42.065 1.008 96.358
ENSMUSG00000039450 Dcxr protein_coding 1.074 56.311 1.041
97.263 0.891 68.266 0.936 84.29
ENSMUSG00000039452 Snx22 protein_coding 0.889 32.378 0.96
92.631 1.176 50.636 0.929 87.182
ENSMUSG00000039456 Morc3 protein_coding 1.073 7.959 0.995 82.848
0.934 51.055 1.071 46.846
ENSMUSG00000039457 Ppl protein_coding 0.996 76.827 0.936
81.443 1.074 62.307 1.063 96.248
ENSMUSG00000039458 Mtmr12 protein_coding 1.104 33.555
0.883 54.06 1.024 64.305 1.022 83.067
ENSMUSG00000039461 Tcta protein_coding 0.883 8.45 1.067 79.055
1.058 21.462 1.052 85.786
ENSMUSG00000039463 Slc9a8 protein_coding 1.012 86.351
0.962 87.857 1.024 79.625 0.997 90.805
ENSMUSG00000039470 Zdhhc2 protein_coding 0.918 37.531
0.997 96.001 1.092 42.199 0.956 92.561
ENSMUSG00000039473 Ubn1 protein_coding 1.055 14.008 NA NA
0.968 22.735 0.967 84.19
ENSMUSG00000039474 Wfs1 protein_coding 1.088 4.925 1.133 71.222
0.811 0.72 1.052 92.903
ENSMUSG00000039476 Prrx2 protein_coding 1.873 1.129 0.913 96.268
0.586 1.705 1.406 46.846
ENSMUSG00000039477 Tnrc18 protein_coding 0.975 62.888
1.059 85.756 0.97 82.31 0.932 83.724
ENSMUSG00000039478 Micu3 protein_coding 1.026 75.399 0.988
96.984 0.986 84.669 1.029 82.091
ENSMUSG00000039480 Nt5dc1 protein_coding 1.014 85.59 0.971
88.897 1.012 88.657 0.968 93.584
ENSMUSG00000039481 Nrtn protein_coding 0.832 65.622 0.963
90.712 1.251 38.152 0.917 83.724
ENSMUSG00000039483 Asb6 protein_coding 0.909 3.009 1.106 72.546
0.997 51.243 0.982 95.74
ENSMUSG00000039485 Tspyl4 protein_coding 1.113 45.61 0.811
61.625 1.102 48.935 0.902 91.625
ENSMUSG00000039492 Ccdc27 protein_coding 0.9 76.968
0.827 85.242 1.344 44.125 0.926 93.132
ENSMUSG00000039496 Cdnf protein_coding 1.07 81.196 0.823
79.807 1.132 55.109 1.166 82.66
ENSMUSG00000039497 Dse protein_coding 1.171 1.009 1.007 95.809
0.845 2.361 1.073 65.845
ENSMUSG00000039501 Znfx1 protein_coding 1.025 43.654 1.096

76.086 0.891 9.347 1.089 74.193
ENSMUSG00000039509 Nup133 protein_coding 1.105 7.553 0.978
90.576 0.928 14.409 1.038 76.347
ENSMUSG00000039512 Uhrf1bp1 protein_coding 1.051 53.228
1.028 89.014 0.928 23.48 1.048 68.696
ENSMUSG00000039515 Ppp2r4 protein_coding 0.963 57.227
1.041 83.104 0.996 87.431 1.011 94.215
ENSMUSG00000039518 Cdsn protein_coding 0.575 1.017 1.395 63.504
1.244 25.93 1.002 92.181
ENSMUSG00000039519 Cyp7b1 protein_coding 1.476 14.59 1.119
79.579 0.605 1.58 1.346 49.239
ENSMUSG00000039521 Foxp3 protein_coding 0.758 16.183 1.103
84.126 1.193 38.926 1.051 90.463
ENSMUSG00000039523 Cep104 protein_coding 0.935 13.952
1.058 82.904 1.009 60.789 1.021 73.401
ENSMUSG00000039529 Atp8b1 protein_coding 1.317 0 0.879
55.71 0.863 7.329 1.102 57.1
ENSMUSG00000039530 Tusc3 protein_coding 1.136 6.61 0.995 93.654
0.882 4.538 1.098 48.119
ENSMUSG00000039531 Zufsp protein_coding 1.077 48.462 0.923
82.671 1.007 81.857 1.095 54.153
ENSMUSG00000039533 Mmd2 protein_coding 1.021 60.435 2.762
72.546 0.355 6.202 2.168 83.067
ENSMUSG00000039536 Stau1 protein_coding 1.019 58.38 1.011 94.367
0.971 46.575 0.985 93.457
ENSMUSG00000039539 Sgcz protein_coding 1 52.986 1
96.216 1 52.972 1 86.699
ENSMUSG00000039540 4921524L21Rik protein_coding 1 82.891
1 95.708 NA NA 1 96.358
ENSMUSG00000039542 Ncam1 protein_coding 0.917 39.612 1.2
71.98 0.907 55.9 1.14 60.31
ENSMUSG00000039543 Ttc18 protein_coding 0.997 81.24 0.971 93.654
1.028 85.464 0.789 85.493
ENSMUSG00000039545 4930524L23Rik antisense 0.889 46.289
0.854 60.678 1.322 16.446 0.844 90.157
ENSMUSG00000039546 Ajap1 protein_coding 0.304 28.998 1.392
82.904 2.361 75.155 2.416 92.263
ENSMUSG00000039555 Cylc2 protein_coding 1 77.407 1
96.843 1 86.985 1 95.273
ENSMUSG00000039556 Ppp1r3f protein_coding 0.862 6.155 0.96
93.765 1.207 8.586 1.011 96.358
ENSMUSG00000039563 2210406O10Rik processed_transcript 0.839
23.13 1.318 88.551 0.905 82.74 0.837 84.29
ENSMUSG00000039568 Ubald1 protein_coding 0.929 37.531
1.005 87.029 1.069 75.009 0.895 83.724
ENSMUSG00000039577 Nphp4 protein_coding 0.996 66.406 1.028
95.088 0.973 86.144 1.213 67.169
ENSMUSG00000039578 Ccser1 protein_coding 1.062 78.054
0.842 83.58 1.115 76.225 0.976 92.742
ENSMUSG00000039579 Grin3a protein_coding 1.285 63.385
0.976 97.263 0.797 39.079 1.166 60.466
ENSMUSG00000039585 Myo9a protein_coding 0.969 82.092 0.996
96.335 1.039 87.786 1.056 83.067
ENSMUSG00000039599 Fam149b protein_coding 0.949 5.544 1.1

72.642	0.954	86.666	0.928	83.724
ENSMUSG00000039601	Rcan2	protein_coding	1.218	40.333	0.893
87.857	0.918	71.734	1.036	95.832
ENSMUSG00000039607	Rbms3	protein_coding	0.966	65.199	1.081
90.454	0.958	84.669	0.927	85.493
ENSMUSG00000039608	Olfr303	protein_coding	1	86.333	1
95.941	1	78.864	1	96.248
ENSMUSG00000039611	Tmem246	protein_coding	1.057	58.896
1.004	97.155	0.941	64.427	0.867	84.29
ENSMUSG00000039615	Stub1	protein_coding	0.848	1.031	1.106	82.904
1.067	25.966	1.085	72.369
ENSMUSG00000039616	Mocos	protein_coding	1.135	1.292	1.029	95.809
0.858	2.294	0.976	85.493
ENSMUSG00000039617	Gm7488	pseudogene	1	82.982	1
96.763	1	82.778	1	95.92
ENSMUSG00000039620	6430573F11Rik	protein_coding	1.114	68.727
0.864	86.728	1.036	86.602	0.776	83.724
ENSMUSG00000039621	Prex1	protein_coding	1.073	45.096	0.899
56.781	1.033	58.414	0.938	83.724
ENSMUSG00000039623	Ap5z1	protein_coding	0.894	15.76	1.001	87.452
1.117	14.131	1.019	96.183
ENSMUSG00000039628	Hs3st6	protein_coding	0.898	34.305	1.22
71.617	0.911	87.686	0.96	93.045
ENSMUSG00000039629	Strip2	protein_coding	0.783	2.009	1.338
62.898	0.952	82.161	0.859	96.183
ENSMUSG00000039630	Hnrnpu	protein_coding	0.953	75.567
1.009	95.708	1.037	72.695	1.07	57.1
ENSMUSG00000039632	Ccdc151	protein_coding	1.021	81.52	0.875
81.731	1.122	63.665	0.804	87.331
ENSMUSG00000039633	Lonrf1	protein_coding	0.755	16.895	1.18
93.994	1.119	50.636	1.045	82.85
ENSMUSG00000039634	Zfp189	protein_coding	1.036	78.744
0.984	95.301	0.982	63.801	0.997	90.397
ENSMUSG00000039637	Coro7	protein_coding	0.981	65.199	1.043
91.584	0.978	78.642	1.089	32.045
ENSMUSG00000039640	Mrpl12	protein_coding	0.801	1.175	1.065
71.617	1.17	37.741	1.009	90.455
ENSMUSG00000039646	Vasn	protein_coding	1.178	7.959	0.997	96.843
0.849	15.465	0.96	92.181
ENSMUSG00000039648	Ccbl1	protein_coding	0.841	11.726	1.016
93.094	1.166	16.808	1.032	96.183
ENSMUSG00000039652	Cpeb3	protein_coding	0.989	48.462	1.113
82.76	0.909	11.64	0.989	92.181
ENSMUSG00000039653	Baat	protein_coding	1.285	75.399	0.944
92.631	0.824	82.74	0.818	95.92
ENSMUSG00000039656	Rxrb	protein_coding	0.983	84.865	1.027
93.707	0.992	76.768	0.935	83.724
ENSMUSG00000039660	D2Wsu81e	protein_coding	0.862	2.668	1.04
92.931	1.114	5.864	1.065	71.305
ENSMUSG00000039661	Dusp26	protein_coding	1.227	46.533
0.819	90.256	0.996	83.158	0.918	85.493
ENSMUSG00000039662	Icmt	protein_coding	0.933	7.881	1.044	71.98	1.03
56.931	0.985	95.273
ENSMUSG00000039670	Oxld1	protein_coding	1.079	40.333	0.932

90.744 0.994 84.552 0.929 84.29
ENSMUSG00000039671 Zmynd8 protein_coding 0.995 86.199
0.949 53.79 1.061 45.208 0.954 86.699
ENSMUSG00000039676 Capsl protein_coding 1.045 86.474 0.677
54.427 1.413 25.966 0.779 84.19
ENSMUSG00000039678 Tbc1d13 protein_coding 0.929 5.967 1.088
71.617 0.988 86.552 1.034 67.691
ENSMUSG00000039680 Mrps6 protein_coding 0.913 17.276 1.141
79.29 NA NA 0.986 91.011
ENSMUSG00000039682 Lap3 protein_coding 0.961 4.825 0.992 97.263
1.044 16.808 1.017 71.855
ENSMUSG00000039683 Sdk1 protein_coding 1.123 8.281 0.961 96.138
0.927 29.451 1.041 93.132
ENSMUSG00000039684 Gm5422 protein_coding 1 68.485 1
97.049 1 72.464 1 86.991
ENSMUSG00000039686 Zer1 protein_coding 0.993 83.333 0.983
95.088 1.026 72.148 0.96 83.724
ENSMUSG00000039693 Msantd3 protein_coding 1.027 80.688
0.885 65.819 1.103 18.411 0.974 92.742
ENSMUSG00000039697 Ncoa7 protein_coding 1.2 1.493 0.979 94.597
0.848 3.969 1.072 92.748
ENSMUSG00000039699 Batf2 protein_coding 0.845 12.624 1.12
79.29 1.056 85.352 0.978 94.904
ENSMUSG00000039701 Usp53 protein_coding 0.938 40.786 0.995
95.708 1.071 34.727 1.026 83.067
ENSMUSG00000039703 Nploc4 protein_coding 0.942 51.447
1.059 71.617 1.003 54.037 1.055 78.379
ENSMUSG00000039704 Lmbrd2 protein_coding 0.838 20.659 0.97
96.138 1.229 27.047 0.995 93.627
ENSMUSG00000039706 Ldb2 protein_coding 1.233 2.233 1.206 82.809
0.673 0.72 1.085 70.624
ENSMUSG00000039710 Slc7a12 protein_coding 1 85.499 1
91.661 1 74.325 1 95.72
ENSMUSG00000039713 Plekhg5 protein_coding 0.88 6.822 0.96
85.95 1.181 7.635 1.044 95.559
ENSMUSG00000039714 NA NA NA NA NA NA 1 88.601
1 92.691
ENSMUSG00000039715 Wdr34 protein_coding 0.884 3.009 1.031 96.407
1.099 13.047 1.006 95.832
ENSMUSG00000039716 Dock3 protein_coding 0.877 63.385 0.859
83.58 1.329 70.623 1.278 83.067
ENSMUSG00000039717 Ralyl protein_coding 1 74.958 1
93.765 1 77.17 1 95.877
ENSMUSG00000039720 Got1l1 protein_coding 1.755 24.253
0.604 84.017 0.94 82.575 0.845 89.294
ENSMUSG00000039725 2810408M09Rik protein_coding 0.963 49.749
0.999 96.948 1.037 50.091 0.977 93.612
ENSMUSG00000039728 Slc6a5 protein_coding 1 73.292 1
94.96 1 70.534 1 86.301
ENSMUSG00000039735 Fnbp1l protein_coding 0.959 30.035
1.016 95.941 1.027 42.199 0.955 86.991
ENSMUSG00000039737 Prkrip1 protein_coding 0.89 10.704
0.921 54.06 1.222 6.449 0.992 93.261
ENSMUSG00000039738 Slx4 protein_coding 1.108 3.176 1.166 60.309

0.777 0.72 1.06 69.965
ENSMUSG00000039740 Alg2 protein_coding 1.038 42.134 1.101
80.806 0.878 7.339 0.991 91.928
ENSMUSG00000039741 Bahcc1 protein_coding 0.736 5.686 1.274
60.309 1.064 75.009 0.989 94.99
ENSMUSG00000039745 Htatip2 protein_coding 0.897 22.142 0.98
86.277 1.136 13.174 0.989 93.584
ENSMUSG00000039747 Orai2 protein_coding 1.029 45.307 0.948
90.378 1.027 81.62 1.056 86.699
ENSMUSG00000039748 Exo1 protein_coding 1.601 41.211 0.751
85.116 0.83 87.744 1.691 51.908
ENSMUSG00000039753 Fbxl5 protein_coding 1.032 63.126 1.017
88.345 0.953 77.294 0.996 96.183
ENSMUSG00000039754 Alkbh4 protein_coding 0.978 74.482
1.008 87.604 1.011 79.776 1.068 86.209
ENSMUSG00000039756 Dnttip2 protein_coding 1.08 33.555
0.922 56.027 1 79.554 1.074 52.366
ENSMUSG00000039759 Thap3 protein_coding 0.888 18.379 0.96
80.052 1.17 10.523 0.985 84.29
ENSMUSG00000039760 Il22ra2 protein_coding 0.7 41.617
0.786 85.116 1.809 23.334 0.925 93.612
ENSMUSG00000039763 Dnajc28 protein_coding 1.097 46.875
0.992 92.404 0.921 63.919 0.986 94.215
ENSMUSG00000039765 Cc2d2a protein_coding 1.19 10.687
1.025 96.138 0.817 6.858 0.967 91.291
ENSMUSG00000039768 Dnajc11 protein_coding 0.927 2.009 0.98
95.708 1.099 7.635 1.017 80.324
ENSMUSG00000039770 Ypel5 protein_coding 1.103 46.097 0.795
65.819 1.136 71.615 1.049 79.268
ENSMUSG00000039771 Polr2j protein_coding 1.019 84.865
1.019 84.007 0.966 35.264 0.99 96.358
ENSMUSG00000039774 Galnt12 protein_coding 1.289 4.675 0.937
96.216 0.827 6.858 1.068 83.865
ENSMUSG00000039775 Defb3 protein_coding 1 83.523 NA NA
1 83.719 1 96.358
ENSMUSG00000039781 Azi1 protein_coding 0.835 3.623 NA NA
1.187 10.819 0.988 89.892
ENSMUSG00000039782 Cpeb2 protein_coding 1.009 84.865 0.968
86.538 1.023 62.237 0.946 93.964
ENSMUSG00000039783 Kmo protein_coding 1.469 3.152 0.823 38.923
0.828 55.9 0.891 84.29
ENSMUSG00000039785 Defb5 protein_coding 1 66.272 1
96.847 1 84.308 1 96.287
ENSMUSG00000039787 Cercam protein_coding 1.081 42.555
0.943 73.766 0.976 86.346 1.133 80.573
ENSMUSG00000039789 Zfp597 protein_coding 1.085 31.24 0.987
97.155 0.932 37.103 1.02 83.067
ENSMUSG00000039795 Zfand1 protein_coding 1.11 21.599
0.851 15.364 1.062 17.412 0.936 93.612
ENSMUSG00000039798 2600006K01Rik protein_coding 0.84 68.66
1.178 95.196 1.012 87.786 0.984 91.217
ENSMUSG00000039801 2410089E03Rik protein_coding 0.979 85.728
0.98 96.001 1.042 78.864 0.939 83.724
ENSMUSG00000039804 Ncoa5 protein_coding 1.011 70.36 1.03 96.013

0.955 77.704 0.997 94.99
ENSMUSG00000039809 Gabbr2 protein_coding 0.919 84.865
1.032 93.094 1.057 83.275 1.242 94.384
ENSMUSG00000039810 Zc3h10 protein_coding 0.887 0.987 1.069
61.032 1.051 30.645 1.033 83.067
ENSMUSG00000039813 Tbc1d2 protein_coding 0.821 5.544 1.049
87.609 1.156 37.369 0.902 83.724
ENSMUSG00000039814 Xkr5 protein_coding 1.736 6.135 0.719 72.576
0.803 58.775 1.108 94.654
ENSMUSG00000039824 Myl6b protein_coding 0.784 2.009 1.238 60.309
1.031 87.103 0.972 96.248
ENSMUSG00000039826 Trub2 protein_coding 0.975 67.477 0.984
95.708 1.044 74.325 0.983 89.587
ENSMUSG00000039828 Wdr70 protein_coding 0.874 6.484 0.972 85.418
1.18 10.76 1.096 48.119
ENSMUSG00000039831 Arhgap29 protein_coding 0.976 66.406
0.969 95.708 1.053 69.825 0.918 95.832
ENSMUSG00000039834 Zfp335 protein_coding 0.989 79.93 1.064
90.116 0.952 73.598 1.031 93.722
ENSMUSG00000039835 Nhsl1 protein_coding 0.865 11.973 0.957
88.704 1.211 5.864 0.971 86.699
ENSMUSG00000039840 Epg5 protein_coding 1.031 62.007 0.944
79.475 1.029 83.275 0.985 91.105
ENSMUSG00000039841 Zfp800 protein_coding 1.033 37.702
1.068 82.904 0.909 14.583 0.964 91.105
ENSMUSG00000039842 Mcph1 protein_coding 0.906 33.906 1.065
85.756 1.032 61.841 1.022 94.567
ENSMUSG00000039844 Rapgef1 protein_coding 1.012 78.054
1.046 72.642 0.946 17.258 0.995 95.72
ENSMUSG00000039849 Pcif1 protein_coding 0.884 15.456 1.042
87.029 1.084 60.104 0.991 86.445
ENSMUSG00000039850 Endov protein_coding 0.829 0.987 1.031 96.216
1.173 5.964 0.998 95.72
ENSMUSG00000039851 4932438H23Rik protein_coding 0.57 38.827
1.597 96.948 1.095 55.512 2.462 77.245
ENSMUSG00000039852 Rere protein_coding 1.053 50.627 1.015
95.211 0.938 41.506 0.964 96.183
ENSMUSG00000039853 Trim14 protein_coding 1.048 76.319
0.892 73.094 1.073 40.875 1.004 86.991
ENSMUSG00000039860 Srrm3 protein_coding 0.769 56.242 0.559 75.4
2.319 24.985 1.034 90.463
ENSMUSG00000039865 Slc44a3 protein_coding 0.845 3.641 0.977
96.847 1.209 11.665 0.959 96.358
ENSMUSG00000039873 Neurl2 protein_coding 1.11 13.105
0.993 91.897 0.909 37.617 0.997 96.358
ENSMUSG00000039878 Slc39a5 protein_coding 0.909 74.8 0.836
84.594 1.316 36.839 1.198 95.273
ENSMUSG00000039879 Heca protein_coding 1.084 13.363 0.9
60.678 1.023 85.757 0.921 77.012
ENSMUSG00000039883 Lrrc17 protein_coding 1.489 0.976 1.023
97.155 0.657 3.073 1.097 63.708
ENSMUSG00000039886 Tmem120a protein_coding 0.864 1.815 1.149
71.98 1.003 69.825 0.912 95.877
ENSMUSG00000039887 Alg14 protein_coding 1.081 27.32 0.979 65.819

0.944 74.853 0.959 93.685
ENSMUSG00000039891 Txlnb protein_coding 3.322 8.847 1.279 82.904
0.234 0.72 0.906 95.777
ENSMUSG00000039899 Fgl2 protein_coding 1.312 0 0.85 79.807
0.893 23.212 0.919 87.735
ENSMUSG00000039901 9130011E15Rik protein_coding 1.011 72
1.097 87.029 0.904 25.512 1.043 76.467
ENSMUSG00000039903 Eva1c protein_coding 1.024 85.283 1.134
89.434 0.862 49.217 0.937 85.493
ENSMUSG00000039908 Slc26a11 protein_coding 1.041 53.809
0.938 74.851 1.025 63.665 0.904 85.493
ENSMUSG00000039910 Cited2 protein_coding 0.838 7.574 1.035
88.923 1.149 13.259 0.891 83.724
ENSMUSG00000039911 Spsb1 protein_coding 1.275 1.846 0.912 68.848
0.863 19.765 1.096 73.823
ENSMUSG00000039914 Coq10a protein_coding 1.036 81.196
1.102 85.378 0.879 49.453 0.954 93.457
ENSMUSG00000039917 Rhbdd2 protein_coding 0.908 20.659
0.972 91.361 1.132 17.279 1.026 95.74
ENSMUSG00000039929 Urb1 protein_coding 1.073 19.746 0.973
76.978 0.955 80.015 0.943 86.445
ENSMUSG00000039934 Gsap protein_coding 1.155 20.659 1.064
96.001 0.809 36.786 0.968 86.699
ENSMUSG00000039936 Pik3cd protein_coding 1.377 0 0.861
81.02 0.845 7.647 1.049 72.826
ENSMUSG00000039942 Ptger4 protein_coding 1.068 62.354 1.04
96.843 0.898 65.688 0.904 88.387
ENSMUSG00000039943 Plcb4 protein_coding 0.853 1.274 0.99 95.301
1.185 5.864 1.01 90.674
ENSMUSG00000039951 Wfdc3 protein_coding 1 76.319 1
97.155 1 79.625 1 96.042
ENSMUSG00000039952 Dag1 protein_coding 0.983 41.021 1.02
85.756 0.994 88.729 0.965 83.724
ENSMUSG00000039953 Clstn1 protein_coding 1.075 16.436
0.964 95.088 0.963 24.918 0.968 86.991
ENSMUSG00000039954 Stk32a protein_coding 1.476 37.941
1.312 95.708 0.514 51.715 1.063 93.734
ENSMUSG00000039956 Mrap protein_coding 0.79 0.987 1.122 75.62
1.126 51.851 0.976 94.654
ENSMUSG00000039958 Mettl20 protein_coding 0.872 44.787
0.961 89.509 1.193 13.356 1.077 57.1
ENSMUSG00000039959 Hip1 protein_coding 0.991 69.24 NA NA 1.03
72.695 1.077 67.94
ENSMUSG00000039960 Rhou protein_coding 0.943 85.946 1.057
96.308 1.005 86.258 0.948 91.372
ENSMUSG00000039962 Olfr906 protein_coding 1 84.865 1
95.504 NA NA 1 96.183
ENSMUSG00000039963 Ccdc40 protein_coding 0.942 86.464
0.848 87.452 1.255 54.809 0.764 83.724
ENSMUSG00000039967 Zfp292 protein_coding 0.99 47.174
0.891 81.284 1.13 11.348 0.94 89.892
ENSMUSG00000039968 Rsbn1l protein_coding 0.997 67.867
0.969 88.705 1.03 54.809 0.96 84.29
ENSMUSG00000039976 Tbc1d16 protein_coding 1.061 17.492

1.058 84.832 0.893 6.858 0.865 84.29
ENSMUSG00000039977 Ccdc67 protein_coding 1.334 37.702
1.085 96.68 0.693 22.279 0.965 91.217
ENSMUSG00000039981 Zc3h12d protein_coding 1.341 24.689
0.931 92.355 0.799 43.842 1.087 95.559
ENSMUSG00000039982 Dtx4 protein_coding 0.993 72.743 0.972
90.29 NA NA 0.803 83.724
ENSMUSG00000039983 Ccdc32 protein_coding 0.991 82.231
1.043 95.196 0.965 73.027 1.011 76.347
ENSMUSG00000039985 Fam60a protein_coding 0.895 43.435
0.933 92.355 1.197 40.511 1.054 93.584
ENSMUSG00000039987 Phtf2 protein_coding 1.112 7.501 1.057 83.104
0.851 2.889 1.15 48.119
ENSMUSG00000039988 Ankrd13c protein_coding 0.998 83.169
1.053 72.642 0.949 20.751 0.956 83.724
ENSMUSG00000039989 Cbx4 protein_coding 1.068 62.689 1.263
71.222 0.74 1.58 0.826 83.724
ENSMUSG00000039990 2700050L05Rik protein_coding 1.03 77.497
0.974 95.504 0.994 86.346 0.96 85.947
ENSMUSG00000039994 Timeless protein_coding 1.123 33.854
1.104 92.561 0.806 19.449 1.178 44.213
ENSMUSG00000039997 Ifi203 protein_coding 1.218 3.176 1.061
96.013 0.775 21.852 1.055 64.513
ENSMUSG00000040003 Magi2 protein_coding 0.977 70.213 1.107
90.68 0.921 79.524 0.947 96.287
ENSMUSG00000040006 Ginm1 protein_coding 0.925 24.817 NA NA
1.06 40.575 0.976 95.74
ENSMUSG00000040007 Bahd1 protein_coding 1 80.51 1.029 82.904
0.974 58.271 0.952 86.991
ENSMUSG00000040009 Gnaz protein_coding 0.822 43.167 1.213
71.617 0.999 79.776 0.879 86.699
ENSMUSG00000040010 Slc7a5 protein_coding 1.262 38.386
0.845 38.699 0.934 87.744 1.218 55.841
ENSMUSG00000040016 Ptger3 protein_coding 0.871 81.01 1.295
82.904 0.887 76.388 1.01 91.979
ENSMUSG00000040018 Cox15 protein_coding 0.967 63.126 1.029
92.793 1.005 86.258 1.05 73.401
ENSMUSG00000040021 Lats1 protein_coding 1.058 14.194 0.995
95.504 0.952 24.163 1.002 86.254
ENSMUSG00000040022 Rab11fip2 protein_coding 0.973 53.138
0.984 93.654 1.04 40.511 0.995 96.287
ENSMUSG00000040025 Ythdf2 protein_coding 1.114 22.205
1.027 95.735 0.872 16.719 0.994 95.74
ENSMUSG00000040026 Saa3 protein_coding 0.733 56.242 1.068
96.407 NA NA 1.007 92.561
ENSMUSG00000040028 Elavl1 protein_coding 1.131 18.635
1.006 95.431 0.88 14.409 1.021 60.466
ENSMUSG00000040029 Ipo8 protein_coding 1.046 21.599 1.006
89.996 0.95 15.778 0.985 93.156
ENSMUSG00000040033 Stat2 protein_coding 1.161 28.395 1.099
83.104 0.783 21.331 1.078 49.769
ENSMUSG00000040034 Nup43 protein_coding 1.249 2.655 0.939 83.392
0.853 15.024 1.014 77.245
ENSMUSG00000040035 Disp2 protein_coding 0.717 17.276 1.367

64.304	NA	NA	1.947	54.265
ENSMUSG00000040037	Negr1	protein_coding	0.882	32.168	1.013
93.899	1.115	78.498	0.981	94.608
ENSMUSG00000040040	Ift88	protein_coding	1.022	74.147	0.921
71.889	1.058	41.331	0.986	96.183
ENSMUSG00000040043	Rbms2	protein_coding	0.89	22.731	1.005
88.533	1.12	54.809	0.962	88.053
ENSMUSG00000040044	Orc3	protein_coding	1.048	35.024	1.004
94.48	0.95	52.213	0.973	94.001
ENSMUSG00000040046	Tph1	protein_coding	1.566	62.689	0.591
90.712	1.082	79.524	1.538	74.047
ENSMUSG00000040048	Ndufb10	protein_coding	0.873	7.118	1.052
90.454	1.092	30.561	1.003	95.273
ENSMUSG00000040054	Baz2a	protein_coding	0.948	15.486	1.037
85.756	1.019	57.286	0.972	86.254
ENSMUSG00000040055	Gjb6	protein_coding	0.32	85.728	0.692
87.857	4.52	54.809	6.989	67.169
ENSMUSG00000040061	Plcb2	protein_coding	1.078	51.737	1.414
71.617	0.657	6.858	0.836	85.636
ENSMUSG00000040065	Pfpl	protein_coding	1	74.745	1
94.262	1	86.112	1	96.287
ENSMUSG00000040084	Bub1b	protein_coding	1.08	29.057	0.973
85.116	0.949	67.962	1.302	92.018
ENSMUSG00000040086	Tnni3k	protein_coding	1	64.893	1
96.897	1	78.369	1	85.629
ENSMUSG00000040093	Bmf	protein_coding	1.024	79.577	0.794
70.693	1.226	55.81	0.8	92.675
ENSMUSG00000040097	Flywch1	protein_coding	0.911	6.632	1.125
79.29	0.974	80.015	0.911	83.724
ENSMUSG00000040102	Klhl42	protein_coding	1.037	18.635
1.073	72.642	0.895	1.58	0.946	83.724
ENSMUSG00000040105	Ppapdc2	protein_coding	0.782	2.009	1.124
71.617	1.133	33.508	1.067	95.273
ENSMUSG00000040111	Gramd1b	protein_coding	0.955	84.738
0.793	61.883	1.321	32.524	1.003	93.964
ENSMUSG00000040112	Mrps35	protein_coding	1.019	62.007
0.972	73.166	1.01	54.363	0.976	92.742
ENSMUSG00000040113	Mettl11b	protein_coding	1	73.734	1
91.776	1	47.12	1	95.72
ENSMUSG00000040118	Cacna2d1	protein_coding	1.204	12.295
1.035	83.104	0.805	1.48	1.045	81.56
ENSMUSG00000040121	Rep15	protein_coding	0.736	14.221	1.089
96.407	1.243	28.603	1.16	86.445
ENSMUSG00000040123	Zmym5	protein_coding	1.009	84.977	0.989
83.58	1	61.841	0.927	86.445
ENSMUSG00000040124	Gorab	protein_coding	1.038	76.546	0.975
94.96	0.989	79.776	1.061	83.067
ENSMUSG00000040125	Gpr26	protein_coding	1	84.534	1
90.138	1	88.203	1	96.183
ENSMUSG00000040128	Pnrc1	protein_coding	0.989	81.196	1.066
80.806	0.946	39.079	0.988	96.071
ENSMUSG00000040133	Gpr176	protein_coding	1.258	9.695	0.867
91.897	0.919	44.211	1.172	96.139
ENSMUSG00000040134	Rdh7	protein_coding	1	81.925	NA	NA

1 82.436 1 92.762
ENSMUSG00000040136 Abcc8 protein_coding 0.677 2.174 1.123 75.21
1.312 20.315 0.965 92.181
ENSMUSG00000040138 Ndp protein_coding 0.977 64.284 0.979
90.378 1.045 57.907 1.16 83.067
ENSMUSG00000040139 9430038I01Rik protein_coding 0.922 49.749
0.897 74.483 1.21 30.948 1.418 55.841
ENSMUSG00000040140 Tdrd6 protein_coding 0.633 65.388 0.78
87.857 2.029 33.042 1.084 96.287
ENSMUSG00000040146 Rgl3 protein_coding 0.909 13.199 1.03
96.335 1.067 38.099 0.852 83.724
ENSMUSG00000040147 Maob protein_coding 0.939 51.447 NA NA
1.043 58.414 0.992 92.675
ENSMUSG00000040148 Hmx3 protein_coding 1 85.355 1
96.013 1 87.744 1 96.086
ENSMUSG00000040151 Hs2st1 protein_coding 1.065 1.323 1.048
95.735 0.894 1.342 0.982 91.372
ENSMUSG00000040152 Thbs1 protein_coding 1.349 30.497 0.813
68.818 0.909 70.791 0.983 93.413
ENSMUSG00000040158 Tax1bp3 protein_coding 1.03 67.163
1.102 79.579 0.881 12.41 0.997 95.777
ENSMUSG00000040163 1700034J05Rik protein_coding 1 78.299
1 96.763 1 85.58 1 95.877
ENSMUSG00000040164 Kcns1 protein_coding 0.552 20.369 0.89
88.897 2.04 22.131 1.112 93.722
ENSMUSG00000040165 Cd209c protein_coding 1.053 86.819
0.917 85.921 1.037 55.352 0.999 92.675
ENSMUSG00000040167 Ikzf5 protein_coding 1.061 22.349 1.026
89.494 0.921 7.742 0.984 95.72
ENSMUSG00000040170 Fmo2 protein_coding 0.996 84.661 0.687
82.503 1.463 38.926 1.224 83.581
ENSMUSG00000040174 Alkbh3 protein_coding 0.921 19.595 NA
NA 1.112 31.4 1.039 70.85
ENSMUSG00000040177 2310057M21Rik protein_coding 0.996 67.163
0.951 96.216 1.057 83.397 1.082 73.401
ENSMUSG00000040181 Fmo1 protein_coding 0.801 48.354 0.934
96.216 1.338 77.17 1.093 49.806
ENSMUSG00000040183 Ankrd6 protein_coding 0.968 84.397
1.034 96.68 0.995 84.83 0.92 88.505
ENSMUSG00000040187 Arntl2 protein_coding 0.965 69.715
0.856 71.889 1.212 10.76 0.918 91.105
ENSMUSG00000040188 Scamp2 protein_coding 1.139 0 NA NA
0.871 2.889 0.999 92.903
ENSMUSG00000040189 Ccdc114 protein_coding 0.782 0.987 1.009
96.68 1.264 5.864 1.08 83.276
ENSMUSG00000040195 Tmem194 protein_coding 1.061 39.075
1.006 96.378 0.939 41.662 1.006 95.405
ENSMUSG00000040197 Cd209e protein_coding 1.07 38.195 1.32
95.196 0.706 16.949 1.066 95.35
ENSMUSG00000040204 2810417H13Rik protein_coding 1.238 40.635
0.888 87.313 0.909 78.045 1.749 50.74
ENSMUSG00000040209 Zfp704 protein_coding 0.992 65.733
0.954 78.877 1.056 62.237 0.917 83.724
ENSMUSG00000040212 Emp3 protein_coding 1.499 0 0.881 88.705

0.754 0.72 1.229 64.513
ENSMUSG00000040213 Ccbl2 protein_coding 1.059 43.654 0.973
96.378 0.972 64.155 0.949 91.105
ENSMUSG00000040217 Gm6369 pseudogene 1 76.135 1
96.847 NA NA 1 96.183
ENSMUSG00000040219 Ttc12 protein_coding 1.297 5.848 0.978 93.654
0.79 13.848 0.908 85.493
ENSMUSG00000040220 Gas8 protein_coding 0.917 26.013 0.955
92.137 1.144 26.498 1.049 69.708
ENSMUSG00000040225 Prrc2c protein_coding 1.027 67.163
0.883 75.618 1.102 63.801 1.001 93.612
ENSMUSG00000040229 Gpr34 protein_coding 1.822 2.651 0.825 76.431
0.664 13.848 0.743 83.952
ENSMUSG00000040231 Syngr4 protein_coding 0.908 80.146 NA
NA 1.017 83.925 1.053 91.872
ENSMUSG00000040234 Tm7sf3 protein_coding 1.189 0 1
85.671 0.842 0.72 0.95 83.724
ENSMUSG00000040236 Trappc5 protein_coding 0.984 84.977
0.992 95.088 1.019 86.666 1.016 90.289
ENSMUSG00000040242 Fgfr1op2 protein_coding 1.014 74.482
0.996 94.848 0.988 75.967 1.09 32.045
ENSMUSG00000040247 Tbc1d10c protein_coding 0.968 82.982
1.184 85.756 0.867 40.794 0.944 83.724
ENSMUSG00000040249 Lrp1 protein_coding 1.02 55.789 0.996
96.268 0.984 42.621 1.439 40.426
ENSMUSG00000040250 Asun protein_coding 1.012 78.639 0.989
97.049 0.999 71.004 0.981 94.174
ENSMUSG00000040253 Gbp7 protein_coding 1.124 23.019 NA NA
0.922 19.552 1.08 96.358
ENSMUSG00000040254 Sema3d protein_coding 0.869 63.126
1.086 94.89 1.053 88.161 1.14 70.624
ENSMUSG00000040258 Nxph4 protein_coding 1.58 46.097 1.728
86.34 0.367 15.024 0.732 90.611
ENSMUSG00000040260 Daam2 protein_coding 0.964 46.289 1.068
90.116 0.97 87.37 0.997 93.264
ENSMUSG00000040263 Klhdc4 protein_coding 0.805 0.987 1.086
82.904 1.141 10.523 1.121 31.133
ENSMUSG00000040264 Gbp5 protein_coding 1.174 6.683 1.234 83.104
0.687 3.943 1.018 95.273
ENSMUSG00000040265 Dnm3 protein_coding 1.102 19.477 1.075
93.707 0.844 17.662 1.014 96.086
ENSMUSG00000040268 Plekha1 protein_coding 1.109 2.085 0.987
95.504 0.913 20.751 0.921 84.29
ENSMUSG00000040269 Mrps28 protein_coding 0.933 8.751 NA NA
1.104 19.286 0.948 96.287
ENSMUSG00000040270 Bach2 protein_coding 1.006 71.36 0.95 78.877
1.045 46.575 0.927 86.445
ENSMUSG00000040272 Accs protein_coding 1.029 78.909 0.908
71.41 1.07 23.454 0.858 84.29
ENSMUSG00000040274 Cdk6 protein_coding 0.964 71.915 0.77
84.017 1.348 30.645 0.916 89.892
ENSMUSG00000040276 Pacsin1 protein_coding 0.976 79.165
1.055 90.389 0.969 81.525 1.241 90.289
ENSMUSG00000040280 Ndufa4l2 protein_coding 0.925 61.334

1.212 72.546 0.889 45.573 1.084 94.904
ENSMUSG00000040282 BC052040 protein_coding 1.239 11.459
0.911 85.242 0.884 33.971 1.084 83.581
ENSMUSG00000040283 Btnl9 protein_coding 0.906 48.354 0.972
87.998 1.137 20.085 0.912 83.724
ENSMUSG00000040284 Gzmg protein_coding 1 67.374 1
96.68 1 83.538 1 95.273
ENSMUSG00000040287 Stac3 protein_coding 1.344 36.124 1.131
85.501 0.656 14.206 0.898 95.273
ENSMUSG00000040289 Hey1 protein_coding 0.632 1.175 0.954 95.504
1.659 7.635 0.953 94.99
ENSMUSG00000040296 Ddx58 protein_coding 1.025 34.861 NA NA
0.992 43.842 1.105 77.854
ENSMUSG00000040297 Suco protein_coding 1.116 3.393 0.917 34.058
0.978 57.091 1 87.7
ENSMUSG00000040298 Btbd16 protein_coding 2.509 37.702
0.938 95.504 0.426 48.387 0.519 88.955
ENSMUSG00000040302 Rbm48 protein_coding 0.928 25.492 1.018
96.843 1.06 40.511 1.023 85.577
ENSMUSG00000040310 Alx4 protein_coding 2.15 1.815 1.069 97.097
0.436 6.858 1.403 44.882
ENSMUSG00000040312 Cchcr1 protein_coding 0.917 43.495
0.994 96.897 1.097 54.673 0.955 88.245
ENSMUSG00000040321 Zfp770 protein_coding 1.124 16.436
1.016 95.301 0.878 21.852 1.002 96.192
ENSMUSG00000040322 Slc25a24 protein_coding 1.244 0.355 0.902
79.807 0.888 12.907 1.04 80.573
ENSMUSG00000040323 Gm15429 pseudogene 1.353 84.564 1.144
83.104 0.644 42.997 0.811 90.628
ENSMUSG00000040325 Vprbp protein_coding 1.06 19.746 0.978
93.654 0.962 57.446 0.981 95.92
ENSMUSG00000040327 Cul9 protein_coding 0.924 19.595 0.96
84.594 1.13 16.666 0.946 87.705
ENSMUSG00000040328 Olfr56 protein_coding 2.163 7.908 0.548
68.818 0.845 86.727 1.122 92.018
ENSMUSG00000040329 Il7 protein_coding 1.035 47.174 1.211
83.104 0.796 20.009 0.856 84.19
ENSMUSG00000040331 Nsmce4a protein_coding 0.988 79.068
0.997 93.654 1.018 81.492 1.111 69.965
ENSMUSG00000040339 Fam102b protein_coding 1.151 10.391
1.001 96.216 0.867 14.583 1.042 96.042
ENSMUSG00000040340 1700019B03Rik protein_coding 0.273 53.138
1.157 96.843 3.176 57.227 2.389 94.016
ENSMUSG00000040345 Arhgap9 protein_coding 1.273 7.174 0.892
61.883 0.877 48.577 0.994 95.72
ENSMUSG00000040350 Trim7 protein_coding 0.899 49.749 1.161
83.104 0.955 78.293 0.929 89.892
ENSMUSG00000040351 Ankib1 protein_coding 1.052 38.195
1.029 87.185 0.924 23.1 1.062 82.85
ENSMUSG00000040354 Mars protein_coding 0.87 4.055 1.009 96.013
1.134 13.259 1.059 57.075
ENSMUSG00000040356 Skiv2l protein_coding 0.815 0.987 1.038
75.612 1.181 5.864 1.08 54.153
ENSMUSG00000040359 Ufl1 protein_coding 1.06 14.966 0.978

87.452	0.966	32.703	0.996	87.134
ENSMUSG00000040363	Bcor	protein_coding	1.064	49.517	1.118
63.504	0.843	0.72	0.942	88.505
ENSMUSG00000040364	Sec1	protein_coding	1.419	82.352	0.778
91.897	0.906	86.144	1.129	91.928
ENSMUSG00000040365	Trim41	protein_coding	0.927	51.056
1.026	93.654	1.053	40.052	1.003	94.608
ENSMUSG00000040367	Lrrd1	protein_coding	0.673	12.785	1.252
82.904	1.188	64.305	1.036	89.72
ENSMUSG00000040370	Lyrm5	protein_coding	0.964	48.646	0.897
86.728	1.158	22.131	1.061	63.708
ENSMUSG00000040372	NA	NA	NA	NA	0.953	97.049	NA	NA
1.132	82.66
ENSMUSG00000040374	Pex2	protein_coding	1.029	14.966	0.971
87.147	0.995	64.155	0.983	85.493
ENSMUSG00000040380	Cbln3	protein_coding	1.121	26.338	0.796
58.64	1.119	76.498	0.977	89.587
ENSMUSG00000040383	Aqr	protein_coding	0.992	86.819	1.038
83.464	0.97	28.237	1.037	77.621
ENSMUSG00000040385	Ppp1ca	protein_coding	0.957	29.21	1.032
79.055	1.013	84.552	0.951	94.654
ENSMUSG00000040387	Klhl32	protein_coding	0.803	79.93	2.408
83.104	0.517	42.621	1.385	91.928
ENSMUSG00000040389	Wdr47	protein_coding	1.024	61.171	1.034
82.103	0.943	14.583	1.029	95.74
ENSMUSG00000040390	Map3k10	protein_coding	0.861	5.686	1.075
93.574	1.081	14.169	0.966	92.31
ENSMUSG00000040396	Abhd13	protein_coding	1.036	62.689
0.966	88.897	0.993	85.675	0.964	85.493
ENSMUSG00000040399	C630050I24Rik	protein_coding	1	85.946
1	95.088	1	84.896	1	96.071
ENSMUSG00000040407	Akap9	protein_coding	0.842	4.268	0.938	86.728
1.264	6.202	1.067	92.31
ENSMUSG00000040410	Fbxl4	protein_coding	1.106	15.602	0.975
88.705	0.928	27.321	0.905	83.724
ENSMUSG00000040412	5330417C22Rik	protein_coding	1.001	73.401
0.931	83.733	1.074	79.426	0.795	83.724
ENSMUSG00000040413	Timd2	protein_coding	0.497	8.365	1.563	86.277
1.28	44.787	0.742	83.724
ENSMUSG00000040414	Slc25a28	protein_coding	0.957	82.479
0.978	96.138	1.07	80.015	0.961	86.991
ENSMUSG00000040415	Dtx3	protein_coding	0.887	1.15	0.962	95.088
1.174	7.024	0.971	90.157
ENSMUSG00000040420	Cdh18	protein_coding	1	82.543	NA	NA
1	88.601	1	96.071
ENSMUSG00000040423	Rc3h1	protein_coding	0.911	11.519	1.038
83.104	1.059	38.099	0.99	91.979
ENSMUSG00000040424	Hipk4	protein_coding	0.65	5.946	1.097	95.431
1.402	24.791	0.957	93.584
ENSMUSG00000040428	Plekha4	protein_coding	1.012	71.36	0.972
93.574	1.017	83.811	1.083	46.412
ENSMUSG00000040429	Mterf	protein_coding	0.911	38.555	1.023
95.708	1.074	34.727	1.03	92.381
ENSMUSG00000040430	Pitpnc1	protein_coding	1.125	4.228	0.976

91.661 0.908 3.428 1.033 93.734
ENSMUSG00000040432 Ltb4r2 protein_coding 0.852 85.637 NA
NA NA NA 0.713 92.31
ENSMUSG00000040433 Zbtb38 protein_coding 1.024 67.374
0.978 88.345 0.995 73.598 0.953 84.29
ENSMUSG00000040434 Gyltl1b protein_coding 0.884 23.641
1.023 96.216 1.104 30.213 0.961 88.552
ENSMUSG00000040435 Ppp1r15a protein_coding 0.981 51.056
0.935 72.576 1.09 27.533 0.953 88.505
ENSMUSG00000040441 Slc26a10 protein_coding 1.617 0 0.827
55.71 0.747 2.333 0.941 91.105
ENSMUSG00000040446 Rprd1a protein_coding 0.998 76.686
0.938 34.058 1.066 18.208 1.014 73.368
ENSMUSG00000040447 Spns2 protein_coding 0.934 25.85 1.064 83.464
1.007 79.426 0.952 84.29
ENSMUSG00000040451 Sgms1 protein_coding 1.143 43.654 0.834
20.272 1.044 29.777 1.036 86.118
ENSMUSG00000040452 Cdh12 protein_coding 1 80.408 1
94.597 1 86.112 1 94.19
ENSMUSG00000040455 Usp45 protein_coding 0.922 26.013 1.005
96.68 1.074 47.776 1.119 31.133
ENSMUSG00000040456 1700054O13Rik protein_coding 1 85.092
NA NA 1 86.144 1 94.016
ENSMUSG00000040459 Arglu1 protein_coding 1.013 76.319 0.96
53.434 1.028 24.383 0.991 94.19
ENSMUSG00000040462 Os9 protein_coding 0.952 71.142 0.982
81.284 1.068 43.842 0.971 86.699
ENSMUSG00000040463 Mybbp1a protein_coding 0.909 25.492
0.939 52.266 1.17 8.586 1.076 46.846
ENSMUSG00000040464 Gtpbp10 protein_coding 1.01 46.013
0.982 72.399 1.011 64.624 0.973 86.699
ENSMUSG00000040466 Blvrb protein_coding 0.991 81.657 1.044
92.561 0.967 65.688 0.974 92.181
ENSMUSG00000040467 4921522P10Rik protein_coding 1 71.915
1 83.104 1 79.776 1 90.289
ENSMUSG00000040471 Ggt6 protein_coding 1.024 65.537 0.807
72.399 1.211 60.789 0.757 83.724
ENSMUSG00000040472 Rabggta protein_coding 0.94 31.614
1.038 90.895 1.027 51.851 1.002 92.564
ENSMUSG00000040473 A330021E22Rik protein_coding 0.984 38.286
0.919 92.355 1.101 30.107 0.747 84.29
ENSMUSG00000040478 Prdm13 protein_coding 1 80.51 1
93.994 1 85.675 1 92.675
ENSMUSG00000040479 Dgkz protein_coding 0.988 49.944 0.973
94.102 1.04 49.058 0.846 83.724
ENSMUSG00000040481 Bptf protein_coding 1.111 5.481 0.978 90.046
0.922 10.726 0.944 86.699
ENSMUSG00000040482 Dxo protein_coding 0.841 8.122 1.071 72.546
1.11 57.286 1.057 93.264
ENSMUSG00000040483 Xaf1 protein_coding 1.091 35.787 1.023
96.013 0.896 49.453 1.171 43.377
ENSMUSG00000040488 Ltbp4 protein_coding 1.033 46.013 0.907
72.399 1.069 72.378 0.999 93.584
ENSMUSG00000040489 Sox30 protein_coding 1.043 59.527 1.411

82.904	0.679	18.965	0.932	93.612
ENSMUSG00000040490	Lrfn2	protein_coding	1.422	11.879	1.682
60.309	0.417	0.72	1.061	90.455
ENSMUSG00000040495	Chrm4	protein_coding	0.883	63.126	NA	NA
1.369	66.928	1.177	95.74
ENSMUSG00000040502	9-Mar	protein_coding	0.907	81.125	1.118
83.819	0.987	36.786	0.995	83.724
ENSMUSG00000040505	Abcg5	protein_coding	0.946	34.954	0.826
95.088	1.28	25.966	0.879	87.624
ENSMUSG00000040506	Ambra1	protein_coding	1.005	58.56	0.998
96.138	0.995	77.888	0.955	90.611
ENSMUSG00000040511	Pvr	protein_coding	0.956	41.759	1.069
71.617	0.981	76.768	1.012	94.654
ENSMUSG00000040514	4921511H03Rik	protein_coding	1	84.977
1	91.661	1	87.431	1	91.372
ENSMUSG00000040520	Manea	protein_coding	1.003	51.189	1.041
96.847	0.96	66.201	0.981	92.181
ENSMUSG00000040521	Tsfm	protein_coding	0.881	2.142	1.117	60.309
1.014	75.967	0.97	90.611
ENSMUSG00000040522	Tlr8	protein_coding	1.781	0	0.871	70.971
0.647	5.701	1.062	93.964
ENSMUSG00000040524	Zfp609	protein_coding	1.029	51.737
0.979	91.532	0.99	57.819	0.983	92.561
ENSMUSG00000040525	Cblc	protein_coding	0.866	15.912	0.887
79.475	1.303	18.208	0.838	83.724
ENSMUSG00000040528	Milr1	protein_coding	1.352	2.361	1.011	93.817
0.729	10.602	1.214	43.377
ENSMUSG00000040532	Abhd11	protein_coding	0.874	1.031	1.05
72.546	1.093	22.444	0.997	94.19
ENSMUSG00000040533	Matn1	protein_coding	1	84.564	NA	NA
1	73.759	1	92.018
ENSMUSG00000040536	Necab1	protein_coding	0.804	53.562	0.4
56.955	3.12	46.206	1.958	94.904
ENSMUSG00000040537	Adam22	protein_coding	1.105	35.499	NA
NA	0.918	22.009	1.071	82.85
ENSMUSG00000040540	Gm9770	pseudogene	1	80.815	NA	NA
1	70.623	1	95.72
ENSMUSG00000040541	Tmem225	protein_coding	1	81.52	1
96.308	1	76.225	1	87.7
ENSMUSG00000040543	Pitpnm3	protein_coding	0.973	72.546
1.066	97.155	0.961	84.626	0.912	83.724
ENSMUSG00000040548	Tex2	protein_coding	1.095	27.545	1.073
63.504	0.852	4.963	1.045	92.564
ENSMUSG00000040549	Ckap5	protein_coding	0.992	68.955	0.938
77.598	1.072	45.208	1.026	66.944
ENSMUSG00000040550	Otud6b	protein_coding	1.017	69.089
1.016	88.923	0.969	21.666	1.077	44.882
ENSMUSG00000040552	C3ar1	protein_coding	1.589	0	0.951	81.731
0.659	3.123	1.062	93.156
ENSMUSG00000040554	Aipl1	protein_coding	1.316	69.489	0.729
72.576	1.043	83.066	0.914	92.389
ENSMUSG00000040557	Wbscr27	protein_coding	1.003	82.891
0.918	85.116	1.086	50.636	0.972	86.254
ENSMUSG00000040560	Wdr7	protein_coding	0.981	82.543	1.087

83.104 0.933 34.21 1.048 60.466
ENSMUSG00000040562 Gstm2 protein_coding 0.846 13.45 1.15 60.309
1.026 88.349 1.126 88.755
ENSMUSG00000040563 BC018242 protein_coding 0.974 44.562
1.057 85.985 NA NA 0.924 86.991
ENSMUSG00000040564 Apoc1 protein_coding 0.727 3.641 1.167 75.612
1.175 43.481 0.874 88.505
ENSMUSG00000040565 Btaf1 protein_coding 1.072 63.534 1.029
97.155 0.903 66.753 1.093 83.067
ENSMUSG00000040569 Slc26a7 protein_coding 1.419 74.516
0.947 95.196 0.746 79.255 0.844 91.291
ENSMUSG00000040570 Rundc3b protein_coding 1.232 34.439
1.047 95.211 0.773 45.573 1.302 86.209
ENSMUSG00000040576 Wbscr28 protein_coding 1 78.224 NA
NA 1 86.552 1 96.287
ENSMUSG00000040583 Cyp2b13 protein_coding 1 82.231 1
95.708 1 48.451 1 88.797
ENSMUSG00000040584 Abcb1a protein_coding 1.287 0.976 0.924
87.452 0.839 27.86 1.055 89.428
ENSMUSG00000040586 Ofd1 protein_coding 1.015 56.311 0.936
54.06 1.054 43.307 1.092 92.903
ENSMUSG00000040591 1110051M20Rik protein_coding 0.841 7.218
1.092 81.127 1.09 34.07 0.994 86.991
ENSMUSG00000040592 Cd79b protein_coding 1.339 24.253 0.954
90.378 0.781 38.534 1.238 92.381
ENSMUSG00000040594 Ranbp17 protein_coding 1.235 12.072
1.041 83.104 0.776 4.48 1.021 87.447
ENSMUSG00000040596 Pogk protein_coding 1.006 74.482 1.032
83.104 0.962 42.997 0.987 86.699
ENSMUSG00000040599 Mis12 protein_coding 1.144 5.109 1.031 96.847
0.846 3.073 1.003 82.66
ENSMUSG00000040600 Eps8l3 protein_coding 1.008 76.686 NA
NA 0.964 86.258 0.733 87.735
ENSMUSG00000040601 Nlrp4a protein_coding 1 82.732 1
97.263 1 88.601 1 94.016
ENSMUSG00000040605 Bace2 protein_coding 1.119 2.446 1.046 83.104
0.852 1.58 1.014 92.181
ENSMUSG00000040606 Kazn protein_coding 0.941 55.939 1.022
96.407 1.043 74.686 0.928 86.699
ENSMUSG00000040612 Ildr2 protein_coding 1.025 86.474 0.961
91.532 1.011 85.935 1.05 73.401
ENSMUSG00000040613 Apobec1 protein_coding 1.328 0.664 0.828
50.715 0.907 26.369 1.038 89.72
ENSMUSG00000040614 Nlrp9c protein_coding 1 86.474 1
96.138 1 81.525 1 96.358
ENSMUSG00000040616 Tmem51 protein_coding 1.121 57.133
0.871 74.483 1.027 80.634 0.8 83.952
ENSMUSG00000040618 Pck2 protein_coding 0.933 80.608 0.82
27.163 1.303 10.819 0.921 93.045
ENSMUSG00000040620 Dhx33 protein_coding 1.015 71.516 0.983
95.088 1.004 86.112 0.99 96.183
ENSMUSG00000040621 Gemin8 protein_coding 0.99 81.76 0.939
73.766 1.075 43.185 1.076 89.108
ENSMUSG00000040624 Plekhg1 protein_coding 0.954 40.217

0.989 95.301 1.058 45.035 0.917 84.29
ENSMUSG00000040631 Dok4 protein_coding 0.934 28.584 1.061
79.261 1.009 84.724 0.93 83.724
ENSMUSG00000040632 Nrl protein_coding 1 75.858 1
95.708 1 77.17 1 92.263
ENSMUSG00000040640 Erc2 protein_coding 1.467 48.857 0.957
90.712 0.711 69.583 0.889 95.877
ENSMUSG00000040648 Ppip5k2 protein_coding 1.022 78.639
0.989 90.287 0.989 72.853 1.038 82.85
ENSMUSG00000040649 Rimklb protein_coding 1.022 68.485
1.117 93.863 0.872 59.383 0.886 85.493
ENSMUSG00000040651 D14Abb1e protein_coding 0.928 21.897
0.944 84.594 1.141 8.586 1.003 96.086
ENSMUSG00000040652 Oaz2 protein_coding 0.932 24.344 1.058
87.037 1.015 57.286 1.032 93.734
ENSMUSG00000040653 Ppp1r14c protein_coding 3.978 9.695 1.23
83.819 0.205 0.879 0.926 95.35
ENSMUSG00000040657 1700063H04Rik protein_coding 1 54.44 1
94.887 1 64.778 1 93.316
ENSMUSG00000040658 Dnph1 protein_coding 0.911 79.709 0.879
81.284 1.243 49.898 1.003 95.273
ENSMUSG00000040659 Efhd2 protein_coding 1.188 0.767 0.999 87.553
0.84 0.879 1.017 79.268
ENSMUSG00000040661 Rad54l2 protein_coding 0.966 64.284
0.996 83.733 1.035 31.479 1.017 94.904
ENSMUSG00000040663 Clcf1 protein_coding 0.71 12.436 1.069
91.361 1.318 7.024 1.052 93.612
ENSMUSG00000040666 Sh3bgr protein_coding 2.585 11.638
1.295 71.617 0.298 0.72 0.793 95.832
ENSMUSG00000040667 Nup88 protein_coding 1.054 22.205 0.973
81.284 0.974 54.037 1.002 93.685
ENSMUSG00000040669 Phc1 protein_coding 0.893 1.031 1.061 79.411
1.054 30.213 1.059 83.067
ENSMUSG00000040675 Mthfd1l protein_coding 1.267 37.941
0.871 95.504 0.904 49.217 1.267 33.269
ENSMUSG00000040680 Kremen2 protein_coding 0.5 16.183
1.383 83.464 1.451 38.926 1.331 87.134
ENSMUSG00000040681 Hmgn1 protein_coding 1.016 68.485 0.99
88.345 0.992 85.21 1.031 56.339
ENSMUSG00000040687 Madd protein_coding 0.977 58.56 0.998 94.102
1.027 67.562 0.923 83.724
ENSMUSG00000040688 Tbl3 protein_coding 0.961 23.319 1.014
93.707 1.026 49.058 1.021 82.091
ENSMUSG00000040690 Col16a1 protein_coding 0.921 22.481
1.003 96.847 1.083 32.433 1.077 68.696
ENSMUSG00000040694 Apobec2 protein_coding 1.774 64.193
1.265 83.104 0.444 18.965 1.348 79.545
ENSMUSG00000040697 Dnajc16 protein_coding 0.948 51.56 1.054
82.904 NA NA 0.966 83.724
ENSMUSG00000040699 Limd2 protein_coding 1.098 37.941 0.99
82.848 0.921 75.637 0.94 86.699
ENSMUSG00000040701 Ap1g2 protein_coding 0.894 1.379 0.978 96.847
1.141 7.855 0.986 96.287
ENSMUSG00000040703 Cyp2s1 protein_coding 1.172 33.854

1.052 95.196 0.812 17.898 1.06 93.261
ENSMUSG00000040705 A930016O22Rik protein_coding 5.764 13.734
1.611 83.104 0.108 0.72 0.937 93.685
ENSMUSG00000040706 Agmat protein_coding 1 83.09 1 96.847
1 87.37 1 96.183
ENSMUSG00000040710 St8sia4 protein_coding 1.224 6.853 1.052
83.104 0.776 0.72 0.903 83.724
ENSMUSG00000040711 Sh3pxd2b protein_coding 1.281 2.998 0.956
93.654 0.817 57.446 1.064 83.067
ENSMUSG00000040712 Camta2 protein_coding 1 70.213
1.044 71.617 0.959 11.64 1.006 90.177
ENSMUSG00000040713 Creg1 protein_coding 1.185 2.784 0.966 95.211
0.87 10.23 0.997 95.273
ENSMUSG00000040714 Klc3 protein_coding 0.763 6.34 NA NA
1.205 12.467 0.847 84.29
ENSMUSG00000040715 Rsc1a1 protein_coding 1 86.351 1
96.216 NA NA 1 95.72
ENSMUSG00000040717 Il17rd protein_coding 1.013 50.377
0.963 43.505 1.026 57.907 0.967 92.675
ENSMUSG00000040720 1110037F02Rik protein_coding 1.073 15.456
0.949 72.576 0.982 77.037 0.978 96.287
ENSMUSG00000040721 Zfhx2 protein_coding 0.795 1.422 0.995 94.89 1.26
18.411 0.869 83.724
ENSMUSG00000040722 Scamp5 protein_coding 1.273 0 0.942
82.848 0.836 0.879 1.04 93.156
ENSMUSG00000040723 Rcsd1 protein_coding 1.071 26.338 0.937
82.848 0.993 60.96 1.072 73.939
ENSMUSG00000040724 Kcna2 protein_coding 0.768 27.664 0.992
95.941 1.304 38.926 1.028 91.985
ENSMUSG00000040725 Hnrnpul1 protein_coding 0.998 81.125 1.02
95.809 0.985 56.822 0.993 93.045
ENSMUSG00000040726 Hesx1 protein_coding 1.269 38.386 1.161
95.329 0.678 30.827 0.95 88.136
ENSMUSG00000040728 Esrp1 protein_coding 1.134 71.516 0.875
71.889 1.007 78.155 0.892 96.183
ENSMUSG00000040729 AK129341 protein_coding 1.046 85.283 NA
NA 0.948 84.724 1.131 83.067
ENSMUSG00000040731 Eif4h protein_coding 0.958 23.556 1.025
79.864 1.019 77.532 1.02 86.301
ENSMUSG00000040732 Erg protein_coding 1.037 69.408 0.965
90.744 NA NA 0.979 84.29
ENSMUSG00000040734 Ppp1r13l protein_coding 0.862 5.491 0.937
84.858 1.239 6.202 0.978 85.493
ENSMUSG00000040738 Ints8 protein_coding 1.094 34.077 1.072
79.411 0.854 8.027 1.015 91.011
ENSMUSG00000040740 Slc25a34 protein_coding 2.06 4.825 0.849
96.984 0.572 4.521 0.566 83.724
ENSMUSG00000040746 Rnf167 protein_coding 0.986 58.802
0.977 92.631 1.039 46.206 0.957 83.724
ENSMUSG00000040747 Cd53 protein_coding 1.56 0 0.904 62.322
0.707 4.389 1.203 72.116
ENSMUSG00000040749 Siah1b protein_coding 0.918 49.368
1.228 79.055 0.886 69.134 1.081 80.77
ENSMUSG00000040751 Lat2 protein_coding 1.26 2.651 0.935 95.708

0.844 6.858 1.115 65.558
ENSMUSG00000040752 Myh6 protein_coding 0.502 41.021 1.283
96.847 1.546 62.237 2.023 93.132
ENSMUSG00000040759 Cmtm5 protein_coding 0.792 41.759 1.178
92.111 1.074 84.961 1.11 90.455
ENSMUSG00000040760 Appl1 protein_coding 1.054 29.057 0.955
90.256 0.993 49.967 1.012 95.832
ENSMUSG00000040761 Spen protein_coding 0.931 23.319 1.088
74.663 0.99 82.685 0.949 83.724
ENSMUSG00000040767 Snrnp25 protein_coding 1.11 44.061
0.854 78.877 1.05 76.923 0.989 84.499
ENSMUSG00000040770 Il25 protein_coding 0.994 85.499 1.028
93.529 0.977 65.902 1.383 95.35
ENSMUSG00000040771 Oard1 protein_coding 1.02 35.787 0.962
93.654 1.021 81.388 0.998 86.445
ENSMUSG00000040774 Cept1 protein_coding 0.995 69.715 NA NA
1.034 63.801 NA NA
ENSMUSG00000040775 Gm9772 protein_coding 1 83.823 NA
NA 1 86.346 1 94.825
ENSMUSG00000040782 Rfwd2 protein_coding 1.068 26.77 0.958 81.731
0.98 60.614 1.028 86.133
ENSMUSG00000040785 Ttc3 protein_coding 1.109 1.292 0.971 81.443
0.93 24.309 1.011 92.381
ENSMUSG00000040794 C1qtnf4 protein_coding 0.764 41.617
1.176 97.155 1.113 68.129 0.913 92.31
ENSMUSG00000040795 Iqcc protein_coding 1.025 64.401 0.933
61.883 1.048 28.997 0.987 96.071
ENSMUSG00000040797 Iqsec3 protein_coding 0.701 7.574 1.256
60.309 1.132 66.269 1.081 89.108
ENSMUSG00000040808 S100g protein_coding 0.426 39.712 1.093
93.908 2.149 36.388 2.106 95.74
ENSMUSG00000040811 Eml2 protein_coding 0.88 4.634 0.939 74.483
1.208 7.635 1.041 91.335
ENSMUSG00000040812 Agbl2 protein_coding 1.516 21.599 1.154
84.768 0.569 5.323 1.028 91.105
ENSMUSG00000040813 Tex264 protein_coding 0.931 33.224
1.075 93.899 1.001 64.305 0.994 95.92
ENSMUSG00000040818 Dennd6a protein_coding 1.032 25.207
1.035 89.434 0.932 10.23 0.954 83.724
ENSMUSG00000040820 Hlcs protein_coding 1.18 0.767 0.9 20.272
0.944 31.887 0.918 85.493
ENSMUSG00000040822 1700123O20Rik protein_coding 0.996 72.546
0.972 88.705 1.032 69.315 0.937 85.493
ENSMUSG00000040824 Snrpd2 protein_coding 0.884 18.795
1.089 89.494 1.043 41.6 0.986 94.904
ENSMUSG00000040828 Catsperd protein_coding 0.78 51.089
1.073 79.475 1.195 28.979 0.742 87.735
ENSMUSG00000040829 Zmynd15 protein_coding 1.019 72.372
1.152 82.809 0.849 9.347 1.061 92.018
ENSMUSG00000040836 Gpr161 protein_coding 1.118 14.746 0.86
77.598 1.04 61.288 1.169 72.369
ENSMUSG00000040838 Gm11639 protein_coding 1 74.299 1
96.335 1 84.83 1 95.273
ENSMUSG00000040840 4930579G18Rik antisense 0.78 10.143

1.015 97.155 1.266 8.007 0.844 84.29
ENSMUSG00000040841 Six5 protein_coding 0.936 49.047 1.095
79.055 0.978 65.409 0.957 90.611
ENSMUSG00000040842 Szrd1 protein_coding 1.033 35.499 1.017
92.062 0.954 7.742 1.037 63.114
ENSMUSG00000040843 Tiprl protein_coding 1.121 5.559 0.965 73.766
0.926 69.134 0.972 95.72
ENSMUSG00000040848 Sft2d2 protein_coding 1.019 42.255
1.015 96.897 0.966 50.523 0.915 75.345
ENSMUSG00000040850 Psme4 protein_coding 0.919 28.514 1.046
91.584 1.036 50.636 1.067 60.968
ENSMUSG00000040852 Plekhh2 protein_coding 1.065 72 0.933
91.532 1.007 82.74 0.945 93.612
ENSMUSG00000040855 Reps2 protein_coding 1.266 14.59 0.866 32.358
0.908 78.642 0.972 87.542
ENSMUSG00000040856 Dlk1 protein_coding 0.947 51.056 1.512
71.617 0.696 29.765 0.972 95.593
ENSMUSG00000040857 Erf protein_coding 0.918 38.555 1.121
82.904 0.972 87.431 0.986 96.192
ENSMUSG00000040859 Bsdc1 protein_coding 0.989 51.56 0.977 91.361
1.034 52.574 0.954 95.92
ENSMUSG00000040860 Crocc protein_coding 0.841 14.403 1.004
84.594 1.185 18.411 0.957 88.053
ENSMUSG00000040865 Ino80d protein_coding 0.892 7.881 0.999
96.948 1.126 13.356 0.935 83.724
ENSMUSG00000040866 Rsph6a protein_coding 1 77.615 NA
NA 1 86.258 1 96.358
ENSMUSG00000040867 Begain protein_coding 0.999 73.292
1.196 83.104 0.838 64.778 1.152 35.053
ENSMUSG00000040875 Osbpl10 protein_coding 0.807 8.751 0.975
90.053 1.271 13.356 0.94 87.218
ENSMUSG00000040877 Wdr25 protein_coding 1.014 73.124 0.963
92.545 1.024 86.258 1.008 94.82
ENSMUSG00000040883 Tmem205 protein_coding 0.835 4.874 1.072
84.007 1.116 27.047 0.927 83.952
ENSMUSG00000040888 Gfer protein_coding 0.975 49.749 0.973
89.509 1.057 44.515 1.003 94.174
ENSMUSG00000040891 Foxa3 protein_coding 0.793 78.538 0.505 75.4
2.503 25.144 1.521 78.948
ENSMUSG00000040896 Kcnd3 protein_coding 0.692 32.749 1.132
87.609 1.279 81.032 1.054 92.018
ENSMUSG00000040901 Kcnk18 protein_coding 1 84.397 1
95.35 1 88.274 1 92.675
ENSMUSG00000040904 Gm21988 protein_coding 1.347 82.092
0.521 92.914 1.42 87.744 0.971 94.99
ENSMUSG00000040907 Atp1a3 protein_coding 0.42 0.987 1.15
95.431 2.072 5.864 0.979 96.183
ENSMUSG00000040913 Fbxw4 protein_coding 0.889 21.025 0.964
89.509 1.168 23.604 0.976 84.29
ENSMUSG00000040918 Slc19a2 protein_coding 1.072 46.875
1.029 82.219 0.909 12.948 1.028 78.948
ENSMUSG00000040928 S100pbp protein_coding 1.106 2.085 1.036
93.863 0.87 8.313 0.964 94.654
ENSMUSG00000040929 Rfx3 protein_coding 0.813 2.009 0.956 95.088

1.29 7.635 1.002 92.181
ENSMUSG00000040935 Padi6 protein_coding 1 77.182 1
95.088 1 83.538 1 88.505
ENSMUSG00000040936 Ulk4 protein_coding 1.042 74.482 0.932
94.597 1.032 87.274 0.942 88.505
ENSMUSG00000040938 Slc16a11 protein_coding 0.907 27.202
1.121 83.104 0.983 79.897 0.774 83.724
ENSMUSG00000040940 Arhgef1 protein_coding 0.834 0.987 1.036
87.029 1.157 6.202 0.989 87.992
ENSMUSG00000040943 Tet2 protein_coding 0.973 46.413 0.934
85.671 1.104 20.572 0.989 89.525
ENSMUSG00000040945 Rcc2 protein_coding 0.94 32.749 NA NA
1.012 51.851 1.026 93.627
ENSMUSG00000040950 Mgl2 protein_coding 1.247 0.2 1.094 91.584
0.735 2.563 1.149 86.301
ENSMUSG00000040952 Rps19 protein_coding 0.78 0.987 1.069 78.494
1.195 7.635 1.066 76.831
ENSMUSG00000040957 Cables1 protein_coding 0.992 82.543
0.947 82.073 1.065 66.043 1.118 96.358
ENSMUSG00000040963 Asgr2 protein_coding 0.98 83.418 0.99
85.418 1.027 55.109 0.823 84.29
ENSMUSG00000040964 Arhgef10l protein_coding 0.961 86.172
1.025 94.96 1.016 81.492 1.029 96.248
ENSMUSG00000040966 Slc22a2 protein_coding 0.761 47.266
1.176 93.899 1.115 87.274 1.367 81.251
ENSMUSG00000040969 Arhgef38 protein_coding 0.89 65.106
0.899 95.088 1.251 72.378 0.84 85.493
ENSMUSG00000040972 Igsf21 protein_coding 0.972 83.418 1.19
90.952 0.862 77.294 0.96 95.273
ENSMUSG00000040978 Gm11992 protein_coding 1.658 10.595
0.708 71.889 0.848 52.087 0.999 95.593
ENSMUSG00000040987 Mill2 protein_coding 0.94 85.637 0.942
75.345 1.126 40.875 1.092 90.854
ENSMUSG00000040990 Sh3kbp1 protein_coding 0.884 6.221 1.163
60.309 0.975 82.436 1.091 51.967
ENSMUSG00000040997 Abhd4 protein_coding 1.042 60.435 1.023
89.014 0.939 41.662 0.952 84.29
ENSMUSG00000040998 Npnt protein_coding 1.179 25.207 0.932
87.313 0.91 71.734 0.698 87.331
ENSMUSG00000041000 Trim62 protein_coding 0.776 0.987 1.073
83.104 1.196 10.76 0.914 86.991
ENSMUSG00000041012 Cmtm8 protein_coding 0.989 83.169 1.044
84.858 0.969 76.754 1.008 92.675
ENSMUSG00000041014 Nrg3 protein_coding 1 86.843 1
94.262 1 87.744 1 88.755
ENSMUSG00000041020 Map7d2 protein_coding 0.865 78.744
0.974 87.553 1.188 51.596 1.286 73.401
ENSMUSG00000041025 Iffo2 protein_coding 0.854 7.857 1.237 60.309
0.946 46.32 0.969 90.805
ENSMUSG00000041028 Ghitm protein_coding 0.898 11.384 1.025
83.104 1.083 33.787 0.993 93.264
ENSMUSG00000041035 Dpcd protein_coding 0.834 3.688 0.979 84.017
1.227 6.506 1.049 90.674
ENSMUSG00000041037 Irgq protein_coding 0.948 35.646 1.147

62.898 0.916 29.451 1.003 94.82
ENSMUSG00000041040 Fam117b protein_coding 1.216 10.595
0.828 26.151 NA NA 1.001 96.086
ENSMUSG00000041044 Lrit1 protein_coding 0.619 39.612 0.979
96.843 1.649 55.352 1.436 87.134
ENSMUSG00000041046 Ramp3 protein_coding 1.012 82.479 1.044
85.501 0.947 73.531 1.064 96.139
ENSMUSG00000041052 Slc7a13 protein_coding 1.431 20.799
0.923 95.708 0.755 24.918 1.01 88.136
ENSMUSG00000041057 Wdr43 protein_coding 1.033 51.826 0.976
72.399 0.993 81.492 1.05 71.653
ENSMUSG00000041058 Wwp1 protein_coding 1.13 6.61 0.976 87.857
0.902 21.117 1.068 91.525
ENSMUSG00000041062 Mslnl protein_coding 0.565 21.897 2.085
81.127 0.847 86.985 1.4 65.845
ENSMUSG00000041064 Pif1 protein_coding 0.944 71.217 0.819
65.819 1.29 81.976 1.961 68.502
ENSMUSG00000041068 4930596D02Rik protein_coding 1 77.182
1 96.847 1 87.744 1 96.183
ENSMUSG00000041073 Nacad protein_coding 1.002 70.597 1.25
72.642 0.8 17.662 1.001 94.608
ENSMUSG00000041075 Fzd7 protein_coding 0.945 30.352 1.058
88.136 1 80.089 0.951 84.29
ENSMUSG00000041078 Grid1 protein_coding 2.238 20.032 1.203
95.301 0.37 32.082 0.604 85.493
ENSMUSG00000041079 Rwdd2b protein_coding 0.989 74.689
1.026 88.823 0.98 82.575 1.054 72.992
ENSMUSG00000041084 Ostc protein_coding 1.132 24.253 1.016
91.584 0.87 6.858 1.158 31.133
ENSMUSG00000041096 Tspyl2 protein_coding 0.994 70.741
1.034 92.062 0.973 11.64 1.013 95.832
ENSMUSG00000041112 Elmo1 protein_coding 0.945 85.946 1.071
83.104 0.989 54.521 1.091 91.872
ENSMUSG00000041115 Iqsec2 protein_coding 0.988 78.299
0.969 87.919 1.04 53.587 1.023 93.734
ENSMUSG00000041117 Ccdc8 protein_coding 1.12 19.477 1.123
93.707 0.792 6.858 1.101 66.068
ENSMUSG00000041119 Pde9a protein_coding 1.099 80.815 0.85
56.027 1.075 22.994 0.984 96.183
ENSMUSG00000041120 Nbl1 protein_coding 1.016 79.068 0.856
94.102 1.147 83.538 0.991 92.561
ENSMUSG00000041124 Msantd4 protein_coding 1.13 15.85 0.95
70.971 0.928 58.516 0.996 82.85
ENSMUSG00000041126 H2afv protein_coding 0.905 12.785 1.098
79.367 1.003 83.275 1.021 87.134
ENSMUSG00000041130 Zfp598 protein_coding 0.925 17.705
1.023 93.863 1.052 44.787 1.016 85.577
ENSMUSG00000041132 N4bp2l1 protein_coding 1.103 43.939
0.961 96.138 0.94 48.577 0.837 85.493
ENSMUSG00000041133 Smc1a protein_coding 0.912 13.581 0.975
89.509 1.125 11.518 1.065 60.466
ENSMUSG00000041134 Cyyr1 protein_coding 0.995 85.129 0.959
96.68 1.046 85.675 0.906 83.724
ENSMUSG00000041135 Ripk2 protein_coding 0.894 30.035 1.108

82.904 NA NA 0.937 94.19
ENSMUSG00000041138 Nme8 protein_coding 1 55.462 1
96.652 1 75.353 1 95.74
ENSMUSG00000041143 Tmco4 protein_coding 1.055 13.557 0.986
83.733 0.963 63.075 1 91.105
ENSMUSG00000041144 Dnah7b protein_coding 1.279 15.85 0.786
60.678 0.995 80.163 0.793 86.699
ENSMUSG00000041147 Brca2 protein_coding 0.936 57.544 0.966
97.155 1.104 58.915 1.12 57.1
ENSMUSG00000041153 Osgin2 protein_coding 0.994 79.433
0.971 89.509 1.035 50.375 0.868 83.724
ENSMUSG00000041161 Otud3 protein_coding 0.96 56.242 1.085
79.055 0.956 67.029 0.936 84.29
ENSMUSG00000041164 Zmiz2 protein_coding 0.912 13.74 1.069 83.104
1.022 76.754 0.969 83.724
ENSMUSG00000041165 Spem1 protein_coding 1.183 64.956 1.227
97.263 0.687 55.214 1.118 95.405
ENSMUSG00000041168 Lonp1 protein_coding 0.938 7.58 NA NA
1.082 24.791 0.987 93.685
ENSMUSG00000041180 Hectd2 protein_coding 1.083 20.659
0.988 91.361 0.932 50.523 1.171 37.634
ENSMUSG00000041187 Prkd2 protein_coding 0.897 2.822 1.018 89.996
1.098 28.997 0.944 83.724
ENSMUSG00000041189 Chrnb1 protein_coding 0.748 0.987 1.011
96.843 1.323 5.864 0.904 84.29
ENSMUSG00000041193 Pla2g5 protein_coding 0.749 46.693
1.502 83.819 NA NA 1.608 70.624
ENSMUSG00000041199 Rpusd1 protein_coding 1.083 25.421
0.981 91.661 0.943 53.7 0.998 87.331
ENSMUSG00000041202 Pla2g2d protein_coding 2.003 27.545 0.76
82.848 0.655 21.431 0.555 84.19
ENSMUSG00000041203 2310036O22Rik protein_coding 0.934 9.752
1.037 83.104 1.036 56.931 0.932 96.183
ENSMUSG00000041205 Map6d1 protein_coding 1.419 84.168
1.015 96.138 0.693 88.349 1.017 91.872
ENSMUSG00000041215 Yeats2 protein_coding 1.018 82.732
0.971 96.68 1.012 84.434 1.005 92.381
ENSMUSG00000041216 Clvs1 protein_coding 0.73 8.267 0.866 90.576
1.587 11.665 0.906 91.105
ENSMUSG00000041219 Arhgap11a protein_coding 1.284 19.893
0.889 44.566 0.877 80.163 1.13 91.011
ENSMUSG00000041220 Elovl6 protein_coding 0.177 0.987 1.368
92.931 4.123 6.202 0.761 90.628
ENSMUSG00000041225 Arhgap12 protein_coding 1.107 0.577 0.938
85.116 0.963 5.336 1.012 84.29
ENSMUSG00000041229 Phf8 protein_coding 1.021 83.169 1.008
92.931 0.969 63.919 0.994 94.608
ENSMUSG00000041231 Ublcp1 protein_coding 0.986 52.258
1.035 84.007 NA NA 1.011 91.928
ENSMUSG00000041235 Chd7 protein_coding 0.878 8.122 0.966 95.301
1.178 11.348 1.011 95.92
ENSMUSG00000041236 Vps41 protein_coding 1.099 11.044 0.925
79.807 0.984 29.765 1.034 79.268
ENSMUSG00000041238 Rbbp8 protein_coding 1.135 11.316 0.963

96.897	0.912	20.258	1.04	85.577
ENSMUSG00000041241	Mul1	protein_coding	0.956	21.176	1.028
82.76	1.02	55.352	1.009	94.654
ENSMUSG00000041245	Wnk3	protein_coding	0.874	39.712	1.21
92.561	0.941	85.935	0.979	88.111
ENSMUSG00000041255	Tmco5b	protein_coding	1	79.068	1
96.216	1	85.352	1	94.567
ENSMUSG00000041258	Zfp236	protein_coding	0.974	64.589	1
94.848	1.028	50.375	1.043	83.067
ENSMUSG00000041261	Car8	protein_coding	1.133	23.82	1.1	97.263
0.799	33.971	0.885	84.29
ENSMUSG00000041263	Rusc1	protein_coding	1.043	20.032	0.95
81.731	1.011	77.037	0.945	83.724
ENSMUSG00000041264	Uspl1	protein_coding	1.088	29.536	0.95
71.41	0.969	87.744	1.016	68.354
ENSMUSG00000041268	Dmxl2	protein_coding	1.022	86.464	0.944
90.378	1.036	69.196	1.038	95.74
ENSMUSG00000041272	Tox	protein_coding	1.314	22.349	NA	NA
0.722	32.703	1.004	95.877
ENSMUSG00000041278	Ttc1	protein_coding	1.104	7.842	0.967	79.807
0.935	45.799	1.034	65.558
ENSMUSG00000041287	Sox15	protein_coding	0.845	38.555	NA	NA
1.199	45.667	0.971	92.181
ENSMUSG00000041293	Gpr110	protein_coding	0.887	83.726
1.482	83.104	0.763	88.203	0.483	83.724
ENSMUSG00000041297	Cdk13	protein_coding	1.006	69.777	1.006
89.996	0.985	35.264	0.978	86.991
ENSMUSG00000041298	Katnal1	protein_coding	0.995	81.76	1.042
95.809	0.966	53.7	1.025	92.181
ENSMUSG00000041303	Gtf3c3	protein_coding	0.982	86.464
0.923	72.576	1.1	31.317	1.01	85.577
ENSMUSG00000041308	Sntb2	protein_coding	1.115	69.089	0.871
90.287	1.027	84.724	1.053	96.183
ENSMUSG00000041309	Nkx6-2	protein_coding	0.774	27.664
1.116	97.155	1.158	39.925	0.987	92.903
ENSMUSG00000041313	Slc7a1	protein_coding	0.935	74.482
1.049	85.501	1.019	74.535	1.174	66.468
ENSMUSG00000041319	Thoc6	protein_coding	0.939	66.406	1.018
95.211	1.042	79.897	1.075	81.251
ENSMUSG00000041323	Ak7	protein_coding	1.52	41.211	0.492
47.831	1.339	52.574	0.667	86.991
ENSMUSG00000041324	Inhba	protein_coding	0.885	66.652	1.037
95.504	1.086	57.907	1.218	83.067
ENSMUSG00000041328	Pcf11	protein_coding	0.988	85.832	1.073
72.642	0.942	18.122	1.016	91.078
ENSMUSG00000041329	Atp1b2	protein_coding	1.295	32.238
1.173	61.943	0.656	5.042	0.818	94.608
ENSMUSG00000041333	Mup4	protein_coding	1	85.203	1
94.96	1	86.258	1	96.287
ENSMUSG00000041341	Atg2b	protein_coding	0.961	71.36	1.009	89.494
1.032	76.754	0.958	83.724
ENSMUSG00000041343	Ankrd42	protein_coding	0.912	32.378
1.092	83.104	1	77.17	1.059	76.831
ENSMUSG00000041346	Wrap53	protein_coding	0.921	7.916	0.963

92.062	1.13	36.578	1.024	76.046
ENSMUSG00000041351	Rap1gap	protein_coding	0.86	30.637
0.984	93.765	1.179	33.787	0.917	83.724
ENSMUSG00000041353	Tmem29	protein_coding	0.499	4.055	1.037
92.062	1.939	13.174	NA	NA
ENSMUSG00000041354	Rgl2	protein_coding	0.819	0.987	1.006	96.68
1.217	5.977	0.966	91.105
ENSMUSG00000041355	Ssr2	protein_coding	0.923	3.009	1.135	75.21
0.952	80.089	1.133	31.133
ENSMUSG00000041358	Nutm1	protein_coding	1	73.487	1
95.708	1	69.196	1	78.042
ENSMUSG00000041359	Tcl1	protein_coding	1	58.802	1
96.407	1	87.103	1	96.042
ENSMUSG00000041360	D19Bwg1357e	protein_coding	0.985	74.482
0.913	66.651	1.11	41.6	1.102	55.054
ENSMUSG00000041361	Myzap	protein_coding	0.852	40.217	0.763
61.625	1.544	11.518	1.013	94.904
ENSMUSG00000041362	4930506M07Rik	protein_coding	1.316	0
0.827	15.364	0.916	25.625	0.932	90.289
ENSMUSG00000041372	B4galnt3	protein_coding	1.127	21.278	0.99
87.322	0.894	59.726	0.973	88.505
ENSMUSG00000041375	Ccdc9	protein_coding	0.812	11.384	0.956
89.277	1.288	10.079	1.028	88.505
ENSMUSG00000041377	Ninj2	protein_coding	2.556	45.61	0.861	95.504
0.452	49.453	0.903	96.071
ENSMUSG00000041378	Cldn5	protein_coding	0.594	3.07	1.084	96.984
1.545	7.635	0.831	85.493
ENSMUSG00000041380	Htr2c	protein_coding	1	86.74	1	97.263
1	78.369	1	94.904
ENSMUSG00000041390	Mdfic	protein_coding	1.179	1.81	1.085	83.104
0.779	0.945	NA	NA
ENSMUSG00000041396	Mettl18	protein_coding	0.972	85.355
0.927	81.443	1.109	36.578	1.017	91.525
ENSMUSG00000041399	NA	NA	NA	NA	1	93.046	1
81.169	1	95.273
ENSMUSG00000041406	BC055324	protein_coding	1.096	54.717
0.929	93.654	0.98	83.397	1.029	94.904
ENSMUSG00000041408	Wapal	protein_coding	1.039	32.411	0.979
83.58	0.985	78.045	0.967	85.493
ENSMUSG00000041415	Dicer1	protein_coding	1.011	69.408
0.996	95.504	0.993	87.971	0.989	88.955
ENSMUSG00000041417	Pik3r1	protein_coding	0.94	44.922
1.182	82.904	0.901	85.352	0.895	91.217
ENSMUSG00000041420	Meis3	protein_coding	0.941	34.305	0.984
95.301	1.077	33.042	1.004	95.35
ENSMUSG00000041423	Paqr6	protein_coding	1.043	86.199	0.988
95.211	0.971	79.524	0.876	84.29
ENSMUSG00000041426	Hibch	protein_coding	1.061	85.59	0.962	87.322
0.974	73.309	0.948	85.947
ENSMUSG00000041429	Nthl1	protein_coding	0.892	30.136	0.99
95.504	1.13	31.761	1.017	85.493
ENSMUSG00000041431	Ccnb1	protein_coding	1.24	43.654	0.64
55.265	1.259	84.308	1.849	65.845
ENSMUSG00000041438	Cirh1a	protein_coding	1.088	9.695	0.986

92.474 0.935 13.202 1 96.287
ENSMUSG00000041439 Mfsd6 protein_coding 1.151 0 0.911 56.027
0.955 17.898 0.802 84.29
ENSMUSG00000041440 Gk5 protein_coding 1.144 17.336 0.907
78.51 0.96 68.584 0.925 85.493
ENSMUSG00000041444 Arhgap32 protein_coding 1.096 22.349 0.94
81.443 0.967 54.521 0.945 83.724
ENSMUSG00000041445 Mmrn2 protein_coding 0.962 75.567 1.06
87.609 0.98 82.575 0.973 91.291
ENSMUSG00000041449 Serpina3h pseudogene 1.883 20.032 0.758
79.807 0.701 56.365 1.056 95.74
ENSMUSG00000041453 Rpl21 protein_coding 1.102 22.025 0.934
67.739 0.973 59.726 1.03 83.067
ENSMUSG00000041459 Tardbp protein_coding 0.923 14.427
0.969 86.604 1.12 11.518 1.098 31.133
ENSMUSG00000041460 Cacna2d4 protein_coding 1.01 47.174
1.329 93.765 0.744 72.464 1.592 62.918
ENSMUSG00000041468 Gpr12 protein_coding 1 85.283 NA NA
1 88.161 1 93.132
ENSMUSG00000041471 Fam35a protein_coding 0.998 79.068
0.957 87.919 1.044 75.155 1.029 80.497
ENSMUSG00000041476 Smpx protein_coding 2.639 21.278 1.981
92.062 0.191 3.428 1.205 77.621
ENSMUSG00000041477 Dcp1b protein_coding 0.977 80.608 0.984
95.088 1.037 69.315 0.964 83.724
ENSMUSG00000041479 Syt15 protein_coding 1.151 22.871 1.011
95.504 0.861 6.858 0.992 92.181
ENSMUSG00000041481 Serpina3g protein_coding 1.246 50.377
1.027 95.088 0.78 51.715 1.232 91.872
ENSMUSG00000041482 Piezo2 protein_coding 0.829 34.954
0.975 85.95 1.237 31.4 1.149 88.512
ENSMUSG00000041483 Zfp281 protein_coding 0.949 66.406
1.042 82.904 1.007 56.822 0.998 90.674
ENSMUSG00000041488 Stx3 protein_coding 1.02 53.228 0.932
68.818 1.053 72.148 0.885 84.29
ENSMUSG00000041491 Cep78 protein_coding 0.999 86.918 1.065
89.434 0.943 67.339 1.015 93.964
ENSMUSG00000041498 Kif14 protein_coding 2.092 3.176 0.488 35.072
0.975 79.255 1.205 74.047
ENSMUSG00000041505 Gm3402 protein_coding 1 78.639 1
96.001 1 82.74 1 95.273
ENSMUSG00000041506 Rrp9 protein_coding 0.935 73.585 0.93
93.654 1.143 80.634 1.062 96.248
ENSMUSG00000041512 4930569F06Rik protein_coding 1 81.196
1 95.088 1 87.786 1 93.722
ENSMUSG00000041515 Irf8 protein_coding 1.1 29.057 0.984
82.671 0.925 66.425 0.902 86.699
ENSMUSG00000041523 Upk2 protein_coding 1.322 39.295 0.592
27.163 1.278 52.574 0.614 88.053
ENSMUSG00000041528 Rnf123 protein_coding 0.87 0.987 1.079
71.617 1.068 24.383 1.047 72.992
ENSMUSG00000041530 Ago1 protein_coding 1.123 4.362 0.959 91.532
0.925 6.858 0.863 83.724
ENSMUSG00000041534 Rbp3 protein_coding 1 84.168 1

96.897 1 86.666 1 96.183
ENSMUSG00000041536 Serpina3a protein_coding 1 62.354 1
93.046 1 88.729 1 96.183
ENSMUSG00000041538 H2-Ob protein_coding 1.184 46.013 NA NA
0.83 53.7 0.861 86.699
ENSMUSG00000041540 Sox5 protein_coding 1.022 68.727 1.005
92.793 0.974 32.297 1.183 87.7
ENSMUSG00000041544 Ptchd2 protein_coding 2.432 63.804
0.455 88.897 0.904 70.328 NA NA
ENSMUSG00000041548 Hspb8 protein_coding 0.724 0.987 1.198 81.039
1.148 22.444 1.24 44.679
ENSMUSG00000041552 Ptchd1 protein_coding 0.906 72.985
1.308 83.104 0.841 57.091 0.722 84.29
ENSMUSG00000041556 Fbxo2 protein_coding 1.165 14.008 1.026
95.504 0.836 28.669 0.855 85.493
ENSMUSG00000041559 Fmod protein_coding 1.309 4.675 1.097 79.055
0.695 1.408 0.949 94.904
ENSMUSG00000041560 Gltscr2 protein_coding 0.993 57.133
0.905 34.058 1.114 43.481 0.966 83.724
ENSMUSG00000041566 Tssk1 protein_coding 1.041 81.647 0.998
88.533 0.959 82.31 0.859 92.381
ENSMUSG00000041570 Camsap2 protein_coding 1.057 21.599
1.049 95.809 0.899 35.264 0.886 83.724
ENSMUSG00000041571 Sepw1 protein_coding 0.906 3.325 1.055 79.367
1.048 43.481 1.076 61.316
ENSMUSG00000041577 Prelp protein_coding 0.997 58.56 1.039 84.768
0.965 82.903 1.051 81.901
ENSMUSG00000041578 Crx protein_coding 1 81.925 NA NA
1 88.161 1 95.273
ENSMUSG00000041583 Obox6 protein_coding 1 83.169 NA NA
1 87.744 1 95.273
ENSMUSG00000041592 Sdk2 protein_coding 0.759 3.688 0.92 89.509
1.433 10.819 1.016 94.904
ENSMUSG00000041594 Tmtc4 protein_coding 1.023 80.408 1.09
89.494 0.896 66.425 0.915 83.724
ENSMUSG00000041596 Nlrp5-ps pseudogene 0.923 71.674 0.719
61.883 1.501 12.35 1.19 64.513
ENSMUSG00000041598 Cdc42ep4 protein_coding 0.934 34.795
1.018 96.001 1.047 40.575 0.98 88.505
ENSMUSG00000041605 5730559C18Rik protein_coding 1.266 36.686
0.89 81.284 0.889 72.464 1.094 95.273
ENSMUSG00000041607 Mbp protein_coding 0.964 86.474 NA NA
1.041 88.601 1.002 88.505
ENSMUSG00000041608 Entpd3 protein_coding 1.087 59.527
1.015 94.597 0.909 76.388 1.088 91.625
ENSMUSG00000041609 Ccdc64 protein_coding 1.022 85.77 1.04
94.89 0.938 78.642 0.855 85.493
ENSMUSG00000041617 Ccdc74a protein_coding 1.093 60.435 NA
NA 0.896 64.427 1.092 74.193
ENSMUSG00000041620 Mmp1b protein_coding 1 84.263 1
95.504 1 85.935 1 92.181
ENSMUSG00000041623 D11Wsu47e protein_coding 1.019 83.169 0.99
96.68 0.991 84.552 0.983 96.183
ENSMUSG00000041624 Gucy1a2 protein_coding 0.748 11.707

1.419 71.617 0.938 88.274 0.975 87.088
ENSMUSG00000041625 Ggact protein_coding 1.016 75.671 1.079
82.904 0.909 29.451 0.966 90.854
ENSMUSG00000041629 Fam104a protein_coding 0.932 29.21 1.096
79.411 0.978 78.954 0.956 86.699
ENSMUSG00000041632 Mrps27 protein_coding 1.035 41.951
1.027 94.48 0.939 67.029 0.983 94.19
ENSMUSG00000041633 Kctd12b protein_coding 1.012 71.217
0.941 92.062 1.047 71.388 0.94 77.245
ENSMUSG00000041638 Gcn1l1 protein_coding 0.909 0.987 0.968
87.857 1.132 5.864 1.006 96.071
ENSMUSG00000041642 Kif21b protein_coding 1.068 72 1.01
90.116 0.926 32.297 0.892 83.724
ENSMUSG00000041644 Slc5a12 protein_coding 1 84.564 1
95.504 1 86.144 1 95.92
ENSMUSG00000041645 Ddx24 protein_coding 1.131 4.925 0.961 59.946
0.921 15.169 1.024 71.305
ENSMUSG00000041649 Klf8 protein_coding 0.865 28.998 NA NA
1.116 32.524 0.987 95.877
ENSMUSG00000041650 Pcca protein_coding 0.943 40.568 1.033
89.434 1.024 85.352 0.946 85.493
ENSMUSG00000041653 Pnpla3 protein_coding 1.009 66.897
1.386 81.127 0.713 46.575 0.924 94.904
ENSMUSG00000041654 Slc39a11 protein_coding 0.949 25.492
0.989 91.584 1.061 55.109 1.036 70.624
ENSMUSG00000041658 Rragb protein_coding 0.985 82.982 0.989
94.848 1.028 77.037 0.916 94.174
ENSMUSG00000041660 Bbox1 protein_coding 0.823 79.93 1.182 92.062
1.03 85.935 0.995 95.593
ENSMUSG00000041669 Prima1 protein_coding 0.92 80.146 0.96
96.407 1.132 84.552 1.006 95.877
ENSMUSG00000041670 Rims1 protein_coding 0.518 46.413 0.772
96.138 2.503 44.787 1.327 90.455
ENSMUSG00000041671 Pyroxd1 protein_coding 1.035 76.827
0.932 55.265 1.038 35.441 0.943 91.625
ENSMUSG00000041673 Lrrc18 protein_coding 1.357 79.577
0.783 84.594 0.944 58.414 1.14 95.273
ENSMUSG00000041674 BC006965 processed_transcript 0.792 21.658
0.903 91.661 1.399 22.517 0.968 87.735
ENSMUSG00000041679 Lrrc29 protein_coding 1.117 47.847
0.896 74.483 1.002 82.74 1.012 96.183
ENSMUSG00000041684 Bivm protein_coding 1.04 24.689 0.932 75.4
1.029 86.552 1.029 94.99
ENSMUSG00000041685 Fcho2 protein_coding 1.021 44.694 0.979
84.017 0.996 87.336 0.983 89.294
ENSMUSG00000041688 Amot protein_coding 1.016 73.841 0.995
96.138 0.989 81.62 1.176 78.948
ENSMUSG00000041695 Kcnj2 protein_coding 0.988 84.865 1.107
87.029 0.911 59.383 0.923 93.264
ENSMUSG00000041696 Rasl12 protein_coding 1.232 19.132
1.097 83.104 0.737 0.72 1.062 72.826
ENSMUSG00000041697 Cox6a1 protein_coding 0.786 1.15 1.112
83.104 1.146 19.877 1.047 92.31
ENSMUSG00000041698 Slco1a1 protein_coding 1 82.982 1

94.848	1	84.961	1	92.748
ENSMUSG00000041700	Lhfpl1	protein_coding	0.287	28.514
1.783	83.104	1.947	56.931	2.801	60.466
ENSMUSG00000041702	Btbd7	protein_coding	0.936	36.945	1.017
92.111	1.05	71.232	1.019	92.181
ENSMUSG00000041703	Zic5	protein_coding	1	86.172	1
97.263	1	85.464	1	96.183
ENSMUSG00000041707	1810011H11Rik	protein_coding	0.831	4.385
0.876	84.017	1.372	5.864	1.023	72.992
ENSMUSG00000041708	Mpped1	protein_coding	1	80.146	1
96.335	1	81.525	1	93.584
ENSMUSG00000041710	Trpc5	protein_coding	1	86.843	1
94.848	1	84.724	1	95.273
ENSMUSG00000041712	Ubr7	protein_coding	1.176	5.527	0.914	70.971
0.931	32.297	0.967	87.182
ENSMUSG00000041716	Gm20604	protein_coding	0.88	61.043
1.074	95.211	1.057	82.161	0.977	86.991
ENSMUSG00000041717	Golt1a	protein_coding	1	80.51	1
96.847	1	79.897	1	95.273
ENSMUSG00000041718	Alg13	protein_coding	0.976	69.24	0.954	82.073
1.078	38.926	0.993	85.493
ENSMUSG00000041720	Pi4ka	protein_coding	1.021	46.875	1.029
95.708	0.953	66.753	1.045	87.7
ENSMUSG00000041722	Khdc1c	protein_coding	1	80.51	NA	NA
1	82.74	1	95.35
ENSMUSG00000041729	Coro2b	protein_coding	0.955	54.604
0.977	88.897	1.066	54.363	0.919	95.74
ENSMUSG00000041730	Prrxl1	protein_coding	1	86.74	1
97.155	1	86.346	1	90.674
ENSMUSG00000041731	Pgm5	protein_coding	1.059	46.097	1.083
79.29	0.869	11.314	0.856	83.724
ENSMUSG00000041733	Coq5	protein_coding	1.015	82.231	NA	NA
1.006	81.525	0.98	91.372
ENSMUSG00000041734	Kirrel	protein_coding	1.038	69.408
0.947	83.392	NA	NA	0.971	95.593
ENSMUSG00000041735	Gm13178	protein_coding	1	71.763	1
97.097	1	74.325	1	96.248
ENSMUSG00000041736	Tspo	protein_coding	0.796	0.987	1.019	92.062
1.236	11.518	1.074	93.261
ENSMUSG00000041737	Tmem45b	protein_coding	0.986	80.751
1.152	96.308	0.881	88.693	0.897	96.358
ENSMUSG00000041740	Rnf10	protein_coding	0.938	1.031	1.087	40.99
0.982	82.903	1.015	76.347
ENSMUSG00000041741	Pde3a	protein_coding	1.097	79.165	1.024
96.847	0.888	86.258	0.836	84.29
ENSMUSG00000041747	Utp15	protein_coding	1.042	40.333	0.984
92.137	0.973	64.427	1.032	93.722
ENSMUSG00000041748	Ackr4	protein_coding	0.886	49.749	1.064
92.137	1.058	41.806	0.943	93.685
ENSMUSG00000041750	Cd1d2	protein_coding	0.966	62.888	0.867
93.469	1.196	44.515	0.961	82.85
ENSMUSG00000041757	Plekha6	protein_coding	0.909	31.11	1.021
95.504	1.078	38.975	0.893	84.29
ENSMUSG00000041762	Gpr155	protein_coding	0.935	67.163

0.995 88.691 1.072 40.875 0.911 86.699
ENSMUSG00000041763 Tpp2 protein_coding 1.092 4.675 0.953 67.941
0.958 39.245 0.981 88.512
ENSMUSG00000041765 Ubac2 protein_coding 1.043 38.682 NA NA
0.958 36.248 0.959 86.699
ENSMUSG00000041769 Ppp2r2d protein_coding 1.088 18.067 0.96
79.261 0.96 59.922 0.975 93.156
ENSMUSG00000041773 Enc1 protein_coding 1.075 40.635 1.093
92.062 0.853 15.024 0.896 96.287
ENSMUSG00000041774 Ydjc protein_coding 0.765 10.014 1.047
95.708 1.252 15.241 1.018 95.74
ENSMUSG00000041775 Mapk1ip1 protein_coding 1.047 41.454
0.888 75.052 1.076 83.538 0.955 83.724
ENSMUSG00000041777 Cir1 protein_coding 0.977 74.299 0.928
73.766 1.104 21.705 1.018 91.985
ENSMUSG00000041779 Tram2 protein_coding 1.211 10.391 1.105
60.309 0.748 0.72 1.013 92.381
ENSMUSG00000041781 Cpsf2 protein_coding 1.075 37.414 0.985
95.941 0.945 46.32 1.05 64.905
ENSMUSG00000041782 Lad1 protein_coding 0.912 31.921 0.878
58.277 1.247 9.567 0.853 92.31
ENSMUSG00000041789 2700046A07Rik protein_coding 0.596 38.983
1.21 96.843 1.389 57.286 1.413 83.067
ENSMUSG00000041791 Capza3 protein_coding 1 83.418 1
84.017 1 67.562 1 92.018
ENSMUSG00000041794 Myrip protein_coding 0.736 12.084 0.938
85.921 1.455 19.877 0.949 83.276
ENSMUSG00000041797 Abca9 protein_coding 1.152 30.788 0.895
61.883 0.969 84.724 0.997 94.654
ENSMUSG00000041798 Gck protein_coding 0.734 85.77 1.28 96.948
1.069 85.792 1.227 90.177
ENSMUSG00000041801 Phlda3 protein_coding 0.925 15.912 1.15
71.617 0.941 76.768 1.149 67.691
ENSMUSG00000041805 Pramel1 protein_coding 1 71.763 1
95.809 1 88.461 1 95.74
ENSMUSG00000041809 Efhc1 protein_coding 1.018 65.733 0.964
72.399 1.017 73.428 0.975 96.183
ENSMUSG00000041815 Poldip3 protein_coding 1.038 13.363
1.009 87.609 0.955 6.858 0.996 93.264
ENSMUSG00000041817 Fam169a protein_coding 1.438 42.555
0.902 92.404 0.771 39.245 0.894 90.289
ENSMUSG00000041827 Oasl1 protein_coding 1.622 0.976 0.853 82.503
0.719 25.345 1.891 48.119
ENSMUSG00000041828 Abca8a protein_coding 0.989 73.585
0.953 87.322 1.065 49.601 1.04 93.734
ENSMUSG00000041831 Sytl3 protein_coding 0.851 26.217 0.852
88.691 1.38 35.441 0.651 83.724
ENSMUSG00000041836 Ptpre protein_coding 0.95 85.092 1.076
92.062 0.979 59.922 1.029 95.72
ENSMUSG00000041837 Pdcd7 protein_coding 0.952 56.242 1.08
82.904 0.97 68.266 0.962 88.505
ENSMUSG00000041840 Haus1 protein_coding 0.983 85.092 0.934
72.576 1.09 28.74 1.065 83.067
ENSMUSG00000041841 Rpl37 protein_coding 1.108 15.972 0.901

54.427 1.004 83.607 1.01 93.612
ENSMUSG00000041842 Fhdc1 protein_coding 1.06 85.355 1.007
93.574 0.938 79.255 0.928 85.493
ENSMUSG00000041845 Rhod protein_coding 1.016 72.985 0.908
91.955 1.087 51.596 0.997 95.405
ENSMUSG00000041846 Smek1 protein_coding 1.087 6.399 0.994 95.196
0.924 6.524 0.996 94.82
ENSMUSG00000041849 Card6 protein_coding 0.931 42.361 0.955
73.766 1.127 20.085 0.966 90.805
ENSMUSG00000041852 Tcf20 protein_coding 1.074 26.863 1.039
89.434 0.895 11.314 0.953 90.611
ENSMUSG00000041857 Oosp1 protein_coding 1 85.728 1
96.378 1 86.831 1 94.654
ENSMUSG00000041859 Mcm3 protein_coding 1.126 29.596 0.999
90.046 0.89 75.353 1.287 31.133
ENSMUSG00000041870 Ankrd13a protein_coding 1.003 77.291
1.005 93.654 0.993 84.626 1.072 50.74
ENSMUSG00000041878 8430432A02Rik protein_coding 0.816 44.467
1.017 94.767 1.202 48.935 0.946 94.608
ENSMUSG00000041879 Ipo9 protein_coding 1.068 37.414 0.994
95.504 0.939 38.799 0.931 86.254
ENSMUSG00000041881 Ndufa7 protein_coding 0.875 4.874 1.035
82.904 1.104 43.842 1.049 78.042
ENSMUSG00000041885 Olfr680-ps1 pseudogene 1 85.77 1 96.335
1 82.436 1 96.358
ENSMUSG00000041886 Macc1 protein_coding 1.143 40.88 0.974 93.908
0.896 52.213 1.055 95.273
ENSMUSG00000041889 Shisa4 protein_coding 1.231 58.198
1.273 63.504 0.635 1.705 0.972 95.74
ENSMUSG00000041890 Git2 protein_coding 0.985 84.294 1.027
83.104 0.986 49.453 0.982 83.952
ENSMUSG00000041891 Lman1 protein_coding 0.978 74.021 1.041
83.104 0.984 69.134 1.071 64.513
ENSMUSG00000041895 Wipi1 protein_coding 0.898 8.002 0.981 90.138
1.14 12.831 1.123 40.894
ENSMUSG00000041907 Gpr45 protein_coding 1 81.823 1
96.652 1 87.274 1 95.273
ENSMUSG00000041911 Dlx1 protein_coding 1.082 73.841 0.981
92.545 0.94 86.258 0.939 86.699
ENSMUSG00000041912 Tdrkh protein_coding 1.017 82.732 1.071
82.904 0.914 25.052 1.036 95.273
ENSMUSG00000041915 Ammecr1l protein_coding 1.041 21.278
1.022 83.104 0.941 2.087 0.985 96.248
ENSMUSG00000041920 Slc16a6 protein_coding 1.301 0.355 NA NA
0.738 3.718 1.072 90.463
ENSMUSG00000041921 Metap1d protein_coding 0.976 41.33 1.018
88.092 1.009 86.258 1.014 91.372
ENSMUSG00000041923 Nol4 protein_coding 1 67.334 1
89.509 1 85.921 1 94.904
ENSMUSG00000041926 Rnpep protein_coding 0.938 44.467 0.984
75.345 1.083 24.791 1.055 56.079
ENSMUSG00000041930 Fam222a protein_coding 1.41 20.659
1.294 72.642 0.546 2.722 0.968 81.061
ENSMUSG00000041935 AW549877 protein_coding 0.931 21.637 1.02

96.847 1.05 23.668 0.919 83.724
ENSMUSG00000041936 Agrn protein_coding 0.959 46.693 1.032
95.504 1.014 56.497 0.908 88.505
ENSMUSG00000041939 Mvk protein_coding 0.9 10.739 1.084
72.546 1.026 78.293 1.002 91.525
ENSMUSG00000041945 Mfsd9 protein_coding 1.039 70.121 0.902
94.597 1.069 71.004 0.916 87.992
ENSMUSG00000041949 Tango6 protein_coding 0.999 73.585 NA
NA 1.005 75.809 0.982 90.805
ENSMUSG00000041954 Tnfrsf18 protein_coding 0.836 17.276
1.008 95.088 1.189 38.152 0.953 96.287
ENSMUSG00000041957 Pkp2 protein_coding 0.946 71.516 0.98
91.897 1.074 87.37 1.105 95.273
ENSMUSG00000041958 Pigs protein_coding 0.969 78.299 1.039
96.847 0.994 85.935 1.046 83.067
ENSMUSG00000041959 S100a10 protein_coding 0.96 52.726 0.97
94.597 1.076 52.8 1.128 37.634
ENSMUSG00000041961 Znrf3 protein_coding 1.08 25.944 0.982
84.594 0.939 46.575 0.855 75.345
ENSMUSG00000041966 Dcaf17 protein_coding 0.913 14.526
0.986 95.211 1.112 18.565 0.939 83.724
ENSMUSG00000041974 Spidr protein_coding 0.952 45.894 0.913
68.115 1.153 7.635 1.118 40.024
ENSMUSG00000041975 Mettl8 protein_coding 1.006 84.294
0.988 89.996 1.007 80.381 1.002 92.389
ENSMUSG00000041977 Arhgef11 protein_coding 0.991 44.467
1.051 62.898 0.957 49.453 0.938 84.29
ENSMUSG00000041986 Elmod1 protein_coding 0.538 20.116
1.331 96.843 1.391 18.411 1.049 90.674
ENSMUSG00000041991 Hrnr protein_coding 1 75.567 1
95.504 1 87.744 1 96.287
ENSMUSG00000041992 Rapgef5 protein_coding 0.993 70.597
1.046 89.434 0.962 77.294 1.004 93.722
ENSMUSG00000041995 Zbed3 protein_coding 0.899 9.341 1.009 95.431
1.102 31.975 0.975 93.584
ENSMUSG00000041997 Tlk1 protein_coding 1.015 28.693 1.046
96.68 0.939 7.948 1.017 95.92
ENSMUSG00000042002 Foxn4 protein_coding 1 76.319 1
95.211 1 76.388 1 93.156
ENSMUSG00000042010 Acacb protein_coding 0.652 1.803 1.063 95.708
1.444 11.782 0.824 91.625
ENSMUSG00000042015 Wdr41 protein_coding 1.005 76.319 1.034
87.029 0.96 25.052 1.045 50.452
ENSMUSG00000042029 Ncapg2 protein_coding 1.087 49.868
0.992 78.877 0.923 86.666 1.147 83.067
ENSMUSG00000042031 Lce3b protein_coding 1 85.832 1
95.088 1 84.961 1 96.183
ENSMUSG00000042032 Mat2b protein_coding 1.032 49.202 0.933
70.971 1.04 62.237 0.999 86.699
ENSMUSG00000042035 Igsf3 protein_coding 1.215 38.386 1.077
96.138 0.764 59.383 0.897 90.805
ENSMUSG00000042041 2010003K11Rik protein_coding 0.444 0.987
1.223 78.235 1.833 10.819 0.684 85.947
ENSMUSG00000042042 Csgalnact2 protein_coding 1.105 0.355 0.881

61.625 1.025 83.607 1.015 92.381
ENSMUSG00000042043 Tbca protein_coding 0.999 77.291 1.007
95.809 0.996 78.713 1.054 86.445
ENSMUSG00000042046 Dstyk protein_coding 1.057 16.74 0.91 55.71
1.042 78.498 0.988 86.699
ENSMUSG00000042050 Wdr60 protein_coding 0.916 4.115 NA NA 1.12
10.281 1.016 68.696
ENSMUSG00000042055 Wdr11 protein_coding 1.003 75.671 0.92
15.938 1.087 5.864 0.979 92.31
ENSMUSG00000042063 Zfp386 protein_coding 1.06 41.951
1.021 95.809 0.927 46.956 1.04 95.676
ENSMUSG00000042066 Tmcc2 protein_coding 1.033 82.891 1.025
97.155 NA NA 0.962 94.904
ENSMUSG00000042073 Abhd14b protein_coding 1.014 71.06 1.084
85.501 0.911 39.245 0.961 85.493
ENSMUSG00000042078 Svop protein_coding 1 63.126 1
95.735 1 84.229 1 93.132
ENSMUSG00000042079 Hnrnpf protein_coding 1.104 14.34 0.972
66.651 0.931 36.786 1.043 40.894
ENSMUSG00000042082 Arsb protein_coding 1.084 20.942 1.086
82.809 0.847 5.336 1.005 83.581
ENSMUSG00000042087 4933440N22Rik protein_coding 0.974 78.744
1.179 73.092 0.869 28.669 0.882 92.31
ENSMUSG00000042092 Lce1c protein_coding 1 83.823 1
95.809 1 80.015 1 87.624
ENSMUSG00000042097 Zfp239 protein_coding 0.977 77.763
0.992 96.843 1.028 86.144 0.667 83.724
ENSMUSG00000042099 Kank3 protein_coding 0.793 4.874 0.941 71.889
1.338 6.069 0.98 89.892
ENSMUSG00000042102 Dmgdh protein_coding 1 48.939 1
96.847 1 85.757 1 92.675
ENSMUSG00000042104 Uggt2 protein_coding 0.943 32.168 0.976
90.378 1.089 23.454 1.113 31.133
ENSMUSG00000042105 Inpp5f protein_coding 1.158 0 0.873
20.174 0.986 83.158 0.971 84.29
ENSMUSG00000042106 Fam212a protein_coding 0.939 17.401
0.915 72.399 1.164 11.932 1.021 94.904
ENSMUSG00000042109 Csdc2 protein_coding 0.934 56.477 1.093
96.847 0.982 78.369 0.896 85.493
ENSMUSG00000042111 Ccdc115 protein_coding 1.031 42.255
0.951 59.946 1.02 52.8 1.016 72.992
ENSMUSG00000042115 Klhdc8a protein_coding 1.043 69.777
0.664 66.651 1.443 48.772 1.003 94.608
ENSMUSG00000042116 Vwa1 protein_coding 0.987 71.142 0.947
93.654 1.066 43.481 1.083 61.753
ENSMUSG00000042118 Bhmt2 protein_coding 1 80.408 1
95.211 1 86.666 1 94.654
ENSMUSG00000042121 Ssh1 protein_coding 1.035 63.534 1.058
79.055 0.909 7.329 0.934 83.724
ENSMUSG00000042124 Lce1f protein_coding 1 74.299 1
95.504 1 85.21 1 93.156
ENSMUSG00000042129 Rassf4 protein_coding 1.258 0 0.863
35.072 0.922 32.082 1.039 95.35
ENSMUSG00000042133 Ppig protein_coding 1.083 3.713 0.962 69.611

0.961 35.559 1.021 84.246
ENSMUSG00000042138 Msantd2 protein_coding 1.069 52.986
0.998 95.431 0.938 55.214 1.006 86.445
ENSMUSG00000042148 Cox10 protein_coding 1.016 76.827 1.113
82.809 0.883 8.907 1.053 87.624
ENSMUSG00000042155 Klhl23 protein_coding 0.966 65.622
0.972 92.404 1.061 66.043 0.996 89.498
ENSMUSG00000042156 Dzip1 protein_coding 1.194 9.983 0.981 91.361
0.852 28.237 0.887 86.991
ENSMUSG00000042157 Sprr2i protein_coding 1 74.689 NA
NA 1 87.744 1 93.734
ENSMUSG00000042165 Gm9774 protein_coding 3.305 5.932 0.728
88.897 0.415 8.382 0.89 96.071
ENSMUSG00000042167 Papd4 protein_coding 1.155 0.944 0.912 60.678
0.946 37.617 1.038 74.607
ENSMUSG00000042178 Armc5 protein_coding 0.976 47.266 1.047
92.931 0.977 84.961 0.945 94.654
ENSMUSG00000042182 Bend6 protein_coding 1.156 34.077 1.08
90.378 0.802 46.32 0.853 86.445
ENSMUSG00000042185 Nfrkb protein_coding 1.007 82.982 0.979
94.48 1.016 88.203 0.982 86.991
ENSMUSG00000042189 Tekt3 protein_coding 1 63.736 1
94.48 1 76.225 1 83.581
ENSMUSG00000042190 Cmklr1 protein_coding 1.043 82.231
1.021 89.996 0.942 71.734 1.058 56.769
ENSMUSG00000042195 Slc35f2 protein_coding 0.799 59.358
0.771 96.216 1.627 50.375 1.06 94.654
ENSMUSG00000042197 Zfp451 protein_coding 1.104 22.205
0.926 93.094 0.982 59.922 0.931 85.493
ENSMUSG00000042198 Chchd7 protein_coding 0.869 19.365
1.075 87.609 1.073 56.931 0.961 85.493
ENSMUSG00000042200 Cdrt4 protein_coding 1.332 76.546 0.722
84.594 1.042 69.728 0.554 83.724
ENSMUSG00000042202 Slc35e2 protein_coding 1.031 65.733
1.071 78.494 0.902 8.027 0.934 83.724
ENSMUSG00000042203 Tbc1d22b protein_coding 0.979 60.569
1.116 64.304 0.916 11.314 1.001 89.525
ENSMUSG00000042207 Kdm5b protein_coding 0.981 85.391 0.975
90.046 1.045 62.648 1.014 95.559
ENSMUSG00000042208 0610010F05Rik protein_coding 0.883 14.221
1.04 83.104 1.088 34.07 1.13 52.324
ENSMUSG00000042210 Abhd14a protein_coding 0.922 12.309
1.011 97.263 1.071 17.412 1.015 92.018
ENSMUSG00000042211 Fbxo38 protein_coding 0.999 73.734
0.948 54.06 1.057 32.173 0.989 96.358
ENSMUSG00000042212 Sprr2d protein_coding 1 86.474 1
95.504 1 87.519 1 92.564
ENSMUSG00000042213 Zfand4 protein_coding 1.15 43.019
1.163 95.708 0.748 48.851 0.89 93.045
ENSMUSG00000042215 Bag2 protein_coding 0.939 44.467 0.969
84.594 1.098 28.979 1.076 83.067
ENSMUSG00000042216 Sgsm1 protein_coding 0.732 15.06 NA NA 1.36
29.777 1.033 94.608
ENSMUSG00000042219 Olfr631 protein_coding 1 70.213 NA

NA 1 82.31 1 96.071
ENSMUSG00000042225 Ammecr1 protein_coding 1.125 21.539
1.025 96.68 0.867 18.336 1.053 95.777
ENSMUSG00000042228 Lyn protein_coding 1.217 0 0.95 55.265
0.863 2.563 1.107 40.127
ENSMUSG00000042229 Rabif protein_coding 1.038 70.741 1.018
83.104 0.943 6.858 1.002 68.696
ENSMUSG00000042233 2010015L04Rik protein_coding 0.798 49.944
1.667 60.309 0.751 18.965 0.981 96.183
ENSMUSG00000042240 Crybb2 protein_coding 1 71.763 1
96.68 1 75.527 1 95.877
ENSMUSG00000042243 BC051665 protein_coding 1 77.182 NA
NA 1 88.729 1 93.964
ENSMUSG00000042244 Pglyrp3 protein_coding 1 82.231 1
96.652 1 85.352 1 95.74
ENSMUSG00000042246 Tmc7 protein_coding 1.374 3.598 0.882 95.088
0.821 20.712 1.15 88.755
ENSMUSG00000042248 Cyp2c37 protein_coding 1 74.745 1
97.263 1 70.623 1 93.316
ENSMUSG00000042249 Adrbk2 protein_coding 1.043 56.311
1.017 94.102 0.944 78.713 0.751 83.724
ENSMUSG00000042251 Pm20d1 protein_coding 0.56 2.009 1.1
85.756 1.619 8.339 1.137 81.251
ENSMUSG00000042254 Cilp protein_coding 1.362 23.254 0.773
94.848 0.953 67.962 1.43 57.1
ENSMUSG00000042256 Ptchd4 protein_coding 0.861 30.136
1.774 60.309 0.653 44.019 0.937 93.964
ENSMUSG00000042269 Fam92b protein_coding 1 74.8 1
90.68 1 87.519 1 93.264
ENSMUSG00000042271 Nxt2 protein_coding 1.146 4.825 1.009 87.857
0.861 28.489 0.987 91.372
ENSMUSG00000042272 Sestd1 protein_coding 1.006 50.377
0.958 62.322 1.036 45.449 1.006 95.92
ENSMUSG00000042275 Pelo protein_coding 0.915 27.836 1.126
71.98 0.967 82.575 0.943 91.372
ENSMUSG00000042279 H1foo protein_coding 1 71.217 NA NA
1 87.173 1 96.248
ENSMUSG00000042284 Itga1 protein_coding 0.902 21.957 1.018
92.253 1.088 51.851 1.002 86.991
ENSMUSG00000042286 Stab1 protein_coding 1.058 19.893 0.999
94.887 0.948 68.584 1.125 74.193
ENSMUSG00000042289 Hsd3b7 protein_coding 1.076 18.314
1.025 91.643 0.903 11.504 0.952 85.493
ENSMUSG00000042292 Mkl1 protein_coding 1.098 39.295 0.993
97.097 0.912 24.309 0.949 96.183
ENSMUSG00000042293 Gm5617 protein_coding 0.91 34.162
0.968 93.654 1.135 29.568 0.851 83.724
ENSMUSG00000042298 Ttc19 protein_coding 0.913 17.705 1.011
93.765 1.085 25.93 0.998 95.74
ENSMUSG00000042302 Ehbp1 protein_coding 0.933 40.786 0.969
73.461 1.102 21.873 1.044 70.539
ENSMUSG00000042303 Sgsm3 protein_coding 0.978 51.447 0.856
75.345 1.191 26.663 1.101 83.276
ENSMUSG00000042305 Tmem183a protein_coding 0.941 41.617

1.007 96.268	1.06	37.764	0.987 87.331
ENSMUSG00000042306	S100a14	protein_coding	1.426 22.349
0.559 12.804	1.251 71.839	1.254 89.428
ENSMUSG00000042308	Setd1a	protein_coding	0.938 8.45	NA	NA
1.076 43.185	0.97	90.611
ENSMUSG00000042312	S100a13	protein_coding	0.93	25.492
1.134 76.634	0.951 85.675	0.971 92.748
ENSMUSG00000042320	Prox2 protein_coding	1.069 56.085	0.933
87.041	0.999 85.675	0.926 85.493
ENSMUSG00000042323	Pbrm1 protein_coding	0.984 60.707	0.982
88.345	1.034 43.307	1.031 93.132
ENSMUSG00000042328	Hps4	protein_coding	1.011 84.049	1.008
97.049	0.982 88.693	0.985 90.854
ENSMUSG00000042331	Specc1	protein_coding	0.996 81.465
0.958 92.474	1.045 72.148	0.928 89.294
ENSMUSG00000042333	Tnfrsf14	protein_coding	0.979 66.272
1.023 95.809	1	57.446	0.972 84.29
ENSMUSG00000042340	Ctf1	protein_coding	0.786 1.803 1.06	82.904
1.198 24.383	1.04	92.903
ENSMUSG00000042345	Ubash3a	protein_coding	1.967 12.855
1.394 91.776	0.363 2.189 0.363 83.724
ENSMUSG00000042348	Arl15 protein_coding	0.98	82.891	0.993
91.661	1.029 54.673	0.998 94.016
ENSMUSG00000042349	Ikbke protein_coding	1.054 4.825 0.917 61.625
1.035 78.642	1.182 72.369
ENSMUSG00000042350	Arel1 protein_coding	0.952 59.358	1.031
88.823	1.021 77.037	1.044 71.305
ENSMUSG00000042351	Grap2 protein_coding	1.408 35.024	1.229
79.864	0.577 12.571	0.871 86.699
ENSMUSG00000042354	Gnl3	protein_coding	1.12	37.091	0.929
66.651	0.96	84.896	1.07	75.198
ENSMUSG00000042357	Gjb5	protein_coding	1.054 66.76 1.103 93.707
0.858 48.387	1.164 96.248
ENSMUSG00000042359	Osbpl6	protein_coding	1.148 51.189
0.689 70.29 1.26	51.851	1.026 74.193
ENSMUSG00000042360	4930433N12Rik	protein_coding	1	77.113
1	96.268	1	75.155	1	92.018
ENSMUSG00000042363	Lgalsl	protein_coding	1.135 5.657 0.949
67.941	0.929 42.895	0.979 93.612
ENSMUSG00000042364	Snx18 protein_coding	1.024 48.462	1.001
97.049	0.976 31.887	0.907 83.724
ENSMUSG00000042367	Gjb3	protein_coding	1.071 65.537	0.938
93.469	0.99	86.084	1.02	95.593
ENSMUSG00000042369	Rbm45 protein_coding	0.969 28.584	0.958
61.883	1.077 6.449 0.982 93.734
ENSMUSG00000042371	Slc5a10	protein_coding	0.719 67.334
0.939 86.604	1.477 41.806	0.735 92.466
ENSMUSG00000042372	Dmrt3 protein_coding	1	86.351	1
92.404	NA	NA	1	94.297
ENSMUSG00000042377	Fam83g	protein_coding	0.863 44.787	NA
NA	1.141 48.935	0.936 84.29
ENSMUSG00000042379	Esm1	protein_coding	0.81	13.74 1.126 81.54
1.093 67.562	0.85	84.29
ENSMUSG00000042380	Smim12	protein_coding	0.909 11.128

0.958 92.474 1.146 9.502 1.048 83.067
ENSMUSG00000042388 Dlgap3 protein_coding 1.172 60.168 NA
NA 0.801 67.339 0.481 83.724
ENSMUSG00000042389 Tsen2 protein_coding 1.041 66.272 0.969
93.654 0.991 88.601 0.983 96.086
ENSMUSG00000042390 Gatad2b protein_coding 0.93 11.519
1.052 89.494 1.02 33.508 0.95 90.805
ENSMUSG00000042396 Rbm7 protein_coding 1.195 0 0.914 48.565
0.91 6.858 0.952 83.724
ENSMUSG00000042401 Crtac1 protein_coding 0.756 25.551
0.985 97.263 1.345 31.975 1.366 36.988
ENSMUSG00000042402 Olfr607 protein_coding 1 79.93 1
95.211 1 84.669 1 96.183
ENSMUSG00000042404 Dennd4b protein_coding 1.167 6.399 0.957
95.088 0.892 6.858 1.05 78.042
ENSMUSG00000042406 Atf4 protein_coding 0.979 68.955 1.086
72.642 0.942 44.019 1.03 82.091
ENSMUSG00000042408 Zmym6 protein_coding 0.987 80.251 NA NA
1.04 86.144 0.966 86.699
ENSMUSG00000042410 Agps protein_coding 0.802 16.652 0.903
95.088 1.381 50.851 1.221 91.65
ENSMUSG00000042414 Prdm14 protein_coding 1 82.092 1
95.088 1 84.504 1 93.132
ENSMUSG00000042417 Ccno protein_coding 0.606 9.841 1.201 95.196
1.376 32.173 0.708 84.19
ENSMUSG00000042419 Nfkbil1 protein_coding 0.808 18.731 0.95
70.29 1.302 7.24 1.029 96.287
ENSMUSG00000042423 Fbrs protein_coding 0.954 26.013 1.067
77.618 0.978 86.727 0.961 85.493
ENSMUSG00000042426 Dhx29 protein_coding 0.883 28.514 1.126
72.642 1.01 84.961 1.176 60.466
ENSMUSG00000042428 Mgat3 protein_coding 1.148 14.205 1.05
84.832 0.827 2.563 0.89 85.493
ENSMUSG00000042429 Adora1 protein_coding 0.877 40.217
0.994 96.68 1.145 84.961 1.247 60.968
ENSMUSG00000042433 E230019M04Rik protein_coding 1 80.251
NA NA 1 88.076 1 80.324
ENSMUSG00000042436 Mfap4 protein_coding 0.802 14.526 0.804
94.597 1.555 23.41 0.924 94.904
ENSMUSG00000042439 Zfp532 protein_coding 0.873 3.401 1.007
95.735 1.137 9.301 0.964 91.985
ENSMUSG00000042444 Fam63b protein_coding 1.056 81.657 0.93
70.693 1.02 73.759 0.971 95.72
ENSMUSG00000042446 Zmym4 protein_coding 1.048 35.499 0.968
83.58 0.987 80.749 0.973 95.273
ENSMUSG00000042447 Mios protein_coding 1.097 9.259 0.948 66.651
0.964 62.533 1.033 91.525
ENSMUSG00000042448 Hoxd1 protein_coding 1 38.555 1
91.065 1 87.786 1 89.152
ENSMUSG00000042451 Mybph protein_coding 1.616 15.662 1.136
87.604 0.545 4.963 0.697 91.105
ENSMUSG00000042453 Reln protein_coding 0.979 86.199 0.998
97.155 1.02 87.898 1.036 93.964
ENSMUSG00000042459 Bpifa2 protein_coding 1 78.299 1

95.708 1 83.719 1 92.018
ENSMUSG00000042460 C1galt1 protein_coding 0.963 83.658 NA
NA 1.05 84.308 1.171 65.401
ENSMUSG00000042462 Dctpp1 protein_coding 0.906 67.163
1.005 89.494 1.094 86.475 1.048 93.132
ENSMUSG00000042472 Zfp410 protein_coding 1.054 54.717 0.97
94.102 0.978 72.464 0.967 91.372
ENSMUSG00000042473 Tbc1d8b protein_coding 1.012 67.991
1.012 96.847 0.979 65.409 0.964 91.625
ENSMUSG00000042474 Faim3 protein_coding 1.217 36.195 0.659
85.95 1.247 85.21 0.782 85.493
ENSMUSG00000042476 Abcb4 protein_coding 2.559 0.2 1.043 82.904
0.374 0.72 0.808 92.12
ENSMUSG00000042477 Tfap2e protein_coding 2.901 11.638
0.431 73.461 0.797 73.874 0.696 84.19
ENSMUSG00000042485 Mustn1 protein_coding 0.935 35.158
0.849 66.651 1.262 20.315 0.998 95.877
ENSMUSG00000042487 Leo1 protein_coding 0.976 66.652 1.157
86.793 0.885 68.584 1.006 94.016
ENSMUSG00000042489 Clspn protein_coding 1.513 32.411 0.959
86.604 0.686 69.445 1.582 55.485
ENSMUSG00000042492 Tbc1d10b protein_coding 0.981 54.983
1.031 79.367 0.985 52.087 1.011 92.018
ENSMUSG00000042496 Prdm10 protein_coding 0.839 9.752 1.031
88.136 1.154 32.776 1.001 91.65
ENSMUSG00000042498 D330045A20Rik protein_coding 0.809 78.538
NA NA 1.433 54.809 1.319 94.384
ENSMUSG00000042499 Hoxd11 protein_coding 1 56.66 1
95.708 1 49.733 1 96.287
ENSMUSG00000042500 Ago4 protein_coding 1.086 60.853 0.854
35.072 1.077 52.574 0.85 85.493
ENSMUSG00000042501 Cpa6 protein_coding 1.07 60.853 1.467
71.617 0.639 7.948 1.065 96.358
ENSMUSG00000042502 Cd2bp2 protein_coding 0.97 42.922
0.992 82.947 1.04 13.082 1.05 35.053
ENSMUSG00000042505 Acn9 protein_coding 1.073 52.134 1.016
96.68 0.919 40.19 0.941 84.29
ENSMUSG00000042506 Usp22 protein_coding 1.077 7.174 1.021 95.211
0.909 16.051 0.978 90.611
ENSMUSG00000042507 Elmsan1 protein_coding 1.04 58.38 0.984
96.843 0.979 42.621 0.976 96.287
ENSMUSG00000042508 Dmtf1 protein_coding 1.038 34.077 0.987
94.262 0.975 81.388 1.01 96.086
ENSMUSG00000042510 AA986860 protein_coding 0.86 23.923
0.948 84.594 1.221 9.334 0.765 87.331
ENSMUSG00000042515 Mum1l1 protein_coding 0.978 74.958
0.636 26.053 1.609 24.985 0.517 83.724
ENSMUSG00000042520 Ubap2l protein_coding 0.997 75.196
1.047 92.793 0.952 63.075 0.999 91.011
ENSMUSG00000042523 Dnal1 protein_coding 1.006 85.391 0.856
42.923 1.158 6.202 0.894 83.724
ENSMUSG00000042524 Sun2 protein_coding 0.902 11.103 1.015
96.407 1.091 17.662 0.959 87.651
ENSMUSG00000042525 4933428M09Rik protein_coding 1 86.474

1 96.843 1 83.857 1 95.273
ENSMUSG00000042529 Kcnj12 protein_coding 1.773 14.205
1.347 79.055 0.419 1.061 0.651 92.31
ENSMUSG00000042532 Golga7b protein_coding 0.477 0.987 1.188
86.387 1.766 7.24 0.896 95.593
ENSMUSG00000042535 Gtpbp1 protein_coding 0.973 47.266
1.022 90.116 1.008 81.29 0.994 90.691
ENSMUSG00000042540 Acot5 protein_coding 0.432 24.817 0.858
88.897 2.694 17.662 0.962 96.287
ENSMUSG00000042541 Shfm1 protein_coding 0.932 39.612 0.991
95.504 1.077 43.481 1.037 71.305
ENSMUSG00000042548 Asxl1 protein_coding 0.948 7.004 NA NA
1.039 18.565 0.971 83.724
ENSMUSG00000042549 Gm16516 antisense 0.932 69.943 1.073
88.136 1 84.552 0.948 95.777
ENSMUSG00000042554 Zp3r protein_coding 1 75.858 1
96.847 1 82.436 1 88.512
ENSMUSG00000042557 Sin3a protein_coding 1.048 28.395 0.999
93.707 0.958 18.797 0.93 83.724
ENSMUSG00000042558 Adprhl2 protein_coding 0.951 18.379
0.976 95.088 1.078 29.819 0.957 86.991
ENSMUSG00000042564 Fam227a protein_coding 0.865 8.751 1.07
89.014 1.084 26.663 0.869 85.493
ENSMUSG00000042569 Dhrs7b protein_coding 0.97 81.52 0.98
82.848 1.05 79.625 0.912 83.724
ENSMUSG00000042570 Mier2 protein_coding 0.912 18.795 1.024
91.361 1.068 23.334 1.055 74.193
ENSMUSG00000042572 Ube2q1 protein_coding 1.03 16.539
1.018 92.253 0.956 21.666 1.009 77.854
ENSMUSG00000042579 4632404H12Rik protein_coding 0.814 1.703
1.012 96.897 1.213 7.635 0.963 90.157
ENSMUSG00000042581 Thsd7b protein_coding 1.425 40.333
0.752 82.671 0.93 84.669 0.77 92.31
ENSMUSG00000042589 Cux2 protein_coding 1.381 13.363 0.784
68.115 0.925 52.972 1.003 96.086
ENSMUSG00000042590 Ipo11 protein_coding 0.998 78.224 0.95
82.671 1.052 45.667 1.03 86.209
ENSMUSG00000042594 Sh2b3 protein_coding 0.978 75.567 1.041
92.561 0.981 86.112 1.009 92.748
ENSMUSG00000042595 Fam199x protein_coding 0.914 20.116
0.973 95.088 1.127 34.727 0.936 83.724
ENSMUSG00000042596 Tfap2d protein_coding 1 83.09 1
95.088 1 84.961 1 94.904
ENSMUSG00000042599 Jhdm1d protein_coding 1.102 51.189
0.815 58.64 1.11 36.011 0.979 89.892
ENSMUSG00000042604 Kcna4 protein_coding 1 86.464 NA NA
1 84.308 1 94.904
ENSMUSG00000042605 Atxn2 protein_coding 1.046 41.211 1.094
79.29 0.87 8.907 0.979 92.181
ENSMUSG00000042606 Hirip3 protein_coding 0.948 44.787
0.978 95.211 1.079 79.897 1.109 40.894
ENSMUSG00000042607 Asb4 protein_coding 1.688 1.493 0.712 67.165
0.828 23.938 1.009 94.384
ENSMUSG00000042608 Stk40 protein_coding 1.011 84.049 1.162

60.309 0.847 2.361 0.974 95.72
ENSMUSG00000042613 Pbxip1 protein_coding 1.043 32.676
0.907 75.993 1.061 85.757 0.997 93.964
ENSMUSG00000042616 Oscp1 protein_coding 0.712 5.686 1.171 95.809
1.201 14.597 1.009 96.248
ENSMUSG00000042622 Maff protein_coding 0.851 24.344 0.856
55.71 1.369 13.259 1.034 93.261
ENSMUSG00000042625 Safb2 protein_coding 0.907 39.953 1.019
91.584 1.084 66.928 0.967 84.29
ENSMUSG00000042626 Shc1 protein_coding 0.995 65.199 1.053
72.642 0.955 47.641 0.996 95.35
ENSMUSG00000042628 Zfyve1 protein_coding 0.972 34.162
1.016 96.843 1.013 51.977 0.947 83.724
ENSMUSG00000042631 Xkr7 protein_coding 1 65.284 1
92.137 1 63.919 1 86.991
ENSMUSG00000042632 Pla2g6 protein_coding 0.816 0.987 1.11
65.165 1.104 25.455 0.974 95.405
ENSMUSG00000042641 Rgsl1 protein_coding 0.65 11.384 1.265
86.34 1.219 37.741 0.972 96.358
ENSMUSG00000042642 Flad1 protein_coding 0.929 14.403 0.983
87.029 1.093 61.418 1.005 96.192
ENSMUSG00000042644 Itpr3 protein_coding 0.995 74.516 0.909
65.819 1.104 38.683 0.938 84.29
ENSMUSG00000042647 Acad12 protein_coding 1.045 67.991
0.887 44.566 1.077 35.673 0.978 90.157
ENSMUSG00000042650 Alkbh5 protein_coding 1.06 13.363
1.078 71.98 0.877 1.061 0.952 83.724
ENSMUSG00000042655 Fam159b protein_coding 1 85.832 1
94.767 1 69.974 NA NA
ENSMUSG00000042659 Arrdc4 protein_coding 0.988 80.251
0.962 95.504 1.052 78.864 0.933 83.724
ENSMUSG00000042660 Wdr55 protein_coding 1.044 69.089 0.966
92.474 0.993 84.961 1.033 90.177
ENSMUSG00000042662 Dusp15 protein_coding 0.919 18.907 NA
NA 1.184 42.522 0.984 96.183
ENSMUSG00000042670 Immp1l protein_coding 1.041 46.013
1.053 94.767 0.915 25.052 0.959 94.654
ENSMUSG00000042672 Dcst1 protein_coding 1.021 76.546 1.104
82.904 0.883 60.233 1.093 95.273
ENSMUSG00000042675 Ypel3 protein_coding 0.942 49.621 0.937
68.818 1.133 24.791 0.851 83.724
ENSMUSG00000042677 Zc3h12a protein_coding 0.903 56.242
1.229 83.104 0.9 63.075 0.986 88.053
ENSMUSG00000042678 Myo15 protein_coding 1.097 63.804 0.79
83.58 1.155 73.759 1.044 90.125
ENSMUSG00000042680 Garem protein_coding 1.058 52.986 1.001
95.504 0.945 42.376 0.866 83.724
ENSMUSG00000042682 Selk protein_coding 1.006 85.655 0.96
90.712 1.037 66.269 1.006 88.797
ENSMUSG00000042684 Npl protein_coding 1.465 1.323 NA NA
0.703 8.472 1.636 44.882
ENSMUSG00000042686 Jph1 protein_coding 1.989 8.477 1.133 83.104
0.443 0.945 0.791 94.608
ENSMUSG00000042688 Mapk6 protein_coding 0.997 86.843 1.034

96.843 0.972 78.954 0.994 95.676
ENSMUSG00000042694 NA NA NA NA 1.06 87.609 0.948
55.214 1.111 44.213
ENSMUSG00000042699 Dhx9 protein_coding 1.026 72.85 0.953 85.921
1.021 80.381 1.062 31.133
ENSMUSG00000042700 Sipa1l1 protein_coding 1.02 66.272
1.149 64.123 0.852 7.809 1.206 83.276
ENSMUSG00000042705 Commd10 protein_coding 1.085 19.315
0.978 95.504 0.945 15.778 1.039 83.067
ENSMUSG00000042707 Dnali1 protein_coding 0.89 76.319
0.747 89.713 1.498 61.171 1.026 86.445
ENSMUSG00000042708 Shcbp1l protein_coding 1.397 28.927
0.736 72.399 0.975 87.431 1.112 95.405
ENSMUSG00000042709 Atpaf2 protein_coding 0.939 11.769
1.056 79.261 1.009 79.625 0.94 86.699
ENSMUSG00000042712 Wbp5 protein_coding 0.827 22.481 0.922
50.664 1.309 5.864 1.05 93.156
ENSMUSG00000042717 Ppp1r3a protein_coding 5.3 5.848 1.436
85.756 0.132 0.72 0.959 95.273
ENSMUSG00000042719 Naa25 protein_coding 1.017 72.655 0.999
95.211 0.983 82.685 1.028 83.276
ENSMUSG00000042724 Map3k9 protein_coding 0.742 1.175 1.041
83.104 1.298 11.518 0.935 86.699
ENSMUSG00000042726 Trafd1 protein_coding 1.023 62.689
0.899 79.475 1.089 81.857 1.034 96.248
ENSMUSG00000042729 Wdr74 protein_coding 0.913 23.641 0.977
86.277 1.124 23.604 1.069 83.067
ENSMUSG00000042734 Ttc9 protein_coding 0.811 45.728 1.024
95.735 1.201 58.616 0.992 92.181
ENSMUSG00000042737 Dpm3 protein_coding 0.895 10.704 1.039
84.832 1.076 37.741 1.077 73.823
ENSMUSG00000042742 B630005N14Rik protein_coding 1.132 2.512
1.001 95.088 0.879 8.192 0.961 86.699
ENSMUSG00000042743 Sgtb protein_coding 1.052 33.325 0.96
75.052 0.985 83.066 1.057 92.018
ENSMUSG00000042744 Gm15800 protein_coding 0.903 1.703 1.096
71.222 1.005 39.925 0.988 96.358
ENSMUSG00000042745 Id1 protein_coding 0.812 10.704 0.9
56.781 1.369 5.864 1.072 79.713
ENSMUSG00000042747 Krtcap2 protein_coding 0.991 85.203
1.073 72.642 0.941 13.848 1.039 83.276
ENSMUSG00000042750 Bex2 protein_coding 0.751 47.266 0.996
95.196 1.334 72.378 0.893 93.722
ENSMUSG00000042751 Nmnat2 protein_coding 1.4 5.313 0.893
91.275 0.798 44.865 1.019 96.192
ENSMUSG00000042757 NA NA NA NA NA NA 0.936 84.434
0.465 91.291
ENSMUSG00000042759 Apobr protein_coding 1.224 7.492 0.889 87.919
0.92 38.434 1.187 82.85
ENSMUSG00000042761 Mrap2 protein_coding 1 66.272 1
94.96 NA NA 1 93.132
ENSMUSG00000042763 Maneal protein_coding 0.921 68.353
1.468 79.367 0.738 69.974 0.924 95.74
ENSMUSG00000042766 Trim46 protein_coding 0.704 5.686 1.269

79.055	1.116 60.104	1.043 93.685
ENSMUSG00000042770	Hebp1 protein_coding	0.82	3.623 1.051 89.494
1.154 17.412	1.077 83.067
ENSMUSG00000042772	Smg7	protein_coding	1.033 21.836	1.076
63.504	0.9	0.72	1.038 54.265
ENSMUSG00000042774	NA	NA	NA	NA	NA	NA	1	81.492
1	95.74
ENSMUSG00000042784	Muc1	protein_coding	0.75	4.115 1.204 71.617
1.11	71.615	0.845 83.724
ENSMUSG00000042787	Exog	protein_coding	0.989 80.815	1.039
85.756	0.975 39.528	1.167 68.502
ENSMUSG00000042788	Fam166b	protein_coding	0.588 46.413
0.945 94.48 1.802 38.152	0.79	87.735
ENSMUSG00000042790	Rnf214	protein_coding	0.945 32.168
1.022 94.367	1.038 64.024	0.977 86.991
ENSMUSG00000042793	Lgr6	protein_coding	0.815 18.795	1.133
91.643	1.084 50.636	0.774 83.724
ENSMUSG00000042796	Olfr1032	protein_coding	1	78.538	1
96.138	1	83.719	1	95.273
ENSMUSG00000042797	Aqp11 protein_coding	0.653 8.181 1.18	95.735
1.3	23.454	1.034 94.567
ENSMUSG00000042800	1700015E13Rik	protein_coding	1.071 77.763
1.189 97.263	0.787 76.225	0.491 84.19
ENSMUSG00000042801	Olfr769	protein_coding	1	84.049	NA
NA	1	81.525	1	95.72
ENSMUSG00000042804	Gpr153	protein_coding	1.096 62.574
1.037 97.049	0.882 70.44 1.306 31.133
ENSMUSG00000042807	Hecw2 protein_coding	0.943 79.709	0.796
37.426	1.329 21.522	1.12	90.177
ENSMUSG00000042808	Gpx2	protein_coding	1.418 54.717	1.019
87.313	0.694 75.353	1.691 72.826
ENSMUSG00000042810	Krba1 protein_coding	0.864 5.962 1.138 83.104
1.02	32.173	0.994 96.071
ENSMUSG00000042812	Foxf1 protein_coding	1.349 21.278	1.058
96.984	0.698 26.809	0.934 87.496
ENSMUSG00000042814	Mcts2 protein_coding	0.887 44.562	1.06
87.604	1.06	84.308	0.92	95.877
ENSMUSG00000042817	Flt3	protein_coding	0.849 39.953	1.078
85.756	1.09	67.823	0.974 93.584
ENSMUSG00000042821	NA	NA	NA	NA	1.095 88.551	0.929
39.755	1.323 42.736
ENSMUSG00000042826	Fgf11 protein_coding	0.975 80.251	1.084
76.065	0.942 26.369	1.006 87.182
ENSMUSG00000042828	Trim72	protein_coding	2.976 8.102 1.312
90.952	0.255 0.72	1.207 86.445
ENSMUSG00000042831	Alkbh6	protein_coding	0.877 14.849
1.031 92.111	1.105 50.091	1.109 83.067
ENSMUSG00000042834	Nrep	protein_coding	1.187 6.541 1.312 79.055
0.641 2.087 1.071 73.597
ENSMUSG00000042842	Serpinb6b	protein_coding	0.952 70.862
1.176 67.843	0.893 33.971	1.037 96.183
ENSMUSG00000042845	Wfdc12	protein_coding	0.53	19.023
1.163 92.561	1.621 45.208	1.63	68.502
ENSMUSG00000042848	Vmn1r226	protein_coding	1	82.732	1

93.654	1	84.071	1	93.722
ENSMUSG00000042849	Olfr1414	protein_coding	1	86.145	1
96.216	1	80.015	1	95.273
ENSMUSG00000042851	Zc3h6	protein_coding	0.765	53.407	0.947
91.532	1.374	34.807	1.005	93.685
ENSMUSG00000042854	Trp53rk	protein_coding	0.822	17.664
1.093	83.104	1.113	43.586	0.996	95.273
ENSMUSG00000042857	Gm9776	protein_coding	0.846	11.232
1.007	96.897	1.174	38.26	1.31	33.269
ENSMUSG00000042861	Kcna10	protein_coding	1	50.913	NA
NA	1	88.274	1	96.183
ENSMUSG00000042863	Olfr1026	protein_coding	1	85.655	NA
NA	1	88.601	1	95.559
ENSMUSG00000042869	Olfr1370	protein_coding	1	84.049	NA
NA	1	84.504	1	94.468
ENSMUSG00000042870	Tom1	protein_coding	0.939	17.031	1.085
67.843	0.983	78.045	1.069	78.042
ENSMUSG00000042873	Lhfpl4	protein_coding	1.246	21.146	NA
NA	0.862	46.071	0.977	83.067
ENSMUSG00000042874	D930007J09Rik	protein_coding	1.045	73.487
1.057	95.735	0.903	84.552	0.779	84.29
ENSMUSG00000042888	1700110M21Rik	protein_coding	1	86.199
1	96.843	NA	NA	1	96.183
ENSMUSG00000042894	Olfr1260	protein_coding	1	86.918	1
93.046	1	72.464	1	96.287
ENSMUSG00000042901	Aida	protein_coding	1.126	1.161	1.013	90.952
0.877	4.538	1.156	61.316
ENSMUSG00000042903	Foxo4	protein_coding	0.974	79.165	0.927
63.912	1.107	62.237	0.989	92.748
ENSMUSG00000042909	Olfr648	protein_coding	1	77.763	1
95.504	1	81.29	1	93.156
ENSMUSG00000042918	Mamstr	protein_coding	1.129	26.863
0.872	70.29	1.012	87.173	1.064	86.445
ENSMUSG00000042938	Gm14117	pseudogene	1	83.523	1
96.216	1	86.831	1	94.904
ENSMUSG00000042940	9230106D20Rik	protein_coding	1	80.751
1	96.138	1	81.492	1	92.31
ENSMUSG00000042942	Greb1l	protein_coding	0.941	60.435
0.938	71.331	1.134	60.5	1.322	85.577
ENSMUSG00000042961	Egflam	protein_coding	1.247	3.536	1.011
87.998	0.791	12.948	1.038	96.192
ENSMUSG00000042962	Gm5436	pseudogene	1	84.977	1
97.263	1	76.754	1	94.016
ENSMUSG00000042976	9930038B18Rik	lincRNA	0.738	53.138
1.049	96.843	1.291	62.307	1.316	76.347
ENSMUSG00000042978	Sbk1	protein_coding	0.743	1.175	1.314	72.546
1.019	43.842	1.043	63.708
ENSMUSG00000042988	Notum	protein_coding	0.552	68.66	1.157	84.126
1.569	85.935	1.632	86.133
ENSMUSG00000042992	Loh12cr1	protein_coding	0.915	11.924	1
96.843	1.096	23.604	1.155	31.133
ENSMUSG00000042993	Ifnk	protein_coding	1	85.355	1
90.053	1	56.125	1	94.384
ENSMUSG00000042997	Nhlrc3	protein_coding	1.041	39.851

0.902 45.961 1.062 38.099 1.003 92.018
ENSMUSG00000043003 Rasef protein_coding 1.381 43.939 NA NA
0.679 63.919 1.106 92.762
ENSMUSG00000043004 Gng2 protein_coding 1.224 0 0.908 37.187
0.897 6.858 1.038 96.042
ENSMUSG00000043008 Klhl6 protein_coding 1.273 0.767 0.766 43.505
1.026 71.999 1.21 74.047
ENSMUSG00000043013 Onecut1 protein_coding 1 86.474 NA
NA 1 84.229 1 96.358
ENSMUSG00000043015 Tmem194b protein_coding 0.943 45.229 0.9
85.075 1.176 40.575 0.995 92.263
ENSMUSG00000043017 Ptgir protein_coding 1.114 29.536 0.878
85.95 1.019 60.233 1.229 37.634
ENSMUSG00000043019 Edem3 protein_coding 0.993 83.823 0.982
93.654 1.027 78.864 0.958 96.183
ENSMUSG00000043020 Wdr63 protein_coding 0.785 68.66 0.98 95.504
1.3 88.729 1.735 87.134
ENSMUSG00000043024 Gm16433 pseudogene 1 62.888 1
96.897 1 75.155 1 95.273
ENSMUSG00000043029 Trpv3 protein_coding 0.617 16.183 1.318
71.617 1.231 72.695 1.05 92.675
ENSMUSG00000043036 Ccdc63 protein_coding 2.874 72.85 0.502
97.155 0.696 88.729 1.045 91.525
ENSMUSG00000043050 Tnp2 protein_coding 1 78.299 1
91.955 1 64.024 1 93.964
ENSMUSG00000043051 Disc1 protein_coding 0.974 81.01 0.907 79.475
1.132 43.481 1.093 95.92
ENSMUSG00000043059 Zfp513 protein_coding 0.907 9.752 1.018
96.138 1.082 9.502 0.984 90.674
ENSMUSG00000043060 Fscb protein_coding 1 83.658 1
97.155 1 82.74 1 96.287
ENSMUSG00000043061 Tmem18 protein_coding 1.133 0.664 0.965
85.075 0.912 5.336 0.981 91.291
ENSMUSG00000043065 Spice1 protein_coding 1.03 83.169 0.95
85.418 1.023 77.532 0.925 85.493
ENSMUSG00000043066 Vmn1r66 protein_coding 1 76.396 1
92.137 1 72.853 1 92.181
ENSMUSG00000043067 Dpy19l1 protein_coding 1.038 34.696 1.02
90.092 0.946 30.024 1.132 40.024
ENSMUSG00000043068 Fam89a protein_coding 0.95 26.483
0.988 96.335 1.069 37.764 0.792 86.991
ENSMUSG00000043073 Usp17le protein_coding 1 75.858 NA
NA 1 86.552 1 92.903
ENSMUSG00000043079 Synpo protein_coding 1.02 74.482 0.975
95.504 1.008 78.045 1.316 35.053
ENSMUSG00000043085 Tmem82 protein_coding 1.13 32.873
0.837 56.781 1.055 71.615 0.755 84.19
ENSMUSG00000043087 Olfr855 protein_coding 1 81.24 1
96.216 NA NA 1 94.904
ENSMUSG00000043088 Il17re protein_coding 0.966 28.584
0.876 71.889 1.185 20.572 0.885 85.493
ENSMUSG00000043089 Mmp1a protein_coding 1 83.658 1
97.155 1 84.552 1 89.827
ENSMUSG00000043090 Zfp866 protein_coding 1.035 46.875 NA

NA	0.984 64.778	1.012 94.016
ENSMUSG00000043091	Tuba1c	protein_coding	1.037 64.714
1.006 94.848	0.957 78.293	1.223 56.863
ENSMUSG00000043099	Hic1	protein_coding	1.036 67.608	1.088
90.116	0.885 24.163	1.238 42.736
ENSMUSG00000043102	Qrfp	protein_coding	0.373 33.002	1.143
96.308	2.347 56.125	2.943 83.067
ENSMUSG00000043110	Lrrn4 protein_coding	1.234 9.259 0.936 95.504
0.864 11.997	0.887 84.29
ENSMUSG00000043119	Olfr448	protein_coding	1	83.418	1
97.263	1	87.744	1	93.627
ENSMUSG00000043122	A530016L24Rik	protein_coding	0.85	13.45
1.049 82.904	1.119 45.208	0.956 94.654
ENSMUSG00000043126	D830039M14Rik	protein_coding	1.411 78.428
1.014 84.832	0.697 54.521	1.411 72.992
ENSMUSG00000043131	Mob1a protein_coding	1.166 0	0.989 96.68
0.866 1.58	0.964 88.955
ENSMUSG00000043140	Tmem186	protein_coding	1.029 66.58 0.987
88.705	0.983 87.173	0.983 92.181
ENSMUSG00000043145	Gm11292	processed_transcript	1	76.546
1	95.708	1	78.642	1	96.287
ENSMUSG00000043153	Ispd	protein_coding	1.071 24.253	0.962
87.857	0.969 85.757	0.924 83.724
ENSMUSG00000043154	Ppp2r3a	protein_coding	1.199 0.355 1.055
76.086	0.79	0.72	0.974 95.593
ENSMUSG00000043155	Hpdl	protein_coding	0.709 50.768	0.732
59.946	1.924 16.808	1.059 96.183
ENSMUSG00000043157	Arl11 protein_coding	1.785 0	0.994 90.046
0.562 2.563 1.067 92.263
ENSMUSG00000043162	Pyurf protein_coding	1	86.145	1
95.941	1	78.954	1	95.74
ENSMUSG00000043165	Lor	protein_coding	0.87	63.736	0.923
96.948	1.242 81.492	0.906 86.699
ENSMUSG00000043177	Gm11993	pseudogene	1	83.333	1
96.652	1	84.229	1	95.273
ENSMUSG00000043183	Simc1 protein_coding	1.053 25.944	0.985
72.712	0.96	78.713	0.978 95.72
ENSMUSG00000043190	Rfesd protein_coding	0.905 27.431	0.959
93.094	1.147 13.259	0.999 91.335
ENSMUSG00000043192	Gm1840	protein_coding	1	81.178	1
94.887	1	84.626	1	95.273
ENSMUSG00000043207	Zmpste24	protein_coding	0.993 72.655
1.026 96.378	0.979 55.9	1.033 52.366
ENSMUSG00000043219	Hoxa6 protein_coding	0.824 48.146	1.181
71.617	1.029 54.037	1.052 86.699
ENSMUSG00000043223	Gm4835	pseudogene	1	83.823	1
97.155	1	85.757	1	93.964
ENSMUSG00000043226	Olfr1009	protein_coding	1	76.968	1
97.263	1	80.749	1	96.287
ENSMUSG00000043230	Fam124b	protein_coding	0.374 13.062
1.052 95.431	2.527 42.522	0.628 87.735
ENSMUSG00000043241	Upf2	protein_coding	1.095 0.577 0.974 83.733
0.936 20.751	0.981 96.086
ENSMUSG00000043243	Fam129c	protein_coding	0.93	43.495

1.352 87.037 0.792 86.985 1.012 83.067
ENSMUSG00000043251 Exoc3l protein_coding 0.735 5.962 1.064
90.116 1.282 20.572 1.003 91.372
ENSMUSG00000043252 Tmem64 protein_coding 1.019 81.76 0.981
96.68 1.001 71.734 0.889 83.724
ENSMUSG00000043257 Pigv protein_coding 0.94 34.162 1.029
88.551 1.036 84.308 0.949 84.29
ENSMUSG00000043259 Fam13c protein_coding 1.286 0.767 0.931
93.654 0.833 0.72 1.068 88.197
ENSMUSG00000043262 Uevld protein_coding 1.092 7.083 0.959 79.475
0.956 28.901 0.978 88.505
ENSMUSG00000043263 Pyhin1 protein_coding 1.692 0.355 0.866
84.594 0.682 15.778 1.406 64.513
ENSMUSG00000043267 Olfr1031 protein_coding 1 84.397 1
89.277 1 84.071 1 94.904
ENSMUSG00000043274 Olfr1123 protein_coding 1 82.092 1
97.288 1 87.744 1 93.964
ENSMUSG00000043279 Trim56 protein_coding 1.013 72 0.969
77.598 1.014 75.155 1.017 95.877
ENSMUSG00000043284 Tmem11 protein_coding 0.966 52.018 NA
NA 1.031 62.995 0.932 86.699
ENSMUSG00000043290 Zfp784 protein_coding 0.877 14.221
0.901 71.98 1.27 7.024 1.006 92.903
ENSMUSG00000043295 Gm16373 pseudogene 1 85.637 1
95.329 1 86.346 1 95.877
ENSMUSG00000043298 Smco3 protein_coding 1.05 66.272 1.104
93.899 0.861 33.689 0.824 84.29
ENSMUSG00000043300 B3galnt1 protein_coding 1.159 14.59 0.924
86.071 0.933 41.154 1.099 60.31
ENSMUSG00000043301 Kcnj6 protein_coding 1 85.77 1 92.561
1 76.388 1 95.74
ENSMUSG00000043308 Vmn1r75 protein_coding 1 84.324 1
93.27 1 87.786 1 92.742
ENSMUSG00000043310 Olfr571 protein_coding 1 80.146 1
97.263 1 81.857 1 94.904
ENSMUSG00000043311 D17H6S53E protein_coding 0.902 11.769
1.113 60.309 0.993 60.96 1.097 52.366
ENSMUSG00000043312 Olfr131 protein_coding 1 84.865 1
94.597 1 83.275 1 96.358
ENSMUSG00000043313 Pcdhb19 protein_coding 0.896 22.142
1.154 82.904 0.964 75.009 1.012 75.198
ENSMUSG00000043314 Olfr30 protein_coding 1 86.351 1
95.35 1 82.436 1 96.287
ENSMUSG00000043319 Cox8c protein_coding 1 74.482 1
95.809 1 84.83 1 95.877
ENSMUSG00000043323 Fbrsl1 protein_coding 0.854 8.122 1.134
82.904 1.035 56.497 0.876 83.724
ENSMUSG00000043329 Gm8849 pseudogene 1 85.283 1
96.847 1 88.41 1 95.35
ENSMUSG00000043331 Olfr975 protein_coding 1 80.815 1
94.96 1 84.896 NA NA
ENSMUSG00000043333 Rhbdl2 protein_coding 0.928 85.857
0.934 83.876 1.15 81.032 1.178 96.071
ENSMUSG00000043336 Filip1l protein_coding 1.228 10.687

1.017 93.899 0.8 7.742 0.971 93.734
ENSMUSG00000043340 6530409C15Rik protein_coding 0.924 62.437
1.014 96.138 1.068 75.809 0.87 88.505
ENSMUSG00000043342 Hoxd9 protein_coding 0.945 34.572 0.962
96.847 1.102 41.235 0.957 92.675
ENSMUSG00000043346 Gm6741 protein_coding 1 71.06 1
95.708 1 70.791 1 93.584
ENSMUSG00000043354 Olfr577 protein_coding 1 73.585 1
92.914 1 86.475 1 91.928
ENSMUSG00000043357 Olfr187 protein_coding 1 80.751 1
96.138 1 80.5 1 95.35
ENSMUSG00000043366 Olfr78 protein_coding 1.41 0.976 1.025
84.832 0.693 1.061 0.874 83.724
ENSMUSG00000043372 Hexim2 protein_coding 0.919 25.492 0.92
92.355 1.18 30.292 0.897 86.991
ENSMUSG00000043383 Olfr1342 protein_coding 1 75.298 NA
NA 1 66.928 1 96.183
ENSMUSG00000043384 Gprasp1 protein_coding 1.074 61.725
0.894 85.793 1.038 76.923 1.011 95.92
ENSMUSG00000043385 Olfr267 protein_coding 1 69.489 1
94.262 1 74.535 1 89.525
ENSMUSG00000043388 Tmem130 protein_coding 0.855 55.939
0.855 88.705 1.37 43.586 1.365 94.153
ENSMUSG00000043391 2510009E07Rik protein_coding 1.11 5.527 NA
NA 0.919 4.389 1.027 96.287
ENSMUSG00000043398 Gpr135 protein_coding 0.936 36.034
0.988 83.104 1.078 83.397 0.891 90.289
ENSMUSG00000043411 Usp48 protein_coding 1.028 24.147 0.961
71.889 1.012 83.066 0.996 95.832
ENSMUSG00000043415 Otud1 protein_coding 0.978 49.047 0.875
82.947 1.165 34.807 0.939 86.699
ENSMUSG00000043419 A030009H04Rik processed_transcript 1.313
7.799 1.054 97.155 0.723 7.647 0.951 85.493
ENSMUSG00000043421 Hilpda protein_coding 1.009 84.452
0.843 49.859 1.171 10.76 1.087 86.301
ENSMUSG00000043429 NA NA NA NA 1 94.102 1
57.637 1 95.273
ENSMUSG00000043430 Psapl1 protein_coding 0.787 45.728
0.624 38.699 2.03 7.635 1.067 91.807
ENSMUSG00000043432 Leng9 protein_coding 0.87 28.584 1.112
78.705 1.036 79.119 1.026 92.748
ENSMUSG00000043439 E130012A19Rik protein_coding 0.763 18.138
0.971 93.765 1.348 23.454 1.222 74.607
ENSMUSG00000043445 Pgp protein_coding 0.938 85.129 1.12
91.643 0.952 88.161 0.978 94.19
ENSMUSG00000043448 Gjc2 protein_coding 1.223 30.788 0.961
95.504 0.852 47.12 0.874 92.12
ENSMUSG00000043453 Magea10 protein_coding 1 84.049 NA
NA 1 88.657 1 95.676
ENSMUSG00000043456 Zfp536 protein_coding 0.928 78.744
1.119 84.768 0.958 78.045 0.941 84.29
ENSMUSG00000043458 Pcdhb12 protein_coding 0.991 74.299
1.343 65.165 0.748 10.23 1.032 85.577
ENSMUSG00000043460 Elfn2 protein_coding 0.601 76.968 1.485

95.504 1.116 68.129 1.3 86.133
ENSMUSG00000043463 Rab9b protein_coding 1.137 82.231 0.951
92.931 0.925 68.82 0.972 95.273
ENSMUSG00000043467 Zbtb37 protein_coding 0.959 82.092
1.169 88.551 0.894 78.954 0.925 91.372
ENSMUSG00000043468 Adam30 protein_coding 1 58.198 1
90.053 1 79.524 1 91.078
ENSMUSG00000043472 Lce3d protein_coding 1 84.049 1
93.469 1 77.704 1 93.734
ENSMUSG00000043484 Gm5867 pseudogene 1 84.977 1
94.848 1 87.431 1 93.612
ENSMUSG00000043485 Krt34 protein_coding 1 86.199 1
96.268 1 80.899 1 95.877
ENSMUSG00000043487 Acot6 protein_coding 1.053 74.482 0.645
33.301 1.474 11.518 0.945 86.699
ENSMUSG00000043488 Gm9783 antisense 0.901 84.819 1.171
96.843 0.951 86.258 0.92 84.29
ENSMUSG00000043496 Tril protein_coding 1.063 38.682 0.959
84.017 0.98 85.58 0.866 83.724
ENSMUSG00000043498 9330132A10Rik pseudogene 1.049 57.133 NA
NA 0.859 54.521 0.969 86.991
ENSMUSG00000043501 Lgals2 protein_coding 0.71 66.129
1.086 94.262 1.302 48.772 0.906 91.372
ENSMUSG00000043505 Gimap5 protein_coding 1.082 72.27 1.063
88.823 0.865 25.688 0.905 84.19
ENSMUSG00000043510 Hscb protein_coding 1.106 20.444 0.962
88.691 0.94 71.388 0.965 85.493
ENSMUSG00000043518 Rai2 protein_coding 0.941 28.584 1.127
82.904 0.94 74.535 1.024 83.067
ENSMUSG00000043529 Olfr266 protein_coding 1 84.865 1
95.708 1 70.9 1 90.674
ENSMUSG00000043531 Sorcs1 protein_coding 1.07 78.428
1.085 82.904 0.859 18.965 0.923 84.29
ENSMUSG00000043535 Setx protein_coding 0.77 4.874 0.919 72.399
1.418 5.864 1.054 95.273
ENSMUSG00000043537 Vmn1r225 protein_coding 1 84.324 1
94.262 1 81.857 1 96.358
ENSMUSG00000043541 Casc1 protein_coding 0.993 86.172 0.694
81.02 1.45 42.522 0.711 86.699
ENSMUSG00000043542 Zc2hc1a protein_coding 1.098 57.133
1.085 79.367 0.836 10.726 0.876 83.724
ENSMUSG00000043549 4932442L08Rik protein_coding 1 78.639
1 95.211 1 79.625 1 95.35
ENSMUSG00000043556 Fbxl7 protein_coding 1.087 42.255 0.92
82.503 0.996 86.258 0.915 84.19
ENSMUSG00000043557 Mdga1 protein_coding 1.566 7.083 NA NA
0.567 7.809 0.901 94.904
ENSMUSG00000043569 4930412D23Rik protein_coding 1 78.224
1 96.763 1 86.258 1 96.183
ENSMUSG00000043572 Pars2 protein_coding 1.015 82.231 1.052
96.843 0.931 88.076 0.92 85.493
ENSMUSG00000043583 4930470P17Rik protein_coding 1 86.918
NA NA NA NA 1 93.264
ENSMUSG00000043587 Acpl2 protein_coding 0.92 31.11 NA NA

1.091 42.199 0.979 92.466
ENSMUSG00000043592 Unc5cl protein_coding 0.825 51.56 1.103
87.609 1.101 81.492 0.952 86.991
ENSMUSG00000043602 Zfp3 protein_coding 0.982 71.763 0.919
87.322 1.11 42.75 0.99 96.248
ENSMUSG00000043605 Olfr13 protein_coding 1 82.891 1
95.708 1 87.971 1 94.825
ENSMUSG00000043613 Mmp3 protein_coding 1.504 1.689 0.873 58.64
0.763 23.938 1.057 83.067
ENSMUSG00000043614 Vps37d protein_coding 0.863 70.862
1.014 92.404 1.143 40.955 0.986 88.505
ENSMUSG00000043618 Eif5al3-ps pseudogene 1 81.465 NA NA
1 85.675 1 93.685
ENSMUSG00000043621 Ubxn10 protein_coding 0.774 58.802
0.883 84.017 1.461 36.388 0.929 94.016
ENSMUSG00000043623 4933412A08Rik pseudogene 0.801 8.139 0.989
88.533 1.262 27.431 0.837 83.724
ENSMUSG00000043629 1700019D03Rik protein_coding 1.24 13.492
1.036 92.111 0.775 6.858 1.073 82.85
ENSMUSG00000043631 Ecm2 protein_coding 1.129 24.253 1.047
95.211 0.848 29.765 1.014 94.174
ENSMUSG00000043633 Fam221b protein_coding 1.047 84.661 1.04
92.914 NA NA 1.176 92.381
ENSMUSG00000043635 Adamts3 protein_coding 0.912 69.24 0.913
72.712 1.196 33.287 0.816 90.611
ENSMUSG00000043639 Rbm20 protein_coding 0.591 6.952 1.306 60.309
1.291 47.31 1.01 83.067
ENSMUSG00000043644 0610009L18Rik protein_coding 0.951 76.968
0.951 87.452 1.104 61.841 0.876 86.699
ENSMUSG00000043648 Pld6 protein_coding 1.416 34.439 0.895
94.262 0.787 44.019 0.731 83.724
ENSMUSG00000043659 Npsr1 protein_coding 1 73.124 1
96.013 1 75.009 1 95.74
ENSMUSG00000043661 Gm9785 pseudogene 1.116 82.352 0.762
91.361 1.171 65.8 0.606 92.31
ENSMUSG00000043664 Tmem221 protein_coding 1.205 21.703
0.981 96.013 0.846 31.887 0.928 88.136
ENSMUSG00000043668 Tox3 protein_coding 1.355 12.855 0.886
69.175 0.831 76.768 0.732 83.724
ENSMUSG00000043670 Diras1 protein_coding 0.883 73.292
1.415 95.196 0.801 84.724 0.364 88.505
ENSMUSG00000043671 Dpy19l3 protein_coding 1.312 2.512 0.963
96.216 0.789 1.973 0.912 86.991
ENSMUSG00000043673 Kcns3 protein_coding 1.054 84.294 NA NA
0.957 79.776 0.909 91.372
ENSMUSG00000043681 Fam25c protein_coding 0.905 68.353
0.647 32.358 1.712 8.586 0.677 90.289
ENSMUSG00000043683 Fem1a protein_coding 1.02 56.907 1.091
64.304 0.901 1.58 1.032 92.018
ENSMUSG00000043687 1190005I06Rik protein_coding 0.798 32.749
1.125 87.037 1.117 65.567 0.784 90.157
ENSMUSG00000043692 Olfr399 protein_coding 1 81.925 1
96.216 1 81.29 1 94.904
ENSMUSG00000043698 Olfr62 protein_coding 1 73.292 1

97.263	1	87.103	1	93.964
ENSMUSG00000043702	Pde12	protein_coding	1.012	77.291	1.036
82.76	0.952	33.137	0.992	89.525
ENSMUSG00000043705	Capn13	protein_coding	1.566	81.24	0.481
72.399	1.323	43.586	0.503	84.29
ENSMUSG00000043715	Olfr1326-ps1	protein_coding	1	71.36	1
97.155	NA	NA	1	94.654
ENSMUSG00000043716	Rpl7	protein_coding	1.165	0.355	0.839	25.341
1.021	84.071	1.012	93.156
ENSMUSG00000043719	Col6a6	protein_coding	1.036	76.827
0.942	91.532	1.023	79.897	0.772	92.561
ENSMUSG00000043733	Ptpn11	protein_coding	0.989	81.465
1.021	96.68	0.985	81.525	1.062	49.514
ENSMUSG00000043740	B430306N03Rik	protein_coding	1.692	4.052
0.748	62.692	0.79	57.091	1.551	31.133
ENSMUSG00000043747	1520401A03Rik	processed_transcript	0.416
4.351	1.16	95.708	2.076	10.615	1.033	96.287
ENSMUSG00000043773	1700048O20Rik	protein_coding	1.127	76.827
0.879	84.017	1.005	70.9	1.088	94.825
ENSMUSG00000043782	Ccdc64b	protein_coding	0.94	59.674
0.855	57.613	1.245	19.609	1.094	92.31
ENSMUSG00000043787	Defb12	protein_coding	1	86.918	1
96.847	1	87.274	1	96.183
ENSMUSG00000043789	Vwce	protein_coding	1.2	31.732	NA	NA
0.597	43.426	0.793	86.991
ENSMUSG00000043794	D830025C05Rik	pseudogene	0.76	12.309
0.973	91.532	1.351	17.662	0.908	84.29
ENSMUSG00000043795	Gm14492	protein_coding	0.824	57.227
1.077	95.431	1.124	84.308	1.188	70.539
ENSMUSG00000043800	Kifc5c-ps	pseudogene	1	81.01	1	95.504
NA	NA	1	93.457
ENSMUSG00000043801	Oaz1-ps	pseudogene	1	49.749	NA	NA
1	80.634	1	95.877
ENSMUSG00000043807	Ly6g5b	protein_coding	0.975	69.24	1.053
97.155	0.973	85.921	0.952	95.74
ENSMUSG00000043811	Rtn4r	protein_coding	0.929	85.092	1.315
82.904	0.82	44.211	1.067	92.381
ENSMUSG00000043822	Adamtsl5	protein_coding	1.058	49.202
1.083	90.29	0.87	37.103	1.081	71.855
ENSMUSG00000043827	Olfr94	protein_coding	1	81.125	NA
NA	1	86.727	1	95.273
ENSMUSG00000043831	Lysmd4	protein_coding	0.982	85.832
0.994	96.138	1.026	82.161	0.911	85.493
ENSMUSG00000043832	Clec4a3	protein_coding	1.888	0	0.757
55.71	0.699	10.602	1.147	93.132
ENSMUSG00000043833	2900005J15Rik	processed_transcript	0.962
72.743	0.922	83.876	1.123	19.452	1.023	93.734
ENSMUSG00000043843	Tmem145	protein_coding	1.728	64.401
0.895	96.013	0.647	73.598	0.585	85.493
ENSMUSG00000043850	Clrn1	protein_coding	0.683	57.227	0.905
95.211	1.612	45.449	0.755	96.139
ENSMUSG00000043855	Olfr479	protein_coding	1	78.224	1
95.735	1	88.349	1	96.071
ENSMUSG00000043857	Mgat5b	protein_coding	1.54	64.401

1.389 96.843 0.466 50.184 0.926 81.061
ENSMUSG00000043858 Nup62 protein_coding 1.013 56.085 1.013
85.756 0.975 18.336 1.062 83.067
ENSMUSG00000043865 NA NA NA NA 1 96.216 1
88.076 1 95.777
ENSMUSG00000043866 Taf10 protein_coding 0.819 0.987 1.097 83.104
1.113 8.209 0.964 91.625
ENSMUSG00000043867 Foxl1 pseudogene 1 77.615 1 96.001
1 84.308 1 95.877
ENSMUSG00000043870 Gm5809 pseudogene 1 72.743 1
95.211 1 88.601 1 96.248
ENSMUSG00000043872 Zmym1 protein_coding 1.077 21.836 0.94
87.998 0.989 52.213 1.094 88.755
ENSMUSG00000043873 Chi3l7 protein_coding 1.594 27.731 0.68
62.692 0.926 88.203 0.551 83.724
ENSMUSG00000043880 Olfr323 protein_coding 1 59.028 1
96.984 1 87.686 1 96.192
ENSMUSG00000043881 Kbtbd7 protein_coding 0.991 80.688
1.057 92.111 0.956 47.12 1.004 93.612
ENSMUSG00000043885 Slc36a4 protein_coding 1.076 2.569 0.963
72.399 0.966 53.224 0.946 85.493
ENSMUSG00000043889 Gm8399 pseudogene 1 61.725 1
97.155 1 58.775 1 94.904
ENSMUSG00000043892 Olfr1018 protein_coding 1 83.169 NA
NA 1 86.258 1 96.183
ENSMUSG00000043895 S1pr2 protein_coding 1.197 5.481 1.012 97.049
0.828 2.563 0.962 96.086
ENSMUSG00000043897 Vmn2r2 protein_coding 1 84.452 1
96.216 1 85.935 1 94.654
ENSMUSG00000043903 Gm22 protein_coding 1.29 15.85 1.042 93.146
0.74 6.524 1.273 58.333
ENSMUSG00000043909 Trp53bp1 protein_coding 0.953 35.646
0.919 87.322 1.143 33.042 0.964 88.387
ENSMUSG00000043911 Olfr922 protein_coding 1 76.396 1
96.216 NA NA 1 92.564
ENSMUSG00000043913 Ccdc60 protein_coding 0.92 73.585
0.641 82.947 1.699 37.741 0.779 88.505
ENSMUSG00000043916 D630028G08Rik pseudogene 1 84.452 NA
NA 1 88.729 NA NA
ENSMUSG00000043923 Ccdc84 protein_coding 1.003 83.333
0.989 93.654 1.005 76.225 0.975 93.156
ENSMUSG00000043924 Ncmap protein_coding 0.891 13.952 NA NA
1.224 25.564 1.028 95.559
ENSMUSG00000043925 Olfr544 protein_coding 1 86.351 1
90.378 1 66.269 1 96.358
ENSMUSG00000043929 Klhl15 protein_coding 1.099 10.464
1.001 80.463 0.909 38.434 0.949 83.724
ENSMUSG00000043931 Gimap7 protein_coding 0.756 65.199
1.801 62.898 0.736 15.63 0.889 86.699
ENSMUSG00000043932 Klri2 protein_coding 1.114 82.092 1.662
60.309 0.541 9.563 0.623 83.724
ENSMUSG00000043939 A530064D06Rik protein_coding 1.746 2.651 0.59
61.883 0.967 81.388 1.122 91.928
ENSMUSG00000043940 Wdfy3 protein_coding 0.988 78.538 0.954

72.712 1.058 38.038 1.008 92.31
ENSMUSG00000043943 Naalad2 protein_coding 1.194 7.65 0.909 93.469 0.916 8.192 1.031 90.397
ENSMUSG00000043945 Adam6a protein_coding 1 77.763 NA NA 1 81.29 1 95.877
ENSMUSG00000043948 Olfr691 protein_coding 1 70.862 1 85.378 1 84.961 1 94.654
ENSMUSG00000043953 Ccrl2 protein_coding 0.802 10.704 1.084 96.268 1.149 24.375 1.031 95.273
ENSMUSG00000043962 Thrap3 protein_coding 1.016 72.27 0.949 37.376 1.032 26.498 0.987 96.086
ENSMUSG00000043964 Orai3 protein_coding 1.078 14.194 1.02 83.104 0.906 7.399 0.961 85.493
ENSMUSG00000043969 Emx2 protein_coding 0.972 33.002 0.898 91.532 1.142 34.807 1.031 54.265
ENSMUSG00000043972 Opn5 protein_coding 1 73.487 1 94.597 1 71.232 1 96.139
ENSMUSG00000043982 Krtap19-4 protein_coding 1 78.299 1 91.661 1 78.045 1 96.192
ENSMUSG00000043987 Cep164 protein_coding 0.876 1.6 1.038 72.546 1.101 25.93 1.008 94.904
ENSMUSG00000043991 Pura protein_coding 1.066 14.59 1.024 91.584 0.913 6.524 0.958 85.493
ENSMUSG00000043993 2900052L18Rik processed_transcript 0.665 3.325 1.07 97.288 1.407 36.578 0.971 93.045
ENSMUSG00000043998 Mgat2 protein_coding 1.103 12.985 1.013 90.29 0.889 6.858 0.972 92.263
ENSMUSG00000043999 Gpr75 protein_coding 0.832 39.712 1.168 83.104 1.028 88.693 1.123 89.052
ENSMUSG00000044005 Gls2 protein_coding 1.015 83.968 1.003 96.652 0.982 86.258 1.146 63.018
ENSMUSG00000044006 Cilp2 protein_coding 1.112 82.982 0.77 85.95 1.17 72.148 0.724 87.651
ENSMUSG00000044014 Npy5r protein_coding 1 69.24 1 93.899 1 88.657 NA NA
ENSMUSG00000044017 Gpr133 protein_coding 1.202 6.958 0.909 90.576 0.917 39.245 1.262 40.024
ENSMUSG00000044018 Mrpl50 protein_coding 1.03 84.865 1.005 96.268 0.965 86.602 1.004 84.499
ENSMUSG00000044021 Muc19 protein_coding 0.798 81.657 1.086 96.763 1.157 83.397 0.537 83.952
ENSMUSG00000044022 Pcdhb21 protein_coding 0.87 39.712 1.185 71.617 0.972 81.62 1.119 85.786
ENSMUSG00000044024 Rell2 protein_coding 0.818 29.725 0.993 96.138 1.232 30.292 0.932 92.181
ENSMUSG00000044025 Olfr790 protein_coding 1 82.231 1 96.843 1 87.744 1 96.183
ENSMUSG00000044026 Slc35g1 protein_coding 0.961 70.597 1.085 91.584 0.957 80.749 1.039 94.904
ENSMUSG00000044029 Olfr178 protein_coding 1 86.006 1 88.705 1 82.161 1 96.184
ENSMUSG00000044030 Irf2bp1 protein_coding 1.04 53.407 0.995 91.065 0.969 85.134 0.874 83.724
ENSMUSG00000044033 Ccdc141 protein_coding 0.933 44.922

1.049 88.91 1.023 78.155 0.929 85.493
ENSMUSG00000044034 Npb protein_coding 0.788 36.823 NA NA
1.312 40.955 0.647 83.724
ENSMUSG00000044037 Als2cl protein_coding 0.75 0.987 0.952
79.894 1.396 5.864 0.948 85.493
ENSMUSG00000044039 Olfr1288 protein_coding 1 74.958 1
95.35 NA NA 1 94.384
ENSMUSG00000044040 Olfr1497 protein_coding 1 85.203 1
94.597 1 86.985 1 95.676
ENSMUSG00000044041 Krt13 protein_coding 1 83.333 1
96.68 1 84.724 1 96.183
ENSMUSG00000044042 Fmn1 protein_coding 1.046 62.689 0.931
84.017 1.027 75.155 1.006 96.287
ENSMUSG00000044043 Pcdhb14 protein_coding 0.912 42.922
1.153 72.642 0.954 36.248 1.006 92.52
ENSMUSG00000044052 Ccr10 protein_coding 0.683 26.217 1.302
85.756 1.125 51.243 0.993 88.387
ENSMUSG00000044055 Otos protein_coding 0.902 76.396 NA NA
0.987 83.538 0.897 94.904
ENSMUSG00000044056 Efcab9 protein_coding 0.98 67.163
1.168 92.931 0.874 39.528 0.904 91.625
ENSMUSG00000044058 Mthfr-ps1 pseudogene 1 84.865 1
96.138 NA NA 1 96.287
ENSMUSG00000044060 A830010M20Rik protein_coding 1.159 24.689
1.006 96.216 0.858 32.297 0.863 88.505
ENSMUSG00000044061 Olfr225 protein_coding 1 81.196 1
94.48 1 88.41 1 94.567
ENSMUSG00000044062 0610009B14Rik antisense 0.594 61.633
1.084 96.68 1.558 77.17 1.035 95.35
ENSMUSG00000044065 Gm9788 pseudogene 1 59.211 1
95.708 1 83.719 1 94.825
ENSMUSG00000044066 Cep68 protein_coding 1.147 0.2 0.982 96.013
0.889 0.879 0.939 83.724
ENSMUSG00000044067 Gpr22 protein_coding 1 83.333 1
96.138 1 85.352 1 96.358
ENSMUSG00000044068 Zrsr1 protein_coding 0.976 71.674 1.006
92.914 1.019 54.363 0.926 84.29
ENSMUSG00000044072 Eml6 protein_coding 1.02 64.401 0.928
88.705 1.055 88.693 1.056 92.018
ENSMUSG00000044080 S100a1 protein_coding 0.881 3.009 1.112
82.904 1.024 41.806 0.955 91.217
ENSMUSG00000044081 4930441O14Rik antisense 0.937 84.534
1.038 95.088 1.029 80.634 1.034 93.734
ENSMUSG00000044083 Efcab8 protein_coding 0.606 7.881 1.684
72.642 0.982 87.744 1.214 78.042
ENSMUSG00000044086 Lmod3 protein_coding 2.794 18.388 7.311
71.98 0.049 0.72 0.933 95.74
ENSMUSG00000044092 C130050O18Rik protein_coding 1.161 39.416
0.927 95.708 0.926 40.794 1.048 93.132
ENSMUSG00000044095 Ccdc166 pseudogene 1 81.465 1
91.83 NA NA 1 95.676
ENSMUSG00000044098 Rsbn1 protein_coding 1.049 65.537 0.946
89.996 NA NA 0.949 83.724
ENSMUSG00000044103 Il1f9 protein_coding 1.086 84.168 1.183

96.378	0.78	79.524	2.823	40.894
ENSMUSG00000044106	Olfr868	protein_coding	1	84.263	1
94.102	1	88.693	1	94.384
ENSMUSG00000044107	Olfr964-ps1	pseudogene	1	82.732	NA	NA
1	80.5	1	95.877
ENSMUSG00000044117	2900011O08Rik	protein_coding	1.143	63.804
1.147	93.574	0.763	30.827	0.919	96.071
ENSMUSG00000044120	Olfr683	protein_coding	1	72.655	1
92.111	NA	NA	1	96.287
ENSMUSG00000044121	5430402E10Rik	protein_coding	1	81.823
1	95.211	1	79.554	1	95.74
ENSMUSG00000044122	Proca1	protein_coding	0.944	19.224
0.946	95.211	1.115	49.898	0.964	91.078
ENSMUSG00000044125	9530080O11Rik	processed_transcript	0.768
64.014	NA	NA	1.54	69.196	1.131	93.316
ENSMUSG00000044134	Fam109a	protein_coding	0.939	38.286
0.971	74.483	1.098	16.26	0.917	84.29
ENSMUSG00000044139	Prss53	protein_coding	0.713	27.968
1.582	82.904	0.884	85.464	1.207	71.855
ENSMUSG00000044141	E130201H02Rik	pseudogene	1	82.982	1
75.993	1	58.051	1	92.12
ENSMUSG00000044145	1810024B03Rik	protein_coding	1.045	68.16
0.892	90.053	1.071	86.144	1.031	93.964
ENSMUSG00000044147	Arf6	protein_coding	1.106	61.725	0.97
95.431	0.933	48.577	0.917	84.19
ENSMUSG00000044148	1810030O07Rik	protein_coding	1.079	2.233
1.037	85.756	0.89	0.72	1.021	87.7
ENSMUSG00000044149	Nkrf	protein_coding	1.096	19.315	0.924
90.744	0.986	65.902	1.025	82.66
ENSMUSG00000044150	A830080D01Rik	protein_coding	1.051	31.24
0.982	79.475	0.965	83.158	1.067	89.108
ENSMUSG00000044155	Naa38	protein_coding	1.097	33.325	0.959
78.877	0.953	87.336	1.1	46.412
ENSMUSG00000044156	Hepacam2	protein_coding	1.705	4.228	0.627
56.781	0.936	69.445	0.988	89.892
ENSMUSG00000044162	Tnip3	protein_coding	1.2	51.189	0.7
63.773	1.187	70.328	1.45	76.831
ENSMUSG00000044164	Rnf182	protein_coding	1.021	77.497	1.18
90.389	0.827	62.533	1.511	50.452
ENSMUSG00000044165	Bcl2l15	protein_coding	1.15	83.523
0.703	92.545	1.235	77.17	1.035	90.674
ENSMUSG00000044167	Foxo1	protein_coding	1.204	28.927	0.887
60.678	0.937	79.524	1.126	96.192
ENSMUSG00000044170	Olfr1384	protein_coding	1	85.092	1
93.707	1	87.898	1	94.82
ENSMUSG00000044172	Ptx4	protein_coding	1	80.03	1	92.931
1	46.575	1	96.287
ENSMUSG00000044177	Wfikkn2	protein_coding	1.209	22.025
0.877	82.503	0.939	59.125	0.747	83.724
ENSMUSG00000044186	Nkx2-6	protein_coding	1	82.891	NA
NA	1	84.626	1	96.183
ENSMUSG00000044197	Gpr146	protein_coding	1.074	43.344
1.145	88.551	0.813	3.234	0.979	92.181
ENSMUSG00000044199	S1pr4	protein_coding	0.958	42.922	1.077

96.308	0.97	78.155	0.892	86.991
ENSMUSG00000044201	Cdc25c	protein_coding	2.68	10.464
0.537	33.301	0.694	86.985	1.513	78.379
ENSMUSG00000044205	Olfr983	protein_coding	1	86.843	1
96.268	1	79.554	1	95.273
ENSMUSG00000044206	Vsig4	protein_coding	2.966	0	0.671	62.692
0.502	3.718	1.403	76.467
ENSMUSG00000044213	Olfr1094	protein_coding	1	81.823	1
96.013	1	86.258	1	94.82
ENSMUSG00000044216	Kcnj4	protein_coding	0.167	36.823	1.084
93.574	5.549	56.708	4.417	81.251
ENSMUSG00000044217	Aqp5	protein_coding	0.988	75.196	1.11
85.501	0.912	54.037	1.281	65.175
ENSMUSG00000044220	Nkx2-3	protein_coding	1	80.408	1
96.68	1	83.857	1	93.413
ENSMUSG00000044221	Grsf1	protein_coding	1.076	15.85	0.992	95.211
0.939	41.928	1.039	78.042
ENSMUSG00000044224	Dnajc21	protein_coding	0.968	49.368
0.989	93.654	1.045	36.839	1.067	61.984
ENSMUSG00000044227	Gm9789	protein_coding	1	63.534	1
96.843	NA	NA	1	96.071
ENSMUSG00000044228	Olfr1010	pseudogene	1	85.728	1
91.661	1	80.089	1	94.384
ENSMUSG00000044229	Nxpe4	protein_coding	1.068	77.497	1.189
79.055	0.787	11.504	0.932	84.29
ENSMUSG00000044231	Nhlrc1	protein_coding	1.1	31.477
0.966	84.017	0.941	81.525	0.929	86.445
ENSMUSG00000044244	Il20rb	protein_coding	0.861	25.551
0.921	61.883	1.263	7.365	1.134	53.923
ENSMUSG00000044248	Vmn1r45	protein_coding	1	74.745	NA
NA	NA	NA	1	96.248
ENSMUSG00000044250	Pced1b	protein_coding	1.027	64.956
0.962	91.532	1.014	86.084	0.964	84.29
ENSMUSG00000044252	Osbpl1a	protein_coding	0.896	33.599
1.027	94.262	1.081	28.603	0.967	91.625
ENSMUSG00000044254	Pcsk9	protein_coding	0.804	83.09	1.232	96.763
1.009	87.431	2.18	54.153
ENSMUSG00000044258	Ctla2a	protein_coding	0.91	19.023
0.996	95.504	1.104	47.512	0.942	93.964
ENSMUSG00000044265	E030002O03Rik	protein_coding	1	85.59	1
95.708	1	87.37	1	90.125
ENSMUSG00000044268	Gm4895	pseudogene	1	70.862	1
92.561	1	81.492	1	96.183
ENSMUSG00000044276	4933427E11Rik	protein_coding	0.712	47.266
1.073	89.494	1.303	64.024	1.432	89.152
ENSMUSG00000044279	Crb3	protein_coding	0.963	74.8	0.935	94.597
1.109	54.809	0.927	92.12
ENSMUSG00000044285	Gm1821	pseudogene	0.82	10.29	0.897	81.134
1.358	10.819	1.067	74.193
ENSMUSG00000044286	Olfr221	protein_coding	1	86.464	1
97.155	NA	NA	1	96.248
ENSMUSG00000044287	Nrn1l	protein_coding	1	79.433	1
94.848	1	86.112	1	94.904
ENSMUSG00000044288	Cnr1	protein_coding	1.382	30.984	1.096

86.34 0.662 9.901 0.591 83.724
ENSMUSG00000044292 Olfr978 protein_coding 1 80.815 1
96.216 1 81.032 1 91.525
ENSMUSG00000044293 Olfr794 protein_coding 1 86.658 1
95.708 1 86.831 1 96.248
ENSMUSG00000044294 Krt84 protein_coding 1 86.74 1 95.35 1
87.37 1 93.156
ENSMUSG00000044296 Zfp879 protein_coding 1.015 85.092
0.927 81.134 1.064 57.286 1.147 95.559
ENSMUSG00000044303 Cdkn2a protein_coding 2.038 15.972 0.6
42.923 0.819 76.388 1.149 94.016
ENSMUSG00000044306 4930500M09Rik protein_coding 0.847 51.56
0.795 91.361 1.485 40.511 0.897 84.29
ENSMUSG00000044308 Ubr3 protein_coding 0.96 53.138 NA NA
1.061 44.515 1.016 87.447
ENSMUSG00000044313 Mab21l3 protein_coding 0.644 33.002 1.12
96.68 1.378 51.977 0.92 94.654
ENSMUSG00000044317 Gpr4 protein_coding 0.831 0.987 1.124 78.629
1.07 35.441 0.845 83.724
ENSMUSG00000044320 1700001O22Rik protein_coding 1.153 67.608
0.799 64.123 1.087 47.31 0.787 90.611
ENSMUSG00000044322 Dsc1 protein_coding 1 82.543 1
92.404 1 85.464 1 93.316
ENSMUSG00000044328 Trp53i13 protein_coding 0.896 3.641 1.056
95.211 1.056 18.208 0.924 83.724
ENSMUSG00000044337 Ackr3 protein_coding 1.045 39.295 1.129
96.843 0.847 32.963 1.229 69.404
ENSMUSG00000044338 Aplnr protein_coding 1.171 33.854 1.334
61.943 0.64 0.72 0.949 90.854
ENSMUSG00000044339 Alkbh2 protein_coding 1.096 44.061
0.954 74.483 0.959 81.62 0.871 85.493
ENSMUSG00000044340 Phlpp1 protein_coding 1.028 86.112
0.995 95.941 0.976 86.475 0.901 83.724
ENSMUSG00000044341 Gm5601 pseudogene 1.469 42.255 1.085
97.155 0.629 27.321 0.944 94.99
ENSMUSG00000044345 Marveld1 protein_coding 0.954 65.622
1.022 85.756 1.027 84.896 1.079 83.067
ENSMUSG00000044348 Slc25a53 protein_coding 0.911 22.731
0.917 83.392 1.199 11.932 0.984 96.358
ENSMUSG00000044349 Snhg11 protein_coding 0.948 69.24 0.924
51.159 1.139 26.663 0.97 94.904
ENSMUSG00000044350 Lacc1 protein_coding 1.255 5.932 0.946 85.075
0.843 11.751 1.061 78.379
ENSMUSG00000044352 Sowaha protein_coding 0.789 52.9 1.544
72.642 0.823 62.533 1.009 94.215
ENSMUSG00000044361 BC024139 protein_coding 0.839 47.174
1.132 82.904 1.054 81.388 0.968 90.455
ENSMUSG00000044362 Ccdc89 protein_coding 0.98 85.857
0.679 72.399 1.498 25.455 0.926 90.611
ENSMUSG00000044364 Tmem74b protein_coding 1.365 41.211
0.867 78.51 0.846 66.647 NA NA
ENSMUSG00000044365 Cxxc4 protein_coding 1.15 84.564 0.782
76.978 1.108 49.058 0.874 90.289
ENSMUSG00000044367 Slc16a13 protein_coding 0.937 86.918

1.061 91.643 1.001 84.669 0.932 83.952
ENSMUSG00000044377 Gm13080 pseudogene 0.789 55.641 1.672
83.104 0.758 77.037 1.182 87.134
ENSMUSG00000044378 Slc15a5 protein_coding 0.077 0.987 1.566
79.055 8.25 7.365 1.032 92.742
ENSMUSG00000044387 2410080I02Rik lincRNA 1.069 86.819
0.661 80.052 1.415 51.243 0.883 96.287
ENSMUSG00000044390 Tigd3 protein_coding 0.85 75.567 1.018
87.452 1.157 71.839 1.127 94.904
ENSMUSG00000044393 Dsg2 protein_coding 1.174 25.944 0.843
67.165 1.013 86.985 0.786 84.29
ENSMUSG00000044400 NA NA NA NA 1 96.984 1
79.554 1 94.904
ENSMUSG00000044405 Adig protein_coding 0.955 70.213 1.119
72.642 0.932 22.735 0.956 95.832
ENSMUSG00000044407 Qk protein_coding 0.778 62.437 1.107
96.843 1.163 71.615 0.776 83.724
ENSMUSG00000044408 Sptssa protein_coding 1.066 41.177
1.066 65.165 0.882 2.563 0.97 90.95
ENSMUSG00000044424 Gm9493 protein_coding 1 84.263 1
96.013 1 63.801 1 95.273
ENSMUSG00000044429 Cryga protein_coding 1 85.59 NA NA 1
86.666 1 94.99
ENSMUSG00000044430 Klk12 protein_coding 1 59.674 1
80.14 1 71.004 1 86.991
ENSMUSG00000044433 Camsap3 protein_coding 0.94 51.447
1.097 92.561 0.967 81.525 0.999 85.493
ENSMUSG00000044441 Olfr1442 protein_coding 1 79.93 1
95.504 1 81.169 1 95.877
ENSMUSG00000044442 N6amt1 protein_coding 1.023 81.196
0.938 87.998 1.045 58.414 1.014 90.125
ENSMUSG00000044444 Pfn3 protein_coding 1 86.658 1
94.102 1 83.158 1 93.584
ENSMUSG00000044447 Dock5 protein_coding 1.003 86.006 0.977
91.361 1.02 73.309 0.965 89.827
ENSMUSG00000044452 Zfp507 protein_coding 1.01 54.344
0.911 51.322 1.082 54.363 1.058 86.118
ENSMUSG00000044454 Olfr867 protein_coding 1 79.277 NA
NA 1 86.112 1 96.358
ENSMUSG00000044456 Rin3 protein_coding 0.941 55.641 0.966
81.443 1.094 33.787 1.047 76.697
ENSMUSG00000044457 Gm4776 protein_coding 1 36.438 1
85.671 1 79.625 1 92.12
ENSMUSG00000044461 Shisa2 protein_coding 0.918 49.621
0.959 93.908 1.137 55.512 1.166 96.192
ENSMUSG00000044465 Fam160a2 protein_coding 0.952 23.641
0.972 86.861 1.08 23.604 0.991 90.854
ENSMUSG00000044468 Fam46c protein_coding 1.265 4.675 1.059
97.263 0.747 6.68 0.942 88.053
ENSMUSG00000044469 Tnfaip8l1 protein_coding 0.809 2.009 0.98
96.68 1.263 9.176 0.927 88.245
ENSMUSG00000044471 AB041803 protein_coding 1.117 85.946
0.782 87.998 1.14 57.637 1.051 83.067
ENSMUSG00000044475 Ascc1 protein_coding 0.934 48.146 0.97

70.29 1.101 20.315 1.01 94.904
ENSMUSG00000044477 Zfand3 protein_coding 0.978 53.407
1.024 85.378 NA NA 0.948 95.405
ENSMUSG00000044480 4930578I07Rik protein_coding 1 71.516
1 96.407 1 86.258 1 95.676
ENSMUSG00000044487 Olfr1112 protein_coding 1 84.977 NA
NA 1 87.37 1 96.086
ENSMUSG00000044496 2510039O18Rik protein_coding 1.007 71.915
1.034 95.735 0.963 87.336 0.921 91.291
ENSMUSG00000044499 Hs3st5 protein_coding 4.048 14.966
0.895 94.848 0.276 25.625 1.156 92.381
ENSMUSG00000044501 Zfp758 protein_coding 1.281 1.545 0.988
93.817 0.791 11.997 0.887 84.29
ENSMUSG00000044502 Bod1 protein_coding 0.971 37.82 0.973 87.998
1.057 31.317 1.024 83.581
ENSMUSG00000044505 Lingo4 protein_coding 1.114 82.352
0.901 79.807 0.996 59.274 1.036 92.748
ENSMUSG00000044515 Gm8385 pseudogene 1 85.728 NA NA
1 80.163 1 94.19
ENSMUSG00000044518 Foxe3 protein_coding 1 86.333 1
93.908 1 87.431 1 94.904
ENSMUSG00000044519 Zfp488 protein_coding 0.947 24.577
0.945 72.399 1.116 12.831 0.87 83.724
ENSMUSG00000044522 A730020M07Rik protein_coding 0.65 24
1.071 92.931 1.437 51.596 1.578 69.708
ENSMUSG00000044526 Znrf4 protein_coding 1 74.8 1 92.253
1 86.112 1 96.358
ENSMUSG00000044528 Tram1l1 protein_coding 1.087 81.52 NA NA
0.993 86.666 1.031 91.625
ENSMUSG00000044533 Rps2 protein_coding 0.985 86.006 1.019
97.155 0.998 88.729 1.075 64.513
ENSMUSG00000044534 Ackr2 protein_coding 0.909 65.106 0.947
73.166 1.163 24.691 1.023 92.762
ENSMUSG00000044537 Olfr779 protein_coding 1 80.408 1
96.843 1 85.757 1 93.156
ENSMUSG00000044538 Gm4830 pseudogene 1 81.76 1 93.908
1 79.625 1 93.734
ENSMUSG00000044542 Prop1 protein_coding 1 81.24 1 96.407
NA NA 1 89.428
ENSMUSG00000044548 Dact1 protein_coding 0.776 8.365 0.928 94.262
1.39 20.886 1.023 86.254
ENSMUSG00000044550 Tceal3 protein_coding 1.116 76.042
1.186 94.367 0.756 65.688 0.869 92.181
ENSMUSG00000044551 9930012K11Rik protein_coding 1.181 64.401
0.737 65.165 1.146 43.586 1.302 44.679
ENSMUSG00000044556 Tex38 protein_coding 1.591 29.057 0.986
95.809 0.635 29.217 0.632 91.078
ENSMUSG00000044560 Olfr1302 protein_coding 1 80.688 1
96.138 1 70.623 1 95.72
ENSMUSG00000044562 Rasip1 protein_coding 0.858 4.115 1.114
92.793 1.043 25.808 0.948 85.493
ENSMUSG00000044566 Cage1 protein_coding 1.126 40.333 0.833
74.851 1.064 76.754 0.884 91.65
ENSMUSG00000044573 Acp1 protein_coding 1.033 29.811 0.999

87.857 0.971 69.583 1.059 60.31
ENSMUSG00000044574 5031434C07Rik protein_coding 1 81.925
1 94.597 NA NA 1 70.624
ENSMUSG00000044576 Gareml protein_coding 0.486 72.743
1.415 96.407 1.455 84.626 1.436 96.192
ENSMUSG00000044581 4932415D10Rik protein_coding 1 77.916
1 89.996 1 83.538 1 96.192
ENSMUSG00000044583 Tlr7 protein_coding 1.52 0 0.797 21.315
0.826 41.662 1.178 70.36
ENSMUSG00000044594 Serpinb3a protein_coding 1 84.865 1
97.263 1 84.724 1 94.608
ENSMUSG00000044595 Dnd1 protein_coding 0.812 28.242 1.089
85.501 1.129 85.757 1.097 83.067
ENSMUSG00000044597 Mycs protein_coding 1 79.277 NA NA
1 88.729 1 94.215
ENSMUSG00000044600 Smim7 protein_coding 1.095 7.255 1.011 82.904
0.904 0.72 0.973 88.505
ENSMUSG00000044609 Gm9294 pseudogene 1 86.464 1
95.504 1 88.693 1 92.018
ENSMUSG00000044617 Zbtb39 protein_coding 0.989 78.909
1.002 95.735 NA NA 0.988 96.287
ENSMUSG00000044624 Gm4922 protein_coding 1 64.714 1
97.049 1 88.161 1 93.627
ENSMUSG00000044626 Liph protein_coding 1.05 83.968 0.838
88.881 1.138 72.378 0.786 83.724
ENSMUSG00000044627 Swi5 protein_coding 0.993 62.437 1.033
90.454 0.971 84.83 1.02 91.525
ENSMUSG00000044628 Rnf208 protein_coding 0.798 17.276
1.026 96.847 1.223 25.144 0.905 86.991
ENSMUSG00000044629 Cnrip1 protein_coding 1.281 4.916 0.822
54.245 0.951 40.794 1.104 33.269
ENSMUSG00000044633 B530045E10Rik protein_coding 1 66.652
1 96.897 1 84.724 1 91.525
ENSMUSG00000044636 Csrnp2 protein_coding 0.938 66.406
1.029 95.708 1.037 69.315 1.038 94.904
ENSMUSG00000044639 Gm11507 pseudogene 1 68.485 1
93.654 1 79.119 1 92.018
ENSMUSG00000044641 Pard6b protein_coding 0.843 48.354
1.058 95.431 1.123 71.232 0.944 87.705
ENSMUSG00000044646 Zbtb7c protein_coding 1.092 45.307
1.013 92.561 0.902 15.376 1.16 55.841
ENSMUSG00000044647 Csrnp3 protein_coding 1.044 63.126
0.778 87.322 1.233 79.325 0.723 83.724
ENSMUSG00000044649 Krtap4-2 protein_coding 1 85.355 1
94.848 1 87.898 1 96.192
ENSMUSG00000044667 D3Bwg0562e protein_coding 1.377 33.099
0.916 95.504 0.791 42.997 1.073 79.268
ENSMUSG00000044674 Fzd1 protein_coding 0.977 77.407 1.084
93.994 0.941 85.675 1.049 83.276
ENSMUSG00000044676 Zfp612 protein_coding 0.895 17.062
0.937 88.897 1.19 19.877 0.848 83.724
ENSMUSG00000044678 Ly6k protein_coding 0.467 45.502 0.79
82.503 2.701 24.791 1.318 76.046
ENSMUSG00000044689 Gm13749 lincRNA 1.044 83.523 1.155

93.574 0.827 83.158 1.187 81.901
ENSMUSG00000044694 2010007H06Rik protein_coding 1.127 51.297
0.778 81.134 1.143 69.825 0.938 88.245
ENSMUSG00000044697 Gm5479 pseudogene 1 76.396 1
89.494 1 86.985 1 95.74
ENSMUSG00000044700 Tmem201 protein_coding 1.033 31.24 1.083
86.34 0.893 5.323 0.951 85.493
ENSMUSG00000044701 Il27 protein_coding 1.043 85.355 0.935
95.196 1.023 86.552 1.03 95.676
ENSMUSG00000044702 Palb2 protein_coding 1.227 30.788 0.934
75.493 0.869 69.134 1.021 92.389
ENSMUSG00000044703 Phf11a protein_coding 1.413 0.577 0.907
79.475 0.777 53.224 1.575 52.982
ENSMUSG00000044705 Olfr670 protein_coding 1 75.761 1
96.652 1 83.925 1 93.413
ENSMUSG00000044707 Ccnjl protein_coding 1.393 37.414 1.055
95.708 0.68 45.573 0.76 85.493
ENSMUSG00000044708 Kcnj10 protein_coding 1.087 66 1.843
64.304 0.5 4.935 0.718 87.182
ENSMUSG00000044709 Gemin7 protein_coding 0.941 36.541
1.064 71.617 1 77.704 0.962 86.991
ENSMUSG00000044712 Slc38a6 protein_coding 1.181 0 0.949
76.978 0.893 23.86 0.952 84.29
ENSMUSG00000044715 Gskip protein_coding 1.071 44.061 0.969
95.504 0.961 55.9 1.002 92.903
ENSMUSG00000044716 Dok7 protein_coding 0.98 82.732 0.986 75.4
1.032 69.002 1.034 78.546
ENSMUSG00000044719 E230025N22Rik protein_coding 1.098 66.944
0.811 81.443 1.126 70.9 0.748 83.724
ENSMUSG00000044724 Gpr152 protein_coding 0.658 11.519
1.215 82.904 1.253 63.665 0.905 96.184
ENSMUSG00000044726 BC030476 protein_coding 1 83.658 1
87.919 1 87.744 1 91.807
ENSMUSG00000044730 9930104L06Rik processed_transcript 0.955
38.286 1.042 95.088 1.007 86.666 1.034 87.447
ENSMUSG00000044734 Serpinb1a protein_coding 1.586 0 0.877
92.914 0.717 0.879 1.271 40.024
ENSMUSG00000044737 Klk14 protein_coding 1.064 68.727 1.088
96.138 0.863 78.713 0.43 83.724
ENSMUSG00000044743 Defb10 protein_coding 1 81.657 1
96.138 1 67.163 1 95.92
ENSMUSG00000044744 1700001G17Rik protein_coding 0.982 72
0.746 79.807 1.361 67.823 0.843 86.991
ENSMUSG00000044748 Defb1 protein_coding 0.464 41.617 NA NA
1.905 45.208 1.514 93.156
ENSMUSG00000044749 Abca6 protein_coding 1.002 84.819 0.758
61.883 1.316 15.411 0.941 86.699
ENSMUSG00000044751 Gm12231 pseudogene 1 81.01 1 96.138
1 86.831 1 94.825
ENSMUSG00000044757 Gm6430 protein_coding 1 69.943 1
95.504 1 87.173 1 93.316
ENSMUSG00000044763 Trmt10c protein_coding 1.173 6.61 0.947
93.654 0.899 15.169 0.95 84.29
ENSMUSG00000044768 D1Ertd622e protein_coding 1.046 50.627 NA

NA 0.908 31.676 1.006 95.35
ENSMUSG00000044770 Scml4 protein_coding 1.083 80.815 0.893
79.475 1.031 43.586 0.978 95.35
ENSMUSG00000044772 NA NA NA NA 1 94.848 NA NA
1 94.904
ENSMUSG00000044783 Hjurp protein_coding 1 56.311 NA NA
0.97 54.037 1.039 96.248
ENSMUSG00000044786 Zfp36 protein_coding 1.103 13.734 0.933
85.671 0.972 42.621 0.919 84.29
ENSMUSG00000044788 Fads6 protein_coding 0.863 42.034 1.027
93.654 1.128 34.807 0.771 83.724
ENSMUSG00000044791 Setd2 protein_coding 0.975 55.641 0.943
69.611 1.086 19.286 0.988 96.139
ENSMUSG00000044792 Isca1 protein_coding 0.998 80.146 1.027
96.843 0.978 84.669 1.045 63.708
ENSMUSG00000044794 9330133O14Rik pseudogene 0.987 56.907 1.03
88.705 0.986 87.744 0.994 85.493
ENSMUSG00000044795 Cyb5d1 protein_coding 0.999 80.146
1.062 71.617 0.943 16.643 0.966 96.287
ENSMUSG00000044798 Olfr923 protein_coding 1 86.74 NA NA
1 79.554 1 95.877
ENSMUSG00000044807 Zfp354c protein_coding 0.953 68.846
0.994 95.708 1.056 82.74 1.066 91.492
ENSMUSG00000044811 AF251705 protein_coding 1.384 3.434 0.816
70.693 0.886 45.573 0.978 95.273
ENSMUSG00000044813 Shb protein_coding 0.961 78.224 1.027
97.155 1.01 84.83 1.045 83.067
ENSMUSG00000044816 D630023F18Rik protein_coding 0.861 76.686
1.652 93.994 0.701 88.076 0.613 93.734
ENSMUSG00000044819 Oxgr1 protein_coding 1 83.333 1
95.088 1 86.666 1 95.72
ENSMUSG00000044820 AY074887 protein_coding 0.636 17.031
0.825 89.277 1.898 8.586 0.867 92.31
ENSMUSG00000044824 Olfr545 protein_coding 1 65.388 1
94.48 1 63.301 1 90.865
ENSMUSG00000044827 Tlr1 protein_coding 1.737 0.461 0.842 96.013
0.68 0.72 1.42 57.1
ENSMUSG00000044847 Lsm11 protein_coding 0.979 69.985 0.989
93.863 1.029 87.686 1.027 80.573
ENSMUSG00000044854 1700056E22Rik protein_coding 1.148 55.789
0.935 87.553 0.933 80.163 0.839 85.947
ENSMUSG00000044857 Lemd2 protein_coding 0.988 48.646 0.972
93.817 1.04 37.741 0.966 93.612
ENSMUSG00000044860 Gm1123 protein_coding 1.728 71.217
0.978 95.329 0.589 87.173 0.585 91.291
ENSMUSG00000044864 Ankrd50 protein_coding 1.136 15.602 1
95.735 0.879 18.336 0.937 84.29
ENSMUSG00000044867 Gm3345 antisense 0.919 82.231 1.226
95.211 0.886 64.427 0.832 84.19
ENSMUSG00000044876 Zfp444 protein_coding 0.898 43.167 0.94
85.418 1.188 26.179 1.024 91.985
ENSMUSG00000044881 Coa4 protein_coding 1.019 62.007 1.088
82.904 0.902 13.551 0.957 96.192
ENSMUSG00000044894 Uqcrq protein_coding 0.655 0.987 1.109 83.104

1.373 6.202 1.137 90.463
ENSMUSG00000044897 Olfr821 protein_coding 1 82.982 1
95.504 NA NA 1 95.92
ENSMUSG00000044899 NA NA NA NA 1 94.597 1
84.83 1 92.742
ENSMUSG00000044903 Psg22 protein_coding 1 73.734 NA NA
1 70.791 1 85.493
ENSMUSG00000044906 4930503L19Rik protein_coding 1.002 56.311
1.08 96.335 0.927 30.827 1.097 68.696
ENSMUSG00000044912 Syt16 protein_coding 1 86.658 1
97.263 NA NA 1 85.629
ENSMUSG00000044916 1700029I15Rik protein_coding 0.598 0.987
1.141 79.411 1.468 5.964 0.985 91.217
ENSMUSG00000044921 Rassf9 protein_coding 1.104 84.294
0.909 90.378 0.998 86.144 0.953 93.612
ENSMUSG00000044923 Olfr1030 protein_coding 1 55.303 1
90.952 1 86.985 1 96.071
ENSMUSG00000044927 H1fx protein_coding 0.807 17.705 1.426
78.629 0.866 71.004 1.195 89.108
ENSMUSG00000044933 Sstr3 protein_coding 0.787 64.284 0.996
96.216 1.27 83.719 1.122 95.832
ENSMUSG00000044934 Zfp367 protein_coding 1.131 21.146
0.945 89.509 0.938 33.971 1.02 86.445
ENSMUSG00000044937 BC030307 protein_coding 0.863 13.062
1.009 97.155 1.152 28.301 0.96 95.74
ENSMUSG00000044938 Klhl31 protein_coding 7.896 9.695 1.949
79.864 0.065 0.72 0.643 95.593
ENSMUSG00000044948 Wdr96 protein_coding 1.309 74.147 0.875
92.561 0.871 46.575 1.166 94.904
ENSMUSG00000044949 Ubtd2 protein_coding 1.134 17.582 1.023
95.329 0.862 31.887 0.991 95.593
ENSMUSG00000044950 Pwwp2a protein_coding 1.039 68.485
0.985 92.631 0.978 83.607 0.937 83.724
ENSMUSG00000044951 Mylk4 protein_coding 5.124 7.492 1.873 85.756
0.104 0.72 0.902 94.904
ENSMUSG00000044952 Kctd21 protein_coding 0.919 34.305
1.179 60.309 0.922 25.512 0.933 86.991
ENSMUSG00000044957 Pp2d1 protein_coding 1.036 82.092 NA NA
1.125 79.119 0.777 96.183
ENSMUSG00000044966 Fbxo48 protein_coding 1.684 24.689
0.934 93.654 0.634 33.137 0.98 94.567
ENSMUSG00000044968 Napepld protein_coding 0.938 52.258
1.007 92.561 1.059 86.475 0.975 86.991
ENSMUSG00000044976 Wdr72 protein_coding 1.023 77.291 0.89
82.073 1.098 72.853 0.974 86.991
ENSMUSG00000044982 Sft2d3 protein_coding 0.884 17.839
0.964 82.503 1.176 19.609 1.045 83.276
ENSMUSG00000044985 Olfr124 protein_coding 1 78.299 NA
NA 1 85.675 1 96.086
ENSMUSG00000044986 Tst protein_coding 1.07 79.068 0.919
94.102 1.018 87.971 0.709 83.724
ENSMUSG00000044988 Ucn3 protein_coding 1 79.277 1
95.708 1 84.434 1 96.248
ENSMUSG00000044991 1110034G24Rik protein_coding 1.061 56.907

1.101 91.643 0.857 21.331 0.911 84.19
ENSMUSG00000044994 Olfr1426 protein_coding 1 85.946 1
97.129 1 88.601 1 95.593
ENSMUSG00000044997 E130304I02Rik protein_coding 1.012 84.661
0.785 80.052 1.254 59.274 0.678 88.505
ENSMUSG00000045005 Fzd5 protein_coding 1.084 50.111 1.011
95.211 0.911 73.598 1.22 83.067
ENSMUSG00000045007 Tubg2 protein_coding 1.001 79.93 0.967 90.046
1.033 66.928 0.937 85.493
ENSMUSG00000045008 9030612E09Rik protein_coding 1.361 33.099
0.703 79.807 1.044 81.169 0.932 85.636
ENSMUSG00000045009 Prrt3 protein_coding 1.426 59.527 NA NA
0.963 67.339 0.607 85.493
ENSMUSG00000045010 Gm4779 antisense 1 71.36 1 97.155
1 88.693 1 91.525
ENSMUSG00000045013 Olfr711 protein_coding 1 84.452 1
93.654 1 80.634 1 95.877
ENSMUSG00000045022 1700024P04Rik protein_coding 1 86.819
1 82.947 1 80.634 1 95.72
ENSMUSG00000045027 Prss22 protein_coding 0.553 10.903
0.826 87.313 2.179 9.567 0.945 91.372
ENSMUSG00000045030 Olfr1443 protein_coding 1 82.092 1
97.155 1 71.004 1 92.561
ENSMUSG00000045031 Cetn4 protein_coding 1.265 0.577 0.886 55.265
0.892 27.194 0.888 83.724
ENSMUSG00000045038 Prkce protein_coding 0.787 17.062 1.018
89.434 1.248 74.686 1.536 42.736
ENSMUSG00000045039 Megf8 protein_coding 0.896 14.221 1.06
90.68 1.053 43.185 1.015 96.248
ENSMUSG00000045045 Lrfn4 protein_coding 0.754 6.34 1.025 94.767
1.296 10.079 0.963 90.289
ENSMUSG00000045048 Gm9795 pseudogene 1 77.182 1
83.321 1 79.119 1 95.877
ENSMUSG00000045052 Prlhr protein_coding 1 80.408 1
83.733 1 86.144 1 89.294
ENSMUSG00000045055 Rpsa-ps2 pseudogene 1 67.334 1
96.984 1 74.082 1 92.018
ENSMUSG00000045062 Pcdhb7 protein_coding 1.106 39.075
1.201 79.29 0.754 8.634 0.776 83.724
ENSMUSG00000045064 Zc2hc1c protein_coding 1.016 74.482
1.127 92.111 0.876 41.662 0.929 86.991
ENSMUSG00000045065 9930022D16Rik protein_coding 1 83.169
1 93.654 1 81.976 1 82.328
ENSMUSG00000045071 E130308A19Rik protein_coding 0.801 1.965 0.96
94.597 1.303 5.864 0.967 86.699
ENSMUSG00000045075 Gm9796 protein_coding 0.84 71.674
1.051 96.948 1.134 69.315 1.118 88.797
ENSMUSG00000045078 Rnf216 protein_coding 1.086 8.685 0.979
91.275 0.94 15.024 0.989 96.183
ENSMUSG00000045087 S1pr5 protein_coding 1.041 62.354 1.327
85.756 0.72 35.815 1.038 90.463
ENSMUSG00000045091 Aqp12 protein_coding 1 83.169 NA NA
1 81.388 1 95.72
ENSMUSG00000045092 S1pr1 protein_coding 1.13 44.061 0.951

81.02 0.925 56.365 0.947 83.724
ENSMUSG00000045094 Arhgef37 protein_coding 0.938 81.01 0.826
60.678 1.29 22.444 0.974 93.045
ENSMUSG00000045095 Magi1 protein_coding 0.942 57.544 1.056
93.27 1.006 79.897 0.895 83.724
ENSMUSG00000045098 Suv420h1 protein_coding 0.992 82.352
1.026 83.104 0.981 41.662 1.004 91.625
ENSMUSG00000045100 Slc25a26 protein_coding 0.95 16.895
1.042 90.68 1.006 55.352 1.01 93.156
ENSMUSG00000045102 Poln protein_coding 0.808 10.704 1.219
71.98 1.012 75.009 1.005 96.287
ENSMUSG00000045103 Dmd protein_coding 1.103 1.323 NA NA
0.905 3.428 0.946 85.493
ENSMUSG00000045104 Gm5514 pseudogene 1 86.918 1
91.584 1 84.669 1 93.612
ENSMUSG00000045106 Ccdc73 protein_coding 0.765 22.431
1.229 82.904 1.064 87.744 1.149 84.499
ENSMUSG00000045107 Saysd1 protein_coding 1.02 72 1.04
96.984 0.945 59.383 0.996 86.699
ENSMUSG00000045109 Krtap4-7 protein_coding 1 84.977 NA
NA 1 66.269 1 95.832
ENSMUSG00000045110 Rassf8 protein_coding 1 86.843 NA
NA 1 81.492 1 96.183
ENSMUSG00000045111 Taar6 protein_coding 1 86.006 NA NA
1 75.527 1 94.752
ENSMUSG00000045114 Prrt2 protein_coding 0.898 51.089 1.117
83.104 0.994 83.397 1.017 96.192
ENSMUSG00000045126 Olfr1445 protein_coding 1 85.203 NA
NA 1 85.921 1 94.153
ENSMUSG00000045128 Rpl18a protein_coding 1.032 25.026
0.984 74.483 0.985 73.096 1.049 96.287
ENSMUSG00000045132 Olfr620 protein_coding 1 78.428 NA
NA 1 84.724 1 95.273
ENSMUSG00000045136 Tubb2b protein_coding 0.922 70.862
1.016 95.088 1.063 72.148 1.156 42.736
ENSMUSG00000045140 Pigw protein_coding 0.977 86.145 1.09
84.999 0.936 64.427 0.97 95.559
ENSMUSG00000045148 Olfr1255 protein_coding 1 83.169 1
95.504 1 84.724 1 95.777
ENSMUSG00000045149 Gm8946 pseudogene 1 84.168 NA NA
1 68.82 1 94.904
ENSMUSG00000045150 Olfr1161 protein_coding 1 80.251 1
97.263 1 88.274 1 95.877
ENSMUSG00000045160 Bola3 protein_coding 0.855 7.004 1.078 89.014
1.085 37.259 0.977 96.183
ENSMUSG00000045165 AI467606 protein_coding 1.055 24.689
0.985 95.708 0.958 67.962 0.966 91.372
ENSMUSG00000045174 Amer3 protein_coding 1 76.686 1
97.097 1 87.37 1 95.273
ENSMUSG00000045176 2310047M10Rik protein_coding 0.995 82.705
0.974 88.881 NA NA 1.026 96.042
ENSMUSG00000045178 4933411O13Rik antisense 1 85.637 1
95.708 NA NA 1 96.287
ENSMUSG00000045179 Sox3 protein_coding 1 69.489 1

91.661	1	80.749	1	92.263
ENSMUSG00000045180	Shroom2	protein_coding	0.915	43.495
0.889	72.399	1.231	27.89	0.975	88.505
ENSMUSG00000045193	Cirbp	protein_coding	0.873	43.816	0.822
80.052	1.388	15.688	1.013	85.493
ENSMUSG00000045201	Lrrc3b	protein_coding	1.654	0.664	0.881
90.744	0.684	9.563	0.848	90.628
ENSMUSG00000045202	Olfr123	protein_coding	1	82.231	NA
NA	1	82.74	1	96.248
ENSMUSG00000045204	Olfr835	protein_coding	1	70.741	1
96.001	1	82.575	1	93.156
ENSMUSG00000045205	Dpy19l4	protein_coding	1.013	74.958
1.013	97.097	0.976	61.288	0.986	90.611
ENSMUSG00000045210	Vcpip1	protein_coding	1.015	48.857
0.966	91.661	1.02	85.21	1.019	93.264
ENSMUSG00000045211	Nudt18	protein_coding	0.897	43.435
1.075	86.793	1.039	85.352	1.03	95.273
ENSMUSG00000045215	Asxl3	protein_coding	0.996	67.991	0.867
72.399	1.152	48.772	0.828	85.493
ENSMUSG00000045216	Hs6st1	protein_coding	1.045	46.533
0.963	94.48	0.992	65.409	0.945	89.294
ENSMUSG00000045217	Ppp1r2-ps9	pseudogene	1	82.352	1
97.049	1	87.786	1	96.183
ENSMUSG00000045225	Olfr1152	protein_coding	1	70.741	NA
NA	1	73.428	1	95.832
ENSMUSG00000045231	BC106179	protein_coding	0.982	77.113
0.817	68.115	1.248	20.572	0.875	96.071
ENSMUSG00000045236	Krtap5-4	protein_coding	1	86.199	1
97.097	1	84.896	1	96.248
ENSMUSG00000045237	1110012L19Rik	protein_coding	0.994	71.142
1.01	96.847	0.996	86.985	1.014	96.192
ENSMUSG00000045238	A730035I17Rik	pseudogene	0.58	33.745
0.683	71.98	2.514	25.966	1.218	92.675
ENSMUSG00000045246	Kcng4	protein_coding	0.844	63.126	2.157
83.104	0.55	57.446	0.512	90.611
ENSMUSG00000045248	Med26	protein_coding	1.067	57.655	1.005
95.211	0.93	87.786	1.01	85.786
ENSMUSG00000045251	Zfp688	protein_coding	0.937	47.266	0.97
89.509	1.101	32.173	1.023	92.31
ENSMUSG00000045252	Zfp574	protein_coding	0.953	24	1.067
71.617	0.983	63.075	0.96	83.724
ENSMUSG00000045257	Morn2	protein_coding	1.144	20.444	0.945
79.475	0.925	69.134	0.995	96.287
ENSMUSG00000045259	Klhdc9	protein_coding	1.224	50.627
0.998	87.147	0.821	82.685	0.953	89.428
ENSMUSG00000045267	Tas2r119	protein_coding	1	84.865	NA
NA	1	83.275	1	93.045
ENSMUSG00000045268	Zfp691	protein_coding	1.059	7.255	0.925
68.115	1.019	73.096	0.984	86.699
ENSMUSG00000045273	Cenph	protein_coding	1.085	41.454	0.926
75.993	0.993	87.744	1.569	71.653
ENSMUSG00000045275	Lca5l	protein_coding	0.892	69.24	0.931	95.504
1.202	61.171	0.867	85.493
ENSMUSG00000045281	Gpr20	protein_coding	1.369	20.659	NA	NA

0.605 19.765 0.992 94.904
ENSMUSG00000045282 Tmem86b protein_coding 0.856 35.646
1.048 93.899 1.111 49.601 0.939 88.053
ENSMUSG00000045284 Dcaf12l1 protein_coding 0.795 26.013
1.133 93.994 1.11 68.425 1.028 94.654
ENSMUSG00000045287 Rtn4rl1 protein_coding 1.179 8.253 1.074
90.116 0.792 4.935 1.07 91.525
ENSMUSG00000045291 E330021D16Rik protein_coding 1 82.705
1 97.049 1 80.015 1 91.872
ENSMUSG00000045294 Insig1 protein_coding 0.939 66.129
0.972 96.001 1.092 78.864 0.946 90.674
ENSMUSG00000045302 Preb protein_coding 0.921 24.817 1.052
82.904 1.032 60.104 1.058 60.466
ENSMUSG00000045306 Olfr734 protein_coding 1 85.391 NA
NA 1 84.071 1 91.492
ENSMUSG00000045312 Lhfpl2 protein_coding 1.567 3.932 0.932
95.504 0.686 6.858 0.971 93.612
ENSMUSG00000045314 Sowahb protein_coding 0.963 77.763
0.877 72.399 1.183 34.727 0.845 84.29
ENSMUSG00000045316 Fahd1 protein_coding 0.855 5.544 1.108 80.14
1.057 43.586 1.033 87.134
ENSMUSG00000045318 Adra2c protein_coding 0.689 30.136
1.151 97.263 1.259 33.042 1.533 51.967
ENSMUSG00000045319 Proser2 protein_coding 0.746 5.311 0.882
93.046 1.517 18.208 1.031 86.991
ENSMUSG00000045322 Tlr9 protein_coding 1.321 17.191 0.996
97.263 0.758 14.079 0.975 94.468
ENSMUSG00000045326 Fndc7 protein_coding 0.727 50.229 1.137
92.062 1.204 86.666 0.958 86.991
ENSMUSG00000045327 6330549D23Rik pseudogene 1.057 53.562 1
86.604 0.948 80.749 0.792 83.724
ENSMUSG00000045328 Cenpe protein_coding 1.247 39.851 0.724
70.29 1.104 87.898 1.712 51.967
ENSMUSG00000045330 4933402E13Rik protein_coding 1 86.351
NA NA 1 80.749 1 92.018
ENSMUSG00000045331 2310079G19Rik protein_coding 1 85.499
1 96.268 1 86.666 1 94.608
ENSMUSG00000045333 Zfp423 protein_coding 0.769 6.409 1.123
71.617 1.155 55.81 0.958 92.12
ENSMUSG00000045336 Hsfy2 protein_coding 1 82.092 1
96.847 1 77.704 1 94.654
ENSMUSG00000045337 Defb11 protein_coding 1 76.686 1
96.843 1 86.831 1 91.372
ENSMUSG00000045339 A930104D05Rik lincRNA 1 81.01 1
96.843 NA NA 1 89.294
ENSMUSG00000045340 Vmn1r70 protein_coding 1 79.853 1
95.088 1 72.853 1 95.72
ENSMUSG00000045341 Olfr167 protein_coding 1 78.299 1
95.504 1 87.686 1 94.904
ENSMUSG00000045345 9530056K15Rik pseudogene 0.905 51.056
0.929 92.631 1.191 34.07 1.009 81.56
ENSMUSG00000045348 Nyap1 protein_coding 0.777 13.45 1.05 91.643
1.23 56.125 0.942 86.991
ENSMUSG00000045349 Sh2d5 protein_coding 0.899 54.604 1.121

86.34 0.991 88.274 0.883 90.805
ENSMUSG00000045350 Fam186a protein_coding 0.849 60.003
1.099 96.843 1.074 70.1 0.8 91.372
ENSMUSG00000045362 Tnfrsf26 protein_coding 1.57 9.805 0.622
55.265 1.018 85.675 0.993 90.865
ENSMUSG00000045364 Ifne protein_coding 1 86.145 1
90.256 1 69.315 1 93.156
ENSMUSG00000045365 Rbm15b protein_coding 1.049 43.939
0.985 93.574 0.964 27.194 0.912 83.724
ENSMUSG00000045374 Wdr81 protein_coding 1.064 10.19 0.978 87.452
0.957 43.426 0.994 96.183
ENSMUSG00000045377 Tmem88 protein_coding 1.065 80.03 1.127
82.904 0.833 39.528 0.91 89.587
ENSMUSG00000045378 Pxt1 protein_coding 1.367 46.097 0.819
75.345 0.889 78.954 1.11 82.091
ENSMUSG00000045382 Cxcr4 protein_coding 1.064 60.435 1.162
83.104 0.81 23.48 0.899 84.29
ENSMUSG00000045391 1700120B22Rik protein_coding 0.95 71.36 1.04
96.68 1.012 83.925 0.747 83.724
ENSMUSG00000045392 Olfr1033 protein_coding 0.865 44.787
0.852 71.889 1.352 9.86 1.147 83.067
ENSMUSG00000045394 Epcam protein_coding 0.971 60.569 0.937
72.399 1.096 37.259 0.845 86.991
ENSMUSG00000045395 Olfr1499 protein_coding 1 81.823 1
95.735 NA NA 1 96.183
ENSMUSG00000045404 Kcnk13 protein_coding 1.327 11.197 0.82
90.053 0.921 59.383 1.098 77.331
ENSMUSG00000045409 Trim39 protein_coding 0.979 22.431
0.975 95.941 1.044 21.873 0.998 88.657
ENSMUSG00000045410 Akr1e1 protein_coding 0.977 32.749
0.973 91.361 1.048 32.433 1.019 71.855
ENSMUSG00000045411 2410002F23Rik protein_coding 0.899 22.237
0.869 56.955 1.282 5.964 1.027 73.401
ENSMUSG00000045414 1190002N15Rik protein_coding 1.078 52.799
1.017 93.574 0.914 38.799 0.921 91.625
ENSMUSG00000045417 Vmn1r230 protein_coding 1 84.294 1
95.708 1 80.015 1 95.676
ENSMUSG00000045421 Olfr1390 protein_coding 1 84.049 1
95.708 1 88.693 1 95.273
ENSMUSG00000045427 Hnrnph2 protein_coding 1.052 71.06 0.924
68.115 1.029 46.956 1.087 33.269
ENSMUSG00000045435 Tmem60 protein_coding 0.982 77.763 1.08
82.809 0.946 48.851 0.967 96.358
ENSMUSG00000045438 Cox19 protein_coding 0.853 0.987 0.988 96.68
1.183 5.864 0.999 94.215
ENSMUSG00000045441 Gprin3 protein_coding 0.608 3.009 1.292
82.904 1.276 38.683 1.185 94.904
ENSMUSG00000045463 Slco6b1 protein_coding 1 85.728 1
90.256 1 87.971 1 96.071
ENSMUSG00000045464 2810002D19Rik processed_transcript 1.014
86.57 0.971 87.553 1.018 84.504 0.929 84.29
ENSMUSG00000045466 Zfp956 protein_coding 0.812 3.101 1.054
91.643 1.168 10.819 1.064 95.74
ENSMUSG00000045467 Ttll13 protein_coding 0.955 36.259

0.933 95.088 1.124 41.806 1.107 94.904
ENSMUSG00000045471 Hcrt protein_coding 1 81.178 1
79.475 1 63.801 1 96.086
ENSMUSG00000045474 Olfr1368 protein_coding 1 79.709 1
96.68 1 86.666 1 94.904
ENSMUSG00000045475 Lce3c protein_coding 1 80.251 1
96.013 1 82.161 1 96.086
ENSMUSG00000045479 Olfr459 protein_coding 1 82.231 1
96.407 1 73.309 1 95.74
ENSMUSG00000045482 Trrap protein_coding 0.96 14.403 0.974
93.574 1.071 28.301 0.953 84.19
ENSMUSG00000045493 Bhlhe23 protein_coding 1 81.823 1
95.301 1 86.258 1 94.654
ENSMUSG00000045498 Pcdhb3 protein_coding 1.27 14.205
1.052 87.609 0.745 12.948 0.867 92.181
ENSMUSG00000045502 Niacr1 protein_coding 0.786 14.086
1.028 96.013 1.241 19.106 0.856 91.979
ENSMUSG00000045503 Sys1 protein_coding 0.978 80.51 1.012 96.335
1.011 86.258 0.999 95.877
ENSMUSG00000045506 A930002H24Rik protein_coding 0.927 54.176
0.865 96.68 1.247 88.274 1.07 94.904
ENSMUSG00000045508 Olfr1367 protein_coding 1 78.744 1
96.843 1 86.985 1 90.463
ENSMUSG00000045518 Onecut3 protein_coding 1 67.334 1
96.843 1 84.229 1 95.74
ENSMUSG00000045519 Zfp560 protein_coding 1.115 6.281 0.934
94.102 0.961 19.449 0.984 90.157
ENSMUSG00000045520 NA NA NA NA 1 93.469 1
86.144 1 91.525
ENSMUSG00000045528 Olfr891 protein_coding 1 80.251 1
95.708 1 86.258 1 95.273
ENSMUSG00000045534 Kcna5 protein_coding 0.949 64.284 0.987
97.155 1.071 80.015 0.974 92.181
ENSMUSG00000045538 Ddx28 protein_coding 0.847 27.431 1.098
97.155 1.075 33.802 1.048 83.865
ENSMUSG00000045539 Sprr3 protein_coding 1 86.006 1
96.407 1 85.792 1 95.273
ENSMUSG00000045540 Olfr600 protein_coding 1 85.59 1
95.088 1 85.675 1 95.877
ENSMUSG00000045545 Krt14 protein_coding 0.843 15.912 1.015
96.68 1.172 43.307 0.983 87.182
ENSMUSG00000045546 Rnf113a2 pseudogene 1 78.909 NA NA
1 87.431 1 95.273
ENSMUSG00000045551 Fpr1 protein_coding 1.416 31.24 0.551 72.712
1.28 87.744 1.139 93.964
ENSMUSG00000045555 Mettl24 protein_coding 1.066 86.006
1.052 93.574 0.892 81.169 0.86 83.724
ENSMUSG00000045559 Olfr827 protein_coding 1 72.655 1
95.708 1 87.744 1 96.086
ENSMUSG00000045566 Sprr4 protein_coding 1 74.745 1
87.313 NA NA 1 96.358
ENSMUSG00000045569 Mc2r protein_coding 1.333 1.323 1.023 88.897
0.734 6.858 0.907 83.724
ENSMUSG00000045573 Penk protein_coding 0.803 57.014 0.934

92.545 1.335 64.024 0.972 96.358
ENSMUSG00000045575 Vmn1r233 protein_coding 1 80.251 1
95.504 1 80.899 1 95.273
ENSMUSG00000045576 St7l protein_coding 1.05 27.731 0.968
86.604 0.979 76.64 0.977 91.372
ENSMUSG00000045581 Olfr710 protein_coding 1 85.203 1
95.941 1 82.74 1 94.904
ENSMUSG00000045584 Olfr610 protein_coding 1 75.567 1
90.378 1 87.519 1 96.139
ENSMUSG00000045587 BC049730 protein_coding 1 69.943 1
94.597 1 80.381 1 93.156
ENSMUSG00000045589 Frrs1l protein_coding 0.941 77.113
0.737 94.597 1.447 73.759 0.706 88.552
ENSMUSG00000045591 Olig3 protein_coding 1 84.977 1
94.597 1 85.757 1 91.872
ENSMUSG00000045594 Glb1 protein_coding 1.132 19.746 0.942
87.452 0.937 37.906 0.972 93.584
ENSMUSG00000045598 Zfp553 protein_coding 1.034 86.843
0.953 95.088 1.018 79.897 0.967 90.289
ENSMUSG00000045609 Gm5455 pseudogene 1 86.658 NA NA
1 85.352 1 94.468
ENSMUSG00000045613 Chrm2 protein_coding 2.246 12.295 0.815
95.329 0.545 16.643 0.676 87.651
ENSMUSG00000045620 Odf3l1 protein_coding 0.435 0.987 1.42
40.99 1.614 7.635 1.199 83.067
ENSMUSG00000045624 Esf1 protein_coding 1.119 24.253 0.945
55.71 0.946 85.935 1.078 61.984
ENSMUSG00000045625 Pigz protein_coding 1.637 6.281 NA NA
0.637 9.901 0.79 85.493
ENSMUSG00000045629 Sh3tc2 protein_coding 0.813 11.103
1.152 90.895 1.066 10.76 1.069 91.872
ENSMUSG00000045636 Mtus1 protein_coding 0.877 5.311 1.008 83.104
1.125 29.302 0.957 84.29
ENSMUSG00000045639 Zfp629 protein_coding 0.978 79.709
0.991 94.848 1.029 63.301 0.946 86.445
ENSMUSG00000045648 Vwc2l protein_coding 1 82.982 1
95.708 1 80.015 1 96.358
ENSMUSG00000045655 Fam216b protein_coding 1 82.705 1
96.216 1 83.397 1 94.567
ENSMUSG00000045657 Pcdhb10 protein_coding 0.868 21.658 1.36
71.98 0.847 58.516 1.222 71.305
ENSMUSG00000045658 Pid1 protein_coding 0.972 77.615 0.891
79.807 1.154 33.287 1.079 59.464
ENSMUSG00000045659 Plekha7 protein_coding 0.981 39.712
1.002 93.094 1.02 38.152 0.894 83.724
ENSMUSG00000045662 Henmt1 protein_coding 0.608 63.736 NA
NA 0.934 86.258 1.389 87.447
ENSMUSG00000045664 Cdc42ep2 protein_coding 1.039 66.944
1.122 82.904 0.858 18.797 1.08 67.691
ENSMUSG00000045665 NA NA NA NA 1.098 84.832 0.887
51.055 1.03 90.397
ENSMUSG00000045667 Smtnl2 protein_coding 1.686 41.951
1.864 64.304 0.319 1.705 1.026 91.928
ENSMUSG00000045671 Spred2 protein_coding 1.009 85.129

1.068 71.617 0.929 35.815 0.919 84.29
ENSMUSG00000045672 Col27a1 protein_coding 1.085 11.459
0.895 91.661 1.03 62.058 1.413 31.133
ENSMUSG00000045678 Olfr1489 protein_coding 1 81.125 1
96.216 1 81.976 1 92.31
ENSMUSG00000045679 Pqlc3 protein_coding 0.862 3.688 1.096 76.634
1.055 45.667 0.976 94.904
ENSMUSG00000045684 Lcn6 protein_coding 1 84.661 1
96.216 1 88.693 1 94.904
ENSMUSG00000045689 Pcdhb4 protein_coding 0.954 52.9 0.859
95.708 1.219 47.512 1.228 72.992
ENSMUSG00000045690 Wdr89 protein_coding 1.079 26.77 0.971 78.877
0.953 87.744 1.068 57.1
ENSMUSG00000045691 Thtpa protein_coding 0.956 47.266 0.887
77.944 1.178 9.502 0.982 96.184
ENSMUSG00000045693 Nlrp4e protein_coding 1 86.199 1
96.847 1 86.727 1 94.654
ENSMUSG00000045694 Gm4997 pseudogene 1 75.858 1
97.263 1 81.169 1 96.287
ENSMUSG00000045699 Gm21411 protein_coding 1 85.59 NA NA
1 88.657 1 96.183
ENSMUSG00000045708 Olfr447 protein_coding 1 86.843 1
96.216 1 80.634 1 94.297
ENSMUSG00000045709 1700023L04Rik lincRNA 0.889 35.449 0.7
48.565 1.601 5.864 0.99 90.177
ENSMUSG00000045713 Vmn1r76 protein_coding 1 82.543 1
96.268 1 82.161 1 96.192
ENSMUSG00000045725 Prr15 protein_coding 1.081 55.789 1.03
91.532 0.899 78.045 1.059 95.74
ENSMUSG00000045730 Adrb2 protein_coding 1.109 50.627 0.952
74.483 0.942 79.325 0.938 92.181
ENSMUSG00000045731 Pnoc protein_coding 1 64.014 1
96.763 1 82.74 1 86.209
ENSMUSG00000045733 Sprn protein_coding 1.27 26.136 0.86
90.378 0.911 31.676 1.061 95.92
ENSMUSG00000045744 Bricd5 protein_coding 0.818 11.128
0.986 93.707 1.236 25.564 0.838 86.445
ENSMUSG00000045746 B230317F23Rik protein_coding 1.19 51.826
NA NA 0.853 62.791 0.744 86.699
ENSMUSG00000045750 D830014E11Rik protein_coding 1 84.452
NA NA 1 84.961 1 96.183
ENSMUSG00000045751 Mms22l protein_coding 0.807 28.804
0.916 88.705 1.349 32.433 1.452 44.679
ENSMUSG00000045752 Tssc4 protein_coding 0.894 1.15 1.051 87.185
1.065 16.808 1.121 50.74
ENSMUSG00000045757 Zfp764 protein_coding 1.108 19.746
0.975 96.013 0.927 28.901 0.952 86.699
ENSMUSG00000045761 Fam179a protein_coding 1.169 78.639
0.791 75.4 1.079 49.601 0.651 84.29
ENSMUSG00000045763 Basp1 protein_coding 1.011 82.092 1.059
97.263 0.931 85.134 1.02 88.111
ENSMUSG00000045767 B230219D22Rik protein_coding 1.026 7.908
1.004 96.843 0.969 7.809 0.978 86.699
ENSMUSG00000045775 Slc16a5 protein_coding 1.691 3.598 1.058

97.129	0.559	3.943	0.989	92.181
ENSMUSG00000045776	Lrtm1	protein_coding	0.968	56.768	1.165
79.261	0.886	53.224	0.93	93.734
ENSMUSG00000045777	Ifitm10	protein_coding	0.597	0.987	0.789
82.947	2.12	5.864	1.159	72.369
ENSMUSG00000045780	Olfr624	protein_coding	1	75.567	1
97.049	1	84.724	1	95.877
ENSMUSG00000045790	Ccdc149	protein_coding	1.016	73.841
0.966	92.474	1.017	88.161	1.083	93.132
ENSMUSG00000045792	NA	NA	NA	NA	1	96.407	1
88.349	1	95.72
ENSMUSG00000045795	Whamm	protein_coding	0.975	43.167	0.84
61.625	1.216	9.502	1.033	93.722
ENSMUSG00000045797	4930402K13Rik	protein_coding	1	85.283
1	97.155	NA	NA	1	92.181
ENSMUSG00000045799	Gm9800	pseudogene	0.669	54.604	1.011
95.708	1.473	57.227	0.736	90.611
ENSMUSG00000045802	Hsf3	protein_coding	1.289	21.836	0.93
94.102	0.832	48.387	1.022	88.505
ENSMUSG00000045812	Olfr984	protein_coding	1	81.01	1
97.155	1	76.923	1	95.273
ENSMUSG00000045815	1700101I19Rik	protein_coding	1	86.006
1	96.268	1	62.237	1	94.904
ENSMUSG00000045817	Zfp36l2	protein_coding	0.996	76.546
1.087	83.104	0.921	16.719	0.962	86.991
ENSMUSG00000045822	Zswim3	protein_coding	0.944	23.556
1.066	80.678	0.992	62.648	0.961	95.405
ENSMUSG00000045824	Olfr574	protein_coding	1	79.068	1
97.049	1	88.203	1	94.904
ENSMUSG00000045826	Ptprcap	protein_coding	0.726	30.035
1.533	64.304	0.896	66.201	0.978	96.183
ENSMUSG00000045827	Serpinb9	protein_coding	1.164	2.233	1.026
96.847	0.834	6.935	0.931	89.827
ENSMUSG00000045835	Hdgfl1	protein_coding	1	68.66	1
96.216	1	86.602	1	93.685
ENSMUSG00000045838	A430105I19Rik	protein_coding	0.94	64.014
1.056	88.533	1.008	80.5	1.223	51.967
ENSMUSG00000045842	Gm7148	protein_coding	1	74.8	1
96.948	1	88.693	1	96.287
ENSMUSG00000045854	Lyrm2	protein_coding	1.034	81.823	0.974
97.263	0.988	88.41	1.007	92.018
ENSMUSG00000045867	Cradd	protein_coding	0.984	81.125	1.012
94.887	1.001	80.899	1.222	72.826
ENSMUSG00000045868	Gvin1	protein_coding	1.165	65.733	0.951
96.407	0.9	46.575	0.836	86.699
ENSMUSG00000045871	Slitrk6	protein_coding	1.219	43.939	1.47
87.185	0.559	36.786	1.472	75.81
ENSMUSG00000045875	Adra1a	protein_coding	0.963	77.113
1.052	83.104	0.987	84.434	1.283	77.621
ENSMUSG00000045876	Pcdhb8	protein_coding	0.836	61.887
1.223	90.29	0.98	80.381	1.056	78.379
ENSMUSG00000045877	4933415A04Rik	protein_coding	0.61	52.258
1.625	96.847	1.01	52.574	0.332	89.525
ENSMUSG00000045883	Olfr1461	protein_coding	1	84.865	1

93.908 1 82.903 1 96.086
ENSMUSG00000045896 Paip2b protein_coding 1.08 10.687
0.959 89.277 0.969 38.534 1.042 92.748
ENSMUSG00000045903 Npas4 protein_coding 1.115 76.042 0.977
96.407 0.917 87.786 0.851 88.053
ENSMUSG00000045912 C2cd4c protein_coding 0.938 86.474
0.924 79.475 1.147 69.196 1.335 74.193
ENSMUSG00000045915 Ccdc42 protein_coding 0.838 66.406
1.606 89.494 0.741 86.985 1.136 91.525
ENSMUSG00000045917 6330416G13Rik protein_coding 0.997 86.172
1.017 82.904 0.983 28.669 1.071 59.464
ENSMUSG00000045930 Clec14a protein_coding 1.158 2.655 0.974
95.708 0.884 45.573 1.016 96.183
ENSMUSG00000045932 Ifit2 protein_coding 1.186 4.228 1.04 92.474
0.81 35.815 1.206 51.967
ENSMUSG00000045934 Mtmr11 protein_coding 1.103 6.76 1.033
89.434 0.879 5.701 1.07 93.016
ENSMUSG00000045942 BC049762 protein_coding 0.946 62.354
0.846 78.877 1.249 58.616 0.557 83.724
ENSMUSG00000045946 Gm9804 protein_coding 1 83.726 1
90.378 NA NA 1 96.184
ENSMUSG00000045948 Mrps12 protein_coding 0.873 7.881 1
92.062 1.146 30.213 1.013 96.086
ENSMUSG00000045952 Gm5548 pseudogene 1 77.615 1
95.329 1 87.898 1 96.086
ENSMUSG00000045954 Sdpr protein_coding 0.879 3.641 NA NA
1.133 12.831 0.898 93.612
ENSMUSG00000045962 Wnk1 protein_coding 1.024 63.534 NA NA
NA NA 0.978 87.447
ENSMUSG00000045967 Gpr158 protein_coding 1 78.909 1
97.155 1 75.155 1 87.447
ENSMUSG00000045968 5830403L16Rik protein_coding 0.716 5.491 NA
NA 1.335 14.169 0.887 94.99
ENSMUSG00000045969 Ing1 protein_coding 0.998 71.06 0.968 93.908
1.037 88.274 0.987 86.991
ENSMUSG00000045973 Slc25a51 protein_coding 0.947 44.562
0.952 88.705 1.11 36.839 1.064 61.753
ENSMUSG00000045975 C2cd2 protein_coding 0.988 63.736 1.052
77.085 0.964 39.528 1.025 89.052
ENSMUSG00000045980 Tmem104 protein_coding 1.152 4.362 1.04
87.037 0.836 0.879 1.004 80.573
ENSMUSG00000045983 Eif4g1 protein_coding 0.797 0.987 1.04
83.464 1.206 5.864 1.114 31.133
ENSMUSG00000045989 4930451I11Rik protein_coding 1 86.145
1 94.597 1 88.729 1 96.358
ENSMUSG00000045994 B3gat1 protein_coding 0.694 45.728
1.055 82.904 1.361 85.921 0.911 87.992
ENSMUSG00000045996 Polr2k protein_coding 0.981 39.712 NA
NA 1.027 66.647 1.072 64.513
ENSMUSG00000045999 AY036118 pseudogene 0.855 59.674 0.96
85.418 1.221 25.966 0.823 83.724
ENSMUSG00000046000 Naa11 protein_coding 1 86.474 1
97.155 1 83.811 1 94.904
ENSMUSG00000046005 D830044D21Rik lincRNA 1 73.734 1

86.861 1 82.74 1 93.685
ENSMUSG00000046006 Gapt protein_coding 1.67 0.355 1.221 93.994
0.49 6.524 1.169 71.855
ENSMUSG00000046008 Pnlip protein_coding 1 61.043 1
95.211 1 85.935 1 96.287
ENSMUSG00000046010 Zfp830 protein_coding 1.062 14.59 0.905
72.576 NA NA 1.047 80.324
ENSMUSG00000046016 Olfr1364 protein_coding 1 79.93 1
96.68 1 88.601 1 94.384
ENSMUSG00000046020 Pofut1 protein_coding 1.024 56.085
1.007 96.843 0.972 39.245 1.014 56.863
ENSMUSG00000046027 Stard5 protein_coding 1.036 32.676
0.942 85.116 1.027 84.552 0.994 94.654
ENSMUSG00000046031 Fam26f protein_coding 1.336 2.651 0.938
96.216 0.799 13.848 1.044 96.358
ENSMUSG00000046032 Snx12 protein_coding 0.959 22.481 NA NA
1.047 37.741 1.069 31.133
ENSMUSG00000046034 Fam105b protein_coding 1.11 3.152 0.92
36.617 0.98 87.786 0.99 83.581
ENSMUSG00000046041 Olfr803 protein_coding 1 86.333 1
95.504 1 87.173 1 94.904
ENSMUSG00000046049 Rp1l1 protein_coding 1 65.284 1
93.654 1 85.21 1 96.071
ENSMUSG00000046056 Sbsn protein_coding 1.013 61.334 0.823
56.027 1.197 9.429 0.958 91.372
ENSMUSG00000046057 Gm15428 pseudogene 1 78.909 1
96.897 1 79.426 1 96.086
ENSMUSG00000046058 Eid2 protein_coding 1.066 80.608 0.971
96.268 0.963 86.258 0.912 89.892
ENSMUSG00000046062 Ppp1r15b protein_coding 0.963 61.887
1.158 71.617 0.899 22.279 1.025 92.691
ENSMUSG00000046070 Igfals protein_coding 1.071 57.655
1.511 63.504 0.616 0.72 0.858 83.724
ENSMUSG00000046076 Olfr998 protein_coding 1 85.946 1
93.654 NA NA 1 95.74
ENSMUSG00000046079 Lrrc8d protein_coding 1.216 7.255 0.979
85.075 0.835 17.258 0.891 83.724
ENSMUSG00000046080 Clec9a protein_coding 0.947 67.477
1.105 90.116 0.951 84.434 0.772 84.19
ENSMUSG00000046082 Tmem174 protein_coding 1 84.564 1
86.604 1 87.786 1 93.457
ENSMUSG00000046085 4931422A03Rik protein_coding 0.765 15.227
0.859 87.452 1.522 8.647 0.946 87.992
ENSMUSG00000046088 Gm9805 protein_coding 1 86.006 1
96.216 1 74.535 1 94.99
ENSMUSG00000046093 Hpcal4 protein_coding 0.794 29.454
1.284 80.775 0.978 83.538 1.01 94.904
ENSMUSG00000046095 Krt32 protein_coding 0.306 34.572 1.134
90.378 2.874 25.425 2.693 86.209
ENSMUSG00000046096 BC030336 protein_coding 1.073 25.944
0.972 82.503 0.957 37.906 1.021 93.964
ENSMUSG00000046108 Il17c protein_coding 0.575 48.939 1.381
91.643 1.261 57.227 0.95 92.851
ENSMUSG00000046110 Serinc4 protein_coding 0.796 7.916 1.13

						87.282	1.113	28.979	0.907	85.493
ENSMUSG00000046111	5830418K08Rik	protein_coding	0.891	22.92	1.038	96.378	1.076	43.481	1.091	64.513
ENSMUSG00000046119	1110008E08Rik	lincRNA	1	84.564	1	95.708	1	82.903	1	94.608
ENSMUSG00000046130	Vmn1r184	protein_coding	1	75.399	1	97.155	1	85.757	1	95.35
ENSMUSG00000046133	C130073F10Rik	protein_coding	1	84.661	1	94.597	1	88.274	1	95.832
ENSMUSG00000046138	9930021J03Rik	protein_coding	1.022	77.407	1.003	87.029	0.975	66.425	0.92	85.493
ENSMUSG00000046139	Patl1	protein_coding	1.139	0.355	NA	NA	0.868	0.72	0.936	83.724
ENSMUSG00000046145	1700018M17Rik	pseudogene	0.67	14.427	1.26	91.776	1.188	52.8	0.63	84.19
ENSMUSG00000046150	Olfr918	protein_coding	1	80.146	1	96.216	1	85.352	1	93.457
ENSMUSG00000046152	Fut10	protein_coding	1.151	9.435	1.026	97.155	0.847	16.643	0.976	95.877
ENSMUSG00000046157	Tmem229b	protein_coding	1.21	0	0.952	91.275	0.869	2.087	0.987	95.273
ENSMUSG00000046159	Chrm3	protein_coding	1.497	34.861	0.882	94.48	0.755	44.211	0.685	83.724
ENSMUSG00000046160	Olig1	protein_coding	1.103	46.289	1.812	78.705	0.498	71.388	0.379	87.735
ENSMUSG00000046167	Gldn	protein_coding	1.106	72.27	0.931	95.504	0.972	70.44	0.924	85.493
ENSMUSG00000046168	Kcnrg	protein_coding	0.925	50.768	0.594	93.654	1.823	62.307	1.045	95.72
ENSMUSG00000046169	Adamts6	protein_coding	1.641	1.292	0.728	82.671	0.838	9.288	1.022	87.735
ENSMUSG00000046173	Pabpc6	protein_coding	1	72.139	1	96.216	1	84.434	1	94.904
ENSMUSG00000046178	Nxph1	protein_coding	1.135	75.196	0.987	93.654	NA	NA	0.662	86.991
ENSMUSG00000046179	E2f8	protein_coding	1.479	10.595	0.901	78.877	0.749	54.25	2.105	42.736
ENSMUSG00000046180	4930550L24Rik	protein_coding	1	84.263	1	94.887	1	75.967	1	96.248
ENSMUSG00000046182	Gsg1l	protein_coding	1.228	86.57	0.563	94.597	1.45	67.163	0.866	90.611
ENSMUSG00000046185	Zfp84	protein_coding	1.011	86.464	1.027	96.843	0.963	88.161	0.96	96.287
ENSMUSG00000046186	Cd109	protein_coding	1.354	1.828	1.095	83.104	0.672	1.871	1.098	86.445
ENSMUSG00000046191	Pcdhb20	protein_coding	0.972	73.487	1.047	83.104	0.979	81.388	1.086	90.125
ENSMUSG00000046196	Ttc39d	protein_coding	0.8	86.464	1.798	96.843	0.692	88.274	0.6	87.992
ENSMUSG00000046201	Scaf8	protein_coding	1.093	7.179	1.001	95.329	0.914	10.003	0.977	91.625
ENSMUSG00000046203	Sprr2g	protein_coding	1	77.407	1	95.504	1	82.685	1	94.82
ENSMUSG00000046204	Pnma2	protein_coding	1.675	49.202	2.456					

82.809	0.242	11.64	0.791	91.372
ENSMUSG00000046207	Pik3r6	protein_coding	1.08	37.941
1.044	95.301	0.883	68.584	0.974	96.358
ENSMUSG00000046213	Cym	protein_coding	1.092	85.203	0.681
79.475	1.345	69.002	1.044	96.358
ENSMUSG00000046215	Rprml	protein_coding	0.85	55.303	0.919
84.594	1.281	36.839	1.767	46.412
ENSMUSG00000046219	C230012O17Rik	processed_transcript	1.432
73.841	0.729	96.847	0.96	85.21	0.957	83.067
ENSMUSG00000046223	Plaur	protein_coding	1.253	28.693	0.868
67.513	0.922	74.853	1.022	86.133
ENSMUSG00000046229	Scand1	protein_coding	0.859	20.799
1.231	80.14	0.949	85.935	0.936	86.699
ENSMUSG00000046230	Vps13a	protein_coding	0.696	0.987	0.965
85.116	1.486	5.864	1.165	60.31
ENSMUSG00000046240	Hepacam	protein_coding	0.967	82.092
1.304	92.062	0.794	73.027	1.02	96.086
ENSMUSG00000046245	Pilra	protein_coding	1.43	8.685	0.759	73.166
0.92	67.029	1.046	82.66
ENSMUSG00000046248	Krtap5-3	protein_coding	1	56.242	1
93.899	1	85.352	1	92.263
ENSMUSG00000046258	Adam29	protein_coding	1	73.585	1
92.931	1	69.196	1	96.183
ENSMUSG00000046259	Sprr2h	protein_coding	1	84.977	1
96.138	1	86.258	1	96.287
ENSMUSG00000046262	C87977	protein_coding	1	82.705	1
96.001	1	86.666	1	96.192
ENSMUSG00000046269	Usp27x	protein_coding	0.953	52.9	0.946
93.574	1.105	72.378	0.978	86.699
ENSMUSG00000046275	Tusc5	protein_coding	0.94	57.544	1.046
82.904	1.018	78.713	1.114	52.324
ENSMUSG00000046280	She	protein_coding	0.915	47.266	1.149
71.617	0.948	74.535	0.863	83.724
ENSMUSG00000046282	Adam20	protein_coding	1	65.199	NA
NA	1	84.552	1	95.92
ENSMUSG00000046292	Prps1l1	pseudogene	1	85.655	NA	NA
1	79.119	1	96.358
ENSMUSG00000046295	Ankle1	protein_coding	1.265	66.58	0.893
67.165	0.884	79.426	1.513	78.379
ENSMUSG00000046300	Olfr1412	protein_coding	1	68.485	1
95.088	1	87.971	1	95.877
ENSMUSG00000046311	Zfp62	protein_coding	1.085	22.205	0.977
90.138	0.945	56.625	1.02	65.175
ENSMUSG00000046312	AI464131	protein_coding	0.815	19.023
1.136	82.904	1.082	45.667	1.193	46.412
ENSMUSG00000046314	Stxbp6	protein_coding	1.076	32.058	NA
NA	0.918	48.133	1.082	83.067
ENSMUSG00000046317	BC107364	protein_coding	1.57	18.314
1.013	92.561	0.629	8.382	0.587	83.724
ENSMUSG00000046318	Ccbe1	protein_coding	0.927	69.985	0.644
72.399	1.677	34.285	0.603	86.991
ENSMUSG00000046321	Hs3st2	protein_coding	1	82.231	1
95.504	1	85.464	1	91.985
ENSMUSG00000046323	Dppa3	protein_coding	2.801	31.477	0.446

94.102 0.799 65.688 0.961 95.877
ENSMUSG00000046324 NA NA NA NA 0.961 92.914 1.039
78.864 1.027 83.581
ENSMUSG00000046329 Slc25a23 protein_coding 0.953 56.477
1.082 87.282 0.967 83.811 0.932 85.493
ENSMUSG00000046330 Rpl37a protein_coding 0.935 60.707 0.89
64.123 1.206 22.994 1.054 90.177
ENSMUSG00000046334 Gm6195 pseudogene 1 78.909 1
95.708 1 83.066 1 95.92
ENSMUSG00000046337 Fam178b protein_coding 1.04 60.707
1.045 96.763 0.917 81.032 0.898 94.654
ENSMUSG00000046338 Gpat2 protein_coding 0.578 44.562 1.124
93.899 1.536 54.809 1.459 92.748
ENSMUSG00000046341 Gm11223 pseudogene 1 83.418 1
93.574 1 81.62 1 94.297
ENSMUSG00000046345 Smco1 protein_coding 17.432 6.76 0.12
68.115 0.48 14.262 1.34 87.134
ENSMUSG00000046351 Zfp322a protein_coding 1.105 26.863
0.945 92.137 0.959 65.688 1.013 83.865
ENSMUSG00000046352 Gjb2 protein_coding 1.044 83.726 0.803
86.277 1.188 66.647 1.092 92.018
ENSMUSG00000046364 Rpl27a protein_coding 1.118 1.949 0.915
46.764 0.977 69.445 1.007 83.276
ENSMUSG00000046367 4931440L10Rik protein_coding 1 77.407
1 90.092 1 87.898 1 94.904
ENSMUSG00000046378 Asphd1 protein_coding 1.067 74.482
1.426 95.431 0.658 57.446 0.546 84.29
ENSMUSG00000046380 NA NA NA NA 0.949 77.598 1.078
38.926 1.015 92.389
ENSMUSG00000046387 Pcdhb17 protein_coding 0.863 13.952 1.17
76.065 0.988 88.729 1.029 96.183
ENSMUSG00000046388 Gm9806 pseudogene 0.193 18.487 1.242
92.355 4.168 12.35 1.976 92.018
ENSMUSG00000046396 Olfr609 protein_coding 1 85.832 1
96.216 1 84.504 1 95.72
ENSMUSG00000046402 Rbp1 protein_coding 1.22 20.659 0.838
62.692 0.98 84.552 1.037 93.722
ENSMUSG00000046404 Yod1 protein_coding 0.961 46.289 1.06
85.756 NA NA 0.888 83.724
ENSMUSG00000046408 1700067K01Rik protein_coding 0.866 53.679
1.31 80.678 0.881 66.425 0.725 83.724
ENSMUSG00000046410 Kcnk6 protein_coding 1.292 0 0.849 62.692
0.911 23.48 1.248 72.369
ENSMUSG00000046411 Gm6816 protein_coding 1 86.918 1
95.504 1 86.552 1 92.181
ENSMUSG00000046413 D230002A01Rik antisense 1.034 65.733
0.985 90.712 NA NA 0.874 83.724
ENSMUSG00000046415 B430212C06Rik processed_transcript 0.864
70.213 1.095 88.91 NA NA 1.086 86.118
ENSMUSG00000046417 Fam211a protein_coding 1.126 34.861 NA
NA 0.877 24.523 0.983 93.264
ENSMUSG00000046431 Olfr518 protein_coding 1 82.352 NA
NA 1 86.985 1 92.762
ENSMUSG00000046432 Ngfrap1 protein_coding 1.003 85.59 0.819

21.315	1.218	12.831	0.899	87.542
ENSMUSG00000046434	Hnrnpa1	protein_coding	1.164	3.434	0.861
8.462	0.994	80.634	1.094	0
ENSMUSG00000046435	Gm13078	protein_coding	1	83.169	NA
NA	1	87.898	1	88.755
ENSMUSG00000046438	Scgb2b24	protein_coding	1	85.832	1
96.216	1	86.727	1	96.358
ENSMUSG00000046440	Gm5564	pseudogene	1	85.092	NA	NA
1	88.349	1	92.851
ENSMUSG00000046441	Cmtr2	protein_coding	1.145	9.259	0.979	96.68
0.893	3.969	0.913	83.724
ENSMUSG00000046442	Ppm1e	protein_coding	1.07	77.916	0.812
70.693	1.154	51.851	1.153	86.133
ENSMUSG00000046447	Camk2n1	protein_coding	0.933	82.479
0.978	85.116	1.092	36.011	0.874	83.724
ENSMUSG00000046449	C77370	protein_coding	0.992	51.297
1.447	79.367	0.695	9.746	2.497	67.169
ENSMUSG00000046450	Olfr71	protein_coding	1	71.06	1
96.268	1	84.896	1	94.608
ENSMUSG00000046456	Tmem150b	protein_coding	0.98	80.51	1.005
94.102	1.013	62.648	0.82	83.724
ENSMUSG00000046460	Sh2d7	protein_coding	0.574	12.785	1.296
82.904	1.341	49.058	0.98	84.29
ENSMUSG00000046463	5930403N24Rik	protein_coding	0.688	25.551
1.017	95.196	1.432	34.07	0.905	94.19
ENSMUSG00000046470	Sox18	protein_coding	0.842	9.341	0.837	83.392
1.422	7.24	0.938	93.264
ENSMUSG00000046474	Krtap4-16	protein_coding	1	86.351	1
97.263	1	88.461	1	93.316
ENSMUSG00000046480	Scn4b	protein_coding	2.398	10.105	1.069
89.494	0.391	1.92	0.914	92.564
ENSMUSG00000046486	Olfr231	protein_coding	1	81.465	1
94.597	1	82.685	1	96.042
ENSMUSG00000046487	Mospd4	pseudogene	0.654	41.759	1.549
82.904	0.984	75.809	0.849	90.674
ENSMUSG00000046491	C1qtnf2	protein_coding	1.627	8.537	0.888
94.848	0.689	9.563	0.787	84.19
ENSMUSG00000046493	Olfr1352	protein_coding	1	85.283	1
96.847	NA	NA	1	95.877
ENSMUSG00000046500	Fam19a4	protein_coding	1	85.203	1
96.138	1	85.935	1	83.067
ENSMUSG00000046516	Cox17	protein_coding	1.129	10.464	0.903
66.651	0.976	77.037	0.988	90.611
ENSMUSG00000046518	Ferd3l	protein_coding	1	85.203	1
95.211	1	74.179	1	95.92
ENSMUSG00000046519	Golph3l	protein_coding	1.153	1.441	0.915
85.418	0.944	4.23	1.043	90.455
ENSMUSG00000046523	Kctd4	protein_coding	0.937	58.56	1.154	81.54
0.927	62.791	0.974	86.991
ENSMUSG00000046532	Ar	protein_coding	1.161	39.851	1.024
90.092	0.843	14.262	0.986	95.676
ENSMUSG00000046541	Zfp526	protein_coding	1.058	40.333
1.067	79.864	0.886	9.573	0.991	93.612
ENSMUSG00000046546	Fam43a	protein_coding	1.07	83.169

1.015 96.68 0.922 66.753 1.017 96.248
ENSMUSG00000046548 4731417B20Rik protein_coding 1 83.523
1 96.216 1 63.801 1 96.183
ENSMUSG00000046550 Spin2c protein_coding 0.935 85.499
0.937 93.654 1.143 70.534 1.215 91.011
ENSMUSG00000046556 Zfp319 protein_coding 0.995 81.196
1.037 83.104 0.968 28.901 0.984 86.699
ENSMUSG00000046561 Arsj protein_coding 1.263 51.297 1.074
96.763 0.736 49.733 1.255 50.452
ENSMUSG00000046562 Unc119b protein_coding 1.054 22.205
0.974 75.493 0.975 74.535 0.996 96.184
ENSMUSG00000046567 4930430F08Rik protein_coding 0.934 18.907
0.897 95.504 1.194 28.979 1.027 96.192
ENSMUSG00000046572 Zfp518b protein_coding 1.015 72.85 1.015
97.155 0.972 59.125 1.048 90.674
ENSMUSG00000046573 Lyrm4 protein_coding 1.131 12.985 1.1
84.832 0.805 8.634 1 88.755
ENSMUSG00000046574 Prr12 protein_coding 0.966 28.804 1.129
71.617 0.916 24.523 0.996 84.29
ENSMUSG00000046575 Olfr1389 protein_coding 1 86.658 1
87.857 1 83.158 1 94.297
ENSMUSG00000046580 Gm7862 pseudogene 1 83.263 1
92.561 1 71.004 1 95.273
ENSMUSG00000046585 Ccdc147 protein_coding 1 85.203 1
96.68 1 72.853 1 93.457
ENSMUSG00000046589 Lrrc8e protein_coding 0.852 52.726
1.004 95.504 1.172 62.237 0.943 92.181
ENSMUSG00000046590 Olfr1165-ps pseudogene 1 84.534 1
97.155 1 78.293 1 95.832
ENSMUSG00000046591 Ticrr protein_coding 1.28 52.799 0.781
75.345 0.996 86.727 1.537 55.841
ENSMUSG00000046593 Tmem215 protein_coding 0.967 55.939 NA
NA 1.144 70.1 1.343 40.024
ENSMUSG00000046598 Bdh1 protein_coding 0.895 53.407 0.817
64.624 1.362 21.873 1.217 88.755
ENSMUSG00000046603 Tcaim protein_coding 0.889 14.403 1.028
83.104 1.091 53.882 1.003 95.74
ENSMUSG00000046605 B3gntl1 protein_coding 1.001 59.358
0.835 62.025 1.192 6.693 1.096 72.116
ENSMUSG00000046607 Hrk protein_coding 1.028 67.163 0.809
71.98 1.205 58.915 1.092 90.397
ENSMUSG00000046610 Oacyl protein_coding 0.787 27.202 1.226
93.574 1.04 51.851 1.001 92.018
ENSMUSG00000046613 Vwa5b2 protein_coding 0.594 80.51 1.941
90.116 0.869 83.607 1.001 85.493
ENSMUSG00000046615 Actrt1 protein_coding 1 86.74 NA NA
1 88.729 1 92.181
ENSMUSG00000046618 Olfml2a protein_coding 1.509 1.395 0.906
81.134 0.729 5.323 0.93 87.992
ENSMUSG00000046620 Rps11-ps4 pseudogene 1 84.977 1
92.545 1 74.853 1 93.156
ENSMUSG00000046623 Gjb4 protein_coding 0.619 12.309 1.376
83.104 1.174 45.449 1.115 96.287
ENSMUSG00000046636 Gm7729 protein_coding 1 85.283 1

97.155	1	76.754	1	92.381
ENSMUSG00000046643	Olfr218	protein_coding	1	82.231	1
96.407	1	82.778	1	95.877
ENSMUSG00000046650	Olfr1440	protein_coding	1	80.688	1
96.843	1	85.757	1	96.183
ENSMUSG00000046658	Zfp316	protein_coding	0.894	23.319	0.97
95.211	1.153	43.129	0.949	83.724
ENSMUSG00000046667	Rbm12b1	protein_coding	1.015	63.534
1.342	79.29	0.732	4.538	0.964	84.29
ENSMUSG00000046668	Cxxc5	protein_coding	1.059	51.826	1.042
71.98	0.903	7.329	1.028	83.067
ENSMUSG00000046671	Mtfr1l	protein_coding	0.97	76.968
1.022	83.464	1.009	54.521	1.002	96.086
ENSMUSG00000046675	Tmem251	protein_coding	1.095	37.414
0.991	86.793	0.923	18.965	0.949	93.156
ENSMUSG00000046676	Lce1l	protein_coding	1	81.657	1
97.155	1	86.602	1	94.608
ENSMUSG00000046678	Olfr981	protein_coding	1	85.77	1
96.216	1	88.729	1	92.181
ENSMUSG00000046679	C87436	protein_coding	1.002	76.827
1.032	97.263	0.965	63.075	0.975	93.612
ENSMUSG00000046683	0610025J13Rik	antisense	0.762	27	1.624
82.904	0.809	86.112	0.999	94.904
ENSMUSG00000046687	Gm5424	protein_coding	1	84.324	NA
NA	1	87.686	1	95.74
ENSMUSG00000046688	Tifa	protein_coding	1.299	4.228	1.022	94.597
0.751	26.057	0.892	91.372
ENSMUSG00000046691	Chtf8	protein_coding	0.968	75.115	1.143
78.494	0.899	49.453	1.036	72.826
ENSMUSG00000046694	Fam46b	protein_coding	1.13	56.907
1.128	88.923	0.786	29.765	0.903	92.181
ENSMUSG00000046697	Enpp7	protein_coding	1	80.03	1	96.268
1	79.325	1	95.74
ENSMUSG00000046699	Slitrk4	protein_coding	0.658	14.221
0.894	91.83	1.704	17.751	0.976	90.95
ENSMUSG00000046707	Csnk2a2	protein_coding	1.085	10.687
0.934	39.056	0.988	71.615	0.997	83.067
ENSMUSG00000046709	Mapk10	protein_coding	1.43	64.956
1.008	95.088	0.695	59.383	1.396	77.621
ENSMUSG00000046711	Hmga1	protein_coding	1.042	56.907	0.996
90.576	0.968	83.857	0.971	86.699
ENSMUSG00000046714	Foxc2	protein_coding	1.129	75.196	1.222
83.104	0.726	28.669	0.901	92.181
ENSMUSG00000046716	Vmn1r67	protein_coding	1	72.372	1
96.68	1	86.346	1	96.071
ENSMUSG00000046717	Igbp1b	protein_coding	1	81.925	1
96.407	1	88.693	1	93.584
ENSMUSG00000046718	Bst2	protein_coding	1.186	23.254	1.139
89.014	0.74	52.972	1.177	46.412
ENSMUSG00000046719	Nxph3	protein_coding	1.236	10.464	1.201
96.138	0.672	21.331	2.275	48.177
ENSMUSG00000046722	Cdc42se1	protein_coding	1.021	29.057
1.056	80.678	0.927	7.509	1.041	76.467
ENSMUSG00000046723	Adam24	protein_coding	1	61.334	1

91.361 1 45.208 1 70.36
ENSMUSG00000046727 Cystm1 protein_coding 1.061 55.789
0.916 90.138 1.026 86.084 1.079 92.018
ENSMUSG00000046731 Kctd11 protein_coding 1.027 84.564
1.075 83.104 0.903 20.258 1.096 59.441
ENSMUSG00000046733 Gprc5a protein_coding 1.05 86.464
0.773 71.889 1.228 30.561 1.075 92.742
ENSMUSG00000046743 Fat4 protein_coding 1.271 12.985 0.914
86.538 0.86 6.858 0.931 84.29
ENSMUSG00000046747 Gm9812 pseudogene 0.795 18.795 0.98
96.68 1.285 45.035 0.962 95.877
ENSMUSG00000046748 2310005G13Rik protein_coding 4.298 17.492
0.387 68.115 0.599 53.7 0.567 90.805
ENSMUSG00000046750 BC089491 protein_coding 0.812 84.324
1.712 95.088 0.717 88.161 0.66 87.331
ENSMUSG00000046753 Ccdc66 protein_coding 1.026 50.627
1.029 96.407 0.943 40.338 1.063 79.268
ENSMUSG00000046755 Kif2b protein_coding 1 83.658 NA NA
1 86.258 1 96.358
ENSMUSG00000046756 Mrps7 protein_coding 0.838 1.15 1.085 81.54
1.098 20.572 1.023 90.674
ENSMUSG00000046761 Fam83h protein_coding 0.898 24.344 NA
NA 1.12 44.515 0.934 83.724
ENSMUSG00000046764 A530053G22Rik protein_coding 0.889 39.712
1.257 60.309 0.894 7.329 0.94 86.699
ENSMUSG00000046768 Rhoj protein_coding 0.947 46.413 1.044
96.138 1.008 55.512 0.98 96.248
ENSMUSG00000046782 Ttc6 protein_coding 0.835 13.199 0.901
95.504 1.334 29.302 0.884 83.724
ENSMUSG00000046785 Epm2aip1 protein_coding 0.943 23.13 1.045
92.931 1.016 62.648 0.957 94.99
ENSMUSG00000046787 Gm9814 protein_coding 1 85.728 1
96.138 1 85.134 1 92.12
ENSMUSG00000046790 Olfr1341 protein_coding 1 86.843 1
95.708 1 81.62 1 95.72
ENSMUSG00000046791 2410016O06Rik protein_coding 0.961 85.77
1.013 94.848 1.029 86.258 1.01 93.264
ENSMUSG00000046792 Zfp787 protein_coding 0.889 38.983
0.986 92.355 1.144 32.776 1.038 95.74
ENSMUSG00000046794 Ppp1r3b protein_coding 0.75 57.014
1.198 84.126 1.111 87.103 1.09 92.52
ENSMUSG00000046798 Cldn12 protein_coding 0.956 74.299
0.935 95.941 1.114 79.897 1.009 96.086
ENSMUSG00000046804 Phgr1 protein_coding 1 78.744 1
96.68 1 81.492 1 96.042
ENSMUSG00000046805 Mpeg1 protein_coding 1.514 0 0.848 56.955
0.78 8.907 0.866 88.387
ENSMUSG00000046806 3110062M04Rik protein_coding 0.845 16.02 1.01
95.431 1.173 28.997 0.944 90.157
ENSMUSG00000046807 Fam211b protein_coding 1.091 25.735
1.093 86.156 0.835 20.751 0.986 90.805
ENSMUSG00000046808 Atp10d processed_transcript 1.045 84.324
0.851 67.513 1.125 25.966 0.987 95.877
ENSMUSG00000046814 Gchfr protein_coding 1.004 71.915 1.189

83.819 0.836 57.446 0.91 95.92
ENSMUSG00000046818 Ddit4l protein_coding 2.294 10.595
1.991 71.617 0.218 1.48 0.77 96.358
ENSMUSG00000046822 Slc39a3 protein_coding 1.014 80.815
1.019 83.104 0.97 41.154 1.035 88.755
ENSMUSG00000046826 Fam187b protein_coding 1.159 82.352 NA
NA 0.961 74.179 0.609 84.29
ENSMUSG00000046834 Krt1 protein_coding 0.574 33.906 1.019
86.071 1.704 26.663 1.251 93.264
ENSMUSG00000046836 Brox protein_coding 1.072 7.083 1.012 94.767
0.923 20.009 1.029 84.31
ENSMUSG00000046841 Ckap4 protein_coding 1.102 30.282 NA NA
0.884 27.675 1.369 31.133
ENSMUSG00000046844 Vat1l protein_coding 0.803 10.903 1.19
82.904 1.045 58.414 1.019 87.7
ENSMUSG00000046845 Il1f10 protein_coding 1 64.893 1
97.155 1 67.962 1 96.086
ENSMUSG00000046846 Spesp1 protein_coding 1 81.01 NA NA
1 80.089 1 93.457
ENSMUSG00000046854 Pip5kl1 protein_coding 1.116 69.777
1.926 86.277 0.466 36.786 0.914 92.851
ENSMUSG00000046856 Gpr1 protein_coding 1.029 86.474 0.757
65.819 1.279 17.279 0.933 95.593
ENSMUSG00000046858 C230030N03Rik pseudogene 1 85.728 NA
NA 1 84.308 1 96.358
ENSMUSG00000046861 Hectd3 protein_coding 0.946 24.344
1.031 87.185 1.025 63.665 0.975 87.331
ENSMUSG00000046862 Pramef8 protein_coding 1.081 13.8 0.976
64.123 0.948 83.158 1 94.016
ENSMUSG00000046865 Fbl protein_coding 0.96 63.126 0.963
82.848 1.084 47.512 1.021 80.187
ENSMUSG00000046873 Mbtps2 protein_coding 1.072 9.805 0.974
66.09 0.954 87.519 0.97 93.045
ENSMUSG00000046876 Atxn1 protein_coding 0.924 58.264 1.063
90.68 1.02 79.325 1.071 78.379
ENSMUSG00000046879 Irgm1 protein_coding 1.194 7.501 1.165 79.261
0.719 7.742 1.092 59.464
ENSMUSG00000046881 Olfr374 protein_coding 1 80.03 1
96.216 1 86.666 1 95.273
ENSMUSG00000046886 Zfp474 protein_coding 1 85.499 1
97.097 1 79.426 1 93.132
ENSMUSG00000046897 Zfp740 protein_coding 0.933 1.274 0.905
41.951 1.184 5.864 0.955 92.181
ENSMUSG00000046899 Prl7a2 protein_coding 1 84.738 1
93.654 1 86.144 1 96.287
ENSMUSG00000046908 Ltb4r1 protein_coding 1.136 58.709
1.074 95.708 0.819 72.269 0.923 96.248
ENSMUSG00000046909 Tefm protein_coding 0.924 38.983 0.95
87.147 1.136 25.144 1.118 51.908
ENSMUSG00000046913 Olfr1451 protein_coding 1 77.763 NA
NA 1 85.935 1 94.752
ENSMUSG00000046916 Myct1 protein_coding 1.088 24.689 1.228
83.104 0.749 2.563 0.874 84.29
ENSMUSG00000046922 Gpr6 protein_coding 1 79.068 1

95.196 1 83.857 1 95.74
ENSMUSG00000046924 Vmn1r179 protein_coding 1 83.263 1
97.155 1 88.349 1 95.74
ENSMUSG00000046932 Vmn1r193 protein_coding 1 84.865 1
94.597 1 82.903 1 96.358
ENSMUSG00000046942 Mageb16 protein_coding 1 86.658 1
95.196 1 76.768 1 92.018
ENSMUSG00000046947 Adck2 protein_coding 0.96 38.983 0.966
93.046 1.079 36.011 0.955 88.053
ENSMUSG00000046949 Nqo2 protein_coding 1.027 25.735 NA NA
0.956 25.512 1.234 0
ENSMUSG00000046952 Gm5815 pseudogene 1 85.857 1
96.308 1 86.831 1 93.264
ENSMUSG00000046957 Spz1 protein_coding 1 83.726 NA NA
1 87.336 1 94.016
ENSMUSG00000046959 Slc26a1 protein_coding 0.904 59.674 1.23
83.104 0.897 86.084 0.984 95.832
ENSMUSG00000046961 Gpr156 protein_coding 1.537 34.696
0.967 96.843 0.673 31.135 1.334 57.075
ENSMUSG00000046962 Zbtb21 protein_coding 1.007 79.433
1.041 79.411 0.952 48.387 1.008 96.287
ENSMUSG00000046971 Pla2g4f protein_coding 3.22 46.533
1.026 96.763 0.302 52.658 0.601 92.675
ENSMUSG00000046974 BC053393 protein_coding 1 84.324 NA
NA 1 84.552 1 94.904
ENSMUSG00000046975 Olfr1020 protein_coding 1 72.372 1
96.216 1 84.552 1 96.248
ENSMUSG00000046980 Pcdh15 pseudogene 1 67.991 1
96.216 1 86.144 1 93.722
ENSMUSG00000046982 Tshz1 protein_coding 0.998 57.655 0.992
95.35 1.013 70.44 0.935 83.724
ENSMUSG00000046985 Tapt1 protein_coding 1.19 1.009 NA NA
0.846 1.898 0.953 90.674
ENSMUSG00000046991 Wdr27 protein_coding 0.844 70.213 0.824
91.661 1.438 41.806 1.038 93.316
ENSMUSG00000046993 Gm5637 pseudogene 1 86.006 1
92.355 1 88.203 1 95.877
ENSMUSG00000046994 Mars2 protein_coding 1.091 63.385 1.022
95.504 0.894 58.775 0.849 84.29
ENSMUSG00000046995 2610303G11Rik protein_coding 1 82.732
1 96.138 1 87.898 1 95.74
ENSMUSG00000046997 Spsb4 protein_coding 0.735 20.659 1.391
82.904 0.98 79.554 1.268 81.061
ENSMUSG00000046999 1110032F04Rik protein_coding 3.196 46.875
0.251 90.378 1.25 76.768 1.875 83.581
ENSMUSG00000047002 Msgn1 protein_coding 1 72.85 1 94.48 1
78.954 1 94.99
ENSMUSG00000047003 Zfp41 protein_coding 1.106 20.296 0.939
75.052 0.964 88.729 0.946 86.699
ENSMUSG00000047011 Map4 pseudogene 1 86.474 1 96.763
1 84.308 1 96.183
ENSMUSG00000047013 Fbxo41 protein_coding 0.621 39.174
1.273 92.931 1.262 65.235 1.313 95.877
ENSMUSG00000047014 Catsperb protein_coding 1 69.715 1

96.138 NA NA 1 96.086
ENSMUSG00000047022 Mipol1 protein_coding 1.109 51.737 1
93.654 0.898 69.974 0.994 95.559
ENSMUSG00000047025 Ccer1 protein_coding 1 74.958 NA NA
1 86.112 1 96.183
ENSMUSG00000047026 Acsm4 protein_coding 1 81.465 1
97.263 1 86.727 1 91.492
ENSMUSG00000047030 Spata2 protein_coding 0.969 69.943
1.194 40.99 0.867 7.339 1.004 95.559
ENSMUSG00000047031 Vmn1r68 protein_coding 1 82.479 1
95.708 1 88.349 1 95.405
ENSMUSG00000047033 Pcdhb15 protein_coding 0.924 82.479
1.092 95.809 0.99 86.112 1.083 95.273
ENSMUSG00000047036 Zfp445 protein_coding 0.898 29.725 0.94
79.475 1.186 11.782 1.069 92.018
ENSMUSG00000047037 Nipa1 protein_coding 1.112 2.352 NA NA
0.818 12.571 1.06 95.273
ENSMUSG00000047039 Olfr1151 protein_coding 1 75.399 1
94.96 1 78.498 1 96.184
ENSMUSG00000047040 Prr15l protein_coding 0.843 19.365
0.811 60.678 1.46 5.864 0.858 91.979
ENSMUSG00000047044 D030056L22Rik protein_coding 1.009 65.733
1.057 84.126 0.939 28.669 0.958 86.699
ENSMUSG00000047045 Tmem164 protein_coding 1.021 61.49 0.98
92.404 0.997 78.642 1.078 91.335
ENSMUSG00000047046 1700031F10Rik antisense 1 74.958 1
96.847 1 58.516 1 90.611
ENSMUSG00000047048 Olfr432 protein_coding 1 81.465 1
85.921 1 61.743 1 95.676
ENSMUSG00000047050 Olfr914 protein_coding 1 84.324 NA
NA 1 84.071 1 96.183
ENSMUSG00000047053 Teddm1 protein_coding 1 79.165 1
96.843 1 88.601 1 95.74
ENSMUSG00000047067 Dusp28 protein_coding 1.016 80.608
1.021 94.767 0.966 84.552 0.889 85.493
ENSMUSG00000047079 Ube2dnl2 protein_coding 1 84.661 1
92.404 1 80.899 1 96.184
ENSMUSG00000047084 Ngrn protein_coding 0.948 57.544 0.978
67.941 1.078 10.819 1.023 95.35
ENSMUSG00000047085 Lrrc4b protein_coding 0.978 71.217
0.889 84.594 1.144 88.693 1.022 96.183
ENSMUSG00000047090 Tmem198b protein_coding 1.038 69.408
0.932 87.452 1.036 85.792 1.101 60.466
ENSMUSG00000047094 Ofcc1 protein_coding 0.898 76.686 1.696
82.904 0.658 55.214 0.873 96.183
ENSMUSG00000047098 Rnf31 protein_coding 0.987 33.906 1.013
78.494 1.001 83.811 0.984 94.99
ENSMUSG00000047102 Tas2r139 protein_coding 1 83.263 1
96.138 NA NA 1 93.264
ENSMUSG00000047104 Pbp2 protein_coding 1 78.054 1
95.088 1 88.601 1 82.328
ENSMUSG00000047108 Dnajb7 protein_coding 1.536 81.01 0.639
93.094 1.015 81.29 1.095 88.111
ENSMUSG00000047115 Fam221a protein_coding 0.897 54.043

0.921 69.611 1.214 27.047 0.857 86.699
ENSMUSG00000047116 9430041J12Rik pseudogene 1 86.658 1
96.843 NA NA 1 96.358
ENSMUSG00000047117 Ankdd1b protein_coding 1.122 77.916
0.848 82.848 1.046 70.9 0.81 91.291
ENSMUSG00000047123 Ticam1 protein_coding 1.004 86.351
0.974 73.166 1.024 40.511 1.106 31.133
ENSMUSG00000047126 Cltc protein_coding 1.067 7.908 0.971 85.116
0.962 32.297 1.069 62.594
ENSMUSG00000047139 Cd24a protein_coding 1.22 22.205 1.068
96.68 0.763 23.212 1.133 75.81
ENSMUSG00000047141 Zfp654 protein_coding 1.102 5.657 0.95
82.947 0.952 67.962 0.992 90.157
ENSMUSG00000047143 Dmrta2 protein_coding 0.746 29.21 0.895
96.68 1.495 50.636 1 96.086
ENSMUSG00000047146 Tet1 protein_coding 0.958 56.477 1.023
93.529 1.018 84.229 1.036 92.181
ENSMUSG00000047149 Olfr1052 protein_coding 1 72.743 1
95.708 1 80.015 1 95.273
ENSMUSG00000047150 1700001C19Rik protein_coding 0.769 34.954
1.05 96.847 1.241 37.369 0.818 87.735
ENSMUSG00000047153 Khnyn protein_coding 1.004 67.163 0.967
94.96 1.026 65.235 0.977 85.493
ENSMUSG00000047161 Chst9 protein_coding 2.68 43.019 0.588
91.532 0.632 59.383 0.629 91.105
ENSMUSG00000047163 Olfr65 protein_coding 1 83.169 1
88.691 NA NA 1 95.877
ENSMUSG00000047166 Tigd5 pseudogene 0.838 9.508 1.073 95.35 1.113
17.662 1.012 90.177
ENSMUSG00000047168 Gm6684 pseudogene 1.429 68.485 0.472
75.618 1.48 66.647 0.646 89.892
ENSMUSG00000047171 Helt protein_coding 1 72.27 1 95.329
1 78.713 1 90.674
ENSMUSG00000047180 Neurl3 protein_coding 1.306 29.536
0.942 85.116 0.811 68.584 0.633 83.724
ENSMUSG00000047181 Samd14 protein_coding 0.993 60.339
0.931 94.102 1.078 48.935 0.971 87.651
ENSMUSG00000047182 Irs3 protein_coding 1.004 83.333 1.336
79.367 0.745 12.571 0.964 84.29
ENSMUSG00000047187 Rab2a protein_coding 1.033 79.277 0.945
82.671 1.027 62.648 1.04 59.464
ENSMUSG00000047189 Gm9818 pseudogene 1 74.021 1
96.843 1 84.504 1 96.287
ENSMUSG00000047193 Dync2h1 protein_coding 0.93 43.435 NA
NA 1.077 39.471 0.902 90.854
ENSMUSG00000047203 Vmn1r54 protein_coding 1 86.74 1
95.211 1 85.58 1 94.99
ENSMUSG00000047205 Dusp18 protein_coding 1 74.482
0.938 87.313 1.064 62.995 0.964 93.734
ENSMUSG00000047207 Olfr1495 protein_coding 1 83.418 1
95.708 1 86.552 1 95.72
ENSMUSG00000047213 Ythdf3 protein_coding 1.038 10.595
1.006 96.897 0.959 13.643 1.006 90.805
ENSMUSG00000047215 Rpl9 protein_coding 1.09 8.996 0.899 12.804

1.018 88.41 1.024 94.016
ENSMUSG00000047216 Cdh19 protein_coding 0.904 56.768 NA NA
1.058 68.129 1.04 65.401
ENSMUSG00000047221 Fam185a protein_coding 1.02 62.574
0.995 96.216 0.984 46.575 0.95 87.542
ENSMUSG00000047222 Ear11 protein_coding 2.577 37.091 0.131
55.265 2.953 39.925 2.548 78.042
ENSMUSG00000047225 Olfr684 protein_coding 1 84.661 1
95.504 NA NA 1 96.183
ENSMUSG00000047227 Gm527 protein_coding 1.171 21.599 0.888
86.604 0.962 38.799 0.928 94.654
ENSMUSG00000047228 BC048546 protein_coding 1 75.858 1
96.335 1 79.426 1 91.492
ENSMUSG00000047230 Cldn2 protein_coding 0.547 18.731 1.101
92.793 1.654 43.586 0.832 84.29
ENSMUSG00000047237 Fbxw21 protein_coding 1 86.918 1
95.708 1 84.504 1 96.183
ENSMUSG00000047238 Mageh1 protein_coding 1.037 67.163
1.085 90.116 0.89 44.865 1.008 86.445
ENSMUSG00000047242 Taf9b protein_coding 1.166 22.205 1.028
81.127 0.833 4.48 0.956 90.628
ENSMUSG00000047246 Hist1h2be protein_coding 0.813 11.973
1.143 83.104 1.074 78.864 0.934 89.525
ENSMUSG00000047248 C2cd3 protein_coding 0.934 3.513 1.05 74.663
1.019 46.575 0.903 83.724
ENSMUSG00000047250 Ptgs1 protein_coding 1.119 1.323 0.949 68.818
0.939 68.584 1.1 77.331
ENSMUSG00000047253 Krtap1-5 protein_coding 1 86.57 1
97.129 1 78.954 1 93.685
ENSMUSG00000047259 Mc4r protein_coding 1 84.397 1
95.211 1 76.225 1 95.273
ENSMUSG00000047260 Emc6 protein_coding 0.929 14.221 1.122
75.108 0.958 88.076 0.98 90.289
ENSMUSG00000047261 Gap43 protein_coding 1.362 26.863 0.826
85.95 0.888 67.029 1.004 92.018
ENSMUSG00000047264 Zfp358 protein_coding 0.967 84.263 NA
NA 1.011 84.504 0.958 85.493
ENSMUSG00000047281 Sfn protein_coding 0.754 54.87 NA NA 1.23
38.683 1.068 93.261
ENSMUSG00000047284 Neurl4 protein_coding 0.89 0.987 1.006
92.561 1.116 5.864 0.98 92.31
ENSMUSG00000047286 Olfr370 protein_coding 1 84.452 1
90.287 1 88.461 1 95.74
ENSMUSG00000047295 Smgc protein_coding 1 86.464 1
95.708 1 83.275 1 94.904
ENSMUSG00000047298 Kcnv2 protein_coding 1.686 21.836 1.144
90.895 0.52 13.848 1.246 83.067
ENSMUSG00000047307 Pcdhb13 protein_coding 0.847 47.174
1.226 82.904 0.965 78.713 0.986 92.564
ENSMUSG00000047330 Kcne4 protein_coding 1.106 29.372 0.941
83.876 0.962 87.898 0.935 86.445
ENSMUSG00000047342 Zfp286 protein_coding 1.072 82.479 NA
NA 0.908 77.532 0.996 96.248
ENSMUSG00000047344 Lancl3 protein_coding 0.798 57.544

1.214 97.263 1.029 68.129 1.107 96.358
ENSMUSG00000047347 Gm5806 pseudogene 1 77.182 NA NA
1 86.346 1 92.748
ENSMUSG00000047352 Olfr976 protein_coding 1 84.865 1
93.574 1 79.625 1 95.92
ENSMUSG00000047356 Lcn10 protein_coding 1 71.36 1 96.407
1 88.657 1 94.82
ENSMUSG00000047361 Gm973 protein_coding 1.898 2.969 0.785 94.597
0.671 5.042 0.758 83.724
ENSMUSG00000047363 Cstad protein_coding 1.357 52.429 0.687
92.137 1.072 87.336 0.501 83.724
ENSMUSG00000047368 Abhd17b protein_coding 1.074 47.174
1.022 87.029 0.907 15.904 0.974 85.493
ENSMUSG00000047370 Gm7367 protein_coding 1 82.732 1
96.68 1 78.369 1 94.904
ENSMUSG00000047371 Zfp768 protein_coding 0.958 57.818
1.025 94.367 1.021 84.83 1.064 93.016
ENSMUSG00000047379 B3gnt1 protein_coding 1.013 72.27 1.008
92.111 0.979 60.96 1.073 57.075
ENSMUSG00000047383 Als2cr11 protein_coding 1 85.391 1
95.708 1 83.719 1 96.358
ENSMUSG00000047384 A730013G03Rik protein_coding 1 83.09 NA
NA 1 88.076 1 86.301
ENSMUSG00000047388 Atmin protein_coding 1.022 62.689 0.965
83.58 1.008 69.315 0.978 94.99
ENSMUSG00000047390 Defb9 protein_coding 1 77.407 1
94.848 1 82.778 1 95.72
ENSMUSG00000047394 Odf3b protein_coding 1.091 78.639 1.014
96.216 0.902 86.985 1 95.832
ENSMUSG00000047407 Tgif1 protein_coding 1.043 46.097 0.915
47.716 1.049 40.575 0.813 85.493
ENSMUSG00000047409 Ctdspl protein_coding 0.91 35.894 0.95
93.654 1.153 45.035 1.102 61.984
ENSMUSG00000047412 Zbtb44 protein_coding 1.041 46.097
0.997 94.597 0.96 67.339 0.91 83.724
ENSMUSG00000047414 Flrt2 protein_coding 1.183 8.477 0.931 94.887
0.905 29.217 1.063 79.442
ENSMUSG00000047415 Gpr68 protein_coding 1.4 8.996 0.968 90.256
0.738 43.842 0.894 83.724
ENSMUSG00000047417 Rexo1 protein_coding 0.891 2.009 1.017 93.574
1.107 7.482 0.984 95.832
ENSMUSG00000047419 Cmya5 protein_coding 3.657 6.135 1.053 83.104
0.26 0.72 0.901 95.35
ENSMUSG00000047420 Fam180a protein_coding 1.028 71.763
0.776 85.242 1.251 50.636 0.697 85.493
ENSMUSG00000047423 AI837181 protein_coding 0.945 67.334
1.147 81.039 0.92 41.506 0.965 83.724
ENSMUSG00000047428 Dlk2 protein_coding 0.821 40.422 0.765
84.017 1.59 25.144 1.066 83.067
ENSMUSG00000047434 Xxylt1 protein_coding 1.104 0.461 NA NA
0.911 1.48 1.033 92.762
ENSMUSG00000047443 Fam132b protein_coding 0.726 86.918
0.862 91.661 1.595 82.778 2.089 83.276
ENSMUSG00000047444 Olfr139 protein_coding 1 81.01 1

97.263 1 85.935 1 95.35
ENSMUSG00000047446 Arl4a protein_coding 1.124 46.097 0.77
67.839 1.153 68.129 1.013 96.071
ENSMUSG00000047454 Gphn protein_coding 0.958 65.388 0.988
91.83 1.058 33.508 0.973 88.505
ENSMUSG00000047459 Dynlrb1 protein_coding 0.957 80.751
0.966 93.908 1.082 70.623 1.096 83.276
ENSMUSG00000047462 A530099J19Rik protein_coding 1.218 24.253
0.922 93.654 0.888 48.577 1.073 93.016
ENSMUSG00000047466 8030462N17Rik protein_coding 1.075 2.723
1.025 96.216 0.907 3.234 0.944 83.724
ENSMUSG00000047473 Zfp30 protein_coding 0.989 85.77 1.047 93.899
0.966 87.336 0.957 91.625
ENSMUSG00000047485 Klhl34 protein_coding 1.605 50.627
0.656 90.378 0.946 87.173 1.064 93.457
ENSMUSG00000047490 4932411E22Rik protein_coding 0.82 75.115
NA NA 0.986 85.921 0.942 96.192
ENSMUSG00000047492 Inhbe protein_coding 1 69.408 1
96.216 1 80.749 1 94.608
ENSMUSG00000047496 Rnf152 protein_coding 1.063 58.709
1.084 81.039 0.868 16.409 0.868 83.724
ENSMUSG00000047497 Adamts12 protein_coding 1.096 20.296
0.916 54.427 0.996 86.112 1.142 92.903
ENSMUSG00000047501 Cldn4 protein_coding 0.804 39.712 NA NA
1.174 30.948 0.942 93.316
ENSMUSG00000047502 Mroh7 protein_coding 0.877 71.36 0.617 55.71
1.839 21.873 0.966 93.612
ENSMUSG00000047507 Baiap3 protein_coding 1.132 24.958
0.837 79.894 1.05 80.015 1.131 85.577
ENSMUSG00000047508 Mbd3l2 protein_coding 1 86.145 1
96.763 1 85.464 1 93.964
ENSMUSG00000047509 Gm6776 pseudogene 1 82.732 1
96.847 NA NA 1 96.358
ENSMUSG00000047511 Olfr1396 protein_coding 1.238 50.913
1.154 97.263 0.698 44.211 0.805 86.699
ENSMUSG00000047514 Tspyl1 protein_coding 1.062 50.627
0.996 83.104 0.946 7.155 0.948 87.496
ENSMUSG00000047515 BC049715 protein_coding 0.702 61.633 NA
NA 1.241 70.328 1.659 53.923
ENSMUSG00000047517 Dmbt1 protein_coding 0.492 4.385 1.376 71.98
1.478 67.163 1.015 92.381
ENSMUSG00000047518 Slfnl1 protein_coding 1.004 84.294
0.764 96.138 1.304 79.325 0.756 94.904
ENSMUSG00000047528 Als2cr12 protein_coding 0.958 69.985
1.255 82.904 0.83 39.245 0.86 86.991
ENSMUSG00000047531 Rtp2 protein_coding 1 56.907 NA NA
1 77.532 1 96.183
ENSMUSG00000047534 Mis18bp1 protein_coding 1.542 48.288
0.722 58.64 NA NA 1.09 73.823
ENSMUSG00000047535 Olfr67 protein_coding 1 84.977 1
95.708 1 79.524 1 96.287
ENSMUSG00000047539 Fbxo28 protein_coding 1.044 13.363
1.029 86.156 0.93 7.399 0.975 86.991
ENSMUSG00000047544 Olfr616 protein_coding 1 68.727 1

95.211 1 87.898 1 96.183
ENSMUSG00000047545 Olfr629 protein_coding 1 81.01 1
94.48 1 86.084 1 96.086
ENSMUSG00000047547 Cltb protein_coding 0.965 74.299 NA NA
1.01 76.923 1.008 71.653
ENSMUSG00000047554 Tmem41b protein_coding 0.995 72.27 1.054
78.705 0.955 19.552 1.015 94.174
ENSMUSG00000047557 Lxn protein_coding 1.028 44.694 1.007
95.329 0.963 80.5 1.064 67.169
ENSMUSG00000047562 Mmp10 protein_coding 1 82.982 NA NA
1 81.388 1 90.611
ENSMUSG00000047564 Krtap3-1 protein_coding 1 83.418 1
96.216 1 84.724 1 94.904
ENSMUSG00000047565 Acot10 protein_coding 1 82.891 1
92.631 NA NA 1 93.261
ENSMUSG00000047583 Tyw3 protein_coding 0.961 71.674 0.987
96.268 1.051 79.426 1.058 94.904
ENSMUSG00000047586 Nccrp1 protein_coding 1.387 56.66 0.604
90.576 1.191 86.552 0.865 90.628
ENSMUSG00000047591 Mafa protein_coding 2.009 5.313 1.032 90.389
0.482 1.061 0.879 95.92
ENSMUSG00000047592 Nxpe5 protein_coding 1.392 2.233 0.906 78.877
0.795 21.331 1.252 69.918
ENSMUSG00000047594 Olfr1122 protein_coding 1 79.709 1
96.407 1 86.985 1 96.139
ENSMUSG00000047603 Zfp235 protein_coding 1.007 86.819
1.017 95.504 0.977 84.504 1.053 78.042
ENSMUSG00000047604 Frat2 protein_coding 0.878 40.217 0.961
85.418 1.182 17.751 1.008 96.287
ENSMUSG00000047613 A430005L14Rik protein_coding 1.009 81.657
1.026 92.561 0.967 84.434 1.011 79.545
ENSMUSG00000047617 BC029214 protein_coding 0.865 3.325 1.066
90.454 1.084 18.797 1.018 81.251
ENSMUSG00000047619 Ddi1 protein_coding 1 79.165 1
96.847 1 78.713 1 95.273
ENSMUSG00000047626 Olfr791 protein_coding 1 84.324 1
95.088 1 82.31 1 95.273
ENSMUSG00000047631 Apof protein_coding 1.224 28.927 0.517
46.547 1.582 35.894 0.561 84.29
ENSMUSG00000047632 Fgfbp3 protein_coding 0.92 86.918
1.219 96.216 0.889 84.071 1.036 94.153
ENSMUSG00000047635 2810006K23Rik protein_coding 0.98 86.333
1.034 93.574 0.989 84.308 1.041 75.198
ENSMUSG00000047638 Nr1h4 protein_coding 0.918 63.804 0.787
70.693 1.378 36.95 0.99 94.001
ENSMUSG00000047641 Krt83 protein_coding 0.945 56.907 0.758
68.818 1.401 62.237 1.094 95.593
ENSMUSG00000047643 Gm5454 pseudogene 1 85.728 1
94.48 1 83.811 1 96.183
ENSMUSG00000047648 Fbxo30 protein_coding 1.121 14.008
1.043 85.756 0.854 5.087 0.978 96.287
ENSMUSG00000047649 Cd3eap protein_coding 1.03 85.728
0.949 87.998 1.025 84.896 1.009 94.825
ENSMUSG00000047652 Pea15b pseudogene 1 83.726 1

96.68 1 74.082 1 96.042
ENSMUSG00000047654 Tssk6 protein_coding 0.569 23.641 1.12
96.843 1.568 37.259 1.026 94.608
ENSMUSG00000047655 Vmn1r74 protein_coding 1 75.761 1
96.763 1 84.308 1 94.904
ENSMUSG00000047656 Trpt1 protein_coding 0.956 66.129 1.073
94.367 0.976 88.729 0.958 86.991
ENSMUSG00000047657 Crry-ps pseudogene 1 84.049 NA NA
1 81.032 1 95.676
ENSMUSG00000047658 Gal3st3 protein_coding 0.899 78.639
0.655 82.073 1.693 57.286 0.919 95.877
ENSMUSG00000047661 1600012P17Rik protein_coding 1 75.567
1 93.765 1 84.961 1 93.612
ENSMUSG00000047667 Olfr26 protein_coding 1 86.658 1
95.504 1 79.625 1 91.872
ENSMUSG00000047669 Msl3l2 protein_coding 1.138 7.842 0.843
42.923 NA NA 1.004 92.018
ENSMUSG00000047671 Tctex1d4 protein_coding 1.272 81.76 0.765
82.848 1.026 57.227 0.786 90.691
ENSMUSG00000047674 Pdha2 protein_coding 1 77.763 1
95.708 1 86.831 1 92.018
ENSMUSG00000047675 Rps8 protein_coding 0.974 71.142 0.931
36.617 1.106 19.106 1.129 63.708
ENSMUSG00000047678 Gpr82 protein_coding 1.344 65.537 0.729
82.671 1.024 86.985 1.022 89.587
ENSMUSG00000047686 Zcchc5 protein_coding 1.66 13.492
1.029 96.68 0.587 15.63 0.834 94.904
ENSMUSG00000047692 4930533K18Rik protein_coding 1.003 81.657
NA NA 0.954 86.552 1.008 93.612
ENSMUSG00000047694 Yipf6 protein_coding 0.957 29.21 0.963 68.115
1.081 8.586 0.972 92.018
ENSMUSG00000047696 Ccdc144b protein_coding 0.734 7.887 1.323
67.23 1.03 83.857 0.741 85.493
ENSMUSG00000047710 Champ1 protein_coding 1.114 4.675 0.979
94.597 0.917 6.524 0.996 87.447
ENSMUSG00000047712 Ust protein_coding 1.094 48.288 0.989
92.111 0.923 29.451 1.018 94.904
ENSMUSG00000047714 Ppp1r2 protein_coding 1.089 1.292 1
95.35 0.913 3.969 0.949 83.724
ENSMUSG00000047716 Olfr736 protein_coding 1 80.751 1
96.652 1 77.294 1 95.273
ENSMUSG00000047719 Ubiad1 protein_coding 1.077 19.477
1.083 88.91 0.854 18.122 0.99 94.82
ENSMUSG00000047720 4922502D21Rik protein_coding 1 84.865
1 95.941 1 78.498 1 93.413
ENSMUSG00000047721 Bola2 protein_coding 0.904 13.45 1.091 95.431
1.015 31.317 0.905 95.832
ENSMUSG00000047728 BC025446 protein_coding 1 79.433 1
94.367 1 87.173 1 94.904
ENSMUSG00000047730 Fcgbp protein_coding 1.113 70.741 1.307
72.546 0.688 16.949 0.644 83.724
ENSMUSG00000047731 Wbp1l protein_coding 0.963 41.759 1.02
96.216 1.019 49.058 1.047 83.067
ENSMUSG00000047733 BC048609 protein_coding 0.891 54.983

0.942 87.029 1.188 76.64 0.961 93.612
ENSMUSG00000047735 Samd9l protein_coding 1.116 14.59 1.049
96.68 0.855 14.079 1.168 59.464
ENSMUSG00000047746 Fbxo40 protein_coding 5.345 8.685 1.167
83.104 0.16 0.72 1.15 93.457
ENSMUSG00000047747 Rnf150 protein_coding 0.978 63.804
0.974 85.793 NA NA 1.135 96.358
ENSMUSG00000047749 Zc3hav1l protein_coding 1.13 36.124 NA
NA 0.872 41.154 0.964 90.611
ENSMUSG00000047751 Utf1 protein_coding 0.545 79.709 0.88
92.474 2.091 52.8 1.033 92.748
ENSMUSG00000047757 Fancb protein_coding 1.048 49.517 1.177
95.211 0.81 22.009 1.037 92.742
ENSMUSG00000047759 Hs3st3a1 protein_coding 1.016 86.006
1.045 96.013 0.944 87.173 1.16 74.607
ENSMUSG00000047766 Lrrc49 protein_coding 1.158 16.539
0.904 74.851 0.957 74.853 0.96 90.157
ENSMUSG00000047767 Atg16l2 protein_coding 1.043 41.211
1.079 83.104 0.89 14.583 0.925 85.493
ENSMUSG00000047773 Ankfn1 protein_coding 0.855 40.217
1.085 96.68 1.081 49.601 1.291 79.268
ENSMUSG00000047777 Phf13 protein_coding 0.988 74.482 1.07
71.98 0.947 25.688 0.948 85.493
ENSMUSG00000047786 Lix1 protein_coding 1.259 37.941 0.928
95.809 0.858 27.194 0.92 92.675
ENSMUSG00000047787 Flrt1 protein_coding 0.74 38.983 1.272
92.793 1.061 81.857 0.912 96.192
ENSMUSG00000047789 Slc38a9 protein_coding 1.004 78.639
0.908 60.678 1.099 14.376 1.027 83.276
ENSMUSG00000047793 Sned1 protein_coding 1.141 16.539 1.151
63.504 0.764 0.879 1.229 42.736
ENSMUSG00000047794 Olfr685 protein_coding 1 84.397 1
96.652 1 85.464 1 96.183
ENSMUSG00000047797 Gjb1 protein_coding 1.155 84.977 0.861
93.654 1.004 82.74 1.042 95.273
ENSMUSG00000047798 Cd300lf protein_coding 1.015 75.671
0.792 78.471 1.247 49.058 1.034 92.52
ENSMUSG00000047799 Oog4 protein_coding 1 86.74 NA NA NA
NA 1 93.132
ENSMUSG00000047804 Akap10 protein_coding 1.032 19.893 NA
NA 1.001 46.071 1.02 92.181
ENSMUSG00000047807 9330154K18Rik antisense 1 79.165 NA
NA 1 88.601 1 95.72
ENSMUSG00000047810 Ccdc88b protein_coding 1.08 77.916 NA
NA 0.968 86.666 0.922 88.505
ENSMUSG00000047819 Tigd4 protein_coding 1.367 76.042 1.045
86.387 0.702 42.376 1.03 92.52
ENSMUSG00000047821 Trim16 protein_coding 1.059 45.61 0.965
90.744 0.976 69.583 1.119 72.369
ENSMUSG00000047822 Gm6484 protein_coding 0.365 1.117 1.818
64.304 1.509 27.475 1.109 86.209
ENSMUSG00000047824 Pygo2 protein_coding 1.027 65.733 1.086
71.617 0.897 0.72 0.994 96.183
ENSMUSG00000047828 A730018C14Rik protein_coding 1 66.129

1 96.407 1 87.686 1 96.086
ENSMUSG00000047832 Cdca4 protein_coding 1.08 40.065 0.997
93.899 0.925 15.465 1.032 81.061
ENSMUSG00000047841 BC051628 protein_coding 1.224 75.196
0.661 79.807 1.231 66.928 0.723 87.651
ENSMUSG00000047842 Diras2 protein_coding 1.801 7.083 0.701
84.594 0.794 33.689 0.895 87.331
ENSMUSG00000047843 Bri3 protein_coding 1.171 4.874 1.007 97.097
0.849 4.48 0.888 83.724
ENSMUSG00000047844 Bex4 protein_coding 0.982 86.918 0.731
85.242 1.394 58.915 0.881 91.985
ENSMUSG00000047854 Stx19 protein_coding 0.566 43.435 0.567
74.851 3.125 18.411 0.762 96.358
ENSMUSG00000047861 Foxi1 protein_coding 0.602 52.726 1.405
96.847 1.177 43.842 1.065 94.99
ENSMUSG00000047866 Lonp2 protein_coding 0.875 2.174 1.049 88.923
1.085 19.286 1.036 92.389
ENSMUSG00000047867 Gimap6 protein_coding 1.092 27.731
1.047 90.116 0.873 3.718 0.881 77.245
ENSMUSG00000047868 Olfr770 protein_coding 1 81.01 1
95.504 NA NA 1 94.016
ENSMUSG00000047875 Gpr157 protein_coding 1.078 48.462
0.946 87.553 0.977 86.831 1.018 85.577
ENSMUSG00000047878 A4galt protein_coding 1.096 7.65 0.899
47.831 1.014 88.693 1.184 51.901
ENSMUSG00000047879 Usp14 protein_coding 1.016 84.661 1.021
96.268 0.964 88.274 1.003 78.379
ENSMUSG00000047880 Cxcr5 protein_coding 0.993 69.489 0.908
95.708 1.106 78.045 0.785 86.445
ENSMUSG00000047881 Rell1 protein_coding 1.109 5.253 0.959 79.807
0.943 31.887 1.043 90.463
ENSMUSG00000047884 Klk9 protein_coding 0.988 65.622 0.485
80.463 2.094 27.475 1.123 95.273
ENSMUSG00000047888 Tnrc6b protein_coding 0.986 63.283
1.083 86.387 0.938 80.015 0.909 83.724
ENSMUSG00000047889 Serpinb6d protein_coding 1 79.277 1
96.948 NA NA 1 92.263
ENSMUSG00000047894 Ang2 protein_coding 1 85.283 1
96.013 1 84.229 1 96.183
ENSMUSG00000047897 Ripply2 protein_coding 1 84.865 1
97.288 1 87.173 1 93.627
ENSMUSG00000047898 Ccr4 protein_coding 0.504 82.891 1.103
96.138 1.8 86.985 1.74 95.273
ENSMUSG00000047904 Sstr2 protein_coding 1 71.217 1
95.708 1 71.388 1 96.183
ENSMUSG00000047905 Gm8566 pseudogene 1 77.497 NA NA
1 86.258 1 93.685
ENSMUSG00000047907 Tshz2 protein_coding 0.992 81.925 0.978
96.216 1.029 83.719 1.022 96.071
ENSMUSG00000047909 Ankrd16 protein_coding 1.006 82.479 0.92
81.731 1.075 33.042 1.001 72.369
ENSMUSG00000047910 Pcdhb16 protein_coding 0.97 82.092
1.169 72.642 0.879 32.297 1.097 83.067
ENSMUSG00000047911 Npm2 protein_coding 0.814 28.804 0.904

97.129 1.358 74.082 0.967 96.183
ENSMUSG00000047921 Trappc9 protein_coding 0.886 1.031 1.057
71.222 1.07 44.787 1.018 95.92
ENSMUSG00000047935 Gm5607 protein_coding 1 69.777 1
93.654 1 84.552 1 90.611
ENSMUSG00000047945 Marcksl1 protein_coding 1.185 20.659
1.003 93.654 0.841 32.963 1.085 63.708
ENSMUSG00000047953 Gp5 protein_coding 0.984 56.768 1.306
71.222 0.775 84.669 1.037 82.328
ENSMUSG00000047959 Kcna3 protein_coding 1.17 76.546 0.757
80.463 1.127 72.148 0.538 87.651
ENSMUSG00000047960 Olfr186 protein_coding 1 85.203 NA
NA 1 81.857 1 95.559
ENSMUSG00000047963 Stbd1 protein_coding 1.161 47.847 0.872
72.399 0.986 81.976 1.129 48.119
ENSMUSG00000047965 Rpl9-ps7 pseudogene 1.273 56.085 0.569
62.322 1.384 63.665 1.16 90.865
ENSMUSG00000047969 Olfr1093 protein_coding 1 85.728 1
96.68 1 86.346 1 95.72
ENSMUSG00000047976 Kcna1 protein_coding 0.897 29.972 0.86
63.912 1.289 7.365 0.886 83.724
ENSMUSG00000047977 Synb protein_coding 1 82.231 1
95.735 NA NA 1 94.904
ENSMUSG00000047980 9230110F15Rik protein_coding 1 84.819
1 95.708 1 84.308 1 93.722
ENSMUSG00000047986 Palm3 protein_coding 1.465 5.313 0.651 33.301
1.046 81.976 1.134 83.067
ENSMUSG00000047988 4933428G20Rik protein_coding 0.781 16.895
1.278 82.904 1.004 82.778 0.865 86.699
ENSMUSG00000047989 Ino80c protein_coding 0.989 71.674
1.068 71.98 0.947 41.506 1.119 55.485
ENSMUSG00000047990 C2cd4a protein_coding 0.514 32.749
2.288 72.642 0.848 84.896 0.336 88.136
ENSMUSG00000047992 Fam69c protein_coding 1.592 7.65 1.297
96.407 0.486 14.583 1.408 49.514
ENSMUSG00000047995 Cypt4 protein_coding 1 80.608 1
95.35 1 84.896 1 96.183
ENSMUSG00000047996 Prrg1 protein_coding 1.329 0 0.989 96.407
0.758 0.72 1.024 95.92
ENSMUSG00000048000 Gigyf2 protein_coding 0.99 25.3 0.976
90.053 1.031 31.4 0.985 88.657
ENSMUSG00000048003 Catsper4 protein_coding 0.845 46.693
1.068 86.156 1.108 88.693 0.998 96.287
ENSMUSG00000048004 Tmem196 protein_coding 3.852 63.804
0.446 95.504 0.582 77.704 0.535 92.181
ENSMUSG00000048007 Timm8a1 protein_coding 0.891 45.229
1.024 96.847 1.094 54.363 1.127 65.175
ENSMUSG00000048012 Zfp473 protein_coding 1.115 81.925
0.926 90.138 0.965 77.17 1.036 96.071
ENSMUSG00000048013 NA NA NA NA 1 83.392 1
46.206 1 87.624
ENSMUSG00000048015 Neurod4 protein_coding 1 82.479 1
95.301 1 61.418 1 96.192
ENSMUSG00000048022 Tmem229a protein_coding 0.7 18.487

1.203 83.819 1.181 41.331 0.902 95.273
ENSMUSG00000048027 Rgmb protein_coding 1.014 58.198 1.021 91.643 0.963 25.625 1.006 84.29
ENSMUSG00000048029 Eno4 protein_coding 1.036 85.728 0.853 83.58 1.131 62.237 0.899 93.722
ENSMUSG00000048031 Fcrl5 protein_coding 1 79.577 1 90.895 1 60.614 1 93.964
ENSMUSG00000048038 4932418E24Rik protein_coding 0.887 42.361 1.415 74.663 0.799 68.82 0.929 94.19
ENSMUSG00000048039 Isg20l2 protein_coding 0.967 72.139 1.024 95.431 1.009 83.811 1.059 57.1
ENSMUSG00000048040 Arxes2 protein_coding 0.821 12.084 NA NA 1.158 28.997 0.934 95.676
ENSMUSG00000048047 Zbtb33 protein_coding 1.043 24.481 0.979 96.378 0.977 28.237 0.98 86.699
ENSMUSG00000048058 Ldlrad3 protein_coding 0.934 48.354 0.934 91.275 1.149 42.522 0.962 85.493
ENSMUSG00000048062 Fpr-rs4 protein_coding 1 86.351 NA NA 1 86.666 1 96.183
ENSMUSG00000048065 Cyb5r2 protein_coding 1.187 52.258 NA NA 0.964 76.923 0.964 94.904
ENSMUSG00000048067 Olfr1349 protein_coding 1 85.728 1 96.68 1 87.173 1 95.777
ENSMUSG00000048070 Pirt protein_coding 0.355 31.333 1.245 96.407 2.266 10.281 1.16 96.358
ENSMUSG00000048076 Arf1 protein_coding 1.014 76.968 1.021 89.434 0.968 63.919 1.073 36.988
ENSMUSG00000048077 H1fnt protein_coding 1 77.615 1 96.268 NA NA 1 95.35
ENSMUSG00000048078 Tenm4 protein_coding 0.921 71.915 1.3 71.222 0.838 30.827 1.172 93.117
ENSMUSG00000048096 Lmod1 protein_coding 0.98 69.24 1.119 81.039 0.914 44.019 0.949 87.542
ENSMUSG00000048100 Taf13 protein_coding 0.964 48.354 0.986 84.594 1.053 28.055 1.011 83.581
ENSMUSG00000048101 Olfr19 protein_coding 1 81.465 1 91.776 1 78.045 1 96.042
ENSMUSG00000048106 4632415L05Rik protein_coding 0.972 75.298 1.061 96.68 0.967 86.475 0.884 84.29
ENSMUSG00000048107 Kctd12 pseudogene 1 81.76 1 97.263 1 83.538 1 92.748
ENSMUSG00000048108 Tmem72 protein_coding 1 61.725 1 96.897 1 75.353 1 95.72
ENSMUSG00000048109 Rbm15 protein_coding 1.121 7.174 1.146 82.103 0.775 0.945 0.98 95.877
ENSMUSG00000048118 Arid4a protein_coding 1.016 41.211 1.017 96.268 0.967 51.443 0.977 83.724
ENSMUSG00000048120 Entpd1 protein_coding 1.14 0.2 NA NA 0.865 5.453 1.056 76.347
ENSMUSG00000048126 Col6a3 protein_coding 1.15 28.098 0.98 96.407 0.887 14.262 1.484 33.269
ENSMUSG00000048138 Dmrt2 protein_coding 0.795 31.333 1.223 79.29 1.023 87.898 1.109 93.627
ENSMUSG00000048142 Nat8l protein_coding 0.921 68.846 1.093

82.904 0.993 82.903 0.852 83.724
ENSMUSG00000048148 Nwd1 protein_coding 1.398 55.124 0.81
92.631 0.882 54.964 0.804 87.735
ENSMUSG00000048149 Gm12663 pseudogene 1 58.38 1 97.263
1 87.971 1 96.358
ENSMUSG00000048153 Olfr49 protein_coding 1 80.408 1
95.708 1 81.032 1 96.287
ENSMUSG00000048154 Kmt2d protein_coding 0.762 0.987 1.099 63.504
1.197 5.864 0.956 85.493
ENSMUSG00000048155 H2bfm protein_coding 1 78.639 1
95.504 1 88.601 1 96.086
ENSMUSG00000048163 Selplg protein_coding 1.398 1.412 0.857
81.284 0.836 17.898 0.995 96.183
ENSMUSG00000048164 Gm7204 pseudogene 1 83.169 1
95.708 NA NA 1 96.287
ENSMUSG00000048170 Mcmbp protein_coding 1.119 8.281 0.958 87.322
0.934 23.938 1.008 89.152
ENSMUSG00000048173 Olfr1324 protein_coding 1 77.407 1
95.431 1 86.831 1 94.153
ENSMUSG00000048174 Tmem81 protein_coding 0.972 84.977
1.003 85.075 1.022 55.109 0.95 89.475
ENSMUSG00000048175 Asb8 protein_coding 0.963 60.003 1.06
83.104 0.981 87.744 1.1 42.736
ENSMUSG00000048186 Bend7 protein_coding 0.865 28.998 0.95
89.509 1.213 18.411 0.849 83.724
ENSMUSG00000048188 Gm8181 pseudogene 1 86.918 1
96.407 1 81.032 1 94.654
ENSMUSG00000048191 Muc6 protein_coding 0.798 24 0.912 91.661
1.376 43.129 0.684 83.724
ENSMUSG00000048197 Olfr1153 protein_coding 1 79.93 1
92.545 NA NA 1 92.742
ENSMUSG00000048200 Efcab4a protein_coding 0.914 29.21 0.98
92.137 1.111 21.873 0.939 83.724
ENSMUSG00000048206 NA NA NA NA 1 97.097 1
86.666 1 96.358
ENSMUSG00000048215 A630023P12Rik lincRNA 1.308 73.124
0.667 87.452 1.149 85.792 0.521 88.136
ENSMUSG00000048216 Gpr85 protein_coding 1.283 39.075 0.997
95.211 0.784 42.997 1.172 87.088
ENSMUSG00000048218 Amigo2 protein_coding 1.233 1.626 0.951
75.052 0.854 9.131 0.91 87.542
ENSMUSG00000048222 Mfap1b protein_coding 1.036 72.372
0.968 95.708 NA NA 1.043 57.1
ENSMUSG00000048226 Olfr1212 protein_coding 1 84.263 1
97.155 1 86.727 1 94.99
ENSMUSG00000048231 H2-M10.4 protein_coding 1 86.918 NA
NA 1 86.144 1 94.19
ENSMUSG00000048232 Fbxo10 protein_coding 0.982 85.637
0.968 86.277 1.052 61.171 0.999 85.493
ENSMUSG00000048234 Rnf149 protein_coding 0.982 74.745
0.894 31.637 1.138 18.565 1.029 93.584
ENSMUSG00000048240 Gng7 protein_coding 1.155 2.723 0.983 95.504
0.876 1.898 0.981 85.493
ENSMUSG00000048248 D630032N06Rik pseudogene 0.673 25.551

0.971 95.708 1.525 20.556 0.974 96.248
ENSMUSG00000048249 Crebrf protein_coding 0.924 67.163 0.96
85.793 1.123 17.751 0.986 87.182
ENSMUSG00000048251 Bcl11b protein_coding 0.803 81.24 1.239
96.68 1.002 87.686 0.795 83.724
ENSMUSG00000048261 Gm4879 pseudogene 1 84.397 1
94.597 1 77.532 1 95.273
ENSMUSG00000048264 Dip2c protein_coding 1.057 34.696 1.016
93.899 0.933 15.904 0.999 92.381
ENSMUSG00000048271 Rbm33 protein_coding 0.928 11.769 0.87
50.664 1.238 5.864 0.988 90.805
ENSMUSG00000048277 Syngr2 protein_coding 1.022 83.169
1.008 96.843 0.966 78.045 0.959 93.584
ENSMUSG00000048279 Sacs protein_coding 0.893 48.146 1.123
79.261 0.993 66.753 1.035 95.92
ENSMUSG00000048280 Zfp738 protein_coding 1.063 75.196
0.935 91.661 1.004 87.744 1.007 92.381
ENSMUSG00000048281 Dleu7 protein_coding 1.195 69.489 0.635
93.817 1.312 88.693 0.784 94.904
ENSMUSG00000048284 Tas2r126 protein_coding 1 85.728 1
96.763 1 69.315 1 96.287
ENSMUSG00000048285 Frmd6 protein_coding 1.131 5.848 1.014 95.431
0.867 3.073 0.969 87.651
ENSMUSG00000048292 Olfr1417 protein_coding 1 84.661 1
90.378 1 65.8 1 94.904
ENSMUSG00000048294 Krtap4-13 protein_coding 1 78.538 1
94.597 1 85.464 1 94.297
ENSMUSG00000048299 Olfr148 protein_coding 1 83.333 1
95.708 1 83.607 1 95.273
ENSMUSG00000048307 Ankrd46 protein_coding 0.962 68.66 1.019
92.931 1.021 82.74 1.089 72.616
ENSMUSG00000048310 Pskh1 protein_coding 0.952 65.199 1.053
88.533 0.996 87.336 0.954 83.724
ENSMUSG00000048312 Gm4884 protein_coding 1 85.499 1
96.652 1 86.144 1 96.192
ENSMUSG00000048316 Gm9823 pseudogene 0.8 13.74 1.085 85.756
1.148 43.307 0.812 85.493
ENSMUSG00000048327 Ckap2l protein_coding 1.838 26.136
0.688 54.245 0.794 88.461 1.319 74.193
ENSMUSG00000048329 Mfsd6l protein_coding 0.938 84.661
0.793 66.651 1.344 21.462 0.98 89.475
ENSMUSG00000048330 Ric3 protein_coding 1.257 22.025 0.932
80.052 0.856 38.534 NA NA
ENSMUSG00000048332 Lhfp protein_coding 1.074 62.007 0.99
96.013 0.937 67.648 0.999 95.92
ENSMUSG00000048334 Gm8258 protein_coding 0.909 86.464
0.735 88.691 1.495 63.801 0.47 90.854
ENSMUSG00000048347 Pcdhb18 protein_coding 0.962 65.106
1.169 79.055 0.89 29.765 1.065 93.156
ENSMUSG00000048349 Pou4f1 protein_coding 1.026 76.396
2.301 93.574 0.425 67.962 0.957 95.273
ENSMUSG00000048351 Selrc1 protein_coding 0.995 61.633
1.066 85.501 0.945 41.928 0.988 94.904
ENSMUSG00000048355 Arxes1 protein_coding 0.64 2.524 1.168

82.103 1.342 7.635 1.089 78.379
ENSMUSG00000048356 Olfr1496 protein_coding 1 79.93 1
92.545 1 86.602 1 92.381
ENSMUSG00000048368 Omd protein_coding 0.911 34.795 1.012
95.708 1.08 38.26 1.124 82.66
ENSMUSG00000048371 Pdp2 protein_coding 1.107 3.644 1.091 84.126
0.826 2.294 0.98 88.053
ENSMUSG00000048373 Fgfbp1 protein_coding 1.47 23.82 NA NA
0.579 19.552 0.763 92.12
ENSMUSG00000048376 F2r protein_coding 1.1 84.324 0.939
95.35 0.965 79.325 0.888 91.985
ENSMUSG00000048377 Foxi2 protein_coding 1 77.291 1
97.155 1 79.255 1 95.559
ENSMUSG00000048378 Olfr1394 protein_coding 1 75.399 1
95.708 NA NA 1 96.287
ENSMUSG00000048379 Socs4 protein_coding 1.056 38.386 0.952
85.671 0.992 64.155 1.018 91.872
ENSMUSG00000048385 Scrt1 protein_coding 0.551 37.248 0.897
90.389 2.024 70.534 2.054 93.132
ENSMUSG00000048387 Osr1 protein_coding 1.14 18.985 NA NA
0.899 15.778 0.972 91.985
ENSMUSG00000048388 Fam171b protein_coding 1.481 0 1.034
96.138 0.652 3.718 1.074 85.786
ENSMUSG00000048391 Olfr843 protein_coding 1 73.401 1
96.308 1 83.857 1 95.559
ENSMUSG00000048398 Gm5065 protein_coding 1 79.577 1
96.68 1 84.669 1 92.762
ENSMUSG00000048399 Tprg protein_coding 1.504 24.689 0.862
87.998 0.771 66.753 0.788 89.294
ENSMUSG00000048400 Prss54 protein_coding 1 82.231 1
97.263 1 83.538 1 94.904
ENSMUSG00000048402 Gli2 protein_coding 1.326 6.958 0.927 86.604
0.812 1.58 1.233 48.119
ENSMUSG00000048406 B330016D10Rik pseudogene 0.811 26.77 1.041
97.155 1.185 41.6 0.975 86.699
ENSMUSG00000048410 Zfp407 protein_coding 0.886 15.486 NA
NA 1.123 20.085 1.085 59.441
ENSMUSG00000048411 Gm597 protein_coding 1 84.294 NA NA
1 88.601 1 96.183
ENSMUSG00000048416 Mlf1 protein_coding 3.274 5.253 1.502 82.904
0.202 0.72 1.394 89.428
ENSMUSG00000048424 Ranbp3l protein_coding 1.314 9.695 1.047
94.767 0.728 9.573 1.02 96.248
ENSMUSG00000048425 Olfr686 protein_coding 1 82.891 1
92.404 1 84.896 1 95.74
ENSMUSG00000048429 1810026J23Rik protein_coding 1.018 81.465
1.019 83.819 0.966 54.25 0.973 94.99
ENSMUSG00000048439 Nupl2 protein_coding 1.078 42.555 0.932
60.678 0.992 57.637 0.938 87.651
ENSMUSG00000048440 Cyp4f16 protein_coding 0.988 86.474
1.056 89.996 0.959 57.091 1.024 49.806
ENSMUSG00000048442 Smim5 protein_coding 1.581 14.59 0.705 89.996
0.898 45.799 1.184 94.384
ENSMUSG00000048445 Ccdc57 protein_coding 0.722 3.641 1.051

91.897 1.322 7.635 1.123 95.559
ENSMUSG00000048450 Msx1 protein_coding 1.336 3.151 1.024 95.431
0.728 1.408 0.878 84.29
ENSMUSG00000048455 Sprr1b protein_coding 1 83.523 NA
NA 1 87.173 1 91.65
ENSMUSG00000048456 Olfr1448 protein_coding 1 78.744 1
96.897 1 79.524 1 87.886
ENSMUSG00000048458 Fam212b protein_coding 0.874 16.02 1.259
63.504 0.905 48.387 1.013 94.297
ENSMUSG00000048469 Olfr564 protein_coding 1 76.968 1
97.155 1 86.831 1 90.177
ENSMUSG00000048473 Gm766 protein_coding 1 85.728 1
95.809 1 81.976 1 96.248
ENSMUSG00000048478 Spata33 protein_coding 0.868 70.862
1.077 94.367 1.071 84.229 0.935 96.248
ENSMUSG00000048480 Cxcr1 protein_coding 1 86.918 1
96.847 NA NA 1 94.384
ENSMUSG00000048481 Mypop protein_coding 1.154 37.702 1.012
82.904 0.857 6.858 0.93 86.991
ENSMUSG00000048482 Bdnf protein_coding 1.426 11.928 0.757
60.678 0.927 86.727 1.043 93.964
ENSMUSG00000048484 Gm7461 protein_coding 0.451 52.726
1.619 96.68 1.373 61.418 0.716 95.777
ENSMUSG00000048485 Zbtb8b protein_coding 1.542 3.498 0.979
95.504 0.661 16.051 1.398 92.018
ENSMUSG00000048486 Fitm2 protein_coding 0.873 13.952 1.047
90.116 1.09 25.564 1.009 92.018
ENSMUSG00000048489 8430408G22Rik protein_coding 1.156 36.195
0.889 81.731 0.969 86.666 0.857 83.724
ENSMUSG00000048490 Nrip1 protein_coding 0.906 20.116 1.041
88.823 1.057 36.578 1.008 96.358
ENSMUSG00000048495 Tyw5 protein_coding 0.89 31.921 1.088
85.756 1.035 66.043 0.95 83.724
ENSMUSG00000048497 Mmgt2 protein_coding 1.01 81.52 0.922 71.98
1.073 54.673 0.961 85.493
ENSMUSG00000048501 Olfr938 protein_coding 1 81.24 1
95.504 1 83.925 1 92.381
ENSMUSG00000048503 Tmem136 protein_coding 1.132 35.371
0.876 38.923 1.008 82.436 1.005 94.001
ENSMUSG00000048516 NA NA NA NA 1 96.268 1
86.144 1 96.287
ENSMUSG00000048520 Fbxl13 protein_coding 1.173 16.436
0.776 75.345 1.099 73.531 0.921 95.72
ENSMUSG00000048521 Cxcr6 protein_coding 1.51 10.595 1.012
96.216 0.656 28.489 0.687 83.724
ENSMUSG00000048528 Nkx1-2 protein_coding 1 82.231 1
96.216 1 80.163 1 95.593
ENSMUSG00000048534 Amica1 protein_coding 1.071 82.982
0.772 62.71 1.21 43.185 1.076 95.777
ENSMUSG00000048537 Phldb1 protein_coding 0.905 13.062
1.026 95.735 1.079 28.055 1.051 81.251
ENSMUSG00000048538 Gm9826 pseudogene 1 85.637 1
93.469 1 77.17 1 82.66
ENSMUSG00000048540 Nlhlh2 protein_coding 1 85.59 1 96.68 1

83.857	1	95.273
ENSMUSG00000048544	5930422O12Rik	protein_coding	0.923	85.59
0.707	79.475	1.528	49.384	1.266	78.379
ENSMUSG00000048546	Tob2	protein_coding	1.221	1.161	0.959	86.861
0.855	11.64	0.974	91.269
ENSMUSG00000048550	Thnsl1	protein_coding	0.981	64.284
0.993	96.984	1.027	70.328	0.989	93.132
ENSMUSG00000048559	4930555K19Rik	pseudogene	1	83.263	1
95.088	1	86.144	1	93.156
ENSMUSG00000048562	Sp8	protein_coding	1	86.464	1
95.088	1	58.616	1	96.287
ENSMUSG00000048572	Tmem252	protein_coding	1.192	61.171
0.764	96.216	1.094	72.464	1.306	86.118
ENSMUSG00000048573	Cypt3	protein_coding	1	85.728	1
95.708	1	86.666	1	95.273
ENSMUSG00000048574	Gm5593	pseudogene	1	82.705	1
92.137	1	70.534	1	94.19
ENSMUSG00000048578	Mlec	protein_coding	1.03	33.555	0.993
96.843	0.977	14.409	1.045	32.045
ENSMUSG00000048581	E130311K13Rik	protein_coding	1.227	0.2
0.867	47.831	0.939	35.559	1.08	81.251
ENSMUSG00000048583	Igf2	protein_coding	1.167	6.135	NA	NA	0.85
14.409	1.013	93.413
ENSMUSG00000048592	Gm5946	pseudogene	1	86.819	1
88.897	1	85.921	1	90.674
ENSMUSG00000048600	Gm5763	pseudogene	1.382	71.217	0.336
50.664	2.143	25.93	0.504	90.805
ENSMUSG00000048603	Gm9828	antisense	0.793	14.221	1.036
95.504	1.217	23.668	0.895	83.952
ENSMUSG00000048612	Myof	protein_coding	1.031	55.124	0.843
20.174	1.153	16.26	1.046	70.624
ENSMUSG00000048616	Nog	protein_coding	1.248	34.077	0.992
93.899	0.804	26.809	1.215	82.66
ENSMUSG00000048620	Olfr1336	protein_coding	1	84.661	1
96.948	1	85.792	1	94.567
ENSMUSG00000048621	Gm6377	protein_coding	1.45	14.59	0.668
70.29	1.03	84.724	1.269	62.594
ENSMUSG00000048626	Klf17	protein_coding	1	81.76	1	95.301
1	76.923	1	89.108
ENSMUSG00000048628	4930542C16Rik	lincRNA	1	84.819	1
97.263	1	86.666	1	95.74
ENSMUSG00000048636	A730049H05Rik	protein_coding	0.841	22.92
0.833	91.661	1.429	15.688	1.244	80.187
ENSMUSG00000048643	NA	NA	NA	NA	NA	NA	1	86.144
1	95.72
ENSMUSG00000048644	Ctxn1	protein_coding	0.749	25.111	1.119
96.68	1.196	24.07	0.863	86.699
ENSMUSG00000048647	Exd1	protein_coding	1.039	85.59	0.967	92.137
0.992	74.686	0.84	83.724
ENSMUSG00000048652	Samd13	protein_coding	0.761	51.056	3.02
71.98	0.435	44.019	0.944	92.675
ENSMUSG00000048655	Ccdc169	protein_coding	0.756	67.477
2.015	83.104	0.655	77.704	1.877	67.94
ENSMUSG00000048661	Lemd3	protein_coding	0.987	69.985	NA	NA

0.998 76.225 0.957 84.29
ENSMUSG00000048668 Rhno1 protein_coding 0.921 8.709 1.021 83.104
1.065 36.388 1.036 92.018
ENSMUSG00000048677 Tpcn2 protein_coding 1.16 6.61 1.012 96.268
0.85 11.751 1.05 52.982
ENSMUSG00000048686 Hmgb4 protein_coding 1 86.199 1
91.661 1 84.308 1 91.783
ENSMUSG00000048693 Olfr435 protein_coding 1 85.283 1
95.329 1 88.729 1 96.183
ENSMUSG00000048696 Mex3d protein_coding 0.924 50.768 1.248
72.642 0.867 52.972 0.906 84.29
ENSMUSG00000048697 Vmn1r26 protein_coding 1 80.608 1
95.504 1 87.37 1 93.964
ENSMUSG00000048701 Ccdc6 protein_coding 1.137 0 0.993 96.216
0.885 0.72 1.035 60.968
ENSMUSG00000048703 Speer4b protein_coding 1 81.125 1
96.843 1 83.275 1 93.457
ENSMUSG00000048706 Lurap1l protein_coding 0.89 41.759
0.996 96.843 1.131 46.575 0.917 85.493
ENSMUSG00000048707 Tprn protein_coding 0.862 17.276 1.08
94.367 1.073 50.375 1.011 92.181
ENSMUSG00000048709 Gm8666 pseudogene 1 53.809 1
95.35 1 84.434 1 94.654
ENSMUSG00000048720 Tbc1d12 protein_coding 1.137 3.176 NA NA
0.871 8.634 0.908 84.29
ENSMUSG00000048728 Zfp454 protein_coding 1.212 45.746
1.121 96.407 0.736 38.534 0.932 95.74
ENSMUSG00000048731 Ggnbp1 protein_coding 0.842 2.142 1.014
96.216 1.172 10.615 1.002 84.29
ENSMUSG00000048732 Klhl11 protein_coding 0.864 17.031
0.946 95.211 1.219 28.979 0.898 86.991
ENSMUSG00000048745 Olfr820 protein_coding 1 86.819 1
96.847 1 85.464 1 94.82
ENSMUSG00000048747 E130114P18Rik protein_coding 1.073 85.092
1.002 95.735 NA NA 0.675 83.724
ENSMUSG00000048755 Mcat protein_coding 0.945 12.181 1.031
82.904 1.025 82.436 0.982 86.699
ENSMUSG00000048756 Foxo3 protein_coding 1.141 25.207 0.871
33.183 1.006 72.853 1.035 93.045
ENSMUSG00000048758 Rpl29 protein_coding 0.876 10.143 1.001
96.216 1.144 7.635 1.095 54.153
ENSMUSG00000048763 Hoxb3 protein_coding 0.805 2.009 0.986 96.843
1.254 8.339 0.893 87.182
ENSMUSG00000048766 Skint10 protein_coding 1 83.968 NA
NA NA NA 1 92.691
ENSMUSG00000048772 Tmem53 protein_coding 0.918 24 1.121
78.705 0.971 75.637 0.9 83.724
ENSMUSG00000048775 Serpinb13 protein_coding 1 81.24 1
96.843 1 84.071 1 93.685
ENSMUSG00000048776 Pthlh protein_coding 1.096 50.627 0.957
94.887 0.956 79.776 1.193 89.108
ENSMUSG00000048779 P2ry6 protein_coding 1.511 0.824 0.96 92.137
0.69 1.973 1.093 83.067
ENSMUSG00000048782 Insc protein_coding 1.243 49.202 1.319

85.756 0.609 13.848 0.858 87.331
ENSMUSG00000048787 Dcun1d3 protein_coding 1.156 40.333
0.937 93.654 0.92 47.641 0.92 84.29
ENSMUSG00000048790 E030011O05Rik protein_coding 1 85.355
1 95.941 1 80.899 1 92.748
ENSMUSG00000048794 Ccdc37 protein_coding 0.9 8.405 0.896
88.881 1.24 7.635 1.026 95.273
ENSMUSG00000048796 Cyb561d1 protein_coding 0.945 22.142
0.959 81.02 1.104 17.751 1.005 93.734
ENSMUSG00000048799 Cep120 protein_coding 1.029 66.272
0.998 95.301 0.976 75.967 0.978 95.273
ENSMUSG00000048806 Ifnb1 protein_coding 1 80.03 1 95.708
1 87.103 1 94.19
ENSMUSG00000048807 Slc35e4 protein_coding 1.536 2.569 0.727
48.565 0.894 73.598 0.909 95.273
ENSMUSG00000048810 Olfr202 protein_coding 1 81.196 1
97.129 1 86.552 1 96.248
ENSMUSG00000048814 Lonrf2 protein_coding 0.937 81.823 1.01
87.553 1.059 75.009 0.912 84.29
ENSMUSG00000048826 Dact2 protein_coding 0.818 12.084 1.05
95.708 1.168 30.107 0.88 85.493
ENSMUSG00000048827 Pkd1l3 protein_coding 0.786 11.726
1.044 97.129 1.216 33.042 0.931 92.181
ENSMUSG00000048830 2310057N15Rik protein_coding 1 75.298
1 95.941 1 70.791 1 95.593
ENSMUSG00000048832 Vps37c protein_coding 0.949 25.492
0.997 95.301 1.06 19.025 1.034 70.539
ENSMUSG00000048833 Slc39a9 protein_coding 1.005 71.674
0.967 75.618 1.03 27.047 0.991 90.628
ENSMUSG00000048852 Gm12185 protein_coding 1.194 22.633
1.439 76.086 0.581 2.687 0.859 83.724
ENSMUSG00000048856 Slc25a47 protein_coding 0.91 37.248
0.861 67.839 1.278 10.76 1.051 92.381
ENSMUSG00000048865 Arhgap30 protein_coding 1.28 0 1.042
96.216 0.747 4.521 1.041 85.629
ENSMUSG00000048874 Phf3 protein_coding 1.018 39.295 0.997
93.765 0.986 66.753 1.008 96.183
ENSMUSG00000048875 Hdhd1a protein_coding 1 83.169 1
93.765 1 87.173 1 93.964
ENSMUSG00000048878 Hexim1 protein_coding 0.902 65.388
1.121 83.104 0.985 87.971 0.974 83.724
ENSMUSG00000048895 Cdk5r1 protein_coding 1.035 85.203
0.772 72.399 1.254 41.331 1.081 94.904
ENSMUSG00000048897 Zfp710 protein_coding 1.033 62.574
0.985 93.654 0.984 65.688 1.024 95.559
ENSMUSG00000048899 Rimkla protein_coding 1.047 74.482
1.207 90.895 0.789 50.523 0.813 84.29
ENSMUSG00000048904 Neurog1 protein_coding 1 81.178 1
96.268 1 84.896 1 95.832
ENSMUSG00000048905 4930539E08Rik protein_coding 1.29 38.386
0.779 65.165 0.99 88.076 0.828 95.593
ENSMUSG00000048910 Fam220a protein_coding 1 85.857 1
95.708 1 87.744 1 96.287
ENSMUSG00000048911 Rnf24 protein_coding 0.977 79.853 1.129

82.219 0.907 20.258 0.99 92.181
ENSMUSG00000048915 Efna5 protein_coding 1.05 86.74 0.988 95.431
0.965 80.015 0.952 86.991
ENSMUSG00000048919 Olfr223 protein_coding 1 86.57 1
96.847 1 87.744 1 90.397
ENSMUSG00000048920 Fkrp protein_coding 0.957 54.176 1.086
71.617 0.965 44.211 0.984 94.19
ENSMUSG00000048921 Zfp689 protein_coding 0.955 57.544 1.11
85.756 0.944 85.921 0.902 95.593
ENSMUSG00000048922 Cdca2 protein_coding 1.071 64.193 0.757
69.611 1.229 69.728 1.8 61.753
ENSMUSG00000048924 Ccdc125 protein_coding 0.946 66.129 0.93
88.345 1.131 54.363 0.95 88.053
ENSMUSG00000048930 Tada3 protein_coding 0.966 27.431 1.013
95.211 1.016 43.185 1.013 89.428
ENSMUSG00000048933 Olfr722 protein_coding 1 83.169 1
96.308 1 81.976 1 87.447
ENSMUSG00000048938 Nr1h5 protein_coding 1 50.913 1
92.474 1 74.853 1 87.992
ENSMUSG00000048949 Gm6206 pseudogene 0.748 56.477 1.188
88.136 1.13 66.043 1.115 94.904
ENSMUSG00000048960 Prex2 protein_coding 0.885 22.237 0.935
75.345 1.204 10.76 1.078 67.691
ENSMUSG00000048962 Rassf10 pseudogene 1 81.24 1 95.088
1 60.789 1 93.016
ENSMUSG00000048965 Mrgpre protein_coding 0.89 44.467
0.832 52.266 1.346 6.916 1.172 86.133
ENSMUSG00000048967 Yjefn3 protein_coding 0.83 28.242
1.023 96.138 1.173 27.431 0.984 91.625
ENSMUSG00000048970 C1galt1c1 protein_coding 1.073 55.462
0.987 97.263 0.945 54.25 1.096 48.119
ENSMUSG00000048981 Krt31 protein_coding 0.32 2.009 1.128 85.501
2.773 17.412 0.629 89.294
ENSMUSG00000048982 Gphb5 protein_coding 1 57.655 NA NA
1 71.999 1 91.217
ENSMUSG00000048988 Elfn1 protein_coding 1.749 10.595 0.58
81.731 0.983 80.749 0.878 93.685
ENSMUSG00000048994 4930557A04Rik protein_coding 1 77.113
1 95.504 1 84.071 1 93.612
ENSMUSG00000048996 Olfr1366 protein_coding 1 84.564 1
91.361 1 49.898 1 95.877
ENSMUSG00000048997 Atxn7l2 protein_coding 0.884 5.946 1.039
96.843 1.092 31.479 1.008 95.777
ENSMUSG00000049001 Ndnf protein_coding 1.368 13.105 0.772
85.116 0.946 45.799 1.046 93.156
ENSMUSG00000049008 BB014433 protein_coding 1 86.74 1
96.68 1 88.601 1 93.016
ENSMUSG00000049010 Olfr982 protein_coding 1 71.516 1
94.597 1 86.475 1 93.413
ENSMUSG00000049011 Olfr729 protein_coding 1 72.546 1
97.049 1 86.258 1 93.132
ENSMUSG00000049015 Olfr1467 protein_coding 1 86.658 1
94.848 1 86.602 1 96.183
ENSMUSG00000049018 Olfr368 protein_coding 1 86.918 1

97.263	1	87.686	1	93.132
ENSMUSG00000049028	Olfr873	protein_coding	1	85.499	1
92.545	1	82.436	1	95.74
ENSMUSG00000049036	Tmem121	protein_coding	1.133	84.865
1.045	95.708	0.845	83.066	0.791	93.612
ENSMUSG00000049037	Clec4a1	protein_coding	1.728	0	0.88
54.245	0.654	6.935	1.048	92.381
ENSMUSG00000049038	Mterfd3	protein_coding	0.928	64.893
1.083	85.378	NA	NA	1.172	61.984
ENSMUSG00000049041	Olfr398	protein_coding	1	72.546	NA
NA	1	83.719	1	94.904
ENSMUSG00000049044	Rapgef4	protein_coding	1.135	10.464
0.922	85.075	0.957	60.614	0.866	83.724
ENSMUSG00000049047	Armcx3	protein_coding	1.162	0.461	1
97.263	0.861	2.361	0.984	84.31
ENSMUSG00000049052	Olfr812	protein_coding	1	62.007	1
96.001	1	76.754	1	91.625
ENSMUSG00000049057	Olfr1257	protein_coding	1	86.172	1
91.83	1	72.695	1	93.261
ENSMUSG00000049062	Gm9244	pseudogene	1	85.092	1
96.013	1	88.349	1	93.156
ENSMUSG00000049073	Olfr985	protein_coding	1	83.523	1
93.654	1	86.552	1	95.593
ENSMUSG00000049076	Acap2	protein_coding	1.079	18.067	NA	NA
0.925	18.539	1.012	82.328
ENSMUSG00000049086	Bmyc	protein_coding	0.845	11.103	1.128
89.494	1.052	32.173	0.903	95.832
ENSMUSG00000049090	Zadh2	protein_coding	1.023	85.655	0.991
93.707	0.987	78.954	0.976	96.287
ENSMUSG00000049091	Sephs2	protein_coding	0.896	12.181
1.029	96.843	1.087	47.31	1.045	81.901
ENSMUSG00000049092	Gpr137c	protein_coding	0.951	58.56	0.809
85.671	1.303	43.129	0.719	83.724
ENSMUSG00000049093	Il23r	protein_coding	0.629	80.146	2.112
79.29	0.751	46.32	0.732	89.525
ENSMUSG00000049094	Olfr367-ps	pseudogene	1	81.925	1
92.137	1	80.381	1	95.74
ENSMUSG00000049097	Ankrd34a	protein_coding	1.446	14.205
0.706	74.483	NA	NA	0.64	89.294
ENSMUSG00000049098	Olfr147	protein_coding	1	68.353	1
96.407	1	87.103	1	93.685
ENSMUSG00000049100	Pcdh10	protein_coding	0.891	72.139
1.444	82.219	0.778	51.715	0.924	93.045
ENSMUSG00000049103	Ccr2	protein_coding	1.595	0.355	0.802	60.678
0.78	19.449	0.996	93.457
ENSMUSG00000049106	Dcaf5	protein_coding	1.053	29.057	0.948
74.851	1.004	84.552	1.017	96.358
ENSMUSG00000049107	Ntf3	protein_coding	1.476	4.362	0.933	96.216
0.725	3.428	0.886	84.29
ENSMUSG00000049109	Themis	protein_coding	2.566	18.314
0.878	83.733	0.441	30.024	0.812	92.675
ENSMUSG00000049112	Oxtr	protein_coding	0.896	75.761	1.366
60.309	0.814	21.331	1.91	74.193
ENSMUSG00000049115	Agtr1a	protein_coding	0.722	22.431

0.744 61.883 1.866 20.085 0.991 91.105
ENSMUSG00000049119 Fam110b protein_coding 1.227 9.259 0.982 95.708 0.829 10.23 0.9 83.724
ENSMUSG00000049122 Frmd3 protein_coding 0.95 79.433 1.08 94.89 0.973 86.602 1.229 60.466
ENSMUSG00000049123 Catsperg2 protein_coding 1 72.743 NA NA 1 79.554 1 96.358
ENSMUSG00000049130 C5ar1 protein_coding 1.334 3.598 0.912 83.58 0.822 28.901 1.127 49.514
ENSMUSG00000049133 Flg2 protein_coding 1 75.298 NA NA 1 88.076 1 95.559
ENSMUSG00000049134 Nrap protein_coding 6.725 6.23 1.156 87.037 0.128 0.72 0.739 94.99
ENSMUSG00000049149 Olfr1258 protein_coding 1 85.77 NA NA 1 87.336 1 96.086
ENSMUSG00000049152 Ugt3a2 protein_coding 0.761 7.881 1.056 86.156 1.242 23.454 0.916 95.72
ENSMUSG00000049160 4930469G21Rik antisense 1 58.264 1 90.046 1 55.512 1 96.287
ENSMUSG00000049164 Zfp518a protein_coding 1.015 58.198 0.973 90.287 1.014 88.076 0.997 92.181
ENSMUSG00000049168 Olfr449 protein_coding 1 85.857 NA NA 1 87.898 1 93.722
ENSMUSG00000049176 Frmpd4 protein_coding 1.98 18.067 1.026 95.504 0.49 30.024 0.817 86.699
ENSMUSG00000049184 Purg protein_coding 1.071 54.344 0.972 93.654 0.964 81.169 0.908 83.724
ENSMUSG00000049191 Rgag4 protein_coding 1.155 0.577 0.969 90.744 0.894 15.904 1.028 82.328
ENSMUSG00000049202 4930515G13Rik pseudogene 1 64.714 1 91.661 1 86.602 1 88.387
ENSMUSG00000049214 Skint7 protein_coding 1 49.047 1 96.407 1 80.899 1 93.612
ENSMUSG00000049217 Olfr788 protein_coding 1 85.355 NA NA 1 84.83 1 93.261
ENSMUSG00000049225 Pdp1 protein_coding 1.038 40.065 0.999 95.211 0.962 36.248 1.105 63.708
ENSMUSG00000049229 Olfr844 protein_coding 1 84.865 1 95.504 1 88.601 1 94.654
ENSMUSG00000049230 Gm9833 protein_coding 2.213 57.38 0.88 93.765 0.514 74.853 0.606 91.625
ENSMUSG00000049231 Gm12497 pseudogene 1 73.487 NA NA 1 88.657 1 93.612
ENSMUSG00000049232 Tigd2 protein_coding 1.068 6.135 0.99 87.553 0.947 32.082 0.989 85.493
ENSMUSG00000049235 Gm7324 pseudogene 1 55.462 1 97.129 NA NA 1 94.608
ENSMUSG00000049241 Gpr81 protein_coding 0.759 8.751 1.272 79.864 1.032 73.428 0.78 83.724
ENSMUSG00000049246 Hspe1-ps2 pseudogene 1 75.115 1 97.049 1 82.161 1 95.74
ENSMUSG00000049249 Gm5316 protein_coding 1 74.8 1 97.129 1 88.274 1 95.676
ENSMUSG00000049265 Kcnk3 protein_coding 0.679 7.207 1.082 93.994

1.357 22.444 0.849 86.699
ENSMUSG00000049280 Olfr509 protein_coding 1 78.054 1
95.504 1 84.896 1 95.74
ENSMUSG00000049281 Scn3b protein_coding 1.786 2.723 1.047 95.708
0.533 3.234 0.907 94.016
ENSMUSG00000049285 Mblac1 protein_coding 1.044 83.523
0.932 91.532 1.024 79.426 0.929 84.19
ENSMUSG00000049287 Iba57 protein_coding 1.055 68.955 1.04
82.904 0.911 35.022 0.941 92.31
ENSMUSG00000049288 Lix1l protein_coding 1.304 0.577 NA NA 0.78
0.879 1.04 94.904
ENSMUSG00000049295 Zfp219 protein_coding 0.887 7.881 1.016
91.456 1.111 26.978 0.953 85.493
ENSMUSG00000049299 Trappc1 protein_coding 1.001 73.734
1.053 88.551 0.947 57.446 1.012 91.928
ENSMUSG00000049300 Prmt6 protein_coding 1.069 37.091 0.933
87.998 0.998 66.201 0.968 92.742
ENSMUSG00000049303 Syt12 protein_coding 0.746 4.895 1.162 83.104
1.152 41.331 1.041 95.74
ENSMUSG00000049305 Ccdc71 protein_coding 0.957 34.162 NA
NA 1.05 55.109 0.999 96.287
ENSMUSG00000049307 Fut4 protein_coding 0.992 69.985 1.091
72.642 0.926 73.096 1.137 71.008
ENSMUSG00000049313 Sorl1 protein_coding 0.763 4.268 1.06 97.155
1.233 13.082 0.913 85.493
ENSMUSG00000049314 Fbxw13 protein_coding 1 82.479 1
96.268 1 84.961 1 94.904
ENSMUSG00000049315 Olfr348 protein_coding 1 72.139 1
93.765 1 88.657 1 96.358
ENSMUSG00000049321 Zfp2 protein_coding 0.961 30.352 0.951
96.68 1.09 47.512 0.88 85.493
ENSMUSG00000049323 Smcr8 protein_coding 1.009 86.658 1.023
96.216 0.967 88.203 0.981 92.181
ENSMUSG00000049327 Setd8 protein_coding 0.84 1.239 1.155 61.943
1.029 57.637 0.955 89.294
ENSMUSG00000049334 Olfr902 protein_coding 1 77.291 1
97.263 1 85.352 1 96.287
ENSMUSG00000049336 Tenm2 protein_coding 1.242 11.638 NA NA
0.801 14.788 0.737 77.245
ENSMUSG00000049339 Fam134a protein_coding 1.006 62.689
1.021 89.434 0.97 23.938 0.956 83.724
ENSMUSG00000049353 Rd3 protein_coding 1.653 33.555 0.817
90.287 0.739 55.214 0.75 90.674
ENSMUSG00000049354 Dcaf7 protein_coding 1.141 0 0.957 67.165
0.91 8.313 0.962 90.805
ENSMUSG00000049357 4933408B17Rik protein_coding 0.846 36.823
0.968 92.474 1.215 28.74 1.091 96.183
ENSMUSG00000049362 Olfr173 protein_coding 1 85.129 1
97.097 1 70.44 1 94.904
ENSMUSG00000049372 Olfr1183 protein_coding 1 82.092 1
94.597 1 82.161 1 95.877
ENSMUSG00000049382 Krt8 protein_coding 0.994 75.196 0.876
63.773 1.151 67.823 1.091 93.964
ENSMUSG00000049387 Cox7b2 protein_coding 1 84.049 1

97.155 1 83.275 1 92.742
ENSMUSG00000049396 Gemin4 protein_coding 0.519 17.839
1.344 82.904 1.427 84.308 0.993 91.217
ENSMUSG00000049401 Ogfr protein_coding 1.02 57.655 1.011
92.404 0.973 77.294 1.053 82.328
ENSMUSG00000049404 Rarres1 protein_coding 0.993 85.203
1.214 97.263 0.828 78.713 0.912 92.181
ENSMUSG00000049409 Prokr1 protein_coding 1.368 16.249
1.395 79.367 0.523 9.563 0.899 88.245
ENSMUSG00000049410 Zfp683 protein_coding 0.881 86.918
1.932 93.146 0.589 58.775 0.814 89.892
ENSMUSG00000049411 Tmem241 protein_coding 1.06 38.386
1.007 97.129 0.938 40.338 0.98 86.991
ENSMUSG00000049414 Gm5417 pseudogene 1 85.129 1
95.708 1 85.675 1 93.413
ENSMUSG00000049420 Tmem200a protein_coding 1.122 84.819
1.112 83.104 0.8 44.019 0.821 84.29
ENSMUSG00000049421 Zfp260 protein_coding 1.175 1.441 0.943
93.046 0.9 3.073 0.971 93.156
ENSMUSG00000049422 Chchd10 protein_coding 0.831 41.617
1.201 86.387 1.002 87.519 1.014 96.183
ENSMUSG00000049436 Upk1b protein_coding 0.684 51.089 1.065
95.708 1.368 38.926 1.125 94.904
ENSMUSG00000049439 Cyp20a1 protein_coding 1.022 78.639 NA
NA 0.97 79.524 1.136 57.075
ENSMUSG00000049456 Olfr1404 protein_coding 1 86.843 1
97.263 1 84.83 1 96.287
ENSMUSG00000049460 Gm8396 pseudogene 1 78.744 1
97.097 1 86.144 1 96.358
ENSMUSG00000049470 Aff4 protein_coding 0.881 11.232 1.032
92.931 1.098 30.948 1.033 95.273
ENSMUSG00000049477 Gm8508 pseudogene 1 79.068 1
92.355 1 69.315 1 94.904
ENSMUSG00000049482 Ctu2 protein_coding 0.906 7.58 1 96.68
1.104 17.751 0.866 92.748
ENSMUSG00000049488 Tmem67 protein_coding 1.072 52.986
0.886 89.277 1.055 84.071 1.002 90.289
ENSMUSG00000049489 Fam58b protein_coding 1.021 76.319
1.024 95.708 0.954 87.336 1.015 93.016
ENSMUSG00000049493 Pls1 protein_coding 1.125 30.984 0.81
50.715 1.093 83.066 1.196 87.624
ENSMUSG00000049494 Gm12669 pseudogene 1 68.955 1
94.367 1 87.744 1 96.086
ENSMUSG00000049498 Olfr1449 protein_coding 1 82.982 1
93.899 1 86.475 1 94.904
ENSMUSG00000049502 Dtx3l protein_coding 1.429 0 0.934 91.532
0.75 0.879 0.878 87.331
ENSMUSG00000049504 Proser1 protein_coding 1.004 86.006
1.012 94.89 0.987 85.675 0.946 85.493
ENSMUSG00000049506 Sppl2c protein_coding 1 84.324 1
95.708 1 88.601 1 95.74
ENSMUSG00000049507 4930429B21Rik protein_coding 1.037 75.671
0.794 85.95 1.209 81.032 1.062 94.608
ENSMUSG00000049511 Htr1b protein_coding 1.56 20.942 1.019

91.361	0.627	12.948	0.753	85.493
ENSMUSG00000049515	Espnl	protein_coding	0.857	65.106	1.019
95.504	1.148	70.9	0.761	89.892
ENSMUSG00000049516	Spty2d1	protein_coding	1.099	1.242	1.033
81.039	0.882	0.72	1.004	96.071
ENSMUSG00000049517	Rps23	protein_coding	1.115	0.577	0.931	21.315
0.96	25.625	0.977	89.892
ENSMUSG00000049521	Cdc42ep1	protein_coding	0.972	61.887
1.138	71.222	0.903	42.895	NA	NA
ENSMUSG00000049526	Tmem202	protein_coding	0.784	39.612
0.962	95.504	1.322	53.882	1.256	94.904
ENSMUSG00000049528	Olfr429	protein_coding	1	85.728	1
95.431	1	86.346	1	96.358
ENSMUSG00000049530	Clrn2	protein_coding	1	76.968	NA	NA
1	79.524	1	95.832
ENSMUSG00000049532	Sall2	protein_coding	0.948	31.921	0.986
96.216	1.067	43.307	1.021	95.676
ENSMUSG00000049536	Tceal1	protein_coding	1.051	62.007
0.972	91.532	0.98	64.778	0.916	85.493
ENSMUSG00000049537	Tecrl	protein_coding	1	72.372	1
95.211	1	67.962	1	94.384
ENSMUSG00000049538	Adamts16	protein_coding	1.952	26.863
0.809	95.708	0.635	63.919	1.021	94.608
ENSMUSG00000049548	Krt82	protein_coding	1	83.263	1
92.137	1	88.076	1	83.581
ENSMUSG00000049550	Clip1	protein_coding	0.936	35.449	1.045
87.029	1.018	64.024	1.084	32.698
ENSMUSG00000049551	Fzd9	protein_coding	1.756	19.746	NA	NA
0.69	19.236	0.902	93.627
ENSMUSG00000049553	Polr1a	protein_coding	0.965	81.647
0.981	93.094	1.058	74.325	1.126	44.882
ENSMUSG00000049555	Tmie	protein_coding	0.825	22.237	0.783
75.345	1.553	15.241	0.853	83.724
ENSMUSG00000049556	Lingo1	protein_coding	1.233	41.951
1.229	83.104	0.66	22.009	1.06	83.067
ENSMUSG00000049560	Defb20	protein_coding	1	56.477	1
96.847	1	86.144	1	95.832
ENSMUSG00000049561	Olfr95	protein_coding	1	83.823	1
90.378	1	88.076	1	93.264
ENSMUSG00000049562	Ap5b1	protein_coding	0.979	73.292	1.089
88.91	0.936	52.087	1.03	93.045
ENSMUSG00000049565	Aknad1	protein_coding	1.485	4.925	0.944
97.288	0.712	5.042	1.072	96.358
ENSMUSG00000049571	9330101J02Rik	protein_coding	1.115	62.574
0.851	81.731	1.057	71.615	0.792	83.724
ENSMUSG00000049573	Olfr780	protein_coding	1	78.909	NA
NA	1	85.792	1	95.72
ENSMUSG00000049576	Zfa-ps	pseudogene	1	83.169	1
87.322	1	72.148	1	80.497
ENSMUSG00000049577	Zfpm1	protein_coding	0.963	83.523	1.106
88.823	0.94	50.184	0.933	83.724
ENSMUSG00000049580	Tsku	protein_coding	1.06	79.853	0.79
72.399	1.19	40.875	1.397	31.133
ENSMUSG00000049583	Grm5	protein_coding	1	76.135	1

						95.35	1	79.554	1	77.245
ENSMUSG00000049588	Ccdc69	protein_coding	1.041	71.896	1.127	87.282	0.853	28.237	0.915	83.724
ENSMUSG00000049593	Lce1h	protein_coding	1	85.59	1	95.301	1	81.62	1	93.457
ENSMUSG00000049598	Vsig8	protein_coding	1.775	33.325	0.537	64.555	1.047	56.931	1.006	94.001
ENSMUSG00000049600	Zbtb45	protein_coding	1.059	61.171	1.042	79.29	0.906	11.997	1.012	95.832
ENSMUSG00000049604	Hoxb13	protein_coding	1	81.76	1	95.211	1	81.29	1	95.593
ENSMUSG00000049605	Olfr418-ps1	protein_coding	1	78.744	1	95.504	1	87.898	1	93.156
ENSMUSG00000049606	Zfp644	protein_coding	1.059	1.81	1.053	97.155	0.892	16.051	0.953	83.724
ENSMUSG00000049608	Gpr55	protein_coding	1.643	66.944	0.46	78.471	1.326	70.1	0.716	95.74
ENSMUSG00000049618	Olfr103	protein_coding	1	64.193	1	94.96	1	85.352	1	94.904
ENSMUSG00000049624	Slc17a5	protein_coding	1.003	67.163	0.987	94.48	NA	NA	0.866	77.245
ENSMUSG00000049625	Tifab	protein_coding	1.508	3.176	0.91	68.115	0.728	44.865	0.931	95.273
ENSMUSG00000049630	C1ql3	protein_coding	1.357	76.827	0.723	95.211	1.02	81.29	1.174	96.192
ENSMUSG00000049643	2310022A10Rik	protein_coding	1.119	2.969	0.998	95.504	0.896	8.907	1.099	70.539
ENSMUSG00000049648	Olfr273	protein_coding	1	77.615	1	96.268	1	83.066	1	94.016
ENSMUSG00000049649	Gpr3	protein_coding	0.808	51.56	1.04	96.216	1.194	53.882	1.039	93.457
ENSMUSG00000049653	Spatc1	protein_coding	0.674	11.924	1.342	83.104	1.107	41.6	1.188	96.358
ENSMUSG00000049657	Zbtb5	protein_coding	1.104	32.058	0.871	45.262	1.039	56.708	0.869	83.952
ENSMUSG00000049658	Bdp1	protein_coding	0.948	55.303	NA	NA	1.089	72.695	1.015	95.35
ENSMUSG00000049659	Aftph	protein_coding	1.066	7.083	0.943	60.678	0.996	73.531	1.022	93.734
ENSMUSG00000049661	Mtag2	processed_transcript	1	86.333	1	97.155	1	75.009	1	94.904
ENSMUSG00000049670	Morn4	protein_coding	0.938	74.482	0.992	93.654	1.077	70.328	0.865	83.724
ENSMUSG00000049672	Zbtb14	protein_coding	0.882	24	1.053	95.301	1.073	48.451	1.115	37.634
ENSMUSG00000049674	Olfr714	protein_coding	1	86.172	1	97.263	1	84.724	1	96.183
ENSMUSG00000049676	Catsperg1	protein_coding	1.057	86.57	0.957	91.361	0.99	71.232	0.807	84.29
ENSMUSG00000049680	Urgcp	protein_coding	0.965	53.138	NA	NA	1.017	67.562	1	94.468
ENSMUSG00000049685	Cyp2g1	protein_coding	1	85.728	1	95.211	1	78.293	1	94.904
ENSMUSG00000049686	Orai1	protein_coding	1.232	1.395	1.01	97.263				

0.804 1.061 0.993 93.612
ENSMUSG00000049687 Fam109b protein_coding 1.221 33.325
0.858 79.261 0.956 73.096 0.998 83.067
ENSMUSG00000049690 Nckap5 protein_coding 0.883 50.519
0.786 62.692 1.442 13.259 0.893 83.724
ENSMUSG00000049694 BC048671 protein_coding 1 81.647 1
94.848 1 84.308 1 94.99
ENSMUSG00000049699 Ucn2 protein_coding 0.501 14.526 1.537
94.367 1.298 22.93 1.182 92.263
ENSMUSG00000049708 NA NA NA NA 1 95.211 1
88.076 1 93.132
ENSMUSG00000049709 Nlrp10 protein_coding 1.123 74.147
0.906 85.921 0.986 85.352 0.977 96.358
ENSMUSG00000049717 Lig4 protein_coding 1.023 76.135 1.007
89.996 0.973 62.791 1.015 88.657
ENSMUSG00000049721 Gal3st1 protein_coding 1.053 75.671
1.093 83.104 0.866 49.453 0.872 92.31
ENSMUSG00000049723 Mmp12 protein_coding 1.233 14.59 1.279 95.088
0.636 10.934 1.154 95.273
ENSMUSG00000049728 Zfp668 protein_coding 0.999 81.823
0.987 88.691 1.01 50.091 1.044 86.133
ENSMUSG00000049734 Trex1 protein_coding 1 84.294 1
95.211 1 80.381 1 94.825
ENSMUSG00000049737 Olfr1361 protein_coding 1 80.751 1
95.504 1 83.066 1 95.832
ENSMUSG00000049739 Zfp646 protein_coding 1.028 47.174
1.045 77.598 0.929 13.202 0.97 95.593
ENSMUSG00000049740 5330417H12Rik protein_coding 1 56.242
1 96.308 1 84.724 1 86.133
ENSMUSG00000049741 Rxfp4 protein_coding 1 51.056 1
96.407 1 79.625 1 83.067
ENSMUSG00000049742 C330006D17Rik protein_coding 0.76 16.183
0.93 93.908 1.418 16.808 1.008 92.31
ENSMUSG00000049744 Arhgap15 protein_coding 1.464 0.2 0.918
82.947 0.744 8.382 0.983 94.468
ENSMUSG00000049751 Rpl36al protein_coding 0.984 68.846
0.995 93.654 1.018 62.995 1.066 76.046
ENSMUSG00000049755 Zfp672 protein_coding 1.005 79.577
1.159 79.864 0.859 6.858 0.989 96.086
ENSMUSG00000049758 Olfr1318 protein_coding 1 86.57 1
96.013 1 86.831 1 94.654
ENSMUSG00000049760 2410015M20Rik protein_coding 0.794 1.15
1.064 79.411 1.183 10.819 1.027 87.134
ENSMUSG00000049761 Pmis2 protein_coding 0.843 70.597 0.502
95.431 2.37 77.888 1.829 95.832
ENSMUSG00000049764 Zfp280b protein_coding 0.922 32.749
1.068 83.104 1.012 86.985 1.026 60.968
ENSMUSG00000049775 Tmsb4x protein_coding 1.016 56.085
0.907 35.072 1.085 37.764 1.038 95.593
ENSMUSG00000049791 Fzd4 protein_coding 0.995 82.982 1.073
96.652 0.935 65.688 1.038 92.762
ENSMUSG00000049792 Bag5 protein_coding 1.065 21.703 0.978
84.594 0.957 39.079 0.961 83.724
ENSMUSG00000049796 Crh protein_coding 1 76.686 1

96.897 1 86.831 1 95.74
ENSMUSG00000049797 Olfr642 protein_coding 1 81.925 1
96.216 1 87.744 1 93.132
ENSMUSG00000049799 Lrrc19 protein_coding 1 82.479 1
87.857 1 76.754 1 92.748
ENSMUSG00000049800 Sertad2 protein_coding 1.021 65.537
0.955 93.654 NA NA 0.949 85.493
ENSMUSG00000049804 Armcx4 protein_coding 1.011 84.819
0.873 71.41 1.135 39.439 1.076 91.525
ENSMUSG00000049806 Olfr466 protein_coding 1 81.52 NA NA
1 79.255 1 93.132
ENSMUSG00000049807 Arhgap23 protein_coding 0.95 55.303
1.121 82.904 0.942 87.898 0.992 88.755
ENSMUSG00000049809 Krtap9-3 protein_coding 1 85.59 1
95.211 1 85.792 1 96.183
ENSMUSG00000049811 Fam161a protein_coding 1.055 34.861
0.936 87.919 1.015 80.015 0.874 87.735
ENSMUSG00000049815 4921511C20Rik protein_coding 1 86.145
1 97.263 1 81.976 1 94.904
ENSMUSG00000049823 Zbtb12 protein_coding 1.009 86.918
1.048 83.104 0.949 40.19 0.968 86.445
ENSMUSG00000049830 Gm9839 pseudogene 1 78.299 1
95.735 1 86.112 1 94.82
ENSMUSG00000049832 Gm9840 pseudogene 1 79.93 1 94.48 1
87.103 1 96.183
ENSMUSG00000049843 Olfr1102 protein_coding 1 82.705 1
95.708 1 88.601 1 92.748
ENSMUSG00000049848 Ceacam19 protein_coding 2.17 5.481 0.611
60.678 0.755 59.922 1.041 86.699
ENSMUSG00000049858 Suox protein_coding 0.926 63.126 0.901
62.692 1.197 15.241 1.009 93.457
ENSMUSG00000049864 Olfr250 protein_coding 1 80.51 1
94.887 1 79.119 1 93.722
ENSMUSG00000049866 Arl4c protein_coding 0.938 38.555 1.161
66.01 0.921 31.887 0.958 84.19
ENSMUSG00000049871 Nlrc3 protein_coding 1.109 62.354 1.036
88.092 0.867 32.703 0.847 86.699
ENSMUSG00000049872 Fam26e protein_coding 1.356 4.465 0.966
95.504 0.763 4.23 0.963 89.498
ENSMUSG00000049878 Rlf protein_coding 1.05 30.497 0.917
37.426 1.038 37.369 1.012 95.877
ENSMUSG00000049881 2810025M15Rik protein_coding 1.021 41.211
0.98 83.58 1.003 88.657 1.069 64.513
ENSMUSG00000049882 Mettl21d protein_coding 1.107 18.388
0.931 60.678 0.97 65.567 1.124 62.594
ENSMUSG00000049891 Gm7984 pseudogene 1 52.799 1
91.955 1 41.928 1 92.181
ENSMUSG00000049892 Rasd1 protein_coding 1.297 17.336 0.973
94.48 0.792 49.967 1.37 49.514
ENSMUSG00000049894 Olfr808 protein_coding 1 77.497 NA
NA 1 88.461 1 94.904
ENSMUSG00000049897 Gm711 protein_coding 0.685 25.551 1.117
93.27 1.304 40.511 1.168 95.676
ENSMUSG00000049902 4921517D22Rik protein_coding 1 77.291

1 96.138 1 88.349 1 91.979
ENSMUSG00000049904 Tmem17 protein_coding 1.036 86.333
1.168 82.103 0.826 54.964 0.943 96.358
ENSMUSG00000049907 Rasl11b protein_coding 0.867 41.617
1.108 83.104 1.04 88.601 0.98 95.676
ENSMUSG00000049916 2610318N02Rik protein_coding 3.77 15.662
0.181 41.141 1.458 63.801 0.906 93.016
ENSMUSG00000049922 Slc35c1 protein_coding 1.003 77.291
1.033 90.29 0.968 61.288 1.06 83.276
ENSMUSG00000049928 Glp2r protein_coding 1.235 47.847 1.014
93.765 0.797 70.791 0.944 96.086
ENSMUSG00000049929 Lpar4 protein_coding 1.054 82.732 0.893
95.504 1.063 88.601 0.877 84.29
ENSMUSG00000049932 H2afx protein_coding 0.961 75.567 1.111
83.104 0.932 64.778 0.996 87.624
ENSMUSG00000049939 Lrrc4 protein_coding 2.168 0 0.921 67.513
0.499 0.72 0.874 84.29
ENSMUSG00000049940 Pgrmc2 protein_coding 0.99 74.147
1.056 83.104 0.957 52.087 0.988 83.952
ENSMUSG00000049946 BC030500 protein_coding 1 77.916 1
96.268 1 84.724 1 95.273
ENSMUSG00000049950 Rpp38 protein_coding 1.037 73.124 NA NA
1 75.967 1.075 71.305
ENSMUSG00000049957 Ccdc137 protein_coding 0.927 63.736 NA
NA 1.021 82.778 1.048 80.77
ENSMUSG00000049960 Mrps16 protein_coding 0.896 41.021 1.08
83.104 1.033 80.381 1.099 86.445
ENSMUSG00000049969 Plekhf2 protein_coding 1.112 16.436
0.973 88.704 0.919 38.534 0.997 93.627
ENSMUSG00000049971 Glt1d1 protein_coding 0.637 0.987 0.847
69.175 1.858 5.864 NA NA
ENSMUSG00000049972 Skint9 protein_coding 1 84.564 1
96.216 1 88.161 1 91.492
ENSMUSG00000049985 Ankrd55 protein_coding 1.426 32.058
1.062 87.282 0.66 11.751 1.148 86.118
ENSMUSG00000049988 Lrrc25 protein_coding 1.716 0 0.927
95.088 0.629 2.144 1.164 67.691
ENSMUSG00000049999 Ppp1r3d protein_coding 0.969 75.671
1.429 65.165 0.72 0.72 1.135 93.132
ENSMUSG00000050002 Idnk protein_coding 0.98 65.388 1.057
89.434 0.966 82.575 0.925 83.724
ENSMUSG00000050010 Shisa3 protein_coding 0.916 48.354 1.08
95.809 1.01 85.792 0.878 83.724
ENSMUSG00000050014 Apol10b protein_coding 1.009 58.802
1.025 83.104 0.968 73.874 0.805 83.724
ENSMUSG00000050015 Olfr350 protein_coding 1 79.709 1
97.129 1 84.896 1 95.832
ENSMUSG00000050017 Pitpnb protein_coding 1.055 67.991
0.978 97.263 0.969 74.179 1.016 87.134
ENSMUSG00000050022 Amz1 protein_coding 0.804 14.221 1.073
86.277 1.16 40.955 1.078 96.183
ENSMUSG00000050023 Olfr1176 protein_coding 1 82.705 1
95.211 1 80.899 1 95.405
ENSMUSG00000050028 Olfr745 protein_coding 1 77.497 1

97.155 1 87.103 1 93.132
ENSMUSG00000050029 Rap2c protein_coding 1.178 1.828 0.964 97.263
0.878 3.428 1.019 84.499
ENSMUSG00000050030 Olfr728 protein_coding 1 76.396 1
96.335 1 86.831 1 95.593
ENSMUSG00000050035 Fhl4 protein_coding 1.425 13.492 0.747
65.165 0.934 81.169 0.647 85.493
ENSMUSG00000050043 Tmx2 protein_coding 0.972 60.707 1.032
84.768 0.992 86.552 0.995 88.197
ENSMUSG00000050050 Ccdc158 protein_coding 1.098 50.627
0.847 71.889 1.072 78.498 0.894 92.181
ENSMUSG00000050052 Tdrp protein_coding 0.754 8.405 1.241 71.98
1.066 64.624 0.937 93.612
ENSMUSG00000050058 Prm3 protein_coding 1 84.324 NA NA
1 88.461 1 96.192
ENSMUSG00000050063 Klk6 protein_coding 1 85.946 1
96.847 1 81.388 1 90.177
ENSMUSG00000050064 Zfp697 protein_coding 0.996 82.231
1.366 82.904 0.737 27.86 0.867 83.724
ENSMUSG00000050066 A130023I24Rik sense_intronic 1 80.688
1 95.504 1 81.29 1 95.74
ENSMUSG00000050069 Grem2 protein_coding 1.082 30.497 1.776
71.617 0.521 3.943 1.479 43.377
ENSMUSG00000050071 Bex1 protein_coding 0.884 42.361 0.891
94.102 1.271 44.515 1.066 95.273
ENSMUSG00000050074 Spink8 protein_coding 1 82.732 1
97.097 1 86.666 1 94.99
ENSMUSG00000050075 Gpr171 protein_coding 1.019 65.284 1.26
83.104 0.777 27.86 0.89 92.675
ENSMUSG00000050079 Rspry1 protein_coding 1.123 1.292 1.017
93.994 0.876 3.147 1.009 92.018
ENSMUSG00000050085 Olfr622 protein_coding 1 70.121 1
95.301 1 73.759 1 96.183
ENSMUSG00000050088 1600012H06Rik protein_coding 0.983 83.726
1 86.071 1.02 79.554 0.959 85.493
ENSMUSG00000050089 Akap4 protein_coding 1 59.674 1
97.263 1 79.625 1 94.752
ENSMUSG00000050092 Sprr2b protein_coding 1 86.74 NA NA
1 83.607 1 92.181
ENSMUSG00000050097 Ces2b protein_coding 1.526 45.746 0.876
90.287 0.748 71.004 0.806 84.29
ENSMUSG00000050100 Hmx2 protein_coding 1 74.689 1
96.268 1 85.58 1 95.273
ENSMUSG00000050102 Vmn1r235 protein_coding 1 73.585 1
92.631 1 86.985 1 93.457
ENSMUSG00000050103 Agmo protein_coding 1.176 27.731 0.905
86.728 0.935 62.058 0.892 88.053
ENSMUSG00000050105 Grrp1 protein_coding 0.978 81.823 1.068
93.654 0.957 76.923 1.063 85.629
ENSMUSG00000050106 Tmc8 protein_coding 0.836 23.556 1.045
96.268 1.146 32.173 0.975 85.493
ENSMUSG00000050107 Gsg2 protein_coding 1.294 57.38 1.446 72.546
0.533 15.904 1.014 92.748
ENSMUSG00000050114 Prdx6b protein_coding 1 80.03 1

95.088 1 83.397 1 95.676
ENSMUSG00000050121 Opalin protein_coding 1 83.658 1
95.211 1 88.601 1 95.273
ENSMUSG00000050122 Vwa3b protein_coding 1.032 70.121 1.081
96.268 0.893 84.552 0.966 96.358
ENSMUSG00000050128 Olfr1023 protein_coding 1 71.896 1
95.431 1 79.897 1 94.904
ENSMUSG00000050132 Sarm1 protein_coding 1.431 28.693 1.135
83.104 0.614 6.858 0.805 88.245
ENSMUSG00000050134 Olfr430 protein_coding 1 78.639 NA
NA 1 83.158 1 96.139
ENSMUSG00000050144 Slc25a44 protein_coding 0.951 37.531
1.017 93.27 1.031 79.119 1.055 84.31
ENSMUSG00000050147 F2rl3 protein_coding 0.707 15.76 1.305 71.98 1.08
77.294 1.087 93.045
ENSMUSG00000050148 Ubqln2 protein_coding 1.091 4.159 NA NA
0.902 6.858 0.947 84.29
ENSMUSG00000050150 Slc9b1 protein_coding 0.573 51.056
2.167 85.756 0.802 87.431 1.902 64.513
ENSMUSG00000050157 Gm867 protein_coding 1.044 60.435 0.824
97.155 1.161 63.075 0.693 83.724
ENSMUSG00000050158 Olfr165 protein_coding 1 85.499 NA
NA 1 87.971 1 92.691
ENSMUSG00000050164 Mchr1 protein_coding 0.554 21.176 0.879
79.261 2.058 11.518 1.501 79.268
ENSMUSG00000050174 Nudt6 protein_coding 0.877 17.898 1.055
97.263 1.08 32.173 0.993 91.291
ENSMUSG00000050179 A930002I21Rik lincRNA 1 74.516 1
95.708 1 84.896 1 94.904
ENSMUSG00000050188 Lsm10 protein_coding 0.955 51.56 1.06 90.116
0.989 84.552 1.027 93.156
ENSMUSG00000050189 Etos1 protein_coding 1 86.843 1
96.138 1 88.349 1 91.291
ENSMUSG00000050190 Gm5346 protein_coding 1 86.112 1
97.097 1 86.475 1 95.273
ENSMUSG00000050192 Eif5a2 protein_coding 0.996 75.196
0.885 82.503 1.13 62.995 1.053 79.268
ENSMUSG00000050195 Scd4 protein_coding 0.999 65.106 0.599
35.895 1.67 5.864 0.906 95.74
ENSMUSG00000050197 Rhox13 protein_coding 1 85.59 1
96.68 1 88.349 1 92.762
ENSMUSG00000050198 Olfr768 protein_coding 1 82.891 1
95.708 1 84.308 1 91.372
ENSMUSG00000050199 Lgr4 protein_coding 1.038 69.777 1.114
87.037 0.865 37.103 0.908 86.991
ENSMUSG00000050201 Otop2 protein_coding 1 55.303 1
96.843 1 65.567 1 96.192
ENSMUSG00000050211 Pla2g4e protein_coding 5.07 10.595 NA
NA 0.117 4.521 1.02 93.964
ENSMUSG00000050212 Eva1b protein_coding 1.026 81.196 0.979
93.469 0.996 86.985 0.958 84.29
ENSMUSG00000050213 Snip1 protein_coding 1.101 2.784 0.983 96.013
0.925 5.336 0.945 84.29
ENSMUSG00000050215 Olfr70 protein_coding 1 85.832 NA

NA 1 81.169 1 95.74
ENSMUSG00000050222 Il17d protein_coding 1.022 78.054 1.072
95.196 0.916 63.075 1.002 93.612
ENSMUSG00000050224 Krtap13 protein_coding 1 76.968 1
97.155 NA NA 1 94.904
ENSMUSG00000050229 Pigm protein_coding 0.986 65.388 0.912
72.576 1.113 11.348 0.982 89.892
ENSMUSG00000050232 Cxcr3 protein_coding 1.202 27.545 1.091
91.643 0.764 15.376 0.842 85.493
ENSMUSG00000050234 Gja4 protein_coding 0.93 60.569 0.819
87.322 1.31 35.13 1.1 92.691
ENSMUSG00000050239 Krtap24-1 protein_coding 1 82.479 1
94.597 1 67.823 1 93.261
ENSMUSG00000050240 Hic2 protein_coding 1.034 65.869 0.857
59.946 1.128 25.455 0.987 93.117
ENSMUSG00000050241 Klre1 protein_coding 0.845 59.358 1.765
61.032 0.671 32.963 1.044 94.654
ENSMUSG00000050243 Gm5446 pseudogene 1.151 82.479 2.556
72.642 0.338 25.688 1.028 94.19
ENSMUSG00000050244 Heatr1 protein_coding 0.978 84.564
1.039 95.708 NA NA 1.075 77.621
ENSMUSG00000050248 Evc2 protein_coding 1.078 26.863 0.945
71.41 0.983 83.275 1.03 84.246
ENSMUSG00000050251 Olfr809 protein_coding 1 85.283 1
94.887 1 83.538 1 96.287
ENSMUSG00000050266 Olfr690 protein_coding 1 82.891 1
96.847 1 88.693 1 84.499
ENSMUSG00000050270 Tmem220 protein_coding 1.153 7.179 0.865
72.399 0.998 78.713 0.9 83.724
ENSMUSG00000050271 D8Ertd82e protein_coding 0.808 49.047 1.2
83.464 1.031 77.294 1.26 96.183
ENSMUSG00000050272 Dscam protein_coding 0.388 9.341 2.152 65.165
1.195 63.801 2.856 64.513
ENSMUSG00000050276 Mrgprg protein_coding 0.27 3.559 0.281
47.831 13.123 5.864 0.961 92.748
ENSMUSG00000050281 Olfr630 protein_coding 1 86.464 1
97.155 1 88.349 1 93.117
ENSMUSG00000050288 Fzd2 protein_coding 1.19 7.397 1.122 92.062
0.746 1.48 0.958 86.699
ENSMUSG00000050295 Foxc1 protein_coding 1.026 57.38 1.005 97.288
0.971 56.625 0.878 83.724
ENSMUSG00000050296 Abca12 protein_coding 1.037 75.761
1.168 94.367 0.823 80.749 0.446 83.724
ENSMUSG00000050299 Gm9843 protein_coding 7.062 0 1.612
90.29 0.088 0.72 0.959 90.854
ENSMUSG00000050304 Slc25a2 protein_coding 0.757 59.674
0.727 90.256 1.814 30.292 0.906 96.183
ENSMUSG00000050310 Rictor protein_coding 0.849 6.822 1.001
93.654 1.176 5.864 0.952 85.493
ENSMUSG00000050312 Nsun3 protein_coding 0.948 51.089 1.015
93.046 1.035 51.851 0.951 89.892
ENSMUSG00000050315 Synpo2 protein_coding 1.149 17.191
0.953 86.861 0.911 44.211 1.134 67.691
ENSMUSG00000050321 Neto1 protein_coding 1.36 36.438 1.143

96.763 0.643 27.675 1.323 71.855
ENSMUSG00000050323 Ndufaf6 protein_coding 0.995 82.543
0.952 95.504 1.057 68.129 1.017 83.067
ENSMUSG00000050325 1700034J04Rik protein_coding 1 85.637
1 97.155 1 81.525 1 94.608
ENSMUSG00000050328 Hoxc12 protein_coding 1 71.915 1
89.434 1 85.134 1 96.248
ENSMUSG00000050332 Amer1 protein_coding 0.968 50.768 0.983
85.756 1.053 87.173 0.939 83.952
ENSMUSG00000050335 Lgals3 protein_coding 0.837 13.239
1.036 96.68 1.155 26.498 1.192 44.679
ENSMUSG00000050343 Olfr1378 protein_coding 1 85.728 1
91.83 1 80.5 1 95.273
ENSMUSG00000050345 4930486L24Rik protein_coding 0.99 56.242
0.87 67.839 1.158 10.819 0.987 63.708
ENSMUSG00000050347 Gm9844 protein_coding 1 84.294 1
93.094 1 71.388 1 94.904
ENSMUSG00000050350 Gpr18 protein_coding 1.022 83.968 1.578
92.111 0.621 46.071 0.76 83.724
ENSMUSG00000050357 Rltpr protein_coding 0.824 45.728 1.083
93.574 1.122 66.269 0.915 86.991
ENSMUSG00000050359 Sprr1a protein_coding 2.276 12.72 0.909
93.654 0.483 33.402 1.1 84.246
ENSMUSG00000050366 Olfr524 protein_coding 1 76.686 1
97.263 NA NA 1 95.273
ENSMUSG00000050368 Hoxd10 protein_coding 0.806 40.422
1.015 83.104 1.221 63.801 1.092 91.335
ENSMUSG00000050370 Ch25h protein_coding 1.441 8.102 1.308 77.618
0.531 2.687 1 94.904
ENSMUSG00000050373 Snx21 protein_coding 1.008 86.351 0.961
92.137 1.03 74.325 0.886 83.724
ENSMUSG00000050377 Il31ra protein_coding 0.813 83.263
1.143 86.156 1.078 69.583 2.08 65.558
ENSMUSG00000050379 6-Sep protein_coding 1.147 66.944 1.023
82.904 0.854 39.755 0.92 91.078
ENSMUSG00000050382 Kif7 protein_coding 0.965 69.985 1.014
96.268 1.024 77.17 1.058 80.77
ENSMUSG00000050383 Svs3b protein_coding 1 85.728 1
96.216 1 80.381 1 96.071
ENSMUSG00000050390 C77080 protein_coding 0.873 22.481
1.017 96.308 1.128 40.511 0.865 85.493
ENSMUSG00000050394 Armcx6 protein_coding 1.06 58.38 0.978
95.211 0.962 85.21 0.94 96.184
ENSMUSG00000050395 Tnfsf15 protein_coding 1.145 65.284
1.365 88.91 0.637 42.895 0.904 92.31
ENSMUSG00000050410 Tcf19 protein_coding 1.222 7.842 0.962 85.921
0.853 27.675 1.206 63.708
ENSMUSG00000050423 Ppp1r3g protein_coding 1.304 67.991 0.66
78.877 1.164 67.823 0.79 94.608
ENSMUSG00000050425 Mrgprb2 protein_coding 1.442 8.477 0.807
79.475 0.86 38.799 1.065 95.92
ENSMUSG00000050428 Fbxo46 protein_coding 1.047 59.806
1.023 92.561 0.929 13.643 0.869 83.724
ENSMUSG00000050431 Olfr730 protein_coding 1 72.85 1

94.48 1 80.015 1 94.99
ENSMUSG00000050435 Ube2dnl1 protein_coding 1 82.092 1
96.001 1 86.258 1 94.99
ENSMUSG00000050445 Cyp8b1 protein_coding 0.76 64.589
0.822 96.138 1.589 49.058 1.191 96.183
ENSMUSG00000050447 Lypd6 protein_coding 1.346 37.941 1.05
93.899 0.705 25.688 0.56 92.12
ENSMUSG00000050463 Krt78 protein_coding 0.914 74.745 0.63
12.804 1.738 19.025 1.148 95.35
ENSMUSG00000050468 Astl protein_coding 1 66.944 NA NA
1 80.749 1 96.287
ENSMUSG00000050471 Fam118b protein_coding 1.065 49.517
0.996 95.211 0.944 38.534 1.031 76.697
ENSMUSG00000050473 Slc35d3 protein_coding 0.707 15.456
0.909 88.704 1.553 13.709 0.667 83.724
ENSMUSG00000050478 Olfr807 protein_coding 1 81.647 1
96.216 1 78.155 1 95.593
ENSMUSG00000050490 Gm8394 protein_coding 1 77.497 1
96.268 1 82.685 1 95.72
ENSMUSG00000050493 Fam167b protein_coding 1.159 59.806
1.108 92.111 0.779 23.212 0.967 92.675
ENSMUSG00000050503 Fbxl22 protein_coding 0.992 80.688
1.196 80.806 0.842 10.602 1.039 96.287
ENSMUSG00000050504 Olfr1323 protein_coding 1 72.139 NA
NA 1 88.41 1 96.192
ENSMUSG00000050505 Pcdh20 protein_coding 0.962 86.145 1.33
82.904 0.781 22.864 1.047 90.177
ENSMUSG00000050520 Cldn8 protein_coding 1.918 0 0.969 85.793
0.537 6.68 1.005 95.273
ENSMUSG00000050530 Fam171a1 protein_coding 1.019 54.717
0.979 88.345 0.998 86.258 1.082 43.377
ENSMUSG00000050533 Gm9845 protein_coding 0.659 60.569 NA
NA 1.43 30.561 0.973 91.525
ENSMUSG00000050538 B230217C12Rik processed_transcript 0.843 74.8
0.745 80.463 1.592 47.512 1.723 52.324
ENSMUSG00000050541 Adra1b protein_coding 0.97 86.172
0.892 72.576 1.153 46.956 1.042 86.445
ENSMUSG00000050545 Fam228b protein_coding 1.234 72.85 0.693
74.483 1.17 57.637 0.859 88.505
ENSMUSG00000050549 5730508B09Rik protein_coding 0.895 39.612
1.316 62.898 0.846 24.918 0.98 88.387
ENSMUSG00000050550 Gm11868 pseudogene 1 85.857 1
94.597 1 83.925 1 94.82
ENSMUSG00000050552 Lamtor4 protein_coding 0.872 0.987 1.018
83.104 1.127 22.93 1.014 94.297
ENSMUSG00000050553 Gk2 protein_coding 1 80.408 1
96.216 1 80.5 1 96.287
ENSMUSG00000050555 Hyls1 protein_coding 0.996 86.658 0.942
87.322 1.064 76.923 1.069 86.209
ENSMUSG00000050556 Kcnb1 protein_coding 0.714 4.993 1.25 75.108
1.122 55.109 1.019 93.964
ENSMUSG00000050565 Tor1aip2 protein_coding 1.062 12.549
0.994 95.301 0.95 14.262 1.061 87.7
ENSMUSG00000050567 Maml1 protein_coding 1.009 72.372 1.083

						71.617	0.916	5.042	0.96	86.445
ENSMUSG00000050577	Olfr1038-ps	pseudogene	1	81.657	1	97.097	1	83.925	1	95.273
ENSMUSG00000050578	Mmp13	protein_coding	1.13	66.944	0.972	97.263	0.909	49.217	1.244	96.183
ENSMUSG00000050587	Lrrc4c	protein_coding	1.091	39.851	0.962	94.848	0.951	71.388	1.099	95.92
ENSMUSG00000050592	Fam78a	protein_coding	1.481	0.2	1.01	91.776	0.666	0.72	0.968	83.276
ENSMUSG00000050600	Zfp831	protein_coding	0.921	57.227	1.43	93.707	0.761	88.076	0.768	84.29
ENSMUSG00000050603	Olfr1008	protein_coding	1	84.049	1	92.355	1	87.431	1	92.389
ENSMUSG00000050604	3110021A11Rik	protein_coding	0.973	74.8	0.959	93.994	1.072	88.461	1.017	96.248
ENSMUSG00000050605	Zfp61	protein_coding	0.974	29.725	0.918	81.731	1.12	10.819	1.021	78.546
ENSMUSG00000050608	Minos1	protein_coding	0.936	14.221	0.973	93.654	1.098	22.517	0.998	89.108
ENSMUSG00000050613	Olfr125	protein_coding	1	85.637	1	95.35	1	75.637	1	94.904
ENSMUSG00000050619	Zscan29	protein_coding	0.975	65.199	0.98	85.793	1.048	46.206	0.966	93.045
ENSMUSG00000050623	Tex40	protein_coding	0.458	18.379	1.445	96.897	1.512	24.375	1.316	88.512
ENSMUSG00000050627	Gpd1l	protein_coding	0.952	33.745	0.977	94.848	1.071	42.75	0.978	86.699
ENSMUSG00000050628	Ubald2	protein_coding	0.8	5.491	1.062	88.705	1.18	7.24	0.999	86.699
ENSMUSG00000050633	1110035H17Rik	pseudogene	1	75.196	1	96.216	1	84.83	1	95.35
ENSMUSG00000050640	Tmem150c	protein_coding	1.162	72	0.895	77.598	0.958	56.497	0.861	91.372
ENSMUSG00000050641	BC048562	protein_coding	1	83.658	1	97.263	NA	NA	1	96.287
ENSMUSG00000050645	Defb19	protein_coding	1	76.396	1	96.984	1	86.144	1	93.156
ENSMUSG00000050650	Mrgpra1	protein_coding	1	86.333	1	95.504	1	88.601	1	93.964
ENSMUSG00000050654	Olfr215	protein_coding	1	84.397	1	96.001	1	78.498	1	95.877
ENSMUSG00000050663	Trhde	protein_coding	0.804	13.45	NA	NA	1.387	20.085	0.93	88.245
ENSMUSG00000050666	Vstm4	protein_coding	1.057	58.198	0.927	82.671	1.023	87.971	1.039	95.676
ENSMUSG00000050668	Gpatch11	protein_coding	0.999	84.168	1.039	96.897	0.959	87.744	0.985	90.177
ENSMUSG00000050671	Ism2	protein_coding	1.144	33.099	0.774	55.265	1.127	72.695	1.196	72.826
ENSMUSG00000050675	Gp1ba	protein_coding	0.526	6.34	NA	NA	1.933	26.663	1.312	93.132
ENSMUSG00000050677	Ccdc96	protein_coding	0.941	74.8	0.981	92.111	1.078	80.089	0.947	94.608
ENSMUSG00000050685	NA	NA	NA	NA	1	96.216	1			

66.269	1	95.273
ENSMUSG00000050697	Prkaa1	protein_coding	1.143	0.2	0.937
85.671	0.931	4.521	0.889	77.331
ENSMUSG00000050700	Emilin3	protein_coding	1.849	29.811
1.023	95.809	0.53	39.245	0.817	91.372
ENSMUSG00000050702	Rfpl3s	protein_coding	1	71.36	1
92.137	1	69.315	1	93.156
ENSMUSG00000050704	2310061N02Rik	protein_coding	1	75.567
1	94.367	NA	NA	1	95.877
ENSMUSG00000050705	2310061I04Rik	protein_coding	0.854	1.6
1.101	71.98	1.063	77.888	1.005	92.181
ENSMUSG00000050708	Ftl1	protein_coding	1.071	39.851	1.1
79.055	0.851	7.399	0.879	83.724
ENSMUSG00000050709	4932442E05Rik	protein_coding	0.85	45.502
1.049	93.654	1.122	45.667	0.847	84.19
ENSMUSG00000050714	Zbtb26	protein_coding	0.987	67.477
1.031	96.68	0.982	88.693	0.959	90.157
ENSMUSG00000050721	Plekho2	protein_coding	1.207	24.147
0.998	96.216	0.83	36.786	0.949	96.071
ENSMUSG00000050730	Arhgap42	protein_coding	1.03	64.401
1.103	97.155	0.874	40.338	0.931	85.493
ENSMUSG00000050732	Vamp8	protein_coding	0.999	81.657	0.966
69.175	1.036	53.587	1.048	80.187
ENSMUSG00000050737	Ptges	protein_coding	0.692	2.559	1.256	72.546
1.147	51.243	0.967	95.593
ENSMUSG00000050742	Olfr164	protein_coding	1	76.546	1
96.268	1	79.897	1	96.248
ENSMUSG00000050751	Pgbd5	protein_coding	0.514	33.415	2.145
75.62	0.908	85.58	1.212	96.071
ENSMUSG00000050756	Defb6	protein_coding	1	86.658	1
96.407	1	77.294	1	95.832
ENSMUSG00000050761	Gp1bb	protein_coding	0.989	76.968	1.039
95.708	0.972	77.888	1.225	87.134
ENSMUSG00000050762	Prss27	protein_coding	0.632	3.009	1.188
83.104	1.329	23.41	1.052	92.263
ENSMUSG00000050763	Olfr1395	protein_coding	1	83.09	NA	NA
1	85.464	1	93.261
ENSMUSG00000050765	Gm5084	protein_coding	0.699	84.819	NA
NA	1.486	59.274	1.299	90.674
ENSMUSG00000050766	Ear14	protein_coding	1	85.499	1
96.216	1	84.504	1	96.183
ENSMUSG00000050772	Olfr1124	protein_coding	1	81.657	1
94.597	1	87.431	1	93.964
ENSMUSG00000050776	Olfr1317	protein_coding	1	59.028	1
96.652	NA	NA	1	95.273
ENSMUSG00000050777	Tmem37	protein_coding	1.31	2.784	0.792
66.09	0.964	73.531	1.163	72.992
ENSMUSG00000050781	Olfr1284	protein_coding	1	82.891	1
95.708	1	85.921	1	95.273
ENSMUSG00000050783	Htr1f	protein_coding	1	81.24	NA	NA	NA
NA	1	95.74
ENSMUSG00000050786	Ccdc126	protein_coding	1.098	20.032
1.238	80.277	0.737	3.147	0.953	86.699
ENSMUSG00000050788	Olfr419	protein_coding	1	83.968	NA

NA	1	87.786	1	95.593
ENSMUSG00000050796	B3galt6	protein_coding	0.997	75.671
1.083	91.643	0.923	30.468	1.033	93.261
ENSMUSG00000050799	Hist1h2ba	protein_coding	1	83.658	1
95.708	1	85.352	1	96.287
ENSMUSG00000050803	Olfr866	protein_coding	1	72.372	1
95.708	1	81.857	1	93.964
ENSMUSG00000050808	Muc15	protein_coding	0.979	76.042	1.153
96.843	0.887	77.037	0.798	83.724
ENSMUSG00000050810	Oog3	protein_coding	1	85.203	1
97.097	1	74.179	1	95.777
ENSMUSG00000050812	AI314180	protein_coding	1.017	59.527
0.977	93.654	1.004	83.158	1.055	70.539
ENSMUSG00000050813	Olfr332	protein_coding	1	84.738	1
97.155	1	84.434	1	96.358
ENSMUSG00000050815	Olfr1441	protein_coding	1	85.129	NA
NA	NA	NA	1	93.156
ENSMUSG00000050818	NA	NA	NA	NA	1	96.652	1
84.896	1	94.904
ENSMUSG00000050821	Fam131a	protein_coding	1.228	10.687
1.011	80.806	0.807	0.72	0.893	91.625
ENSMUSG00000050822	Slc29a4	protein_coding	1.143	42.134
0.639	79.475	1.374	70.1	0.687	83.724
ENSMUSG00000050824	Sstr5	protein_coding	1	76.135	1
95.708	1	81.169	1	96.287
ENSMUSG00000050830	Vwc2	protein_coding	0.861	74.147	1.085
96.843	1.072	76.64	1.375	89.052
ENSMUSG00000050836	Ntng1	protein_coding	1.216	50.627	1.658
82.904	0.495	27.321	0.728	84.29
ENSMUSG00000050844	1700020N01Rik	protein_coding	1	85.655
1	95.329	1	83.538	1	83.067
ENSMUSG00000050846	Zfp623	protein_coding	1.075	17.492
1.014	95.735	0.92	18.336	0.988	95.832
ENSMUSG00000050852	Gm12115	pseudogene	1	67.374	1
96.68	1	66.425	1	90.289
ENSMUSG00000050853	Olfr958	protein_coding	1	75.567	NA
NA	1	85.757	1	96.139
ENSMUSG00000050854	Tmem125	protein_coding	0.947	80.251
0.992	95.504	1.065	77.17	1.138	79.713
ENSMUSG00000050855	Zfp940	protein_coding	1.035	57.133
1.111	96.407	0.871	36.248	0.779	85.493
ENSMUSG00000050856	Atp5k	protein_coding	0.656	2.174	1.164	60.309
1.311	33.042	1.116	90.455
ENSMUSG00000050860	Phospho1	protein_coding	0.6	57.014
1.256	97.155	1.324	56.497	1.038	96.248
ENSMUSG00000050865	Olfr1494	protein_coding	1	77.916	1
93.046	1	87.173	1	95.74
ENSMUSG00000050870	Mrgprb8	protein_coding	1	69.408	1
93.654	1	88.657	1	95.559
ENSMUSG00000050875	A730017C20Rik	protein_coding	1.016	79.068
NA	NA	1.009	87.336	1.709	49.514
ENSMUSG00000050883	4930523C07Rik	protein_coding	1	83.169
1	96.843	1	87.898	1	94.904
ENSMUSG00000050890	Pdik1l	protein_coding	1.013	70.741

1.046 90.68 0.946 48.133 1.012 94.174
ENSMUSG00000050891 Tatdn1 protein_coding 1.07 71.896 1.01
90.287 0.923 83.719 1.029 70.539
ENSMUSG00000050896 Rtn4rl2 protein_coding 0.799 27.202
1.325 79.411 0.946 84.626 1.068 91.525
ENSMUSG00000050900 Gm7327 pseudogene 1 86.464 1
93.899 1 84.669 1 95.72
ENSMUSG00000050901 Mtnr1b protein_coding 1 78.909 NA
NA 1 88.161 1 96.183
ENSMUSG00000050908 Tvp23a protein_coding 1.057 84.397
1.152 80.678 0.823 35.815 1.281 31.133
ENSMUSG00000050910 Cdr2l protein_coding 1.036 41.454 1.136
79.29 0.846 4.935 1.208 46.412
ENSMUSG00000050912 Tmem123 protein_coding 1.125 1.99 0.934
82.848 0.948 25.512 0.887 86.991
ENSMUSG00000050914 Ankrd37 protein_coding 1.124 73.124
1.261 94.367 0.702 69.974 0.945 95.676
ENSMUSG00000050917 Fgf4 protein_coding 0.7 40.786 NA NA
1.54 61.171 1.075 84.246
ENSMUSG00000050919 Zfp366 protein_coding 1.097 34.696
1.152 88.551 0.789 5.087 0.91 85.493
ENSMUSG00000050921 P2ry10 protein_coding 1.134 51.826
1.187 92.062 0.744 39.079 0.897 83.724
ENSMUSG00000050926 Dcaf12l2 protein_coding 1.862 53.809
0.891 88.691 0.601 88.41 0.482 83.724
ENSMUSG00000050930 Map10 protein_coding 0.89 45.502 NA NA
1.153 47.776 1.083 91.928
ENSMUSG00000050931 Sgms2 protein_coding 1.52 1.913 1.061 96.897
0.619 7.509 1.357 65.401
ENSMUSG00000050933 Vmn1r231 protein_coding 1 71.06 NA NA
1 85.58 1 94.384
ENSMUSG00000050936 Hist2h2bb protein_coding 1.567 62.574
0.542 96.216 1.181 88.076 0.906 96.086
ENSMUSG00000050944 Efcab5 protein_coding 1.229 52.429
0.719 72.576 1.128 58.915 0.958 85.493
ENSMUSG00000050945 Zfp438 protein_coding 1.153 27.32 0.992
92.111 0.871 8.626 0.949 90.157
ENSMUSG00000050947 Amigo1 protein_coding 1.12 13.557
1.039 95.735 0.86 12.571 0.796 83.724
ENSMUSG00000050953 Gja1 protein_coding 1.035 79.853 1.033
83.104 0.937 38.534 1.006 92.018
ENSMUSG00000050954 Zfp169 protein_coding 1.02 73.487
0.954 90.952 1.03 84.434 0.928 84.29
ENSMUSG00000050957 Insl6 protein_coding 1.263 43.019 0.79
75.618 1.003 79.554 1.064 88.512
ENSMUSG00000050961 AY358078 protein_coding 1 84.452 1
95.504 1 86.602 1 96.192
ENSMUSG00000050963 Kcns2 protein_coding 1 83.169 1
96.763 1 81.29 1 96.192
ENSMUSG00000050965 Prkca protein_coding 0.847 0.987 1.123 60.309
1.054 12.35 1.061 46.846
ENSMUSG00000050967 Creg2 protein_coding 1.127 85.946 0.882
91.361 1.006 61.743 1.259 93.132
ENSMUSG00000050973 Gdpgp1 protein_coding 0.86 15.76 1.063

83.104	1.093	62.648	0.998	92.762
ENSMUSG00000050974	Gm9847	protein_coding	1	79.853	1
96.216	1	88.601	1	92.181
ENSMUSG00000050978	Gm5215	pseudogene	1	85.728	1
96.843	NA	NA	1	96.358
ENSMUSG00000050982	Apol10a	protein_coding	1	82.982	NA
NA	1	86.084	1	94.384
ENSMUSG00000050989	Sepn1	protein_coding	1.069	12.396	0.991
92.793	0.947	5.042	1.08	60.31
ENSMUSG00000050994	Adgb	protein_coding	0.817	11.726	1.028
91.065	1.194	42.75	0.933	93.045
ENSMUSG00000050996	Cetn1	protein_coding	1	82.982	1
97.288	1	83.158	1	95.273
ENSMUSG00000051000	Fam160a1	protein_coding	0.958	57.818
1.309	60.309	0.798	29.451	0.928	94.001
ENSMUSG00000051002	Gm9848	protein_coding	1	81.125	1
96.001	1	85.935	1	95.72
ENSMUSG00000051003	Olfr161	protein_coding	1	81.01	1
94.597	1	81.29	1	93.627
ENSMUSG00000051007	Pddc1	protein_coding	1.151	3.932	0.917	78.471
0.947	15.376	0.977	90.674
ENSMUSG00000051008	4930412M03Rik	lincRNA	0.492	17.062
1.295	91.456	1.565	47.31	1.213	93.156
ENSMUSG00000051022	Hs3st1	protein_coding	1.088	23.493
0.894	94.887	1.026	71.734	0.907	83.724
ENSMUSG00000051034	Zfp11	protein_coding	0.985	83.169	NA	NA
0.969	86.727	1.106	57.075
ENSMUSG00000051037	Zfp455	protein_coding	1.154	21.278
0.888	50.715	0.977	82.161	1.082	91.928
ENSMUSG00000051038	Rhox11	protein_coding	1	80.751	1
96.68	1	88.41	1	96.358
ENSMUSG00000051041	Olfml1	protein_coding	1.151	6.541	1.135
71.617	0.766	0.72	1.032	96.287
ENSMUSG00000051043	Gprc5c	protein_coding	1.171	0.2	0.97
82.947	0.88	1.92	1.051	92.181
ENSMUSG00000051046	Olfr214	protein_coding	1	85.391	NA
NA	1	83.857	1	95.676
ENSMUSG00000051048	P4ha3	protein_coding	1.559	2.784	0.989	96.216
0.649	9.494	1.816	50.452
ENSMUSG00000051050	Spink14	protein_coding	1	84.819	1
97.049	1	88.729	1	94.904
ENSMUSG00000051051	Olfr523	protein_coding	1	85.655	1
95.708	1	83.811	1	95.877
ENSMUSG00000051052	Olfr335-ps	pseudogene	1	85.129	1
90.287	1	61.841	1	94.904
ENSMUSG00000051056	Gja10	protein_coding	1	76.546	1
91.661	1	69.825	1	72.116
ENSMUSG00000051062	Fbll1	protein_coding	1	85.728	1
91.361	1	84.229	1	96.071
ENSMUSG00000051065	Mb21d2	protein_coding	0.982	81.196
1.127	82.904	0.906	51.715	0.9	83.724
ENSMUSG00000051067	Lingo3	protein_coding	8.505	2.569	NA	NA
0.245	1.898	0.468	92.181
ENSMUSG00000051074	4930579K19Rik	protein_coding	0.913	44.787

0.878 91.643 1.25 55.109 1.027 93.964
ENSMUSG00000051076 Vtcn1 protein_coding 0.759 14.645 1.115
82.904 1.184 41.6 1.271 57.075
ENSMUSG00000051079 Rgs13 protein_coding 6.456 7.842 0.196 79.807
0.785 52.972 1.047 91.105
ENSMUSG00000051095 Olfr986 protein_coding 1 84.661 1
90.712 1 85.921 1 96.287
ENSMUSG00000051098 Mblac2 protein_coding 1.071 41.177
1.108 96.216 0.84 22.735 0.962 86.699
ENSMUSG00000051107 Gm15440 protein_coding 1.01 84.263
1.225 91.643 0.811 28.237 1.065 85.577
ENSMUSG00000051111 Sv2c protein_coding 0.502 10.955 0.652
82.947 3.064 10.819 1.004 83.581
ENSMUSG00000051113 Fam71e1 protein_coding 0.945 50.229
0.789 70.29 1.337 15.185 0.789 83.724
ENSMUSG00000051116 Gm8121 pseudogene 1 85.857 1
95.088 1 88.657 1 95.832
ENSMUSG00000051118 Olfr77 protein_coding 1 86.145 1
97.155 1 87.898 1 90.95
ENSMUSG00000051124 Gimap9 protein_coding 1.033 69.489
1.026 96.68 0.945 65.902 1.007 91.525
ENSMUSG00000051133 Gm6293 pseudogene 0.922 80.51 1.008 91.643
1.078 84.552 0.707 83.724
ENSMUSG00000051136 Ghsr protein_coding 1 78.428 1
94.89 1 83.397 1 86.991
ENSMUSG00000051146 Camk2n2 protein_coding 0.7 3.956 1.104
83.104 1.296 29.777 0.942 96.358
ENSMUSG00000051147 Nat2 protein_coding 1.101 23.82 0.915 92.545
0.99 78.954 0.855 83.724
ENSMUSG00000051149 Adnp protein_coding 1.234 27.731 1.248
86.793 0.65 9.573 0.685 83.724
ENSMUSG00000051153 Tas2r105 protein_coding 1 80.146 1
97.263 1 87.744 1 92.389
ENSMUSG00000051154 Commd3 protein_coding 1.02 85.728
1.001 90.378 0.977 87.274 0.988 89.152
ENSMUSG00000051156 Olfr1491 protein_coding 1 81.178 1
95.708 1 84.308 1 92.903
ENSMUSG00000051159 Cited1 protein_coding 0.518 0.987 0.729
75.052 2.648 5.864 0.997 96.086
ENSMUSG00000051160 Olfr853 protein_coding 1 72 1
95.301 1 88.349 1 89.108
ENSMUSG00000051166 Eml5 protein_coding 0.949 71.142 1.105
95.431 0.954 87.744 0.803 83.724
ENSMUSG00000051169 Rpusd3 protein_coding 0.881 21.637
1.066 89.434 1.067 73.428 1.053 94.016
ENSMUSG00000051172 Olfr672 protein_coding 1 82.092 1
95.504 1 88.161 1 96.192
ENSMUSG00000051176 Zfp42 protein_coding 1 84.865 1
95.211 1 86.112 1 95.273
ENSMUSG00000051177 Plcb1 protein_coding 0.954 38.827 0.992
96.763 1.051 51.851 1.134 48.119
ENSMUSG00000051180 Olfr522 protein_coding 1 85.499 NA
NA 1 69.002 1 93.156
ENSMUSG00000051182 NA NA NA NA 1 97.155 1

84.961	1	96.183
ENSMUSG00000051184	Zfp524	protein_coding	0.993	58.38	0.956
78.471	1.055	71.615	0.986	87.218
ENSMUSG00000051185	Fam174a	protein_coding	0.96	39.174
0.978	96.68	1.065	72.378	1.005	92.181
ENSMUSG00000051190	Olfr1356	protein_coding	1	72.27	1
97.097	1	72.378	1	96.358
ENSMUSG00000051198	NA	NA	NA	NA	1	96.763	1
77.294	1	93.722
ENSMUSG00000051200	Olfr513	protein_coding	1	86.474	NA
NA	NA	NA	1	96.248
ENSMUSG00000051207	Mrgprd	protein_coding	1	73.124	1
94.262	1	77.17	1	96.183
ENSMUSG00000051209	Gpr119	protein_coding	1	79.577	1
96.216	1	86.475	1	92.466
ENSMUSG00000051212	Gpr183	protein_coding	1.054	27.545
1.544	78.629	0.616	2.563	1.313	67.94
ENSMUSG00000051219	3100002H09Rik	protein_coding	1	82.732
1	97.129	1	79.255	1	92.742
ENSMUSG00000051220	Ercc6l	protein_coding	1.476	13.557
0.719	81.731	0.945	65.902	1.413	64.513
ENSMUSG00000051223	Bzw1	protein_coding	1.158	15.178	1.034
96.407	0.835	7.948	0.977	80.187
ENSMUSG00000051224	Tceanc	protein_coding	0.951	23.641
0.953	95.708	1.106	46.575	1.031	82.66
ENSMUSG00000051225	Fam83a	protein_coding	0.558	0.987	1.334
93.574	1.348	5.864	1.256	31.133
ENSMUSG00000051228	Nyx	protein_coding	1.206	47.174	0.938
95.504	0.887	83.811	0.77	86.991
ENSMUSG00000051232	Tmem199	protein_coding	0.916	36.823
0.949	96.138	1.15	49.898	1.002	85.493
ENSMUSG00000051234	Rnf7	protein_coding	1.081	8.281	1.024	88.923
0.904	2.722	0.98	93.584
ENSMUSG00000051235	Gen1	protein_coding	0.965	86.172	1.018
96.001	1.013	87.898	1.15	77.854
ENSMUSG00000051236	Msrb3	protein_coding	1.117	13.557	0.987
95.708	0.906	8.027	0.967	86.699
ENSMUSG00000051238	Swsap1	protein_coding	0.994	64.284
1.023	82.904	0.981	41.506	1.026	93.964
ENSMUSG00000051242	Pcdhb9	protein_coding	1.066	34.696
1.053	92.561	0.893	23.607	1.065	94.82
ENSMUSG00000051243	Islr2	protein_coding	0.95	35.449	1
88.691	1.054	34.07	0.88	91.269
ENSMUSG00000051246	Msantd1	protein_coding	0.89	34.795
0.968	96.68	1.157	69.196	1.063	94.654
ENSMUSG00000051251	Nhlh1	protein_coding	1	86.464	1
97.155	1	88.349	1	92.31
ENSMUSG00000051255	Gm6563	protein_coding	1	81.24	1
94.848	1	76.768	1	94.608
ENSMUSG00000051256	Jagn1	protein_coding	0.964	79.433	1.086
82.904	0.957	68.82	1.036	91.525
ENSMUSG00000051257	Trap1a	protein_coding	1	84.819	1
96.763	1	81.388	1	95.92
ENSMUSG00000051258	Olfr1362	protein_coding	1	63.126	1

95.088 1 88.601 1 88.657
ENSMUSG00000051262 Cml3 protein_coding 1.176 69.408 1.116
83.104 0.759 29.765 1.007 94.904
ENSMUSG00000051276 Actrt2 protein_coding 1 75.196 1
96.216 1 73.096 1 95.74
ENSMUSG00000051278 4930422G04Rik protein_coding 1.104 58.896
1.105 96.68 0.821 49.453 1.136 51.238
ENSMUSG00000051279 Gdf6 protein_coding 1.149 39.295 0.779
91.83 1.113 76.768 1.183 77.621
ENSMUSG00000051285 Pcmtd1 protein_coding 1.158 14.746 0.92
85.116 0.94 60.614 0.937 85.493
ENSMUSG00000051295 9630028B13Rik antisense 1 72.743 NA
NA 1 85.921 1 95.593
ENSMUSG00000051297 2410124H12Rik protein_coding 1 81.823
NA NA 1 84.896 1 96.358
ENSMUSG00000051306 Usp42 protein_coding 0.916 6.484 1.127 60.309
0.968 48.387 1.043 81.251
ENSMUSG00000051313 Olfr1262 protein_coding 1 66.897 1
96.652 1 86.346 1 95.74
ENSMUSG00000051314 Ffar2 protein_coding 1.513 12.396 1.052
94.367 0.628 10.602 0.721 83.724
ENSMUSG00000051316 Taf7 protein_coding 1.057 27.32 1.076 96.216
0.878 40.338 0.942 89.892
ENSMUSG00000051319 1500011K16Rik lincRNA 0.751 1.698 1.082
92.253 1.232 5.964 0.974 95.35
ENSMUSG00000051323 Pcdh19 protein_coding 1.06 50.377
0.992 95.211 0.953 30.827 1.188 44.679
ENSMUSG00000051329 Nup160 protein_coding 1.02 35.371
1.001 85.075 0.976 54.25 0.98 95.877
ENSMUSG00000051331 Cacna1c protein_coding 1.05 54.717
1.125 74.663 0.846 9.746 1.019 95.593
ENSMUSG00000051335 Gfod1 protein_coding 0.762 3.07 1.094 82.904
1.194 17.751 0.953 87.331
ENSMUSG00000051339 2900026A02Rik protein_coding 0.887 13.45
1.024 85.378 1.105 36.839 0.959 83.724
ENSMUSG00000051340 Olfr645 protein_coding 1 84.263 1
94.848 1 81.976 1 96.086
ENSMUSG00000051341 Zfp52 protein_coding 1.136 13.292 0.883
71.889 0.993 74.853 1.124 61.984
ENSMUSG00000051343 Rab11fip5 protein_coding 1.224 2.567 0.88
85.242 0.929 31.887 1.006 95.832
ENSMUSG00000051344 Plekhm3 protein_coding 1.207 6.135 0.991
93.899 0.832 4.935 0.922 90.805
ENSMUSG00000051346 Spryd4 protein_coding 1.014 67.608
0.994 95.301 0.991 73.874 1.157 57.075
ENSMUSG00000051351 Zfp46 protein_coding 1.081 25.207 1.029
91.584 0.894 6.858 0.982 86.991
ENSMUSG00000051354 Samd3 protein_coding 1.058 86.145 1.36
83.464 0.694 51.715 0.96 95.832
ENSMUSG00000051355 Commd1 protein_coding 0.909 14.526
1.029 91.065 1.071 37.764 1.071 57.075
ENSMUSG00000051359 Ncald protein_coding 1.081 51.737 1.187
79.055 0.776 23.938 0.88 83.724
ENSMUSG00000051361 6030498E09Rik protein_coding 1 75.399

1	95.088	1	46.575	1	94.904
ENSMUSG00000051362	Olfr589	protein_coding	1	81.125	1
95.708	1	81.976	1	95.92
ENSMUSG00000051367	Six1	protein_coding	0.954	55.641	1.231
60.309	0.85	17.381	NA	NA
ENSMUSG00000051373	Ppapdc3	protein_coding	1.306	18.985
1.066	83.104	0.715	3.718	1.269	82.85
ENSMUSG00000051375	Pcdh1	protein_coding	0.885	2.536	1.104	66.01	1.02
66.928	0.923	83.724
ENSMUSG00000051378	Kif18b	protein_coding	1.2	40.88	0.85
74.851	0.98	84.434	1.295	81.901
ENSMUSG00000051379	NA	NA	NA	NA	1.174	74.663	0.841
15.376	1.042	83.581
ENSMUSG00000051390	Zbtb22	protein_coding	0.942	33.599
0.953	86.728	1.112	32.776	0.956	92.31
ENSMUSG00000051391	Ywhag	protein_coding	0.797	0.987	1.071	72.546
1.166	14.131	1.068	65.175
ENSMUSG00000051392	Olfr1402	protein_coding	1	84.397	1
95.504	1	86.144	1	96.287
ENSMUSG00000051396	Hspa14	protein_coding	1.003	80.251
1.052	90.092	0.945	73.874	1.121	33.269
ENSMUSG00000051397	Tacstd2	protein_coding	0.819	4.825	1.061
91.065	1.154	21.873	0.908	86.699
ENSMUSG00000051403	Ppp1r37	protein_coding	0.9	1.017	1.031
83.104	1.077	7.964	0.953	93.685
ENSMUSG00000051413	Plagl2	protein_coding	1.149	26.77	0.94
90.046	0.924	44.211	0.884	83.724
ENSMUSG00000051414	Olfr829	protein_coding	1	85.77	NA	NA
1	87.786	1	95.92
ENSMUSG00000051424	Olfr1184	protein_coding	1	79.853	NA
NA	1	83.538	1	94.608
ENSMUSG00000051427	Ccdc157	protein_coding	0.903	0.987	1.058
87.185	1.047	9.807	1.077	83.067
ENSMUSG00000051428	Gm5644	pseudogene	1	75.858	1
95.504	1	88.076	1	92.748
ENSMUSG00000051431	Gpr87	protein_coding	1.04	72.27	1.284	94.102
0.746	85.134	0.511	86.699
ENSMUSG00000051435	Fhad1	protein_coding	1.189	45.746	0.892
91.361	0.939	71.734	1.036	91.372
ENSMUSG00000051437	Ubqlnl	protein_coding	1	85.857	1
95.504	1	87.744	1	92.564
ENSMUSG00000051439	Cd14	protein_coding	0.979	73.487	0.922
72.576	1.11	25.425	0.917	83.724
ENSMUSG00000051444	Bbs12	protein_coding	0.962	78.744	0.992
87.919	1.05	55.352	1.011	95.273
ENSMUSG00000051451	Crebzf	protein_coding	1.004	65.622
0.971	85.671	1.026	43.129	0.876	83.724
ENSMUSG00000051455	Gm1564	protein_coding	1	86.74	NA	NA
1	86.144	1	95.676
ENSMUSG00000051457	Spn	protein_coding	1.321	1.161	1.045	92.561
0.721	2.333	1.014	88.387
ENSMUSG00000051469	Zfp191	protein_coding	1.077	45.307
0.969	92.137	0.958	67.648	0.967	95.72
ENSMUSG00000051480	A130050O07Rik	protein_coding	0.663	66.129

1.139 89.509 1.323 54.673 2.4 50.452
ENSMUSG00000051481 Krtap31-2 protein_coding 1 84.452 1
94.597 1 79.625 1 96.248
ENSMUSG00000051483 Cbr1 protein_coding 1.033 73.734 0.934
82.073 1.038 54.809 0.898 83.724
ENSMUSG00000051486 Pcdhb11 protein_coding 0.921 64.284
1.205 83.104 0.903 43.426 0.882 91.372
ENSMUSG00000051490 Foxd4 protein_coding 1 82.231 1
96.013 1 82.778 1 96.358
ENSMUSG00000051493 Olfr974 protein_coding 1 80.251 1
96.216 NA NA 1 95.74
ENSMUSG00000051495 Irf2bp2 protein_coding 1.086 81.196
0.987 97.263 0.934 59.922 0.877 85.493
ENSMUSG00000051498 Tlr6 protein_coding 1.163 12.72 0.841 85.671
1.018 48.851 1.233 83.067
ENSMUSG00000051499 Zfp786 protein_coding 1.058 56.085
0.784 75.345 1.2 68.425 1.206 83.067
ENSMUSG00000051502 Ufsp1 protein_coding 0.779 8.302 0.903 90.378
1.415 14.597 0.865 92.181
ENSMUSG00000051504 Siglech protein_coding 1.105 66.76 0.916
94.89 0.99 59.726 0.6 83.724
ENSMUSG00000051506 Wdfy4 protein_coding 1.415 0.355 1.049 94.597
0.673 1.898 1.037 92.018
ENSMUSG00000051509 Olfr414 protein_coding 1 81.01 1
95.431 1 87.971 1 94.567
ENSMUSG00000051510 Mafg protein_coding 0.912 0.987 0.983 85.921
1.11 5.864 1.01 79.268
ENSMUSG00000051515 Fam181b pseudogene 0.855 41.021 0.98
96.652 1.191 69.728 0.926 86.991
ENSMUSG00000051517 Arhgef39 protein_coding 0.903 77.497 0.91
83.58 1.22 81.492 1.63 83.276
ENSMUSG00000051518 Rps19bp1 protein_coding 0.979 77.763
0.989 96.847 1.028 78.864 1.022 96.183
ENSMUSG00000051527 Usp29 protein_coding 1 57.949 1
96.763 1 69.583 1 96.287
ENSMUSG00000051528 Olfr424 protein_coding 1 84.865 1
95.504 1 83.719 1 96.192
ENSMUSG00000051537 Gm5124 pseudogene 0.724 2.009 1.167 86.793
1.176 24.07 0.836 85.493
ENSMUSG00000051548 Gm6365 pseudogene 0.978 79.165 0.673
95.504 1.514 70.534 0.739 94.904
ENSMUSG00000051550 Zfp579 protein_coding 0.904 38.195
1.094 88.823 1.011 55.81 0.982 90.805
ENSMUSG00000051557 Pusl1 protein_coding 1.029 67.608 1.028
85.985 0.942 45.573 0.987 88.797
ENSMUSG00000051579 Tceal8 protein_coding 1.027 57.38 0.981
85.075 0.993 83.857 1.098 57.1
ENSMUSG00000051586 Mical3 protein_coding 0.964 32.749
1.067 79.864 0.974 86.144 0.95 93.612
ENSMUSG00000051590 Map3k19 protein_coding 1.129 27.545
0.768 68.115 1.152 88.274 1.128 90.455
ENSMUSG00000051591 Olfr697 protein_coding 1 81.925 1
94.767 1 63.665 1 94.99
ENSMUSG00000051592 Ccnb3 protein_coding 1 76.396 1

96.216 1 71.999 1 93.457
ENSMUSG00000051593 Olfr272 protein_coding 1 82.982 1
95.211 1 86.602 1 93.457
ENSMUSG00000051596 Otop1 protein_coding 0.759 46.413 1.202
79.864 1.092 86.112 1.225 90.397
ENSMUSG00000051599 Pcdhb2 protein_coding 0.83 73.585
1.681 92.062 0.717 88.461 0.812 85.493
ENSMUSG00000051611 Olfr112 protein_coding 1 80.751 1
94.48 1 51.977 1 90.674
ENSMUSG00000051614 4930533L02Rik protein_coding 1 83.726
1 96.138 1 86.666 1 96.183
ENSMUSG00000051615 Rap2a protein_coding 1.031 44.334 1.135
60.309 0.852 0.72 0.998 95.273
ENSMUSG00000051616 C230029F24Rik protein_coding 1 84.564
1 95.708 1 68.266 1 95.832
ENSMUSG00000051617 Krt9 protein_coding 0.653 43.816 NA NA
1.311 35.13 1.346 85.577
ENSMUSG00000051618 Ubqln3 protein_coding 1 83.418 1
96.216 1 86.112 1 96.183
ENSMUSG00000051627 Hist1h1e protein_coding 0.539 1.379 1.501
71.98 1.233 33.802 1.006 96.183
ENSMUSG00000051638 Gm9857 protein_coding 1 85.946 1
79.475 1 84.434 1 95.676
ENSMUSG00000051639 Gm5812 pseudogene 0.869 50.229 1.087
97.049 1.062 71.839 1.061 96.183
ENSMUSG00000051648 Kctd19 protein_coding 1.023 68.16 1.268
97.049 0.773 87.431 0.906 93.685
ENSMUSG00000051650 B3gnt2 protein_coding 1.555 52.429
1.073 93.899 0.6 44.019 0.757 92.31
ENSMUSG00000051652 Lrrc3 protein_coding 1.267 24.253 NA NA
0.75 12.948 0.88 90.691
ENSMUSG00000051669 AU021092 protein_coding 1.177 22.633
0.924 90.053 0.915 52.658 0.91 85.493
ENSMUSG00000051671 Coa6 protein_coding 0.907 36.259 1.033
94.767 1.069 53.882 0.937 93.316
ENSMUSG00000051674 Dcun1d4 protein_coding 1.206 0.999 0.968
92.404 0.858 0.72 0.95 83.724
ENSMUSG00000051675 Trim32 protein_coding 1.074 57.655
1.002 91.584 0.932 20.751 0.996 93.964
ENSMUSG00000051678 Pcdhb6 protein_coding 0.686 71.763 NA
NA 1.029 85.464 1.132 96.086
ENSMUSG00000051680 Olfr693 protein_coding 1 75.567 1
96.013 1 86.144 1 95.593
ENSMUSG00000051682 Treml4 protein_coding 0.992 60.435
1.003 95.301 1.005 79.255 0.778 85.493
ENSMUSG00000051687 Vmn1r73 protein_coding 1 79.577 1
94.848 1 86.258 1 93.156
ENSMUSG00000051695 Pcbp1 protein_coding 0.994 80.688 1.037
82.904 0.971 27.994 1.018 95.273
ENSMUSG00000051703 Tmem198 protein_coding 0.9 47.677
1.052 92.931 1.057 62.237 1.028 83.276
ENSMUSG00000051705 Senp8 protein_coding 1.044 76.686 0.927
90.712 1.036 74.686 1.043 85.577
ENSMUSG00000051706 Olfr1325 protein_coding 1 84.263 1

94.597	1	88.657	1	92.748
ENSMUSG00000051716	Apon	protein_coding	1.593	50.627	0.742
95.088	0.847	84.669	1.449	72.204
ENSMUSG00000051721	BC068281	protein_coding	0.961	83.333
1.094	79.29	0.946	31.887	0.97	91.105
ENSMUSG00000051726	Kcnf1	protein_coding	1.118	74.147	1.087
95.301	0.821	65.902	1.203	91.525
ENSMUSG00000051727	Kctd14	protein_coding	0.78	17.401
0.833	55.265	1.54	6.449	0.727	90.611
ENSMUSG00000051728	4930563D23Rik	protein_coding	1	82.092
1	96.897	1	85.21	1	93.964
ENSMUSG00000051729	Gm5087	protein_coding	1	85.77	1
93.27	1	85.921	1	96.287
ENSMUSG00000051730	Mettl5	protein_coding	0.925	13.952
1.043	91.584	1.033	45.035	1.02	73.939
ENSMUSG00000051732	Pabpc2	protein_coding	1	86.57	NA	NA
1	85.757	1	95.273
ENSMUSG00000051735	Rinl	protein_coding	1.199	12.985	0.966
92.137	0.865	25.512	1.021	95.877
ENSMUSG00000051736	Fam229b	protein_coding	0.971	85.946
1.106	96.138	0.93	86.112	1.083	79.442
ENSMUSG00000051747	Ttn	protein_coding	5.568	7.553	1.106	85.378
0.163	0.72	1.02	95.273
ENSMUSG00000051748	1100001G20Rik	protein_coding	0.915	24.344
1.107	78.705	0.983	87.173	0.958	92.31
ENSMUSG00000051758	4930544M13Rik	lincRNA	1	73.841	NA
NA	1	87.37	1	95.72
ENSMUSG00000051768	Xrcc1	protein_coding	0.902	4.385	NA	NA
1.101	14.449	0.99	96.358
ENSMUSG00000051769	Wfdc15a	protein_coding	1	80.408	1
95.504	1	84.896	1	95.273
ENSMUSG00000051777	Iqcj	protein_coding	1	78.909	1
95.504	1	74.179	1	90.865
ENSMUSG00000051786	Tubgcp6	protein_coding	0.993	67.867
1.075	78.705	0.932	23.48	1.003	94.904
ENSMUSG00000051788	4930564D02Rik	protein_coding	1	86.199
1	96.68	1	85.935	1	92.675
ENSMUSG00000051790	Nlgn2	protein_coding	1.038	42.134	1.027
95.196	0.935	11.314	1.051	93.132
ENSMUSG00000051793	NA	NA	NA	NA	1	97.129	1
64.624	1	93.685
ENSMUSG00000051802	Krtap19-5	protein_coding	1	74.745	NA
NA	1	84.961	1	96.183
ENSMUSG00000051804	Adam6b	protein_coding	1	82.352	1
96.68	1	66.425	1	91.807
ENSMUSG00000051811	Cox6b2	protein_coding	1.181	23.019
0.883	72.576	0.96	66.753	0.999	85.493
ENSMUSG00000051817	Sox12	protein_coding	0.837	48.146	1.067
96.216	1.122	39.925	1.02	96.183
ENSMUSG00000051827	Rhox2a	protein_coding	1	63.804	1
96.138	1	66.201	1	86.699
ENSMUSG00000051832	E230016K23Rik	processed_transcript	1.524
0.577	0.61	33.301	1.076	88.601	1.089	88.197
ENSMUSG00000051844	B230319C09Rik	processed_transcript	0.775

17.062 1.361 81.127 0.946 79.426 0.859 92.181
ENSMUSG00000051851 Cxx1c protein_coding 1.019 84.564 1.013
91.643 0.972 53.224 1.031 89.052
ENSMUSG00000051853 Arf3 protein_coding 1.035 48.288 1.033
83.104 0.934 23.1 0.984 93.685
ENSMUSG00000051855 NA NA NA NA 0.935 96.652 1.047
78.045 1.363 71.305
ENSMUSG00000051860 Samd7 protein_coding 1.017 49.047 1.593
92.111 0.615 87.786 0.58 96.183
ENSMUSG00000051864 Tbc1d22a protein_coding 1.059 6.683 0.983
90.378 0.962 44.678 1.018 94.608
ENSMUSG00000051877 Vmn2r72 protein_coding 1 82.982 1
96.216 NA NA 1 96.183
ENSMUSG00000051879 Krt71 protein_coding 1.302 86.199 0.703
86.728 1.092 63.301 0.181 83.724
ENSMUSG00000051885 Olfr664 pseudogene 1 85.832 1
95.708 1 80.634 1 93.413
ENSMUSG00000051890 Klhdc1 protein_coding 1.12 7.332 0.996
79.475 0.899 21.666 0.938 85.493
ENSMUSG00000051896 Tex37 protein_coding 1 80.751 1
95.708 1 86.666 1 96.358
ENSMUSG00000051906 Cd209f protein_coding 1.369 0.461 0.833
68.115 0.876 16.949 1.188 67.691
ENSMUSG00000051910 Sox6 protein_coding 1.208 7.083 1.069 71.617
0.775 1.705 0.998 92.31
ENSMUSG00000051917 Tas2r144 protein_coding 1 81.52 1
95.504 1 86.475 1 94.82
ENSMUSG00000051920 Rspo2 protein_coding 1.342 65.284 0.957
91.584 0.776 60.96 2.613 65.845
ENSMUSG00000051934 Spats2 protein_coding 1.015 84.452
0.941 72.399 1.05 53.587 1.03 78.546
ENSMUSG00000051936 Prss58 protein_coding 1 84.049 1
90.378 1 79.625 1 94.904
ENSMUSG00000051940 5031410I06Rik protein_coding 1 86.145
1 96.652 1 87.898 1 96.139
ENSMUSG00000051950 B3galtl protein_coding 0.766 0.987 1.229
71.617 1.065 26.912 1.037 92.018
ENSMUSG00000051951 Xkr4 protein_coding 0.797 43.816 1.039
96.68 1.208 57.286 0.897 94.608
ENSMUSG00000051952 Olfr371 protein_coding 1 66.897 1
95.504 NA NA 1 95.273
ENSMUSG00000051956 Rnf133 protein_coding 1 70.121 1
95.301 1 75.967 1 94.904
ENSMUSG00000051969 Tlr11 protein_coding 1.393 44.061 0.827
97.263 0.868 41.662 0.762 83.724
ENSMUSG00000051977 Prdm9 protein_coding 0.954 41.759 1.029
86.156 1.016 80.381 1.005 95.74
ENSMUSG00000051978 Erich1 protein_coding 0.985 78.299
1.044 82.904 0.974 38.434 1.008 96.183
ENSMUSG00000051984 Sec31b protein_coding 0.931 28.998
1.017 82.904 1.058 82.161 1.001 91.928
ENSMUSG00000051986 A530006G24Rik lincRNA 1 84.452 1
93.469 1 83.275 1 93.685
ENSMUSG00000051989 Smim11 protein_coding 0.948 57.014 1.14

71.617 0.927 39.755 1.049 83.067
ENSMUSG00000051998 Lax1 protein_coding 1.054 77.113 1.176
83.104 0.806 40.338 0.967 93.612
ENSMUSG00000052012 Olfr796 protein_coding 1 83.726 1
95.088 1 84.504 1 95.74
ENSMUSG00000052013 Btla protein_coding 0.916 84.324 0.992
96.216 1.103 87.274 0.956 93.685
ENSMUSG00000052014 A330070K13Rik protein_coding 1 75.858
1 96.763 1 80.015 1 95.273
ENSMUSG00000052025 Ppp1r2-ps7 pseudogene 1 83.968 1
96.68 1 83.857 1 96.183
ENSMUSG00000052026 Slc6a7 protein_coding 1.13 58.38 NA NA
1.012 78.045 0.718 83.952
ENSMUSG00000052031 Tagap1 protein_coding 0.847 11.924
1.065 84.999 1.108 21.873 0.999 95.273
ENSMUSG00000052033 Pfdn4 protein_coding 1.116 28.927 0.998
96.138 0.898 51.055 0.987 93.413
ENSMUSG00000052040 Klf13 protein_coding 1.016 74.147 1.032
90.053 0.953 75.527 1.017 94.752
ENSMUSG00000052056 Zfp217 protein_coding 1.019 65.537 NA
NA 0.965 76.64 1.005 96.248
ENSMUSG00000052058 Olfr901 protein_coding 1 84.168 1
96.216 1 83.607 1 95.35
ENSMUSG00000052062 Pard3b protein_coding 1.117 8.102 0.919
79.475 0.974 37.617 0.987 95.676
ENSMUSG00000052075 1700029F12Rik protein_coding 1 85.637
1 95.088 1 78.293 1 95.676
ENSMUSG00000052085 Dock8 protein_coding 1.528 0 0.904 56.781
0.725 0.945 0.884 85.493
ENSMUSG00000052087 Rgs14 protein_coding 1.771 0 0.749 79.475
0.756 3.073 1.054 85.786
ENSMUSG00000052098 Gm9867 protein_coding 1 81.196 1
95.708 1 87.519 1 94.82
ENSMUSG00000052099 Prss51 protein_coding 1 86.74 1
95.088 1 84.724 1 95.74
ENSMUSG00000052102 Gnpda1 protein_coding 1.298 0 0.924
42.923 0.832 7.742 1.035 87.134
ENSMUSG00000052105 Soga2 protein_coding 1.057 53.228 0.959
87.041 0.989 86.112 1.068 86.445
ENSMUSG00000052117 D630039A03Rik protein_coding 1.041 73.124
0.77 47.831 1.243 45.035 1.023 92.748
ENSMUSG00000052125 F730043M19Rik processed_transcript 1.091
50.377 1.371 79.411 0.669 10.23 0.995 95.273
ENSMUSG00000052131 Akr1b7 protein_coding 0.658 76.135
0.749 92.914 2.032 54.363 1.256 94.174
ENSMUSG00000052133 Sema5b protein_coding 1.301 16.74 1.125
85.756 0.681 2.817 1.004 93.045
ENSMUSG00000052135 Foxo6 protein_coding 1.487 52.799 0.979
87.609 0.689 18.122 0.884 96.287
ENSMUSG00000052137 Rbm12b2 protein_coding 1.086 20.032 1.04
90.092 0.883 11.997 0.922 83.724
ENSMUSG00000052139 Bre protein_coding 0.886 15.486 1.111
60.309 1.016 87.519 1.167 73.597
ENSMUSG00000052142 Rasal3 protein_coding 1.018 85.728

1.168 60.309 0.843 4.963 0.945 85.493
ENSMUSG00000052143 Gm9869 protein_coding 0.863 51.447
0.681 87.919 1.695 29.568 0.875 83.724
ENSMUSG00000052144 Ppp4r2 protein_coding 1.23 0 0.978
85.116 0.829 0.72 0.981 93.612
ENSMUSG00000052146 Rps10 protein_coding 0.911 31.333 0.967
79.261 1.133 20.968 1.019 91.525
ENSMUSG00000052151 Ppap2c protein_coding 0.899 47.677 0.97
81.731 1.149 24.383 0.994 93.612
ENSMUSG00000052154 Gm9870 pseudogene 0.84 29.725 1.142
85.756 1.045 77.888 0.87 89.525
ENSMUSG00000052155 Acvr2a protein_coding 1.074 34.077
1.081 83.104 0.861 11.314 0.941 83.724
ENSMUSG00000052157 Gm650 pseudogene 1 85.728 1 96.847
1 83.719 1 94.82
ENSMUSG00000052160 Pld4 protein_coding 1.472 0 0.819 68.818
0.828 20.511 1.069 94.016
ENSMUSG00000052180 Serpinb6c protein_coding 1.223 83.263
1.026 95.735 0.794 61.743 0.962 95.877
ENSMUSG00000052182 Olfr849 protein_coding 1 71.142 1
97.263 1 85.935 1 88.797
ENSMUSG00000052187 Hbb-y protein_coding 1 84.534 NA NA
1 88.076 1 96.071
ENSMUSG00000052188 Gm14964 antisense 1.108 63.385 0.699
67.839 1.293 49.601 0.844 83.724
ENSMUSG00000052192 Gm5963 pseudogene 1 84.661 1
94.262 1 81.62 1 91.65
ENSMUSG00000052212 Cd177 protein_coding 1.095 57.949 0.801
85.671 1.144 88.461 1.373 68.696
ENSMUSG00000052214 Opa3 protein_coding 0.824 4.099 1.272 71.617
NA NA 1.083 40.426
ENSMUSG00000052217 Hbb-bh1 protein_coding 1 86.112 1
96.847 1 80.634 1 95.273
ENSMUSG00000052221 Ppp1r36 protein_coding 1.174 23.019 0.87
72.399 0.979 79.524 1.09 95.72
ENSMUSG00000052222 1700084E18Rik pseudogene 0.793 74.021
0.896 83.58 1.409 20.085 0.943 96.183
ENSMUSG00000052229 Gpr17 protein_coding 0.705 8.139 0.831 26.053
1.697 5.864 0.98 85.493
ENSMUSG00000052234 Epx protein_coding 1 74.021 1
91.361 1 69.728 1 94.468
ENSMUSG00000052236 Gm9871 protein_coding 1 83.418 1
90.378 1 85.58 1 95.832
ENSMUSG00000052241 A930035D04Rik pseudogene 0.845 69.715
1.208 87.029 0.978 84.071 1.02 95.74
ENSMUSG00000052248 Gm13476 antisense 0.935 59.358 1.07
90.116 0.999 84.724 0.927 93.316
ENSMUSG00000052253 Zfp622 protein_coding 1.013 53.809
0.933 65.165 1.054 61.743 0.965 84.29
ENSMUSG00000052262 Prpmp5 protein_coding 1 85.832 1
91.661 NA NA 1 95.74
ENSMUSG00000052270 Fpr2 protein_coding 1.633 16.249 0.745
78.51 0.821 57.819 1.322 88.512
ENSMUSG00000052271 Bhlha15 protein_coding 1.153 62.007

0.899 82.671 0.967 88.693 1.323 73.368
ENSMUSG00000052273 Dnah3 protein_coding 0.533 15.456 1.139
91.584 1.647 21.705 1.019 93.132
ENSMUSG00000052281 Gm15024 antisense 1.077 57.133 0.745
85.671 NA NA 1.039 90.854
ENSMUSG00000052291 5330438D12Rik pseudogene 0.763 9.508 1.114
92.253 1.175 30.213 0.998 86.699
ENSMUSG00000052293 Taf9 protein_coding 1.079 34.861 0.99
97.263 0.937 35.815 1.147 70.624
ENSMUSG00000052296 Ppp6r1 protein_coding 1.03 77.407
1.015 79.261 0.958 33.402 0.949 84.29
ENSMUSG00000052298 Cdc42se2 protein_coding 1.162 0 0.998
85.418 0.857 5.336 1.006 85.629
ENSMUSG00000052299 Ltn1 protein_coding 0.973 66.406 1.029
85.378 1.001 80.749 1.027 94.904
ENSMUSG00000052301 Doc2a protein_coding 0.804 20.799 1.27
79.367 0.981 82.778 0.885 84.29
ENSMUSG00000052302 Tbc1d30 protein_coding 0.919 23.923
0.881 70.693 1.239 14.597 0.709 86.991
ENSMUSG00000052303 Mrgpra6 protein_coding 1 75.115 NA
NA 1 81.032 1 92.31
ENSMUSG00000052305 NA NA NA NA 0.87 61.883 1.161
30.645 1.208 88.512
ENSMUSG00000052310 Slc39a1 protein_coding 1.013 76.827
1.046 82.904 0.945 20.712 1.071 44.679
ENSMUSG00000052316 Lrrc15 protein_coding 1.673 8.685 0.937
96.216 0.637 14.409 1.183 88.755
ENSMUSG00000052331 Ankrd44 protein_coding 1.06 37.414
0.959 88.691 0.98 74.853 0.959 86.699
ENSMUSG00000052334 1700024B05Rik protein_coding 1 86.351
NA NA 1 88.203 1 95.877
ENSMUSG00000052336 Cx3cr1 protein_coding 0.975 64.193
1.334 79.367 0.769 0.945 1.076 93.156
ENSMUSG00000052337 Immt protein_coding 0.902 9.863 1.041 83.104
1.066 57.286 1.087 60.968
ENSMUSG00000052353 9930013L23Rik protein_coding 1.304 85.59
0.325 43.505 2.365 16.808 0.464 85.493
ENSMUSG00000052364 B630019K06Rik protein_coding 0.991 71.915
0.992 94.887 1.017 73.309 0.781 86.254
ENSMUSG00000052368 Gm9873 lincRNA 0.864 47.677 0.85
87.185 1.362 68.425 0.631 87.218
ENSMUSG00000052369 Tmem106c protein_coding 1.051 22.205
1.014 95.504 0.94 32.963 0.847 84.19
ENSMUSG00000052371 1700109F18Rik antisense 0.877 34.795 1.22
72.546 0.936 66.201 1.055 91.928
ENSMUSG00000052373 Mpp3 protein_coding 0.983 83.333 0.943
88.881 1.078 56.125 1.051 96.183
ENSMUSG00000052374 Actn2 protein_coding 0.725 80.815 1.394
93.994 0.989 51.715 2.65 83.067
ENSMUSG00000052376 4921508M14Rik protein_coding 1 74.516
1 95.504 1 87.898 1 88.111
ENSMUSG00000052382 Rnase9 protein_coding 1 59.211 1
96.378 1 88.693 1 93.612
ENSMUSG00000052384 Nrros protein_coding 1.397 0 1.004 92.631

0.711 1.871 1.074 73.823
ENSMUSG00000052387 Trpm3 protein_coding 1.168 51.826 0.974
95.35 0.877 81.388 0.775 85.493
ENSMUSG00000052388 Gm9874 protein_coding 1 71.06 1
71.889 1 47.935 1 93.734
ENSMUSG00000052392 Acot4 protein_coding 0.786 28.584 0.691
90.256 1.84 52.8 1.136 57.952
ENSMUSG00000052395 Rft1 protein_coding 0.99 74.745 0.991
94.96 1.02 80.015 0.988 94.19
ENSMUSG00000052396 Mogat2 protein_coding 0.355 0.987 1.502
82.904 1.868 5.864 0.929 95.832
ENSMUSG00000052397 Ezr protein_coding 1.51 4.675 0.702 54.245
0.944 49.733 1.19 83.067
ENSMUSG00000052403 Gm13520 antisense 1.246 8.477 0.687 30.872
1.166 57.286 1.034 94.904
ENSMUSG00000052406 Rexo4 protein_coding 1.05 54.44 0.974 93.654
0.979 81.169 1.043 42.736
ENSMUSG00000052407 Ccdc171 protein_coding 0.887 19.224
1.048 95.504 1.077 34.285 0.893 90.157
ENSMUSG00000052414 Atf7 protein_coding 1 81.657 NA NA
NA NA 1 96.086
ENSMUSG00000052415 Tchh protein_coding 0.928 45.894 0.903
90.378 1.19 44.361 1.072 78.379
ENSMUSG00000052417 Olfr720 protein_coding 1 81.178 1
97.155 1 86.346 1 94.904
ENSMUSG00000052419 2610001J05Rik protein_coding 1.051 31.732
1.027 79.367 0.923 2.563 1.057 49.514
ENSMUSG00000052423 B4galt3 protein_coding 0.922 18.138
1.034 88.533 1.047 43.481 0.984 91.372
ENSMUSG00000052426 Gm9877 pseudogene 0.559 32.749 1.601
82.904 1.115 83.538 0.542 90.805
ENSMUSG00000052428 Tmco1 protein_coding 0.991 70.213 1.046
84.832 0.965 82.903 0.971 88.136
ENSMUSG00000052429 Prmt1 protein_coding 0.967 45.728 0.977
94.262 1.061 38.099 1.045 62.594
ENSMUSG00000052430 Bmpr1b protein_coding 1.128 82.352
1.684 79.411 0.524 18.797 0.83 94.608
ENSMUSG00000052435 Cebpe protein_coding 1.461 56.66 0.52 81.731
1.316 62.648 1.427 77.621
ENSMUSG00000052446 Zfp961 protein_coding 1.155 3.061 0.977
83.321 0.887 32.297 1.008 93.964
ENSMUSG00000052456 Asna1 protein_coding 0.886 3.688 1.037 82.904
1.089 46.575 1.052 74.896
ENSMUSG00000052459 Atp6v1a protein_coding 0.985 85.355
1.041 96.407 0.979 62.791 1.005 92.762
ENSMUSG00000052467 D630004N19Rik protein_coding 1 84.049
1 95.735 1 87.274 1 86.118
ENSMUSG00000052468 Pmp2 protein_coding 0.803 61.633 1.077
95.708 1.158 71.839 0.943 88.505
ENSMUSG00000052469 Tcp10c protein_coding 1 85.946 1
91.275 1 81.492 1 96.287
ENSMUSG00000052471 Gm9881 protein_coding 1 84.865 1
97.288 1 81.976 1 94.904
ENSMUSG00000052477 C130026I21Rik protein_coding 2.458 0.2 0.63

28.822 0.642 16.129 1.203 78.546
ENSMUSG00000052479 A330008L17Rik protein_coding 1 85.946
1 96.138 1 86.258 1 95.777
ENSMUSG00000052482 Gm8174 pseudogene 1 79.277 1
96.948 1 79.524 1 96.287
ENSMUSG00000052488 Cherp protein_coding 0.999 60.339 1.024
83.104 0.978 61.288 0.986 92.31
ENSMUSG00000052496 Pkdrej protein_coding 0.827 82.543
1.089 95.809 1.112 86.666 0.932 96.183
ENSMUSG00000052504 Epha3 protein_coding 1.344 7.083 0.943 95.504
0.789 10.362 1.111 46.412
ENSMUSG00000052508 Olfr536 protein_coding 1 81.647 NA
NA 1 81.492 1 93.316
ENSMUSG00000052512 Nav2 protein_coding 1.116 18.635 1.015
90.092 0.885 0.879 1.028 96.248
ENSMUSG00000052516 Robo2 protein_coding 0.954 81.647 1.056
87.029 0.993 62.791 1.165 94.016
ENSMUSG00000052520 Cyp2j5 protein_coding 1 81.52 1
94.887 1 86.144 1 94.752
ENSMUSG00000052525 Spdya protein_coding 1.067 70.213 1.313
82.904 0.715 43.842 0.82 86.699
ENSMUSG00000052533 Nup188 protein_coding 1.045 45.096
0.976 87.322 0.981 87.744 1.044 88.755
ENSMUSG00000052534 Pbx1 protein_coding 1.095 10.105 0.944
79.807 0.969 22.279 0.936 83.724
ENSMUSG00000052537 Olfr198 protein_coding 1 81.178 1
96.216 1 70.534 1 93.734
ENSMUSG00000052539 Magi3 protein_coding 1.012 75.196 1.035
96.68 0.956 66.753 0.963 84.29
ENSMUSG00000052544 St6galnac3 protein_coding 1.996 0.2 0.898
82.947 0.557 2.986 0.877 83.724
ENSMUSG00000052549 Arl13a protein_coding 1 84.564 1
97.155 1 85.921 1 94.904
ENSMUSG00000052551 Adarb2 protein_coding 0.907 80.51 0.919
94.597 1.204 76.754 0.668 83.724
ENSMUSG00000052554 Defb34 protein_coding 1 69.943 1
97.155 1 82.436 1 93.685
ENSMUSG00000052557 Gan protein_coding 0.911 83.09 1.031 97.263
1.063 70.534 1.253 87.7
ENSMUSG00000052558 Gm9884 protein_coding 1 85.728 1
92.355 1 73.759 1 92.381
ENSMUSG00000052560 Cpne8 protein_coding 1.084 13.633 0.973
93.654 0.948 25.625 1.032 91.872
ENSMUSG00000052562 Slc22a30 protein_coding 1 86.333 1
94.887 1 76.498 1 94.99
ENSMUSG00000052563 D930048N14Rik protein_coding 0.749 3.009
1.066 71.617 1.249 20.968 0.978 93.964
ENSMUSG00000052565 Hist1h1d protein_coding 0.451 4.941 1.394
71.98 1.585 53.134 0.917 90.157
ENSMUSG00000052566 Hook2 protein_coding 0.833 4.941 0.953 87.041
1.256 8.007 0.909 84.19
ENSMUSG00000052572 Dlg2 protein_coding 1.287 15.662 0.822
87.322 0.949 46.575 0.861 85.493
ENSMUSG00000052581 Lrrtm4 protein_coding 1.119 86.112

1.549 86.387 0.576 49.967 0.95 96.183
ENSMUSG00000052584 Serp2 protein_coding 1.235 76.319 0.552
68.818 1.461 51.977 0.873 87.651
ENSMUSG00000052593 Adam17 protein_coding 1.158 0.577 0.958
81.731 0.901 35.264 0.978 93.685
ENSMUSG00000052595 A1cf protein_coding 1 80.751 1
95.941 1 86.666 1 93.156
ENSMUSG00000052605 Isoc2b protein_coding 0.894 23.923
0.974 87.857 1.147 26.498 0.983 86.991
ENSMUSG00000052609 Plekhg3 protein_coding 0.929 44.787
0.897 58.277 1.204 22.131 1.124 59.394
ENSMUSG00000052616 Ccdc79 protein_coding 0.553 7.58 0.791
88.691 2.279 16.26 0.786 88.245
ENSMUSG00000052625 Olfr851 protein_coding 1 84.397 1
96.138 1 88.601 1 94.654
ENSMUSG00000052629 Gm9885 protein_coding 0.827 52.726
1.019 93.469 1.189 36.839 1.002 87.705
ENSMUSG00000052631 Sh2d6 protein_coding 1.889 7.174 0.61 55.71
0.869 82.685 1.153 60.31
ENSMUSG00000052632 Asap2 protein_coding 0.948 20.369 0.913
87.452 1.151 24.07 0.942 86.991
ENSMUSG00000052642 4930504O13Rik protein_coding 1 82.352
1 96.216 1 80.749 1 94.99
ENSMUSG00000052656 Rnf103 protein_coding 0.94 49.944
0.787 21.426 1.348 5.864 0.905 94.654
ENSMUSG00000052658 5830454E08Rik protein_coding 0.688 31.614
0.958 96.843 1.515 25.966 1.31 76.831
ENSMUSG00000052673 Gm9887 protein_coding 1.141 81.24 NA NA
0.895 87.336 0.737 85.947
ENSMUSG00000052675 Zfp112 protein_coding 1.044 84.049 0.95
95.301 1.005 73.759 1.038 84.246
ENSMUSG00000052676 Zmat1 protein_coding 1.025 51.297 0.921
95.088 1.054 69.583 1.008 86.699
ENSMUSG00000052681 Rap1b protein_coding 1.237 0 0.996 97.155
0.812 0.72 1.111 63.114
ENSMUSG00000052684 Jun protein_coding 1.14 25.026 0.979
95.301 0.892 24.309 1.095 78.948
ENSMUSG00000052688 5430435G22Rik protein_coding 1.292 0
0.972 91.361 0.797 4.389 1.116 35.053
ENSMUSG00000052698 Tln2 protein_coding 1.02 80.408 1.094
72.642 0.898 29.451 1.04 83.276
ENSMUSG00000052707 Tnrc6a protein_coding 1.093 1.412 0.975
91.532 0.935 3.969 0.967 84.29
ENSMUSG00000052712 BC004004 protein_coding 0.957 8.938 1.075
71.617 0.974 69.583 1.005 89.052
ENSMUSG00000052713 Zfp608 protein_coding 0.909 65.199
1.073 75.62 1.023 67.339 0.941 85.493
ENSMUSG00000052724 Gm9888 antisense 0.878 83.09 1.023 90.256
1.113 63.665 1.207 96.248
ENSMUSG00000052726 Kcnt2 protein_coding 1.569 0.355 0.77 85.793
0.827 9.494 1.056 95.273
ENSMUSG00000052727 Map1b protein_coding 1.079 66 0.994 93.817
0.928 78.045 1.009 93.413
ENSMUSG00000052736 Klrc2 protein_coding 1.114 82.092 1.134

91.065 0.789 52.213 0.769 95.72
ENSMUSG00000052738 Suclg1 protein_coding 0.974 34.954
0.957 96.948 1.072 49.601 1.084 67.691
ENSMUSG00000052748 Swt1 protein_coding 1.035 52.429 1.025
93.654 0.938 77.037 0.971 91.65
ENSMUSG00000052749 Trim30b protein_coding 1.337 33.555 0.71
85.418 1.053 77.294 1.64 68.502
ENSMUSG00000052751 Repin1 protein_coding 0.975 47.266 1.07
71.98 0.957 58.775 0.981 89.475
ENSMUSG00000052752 Traf7 protein_coding 0.996 71.763 1.023
83.104 0.978 62.058 1.013 74.193
ENSMUSG00000052760 A630001G21Rik protein_coding 1.181 12.72
0.966 95.941 0.873 19.236 1.097 88.797
ENSMUSG00000052763 Zfp212 protein_coding 0.967 30.637 0.96
85.116 1.081 25.966 1.02 95.832
ENSMUSG00000052767 Gm12703 processed_transcript 1 82.231
1 97.129 1 76.225 1 88.797
ENSMUSG00000052769 Gm9889 pseudogene 0.972 82.891 0.72
79.475 1.427 37.259 1.127 94.608
ENSMUSG00000052776 Oas1a protein_coding 1.499 1.712 0.886 95.211
0.752 18.965 1.46 44.213
ENSMUSG00000052783 Grk4 protein_coding 0.974 65.106 0.952
96.268 1.076 81.492 1.02 90.611
ENSMUSG00000052785 Olfr573-ps1 protein_coding 1 75.298 1
96.843 1 80.015 1 93.734
ENSMUSG00000052790 Gm9890 pseudogene 1 80.608 1
97.155 1 86.727 1 96.248
ENSMUSG00000052794 1700030K09Rik protein_coding 1.032 86.57
0.961 97.263 1.009 85.921 0.912 83.724
ENSMUSG00000052798 Nup107 protein_coding 1.048 33.099
1.025 92.793 0.927 13.848 1.121 69.965
ENSMUSG00000052812 Atad2b protein_coding 0.831 6.822 0.951
86.071 1.266 9.567 0.957 92.31
ENSMUSG00000052818 Olfr813 protein_coding 1 83.968 1
96.268 1 88.41 1 94.904
ENSMUSG00000052821 Cysltr1 protein_coding 1.216 1.689 1.041
96.847 0.789 3.718 1.078 83.067
ENSMUSG00000052825 Gm9892 pseudogene 1 75.671 1
94.102 1 86.346 1 94.384
ENSMUSG00000052833 Sae1 protein_coding 1.036 62.354 1.003
95.504 0.962 64.778 1.038 49.514
ENSMUSG00000052837 Junb protein_coding 0.823 25.492 1.044
95.504 1.164 28.603 1.02 90.611
ENSMUSG00000052852 Reep1 protein_coding 1.011 81.01 1.308 66.01
0.754 15.778 1.153 92.381
ENSMUSG00000052854 Nrk protein_coding 1.39 50.913 1.29
95.196 0.559 33.689 1.212 65.175
ENSMUSG00000052861 Dnah6 protein_coding 1.221 67.608 0.736
61.625 1.112 45.449 0.773 83.952
ENSMUSG00000052865 Gm13619 antisense 1 86.658 NA NA
1 84.724 1 93.261
ENSMUSG00000052889 Prkcb protein_coding 1.171 14.34 1.232 71.98 0.69
1.061 1.034 94.384
ENSMUSG00000052906 Ubxn8 protein_coding 1.001 72.743 1.022

72.642 0.974 45.799 0.997 91.269
ENSMUSG00000052909 Gm9894 protein_coding 1 78.054 1
95.211 1 87.103 1 92.181
ENSMUSG00000052911 Lamb2 protein_coding 0.947 19.83 0.922 83.876
1.147 22.93 0.997 93.016
ENSMUSG00000052912 Smarca5-ps pseudogene 0.879 81.76 0.974 87.998
1.165 62.995 1.3 92.762
ENSMUSG00000052914 Cyp2j6 protein_coding 1.049 29.057 NA
NA 0.95 42.621 0.956 85.493
ENSMUSG00000052915 Msl1 protein_coding 1.069 40.065 1.048
72.546 0.891 0.72 0.97 84.29
ENSMUSG00000052917 Senp7 protein_coding 1.051 54.717 0.982
91.361 0.968 87.744 0.953 86.991
ENSMUSG00000052920 Prkg1 protein_coding 1.163 35.787 0.99
92.793 0.869 15.465 1.035 83.865
ENSMUSG00000052921 Arhgef15 protein_coding 0.976 82.479
1.119 79.367 0.916 8.907 0.882 83.724
ENSMUSG00000052926 Rnaseh2a protein_coding 0.919 22.142 0.97
85.075 1.122 17.412 1.063 75.81
ENSMUSG00000052928 Ctif protein_coding 0.953 63.736 0.903
83.392 1.161 45.208 0.921 86.699
ENSMUSG00000052934 Fbxo31 protein_coding 0.901 83.726 1.19
95.211 0.935 84.626 1.131 83.067
ENSMUSG00000052942 Glis3 protein_coding 1.028 82.092 0.967
95.211 1.003 83.925 0.869 83.724
ENSMUSG00000052949 Rnf157 protein_coding 1.133 21.146
1.075 90.092 0.822 11.64 1.129 88.197
ENSMUSG00000052951 C130021I20Rik antisense 0.659 0.987 0.939
90.744 1.615 10.079 0.818 84.29
ENSMUSG00000052957 Gas1 protein_coding 1.036 74.482 0.976
91.361 0.988 85.464 0.995 96.183
ENSMUSG00000052961 Lingo2 pseudogene 1 83.823 1
91.955 1 88.729 1 94.016
ENSMUSG00000052962 Mrpl35 protein_coding 0.995 58.077
0.993 96.843 1.01 88.601 0.993 78.042
ENSMUSG00000052974 Cyp2f2 protein_coding 0.686 13.239 0.82
84.594 1.781 12.467 0.74 92.12
ENSMUSG00000052979 6530403G13Rik protein_coding 1 82.092
1 96.763 1 86.666 1 95.35
ENSMUSG00000052981 Ube2ql1 protein_coding 1.27 63.385 0.92
85.671 0.856 58.516 0.847 83.724
ENSMUSG00000052997 Uba2 protein_coding 1.086 16.74 0.963 86.538
0.957 35.022 1.091 46.412
ENSMUSG00000053004 Hrh1 protein_coding 1.125 28.927 1.094
87.282 0.814 7.329 0.995 96.183
ENSMUSG00000053007 Creb5 protein_coding 0.893 23.13 1.028 94.96
1.084 35.441 1.005 95.72
ENSMUSG00000053012 Krcc1 protein_coding 0.992 85.637 1.095
72.546 0.917 27.675 0.977 94.19
ENSMUSG00000053024 Cntn2 protein_coding 0.892 39.953 1.283
80.678 0.87 87.519 0.89 92.561
ENSMUSG00000053025 Sv2b protein_coding 1.398 77.182 0.667
72.712 1.073 36.95 0.954 96.358
ENSMUSG00000053030 Spink2 protein_coding 1.038 86.57 1.037

94.102	0.925	86.258	1.928	67.94
ENSMUSG00000053038	Gm6180	pseudogene	1	84.294	1
96.407	1	86.602	1	91.783
ENSMUSG00000053040	Aph1c	protein_coding	1.253	20.296	0.825
55.71	0.968	87.274	0.623	83.724
ENSMUSG00000053044	Cd8b1	protein_coding	1.156	49.868	1.425
87.609	0.604	17.258	1.295	94.654
ENSMUSG00000053046	Brsk2	protein_coding	0.81	21.637	0.905
94.767	1.356	52.574	1.241	72.369
ENSMUSG00000053062	Jam2	protein_coding	1.194	0	1.004	95.708
0.836	0.72	0.906	83.724
ENSMUSG00000053063	Clec12a	protein_coding	2.062	3.825	0.923
86.604	0.526	11.314	0.747	89.892
ENSMUSG00000053070	9230110C19Rik	protein_coding	1.108	48.857
NA	NA	0.987	71.388	0.973	91.979
ENSMUSG00000053080	2700081O15Rik	protein_coding	1	84.865
1.126	62.898	0.886	2.144	1.006	90.289
ENSMUSG00000053081	1700069B07Rik	protein_coding	0.599	63.736
1.283	95.431	1.296	78.498	1.239	94.82
ENSMUSG00000053091	Lins	protein_coding	0.971	84.865	1.191
71.617	0.865	21.431	1.019	96.287
ENSMUSG00000053093	Myh7	protein_coding	2.179	9.695	0.875	87.041
0.523	14.409	0.579	96.192
ENSMUSG00000053094	Tmem248	protein_coding	1.072	12.985
1.043	74.663	0.895	1.58	1.033	83.067
ENSMUSG00000053101	Gpr141	protein_coding	2.068	1.846	0.661
41.141	0.729	27.994	1.034	95.92
ENSMUSG00000053110	Yap1	protein_coding	1.066	1.441	NA	NA
0.943	26.369	0.952	83.724
ENSMUSG00000053111	Fank1	protein_coding	1.688	14.59	0.702	67.941
0.845	81.169	1.18	86.301
ENSMUSG00000053113	Socs3	protein_coding	0.715	40.568	0.962
94.848	1.453	40.955	1.43	87.624
ENSMUSG00000053117	NA	NA	NA	NA	1.271	83.104	0.829
69.445	1.609	44.679
ENSMUSG00000053119	Chmp3	protein_coding	0.993	67.334	0.92
20.272	1.095	5.864	1.008	94.654
ENSMUSG00000053121	Gm5129	protein_coding	1.006	77.291	1.16
92.631	0.852	84.071	1.11	94.654
ENSMUSG00000053128	Rnf26	protein_coding	0.998	77.291	1.069
72.642	0.938	23.48	1.027	95.273
ENSMUSG00000053129	Gsx1	protein_coding	1	81.465	1
95.708	NA	NA	1	93.117
ENSMUSG00000053134	Supt7l	protein_coding	1.058	40.333
0.881	58.277	1.072	37.259	1.012	91.928
ENSMUSG00000053137	Mapk11	protein_coding	0.81	25.3	1.146
83.464	1.073	61.418	1.047	79.268
ENSMUSG00000053141	Ptprt	protein_coding	1.097	63.804	1.092
83.104	0.834	27.194	0.905	83.724
ENSMUSG00000053146	Olfr352	protein_coding	1	75.567	1
97.155	1	76.225	1	95.593
ENSMUSG00000053153	Spag16	protein_coding	1	73.734	1
94.48	1	68.82	1	94.654
ENSMUSG00000053158	Fes	protein_coding	1.075	15.602	0.93

85.95 0.996 69.445 1.088 63.114
ENSMUSG00000053164 Gpr21 protein_coding 1.073 71.516 1.281
96.847 0.728 59.125 1.025 96.183
ENSMUSG00000053168 9030619P08Rik protein_coding 0.562 43.435
1.471 82.904 1.207 82.74 1.47 96.358
ENSMUSG00000053173 Rpl18-ps2 pseudogene 1 80.408 1
93.469 1 84.961 1 96.183
ENSMUSG00000053175 Bcl3 protein_coding 0.824 12.084 0.947
95.211 1.275 37.741 1.079 92.381
ENSMUSG00000053178 Gm9897 protein_coding 1.027 81.823
0.921 83.733 1.058 39.925 0.893 87.735
ENSMUSG00000053181 A830005F24Rik protein_coding 0.828 86.843
0.979 96.847 1.231 84.626 1.071 96.183
ENSMUSG00000053182 Gm609 protein_coding 0.82 66.129 0.966
95.088 1.257 72.695 1.181 92.742
ENSMUSG00000053184 Spaca3 protein_coding 1 57.544 1
86.156 1 86.985 1 92.381
ENSMUSG00000053185 Gm9898 protein_coding 1 79.068 1
91.643 1 70.44 1 90.628
ENSMUSG00000053192 Mllt11 protein_coding 1.051 84.564
0.996 96.68 0.955 43.842 1.047 75.198
ENSMUSG00000053194 Cib4 protein_coding 0.694 34.305 1.148
92.561 1.258 71.615 1.091 89.152
ENSMUSG00000053198 Prx protein_coding 0.84 23.319 NA NA
1.094 32.433 1.043 91.872
ENSMUSG00000053199 Arhgap20 protein_coding 1.065 74.147
0.964 95.504 0.972 86.144 1.007 95.35
ENSMUSG00000053205 Styx protein_coding 1.036 30.984 0.934
90.378 1.031 46.575 1.046 94.99
ENSMUSG00000053208 Kat2b-ps pseudogene 1 72 1 92.404
1 86.602 1 95.74
ENSMUSG00000053211 Zfy1 protein_coding 1 83.263 1
96.268 1 81.032 1 96.042
ENSMUSG00000053214 Gm9899 pseudogene 0.661 36.034 0.909
93.469 1.662 30.107 0.71 85.493
ENSMUSG00000053216 Btn2a2 protein_coding 1.422 58.896 0.71
79.475 0.988 85.792 0.997 96.287
ENSMUSG00000053217 Tas2r136 protein_coding 1 76.968 1
96.68 1 83.066 1 96.358
ENSMUSG00000053218 Gm9900 protein_coding 0.833 7.857 0.989
96.68 1.209 27.533 0.93 92.31
ENSMUSG00000053219 Raet1e protein_coding 1.445 58.896
0.854 90.256 NA NA 0.747 86.699
ENSMUSG00000053226 Dand5 protein_coding 1.019 58.198 1.01
95.329 0.972 49.967 0.938 86.699
ENSMUSG00000053244 Gm9901 pseudogene 0.584 18.731 0.825
93.654 2.073 25.966 1.601 81.251
ENSMUSG00000053253 Ndfip2 protein_coding 1.03 40.635
0.975 87.041 0.993 58.516 0.97 91.625
ENSMUSG00000053263 Gm12592 processed_transcript 0.888 21.637
1.124 84.999 0.999 58.915 0.688 84.29
ENSMUSG00000053268 Dspp protein_coding 1 82.231 1
96.308 1 85.675 1 93.964
ENSMUSG00000053277 Gm14496 protein_coding 1 63.126 NA

NA	1	84.669	1	92.389
ENSMUSG00000053279	Aldh1a1	protein_coding	0.707	3.848	1.031
96.216	1.375	5.964	1.171	31.133
ENSMUSG00000053286	Trmt1l	protein_coding	0.97	53.138
1.033	97.263	0.995	75.009	0.989	96.287
ENSMUSG00000053287	Olfr1013	protein_coding	1	82.732	1
95.809	1	86.602	1	93.685
ENSMUSG00000053289	Ddx10	protein_coding	1.029	69.089	0.945
79.475	1.027	54.363	1.045	71.305
ENSMUSG00000053291	Rab4b	protein_coding	0.917	21.176	0.849
78.51	1.287	11.932	1.028	94.904
ENSMUSG00000053293	Pom121	protein_coding	0.969	40.786
1.097	71.98	0.944	61.743	0.922	90.289
ENSMUSG00000053297	AI854703	lincRNA	0.988	74.745	0.847
79.475	1.197	70.328	1.072	89.892
ENSMUSG00000053303	Slc22a26	protein_coding	0.533	43.495	NA
NA	1.571	38.099	0.942	83.581
ENSMUSG00000053310	Nrgn	protein_coding	0.874	48.146	1.167
83.104	0.979	87.336	0.937	94.654
ENSMUSG00000053317	Sec61b	protein_coding	0.912	8.181	1.071
74.663	1.021	64.024	1.082	68.696
ENSMUSG00000053318	Slamf8	protein_coding	1.172	29.536
1.203	84.768	0.708	14.206	1.022	96.071
ENSMUSG00000053329	D10Jhu81e	protein_coding	0.868	0.987	1.033
90.68	1.116	11.518	0.996	94.608
ENSMUSG00000053332	Gas5	processed_transcript	1.061	33.854
0.846	22	1.117	36.578	1.127	78.948
ENSMUSG00000053333	Dis3l2	protein_coding	1.078	9.259	0.965
84.594	0.964	36.499	1.042	92.018
ENSMUSG00000053334	Ficd	protein_coding	1.101	19.477	1.124
81.127	0.808	4.48	0.989	96.248
ENSMUSG00000053338	Tarm1	protein_coding	0.794	75.298	0.827
93.654	1.518	54.163	2.797	31.133
ENSMUSG00000053347	Zfp943	protein_coding	1.15	10.687
0.932	74.483	0.935	41.928	0.971	93.964
ENSMUSG00000053353	2310001K24Rik	protein_coding	0.914	33.745
1.166	96.68	0.936	59.274	0.78	84.29
ENSMUSG00000053358	Gm9905	protein_coding	0.88	50.519
1.354	72.642	0.836	49.453	0.837	85.493
ENSMUSG00000053360	F730035P03Rik	protein_coding	1	68.16	1
94.262	1	87.971	1	96.183
ENSMUSG00000053375	Atp6v1e2	protein_coding	1	82.352	1
97.155	1	78.293	1	88.387
ENSMUSG00000053388	Trim50	protein_coding	1	72.546	1
91.897	1	61.171	1	96.183
ENSMUSG00000053389	Tas2r107	protein_coding	1	86.112	1
95.504	1	86.831	1	96.287
ENSMUSG00000053390	Zfp952	protein_coding	1.019	57.38	1.021
92.914	0.957	78.293	1.023	96.248
ENSMUSG00000053391	Olfr211	protein_coding	1	80.03	NA	NA
1	87.786	1	95.405
ENSMUSG00000053395	Cacng8	protein_coding	1.942	46.013
0.689	94.102	0.749	69.974	0.747	90.805
ENSMUSG00000053398	Phgdh	protein_coding	0.807	0.987	1.148	60.309

1.078 23.41 0.933 95.273
ENSMUSG00000053399 Adamts18 protein_coding 0.979 86.819 1.13
91.643 0.903 67.962 1.148 78.379
ENSMUSG00000053411 Cbx7 protein_coding 1.103 33.099 0.958
93.094 0.948 49.967 0.806 77.245
ENSMUSG00000053414 Hunk protein_coding 0.944 60.003 1.326
60.309 0.796 15.465 1.004 83.276
ENSMUSG00000053420 Gm4792 protein_coding 1 57.544 1
89.996 1 88.657 1 92.762
ENSMUSG00000053436 Mapk14 protein_coding 1.056 10.687
0.982 96.268 0.965 4.48 1.021 87.134
ENSMUSG00000053441 Adamts19 protein_coding 1.751 3.893 0.951
91.532 0.601 15.465 1.925 31.133
ENSMUSG00000053442 4930597O21Rik protein_coding 0.814 48.036
0.978 97.155 1.252 67.562 1.038 90.865
ENSMUSG00000053453 Thoc7 protein_coding 1.096 21.539 0.978
91.532 0.933 40.338 1.046 76.697
ENSMUSG00000053460 Ggcx protein_coding 0.968 44.922 1.064
67.462 0.973 48.577 1.003 96.358
ENSMUSG00000053461 Hhipl2 protein_coding 0.816 35.449
0.921 93.146 1.325 63.665 0.914 86.445
ENSMUSG00000053465 Hs6st3 protein_coding 0.836 82.543 1.91
96.335 0.624 80.163 0.564 88.053
ENSMUSG00000053467 A530088E08Rik protein_coding 0.909 71.915
NA NA 1.22 86.727 0.963 93.584
ENSMUSG00000053469 Tg protein_coding 0.753 78.224 0.898
91.83 1.472 51.596 1.12 90.611
ENSMUSG00000053470 Kdm3a protein_coding 0.942 40.786 0.917
65.819 1.153 7.855 0.933 86.699
ENSMUSG00000053472 Gm13534 pseudogene 1 85.129 NA NA
1 88.161 1 95.273
ENSMUSG00000053475 Tnfaip6 protein_coding 1.237 54.44 0.968
93.27 0.833 29.217 1.027 61.984
ENSMUSG00000053477 Tcf4 protein_coding 1.043 19.893 1.018
93.654 0.943 29.217 1.034 94.904
ENSMUSG00000053483 Usp21 protein_coding 0.951 77.407 1.019
96.763 1.033 78.864 1.018 92.851
ENSMUSG00000053490 Trim60 protein_coding 1 81.178 1
93.908 1 74.082 1 94.654
ENSMUSG00000053499 Gm5444 protein_coding 1 73.401 1
95.504 1 88.461 1 95.676
ENSMUSG00000053508 BC048502 protein_coding 1 71.763 1
97.155 1 65.8 1 93.734
ENSMUSG00000053510 Nrd1 protein_coding 1.038 59.527 0.957
92.631 1.007 82.31 1.066 31.133
ENSMUSG00000053522 Lgals7 protein_coding 0.568 5.962 1.109
92.793 1.581 13.709 1.074 96.287
ENSMUSG00000053528 A530021J07Rik protein_coding 1 76.396
1 95.708 1 80.015 1 95.877
ENSMUSG00000053536 Cstf2t protein_coding 1.061 17.582
1.019 93.899 0.923 10.934 1.01 91.525
ENSMUSG00000053541 Gm4759 pseudogene 0.245 22.142 1.26
87.609 3.237 54.809 3.21 92.018
ENSMUSG00000053545 6430503K07Rik protein_coding 1 85.355

1 96.216 1 72.695 1 95.74
ENSMUSG00000053550 Shisa7 protein_coding 1 76.396
1.026 96.948 NA NA 0.752 84.19
ENSMUSG00000053552 Ebf4 protein_coding 1.143 66.272 0.818
85.242 1.07 80.089 0.898 83.952
ENSMUSG00000053553 3110082I17Rik protein_coding 0.92 24.344
0.815 28.822 1.334 5.864 1.044 80.573
ENSMUSG00000053559 Smagp protein_coding 0.939 19.83 1.002 96.68
1.062 39.439 1.01 95.273
ENSMUSG00000053560 Ier2 protein_coding 0.839 22.237 1.101
96.847 1.082 19.877 1.041 89.108
ENSMUSG00000053565 Eif3k protein_coding 1.028 45.746 0.954
45.961 1.019 67.163 1.003 96.183
ENSMUSG00000053574 4930563E22Rik processed_transcript 1.037
74.147 0.913 87.452 1.058 88.729 0.743 85.493
ENSMUSG00000053580 Tanc2 protein_coding 0.951 40.568 0.882
86.538 1.193 47.512 1.04 93.964
ENSMUSG00000053581 Zfand2a protein_coding 1.019 66.272
1.004 93.654 0.98 86.084 1.091 76.831
ENSMUSG00000053583 Gm9912 protein_coding 1 84.534 1
95.504 1 83.158 1 94.82
ENSMUSG00000053588 A730061H03Rik protein_coding 0.737 39.612
1.011 97.263 1.343 42.522 1.039 87.651
ENSMUSG00000053600 Zfp472 protein_coding 1.207 2.969 0.865
35.072 0.954 86.727 1.026 96.192
ENSMUSG00000053603 4930442H23Rik protein_coding 0.687 10.143
1.319 79.411 1.099 70.534 0.923 86.991
ENSMUSG00000053604 Rpia protein_coding 1.038 39.416 1.032
88.823 0.934 6.858 1.008 85.493
ENSMUSG00000053615 Gm9913 protein_coding 0.934 80.608 NA
NA 1.14 86.831 0.866 86.699
ENSMUSG00000053617 Sh3pxd2a protein_coding 1.181 40.333 1.12
92.062 0.757 27.321 0.746 83.724
ENSMUSG00000053624 Gykl1 protein_coding 1 86.351 1
95.504 1 84.626 1 93.156
ENSMUSG00000053626 Tll1 protein_coding 1.298 1.242 1.037 90.29
0.742 0.879 0.906 84.29
ENSMUSG00000053640 Gm9915 protein_coding 1 84.534 1
97.155 1 81.492 1 96.287
ENSMUSG00000053641 Dennd4a protein_coding 1.062 66.76 1.027
96.68 0.915 33.137 0.987 96.086
ENSMUSG00000053644 Aldh7a1 protein_coding 0.924 11.232
1.009 97.155 1.072 25.564 1.077 55.485
ENSMUSG00000053646 Plxnb1 protein_coding 0.725 10.704
1.183 82.904 1.167 34.727 0.936 83.724
ENSMUSG00000053647 Gper1 protein_coding 1.004 71.896 NA NA
0.966 68.266 1.033 94.297
ENSMUSG00000053654 Krt42 protein_coding 0.577 32.749 1.183
92.793 1.468 50.636 1.129 93.722
ENSMUSG00000053656 Dancr processed_transcript 0.95 34.162
1.147 79.055 0.92 81.388 1.083 77.621
ENSMUSG00000053664 Uck2 protein_coding 1.021 86.819 0.984
96.216 0.992 78.954 1.03 95.676
ENSMUSG00000053675 Tgm5 protein_coding 1.377 51.737 0.339

56.955 2.138 45.208 0.951 94.654
ENSMUSG00000053678 Defb40 protein_coding 1 85.857 1
96.268 1 83.538 1 96.248
ENSMUSG00000053684 BC048403 protein_coding 0.981 81.657
0.987 90.744 1.026 82.436 0.973 87.182
ENSMUSG00000053687 Dpep2 protein_coding 1.628 3.498 0.919 96.216
0.669 8.907 1.753 70.539
ENSMUSG00000053693 Mast1 protein_coding 0.839 31.11 0.995 91.776
1.2 56.708 0.924 83.724
ENSMUSG00000053695 NA NA NA NA 1 95.211 1
88.601 1 93.132
ENSMUSG00000053702 Nebl protein_coding 1.008 86.74 0.911 86.604
1.09 73.428 0.945 85.493
ENSMUSG00000053706 B430305J03Rik protein_coding 0.768 16.02 1.24
72.546 1.048 79.325 0.833 83.952
ENSMUSG00000053714 4732471J01Rik protein_coding 0.76 1.274 1
94.767 1.316 16.205 0.924 84.29
ENSMUSG00000053716 Dusp7 protein_coding 1.098 35.024 1.162
61.943 0.784 2.563 0.886 83.724
ENSMUSG00000053719 Klk1b26 protein_coding 1 76.968 1
96.847 1 86.985 1 95.273
ENSMUSG00000053720 Vmn2r43 protein_coding 1 85.637 NA
NA 1 81.032 1 95.832
ENSMUSG00000053729 Spinkl protein_coding 1 86.464 NA
NA 1 86.112 1 96.358
ENSMUSG00000053730 Tmem39b protein_coding 1.064 45.746
1.037 82.904 0.904 9.746 0.969 87.735
ENSMUSG00000053742 Gm5114 protein_coding 1 84.738 1
97.155 1 86.258 1 92.691
ENSMUSG00000053746 Ptrh1 protein_coding 0.999 63.126 1.045
97.155 0.955 79.554 1.031 93.261
ENSMUSG00000053747 Sox14 protein_coding 0.855 76.827 1.5
94.367 0.781 61.288 0.9 85.493
ENSMUSG00000053749 Gm9920 protein_coding 0.957 58.56 1.172
79.29 0.894 64.427 0.903 91.105
ENSMUSG00000053754 Chd8 protein_coding 1.003 80.751 0.943
11.228 1.055 5.864 0.955 91.979
ENSMUSG00000053768 Chchd3 protein_coding 0.954 38.286 NA
NA 1.066 44.515 0.973 95.832
ENSMUSG00000053769 Lysmd1 protein_coding 0.917 3.47 0.931
56.027 1.173 5.864 1.011 90.674
ENSMUSG00000053773 Rdh8 protein_coding 1 81.823 1
95.708 1 88.274 1 96.248
ENSMUSG00000053774 Ubxn7 protein_coding 0.998 84.661 0.898
74.851 1.116 36.578 0.964 83.724
ENSMUSG00000053783 1700016K19Rik protein_coding 0.996 81.52
0.882 97.155 1.138 87.686 1.156 95.92
ENSMUSG00000053790 Defb38 protein_coding 1 83.169 1
96.216 1 88.729 1 95.35
ENSMUSG00000053797 Krt16 protein_coding 1 84.564 1
95.941 1 87.971 1 90.95
ENSMUSG00000053799 Exoc6 protein_coding 1.131 7.908 0.963 92.631
0.919 18.539 0.954 94.654
ENSMUSG00000053801 Grwd1 protein_coding 0.892 81.01 1.12 92.561

1 86.831 1.141 71.305
ENSMUSG00000053815 Olfr744 protein_coding 1 85.728 1
95.211 NA NA 1 93.132
ENSMUSG00000053819 Camk2d protein_coding 1.064 27.113
1.112 83.104 0.848 3.943 1.004 88.955
ENSMUSG00000053820 Bcl2a1c protein_coding 1 82.982 NA
NA 1 85.921 1 95.273
ENSMUSG00000053821 Gm9922 protein_coding 0.645 8.405 1.086
89.434 1.424 36.011 0.92 95.72
ENSMUSG00000053825 Ppfia2 protein_coding 0.999 81.01 1.123
88.551 0.889 72.464 1.106 93.734
ENSMUSG00000053830 Gm9923 protein_coding 1 77.182 1
92.474 1 74.082 1 95.273
ENSMUSG00000053835 H2-T24 protein_coding 1.484 2.361 0.811
60.678 0.83 76.768 1.043 74.607
ENSMUSG00000053838 Nudcd3 protein_coding 0.968 19.224
1.017 83.104 1.016 77.532 0.979 86.991
ENSMUSG00000053839 Gm9924 protein_coding 1 83.968 NA
NA 1 88.729 1 92.742
ENSMUSG00000053841 Txlna protein_coding 0.935 7.58 1.052 82.904
1.011 61.743 0.992 96.248
ENSMUSG00000053846 Lipg protein_coding 0.778 8.267 1.306 82.904
0.981 68.643 1.703 83.067
ENSMUSG00000053852 NA NA NA NA 1 95.504 1
84.071 1 91.872
ENSMUSG00000053856 Dnajc5g protein_coding 1 81.925 1
96.68 1 86.144 1 94.654
ENSMUSG00000053862 Slc51b protein_coding 0.99 83.263
0.907 97.155 1.112 72.378 0.717 84.29
ENSMUSG00000053863 Mepe protein_coding 1 84.865 1
90.576 1 70.1 1 95.777
ENSMUSG00000053868 Gm5142 protein_coding 1 68.353 1
96.847 NA NA 1 96.183
ENSMUSG00000053870 Fpgt protein_coding 1.183 0.577 1.003 97.129
0.841 1.342 0.91 84.19
ENSMUSG00000053873 Aym1 protein_coding 1 78.744 1
97.155 1 74.686 1 95.559
ENSMUSG00000053877 Srcap protein_coding 0.937 43.495 1.039
83.104 1.028 86.602 0.995 88.387
ENSMUSG00000053886 Sh2d4a protein_coding 1.252 3.061 0.932
67.165 0.858 24.163 0.926 90.157
ENSMUSG00000053889 A130066N16Rik antisense 0.632 11.103
1.688 79.29 0.939 81.032 0.816 91.105
ENSMUSG00000053896 4933409G03Rik protein_coding 1 83.333
NA NA 1 86.144 1 95.273
ENSMUSG00000053897 Slc39a8 protein_coding 1.234 5.559 0.952
95.504 0.847 14.409 0.956 84.29
ENSMUSG00000053898 Ech1 protein_coding 1.012 77.291 0.975
95.35 1.014 88.203 1.038 89.892
ENSMUSG00000053906 Mafg pseudogene 1 71.36 1 97.129 1
84.071 1 91.65
ENSMUSG00000053907 Mat2a protein_coding 1.181 0.2 0.961 64.555
0.88 11.751 0.957 85.493
ENSMUSG00000053909 Rhox10 protein_coding 1 86.819 NA

NA 1 83.811 1 95.74
ENSMUSG00000053914 Kdm4d protein_coding 1.082 84.738 0.861
94.597 NA NA 0.926 96.086
ENSMUSG00000053916 Nanp protein_coding 0.928 57.014 1.015
95.708 1.061 71.615 1.153 49.769
ENSMUSG00000053925 Gm9929 processed_transcript 0.636 13.062
1.119 95.431 1.404 30.561 0.809 84.19
ENSMUSG00000053929 Cyhr1 protein_coding 0.901 1.698 1.029 82.904
1.077 10.523 0.956 84.29
ENSMUSG00000053930 Shisa6 protein_coding 1.123 66.272
1.429 71.617 0.624 16.719 0.903 92.181
ENSMUSG00000053931 Cnn3 protein_coding 0.972 85.857 1.056
90.68 0.969 70.791 1.139 71.008
ENSMUSG00000053935 Atf7ip protein_coding 1.048 20.799
1.007 91.361 0.945 53.7 0.982 83.724
ENSMUSG00000053950 Adnp2 protein_coding 1.235 0.461 0.935 72.399
0.866 4.723 0.952 85.493
ENSMUSG00000053957 Gm12474 lincRNA 0.653 49.047 1.343
96.68 1.142 61.841 1.08 91.335
ENSMUSG00000053961 Ang5 protein_coding 1 73.585 1
93.654 1 86.666 1 96.358
ENSMUSG00000053963 6330403A02Rik protein_coding 1.292 8.537
1.167 82.904 0.662 1.342 1.007 88.955
ENSMUSG00000053964 Lgals4 protein_coding 0.953 62.888
0.935 31.637 1.119 19.025 0.928 85.493
ENSMUSG00000053965 Pde5a protein_coding 1.014 82.982 0.908
80.463 1.082 55.81 0.95 86.991
ENSMUSG00000053977 Cd8a protein_coding 1.033 45.096 1.738
95.809 0.554 30.024 0.867 90.157
ENSMUSG00000053979 A730045E13Rik protein_coding 1 80.408
1 97.155 1 79.897 1 92.181
ENSMUSG00000053985 Zfp14 protein_coding 1.009 85.129 0.908
75.345 1.093 58.414 1.056 89.152
ENSMUSG00000053988 Gm9931 protein_coding 1 83.523 1
95.211 1 75.809 1 95.676
ENSMUSG00000054000 Tusc1 protein_coding 0.785 4.993 1.011 96.68
1.262 17.751 0.977 92.12
ENSMUSG00000054005 Mill1 protein_coding 0.725 18.379 0.931
85.418 1.482 18.411 0.782 84.29
ENSMUSG00000054006 D630008O14Rik antisense 0.739 51.089
1.458 93.146 0.928 81.492 0.64 85.493
ENSMUSG00000054008 Ndst1 protein_coding 1.176 6.853 0.976 91.532
0.875 9.563 0.951 87.735
ENSMUSG00000054013 Tmem179 protein_coding 0.58 1.239 1.258
82.904 1.371 11.151 0.753 86.445
ENSMUSG00000054021 Sirt5 protein_coding 0.894 8.302 NA NA
1.039 32.524 0.982 94.904
ENSMUSG00000054027 Nt5dc3 protein_coding 1.031 14.59 1.078
83.104 0.902 5.628 0.998 96.287
ENSMUSG00000054031 Rdh18-ps pseudogene 1 56.242 1
95.196 1 69.728 1 96.248
ENSMUSG00000054032 Gm9932 protein_coding 1 81.647 1
96.407 1 83.925 1 95.877
ENSMUSG00000054033 Adam39 protein_coding 1 81.76 1

95.504	1	86.666	1	96.086
ENSMUSG00000054034	Tceal5	protein_coding	1.465	17.761	1.02
91.456	0.668	15.169	1.026	93.117
ENSMUSG00000054036	Olfr279	protein_coding	1	63.534	NA
NA	1	82.778	1	91.105
ENSMUSG00000054044	Gm9933	protein_coding	0.823	34.305
1.078	97.155	1.128	28.301	0.891	92.466
ENSMUSG00000054046	Klk13	protein_coding	1.264	74.482	0.922
88.705	0.855	86.144	0.51	84.19
ENSMUSG00000054051	Ercc6	protein_coding	1.024	76.827	0.975
86.277	1.002	80.634	0.896	83.724
ENSMUSG00000054054	Olfr309	protein_coding	1	84.168	1
95.504	1	87.431	1	94.904
ENSMUSG00000054057	A930004D18Rik	protein_coding	0.858	28.242
1.109	82.904	1.046	57.286	0.927	95.593
ENSMUSG00000054061	Gm9934	protein_coding	0.885	54.043
1.158	95.735	0.972	84.961	0.68	83.724
ENSMUSG00000054065	Pkp3	protein_coding	0.87	21.176	0.91
85.418	1.266	16.808	0.797	84.29
ENSMUSG00000054069	6030452D12Rik	protein_coding	1	82.982
1	95.431	1	73.759	1	83.067
ENSMUSG00000054072	Iigp1	protein_coding	1.409	3.598	0.968	96.335
0.732	6.858	0.825	83.724
ENSMUSG00000054074	Skida1	protein_coding	0.725	7.118	1.148
82.904	1.204	29.302	0.982	92.851
ENSMUSG00000054079	Utp18	protein_coding	1.065	28.395	0.956
87.857	0.982	46.575	1.177	51.908
ENSMUSG00000054083	Capn12	protein_coding	0.819	33.002
1.099	91.065	1.113	48.451	0.806	85.493
ENSMUSG00000054087	Fbxw14	protein_coding	1	69.489	1
96.216	1	84.961	1	87.624
ENSMUSG00000054091	1810037I17Rik	protein_coding	0.971	44.787
1.039	82.904	0.992	85.675	0.939	92.561
ENSMUSG00000054099	Slc25a40	protein_coding	0.995	80.815
1.003	94.597	1.004	86.084	0.973	93.264
ENSMUSG00000054102	Nlrp9a	protein_coding	1	78.224	NA
NA	1	85.921	1	95.877
ENSMUSG00000054106	Try4	protein_coding	1	86.474	1
96.984	1	87.686	1	93.457
ENSMUSG00000054115	Skp2	protein_coding	1.092	33.854	1.092
96.308	0.839	7.647	1.039	76.697
ENSMUSG00000054116	E130116L18Rik	protein_coding	0.913	74.8
0.926	95.35	1.18	64.024	0.836	83.724
ENSMUSG00000054117	Zdhhc25	protein_coding	1	72.743	NA
NA	1	78.498	1	91.372
ENSMUSG00000054135	A430110L20Rik	lincRNA	0.971	74.021
1.071	90.389	0.959	78.713	0.922	90.691
ENSMUSG00000054141	Olfr24	protein_coding	1	80.251	1
96.652	1	85.935	1	96.183
ENSMUSG00000054142	Vmn1r236	protein_coding	1	77.916	1
96.407	1	88.274	1	96.183
ENSMUSG00000054146	Krt15	protein_coding	0.583	3.641	0.964	96.268
1.783	7.365	0.96	91.335
ENSMUSG00000054150	Syne3	protein_coding	0.865	19.023	0.869

94.262 1.326 7.635 0.847 83.724
ENSMUSG00000054156 Gm4894 protein_coding 1 85.77 NA NA
1 78.713 1 94.016
ENSMUSG00000054160 Nkx2-4 protein_coding 1 83.263 1
96.847 1 84.83 1 96.184
ENSMUSG00000054161 Fam83e protein_coding 2.531 5.559 0.656
60.678 0.604 50.184 1.628 81.56
ENSMUSG00000054162 Spock3 protein_coding 1.213 54.344
1.057 96.847 0.777 55.701 0.999 90.865
ENSMUSG00000054165 4922502B01Rik protein_coding 1 85.355
1 96.948 1 75.637 1 96.287
ENSMUSG00000054169 Ceacam10 protein_coding 0.933 81.52 0.62
71.41 1.733 42.199 0.755 92.181
ENSMUSG00000054178 Gm9938 protein_coding 0.628 3.009 1.003
91.361 1.589 5.864 1.105 73.939
ENSMUSG00000054181 A930012O16Rik processed_transcript 0.943
71.142 1.039 91.532 1.022 62.648 0.93 90.805
ENSMUSG00000054191 Klf1 protein_coding 1.239 38.386 0.88
79.807 0.916 83.925 0.757 83.952
ENSMUSG00000054196 Cthrc1 protein_coding 1.015 67.163 0.89
72.712 1.104 57.227 0.755 95.593
ENSMUSG00000054199 Gon4l protein_coding 0.944 12.181 0.964
96.138 1.099 13.174 1.04 67.691
ENSMUSG00000054200 Ffar4 protein_coding 0.839 7.004 1.067 79.29
1.114 43.185 0.942 86.991
ENSMUSG00000054203 Ifi205 protein_coding 1.148 59.806
1.139 97.155 0.763 80.015 1.139 60.968
ENSMUSG00000054204 Fam150b protein_coding 0.877 44.562
0.659 61.883 1.725 7.143 0.942 92.903
ENSMUSG00000054206 Gzmm protein_coding 0.698 6.677 1.202 83.104
1.191 35.673 1.316 60.466
ENSMUSG00000054215 Sprr2k protein_coding 1 66.129 1
96.216 1 84.83 1 87.134
ENSMUSG00000054226 Tprkb protein_coding 1.067 27.731 1.004
95.301 0.935 23.212 1.044 67.169
ENSMUSG00000054236 Olfr481 protein_coding 1 86.112 1
95.708 1 84.626 1 92.31
ENSMUSG00000054237 Fra10ac1 protein_coding 0.979 81.925
0.948 82.503 1.078 26.978 1.062 79.268
ENSMUSG00000054247 Gm9939 pseudogene 0.574 9.752 1.164 93.899
1.49 19.106 1.012 92.181
ENSMUSG00000054252 Fgfr3 protein_coding 0.824 13.062 0.937
75.052 1.297 10.079 1.103 74.607
ENSMUSG00000054256 Msi1 protein_coding 1.054 84.397 0.852
90.744 1.117 72.695 0.901 86.699
ENSMUSG00000054258 Gm5082 protein_coding 1 84.738 1
95.708 1 80.089 1 95.593
ENSMUSG00000054263 Lifr protein_coding 1.061 73.734 0.844
76.431 1.112 49.384 0.966 90.611
ENSMUSG00000054266 Serpinb9d protein_coding 1 83.523 1
97.263 1 86.258 1 94.215
ENSMUSG00000054272 Zscan4c protein_coding 1 86.006 1
97.263 1 87.519 1 94.904
ENSMUSG00000054277 Arfgap3 protein_coding 1.03 53.562

1.002 91.361 0.969 72.464 1.046 55.485
ENSMUSG00000054280 Prr14l protein_coding 0.99 75.567
0.932 88.704 1.083 40.052 0.977 92.181
ENSMUSG00000054293 A630033H20Rik protein_coding 1.459 8.477
1.139 95.431 0.602 14.788 0.968 93.964
ENSMUSG00000054302 Eapp protein_coding 0.932 14.403 1.019
96.216 1.054 20.556 0.975 90.854
ENSMUSG00000054304 D130007C19Rik pseudogene 1.295 7.332 0.84
64.123 0.916 78.293 0.895 83.952
ENSMUSG00000054309 Cpsf3 protein_coding 1.032 78.639 0.984
87.313 NA NA 1.033 69.708
ENSMUSG00000054310 Obox1 protein_coding 1 86.006 1
95.708 1 85.675 1 96.071
ENSMUSG00000054312 Mrps21 protein_coding 0.878 4.055 1.026
84.126 1.109 22.93 1.017 83.067
ENSMUSG00000054320 Lrrc36 protein_coding 0.906 66.406
0.991 94.887 1.113 49.058 0.946 85.493
ENSMUSG00000054321 Taf4b protein_coding 1.003 82.479 0.943
82.503 1.059 48.451 0.998 79.713
ENSMUSG00000054325 Lce3a protein_coding 1 80.251 1
96.407 1 87.103 1 92.31
ENSMUSG00000054342 Kcnn4 protein_coding 0.849 20.369 0.953
83.58 1.238 10.819 1.031 90.455
ENSMUSG00000054351 4930553M12Rik protein_coding 1 83.169
1 95.504 1 73.598 1 93.964
ENSMUSG00000054360 Bsx protein_coding 1 67.163 1
90.092 1 69.196 1 88.111
ENSMUSG00000054364 Rhob protein_coding 1.011 69.408 0.897
81.134 1.097 75.527 0.901 85.493
ENSMUSG00000054379 NA NA NA NA 1 97.263 1
84.83 1 92.675
ENSMUSG00000054381 Zfp747 protein_coding 0.982 83.726
0.973 94.887 1.049 64.305 0.943 88.245
ENSMUSG00000054383 Pnma1 protein_coding 0.825 84.819 0.88
88.881 1.371 54.673 1.186 86.301
ENSMUSG00000054385 Ceacam2 protein_coding 0.84 81.196
0.979 96.378 1.21 87.431 0.668 92.748
ENSMUSG00000054387 Mdm4 protein_coding 0.928 17.031 0.891
91.361 1.214 10.76 1.029 95.273
ENSMUSG00000054391 4930517O19Rik processed_transcript 1
86.006 1 93.574 1 83.857 1 95.92
ENSMUSG00000054400 Cklf protein_coding 1.151 9.206 1.045 96.216
0.829 15.904 1.014 86.991
ENSMUSG00000054404 Slfn5 protein_coding 1.136 3.061 0.921 91.532
0.956 30.827 1.122 78.379
ENSMUSG00000054405 Dnajc8 protein_coding 0.98 85.946
0.935 24.911 1.093 15.945 1.063 46.846
ENSMUSG00000054406 Olfr411 protein_coding 1 76.686 1
95.708 1 86.144 1 94.904
ENSMUSG00000054408 Spcs3 protein_coding 1.126 7.959 0.964 92.914
0.919 21.666 1.083 50.452
ENSMUSG00000054412 Gm4793 protein_coding 0.851 77.407
0.897 94.848 1.306 53.134 0.898 86.445
ENSMUSG00000054414 Slc30a7 protein_coding 1.077 54.717

0.965 93.654 0.96 80.015 0.999 89.72
ENSMUSG00000054417 Cyp3a44 protein_coding 1 84.263 NA
NA 1 84.896 1 96.248
ENSMUSG00000054418 2900041M22Rik processed_transcript 1.165
71.516 0.956 95.504 0.895 83.811 0.892 94.654
ENSMUSG00000054423 NA NA NA NA 1 93.529 1
83.719 1 95.74
ENSMUSG00000054426 A930005H10Rik protein_coding 0.781 31.11
1.091 82.904 1.171 85.352 0.954 92.181
ENSMUSG00000054428 Atpif1 protein_coding 1.06 72.85 0.853
40.053 1.108 31.4 1.087 72.116
ENSMUSG00000054431 Olfr450 protein_coding 1 86.172 1
91.643 1 86.144 1 96.086
ENSMUSG00000054434 Tmem120b protein_coding 0.625 0.987 1.082
84.999 1.48 5.864 0.936 86.699
ENSMUSG00000054435 Gimap4 protein_coding 1.047 61.725
1.081 82.904 0.885 10.003 1.012 94.19
ENSMUSG00000054446 Cpa1 protein_coding 1 77.407 1
92.111 1 88.076 1 83.067
ENSMUSG00000054452 Aes protein_coding 0.807 1.274 1.081 77.618
1.148 22.517 1.088 93.734
ENSMUSG00000054453 Sytl5 protein_coding 1.047 75.115 0.872
87.857 1.097 57.907 1.056 92.748
ENSMUSG00000054455 Vapb protein_coding 0.937 1.031 1.036 83.104
1.029 19.025 0.983 91.525
ENSMUSG00000054457 9430021M05Rik processed_transcript 1
84.324 1 94.848 1 80.634 1 93.612
ENSMUSG00000054459 Vsnl1 protein_coding 1.217 37.091 1.232
83.104 0.667 12.288 0.993 93.685
ENSMUSG00000054469 Lclat1 protein_coding 1.064 18.314
0.992 96.763 0.948 12.41 1.035 86.209
ENSMUSG00000054474 Thnsl2 protein_coding 0.881 19.985
0.927 86.604 1.224 22.93 0.997 90.611
ENSMUSG00000054477 Kcnn2 protein_coding 0.9 78.538 0.859
95.809 1.294 76.498 0.986 89.72
ENSMUSG00000054484 Tmem62 protein_coding 0.965 39.712
0.946 79.475 1.095 20.051 0.86 77.331
ENSMUSG00000054493 Gm9947 protein_coding 1.14 72.372
0.723 73.094 1.217 72.378 0.736 83.724
ENSMUSG00000054497 NA NA NA NA 1 93.908 1
88.203 1 94.82
ENSMUSG00000054498 Olfr308 protein_coding 1 86.843 NA
NA 1 82.903 1 93.734
ENSMUSG00000054499 Dedd2 protein_coding 0.895 9.841 0.957 87.553
1.164 7.635 0.994 95.877
ENSMUSG00000054509 Parp4 protein_coding 0.986 58.802 1.052
83.104 0.967 66.201 0.939 83.724
ENSMUSG00000054510 Gm14461 lincRNA 1 76.135 NA NA
1 68.643 1 95.877
ENSMUSG00000054514 2610204G22Rik antisense 1.069 85.655
0.753 79.475 1.243 27.732 0.953 89.294
ENSMUSG00000054517 Trim65 protein_coding 1.058 43.939 1.1
92.561 0.859 16.051 0.979 92.675
ENSMUSG00000054519 Zfp867 protein_coding 1.076 72.372

0.966 82.848	0.963 81.032	0.981 86.991
ENSMUSG00000054520	Sh3bp2	protein_coding	1.222 8.102 0.962
89.996	0.847 28.237	1.028 92.564
ENSMUSG00000054523	Ms4a5 protein_coding	1	80.688	1
96.68 1	87.173	1	92.31
ENSMUSG00000054526	Olfr1500	protein_coding	1	86.199	1
95.504	1	86.475	1	95.273
ENSMUSG00000054545	Ugt1a6a	protein_coding	0.898 56.242
0.914 90.378	1.221 37.741	1.084 94.654
ENSMUSG00000054555	Adam12	protein_coding	0.762 19.595
1.289 71.617	1.016 79.325	1.188 51.967
ENSMUSG00000054556	Gm4876	antisense	0.84	29.454	1.231
74.663	NA	NA	1.014 96.086
ENSMUSG00000054568	Usp17la	protein_coding	1	81.465	1
95.708	1	79.776	1	94.904
ENSMUSG00000054580	Pla2r1	protein_coding	1.112 19.315	0.97
81.731	0.93	77.704	0.959 93.612
ENSMUSG00000054582	Pabpc1l	protein_coding	0.687 0.987 0.959
90.138	1.516 5.864 0.967 83.067
ENSMUSG00000054588	Gbp10 protein_coding	0.63	23.319	1.062
88.91 1.49	48.772	1.095 96.287
ENSMUSG00000054589	Gm9949	protein_coding	1.167 58.198
0.947 93.765	0.904 54.25 0.996 93.612
ENSMUSG00000054594	Oscar protein_coding	0.821 39.174	1.522
78.705	0.801 64.155	0.953 95.593
ENSMUSG00000054598	9130230L23Rik	protein_coding	0.351 2.009
1.538 77.598	1.857 26.912	0.944 92.181
ENSMUSG00000054604	Cggbp1	protein_coding	1.058 26.863
0.955 92.137	0.986 48.851	0.957 84.29
ENSMUSG00000054611	Kdm2a protein_coding	1.06	22.205	1.05
82.904	0.895 7.809 0.958 88.505
ENSMUSG00000054612	Mgmt	protein_coding	0.913 22.237	1.048
90.116	1.041 58.915	0.985 94.904
ENSMUSG00000054618	Gm9951	lincRNA	0.704 39.612	1.532
60.309	0.925 45.093	1.936 59.394
ENSMUSG00000054619	Mettl7a1	protein_coding	1.016 75.196	NA
NA	0.973 74.179	1.185 76.831
ENSMUSG00000054626	Xlr	protein_coding	1.861 0.664 0.797 79.475
0.674 9.746 0.95	95.877
ENSMUSG00000054630	Ugt2b5	protein_coding	1	79.068	1
95.708	1	88.657	1	94.99
ENSMUSG00000054640	Slc8a1	protein_coding	1.52	2.651 0.925
82.503	0.712 16.719	1.02	83.067
ENSMUSG00000054641	Mmrn1 protein_coding	1.165 12.855	1.102
95.35 0.781 39.245	0.959 89.892
ENSMUSG00000054648	Zfp869	protein_coding	1.136 16.436
0.848 38.699	1.034 52.8	1.062 70.539
ENSMUSG00000054659	Pm20d2	protein_coding	1.452 7.492 0.955
95.301	0.722 2.722 0.921 86.699
ENSMUSG00000054662	Ano9	protein_coding	0.899 43.435	0.808
74.851	1.377 13.174	0.845 93.316
ENSMUSG00000054666	Olfr63	protein_coding	1	81.24 1
96.847	1	85.921	1	95.273
ENSMUSG00000054667	Irs4	protein_coding	1	86.145	1

90.712 1 70.328 1 93.734
ENSMUSG00000054672 5830411N06Rik protein_coding 1.648 83.169
0.514 96.138 1.181 88.274 0.638 89.587
ENSMUSG00000054675 Tmem119 protein_coding 1.54 0.461 0.929
96.407 0.7 0.72 1.268 32.698
ENSMUSG00000054676 1600014C10Rik protein_coding 0.765 2.524
1.083 84.126 1.206 5.864 1.002 90.611
ENSMUSG00000054679 Srsf12 protein_coding 2.037 10.595
0.397 61.883 1.233 79.426 0.794 85.493
ENSMUSG00000054690 Emcn protein_coding 1.123 3.498 NA NA
0.894 0.879 0.998 87.331
ENSMUSG00000054693 Adam10 protein_coding 1.128 25.944
0.867 37.426 1.019 58.915 0.932 92.12
ENSMUSG00000054702 Ap1s3 protein_coding 0.967 59.674 0.874
70.29 1.184 33.787 1.013 93.722
ENSMUSG00000054708 Ankrd24 protein_coding 0.904 26.77 0.979
91.532 1.133 22.994 0.884 83.724
ENSMUSG00000054714 Gm9954 protein_coding 1 72.372 1
86.538 1 88.203 1 96.086
ENSMUSG00000054715 Zscan22 protein_coding 1.027 53.562
1.059 83.104 0.917 10.362 1.01 93.964
ENSMUSG00000054716 Zfp771 protein_coding 0.899 17.401
1.078 93.707 1.03 47.776 0.998 87.331
ENSMUSG00000054717 Hmgb2 protein_coding 0.919 79.709 0.861
50.715 1.262 51.243 1.199 74.193
ENSMUSG00000054720 Lrrc8c protein_coding 1.122 6.541 0.885
45.262 NA NA 1.068 91.928
ENSMUSG00000054723 Vmac protein_coding 0.944 23.319 1.015
82.904 1.04 86.258 1.025 95.273
ENSMUSG00000054727 1700013H16Rik protein_coding 0.809 72.743
1.124 95.809 1.095 87.971 1.274 93.117
ENSMUSG00000054728 Phactr1 protein_coding 1.375 0 0.941
93.046 0.775 2.687 1.005 92.389
ENSMUSG00000054733 Msra protein_coding 0.789 0.987 1.127 71.222
1.125 28.979 1.09 93.722
ENSMUSG00000054737 Zfp182 protein_coding 1.073 34.077
0.946 85.418 0.985 78.293 0.959 96.358
ENSMUSG00000054746 Abca15 protein_coding 1 74.482 1
96.378 1 87.744 1 95.74
ENSMUSG00000054752 Fsd1l protein_coding 0.97 65.622 0.89
86.277 1.16 40.575 0.988 92.018
ENSMUSG00000054753 AU018091 protein_coding 0.721 23.923
1.544 71.98 0.895 88.601 0.742 85.493
ENSMUSG00000054757 Akr1c20 protein_coding 1 79.277 1
94.848 1 82.903 1 80.187
ENSMUSG00000054758 Gm9956 protein_coding 0.931 86.145
0.726 90.712 1.477 39.439 0.496 83.952
ENSMUSG00000054759 Krtap5-2 protein_coding 1 80.146 1
96.984 1 84.229 1 96.183
ENSMUSG00000054764 Mtnr1a protein_coding 1 80.751 1
93.654 1 87.898 1 92.762
ENSMUSG00000054766 Set protein_coding 1 64.284 1.021
91.776 0.978 83.538 1.056 57.1
ENSMUSG00000054770 Kctd18 protein_coding 0.976 44.252 0.9

15.364	1.136	5.864	0.963	96.183
ENSMUSG00000054773	4930566N20Rik	protein_coding	1	79.433
1	94.597	1	82.74	1	96.086
ENSMUSG00000054779	Gm12541	antisense	1.34	62.689	0.861
95.708	0.867	80.015	1.514	78.948
ENSMUSG00000054792	Klhl18	protein_coding	0.999	62.007
1.018	91.776	0.981	46.071	1.002	91.291
ENSMUSG00000054793	Cadm4	protein_coding	0.977	69.24	0.979	93.654
1.045	65.567	0.917	83.724
ENSMUSG00000054808	Actn4	protein_coding	0.919	7.881	1.002	93.654
1.088	10.76	0.969	84.29
ENSMUSG00000054814	Usp46	protein_coding	1.016	69.089	1.067
92.561	0.923	63.504	0.961	95.273
ENSMUSG00000054822	1700041G16Rik	protein_coding	1.028	78.538
0.934	95.301	1.043	80.015	0.968	93.612
ENSMUSG00000054823	Whsc1l1	protein_coding	1.138	0.2	0.962
54.427	0.91	21.431	1.02	93.156
ENSMUSG00000054827	Cyp2c50	protein_coding	1	81.657	1
96.138	1	87.519	1	90.397
ENSMUSG00000054836	Elp6	protein_coding	0.931	40.568	1.022
96.68	1.048	65.8	1.028	87.624
ENSMUSG00000054843	Atrnl1	protein_coding	1.064	54.44	1.087
81.039	0.861	20.511	0.978	92.748
ENSMUSG00000054850	6330419J24Rik	protein_coding	1.551	0.461
0.712	52.266	0.9	62.791	1.172	79.442
ENSMUSG00000054855	Rnd1	protein_coding	0.836	41.617	0.921
87.857	1.3	27.047	1.125	95.273
ENSMUSG00000054863	Fam19a5	protein_coding	1.067	81.196
1.086	85.756	0.861	7.329	0.841	83.724
ENSMUSG00000054871	Tmem158	protein_coding	0.968	73.401
1.176	82.904	0.881	16.051	1.066	91.105
ENSMUSG00000054874	Pcnxl3	protein_coding	0.963	38.827
1.024	81.039	1.014	86.258	0.974	83.724
ENSMUSG00000054889	Dsp	protein_coding	0.825	10.014	0.909
88.881	1.336	7.365	0.818	83.724
ENSMUSG00000054890	Olfr1535	protein_coding	1	82.891	1
97.097	NA	NA	1	96.183
ENSMUSG00000054892	Txk	protein_coding	1.201	74.745	0.909
96.216	0.914	75.967	0.835	85.636
ENSMUSG00000054893	Zfp667	protein_coding	1.441	0.664	0.915
65.165	0.757	10.362	0.978	94.468
ENSMUSG00000054894	Atp5s	protein_coding	1.13	6.026	0.973	95.088
0.91	10.602	0.927	83.724
ENSMUSG00000054901	Arhgef33	protein_coding	0.716	71.915	NA
NA	1.399	79.897	1.031	87.218
ENSMUSG00000054905	Stfa3	protein_coding	1	71.896	1
97.049	1	86.727	1	89.294
ENSMUSG00000054909	Wbscr25	processed_transcript	0.629	41.759
0.776	85.671	2.042	27.431	0.921	86.699
ENSMUSG00000054910	4931415C17Rik	processed_transcript	1.086
85.77	1.23	95.708	0.746	87.744	1.328	89.152
ENSMUSG00000054920	Klhl5	protein_coding	1.166	0.984	1.003	96.001
0.857	1.061	1.014	95.593
ENSMUSG00000054931	Zkscan4	protein_coding	0.974	81.465

1.013 93.654 1.016 68.643 0.968 95.72
ENSMUSG00000054934 Kcnmb4 protein_coding 1.231 43.939 0.94
95.211 0.865 87.971 0.739 83.724
ENSMUSG00000054938 Olfr1346 protein_coding 1 85.832 NA
NA 1 82.74 1 95.559
ENSMUSG00000054939 Zfp174 protein_coding 0.94 48.939
0.901 85.116 1.176 47.31 0.988 85.493
ENSMUSG00000054940 Olfr137 protein_coding 1 76.042 NA
NA 1 88.601 1 92.561
ENSMUSG00000054942 Fam73a protein_coding 1.177 7.553 0.926
82.671 0.92 49.967 0.824 84.29
ENSMUSG00000054945 Gm9958 protein_coding 1.052 66 NA NA
0.897 85.21 0.981 96.183
ENSMUSG00000054951 9130008F23Rik protein_coding 0.753 9.599
1.098 87.185 1.206 34.403 1.032 94.153
ENSMUSG00000054957 Gm9959 pseudogene 0.975 65.106 1.029
90.092 0.995 79.325 0.787 86.699
ENSMUSG00000054958 Nt5c1a protein_coding 1.761 35.024
2.111 82.904 0.269 4.48 0.666 90.805
ENSMUSG00000054966 Ifltd1 protein_coding 0.898 86.474
0.917 95.211 1.217 82.685 1.242 92.748
ENSMUSG00000054967 Zfp647 protein_coding 1.026 49.047
0.909 92.355 1.073 38.152 0.996 93.132
ENSMUSG00000054976 Nyap2 protein_coding 1 71.516 1
78.471 1 66.928 1 89.108
ENSMUSG00000054978 Kbtbd13 protein_coding 1.161 22.025
1.254 73.092 0.686 1.342 0.997 94.016
ENSMUSG00000054986 Sec14l3 protein_coding 1 86.145 1
94.848 1 87.336 1 96.183
ENSMUSG00000054994 AV320801 protein_coding 1 81.52 NA NA
1 86.666 1 95.273
ENSMUSG00000054999 Naaladl1 protein_coding 0.758 83.823
1.016 91.532 1.297 59.274 1.835 69.404
ENSMUSG00000055003 Lrtm2 protein_coding 2.597 10.464 0.602
91.532 0.64 14.079 0.88 92.181
ENSMUSG00000055010 A830031A19Rik protein_coding 1 75.858
NA NA 1 85.757 1 95.35
ENSMUSG00000055013 Agap1 protein_coding 0.981 85.499 0.952
42.923 1.067 13.259 1.101 68.696
ENSMUSG00000055015 Gm9961 protein_coding 1 85.857 1
97.155 1 69.583 1 94.016
ENSMUSG00000055022 Cntn1 protein_coding 2.447 3.498 0.796 79.894
0.513 7.742 0.934 95.35
ENSMUSG00000055024 Ep300 protein_coding 1.042 59.527 1.052
79.261 0.914 17.381 0.981 85.493
ENSMUSG00000055026 Gabrg3 protein_coding 0.792 58.802
1.838 63.504 0.685 19.765 0.867 86.991
ENSMUSG00000055027 Smyd1 protein_coding 2.441 15.662 1.722
82.904 0.237 0.879 0.906 93.964
ENSMUSG00000055030 Sprr2e protein_coding 1 82.982 1
95.35 1 78.954 1 95.74
ENSMUSG00000055033 NA NA NA NA NA NA 1 88.657
1 96.086
ENSMUSG00000055041 Commd5 protein_coding 1.038 85.59 0.941

85.075 1.022 70.9 1.033 75.81
ENSMUSG00000055044 Pdlim1 protein_coding 1.054 75.399
1.064 89.434 0.889 72.269 1.057 57.1
ENSMUSG00000055045 Gm11190 lincRNA 1 72.139 1
93.654 1 79.426 1 94.904
ENSMUSG00000055048 Gm9962 protein_coding 1 86.918 1
97.263 1 84.308 1 96.192
ENSMUSG00000055053 Nfic protein_coding 1.031 47.174 1.094
72.642 0.886 2.294 1.091 71.653
ENSMUSG00000055061 4931431C16Rik processed_transcript 0.762
27.431 1.071 87.609 1.222 54.163 0.817 84.19
ENSMUSG00000055065 Ddx17 protein_coding 0.968 1.343 0.978 91.661
1.054 8.647 0.955 86.699
ENSMUSG00000055067 Smyd3 protein_coding 1.208 1.846 0.847 38.699
0.977 80.163 0.954 89.525
ENSMUSG00000055069 Rab39 protein_coding 1.683 0.461 0.814 90.053
0.732 5.884 1.376 80.573
ENSMUSG00000055072 Gm9964 protein_coding 1 82.543 1
96.138 1 80.381 1 96.184
ENSMUSG00000055078 Gabra5 protein_coding 1 81.647 1
97.263 NA NA 1 95.877
ENSMUSG00000055088 Olfr354 protein_coding 1 79.709 NA
NA 1 86.727 1 96.183
ENSMUSG00000055095 Spink6 protein_coding 1 82.231 1
96.847 NA NA 1 92.018
ENSMUSG00000055099 Gm9966 protein_coding 1 66.652 1
97.263 1 86.144 1 94.016
ENSMUSG00000055108 Phxr2 protein_coding 1 73.585 1
92.931 1 85.21 1 95.405
ENSMUSG00000055109 Gm15155 protein_coding 1 82.231 1
97.155 1 88.41 1 96.248
ENSMUSG00000055116 Arntl protein_coding 0.912 64.284 1.238
96.897 0.886 86.985 0.837 91.105
ENSMUSG00000055124 Olfr623 protein_coding 1 74.299 1
92.137 1 84.896 1 95.832
ENSMUSG00000055128 Cgrrf1 protein_coding 0.908 57.227
0.939 95.329 1.17 47.776 1.134 81.251
ENSMUSG00000055134 9130017K11Rik processed_transcript 0.813
26.77 0.747 88.691 1.64 21.873 1.037 95.74
ENSMUSG00000055137 5033411D12Rik protein_coding 0.828 19.985
0.95 88.897 1.272 18.208 0.939 88.053
ENSMUSG00000055148 Klf2 protein_coding 1.16 29.596 1.01
91.532 0.851 48.133 0.989 95.92
ENSMUSG00000055150 Zfp78 protein_coding 1.122 11.044 1.14
83.104 0.783 3.428 1.074 91.492
ENSMUSG00000055159 4930583K01Rik processed_transcript 1.151
85.092 1.252 95.735 0.695 73.598 0.403 83.952
ENSMUSG00000055172 C1ra protein_coding 1.126 22.871 1.029
97.288 0.863 17.258 1.053 73.823
ENSMUSG00000055184 Fam72a protein_coding 1.09 51.189
0.777 68.115 1.183 64.024 1.041 90.865
ENSMUSG00000055188 2900002K06Rik antisense 0.845 67.334
1.053 96.897 1.124 70.9 1.161 87.134
ENSMUSG00000055193 Klk15 protein_coding 1 72.743 1

93.654	1	87.274	1	91.928
ENSMUSG00000055197	Fev	protein_coding	1	85.283	1
96.013	1	79.897	1	94.654
ENSMUSG00000055198	Gm12830	protein_coding	1	60.435	1
91.361	1	68.82	1	95.676
ENSMUSG00000055200	Sertad3	protein_coding	1.065	77.916
0.977	93.899	0.963	65.688	0.871	85.493
ENSMUSG00000055202	Zfp811	protein_coding	1.367	11.197
1.202	93.654	0.609	17.898	0.699	83.724
ENSMUSG00000055204	Ankrd17	protein_coding	1.057	13.105
0.992	93.654	0.952	19.449	1.016	96.183
ENSMUSG00000055210	Foxd2	protein_coding	1.382	32.058	1.175
88.136	0.615	12.571	0.735	84.29
ENSMUSG00000055216	9430025C20Rik	pseudogene	0.609	20.116
1.071	87.029	1.532	81.857	0.892	95.593
ENSMUSG00000055228	Zfp935	protein_coding	1.178	5.848	0.84
78.471	1.012	75.967	1.026	95.273
ENSMUSG00000055235	Wdr86	protein_coding	1.322	4.825	1.127	83.104
0.67	0.879	1.035	93.457
ENSMUSG00000055239	Kcmf1	protein_coding	0.936	24.817	0.987
97.263	1.082	38.038	0.967	90.674
ENSMUSG00000055240	Zfp101	protein_coding	1.024	82.705
0.947	90.046	1.033	69.825	0.979	96.248
ENSMUSG00000055254	Ntrk2	protein_coding	1.072	63.126	1.04
83.104	0.9	38.799	1.141	91.525
ENSMUSG00000055271	9330161L09Rik	protein_coding	1.332	45.61
1.015	95.708	0.739	69.445	0.666	84.29
ENSMUSG00000055296	Tmem245	protein_coding	0.847	16.895
0.973	91.361	1.208	7.964	0.96	89.827
ENSMUSG00000055301	Adh7	protein_coding	0.979	85.77	0.952	94.96
1.067	72.853	1.16	61.984
ENSMUSG00000055302	Mrfap1	protein_coding	0.98	72.546
1.009	90.256	1.014	63.301	1.087	51.238
ENSMUSG00000055305	Zfp93	protein_coding	1.066	35.787	0.943
64.123	0.997	58.616	0.99	95.273
ENSMUSG00000055313	Pgbd1	protein_coding	0.79	15.912	1.037
82.904	1.221	42.522	1.054	96.248
ENSMUSG00000055319	Sec23ip	protein_coding	1.049	37.414
1.013	96.948	0.938	32.082	1.037	61.984
ENSMUSG00000055320	Tead1	protein_coding	0.537	2.009	1.06	94.848
1.758	7.024	1.225	63.114
ENSMUSG00000055322	Tns1	protein_coding	0.858	9.081	0.988	96.68
1.182	19.452	0.988	95.559
ENSMUSG00000055323	Gm9967	protein_coding	0.958	85.283
0.688	56.781	1.511	15.602	0.963	92.12
ENSMUSG00000055333	Fat2	protein_coding	1.021	81.647	1.033
92.931	0.944	88.601	0.945	86.699
ENSMUSG00000055334	Snupn	protein_coding	0.926	48.646	1.047
97.263	1.028	60.5	1.024	76.831
ENSMUSG00000055340	Olfr115	pseudogene	1	80.608	1
97.263	1	76.768	1	94.001
ENSMUSG00000055341	Zfp457	protein_coding	0.556	40.217
1.132	96.847	1.587	84.724	1.714	74.607
ENSMUSG00000055357	4933400A11Rik	protein_coding	1	75.298

1 95.735 1 73.428 1 93.156
ENSMUSG00000055360 Prl2c5 protein_coding 1 81.196 1
95.211 1 76.754 1 78.948
ENSMUSG00000055368 Slc6a2 protein_coding 1.62 3.893 1.024
96.216 0.602 3.718 0.847 92.748
ENSMUSG00000055370 Gm9968 lincRNA 0.862 78.909 1.008
96.984 1.147 88.657 0.981 92.675
ENSMUSG00000055371 Stam2 protein_coding 1.1 0.999 1.035 93.574
0.878 0.879 0.99 96.183
ENSMUSG00000055373 Fut9 protein_coding 0.88 48.354 0.703
62.025 1.616 14.376 0.875 89.587
ENSMUSG00000055385 Rnf212 protein_coding 1 86.57 NA NA
1 83.397 1 94.654
ENSMUSG00000055396 D830050J10Rik protein_coding 0.92 60.569
1.149 80.775 0.95 59.726 0.88 83.724
ENSMUSG00000055401 Fbxo6 protein_coding 0.945 52.9 0.991 88.704
1.068 23.41 0.998 95.74
ENSMUSG00000055403 4933427D06Rik protein_coding 1 79.853
1 94.102 1 88.203 1 95.877
ENSMUSG00000055407 Map6 protein_coding 0.74 2.009 1.085 96.335
1.249 8.339 0.961 93.316
ENSMUSG00000055408 Gm15053 antisense 1 85.499 1
93.654 1 88.203 1 92.181
ENSMUSG00000055409 Nell1 protein_coding 1 84.324 1
84.594 1 58.616 1 93.612
ENSMUSG00000055413 H2-Q5 pseudogene 0.881 22.731 0.944 91.584
1.205 64.624 0.885 83.724
ENSMUSG00000055415 Atp10b protein_coding 0.788 8.302 1.172
83.104 1.085 39.471 1.004 96.183
ENSMUSG00000055416 Fam136b-ps protein_coding 1 84.738 1
96.984 1 88.161 1 96.042
ENSMUSG00000055421 Pcdh9 protein_coding 1.225 22.205 NA NA
1.015 47.641 0.786 83.724
ENSMUSG00000055424 Gm9970 protein_coding 0.944 60.707
0.899 95.211 1.177 57.907 0.9 84.29
ENSMUSG00000055430 Nap1l5 protein_coding 1.361 0 1.502
82.904 0.488 1.58 0.891 83.724
ENSMUSG00000055435 Maf protein_coding 1.278 0 NA NA
0.804 0.879 1.121 59.464
ENSMUSG00000055436 Srsf11 protein_coding 0.887 2.405 0.965
73.166 1.17 5.864 1.027 67.94
ENSMUSG00000055446 A630091E08Rik protein_coding 1 83.823
NA NA 1 87.274 1 95.72
ENSMUSG00000055447 Cd47 protein_coding 1.079 0.767 1.056 92.793
0.876 5.453 1.048 91.783
ENSMUSG00000055452 Gm7353 protein_coding 1 71.142 NA
NA 1 68.129 1 96.192
ENSMUSG00000055456 Gm9972 protein_coding 1 82.092 1
96.847 1 83.857 1 92.31
ENSMUSG00000055471 Alk protein_coding 0.657 2.174 0.99 89.434
1.541 51.851 0.548 83.724
ENSMUSG00000055480 Zfp458 protein_coding 0.86 54.043
1.245 83.104 0.931 86.112 1.086 85.786
ENSMUSG00000055485 Soga1 protein_coding 0.882 57.544 1.041

96.268 1.089 56.125 1.114 91.525
ENSMUSG00000055489 Ano5 protein_coding 15.304 2.897 0.417
90.68 0.157 0.72 1.337 86.118
ENSMUSG00000055491 Pprc1 protein_coding 0.914 83.523 1.165
89.014 0.936 53.224 1.127 87.886
ENSMUSG00000055493 Epm2a protein_coding 1.135 7.083 1.099 83.104
0.798 2.563 0.98 94.19
ENSMUSG00000055494 Gm14168 lincRNA 0.646 32.749 0.872
92.914 1.767 36.839 1.169 83.581
ENSMUSG00000055497 Gm9974 pseudogene 0.947 80.251 0.847
80.052 1.25 33.042 1.077 92.181
ENSMUSG00000055515 Vmn2r81 protein_coding 1 83.263 NA
NA 1 84.434 1 96.248
ENSMUSG00000055523 Gucy2g protein_coding 1.056 83.968 NA
NA 0.966 83.811 0.777 83.724
ENSMUSG00000055531 Cpsf6 protein_coding 1.015 79.93 0.982 88.691
1.002 69.825 1.022 83.276
ENSMUSG00000055538 Zcchc24 protein_coding 1.112 20.659
1.013 91.065 0.888 16.051 1.035 64.513
ENSMUSG00000055541 Lair1 protein_coding 1.407 29.057 1.626
78.705 0.438 3.863 1.126 59.208
ENSMUSG00000055546 Timd4 protein_coding 1.594 0 1.167 96.847
0.537 3.073 1.459 67.691
ENSMUSG00000055547 Apobec4 protein_coding 1 69.985 1
93.654 1 86.475 1 96.183
ENSMUSG00000055553 Kxd1 protein_coding 0.974 60.569 0.99
93.654 1.035 63.301 0.956 85.493
ENSMUSG00000055560 Zfp459 protein_coding 1.132 34.077
0.767 70.864 1.15 85.935 0.888 85.493
ENSMUSG00000055561 Spink5 protein_coding 0.459 16.325
1.324 83.104 1.645 52.8 0.986 92.748
ENSMUSG00000055567 Unc80 protein_coding 0.879 40.786 1.282
82.904 0.885 82.685 1.337 71.305
ENSMUSG00000055571 Olfr305 protein_coding 1 86.819 1
94.48 1 70.328 1 95.877
ENSMUSG00000055594 5530400C23Rik protein_coding 1 70.213
1 97.129 1 82.161 1 94.904
ENSMUSG00000055602 Tcp10b protein_coding 1.12 81.178 1.11
92.062 0.807 66.201 0.653 83.724
ENSMUSG00000055609 Hba-x protein_coding 1 81.76 1 96.216
1 83.397 1 95.92
ENSMUSG00000055610 Olfr307 protein_coding 1 76.042 1
97.263 1 65.902 1 96.358
ENSMUSG00000055612 Cdca7 protein_coding 0.952 78.299 0.946
79.475 1.111 51.851 1.179 46.846
ENSMUSG00000055621 Olfr636-ps1 pseudogene 1 72.743 1
97.263 1 76.754 1 95.877
ENSMUSG00000055629 B4galnt4 protein_coding 0.629 9.863 1.13
95.735 1.408 23.668 0.903 87.705
ENSMUSG00000055632 Hmcn2 protein_coding 0.741 10.29 1.112 94.367
1.209 47.512 0.929 88.053
ENSMUSG00000055633 Zfp580 protein_coding 1.069 47.847
1.017 94.887 0.917 55.9 0.953 91.269
ENSMUSG00000055639 Dach1 protein_coding 1.305 16.124 0.836

91.532	0.913	25.512	1	92.748
ENSMUSG00000055643	4931431F19Rik	protein_coding	1	82.891
NA	NA	1	83.538	1	92.181
ENSMUSG00000055652	Klhl25	protein_coding	1.075	81.178
1.055	83.104	0.879	19.449	0.914	91.65
ENSMUSG00000055653	Gpc3	protein_coding	1.076	67.991	0.983
94.597	0.942	83.607	1.018	93.734
ENSMUSG00000055657	E030030I06Rik	protein_coding	1	81.925
1	95.708	1	61.841	1	93.156
ENSMUSG00000055660	Mettl4	protein_coding	0.985	82.479
1.115	83.819	0.909	71.004	0.98	96.183
ENSMUSG00000055670	Zzef1	protein_coding	0.986	67.477	1.006
95.735	NA	NA	1.025	94.904
ENSMUSG00000055675	Kbtbd11	protein_coding	1.254	4.925	1.037
82.904	0.766	0.945	0.919	84.29
ENSMUSG00000055676	Gm5069	protein_coding	0.784	36.945
1.356	83.104	0.942	81.857	0.776	85.493
ENSMUSG00000055681	Cope	protein_coding	0.843	0.987	1.083	40.99	1.09
38.152	1.144	46.846
ENSMUSG00000055691	Gja6	protein_coding	1.884	47.466	0.906
90.576	0.587	86.552	1.383	94.016
ENSMUSG00000055692	Tmem191c	protein_coding	0.946	49.944
0.998	90.256	1.056	37.741	0.871	84.19
ENSMUSG00000055700	4933401B06Rik	pseudogene	1	83.968	1
96.138	1	84.308	1	95.777
ENSMUSG00000055704	Gm9978	protein_coding	1.015	81.125
1.361	83.104	0.725	46.956	0.96	92.018
ENSMUSG00000055707	Klhl26	protein_coding	0.974	47.266
0.927	70.693	1.101	16.446	1.007	92.12
ENSMUSG00000055717	Slain1	protein_coding	0.806	8.122	1.237
83.464	1.005	62.648	0.78	83.724
ENSMUSG00000055720	Ubl7	protein_coding	0.933	3.101	1.027	83.819
1.044	32.524	1	94.904
ENSMUSG00000055723	Rras2	protein_coding	1.071	18.556	1.006
91.955	0.924	69.134	1.005	90.674
ENSMUSG00000055725	Paqr3	protein_coding	0.85	7.881	0.948	96.216
1.244	19.452	0.932	91.985
ENSMUSG00000055730	Ces2a	protein_coding	1	78.299	NA	NA
1	87.431	1	94.904
ENSMUSG00000055732	Gm9979	protein_coding	1	81.01	1
93.899	1	87.786	1	96.287
ENSMUSG00000055733	Nap1l3	protein_coding	0.905	45.229
1.029	88.136	1.067	80.634	1.109	82.328
ENSMUSG00000055737	Ghr	protein_coding	1.039	19.132	1.215
88.823	0.788	13.848	0.952	84.29
ENSMUSG00000055745	Ldoc1l	protein_coding	1.103	16.249
0.966	96.216	0.938	24.309	1.052	95.35
ENSMUSG00000055746	Magea2	protein_coding	1	85.59	1
95.708	1	86.144	1	93.316
ENSMUSG00000055760	Gemin6	protein_coding	1.154	56.907	0.94
95.504	0.921	60.96	1.048	82.85
ENSMUSG00000055761	Nkain3	protein_coding	0.438	35.158
4.688	71.617	0.487	88.349	2.013	78.042
ENSMUSG00000055762	Eef1d	protein_coding	0.988	83.726	0.926	0

1.091 5.864 1.012 80.77
ENSMUSG00000055763 Gm6910 pseudogene 0.781 27.968 0.828
90.576 1.542 10.76 1.016 96.287
ENSMUSG00000055771 Gm7936 pseudogene 1 75.399 NA NA
1 84.071 1 94.19
ENSMUSG00000055775 Myh8 protein_coding 2.411 3.932 0.748 94.597
0.551 6.858 1.516 85.577
ENSMUSG00000055780 Usp26 protein_coding 1 84.534 1
96.407 1 84.308 1 95.273
ENSMUSG00000055782 Abcd2 protein_coding 1.02 43.019 1.169
66.695 0.84 5.628 0.968 90.611
ENSMUSG00000055789 E330017A01Rik protein_coding 1 84.661
1 93.908 NA NA 1 96.358
ENSMUSG00000055795 Gm5160 protein_coding 1 67.374 1
97.263 1 71.615 1 85.629
ENSMUSG00000055799 Tcf7l1 protein_coding 1.026 79.577
1.046 82.904 0.933 40.338 0.99 96.287
ENSMUSG00000055805 Fmnl1 protein_coding 1.061 57.38 1.121 87.037
0.839 13.202 0.98 87.992
ENSMUSG00000055809 Dnaaf3 protein_coding 1.299 19.893
0.922 94.597 0.835 21.331 1.013 96.071
ENSMUSG00000055817 Mta3 protein_coding 1.05 70.121 0.991
95.211 0.96 78.293 0.985 93.734
ENSMUSG00000055818 A230083G16Rik protein_coding 0.725 7.916
1.068 96.407 1.289 17.412 0.846 96.086
ENSMUSG00000055820 Olfr967 protein_coding 1 75.567 1
94.887 1 84.504 1 95.273
ENSMUSG00000055827 Gsdmc3 protein_coding 1 57.014 1
96.407 1 80.899 1 94.567
ENSMUSG00000055831 Gm9982 protein_coding 1.049 83.333
1.097 86.34 0.87 54.037 0.945 96.358
ENSMUSG00000055833 1700034H15Rik protein_coding 0.838 11.103
NA NA 1.09 21.705 0.931 94.608
ENSMUSG00000055835 Zfp1 protein_coding 1.06 48.462 0.897
68.818 1.044 64.305 0.988 86.991
ENSMUSG00000055838 Olfr357 protein_coding 1 81.24 1
97.263 1 78.713 1 96.086
ENSMUSG00000055839 Tceb2 protein_coding 0.953 25.551 1.026
83.104 1.023 57.907 1.095 59.441
ENSMUSG00000055850 Rnf181 protein_coding 0.926 6.155 1.066
71.222 1.013 84.308 1.01 93.132
ENSMUSG00000055862 Izumo4 protein_coding 0.943 44.787
0.941 85.95 1.128 27.047 0.917 83.724
ENSMUSG00000055865 Fam19a3 protein_coding 0.677 17.031
1.339 79.367 1.098 79.119 0.92 96.287
ENSMUSG00000055866 Per2 protein_coding 0.74 58.802 1.062
88.092 1.275 79.119 1.212 57.1
ENSMUSG00000055872 NA NA NA NA 1.264 94.848 1.054
82.74 1.367 92.018
ENSMUSG00000055874 Foxi3 protein_coding 1 80.608 1
85.756 1 88.461 1 96.183
ENSMUSG00000055882 Abhd16b protein_coding 0.689 46.693
0.945 95.708 1.54 48.772 0.746 95.273
ENSMUSG00000055884 Fancm protein_coding 1.088 43.019 0.991

91.661	0.927	85.58	0.871	84.29
ENSMUSG00000055895	Plac1l	protein_coding	1	82.982	1
95.431	1	85.352	1	94.215
ENSMUSG00000055897	Ppp4r1l-ps	pseudogene	1.056	56.085	0.922
85.116	1.031	86.258	0.994	86.991
ENSMUSG00000055900	Tmem69	protein_coding	0.952	44.562
1.068	89.494	NA	NA	0.954	84.29
ENSMUSG00000055912	Tmem150a	protein_coding	1.021	80.815
1.161	60.309	0.842	8.907	1.038	86.301
ENSMUSG00000055917	Zfp277	protein_coding	1.143	2.655	0.893
60.678	0.982	83.158	1.067	91.492
ENSMUSG00000055923	Aasdh	protein_coding	0.928	4.115	0.982	90.053
1.1	11.518	0.985	94.654
ENSMUSG00000055926	Gm14137	protein_coding	0.989	72.985
0.912	95.708	1.106	86.258	0.598	83.724
ENSMUSG00000055932	Fto	protein_coding	1.009	64.956	NA	NA
0.973	71.004	1.048	60.31
ENSMUSG00000055933	Gm97	protein_coding	1	71.896	NA	NA
1	86.602	1	91.372
ENSMUSG00000055936	AU015836	protein_coding	1	84.661	1
95.708	1	84.896	1	94.904
ENSMUSG00000055937	Krt28	protein_coding	0.647	53.407	1.258
95.35	1.232	58.915	1.634	87.447
ENSMUSG00000055942	Gm4745	protein_coding	1	85.203	NA
NA	1	84.896	1	92.31
ENSMUSG00000055943	Emc7	protein_coding	0.957	29.725	1.049
79.411	0.992	87.103	0.912	83.724
ENSMUSG00000055944	E130018O15Rik	lincRNA	1	78.909	NA
NA	1	87.431	1	95.92
ENSMUSG00000055945	Prr18	protein_coding	0.968	84.397	1.21
96.335	0.855	87.336	0.799	95.273
ENSMUSG00000055951	Ccl25	protein_coding	1	86.843	1
90.378	1	86.831	1	94.19
ENSMUSG00000055958	Gm9987	pseudogene	1.474	83.169	0.906
96.763	0.746	74.325	0.897	92.018
ENSMUSG00000055960	Skint4	protein_coding	1	86.199	1
96.843	NA	NA	1	93.612
ENSMUSG00000055961	BC051076	protein_coding	1	78.224	1
97.097	1	84.83	1	93.627
ENSMUSG00000055963	Triqk	protein_coding	1.302	6.23	0.962	94.887
0.799	6.858	0.919	85.493
ENSMUSG00000055968	Gm9988	pseudogene	0.869	60.339	1.11
85.756	1.036	86.985	0.894	93.612
ENSMUSG00000055971	Olfr378	protein_coding	1	81.196	1
95.211	1	87.971	1	94.904
ENSMUSG00000055972	2810407A14Rik	protein_coding	0.509	57.014
0.755	84.017	2.599	30.213	2.242	90.463
ENSMUSG00000055976	Cldn23	protein_coding	0.836	49.944
0.997	96.407	1.198	86.144	0.854	83.724
ENSMUSG00000055978	Fut2	protein_coding	1.236	46.875	0.547
67.165	1.474	62.237	1.092	94.904
ENSMUSG00000055980	Irs1	protein_coding	0.966	42.922	1.17
39.442	0.887	38.534	1.175	91.872
ENSMUSG00000055991	Zkscan5	protein_coding	0.944	48.939

0.982 89.509 1.073 38.975 1.003 88.955
ENSMUSG00000055994 Nod2 protein_coding 1.098 42.134 0.99
96.013 0.918 23.48 1 95.273
ENSMUSG00000056004 9330182L06Rik protein_coding 1.047 76.135
0.882 96.216 1.083 79.255 0.747 86.699
ENSMUSG00000056008 Gm11541 protein_coding 1 72.27 1
96.407 1 45.799 1 95.877
ENSMUSG00000056014 A430033K04Rik protein_coding 1.096 25.207
0.909 57.613 1.005 84.896 0.943 92.903
ENSMUSG00000056018 1700008F21Rik protein_coding 1 81.823
1 93.765 1 86.831 1 93.156
ENSMUSG00000056019 Zfp709 protein_coding 1.329 1.828 0.708
35.072 1.062 87.274 1.049 93.264
ENSMUSG00000056025 Clca1 protein_coding 0.935 62.888 0.89
90.712 1.197 27.89 0.887 83.724
ENSMUSG00000056031 9330154J02Rik protein_coding 0.834 72.139
1.269 93.574 0.947 87.898 0.933 94.82
ENSMUSG00000056032 BC018473 antisense 1 80.146 1
93.908 1 87.744 1 96.287
ENSMUSG00000056035 Cyp3a11 protein_coding 1 84.661 NA
NA 1 86.258 1 94.825
ENSMUSG00000056043 Rgs9bp protein_coding 0.942 84.661
1.397 75.612 0.757 24.523 1.004 85.493
ENSMUSG00000056050 Mia3 protein_coding 1.053 46.013 0.984
91.275 0.961 67.029 1.045 48.119
ENSMUSG00000056054 S100a8 protein_coding 0.912 78.639
1.346 96.847 0.817 76.768 0.995 83.581
ENSMUSG00000056055 Sag protein_coding 1.14 59.527 0.81
70.29 1.084 50.091 0.761 83.724
ENSMUSG00000056061 Gm14318 antisense 1 85.391 1
95.504 1 88.657 1 95.273
ENSMUSG00000056069 Fam105a protein_coding 1.451 0 0.961
82.947 0.715 0.72 1.083 93.722
ENSMUSG00000056071 S100a9 protein_coding 1.516 52.799
0.716 88.691 0.918 87.37 1.246 96.248
ENSMUSG00000056073 Grik2 protein_coding 1.444 6.399 1.06 95.708
0.654 13.643 0.954 92.181
ENSMUSG00000056076 Eif3b protein_coding 0.973 30.035 1
90.138 1.028 27.475 0.987 79.545
ENSMUSG00000056089 Gm5468 protein_coding 1 73.585 1
94.597 1 74.082 1 94.825
ENSMUSG00000056091 St3gal5 protein_coding 1.374 3.151 0.84
68.848 0.866 36.499 1.485 42.736
ENSMUSG00000056106 Gm9990 protein_coding 0.483 25.492
1.898 83.464 1.093 84.308 1.374 95.559
ENSMUSG00000056115 Tas2r134 protein_coding 1 85.129 1
95.708 1 87.898 1 95.74
ENSMUSG00000056116 H2-T22 protein_coding 1.083 11.879
0.989 95.504 0.933 41.928 1.062 75.198
ENSMUSG00000056121 Fez2 protein_coding 0.969 45.502 1.027
95.211 1.001 84.896 1.056 72.116
ENSMUSG00000056124 B4galt6 protein_coding 1.155 0.2 1.062
96.68 0.812 4.759 1.072 86.133
ENSMUSG00000056130 Ticam2 protein_coding 1.828 0.767 0.976

78.877	0.559 6.858 0.96	95.273
ENSMUSG00000056131	Pgm3	protein_coding	1.086 63.804	0.928
72.712	0.989 75.527	1.055 73.401
ENSMUSG00000056133	Gm9992	protein_coding	1.803 0.2	0.921
79.807	0.604 3.073 0.522 83.724
ENSMUSG00000056144	Trim34a	protein_coding	1.218 0	1.02
92.355	0.805 16.129	1.02	93.156
ENSMUSG00000056145	AI504432	lincRNA	1.365 27.32 0.857 75.345
0.852 86.552	0.803 85.493
ENSMUSG00000056148	Rdh9	protein_coding	1	84.397	1
94.48 1	74.686	1	96.358
ENSMUSG00000056153	Socs6 protein_coding	1.073 7.553 0.974 87.147
0.957 17.381	0.999 94.608
ENSMUSG00000056155	Nanos3	protein_coding	0.613 44.787
0.737 76.431	2.22	28.979	0.567 84.29
ENSMUSG00000056162	Cndp1 protein_coding	1.329 66	0.812 87.857
0.928 84.308	0.806 96.248
ENSMUSG00000056167	Cnot10	protein_coding	1.001 66	0.988
95.504	1.008 65.902	1.005 96.183
ENSMUSG00000056174	Col8a2	protein_coding	1.315 19.315	1.06
93.574	0.717 15.376	0.866 86.991
ENSMUSG00000056184	Olfr283	protein_coding	1	81.925	NA
NA	1	87.431	1	95.273
ENSMUSG00000056185	Snx32 protein_coding	0.905 44.922	1.055
91.643	1.043 62.307	1.049 94.904
ENSMUSG00000056197	4931417E11Rik	protein_coding	1	86.74 NA
NA	1	86.112	1	96.183
ENSMUSG00000056201	Cfl1	protein_coding	0.983 71.674	1.087
60.309	0.938 7.399 1.061 59.394
ENSMUSG00000056203	Tas2r135	protein_coding	0.809 78.909
0.756 90.256	1.635 58.414	0.652 91.291
ENSMUSG00000056204	Pgpep1	protein_coding	1.071 6.541 NA	NA
0.946 11.116	0.974 96.183
ENSMUSG00000056209	Npm3	protein_coding	1.062 49.517	1
88.705	0.944 57.819	0.981 94.567
ENSMUSG00000056211	R3hdm1	protein_coding	1.016 66.272
1.033 80.277	0.952 9.563 1.08	40.426
ENSMUSG00000056214	Pard6g	protein_coding	1.076 22.205
1.102 87.037	0.844 7.329 0.87	77.012
ENSMUSG00000056215	Lrguk protein_coding	0.56	64.589	0.662
94.887	2.69	73.759	1.047 93.156
ENSMUSG00000056216	Cebpg protein_coding	1.153 6.399 0.945 93.094
0.92	1.705 0.962 85.493
ENSMUSG00000056219	Gm5423	protein_coding	1	82.479	1
97.155	1	76.768	1	96.071
ENSMUSG00000056220	Pla2g4a	protein_coding	1.159 45.307
0.884 81.731	0.971 86.475	0.992 77.331
ENSMUSG00000056223	Spata31	protein_coding	1	70.121	NA
NA	1	87.686	1	94.82
ENSMUSG00000056228	Cars2 protein_coding	0.805 4.385 1.097 82.904
1.126 28.74 1.037 94.608
ENSMUSG00000056234	Ncoa4 protein_coding	0.944 53.679	0.913
84.594	1.159 33.508	0.928 94.904
ENSMUSG00000056257	Gm5447	protein_coding	1.029 86.006	1.47

89.996 0.662 57.446 1.03 93.734
ENSMUSG00000056260 Lrif1 protein_coding 1.179 0.577 0.965 81.731
0.879 6.858 1.013 93.722
ENSMUSG00000056267 Cep70 protein_coding 1.032 68.727 0.931
83.321 1.041 83.066 0.963 88.505
ENSMUSG00000056268 Dennd1b protein_coding 1.017 75.567
0.914 41.141 1.073 20.556 1.014 93.457
ENSMUSG00000056270 Prr9 protein_coding 1 81.647 1
91.83 1 63.801 1 93.627
ENSMUSG00000056271 Lman1l protein_coding 1.29 38.195
0.709 79.261 1.088 85.792 0.845 91.217
ENSMUSG00000056281 Olfr1322 protein_coding 1 75.761 1
96.763 1 75.809 1 94.904
ENSMUSG00000056288 Gm11961 antisense 1 73.292 1
96.138 1 57.907 1 95.559
ENSMUSG00000056290 Ms4a4b protein_coding 0.872 85.655
1.278 80.14 0.901 48.133 1.123 93.584
ENSMUSG00000056293 Gsdmc2 protein_coding 1 56.66 1
94.597 1 70.791 1 91.269
ENSMUSG00000056296 Synpr protein_coding 1.356 45.746 1.506
86.793 0.491 28.669 0.568 84.29
ENSMUSG00000056300 Gm13247 protein_coding 0.982 84.564
0.637 83.876 1.594 55.81 1.003 96.192
ENSMUSG00000056305 Usp39 protein_coding 0.937 44.467 1.087
79.261 0.977 88.274 1.032 72.826
ENSMUSG00000056310 Tyw1 protein_coding 1.027 66.944 0.955
64.123 1.017 75.527 1.032 67.169
ENSMUSG00000056313 1810011O10Rik protein_coding 1.084 51.189
0.757 20.174 1.211 22.93 1.027 96.183
ENSMUSG00000056316 Gm9996 protein_coding 1 69.489 1
87.041 1 81.032 1 90.691
ENSMUSG00000056328 Myh1 protein_coding 0.932 75.858 2.459
83.104 0.436 59.125 1.376 83.067
ENSMUSG00000056329 1810010K12Rik processed_transcript 1
84.049 1 96.216 1 77.294 1 96.086
ENSMUSG00000056332 Gm16294 lincRNA 1 84.738 1
96.847 1 85.935 1 96.183
ENSMUSG00000056342 Usp34 protein_coding 0.96 37.531 0.967
91.661 1.077 25.144 0.975 96.183
ENSMUSG00000056350 Krtap13-1 protein_coding 1 86.74 NA NA
1 86.552 1 94.904
ENSMUSG00000056359 D830013O20Rik protein_coding 1 86.006
1 97.155 1 84.724 1 92.748
ENSMUSG00000056367 Actr3b protein_coding 1.362 12.72 0.92
81.731 0.798 25.625 1.187 42.736
ENSMUSG00000056379 Taar1 protein_coding 1 85.499 1
87.452 1 88.657 1 96.358
ENSMUSG00000056383 AI987944 protein_coding 1.084 32.411
1.072 90.454 0.861 25.345 1.083 42.736
ENSMUSG00000056394 Lig1 protein_coding 1.078 40.635 0.912
72.399 1.015 88.461 1.275 68.354
ENSMUSG00000056399 Prss34 protein_coding 1.609 6.958 0.643
57.613 0.969 69.974 0.909 90.805
ENSMUSG00000056411 Gm12500 protein_coding 0.924 66.129

0.997 95.708 1.084 53.882 0.917 91.291
ENSMUSG00000056413 Adap1 protein_coding 0.724 4.115 1.28 71.617
1.076 84.724 1.016 95.92
ENSMUSG00000056418 BC043934 protein_coding 1.067 82.231
0.809 86.861 1.157 58.051 1.044 95.273
ENSMUSG00000056426 Nat3 protein_coding 1 81.925 1
96.68 1 87.658 1 95.273
ENSMUSG00000056427 Slit3 protein_coding 0.956 75.761 0.933
86.071 1.118 45.208 1.008 92.748
ENSMUSG00000056429 Tgoln1 protein_coding 1.041 52.986
1.029 92.111 0.931 27.321 1.017 96.248
ENSMUSG00000056445 5730446D14Rik antisense 0.836 39.953
0.756 73.166 1.577 16.446 0.956 93.964
ENSMUSG00000056458 Stk30 protein_coding 1.078 25.735 1.046
96.68 0.884 19.449 0.907 85.493
ENSMUSG00000056459 Zbtb25 protein_coding 0.905 49.749
0.997 96.268 1.109 41.6 1.025 91.492
ENSMUSG00000056468 5730596B20Rik protein_coding 0.672 4.895
0.905 91.584 1.646 21.201 0.858 95.72
ENSMUSG00000056476 Med12l protein_coding 1.159 39.075
1.183 71.617 0.73 1.705 0.874 83.952
ENSMUSG00000056481 Cd248 protein_coding 1.323 2.085 0.866 96.68
0.875 6.935 1.388 31.133
ENSMUSG00000056486 Chn1 protein_coding 0.749 5.544 NA NA
1.378 16.808 1.108 83.581
ENSMUSG00000056492 Gpr116 protein_coding 0.994 67.163
0.802 62.71 1.252 37.369 0.994 88.245
ENSMUSG00000056493 Foxk1 protein_coding 0.976 61.633 1.026
87.609 0.997 86.258 0.96 85.493
ENSMUSG00000056498 Tmem154 protein_coding 1.234 12.396
0.838 25.341 0.968 88.657 0.949 94.19
ENSMUSG00000056501 Cebpb protein_coding 1.053 67.374 0.94
95.504 1.007 67.962 1.015 91.985
ENSMUSG00000056508 1700001K19Rik protein_coding 1.233 59.211
1.298 96.216 0.625 69.583 0.537 83.724
ENSMUSG00000056509 Gm9999 protein_coding 1 74.147 1
91.361 1 86.602 1 92.181
ENSMUSG00000056515 Rab31 protein_coding 1.45 0 0.937 84.017
0.734 0.72 1.085 83.067
ENSMUSG00000056529 Ptafr protein_coding 1.742 0.2 0.836 51.322
0.685 22.864 0.818 96.183
ENSMUSG00000056531 Ccdc18 protein_coding 1.147 58.38 0.781
88.691 1.114 79.554 0.972 96.183
ENSMUSG00000056536 Pign protein_coding 1.069 15.662 0.917
70.29 1.018 85.935 1.04 88.755
ENSMUSG00000056537 Rlim protein_coding 1.06 26.136 0.927
74.633 1.022 86.112 1.034 91.525
ENSMUSG00000056544 Defb21 protein_coding 1 86.333 1
82.848 1 70.534 1 96.287
ENSMUSG00000056553 Ptprn2 protein_coding 0.719 76.686
1.329 96.268 1.048 79.554 0.669 93.316
ENSMUSG00000056564 Olfr324 protein_coding 1 81.01 1
95.211 NA NA NA NA
ENSMUSG00000056569 Mpz protein_coding 0.952 38.286 0.996

97.263	1.056	47.935	0.99	96.042
ENSMUSG00000056579	Tug1	processed_transcript	1.088	1.949	1.022
96.984	0.901	5.087	0.946	83.724
ENSMUSG00000056583	9930038K12Rik	pseudogene	1	85.092	1
95.504	1	84.504	1	96.086
ENSMUSG00000056586	Zar1l	protein_coding	1	81.823	1
95.301	1	75.009	1	96.358
ENSMUSG00000056590	H2afy3	pseudogene	1	86.145	1
82.848	1	60.789	1	90.674
ENSMUSG00000056592	Zfp658	protein_coding	1.14	20.942
0.958	93.469	0.917	32.297	0.99	96.183
ENSMUSG00000056596	Trnp1	protein_coding	1.035	80.408	1.033
95.431	0.933	59.726	0.797	83.724
ENSMUSG00000056598	Lrrc48	protein_coding	1.051	74.958
0.935	92.137	1.018	84.724	0.874	92.181
ENSMUSG00000056600	Olfr90	protein_coding	1	80.608	NA
NA	1	88.693	1	96.358
ENSMUSG00000056602	Fry	protein_coding	0.964	37.248	1.1
60.309	0.94	44.211	0.962	92.12
ENSMUSG00000056605	Krt72	protein_coding	1	78.909	1
96.407	1	84.896	1	96.183
ENSMUSG00000056606	NA	NA	NA	NA	0.762	79.475	1.255
49.384	0.86	93.685
ENSMUSG00000056608	Chd9	protein_coding	0.959	40.568	0.97
88.691	1.075	31.975	1.04	76.831
ENSMUSG00000056612	Ppp1r14b	protein_coding	0.917	33.415
0.991	95.504	1.099	37.369	1.112	81.56
ENSMUSG00000056617	4931429L15Rik	protein_coding	1	84.865
1	91.584	1	73.027	1	96.071
ENSMUSG00000056629	Fkbp2	protein_coding	0.876	19.595	1.03
95.431	1.11	38.152	1.132	60.968
ENSMUSG00000056643	Chst13	protein_coding	1.207	75.399
0.775	90.287	1.073	85.352	3.003	46.412
ENSMUSG00000056648	Hoxb8	protein_coding	0.944	49.621	1.026
92.404	1.031	35.894	0.887	86.699
ENSMUSG00000056656	Apol8	protein_coding	1.425	25.421	NA	NA
0.713	18.336	1.06	93.261
ENSMUSG00000056665	Them6	protein_coding	1.137	35.499	1.196
66.695	0.734	1.48	0.891	84.29
ENSMUSG00000056666	Retsat	protein_coding	1.183	7.65	0.905
90.378	0.93	17.258	0.962	85.493
ENSMUSG00000056671	Prelid2	protein_coding	1.357	9.259	0.805
82.947	0.916	40.794	1.103	90.177
ENSMUSG00000056673	Kdm5d	protein_coding	1	80.51	NA	NA	1
77.532	1	94.82
ENSMUSG00000056679	Gpr173	protein_coding	1.07	86.199
0.935	93.654	0.995	82.161	1.02	94.215
ENSMUSG00000056682	NA	NA	NA	NA	2.046	96.948	0.867
81.62	1.351	95.72
ENSMUSG00000056687	Gm11696	protein_coding	0.831	30.035
1.316	76.634	0.916	61.288	0.923	87.218
ENSMUSG00000056692	D17Wsu92e	protein_coding	1.002	78.224
1.022	96.847	0.976	86.475	1.03	51.901
ENSMUSG00000056696	Olfr1350	protein_coding	1	74.745	1

95.504	1	87.898	1	95.74
ENSMUSG00000056698	Elmod3	protein_coding	0.982	39.953	0.96
95.211	1.063	75.155	0.988	94.654
ENSMUSG00000056706	Krtap7-1	protein_coding	1	84.168	1
91.584	1	78.045	1	95.92
ENSMUSG00000056708	Ier5	protein_coding	0.88	30.637	1.107
90.952	1.024	58.616	0.982	96.287
ENSMUSG00000056718	Gm13199	protein_coding	0.716	4.895	0.989
92.355	1.414	7.635	1	93.316
ENSMUSG00000056724	Nbeal2	protein_coding	0.966	19.224
0.945	88.704	1.097	10.76	0.937	86.445
ENSMUSG00000056728	Ctsll3	protein_coding	1	80.688	1
95.941	1	77.532	1	96.071
ENSMUSG00000056729	Gm10006	protein_coding	1	74.299	1
96.138	1	84.83	1	96.248
ENSMUSG00000056735	A930024E05Rik	processed_transcript	0.87	74.8
0.937	94.96	1.228	48.772	0.915	86.991
ENSMUSG00000056737	Capg	protein_coding	1.405	0	0.812	47.727
0.874	20.511	1.047	64.513
ENSMUSG00000056738	A730036I17Rik	lincRNA	1	86.006	1
96.013	NA	NA	1	93.413
ENSMUSG00000056749	Nfil3	protein_coding	0.796	51.089	1.268
83.104	0.989	84.724	1.39	67.691
ENSMUSG00000056752	Dnah9	protein_coding	2.515	34.439	0.431
84.594	0.921	63.919	1.094	93.627
ENSMUSG00000056753	C330011M18Rik	protein_coding	0.881	84.661
1.072	97.129	1.056	80.015	0.97	89.892
ENSMUSG00000056755	Grm7	protein_coding	1	83.418	NA	NA
1	74.325	1	93.685
ENSMUSG00000056758	Hmga2	protein_coding	1.203	64.956	0.735
95.211	1.132	84.896	0.911	89.892
ENSMUSG00000056763	Cspp1	protein_coding	0.943	17.031	0.947
70.29	1.119	6.916	0.997	94.904
ENSMUSG00000056770	Setd3	protein_coding	1.033	48.462	0.947
71.98	1.021	83.719	1.004	96.183
ENSMUSG00000056771	Gm10010	protein_coding	1.096	59.806
0.872	93.908	1.047	81.169	0.996	93.316
ENSMUSG00000056772	Rps6-ps2	pseudogene	1	84.865	1
97.263	NA	NA	1	95.74
ENSMUSG00000056777	Rpl21-ps13	pseudogene	1	83.09	1	93.765
1	88.657	1	95.676
ENSMUSG00000056782	Olfr667	protein_coding	1	74.516	1
95.809	1	84.229	1	94.468
ENSMUSG00000056797	Gm4889	protein_coding	1	71.217	1
94.597	1	84.552	1	96.192
ENSMUSG00000056812	St8sia3	protein_coding	1	84.977	NA
NA	1	88.203	1	93.722
ENSMUSG00000056815	Gm6812	protein_coding	1	80.51	1
96.847	1	83.275	1	95.877
ENSMUSG00000056820	Tsnax	protein_coding	1.076	7.799	0.93	75.345
0.997	67.962	1.022	68.696
ENSMUSG00000056822	Olfr166	protein_coding	1	71.142	1
95.35	1	86.727	1	83.276
ENSMUSG00000056829	Foxb2	protein_coding	1	81.24	NA	NA	1

88.076	1	94.99
ENSMUSG00000056832	Ttc26	protein_coding	0.95	86.74	1.13	82.904
0.932	23.1	1.009	90.177
ENSMUSG00000056836	Gm6851	pseudogene	1	83.523	1
95.708	1	87.744	1	92.018
ENSMUSG00000056849	Gm16415	pseudogene	1	82.705	1
97.129	1	83.158	1	95.676
ENSMUSG00000056851	Pcbp2	protein_coding	1.105	6.438	0.985	91.361
0.914	11.997	0.988	95.405
ENSMUSG00000056853	Olfr765	protein_coding	1	83.263	1
96.216	1	80.5	1	90.854
ENSMUSG00000056856	Jakmip3	protein_coding	1.319	30.788
0.824	84.594	0.922	69.583	0.925	89.892
ENSMUSG00000056858	Olfr1502	protein_coding	1	81.657	1
95.211	1	83.719	1	93.132
ENSMUSG00000056863	Olfr702	protein_coding	1	82.982	1
95.708	1	86.258	1	96.042
ENSMUSG00000056870	Gulp1	protein_coding	1.165	10.19	1.135	86.387
0.757	1.061	0.971	85.493
ENSMUSG00000056872	Gm10011	pseudogene	1.402	60.168	0.576
79.807	1.24	77.532	0.7	84.29
ENSMUSG00000056877	Rps15a-ps1	pseudogene	1	73.487	1
95.504	1	87.686	1	95.877
ENSMUSG00000056882	Vmn1r215	protein_coding	1	73.401	1
96.68	1	85.792	1	95.832
ENSMUSG00000056883	Olfr533	protein_coding	1	55.124	NA
NA	1	77.704	1	96.183
ENSMUSG00000056885	Gm4559	protein_coding	1	85.129	1
95.708	1	80.015	1	95.832
ENSMUSG00000056888	Glipr1	protein_coding	1.187	12.985	1.16
96.68	0.727	15.024	0.887	90.289
ENSMUSG00000056895	Hist3h2ba	protein_coding	0.483	1.727	NA	NA
1.886	6.506	1.124	88.512
ENSMUSG00000056899	Immp2l	protein_coding	0.751	3.848	1.006
96.847	1.322	15.241	0.889	93.045
ENSMUSG00000056900	Usp13	protein_coding	1.831	16.249	1.413
82.904	0.385	1.408	0.902	95.273
ENSMUSG00000056901	Tas2r102	protein_coding	1	85.655	NA
NA	1	88.203	1	95.74
ENSMUSG00000056904	Gm5620	pseudogene	0.509	1.15	1.803	71.617
1.086	45.667	1.54	37.634
ENSMUSG00000056910	Vmn2r107	protein_coding	1	86.843	1
96.216	1	88.693	1	95.92
ENSMUSG00000056917	Sipa1	protein_coding	0.871	32.557	1.172
72.642	0.979	85.134	1.019	92.381
ENSMUSG00000056919	4922501C03Rik	protein_coding	0.898	28.584
1.09	96.335	1.023	55.81	1.054	76.831
ENSMUSG00000056921	Olfr394	protein_coding	1	82.732	1
94.102	1	70.328	1	95.593
ENSMUSG00000056926	Tas2r113	protein_coding	1	75.671	1
96.407	1	88.693	1	95.405
ENSMUSG00000056938	Acbd4	protein_coding	0.76	1.488	1.104	72.642
1.194	28.603	0.953	83.724
ENSMUSG00000056941	Commd7	protein_coding	0.998	86.006	NA

NA	0.999	69.445	0.95	83.724
ENSMUSG00000056943	Gm10015	pseudogene	1	70.862	NA	NA
1	88.693	1	96.183
ENSMUSG00000056946	Olfr512	protein_coding	1	67.374	NA
NA	1	87.274	1	95.832
ENSMUSG00000056947	Mab21l1	protein_coding	1.127	19.315
1.312	82.219	0.674	2.361	1.257	55.841
ENSMUSG00000056952	Tatdn2	protein_coding	1.02	59.211
1.039	90.68	0.943	20.751	1.068	93.156
ENSMUSG00000056959	Olfr315	protein_coding	1	84.534	1
93.765	1	87.744	1	92.31
ENSMUSG00000056961	Olfr919	protein_coding	1	83.523	NA
NA	1	87.431	1	93.964
ENSMUSG00000056962	Jmjd6	protein_coding	0.986	81.657	1.087
79.367	0.934	6.858	1.02	96.358
ENSMUSG00000056966	Gjc3	protein_coding	0.844	25.111	1.142
64.304	1.04	84.669	1.134	95.35
ENSMUSG00000056972	Magel2	protein_coding	1	85.832	1
94.767	1	78.045	1	93.734
ENSMUSG00000056973	Ces1d	protein_coding	1.085	76.827	0.866
91.661	1.061	72.853	0.89	90.289
ENSMUSG00000056987	Fam71d	protein_coding	2.094	35.499
1.758	95.211	0.271	15.024	0.249	83.724
ENSMUSG00000056995	Olfr1178	protein_coding	1	80.146	NA
NA	1	86.985	1	95.74
ENSMUSG00000056999	Ide	protein_coding	1.036	86.006	1.285
92.793	0.748	88.203	1.059	78.546
ENSMUSG00000057000	Nxf3	protein_coding	1	50.627	1
93.765	1	78.713	1	88.053
ENSMUSG00000057003	Myh4	protein_coding	7.677	5.481	0.929	83.819
0.141	0.72	0.985	95.72
ENSMUSG00000057021	Vmn2r-ps159	protein_coding	1	79.709	1
97.155	1	78.864	1	92.018
ENSMUSG00000057036	Gm7536	protein_coding	12.482	0
1.765	85.501	0.045	0.72	0.737	91.217
ENSMUSG00000057037	Cfhr1	protein_coding	1	84.324	1
94.96	1	85.921	1	96.287
ENSMUSG00000057047	1700010B08Rik	protein_coding	1	84.294
1	93.654	1	86.475	1	96.086
ENSMUSG00000057050	Olfr397	protein_coding	1	74.689	1
95.35	NA	NA	1	95.676
ENSMUSG00000057054	Inca1	protein_coding	1.024	81.823	0.966
95.431	NA	NA	0.82	83.724
ENSMUSG00000057058	Skap1	protein_coding	0.994	82.092	1.503
82.809	0.67	29.451	0.658	83.724
ENSMUSG00000057060	Slc35f3	protein_coding	0.801	38.195
0.741	86.071	1.685	58.915	1.107	93.734
ENSMUSG00000057067	Olfr297	protein_coding	1	83.169	1
95.211	1	88.349	1	95.74
ENSMUSG00000057068	Fam47e	protein_coding	0.642	56.477	NA
NA	1.314	62.307	1.492	83.581
ENSMUSG00000057069	Ero1lb	protein_coding	1.059	45.307
1.018	92.931	0.925	21.117	0.932	83.724
ENSMUSG00000057072	1700022P22Rik	protein_coding	1.249	75.196

0.944 94.102 0.847 87.336 0.731 86.991
ENSMUSG00000057092 Fxyd3 protein_coding 0.889 29.972 1.012 95.329 1.114 23.41 0.864 84.29
ENSMUSG00000057093 C030039L03Rik protein_coding 1.024 70.741 0.947 95.504 1.028 87.37 1.123 85.786
ENSMUSG00000057098 Ebf1 protein_coding 1.134 5.559 0.922 75.345 0.957 38.799 1.047 95.273
ENSMUSG00000057101 Zfp180 protein_coding 1.009 75.115 1.037 83.104 0.959 55.9 1.034 83.067
ENSMUSG00000057103 Cml1 protein_coding 0.855 23.641 0.936 90.744 1.251 28.997 0.935 84.29
ENSMUSG00000057110 Cep110 protein_coding 0.945 26.77 0.987 97.263 1.072 27.533 0.963 86.991
ENSMUSG00000057113 Npm1 protein_coding 1.196 6.853 0.818 35.072 1.025 87.274 1.069 49.514
ENSMUSG00000057116 AF366264 protein_coding 1 85.092 1 90.389 1 57.446 1 92.12
ENSMUSG00000057123 Gja5 protein_coding 0.922 79.709 1.042 93.146 1.037 87.971 1.065 85.629
ENSMUSG00000057130 Txnl4a protein_coding 0.917 8.405 1.11 87.609 0.978 76.498 0.921 84.29
ENSMUSG00000057132 Rpgrip1 protein_coding 0.912 45.728 0.958 87.313 1.148 25.455 0.837 83.724
ENSMUSG00000057133 Chd6 protein_coding 0.94 13.883 0.925 75.4 1.154 11.932 0.911 84.29
ENSMUSG00000057134 Ado protein_coding 1.078 23.82 1.027 83.104 0.904 5.701 0.978 91.985
ENSMUSG00000057135 Scimp protein_coding 1.063 81.125 1.361 75.62 0.689 12.288 1.138 59.464
ENSMUSG00000057137 Tmem140 protein_coding 1.231 21.146 0.787 20.174 1.03 72.853 0.915 90.157
ENSMUSG00000057143 Trim12c protein_coding 1.225 0.355 0.881 56.027 0.927 42.997 1.033 79.442
ENSMUSG00000057147 Dph6 protein_coding 1.015 81.657 1.047 96.984 0.938 87.744 1.061 75.81
ENSMUSG00000057149 Olfr1301 protein_coding 1 78.428 1 95.301 1 85.757 1 95.273
ENSMUSG00000057156 Homez protein_coding 0.99 65.622 0.972 92.137 1.036 66.928 0.908 88.505
ENSMUSG00000057161 Vmn1r80 protein_coding 1 70.741 NA NA 1 85.921 1 93.612
ENSMUSG00000057163 Prss2 protein_coding 1 74.689 1 90.116 1 74.853 1 90.611
ENSMUSG00000057168 Olfr10 protein_coding 1 67.991 NA NA 1 69.134 1 96.287
ENSMUSG00000057170 Prl3d1 protein_coding 1 84.324 1 95.504 1 85.464 1 96.183
ENSMUSG00000057173 Rfx8 protein_coding 1.531 57.133 0.471 79.475 1.381 80.899 0.944 91.65
ENSMUSG00000057174 Krtap19-9b protein_coding 1 81.125 1 94.89 1 86.258 1 96.086
ENSMUSG00000057176 Gm166 protein_coding 0.844 24.344 0.983 93.908 1.208 20.315 0.985 92.675
ENSMUSG00000057177 Gsk3a protein_coding 0.91 5.491 1.063 87.037

1.036 37.764 0.963 88.136
ENSMUSG00000057179 Olfr747 protein_coding 1 86.351 NA
NA 1 82.74 1 96.358
ENSMUSG00000057181 5730455P16Rik protein_coding 1.026 72
1.033 96.843 0.944 61.743 1.008 93.156
ENSMUSG00000057182 Scn3a protein_coding 0.97 66.58 1.093 95.708
0.939 71.388 0.988 93.964
ENSMUSG00000057191 AB124611 protein_coding 1.548 2.567 0.828
53.79 0.776 46.956 0.932 93.316
ENSMUSG00000057193 Slc44a2 protein_coding 1.147 0.355 1.008
97.155 0.865 0.879 0.895 83.724
ENSMUSG00000057203 Vmn1r234 protein_coding 1 84.452 1
95.708 NA NA 1 91.985
ENSMUSG00000057207 Olfr1028 protein_coding 1 85.129 1
95.504 1 83.607 1 96.183
ENSMUSG00000057215 Gm15514 lincRNA 1 86.843 1
96.652 1 83.719 1 94.016
ENSMUSG00000057216 Gm10807 pseudogene 1 84.865 1
95.088 1 87.971 1 96.183
ENSMUSG00000057219 Armc7 protein_coding 1.039 84.397 1.039
83.464 0.927 42.895 0.924 84.29
ENSMUSG00000057223 Gm6578 protein_coding 1 69.777 1
91.83 1 87.686 1 95.593
ENSMUSG00000057228 Aadat protein_coding 1 81.647 1
96.138 1 86.666 1 90.611
ENSMUSG00000057229 Atp5sl protein_coding 0.994 48.646
0.889 71.41 1.132 18.797 0.967 93.132
ENSMUSG00000057230 Aak1 protein_coding 0.914 23.923 1.022
87.609 1.072 36.011 1.082 95.273
ENSMUSG00000057234 Mettl15 protein_coding 1.061 65.537
0.967 93.094 0.972 68.266 0.855 83.724
ENSMUSG00000057236 Rbbp4 protein_coding 1.062 47.174 0.968
66.651 0.975 86.602 0.992 96.183
ENSMUSG00000057240 Ms4a13 protein_coding 1 86.199 1
95.735 1 87.686 1 95.832
ENSMUSG00000057244 Gm6139 protein_coding 1 68.16 1
96.652 1 84.071 1 91.078
ENSMUSG00000057246 BC051142 protein_coding 1.395 42.255
0.982 88.533 0.729 26.809 0.685 83.724
ENSMUSG00000057262 Gm10020 protein_coding 1.051 84.819 0.86
85.418 1.108 70.623 0.9 89.892
ENSMUSG00000057265 Ccdc176 protein_coding 0.833 14.403
0.957 96.68 1.249 30.561 0.867 83.724
ENSMUSG00000057270 Olfr1501 protein_coding 1 84.452 1
97.097 1 85.58 1 95.273
ENSMUSG00000057278 Snrpg protein_coding 1.109 25.944 0.95
72.399 0.952 84.434 1.029 86.445
ENSMUSG00000057280 Musk protein_coding 0.978 74.299 1.095
91.643 NA NA 0.927 94.016
ENSMUSG00000057286 St6galnac2 protein_coding 0.864 3.641 1.01
93.046 1.149 8.201 1.052 93.261
ENSMUSG00000057290 Gm15732 pseudogene 1 82.982 1
96.216 1 88.729 1 86.991
ENSMUSG00000057299 Vmn1r192 protein_coding 1 86.333 1

97.155	1	88.274	1	94.654
ENSMUSG00000057315	Arhgap24	protein_coding	0.692	0.987	1.155
72.642	1.247	22.366	1.102	72.369
ENSMUSG00000057321	Usp17ld	protein_coding	1	73.401	1
95.301	NA	NA	1	93.964
ENSMUSG00000057322	Rpl38	protein_coding	1.111	31.477	0.87
54.427	NA	NA	1.033	85.629
ENSMUSG00000057329	Bcl2	protein_coding	0.976	50.768	1.057
83.104	0.97	85.792	0.901	86.699
ENSMUSG00000057335	Cep170	protein_coding	1.028	48.857
1.085	88.923	0.899	28.901	1.034	83.067
ENSMUSG00000057337	Chst3	protein_coding	1.169	44.694	0.917
95.211	0.935	63.075	0.987	95.273
ENSMUSG00000057342	Sphk2	protein_coding	0.902	28.514	1.105
89.996	1.006	66.928	0.979	95.92
ENSMUSG00000057346	Apol9a	protein_coding	1.474	2.512	1.11
95.088	0.611	21.852	1.374	55.841
ENSMUSG00000057349	Olfr948	protein_coding	1	86.843	1
96.843	1	80.5	1	93.413
ENSMUSG00000057351	Olfr347	protein_coding	1	84.263	1
96.013	1	82.903	1	94.904
ENSMUSG00000057354	4930430O22Rik	protein_coding	1	79.068
1	93.574	1	85.792	1	89.052
ENSMUSG00000057363	Uxs1	protein_coding	1.085	5.657	NA	NA
0.933	7.329	1.183	43.377
ENSMUSG00000057367	Birc2	protein_coding	1.049	61.725	0.937
64.123	1.02	44.515	0.991	91.928
ENSMUSG00000057370	Rpl21-ps12	pseudogene	1	84.324	1
97.049	NA	NA	1	95.877
ENSMUSG00000057375	Yipf1	protein_coding	0.94	11.103	NA	NA
1.046	13.082	0.987	95.676
ENSMUSG00000057378	Ryr3	protein_coding	0.968	85.637	1.086
92.561	0.95	80.749	1.357	88.797
ENSMUSG00000057381	Tas2r123	protein_coding	1	86.172	1
97.155	1	80.899	1	95.273
ENSMUSG00000057387	4922502B01Rik	protein_coding	1	75.196
1	95.708	1	84.724	1	95.35
ENSMUSG00000057388	Mrpl18	protein_coding	0.958	33.745
1.071	82.809	0.976	71.734	0.995	74.896
ENSMUSG00000057396	Zfp759	protein_coding	1.122	19.315
0.902	60.678	0.992	82.903	0.937	96.358
ENSMUSG00000057400	Ces1c	protein_coding	1	84.452	1
88.345	1	57.286	1	96.248
ENSMUSG00000057402	1700042B14Rik	protein_coding	1	84.534
1	94.96	1	72.695	1	93.045
ENSMUSG00000057406	Whsc1	protein_coding	1.002	78.538	0.943
87.857	1.06	63.665	1.082	60.466
ENSMUSG00000057409	Zfp53	protein_coding	1.136	20.444	0.788
24.911	1.113	34.07	1.071	96.139
ENSMUSG00000057411	Fam173a	protein_coding	0.87	17.401
1.085	83.464	1.061	29.568	1.02	95.593
ENSMUSG00000057417	Dcpp3	protein_coding	0.139	3.009	0.949	92.793
7.606	11.518	1.897	88.111
ENSMUSG00000057421	Las1l	protein_coding	1	59.806	0.924

35.072 1.084 22.444 1.007 93.722
ENSMUSG00000057424 Olfr934 protein_coding 1 86.172 1
95.708 1 86.346 1 96.358
ENSMUSG00000057425 Ugt2b37 protein_coding 1 80.03 1
96.216 1 78.155 1 96.358
ENSMUSG00000057439 Kir3dl2 protein_coding 1 65.622 1
95.708 NA NA 1 92.181
ENSMUSG00000057440 Mpp7 protein_coding 1.054 84.168 0.929
91.361 1.023 69.825 0.942 95.877
ENSMUSG00000057443 Olfr138 protein_coding 1 85.59 1
96.013 1 86.258 1 93.264
ENSMUSG00000057444 Olfr910 protein_coding 1 83.169 1
95.504 1 85.792 1 94.654
ENSMUSG00000057446 Cts8 protein_coding 1 81.76 1 95.809
1 79.554 1 95.877
ENSMUSG00000057447 Olfr1205 protein_coding 1 83.823 1
93.469 1 86.831 1 95.273
ENSMUSG00000057454 Lypd3 protein_coding 0.861 54.983 0.828
78.51 1.405 34.285 0.961 88.245
ENSMUSG00000057455 Rit2 protein_coding 0.92 69.985 1.336
92.253 NA NA 1.018 96.248
ENSMUSG00000057461 Olfr591 protein_coding 1 76.042 1
95.211 1 80.089 1 94.174
ENSMUSG00000057464 Olfr1415 protein_coding 1 84.977 1
95.708 1 87.898 1 94.468
ENSMUSG00000057469 E2f6 protein_coding 0.997 76.546 1.12
71.617 0.898 4.963 1.132 49.239
ENSMUSG00000057497 Fam136a protein_coding 1.051 41.951
1.046 94.96 0.906 73.598 1.014 92.018
ENSMUSG00000057499 Gm6177 pseudogene 1 71.36 1 97.263
1 84.896 1 86.445
ENSMUSG00000057503 Olfr1459 protein_coding 1 81.823 1
96.68 1 87.744 1 93.964
ENSMUSG00000057506 Bloc1s2 protein_coding 1.074 52.429
0.934 88.705 NA NA 1.001 95.273
ENSMUSG00000057513 Vmn1r177 protein_coding 1 85.832 1
96.948 1 86.475 1 95.72
ENSMUSG00000057522 Spop protein_coding 0.975 44.467 0.935
73.461 1.092 25.144 1.011 94.99
ENSMUSG00000057530 Ece1 protein_coding 1.117 3.176 1.01 96.68
0.887 5.628 1.016 89.152
ENSMUSG00000057531 Dtnbp1 protein_coding 1.043 35.499
1.064 96.68 0.903 25.052 0.962 86.445
ENSMUSG00000057534 Gm15698 protein_coding 1 64.893 1
97.263 1 86.831 1 94.016
ENSMUSG00000057540 Olfr310 protein_coding 1 86.464 1
94.89 1 86.144 1 92.018
ENSMUSG00000057541 Pus7 protein_coding 0.893 40.568 1.007
96.335 1.108 48.772 1.096 72.992
ENSMUSG00000057551 Zfp317 protein_coding 0.98 83.263
0.986 96.013 1.035 78.369 1.031 78.948
ENSMUSG00000057554 Lgals8 protein_coding 1.078 6.853 0.974
88.691 0.95 19.765 1.011 83.067
ENSMUSG00000057561 Eif1a protein_coding 0.9 47.174 0.931

86.538	1.19	61.841	1.258	57.075
ENSMUSG00000057564	NA	NA	NA	NA	1	93.817	1
77.037	1	96.183
ENSMUSG00000057572	Zbtb8os	protein_coding	1.033	80.146
0.957	81.731	1.009	50.851	1.015	91.492
ENSMUSG00000057577	Gm10025	pseudogene	1	66.58	1	92.404
1	87.658	1	96.183
ENSMUSG00000057592	Vmn1r48	protein_coding	1	83.726	1
96.268	1	84.724	1	96.287
ENSMUSG00000057594	Arl16	protein_coding	1.217	0.461	0.95	81.731
0.864	11.504	0.963	86.699
ENSMUSG00000057596	Trim30d	protein_coding	1.342	0.461	0.931
85.418	0.8	30.468	1.2	46.846
ENSMUSG00000057604	Lmcd1	protein_coding	1.056	84.865	1.082
92.561	0.873	75.637	0.869	96.192
ENSMUSG00000057606	Colq	protein_coding	0.784	68.353	1.871
83.104	0.68	75.353	2.395	51.908
ENSMUSG00000057609	Lce1a1	protein_coding	1	68.727	1
96.335	1	77.037	1	88.136
ENSMUSG00000057610	Ighv1-73	protein_coding	1	83.726	NA
NA	1	84.896	1	94.19
ENSMUSG00000057612	Vmn1r37	protein_coding	1	85.355	1
96.216	1	88.076	1	96.042
ENSMUSG00000057614	Gnai1	protein_coding	0.997	81.196	1.028
96.216	0.979	76.64	0.843	83.724
ENSMUSG00000057626	NA	NA	NA	NA	1	96.847	NA	NA
1	95.273
ENSMUSG00000057637	Prdm2	protein_coding	0.956	33.002	1.014
91.532	1.03	28.979	1.02	95.35
ENSMUSG00000057649	Brd9	protein_coding	0.961	14.086	1.009
92.561	1.029	50.851	0.988	96.183
ENSMUSG00000057650	Krtap19-2	protein_coding	1	86.464	1
97.263	1	86.985	1	96.192
ENSMUSG00000057654	Olfr328	protein_coding	1	85.092	1
96.268	1	74.853	1	96.248
ENSMUSG00000057666	Gapdh	protein_coding	0.573	1.701	1.287	83.104
1.353	10.819	1.451	48.119
ENSMUSG00000057667	Bloc1s3	protein_coding	0.988	73.292	0.98
85.793	1.034	43.586	0.902	83.724
ENSMUSG00000057672	Pkn1	protein_coding	0.973	78.909	0.98
94.597	1.044	61.418	1.018	95.559
ENSMUSG00000057674	Gm11938	protein_coding	1	80.03	NA	NA
1	86.985	1	96.358
ENSMUSG00000057685	Gm8526	pseudogene	1	76.968	1
94.597	1	85.352	1	95.74
ENSMUSG00000057691	Zfp746	protein_coding	0.97	33.002
1.004	93.863	1.024	62.995	0.964	91.269
ENSMUSG00000057696	Rpl30-ps1	pseudogene	1	69.985	1
90.952	NA	NA	1	88.197
ENSMUSG00000057697	Vmn1r53	protein_coding	1	53.138	1
83.104	1	87.744	1	79.713
ENSMUSG00000057699	Tas2r131	protein_coding	1	81.52	1
96.138	1	88.729	1	94.82
ENSMUSG00000057706	Mex3b	protein_coding	1.017	78.428	1.111

90.454 0.886 57.819 1.072 92.018
ENSMUSG00000057715 A830018L16Rik protein_coding 1.107 69.489 1.123 82.904 0.804 7.948 0.909 85.493
ENSMUSG00000057716 Tmem178b protein_coding 0.944 82.982 0.969 79.475 1.092 65.567 0.906 85.493
ENSMUSG00000057719 Sh3rf2 protein_coding 0.86 75.858 1.609 71.617 0.72 15.465 1.295 95.273
ENSMUSG00000057722 Lepr protein_coding 1.308 0 0.906 73.166 0.841 7.083 0.964 88.245
ENSMUSG00000057723 Krt33b protein_coding 1 83.523 1 95.708 1 84.724 1 96.183
ENSMUSG00000057726 Serpinb9g protein_coding 1 72.546 1 86.604 1 66.928 1 96.358
ENSMUSG00000057729 Prtn3 protein_coding 1.83 0 1.154 93.994 0.472 0.72 1.277 70.539
ENSMUSG00000057735 Olfr1186 protein_coding 1 80.51 1 94.887 1 85.921 1 95.273
ENSMUSG00000057738 Sptan1 protein_coding 0.928 9.863 0.918 24.911 1.174 5.864 1.054 73.368
ENSMUSG00000057751 Megf6 protein_coding 0.935 73.585 0.905 76.431 1.183 36.839 0.894 85.493
ENSMUSG00000057754 Tas2r106 protein_coding 1 80.751 1 95.941 1 72.148 1 93.156
ENSMUSG00000057761 Olfr1022 protein_coding 1 85.655 NA NA 1 78.498 1 94.468
ENSMUSG00000057762 Gm6169 protein_coding 2.193 57.949 0.271 51.322 1.676 49.384 0.902 94.99
ENSMUSG00000057766 Ankrd29 protein_coding 1.03 71.896 NA NA 0.991 77.704 1.01 92.748
ENSMUSG00000057767 Olfr1383 protein_coding 1 76.042 1 95.301 1 84.626 1 90.865
ENSMUSG00000057770 Olfr668 protein_coding 1 84.534 1 95.301 NA NA 1 96.086
ENSMUSG00000057778 Cyb5d2 protein_coding 0.987 84.294 1.021 95.735 0.995 78.045 1.027 95.777
ENSMUSG00000057788 Ddx49 protein_coding 0.915 11.128 1.054 83.104 1.039 41.235 1.013 75.81
ENSMUSG00000057789 Bak1 protein_coding 0.942 15.912 1.003 96.138 1.058 24.029 1.046 46.412
ENSMUSG00000057799 Vmn1r216 protein_coding 1 77.182 1 89.509 1 86.084 1 94.654
ENSMUSG00000057801 Olfr135 protein_coding 1 86.145 1 96.847 1 78.155 1 96.183
ENSMUSG00000057802 Gm10030 protein_coding 1.14 83.968 0.963 95.504 0.911 86.346 1.179 93.457
ENSMUSG00000057805 1700084M14Rik protein_coding 1 86.57 1 93.765 1 84.071 1 95.676
ENSMUSG00000057816 1700007G11Rik protein_coding 1.127 82.231 0.938 97.097 0.949 88.601 1.539 83.276
ENSMUSG00000057817 Olfr1446 protein_coding 1 85.655 1 96.68 1 86.666 1 96.287
ENSMUSG00000057818 1600029O15Rik pseudogene 1.134 57.38 0.77 67.739 1.145 70.1 0.736 84.29
ENSMUSG00000057829 Gm5278 pseudogene 1 54.176 1

93.908	1	48.304	1	87.447
ENSMUSG00000057832	Olfr695	protein_coding	1	80.688	1
96.897	1	82.161	1	95.273
ENSMUSG00000057835	Zfp119a	protein_coding	1.199	8.537	0.905
59.946	0.925	76.768	0.956	91.65
ENSMUSG00000057836	Xlr3a	protein_coding	0.443	40.217	1.704
86.34	1.319	75.809	2.389	60.31
ENSMUSG00000057841	Rpl32	protein_coding	0.929	67.163	1.024
86.861	1.051	36.011	1.054	64.905
ENSMUSG00000057842	Zfp595	protein_coding	1.091	39.075
0.957	81.443	NA	NA	0.958	93.261
ENSMUSG00000057855	Krtap6-3	protein_coding	1	86.843	NA
NA	1	88.601	1	96.071
ENSMUSG00000057858	D19Ertd737e	protein_coding	0.957	36.945
0.982	95.211	1.059	33.287	0.994	92.564
ENSMUSG00000057863	Rpl36	protein_coding	0.918	27.968	1.016
95.708	1.074	36.95	0.953	88.552
ENSMUSG00000057880	Abat	protein_coding	1.412	2.784	0.876	83.58	0.81
7.809	0.94	86.699
ENSMUSG00000057890	Olfr1375-ps1	pseudogene	1	83.09	1
97.263	1	81.525	1	96.248
ENSMUSG00000057894	Zfp329	protein_coding	0.968	40.568
0.957	93.654	1.08	35.673	0.976	93.612
ENSMUSG00000057895	Zfp105	protein_coding	1.062	32.411
0.879	59.946	1.075	53.449	1.042	90.674
ENSMUSG00000057897	Camk2b	protein_coding	1.429	36.195
0.904	87.322	0.773	59.922	1.139	83.276
ENSMUSG00000057899	Gpr111	protein_coding	0.966	82.231
0.946	87.452	1.097	67.163	0.648	83.724
ENSMUSG00000057903	Olfr739	protein_coding	1	86.199	1
96.68	1	88.076	1	93.612
ENSMUSG00000057913	Gm10032	protein_coding	0.417	19.595
0.778	83.733	3.073	8.466	1.302	93.722
ENSMUSG00000057914	Cacnb2	protein_coding	1.015	86.819
0.863	74.633	1.143	54.673	NA	NA
ENSMUSG00000057924	Gm10033	protein_coding	1.217	15.602	0.91
87.919	0.899	67.962	0.955	92.675
ENSMUSG00000057945	Gm17136	pseudogene	1	84.661	1
88.881	1	76.768	1	96.248
ENSMUSG00000057946	Vmn1r181	protein_coding	1	86.474	1
96.68	1	88.461	1	95.92
ENSMUSG00000057948	Unc13d	protein_coding	1.026	66.944
0.947	72.399	1.031	77.532	0.887	85.493
ENSMUSG00000057963	Itpk1	protein_coding	1.017	84.452	1.021
90.389	0.965	59.726	1.199	70.539
ENSMUSG00000057967	Fgf18	protein_coding	1.351	34.439	1.61
83.464	0.459	42.895	1.066	61.984
ENSMUSG00000057969	Sema3b	protein_coding	0.902	48.939
0.967	94.48	1.147	85.792	1.089	76.831
ENSMUSG00000057977	Skint11	protein_coding	1	73.401	1
97.263	1	87.686	1	93.612
ENSMUSG00000057981	Vmn1r12	protein_coding	1	82.732	1
97.097	1	87.898	1	95.559
ENSMUSG00000057982	Zfp809	protein_coding	0.936	73.292

1.013 96.013 1.053 76.498 1.069 71.305
ENSMUSG00000057990 E030024N20Rik pseudogene 0.961 59.028
0.956 89.509 1.086 41.235 0.749 86.991
ENSMUSG00000057997 Olfr541 protein_coding 1 81.196 1
95.504 1 81.525 1 94.904
ENSMUSG00000058005 Tdpoz3 protein_coding 1 85.637 NA
NA 1 84.229 1 96.183
ENSMUSG00000058006 Mdn1 protein_coding 0.896 18.907 0.943
93.654 1.18 34.285 0.991 89.052
ENSMUSG00000058013 11-Sep protein_coding 1.181 6.23 0.944
90.576 0.899 21.331 1.109 38.624
ENSMUSG00000058014 Olfr502 protein_coding 1 80.146 1
96.216 1 87.431 1 96.086
ENSMUSG00000058017 Serpinb3d protein_coding 1 86.74 1
97.288 1 88.41 1 96.086
ENSMUSG00000058022 Adtrp protein_coding 1.333 8.685 0.826 82.947
0.909 28.489 0.773 83.724
ENSMUSG00000058028 Zscan5b protein_coding 1 84.263 1
97.288 1 88.161 1 95.877
ENSMUSG00000058030 Vmn1r237 protein_coding 1 85.655 1
95.504 1 84.308 1 95.273
ENSMUSG00000058046 4933430I17Rik protein_coding 0.811 41.759
0.857 87.147 1.443 37.369 1.253 90.463
ENSMUSG00000058050 Gm9234 pseudogene 1 75.671 1
95.35 1 55.214 1 92.748
ENSMUSG00000058052 Defb35 protein_coding 1 66.272 1
92.404 1 86.602 1 93.612
ENSMUSG00000058056 Palld protein_coding 1.038 72 1.027 83.104
0.938 27.994 1.221 65.845
ENSMUSG00000058057 Mettl7a3 protein_coding 0.968 83.523
1.814 88.823 0.57 24.523 1.278 93.722
ENSMUSG00000058063 Trim31 protein_coding 1 80.751 NA
NA 1 84.669 1 93.964
ENSMUSG00000058064 Gm10036 protein_coding 1.238 62.689
1.089 97.263 0.742 59.922 1.183 93.627
ENSMUSG00000058070 Eml1 protein_coding 1.141 10.595 0.909
83.58 0.965 38.534 1.109 76.697
ENSMUSG00000058071 Olfr818 protein_coding 1 80.688 1
95.301 1 85.935 1 95.74
ENSMUSG00000058073 Gm11189 pseudogene 1 85.129 NA NA
1 84.434 1 89.498
ENSMUSG00000058076 Sdhc protein_coding 0.9 3.07 1.061 71.617
1.048 27.475 0.989 87.496
ENSMUSG00000058078 Olfr1503-ps1 pseudogene 1 82.231 1
95.211 1 85.21 1 92.181
ENSMUSG00000058079 Olfr605 protein_coding 1 71.36 1
97.155 1 84.724 1 96.042
ENSMUSG00000058084 NA NA NA NA 1 96.216 1
86.258 1 95.273
ENSMUSG00000058093 AA987161 protein_coding 1.125 2.651 0.896
43.505 0.993 84.669 1.059 96.183
ENSMUSG00000058099 Nfam1 protein_coding 1.684 0 0.817 93.765
0.725 1.48 1.149 60.968
ENSMUSG00000058114 Olfr127 protein_coding 1 79.577 1

89.509 1 71.232 1 94.904
ENSMUSG00000058119 Gm5771 protein_coding 1 75.298 1
96.847 1 79.625 1 96.042
ENSMUSG00000058124 H2-M10.3 protein_coding 1 83.169 1
96.138 1 85.935 1 95.72
ENSMUSG00000058126 Tpm3-rs7 protein_coding 1.71 77.182
0.488 86.728 1.196 86.258 1.301 92.018
ENSMUSG00000058132 Vmn1r82 protein_coding 1 82.479 1
94.597 1 86.258 1 91.65
ENSMUSG00000058135 Gstm1 protein_coding 0.857 21.658 1.095
60.309 1.067 79.554 0.869 83.724
ENSMUSG00000058145 Adamts17 protein_coding 0.834 35.158
1.183 83.104 1.01 78.155 1.165 69.404
ENSMUSG00000058147 Xlr3c protein_coding 0.216 5.778 2.378 82.904
1.955 62.648 1.775 72.826
ENSMUSG00000058152 Chsy3 protein_coding 1.597 6.438 0.984 96.407
0.637 9.563 0.947 94.904
ENSMUSG00000058153 Sez6l protein_coding 0.849 74.8 1.333 79.367
0.883 51.443 0.983 96.184
ENSMUSG00000058163 Gm5431 protein_coding 1.687 0 0.703
70.864 0.842 18.797 1.166 70.539
ENSMUSG00000058172 Krtap6-1 protein_coding 1 82.231 1
95.088 1 70.9 1 96.042
ENSMUSG00000058173 Smco4 protein_coding 1.039 84.738 0.938
95.504 1.028 87.686 0.957 84.29
ENSMUSG00000058174 Gm5148 protein_coding 0.756 16.895
0.895 82.848 1.478 7.143 0.705 85.493
ENSMUSG00000058176 Gm5407 protein_coding 1 73.292 1
96.013 1 88.41 1 96.248
ENSMUSG00000058183 Mmel1 protein_coding 0.811 84.168 0.587
91.532 2.102 68.643 1.171 93.734
ENSMUSG00000058186 Gm13242 protein_coding 0.195 22.431
1.927 83.104 2.663 73.309 3.577 86.301
ENSMUSG00000058188 Olfr746 protein_coding 1 72.139 NA
NA 1 87.274 1 96.183
ENSMUSG00000058192 Zfp846 protein_coding 1.022 46.097
1.116 79.411 0.879 8.382 1.057 92.181
ENSMUSG00000058194 Olfr1126 protein_coding 1 85.283 1
96.216 1 81.032 1 96.071
ENSMUSG00000058200 Olfr66 protein_coding 1 75.298 1
96.013 1 84.669 1 92.762
ENSMUSG00000058207 Serpina3k protein_coding 0.295 20.369
1.783 80.678 1.894 77.888 0.872 88.387
ENSMUSG00000058216 BC021614 protein_coding 0.696 54.176
0.786 70.971 1.821 34.403 1.367 83.067
ENSMUSG00000058222 Olfr318 protein_coding 1 82.352 1
95.504 1 84.504 1 96.248
ENSMUSG00000058230 Grlf1 protein_coding 1.035 19.893 1.094
71.222 0.882 0.72 1.012 84.499
ENSMUSG00000058239 Usf2 protein_coding 0.949 44.787 1.025
96.407 1.03 67.823 0.966 83.724
ENSMUSG00000058240 Cryzl1 protein_coding 1.132 0.664 0.941
57.613 0.941 54.25 1.007 94.904
ENSMUSG00000058244 NA NA NA NA 1 96.308 1

87.103	1	96.183
ENSMUSG00000058246	Gm10037	protein_coding	0.929	80.751
1.143	97.263	0.942	88.657	1.062	82.85
ENSMUSG00000058248	Kcnh1	protein_coding	0.65	15.486	0.756
71.331	2.037	7.482	0.677	86.699
ENSMUSG00000058250	Tas2r138	protein_coding	1	82.982	NA
NA	1	86.112	1	90.455
ENSMUSG00000058251	Olfr822	protein_coding	1	76.546	1
97.263	1	85.352	1	94.904
ENSMUSG00000058252	1700008I05Rik	protein_coding	1	84.865
1	83.733	1	69.728	1	85.577
ENSMUSG00000058254	Tspan7	protein_coding	0.922	48.146
1.022	89.494	1.06	87.786	0.983	90.611
ENSMUSG00000058258	Idi1	protein_coding	0.841	12.436	1.284
79.367	0.921	83.719	1.03	60.466
ENSMUSG00000058260	Serpina9	protein_coding	1	62.689	1
97.155	1	66.201	1	85.493
ENSMUSG00000058267	Mrps14	protein_coding	0.887	12.436
0.988	89.713	1.142	13.956	0.98	94.904
ENSMUSG00000058270	Olfr25	protein_coding	1	86.006	1
96.763	1	86.985	1	95.273
ENSMUSG00000058275	Olfr412	protein_coding	1	79.709	1
97.263	1	86.602	1	91.105
ENSMUSG00000058281	Gm10038	pseudogene	1.029	84.534	1.135
90.378	0.852	77.17	0.996	96.183
ENSMUSG00000058290	Espl1	protein_coding	1.511	0.976	0.743	20.174
0.894	73.598	1.178	59.208
ENSMUSG00000058291	Zfp68	protein_coding	1.162	0.767	0.909	74.851
0.948	11.314	0.999	92.12
ENSMUSG00000058295	Prp2	protein_coding	1	79.853	1
97.263	1	88.41	1	95.877
ENSMUSG00000058297	Spock2	protein_coding	0.931	49.621
0.987	94.597	1.092	45.208	0.825	96.287
ENSMUSG00000058298	Mcm9	protein_coding	1.062	34.861	0.864
53.434	1.084	58.616	0.992	94.99
ENSMUSG00000058301	Upf1	protein_coding	0.969	54.87	1.049	60.309
0.983	20.009	1.008	95.92
ENSMUSG00000058317	Ube2e2	protein_coding	1.078	56.311
1.039	83.104	0.895	19.765	0.969	95.273
ENSMUSG00000058318	Phf21a	protein_coding	0.95	67.477
1.016	95.504	1.035	38.975	0.943	83.724
ENSMUSG00000058325	Dock1	protein_coding	1.007	67.991	1.016
93.574	0.972	56.625	1.007	89.498
ENSMUSG00000058328	NA	NA	NA	NA	NA	NA	1	87.744
1	92.762
ENSMUSG00000058331	Zfp85-rs1	protein_coding	1.104	62.007
0.883	75.345	1.021	78.369	1.059	92.389
ENSMUSG00000058349	NA	NA	NA	NA	1	94.367	1
87.744	NA	NA
ENSMUSG00000058351	Smim4	protein_coding	0.844	2.142	1.163	82.904
1.02	54.363	0.984	91.872
ENSMUSG00000058354	Krt6a	protein_coding	3.478	4.052	0.506	34.058
0.568	75.637	2.607	52.366
ENSMUSG00000058355	Abce1	protein_coding	1.006	77.497	1.004

96.847 0.988 71.999 1.148 61.316
ENSMUSG00000058360 Gm10040 pseudogene 1 76.827 1
94.262 1 73.598 1 93.685
ENSMUSG00000058368 Krtap21-1 protein_coding 1 86.006 1
95.504 1 88.41 1 94.654
ENSMUSG00000058385 Hist1h2bg protein_coding 1.083 57.133
0.957 90.744 0.962 83.607 0.773 83.724
ENSMUSG00000058388 Phtf1 protein_coding 0.981 67.867 1.051
76.065 0.968 63.919 1.054 70.36
ENSMUSG00000058392 Rrp1b protein_coding 0.928 68.66 1.044 92.914
1.032 62.307 1.136 56.079
ENSMUSG00000058396 Gpr182 protein_coding 0.905 45.728
0.902 90.378 1.221 38.038 0.967 95.877
ENSMUSG00000058399 Vmn1r64 protein_coding 1 82.982 1
95.088 1 84.552 1 96.358
ENSMUSG00000058400 Qrfpr protein_coding 1 85.655 1
96.68 1 87.658 1 89.428
ENSMUSG00000058402 Zfp420 protein_coding 1.131 6.683 0.998
90.053 0.886 19.552 0.94 92.12
ENSMUSG00000058407 Txndc9 protein_coding 1.099 23.254
0.947 59.946 0.963 82.575 1.041 57.952
ENSMUSG00000058420 Syt17 protein_coding 1.297 33.854 0.98
92.931 0.787 23.212 1.087 86.991
ENSMUSG00000058430 Rpl27a-ps2 pseudogene 1 73.585 1
90.378 1 87.37 1 96.071
ENSMUSG00000058435 Btnl4 protein_coding 0.707 60.707 0.843
96.216 1.68 66.043 1.166 91.872
ENSMUSG00000058440 Nrf1 protein_coding 1.064 11.459 NA NA
0.942 18.336 0.982 91.625
ENSMUSG00000058441 Panx2 protein_coding 0.887 14.403 0.862
92.137 1.306 22.444 0.82 96.183
ENSMUSG00000058444 Map2k5 protein_coding 0.947 53.679
1.028 88.91 1.023 84.961 0.963 87.651
ENSMUSG00000058446 Znrf2 protein_coding 0.978 73.124 1.12
85.756 0.916 19.552 1.103 88.512
ENSMUSG00000058447 Gm26920 protein_coding 1 85.499 NA
NA 1 85.352 1 93.612
ENSMUSG00000058454 Dhcr7 protein_coding 1.014 72.139 1.1
72.546 0.895 44.019 1.061 61.753
ENSMUSG00000058463 Gm5471 pseudogene 1 85.355 1
96.407 1 88.601 1 94.608
ENSMUSG00000058470 Gm8369 protein_coding 1.238 72.655
0.629 70.693 1.282 48.304 1.122 94.215
ENSMUSG00000058486 Wdr91 protein_coding 1.034 76.042 1.043
90.454 0.927 55.701 0.974 93.457
ENSMUSG00000058491 Olfr869 protein_coding 1 71.217 1
97.263 1 87.173 1 95.72
ENSMUSG00000058492 Scp2-ps2 pseudogene 1 71.674 1
96.897 1 87.431 1 89.72
ENSMUSG00000058498 Rnf207 protein_coding 0.941 86.333
1.194 87.604 0.889 67.648 0.904 85.493
ENSMUSG00000058499 Pip protein_coding 1 75.567 1
95.504 1 80.749 1 93.132
ENSMUSG00000058503 Fam133b protein_coding 0.965 58.56 1.036

83.464 0.997 87.686 1.001 96.086
ENSMUSG00000058508 Gm10042 pseudogene 1 77.615 1
95.211 1 88.203 1 96.358
ENSMUSG00000058513 Olfr801 protein_coding 1 81.196 1
96.268 NA NA 1 94.82
ENSMUSG00000058515 Olfr933 protein_coding 1 74.8 1
95.301 1 82.903 1 96.183
ENSMUSG00000058521 Olfr892-ps1 pseudogene 1 86.474 1
96.308 1 86.084 1 92.31
ENSMUSG00000058537 AW822073 protein_coding 1 82.092 NA
NA 1 88.41 1 89.827
ENSMUSG00000058542 Gm15590 pseudogene 1 58.264 1
94.96 1 85.352 1 93.964
ENSMUSG00000058546 Rpl23a protein_coding 1.023 67.991
0.946 69.611 1.036 80.089 1.001 91.872
ENSMUSG00000058550 Dppa4 protein_coding 1 81.823 1
94.848 1 77.294 1 95.273
ENSMUSG00000058558 Rpl5 protein_coding 1.15 6.026 0.946 68.818
0.919 32.297 1.084 44.882
ENSMUSG00000058569 Tmed9 protein_coding 0.973 27 0.981 93.654
1.042 34.727 1.085 44.213
ENSMUSG00000058571 Gpc6 protein_coding 1.046 53.809 1.055
97.263 0.904 56.625 1 87.542
ENSMUSG00000058581 Gm5801 pseudogene 1 73.841 1
92.355 1 87.431 1 96.042
ENSMUSG00000058586 Serhl protein_coding 0.907 18.731 1.056
96.897 1.043 49.058 0.981 92.31
ENSMUSG00000058587 Tmod3 protein_coding 1.073 21.703 1.011
96.268 0.921 19.765 0.988 88.111
ENSMUSG00000058588 Vmn1r21 protein_coding 1 82.479 1
96.216 1 86.475 1 96.248
ENSMUSG00000058589 Anks1b protein_coding 1.669 22.349 NA
NA 0.57 25.345 0.989 95.832
ENSMUSG00000058590 Gm6462 pseudogene 1 81.823 1
96.847 1 73.309 1 96.358
ENSMUSG00000058594 Fbxo18 protein_coding 0.988 50.519
0.958 62.025 1.056 11.348 1.055 33.269
ENSMUSG00000058600 Rpl30 protein_coding 1.09 5.932 0.922 31.138
0.998 81.388 1.053 83.067
ENSMUSG00000058603 Rpl28-ps1 pseudogene 4.598 0 2.028 83.104
0.108 0.72 1.052 96.086
ENSMUSG00000058607 Rpl31-ps20 pseudogene 1 83.169 1
96.652 NA NA 1 95.273
ENSMUSG00000058613 Gm5062 pseudogene 1 84.049 1
96.013 NA NA 1 94.99
ENSMUSG00000058620 Adra2b protein_coding 1.337 64.714
0.772 93.654 0.969 66.425 0.975 91.105
ENSMUSG00000058622 Gm5265 pseudogene 1 82.543 1
97.155 1 86.985 1 92.12
ENSMUSG00000058624 Gda protein_coding 1.016 58.709 0.976
90.378 1.009 86.552 1.283 31.133
ENSMUSG00000058625 Gm17383 pseudogene 1 81.125 NA NA
1 73.531 1 93.261
ENSMUSG00000058626 Capn11 protein_coding 0.349 81.465 3.94

72.642 0.728 47.641 1.156 88.505
ENSMUSG00000058628 Olfr875 protein_coding 1 86.918 1
95.504 1 86.727 1 94.904
ENSMUSG00000058631 Vmn1r63 protein_coding 1 72.985 1
96.138 1 85.921 1 94.82
ENSMUSG00000058638 Zfp110 protein_coding 1.146 0 0.893
33.301 0.978 62.791 1.015 95.273
ENSMUSG00000058643 Speer4f protein_coding 1 86.351 1
96.138 1 84.626 1 92.389
ENSMUSG00000058655 Eif4b protein_coding 1.129 2.352 0.841 20.174
1.053 65.235 0.944 89.892
ENSMUSG00000058656 Samd12 protein_coding 0.908 76.396
0.956 93.094 1.154 48.772 0.912 91.105
ENSMUSG00000058659 Olfr58 protein_coding 1 69.777 NA
NA 1 70.9 1 91.928
ENSMUSG00000058662 Olfr69 protein_coding 1 86.474 1
96.68 1 86.985 1 96.071
ENSMUSG00000058665 En1 protein_coding 0.999 84.263 1.151
82.904 0.87 46.071 1.075 89.108
ENSMUSG00000058669 Nkx2-9 protein_coding 1 84.452 NA
NA 1 80.015 1 94.904
ENSMUSG00000058670 4932411N23Rik protein_coding 1 74.482
NA NA 1 77.704 1 95.92
ENSMUSG00000058672 Tubb2a protein_coding 0.899 69.24 1.099
83.104 1.014 86.084 1.236 58.557
ENSMUSG00000058685 Vmn2r51 protein_coding 1 81.01 1
93.654 1 85.921 1 94.001
ENSMUSG00000058690 Ccser2 protein_coding 0.958 65.388 1.06
72.642 0.985 45.799 1.012 95.74
ENSMUSG00000058692 Olfr846 protein_coding 1 86.351 1
96.138 NA NA 1 94.215
ENSMUSG00000058700 Gm8724 pseudogene 1 86.006 1
94.102 1 85.134 1 95.559
ENSMUSG00000058704 Memo1 protein_coding 1.011 79.068 1.097
60.309 0.9 18.122 1.093 60.466
ENSMUSG00000058706 0610030E20Rik protein_coding 0.976 64.589
0.99 96.308 1.032 80.634 0.997 92.018
ENSMUSG00000058709 Egln2 protein_coding 0.922 33.906 1.01
92.137 1.071 40.955 1.017 92.31
ENSMUSG00000058715 Fcer1g protein_coding 1.651 0 0.841
77.598 0.72 0.945 1.21 60.466
ENSMUSG00000058717 Gm4763 protein_coding 1 83.823 1
96.308 1 82.778 1 93.132
ENSMUSG00000058725 Gm11937 protein_coding 1 74.516 1
95.088 1 86.985 1 93.964
ENSMUSG00000058728 Cd300c protein_coding 0.617 41.759
0.791 87.857 2.052 21.462 1.63 73.939
ENSMUSG00000058729 Lin9 protein_coding 0.89 49.944 1.099
96.847 1.025 50.636 1.035 91.011
ENSMUSG00000058731 NA NA NA NA NA NA NA NA 1
95.832
ENSMUSG00000058736 Gm10047 protein_coding 0.846 80.608
1.254 90.092 0.939 87.173 1.085 93.261
ENSMUSG00000058740 Kcnt1 protein_coding 0.864 45.894 0.748

88.691 1.552 25.93 1.325 72.992
ENSMUSG00000058741 Prr19 protein_coding 1 82.231 1
96.68 1 37.906 1 91.372
ENSMUSG00000058743 Kcnj14 protein_coding 0.821 22.481
1.236 79.055 NA NA 0.838 93.413
ENSMUSG00000058745 Gm9174 pseudogene 1 72.27 1 96.847
1 87.786 1 95.777
ENSMUSG00000058748 Zfp958 protein_coding 1.43 0.2 0.894
63.773 0.782 6.935 0.938 87.992
ENSMUSG00000058754 Gm10048 pseudogene 1 84.738 NA NA
1 88.601 1 95.273
ENSMUSG00000058755 Osm protein_coding 1.58 7.255 1.057 87.452
0.597 44.019 1.117 96.184
ENSMUSG00000058756 Thra protein_coding 1.193 17.582 NA NA
0.825 7.509 0.91 83.724
ENSMUSG00000058761 Rnf169 protein_coding 1.231 7.959 0.978
82.947 0.829 25.625 0.977 87.886
ENSMUSG00000058762 Btnl6 protein_coding 1 84.534 1
95.431 1 88.161 1 96.287
ENSMUSG00000058773 Hist1h1b protein_coding 0.927 80.608
0.889 92.793 1.208 84.724 0.762 96.086
ENSMUSG00000058793 Cds2 protein_coding 1.093 0 1.012 96.013
0.901 0.72 0.957 83.724
ENSMUSG00000058794 Nfe2 protein_coding 1.957 5.253 0.594 55.71
0.859 79.255 0.826 88.505
ENSMUSG00000058799 Nap1l1 protein_coding 1.187 12.985
0.929 85.075 0.909 38.799 1.057 68.502
ENSMUSG00000058802 Olfr97 protein_coding 1 76.396 1
97.155 1 81.525 1 94.825
ENSMUSG00000058806 Col13a1 protein_coding 0.821 30.637
1.123 82.904 1.087 74.686 1.395 57.839
ENSMUSG00000058807 Olfr331 protein_coding 1 82.982 1
97.263 1 67.562 1 94.153
ENSMUSG00000058812 0610039K10Rik antisense 0.75 3.641 NA NA
1.321 10.079 0.797 83.724
ENSMUSG00000058816 Ppp1r2-ps3 pseudogene 1 67.374 1
97.129 1 88.41 1 91.291
ENSMUSG00000058818 Pirb protein_coding 1.34 0.984 0.832 66.651
0.897 46.575 1.298 72.826
ENSMUSG00000058820 Olfr146 protein_coding 1 83.726 1
90.287 1 78.369 1 93.316
ENSMUSG00000058831 Opn1sw protein_coding 0.518 52.258
1.279 95.211 1.511 75.527 1.74 78.546
ENSMUSG00000058833 2810428I15Rik protein_coding 0.798 9.752
1.149 79.864 1.092 79.119 1.059 95.74
ENSMUSG00000058835 Abi1 protein_coding 1.134 0 0.961 60.678
0.919 2.889 0.989 93.964
ENSMUSG00000058838 NA NA NA NA 1 96.948 NA NA
1 94.016
ENSMUSG00000058856 Olfr952 protein_coding 1 78.909 1
94.102 1 88.693 NA NA
ENSMUSG00000058873 Gm5582 pseudogene 1 74.147 1
96.68 1 88.601 1 95.832
ENSMUSG00000058881 Zfp516 protein_coding 0.999 86.006

0.984 90.046 1.018 69.002 0.975 88.245
ENSMUSG00000058883 Zfp708 protein_coding 1.197 29.811
0.918 78.877 0.906 87.103 0.956 92.12
ENSMUSG00000058884 Olfr1025-ps1 protein_coding 1 86.112
1 97.263 NA NA 1 95.74
ENSMUSG00000058886 Deaf1 protein_coding 1.017 84.534 1.043
82.809 0.943 24.309 1.034 64.513
ENSMUSG00000058897 Col25a1 protein_coding 1.171 62.689 1.05
84.007 0.811 24.918 1.114 96.287
ENSMUSG00000058900 Rsl1 protein_coding 1.156 5.559 0.914 92.137
0.943 18.797 0.932 83.724
ENSMUSG00000058904 Olfr1413 protein_coding 1 85.283 1
95.088 1 86.258 1 96.287
ENSMUSG00000058905 Gm10051 pseudogene 1.143 69.489 0.607
38.699 1.445 7.635 1.008 95.74
ENSMUSG00000058908 Pla2g2a polymorphic_pseudogene 1 86.351
1 96.268 1 87.37 1 95.405
ENSMUSG00000058914 C1qtnf3 protein_coding 1.081 69.777
1.044 74.851 0.881 80.634 0.552 83.724
ENSMUSG00000058921 Slc10a5 protein_coding 0.934 60.569
1.027 97.155 1.039 73.309 0.839 85.493
ENSMUSG00000058922 Gm10052 pseudogene 1.154 48.288 0.766
90.287 1.129 80.015 0.855 91.372
ENSMUSG00000058923 Vmn1r6 protein_coding 1 86.351 1
95.504 NA NA 1 94.904
ENSMUSG00000058927 Gm10053 protein_coding 0.515 3.009 1.309
72.642 1.482 21.201 1.15 67.691
ENSMUSG00000058932 Gm2174 protein_coding 1 83.169 1
92.137 1 79.776 1 77.621
ENSMUSG00000058933 Gm5185 pseudogene 1 86.464 1
96.843 1 85.757 1 94.904
ENSMUSG00000058934 Gm16176 antisense 0.72 25.3 0.783 82.947
1.777 10.523 1.176 94.567
ENSMUSG00000058935 1700018C11Rik protein_coding 1 59.674
1 93.863 1 84.896 1 96.358
ENSMUSG00000058945 Gm10056 pseudogene 0.488 18.138 1.472
91.065 1.388 64.305 1.102 87.134
ENSMUSG00000058952 Cfi protein_coding 1.919 16.539 0.527
68.115 0.99 86.552 1.352 83.581
ENSMUSG00000058953 Gm4822 pseudogene 1 78.744 1
94.102 1 84.626 1 95.877
ENSMUSG00000058966 Fam57b protein_coding 0.538 0.987 1.208
90.116 1.541 5.864 0.958 87.705
ENSMUSG00000058976 Usp17lc protein_coding 1 86.199 NA
NA 1 86.727 1 87.447
ENSMUSG00000058979 Cecr5 protein_coding 0.842 2.009 1.077 71.98
1.103 24.07 1.017 96.358
ENSMUSG00000058981 Olfr1406 protein_coding 1 84.977 1
95.504 1 86.727 1 93.413
ENSMUSG00000058997 Vwa8 protein_coding 1.003 61.725 0.933
86.071 1.069 56.708 0.997 94.608
ENSMUSG00000059003 Grin2a protein_coding 1 46.693 1
93.994 1 75.155 1 94.654
ENSMUSG00000059005 Hnrnpa3 protein_coding 1.027 66.76 0.915

78.471 1.066 58.051 1.172 31.133
ENSMUSG00000059013 Sh2d3c protein_coding 0.858 13.239 NA
NA 1.074 27.732 0.929 86.991
ENSMUSG00000059014 Gm5409 pseudogene 1 82.092 1
96.68 1 88.693 1 96.358
ENSMUSG00000059022 Kcp protein_coding 0.9 33.415 0.906
96.216 1.228 48.304 1.048 71.008
ENSMUSG00000059023 Olfr1201 protein_coding 1 72.372 1
95.431 NA NA 1 95.92
ENSMUSG00000059027 9630013D21Rik processed_transcript 0.749
3.956 1.328 83.104 1.002 40.575 0.912 94.19
ENSMUSG00000059030 Olfr128 protein_coding 1 81.465 1
96.268 1 87.173 1 96.358
ENSMUSG00000059031 Olfr482 protein_coding 1 84.738 1
95.211 1 86.475 1 96.358
ENSMUSG00000059033 Rpl18a-ps1 pseudogene 1 85.129 1
95.088 1 86.727 1 89.72
ENSMUSG00000059040 Gm5506 protein_coding 0.509 3.07 1.194
92.561 1.646 20.051 1.355 92.748
ENSMUSG00000059041 Olfr192 pseudogene 1 81.01 1 95.708
1 87.686 1 95.593
ENSMUSG00000059042 Klk1b9 protein_coding 1 81.657 NA
NA 1 78.954 1 86.445
ENSMUSG00000059043 Olfr15 protein_coding 1 86.199 1
95.708 1 88.349 1 95.405
ENSMUSG00000059049 Frem1 protein_coding 1.319 45.096 1.066
95.708 0.71 56.822 1.416 72.616
ENSMUSG00000059060 Rad51b protein_coding 1.699 57.133
0.747 94.102 0.789 77.037 1.213 75.198
ENSMUSG00000059064 NA NA NA NA 1 96.216 1
73.531 1 89.892
ENSMUSG00000059069 Olfr749 protein_coding 1 72.655 1
95.504 1 77.294 1 95.877
ENSMUSG00000059070 Rpl18 protein_coding 0.931 30.352 0.942
60.678 1.14 10.615 1.055 89.428
ENSMUSG00000059077 Pth protein_coding 1 76.546 1
97.155 1 82.74 1 94.904
ENSMUSG00000059087 Olfr698 protein_coding 1 72.546 1
94.597 1 79.426 1 94.82
ENSMUSG00000059089 Fcgr4 protein_coding 1.859 7.255 1.052 85.793
0.512 20.751 1.355 68.696
ENSMUSG00000059101 Olfr157 protein_coding 1 64.714 1
95.735 1 59.922 1 91.105
ENSMUSG00000059105 Olfr1504 protein_coding 1 86.172 1
96.407 1 84.504 1 93.964
ENSMUSG00000059106 Olfr961 protein_coding 1 86.843 1
95.35 1 87.519 1 96.358
ENSMUSG00000059108 Ifitm6 protein_coding 1.5 6.135 0.753
70.693 0.883 59.383 1.158 84.31
ENSMUSG00000059112 Olfr1263 protein_coding 1 81.925 1
96.138 1 79.897 1 96.183
ENSMUSG00000059113 Gm10061 protein_coding 1 83.169 1
96.407 1 88.076 1 94.99
ENSMUSG00000059114 Gm6772 protein_coding 1 67.163 1

97.049 1 87.336 1 93.264
ENSMUSG00000059119 Nap1l4 protein_coding 0.948 4.73 1.019
96.68 1.033 13.082 1.055 31.133
ENSMUSG00000059128 Ifnl2 protein_coding 1 79.709 1
95.431 1 82.74 1 96.192
ENSMUSG00000059134 Olfr814 protein_coding 1 81.01 1
96.652 NA NA 1 93.156
ENSMUSG00000059136 Olfr539 protein_coding 1 86.112 1
95.35 1 82.685 1 94.001
ENSMUSG00000059142 Zfp945 protein_coding 0.982 73.585
0.895 87.322 1.133 33.802 0.977 86.699
ENSMUSG00000059146 Ntrk3 protein_coding 0.631 0.987 0.891 95.088
1.78 5.864 1.031 87.7
ENSMUSG00000059149 Mfsd4 protein_coding 0.949 65.733 1.236
82.904 0.854 18.539 0.899 86.991
ENSMUSG00000059150 Rpl26-ps4 pseudogene 1 58.264 1
97.155 1 81.032 1 96.248
ENSMUSG00000059159 Gm8129 pseudogene 10.012 0 0.838
86.538 0.119 0.72 0.957 92.263
ENSMUSG00000059169 Krt40 protein_coding 1 66.58 1 87.041
1 74.325 1 86.445
ENSMUSG00000059173 Pde1a protein_coding 0.904 57.544 0.977
93.817 1.129 49.058 1.057 85.786
ENSMUSG00000059179 Gm8991 protein_coding 1 81.24 1
96.216 1 87.431 1 96.358
ENSMUSG00000059182 Skap2 protein_coding 1.187 0.2 0.901 70.29
0.932 32.703 1.085 43.377
ENSMUSG00000059183 Mtfmt protein_coding 1.031 42.134 0.927
92.137 1.05 84.669 1.016 93.964
ENSMUSG00000059187 Fam19a1 protein_coding 1.155 68.16 3.385
87.037 0.255 25.052 0.985 94.153
ENSMUSG00000059189 Olfr913 protein_coding 1 78.639 1
94.367 NA NA 1 95.832
ENSMUSG00000059195 Gm12715 pseudogene 0.744 55.303 1.06
90.68 1.264 88.601 1.285 92.748
ENSMUSG00000059201 Lep protein_coding 0.849 58.077 1.135
82.904 1.038 81.169 1.089 86.445
ENSMUSG00000059203 Il1rapl2 protein_coding 1 45.746 NA
NA 1 50.184 1 86.991
ENSMUSG00000059205 Olfr1130 protein_coding 1 81.01 1
91.661 NA NA 1 96.358
ENSMUSG00000059206 Vmn1r71 protein_coding 1 84.294 1
95.735 NA NA 1 95.74
ENSMUSG00000059208 Hnrnpm protein_coding 0.978 55.303
0.932 73.166 1.093 27.732 1.107 0
ENSMUSG00000059213 Ddn protein_coding 0.661 23.923 1.454
72.642 1.039 87.686 1.077 83.067
ENSMUSG00000059218 Gm13084 protein_coding 1 66.944 1
96.138 1 81.525 1 91.291
ENSMUSG00000059219 Olfr113 pseudogene 1 82.479 NA NA
1 86.084 1 93.156
ENSMUSG00000059227 Olfr494 protein_coding 1 86.172 1
95.504 1 71.839 1 94.384
ENSMUSG00000059229 Gm6802 protein_coding 1 79.277 1

94.597 1 82.31 1 92.851
ENSMUSG00000059230 Defb4 protein_coding 1 80.815 NA NA
1 71.615 1 94.567
ENSMUSG00000059244 Gm10062 lincRNA 1.037 44.334 1.356
90.712 0.712 69.445 0.806 91.372
ENSMUSG00000059246 Foxb1 protein_coding 1 81.823 1
96.407 1 86.144 1 95.593
ENSMUSG00000059248 9-Sep protein_coding 1.176 4.159 0.911 67.739
0.93 27.994 1.096 49.806
ENSMUSG00000059251 Olfr345 protein_coding 1 86.333 1
96.652 1 88.461 1 94.654
ENSMUSG00000059252 Tpt1-ps5 pseudogene 1 68.955 1
96.378 1 86.552 1 95.72
ENSMUSG00000059256 Gzmd protein_coding 1 84.397 1
93.654 1 84.961 1 92.263
ENSMUSG00000059263 Usp47 protein_coding 1.088 3.498 0.976 85.418
0.941 16.051 1.024 75.198
ENSMUSG00000059273 Zc3h4 protein_coding 1.025 50.913 NA NA
0.986 63.075 1.015 94.904
ENSMUSG00000059277 R74862 processed_transcript 0.724 11.384
1.118 93.654 1.234 10.819 1.01 86.445
ENSMUSG00000059278 Lsmd1 protein_coding 0.859 21.957 1.061
83.104 1.096 48.451 1.025 95.273
ENSMUSG00000059279 Olfr224 protein_coding 1 81.925 1
96.68 1 84.896 1 96.183
ENSMUSG00000059280 Vpreb2 protein_coding 1 83.968 1
94.367 1 77.17 1 90.691
ENSMUSG00000059288 Cdyl protein_coding 1.011 86.112 0.995
95.708 0.995 75.637 0.988 89.72
ENSMUSG00000059291 Rpl11 protein_coding 1.006 62.689 0.953
38.923 1.045 32.524 1.034 87.624
ENSMUSG00000059301 Gm5434 protein_coding 1 76.968 1
96.68 1 82.575 1 95.74
ENSMUSG00000059303 Olfr836 protein_coding 1 86.112 1
95.504 1 87.37 1 96.183
ENSMUSG00000059305 Vpreb1 protein_coding 0.648 12.084
1.095 82.904 1.411 38.975 1.272 79.268
ENSMUSG00000059309 Hist1h3f protein_coding 0.36 10.143
3.344 72.546 0.828 78.864 1.478 83.067
ENSMUSG00000059316 Slc27a4 protein_coding 0.92 5.204 1.084
64.123 1.004 79.554 0.969 89.892
ENSMUSG00000059319 Olfr295 protein_coding 1 86.658 1
96.216 1 76.923 1 96.287
ENSMUSG00000059323 Tonsl protein_coding 0.982 85.637 1.08
90.116 0.94 80.899 1.13 83.276
ENSMUSG00000059325 Hopx protein_coding 1.017 57.655 1.117
86.156 0.881 28.669 0.889 83.724
ENSMUSG00000059326 Csf2ra protein_coding 0.91 69.943
1.024 95.504 1.076 75.009 1.106 74.193
ENSMUSG00000059327 Eda protein_coding 1.24 2.784 NA NA
0.788 6.858 1.068 93.627
ENSMUSG00000059334 Zfp36l3 protein_coding 2.807 30.282
0.836 86.861 0.424 82.685 0.367 83.724
ENSMUSG00000059336 Slc14a1 protein_coding 1.701 24.689

0.767 75.993 0.763 42.621 0.609 83.724
ENSMUSG00000059343 Aldoart1 protein_coding 1 81.925 1
95.504 1 77.037 1 95.559
ENSMUSG00000059352 Gm10064 protein_coding 1 85.77 1
96.216 1 83.925 1 95.273
ENSMUSG00000059355 BC056474 protein_coding 0.95 16.325
1.037 83.104 1.015 68.129 0.948 93.261
ENSMUSG00000059363 Fxn protein_coding 0.965 78.054 1.028
90.378 1.002 84.626 0.908 86.991
ENSMUSG00000059366 Olfr959 protein_coding 1 85.728 1
96.001 1 88.461 1 96.184
ENSMUSG00000059371 Olfr427 protein_coding 1 86.464 1
96.138 1 81.525 1 93.964
ENSMUSG00000059375 Vmn1r33 protein_coding 1 60.003 1
96.652 1 87.658 1 95.74
ENSMUSG00000059379 Olfr1014 protein_coding 1 75.196 1
97.155 NA NA 1 95.877
ENSMUSG00000059382 Tas2r120 protein_coding 1 77.291 1
97.263 1 86.666 1 92.263
ENSMUSG00000059383 Gfral protein_coding 1 82.231 1
96.138 1 83.607 1 96.248
ENSMUSG00000059395 Nkapl protein_coding 0.79 16.895 1.187
79.411 1.065 87.744 1.105 96.358
ENSMUSG00000059397 Olfr54 protein_coding 1 60.707 1
97.263 1 86.666 1 92.675
ENSMUSG00000059401 Mamld1 protein_coding 1.108 56.085
1.054 79.29 0.856 11.751 0.95 87.542
ENSMUSG00000059406 Tmprss9 protein_coding 0.642 46.693
1.054 96.407 1.476 66.269 0.979 92.675
ENSMUSG00000059408 Mrgprh protein_coding 1.18 26.863
1.303 87.604 0.651 16.643 1.01 84.29
ENSMUSG00000059409 Ppp2r5d protein_coding 0.938 3.623 1.045
79.411 1.021 53.134 0.942 86.699
ENSMUSG00000059410 Tas2r125 protein_coding 1 81.24 1
95.211 1 74.325 1 96.358
ENSMUSG00000059411 Olfr434 protein_coding 1 85.77 1
96.897 1 86.831 1 96.248
ENSMUSG00000059412 Fxyd2 protein_coding 1.112 42.555 0.909
88.881 0.988 84.83 0.682 83.724
ENSMUSG00000059422 Gm8116 pseudogene 1 78.054 1
94.848 1 88.076 1 95.273
ENSMUSG00000059423 Zfp933 protein_coding 1.062 61.725
0.898 86.538 1.046 78.155 0.995 95.92
ENSMUSG00000059429 Olfr365 protein_coding 1 86.918 1
90.138 1 85.757 1 96.358
ENSMUSG00000059430 Actg2 protein_coding 0.983 80.51 1.225 69.228
0.833 26.809 1.14 66.468
ENSMUSG00000059434 Gckr protein_coding 0.815 69.24 0.876 93.765
1.404 38.975 0.633 84.29
ENSMUSG00000059436 Max protein_coding 1.002 72.655 0.955
53.79 1.045 41.235 1.043 72.369
ENSMUSG00000059439 Bcas3 protein_coding 0.99 81.52 1.008 92.062
1.002 50.184 0.995 84.29
ENSMUSG00000059447 Hadhb protein_coding 0.898 83.09 1.039 97.155

1.073 75.527 1.004 88.136
ENSMUSG00000059448 Olfr156 protein_coding 1 86.843 1
91.532 1 85.464 1 95.74
ENSMUSG00000059455 Plac8l1 protein_coding 1 82.092 1
93.765 NA NA 1 96.358
ENSMUSG00000059456 Ptk2b protein_coding 1.029 75.671 0.924
65.819 1.051 44.125 NA NA
ENSMUSG00000059459 Gm10065 protein_coding 0.787 80.251
1.069 92.355 1.186 51.851 1.275 95.273
ENSMUSG00000059460 Olfr286 protein_coding 1 85.857 1
95.504 1 88.601 1 93.316
ENSMUSG00000059461 Gm7331 pseudogene 1.489 72.372 0.622
96.138 1.076 86.727 0.957 94.904
ENSMUSG00000059463 Spag11b protein_coding 1 86.74 1
96.407 1 83.811 1 94.99
ENSMUSG00000059473 Olfr979 protein_coding 1 80.408 1
96.652 1 87.744 1 96.248
ENSMUSG00000059474 Mbtd1 protein_coding 1.064 14.746 0.945
87.553 0.995 82.575 0.943 83.724
ENSMUSG00000059475 Zfp426 protein_coding 1.082 22.349
0.974 88.345 0.946 46.956 1.063 65.401
ENSMUSG00000059479 B3gnt8 protein_coding 1.102 75.671
0.923 96.843 0.985 69.974 1.082 76.697
ENSMUSG00000059482 2610301B20Rik protein_coding 1.057 30.984
0.933 66.651 1.008 88.657 1.011 94.654
ENSMUSG00000059483 Gm5528 pseudogene 1 84.738 NA NA
1 86.602 1 96.086
ENSMUSG00000059486 Kbtbd2 protein_coding 1.076 2.655 1.018
96.68 0.911 1.58 0.958 83.724
ENSMUSG00000059488 Olfr727 protein_coding 1 80.146 1
97.155 1 88.461 1 96.071
ENSMUSG00000059493 Nhs protein_coding 0.86 11.103 0.999
92.137 1.164 17.662 1.011 96.071
ENSMUSG00000059495 Arhgef12 protein_coding 1.045 4.362 0.993
96.68 0.961 7.809 0.923 77.012
ENSMUSG00000059498 Fcgr3 protein_coding 1.441 0.999 0.842 41.268
0.826 47.641 1.175 76.347
ENSMUSG00000059503 Olfr248 protein_coding 1 63.534 1
95.708 1 82.575 1 96.287
ENSMUSG00000059504 Olfr314 protein_coding 1 80.751 1
93.654 NA NA 1 93.964
ENSMUSG00000059511 Gm20563 pseudogene 1 86.57 1 96.847
1 87.173 1 95.832
ENSMUSG00000059518 Znhit1 protein_coding 0.802 2.536 1.048
82.904 1.185 16.26 1.093 82.85
ENSMUSG00000059534 Uqcr10 protein_coding 0.762 4.941 1.126
82.904 1.165 67.163 1.099 94.904
ENSMUSG00000059540 Tcea2 protein_coding 0.99 78.224 0.998
95.088 1.007 79.426 0.988 93.584
ENSMUSG00000059547 Fbxw26 protein_coding 1 85.857 1
95.088 1 86.666 1 91.65
ENSMUSG00000059552 Trp53 protein_coding 0.916 37.248 1.092
93.899 1.001 63.801 0.993 96.192
ENSMUSG00000059554 Ccdc28a protein_coding 1.063 81.465

0.891 86.728 1.053 55.512 0.886 91.105
ENSMUSG00000059555 Tor4a protein_coding 1.087 5.016 0.959 66.09 0.96 68.82 1.033 96.184
ENSMUSG00000059562 Ccdc154 protein_coding 0.479 64.893 0.922 94.102 2.266 53.882 0.74 95.92
ENSMUSG00000059565 Gm5292 pseudogene 1 85.728 1 96.216 1 86.552 1 95.877
ENSMUSG00000059571 Gm16218 pseudogene 1 69.777 NA NA 1 80.749 1 95.35
ENSMUSG00000059585 Ube2nl pseudogene 1 86.006 1 91.661 NA NA 1 94.215
ENSMUSG00000059586 Nsmce2 protein_coding 1.092 2.569 0.979 87.857 0.936 30.827 1.022 95.273
ENSMUSG00000059588 Calcrl protein_coding 1.091 2.446 0.99 88.705 0.928 19.552 1 87.705
ENSMUSG00000059595 Olfr935 protein_coding 1 85.77 1 92.137 1 79.325 1 96.086
ENSMUSG00000059602 Syn3 protein_coding 0.836 26.77 0.979 96.138 1.222 38.099 0.879 90.611
ENSMUSG00000059606 Ear5 protein_coding 1 84.263 NA NA 1 88.693 1 92.389
ENSMUSG00000059623 Olfr39 protein_coding 1 85.728 1 97.155 1 85.757 1 96.248
ENSMUSG00000059625 Sohlh1 protein_coding 1 74.8 1 96.948 NA NA 1 95.559
ENSMUSG00000059626 Rhox3e protein_coding 1 73.734 NA NA 1 85.935 1 96.358
ENSMUSG00000059632 Krtap8-1 protein_coding 1 86.112 1 91.897 1 82.161 1 94.904
ENSMUSG00000059645 Gm7361 protein_coding 1 79.433 1 88.705 1 50.091 1 86.445
ENSMUSG00000059647 Gm10068 protein_coding 1 84.738 1 96.138 1 88.349 1 94.016
ENSMUSG00000059648 Rnase11 protein_coding 1 84.324 1 95.708 1 75.637 1 96.287
ENSMUSG00000059657 Stfa2l1 protein_coding 1 83.823 1 97.155 1 86.084 1 92.12
ENSMUSG00000059658 Gm16379 pseudogene 1.279 66.58 0.729 95.708 1.074 79.255 0.6 85.493
ENSMUSG00000059659 Gm10069 lincRNA 0.555 0.987 1.611 83.104 1.121 37.259 1.043 93.156
ENSMUSG00000059663 4930524N10Rik protein_coding 1 85.655 1 94.48 1 83.811 1 95.273
ENSMUSG00000059668 Krt4 protein_coding 2.02 13.633 0.78 71.889 0.632 56.822 0.79 92.389
ENSMUSG00000059669 Taf1b protein_coding 1.079 16.959 0.938 73.461 0.984 65.688 0.968 89.294
ENSMUSG00000059674 Cdh24 protein_coding 0.838 60.569 0.975 96.68 1.226 85.464 0.96 85.493
ENSMUSG00000059687 Olfr108 protein_coding 1 85.637 NA NA 1 87.898 1 96.183
ENSMUSG00000059689 Zfp637 protein_coding 1.033 28.927 0.984 87.553 0.985 77.294 1.02 95.35
ENSMUSG00000059690 1700020N15Rik protein_coding 1 74.958

1 93.817 1 85.21 1 92.263
ENSMUSG00000059695 1700019M22Rik protein_coding 1 75.671
1 96.407 1 84.229 1 95.273
ENSMUSG00000059708 Akap17b protein_coding 1.012 83.658
1.139 64.304 0.87 23.938 1.034 94.001
ENSMUSG00000059713 Rcan3 protein_coding 1.252 0.767 0.965 61.625
0.829 3.943 0.899 86.991
ENSMUSG00000059714 Flot1 protein_coding 0.885 3.009 1.115 71.617
1.015 48.451 1.026 77.621
ENSMUSG00000059729 Olfr1385 protein_coding 1 72.546 1
95.329 1 78.155 1 93.132
ENSMUSG00000059731 Olfr837 pseudogene 1 76.968 1
96.013 1 86.985 1 95.72
ENSMUSG00000059734 Ndufs8 protein_coding 0.829 0.987 1.056
71.98 1.139 20.886 1.021 86.133
ENSMUSG00000059741 Myl3 protein_coding 0.617 66.652 1.394
92.561 1.165 80.899 1.826 60.31
ENSMUSG00000059742 Kcnh7 protein_coding 0.702 25.111 1.193
92.062 1.196 37.369 0.53 84.29
ENSMUSG00000059743 Fdps protein_coding 0.65 0.987 1.261 73.092
1.218 20.572 1.139 65.558
ENSMUSG00000059762 Olfr767 protein_coding 1 86.57 1
94.48 1 81.388 1 87.7
ENSMUSG00000059768 Olfr682-ps1 protein_coding 1 80.608 1
96.407 1 86.112 1 95.273
ENSMUSG00000059772 Slx1b protein_coding 0.97 50.768 0.945
96.001 1.088 64.624 0.924 86.699
ENSMUSG00000059775 Rps26-ps1 protein_coding 31.758 0
0.393 78.877 0.08 0.72 0.909 95.74
ENSMUSG00000059776 Gm10071 pseudogene 1 85.391 1
96.897 1 86.602 1 96.042
ENSMUSG00000059791 Nrm protein_coding 1.137 13.633 0.852
54.427 1.03 83.925 1.155 60.968
ENSMUSG00000059796 Eif4a1 protein_coding 1.01 83.09 0.989
96.847 1.002 86.666 1.057 55.841
ENSMUSG00000059810 Rgs3 protein_coding 0.887 48.146 1.039
95.431 1.086 42.199 0.947 91.625
ENSMUSG00000059811 Atl2 protein_coding 1.049 53.562 0.988
88.691 0.965 84.434 0.936 94.608
ENSMUSG00000059820 AU019823 protein_coding 0.984 84.564
1.045 96.652 0.969 84.434 1.003 94.904
ENSMUSG00000059821 Olfr847 protein_coding 1 76.968 1
93.046 1 80.015 1 95.676
ENSMUSG00000059824 Dbp protein_coding 0.953 57.38 1.209 83.104
0.869 7.742 1.086 85.629
ENSMUSG00000059827 Gm5670 pseudogene 1 81.24 1 95.809
1 75.155 1 96.358
ENSMUSG00000059830 Gm16380 protein_coding 1 84.819 NA
NA 1 86.112 1 95.92
ENSMUSG00000059832 Kprp protein_coding 1 70.213 1
87.609 NA NA 1 88.197
ENSMUSG00000059834 Sclt1 protein_coding 1.102 58.896 0.88
79.894 1.032 76.923 1.007 95.92
ENSMUSG00000059835 Rpl13-ps3 protein_coding 1 78.744 1

97.263	1	88.657	1	94.468
ENSMUSG00000059839	Zfp874b	protein_coding	1.056	32.873
0.942	71.331	1.003	87.786	1.063	89.498
ENSMUSG00000059842	Zfp341	protein_coding	0.98	43.816
1.014	92.561	1.007	84.896	0.946	86.991
ENSMUSG00000059845	Gm11567	protein_coding	1	75.761	1
95.431	1	87.744	1	93.457
ENSMUSG00000059851	Suv420h2	protein_coding	0.916	19.224
0.996	96.897	1.097	28.979	0.894	92.181
ENSMUSG00000059852	Kcng2	protein_coding	1.066	72.85	1.267	95.196
0.737	34.21	0.769	83.724
ENSMUSG00000059854	Hydin	protein_coding	0.769	67.477	1.321
91.643	0.984	84.229	0.965	86.699
ENSMUSG00000059857	Ntng1	protein_coding	1.532	7.255	NA	NA
0.702	9.746	0.792	85.493
ENSMUSG00000059862	Olfr826	protein_coding	1	86.658	1
93.817	1	86.602	1	94.99
ENSMUSG00000059866	Tnip2	protein_coding	1.036	55.462	0.862
61.883	1.115	26.663	0.978	92.181
ENSMUSG00000059867	Olfr960	protein_coding	1	83.263	1
96.843	1	83.719	1	96.086
ENSMUSG00000059873	Olfr1029	protein_coding	1	83.823	1
90.378	1	76.923	1	95.273
ENSMUSG00000059874	Olfr603	protein_coding	1	85.283	1
96.847	1	84.308	1	95.273
ENSMUSG00000059877	Dph3b-ps	protein_coding	1	85.728	1
95.329	1	79.524	1	94.904
ENSMUSG00000059878	Zfp422	protein_coding	1.011	65.284
1.021	91.275	0.97	88.274	0.928	83.724
ENSMUSG00000059883	Irak4	protein_coding	0.972	86.172	1.028
95.504	1.003	87.103	1.053	71.305
ENSMUSG00000059887	Olfr1507	protein_coding	1	81.823	NA
NA	NA	NA	1	96.183
ENSMUSG00000059890	Ube4a	protein_coding	0.887	4.385	1.053	83.104
1.067	16.808	1.012	95.559
ENSMUSG00000059891	Tsks	protein_coding	0.411	11.128	1.087
94.367	2.237	28.997	1.294	95.273
ENSMUSG00000059895	Ptp4a3	protein_coding	0.872	27.836
1.142	71.617	1.005	87.37	1.104	83.067
ENSMUSG00000059897	Zfp930	protein_coding	1.332	0	0.858
35.895	0.879	25.345	0.893	84.29
ENSMUSG00000059898	Dsc3	protein_coding	1.332	46.013	0.651
85.418	1.153	85.352	0.695	92.181
ENSMUSG00000059900	Tmem40	protein_coding	0.929	47.677
0.792	65.819	1.357	12.703	0.953	85.493
ENSMUSG00000059901	Adamts14	protein_coding	1.081	24.147
1.083	94.48	0.852	44.211	1.225	44.679
ENSMUSG00000059910	Olfr1265	protein_coding	1	78.744	1
96.013	1	86.475	1	90.865
ENSMUSG00000059920	4930453N24Rik	protein_coding	0.964	33.599
1.065	80.775	0.976	88.203	0.987	91.928
ENSMUSG00000059921	Unc5c	protein_coding	1.491	7.65	0.897	90.712
0.749	33.971	0.859	95.273
ENSMUSG00000059923	Grb2	protein_coding	1.041	33.325	1.026

83.819	0.934	10.934	0.986	95.832
ENSMUSG00000059934	Prh1	protein_coding	1	86.172	1
96.216	1	84.896	1	94.82
ENSMUSG00000059939	9430015G10Rik	protein_coding	0.895	14.645
1.097	90.29	1.014	51.977	1.039	94.567
ENSMUSG00000059964	NA	NA	NA	NA	1	97.155	1
88.693	1	96.086
ENSMUSG00000059970	Hspa2	protein_coding	1.021	77.916	1.021
93.529	0.96	41.928	1.07	83.067
ENSMUSG00000059972	Gm14773	lincRNA	1	57.655	1
97.155	1	86.346	1	95.74
ENSMUSG00000059974	Ntm	protein_coding	1.008	86.464	1.083
83.104	0.917	53.7	0.91	91.291
ENSMUSG00000059975	Zfp74	protein_coding	0.957	53.679	1.057
79.411	0.989	85.675	1.092	91.525
ENSMUSG00000059981	Taok2	protein_coding	0.973	60.003	1.029
89.494	1	84.552	1.007	96.139
ENSMUSG00000059990	NA	NA	NA	NA	1	96.268	1
87.431	1	96.248
ENSMUSG00000059991	Nptx2	protein_coding	1.429	39.075	0.856
93.817	0.817	43.842	2.152	44.679
ENSMUSG00000059994	Fcrl1	protein_coding	1.037	86.145	0.979
92.631	0.981	87.898	0.927	95.74
ENSMUSG00000059995	Atxn7l3	protein_coding	0.939	33.745
1.061	83.104	1.006	85.464	1.043	83.276
ENSMUSG00000059996	Olfr484	protein_coding	1	84.534	1
95.088	1	87.37	1	94.654
ENSMUSG00000060002	Chpt1	protein_coding	0.83	1.274	1.01	96.268
1.196	12.831	0.894	83.724
ENSMUSG00000060012	Kif13b	protein_coding	1.087	34.439	NA
NA	0.916	23.938	0.979	85.493
ENSMUSG00000060017	Olfr121	protein_coding	1	80.251	1
95.211	1	71.615	1	96.358
ENSMUSG00000060019	Gm10073	protein_coding	1	74.516	NA
NA	NA	NA	1	95.92
ENSMUSG00000060024	Vmn1r213	protein_coding	1	85.203	1
97.263	NA	NA	1	95.273
ENSMUSG00000060029	4930473A02Rik	processed_transcript	1.035
74.482	0.948	95.504	1.017	69.825	0.849	84.19
ENSMUSG00000060030	Olfr317	protein_coding	1	86.006	1
96.013	1	85.921	1	95.593
ENSMUSG00000060032	H2afj	protein_coding	0.796	6.155	1.098	92.111
1.142	19.452	0.979	87.182
ENSMUSG00000060034	Ctf2	protein_coding	0.527	31.614	1.746
85.756	1.082	86.144	0.664	84.19
ENSMUSG00000060036	Rpl3	protein_coding	1.077	11.197	0.909
15.938	1.017	51.851	1.028	95.777
ENSMUSG00000060038	Dhps	protein_coding	0.932	3.688	0.966	95.504
1.112	14.169	1.003	86.118
ENSMUSG00000060044	Tmem26	protein_coding	1.853	16.539
0.904	95.708	0.598	19.552	0.964	95.92
ENSMUSG00000060049	Olfr235	protein_coding	1	85.946	1
93.654	1	83.066	1	94.153
ENSMUSG00000060057	Olfr193	protein_coding	1	83.169	NA

NA 1 86.258 1 96.183
ENSMUSG00000060063 Alox5ap protein_coding 1.581 0 0.875
82.503 0.723 2.563 1.218 62.594
ENSMUSG00000060068 Gm12222 pseudogene 1 83.263 1
97.263 1 84.308 1 95.35
ENSMUSG00000060070 Defa26 protein_coding 1 84.397 1
93.469 1 86.475 1 94.904
ENSMUSG00000060073 Psma3 protein_coding 1.104 51.297 0.941
82.947 0.962 80.5 1.051 55.485
ENSMUSG00000060081 Hist1h2aa protein_coding 1 81.196 1
94.597 1 88.076 1 92.389
ENSMUSG00000060084 Olfr748 protein_coding 1 71.915 1
96.847 1 88.161 1 91.65
ENSMUSG00000060087 Gm10074 pseudogene 1 85.637 NA NA
1 86.144 1 95.74
ENSMUSG00000060090 Rp2h protein_coding 1.109 3.498 0.997 87.919
0.901 20.009 0.972 85.493
ENSMUSG00000060093 Hist1h4a protein_coding 1.72 32.411
0.733 90.256 0.794 82.903 0.743 83.952
ENSMUSG00000060096 Amd-ps3 pseudogene 0.844 81.925 0.446
75.618 2.654 44.787 1.178 83.067
ENSMUSG00000060098 Prmt7 protein_coding 0.971 83.823 1.056
93.899 0.976 81.525 1.044 79.268
ENSMUSG00000060105 Olfr504 protein_coding 1 84.819 1
97.155 1 87.971 1 95.35
ENSMUSG00000060112 NA NA NA NA 1 74.483 1
74.082 1 91.011
ENSMUSG00000060114 Olfr912 protein_coding 1 85.092 1
96.216 1 84.626 1 96.086
ENSMUSG00000060121 Gemin2 protein_coding 0.999 77.916
0.962 82.671 1.041 82.436 1.052 95.72
ENSMUSG00000060126 Tpt1 protein_coding 1.038 59.806 NA NA
0.985 61.743 0.992 95.832
ENSMUSG00000060128 Gm10075 pseudogene 1 75.115 NA NA
1 85.21 1 77.331
ENSMUSG00000060131 Atp8b4 protein_coding 1.459 0 0.842
55.265 0.812 9.494 1.175 60.968
ENSMUSG00000060147 Serpinb6a protein_coding 1.3 0 0.884
65.819 0.871 4.48 1.276 31.133
ENSMUSG00000060149 BC002059 protein_coding 1.285 1.395 0.88
79.475 0.886 23.86 0.909 87.331
ENSMUSG00000060152 Pop5 protein_coding 0.988 73.292 1.04
82.809 0.969 33.402 1.01 90.674
ENSMUSG00000060161 Klk1b7-ps pseudogene 1 86.199 1
96.216 1 86.831 1 96.287
ENSMUSG00000060166 Zdhhc8 protein_coding 1.006 63.736
1.042 83.104 0.954 81.62 0.949 86.445
ENSMUSG00000060170 Olfr1371 protein_coding 1 85.499 1
97.263 1 72.378 1 96.248
ENSMUSG00000060176 Kif27 protein_coding 0.993 63.534 0.739
30.872 1.363 26.498 0.967 90.805
ENSMUSG00000060177 Klk1b22 protein_coding 1 67.477 1
90.744 1 76.923 1 95.273
ENSMUSG00000060181 Slc35e3 protein_coding 1.019 40.065

1.028 88.823 0.953 10.23 1.193 48.119
ENSMUSG00000060183 Cxcl11 polymorphic_pseudogene 0.846 35.449
1.431 82.904 0.823 88.657 0.725 86.991
ENSMUSG00000060187 Lrrc10 protein_coding 1 81.01 1
86.604 1 43.307 1 92.018
ENSMUSG00000060188 Cxcl17 protein_coding 0.789 12.436
0.723 74.483 1.747 6.202 0.815 85.493
ENSMUSG00000060198 Gm11353 pseudogene 1 82.092 1
95.708 1 58.616 1 93.964
ENSMUSG00000060201 Spink7 protein_coding 1 78.639 NA
NA 1 87.744 1 94.904
ENSMUSG00000060204 Gm5868 protein_coding 1 85.283 1
94.848 1 69.728 1 95.273
ENSMUSG00000060205 Olfr57 protein_coding 1 67.163 1
95.708 1 77.888 1 96.358
ENSMUSG00000060206 Zfp462 protein_coding 0.885 3.623 1.016
83.104 1.114 34.403 0.933 85.493
ENSMUSG00000060208 Defa17 protein_coding 1 60.853 NA
NA 1 86.258 1 94.654
ENSMUSG00000060209 Gm6570 pseudogene 1 82.732 1
95.504 1 81.29 1 96.358
ENSMUSG00000060212 Pcnxl2 protein_coding 0.667 42.361
0.737 87.919 2.023 40.052 0.783 86.991
ENSMUSG00000060214 Gm8203 pseudogene 3.408 4.628 NA NA
0.256 5.042 0.511 86.991
ENSMUSG00000060216 Arrb2 protein_coding 1.021 32.676 1.066
97.263 0.914 40.19 1.151 57.711
ENSMUSG00000060224 Pyroxd2 protein_coding 1.174 15.602
0.834 56.955 1.022 82.685 0.984 95.273
ENSMUSG00000060227 Casc4 protein_coding 1.164 23.019 0.969
96.138 0.881 28.669 0.959 91.105
ENSMUSG00000060240 Cend1 protein_coding 0.972 81.925 1.023
83.104 1.009 55.701 1.225 93.156
ENSMUSG00000060244 Alyref2 protein_coding 0.993 77.916
0.891 77.598 1.129 46.956 0.999 95.832
ENSMUSG00000060245 Vmn1r228 protein_coding 1 84.294 1
96.843 NA NA 1 95.273
ENSMUSG00000060254 Olfr980 protein_coding 1 86.819 1
94.848 NA NA 1 96.287
ENSMUSG00000060256 Tdpoz4 protein_coding 1 77.916 1
95.708 1 85.935 1 95.74
ENSMUSG00000060257 Scrt2 protein_coding 1 78.299 1
96.68 1 83.857 1 95.273
ENSMUSG00000060260 Pwwp2b protein_coding 0.984 79.433
1.151 82.76 0.885 40.19 1.053 93.156
ENSMUSG00000060261 Gtf2i protein_coding 0.999 82.352 0.993
81.731 1.006 69.196 0.968 93.117
ENSMUSG00000060268 Gm1661 protein_coding 0.767 16.472
1.049 96.763 1.242 42.199 0.805 85.493
ENSMUSG00000060275 Nrg2 protein_coding 1.031 80.688 0.722
34.058 1.34 13.259 1.028 92.851
ENSMUSG00000060279 Ap2a1 protein_coding 1.005 60.339 0.973
91.584 1.022 86.985 1.034 59.394
ENSMUSG00000060284 Sp7 protein_coding 1.311 52.429 0.671

82.503	1.134	84.308	1.032	91.372
ENSMUSG00000060288	Ppih	protein_coding	0.935	53.138	1.055
92.561	1.01	76.754	1.088	76.697
ENSMUSG00000060301	2610008E11Rik	protein_coding	1.101	17.191
1.045	92.931	0.866	17.381	1.034	92.748
ENSMUSG00000060303	Olfr1447	protein_coding	1	76.686	1
93.27	1	86.258	1	95.273
ENSMUSG00000060311	Mucl1	protein_coding	1.962	11.316	0.664
82.073	0.767	50.184	0.674	87.651
ENSMUSG00000060314	Zfp941	protein_coding	0.843	38.286
0.827	89.509	1.435	41.235	0.935	93.734
ENSMUSG00000060317	Acnat2	protein_coding	0.153	6.155	1.636
79.261	4.002	30.213	2.569	60.466
ENSMUSG00000060332	Tmc2	protein_coding	1	73.292	1
97.129	1	85.792	1	94.752
ENSMUSG00000060335	Olfr384	protein_coding	1	84.324	1
97.129	1	84.308	1	94.82
ENSMUSG00000060336	Zfp937	protein_coding	1.027	55.124	1.1
96.68	0.888	58.271	0.964	93.264
ENSMUSG00000060343	Calm5	pseudogene	1	81.178	1	95.504
NA	NA	1	88.755
ENSMUSG00000060371	Caln1	protein_coding	1	82.982	NA	NA
1	58.775	1	96.358
ENSMUSG00000060373	Hnrnpc	protein_coding	0.911	25.111
0.931	73.166	1.176	6.693	1.008	92.263
ENSMUSG00000060375	3110018I06Rik	protein_coding	1	72	1
97.263	1	88.729	1	95.593
ENSMUSG00000060376	Bckdha	protein_coding	0.935	41.759
1.037	96.843	1.033	62.995	0.879	83.724
ENSMUSG00000060380	C030014I23Rik	lincRNA	0.969	48.146
1.079	90.895	0.958	86.144	0.883	86.991
ENSMUSG00000060393	Gm5580	pseudogene	1	86.474	NA	NA
1	85.21	1	95.676
ENSMUSG00000060397	Zfp128	protein_coding	1.098	50.377
0.985	93.469	0.926	63.075	1.042	87.088
ENSMUSG00000060402	Chst8	protein_coding	0.836	83.726	1.114
86.277	1.071	83.811	1.321	81.56
ENSMUSG00000060404	Olfr1369-ps1	protein_coding	1	86.819
1	95.211	1	85.21	1	95.74
ENSMUSG00000060407	Cyp2a12	protein_coding	1	79.93	1
95.504	1	71.839	1	94.297
ENSMUSG00000060411	Npn2	protein_coding	1	63.736	1
95.504	1	56.125	1	89.108
ENSMUSG00000060412	Tas2r124	protein_coding	1	83.09	1
96.68	1	88.076	1	94.654
ENSMUSG00000060416	Gm839	protein_coding	1	83.726	NA	NA
1	82.903	1	92.31
ENSMUSG00000060417	Gm8603	protein_coding	1	86.74	1
93.654	1	75.009	1	93.316
ENSMUSG00000060419	Rps16-ps2	pseudogene	4.667	0	1.208	88.551
0.177	0.72	1.102	86.118
ENSMUSG00000060420	Vmn1r62	pseudogene	1	73.401	1
96.68	1	80.899	1	94.016
ENSMUSG00000060422	Olfr1034	protein_coding	0.976	35.646

					0.402	56.781	2.538	8.466	1.607	46.412
ENSMUSG00000060424	2610017I09Rik	antisense	1	85.092	NA	NA	1	76.388	1	95.273
ENSMUSG00000060427	Zfp868	protein_coding	0.986	81.925	0.935	71.889	1.086	38.26	1.115	44.882
ENSMUSG00000060429	Sntb1	protein_coding	0.945	24.344	1.178	75.62	0.899	54.25	0.954	86.699
ENSMUSG00000060438	Rps10-ps1	protein_coding	0.95	84.534	0.748	90.053	1.404	48.304	1.398	83.067
ENSMUSG00000060441	Trim5	protein_coding	1.222	7.842	1.126	86.34	0.728	20.751	0.935	92.12
ENSMUSG00000060445	Sycp2	protein_coding	0.993	63.283	1.199	84.832	0.837	81.169	0.937	92.181
ENSMUSG00000060450	Rnf14	protein_coding	0.923	15.227	1.028	87.609	1.056	64.024	1.078	52.324
ENSMUSG00000060459	Kng2	protein_coding	0.488	35.158	2.147	81.54	0.95	84.896	1.36	82.85
ENSMUSG00000060461	Dppa5a	protein_coding	1	56.311	1	96.847	1	47.641	1	96.086
ENSMUSG00000060467	Gm10080	pseudogene	0.784	5.311	1.676	60.309	0.758	40.338	1.119	74.193
ENSMUSG00000060469	Krtap19-3	protein_coding	1	83.333	1	95.301	1	79.897	1	95.72
ENSMUSG00000060470	Gpr97	protein_coding	0.648	3.07	1.128	83.104	1.367	15.602	0.865	83.724
ENSMUSG00000060475	Wtap	protein_coding	1.027	50.377	1.011	89.494	0.959	27.194	1.012	83.276
ENSMUSG00000060477	Irak2	protein_coding	1.106	51.189	0.867	70.693	1.044	77.888	0.902	88.955
ENSMUSG00000060480	Olfr171	protein_coding	1	80.815	1	94.96	1	83.719	1	94.384
ENSMUSG00000060487	Samd5	protein_coding	1.446	13.734	1.096	90.378	0.628	16.949	1.082	91.872
ENSMUSG00000060490	Vmn1r218	protein_coding	1	83.263	1	94.48	1	84.071	1	93.261
ENSMUSG00000060499	Rpl10l	protein_coding	1	82.543	NA	NA	1	82.575	1	95.35
ENSMUSG00000060503	Olfr715	protein_coding	1	86.464	NA	NA	1	72.853	1	96.183
ENSMUSG00000060508	Nlrp9b	protein_coding	0.697	79.433	1.944	96.138	0.739	83.719	0.765	94.654
ENSMUSG00000060509	Xcr1	protein_coding	1.018	81.823	1.309	71.617	0.747	28.237	0.766	86.699
ENSMUSG00000060510	Zfp266	protein_coding	0.987	65.622	0.978	70.693	1.037	32.173	0.988	94.608
ENSMUSG00000060512	0610040J01Rik	protein_coding	0.742	44.252	0.965	90.378	1.398	34.07	1.181	90.805
ENSMUSG00000060519	Tor3a	protein_coding	1.118	0.355	1.04	93.046	0.856	14.206	1.088	60.466
ENSMUSG00000060523	Olfr726	protein_coding	1	80.51	1	95.708	1	86.666	1	92.12
ENSMUSG00000060530	A930017M01Rik	pseudogene	1.004	85.857	NA	NA	0.952	87.103	0.947	90.865
ENSMUSG00000060534	Dcc	protein_coding	1	80.408	NA	NA				

1 81.525 1 96.071
ENSMUSG00000060538 Tmem219 protein_coding 0.941 42.361
0.986 94.96 1.079 41.6 1.145 44.882
ENSMUSG00000060548 Tnfrsf19 protein_coding 1.869 6.438 0.873
91.532 0.613 12.288 0.65 86.699
ENSMUSG00000060550 H2-Q7 protein_coding 0.846 36.541 1.183
79.579 0.996 84.961 0.909 84.29
ENSMUSG00000060556 Olfr1418 protein_coding 1 81.52 1
96.268 1 81.29 1 96.071
ENSMUSG00000060559 Gm10083 protein_coding 0.78 23.319
1.756 79.579 0.731 70.214 0.757 83.724
ENSMUSG00000060560 Ces4a protein_coding 0.464 38.286 1.173
96.138 1.835 47.512 0.926 92.748
ENSMUSG00000060565 Gm5591 protein_coding 1 84.397 1
96.216 1 86.831 1 94.99
ENSMUSG00000060566 Rpl21-ps3 pseudogene 1 79.433 1
95.301 1 83.607 1 96.287
ENSMUSG00000060568 Fam78b protein_coding 1.032 73.487
1.118 83.104 0.867 59.726 0.936 96.358
ENSMUSG00000060572 Mfap2 protein_coding 1.128 15.178 0.995
93.817 0.888 14.583 1.171 44.882
ENSMUSG00000060579 Fhit protein_coding 1.085 32.058 0.95
85.116 0.972 84.669 0.944 86.991
ENSMUSG00000060583 Olfr881 protein_coding 1 85.59 1
94.848 1 88.693 1 96.358
ENSMUSG00000060586 NA NA NA NA 1.205 85.756 0.806
77.704 0.776 83.724
ENSMUSG00000060591 Ifitm2 protein_coding 1.091 59.806
1.019 95.431 0.9 7.329 1.02 59.208
ENSMUSG00000060594 Layn protein_coding 1.296 1.815 1.025 81.134
0.752 11.116 1.028 91.492
ENSMUSG00000060600 Eno3 protein_coding 2.754 8.847 1.069 90.116
0.341 0.879 1.039 94.904
ENSMUSG00000060601 Nr1h2 protein_coding 0.927 25.492 1.017
96.68 1.064 45.035 0.97 86.991
ENSMUSG00000060613 Cyp2c70 protein_coding 1 82.092 1
95.504 1 83.275 1 95.72
ENSMUSG00000060615 Ang4 protein_coding 1 84.661 1
95.329 1 76.498 1 95.92
ENSMUSG00000060621 Nkpd1 protein_coding 0.805 26.013 0.902
95.211 1.372 21.201 0.683 94.468
ENSMUSG00000060630 Zfp735 protein_coding 1 81.823 1
97.155 1 84.071 1 93.316
ENSMUSG00000060636 Rpl35a protein_coding 1.147 3.825 0.843
35.072 1.036 84.669 0.949 89.525
ENSMUSG00000060639 Hist1h4i protein_coding 0.912 86.474
1.218 96.407 0.897 80.015 0.933 86.991
ENSMUSG00000060647 Gm7099 pseudogene 1 80.251 1
90.092 1 87.336 1 94.468
ENSMUSG00000060657 Marf1 protein_coding 0.99 59.527 0.998
89.509 1.012 87.658 0.942 83.724
ENSMUSG00000060663 Olfr175-ps1 protein_coding 1 84.452 1
96.013 1 82.74 1 96.248
ENSMUSG00000060671 Atp8b2 protein_coding 1.102 1.323 0.993

97.097	0.915	1.898	1.009	87.134
ENSMUSG00000060673	NA	NA	NA	NA	1	95.431	NA	NA
1	96.183
ENSMUSG00000060675	Pla2g16	protein_coding	1.074	85.728
1.003	97.155	0.928	79.524	1.023	83.581
ENSMUSG00000060679	Mrps9	protein_coding	1.04	35.787	0.934
60.678	1.029	62.237	1	90.95
ENSMUSG00000060680	Gm8894	pseudogene	1	61.043	NA	NA
1	62.648	1	93.132
ENSMUSG00000060681	Slc9a6	protein_coding	0.99	73.734
1.011	97.263	0.999	58.516	1.033	93.117
ENSMUSG00000060685	Gm14511	protein_coding	1	70.213	1
95.504	1	84.896	1	96.139
ENSMUSG00000060688	Olfr292	protein_coding	1	72.139	1
96.308	1	81.388	1	94.297
ENSMUSG00000060691	Krtap19-1	protein_coding	1	84.452	1
96.847	1	83.158	1	93.132
ENSMUSG00000060701	Fpr-rs3	protein_coding	1	85.637	1
95.708	1	88.693	1	93.722
ENSMUSG00000060703	Cd302	protein_coding	1.238	10.19	0.897	90.053
0.899	30.468	1.035	75.198
ENSMUSG00000060708	Bloc1s4	protein_coding	0.984	78.744
1.066	87.029	0.954	54.521	0.875	83.724
ENSMUSG00000060716	Plekhh1	protein_coding	1.025	85.499
0.869	67.165	1.12	29.819	1.013	90.865
ENSMUSG00000060724	Vmn1r47	protein_coding	1	83.169	1
93.765	1	81.29	1	95.273
ENSMUSG00000060726	Tmsb15a	protein_coding	1	70.213	1
95.196	1	82.903	1	86.991
ENSMUSG00000060727	Gm12571	pseudogene	0.47	72.985	0.542
68.115	3.93	43.185	1.956	90.865
ENSMUSG00000060730	Gm10086	pseudogene	1	77.497	1
97.263	1	87.103	1	94.19
ENSMUSG00000060733	Ipmk	protein_coding	1.059	20.296	0.979
88.691	0.96	46.071	0.971	86.991
ENSMUSG00000060735	Rxfp3	protein_coding	1.345	40.333	0.685
69.611	1.08	85.21	1.099	95.273
ENSMUSG00000060738	Prl7c1	protein_coding	1	79.853	1
96.216	1	81.492	1	96.248
ENSMUSG00000060739	Nsa2	protein_coding	1.088	3.498	0.902	12.804
1.022	64.305	1.047	86.445
ENSMUSG00000060742	Olfr1076	protein_coding	1	80.251	1
95.088	1	72.464	1	95.405
ENSMUSG00000060743	H3f3a	protein_coding	1.099	0.355	0.963	79.894
0.946	23.938	0.988	90.805
ENSMUSG00000060747	Ifnl3	protein_coding	1	84.977	1
97.049	1	86.144	1	96.086
ENSMUSG00000060756	Krtap2-4	protein_coding	1	80.51	1
95.211	1	80.5	1	96.287
ENSMUSG00000060759	Olfr503	protein_coding	1	81.465	1
96.138	1	75.527	1	96.287
ENSMUSG00000060765	Olfr325	protein_coding	1	84.397	1
94.367	1	84.961	1	95.72
ENSMUSG00000060771	Tsga10	protein_coding	1.088	29.596	1.02

93.707 0.9 36.786 0.982 92.31
ENSMUSG00000060777 Gm10087 pseudogene 1 72.546 NA NA
1 86.144 1 93.261
ENSMUSG00000060780 Lrrtm1 protein_coding 1.132 64.714
0.999 93.899 0.882 25.512 0.748 83.724
ENSMUSG00000060787 Olfr464 protein_coding 1 83.658 NA
NA 1 84.669 1 95.593
ENSMUSG00000060791 Gmfg protein_coding 1.236 16.74 0.82 26.151
0.988 85.757 1.05 94.016
ENSMUSG00000060794 Tssk5 protein_coding 1 84.452 NA NA
1 52.213 1 94.215
ENSMUSG00000060795 Gm13363 pseudogene 1 86.172 1
94.848 1 84.229 1 94.297
ENSMUSG00000060798 Intu protein_coding 1.134 19.132 0.938
82.848 0.94 76.768 0.898 84.29
ENSMUSG00000060802 B2m protein_coding 1.035 64.893 0.993
95.735 0.974 78.954 0.915 83.724
ENSMUSG00000060803 Gstp1 protein_coding 1.036 68.16 1.035 83.104
0.935 21.852 1 96.248
ENSMUSG00000060808 3110043A19Rik antisense 0.653 7.207 1.048
96.216 1.466 18.565 0.872 96.358
ENSMUSG00000060811 Gm10088 protein_coding 1 74.516 1
95.941 1 82.685 1 96.358
ENSMUSG00000060816 Vmn1r52 protein_coding 1 79.93 1
96.407 NA NA 1 93.685
ENSMUSG00000060827 Olfr1193 protein_coding 1 81.657 1
96.948 1 86.727 1 94.654
ENSMUSG00000060843 Ctnna3 protein_coding 1.023 85.946
1.147 92.931 0.853 57.819 1.329 65.401
ENSMUSG00000060860 Ube2s protein_coding 0.901 13.239 1.035
93.27 1.069 42.75 1.032 74.896
ENSMUSG00000060862 Zbtb40 protein_coding 0.956 76.135
1.128 97.288 0.923 86.084 1.073 83.067
ENSMUSG00000060863 Tcl1b2 protein_coding 1 75.115 1
96.847 1 86.666 1 95.35
ENSMUSG00000060879 Gm6564 pseudogene 1 79.93 NA NA 1
87.173 1 96.358
ENSMUSG00000060882 Kcnd2 protein_coding 1.025 73.401 NA NA
0.708 79.776 0.484 83.724
ENSMUSG00000060887 Gm12960 pseudogene 1 84.977 1
93.654 1 87.971 1 96.139
ENSMUSG00000060888 Olfr566 protein_coding 1 79.068 NA
NA 1 88.161 1 95.593
ENSMUSG00000060899 Rpl21-ps11 pseudogene 1 76.968 NA NA
1 87.336 1 95.273
ENSMUSG00000060904 Arl1 protein_coding 1.036 73.734 0.998
88.533 0.966 45.573 1.119 32.045
ENSMUSG00000060913 Trim55 protein_coding 8.324 14.008
0.939 89.494 0.128 2.189 0.606 94.904
ENSMUSG00000060918 Olfr51 protein_coding 1 73.734 1
96.268 NA NA 1 95.676
ENSMUSG00000060923 Acyp2 protein_coding 0.835 1.175 0.997 91.584
1.195 14.131 1.026 95.273
ENSMUSG00000060924 Csmd1 protein_coding 1.115 47.174 1.053

92.561 0.852 41.928 0.879 83.724
ENSMUSG00000060935 AI597468 protein_coding 1.168 2.512 1.071
95.431 0.798 1.342 0.947 94.608
ENSMUSG00000060938 Rpl26 protein_coding 1.077 34.439 0.874
21.426 1.064 29.568 1.079 56.863
ENSMUSG00000060950 Trmt61a protein_coding 0.847 61.633
1.046 97.155 1.129 68.425 1.03 83.067
ENSMUSG00000060956 Gm10092 protein_coding 1 77.916 1
96.216 1 87.898 1 91.783
ENSMUSG00000060961 Slc4a4 protein_coding 1.076 39.075
0.956 87.998 0.973 65.902 0.996 92.31
ENSMUSG00000060962 Dmkn protein_coding 1.3 58.38 0.862 75.345
0.89 76.754 1.133 92.263
ENSMUSG00000060967 Etd protein_coding 1 82.231 1
94.848 1 69.583 1 94.654
ENSMUSG00000060969 Irx1 protein_coding 0.902 29.972 0.954
94.887 1.164 38.26 0.885 83.724
ENSMUSG00000060974 Olfr530 protein_coding 1 86.351 1
95.708 1 84.669 1 96.358
ENSMUSG00000060981 Hist1h4h protein_coding 0.82 18.379 1.17
82.904 1.042 68.129 0.925 85.493
ENSMUSG00000060985 Tdrd5 protein_coding 1.74 59.211 0.462
44.268 1.24 40.955 0.713 90.157
ENSMUSG00000060988 Galnt13 protein_coding 2.809 21.539
0.787 96.897 0.452 21.666 1.025 87.7
ENSMUSG00000060989 Gm11847 pseudogene 0.324 16.02 1.369 96.68
2.254 42.199 1.101 94.825
ENSMUSG00000060992 Copz1 protein_coding 1.015 69.408 1.026
66.695 0.961 9.746 1.01 83.067
ENSMUSG00000061000 Olfr507 protein_coding 1 86.474 1
96.68 1 84.308 1 96.183
ENSMUSG00000061013 Mkx protein_coding 1.433 13.363 1.143
96.216 0.609 19.236 0.713 83.724
ENSMUSG00000061022 Vmn1r222 protein_coding 1 86.333 1
96.001 1 82.74 1 96.042
ENSMUSG00000061024 Rrs1 protein_coding 0.904 32.557 1.098
75.21 1.009 83.275 1.044 95.273
ENSMUSG00000061028 Clasrp protein_coding 0.833 3.07 0.997
95.735 1.207 9.301 0.976 93.316
ENSMUSG00000061032 Rrp1 protein_coding 0.948 3.688 0.972 84.594
1.083 5.864 1.044 70.36
ENSMUSG00000061039 Olfr920 protein_coding 1.15 25.207
0.896 87.857 0.967 73.096 0.914 85.493
ENSMUSG00000061046 Haghl protein_coding 1.01 81.196 0.944
67.739 1.047 43.307 1.023 83.067
ENSMUSG00000061048 Cdh3 protein_coding 0.883 11.384 1.162
71.617 NA NA 0.928 83.724
ENSMUSG00000061062 Gm10093 protein_coding 0.802 60.339 NA
NA 1.014 79.325 1.166 92.748
ENSMUSG00000061065 Gm5382 protein_coding 1 86.843 1
94.48 1 86.144 1 94.752
ENSMUSG00000061068 Mcpt4 protein_coding 1.903 0 0.921 81.02
0.571 1.764 0.947 91.625
ENSMUSG00000061075 NA NA NA NA 1 96.68 1 85.935

1 96.086
ENSMUSG00000061079 Zfp143 protein_coding 1.038 26.338
0.879 49.266 1.095 29.819 1.019 89.428
ENSMUSG00000061080 Lsamp protein_coding 1.321 24.958 0.935
90.053 0.81 55.214 0.996 93.722
ENSMUSG00000061086 Myl4 protein_coding 0.796 3.009 1.087 81.127
1.159 10.819 1.005 91.783
ENSMUSG00000061099 Gapdhs protein_coding 0.736 15.227 NA
NA 1.35 22.93 0.992 90.611
ENSMUSG00000061100 Retnla protein_coding 1.433 5.848 0.655
25.341 1.068 75.527 0.913 89.587
ENSMUSG00000061111 Fam195b protein_coding 1.013 85.203 NA
NA 1.015 88.349 1.025 83.276
ENSMUSG00000061115 Gm17113 pseudogene 1 82.092 1
95.708 1 87.37 1 95.72
ENSMUSG00000061118 Dnajc30 protein_coding 0.922 8.405 1.113
74.663 0.974 79.897 0.951 85.493
ENSMUSG00000061119 Prcp protein_coding 1.247 0 0.926 81.02
0.863 13.643 0.954 93.612
ENSMUSG00000061126 Cyp4f39 protein_coding 1.997 8.685 0.955
96.68 0.523 1.871 0.865 94.608
ENSMUSG00000061130 Ppm1b protein_coding 1.021 52.429 0.975
96.897 1.007 53.224 0.98 84.19
ENSMUSG00000061132 Blnk protein_coding 1.657 0 0.793 37.376
0.762 11.64 0.951 93.457
ENSMUSG00000061136 Prpf40a protein_coding 1.107 35.499
0.948 71.41 0.952 87.103 1.007 91.872
ENSMUSG00000061143 Maml3 protein_coding 1.268 15.178 0.974
85.116 0.811 22.864 0.853 83.724
ENSMUSG00000061144 Spink12 protein_coding 1 75.115 1
96.216 1 80.089 1 92.675
ENSMUSG00000061150 Vmn1r229 protein_coding 1 82.092 1
93.574 1 73.598 1 95.877
ENSMUSG00000061165 Olfr160 protein_coding 1 74.689 1
93.654 1 86.144 1 94.297
ENSMUSG00000061171 Slc38a11 protein_coding 1.274 45.307
0.996 95.708 0.788 54.037 0.82 92.675
ENSMUSG00000061175 Fnip2 protein_coding 1.252 0 0.947 87.857
0.844 0.72 0.966 83.724
ENSMUSG00000061184 Tmprss11c protein_coding 1 81.465 1
96.216 1 84.552 1 94.001
ENSMUSG00000061186 Sfmbt2 protein_coding 1.253 32.058
0.936 94.102 0.853 38.534 1.07 94.904
ENSMUSG00000061195 Olfr1289 protein_coding 1 85.728 1
96.897 1 87.686 1 95.877
ENSMUSG00000061200 Olfr601 protein_coding 1 78.224 1
93.908 1 81.857 1 82.85
ENSMUSG00000061207 Stk19 protein_coding 0.919 2.524 1.052 79.261
1.038 34.727 0.985 96.071
ENSMUSG00000061208 Vmn1r8 protein_coding 1 80.751 1
92.137 1 71.839 1 94.654
ENSMUSG00000061210 Olfr47 protein_coding 1 83.169 1
95.211 1 84.896 1 92.181
ENSMUSG00000061222 Vmn1r189 protein_coding 1 78.538 NA

NA 1 84.83 1 95.273
ENSMUSG00000061232 H2-K1 protein_coding 0.928 34.305 1.012
91.065 1.065 73.309 0.929 83.724
ENSMUSG00000061242 Gm9104 pseudogene 0.509 26.217 1.102
90.952 1.778 68.129 1.537 67.94
ENSMUSG00000061244 Exoc5 protein_coding 1.14 1.161 0.983 92.474
0.892 5.042 1.009 94.904
ENSMUSG00000061246 Olfr387-ps1 pseudogene 1 84.534 1
96.984 1 86.475 1 95.74
ENSMUSG00000061272 Gm14173 pseudogene 1 83.823 1
97.288 1 78.293 1 94.384
ENSMUSG00000061273 Mmgt1 protein_coding 0.938 21.897 1.09
79.411 NA NA 0.979 92.691
ENSMUSG00000061283 Tgif2lx1 protein_coding 1 71.763 1
96.68 1 85.58 1 94.215
ENSMUSG00000061286 Exosc5 protein_coding 0.89 7.58 1.069
82.904 1.049 35.894 1.013 95.273
ENSMUSG00000061288 Taok3 protein_coding 1.295 1.395 0.947 72.399
0.812 7.155 0.983 92.762
ENSMUSG00000061292 Cyp3a59 protein_coding 1 77.763 1
90.378 1 84.83 1 92.018
ENSMUSG00000061295 Olfr1261 protein_coding 1 86.74 1
96.216 1 86.552 1 96.248
ENSMUSG00000061296 Vmn1r210 protein_coding 1 80.408 1
95.504 1 81.62 1 92.675
ENSMUSG00000061305 NA NA NA NA NA NA 1 86.602
1 96.086
ENSMUSG00000061306 Slc38a10 protein_coding 0.853 0.987 1.055
83.104 1.114 15.185 1.005 83.067
ENSMUSG00000061313 Ddhd2 protein_coding 0.888 15.76 0.971 95.504
1.16 16.205 0.946 83.724
ENSMUSG00000061315 Naca protein_coding 1.094 1.828 0.937 40.463
0.977 67.029 0.987 91.928
ENSMUSG00000061322 Dnaic1 protein_coding 0.782 19.224
0.817 42.923 1.562 6.693 0.617 85.493
ENSMUSG00000061330 Gm11361 pseudogene 5.048 10.464 0.263
87.857 0.751 34.592 2.203 57.1
ENSMUSG00000061331 Gm17132 pseudogene 1 82.982 1
96.407 1 73.874 1 94.904
ENSMUSG00000061336 Olfr116 protein_coding 1 77.615 1
95.708 1 86.985 1 95.273
ENSMUSG00000061342 Olfr1168 protein_coding 1 72.85 1
95.708 1 85.464 1 93.457
ENSMUSG00000061344 Olfr320 protein_coding 1 84.397 1
93.654 1 85.935 1 91.525
ENSMUSG00000061353 Cxcl12 protein_coding 1.208 0.461 0.966
83.876 0.854 14.409 0.945 90.289
ENSMUSG00000061356 Nuggc protein_coding 0.9 49.749 0.708
94.102 1.561 47.776 0.677 86.699
ENSMUSG00000061360 Phf5a protein_coding 1.031 59.211 1.03
95.735 0.944 49.733 1.019 73.823
ENSMUSG00000061361 Olfr168 protein_coding 1 86.474 1
92.931 1 85.464 1 96.192
ENSMUSG00000061367 Olfr771 protein_coding 1 85.129 1

95.504 1 65.567 NA NA
ENSMUSG00000061371 Zfp873 protein_coding 1.032 64.193 NA
NA 0.984 76.388 0.971 96.358
ENSMUSG00000061374 Fiz1 protein_coding 0.881 17.031 0.998
95.708 1.134 40.575 1.013 91.928
ENSMUSG00000061376 Vmn1r219 protein_coding 1 84.564 1
95.211 1 86.666 1 93.964
ENSMUSG00000061387 Olfr1490 protein_coding 1 86.918 1
95.708 1 79.625 1 92.52
ENSMUSG00000061390 NA NA NA NA 1 97.155 1
83.857 1 95.273
ENSMUSG00000061392 Mageb5 protein_coding 1 86.333 1
96.847 1 80.015 1 93.316
ENSMUSG00000061393 Acvr2b protein_coding 0.969 54.604
0.995 96.843 1.039 67.163 0.846 83.724
ENSMUSG00000061397 Krt79 protein_coding 1.109 35.748 0.978
95.329 0.919 33.137 0.992 91.872
ENSMUSG00000061410 Zcchc14 protein_coding 1.02 62.007
0.984 96.216 0.999 74.179 0.987 86.991
ENSMUSG00000061411 8430427H17Rik protein_coding 1.065 43.344
1.066 83.104 0.883 18.797 0.924 84.29
ENSMUSG00000061414 Efcab4b protein_coding 0.799 83.169
1.469 83.104 0.85 44.019 1.013 86.445
ENSMUSG00000061436 Hipk2 protein_coding 0.905 19.365 1.077
83.104 1.021 71.839 0.955 83.724
ENSMUSG00000061451 Tmem151a protein_coding 0.936 83.726
0.829 93.654 1.291 82.436 1.11 96.287
ENSMUSG00000061455 Stx17 protein_coding 1.07 15.662 0.93
65.819 1.001 66.201 0.935 84.29
ENSMUSG00000061457 Olfr871 protein_coding 1 81.178 NA
NA 1 84.83 1 94.904
ENSMUSG00000061458 Nol10 protein_coding 0.915 64.589 0.996
93.094 1.097 56.931 1.093 75.198
ENSMUSG00000061461 Smim20 protein_coding 0.966 59.358
0.985 90.053 1.048 30.213 1.015 93.457
ENSMUSG00000061462 Obscn protein_coding 4.665 11.459 0.967
90.092 0.222 1.705 0.794 95.72
ENSMUSG00000061469 Gm12569 antisense 1 86.145 1
95.196 1 85.675 1 88.512
ENSMUSG00000061474 Mrps36 protein_coding 0.876 20.369
1.138 72.546 1.003 62.648 1.054 89.152
ENSMUSG00000061477 Rps7 protein_coding 1.095 10.464 0.902
13.578 1.009 73.309 1.003 92.389
ENSMUSG00000061479 Snrpa protein_coding 0.912 17.401 1.029
88.823 1.066 43.481 1.067 78.546
ENSMUSG00000061482 Hist1h4d protein_coding 0.852 68.846
1.166 96.378 1.003 83.538 0.923 95.877
ENSMUSG00000061486 Gm5161 pseudogene 1 60.569 1
90.116 1 85.935 1 92.903
ENSMUSG00000061488 Rpl27a-ps1 pseudogene 1 85.728 1
95.708 1 80.899 1 83.581
ENSMUSG00000061489 Olfr525 protein_coding 1 83.523 1
96.335 1 77.532 1 93.734
ENSMUSG00000061490 Ube2d4 protein_coding 1 73.841 1

93.707 1 88.601 1 95.877
ENSMUSG00000061501 Olfr197 pseudogene 1 71.516 1
97.263 1 86.831 1 90.463
ENSMUSG00000061510 Gm10101 lincRNA 1.182 53.562 0.857
94.848 0.987 88.076 1.084 94.904
ENSMUSG00000061518 Cox5b protein_coding 0.828 1.175 1.071 79.29
1.128 33.287 0.997 95.273
ENSMUSG00000061520 Olfr153 protein_coding 1 85.946 1
90.053 1 82.436 1 96.183
ENSMUSG00000061525 4921509C19Rik protein_coding 1 81.647
1 94.96 1 88.274 1 87.7
ENSMUSG00000061527 Krt5 protein_coding 0.977 72.139 1.042
88.551 0.985 88.601 0.87 83.724
ENSMUSG00000061531 Tmem236 protein_coding 0.663 48.354
1.344 91.643 1.118 69.196 1.334 91.928
ENSMUSG00000061533 Cep128 protein_coding 1.179 12.855
0.917 74.633 0.926 54.964 1.092 90.397
ENSMUSG00000061535 C1qtnf7 protein_coding 1.041 82.092
0.877 74.483 1.09 43.129 1.003 95.676
ENSMUSG00000061536 Sec22c protein_coding 0.875 3.47 1.047
92.561 1.089 30.948 0.989 94.016
ENSMUSG00000061540 Orm2 protein_coding 0.492 3.07 0.954 96.378
2.129 12.703 0.828 85.493
ENSMUSG00000061544 Zfp229 protein_coding 1.039 46.533
0.954 92.914 1.008 72.269 0.996 91.985
ENSMUSG00000061549 Olfr301 protein_coding 1 82.982 1
96.216 1 88.729 1 96.248
ENSMUSG00000061559 Wdr61 protein_coding 1.045 74.745 0.989
82.848 0.968 87.274 0.968 94.654
ENSMUSG00000061561 Olfr373 protein_coding 1 74.516 1
95.35 1 87.37 1 96.086
ENSMUSG00000061576 Dpp6 protein_coding 3.621 13.492 0.84
95.329 0.328 26.057 1.072 95.35
ENSMUSG00000061577 Gpr114 protein_coding 1.123 74.516
1.041 92.561 0.853 47.12 0.954 89.892
ENSMUSG00000061578 Ksr2 protein_coding 1.873 0.976 0.583 33.183
0.918 80.015 0.888 88.505
ENSMUSG00000061584 Lyg2 protein_coding 1 86.658 1
94.96 1 81.62 1 89.525
ENSMUSG00000061589 Dot1l protein_coding 0.842 3.325 1.17 60.309
1.016 70.534 0.964 85.493
ENSMUSG00000061601 Pclo protein_coding 0.753 3.853 1.355 70.816
0.975 68.425 0.843 96.183
ENSMUSG00000061602 Vmn1r78 protein_coding 1 82.732 1
96.984 1 83.066 1 96.287
ENSMUSG00000061603 Akap6 protein_coding 1.193 29.057 1.068
94.367 0.783 10.726 0.819 96.183
ENSMUSG00000061607 Mdc1 protein_coding 0.928 26.217 1.051
83.104 NA NA 1.084 59.464
ENSMUSG00000061613 U2af1 protein_coding 0.99 74.689 0.947
75.618 1.068 40.052 1.078 55.485
ENSMUSG00000061614 Olfr845 protein_coding 1 78.909 1
97.155 1 85.352 1 93.722
ENSMUSG00000061616 Olfr12 protein_coding 1 81.925 1

95.211 1 84.308 1 95.273
ENSMUSG00000061619 Vma21-ps protein_coding 1 84.452 1
97.263 1 79.897 NA NA
ENSMUSG00000061626 Olfr68 protein_coding 1 86.819 1
96.216 1 85.921 1 96.358
ENSMUSG00000061633 1700029P11Rik protein_coding 1 71.142
1 96.984 1 86.144 1 96.042
ENSMUSG00000061637 Olfr1457 protein_coding 1 85.728 1
90.712 1 87.173 1 95.593
ENSMUSG00000061650 Med9 protein_coding 0.958 46.289 0.95
86.861 1.101 34.07 0.994 96.184
ENSMUSG00000061653 Vmn1r46 protein_coding 1 86.145 1
94.767 1 82.903 1 94.904
ENSMUSG00000061665 Cd2ap protein_coding 1.122 2.047 0.948 59.946
0.941 27.194 0.996 92.381
ENSMUSG00000061666 Gdpd1 protein_coding 1.19 29.811 0.84
56.781 1.001 76.498 1.035 94.153
ENSMUSG00000061669 Gm6404 pseudogene 1 84.865 1
97.129 1 86.144 1 95.92
ENSMUSG00000061684 Rpl21-ps8 pseudogene 1 55.939 1
95.088 1 82.436 1 94.904
ENSMUSG00000061689 Dlgap4 protein_coding 0.911 40.422
1.077 75.21 1.015 76.64 1.051 76.697
ENSMUSG00000061701 Fbxw20 protein_coding 1 78.639 1
96.268 1 81.032 1 96.358
ENSMUSG00000061702 Tmem91 protein_coding 0.598 17.664
1.133 92.931 1.471 30.561 0.946 84.19
ENSMUSG00000061704 Olfr1036 protein_coding 1 84.534 1
97.263 1 78.713 1 95.92
ENSMUSG00000061707 Gm4871 protein_coding 1 71.516 1
96.843 1 88.076 1 93.964
ENSMUSG00000061718 Ppp1r1b protein_coding 1.234 65.733
0.689 63.912 1.17 69.728 0.964 92.12
ENSMUSG00000061723 Tnnt3 protein_coding 7.511 7.174 1.083 84.126
0.123 0.72 0.959 96.086
ENSMUSG00000061728 Btnl7-ps pseudogene 1.084 62.574 1.691
93.863 0.543 35.559 0.613 86.699
ENSMUSG00000061731 Ext1 protein_coding 1.121 10.19 0.998 91.532
0.895 20.712 1.043 49.514
ENSMUSG00000061740 Cyp2d22 protein_coding 0.955 60.707
0.877 64.123 1.195 13.082 1.056 96.287
ENSMUSG00000061751 Kalrn protein_coding 0.76 5.778 0.88 79.894
1.488 5.864 0.967 96.183
ENSMUSG00000061755 Bod1l protein_coding 0.949 39.953 1.003
95.504 1.053 54.163 1.049 82.328
ENSMUSG00000061758 Akr1b10 protein_coding 1.085 55.789
0.979 83.876 0.943 87.519 0.91 85.493
ENSMUSG00000061759 1700052N19Rik protein_coding 1.041 40.88 0.94
81.731 1.018 84.724 1.015 82.85
ENSMUSG00000061762 Tac1 protein_coding 1.157 56.66 0.938 82.503
0.924 75.155 1.54 57.839
ENSMUSG00000061777 NA NA NA NA 1 95.504 1
85.792 1 96.042
ENSMUSG00000061778 Mospd2 protein_coding 1.052 45.746 1

95.211 0.951 57.819 0.92 83.724
ENSMUSG00000061780 Cfd protein_coding 1.111 27.545 NA NA
0.842 38.434 0.883 83.724
ENSMUSG00000061787 Rps17 protein_coding 1.057 40.88 0.902 54.427
1.05 35.894 1.008 96.086
ENSMUSG00000061798 Olfr1204 protein_coding 1 85.092 1
93.146 1 79.625 1 94.82
ENSMUSG00000061802 Armc4 protein_coding 1 80.751 1
89.277 1 76.754 1 96.287
ENSMUSG00000061808 Ttr protein_coding 1 79.277 NA NA
1 77.704 1 90.397
ENSMUSG00000061809 Tbpl2 protein_coding 1 84.263 1
90.712 NA NA 1 93.734
ENSMUSG00000061815 Rufy4 protein_coding 1.087 65.284 0.907
67.513 1.01 69.196 0.976 95.832
ENSMUSG00000061816 Myl1 protein_coding 2.236 18.388 1.198
71.617 0.373 0.879 0.931 94.016
ENSMUSG00000061829 NA NA NA NA 1 95.504 1
87.103 1 94.825
ENSMUSG00000061835 Olfr316 protein_coding 1 86.172 1
95.504 1 88.693 1 96.071
ENSMUSG00000061838 Suclg2 protein_coding 1.058 73.401
0.977 97.097 0.968 64.427 0.975 95.273
ENSMUSG00000061843 Vmn1r-ps32 pseudogene 1 65.733 1
96.68 1 85.464 1 95.74
ENSMUSG00000061845 Gm10104 protein_coding 1 82.891 1
96.897 1 83.538 1 94.82
ENSMUSG00000061847 Defb39 protein_coding 1 81.01 1
96.407 1 83.857 1 94.153
ENSMUSG00000061852 Gm7582 pseudogene 0.983 66.76 0.8 91.661
1.274 84.552 1.921 60.31
ENSMUSG00000061859 Inadl protein_coding 0.901 37.248 0.935
79.475 1.188 20.315 1.026 93.685
ENSMUSG00000061863 Gm6822 pseudogene 1 83.968 1
96.843 1 86.258 1 93.156
ENSMUSG00000061864 Galntl6 protein_coding 1 86.843 NA
NA 1 84.724 1 90.177
ENSMUSG00000061875 Olfr1148 protein_coding 1 82.092 1
96.216 1 88.461 1 92.181
ENSMUSG00000061877 BC048679 protein_coding 1 82.543 1
95.431 1 62.058 1 91.525
ENSMUSG00000061878 Sphk1 protein_coding 1.083 50.627 NA NA
0.811 43.842 1.218 83.067
ENSMUSG00000061882 Ccdc62 protein_coding 1.083 37.091
0.798 23.703 1.16 10.819 0.922 86.991
ENSMUSG00000061887 Ssbp3 protein_coding 1.033 50.913 1.137
74.663 0.85 6.515 0.999 94.904
ENSMUSG00000061894 Zscan20 protein_coding 0.998 78.538
0.923 93.094 1.082 69.315 0.972 86.991
ENSMUSG00000061898 Rbak protein_coding 1.03 84.738 0.939
81.731 1.035 50.636 0.987 96.042
ENSMUSG00000061904 Slc25a3 protein_coding 0.933 15.06 1.051
83.104 1.02 57.227 1.045 72.826
ENSMUSG00000061906 Ugt2b38 protein_coding 1 86.199 1

96.847	1	81.525	1	95.74
ENSMUSG00000061923	Odf1	protein_coding	1	47.174	NA	NA
1	63.075	1	86.991
ENSMUSG00000061937	Csn1s2a	protein_coding	0.25	32.749
0.727	85.075	5.516	20.968	0.687	91.372
ENSMUSG00000061950	Ppp4r1	protein_coding	1.1	0.461	1.044
79.411	0.872	0.72	1.036	68.696
ENSMUSG00000061952	Olfr1377	protein_coding	1	82.092	1
96.138	1	88.203	1	96.183
ENSMUSG00000061958	Gm14851	protein_coding	1	85.728	1
91.661	1	72.378	1	95.676
ENSMUSG00000061959	Ces1e	protein_coding	1.355	83.658	0.674
93.654	1.096	88.657	0.837	85.493
ENSMUSG00000061961	Olfr815	protein_coding	1	75.761	1
96.216	1	85.21	1	93.627
ENSMUSG00000061969	Gm5581	protein_coding	1	85.857	1
95.504	NA	NA	1	93.016
ENSMUSG00000061974	Cldn24	protein_coding	1	82.543	1
93.574	1	86.985	1	95.35
ENSMUSG00000061975	Olfr492	pseudogene	1	84.977	1
96.216	1	87.744	1	95.273
ENSMUSG00000061977	Tas2r104	protein_coding	1	84.397	1
92.062	NA	NA	1	94.904
ENSMUSG00000061979	Wbscr16	protein_coding	0.975	62.158
0.972	78.51	1.056	20.315	NA	NA
ENSMUSG00000061981	Flot2	protein_coding	0.884	10.704	1.049
83.104	1.078	29.819	1.047	42.736
ENSMUSG00000061983	Rps12	protein_coding	1.18	2.969	0.87	20.272
0.975	61.743	1.13	44.882
ENSMUSG00000061984	Olfr392	protein_coding	1	77.763	NA
NA	1	87.744	1	94.99
ENSMUSG00000061988	Rpl10a-ps2	protein_coding	1	62.689	1
96.216	1	71.388	1	95.593
ENSMUSG00000061992	Gm4953	protein_coding	1	78.909	NA
NA	1	84.83	1	93.612
ENSMUSG00000062006	Rpl34	protein_coding	1.096	40.88	0.884	0
1.031	41.6	1.053	74.047
ENSMUSG00000062007	Hsh2d	protein_coding	1.175	85.655	1.832
83.104	0.463	56.365	0.927	94.608
ENSMUSG00000062012	Zfp13	protein_coding	0.976	48.939	0.969
95.708	1.055	47.512	1.004	96.192
ENSMUSG00000062014	Gmfb	protein_coding	1.05	42.555	0.9
79.261	1.055	70.328	1.057	67.691
ENSMUSG00000062017	Abca14	protein_coding	2.381	57.38	0.459
90.378	0.916	85.134	0.985	92.561
ENSMUSG00000062031	Athl1	protein_coding	0.967	29.21	0.979	93.654
1.053	47.935	1.053	66.468
ENSMUSG00000062036	4932415M13Rik	lincRNA	1	84.661	1
95.708	1	87.744	1	94.82
ENSMUSG00000062037	Olfr285	protein_coding	1	81.823	1
97.155	1	83.275	1	94.904
ENSMUSG00000062038	Gm10108	protein_coding	1	76.686	1
96.216	1	88.693	1	95.593
ENSMUSG00000062040	Zfp27	protein_coding	0.986	65.733	1.005

91.83 1.008 86.666 1.038 84.246
ENSMUSG00000062042 Olfr294 protein_coding 1 84.452 NA
NA 1 83.275 1 94.904
ENSMUSG00000062044 Lmtk3 protein_coding 0.677 7.218 1.087 83.464
1.361 20.051 0.948 85.493
ENSMUSG00000062054 Iah1 protein_coding 0.987 70.213 1.025
91.584 0.985 87.519 0.974 89.892
ENSMUSG00000062061 Obp2a protein_coding 1 48.462 1
95.504 1 82.31 1 96.358
ENSMUSG00000062064 Slc2a7 protein_coding 1 82.982 1
96.407 1 74.535 1 93.016
ENSMUSG00000062070 Pgk1 protein_coding 0.894 21.393 1.092
83.104 1.026 65.8 1.216 46.846
ENSMUSG00000062074 Wisp3 protein_coding 0.675 61.043 NA NA
1.525 44.787 1.023 93.117
ENSMUSG00000062075 Lmnb2 protein_coding 0.88 33.745 1.05
92.062 1.079 30.948 1.081 62.594
ENSMUSG00000062078 Qk protein_coding 1.006 75.671 0.996
92.253 0.995 45.573 0.987 91.872
ENSMUSG00000062082 Cd200r4 protein_coding 2.783 0 0.768
40.946 0.467 5.323 0.955 93.156
ENSMUSG00000062083 Rpl13a-ps1 protein_coding 1 85.77 1
95.35 1 87.744 1 93.964
ENSMUSG00000062087 4930423O20Rik protein_coding 1 71.915
1 95.708 1 62.237 1 96.287
ENSMUSG00000062093 Gm10110 protein_coding 1.228 36.124
0.855 88.881 0.952 87.431 1.151 66.468
ENSMUSG00000062098 Btbd3 protein_coding 1.181 3.825 1.038 83.104
0.814 1.061 1.014 93.261
ENSMUSG00000062101 Zfp119b protein_coding 0.942 72.546
0.926 84.858 1.149 38.099 1.216 72.992
ENSMUSG00000062103 Olfr924 protein_coding 1 86.474 NA
NA 1 78.369 1 94.297
ENSMUSG00000062105 Olfr183 protein_coding 1 76.546 1
93.817 1 79.897 1 92.018
ENSMUSG00000062110 Scfd2 protein_coding 1.009 58.264 NA NA
1.022 62.237 1.062 68.696
ENSMUSG00000062115 Rai1 protein_coding 0.872 5.544 1.041 93.529
1.101 27.431 1.036 91.372
ENSMUSG00000062116 Zfp954 protein_coding 1.028 83.418
0.977 93.654 0.993 70.623 0.955 92.12
ENSMUSG00000062121 Olfr149 protein_coding 1 85.728 NA
NA 1 87.898 1 95.74
ENSMUSG00000062127 Cttnbp2nl protein_coding 1.088 16.959
0.941 73.766 0.979 72.464 0.98 83.724
ENSMUSG00000062128 Olfr20 protein_coding 1 84.865 1
96.216 1 88.601 1 92.018
ENSMUSG00000062132 Gm1082 antisense 2.103 78.054 0.244
59.946 1.947 54.673 1.432 95.273
ENSMUSG00000062142 Olfr569 protein_coding 1 81.196 1
95.088 1 77.704 1 96.287
ENSMUSG00000062148 Ear6 protein_coding 1 83.523 1
96.138 NA NA 1 95.877
ENSMUSG00000062151 Unc13c protein_coding 0.951 67.334

1.141 82.904 0.924 77.294 1.274 78.948
ENSMUSG00000062152 Rpl21-ps14 pseudogene 1 84.738 1
96.843 NA NA 1 93.964
ENSMUSG00000062157 Ifnlr1 protein_coding 0.751 0.987 NA NA
1.423 6.202 0.903 86.699
ENSMUSG00000062162 Mageb1 protein_coding 1 74.958 1
96.948 1 80.381 1 94.904
ENSMUSG00000062165 Vmn1r232 protein_coding 1 86.918 NA
NA 1 76.754 1 95.676
ENSMUSG00000062169 Cnih4 protein_coding 0.979 85.857 1.039
85.756 0.985 72.269 0.999 91.011
ENSMUSG00000062170 Fmr1nb protein_coding 0.615 54.87 2.699
87.609 0.602 67.029 1.523 83.067
ENSMUSG00000062175 Tgif2 protein_coding 0.998 85.391 0.972
93.765 1.032 75.809 0.97 95.74
ENSMUSG00000062181 NA NA NA NA 1 95.504 1
84.724 1 95.273
ENSMUSG00000062184 Hs6st2 protein_coding 1.285 18.388
0.803 73.766 0.965 50.523 1.194 86.133
ENSMUSG00000062186 Olfr395 protein_coding 1 86.843 1
95.35 1 79.897 1 94.384
ENSMUSG00000062190 Lancl2 protein_coding 1.014 83.169
1.025 83.104 0.963 21.331 0.98 95.676
ENSMUSG00000062198 2700097O09Rik protein_coding 0.972 71.674
1.01 95.504 1.021 72.853 0.97 90.805
ENSMUSG00000062199 Olfr1465 protein_coding 1 86.464 1
96.138 1 85.935 1 92.31
ENSMUSG00000062200 Vmn2r7 protein_coding 1 86.145 1
97.288 1 76.388 1 95.676
ENSMUSG00000062201 NA NA NA NA 1 96.843 1
79.897 1 95.559
ENSMUSG00000062202 Btbd9 protein_coding 1.073 2.651 0.975 72.712
0.952 25.512 1.038 89.72
ENSMUSG00000062203 Gspt1 protein_coding 1.019 71.217 1.053
76.086 0.932 20.511 1.09 65.845
ENSMUSG00000062204 Olfr1373 protein_coding 1 79.709 1
93.654 1 86.666 1 92.903
ENSMUSG00000062209 Erbb4 protein_coding 1.16 10.595 0.947
95.211 0.91 42.621 0.677 83.724
ENSMUSG00000062210 Tnfaip8 protein_coding 1.181 0 0.958
68.115 0.885 2.722 0.972 93.261
ENSMUSG00000062224 4933411G06Rik protein_coding 1 84.661
1 90.138 1 88.203 1 95.777
ENSMUSG00000062226 Gm10112 protein_coding 1 85.832 1
95.504 1 86.258 1 96.183
ENSMUSG00000062232 Rapgef2 protein_coding 0.983 81.823
0.911 90.053 1.114 57.907 1.022 90.177
ENSMUSG00000062234 Gak protein_coding 1.027 52.986 1.006
83.321 0.97 83.811 0.984 90.628
ENSMUSG00000062235 Gm5341 pseudogene 1 81.178 1
95.211 1 86.144 1 96.287
ENSMUSG00000062245 Olfr170 protein_coding 1 83.823 1
97.263 1 72.853 1 94.904
ENSMUSG00000062248 Cks2 protein_coding 1.304 39.295 0.814

70.29 0.94 79.119 1.224 78.042
ENSMUSG00000062252 Lhfpl5 protein_coding 1 83.169 1
90.378 1 70.9 1 94.19
ENSMUSG00000062257 Opcml protein_coding 0.978 63.534 1.212
79.411 0.843 17.258 1.071 70.539
ENSMUSG00000062270 Morf4l1 protein_coding 1.002 38.682
0.949 53.434 1.054 58.051 1.036 57.075
ENSMUSG00000062272 Olfr1129 protein_coding 1 79.433 1
96.138 1 88.601 1 96.183
ENSMUSG00000062275 Fbxw24 protein_coding 1 83.523 1
97.049 1 86.666 1 93.264
ENSMUSG00000062278 Gm11562 protein_coding 1 66.406 1
96.216 1 86.258 1 96.183
ENSMUSG00000062279 Gm10237 pseudogene 1 85.832 NA NA
1 80.015 1 96.248
ENSMUSG00000062280 Olfr1285 pseudogene 1 76.686 1
95.708 1 88.349 NA NA
ENSMUSG00000062282 Gm10113 protein_coding 1 81.125 1
96.013 1 87.744 1 93.457
ENSMUSG00000062284 Gm6030 pseudogene 1.041 80.608 0.96
96.268 0.996 87.971 0.872 94.904
ENSMUSG00000062285 Olfr1386 protein_coding 1 81.465 1
95.504 1 84.724 1 96.086
ENSMUSG00000062296 Trank1 protein_coding 1.539 20.799
1.095 96.68 0.592 41.154 0.807 86.699
ENSMUSG00000062300 Pvrl2 protein_coding 1.007 76.135 1.086
82.904 0.917 58.516 0.851 83.724
ENSMUSG00000062309 Rpp25 protein_coding 1.445 22.871 0.87
82.947 0.795 51.055 1.704 31.133
ENSMUSG00000062312 Erbb2 protein_coding 0.869 8.614 0.888 58.277
1.3 7.024 1.01 90.611
ENSMUSG00000062314 Olfr1505 protein_coding 1 82.231 1
94.96 1 81.857 1 95.777
ENSMUSG00000062319 Gm10115 protein_coding 0.634 13.062
0.981 83.104 1.609 60.5 1.202 86.118
ENSMUSG00000062327 T protein_coding 1 67.334 1
85.075 1 41.235 1 92.748
ENSMUSG00000062328 Rpl17 protein_coding 1.066 62.689 0.889
65.165 1.057 45.667 1.053 68.354
ENSMUSG00000062329 Cytl1 protein_coding 1.136 42.555 0.784
79.261 1.122 79.426 1.126 95.273
ENSMUSG00000062342 Serpinb9e protein_coding 1 84.452 1
93.046 1 87.786 1 96.248
ENSMUSG00000062352 Itgb1bp1 protein_coding 1.1 10.391
1.022 83.104 0.888 1.061 0.891 83.724
ENSMUSG00000062365 Gm4968 protein_coding 1 83.726 NA
NA 1 78.642 1 88.512
ENSMUSG00000062369 Usp17lb protein_coding 1 72 1
96.216 1 88.729 1 89.892
ENSMUSG00000062372 Otof protein_coding 1.793 43.654 0.953
90.287 0.583 69.134 0.783 93.612
ENSMUSG00000062373 Tmem65 protein_coding 1.031 77.182
0.985 89.509 0.982 84.724 0.987 95.273
ENSMUSG00000062376 2010012O05Rik protein_coding 1.006 84.564

1.044 87.185 0.948 40.794 1.028 83.067
ENSMUSG00000062380 Tubb3 protein_coding 1.897 6.135 0.796 95.211
0.662 12.288 1.689 43.605
ENSMUSG00000062381 Vps28 protein_coding 0.926 8.122 1.01 88.092
1.066 36.578 1.011 96.183
ENSMUSG00000062383 B130024G19Rik protein_coding 1.596 7.179
0.842 91.661 0.743 12.288 1.04 91.372
ENSMUSG00000062391 4932435O22Rik lincRNA 2.904 4.925 0.628
90.378 0.549 9.573 1.029 86.991
ENSMUSG00000062393 Dgkk protein_coding 0.901 84.049 0.847
82.073 1.309 36.011 0.886 83.067
ENSMUSG00000062397 Zfp706 protein_coding 1.045 37.414
1.031 88.092 0.927 14.788 0.974 89.475
ENSMUSG00000062400 Krtap6-5 protein_coding 1 81.657 1
97.288 1 83.607 1 89.052
ENSMUSG00000062410 Hsd3b3 protein_coding 1 60.168 1
92.355 1 86.666 1 92.181
ENSMUSG00000062421 Arf2 protein_coding 1.035 40.333 1.087
77.618 0.888 7.742 1.05 89.108
ENSMUSG00000062426 Olfr304 protein_coding 1 82.732 1
95.211 1 84.071 1 95.273
ENSMUSG00000062432 Cyp26c1 protein_coding 0.981 60.707
1.723 95.088 0.589 85.792 1.458 88.512
ENSMUSG00000062433 Krtap6-2 pseudogene 1 80.51 1 94.887
1 84.724 1 93.457
ENSMUSG00000062434 Olfr516 protein_coding 1 77.615 1
96.897 NA NA 1 94.82
ENSMUSG00000062438 Adam1b protein_coding 1 81.465 1
91.776 1 84.896 1 95.273
ENSMUSG00000062444 Ap3b2 protein_coding 1.279 56.907 0.803
88.705 0.975 88.729 1.284 83.067
ENSMUSG00000062456 Rpl9-ps6 protein_coding 17.146 0
0.904 94.102 0.064 0.72 0.952 93.612
ENSMUSG00000062461 Gm5453 pseudogene 1 81.178 1
95.708 1 86.258 1 95.273
ENSMUSG00000062470 5730577I03Rik antisense 0.916 54.176
1.203 95.088 0.905 88.349 0.986 96.358
ENSMUSG00000062472 Gm4945 protein_coding 1 85.283 NA
NA 1 76.768 1 95.273
ENSMUSG00000062477 Hmgb1l pseudogene 1 85.728 1
96.68 1 87.519 1 94.001
ENSMUSG00000062478 Ctrc protein_coding 1 84.263 1
93.817 1 76.64 1 93.457
ENSMUSG00000062480 Acat3 protein_coding 0.668 6.221 1.386 74.663
1.075 47.776 0.723 83.724
ENSMUSG00000062483 Vmn1r171 protein_coding 1 75.399 1
95.211 NA NA 1 96.086
ENSMUSG00000062488 I830012O16Rik protein_coding 1.224 17.191
1.219 82.904 0.668 11.504 1.594 42.736
ENSMUSG00000062496 4930431F12Rik protein_coding 1 71.142
1 86.728 1 84.724 1 96.287
ENSMUSG00000062497 Olfr1411 protein_coding 1 80.146 1
96.843 1 87.37 1 95.72
ENSMUSG00000062501 4930425F17Rik protein_coding 1 61.633

NA NA NA NA 1 74.193
ENSMUSG00000062510 Nsl1 protein_coding 1.485 10.687 0.763
62.692 0.884 63.504 1.207 87.447
ENSMUSG00000062511 4930512M02Rik protein_coding 1 76.319
1 95.329 1 86.258 1 95.777
ENSMUSG00000062515 Fabp4 protein_coding 0.881 19.365 0.989
85.242 1.143 10.76 0.958 96.183
ENSMUSG00000062518 Zfp534 protein_coding 1 67.163 1
74.633 1 65.8 1 92.018
ENSMUSG00000062519 Zfp398 protein_coding 0.92 50.519
0.952 89.509 1.143 37.369 0.978 92.561
ENSMUSG00000062524 Ncr1 protein_coding 0.755 30.136 2.001
40.99 0.663 25.625 0.982 95.593
ENSMUSG00000062526 Mppe1 protein_coding 1.111 20.032 0.936
78.51 0.964 53.7 0.879 83.724
ENSMUSG00000062527 Olfr1408 protein_coding 1 85.728 1
96.68 1 87.274 1 95.273
ENSMUSG00000062528 Tas2r109 protein_coding 1 71.674 1
91.361 1 87.786 1 95.72
ENSMUSG00000062542 Syt9 protein_coding 1.447 27.113 1.114
85.756 0.621 12.41 0.735 92.675
ENSMUSG00000062543 Gsdmcl1 protein_coding 1 85.391 1
97.049 1 79.554 1 94.567
ENSMUSG00000062545 Tlr12 protein_coding 1.059 68.485 1.741
62.898 0.54 4.538 0.704 83.724
ENSMUSG00000062546 V1ra8 protein_coding 1 86.474 1
96.335 1 87.519 1 96.287
ENSMUSG00000062551 Prl2c1 protein_coding 1 85.283 1
96.948 1 86.475 1 96.287
ENSMUSG00000062553 Olfr709-ps1 protein_coding 1 69.777 1
96.138 1 86.552 1 95.92
ENSMUSG00000062554 Gm12751 pseudogene 1 81.178 1
92.931 1 75.637 1 90.805
ENSMUSG00000062556 Scgb1b2 protein_coding 1 86.843 1
92.137 1 84.724 1 94.174
ENSMUSG00000062561 Gm10118 protein_coding 1 71.915 1
96.763 1 83.925 1 91.011
ENSMUSG00000062563 Cys1 protein_coding 0.753 38.555 1.015
96.407 1.312 67.823 1.647 59.464
ENSMUSG00000062564 Tex28 protein_coding 1 84.294 1
96.013 1 86.475 1 96.086
ENSMUSG00000062580 Timm17a protein_coding 0.927 21.176
0.931 96.268 1.159 43.842 1.109 60.968
ENSMUSG00000062582 Rpl30-ps8 pseudogene 1 70.597 NA NA
NA NA 1 93.132
ENSMUSG00000062585 Cnr2 protein_coding 1.488 1.323 1.047 94.848
0.641 3.428 0.935 87.331
ENSMUSG00000062588 Gm6104 protein_coding 0.551 68.846
1.419 93.899 1.282 86.727 1.618 93.722
ENSMUSG00000062590 Armc9 protein_coding 0.893 14.403 1.015
90.138 1.105 22.131 1.057 64.513
ENSMUSG00000062591 Tubb4a protein_coding 0.961 33.002
1.305 71.617 0.797 55.9 1.052 90.674
ENSMUSG00000062593 Lilrb4 protein_coding 1.693 0 0.852

84.594 0.694 3.147 1.106 79.442
ENSMUSG00000062604 Srpk2 protein_coding 1.011 79.277 1.01
95.941 0.981 71.388 1.038 65.558
ENSMUSG00000062608 Olfr195 protein_coding 1 73.841 1
94.102 1 87.658 1 96.183
ENSMUSG00000062609 Kcnj15 protein_coding 0.651 3.623 1.129
83.104 1.354 35.13 0.945 93.612
ENSMUSG00000062619 2310039H08Rik protein_coding 0.86 35.449
1.157 86.277 1.006 82.74 1.018 95.559
ENSMUSG00000062621 NA NA NA NA 1 96.897 1
79.625 1 96.183
ENSMUSG00000062624 Cyp2c67 protein_coding 1 85.203 1
95.708 1 83.538 1 95.74
ENSMUSG00000062627 Mysm1 protein_coding 1.015 63.804 0.951
75.345 1.032 79.119 0.944 90.691
ENSMUSG00000062629 Olfr114 protein_coding 1 85.203 1
87.857 1 56.125 1 90.674
ENSMUSG00000062646 Ganc protein_coding 1.092 28.927 0.992
86.277 0.92 78.293 0.902 84.29
ENSMUSG00000062647 Rpl7a protein_coding 1.015 79.277 0.928
24.911 1.059 16.666 1.029 85.577
ENSMUSG00000062649 Olfr44 protein_coding 1 74.299 1
95.329 1 88.729 1 96.358
ENSMUSG00000062651 H2afb1 protein_coding 1 82.982 NA
NA 1 84.071 1 92.12
ENSMUSG00000062661 Ncs1 protein_coding 1.244 16.124 0.883
97.288 0.91 22.009 1.158 69.708
ENSMUSG00000062683 Atp5g2 protein_coding 0.944 19.224
1.013 95.504 1.042 30.107 0.982 96.287
ENSMUSG00000062687 Gm6531 protein_coding 1 75.298 1
96.216 1 84.626 1 95.593
ENSMUSG00000062691 1110001A16Rik protein_coding 1 78.538
0.989 94.96 1.013 85.464 1.064 73.597
ENSMUSG00000062694 Cav3 protein_coding 3.873 20.659 0.576
97.097 0.448 3.718 1.368 85.786
ENSMUSG00000062704 9430002A10Rik protein_coding 0.781 82.982
0.726 52.266 1.768 29.777 1.371 78.948
ENSMUSG00000062705 Tpbpb protein_coding 1 78.639 1
96.216 1 88.203 1 92.381
ENSMUSG00000062712 Olfr531 protein_coding 1 85.728 NA
NA 1 86.666 1 96.358
ENSMUSG00000062713 Sim2 protein_coding 3.89 3.152 0.446 65.165
0.576 7.399 0.499 85.493
ENSMUSG00000062729 Ppox protein_coding 0.974 19.023 0.972
89.509 1.059 16.808 0.959 87.182
ENSMUSG00000062732 Lypd4 protein_coding 1 81.01 1 96.138
1 86.112 1 95.273
ENSMUSG00000062737 Prl3d2 protein_coding 1 86.74 1
95.504 1 88.657 1 95.273
ENSMUSG00000062742 Gm5239 protein_coding 1 81.823 NA
NA 1 87.898 1 96.287
ENSMUSG00000062743 Zfp677 protein_coding 1.147 23.019
0.962 88.881 0.907 56.822 0.96 85.493
ENSMUSG00000062751 Prss1 protein_coding 1 83.523 1

96.216 1 78.293 1 95.72
ENSMUSG00000062753 AI413582 protein_coding 1.03 49.868
1.016 94.887 0.954 71.004 0.982 92.12
ENSMUSG00000062757 Olfr1206 protein_coding 1 78.744 1
96.216 1 87.336 1 92.762
ENSMUSG00000062758 Gm16477 protein_coding 1 71.915 1
96.68 1 81.857 1 92.181
ENSMUSG00000062760 1810041L15Rik protein_coding 2.668 33.555
1.566 96.847 0.239 32.963 0.313 83.724
ENSMUSG00000062761 Zfp512 protein_coding 0.958 85.092
0.941 67.513 1.103 30.561 1.075 68.354
ENSMUSG00000062762 Ei24 protein_coding 1.059 7.492 1.001 90.256
0.946 31.135 0.96 85.493
ENSMUSG00000062782 Olfr527 protein_coding 1 84.168 1
97.288 1 87.103 1 94.904
ENSMUSG00000062785 Kcnc3 protein_coding 0.797 13.952 1.112
95.431 1.13 42.199 1.071 96.192
ENSMUSG00000062791 Gm382 protein_coding 1 86.918 1
95.504 1 88.601 1 95.273
ENSMUSG00000062793 Olfr1158 protein_coding 1 86.112 NA
NA 1 88.693 1 94.001
ENSMUSG00000062794 Zfp599 protein_coding 0.743 43.495
0.837 66.09 1.61 27.431 1.276 86.445
ENSMUSG00000062797 l7Rn6 protein_coding 0.968 42.722 0.975
96.847 1.062 57.637 0.998 68.696
ENSMUSG00000062808 Hist1h3d protein_coding 0.98 45.229
1.173 83.104 0.867 81.29 0.813 96.183
ENSMUSG00000062814 Ssxa1 protein_coding 1 85.728 NA NA
1 86.112 1 96.086
ENSMUSG00000062818 Vmn1r51 protein_coding 1 86.843 1
96.843 1 86.144 1 96.287
ENSMUSG00000062822 4833420G17Rik protein_coding 1.01 77.916
0.967 90.256 1.025 73.759 0.87 83.724
ENSMUSG00000062825 Actg1 protein_coding 1.076 3.713 1.023 96.268
0.906 17.381 1 94.904
ENSMUSG00000062826 Ces2f protein_coding 1 82.891 1
95.708 1 88.41 1 96.183
ENSMUSG00000062833 Sval3 protein_coding 1 80.608 1
95.708 1 86.112 1 95.74
ENSMUSG00000062844 Olfr1458 protein_coding 1 85.283 1
93.654 1 83.275 1 95.92
ENSMUSG00000062846 Gm14176 pseudogene 1 81.76 1 96.897
1 60.96 1 90.95
ENSMUSG00000062859 Tcp11 protein_coding 0.939 74.516 1.153
95.504 0.923 87.173 0.885 86.445
ENSMUSG00000062861 Zfp28 protein_coding 0.947 36.034 0.893
80.463 1.183 20.051 1.044 83.067
ENSMUSG00000062866 Phactr2 protein_coding 1.011 47.466
0.985 83.392 0.999 88.693 0.989 86.991
ENSMUSG00000062867 Impdh2 protein_coding 1.083 23.493
0.913 55.265 1.009 71.839 1.102 35.053
ENSMUSG00000062868 Olfr830 protein_coding 1 77.497 1
96.216 1 79.325 1 88.111
ENSMUSG00000062873 Olfr1355 protein_coding 1 73.401 1

94.102 NA NA 1 96.358
ENSMUSG00000062874 Gm4978 protein_coding 1 86.658 1
95.708 1 87.786 1 96.183
ENSMUSG00000062878 Olfr298 protein_coding 1 72.372 1
96.138 1 85.792 1 96.358
ENSMUSG00000062892 Olfr1450 protein_coding 1 82.982 1
95.211 1 84.961 1 94.608
ENSMUSG00000062901 Klhl24 protein_coding 1.053 66.944
0.929 82.848 1.019 70.534 0.942 83.724
ENSMUSG00000062905 Vmn1r32 protein_coding 0.561 66.129
0.931 90.454 1.918 86.831 3.754 75.81
ENSMUSG00000062906 Hdac10 protein_coding 0.967 33.002
1.056 84.126 0.976 87.898 1.018 68.696
ENSMUSG00000062908 Acadm protein_coding 1.007 72.546 1.002
97.155 0.991 85.792 1.017 95.35
ENSMUSG00000062914 Olfr777 protein_coding 1 83.09 1
93.899 1 84.308 1 95.273
ENSMUSG00000062929 Cfl2 protein_coding 0.989 86.658 1.095
90.116 0.924 39.245 1.077 56.339
ENSMUSG00000062931 Zfp938 protein_coding 1.127 3.932 0.998
90.256 0.887 9.494 1.022 90.865
ENSMUSG00000062937 Mtap protein_coding 0.978 71.896 1.029
96.843 0.993 67.029 1.155 57.952
ENSMUSG00000062939 Stat4 protein_coding 1.485 13.363 0.81
66.651 0.831 63.919 0.892 92.181
ENSMUSG00000062944 9130023H24Rik protein_coding 1.064 52.134
0.995 93.094 0.945 72.464 1.012 94.904
ENSMUSG00000062949 Atp11c protein_coding 1.094 35.499
1.006 90.053 0.908 72.464 1.039 70.36
ENSMUSG00000062952 Tas2r110 protein_coding 1 76.686 NA
NA 1 86.258 1 96.287
ENSMUSG00000062960 Kdr protein_coding 0.854 13.74 1.083 96.68
1.078 70.328 0.925 78.398
ENSMUSG00000062961 Ccdc177 protein_coding 0.557 48.646
0.547 70.864 3.265 13.956 1.223 96.183
ENSMUSG00000062962 Gm6378 pseudogene 1 86.658 NA NA
1 82.778 1 95.273
ENSMUSG00000062963 Ufc1 protein_coding 0.931 23.641 1.02
90.378 1.055 35.894 1.057 51.908
ENSMUSG00000062980 Cped1 protein_coding 1.182 32.238 1.024
83.104 0.826 8.382 1.188 87.886
ENSMUSG00000062981 Mrpl42 protein_coding 0.873 7.43 1.151
63.504 NA NA 1.06 93.964
ENSMUSG00000062987 Gm10081 protein_coding 1 85.728 1
96.216 1 87.744 1 96.086
ENSMUSG00000062991 Nrg1 protein_coding 0.728 71.763 1.095
88.092 1.259 86.084 1.321 87.7
ENSMUSG00000062995 Ica1 protein_coding 0.924 33.906 1.034
96.268 1.049 38.099 0.959 86.991
ENSMUSG00000062997 Rpl35 protein_coding 0.957 60.569 1.001
94.48 1.046 39.439 1.005 96.358
ENSMUSG00000063001 Rps23-ps pseudogene 5.602 0 0.75 92.474
0.238 0.72 0.862 89.892
ENSMUSG00000063015 Ccni protein_coding 1.062 32.676 1.015

93.994	0.929	33.137	0.917	83.724
ENSMUSG00000063018	2010204K13Rik	processed_transcript	0.86
65.388	1.323	89.434	0.876	73.874	1.287	82.328
ENSMUSG00000063019	Manbal	protein_coding	1.039	73.734
0.956	81.284	1.004	79.897	0.954	87.992
ENSMUSG00000063020	Olfr194	protein_coding	1	74.482	1
92.474	1	83.811	1	96.183
ENSMUSG00000063047	Zfp780b	protein_coding	1.159	3.393	0.967
61.625	0.892	83.397	0.933	86.699
ENSMUSG00000063049	Ing2	protein_coding	0.919	34.162	1.158
78.705	0.94	71.004	0.949	89.475
ENSMUSG00000063052	Lrrc40	protein_coding	1.147	7.179	0.938
77.944	0.929	25.512	0.999	95.273
ENSMUSG00000063058	Gm6406	protein_coding	1	83.968	1
96.763	1	70.214	1	96.183
ENSMUSG00000063060	Sox7	protein_coding	1.059	86.74	1.044	96.216
0.906	87.431	0.925	88.053
ENSMUSG00000063063	Ctnna2	protein_coding	1.322	40.065
1.043	90.287	0.725	57.446	0.784	86.445
ENSMUSG00000063065	Mapk3	protein_coding	0.935	67.867	1.067
83.104	1.005	78.045	0.989	84.29
ENSMUSG00000063077	Kif1b	protein_coding	1.021	68.16	1.011	95.196
0.971	56.365	0.999	95.35
ENSMUSG00000063080	Gm14501	protein_coding	1	85.129	NA
NA	1	80.899	1	94.608
ENSMUSG00000063087	Gm10125	processed_transcript	1.109	52.429
0.975	96.843	0.924	35.022	1.073	96.287
ENSMUSG00000063106	Olfr1510	protein_coding	1	80.251	1
93.899	1	84.83	1	96.086
ENSMUSG00000063108	Zfp26	protein_coding	0.983	71.36	0.935	81.284
1.084	38.152	1.051	89.428
ENSMUSG00000063109	A930013B10Rik	antisense	1.072	86.112
1.639	93.863	0.569	46.575	0.703	89.892
ENSMUSG00000063116	Olfr410	protein_coding	1	86.199	1
95.504	1	69.825	1	93.734
ENSMUSG00000063120	Olfr480	protein_coding	1	85.391	1
95.211	1	86.602	1	95.74
ENSMUSG00000063129	Aldoart2	protein_coding	1	67.477	1
95.431	NA	NA	1	96.358
ENSMUSG00000063130	Calml3	protein_coding	1.114	71.06	0.935
92.545	0.963	78.642	0.784	86.699
ENSMUSG00000063133	Klk1b1	protein_coding	1	83.418	1
88.704	1	77.888	1	91.807
ENSMUSG00000063137	Olfr177	protein_coding	1	86.843	NA
NA	1	87.37	1	96.248
ENSMUSG00000063142	Kcnma1	protein_coding	1.273	11.928
1.092	96.216	0.72	18.797	0.928	83.952
ENSMUSG00000063145	Bbs5	protein_coding	1.052	69.777	0.913
79.807	1.037	52.574	0.939	85.493
ENSMUSG00000063146	Clip2	protein_coding	0.964	54.604	1.009
96.013	1.025	55.512	0.97	94.82
ENSMUSG00000063157	Csn2	protein_coding	0.417	33.745	0.865
88.691	2.782	20.085	1.042	83.581
ENSMUSG00000063160	Numbl	protein_coding	1.125	26.136	0.936

95.329 0.945 25.512 1.086 31.133
ENSMUSG00000063163 Speer2 protein_coding 1 57.227 1
95.431 NA NA 1 92.466
ENSMUSG00000063171 Rps4y2 protein_coding 0.941 45.728
1.085 77.618 0.976 73.598 0.851 85.493
ENSMUSG00000063172 Hspb11 protein_coding 1.168 32.238
0.943 93.908 0.905 67.339 0.914 90.611
ENSMUSG00000063173 Olfr811 protein_coding 1 78.909 NA
NA 1 87.658 1 96.192
ENSMUSG00000063176 Olfr955 protein_coding 1 74.745 NA
NA 1 87.658 1 96.358
ENSMUSG00000063179 Pstk protein_coding 0.927 34.795 1.092
79.261 0.99 82.685 1.046 92.263
ENSMUSG00000063180 Gm10126 protein_coding 1 66 NA NA
1 88.657 1 95.832
ENSMUSG00000063188 Olfr107 protein_coding 1 86.333 1
95.504 1 86.346 1 96.086
ENSMUSG00000063193 Cd300lb protein_coding 1.427 2.047 0.887
51.115 0.788 51.715 1.162 60.466
ENSMUSG00000063200 Nol7 protein_coding 1.006 80.815 NA NA
0.972 86.985 1.037 74.607
ENSMUSG00000063206 Gm15315 protein_coding 1 84.977 1
94.262 1 88.203 1 96.183
ENSMUSG00000063216 Olfr1351 protein_coding 1 73.585 1
96.407 NA NA 1 93.132
ENSMUSG00000063221 Olfr930 protein_coding 1 80.51 NA NA
1 87.971 1 94.904
ENSMUSG00000063225 Olfr917 protein_coding 1 78.054 1
92.631 1 76.754 1 91.269
ENSMUSG00000063229 Ldha protein_coding 0.626 0.987 1.226 79.579
1.305 10.76 1.201 54.153
ENSMUSG00000063230 Olfr535 protein_coding 1 81.196 1
95.211 1 86.084 1 96.183
ENSMUSG00000063232 Serpina11 protein_coding 1 63.126 1
91.661 1 83.607 1 92.12
ENSMUSG00000063235 Ptpmt1 processed_transcript 0.936 5.967
1.043 90.29 1.024 36.578 0.927 87.218
ENSMUSG00000063236 1110038F14Rik protein_coding 0.952 31.614
NA NA 1.042 32.776 1.028 93.156
ENSMUSG00000063240 Olfr133 protein_coding 1 83.09 1
94.597 1 80.381 1 94.654
ENSMUSG00000063242 Tgif2lx2 protein_coding 1 70.36 1
96.843 1 88.161 1 92.851
ENSMUSG00000063245 Gm13248 protein_coding 1 86.351 1
95.301 1 66.647 1 89.498
ENSMUSG00000063251 Krtap4-1 protein_coding 1 83.09 1
96.68 1 84.552 1 95.74
ENSMUSG00000063252 Gm4744 protein_coding 1 84.865 1
96.897 1 86.985 1 96.042
ENSMUSG00000063253 Scoc protein_coding 0.967 84.049 1.053
96.843 0.979 88.274 1.053 63.708
ENSMUSG00000063254 B230325K18Rik protein_coding 1.008 68.485
1.006 92.545 0.987 79.325 1.07 93.156
ENSMUSG00000063260 Syt10 protein_coding 1 80.608 1

94.597 1 85.58 1 96.358
ENSMUSG00000063268 Parp10 protein_coding 0.966 63.283
1.062 88.092 0.972 77.294 1.058 46.846
ENSMUSG00000063273 Naa15 protein_coding 1.096 1.161 0.965 85.242
0.944 15.169 1.058 86.118
ENSMUSG00000063275 Ptpla protein_coding 1.126 2.998 0.942 92.137
0.943 10.934 0.956 85.493
ENSMUSG00000063277 Gm10128 protein_coding 1 82.543 1
94.887 1 86.144 1 94.567
ENSMUSG00000063281 Zfp35 protein_coding 1.107 16.249 1.028
92.111 0.879 11.314 1.062 66.944
ENSMUSG00000063286 Gm8995 pseudogene 1.111 29.536 0.973
88.881 0.921 44.865 1.181 57.711
ENSMUSG00000063296 Tmem117 protein_coding 1.398 7.332 1.106
96.763 0.648 6.858 0.881 96.086
ENSMUSG00000063297 Luzp2 protein_coding 0.893 85.728 NA NA
1.123 85.464 1.1 95.92
ENSMUSG00000063303 Gm10129 protein_coding 0.852 49.944
0.884 90.256 1.331 31.4 0.812 86.991
ENSMUSG00000063305 Psg20 protein_coding 1 84.819 1
95.088 1 83.538 1 96.183
ENSMUSG00000063316 Rpl27 protein_coding 0.947 75.761 0.932
75.618 1.133 46.206 1 94.904
ENSMUSG00000063317 Usp31 protein_coding 1.018 85.59 1.036 93.899
0.949 83.397 0.941 91.105
ENSMUSG00000063320 1190007I07Rik protein_coding 1.009 81.465
0.988 87.919 1.006 70.534 0.88 83.724
ENSMUSG00000063328 Gm9396 protein_coding 1 73.841 1
96.897 1 82.31 1 94.468
ENSMUSG00000063334 Krr1 protein_coding 1.085 54.344 0.946
79.475 0.977 88.729 0.989 85.629
ENSMUSG00000063350 Olfr874 protein_coding 1 80.251 NA
NA 1 84.308 1 91.525
ENSMUSG00000063354 Slc39a4 protein_coding 1.667 2.569 0.911
90.256 0.658 12.571 0.661 88.505
ENSMUSG00000063358 Mapk1 protein_coding 1.025 41.211 1.023
87.029 0.954 11.997 1.067 44.679
ENSMUSG00000063362 Alg11 protein_coding 1.106 10.687 1.014
93.765 0.893 24.309 1.012 90.177
ENSMUSG00000063364 3300002I08Rik protein_coding 1.207 84.324
0.913 87.041 0.908 81.29 0.872 94.608
ENSMUSG00000063374 Olfr763 protein_coding 1 74.482 1
95.301 1 86.831 1 94.297
ENSMUSG00000063376 Ifna13 protein_coding 1 80.03 1
97.155 1 82.685 1 95.877
ENSMUSG00000063380 Olfr945 protein_coding 1 81.01 1
95.329 1 84.669 1 94.825
ENSMUSG00000063382 Bcl9l protein_coding 0.917 23.923 1.159
65.165 0.941 16.129 0.999 96.183
ENSMUSG00000063383 Zfp947 protein_coding 1.21 10.19 0.942
76.978 0.875 66.425 1.085 93.117
ENSMUSG00000063386 Olfr1387 protein_coding 1 85.355 1
95.211 1 84.896 1 94.904
ENSMUSG00000063388 BC023105 pseudogene 1.013 85.857 1.529

66.01 0.646 11.902 0.973 95.35
ENSMUSG00000063394 Olfr293 protein_coding 1 80.751 1
94.597 1 88.601 1 95.273
ENSMUSG00000063406 Tmed5 protein_coding 1.091 23.493 0.996
95.088 0.922 54.521 0.978 90.611
ENSMUSG00000063409 Lrrc43 protein_coding 5.328 20.942
0.359 68.115 0.523 85.935 0.516 91.105
ENSMUSG00000063410 Stk24 protein_coding 1.079 7.255 1.014 95.211
0.91 1.705 0.99 96.086
ENSMUSG00000063412 Gm10131 pseudogene 1 77.182 1
94.102 1 83.607 1 96.287
ENSMUSG00000063415 Cyp26b1 protein_coding 1.687 43.939
0.708 79.475 0.837 83.607 0.898 91.872
ENSMUSG00000063427 Rps10-ps2 pseudogene 1 73.734 1
95.708 1 80.899 1 92.181
ENSMUSG00000063428 Ddo protein_coding 0.899 24.748 0.986
96.216 1.124 37.369 0.972 96.248
ENSMUSG00000063430 Wscd2 protein_coding 1.036 83.523 1.299
81.54 0.744 31.887 1.006 91.335
ENSMUSG00000063434 Sorcs3 protein_coding 1.57 36.195 NA
NA 0.508 27.321 0.972 90.463
ENSMUSG00000063439 B9d2 protein_coding 1.001 75.761 1.022
86.277 0.977 67.962 0.989 94.904
ENSMUSG00000063442 Gm7117 pseudogene 1.369 66.944 0.572
66.796 1.28 51.596 0.837 93.156
ENSMUSG00000063445 Nmral1 protein_coding 1.166 20.032
0.908 82.947 0.947 55.9 1.225 57.839
ENSMUSG00000063447 Ube2d2b protein_coding 1 83.333 1
91.532 1 82.778 1 87.7
ENSMUSG00000063450 Syne2 protein_coding 1.062 44.694 0.902
34.058 1.041 27.533 0.941 85.493
ENSMUSG00000063452 Olfr800 protein_coding 1 74.147 1
96.843 1 87.898 1 96.287
ENSMUSG00000063455 D630045J12Rik protein_coding 1.111 75.115
0.914 93.654 0.986 79.325 0.749 86.991
ENSMUSG00000063457 Rps15 protein_coding 0.927 32.749 0.988
90.378 1.091 17.412 0.971 85.493
ENSMUSG00000063458 1700112E06Rik protein_coding 0.983 83.333
0.818 60.678 1.239 21.522 1.058 90.125
ENSMUSG00000063478 Tas2r114 protein_coding 1 73.124 1
96.138 1 72.695 1 96.248
ENSMUSG00000063480 Nhp2l1 protein_coding 1.09 11.459
0.954 73.766 0.963 46.956 1.072 68.696
ENSMUSG00000063488 NA NA NA NA 0.996 85.242 1.085
45.667 0.975 93.264
ENSMUSG00000063506 Arhgap22 protein_coding 0.966 83.09 NA NA
NA NA 0.892 83.724
ENSMUSG00000063507 Gm1330 protein_coding 1 85.391 1
96.847 1 79.426 1 95.72
ENSMUSG00000063511 Snrnp70 protein_coding 0.814 1.175 1.066
90.454 1.147 5.964 1.028 92.381
ENSMUSG00000063514 Gm6756 pseudogene 1 84.324 NA NA
1 86.727 1 96.183
ENSMUSG00000063522 2010109I03Rik protein_coding 0.726 49.368

0.745 83.321 1.85 27.533 0.991 90.95
ENSMUSG00000063524 Eno1 protein_coding 0.55 1.274 1.102 88.136
1.656 6.202 1.109 91.928
ENSMUSG00000063529 Stmnd1 protein_coding 1.121 83.169
0.806 93.654 1.109 84.724 0.705 87.735
ENSMUSG00000063531 Sema3e protein_coding 0.855 27.431
0.743 68.115 1.574 9.502 1.051 96.086
ENSMUSG00000063535 Zfp773 protein_coding 1.043 56.085
1.065 94.367 0.898 56.822 0.856 85.493
ENSMUSG00000063543 Gm5616 protein_coding 1 85.59 1
96.268 1 88.601 1 96.358
ENSMUSG00000063548 Gm4825 protein_coding 1 86.658 1
90.378 1 57.637 1 89.052
ENSMUSG00000063549 Olfr322 protein_coding 1 86.74 1
97.288 1 87.37 1 95.777
ENSMUSG00000063550 Nup98 protein_coding 1.145 4.228 0.965 78.471
0.905 16.643 0.966 93.734
ENSMUSG00000063556 Gm10132 protein_coding 1 68.955 NA
NA 1 88.161 1 95.559
ENSMUSG00000063558 Aox1 protein_coding 0.996 62.888 0.722
94.262 1.385 23.454 1.04 91.525
ENSMUSG00000063564 Col23a1 protein_coding 1 80.608
0.991 95.708 1.007 84.229 1.078 72.369
ENSMUSG00000063568 Jazf1 protein_coding 0.954 69.943 1.171
80.775 0.897 65.409 1.087 83.581
ENSMUSG00000063576 Klhdc3 protein_coding 0.889 0.987 1.05
71.98 1.075 24.383 1.046 59.464
ENSMUSG00000063582 Olfr666 protein_coding 1 74.8 1
92.474 1 67.962 1 83.067
ENSMUSG00000063583 Olfr1410 protein_coding 1 74.482 1
94.48 1 80.381 1 95.405
ENSMUSG00000063586 Gm5513 pseudogene 1 77.113 1
93.765 1 82.685 1 96.042
ENSMUSG00000063590 Slc22a28 protein_coding 1 74.745 NA
NA 1 76.388 1 92.181
ENSMUSG00000063594 Gng8 protein_coding 0.877 64.893 1.415
81.039 0.807 57.446 1.089 95.74
ENSMUSG00000063600 Egfem1 protein_coding 0.871 81.925
0.743 85.418 1.539 39.925 1.374 94.016
ENSMUSG00000063605 Ccdc102a protein_coding 1.02 83.09 1.046
88.551 0.939 56.365 1.06 65.558
ENSMUSG00000063611 Gm10134 protein_coding 1.569 2.651 0.97
94.102 0.656 14.409 1.229 65.558
ENSMUSG00000063615 Olfr64 protein_coding 1 81.01 1
90.287 1 87.971 1 95.676
ENSMUSG00000063623 C230062I16Rik protein_coding 0.797 59.028
1.24 95.196 1.013 67.163 0.829 91.217
ENSMUSG00000063628 Gm7665 pseudogene 1 80.751 1
90.053 1 85.935 1 93.722
ENSMUSG00000063632 Sox11 protein_coding 1.302 73.401 0.988
96.948 0.778 80.015 1.009 95.35
ENSMUSG00000063646 Jakmip1 protein_coding 1.097 73.734 1.15
88.533 0.793 65.409 0.96 90.95
ENSMUSG00000063651 Cnfn protein_coding 1 84.263 1

92.355	1	87.37 1	92.851
ENSMUSG00000063652	Slc22a21	protein_coding	1.072 73.401
0.892 91.361	1.043 86.602	0.88	83.724
ENSMUSG00000063656	Gm10135	protein_coding	0.809 77.113
0.849 93.094	1.454 56.497	1.075 94.016
ENSMUSG00000063659	Zbtb18	protein_coding	1.003 85.832
1.086 75.62 0.919 3.943 0.963 87.735
ENSMUSG00000063660	Olfr98	protein_coding	1	50.377	1
91.661	1	86.831	1	96.287
ENSMUSG00000063661	Krt73 protein_coding	1.273 73.401	0.232
40.463	3.386 31.317	1.4	87.886
ENSMUSG00000063663	Brwd3 protein_coding	0.959 42.361	0.807
79.475	1.295 16.808	1.038 96.358
ENSMUSG00000063672	Nkx6-3	protein_coding	1	80.408	1
96.216	1	87.274	1	82.091
ENSMUSG00000063681	Crb1	protein_coding	1	82.231	1
83.321	1	81.032	1	96.139
ENSMUSG00000063683	Glyat protein_coding	1.637 74.745	0.445
79.475	1.369 61.841	1.692 89.108
ENSMUSG00000063684	Gm13910	pseudogene 0.978 68.727	0.843
93.817	1.209 85.792	1.077 94.904
ENSMUSG00000063687	Pcdhb5	protein_coding	1.14	51.737
1.129 79.29 0.773 4.389 1.029 84.499
ENSMUSG00000063689	Hist2h2ab	protein_coding	1.182 83.418
0.516 90.053	1.637 43.586	1.406 93.132
ENSMUSG00000063694	Cycs	protein_coding	0.779 0.987 1.107 90.29
1.158 15.241	1.104 83.067
ENSMUSG00000063698	Sfxn4 protein_coding	0.958 61.043	0.96
96.216	1.086 62.995	1.08	83.067
ENSMUSG00000063704	Mapk15	protein_coding	0.957 77.763
0.859 85.793	1.218 54.673	0.646 83.724
ENSMUSG00000063713	Klk1b24	protein_coding	1	71.763	1
95.735	1	86.346	1	91.928
ENSMUSG00000063714	1700011J10Rik	antisense	0.931 80.608
1.164 97.049	0.926 84.552	0.949 84.29
ENSMUSG00000063715	Olfr816	protein_coding	1	84.324	1
93.817	1	81.525	1	90.177
ENSMUSG00000063727	Tnfrsf11b	protein_coding	1.153 34.861
1.183 83.104	0.734 6.858 0.853 85.493
ENSMUSG00000063728	Magea6	protein_coding	1	84.534	1
94.96 1	82.685	1	95.74
ENSMUSG00000063730	Hsd3b2	protein_coding	1	83.658	1
92.404	NA	NA	1	95.273
ENSMUSG00000063732	Olfr908	pseudogene 1	86.74 1	96.897
1	84.961	1	93.457
ENSMUSG00000063739	Gm4963	pseudogene 1	80.815	1
92.137	1	84.229	1	93.457
ENSMUSG00000063751	Gm13939	processed_transcript	1	80.815
1	94.262	1	76.64 1	95.74
ENSMUSG00000063754	Gm10136	pseudogene 1.117 83.726	0.839
94.887	1.066 60.789	0.923 95.777
ENSMUSG00000063757	4632433K11Rik	protein_coding	0.856 33.745
0.883 68.818	1.326 8.93	0.93	95.92
ENSMUSG00000063760	Rnf217	protein_coding	0.947 84.263

0.962 95.088 1.094 86.144 0.934 87.542
ENSMUSG00000063762 Tas2r129 protein_coding 1 82.231 NA
NA 1 84.896 1 95.273
ENSMUSG00000063764 Olfr508 protein_coding 1 86.464 1
91.661 1 87.898 1 94.153
ENSMUSG00000063765 Chadl protein_coding 0.789 50.768 1.187
81.127 1.068 81.29 1.065 95.72
ENSMUSG00000063767 S100a7a protein_coding 0.753 32.557
1.032 96.897 1.289 59.612 1.116 90.674
ENSMUSG00000063777 Olfr1469 protein_coding 1 85.655 NA
NA 1 87.786 1 96.139
ENSMUSG00000063779 Chi3l4 protein_coding 1 72.85 1
96.407 1 67.339 1 86.991
ENSMUSG00000063780 Olfr282 protein_coding 1 79.165 1
94.767 1 86.258 1 95.877
ENSMUSG00000063785 Utp14a protein_coding 1.024 81.76 0.921
71.331 1.061 43.307 1.077 60.466
ENSMUSG00000063787 Chchd1 protein_coding 0.977 57.227
0.962 82.848 1.061 27.732 1.055 55.841
ENSMUSG00000063800 Prpf38a protein_coding 1.062 32.676
0.878 40.946 1.075 49.601 1.004 93.117
ENSMUSG00000063801 Ap3s2 protein_coding 0.968 23.319 0.997
97.155 1.033 28.301 0.979 96.086
ENSMUSG00000063802 Hspbp1 protein_coding 0.887 7.71 1.047
88.136 1.078 36.95 1.016 69.404
ENSMUSG00000063804 Lin28b protein_coding 1 71.915 1
95.735 1 88.161 1 91.372
ENSMUSG00000063808 Gpatch1 protein_coding 1.011 81.178 0.94
89.713 1.056 69.002 0.939 84.29
ENSMUSG00000063810 Alms1 protein_coding 0.994 77.497 0.976
97.263 1.03 87.971 0.942 85.493
ENSMUSG00000063816 Gm4987 pseudogene 1 75.761 NA NA
1 88.349 1 93.156
ENSMUSG00000063820 Arl9 protein_coding 0.837 56.768 1.162
95.735 1.026 88.601 0.84 87.651
ENSMUSG00000063821 Dupd1 protein_coding 2.392 37.091 1.687
97.049 0.248 36.499 1.77 63.018
ENSMUSG00000063823 Olfr532 protein_coding 1 82.092 1
97.288 1 87.744 1 96.287
ENSMUSG00000063827 Olfr1382 protein_coding 1 81.647 1
96.138 NA NA 1 93.964
ENSMUSG00000063830 Gm6065 pseudogene 1 86.172 1
97.155 1 88.274 1 94.82
ENSMUSG00000063838 Cdc42ep5 protein_coding 1.047 66.76 0.988
96.013 0.963 85.675 1.057 64.513
ENSMUSG00000063842 Olfr862 protein_coding 1 85.655 1
96.216 1 86.144 1 94.016
ENSMUSG00000063844 Olfr1276 protein_coding 1 85.283 1
89.996 NA NA 1 94.654
ENSMUSG00000063846 Gm21731 protein_coding 1 85.728 NA
NA 1 85.935 1 95.74
ENSMUSG00000063849 Ppcdc protein_coding 0.997 58.198 0.956
87.998 1.051 88.461 1.023 96.183
ENSMUSG00000063856 Gpx1 protein_coding 1.131 1.949 0.896 26.053

0.983 84.626 1.031 94.016
ENSMUSG00000063867 Olfr1511 protein_coding 1 86.819 1
92.914 1 84.961 1 96.248
ENSMUSG00000063870 Chd4 protein_coding 0.977 36.945 1.012
92.561 1.014 81.492 0.955 88.387
ENSMUSG00000063873 Slc24a3 protein_coding 0.771 2.142 1.02
95.431 1.272 10.281 1.045 83.067
ENSMUSG00000063881 NA NA NA NA 1 93.654 1
84.896 1 94.468
ENSMUSG00000063882 Uqcrh protein_coding 0.939 34.572 1.001
93.654 1.061 40.575 1.035 82.328
ENSMUSG00000063884 Ptcd3 protein_coding 1.035 67.163 0.98
94.102 0.984 87.103 1.019 80.187
ENSMUSG00000063885 Gm6498 pseudogene 0.892 79.709 0.74
85.793 1.519 32.173 1.191 83.067
ENSMUSG00000063886 Gm10138 pseudogene 0.938 55.939 0.945
91.532 1.126 45.667 0.782 83.724
ENSMUSG00000063887 Nlgn1 protein_coding 1.504 63.804 0.794
95.088 0.837 82.575 0.746 96.358
ENSMUSG00000063888 Rpl7l1 protein_coding 1.064 26.77 1.054
95.211 0.893 23.48 1.046 60.31
ENSMUSG00000063889 Crem protein_coding 0.954 38.195 1.092
71.617 0.956 50.184 0.977 86.699
ENSMUSG00000063894 Zkscan8 protein_coding 0.82 1.017 NA NA
1.165 5.864 1.046 91.011
ENSMUSG00000063895 Nupl1 protein_coding 1.089 13.292 0.927
89.713 0.993 46.956 1.041 93.016
ENSMUSG00000063900 Adam26b protein_coding 1 84.977 1
94.102 1 88.657 1 93.734
ENSMUSG00000063902 Gm7964 protein_coding 1.23 1.188 0.93
72.712 0.872 19.449 1.051 50.74
ENSMUSG00000063903 Klk1 protein_coding 3.615 15.662 0.482
94.96 0.575 23.1 0.529 84.29
ENSMUSG00000063904 Dpp3 protein_coding 0.93 32.168 1.029
95.211 1.045 47.776 1.072 44.679
ENSMUSG00000063905 Gm10139 protein_coding 1 84.738 1
96.268 1 85.464 1 96.287
ENSMUSG00000063911 Tdgf1-ps2 pseudogene 1 81.24 1 95.708
1 82.31 1 96.086
ENSMUSG00000063919 Srrm4 protein_coding 0.86 34.954 NA NA
1.178 41.331 1.15 83.067
ENSMUSG00000063929 Cyp4a32 protein_coding 0.7 60.569 0.64
80.052 2.238 21.705 0.777 86.991
ENSMUSG00000063931 Pepd protein_coding 0.871 60.003 1.126
93.994 1.02 76.498 1.025 95.832
ENSMUSG00000063932 Poteg protein_coding 1 64.714 1
90.256 1 79.325 1 94.654
ENSMUSG00000063935 Zar1 protein_coding 1 75.858 1
96.68 1 86.552 1 94.82
ENSMUSG00000063952 Brpf3 protein_coding 0.99 57.818 1.018
82.904 0.989 87.898 0.919 84.19
ENSMUSG00000063954 Hist2h2aa1 protein_coding 1 66.58 1
96.138 1 75.637 1 92.31
ENSMUSG00000063971 1700011A15Rik protein_coding 0.547 69.985

0.64 74.483 2.862 20.315 1.767 86.133
ENSMUSG00000063972 Nr6a1 protein_coding 1.027 68.485 0.961
75.345 1.012 69.728 0.902 86.991
ENSMUSG00000063975 Slco1a5 protein_coding 1.102 76.827
0.888 85.075 1.021 86.666 0.915 84.29
ENSMUSG00000063994 Olfr120 protein_coding 1 83.263 NA
NA 1 84.896 1 92.381
ENSMUSG00000063998 Vmn1r211 protein_coding 1 85.283 1
95.301 1 81.388 1 92.018
ENSMUSG00000064006 Olfr190 protein_coding 1 79.277 NA
NA 1 81.857 1 96.287
ENSMUSG00000064010 Dppa1 protein_coding 1 76.396 1
95.211 1 81.976 1 83.067
ENSMUSG00000064023 Klk8 protein_coding 1.013 86.145 1.004
96.847 0.984 70.791 0.909 88.505
ENSMUSG00000064030 Wibg protein_coding 0.9 4.993 0.932 67.941
1.191 5.864 0.963 95.72
ENSMUSG00000064032 Gm10143 processed_transcript 0.979 59.358
1.135 90.29 0.896 81.525 1.202 46.412
ENSMUSG00000064036 Mro protein_coding 0.339 43.435 0.756
87.313 3.89 21.201 1.779 79.268
ENSMUSG00000064037 Gpn1 protein_coding 1.009 78.744 1.005
90.895 0.987 63.919 1.028 44.679
ENSMUSG00000064039 Ccrl1l protein_coding 4.518 5.253 0.609
94.262 0.364 6.858 0.459 83.724
ENSMUSG00000064043 Trerf1 protein_coding 1.223 0.999 0.912
88.691 0.895 17.898 1.04 94.752
ENSMUSG00000064044 Olfr319 protein_coding 1 85.946 1
96.652 1 86.666 1 96.287
ENSMUSG00000064052 Gm5089 protein_coding 1 77.497 1
96.763 1 86.112 1 94.153
ENSMUSG00000064057 Scgb3a1 protein_coding 0.499 58.264 NA
NA 1.694 62.648 1.834 67.691
ENSMUSG00000064061 Dzip3 protein_coding 1.004 46.413 0.967
96.68 1.033 73.428 1.011 78.948
ENSMUSG00000064065 Ipcef1 protein_coding 1.149 29.536
1.155 83.104 0.753 20.751 0.893 83.724
ENSMUSG00000064068 Mtx1 protein_coding 0.91 2.536 1.088 66.01
1.012 51.851 0.953 96.192
ENSMUSG00000064080 Fbln2 protein_coding 1.12 0 0.891 79.475
0.999 52.213 1.145 49.514
ENSMUSG00000064081 Gm8973 protein_coding 1 49.868 1
97.263 1 48.133 1 86.699
ENSMUSG00000064084 Olfr1202 protein_coding 1 77.615 1
96.652 1 85.921 1 96.086
ENSMUSG00000064090 Vrk2 protein_coding 1.125 22.633 1.002
95.708 0.885 22.864 0.978 96.086
ENSMUSG00000064105 Cnnm2 protein_coding 0.892 5.778 1.077 83.104
1.041 30.292 0.919 94.82
ENSMUSG00000064109 Hcst protein_coding 1.185 49.868 0.737
79.807 1.147 84.83 0.954 91.105
ENSMUSG00000064110 Olfr963 protein_coding 1 72.655 NA
NA 1 88.076 1 93.156
ENSMUSG00000064115 Cadm2 protein_coding 0.888 26.217 0.957

90.053 1.175 31.479 1.13 66.468
ENSMUSG00000064120 Mocs1 protein_coding 0.959 59.028 0.855
89.713 1.218 44.125 0.989 94.468
ENSMUSG00000064121 Olfr96 protein_coding 1 82.732 1
92.137 1 83.275 1 89.498
ENSMUSG00000064125 BC068157 protein_coding 0.639 1.449 1.119
92.062 1.401 11.348 0.929 85.493
ENSMUSG00000064127 Med14 protein_coding 1.001 85.59 0.985 91.361
1.01 79.325 1.004 95.74
ENSMUSG00000064128 Cenpj protein_coding 1.072 19.132 0.991
88.136 0.943 8.142 1.043 86.301
ENSMUSG00000064129 Gm14781 protein_coding 1 78.538 1
96.847 NA NA 1 96.358
ENSMUSG00000064137 Rhox8 protein_coding 2.596 50.111 0.244
70.971 1.577 61.171 1.492 86.445
ENSMUSG00000064138 Fam172a protein_coding 1.142 0.355 0.982
78.51 0.892 4.723 0.99 87.218
ENSMUSG00000064140 Trim38 protein_coding 1.47 83.169 1.1
95.504 0.616 87.786 1.066 93.734
ENSMUSG00000064141 Zfp69 protein_coding 0.7 1.698 1.048 94.367
1.367 6.202 0.987 91.872
ENSMUSG00000064145 Arih2 protein_coding 1.035 64.714 0.992
95.708 0.975 55.9 1 91.872
ENSMUSG00000064147 Rab44 protein_coding 1.368 2.655 0.797 84.594
0.917 9.901 1.006 96.287
ENSMUSG00000064158 Izumo1 protein_coding 1.065 85.655
0.978 90.116 0.957 74.853 0.907 94.174
ENSMUSG00000064165 Krt39 protein_coding 1 84.168 1
96.984 1 88.693 1 95.777
ENSMUSG00000064168 Hist1h2bh protein_coding 0.807 69.24 1.175
96.68 1.057 86.727 0.472 84.29
ENSMUSG00000064177 Ghrl protein_coding 0.608 35.894 1.048
95.708 1.566 40.575 1.047 96.183
ENSMUSG00000064179 Tnnt1 protein_coding 1.231 54.717 1.156
88.823 0.701 21.331 1.068 77.245
ENSMUSG00000064181 Rab3ip protein_coding 0.932 42.034
1.038 79.411 1.035 84.308 1.064 95.273
ENSMUSG00000064194 Zfp936 protein_coding 1 76.135 1
91.532 1 84.896 1 96.248
ENSMUSG00000064201 Krt2 protein_coding 1 83.169 1
95.504 1 87.336 1 94.99
ENSMUSG00000064202 4430402I18Rik protein_coding 0.827 7.857
1.189 72.642 1.019 67.562 0.975 94.99
ENSMUSG00000064208 Gm10145 pseudogene 1 86.112 1
95.504 1 86.727 1 86.445
ENSMUSG00000064210 Ano6 protein_coding 1.039 33.854 1.025
95.35 0.942 40.794 1.006 96.287
ENSMUSG00000064213 Defa24 protein_coding 1 86.658 1
95.196 1 81.492 1 96.042
ENSMUSG00000064215 Ifi27 protein_coding 0.806 0.987 1.066 71.617
1.157 9.301 0.989 91.269
ENSMUSG00000064220 Hist2h2aa2 protein_coding 0.646 32.378
1.296 79.864 1.191 83.066 1.204 96.358
ENSMUSG00000064223 Olfr694 protein_coding 1 80.408 1

95.708	NA	NA	1	96.184
ENSMUSG00000064224	Gsdma3	protein_coding	1.513 83.418	0.23
87.147	2.88	56.497	1.465 88.111
ENSMUSG00000064225	Paqr9 protein_coding	0.851 7.887 1.392 60.309
0.841 23.1	0.992 90.95
ENSMUSG00000064231	Gm5321	pseudogene 1	79.577	1
96.138	1	87.431	1	95.92
ENSMUSG00000064232	Gm5414	protein_coding	1	86.57 1
95.301	1	71.999	1	96.248
ENSMUSG00000064243	Vmn1r205	protein_coding	1	85.77 NA	NA
1	85.935	1	93.584
ENSMUSG00000064246	Chi3l1	protein_coding	1.209 59.211
0.655 67.513	1.258 86.602	0.556 83.724
ENSMUSG00000064247	Plcxd1	protein_coding	0.792 4.895 1.107
91.584	1.137 20.315	0.939 85.493
ENSMUSG00000064252	Olfr329-ps	pseudogene 1	75.761	1
96.843	1	83.925	1	93.734
ENSMUSG00000064254	Ethe1 protein_coding	1.104 35.748	NA	NA
0.901 40.19 1.062 91.525
ENSMUSG00000064259	Vmn1r13	protein_coding	1	84.661	1
94.597	1	85.464	1	94.384
ENSMUSG00000064262	Gimap8	protein_coding	0.962 64.589
1.105 80.678	0.939 26.369	0.949 86.991
ENSMUSG00000064264	Zfp428	protein_coding	0.793 15.486
1.143 80.775	1.099 69.315	0.978 88.053
ENSMUSG00000064266	NA	NA	NA	NA	1	97.155	1
87.37 1	96.248
ENSMUSG00000064267	Hvcn1 protein_coding	0.938 79.433	1.143
77.598	0.934 51.443	0.943 84.29
ENSMUSG00000064272	Gpbar1	protein_coding	0.729 77.763
1.209 96.013	1.132 81.857	0.906 93.264
ENSMUSG00000064281	Rpl19-ps1	pseudogene 1	78.538	1
95.329	1	74.686	1	94.001
ENSMUSG00000064284	Cdpf1 protein_coding	0.959 26.013	1.02
94.367	1.02	38.152	1.06	48.857
ENSMUSG00000064288	Hist1h4k	protein_coding	0.796 54.983
0.984 83.104	NA	NA	0.797 85.493
ENSMUSG00000064289	Tank	protein_coding	0.932 42.034	1.06
88.092	1.016 88.41 1.004 96.287
ENSMUSG00000064294	Aox3	protein_coding	1.283 48.857	0.745
89.996	1.04	70.1	1.124 95.273
ENSMUSG00000064299	4921528I07Rik	protein_coding	1	80.03 1
91.955	1	78.369	1	83.067
ENSMUSG00000064302	Clasp1	protein_coding	0.92	7.58	1.059
60.309	1.024 80.381	1.048 76.831
ENSMUSG00000064307	Lrrc51	protein_coding	1.008 75.761	NA
NA	0.975 69.315	0.885 85.493
ENSMUSG00000064310	Zpld1 protein_coding	1	81.178	1
96.216	1	87.744	1	96.358
ENSMUSG00000064315	Rpl21-ps10	pseudogene 1	76.827	1
94.597	1	75.809	1	96.248
ENSMUSG00000064325	Hhip	protein_coding	1.301 18.556	1.411
83.104	0.543 9.901 1.194 66.944
ENSMUSG00000064326	Siva1 protein_coding	1.157 15.602	0.897

79.475 0.961 62.791 1.065 90.397
ENSMUSG00000064333 Olfr926 protein_coding 1 84.738 1
93.899 1 87.898 1 95.74
ENSMUSG00000064336 mt-Tf Mt_tRNA 1 82.982 NA NA 1
80.163 1 95.273
ENSMUSG00000064337 mt-Rnr1 Mt_rRNA 1.194 39.416 0.825
87.452 1.016 83.397 1.015 95.676
ENSMUSG00000064338 mt-Tv Mt_tRNA 1 84.397 1 96.216
NA NA 1 88.755
ENSMUSG00000064339 mt-Rnr2 Mt_rRNA 1.151 30.984 0.922
89.713 0.938 54.25 1.062 71.855
ENSMUSG00000064340 mt-Tl1 Mt_tRNA 1 85.832 NA NA
1 88.657 1 95.74
ENSMUSG00000064341 mt-Nd1 protein_coding 0.93 21.658 1.05
82.904 1.026 76.64 0.986 93.964
ENSMUSG00000064342 mt-Ti Mt_tRNA 1 86.145 1 96.407
1 88.349 1 95.273
ENSMUSG00000064343 mt-Tq Mt_tRNA 0.398 5.591 0.82 92.137
3.069 5.864 0.785 94.99
ENSMUSG00000064344 mt-Tm Mt_tRNA 1 83.418 1 95.329
1 83.857 1 96.071
ENSMUSG00000064345 mt-Nd2 protein_coding 0.915 34.305
1.075 81.54 1.019 87.744 0.965 92.181
ENSMUSG00000064346 mt-Tw Mt_tRNA 0.585 8.122 0.893 95.504
1.918 13.259 1.049 96.358
ENSMUSG00000064348 mt-Tn Mt_tRNA 1 72.546 1 97.049
1 81.29 1 95.273
ENSMUSG00000064349 mt-Tc Mt_tRNA 1 85.728 1 96.013
NA NA 1 94.297
ENSMUSG00000064351 mt-Co1 protein_coding 0.86 5.946 0.966
96.216 1.199 29.819 0.988 95.832
ENSMUSG00000064352 mt-Ts1 Mt_tRNA 1 66.406 NA NA
1 80.089 1 88.755
ENSMUSG00000064353 mt-Td Mt_tRNA 1 86.172 1 93.908
1 82.436 1 96.358
ENSMUSG00000064354 mt-Co2 protein_coding 0.72 30.035
1.051 96.68 1.318 60.104 1.237 93.457
ENSMUSG00000064355 mt-Tk Mt_tRNA 1 85.092 1 92.474
1 87.103 1 96.358
ENSMUSG00000064357 mt-Atp6 protein_coding 0.659 32.168
1.398 79.29 1.087 86.552 0.868 88.136
ENSMUSG00000064358 mt-Co3 protein_coding 0.665 4.874 0.896
86.728 1.673 7.24 1.18 92.389
ENSMUSG00000064359 mt-Tg Mt_tRNA 1 75.567 1 94.102
1 72.695 1 95.92
ENSMUSG00000064360 mt-Nd3 protein_coding 0.786 59.674
0.725 87.313 1.753 40.052 1.585 90.397
ENSMUSG00000064361 mt-Tr Mt_tRNA 1 86.819 1 96.948
1 87.431 1 94.82
ENSMUSG00000064363 mt-Nd4 protein_coding 0.94 46.693
1.029 87.282 NA NA 0.916 85.493
ENSMUSG00000064364 mt-Th Mt_tRNA 1 82.092 1 97.288
1 79.325 1 95.74
ENSMUSG00000064365 mt-Ts2 Mt_tRNA 1 82.092 1

96.652 1 84.724 1 96.183
ENSMUSG00000064366 mt-Tl2 Mt_tRNA 1 71.36 1 91.361
1 81.857 1 94.82
ENSMUSG00000064367 mt-Nd5 protein_coding 0.927 17.839 1.08
82.904 0.999 81.525 0.962 93.261
ENSMUSG00000064368 mt-Nd6 protein_coding 0.459 0.987 0.859
91.065 2.536 5.864 0.968 83.952
ENSMUSG00000064369 mt-Te Mt_tRNA 0.496 34.305 0.923 87.998
2.177 20.315 NA NA
ENSMUSG00000064370 mt-Cytb protein_coding 0.814 0.987 1.039
84.999 1.177 61.743 0.952 94.99
ENSMUSG00000064371 mt-Tt Mt_tRNA 2.536 0.664 0.671 93.654
0.589 2.563 0.917 85.493
ENSMUSG00000064372 mt-Tp Mt_tRNA 0.667 8.938 1.048 88.823
1.427 32.433 1.023 91.979
ENSMUSG00000064373 Sepp1 protein_coding 1.295 1.846 0.86 39.056
0.894 54.964 0.867 87.705
ENSMUSG00000064377 Gm24966 snoRNA 1 86.351 NA NA
1 84.724 1 96.248
ENSMUSG00000064380 Gm26448 snoRNA 0.901 63.736 1.006
95.211 1.105 48.304 1.284 73.823
ENSMUSG00000064382 Gm26447 snoRNA 1 82.092 1
95.211 1 85.921 1 94.904
ENSMUSG00000064386 Gm26449 snRNA 1 86.658 1 93.863
1 85.935 1 93.132
ENSMUSG00000064390 Rnu73b snoRNA 1.135 69.777 0.906
95.211 0.973 75.967 0.909 89.892
ENSMUSG00000064391 Gm23625 snRNA 1 86.474 1 95.088
1 82.778 1 95.273
ENSMUSG00000064392 Gm23626 snoRNA 1 84.534 1
97.263 1 86.666 1 93.722
ENSMUSG00000064393 Gm23627 snRNA 1 68.16 1 97.263 1
79.897 1 94.153
ENSMUSG00000064394 Gm23622 snRNA 1 81.647 1 96.763
1 86.258 1 96.192
ENSMUSG00000064395 Gm23623 snRNA 1 79.93 1 96.268 1
86.112 1 96.071
ENSMUSG00000064397 Gm23624 misc_RNA 1 79.93 1 93.899
1 84.434 1 96.287
ENSMUSG00000064399 Gm23629 snRNA 1 85.499 1 96.847
1 85.921 1 92.748
ENSMUSG00000064400 Gm23927 snoRNA 1 86.172 NA NA
1 83.538 1 96.287
ENSMUSG00000064403 Gm23928 snRNA 1 71.516 NA NA 1
87.744 1 94.82
ENSMUSG00000064405 Gm23925 snoRNA 1 79.853 1
95.211 1 86.552 1 96.287
ENSMUSG00000064407 Gm23926 snRNA 1 85.857 1 96.843
NA NA 1 92.903
ENSMUSG00000064408 Gm23924 snRNA 1 79.277 1 96.897
1 80.089 1 95.877
ENSMUSG00000064409 n-R5s40 rRNA 1.557 46.013 0.469 84.017
1.367 84.724 0.745 89.475
ENSMUSG00000064410 Gm22247 snoRNA 1 74.689 1

90.29 1 80.015 1 93.132
ENSMUSG00000064412 Gm22246 snRNA 1 85.655 1 94.48 1
70.328 1 93.316
ENSMUSG00000064413 Gm22245 snRNA 1 79.433 NA NA 1
87.336 1 96.183
ENSMUSG00000064414 n-R5s215 rRNA 1 81.01 NA NA 1
86.552 1 90.455
ENSMUSG00000064416 Gm22248 snRNA 1 77.113 1 91.361
1 77.888 1 96.086
ENSMUSG00000064417 DQ267102 snoRNA 1.397 47.847 1.679
87.609 0.427 23.86 0.429 86.699
ENSMUSG00000064422 Gm22502 snRNA 1 85.129 1 97.049
1 85.757 1 95.676
ENSMUSG00000064424 Gm22749 snRNA 1 73.841 1 95.088
1 70.623 1 95.273
ENSMUSG00000064427 Gm22748 snoRNA 0.951 84.564 3.954
83.104 0.265 19.236 0.457 88.505
ENSMUSG00000064428 Gm22746 snRNA 1 85.129 1 97.097
1 88.601 1 91.217
ENSMUSG00000064429 Gm22747 snRNA 1 81.657 NA NA 1
86.475 1 96.071
ENSMUSG00000064432 Gm25587 snRNA 1 75.761 1 96.984
1 83.925 1 94.82
ENSMUSG00000064433 Gm25586 misc_RNA 1 85.728 1
97.155 1 74.179 1 94.016
ENSMUSG00000064435 Gm25588 snoRNA 1 80.688 1
94.597 1 87.658 1 93.685
ENSMUSG00000064436 Gm25589 snRNA 1 83.968 1 95.941
1 84.83 1 93.612
ENSMUSG00000064440 Gm26224 snoRNA 1 86.474 1
93.654 1 87.658 1 95.273
ENSMUSG00000064441 Snord37 snoRNA 0.741 15.06 0.825 97.155
1.636 39.925 0.849 95.35
ENSMUSG00000064443 Gm26226 snRNA 0.884 66.129 1.154 85.756
0.977 69.445 0.955 94.904
ENSMUSG00000064444 Gm26227 snRNA 1 74.147 1 95.329
1 85.21 1 95.92
ENSMUSG00000064446 Gm26228 snRNA 1 86.464 1 96.897
1 83.275 1 96.139
ENSMUSG00000064450 Snord68 snoRNA 1 84.661 1
96.138 1 83.158 1 94.654
ENSMUSG00000064451 Snora23 snoRNA 0.968 85.283 1.219
93.863 0.845 88.203 0.753 93.964
ENSMUSG00000064453 Snord21 snoRNA 1 77.916 1
97.097 1 85.58 1 92.263
ENSMUSG00000064455 n-R5s56 rRNA 1 84.263 1 96.013
1 84.724 1 96.071
ENSMUSG00000064460 Gm25080 snRNA 1 84.564 NA NA 1
86.144 1 95.273
ENSMUSG00000064463 Gm25079 snoRNA 1 86.474 1
95.708 1 87.744 1 92.389
ENSMUSG00000064464 Gm25078 snRNA 1 84.977 1 95.708
1 87.786 1 92.181
ENSMUSG00000064467 n-R5s37 rRNA 1 80.51 1 96.847 1

86.144 1 96.287
ENSMUSG00000064468 Gm25081 snRNA 1 78.538 1 95.088
1 88.601 1 95.72
ENSMUSG00000064471 Gm23406 snoRNA 1 81.24 1 97.155
1 86.985 1 95.74
ENSMUSG00000064476 Gm23405 snRNA 1 76.042 NA NA NA
NA 1 94.904
ENSMUSG00000064477 Gm23404 snoRNA 1 69.489 1
96.407 1 71.232 1 96.248
ENSMUSG00000064481 Gm23737 snRNA 1 79.709 1 96.843
1 87.971 1 95.877
ENSMUSG00000064485 Gm23735 snRNA 1 85.832 1 96.847
1 78.864 1 94.215
ENSMUSG00000064486 Gm23293 snRNA 1 84.819 1 96.138
1 86.144 1 96.287
ENSMUSG00000064487 Gm23736 snoRNA 1 80.608 1
97.155 NA NA 1 93.132
ENSMUSG00000064489 Gm23296 snRNA 1 74.689 1 95.735
1 72.464 1 94.608
ENSMUSG00000064490 Gm22283 snRNA 1 83.823 1 95.504
1 87.971 1 96.183
ENSMUSG00000064493 Snora28 snoRNA 1.166 29.811 0.957
94.89 0.897 47.641 0.982 92.851
ENSMUSG00000064494 Gm22063 snRNA 1.809 56.085 0.334 57.613
1.655 50.851 1.139 81.901
ENSMUSG00000064496 DQ267101 snoRNA 1 84.661 1
97.155 1 84.308 1 95.273
ENSMUSG00000064497 Gm22062 snRNA 1 83.418 1 96.652
1 88.601 1 94.904
ENSMUSG00000064500 Gm25296 snoRNA 1 79.853 1
97.129 1 87.744 1 96.192
ENSMUSG00000064507 n-R5s94 rRNA 1 86.843 NA NA 1
84.504 1 96.183
ENSMUSG00000064510 Gm22458 snRNA 1 78.744 1 95.735
1 87.971 1 94.904
ENSMUSG00000064511 Gm22459 snoRNA 1 80.51 1 93.899
1 87.786 1 92.851
ENSMUSG00000064512 Gm22456 snRNA 1 73.487 1 96.843
1 87.786 1 90.854
ENSMUSG00000064513 Gm22457 snoRNA 1 82.543 1
91.532 1 88.161 1 95.72
ENSMUSG00000064514 Gm22455 snoRNA 1.712 12.396 0.652
88.345 0.893 63.075 1.089 83.581
ENSMUSG00000064515 n-R5s162 rRNA 1 84.661 1 97.263
1 80.089 1 96.358
ENSMUSG00000064517 Gm22454 snRNA 1 85.637 1 97.263
1 84.669 1 87.735
ENSMUSG00000064519 n-R5s97 rRNA 1 71.915 NA NA 1
87.744 1 91.625
ENSMUSG00000064522 Gm26442 snRNA 1 82.705 1 96.847
1 78.954 1 94.904
ENSMUSG00000064524 Gm26441 snRNA 1 65.106 1 92.062
1 83.538 1 96.248
ENSMUSG00000064525 Gm26440 snoRNA 1 84.865 1

96.843 1 85.134 1 95.777
ENSMUSG00000064526 Gm26439 snRNA 1 81.647 1 96.763
1 86.258 1 95.593
ENSMUSG00000064527 Gm26438 misc_RNA 1 78.909 1
96.138 1 87.898 1 95.593
ENSMUSG00000064529 Gm26437 snRNA 1 83.658 1 95.809
1 88.203 1 95.273
ENSMUSG00000064532 Gm23614 snRNA 1 78.909 1 94.262
1 84.504 1 95.72
ENSMUSG00000064536 Gm23615 snoRNA 1 86.464 1
95.35 1 84.308 1 96.248
ENSMUSG00000064541 Gm22978 snRNA 1 75.858 1 97.097
1 87.658 1 92.31
ENSMUSG00000064542 Gm22980 snoRNA 1.374 81.01 NA NA
0.913 87.103 1.148 91.872
ENSMUSG00000064543 Gm22979 snRNA 1 81.52 1 94.597 1
88.349 1 96.042
ENSMUSG00000064545 Gm22981 snoRNA 1 86.172 1
94.48 1 85.58 1 94.297
ENSMUSG00000064547 Gm22982 misc_RNA 1 81.52 1 95.431
1 86.258 1 95.74
ENSMUSG00000064548 n-R5s65 rRNA 1 84.397 1 96.68 1
87.274 1 93.156
ENSMUSG00000064549 Gm22977 snRNA 1 86.351 1 95.088
1 86.831 1 96.086
ENSMUSG00000064550 Gm24623 snRNA 1 77.291 1 95.504
1 80.015 1 93.584
ENSMUSG00000064551 Gm24624 snRNA 1 82.705 1 96.897
1 73.428 1 96.287
ENSMUSG00000064553 Gm24622 snRNA 1 72 1 94.89 1
80.163 1 88.552
ENSMUSG00000064554 n-R5s173 rRNA 1 68.955 1 90.256
1 59.274 1 89.428
ENSMUSG00000064555 Gm24626 snRNA 1 83.968 1 93.094
1 87.37 1 96.086
ENSMUSG00000064556 Gm24625 snoRNA 1 85.203 NA NA
1 83.538 1 95.273
ENSMUSG00000064558 Gm24620 snoRNA 0.936 77.113 0.732
90.256 1.453 52.8 1.162 96.358
ENSMUSG00000064563 Snora7a snoRNA 1.194 61.49 0.719 82.848
1.165 78.369 0.923 90.628
ENSMUSG00000064567 Gm24143 snoRNA 1 53.138 1
92.253 1 70.534 1 96.287
ENSMUSG00000064569 Gm25592 snoRNA 1 82.732 1
96.216 1 88.349 1 93.685
ENSMUSG00000064570 Gm25800 snoRNA 1 66 1 94.848
1 88.203 1 95.593
ENSMUSG00000064572 Gm25801 snRNA 1 67.991 1 95.504
1 79.776 1 96.086
ENSMUSG00000064573 Gm25802 snoRNA 1 81.52 NA NA 1
85.58 1 94.904
ENSMUSG00000064574 Gm25797 snRNA 1 84.534 1 96.763
1 87.37 1 94.608
ENSMUSG00000064575 Gm25798 snRNA 1 73.487 NA NA 1

83.719 1 96.184
ENSMUSG00000064577 Gm25799 snRNA 1 83.169 NA NA 1
88.693 1 94.904
ENSMUSG00000064579 Gm25796 snRNA 1 75.298 1 96.68 1
85.757 1 96.183
ENSMUSG00000064580 Gm25134 snRNA 0.937 82.891 0.844 95.088
1.262 45.449 1.152 83.581
ENSMUSG00000064581 Gm25133 snoRNA 1 82.092 1
96.138 1 86.831 1 96.086
ENSMUSG00000064582 Gm25135 snRNA 1 57.655 0.8 60.678
1.251 48.935 1.145 79.442
ENSMUSG00000064584 Gm25130 snRNA 1 74.299 NA NA 1
87.274 1 95.832
ENSMUSG00000064585 Gm25129 snoRNA 0.576 47.266 1.053
91.83 1.649 40.511 1.228 91.525
ENSMUSG00000064588 n-R5s74 rRNA 1 76.319 1 97.155
1 85.352 1 94.19
ENSMUSG00000064590 Gm22301 snRNA 1 59.806 NA NA 1
82.575 1 95.593
ENSMUSG00000064591 Gm22302 snRNA 1 84.534 1 93.654
1 86.602 1 96.183
ENSMUSG00000064595 Gm22300 snoRNA 1 82.982 1
96.407 1 85.935 1 91.525
ENSMUSG00000064596 Gm22298 snoRNA 1 73.841 1
95.708 NA NA 1 95.273
ENSMUSG00000064600 Gm25636 snoRNA 1 53.138 1
96.268 1 61.743 1 84.499
ENSMUSG00000064601 Gm25634 snRNA 1 80.815 1 95.708
1 87.744 1 94.19
ENSMUSG00000064602 Snora41 snoRNA 0.862 38.827 0.846
78.877 1.364 30.561 1.609 57.075
ENSMUSG00000064604 Snora44 snoRNA 1 74.482 NA NA
1 86.144 1 95.832
ENSMUSG00000064605 Gm22220 snRNA 1 78.909 1 95.708
1 86.985 1 95.405
ENSMUSG00000064606 Gm25899 snRNA 1 82.543 1 97.049
1 79.625 1 95.559
ENSMUSG00000064609 Gm25635 snoRNA 1 85.092 1
95.504 1 85.464 1 95.877
ENSMUSG00000064612 Gm26457 snoRNA 1.467 86.006 0.546
87.147 1.243 63.301 1.479 71.305
ENSMUSG00000064613 Gm24621 snRNA 1 84.534 1 96.68 1
87.744 1 96.248
ENSMUSG00000064616 Gm26454 snRNA 1 86.57 1 96.138 1
86.831 1 95.877
ENSMUSG00000064617 n-R5s176 rRNA 1 82.891 1 95.301
1 88.349 1 94.654
ENSMUSG00000064618 n-R5s95 rRNA 1 79.277 1 94.597
1 81.525 1 92.564
ENSMUSG00000064619 Gm23975 snRNA 1 84.452 1 95.301
1 84.626 1 96.287
ENSMUSG00000064620 Gm22303 snoRNA 1 57.818 1
92.137 1 67.163 1 95.273
ENSMUSG00000064621 AF357355 snoRNA 1 81.647 1

97.155 1 78.045 1 95.273
ENSMUSG00000064622 Gm22304 snoRNA 1 83.263 1
91.456 NA NA 1 93.612
ENSMUSG00000064624 Gm22305 snRNA 1 86.57 1 92.914 1
85.792 1 95.273
ENSMUSG00000064626 Gm22306 snRNA 1 82.352 1 94.262
1 87.744 1 91.525
ENSMUSG00000064630 Gm25143 snRNA 1 82.479 1 96.268
1 86.602 1 94.016
ENSMUSG00000064631 n-R5s42 rRNA 1 67.608 1 90.378
1 81.525 1 95.877
ENSMUSG00000064632 Gm25142 snRNA 1 84.661 1 96.216
1 71.232 1 94.608
ENSMUSG00000064634 Gm22620 snoRNA 1.393 25.026 0.738
55.265 0.97 75.009 0.806 86.445
ENSMUSG00000064637 Snora20 snoRNA 3.713 66 0.23 91.275
1.173 87.898 0.914 94.384
ENSMUSG00000064642 Gm23299 snoRNA 1 86.351 1
95.088 1 84.669 1 94.82
ENSMUSG00000064644 Gm23302 snRNA 1 67.163 1 96.68 1
81.492 1 94.654
ENSMUSG00000064646 Gm23300 snoRNA 1 76.042 1
88.704 1 83.066 1 91.625
ENSMUSG00000064647 Gm23301 snoRNA 1.037 74.147 1.071
95.735 0.9 47.12 0.951 95.273
ENSMUSG00000064648 Gm23303 snRNA 0.186 12.084 1.056 83.104
5.069 75.527 3.718 83.067
ENSMUSG00000064652 Gm24165 snoRNA 1 77.615 1
97.263 1 86.985 1 94.904
ENSMUSG00000064655 Gm25788 snoRNA 0.48 15.456 0.954
95.708 2.187 18.797 1.98 74.193
ENSMUSG00000064658 Gm24166 snoRNA 1 77.182 1
91.361 1 71.615 1 95.593
ENSMUSG00000064659 Gm26131 snRNA 1 84.865 1 95.708
1 82.575 1 95.676
ENSMUSG00000064663 Gm24859 snRNA 1 80.51 1 96.843 1
83.538 1 96.086
ENSMUSG00000064664 Gm24459 snRNA 1 86.351 1 95.329
1 86.258 1 94.99
ENSMUSG00000064665 Gm24460 snoRNA 1 78.639 1
95.504 1 87.274 1 92.742
ENSMUSG00000064666 Snora52 snoRNA 1 75.196 1
92.404 1 79.426 1 94.297
ENSMUSG00000064669 Gm24461 snRNA 1 84.168 1 96.847
1 83.066 1 92.903
ENSMUSG00000064670 Gm22808 snRNA 1 75.196 1 95.088
1 87.336 1 96.086
ENSMUSG00000064671 Gm22807 snoRNA 1 86.474 NA NA
1 88.349 1 95.74
ENSMUSG00000064672 Gm22806 snoRNA 2.29 65.284 0.563
91.955 0.777 85.757 1.189 93.964
ENSMUSG00000064673 Gm22805 snRNA 1 85.728 1 96.652
1 88.274 1 95.273
ENSMUSG00000064675 Gm22810 snRNA 1 79.277 1 96.138

1 87.658 1 94.904
ENSMUSG00000064677 Gm22809 snRNA 1 74.745 1 95.35 1
79.625 1 94.654
ENSMUSG00000064682 Gm25813 snRNA 1 85.946 1 96.68 1
86.084 1 94.82
ENSMUSG00000064685 Gm25812 snRNA 1 84.324 1 96.216
1 75.809 1 96.287
ENSMUSG00000064686 Gm25810 snRNA 1 72.139 1 92.631
1 68.82 1 96.287
ENSMUSG00000064687 Gm25811 snRNA 1 83.169 1 96.948
1 77.532 1 96.287
ENSMUSG00000064688 Gm25814 snRNA 1 81.01 1 93.654 1
83.925 1 96.248
ENSMUSG00000064689 Gm25815 snRNA 1 85.728 1 96.138
1 88.693 1 95.832
ENSMUSG00000064691 Gm24149 snRNA 1 85.092 1 92.137
1 88.657 1 93.457
ENSMUSG00000064692 Gm24151 snoRNA 1 81.76 1 97.155
1 87.37 1 95.676
ENSMUSG00000064693 Gm24150 snRNA 1 73.124 NA NA 1
85.792 1 94.297
ENSMUSG00000064694 Gm24146 misc_RNA 1 83.169 NA NA
1 86.112 1 92.181
ENSMUSG00000064696 Gm24148 snoRNA 1.022 79.577 0.694
66.651 1.409 47.31 0.997 95.74
ENSMUSG00000064697 Gm24147 snoRNA 1 85.946 1
96.763 1 79.625 1 95.74
ENSMUSG00000064698 Gm24152 snRNA 1 77.182 1 96.407
1 86.144 1 95.74
ENSMUSG00000064702 Gm24950 snRNA 1 82.982 1 95.301
1 80.089 1 95.72
ENSMUSG00000064704 n-R5s180 rRNA 1 80.815 NA NA 1
86.727 1 92.181
ENSMUSG00000064706 NA NA NA NA 1 97.263 1
81.525 1 96.139
ENSMUSG00000064707 Gm22507 snRNA 1 78.299 1 95.211
1 73.531 1 95.877
ENSMUSG00000064709 Gm22509 snRNA 1 76.686 1 96.843
1 87.744 1 96.287
ENSMUSG00000064714 n-R5s62 rRNA 1 86.145 NA NA 1
82.74 1 94.608
ENSMUSG00000064715 Gm24207 snRNA 1 67.477 NA NA 1
76.923 1 96.183
ENSMUSG00000064717 Gm24208 snoRNA 1 86.351 1
96.013 1 87.744 1 96.086
ENSMUSG00000064718 Gm25929 snRNA 1 86.333 1 95.708
1 80.899 1 91.372
ENSMUSG00000064720 Gm25856 snoRNA 1 81.01 1 96.268
1 86.985 1 95.273
ENSMUSG00000064721 Gm25855 snoRNA 1 81.01 1 96.843
1 84.626 1 95.593
ENSMUSG00000064722 Gm25857 snoRNA 1 82.092 1
96.216 1 80.634 1 94.904
ENSMUSG00000064725 Snord96a snoRNA 1 81.76 NA NA 1

74.686 1 96.287
ENSMUSG00000064726 Gm25854 snoRNA 1 86.819 1
96.216 1 73.759 1 95.74
ENSMUSG00000064727 Gm25853 snoRNA 1 82.092 1
95.708 1 86.666 1 94.82
ENSMUSG00000064728 Gm25851 snRNA 1 85.092 1 95.211
1 79.255 1 94.19
ENSMUSG00000064731 Snord45b snoRNA 1.133 69.089 0.817
92.914 1.082 85.464 1.124 81.901
ENSMUSG00000064732 Gm23447 snRNA 1 85.77 1 95.211 1
86.666 1 93.457
ENSMUSG00000064734 Gm23445 snRNA 1 79.068 1 84.594
1 86.475 1 83.067
ENSMUSG00000064735 Gm23057 snRNA 1 79.433 1 96.897
1 84.552 1 88.797
ENSMUSG00000064737 Gm23056 snRNA 1 84.168 NA NA 1
88.41 1 96.287
ENSMUSG00000064739 Gm23055 snRNA 1 82.231 1 95.211
1 80.5 1 96.183
ENSMUSG00000064740 Gm24706 snoRNA 1 78.639 1
97.155 NA NA 1 95.593
ENSMUSG00000064741 Snord14a snoRNA 0.881 81.01 0.892 75.493
1.27 31.761 1.149 88.111
ENSMUSG00000064742 Gm24705 snRNA 1 74.8 NA NA 1
75.809 1 86.445
ENSMUSG00000064743 Gm24856 snRNA 1 84.534 1 97.263
1 80.089 1 95.832
ENSMUSG00000064745 Gm24708 snRNA 1 72.372 1 96.335
1 87.336 1 93.612
ENSMUSG00000064746 Gm24707 snRNA 1 69.715 1 92.545
1 82.575 1 96.183
ENSMUSG00000064748 n-R5s28 rRNA 1 84.661 NA NA 1
88.693 1 94.153
ENSMUSG00000064751 Gm26330 snoRNA 1 84.324 1
94.887 1 86.258 1 93.156
ENSMUSG00000064755 Gm26328 snRNA 1 82.732 1 96.216
1 87.686 1 96.287
ENSMUSG00000064758 Gm26331 snRNA 1 85.832 NA NA 1
84.896 1 92.851
ENSMUSG00000064759 NA NA NA NA 1 94.848 1
86.258 1 94.016
ENSMUSG00000064761 Gm23514 snoRNA 1 86.474 1
96.013 1 72.695 1 93.722
ENSMUSG00000064762 Gm23738 snRNA 1 85.728 NA NA 1
82.778 1 96.183
ENSMUSG00000064763 Gm23515 snRNA 1 81.465 1 95.708
1 88.729 1 95.273
ENSMUSG00000064764 Gm23517 snRNA 1 82.705 1 94.102
1 84.83 1 92.748
ENSMUSG00000064765 Gm26456 misc_RNA 1 79.165 1
91.584 1 88.693 1 95.92
ENSMUSG00000064766 Gm26458 snoRNA 1 75.761 NA NA
1 74.686 1 96.248
ENSMUSG00000064767 Snord35b snoRNA 0.622 85.129 1.459

95.196 1.1 88.461 1.308 92.675
ENSMUSG00000064768 Gm23519 snoRNA 1 80.751 1
92.355 1 81.857 1 95.273
ENSMUSG00000064769 Gm23518 snRNA 1 82.092 1 96.897
1 86.258 1 95.92
ENSMUSG00000064772 Gm25202 snRNA 1 80.751 1 94.848
1 86.144 1 95.676
ENSMUSG00000064775 Gm25205 snRNA 1 78.909 1 96.138
1 86.985 1 96.358
ENSMUSG00000064776 Gm25203 snoRNA 1 74.689 1
91.532 1 88.657 1 96.287
ENSMUSG00000064777 Gm25204 snRNA 1 67.608 1 96.013
NA NA 1 95.273
ENSMUSG00000064779 Gm22997 snRNA 1 82.732 1 96.268
1 73.759 1 92.181
ENSMUSG00000064780 Snord95 snoRNA 1 86.843 1
97.155 1 86.112 1 93.734
ENSMUSG00000064781 Gm22686 snRNA 1 83.263 1 96.847
1 80.634 1 94.904
ENSMUSG00000064782 Gm22685 snRNA 1 85.499 NA NA 1
86.258 1 95.877
ENSMUSG00000064783 Gm22684 snoRNA 1 81.657 1
96.843 1 86.144 1 95.92
ENSMUSG00000064788 Gm22683 snoRNA 1 83.09 1 95.708
1 88.729 1 92.018
ENSMUSG00000064791 Snord14e snoRNA 1 85.637 1
96.68 1 82.778 1 96.183
ENSMUSG00000064792 Gm24355 snoRNA 1 76.135 1
86.728 1 57.227 1 64.513
ENSMUSG00000064793 Gm24356 snRNA 1 71.896 NA NA 1
87.519 1 96.248
ENSMUSG00000064795 Gm22423 snRNA 1 84.049 1 90.378
1 87.37 1 90.674
ENSMUSG00000064796 Terc misc_RNA 0.54 14.403 1.389 91.643
1.337 55.352 0.495 83.724
ENSMUSG00000064797 Gm24357 snoRNA 1.039 69.777 1.053
97.155 0.911 88.693 0.966 92.748
ENSMUSG00000064798 n-R5s193 rRNA 1 85.728 1 95.504
1 80.381 1 95.559
ENSMUSG00000064800 Gm24948 snoRNA 1 70.741 1
90.744 NA NA 1 92.018
ENSMUSG00000064801 Gm24035 snRNA 1 74.958 1 96.843
1 79.255 1 92.12
ENSMUSG00000064802 Gm24036 snRNA 1 85.832 1 96.216
1 85.921 1 94.384
ENSMUSG00000064805 Gm24037 snRNA 1 80.751 1 96.68 1
88.161 1 94.297
ENSMUSG00000064806 NA NA NA NA 1 92.137 1
84.552 1 94.654
ENSMUSG00000064814 Gm23939 snoRNA 1 81.465 1
94.48 1 80.899 1 91.872
ENSMUSG00000064816 Gm22357 snoRNA 1 79.93 NA NA 1
78.369 1 95.72
ENSMUSG00000064818 Gm22359 snRNA 1 82.231 NA NA 1

85.757 1 96.287
ENSMUSG00000064822 Gm24292 snRNA 1 84.865 1 95.211
1 79.554 1 96.071
ENSMUSG00000064823 Snord82 snoRNA 1 85.203 1
95.35 1 84.626 1 95.35
ENSMUSG00000064824 Gm22443 snRNA 1 85.129 1 93.094
1 85.792 1 96.183
ENSMUSG00000064829 Gm22879 snoRNA 1 86.351 1
93.765 1 88.693 1 95.877
ENSMUSG00000064830 Gm25699 snRNA 1 84.534 1 96.216
1 79.625 1 96.248
ENSMUSG00000064831 Gm25698 snRNA 1 80.51 1 97.288 1
87.786 1 96.358
ENSMUSG00000064833 Gm25926 snRNA 3.972 19.477 0.481 93.654
0.524 40.794 0.716 85.493
ENSMUSG00000064834 Gm25696 snRNA 1 84.324 NA NA 1
84.434 1 96.358
ENSMUSG00000064836 Gm25697 snoRNA 1 85.946 1
93.574 1 76.923 1 96.287
ENSMUSG00000064837 Snora75 snoRNA 0.834 67.334 1.381
79.411 0.87 85.675 0.851 96.183
ENSMUSG00000064840 Gm26204 snRNA 1 84.819 NA NA 1
79.625 1 92.561
ENSMUSG00000064841 Gm26205 snoRNA 1.059 67.163 1.061
95.809 0.89 85.134 1.026 74.193
ENSMUSG00000064842 Gm26206 snRNA 1 72.85 1 97.263 1
79.325 1 93.612
ENSMUSG00000064843 Gm26207 snRNA 1 79.433 NA NA 1
85.792 1 96.183
ENSMUSG00000064844 Gm26202 snoRNA 0.467 55.303 0.683
87.322 3.122 28.301 3.376 60.968
ENSMUSG00000064845 Gm26203 snRNA 1 85.946 NA NA 1
78.293 1 93.734
ENSMUSG00000064850 Gm23443 snoRNA 1 81.52 1 95.196
1 87.37 1 95.92
ENSMUSG00000064851 Gm24525 snRNA 1 60.003 1 96.763
1 81.29 1 93.261
ENSMUSG00000064853 Gm23442 snoRNA 0.944 78.639 0.809
66.651 1.305 65.034 1.001 93.264
ENSMUSG00000064854 NA NA NA NA NA NA 1 88.161
1 93.584
ENSMUSG00000064855 Gm24526 snRNA 1 82.231 1 97.155
1 81.525 1 95.273
ENSMUSG00000064856 Gm23444 snRNA 1 73.841 1 97.049
1 71.999 1 92.181
ENSMUSG00000064857 n-R5s25 rRNA 1 80.408 NA NA 1
85.935 1 95.676
ENSMUSG00000064858 Snora43 snoRNA 1 80.146 1
95.088 1 87.898 1 95.777
ENSMUSG00000064859 Gm24524 snRNA 1 80.751 1 95.941
1 88.693 1 91.783
ENSMUSG00000064860 n-R5s220 rRNA 1 79.93 1 95.708 1
82.575 1 95.273
ENSMUSG00000064862 Gm25038 snoRNA 1 77.291 1

96.308 1 83.066 1 95.676
ENSMUSG00000064867 Gm25039 snRNA 1 64.284 1 93.863
1 75.637 1 96.086
ENSMUSG00000064871 Snord58b snoRNA 0.938 86.843 0.744
79.807 1.436 38.099 0.885 92.561
ENSMUSG00000064872 Gm23376 snRNA 1 81.647 1 97.155
1 83.397 1 93.132
ENSMUSG00000064876 Gm23378 snRNA 1 82.092 1 96.268
1 86.831 1 95.877
ENSMUSG00000064877 Gm23377 snoRNA 1 86.464 1
95.088 1 87.431 1 95.72
ENSMUSG00000064880 Gm24201 snoRNA 1 80.408 1
97.155 1 86.666 1 88.505
ENSMUSG00000064882 Gm24202 snRNA 1 60.707 1 97.129
1 87.898 1 96.086
ENSMUSG00000064883 Gm24203 misc_RNA 1 85.655 1
96.138 1 84.308 1 96.184
ENSMUSG00000064884 NA NA NA NA 1 96.843 1
86.666 1 95.74
ENSMUSG00000064887 Gm24205 snRNA 1 85.59 1 96.138 1
87.274 1 95.35
ENSMUSG00000064889 Gm24200 snRNA 1 79.433 1 97.263
1 88.161 1 91.335
ENSMUSG00000064890 Gm22505 snRNA 1 80.608 1 94.96 1
88.076 1 96.287
ENSMUSG00000064897 Gm22504 snoRNA 0.701 63.126 2.493
82.76 0.571 49.967 0.497 84.29
ENSMUSG00000064899 Snord118 snoRNA 1 75.858 1
96.843 1 86.258 1 94.016
ENSMUSG00000064900 Gm25402 snoRNA 1 64.284 NA NA
1 82.436 1 95.832
ENSMUSG00000064901 Snora21 snoRNA 1.786 36.686 0.729
91.532 0.77 81.525 0.579 91.372
ENSMUSG00000064904 Gm25404 snRNA 1 82.891 1 96.216
1 87.658 1 94.904
ENSMUSG00000064905 Gm25403 misc_RNA 1 81.465 1
91.361 1 82.903 1 95.74
ENSMUSG00000064906 n-R5s204 rRNA 1 85.946 1 93.094
1 88.601 1 93.045
ENSMUSG00000064907 Gm25405 snRNA 1 84.263 1 95.301
1 82.436 1 96.287
ENSMUSG00000064909 Gm25401 snRNA 1 54.717 1 96.407
1 52.972 1 89.892
ENSMUSG00000064915 Gm22575 snRNA 1 82.352 1 97.155
1 87.274 1 95.92
ENSMUSG00000064916 Gm22573 snRNA 0.694 80.146 NA NA
1.496 79.119 1.287 96.358
ENSMUSG00000064918 Gm22571 snoRNA 1.085 74.147 NA NA
0.984 66.425 1.469 86.301
ENSMUSG00000064921 Gm22229 snRNA 1 86.112 NA NA 1
88.461 1 91.65
ENSMUSG00000064923 Gm22042 snRNA 1 84.564 NA NA 1
85.792 1 93.964
ENSMUSG00000064925 Snora62 snoRNA 1.682 54.717 0.757

94.262	0.782	81.525	0.793	90.805
ENSMUSG00000064926	Gm22044	snRNA	1	78.909	1	96.268
1	82.161	1	95.273
ENSMUSG00000064927	Gm22043	snRNA	0.832	81.01	0.815	82.671
1.471	55.352	1.039	96.139
ENSMUSG00000064928	Gm22045	snRNA	1	83.169	NA	NA	1
86.144	1	94.904
ENSMUSG00000064932	n-R5s36	rRNA	1	86.658	1	96.138
NA	NA	1	94.468
ENSMUSG00000064938	Mir3068	snoRNA	0.646	45.728	0.638
86.071	2.415	33.508	1.437	91.335
ENSMUSG00000064939	Gm23721	snRNA	1	84.819	1	96.216
1	84.434	1	92.018
ENSMUSG00000064940	Gm23239	snRNA	1	84.819	NA	NA	1
87.744	1	93.156
ENSMUSG00000064941	Gm23238	snRNA	1	81.647	NA	NA	1
73.759	1	93.156
ENSMUSG00000064943	Gm23240	snRNA	1	84.049	NA	NA	1
86.666	1	94.904
ENSMUSG00000064945	Rny3	misc_RNA	1	84.324	1	94.597
1	84.724	1	96.183
ENSMUSG00000064949	Snora61	snoRNA	1	83.169	1
93.654	1	86.475	1	96.086
ENSMUSG00000064952	Gm24920	snoRNA	1.217	74.745	0.625
73.166	1.316	65.034	0.821	90.611
ENSMUSG00000064954	Gm24919	snRNA	1	84.324	1	94.887
1	77.17	1	92.762
ENSMUSG00000064956	Gm24918	snoRNA	1	61.633	1
92.111	1	87.173	1	94.904
ENSMUSG00000064958	Gm24921	snRNA	1	79.853	1	95.504
NA	NA	1	96.248
ENSMUSG00000064959	n-R5s41	rRNA	1	80.51	1	95.504	1
84.83	1	79.442
ENSMUSG00000064960	Gm24406	snoRNA	1	84.977	1
93.654	1	79.426	1	90.95
ENSMUSG00000064961	Gm22704	snoRNA	1	75.858	1
96.013	1	85.464	1	92.181
ENSMUSG00000064966	Snord15b	snoRNA	1	83.333	1
96.138	1	87.786	1	94.654
ENSMUSG00000064967	Gm24405	snRNA	1	75.298	1	95.809
1	80.634	1	94.174
ENSMUSG00000064968	Snord47	snoRNA	1	83.09	NA	NA	1
80.015	1	92.762
ENSMUSG00000064971	Gm26054	snRNA	1	84.865	1	97.155
1	84.83	1	94.19
ENSMUSG00000064972	Gm26055	snRNA	1	81.01	NA	NA	1
87.336	1	94.19
ENSMUSG00000064977	Gm26056	snRNA	1	86.474	1	91.661
1	84.724	1	94.904
ENSMUSG00000064981	Snora70	snoRNA	1	85.203	NA	NA
1	87.519	1	93.316
ENSMUSG00000064983	Gm25258	snRNA	1	84.977	1	96.897
1	87.898	1	94.904
ENSMUSG00000064984	Snord73a	snoRNA	1.968	1.643	0.525	59.396

0.969 87.519 0.948 94.82
ENSMUSG00000064987 Gm25260 snRNA 1 73.734 1 96.68 1
87.103 1 93.016
ENSMUSG00000064992 Gm22421 snRNA 1 72.985 1 93.094
1 73.759 1 92.564
ENSMUSG00000064995 Gm26003 snoRNA 1 84.263 1
96.268 1 86.602 1 95.273
ENSMUSG00000064999 NA NA NA NA 1 97.288 1
81.525 1 90.805
ENSMUSG00000065004 Gm26326 snoRNA 1 81.01 1 93.899
1 87.37 1 94.904
ENSMUSG00000065005 n-R5s79 rRNA 1 85.129 1 96.68 1
72.378 1 96.287
ENSMUSG00000065006 Gm26325 snRNA 1 80.815 1 96.847
1 70.9 1 96.086
ENSMUSG00000065007 Gm26324 snRNA 1 79.577 1 92.561
NA NA 1 95.273
ENSMUSG00000065011 Gm23506 snRNA 1 84.865 1 95.211
1 84.308 1 92.381
ENSMUSG00000065012 Gm23507 snRNA 1 74.8 1 96.763 1
87.898 1 96.287
ENSMUSG00000065013 Gm23508 snoRNA 1 83.823 NA NA
1 81.388 1 96.248
ENSMUSG00000065014 Gm23509 snRNA 1 85.391 1 95.088
1 86.144 1 93.156
ENSMUSG00000065016 Snora3 snoRNA 1 86.172 1
94.597 NA NA 1 91.625
ENSMUSG00000065017 Gm23510 snRNA 1 86.351 1 95.504
1 86.112 1 96.139
ENSMUSG00000065018 Gm23511 snRNA 1 83.418 1 95.504
1 84.308 1 92.748
ENSMUSG00000065022 AF357341 snoRNA 1 85.77 1 93.899
1 84.229 1 94.904
ENSMUSG00000065027 Gm25201 snRNA 1 66.129 NA NA 1
80.899 1 94.215
ENSMUSG00000065028 Gm25200 snoRNA 1 73.292 1
97.288 NA NA 1 96.183
ENSMUSG00000065029 Gm25199 snRNA 1 78.428 1 94.48 1
84.229 1 91.872
ENSMUSG00000065030 n-R5s160 rRNA 1 84.294 NA NA 1
87.658 1 95.273
ENSMUSG00000065031 Gm22364 snRNA 1 82.092 1 95.735
NA NA 1 94.904
ENSMUSG00000065034 Gm22363 snRNA 1 86.464 1 96.216
1 88.693 1 95.877
ENSMUSG00000065036 Gm22362 snoRNA 0.879 79.433 0.801
87.919 1.415 32.433 0.873 96.358
ENSMUSG00000065037 Rn7sk misc_RNA 0.966 81.125 1.098 95.504
NA NA 1.123 92.181
ENSMUSG00000065040 Gm24977 snRNA 1 81.01 1 96.68 1
74.853 1 96.287
ENSMUSG00000065041 Gm24044 snoRNA 1.301 78.428 1.27
82.904 0.606 58.271 1.021 93.722
ENSMUSG00000065042 Gm24043 snRNA 1 81.01 1 94.887 1

87.744 1 95.273
ENSMUSG00000065044 n-R5s169 rRNA 1 86.918 1 95.941
1 83.607 1 93.316
ENSMUSG00000065047 Gm24045 misc_RNA 0.87 84.865 2.687
89.996 0.428 52.087 0.645 85.493
ENSMUSG00000065048 Gm24042 snRNA 1 83.169 NA NA 1
88.693 1 95.593
ENSMUSG00000065049 Gm24041 snRNA 1 83.968 1 96.013
1 83.538 1 96.358
ENSMUSG00000065050 Gm25704 snRNA 1 82.352 1 92.631
1 87.971 1 94.904
ENSMUSG00000065053 Gm25705 snRNA 1 86.474 1 94.367
1 88.657 1 95.273
ENSMUSG00000065055 n-R5s213 rRNA 1 85.283 1 97.263
1 78.369 1 94.297
ENSMUSG00000065057 Gm25703 snRNA 1 83.726 1 96.138
1 69.196 1 93.734
ENSMUSG00000065061 Gm22884 snoRNA 1 77.763 1
96.843 1 83.066 1 96.192
ENSMUSG00000065068 n-R5s5 rRNA 1 84.865 1 91.955
1 80.089 1 96.183
ENSMUSG00000065070 n-R5s196 rRNA 1 61.633 1 93.529
1 83.397 1 96.183
ENSMUSG00000065072 Gm24533 snoRNA 1 84.049 1
96.216 1 84.896 1 91.269
ENSMUSG00000065074 Gm24535 snRNA 1 81.925 1 95.431
1 88.41 1 95.273
ENSMUSG00000065078 Gm24536 snRNA 1 80.608 1 96.407
1 82.685 1 92.389
ENSMUSG00000065079 Gm23464 snRNA 1 85.283 1 97.097
1 86.552 1 96.183
ENSMUSG00000065083 n-R5s205 rRNA 1 86.474 1 95.708
1 84.669 1 88.797
ENSMUSG00000065084 Gm26496 snRNA 1 86.843 1 96.138
1 84.229 1 95.273
ENSMUSG00000065086 Gm26495 snoRNA 1 85.283 1
95.211 1 85.921 1 93.685
ENSMUSG00000065087 Snord22 snoRNA 1 86.918 1
97.288 1 86.831 1 96.358
ENSMUSG00000065088 Gm26494 snoRNA 1 75.298 NA NA
1 88.161 1 93.964
ENSMUSG00000065089 Gm26493 snoRNA 2.1 64.193 0.432
84.017 1.095 66.647 0.516 96.192
ENSMUSG00000065090 Gm23677 snRNA 1 74.147 1 91.955
1 87.898 1 94.001
ENSMUSG00000065091 Gm23678 snRNA 1 81.647 1 94.597
1 77.888 1 92.31
ENSMUSG00000065094 NA NA NA NA 1 93.899 1
79.255 1 95.273
ENSMUSG00000065095 Gm23679 snoRNA 1 81.01 NA NA 1
79.554 1 95.72
ENSMUSG00000065096 Gm23680 snRNA 1 74.021 1 97.263
1 85.675 1 95.593
ENSMUSG00000065097 Snora16a snoRNA 0.912 80.751 NA NA

0.783 84.896 1.293 44.882
ENSMUSG00000065102 n-R5s50 rRNA 1 78.428 1 94.367
1 86.602 1 93.612
ENSMUSG00000065104 Gm26128 snRNA 1 74.299 NA NA 1
81.29 1 96.086
ENSMUSG00000065105 Gm26130 snoRNA 1 85.637 1
96.268 1 88.729 1 93.734
ENSMUSG00000065107 n-R5s88 rRNA 1 65.869 1 94.48 1
78.369 1 93.132
ENSMUSG00000065110 Snord61 snoRNA 1 86.351 1
96.68 1 87.103 1 94.904
ENSMUSG00000065111 Gm25141 snRNA 1 85.728 1 96.001
1 85.464 1 94.215
ENSMUSG00000065115 Gm23298 snRNA 1 84.452 1 96.68 NA
NA 1 96.183
ENSMUSG00000065118 Gm23297 snoRNA 0.71 42.361 2.07
83.104 0.679 88.076 1.783 91.525
ENSMUSG00000065122 Gm26126 snRNA 1 83.418 1 96.216
1 81.492 1 96.358
ENSMUSG00000065124 Snora65 snoRNA 1 86.145 1
95.941 NA NA 1 93.316
ENSMUSG00000065126 Snord104 snoRNA 0.877 22.431 0.931
87.452 1.226 21.462 0.96 92.691
ENSMUSG00000065127 Gm26127 snRNA 1 84.168 1 95.301
1 86.112 1 92.748
ENSMUSG00000065130 Gm24457 snRNA 1 80.815 1 91.532
1 83.275 1 94.384
ENSMUSG00000065132 Gm24456 snRNA 1 81.76 1 95.809 1
84.83 1 95.273
ENSMUSG00000065138 Gm24458 snRNA 1 80.751 1 95.708
1 81.976 1 93.627
ENSMUSG00000065141 NA NA NA NA NA NA 1 78.642
1 95.877
ENSMUSG00000065143 Gm25644 snRNA 1 82.543 1 93.094
1 87.744 1 92.903
ENSMUSG00000065147 Snora31 snoRNA 0.969 78.538 1.015
96.843 NA NA 0.944 94.654
ENSMUSG00000065151 Gm25480 snoRNA 1 86.474 1
95.504 1 87.431 1 93.734
ENSMUSG00000065155 Gm25481 snRNA 1 63.804 1 97.263
1 78.293 1 91.291
ENSMUSG00000065158 Gm25483 snoRNA 1 71.142 1
91.532 1 87.103 1 85.577
ENSMUSG00000065159 Gm25482 snRNA 1 51.089 1 93.899
NA NA 1 94.904
ENSMUSG00000065160 Mir5117 snoRNA 1 79.709 NA NA
1 88.693 1 91.625
ENSMUSG00000065162 Gm23645 snRNA 0.884 50.768 1.143 87.185
0.99 75.637 0.891 93.734
ENSMUSG00000065164 Gm23814 snRNA 1 81.465 1 95.431
1 87.37 1 93.627
ENSMUSG00000065165 Gm23815 snRNA 1 75.399 1 93.574
1 82.575 1 96.139
ENSMUSG00000065173 Gm22127 snRNA 1 82.352 1 90.053

1	85.792	1	94.904
ENSMUSG00000065178	Gm22126	snRNA	1	79.93	1	97.155	NA	NA	1	96.183
ENSMUSG00000065181	Gm25139	snoRNA	1	82.092	1	97.129	1	87.971	1	93.734
ENSMUSG00000065182	Gm25138	snoRNA	1	70.597	1	94.89	NA	NA	1	96.358
ENSMUSG00000065185	NA	NA	NA	NA	1	94.887	1	70.1	1	94.904
ENSMUSG00000065187	Gm25136	snoRNA	1	82.982	1	96.138	1	81.169	1	96.086
ENSMUSG00000065188	Gm25140	snRNA	1	81.647	1	96.847	1	86.985	1	91.928
ENSMUSG00000065191	Gm23458	snRNA	1.136	50.913	1.136	95.196	0.771	68.82	0.823	85.493
ENSMUSG00000065192	Gm23460	snRNA	1	85.728	1	95.708	1	73.309	1	93.722
ENSMUSG00000065193	Gm23459	snRNA	1	85.655	1	95.211	NA	NA	1	96.248
ENSMUSG00000065195	Gm26071	snRNA	1	72.743	1	96.843	1	86.552	1	91.872
ENSMUSG00000065196	Snord85	snoRNA	1	85.203	1	96.763	1	88.41	1	95.405
ENSMUSG00000065198	Gm23462	snRNA	1	75.298	1	94.48	1	80.015	1	95.35
ENSMUSG00000065199	Gm23461	snRNA	1	80.688	1	95.329	NA	NA	1	96.086
ENSMUSG00000065201	Gm24612	snRNA	1	82.891	1	97.263	1	84.896	1	96.086
ENSMUSG00000065202	Gm24615	snoRNA	1	85.832	NA	NA	1	87.898	1	96.358
ENSMUSG00000065203	Gm24614	snRNA	1	74.147	1	96.847	1	83.719	1	95.72
ENSMUSG00000065206	Gm24611	snRNA	1	82.891	1	96.216	1	87.971	1	95.593
ENSMUSG00000065211	Gm26265	snoRNA	1	80.51	NA	NA	1	81.032	1	94.654
ENSMUSG00000065212	Gm26264	snRNA	1	84.865	1	95.301	1	88.274	NA	NA
ENSMUSG00000065215	Gm26263	snRNA	1	75.761	1	97.263	1	87.431	1	96.287
ENSMUSG00000065219	Snord32a	snoRNA	1	85.946	1	96.013	NA	NA	1	92.762
ENSMUSG00000065220	Gm25794	snRNA	1	71.142	1	95.735	1	88.461	1	96.183
ENSMUSG00000065221	Gm25793	snRNA	1	81.01	1	96.843	1	87.786	1	92.181
ENSMUSG00000065223	Gm25792	snRNA	1	76.319	1	97.155	1	84.896	1	96.287
ENSMUSG00000065226	Gm25791	snoRNA	1.04	24.253	0.884	64.123	1.088	73.309	0.874	84.29
ENSMUSG00000065227	Gm25790	snRNA	1	81.196	1	95.941	1	83.719	1	95.74
ENSMUSG00000065228	Gm25789	snoRNA	1	74.516	1

96.001	1	78.864	1	92.748
ENSMUSG00000065231	Gm22972	snoRNA	1	82.705	NA	NA
1	85.921	1	88.797
ENSMUSG00000065232	Gm22973	snRNA	1	80.251	1	96.847
1	84.669	1	95.72
ENSMUSG00000065233	n-R5s60	rRNA	1	75.196	NA	NA	1
84.308	1	92.181
ENSMUSG00000065235	Gm22974	snRNA	1	81.823	NA	NA	NA
NA	1	94.99
ENSMUSG00000065236	n-R5s197	rRNA	1.009	61.334	1.528	83.104
0.648	51.443	1.316	90.674
ENSMUSG00000065237	NA	NA	NA	NA	1	94.597	1
84.552	1	93.584
ENSMUSG00000065238	Gm22970	snRNA	1	82.479	1	94.48	NA
NA	1	93.156
ENSMUSG00000065239	Gm22971	snRNA	1	81.24	1	95.211	1
79.554	1	95.74
ENSMUSG00000065242	Gm22294	snRNA	1	85.283	1	96.68	1
79.255	1	96.287
ENSMUSG00000065244	Gm22296	snRNA	1	84.397	1	95.211
1	84.308	1	88.505
ENSMUSG00000065245	Gm22295	snRNA	1	84.865	NA	NA	1
69.974	1	87.088
ENSMUSG00000065246	Gm22297	snRNA	1	58.802	NA	NA	1
86.831	1	87.624
ENSMUSG00000065248	Gm22293	snoRNA	1	74.689	1
92.474	1	86.666	1	95.559
ENSMUSG00000065251	Gm23971	snRNA	1	80.688	1	96.216
NA	NA	1	94.654
ENSMUSG00000065253	Gm23970	snRNA	1	85.391	1	95.35	1
87.898	1	96.287
ENSMUSG00000065254	Gm23973	misc_RNA	1	84.865	1
96.138	1	86.258	1	94.82
ENSMUSG00000065255	Gm23974	snoRNA	1	78.054	NA	NA
1	73.598	1	95.35
ENSMUSG00000065256	Gm23972	snRNA	1	83.09	1	95.708	1
78.293	1	93.722
ENSMUSG00000065258	Gm23969	snoRNA	0.773	52.9	NA	NA	1.01
48.451	1.088	90.865
ENSMUSG00000065259	Snora30	snoRNA	0.908	52.258	0.968
94.597	1.134	46.956	0.758	94.19
ENSMUSG00000065260	Gm23453	snRNA	1	74.299	NA	NA	1
87.898	1	95.877
ENSMUSG00000065261	Gm23452	snRNA	1	78.639	1	96.847
1	84.434	1	94.153
ENSMUSG00000065262	Gm23451	snoRNA	1	86.57	1	95.329
1	85.935	1	95.593
ENSMUSG00000065265	Gm23455	snoRNA	0.857	37.248	0.888
79.807	1.318	20.315	1.202	63.114
ENSMUSG00000065266	Gm23454	snoRNA	1	86.464	1
96.268	1	85.757	1	96.248
ENSMUSG00000065269	Gm23456	snoRNA	1	84.324	1
95.708	1	82.74	1	95.405
ENSMUSG00000065272	Snord57	snoRNA	1	82.732	1

91.661 1 88.693 1 92.466
ENSMUSG00000065273 Gm25128 snoRNA 0.673 45.728 1.225
96.407 1.209 67.562 1.113 93.156
ENSMUSG00000065274 Gm25125 snoRNA 1 83.968 1
97.155 NA NA 1 96.248
ENSMUSG00000065275 n-R5s217 rRNA 1 84.819 NA NA 1
83.538 1 96.358
ENSMUSG00000065277 Gm25126 snRNA 1 78.224 1 95.504
1 86.727 1 93.734
ENSMUSG00000065279 Gm25124 snRNA 1 54.983 1 96.138
1 54.673 1 96.248
ENSMUSG00000065280 Gm24453 snoRNA 1 83.169 NA NA
1 87.274 1 94.904
ENSMUSG00000065281 Gm24452 snoRNA 1 86.333 1
95.735 1 86.666 1 94.654
ENSMUSG00000065282 Gm24455 snoRNA 1.537 5.848 0.724 43.505
0.895 80.5 0.913 92.466
ENSMUSG00000065283 NA NA NA NA 1 96.013 1
72.464 1 93.964
ENSMUSG00000065287 Gm24451 snoRNA 1 83.968 1
96.407 1 84.308 1 95.273
ENSMUSG00000065289 Gm23650 snoRNA 1 70.862 1
97.263 1 81.032 1 94.001
ENSMUSG00000065291 Gm26125 snRNA 1 81.01 1 93.765 NA
NA 1 96.192
ENSMUSG00000065293 Gm26124 misc_RNA 1 86.172 1
95.708 1 80.381 1 95.35
ENSMUSG00000065295 Gm24134 snoRNA 1 80.408 1
96.407 1 86.258 1 93.964
ENSMUSG00000065296 Gm26122 snRNA 1 85.77 NA NA 1
80.899 1 95.877
ENSMUSG00000065297 Gm26123 snoRNA 1 80.608 NA NA
NA NA 1 94.468
ENSMUSG00000065298 Gm26121 snRNA 1 78.299 1 94.887
1 76.498 1 94.82
ENSMUSG00000065299 Gm23138 snRNA 1 77.916 1 94.102
1 86.144 1 91.291
ENSMUSG00000065303 Gm23249 snRNA 1 84.738 1 96.984
1 86.985 1 96.358
ENSMUSG00000065304 Gm23245 snoRNA 0.937 52.986 1.15
94.848 0.93 79.255 1.096 88.387
ENSMUSG00000065305 Gm23246 snoRNA 1 86.819 1
96.897 1 87.658 1 95.273
ENSMUSG00000065307 Gm23247 snRNA 1 77.916 1 96.216
1 82.31 1 91.217
ENSMUSG00000065309 Gm23244 snRNA 1 75.567 1 93.469
1 82.161 1 93.734
ENSMUSG00000065311 n-R5s156 rRNA 1 84.263 NA NA 1
88.076 NA NA
ENSMUSG00000065312 Gm26063 snRNA 1 83.418 1 87.029
1 86.144 1 93.964
ENSMUSG00000065313 Gm26062 snRNA 1 81.465 1 97.155
1 88.729 1 88.111
ENSMUSG00000065314 Gm26065 snRNA 1 82.231 1 96.843

1 88.203 1 94.904
ENSMUSG00000065315 Gm26064 snRNA 0.443 48.354 2.003 96.407
1.13 59.612 0.9 93.722
ENSMUSG00000065321 Gm22057 snRNA 1 80.03 1 97.263 1
77.532 1 95.832
ENSMUSG00000065322 Gm22055 snRNA 1 83.523 1 95.708
1 78.498 1 91.372
ENSMUSG00000065327 Gm22058 misc_RNA 1 81.925 1
97.129 1 86.831 1 96.183
ENSMUSG00000065328 Gm22053 snoRNA 1 85.203 1
96.138 1 88.601 1 95.74
ENSMUSG00000065329 Gm22054 snRNA 1 50.768 1 91.897
1 41.331 1 94.016
ENSMUSG00000065330 Gm25755 snRNA 1 82.231 1 96.897
1 83.719 1 93.964
ENSMUSG00000065333 Gm24928 snRNA 1 79.93 1 95.431 1
83.925 1 96.071
ENSMUSG00000065336 Snora34 snoRNA 1.028 70.36 1.063 95.211
0.912 71.734 0.947 86.991
ENSMUSG00000065337 Gm24929 snRNA 1 84.168 1 95.504
1 84.724 1 93.316
ENSMUSG00000065340 Gm25578 snRNA 1 84.324 1 96.378
1 85.921 1 96.183
ENSMUSG00000065343 Gm25580 misc_RNA 1.549 64.956 1.772
96.68 0.365 39.528 1.092 89.72
ENSMUSG00000065344 Gm25581 snRNA 1 86.199 NA NA 1
86.144 1 94.904
ENSMUSG00000065346 Gm25582 snoRNA 1 81.01 1 91.361
1 88.693 1 88.657
ENSMUSG00000065347 Gm25583 snRNA 1 69.985 1 96.407
1 83.607 1 94.608
ENSMUSG00000065348 Gm25577 snRNA 1 72.743 1 96.013
NA NA 1 94.904
ENSMUSG00000065353 Snora73b snoRNA 0.961 74.021 0.889
94.96 1.172 65.8 1.139 65.558
ENSMUSG00000065360 Gm24412 snoRNA 0.794 81.01 0.943 84.594
1.329 56.497 0.661 88.505
ENSMUSG00000065362 Gm24411 snoRNA 1.096 74.482 0.655
73.461 1.396 24.985 1.407 51.908
ENSMUSG00000065367 Gm24410 snRNA 1 82.231 1 87.919
1 79.426 1 95.273
ENSMUSG00000065368 Gm24414 snRNA 1 84.865 NA NA 1
82.161 1 90.177
ENSMUSG00000065372 Gm22741 snRNA 1 81.24 1 97.155 1
78.498 1 95.72
ENSMUSG00000065373 Gm22740 snRNA 1 71.36 1 91.661 1
73.428 1 95.273
ENSMUSG00000065378 Gm22744 snoRNA 1 78.428 1
95.431 1 86.602 1 93.964
ENSMUSG00000065379 Gm22743 snoRNA 1 86.199 1
94.848 1 83.275 1 96.086
ENSMUSG00000065380 Gm23095 snRNA 1 75.399 1 94.102
1 72.464 1 96.183
ENSMUSG00000065381 Gm23096 snRNA 1 81.76 1 95.504 1

86.346	1	96.183
ENSMUSG00000065382	Gm23097	snRNA	1	81.925	1	96.138
1	77.532	1	95.559
ENSMUSG00000065384	Gm23093	snRNA	1	86.006	1	97.155
1	88.657	1	93.156
ENSMUSG00000065385	n-R5s153	rRNA	1	80.688	1	97.097
1	84.83	1	95.777
ENSMUSG00000065388	Gm23099	snRNA	1	83.333	1	96.68	1
86.258	1	96.183
ENSMUSG00000065392	Gm25894	snoRNA	1	85.77	1	95.301
1	88.693	1	95.877
ENSMUSG00000065394	Mir25	miRNA	1	75.858	1	94.48	NA	NA
1	95.273
ENSMUSG00000065395	Mir193	miRNA	1	62.007	1	96.216
1	87.744	1	92.762
ENSMUSG00000065396	Mir99b	miRNA	0.493	2.822	1.294	85.756
1.567	17.998	1.01	93.156
ENSMUSG00000065397	Mir155	miRNA	1	81.52	1	92.631	NA
NA	1	95.273
ENSMUSG00000065398	Mir188	miRNA	1	82.352	1	97.263
NA	NA	1	95.92
ENSMUSG00000065399	Mir133a-1	miRNA	1	69.089	1	97.155
1	62.791	1	92.018
ENSMUSG00000065401	Mir144	miRNA	1	83.263	NA	NA	1
87.971	1	94.82
ENSMUSG00000065402	Mir122a	miRNA	1	82.231	1	97.263
1	86.475	1	95.273
ENSMUSG00000065403	Mir18	miRNA	1	85.355	1	91.361	NA
NA	1	95.35
ENSMUSG00000065404	Mir201	miRNA	1	84.865	NA	NA	1
74.325	1	95.273
ENSMUSG00000065405	Mir30a	miRNA	1	86.333	1	95.088
1	79.325	1	96.183
ENSMUSG00000065406	Mirlet7i	miRNA	1.359	59.211	0.463	62.692
1.592	45.667	1.537	94.016
ENSMUSG00000065407	Mir135a-1	miRNA	0.691	49.368	1.415	83.104
1.024	87.336	1.656	91.928
ENSMUSG00000065408	Mir31	miRNA	1.001	74.8	1.598	91.456	0.626
56.822	0.995	95.92
ENSMUSG00000065409	Mir30e	miRNA	1	83.09	NA	NA	NA	NA
1	94.825
ENSMUSG00000065410	Mir298	miRNA	1	84.564	1	93.908
1	86.346	1	95.92
ENSMUSG00000065412	Mir29b-2	miRNA	1	77.615	NA	NA	1
80.089	1	96.184
ENSMUSG00000065413	Mir367	miRNA	1	85.129	1	95.088
1	79.554	1	94.904
ENSMUSG00000065414	Mir138-1	miRNA	1	84.397	NA	NA	1
83.275	1	92.748
ENSMUSG00000065415	Mir217	miRNA	1	85.655	1	97.155
1	82.436	1	95.405
ENSMUSG00000065416	Mir19a	miRNA	1	72.27	NA	NA	1
80.015	1	94.297
ENSMUSG00000065417	Mir340	miRNA	1	73.734	1	94.848

1 87.898 1 88.387
ENSMUSG00000065418 Mir322 miRNA 1 81.465 1 96.407
1 79.255 1 92.181
ENSMUSG00000065420 Mir142 miRNA 1.22 72.85 0.596 75.4 1.374
62.237 0.809 83.724
ENSMUSG00000065423 Mir181a-2 miRNA 1 74.958 1 90.29 1
68.82 1 91.269
ENSMUSG00000065424 Mir9-2 miRNA 1 78.299 1 93.654
1 84.669 1 96.139
ENSMUSG00000065425 Mir325 miRNA 1 81.01 1 97.263 1
82.575 1 94.825
ENSMUSG00000065426 Mir134 miRNA 1 75.298 1 96.216
1 87.103 1 96.248
ENSMUSG00000065427 Mir429 miRNA 1 85.59 1 95.708 1
79.897 1 92.31
ENSMUSG00000065428 Mir382 miRNA 1 86.351 1 96.216
1 86.666 1 93.316
ENSMUSG00000065429 Mir345 miRNA 1.007 72 0.966 96.948
1.024 84.071 1.021 84.499
ENSMUSG00000065431 Mir186 miRNA 1.066 74.482 0.756 79.475
1.245 57.227 0.792 86.445
ENSMUSG00000065432 Mir208a miRNA 1 75.115 1 97.288
1 74.853 1 96.183
ENSMUSG00000065433 Mir370 miRNA 1 86.333 1 92.931
1 64.427 1 92.748
ENSMUSG00000065434 Mir7-1 miRNA 1.012 86.658 1.311 88.91
0.754 39.079 0.947 87.735
ENSMUSG00000065435 Mir135b miRNA 1 70.213 1 96.407
1 84.83 1 94.608
ENSMUSG00000065436 Mir342 miRNA 1 73.841 NA NA 1
70.44 1 87.331
ENSMUSG00000065437 Mir30d miRNA 1 85.203 1 96.001
NA NA 1 96.086
ENSMUSG00000065438 Mir377 miRNA 1 69.408 1 95.708
1 88.076 1 96.287
ENSMUSG00000065439 Mir140 miRNA 0.447 54.176 1.319 92.793
1.689 79.625 1.749 92.903
ENSMUSG00000065440 Mirlet7g miRNA 1 80.688 NA NA 1
78.293 1 92.466
ENSMUSG00000065441 Mir128-2 miRNA 1 72.27 1 95.088 1
87.519 1 96.287
ENSMUSG00000065442 Mir20a miRNA 1 83.726 1 94.597
1 86.346 1 93.584
ENSMUSG00000065443 Mir196b miRNA 1 81.196 1 92.474
1 79.426 1 95.74
ENSMUSG00000065444 Mir27a miRNA 1 36.034 1 96.216
1 64.305 1 93.117
ENSMUSG00000065445 Mir143 miRNA 0.526 3.009 1.328 90.092
1.427 25.966 0.792 85.493
ENSMUSG00000065446 Mir139 miRNA 1 85.832 NA NA 1
84.552 1 91.372
ENSMUSG00000065447 Mir302d miRNA 1 84.397 1 95.708
NA NA 1 96.071
ENSMUSG00000065448 Mir154 miRNA 1 72.85 1 96.843 1

81.29 1 93.627
ENSMUSG00000065450 Mir448 miRNA 1 82.092 1 96.847 1 80.015 1 94.297
ENSMUSG00000065451 Mir101a miRNA 1 78.428 NA NA 1 88.693 NA NA
ENSMUSG00000065452 Mir207 miRNA 1 78.054 1 96.268 1 78.155 1 94.904
ENSMUSG00000065453 Mirlet7d miRNA 1 86.172 1 96.216 1 86.666 1 95.273
ENSMUSG00000065454 NA NA NA NA NA NA 1 78.155 1 96.248
ENSMUSG00000065455 Mir21 miRNA 1 82.092 1 95.708 1 74.082 1 95.593
ENSMUSG00000065456 Mir106a miRNA 1 81.24 1 96.268 1 83.158 1 95.74
ENSMUSG00000065457 Mir383 miRNA 1 70.121 1 96.001 1 80.015 1 93.612
ENSMUSG00000065458 Mir181b-1 miRNA 1 80.03 1 97.263 1 84.626 1 95.877
ENSMUSG00000065462 Mir200c miRNA 1 77.407 1 94.848 1 88.274 1 88.512
ENSMUSG00000065463 Snord45c snoRNA 1 81.76 1 93.654 1 82.575 1 90.157
ENSMUSG00000065465 Mir33 miRNA 1 62.689 1 95.088 1 85.58 1 93.734
ENSMUSG00000065466 n-R5s89 rRNA 1 76.686 1 93.899 1 87.103 1 93.261
ENSMUSG00000065468 Mir26b miRNA 1.104 66.944 0.526 71.889 1.726 72.148 0.698 86.699
ENSMUSG00000065469 NA NA NA NA NA NA 1 87.971 1 95.273
ENSMUSG00000065470 Mir149 miRNA 1 69.489 1 94.597 1 79.255 1 86.445
ENSMUSG00000065471 Mir222 miRNA 1 81.465 1 95.504 1 87.971 1 72.369
ENSMUSG00000065472 Mir125b-2 miRNA 1 84.294 1 88.691 1 83.719 1 89.108
ENSMUSG00000065473 Mir19b-2 miRNA 1 79.433 1 96.268 1 81.62 1 91.011
ENSMUSG00000065475 Mir27b miRNA 0.553 3.07 1.325 76.634 1.37 38.683 1.122 95.74
ENSMUSG00000065476 Mir30b miRNA 1 81.925 1 94.887 1 73.598 1 86.991
ENSMUSG00000065477 Mir411 miRNA 1 76.135 1 96.216 1 83.857 1 91.372
ENSMUSG00000065479 Mir125a miRNA 1 85.77 1 95.708 1 87.786 1 96.184
ENSMUSG00000065480 Mir133b miRNA 1 80.251 1 96.138 1 56.497 1 93.612
ENSMUSG00000065481 Mir346 miRNA 1 83.523 1 93.817 NA NA 1 95.35
ENSMUSG00000065482 Mir302c miRNA 1 85.655 1 97.263 1 87.274 1 94.297
ENSMUSG00000065483 Mir181c miRNA 1 62.437 1 94.767

1 85.675 1 95.877
ENSMUSG00000065484 Mir130a miRNA 1 84.661 1 92.137
1 57.907 1 95.35
ENSMUSG00000065485 Mir219-2 miRNA 1 81.196 1 91.361
1 86.985 1 92.181
ENSMUSG00000065486 Mir450-1 miRNA 1 81.196 1 93.27 1
85.21 1 94.904
ENSMUSG00000065489 Mir365-2 miRNA 1.059 77.291 1.214 83.104
0.775 44.678 1.139 92.762
ENSMUSG00000065491 Mir7b miRNA 1 82.092 1 96.013 1
74.325 1 95.877
ENSMUSG00000065492 Mir34b miRNA 1 82.231 1 93.654
1 84.83 1 94.567
ENSMUSG00000065493 Mir34a miRNA 1.833 63.804 1.023 82.904
0.534 27.86 1.145 76.831
ENSMUSG00000065494 Mir28 miRNA 1 73.292 1 95.211 1
87.103 1 95.676
ENSMUSG00000065495 Mir150 miRNA 1 73.487 1 94.848
NA NA 1 90.463
ENSMUSG00000065497 Mir410 miRNA 1 86.843 1 97.129
1 82.685 1 96.287
ENSMUSG00000065498 Mir379 miRNA 1 86.474 1 95.708
1 78.864 1 90.125
ENSMUSG00000065499 Mir384 miRNA 1 84.534 1 96.407
1 75.155 1 93.316
ENSMUSG00000065502 Mir202 miRNA 1 85.203 1 97.129
1 64.024 1 91.105
ENSMUSG00000065505 Mir148a miRNA 1.031 81.01 1.47 83.104 0.66
69.445 0.443 83.724
ENSMUSG00000065507 Mir204 miRNA 1 84.294 NA NA 1
88.693 1 93.612
ENSMUSG00000065508 Mir17 miRNA 1 82.231 1 96.897 1
79.625 1 93.457
ENSMUSG00000065509 Mir421 miRNA 0.674 58.264 1.247 85.756
1.194 88.41 1.343 94.153
ENSMUSG00000065510 Mir361 miRNA 1 75.761 1 97.263
1 85.675 1 84.499
ENSMUSG00000065511 Mir129-2 miRNA 1 77.291 1 95.735
1 84.071 1 95.273
ENSMUSG00000065512 Mir138-2 miRNA 1 78.909 1 97.049
1 86.144 1 92.748
ENSMUSG00000065513 Mir26a-1 miRNA 1.09 79.709 0.589 70.29
1.556 50.091 1.767 48.857
ENSMUSG00000065514 Mir106b miRNA 1 77.182 1 96.847
1 77.294 1 94.384
ENSMUSG00000065515 Mir152 miRNA 1 54.983 NA NA 1
64.624 1 71.653
ENSMUSG00000065516 Mir214 miRNA 1 79.165 1 96.68 NA
NA 1 93.734
ENSMUSG00000065518 Mir423 miRNA 1 70.862 1 96.763
1 86.666 1 94.654
ENSMUSG00000065519 Mir10a miRNA 1 86.57 1 93.908 1
86.084 1 95.92
ENSMUSG00000065520 Mir128-1 miRNA 1 86.474 1 93.654

1 72.378 1 95.92
ENSMUSG00000065521 Mir296 miRNA 1 82.891 NA NA 1
85.464 1 96.192
ENSMUSG00000065523 Mir192 miRNA 1 81.823 NA NA 1
84.724 1 93.627
ENSMUSG00000065524 Mir135a-2 miRNA 1 84.564 1 95.708
NA NA 1 96.183
ENSMUSG00000065527 Mir93 miRNA 1 85.499 NA NA 1
77.294 1 95.273
ENSMUSG00000065529 Mir22 miRNA 1 83.169 NA NA 1
77.532 1 96.183
ENSMUSG00000065530 Mir99a miRNA 0.817 56.242 1.18 96.268
1.037 55.81 1.008 96.248
ENSMUSG00000065532 Mir187 miRNA 1 67.477 1 96.68 NA
NA 1 95.273
ENSMUSG00000065533 Mir205 miRNA 1 82.092 1 93.899
1 88.601 1 96.358
ENSMUSG00000065534 Mir324 miRNA 1 82.543 NA NA 1
84.896 1 96.248
ENSMUSG00000065536 Mir98 miRNA 1 84.049 1 97.155 1
87.744 1 95.72
ENSMUSG00000065538 Mir153 miRNA 1 81.925 1 95.431
1 82.685 1 96.248
ENSMUSG00000065539 Mir216a miRNA 1 83.333 1 97.155
1 83.811 1 96.071
ENSMUSG00000065540 Mir126 miRNA 1 72.372 1 94.597
1 87.786 1 95.273
ENSMUSG00000065541 Mir24-2 miRNA 1 85.283 1 96.268
1 84.83 1 95.273
ENSMUSG00000065543 Mir330 miRNA 1 81.01 1 96.948 1
83.158 1 96.192
ENSMUSG00000065544 Mir32 miRNA 0.758 81.647 1.016 85.671
1.297 75.809 0.971 94.608
ENSMUSG00000065546 Mir196a-1 miRNA 0.892 67.867 0.858 95.809
1.31 86.985 1.225 86.445
ENSMUSG00000065547 Mir199a-1 miRNA 0.862 83.09 1.254 96.68 NA NA
1.121 86.991
ENSMUSG00000065548 Mir29c miRNA 1 73.487 NA NA 1
84.724 1 96.287
ENSMUSG00000065551 Mir210 miRNA 1 57.227 NA NA 1
85.921 1 95.273
ENSMUSG00000065552 Mir302a miRNA 1 82.352 1 95.708
1 83.719 1 92.691
ENSMUSG00000065553 Mir103-1 miRNA 1 54.604 1 90.895
1 82.685 1 93.132
ENSMUSG00000065555 Mir219-1 miRNA 1 76.546 1 95.708
1 81.62 1 96.183
ENSMUSG00000065559 Mir206 miRNA 1 79.93 1 95.504 1
82.161 1 96.086
ENSMUSG00000065560 Mir148b miRNA 1 63.804 1 94.597
1 81.525 1 86.699
ENSMUSG00000065561 Mir369 miRNA 1 85.391 NA NA 1
88.274 1 81.251
ENSMUSG00000065562 Mir215 miRNA 1 79.277 1 97.155

1 88.203 1 89.108
ENSMUSG00000065563 Mir103-2 miRNA 0.601 31.333 1.96 79.261
0.847 82.903 1.134 96.183
ENSMUSG00000065564 Mirlet7b miRNA 0.579 11.726 1.42 82.904
1.214 54.809 0.713 89.587
ENSMUSG00000065565 Mir181a-1 miRNA 1 76.686 1 95.708
1 87.686 1 96.358
ENSMUSG00000065567 Mir30c-2 miRNA 1 85.857 1 96.268
1 71.999 1 96.287
ENSMUSG00000065568 Mir344 miRNA 1 85.728 1 96.001
1 85.921 1 94.174
ENSMUSG00000065569 Mir137 miRNA 1 86.819 1 95.708
1 78.155 1 94.153
ENSMUSG00000065570 Mir412 miRNA 1 81.657 1 96.216
1 84.896 1 92.389
ENSMUSG00000065572 Mir130b miRNA 1 82.231 1 96.013
1 80.899 1 95.273
ENSMUSG00000065573 Mir350 miRNA 1 82.092 1 96.847
1 84.724 1 91.372
ENSMUSG00000065574 Mir203 miRNA 1 77.615 1 93.27 1
81.388 1 94.904
ENSMUSG00000065575 Mir449a miRNA 1 86.333 1 95.708
1 78.155 1 93.261
ENSMUSG00000065577 Mir329 miRNA 1 68.846 1 97.263
1 77.037 1 92.762
ENSMUSG00000065578 Mir181b-2 miRNA 1 70.36 1 96.847 1
86.602 1 92.181
ENSMUSG00000065579 Mir425 miRNA 0.684 36.541 1.336 83.104
1.09 80.381 0.865 95.559
ENSMUSG00000065580 Mir15b miRNA 1 85.946 1 95.708
1 83.275 1 96.183
ENSMUSG00000065581 Mir194-1 miRNA 1 82.479 1 94.367
1 84.669 1 93.722
ENSMUSG00000065582 Mir194-2 miRNA 1 74.958 1 92.545
1 76.225 1 95.777
ENSMUSG00000065585 Mir211 miRNA 1 82.982 NA NA 1
88.729 1 94.654
ENSMUSG00000065586 Mir96 miRNA 1 77.916 1 95.211 1
87.744 1 95.832
ENSMUSG00000065587 Mir34c miRNA 1 85.857 1 97.097
1 87.971 1 96.248
ENSMUSG00000065589 Mir301 miRNA 0.863 85.391 NA NA NA
NA 2.341 67.94
ENSMUSG00000065591 Gm22753 snRNA 0.817 82.543 1.018 88.705
1.206 69.002 1.001 91.291
ENSMUSG00000065592 Mir145 miRNA 0.848 31.614 0.961 87.919
1.227 27.732 0.97 93.612
ENSMUSG00000065593 Mir339 miRNA 1.122 70.121 0.932 82.848
0.958 82.436 0.885 92.181
ENSMUSG00000065594 Mir107 miRNA 1 82.543 1 96.847
1 83.538 1 93.156
ENSMUSG00000065596 Mir184 miRNA 1 74.482 NA NA 1
88.693 1 91.807
ENSMUSG00000065597 Mir124a-1 miRNA 1 86.74 NA NA 1

80.899 1 96.287
ENSMUSG00000065599 Mir23b miRNA 0.65 16.02 1.015 92.561
1.515 63.301 0.807 90.611
ENSMUSG00000065600 Mir338 miRNA 1.839 16.124 NA NA
0.325 21.431 0.294 83.724
ENSMUSG00000065602 Mirlet7f-2 miRNA 1 85.391 1 91.532
1 87.103 1 91.335
ENSMUSG00000065603 Mir218-1 miRNA 1 77.763 1 83.104
1 87.971 1 81.56
ENSMUSG00000065604 Mir29b-1 miRNA 1 75.196 1 95.504
1 84.626 1 96.358
ENSMUSG00000065606 Mir16-2 miRNA 1 79.577 1 94.848
1 83.719 1 96.248
ENSMUSG00000065607 Mir331 miRNA 1 84.819 1 91.83 1
88.693 1 95.676
ENSMUSG00000065608 Mirlet7c-2 miRNA 0.631 21.393 1.293 96.843
1.224 37.369 0.817 96.071
ENSMUSG00000065609 Mir7-2 miRNA 1 86.172 1 94.887
1 86.144 1 96.086
ENSMUSG00000065610 Mir29a miRNA 1 45.746 1 95.708
1 76.64 1 85.493
ENSMUSG00000065612 Mir151 miRNA 1 71.763 1 94.597
1 79.625 1 96.192
ENSMUSG00000065613 Mir92-2 miRNA 1 86.333 1 96.843
1 82.903 1 96.248
ENSMUSG00000065616 Mir375 miRNA 1 72.85 NA NA 1
82.436 1 92.181
ENSMUSG00000065617 Mir323 miRNA 1 58.38 1 95.211 1
84.071 1 89.892
ENSMUSG00000065619 Mir183 miRNA 1 79.068 1 96.138
NA NA 1 96.183
ENSMUSG00000065620 Gm24764 snRNA 1 84.564 1 96.847
1 83.719 1 89.052
ENSMUSG00000065622 Gm24763 snRNA 1 82.543 NA NA 1
88.693 1 93.612
ENSMUSG00000065623 Gm24762 snRNA 1 74.958 1 96.216
1 87.898 1 95.877
ENSMUSG00000065625 Gm24765 snRNA 1 86.819 1 90.287
1 85.792 1 96.248
ENSMUSG00000065627 Gm24803 snRNA 1 81.925 NA NA NA
NA 1 95.777
ENSMUSG00000065628 Snord33 snoRNA 1 80.51 1 94.48 1
88.601 1 95.676
ENSMUSG00000065629 Gm24826 snoRNA 1 78.224 1
95.088 1 86.258 1 95.74
ENSMUSG00000065630 Gm24256 snRNA 1 83.968 1 96.763
1 80.163 1 90.455
ENSMUSG00000065634 Gm24252 snoRNA 1 74.689 1
91.584 1 87.173 1 96.192
ENSMUSG00000065635 Gm24253 snoRNA 1 72.655 1
94.96 1 78.642 1 96.287
ENSMUSG00000065636 Gm24254 snRNA 1 81.178 1 93.574
1 86.602 1 95.35
ENSMUSG00000065637 Gm26397 snoRNA 1 67.334 1.037

						92.062	0.962	88.657	0.792	89.525
ENSMUSG00000065640	Snord1c	snoRNA	1	84.324	1	97.263	1	88.349	1	96.287
ENSMUSG00000065642	Snora69	snoRNA	1.863	18.635	0.877	87.452	0.61	27.194	0.708	84.29
ENSMUSG00000065643	Gm25897	snRNA	0.651	30.136	1.218	83.104	1.263	78.864	1.398	83.067
ENSMUSG00000065644	Gm25859	snoRNA	1	81.925	1	91.897	1	85.675	1	95.273
ENSMUSG00000065645	Gm23875	misc_RNA	1	84.564	1	96.378	1	84.83	1	96.287
ENSMUSG00000065647	Gm25896	snoRNA	1	71.896	1	96.216	1	70.214	1	96.287
ENSMUSG00000065648	NA	NA	NA	NA	NA	NA	1	86.666	1	95.92
ENSMUSG00000065649	Snora74a	snoRNA	1.705	80.03	0.753	91.361	NA	NA	0.752	95.676
ENSMUSG00000065651	Gm25973	snoRNA	1	68.727	1	95.708	1	77.037	1	93.685
ENSMUSG00000065653	Gm23105	snoRNA	1	83.09	1	96.268	1	69.728	1	95.877
ENSMUSG00000065656	Gm23104	snRNA	1	82.543	1	96.138	1	84.724	1	96.358
ENSMUSG00000065657	Gm25337	snRNA	1	85.391	1	95.329	NA	NA	1	92.31
ENSMUSG00000065658	Gm23102	snRNA	1	84.049	1	93.654	1	67.823	1	96.042
ENSMUSG00000065659	Gm23103	snRNA	1	84.977	1	96.216	1	87.898	1	94.904
ENSMUSG00000065660	n-R5s170	rRNA	1	75.858	1	90.053	1	85.757	1	96.358
ENSMUSG00000065661	Gm22580	snRNA	1	82.982	1	97.288	1	87.686	1	91.525
ENSMUSG00000065662	n-R5s161	rRNA	1	86.006	1	96.013	1	88.601	1	96.184
ENSMUSG00000065663	Gm22579	snoRNA	0.88	65.388	0.961	96.138	1.18	44.515	1.07	93.016
ENSMUSG00000065664	Gm22578	snRNA	1	44.467	1	96.68	1	87.658	1	96.192
ENSMUSG00000065665	Gm22577	snRNA	1	78.538	1	96.407	1	87.37	1	96.248
ENSMUSG00000065667	Gm22576	snRNA	1	81.657	1	86.604	1	78.155	1	87.447
ENSMUSG00000065669	Gm22581	snoRNA	1.019	85.857	0.953	95.211	1.033	77.17	1.046	92.748
ENSMUSG00000065673	Gm24258	snoRNA	1	86.57	1	95.211	1	62.995	1	96.287
ENSMUSG00000065674	Gm24259	snRNA	1	85.637	1	96.335	1	84.961	1	90.611
ENSMUSG00000065675	Gm24260	snRNA	1	82.705	1	96.68	1	86.985	NA	NA
ENSMUSG00000065676	Snord42b	snoRNA	0.221	40.786	2.082	72.642	2.176	83.397	2.21	95.559
ENSMUSG00000065678	Gm24261	snRNA	1	65.537	1	91.661				

1 64.024 1 96.358
ENSMUSG00000065680 Snord38a snoRNA 1 83.169 1
96.847 1 86.475 1 94.82
ENSMUSG00000065683 Gm23731 snRNA 1 71.896 1 92.253
1 88.693 1 95.273
ENSMUSG00000065684 Gm23733 snRNA 1 72.139 1 97.263
1 75.527 1 95.74
ENSMUSG00000065685 Gm23732 snRNA 1 80.608 1 92.793
1 87.173 1 94.904
ENSMUSG00000065687 Gm23734 snoRNA 1 84.865 1
95.301 1 87.274 1 93.457
ENSMUSG00000065689 n-R5s7 rRNA 1 74.8 1 96.407 1
88.161 1 92.018
ENSMUSG00000065691 n-R5s77 rRNA 1 82.732 1 95.504
1 86.112 1 90.865
ENSMUSG00000065692 Gm25410 snoRNA 1 85.655 1
94.96 1 73.096 1 95.559
ENSMUSG00000065700 Gm22827 snRNA 1 85.77 1 96.847 1
86.666 1 95.72
ENSMUSG00000065701 Rny1 misc_RNA 1 83.09 1 94.597 1
83.275 1 95.92
ENSMUSG00000065702 Gm22828 snRNA 1 84.564 1 95.088
1 79.625 1 95.74
ENSMUSG00000065705 Gm22829 snRNA 1 85.857 1 93.908
1 85.935 1 93.685
ENSMUSG00000065706 Gm22830 snoRNA 1 79.853 1
95.708 1 88.729 1 95.273
ENSMUSG00000065708 NA NA NA NA NA NA 1 87.898
1 91.985
ENSMUSG00000065709 Gm22341 snoRNA 1 65.106 1
96.216 1 87.744 1 93.261
ENSMUSG00000065714 Gm25652 snRNA 1 85.203 1 91.897
1 87.744 1 94.174
ENSMUSG00000065715 Snord7 snoRNA 1.455 66.272 0.684
86.861 1.006 87.898 0.933 93.685
ENSMUSG00000065716 Gm25651 snoRNA 1 73.401 1
96.984 1 86.985 1 95.92
ENSMUSG00000065718 Gm25650 snRNA 1 86.333 1 96.763
1 82.685 1 93.156
ENSMUSG00000065719 n-R5s12 rRNA 1 85.499 1 97.155
1 83.275 1 95.877
ENSMUSG00000065721 Gm26168 misc_RNA 1 84.324 1
92.914 1 80.899 1 96.086
ENSMUSG00000065722 Gm26166 snRNA 1 82.231 1 97.155
1 79.776 1 93.734
ENSMUSG00000065726 Gm26163 snoRNA 1 79.577 1
97.263 1 79.426 1 96.192
ENSMUSG00000065727 Gm26164 snRNA 1 84.263 1 96.013
NA NA 1 95.72
ENSMUSG00000065728 Gm26175 snoRNA 1.999 35.024 1.294
95.088 0.386 48.851 0.998 95.72
ENSMUSG00000065729 NA NA NA NA 1 96.308 1
84.308 1 94.904
ENSMUSG00000065732 Gm24492 snRNA 1 71.896 1 95.301

							1	78.369	1	92.181
ENSMUSG00000065733	Gm24491	snRNA	1	73.585	1	96.378	1	82.74	1	95.273
ENSMUSG00000065734	Snord49a	snoRNA	0.998	71.36	1.22	93.146	0.819	87.519	0.956	90.674
ENSMUSG00000065735	Gm24489	snoRNA	1	86.199	1	96.68	1	83.066	1	93.316
ENSMUSG00000065737	Gm24490	snRNA	1	84.168	1	93.094	1	87.658	1	93.734
ENSMUSG00000065738	Gm24494	snoRNA	1.03	86.57	1.028	94.848	0.945	79.255	0.892	94.82
ENSMUSG00000065739	Gm24493	snoRNA	1	74.745	1	93.654	1	78.293	1	94.99
ENSMUSG00000065742	Gm25007	snRNA	1	75.761	NA	NA	1	88.601	1	95.92
ENSMUSG00000065745	Gm25004	snRNA	1	79.165	1	96.652	1	86.144	1	94.904
ENSMUSG00000065746	Gm25005	snRNA	1	86.474	1	96.407	1	81.169	1	95.74
ENSMUSG00000065747	Gm25006	snRNA	1	83.523	NA	NA	1	86.144	1	96.358
ENSMUSG00000065749	AF357428	snoRNA	1	85.655	1	92.914	1	81.857	1	96.183
ENSMUSG00000065750	Gm23346	snoRNA	0.66	49.047	1.278	93.574	1.184	73.309	0.89	96.248
ENSMUSG00000065751	Gm23345	snRNA	1	81.01	1	97.263	1	87.786	1	95.273
ENSMUSG00000065757	Gm23347	snoRNA	1	80.408	1	97.129	1	86.144	1	92.851
ENSMUSG00000065758	n-R5s10	rRNA	1	80.146	1	95.504	1	83.397	1	95.559
ENSMUSG00000065760	Gm23848	snRNA	1	86.172	1	93.654	1	87.431	1	94.654
ENSMUSG00000065763	Gm23847	snRNA	1	81.01	1	93.899	1	82.161	1	93.612
ENSMUSG00000065764	NA	NA	NA	NA	1	95.088	1	68.266	1	96.184
ENSMUSG00000065767	Gm23849	snRNA	1.494	67.374	0.609	94.597	1.099	81.525	0.877	87.705
ENSMUSG00000065768	Gm23845	snRNA	1	80.751	NA	NA	1	88.41	1	96.183
ENSMUSG00000065769	Gm23846	snRNA	1	85.946	1	95.708	1	79.119	1	92.742
ENSMUSG00000065770	Gm22155	snRNA	1	66.129	1	92.404	1	84.229	1	96.287
ENSMUSG00000065771	n-R5s54	rRNA	1	75.671	1	90.744	1	87.744	1	92.31
ENSMUSG00000065773	Rnu1b6	snRNA	1	86.006	1	95.735	1	86.112	1	95.72
ENSMUSG00000065778	Gm22154	snoRNA	0.814	86.819	1.484	93.574	0.825	60.233	0.847	87.735
ENSMUSG00000065782	Gm22680	snoRNA	1	85.391	1	95.504	1	84.961	1	93.264
ENSMUSG00000065787	Gm22681	snRNA	1	81.52	1	96.763	1			

88.693 1 94.904
ENSMUSG00000065788 Gm22682 snRNA 1 63.126 1 96.013
1 84.724 1 94.654
ENSMUSG00000065789 Gm25869 snRNA 1 85.391 1 96.138
1 88.161 1 95.832
ENSMUSG00000065791 Gm25516 snRNA 1 82.092 NA NA 1
88.274 1 87.7
ENSMUSG00000065792 Gm25515 snRNA 1 86.819 1 96.407
1 83.275 1 95.676
ENSMUSG00000065794 Gm25514 snRNA 1 71.915 1 95.504
1 88.693 1 95.92
ENSMUSG00000065795 Gm25513 snRNA 1 86.145 1 96.308
1 86.831 1 95.877
ENSMUSG00000065797 Gm25512 snRNA 1 81.925 1 92.404
1 86.112 1 94.608
ENSMUSG00000065800 Gm23042 snRNA 1 80.51 1 97.155 1
88.693 1 96.192
ENSMUSG00000065802 Gm23041 snRNA 1 66.76 1 95.735 1
88.601 1 95.72
ENSMUSG00000065803 Gm23040 snRNA 1 80.251 1 93.899
1 88.601 1 96.248
ENSMUSG00000065804 Gm23039 snRNA 1 81.657 1 96.216
1 78.713 1 96.183
ENSMUSG00000065806 n-R5s69 rRNA 1 84.977 1 96.216
1 69.825 1 93.734
ENSMUSG00000065807 Gm23038 snRNA 1 86.333 1 96.897
1 88.693 1 94.904
ENSMUSG00000065811 Gm24695 snRNA 1 80.815 1 96.268
NA NA 1 94.99
ENSMUSG00000065812 Gm24696 snoRNA 1 78.224 1
96.138 1 88.693 1 96.287
ENSMUSG00000065813 n-R5s3 rRNA 1 86.464 1 96.013
1 83.066 1 93.685
ENSMUSG00000065815 Gm24697 snRNA 1 85.637 1 95.708
1 83.925 1 92.381
ENSMUSG00000065817 Gm24698 snoRNA 4.576 23.493 0.24
70.29 0.907 86.985 0.608 93.612
ENSMUSG00000065818 Snord35a snoRNA 0.692 57.227 1.601
93.899 0.901 79.554 0.86 96.248
ENSMUSG00000065819 Gm24693 snRNA 1 67.991 1 96.984
1 84.552 1 94.384
ENSMUSG00000065820 Gm26316 snRNA 1 76.396 1 85.793
1 87.786 1 95.92
ENSMUSG00000065823 n-R5s182 rRNA 1 86.464 NA NA 1
73.759 1 95.72
ENSMUSG00000065825 Gm26314 snRNA 1 81.24 1 94.48 1
87.898 1 96.192
ENSMUSG00000065828 Gm26317 snRNA 1 84.564 1 96.216
1 87.37 1 96.086
ENSMUSG00000065836 NA NA NA NA 1 87.452 1
82.778 1 86.991
ENSMUSG00000065837 Gm23503 snRNA 1 86.112 1 96.68 1
80.749 1 96.139
ENSMUSG00000065840 Gm25187 snoRNA 1 85.203 1

95.301 1 87.971 1 88.197
ENSMUSG00000065843 Gm25186 snRNA 1 71.674 1 83.104
1 54.25 1 96.358
ENSMUSG00000065845 Gm25189 snRNA 1 82.543 1 94.102
1 86.144 1 93.132
ENSMUSG00000065847 Gm25188 snoRNA 1.184 84.452 0.713
71.98 1.18 64.024 0.527 96.287
ENSMUSG00000065851 Gm22352 snRNA 1 83.823 1 92.914
1 67.163 1 96.183
ENSMUSG00000065852 Gm22353 snoRNA 1 81.76 NA NA 1
88.601 1 94.82
ENSMUSG00000065853 NA NA NA NA 1 96.216 1
88.601 1 94.19
ENSMUSG00000065858 Gm22350 snRNA 1 84.452 NA NA 1
87.37 1 95.273
ENSMUSG00000065859 Gm22351 snRNA 1 82.479 1 95.941
1 65.034 1 92.748
ENSMUSG00000065862 Gm24029 snoRNA 1 84.049 1
96.984 1 88.161 1 95.273
ENSMUSG00000065864 Gm24031 snoRNA 1 80.146 1
95.504 1 81.388 1 94.82
ENSMUSG00000065865 Gm24030 snRNA 1 80.815 1 95.708
1 86.144 1 96.248
ENSMUSG00000065866 Gm24032 snRNA 1 86.74 1 95.708 1
86.144 1 96.358
ENSMUSG00000065869 Gm24917 snRNA 1 79.277 1 95.504
1 84.961 1 96.183
ENSMUSG00000065870 Rnu3a snoRNA 2.045 46.013 0.979 89.713
0.499 70.791 0.434 83.724
ENSMUSG00000065872 Gm25681 snRNA 1 84.661 NA NA 1
80.381 1 96.248
ENSMUSG00000065873 n-R5s183 rRNA 1 83.263 1 93.654
1 83.857 1 94.82
ENSMUSG00000065874 Gm25683 snoRNA 1 86.658 1
95.708 1 83.857 1 93.261
ENSMUSG00000065875 Gm25684 snRNA 1 75.196 1 96.652
1 81.976 1 95.877
ENSMUSG00000065876 Gm25682 snRNA 1 77.291 NA NA 1
87.686 1 96.248
ENSMUSG00000065878 Snord34 snoRNA 1 83.263 1
93.765 1 83.719 1 96.183
ENSMUSG00000065881 Gm22866 snRNA 1 72.743 1 95.941
1 77.037 1 96.183
ENSMUSG00000065882 n-R5s168 rRNA 1 84.977 1 95.708
1 84.83 1 96.183
ENSMUSG00000065883 Gm22865 snoRNA 0.665 59.358 0.523
70.29 2.869 16.808 1.632 83.276
ENSMUSG00000065884 Gm22864 snoRNA 1 80.51 1 90.378
1 82.74 1 96.287
ENSMUSG00000065887 n-R5s185 rRNA 1 72.27 1 96.68 1
88.349 1 96.287
ENSMUSG00000065889 Gm22868 snRNA 1 68.16 1 94.48 1
86.666 1 92.52
ENSMUSG00000065892 Gm24521 snRNA 1 84.397 1 97.263

NA NA 1 95.273
ENSMUSG00000065893 n-R5s151 rRNA 1 76.827 1 96.268
1 87.971 1 96.183
ENSMUSG00000065894 n-R5s46 rRNA 1 75.858 1 97.155
1 87.658 1 96.358
ENSMUSG00000065898 Gm24522 snRNA 1 80.608 1 93.654
1 84.626 1 96.139
ENSMUSG00000065899 Gm24523 snoRNA 1 83.169 1
95.504 1 87.686 1 93.457
ENSMUSG00000065903 Gm26111 snRNA 1 85.637 1 95.708
NA NA 1 96.358
ENSMUSG00000065904 Gm26109 snoRNA 0.896 72.139 0.759
91.897 1.469 54.363 1.153 93.722
ENSMUSG00000065905 Gm26110 snRNA 1 49.047 1 95.431
1 86.727 1 92.263
ENSMUSG00000065906 Gm26107 snRNA 1 83.658 1 96.138
1 84.896 1 93.612
ENSMUSG00000065907 Gm26108 snRNA 1 74.689 1 96.847
1 88.274 1 93.722
ENSMUSG00000065911 Gm24447 misc_RNA 1 86.006 1
95.708 NA NA 1 95.676
ENSMUSG00000065913 Gm24448 snRNA 1 83.09 1 94.96 1
85.792 1 94.82
ENSMUSG00000065916 Gm24446 snRNA 1 83.263 1 96.216
1 87.173 1 94.608
ENSMUSG00000065919 Gm24449 snRNA 1.445 37.941 0.628 84.594
1.105 88.203 0.673 92.31
ENSMUSG00000065922 n-R5-8s1 rRNA 1 86.172 NA NA 1
87.103 1 96.358
ENSMUSG00000065924 Gm22797 snoRNA 1 69.715 NA NA
1 86.727 1 96.192
ENSMUSG00000065926 Gm22798 snRNA 1 65.537 1 96.308
1 80.381 1 96.183
ENSMUSG00000065934 Gm25630 snRNA 0.57 41.021 0.947 94.262
1.861 27.047 1.134 96.183
ENSMUSG00000065935 Gm25629 snRNA 1 68.727 NA NA 1
69.974 1 85.493
ENSMUSG00000065936 Gm25628 snRNA 1 73.585 1 91.643
1 87.431 1 95.273
ENSMUSG00000065937 Gm25627 snRNA 1 86.74 1 94.848 1
87.744 1 95.74
ENSMUSG00000065939 Snora2b snoRNA 0.844 68.353 1.011
97.155 NA NA 0.757 83.724
ENSMUSG00000065940 Gm23795 snoRNA 1 83.658 NA NA
1 82.575 1 93.457
ENSMUSG00000065943 Gm23794 snRNA 1 84.977 1 96.68 1
85.757 1 96.086
ENSMUSG00000065945 Gm23796 snRNA 1 79.433 1 96.138
1 87.103 1 95.74
ENSMUSG00000065947 mt-Nd4l protein_coding 1.115 73.292
1.088 91.643 0.823 75.353 0.82 92.181
ENSMUSG00000065950 Gm5908 pseudogene 1 78.428 1
94.262 1 71.839 1 96.183
ENSMUSG00000065952 C330021F23Rik protein_coding 5.45 7.179

0.149 40.053 1.234 73.759 1.228 93.734
ENSMUSG00000065954 Tacc1 protein_coding 0.937 22.731 1.062
72.546 1.007 88.461 1.008 95.273
ENSMUSG00000065956 Gm14850 protein_coding 1 82.705 1
96.216 1 77.17 1 93.734
ENSMUSG00000065979 Cpped1 protein_coding 0.975 75.858
1.005 96.68 NA NA 1.035 86.445
ENSMUSG00000065987 Cd209b protein_coding 1.183 31.477
0.748 42.923 1.128 83.925 1.015 96.287
ENSMUSG00000065990 Aurkaip1 protein_coding 0.903 7.857 0.995
96.68 1.114 28.979 1.034 96.183
ENSMUSG00000065999 Gm13154 protein_coding 1.195 50.913 0.69
40.463 1.21 30.948 1.047 89.72
ENSMUSG00000066000 2610305D13Rik protein_coding 1.173 20.799
0.638 58.277 1.329 63.665 0.892 83.724
ENSMUSG00000066009 Gm13051 protein_coding 1.632 76.546
0.794 94.597 0.77 82.575 0.792 93.584
ENSMUSG00000066026 Dhrs3 protein_coding 0.963 47.266 0.9
55.265 1.148 6.693 1.102 60.31
ENSMUSG00000066027 Gm10436 protein_coding 1 75.196 1
97.263 1 86.144 1 95.559
ENSMUSG00000066030 Oog2 protein_coding 1 81.647 NA NA
1 87.744 1 96.287
ENSMUSG00000066031 Gm13023 protein_coding 1 85.77 1
88.897 NA NA 1 96.192
ENSMUSG00000066036 Ubr4 protein_coding 0.912 1.449 0.962 81.731
1.142 5.864 0.991 96.042
ENSMUSG00000066037 Hnrnpr protein_coding 0.97 85.946 0.98
79.894 1.053 52.432 1.028 93.964
ENSMUSG00000066042 Med18 protein_coding 1.064 35.748 1.014
93.094 0.93 79.524 0.863 85.493
ENSMUSG00000066043 Phactr4 protein_coding 0.869 1.031 1.043
78.705 1.1 29.302 1.022 86.445
ENSMUSG00000066057 Gm1976 lincRNA 1.201 37.091 1.054
95.708 0.788 18.122 1.027 94.153
ENSMUSG00000066058 Cldn19 protein_coding 1.09 69.408
1.115 88.91 0.825 50.184 0.982 92.675
ENSMUSG00000066061 Olfr1335 protein_coding 1 66.897 1
94.96 1 88.461 1 94.654
ENSMUSG00000066068 Gm13611 pseudogene 1 80.815 1
96.216 1 85.134 1 95.832
ENSMUSG00000066071 Cyp4a12a protein_coding 1 84.977 1
93.654 1 66.647 1 94.904
ENSMUSG00000066072 Cyp4a10 protein_coding 2.648 7.397 0.492
56.955 0.767 48.851 1.316 96.184
ENSMUSG00000066097 Cyp2j11 protein_coding 1 81.657 1
87.452 1 74.082 1 93.261
ENSMUSG00000066100 Krtap5-1 protein_coding 1 78.299 1
92.545 1 78.864 1 92.018
ENSMUSG00000066101 Gm10153 protein_coding 1 85.391 NA
NA 1 80.381 1 95.92
ENSMUSG00000066107 Gm12666 protein_coding 1 84.865 1
96.407 1 88.601 1 96.192
ENSMUSG00000066113 Adamtsl1 protein_coding 1.125 44.061

0.921 91.532 0.965 67.648 1.072 76.831
ENSMUSG00000066116 Gm10154 pseudogene 1 83.726 1
96.216 1 83.538 1 94.904
ENSMUSG00000066122 Olfr45 protein_coding 1 77.763 1
96.138 1 78.642 1 95.273
ENSMUSG00000066129 Kndc1 protein_coding 0.399 65.106 NA NA
2.419 74.686 1.317 91.872
ENSMUSG00000066137 Gm11487 protein_coding 1 85.203 1
95.329 1 70.328 1 95.72
ENSMUSG00000066141 Gm11232 protein_coding 1 71.36 1
85.075 1 67.562 1 93.264
ENSMUSG00000066148 Prpf4 protein_coding 1.051 48.462 1.001
95.211 0.948 69.134 0.929 87.992
ENSMUSG00000066149 Cdc26 protein_coding 0.976 51.447 0.992
95.504 1.033 75.527 1.004 93.612
ENSMUSG00000066150 Slc31a1 protein_coding 1.032 47.847
1.082 83.104 0.893 13.848 1.039 75.198
ENSMUSG00000066151 Fkbp15 protein_coding 1.028 37.702
0.906 34.058 1.067 33.787 1.079 42.736
ENSMUSG00000066152 Slc31a2 protein_coding 1.051 8.847 1.037
82.904 0.919 9.456 1.012 93.264
ENSMUSG00000066153 Mup21 protein_coding 0.707 85.499 0.943
93.765 1.494 56.931 0.732 85.493
ENSMUSG00000066154 Mup3 protein_coding 0.34 0.987 1.489 60.309
1.965 5.864 0.93 95.593
ENSMUSG00000066158 AY512931 processed_transcript 1 47.174
1 87.919 1 87.744 1 88.505
ENSMUSG00000066170 E230001N04Rik lincRNA 1.176 78.538 0.75
81.443 1.137 36.95 0.84 81.061
ENSMUSG00000066175 2510046G10Rik protein_coding 0.841 37.248
1.049 88.881 1.134 34.403 0.979 92.31
ENSMUSG00000066178 6030445D17Rik protein_coding 0.992 75.567
0.965 91.83 1.042 67.163 1.049 92.018
ENSMUSG00000066180 Gm10155 pseudogene 1 84.865 1
92.111 1 84.434 1 93.734
ENSMUSG00000066189 Cacng3 protein_coding 1 75.399 1
95.504 1 83.607 1 92.018
ENSMUSG00000066191 Anks6 protein_coding 1.094 35.499 1.04
84.768 0.881 25.688 0.97 95.92
ENSMUSG00000066196 Spag8 protein_coding 0.656 4.115 1.185 83.104
1.288 28.997 1.082 92.903
ENSMUSG00000066197 Gpr139 protein_coding 1 84.534 1
96.407 1 81.492 1 92.181
ENSMUSG00000066232 Ipo7 protein_coding 1.074 37.702 0.961
79.807 0.969 78.045 1.01 83.067
ENSMUSG00000066233 Tmem42 protein_coding 0.956 52.018
1.033 89.434 1.014 82.903 1.085 87.624
ENSMUSG00000066235 Pomgnt2 protein_coding 1.107 12.549
1.049 97.155 0.859 8.634 1.006 96.287
ENSMUSG00000066239 Olfr519 protein_coding 1 84.324 1
97.155 1 86.985 1 96.183
ENSMUSG00000066240 Olfr517 protein_coding 1 84.865 1
96.216 1 86.985 1 95.74
ENSMUSG00000066241 Olfr514 protein_coding 1 86.351 NA

NA 1 86.475 1 94.904
ENSMUSG00000066242 Olfr467 protein_coding 1 80.608 1
96.268 1 76.225 1 96.071
ENSMUSG00000066245 Gm10156 pseudogene 1 84.819 1
96.138 1 87.274 1 95.676
ENSMUSG00000066257 Olfr206 protein_coding 1 84.168 NA
NA 1 83.158 1 92.018
ENSMUSG00000066262 Olfr640 protein_coding 1 77.291 1
96.843 1 87.898 1 96.248
ENSMUSG00000066263 Olfr639 protein_coding 1 85.728 1
95.504 1 84.308 1 96.358
ENSMUSG00000066268 Olfr586 protein_coding 1 83.418 1
95.708 1 73.598 1 94.904
ENSMUSG00000066269 Olfr575 protein_coding 1 86.74 1
96.216 1 88.274 1 93.964
ENSMUSG00000066270 Gm10157 pseudogene 1 84.819 1
96.847 1 81.492 1 94.904
ENSMUSG00000066273 Olfr33 protein_coding 1 75.298 1
97.155 1 84.308 1 72.826
ENSMUSG00000066278 Vps37b protein_coding 1.226 21.278 1.05
84.768 0.778 8.626 0.838 86.991
ENSMUSG00000066306 Numa1 protein_coding 0.927 8.122 0.967 90.287
1.113 14.873 0.953 83.724
ENSMUSG00000066315 Gm12918 pseudogene 1 76.319 1
95.708 1 84.308 1 95.35
ENSMUSG00000066319 Rtp3 protein_coding 1.108 36.438 1.001
90.378 0.899 69.974 0.829 83.724
ENSMUSG00000066324 Impad1 protein_coding 1.037 36.124
0.989 90.287 0.972 54.521 1.008 93.734
ENSMUSG00000066357 Wdr6 protein_coding 1.108 6.23 1.025 93.994
0.879 5.701 0.983 85.493
ENSMUSG00000066359 Tcl1b1 protein_coding 1 75.196 1
95.329 1 86.084 1 95.273
ENSMUSG00000066361 Serpina3c protein_coding 0.969 47.677 1.13
80.806 0.913 86.112 0.989 96.358
ENSMUSG00000066362 Rps13-ps1 pseudogene 1 73.401 1
97.263 1 84.071 1 95.72
ENSMUSG00000066363 Serpina3f protein_coding 1.122 64.956
1.567 85.985 0.567 29.217 0.657 83.724
ENSMUSG00000066366 Serpina1a protein_coding 0.896 38.827
1.671 71.98 0.667 80.163 0.593 88.053
ENSMUSG00000066368 Actl11 protein_coding 1 81.647 1
95.504 1 80.015 1 95.273
ENSMUSG00000066372 Vmn2r65 protein_coding 1 72.985 1
97.155 1 83.066 1 93.722
ENSMUSG00000066378 Gm10160 protein_coding 1 76.546 1
95.196 1 44.865 1 87.992
ENSMUSG00000066382 Iqcf5 protein_coding 1 86.333 1
96.308 NA NA 1 94.608
ENSMUSG00000066389 Rpl31-ps1 pseudogene 1 85.355 1
92.631 1 80.899 1 96.086
ENSMUSG00000066392 Nrxn3 protein_coding 0.37 21.176 1.795
65.165 1.508 73.759 2.065 87.447
ENSMUSG00000066406 Akap13 protein_coding 1.058 66.272

0.905 92.631 1.04 83.275 1.008 95.273
ENSMUSG00000066407 Gm10263 protein_coding 1.446 13.734
0.889 85.418 0.775 49.453 0.974 96.192
ENSMUSG00000066415 Msl2 protein_coding 1.043 15.85 1.023 97.155
0.94 11.314 0.996 91.217
ENSMUSG00000066438 Plekhd1 protein_coding 1.018 84.564
0.765 88.691 1.279 76.923 0.66 85.493
ENSMUSG00000066440 Zfyve26 protein_coding 0.969 38.983
0.995 97.263 1.04 43.481 0.915 83.724
ENSMUSG00000066441 Rdh11 protein_coding 0.921 27.431 1.047
89.434 1.039 42.75 1.014 92.564
ENSMUSG00000066442 Mthfs protein_coding 0.933 74.8 1.04 90.378
1.029 62.995 1.046 92.675
ENSMUSG00000066456 Hmgn3 protein_coding 1.068 15.178 1.048
89.434 0.895 8.027 1.037 82.85
ENSMUSG00000066457 Gm5068 pseudogene 1 85.637 1
88.691 1 74.686 1 95.273
ENSMUSG00000066463 Omt2a protein_coding 1 85.355 1
97.263 1 81.976 1 93.156
ENSMUSG00000066477 Gm16551 lincRNA 1 79.277 1
95.504 1 87.173 1 95.877
ENSMUSG00000066478 Gm5745 pseudogene 1 86.918 1
90.256 NA NA 1 96.086
ENSMUSG00000066489 Rpl26-ps2 pseudogene 1 84.738 1
94.887 NA NA NA NA
ENSMUSG00000066491 Gm6265 pseudogene 1 84.397 1
96.68 NA NA 1 93.734
ENSMUSG00000066500 Izumo2 protein_coding 1 86.351 1
95.941 NA NA 1 92.018
ENSMUSG00000066510 Ankdd1a protein_coding 0.91 50.229
0.573 79.894 1.918 26.663 0.995 87.651
ENSMUSG00000066512 Klk1b5 protein_coding 1 83.658 1
92.561 1 83.397 1 94.904
ENSMUSG00000066513 Klk1b4 protein_coding 1 82.982 1
96.013 NA NA 1 96.184
ENSMUSG00000066515 Klk1b3 protein_coding 1 85.728 NA
NA 1 84.308 1 95.35
ENSMUSG00000066537 Vmn2r57 protein_coding 1 75.761 1
90.116 1 72.464 1 93.132
ENSMUSG00000066538 Gm6254 pseudogene 1.3 86.819 0.719
88.691 1.069 47.776 0.732 94.904
ENSMUSG00000066543 Gm10166 pseudogene 1 80.251 1
96.335 1 86.258 1 94.297
ENSMUSG00000066551 Hmgb1 protein_coding 1.076 9.435 0.977 74.851
0.948 48.851 1.056 44.679
ENSMUSG00000066553 Gm6969 pseudogene 1.32 24.147 0.839
86.277 0.901 73.874 0.984 92.181
ENSMUSG00000066554 Gm10167 pseudogene 1 24 1 83.104
1 80.899 1 94.904
ENSMUSG00000066568 Lsm14a protein_coding 1.059 6.399 0.985
95.504 0.956 10.003 1.036 58.557
ENSMUSG00000066571 4931406P16Rik protein_coding 1.094 71.516
0.755 38.699 1.212 39.439 1.084 71.855
ENSMUSG00000066583 NA NA NA NA 1 97.155 1

82.778	1	96.287
ENSMUSG00000066586	Scgb2b26	protein_coding	1	85.832	1
97.155	1	87.686	1	94.215
ENSMUSG00000066592	Gm7589	protein_coding	1	85.283	1
97.155	1	80.381	1	95.559
ENSMUSG00000066595	Mfsd7b	protein_coding	1.133	4.825	0.974
85.075	0.9	23.607	0.933	84.29
ENSMUSG00000066607	6030419C18Rik	protein_coding	1.077	86.172
1.069	82.904	0.87	38.434	1.35	48.119
ENSMUSG00000066613	Zfp932	protein_coding	1.078	51.189
0.944	90.138	NA	NA	1.046	83.067
ENSMUSG00000066620	Gm4883	pseudogene	1	82.092	1
97.263	1	64.305	1	96.287
ENSMUSG00000066621	Tecpr1	protein_coding	1.052	36.195
1.125	79.261	0.845	3.943	0.897	83.724
ENSMUSG00000066626	Gm5121	pseudogene	1	54.44	1	95.735
1	72.464	1	96.192
ENSMUSG00000066637	Ttc32	protein_coding	0.891	31.614	1.132
87.609	0.986	79.625	0.937	96.192
ENSMUSG00000066640	Fbxl18	protein_coding	0.906	3.009	1.044
71.222	1.058	40.575	1.004	94.608
ENSMUSG00000066643	Wdr35	protein_coding	1.012	73.124	1.008
96.216	0.982	78.954	1.056	69.965
ENSMUSG00000066647	Gm5113	protein_coding	1.004	86.74	NA	NA
1.01	78.864	0.918	84.29
ENSMUSG00000066671	Olfr220	protein_coding	1	80.03	1
90.68	1	85.935	1	83.067
ENSMUSG00000066672	Olfr417	protein_coding	1	69.943	1
95.708	1	86.258	1	93.722
ENSMUSG00000066677	Pydc3	protein_coding	1.109	19.132	1.082
97.288	0.833	11.997	1.862	60.31
ENSMUSG00000066680	Olfr1407-ps1	pseudogene	1	80.608	1
96.268	1	83.925	1	92.31
ENSMUSG00000066682	Pilrb2	protein_coding	1.445	13.492
0.784	70.693	0.884	75.353	1.105	92.018
ENSMUSG00000066684	Pilrb1	protein_coding	1.193	37.702
0.898	65.819	0.935	79.119	1.152	80.573
ENSMUSG00000066687	Zbtb16	protein_coding	0.858	49.047
0.716	54.427	1.631	16.808	1.228	85.629
ENSMUSG00000066688	Gm13083	protein_coding	1	85.857	1
95.088	1	84.724	1	93.722
ENSMUSG00000066693	Gm7493	pseudogene	1	84.397	NA	NA
1	62.791	1	92.181
ENSMUSG00000066697	Gm10172	pseudogene	1	85.59	1	94.887
1	83.066	1	96.071
ENSMUSG00000066704	Cyp2b19	protein_coding	0.863	35.894	0.88
81.443	1.312	33.508	1.459	50.452
ENSMUSG00000066705	Fxyd6	protein_coding	1.042	40.635	1.002
95.088	0.957	58.775	0.969	85.493
ENSMUSG00000066714	Gm8062	pseudogene	1	82.352	1
95.431	1	88.203	1	95.676
ENSMUSG00000066721	Zfp575	protein_coding	1.141	60.435
1.163	93.27	0.755	32.703	1.166	68.696
ENSMUSG00000066723	Vmn1r183	protein_coding	1	84.294	1

96.013 1 76.498 1 95.92
ENSMUSG00000066724 Gm10175 protein_coding 4.58 0.2 0.654
74.483 0.333 4.338 0.731 83.724
ENSMUSG00000066735 Vkorc1l1 protein_coding 0.98 81.01 1.042
91.584 0.982 67.339 1.101 43.377
ENSMUSG00000066747 Olfr878 protein_coding 1 81.76 1
94.262 1 81.857 1 96.287
ENSMUSG00000066748 Olfr145 protein_coding 1 76.686 1
95.088 1 69.974 1 94.904
ENSMUSG00000066749 Olfr877 protein_coding 1 74.689 1
97.263 1 83.811 1 96.183
ENSMUSG00000066750 Olfr876 protein_coding 1 82.982 1
93.899 NA NA 1 95.777
ENSMUSG00000066752 Gm10176 protein_coding 1.119 82.891
0.913 90.116 0.975 71.388 0.772 86.991
ENSMUSG00000066755 Tnfsf18 protein_coding 1.015 81.01 0.729
85.418 1.355 53.449 1.648 73.597
ENSMUSG00000066767 Gm3867 lincRNA 1 82.982 1
95.708 1 63.801 1 95.273
ENSMUSG00000066771 Gm5614 pseudogene 1 66.129 1
95.211 NA NA 1 93.132
ENSMUSG00000066772 Obox3 protein_coding 1 85.655 1
94.597 1 88.274 1 93.612
ENSMUSG00000066797 Zfp648 protein_coding 1 85.59 1
94.262 1 85.935 1 87.705
ENSMUSG00000066798 Zbtb6 protein_coding 1.046 33.854 1.011
94.597 0.941 44.211 0.976 96.183
ENSMUSG00000066800 Rnasel protein_coding 1.271 0 0.878
53.79 0.894 13.551 1.056 91.525
ENSMUSG00000066803 Vmn1r84 protein_coding 1 82.479 1
96.407 1 87.37 1 93.964
ENSMUSG00000066804 Vmn1r83 protein_coding 1 84.168 1
96.138 1 88.161 1 95.273
ENSMUSG00000066807 Gm10179 pseudogene 1 76.319 1
96.308 1 86.985 1 93.261
ENSMUSG00000066810 Gm10181 protein_coding 1 64.014 1
95.211 1 84.504 1 94.016
ENSMUSG00000066820 Vmn2r28 protein_coding 1 73.487 1
95.735 1 86.831 1 94.608
ENSMUSG00000066828 Vmn2r37 protein_coding 1 83.418 NA
NA NA NA 1 96.139
ENSMUSG00000066829 Zfp810 protein_coding 1.042 57.38 1.008
87.147 NA NA 0.942 93.584
ENSMUSG00000066838 Zfp772 protein_coding 0.999 63.385
0.868 60.678 1.155 25.966 0.963 83.952
ENSMUSG00000066839 Ecsit protein_coding 0.877 5.544 1.028 79.864
1.11 61.418 0.994 96.192
ENSMUSG00000066842 Hmcn1 protein_coding 0.994 86.843 0.999
92.561 1.009 73.598 1.21 92.018
ENSMUSG00000066850 Vmn1r65 protein_coding 1 83.418 1
95.708 1 88.693 1 96.184
ENSMUSG00000066861 Oas1g protein_coding 1.483 7.332 1.028 96.013
0.655 38.534 1.758 42.736
ENSMUSG00000066877 Nck2 protein_coding 0.959 72.743 0.922

69.175 1.133 25.455 1.01 93.045
ENSMUSG00000066878 Gm10184 protein_coding 1 86.819 1
92.545 1 79.776 1 84.499
ENSMUSG00000066880 Zfp617 protein_coding 1.119 19.315
0.862 52.266 1.032 86.666 0.988 95.273
ENSMUSG00000066892 Fbxl12 protein_coding 0.972 85.77 0.955
65.165 1.072 37.741 0.909 83.724
ENSMUSG00000066894 Vsig10 protein_coding 0.888 11.973 0.94
64.555 1.201 5.977 0.912 83.724
ENSMUSG00000066897 Olfr872 protein_coding 1 83.968 1
95.735 1 83.066 1 95.273
ENSMUSG00000066899 Olfr870 protein_coding 1 69.715 1
96.847 NA NA 1 95.35
ENSMUSG00000066900 Suds3 protein_coding 0.916 25.111 0.967
84.594 1.13 18.797 0.996 94.99
ENSMUSG00000066905 Olfr860 protein_coding 1 81.01 1
95.301 1 84.961 1 96.248
ENSMUSG00000066912 Olfr833-ps1 pseudogene 1 83.333 1
96.378 1 85.352 NA NA
ENSMUSG00000066914 Olfr831-ps1 protein_coding 1 75.671 1
96.216 1 86.258 1 94.19
ENSMUSG00000066936 Gm10188 protein_coding 1 81.52 1
96.407 NA NA 1 95.72
ENSMUSG00000066938 Gm10190 protein_coding 0.484 44.922 4.06
82.809 0.508 67.029 1.025 94.19
ENSMUSG00000066944 4921513D11Rik protein_coding 3.106 57.133
0.204 75.618 1.586 52.432 0.966 92.903
ENSMUSG00000066952 Myo1h protein_coding 0.795 21.393 0.992
96.013 1.27 23.604 0.886 85.493
ENSMUSG00000066958 Gm16391 pseudogene 1 86.474 1
96.216 1 80.749 1 96.184
ENSMUSG00000066975 Cryba4 protein_coding 1.24 52.799 0.54
60.678 1.497 61.841 1.267 90.177
ENSMUSG00000066979 Bub3 protein_coding 0.964 18.731 1.03
86.156 1.008 76.498 1.03 50.74
ENSMUSG00000066983 Gm16519 protein_coding 1 81.465 1
96.138 1 83.538 1 95.74
ENSMUSG00000066990 Gm10192 protein_coding 1 80.03 1
92.631 1 87.744 1 94.904
ENSMUSG00000067001 Serpinb7 protein_coding 1.417 47.174 1.61
93.654 0.437 45.093 1.569 86.133
ENSMUSG00000067006 Serpinb5 protein_coding 1.128 81.76 0.985
90.378 0.901 87.898 0.75 86.699
ENSMUSG00000067010 Vmn2r10 protein_coding 1 76.968 1
95.504 1 87.37 1 94.608
ENSMUSG00000067028 Cntnap5b protein_coding 1 75.761 1
96.843 1 87.686 1 96.358
ENSMUSG00000067038 Rps12-ps3 protein_coding 1 85.499 1
95.301 1 83.857 1 94.752
ENSMUSG00000067047 Olfr719-ps pseudogene 1 80.146 1
94.262 1 79.554 1 94.904
ENSMUSG00000067049 Unc93a protein_coding 2.18 0.767 0.916
94.262 0.499 3.432 0.373 83.724
ENSMUSG00000067058 Rps15a-ps5 pseudogene 1 85.728 1

93.146 1 87.37 1 88.797
ENSMUSG00000067063 Gm6990 pseudogene 0.765 77.615 0.671
93.654 1.947 60.104 0.44 89.475
ENSMUSG00000067064 Olfr1416 protein_coding 1 72.372 1
95.431 1 81.032 1 93.734
ENSMUSG00000067071 Hes6 protein_coding 0.916 45.728 0.997
97.155 1.095 65.8 1.017 94.654
ENSMUSG00000067085 Gm10197 protein_coding 0.717 18.731 NA
NA 1.46 39.471 0.888 84.29
ENSMUSG00000067101 1700010H22Rik protein_coding 1 86.199
NA NA 1 83.066 1 94.654
ENSMUSG00000067103 AY702103 lincRNA 1 82.092 1
97.097 1 87.971 1 96.358
ENSMUSG00000067106 Gm5529 pseudogene 1 79.709 NA NA
1 75.009 1 95.832
ENSMUSG00000067122 Gm10198 protein_coding 1 71.763 1
97.263 NA NA 1 95.92
ENSMUSG00000067147 Rpl7a-ps11 pseudogene 1 85.77 1 90.053
1 83.719 1 92.381
ENSMUSG00000067148 Polr1c protein_coding 1.003 72.546
0.995 94.848 0.998 78.498 1.059 59.394
ENSMUSG00000067149 Igj protein_coding 1.921 8.477 0.928 95.196
0.558 0.879 0.97 94.82
ENSMUSG00000067150 Xpo5 protein_coding 1.003 65.284 NA NA
0.979 58.775 1.032 93.722
ENSMUSG00000067156 Gm7337 pseudogene 1 78.639 1
95.504 NA NA 1 96.183
ENSMUSG00000067158 Col4a4 protein_coding 1.143 36.438
0.679 55.71 1.283 39.471 1.05 94.174
ENSMUSG00000067161 Gm5560 pseudogene 1 79.165 1
94.96 1 86.475 1 92.181
ENSMUSG00000067173 Mrgpra4 protein_coding 3.493 5.481 NA NA
0.239 3.718 0.618 83.724
ENSMUSG00000067186 Olfr132 protein_coding 1 81.178 1
95.504 1 86.475 1 93.722
ENSMUSG00000067189 Gm7335 pseudogene 1 81.125 1
95.301 1 87.744 1 92.675
ENSMUSG00000067194 Eif1ax protein_coding 0.959 73.585
1.053 89.434 0.993 87.744 1.051 71.855
ENSMUSG00000067199 Frat1 pseudogene 0.782 33.415 1.105 90.68
1.157 62.995 0.86 83.952
ENSMUSG00000067201 H2-M9 protein_coding 1.133 22.871 0.792
67.513 1.112 79.554 1.031 94.608
ENSMUSG00000067203 H2-K2 pseudogene 1.137 24.481 0.964 82.503
0.915 55.9 0.797 83.724
ENSMUSG00000067212 H2-T23 protein_coding 1.324 0 0.827 0
0.913 68.266 0.958 92.181
ENSMUSG00000067215 Usp51 protein_coding 1.554 58.896 0.992
93.899 0.648 42.997 0.567 84.29
ENSMUSG00000067219 Nipal1 protein_coding 1.657 22.205
0.817 85.116 0.738 52.658 1.079 83.581
ENSMUSG00000067224 Gm3695 pseudogene 1.015 84.168 2.214
90.68 0.445 47.12 0.887 90.691
ENSMUSG00000067225 Cyp2c54 protein_coding 1 85.946 1

96.68 1 85.921 1 95.559
ENSMUSG00000067229 Cyp2c66 protein_coding 1 85.655 1
96.948 1 83.397 1 94.904
ENSMUSG00000067231 Cyp2c65 protein_coding 1 75.115 1
94.848 1 81.857 1 95.877
ENSMUSG00000067235 H2-Q10 protein_coding 0.615 0.987 1.039
95.431 1.568 9.176 1.074 94.904
ENSMUSG00000067242 Lgi1 protein_coding 1.06 46.533 1.277
82.904 0.738 6.858 0.87 83.724
ENSMUSG00000067261 Foxd3 protein_coding 0.648 33.415 1.184
96.843 1.301 48.935 0.997 91.625
ENSMUSG00000067262 Vmn1r239-ps pseudogene 1 85.129 1
96.763 1 86.475 1 94.904
ENSMUSG00000067274 Rplp0 protein_coding 1.038 31.732 0.943
15.364 1.022 76.498 0.984 93.685
ENSMUSG00000067276 Capn6 protein_coding 1.115 49.202 1.162
94.367 0.772 45.799 1.026 95.559
ENSMUSG00000067279 Ppp1r3c protein_coding 1.059 52.134
1.172 83.104 0.807 17.381 1.19 92.748
ENSMUSG00000067285 Gm16223 protein_coding 1 86.819 1
96.268 NA NA 1 95.273
ENSMUSG00000067288 Rps28 protein_coding 1.016 86.658 0.945
79.807 1.04 52.432 1.009 96.139
ENSMUSG00000067292 Gm10203 protein_coding 1 85.283 NA
NA 1 82.778 1 94.825
ENSMUSG00000067297 2010002M12Rik protein_coding 1.36 18.635
0.85 90.053 0.866 30.827 1.229 73.823
ENSMUSG00000067299 Crygd protein_coding 1 83.169 1
96.407 1 58.516 1 94.174
ENSMUSG00000067321 Gm7931 pseudogene 1 81.925 1
97.263 1 88.729 1 92.691
ENSMUSG00000067331 Gm10205 pseudogene 1 86.658 1
96.763 1 83.925 1 93.964
ENSMUSG00000067336 Bmpr2 protein_coding 0.703 7.43 0.898 95.301
1.579 5.964 0.959 92.561
ENSMUSG00000067344 Rps25-ps1 pseudogene 1.453 80.815 0.847
87.322 0.814 83.066 1.324 95.877
ENSMUSG00000067351 Rps15a-ps2 pseudogene 1 85.728 1
96.68 1 86.144 1 94.904
ENSMUSG00000067352 Gm14149 lincRNA 1 84.865 1
89.494 1 83.275 1 94.825
ENSMUSG00000067356 B430203G13Rik protein_coding 1.071 85.857
0.714 68.115 1.31 19.025 0.783 86.699
ENSMUSG00000067360 Pramel3 protein_coding 1 81.823 NA
NA 1 86.666 1 96.287
ENSMUSG00000067365 Tmem128 protein_coding 1.024 41.177
0.963 69.175 1.011 65.235 0.984 93.685
ENSMUSG00000067367 Lyar protein_coding 0.993 85.77 0.974 84.594
1.03 50.091 1.072 78.948
ENSMUSG00000067369 Trmt2b protein_coding 1.124 53.809
0.964 96.268 0.922 31.135 0.932 92.675
ENSMUSG00000067370 B3galt4 protein_coding 1.084 28.927 1.02
97.097 0.902 29.217 0.9 83.724
ENSMUSG00000067377 Tspan6 protein_coding 1.094 15.972

0.945 87.919 0.97 52.087 1.243 60.466
ENSMUSG00000067389 Gm17080 antisense 0.708 4.176 0.909 87.998
1.551 8.93 1.1 96.192
ENSMUSG00000067399 Trim43c protein_coding 0.777 84.049
0.671 75.345 1.912 22.444 0.778 84.29
ENSMUSG00000067416 Actl9 pseudogene 1 83.333 NA NA 1
84.896 1 92.762
ENSMUSG00000067424 Zfp563 protein_coding 1.101 72 0.936
81.731 0.971 73.759 1.006 89.428
ENSMUSG00000067430 Zfp763 protein_coding 1.081 25.207 0.9
68.115 1.028 66.043 1.02 90.455
ENSMUSG00000067438 Hmx1 protein_coding 1 82.891 1
97.155 1 82.161 1 93.964
ENSMUSG00000067441 Gm14920 protein_coding 1 86.918 1
96.68 1 87.744 1 94.904
ENSMUSG00000067455 Hist1h4j protein_coding 0.571 25.492
1.455 83.104 1.198 71.232 1.103 91.783
ENSMUSG00000067482 Olfr1456-ps1 pseudogene 1 75.858 1
97.288 1 88.729 1 95.273
ENSMUSG00000067513 Olfr1436 protein_coding 1 81.52 NA NA
1 84.504 1 95.877
ENSMUSG00000067519 Olfr262 protein_coding 1 78.744 1
97.263 1 74.325 1 96.071
ENSMUSG00000067520 Olfr1432 pseudogene 1 71.516 1
96.68 1 87.971 1 95.877
ENSMUSG00000067522 Olfr76 protein_coding 1 83.523 1
95.504 1 88.657 1 93.156
ENSMUSG00000067524 Olfr1428 protein_coding 1 83.418 1
95.329 1 70.623 1 96.184
ENSMUSG00000067525 Olfr1427 protein_coding 1 83.263 1
95.35 NA NA 1 91.872
ENSMUSG00000067526 Olfr1425 protein_coding 1 71.36 NA NA
NA NA 1 94.99
ENSMUSG00000067528 Olfr1424 protein_coding 1 80.688 NA
NA 1 76.754 1 93.132
ENSMUSG00000067529 Olfr1423 protein_coding 1 85.77 1
95.708 1 88.41 1 93.584
ENSMUSG00000067541 A630073D07Rik protein_coding 1 83.823
1 95.708 1 87.744 NA NA
ENSMUSG00000067543 NA NA NA NA 1 95.431 1
85.675 1 94.19
ENSMUSG00000067561 Dmrtc1c2 protein_coding 1 81.657 NA
NA 1 86.258 1 96.071
ENSMUSG00000067562 NA NA NA NA 1 95.431 1
81.857 1 96.184
ENSMUSG00000067567 Hdac8 protein_coding 1.004 51.737 0.927
82.671 1.072 84.724 1.029 95.273
ENSMUSG00000067575 Rpl35a-ps3 pseudogene 1 86.658 1
94.367 1 88.601 1 77.621
ENSMUSG00000067578 Cbln4 protein_coding 1 84.294 1
96.216 1 79.325 1 94.904
ENSMUSG00000067586 S1pr3 protein_coding 1.193 36.438 1.075
88.533 0.78 31.887 1.238 36.174
ENSMUSG00000067591 Klra3 protein_coding 0.706 42.722 2.453

82.904	0.575	87.786	1.257	74.193
ENSMUSG00000067594	Krt77	protein_coding	3.623	6.399	0.911	96.268
0.303	8.472	2.086	76.831
ENSMUSG00000067596	Krt74	protein_coding	1	85.59	NA	NA	1
74.535	1	95.593
ENSMUSG00000067599	Klra7	protein_coding	1.152	83.968	1.424
82.219	0.608	37.906	0.909	93.685
ENSMUSG00000067604	Nms	protein_coding	1	86.464	1
95.504	1	85.935	1	92.851
ENSMUSG00000067608	Pcna-ps2	protein_coding	1	79.165	1
95.196	1	74.853	1	95.35
ENSMUSG00000067610	Klri1	protein_coding	1.383	50.377	NA	NA
0.829	68.82	0.986	96.086
ENSMUSG00000067615	Krt81	protein_coding	1	85.283	1
95.504	1	81.032	1	90.463
ENSMUSG00000067616	Klk11	protein_coding	0.857	38.195	0.978
96.847	1.197	57.227	0.672	83.724
ENSMUSG00000067627	Gm10217	protein_coding	1	72.139	1
96.843	NA	NA	1	93.156
ENSMUSG00000067629	Syngap1	protein_coding	0.893	28.804
1.042	92.561	1.075	61.171	1.128	93.156
ENSMUSG00000067642	Gpr113	protein_coding	1	62.437	1
85.756	1	84.961	1	83.581
ENSMUSG00000067653	Ankrd23	protein_coding	2.081	2.897	1.115
90.092	0.432	0.72	0.867	96.358
ENSMUSG00000067656	Slc22a27	protein_coding	1	83.823	1
97.155	1	82.161	1	95.273
ENSMUSG00000067679	Obp1b	protein_coding	1	73.585	1
96.138	1	69.825	1	96.086
ENSMUSG00000067684	Obp1a	protein_coding	1	83.823	1
96.652	1	85.675	1	96.287
ENSMUSG00000067698	Gm10220	protein_coding	1	75.115	1
93.707	1	87.37	1	94.904
ENSMUSG00000067700	Gm5862	protein_coding	1	82.705	1
97.129	1	84.896	1	96.183
ENSMUSG00000067702	Tuba3a	protein_coding	1	84.661	1
95.504	1	86.602	1	96.139
ENSMUSG00000067713	Prkag1	protein_coding	1.025	39.075
1.007	95.504	0.972	61.288	0.965	85.493
ENSMUSG00000067714	Lpar5	protein_coding	1.467	0.664	0.808	68.115
0.845	9.131	1.03	94.654
ENSMUSG00000067716	Gm7275	protein_coding	1	85.728	1
96.138	1	77.532	1	93.132
ENSMUSG00000067722	BC003965	protein_coding	0.94	12.309
1.016	96.216	1.046	58.616	1.029	92.018
ENSMUSG00000067724	Gbx1	protein_coding	0.816	80.815	1.218
94.597	1.01	87.686	1.322	81.901
ENSMUSG00000067736	Gm10222	protein_coding	0.458	8.122	1.31
87.604	1.669	28.603	0.868	90.177
ENSMUSG00000067745	Gm14243	lincRNA	1	83.09	1	96.843
1	85.792	1	95.273
ENSMUSG00000067750	Khdc1a	protein_coding	4.736	40.065
0.488	87.313	0.432	81.62	0.513	86.445
ENSMUSG00000067764	Xlr5c	protein_coding	1	83.823	NA	NA

1 85.464 1 96.183
ENSMUSG00000067768 Xlr4b protein_coding 1.374 80.03 NA NA
0.889 73.874 1.133 60.31
ENSMUSG00000067773 Defb41 protein_coding 1 63.534 1
95.301 1 85.464 1 95.74
ENSMUSG00000067780 Pi15 protein_coding 1.105 79.577 1.169
72.642 0.774 12.907 1.269 71.653
ENSMUSG00000067786 Nnat protein_coding 0.631 22.481 1.082
83.104 1.464 43.586 1.67 67.94
ENSMUSG00000067787 Blcap protein_coding 0.906 58.077 1.172
71.617 0.939 29.451 1.16 96.086
ENSMUSG00000067798 Magi2 protein_coding 1 82.705 1
94.48 1 85.935 1 96.287
ENSMUSG00000067818 Myl9 protein_coding 1.034 20.942 1.051
89.434 0.921 8.382 0.958 84.29
ENSMUSG00000067825 Pex26 protein_coding 0.977 65.388 1.013
94.767 1.013 84.896 0.928 83.724
ENSMUSG00000067833 2900097C17Rik protein_coding 0.973 66.129
1.155 82.904 0.891 48.133 0.843 83.724
ENSMUSG00000067835 Gm14661 antisense 1 86.57 NA NA 1
83.158 1 94.99
ENSMUSG00000067847 Romo1 protein_coding 0.806 1.343 0.975 92.561
1.273 23.334 1.019 91.872
ENSMUSG00000067848 4933402N22Rik protein_coding 1 82.982
1 97.155 1 74.686 1 95.832
ENSMUSG00000067851 Arfgef1 protein_coding 1.006 86.351
0.938 79.261 1.059 52.574 0.982 93.264
ENSMUSG00000067855 Speer3 protein_coding 1 76.319 1
95.301 1 86.552 1 94.654
ENSMUSG00000067860 Zic3 protein_coding 1 81.52 NA NA 1
85.921 1 95.74
ENSMUSG00000067869 Tcea1-ps1 pseudogene 1 82.092 1
79.807 1 52.8 1 95.273
ENSMUSG00000067870 Rpl31-ps8 pseudogene 14.821 0 NA NA
0.104 0.72 1.318 71.008
ENSMUSG00000067872 Ccdc87 protein_coding 1.856 39.295
0.535 70.864 1.009 84.308 1.293 76.697
ENSMUSG00000067873 Htatsf1 protein_coding 1.006 77.497
0.969 71.889 1.028 62.307 1.073 48.857
ENSMUSG00000067878 Mtap7d3 protein_coding 1.16 45.61 0.908
92.355 0.947 75.637 0.876 87.331
ENSMUSG00000067879 3110035E14Rik protein_coding 1.073 86.333
1.785 84.768 0.52 60.96 1.125 96.287
ENSMUSG00000067882 Casp16 polymorphic_pseudogene 0.55 73.292
0.726 79.807 2.504 36.011 1.89 94.215
ENSMUSG00000067889 Sptbn2 protein_coding 0.667 2.536 0.99
81.731 1.511 7.024 0.835 85.493
ENSMUSG00000067909 Slxl1 protein_coding 1 86.464 1
96.216 1 88.161 1 94.608
ENSMUSG00000067916 Gm13139 protein_coding 1.207 85.77 0.446
79.475 1.85 43.185 1.928 83.276
ENSMUSG00000067924 Cxx1b protein_coding 0.879 14.403 1.14
79.864 0.999 82.74 1.077 55.485
ENSMUSG00000067925 Cxx1a protein_coding 1.038 71.915 0.886

96.138 1.088 70.623 1.064 74.607
ENSMUSG00000067928 Zfp760 protein_coding 1.073 83.968
0.906 78.471 1.029 46.575 0.939 95.273
ENSMUSG00000067929 Gm10226 protein_coding 1.037 44.694
1.207 91.456 0.801 23.48 0.855 87.218
ENSMUSG00000067931 Zfp948 protein_coding 1.09 36.686 NA
NA 0.947 36.248 1.126 46.846
ENSMUSG00000067942 Zfp160 protein_coding 1.107 2.651 0.958
91.661 0.946 36.786 1.039 93.156
ENSMUSG00000067949 Vmn1r-ps144 pseudogene 1 70.121 1
96.268 NA NA 1 96.071
ENSMUSG00000067951 Vmn1r227 processed_transcript 1 82.982
1 93.529 1 88.076 1 94.384
ENSMUSG00000067971 Olfr1321 protein_coding 1 83.263 1
96.984 NA NA NA NA
ENSMUSG00000067995 Gtf2f2 protein_coding 1.059 57.133
0.905 64.624 1.04 57.907 1.123 72.616
ENSMUSG00000067996 Bpifb9b protein_coding 1 84.865 1
96.001 1 88.161 1 94.904
ENSMUSG00000067998 Bpifb9a protein_coding 1 77.615 NA
NA 1 86.475 1 95.74
ENSMUSG00000068008 Bpifb3 protein_coding 1 82.092 1
96.013 1 74.082 1 93.156
ENSMUSG00000068011 2510049J12Rik protein_coding 0.509 46.413
2.536 83.104 0.773 87.431 0.529 83.724
ENSMUSG00000068014 Gm14648 pseudogene 1 86.658 1
96.268 1 86.475 1 92.018
ENSMUSG00000068015 Lrch1 protein_coding 1.057 7.65 0.887 23.928
1.069 45.94 1.012 88.245
ENSMUSG00000068036 Mllt4 protein_coding 0.945 27 0.984 92.062
1.071 58.051 0.986 86.991
ENSMUSG00000068037 Mas1 protein_coding 0.809 86.172 1.224
91.456 1.005 49.967 0.948 85.493
ENSMUSG00000068039 Tcp1 protein_coding 1.069 55.789 0.955
94.96 0.983 74.535 1.028 76.831
ENSMUSG00000068040 Tm9sf4 protein_coding 1.007 82.352
1.052 72.642 0.945 7.329 0.983 96.183
ENSMUSG00000068046 4930430D24Rik protein_coding 1 81.465
1 95.708 1 82.74 1 95.273
ENSMUSG00000068048 Rhox9 protein_coding 1 82.352 1
95.504 1 88.076 1 93.316
ENSMUSG00000068067 1110057P08Rik protein_coding 1 85.946
1 96.68 1 81.032 1 96.183
ENSMUSG00000068068 Gm7735 protein_coding 1 82.891 NA
NA 1 83.857 1 93.016
ENSMUSG00000068071 Gm6358 protein_coding 1 86.145 1
95.329 1 84.308 1 94.752
ENSMUSG00000068073 1110025L11Rik protein_coding 1 85.203
1 94.89 1 82.778 1 95.74
ENSMUSG00000068074 Gm10228 protein_coding 1 85.728 1
97.263 1 80.015 1 95.273
ENSMUSG00000068075 Gm10229 protein_coding 1 68.66 NA NA
1 88.657 1 93.964
ENSMUSG00000068078 2310034C09Rik pseudogene 1 85.857 NA

NA 1 84.896 1 93.457
ENSMUSG00000068079 Tcf15 protein_coding 0.885 39.712 1.072
95.431 1.05 66.269 0.936 92.181
ENSMUSG00000068082 Grxcr1 protein_coding 1 82.891 NA
NA 1 87.744 1 96.287
ENSMUSG00000068083 Cyp2d40 protein_coding 1 67.374 1
95.088 NA NA 1 95.35
ENSMUSG00000068085 Cyp2d11 protein_coding 1 68.66 1
96.013 1 42.065 1 92.381
ENSMUSG00000068086 Cyp2d9 protein_coding 1 81.647 NA
NA 1 86.985 1 86.699
ENSMUSG00000068101 Cenpm protein_coding 1.276 12.72 0.882 83.58
0.892 58.516 1.578 76.831
ENSMUSG00000068105 Tnfrsf13c protein_coding 1.371 83.09 1.605
82.904 0.454 26.057 0.99 93.016
ENSMUSG00000068113 Gm4907 protein_coding 1 86.658 1
92.137 1 67.648 1 93.132
ENSMUSG00000068114 Ccdc134 protein_coding 0.994 83.418 1.07
83.104 0.94 57.091 1.217 44.679
ENSMUSG00000068115 Ninl protein_coding 0.991 69.24 0.897 85.075
1.128 31.479 0.964 85.493
ENSMUSG00000068117 Mei1 protein_coding 1 76.319 NA NA
1 79.325 1 96.086
ENSMUSG00000068120 Gm10231 pseudogene 1.007 74.147 1.997
82.904 0.496 37.103 0.577 96.192
ENSMUSG00000068122 Agtr2 protein_coding 1.251 39.075 1.145
96.763 0.7 20.258 1.259 83.276
ENSMUSG00000068129 Cst7 protein_coding 1.873 21.146 0.968
92.545 0.552 42.376 0.88 92.181
ENSMUSG00000068130 Zfp442 protein_coding 1.027 84.738 NA
NA 0.919 87.898 0.897 92.748
ENSMUSG00000068134 Zfp120 protein_coding 1.17 0.577 0.977
74.483 0.875 15.904 0.895 86.699
ENSMUSG00000068141 Gm10232 pseudogene 1 86.199 1
96.407 NA NA 1 94.82
ENSMUSG00000068149 BC049702 protein_coding 1 85.129 NA
NA 1 88.657 1 95.273
ENSMUSG00000068151 A230006K03Rik lincRNA 1 79.577 1
97.155 1 81.976 1 96.086
ENSMUSG00000068165 Gm10233 pseudogene 1 73.124 1
96.268 1 80.749 1 95.832
ENSMUSG00000068167 Csnka2ip protein_coding 1 84.977 1
91.955 1 84.896 1 92.018
ENSMUSG00000068173 4930525M21Rik pseudogene 1 77.113 NA
NA 1 84.896 1 95.74
ENSMUSG00000068180 AY512915 antisense 1 82.543 1
95.504 1 88.657 1 96.183
ENSMUSG00000068181 Gm10234 protein_coding 1 66.897 1
91.897 1 74.325 1 94.016
ENSMUSG00000068182 Olfr203 protein_coding 1 79.068 1
97.155 1 83.811 1 94.654
ENSMUSG00000068184 Ndufaf2 protein_coding 0.853 0.987 1.089
75.612 1.079 11.348 1.027 73.939
ENSMUSG00000068196 Col8a1 protein_coding 0.674 7.574 0.792

85.116 1.876 10.819 0.808 83.724
ENSMUSG00000068200 Gm5931 pseudogene 1 83.658 1
96.216 1 85.935 1 92.181
ENSMUSG00000068205 Macrod2 protein_coding 1.048 60.435
0.973 82.76 0.977 28.669 0.992 95.273
ENSMUSG00000068206 Pick1 protein_coding 0.877 3.448 0.946 84.858
1.202 5.864 1.029 91.105
ENSMUSG00000068218 Ssxb9 protein_coding 1 73.585 1
95.301 1 80.163 1 89.052
ENSMUSG00000068219 Ssxb10 protein_coding 1 83.823 1
95.211 1 87.898 1 93.316
ENSMUSG00000068220 Lgals1 protein_coding 0.775 0.987 1.259
60.309 1.027 51.851 1.224 38.624
ENSMUSG00000068221 Pdxp protein_coding 0.984 83.09 0.959 96.268
NA NA 0.998 92.018
ENSMUSG00000068226 Gm6723 pseudogene 0.857 62.354 0.706
55.265 1.653 55.512 1.597 92.748
ENSMUSG00000068227 Il2rb protein_coding 0.917 81.823 1.312
60.309 0.831 36.499 0.96 92.181
ENSMUSG00000068228 NA NA NA NA 1 96.984 1
80.163 1 94.174
ENSMUSG00000068231 Vmn1r43 protein_coding 1 82.092 1
95.941 1 86.602 1 95.676
ENSMUSG00000068232 Vmn1r42 protein_coding 1 68.353 1
95.211 1 76.768 1 93.457
ENSMUSG00000068234 Vmn1r44 protein_coding 1 80.51 1
97.129 1 80.634 1 92.018
ENSMUSG00000068235 Vmn1r-ps25 pseudogene 1 75.399 NA NA
1 77.294 1 96.287
ENSMUSG00000068243 Gm7079 pseudogene 1.279 56.311 NA NA
0.517 42.895 1.665 92.018
ENSMUSG00000068245 Phf11d protein_coding 1.154 1.846 NA NA
0.829 16.409 1.297 56.339
ENSMUSG00000068246 Apol9b protein_coding 1.632 7.332 1.111
96.847 0.552 46.32 1.312 44.882
ENSMUSG00000068250 Amn1 protein_coding 0.838 7.207 1.035 93.574
1.149 10.819 0.988 93.264
ENSMUSG00000068259 Olfr461 protein_coding 1 85.203 1
96.138 1 84.896 1 96.248
ENSMUSG00000068263 Efcc1 protein_coding 0.993 83.169 1.04
97.263 0.969 87.744 0.75 84.19
ENSMUSG00000068264 Ap5s1 protein_coding 0.975 85.499 1.032
90.389 0.991 80.749 1.012 94.001
ENSMUSG00000068267 Cenpb protein_coding 0.901 18.602 1.019
96.763 1.087 62.237 1.051 65.845
ENSMUSG00000068270 Shroom4 protein_coding 1.055 35.748
1.098 86.277 0.863 2.563 0.898 83.724
ENSMUSG00000068284 Gm608 protein_coding 0.99 75.298 0.938
93.654 1.074 46.206 0.944 83.952
ENSMUSG00000068290 Ddrgk1 protein_coding 0.971 71.763
0.974 90.378 1.059 57.907 0.93 84.29
ENSMUSG00000068299 1700019G17Rik protein_coding 1.044 54.717
0.89 66.651 1.078 51.977 0.701 83.724
ENSMUSG00000068302 Noto protein_coding 1 76.135 1

96.68 NA NA 1 96.183
ENSMUSG00000068303 Spr-ps1 protein_coding 0.614 31.614
1.205 87.037 1.347 79.325 1.198 94.904
ENSMUSG00000068314 Gm6899 protein_coding 0.947 37.82 0.816
87.452 1.294 41.6 0.82 93.132
ENSMUSG00000068323 Slc4a5 protein_coding 1.581 10.391
0.774 64.624 0.818 87.37 0.663 90.611
ENSMUSG00000068327 Tlx2 protein_coding 1 83.968 NA NA
1 87.431 1 94.19
ENSMUSG00000068328 Aup1 protein_coding 0.966 15.486 1.022
83.104 1.014 64.024 0.985 93.016
ENSMUSG00000068329 Htra2 protein_coding 0.952 22.431 0.981
93.899 1.073 49.898 1.019 93.261
ENSMUSG00000068335 Dok1 protein_coding 0.966 73.487 0.879
79.475 1.173 35.13 1.123 46.412
ENSMUSG00000068341 Reg3d protein_coding 1 85.832 1
96.138 1 79.897 1 96.086
ENSMUSG00000068348 Gm10238 protein_coding 1 86.145 1
96.847 1 83.158 1 95.273
ENSMUSG00000068373 D430041D05Rik protein_coding 1.063 71.896
0.938 96.847 0.998 38.799 0.964 92.181
ENSMUSG00000068391 Chrac1 protein_coding 1.095 12.396
0.964 83.58 0.945 35.264 0.954 89.525
ENSMUSG00000068394 Cep152 protein_coding 1.003 77.182
1.034 96.843 0.961 71.388 1.153 46.846
ENSMUSG00000068396 Rpl34-ps1 pseudogene 1.006 81.01 0.718 79.261
1.385 43.307 0.86 94.153
ENSMUSG00000068397 Gm10240 pseudogene 1 74.516 1
96.216 1 86.985 1 93.016
ENSMUSG00000068399 Gm7247 protein_coding 1 82.891 1
95.504 1 73.874 1 96.071
ENSMUSG00000068407 Rnase12 protein_coding 1 82.705 1
92.793 1 48.133 1 90.397
ENSMUSG00000068417 Pnp2 protein_coding 0.907 73.292 1.224
88.092 0.9 64.155 1.713 40.127
ENSMUSG00000068426 Vmn1r19 protein_coding 1 86.006 1
96.268 1 86.084 1 91.372
ENSMUSG00000068431 Olfr742 protein_coding 1 83.968 1
96.897 1 84.724 1 96.071
ENSMUSG00000068437 Olfr725 protein_coding 1 81.657 1
95.211 1 86.666 1 96.071
ENSMUSG00000068452 Duox2 protein_coding 1.01 71.915 0.826
86.604 1.2 24.07 0.914 94.654
ENSMUSG00000068457 Uty protein_coding 1 86.658 1
97.129 1 79.776 1 94.904
ENSMUSG00000068463 B630019A10Rik processed_transcript 1.596
44.061 0.857 79.261 0.732 88.601 0.577 83.724
ENSMUSG00000068466 Gm5518 pseudogene 1 86.172 NA NA
1 87.786 1 96.287
ENSMUSG00000068479 Mfap1a protein_coding 1.089 5.932 0.973
93.654 0.946 6.858 0.964 84.29
ENSMUSG00000068480 Gm7551 pseudogene 1 66.76 1 96.001
1 87.971 1 93.045
ENSMUSG00000068487 Gm4943 pseudogene 1 80.688 1

89.996 1 86.144 1 94.82
ENSMUSG00000068506 Gm5800 protein_coding 1 79.709 1
95.708 1 74.179 1 91.372
ENSMUSG00000068522 Aard protein_coding 0.519 5.946 0.986 95.504
1.956 16.808 1.132 83.276
ENSMUSG00000068523 Gng5 protein_coding 1.069 19.893 1.111
60.309 0.845 0.72 0.971 94.654
ENSMUSG00000068535 Olfr169 protein_coding 1 80.51 1
95.708 1 84.071 1 96.071
ENSMUSG00000068536 Doxl2 protein_coding 1 84.534 1
94.597 1 84.071 1 92.851
ENSMUSG00000068537 NA NA NA NA 1 96.013 1
88.274 1 95.74
ENSMUSG00000068547 Clca6 protein_coding 1 84.397 1
88.91 1 71.388 1 94.215
ENSMUSG00000068551 Zfp467 protein_coding 0.994 86.57 1.18
64.304 0.85 1.061 0.992 95.72
ENSMUSG00000068566 Myadm protein_coding 0.956 24.577 1.079
40.99 0.972 12.571 1.028 93.156
ENSMUSG00000068574 Olfr458 protein_coding 1 85.655 NA
NA 1 75.009 1 94.904
ENSMUSG00000068579 Rpl7a-ps3 protein_coding 1 71.217 1
96.216 1 87.336 1 93.156
ENSMUSG00000068580 Zfyve19 protein_coding 0.953 35.646
1.022 96.897 1.028 65.567 0.981 96.192
ENSMUSG00000068587 Mgam protein_coding 1.167 71.763 1.045
95.504 0.822 79.625 0.411 92.12
ENSMUSG00000068601 Gm10244 protein_coding 1.032 60.435
0.938 81.731 1.034 84.308 0.913 87.705
ENSMUSG00000068606 Gm4841 protein_coding 1.158 66 1.688
60.309 0.513 3.428 1.14 85.786
ENSMUSG00000068614 Actc1 protein_coding 1.403 36.195 1.445
78.629 0.492 1.58 1.221 93.734
ENSMUSG00000068615 NA NA NA NA 1 96.897 1
87.274 1 92.748
ENSMUSG00000068617 Efcab1 protein_coding 1.014 58.198
1.053 97.155 0.935 80.163 1.065 91.492
ENSMUSG00000068629 Gm6155 pseudogene 1 85.655 1
97.288 1 87.744 1 96.287
ENSMUSG00000068631 Gm7676 protein_coding 1 84.452 1
94.597 1 67.339 1 94.384
ENSMUSG00000068645 Olfr1280 protein_coding 1 86.172 1
97.263 1 86.144 1 95.273
ENSMUSG00000068646 Olfr1279 protein_coding 1 79.165 1
97.288 1 86.346 1 96.183
ENSMUSG00000068647 Olfr1278 protein_coding 1 85.637 1
96.407 1 87.103 1 92.762
ENSMUSG00000068663 Clec16a protein_coding 1.018 73.734
1.081 82.904 0.906 25.345 0.96 83.724
ENSMUSG00000068680 Gm10248 antisense 1 83.169 1
94.597 1 62.995 1 95.273
ENSMUSG00000068686 Cd59b protein_coding 0.857 28.242 NA NA
1.091 48.451 1.042 94.99
ENSMUSG00000068689 Rps19-ps9 pseudogene 1 81.178 1

96.68 1 84.229 1 96.358
ENSMUSG00000068696 Gpr88 protein_coding 0.582 83.333 2.53
80.806 0.677 55.214 1.992 79.442
ENSMUSG00000068697 Myoz1 protein_coding 8.46 6.281 0.984 89.434
0.12 0.72 0.919 96.184
ENSMUSG00000068699 Flnc protein_coding 1.234 11.638 1.32
82.76 0.615 7.155 1.38 35.053
ENSMUSG00000068732 Tmem167b protein_coding 1.135 1.412 0.965
82.073 0.912 1.764 1.032 80.497
ENSMUSG00000068735 Trp53i11 protein_coding 0.865 56.768
1.147 87.609 1.003 86.666 0.935 86.699
ENSMUSG00000068739 Sars protein_coding 0.916 0.987 0.952 79.807
1.15 5.864 1.113 0
ENSMUSG00000068740 Celsr2 protein_coding 0.857 0.987 1
97.263 1.162 20.051 0.855 83.724
ENSMUSG00000068742 Cry2 protein_coding 0.788 0.987 1.07 93.899
1.187 16.666 1.006 83.067
ENSMUSG00000068744 Psrc1 protein_coding 1.157 51.189 0.949
90.053 0.913 72.464 1.53 35.053
ENSMUSG00000068747 Sort1 protein_coding 0.995 53.228 1.013
97.155 0.993 56.625 0.934 83.724
ENSMUSG00000068748 Ptprz1 protein_coding 1.059 51.826
0.987 87.609 0.96 21.852 0.981 92.181
ENSMUSG00000068749 Psma5 protein_coding 1.035 81.657 0.902
84.017 1.072 69.196 1.092 64.513
ENSMUSG00000068758 Il3ra protein_coding 1.124 29.811 0.858
68.818 1.037 86.258 1.046 92.263
ENSMUSG00000068762 Gstm6 protein_coding 0.718 14.221 1.051
97.263 1.323 24.07 0.953 93.584
ENSMUSG00000068776 4930469B13Rik pseudogene 1 82.092 NA
NA 1 83.275 1 96.248
ENSMUSG00000068794 Col28a1 protein_coding 0.978 85.129
0.816 67.739 1.251 12.703 1.187 38.624
ENSMUSG00000068798 Rap1a protein_coding 1.184 1.828 0.885 75.4
0.954 4.935 1.052 68.696
ENSMUSG00000068806 Olfr1259 protein_coding 1 80.03 1
96.843 1 71.004 1 88.387
ENSMUSG00000068808 Olfr1189 protein_coding 1 76.686 1
95.211 1 85.921 NA NA
ENSMUSG00000068809 NA NA NA NA 1 97.263 1
88.349 1 93.132
ENSMUSG00000068810 Olfr1187-ps1 protein_coding 1 76.396
1 96.216 1 86.144 1 96.183
ENSMUSG00000068814 Olfr1145 protein_coding 1 85.655 1
93.654 1 87.786 1 94.904
ENSMUSG00000068815 Olfr1143 protein_coding 1 84.294 1
96.984 1 83.158 1 96.183
ENSMUSG00000068816 Olfr152 protein_coding 1 84.865 1
94.597 1 88.693 1 93.584
ENSMUSG00000068817 Olfr1140 protein_coding 1 86.333 1
91.776 1 88.203 1 95.832
ENSMUSG00000068818 Olfr1132 protein_coding 1 82.982 1
93.817 1 86.346 1 93.132
ENSMUSG00000068819 Olfr1131 protein_coding 1 78.299 1

97.155 1 78.642 1 96.287
ENSMUSG00000068823 Csde1 protein_coding 1.024 43.019 1.011
95.708 0.967 25.688 1.016 89.052
ENSMUSG00000068824 Olfr1083-ps pseudogene 1 86.333 1
96.847 1 86.144 1 92.748
ENSMUSG00000068825 Gm13721 pseudogene 1 85.203 1
96.013 NA NA 1 93.117
ENSMUSG00000068854 Hist2h2be protein_coding 0.933 31.614
1.095 79.29 0.979 78.369 0.848 86.254
ENSMUSG00000068855 Hist2h2ac protein_coding 1.931 21.703
1.765 96.268 0.293 16.949 0.607 91.65
ENSMUSG00000068856 Sf3b4 protein_coding 0.994 86.145 1.111
72.546 0.908 21.117 1.01 88.197
ENSMUSG00000068859 Sp9 protein_coding 0.856 21.658 0.933
97.155 1.253 32.524 0.853 84.29
ENSMUSG00000068860 Gm128 protein_coding 0.687 33.906 1.686
71.617 0.864 71.004 0.95 88.053
ENSMUSG00000068874 Selenbp1 protein_coding 0.978 77.497
1.055 95.708 0.965 88.601 0.971 87.331
ENSMUSG00000068876 Cgn protein_coding 0.891 22.481 0.975
95.708 1.149 30.107 0.992 95.832
ENSMUSG00000068877 Selenbp2 protein_coding 1.069 64.956
0.919 92.545 1.012 84.071 1.025 94.752
ENSMUSG00000068879 Gm5773 protein_coding 1 84.168 NA
NA 1 82.778 1 94.82
ENSMUSG00000068882 Ssb protein_coding 1.046 76.827 0.932
83.392 1.028 83.066 1.129 31.133
ENSMUSG00000068885 Lce3f protein_coding 1 85.637 1
95.431 1 87.431 1 94.904
ENSMUSG00000068887 Lce1j protein_coding 1 84.661 NA NA
1 84.896 1 96.183
ENSMUSG00000068888 Lce1i protein_coding 1 81.465 1
88.881 1 84.308 1 95.74
ENSMUSG00000068889 Lce1e protein_coding 1 81.01 1 97.263
1 84.896 1 94.904
ENSMUSG00000068890 Lce1a2 protein_coding 1 79.433 1
94.102 1 87.431 1 94.384
ENSMUSG00000068917 Clk2 protein_coding 0.946 38.195 1.015
91.584 1.044 66.647 0.955 83.724
ENSMUSG00000068921 Dap3 protein_coding 0.945 11.707 1.029
79.411 1.027 69.315 0.999 80.77
ENSMUSG00000068922 Msto1 protein_coding 1.046 56.907 0.95
82.947 1.005 64.305 0.972 91.372
ENSMUSG00000068923 Syt11 protein_coding 1.104 20.032 0.952
67.839 0.946 85.134 0.946 92.748
ENSMUSG00000068940 Tcl1b3 protein_coding 1 86.843 NA
NA 1 78.369 1 95.877
ENSMUSG00000068947 Olfr366 protein_coding 1 84.819 1
94.89 1 79.776 1 93.584
ENSMUSG00000068948 Olfr363-ps pseudogene 1 82.982 1
96.68 1 87.898 1 92.762
ENSMUSG00000068950 Olfr338 protein_coding 1 86.145 1
93.654 NA NA 1 95.777
ENSMUSG00000068959 Zfp619 protein_coding 1.049 84.865

0.977 95.329 0.978 76.225 0.938 86.991
ENSMUSG00000068962 Zfp114 protein_coding 0.745 27 NA NA
1.2 45.94 1.035 91.525
ENSMUSG00000068964 Gm6361 pseudogene 1 82.891 1
97.155 1 86.144 1 96.184
ENSMUSG00000068966 Zbtb34 protein_coding 1.048 50.377
0.912 79.807 1.048 81.976 1.035 86.445
ENSMUSG00000068969 Rpl32-ps pseudogene 1 74.482 1
95.708 1 75.009 1 93.734
ENSMUSG00000068999 Vmn2r5 protein_coding 1 81.823 NA
NA 1 84.961 1 86.445
ENSMUSG00000069011 Gm10254 pseudogene 1 83.169 1
94.102 1 83.538 1 92.12
ENSMUSG00000069014 Gm5641 protein_coding 1 82.982 1
95.431 1 87.274 1 91.105
ENSMUSG00000069020 Urm1 protein_coding 0.908 12.309 1.067
86.156 1.03 31.317 1.034 78.379
ENSMUSG00000069036 Sry protein_coding 1 82.891 NA NA
1 86.985 1 94.654
ENSMUSG00000069038 Gm4906 protein_coding 1 81.465 1
95.504 1 80.899 1 94.001
ENSMUSG00000069041 Slc25a31 protein_coding 0.27 22.481
2.268 72.642 1.636 68.129 2.548 81.061
ENSMUSG00000069044 Usp9y protein_coding 1 79.853 1
96.407 1 86.985 1 95.593
ENSMUSG00000069045 Ddx3y protein_coding 1 81.465 1
96.001 1 88.657 1 92.12
ENSMUSG00000069049 Eif2s3y protein_coding 1 78.909 1
92.062 1 86.666 1 92.851
ENSMUSG00000069053 Uba1y protein_coding 1 86.819 1
93.654 1 68.425 1 90.865
ENSMUSG00000069072 Slc7a14 protein_coding 1.924 7.397 0.581
44.268 0.895 85.58 0.672 94.608
ENSMUSG00000069074 Gm10258 protein_coding 1 83.169 1
92.355 NA NA 1 96.287
ENSMUSG00000069080 Lcn11 protein_coding 1 81.178 NA NA
1 80.015 1 91.985
ENSMUSG00000069083 Gm10259 protein_coding 1 77.407 1
95.708 1 88.729 1 95.273
ENSMUSG00000069085 Dytn protein_coding 1 74.147 1
93.654 1 85.792 1 96.287
ENSMUSG00000069089 Cdk7 protein_coding 0.964 52.726 0.966
89.509 1.074 38.099 0.986 85.493
ENSMUSG00000069094 Pde7a protein_coding 0.89 11.519 1.056
83.104 1.067 81.29 0.987 85.493
ENSMUSG00000069114 Zbtb10 protein_coding 0.974 36.945
0.983 97.263 1.042 34.807 0.926 83.724
ENSMUSG00000069117 Gm10260 protein_coding 1 71.763 1
95.708 1 74.686 1 96.287
ENSMUSG00000069118 1700008P02Rik protein_coding 1 81.465
1 96.843 1 87.744 1 93.156
ENSMUSG00000069125 Rps24-ps2 pseudogene 1 84.738 1
97.263 1 88.657 1 92.748
ENSMUSG00000069132 Nxph2 protein_coding 1 86.006 1

95.708 1 84.504 1 95.832
ENSMUSG00000069135 Fgfr1op protein_coding 0.951 49.944
1.042 96.407 1.011 68.129 0.939 95.273
ENSMUSG00000069136 A130006I12Rik pseudogene 1.006 78.744
0.933 93.046 1.069 81.032 0.787 89.892
ENSMUSG00000069170 Gpr98 protein_coding 1.083 83.823 1.244
97.155 0.74 87.519 0.803 92.561
ENSMUSG00000069171 Nr2f1 protein_coding 0.913 33.906 1.102
71.617 0.993 81.62 1.29 32.698
ENSMUSG00000069184 Zfp72 protein_coding 1.064 52.799 1.037
96.216 0.909 41.154 1.021 96.248
ENSMUSG00000069188 Gm13192 pseudogene 1.262 36.686 1.096
82.904 0.724 6.858 1.133 93.117
ENSMUSG00000069189 Zdhhc11 protein_coding 1 71.36 1
91.361 1 57.286 1 92.691
ENSMUSG00000069196 Gm10161 pseudogene 1 80.608 1
93.994 1 87.786 1 92.31
ENSMUSG00000069206 Zfp874a protein_coding 1.125 3.434 0.928
55.265 0.955 53.7 1.026 91.625
ENSMUSG00000069208 Zfp825 protein_coding 1.236 13.292
0.955 87.313 0.846 24.309 0.958 96.192
ENSMUSG00000069223 4930451E10Rik antisense 0.977 65.622
1.034 93.863 0.992 82.778 0.806 83.724
ENSMUSG00000069227 Gprin1 protein_coding 0.942 73.585
1.039 95.329 1.018 71.839 1.335 71.653
ENSMUSG00000069236 Gm7251 pseudogene 1 82.231 1
96.216 1 85.921 1 94.153
ENSMUSG00000069237 C78339 protein_coding 1.044 25.421 NA
NA 0.944 34.21 1.001 95.832
ENSMUSG00000069248 Serpinb6e protein_coding 1 83.418 1
95.735 1 68.266 1 90.674
ENSMUSG00000069255 Dusp22 protein_coding 1.012 86.172
0.954 92.914 1.035 87.103 1.019 58.721
ENSMUSG00000069258 Prl2b1 protein_coding 1 80.751 1
95.301 1 84.724 1 94.904
ENSMUSG00000069259 Prl6a1 protein_coding 1 86.172 1
96.268 NA NA 1 96.248
ENSMUSG00000069265 Hist1h3a protein_coding 1.614 83.658
0.521 95.504 1.192 86.666 0.95 95.877
ENSMUSG00000069266 Hist1h4b protein_coding 1.017 77.291
1.438 97.097 0.684 66.425 0.628 90.611
ENSMUSG00000069270 Hist1h2ac protein_coding 0.929 61.334
1.153 88.092 0.934 75.637 0.715 87.705
ENSMUSG00000069272 Hist1h2ae protein_coding 1.504 35.024
1.594 81.127 0.418 6.858 0.775 85.493
ENSMUSG00000069273 Hist1h3e protein_coding 1.867 81.52 0.486
91.897 1.105 83.925 0.57 84.29
ENSMUSG00000069276 Gm10264 protein_coding 1 85.203 1
96.216 1 87.431 1 95.273
ENSMUSG00000069280 Vmn1r223 protein_coding 1 82.231 1
96.013 1 78.369 1 93.685
ENSMUSG00000069289 Vmn1r203 protein_coding 1 83.263 1
96.138 1 87.744 1 96.287
ENSMUSG00000069291 Vmn1r200 protein_coding 1 68.66 1

96.308	NA	NA	1	95.273
ENSMUSG00000069292	Vmn1r199	protein_coding	1	85.59 1
96.843	1	86.602	1	95.74
ENSMUSG00000069294	Vmn1r197	protein_coding	1	85.77 1
94.848	1	88.693	1	96.183
ENSMUSG00000069295	Vmn1r196	protein_coding	1	79.068	NA
NA	1	88.601	1	94.608
ENSMUSG00000069296	Vmn1r195	protein_coding	1	84.661	NA
NA	1	86.666	1	94.99
ENSMUSG00000069297	Vmn1r194	protein_coding	1	83.968	1
95.504	1	88.161	1	96.358
ENSMUSG00000069299	NA	NA	NA	NA	1	93.908	1
88.461	1	92.31
ENSMUSG00000069303	Hist1h2br	protein_coding	1	85.129	1
95.211	1	81.857	1	94.904
ENSMUSG00000069305	Hist1h4n	protein_coding	1	74.689	1
91.584	1	81.388	1	96.287
ENSMUSG00000069306	Hist1h4m	protein_coding	1.583 58.896
1.125 94.96 0.563 65.902	0.327 83.952
ENSMUSG00000069307	NA	NA	NA	NA	1	96.001	1
88.161	1	96.086
ENSMUSG00000069309	Hist1h2an	protein_coding	1	53.228	1
96.68 1	73.598	1	96.358
ENSMUSG00000069312	Hist1h3h	protein_coding	1	83.169	1
83.58 1	49.898	1	94.904
ENSMUSG00000069315	Gm7731	pseudogene 1	83.09 1	94.96 1
79.426	1	96.086
ENSMUSG00000069324	Gm5096	protein_coding	1	79.93 1
96.843	1	79.625	1	96.358
ENSMUSG00000069355	Gm5152	protein_coding	1	84.865	1
97.155	1	84.724	1	95.273
ENSMUSG00000069372	Ctxn3 protein_coding	1.607 75.399	1.043
97.097	0.596 45.799	0.907 83.067
ENSMUSG00000069378	Prdm6 protein_coding	1.236 29.811	0.836
95.211	0.966 42.895	0.839 86.991
ENSMUSG00000069379	Gm4950	protein_coding	1	84.865	1
93.908	1	84.229	1	95.273
ENSMUSG00000069385	Gm10267	protein_coding	1	84.819	1
95.504	1	86.144	1	94.99
ENSMUSG00000069390	Olfr707	protein_coding	1	76.827	NA
NA	1	81.857	1	93.156
ENSMUSG00000069421	Olfr810	protein_coding	1	86.843	1
96.013	1	84.308	1	94.608
ENSMUSG00000069430	Olfr9 protein_coding	1	85.355	1
96.984	1	82.161	1	95.273
ENSMUSG00000069456	Rdh16 protein_coding	0.649 82.543	0.476
71.331	3.235 28.979	1.755 89.052
ENSMUSG00000069475	Gm6020	protein_coding	1.235 81.125	NA
NA	1.36	87.686	1.651 90.674
ENSMUSG00000069476	Zfp616	protein_coding	1	82.479	1
95.301	1	75.527	1	96.183
ENSMUSG00000069495	Epc2	protein_coding	1.112 1.99	0.985 91.532
0.909 6.858 0.994 93.264
ENSMUSG00000069515	Lyz1	protein_coding	1.754 1.81	0.78	38.699

0.732 64.155 0.857 85.493
ENSMUSG00000069516 Lyz2 protein_coding 1.524 8.996 0.817 61.883
0.801 55.9 0.833 88.387
ENSMUSG00000069520 Tmem19 protein_coding 1.043 45.746
0.979 92.355 0.983 71.999 0.985 96.183
ENSMUSG00000069539 Scyl2 protein_coding 1.067 12.985 0.952
70.971 0.981 63.504 0.978 88.505
ENSMUSG00000069540 Gm4925 pseudogene 0.853 60.707 0.685
74.483 1.701 22.517 1.133 83.067
ENSMUSG00000069554 I830134H01Rik lincRNA 0.706 57.818
1.394 83.104 1.011 83.719 1.273 92.018
ENSMUSG00000069565 Dazap1 protein_coding 0.87 13.199
1.023 96.984 1.121 13.259 1.024 83.067
ENSMUSG00000069581 Tspear protein_coding 0.849 81.925
0.752 94.262 1.559 67.562 1.034 94.215
ENSMUSG00000069582 Krtap10-4 pseudogene 1 86.474 1
94.48 1 85.464 1 92.181
ENSMUSG00000069583 Krtap12-1 protein_coding 1 80.688 1
95.211 1 87.37 1 95.273
ENSMUSG00000069584 Gm10272 protein_coding 1 80.608 1
96.216 1 82.161 1 93.964
ENSMUSG00000069601 Ank3 protein_coding 0.908 8.002 1.065 85.756
1.036 37.764 0.998 92.31
ENSMUSG00000069607 Cd300lh protein_coding 2.933 1.712 0.45
73.166 0.755 28.901 0.616 85.493
ENSMUSG00000069620 Gm5250 protein_coding 1 84.452 1
96.652 1 86.112 1 95.273
ENSMUSG00000069622 Gm10273 protein_coding 1 85.203 1
96.843 1 88.657 1 91.525
ENSMUSG00000069631 Strada protein_coding 0.884 4.634 1.033
97.155 1.093 10.523 1.02 96.192
ENSMUSG00000069633 Pex11g protein_coding 0.842 0.987 1.093
71.98 1.086 6.916 0.969 83.724
ENSMUSG00000069662 Marcks protein_coding 1.136 15.602
1.071 85.756 0.823 6.858 1.01 90.95
ENSMUSG00000069668 Sult3a1 protein_coding 1 81.823 1
96.843 1 86.666 1 93.722
ENSMUSG00000069670 Nkain2 protein_coding 3.567 27.113
0.281 84.017 1 84.552 0.517 86.991
ENSMUSG00000069671 Nkain2 protein_coding 1.492 51.297
0.587 72.399 1.136 76.754 0.987 96.183
ENSMUSG00000069678 Pcgf1 protein_coding 1.047 37.414 0.913
67.513 1.049 78.369 1.007 90.611
ENSMUSG00000069703 Taar7e protein_coding 1 84.738 1
95.504 1 80.749 1 94.825
ENSMUSG00000069706 Taar5 protein_coding 1 83.968 1
96.138 1 83.607 1 94.904
ENSMUSG00000069708 Taar3 protein_coding 1 81.657 1
95.504 1 69.196 1 91.928
ENSMUSG00000069712 4930444G20Rik protein_coding 1 79.277
1 96.407 1 83.397 1 88.197
ENSMUSG00000069713 4933406P04Rik protein_coding 0.845 77.615
1.103 95.196 1.074 78.155 0.916 96.183
ENSMUSG00000069717 Gm11568 protein_coding 1 80.608 1

93.994 1 85.464 1 96.358
ENSMUSG00000069718 Gm11563 protein_coding 1 84.452 1
94.48 1 81.976 1 93.964
ENSMUSG00000069720 4930572O03Rik protein_coding 0.577 50.229
0.988 95.088 1.761 48.935 1.1 93.316
ENSMUSG00000069721 Krtap3-2 protein_coding 1 76.396 1
95.708 1 82.31 1 93.734
ENSMUSG00000069722 Krtap3-3 protein_coding 1 84.738 1
95.196 1 83.275 1 96.086
ENSMUSG00000069727 Gm5595 protein_coding 1.003 62.574
1.055 82.904 0.946 20.258 1.012 88.505
ENSMUSG00000069729 Arid1b protein_coding 1.06 68.485
0.996 96.268 0.946 41.662 0.933 89.294
ENSMUSG00000069743 Zfp820 protein_coding 1.13 21.146
0.971 93.094 0.909 41.662 1.029 95.273
ENSMUSG00000069744 Psmb3 protein_coding 0.887 1.175 1.04 82.904
1.085 22.131 1.127 60.466
ENSMUSG00000069755 Zfp125 protein_coding 1.125 20.799 0.95
87.322 0.938 67.029 1.093 81.901
ENSMUSG00000069763 Tmem100 protein_coding 0.925 80.251
1.273 83.104 0.85 30.827 1.238 65.558
ENSMUSG00000069769 Msi2 protein_coding 1.032 77.916 0.935
79.807 1.035 47.31 0.947 83.952
ENSMUSG00000069785 Gm11444 protein_coding 1 78.299 1
95.735 1 88.601 1 93.156
ENSMUSG00000069792 Wfdc17 protein_coding 1.639 0 0.832
85.95 0.733 6.751 1.31 43.377
ENSMUSG00000069793 Slfn9 protein_coding 1.331 13.557 NA NA
0.705 44.865 1.527 37.634
ENSMUSG00000069796 Gm11426 lincRNA 1 79.93 1 95.708
1 84.308 1 94.904
ENSMUSG00000069804 Gm10277 protein_coding 0.662 16.325
0.896 95.504 1.68 38.099 1.124 96.287
ENSMUSG00000069805 Fbp1 protein_coding 0.874 78.909 0.672
49.859 1.705 27.431 1.079 95.877
ENSMUSG00000069806 Cacng7 protein_coding 0.964 42.034
1.127 40.99 0.917 32.082 0.947 86.991
ENSMUSG00000069808 Fam57a protein_coding 1.113 27.113
1.063 93.654 0.84 56.822 1.01 92.851
ENSMUSG00000069814 E130309D14Rik protein_coding 0.807 58.077
1.26 96.847 0.983 85.935 0.864 85.493
ENSMUSG00000069816 Olfr23 protein_coding 1 85.77 1
96.216 1 83.925 1 96.358
ENSMUSG00000069818 Olfr390 protein_coding 1 82.231 1
96.268 1 79.897 1 95.273
ENSMUSG00000069823 Olfr1 protein_coding 1 83.169 1
96.268 1 84.83 1 95.593
ENSMUSG00000069824 Olfr377-ps1 pseudogene 1 84.661 1
97.288 1 84.308 1 96.287
ENSMUSG00000069825 Spata22 protein_coding 1.092 72 0.975
87.609 0.942 21.852 1.093 93.132
ENSMUSG00000069830 Nlrp1a protein_coding 1.928 7.908 0.924
97.263 0.563 11.902 1.054 83.276
ENSMUSG00000069833 Ahnak protein_coding 1.063 29.596 0.97

89.509	0.97	55.9	1.063	96.139
ENSMUSG00000069835	Sat2	protein_coding	1.003	84.397	0.901
88.691	1.105	59.612	1.069	95.273
ENSMUSG00000069844	Sco1	protein_coding	0.95	48.646	1.055
87.282	NA	NA	0.957	87.735
ENSMUSG00000069855	Slc47a2	protein_coding	1.116	63.385
1.154	96.407	0.775	63.075	1.332	84.29
ENSMUSG00000069867	Pabpn1l	protein_coding	0.798	43.435
0.976	96.216	1.28	40.875	1.443	74.607
ENSMUSG00000069873	4930438A08Rik	protein_coding	1.961	63.804
1.183	93.899	0.432	36.786	0.78	90.611
ENSMUSG00000069874	Irgm2	protein_coding	1.107	61.49	1.203	71.617
0.753	8.472	1.017	93.156
ENSMUSG00000069892	9930111J21Rik2	protein_coding	1.179	18.388
0.97	91.584	0.874	7.339	1.201	74.193
ENSMUSG00000069893	9930111J21Rik1	protein_coding	1.109	46.013
1.02	95.211	0.885	60.233	0.866	85.493
ENSMUSG00000069895	Atxn1l	protein_coding	1.077	3.176	0.961
60.806	0.963	40.19	0.972	89.525
ENSMUSG00000069899	Gm12166	protein_coding	1	76.042	NA
NA	1	80.089	1	95.92
ENSMUSG00000069910	Spdl1	protein_coding	1.274	59.527	1.102
95.504	0.713	69.445	1.148	82.66
ENSMUSG00000069913	NA	NA	NA	NA	1	95.504	1
80.381	1	92.381
ENSMUSG00000069917	Hba-a2	protein_coding	1.01	72.27	0.91
90.053	1.083	77.888	0.911	88.552
ENSMUSG00000069919	Hba-a1	protein_coding	1.018	81.178
1.027	96.216	0.957	87.898	0.747	86.445
ENSMUSG00000069920	B3gnt9	protein_coding	1.456	0.664	0.895
95.941	0.764	0.72	1.149	69.918
ENSMUSG00000069922	Ces3a	protein_coding	1	83.263	1
93.899	1	87.274	1	95.777
ENSMUSG00000069925	4932416K20Rik	pseudogene	1	78.744	1
95.329	1	88.461	1	96.358
ENSMUSG00000069939	Gm12070	pseudogene	0.553	22.237	1.357
88.136	1.336	38.152	1.209	87.7
ENSMUSG00000069962	Gm5356	protein_coding	1	84.819	1
95.329	1	76.754	1	94.608
ENSMUSG00000069971	4933402J07Rik	protein_coding	1	81.925
1	95.211	1	71.839	1	84.246
ENSMUSG00000069986	Gm8054	pseudogene	1	83.169	1
95.504	1	85.921	1	96.192
ENSMUSG00000069996	Gm7125	pseudogene	1	81.01	NA	NA	1
87.336	1	94.752
ENSMUSG00000069998	Olfr372	protein_coding	1	86.006	1
95.708	1	87.744	1	95.74
ENSMUSG00000070000	Fcho1	protein_coding	1.191	23.019	0.863
80.052	0.974	86.346	0.835	83.724
ENSMUSG00000070002	Ell	protein_coding	1.001	76.396	0.916
71.41	1.091	29.819	1.01	96.287
ENSMUSG00000070003	Ssbp4	protein_coding	1.003	56.477	1.039
85.985	0.959	79.524	0.987	87.624
ENSMUSG00000070023	Gm10283	protein_coding	1	82.231	NA

NA 1 86.552 1 90.805
ENSMUSG00000070025 Gm10284 pseudogene 1 78.909 1
95.735 1 88.601 1 95.273
ENSMUSG00000070031 Sp140 protein_coding 1.278 0.577 0.973 61.883
0.8 23.86 1.083 73.401
ENSMUSG00000070034 Sp110 protein_coding 1.187 0 1.152 95.196
0.728 21.331 1.226 32.698
ENSMUSG00000070044 Fam149a protein_coding 0.948 54.87 1.096
71.617 0.959 33.402 1.055 92.691
ENSMUSG00000070047 Fat1 protein_coding 0.978 58.56 1.038 94.367
0.983 84.961 0.957 85.493
ENSMUSG00000070056 Mfhas1 protein_coding 1.244 0.577 1.056
90.092 0.759 1.871 0.982 96.358
ENSMUSG00000070063 Snora33 snoRNA 0.74 28.584 1.228
91.584 1.101 79.426 1.101 83.581
ENSMUSG00000070065 Mir451 miRNA 1 84.294 1 90.378
1 58.414 1 94.82
ENSMUSG00000070071 Mir465 miRNA 1 84.168 NA NA 1
87.173 1 96.086
ENSMUSG00000070072 Mir433 miRNA 0.358 51.089 1.654 96.216
1.691 82.436 1.319 95.559
ENSMUSG00000070073 Mir470 miRNA 1 84.324 1 96.763
1 86.258 1 96.287
ENSMUSG00000070074 Mir484 miRNA 1 77.916 1 94.848
1 78.864 1 94.904
ENSMUSG00000070075 Mir490 miRNA 1 78.428 1 97.263
1 84.626 1 96.287
ENSMUSG00000070076 Mir127 miRNA 1 51.56 1 95.708 1
57.637 1 96.287
ENSMUSG00000070077 Mir491 miRNA 1 33.415 1 96.68 1
56.931 1 70.539
ENSMUSG00000070078 Mir463 miRNA 1 85.728 1 93.863
1 82.74 1 93.316
ENSMUSG00000070081 Mir505 miRNA 1 85.946 1 96.216
1 86.144 1 91.525
ENSMUSG00000070099 Mir466 miRNA 1 86.658 1 96.652
1 77.037 1 95.74
ENSMUSG00000070100 Mir362 miRNA 1 82.543 1 96.68 1
81.525 1 95.593
ENSMUSG00000070102 Mir455 miRNA 1 72.27 NA NA 1
84.308 1 92.31
ENSMUSG00000070103 Mir488 miRNA 1 78.538 1 90.116
1 84.896 1 92.31
ENSMUSG00000070104 Mir471 miRNA 1 81.76 1 97.097 1
86.602 1 95.74
ENSMUSG00000070105 Mir495 miRNA 1 83.169 1 87.553
1 87.744 1 94.82
ENSMUSG00000070106 Mir363 miRNA 1 68.66 1 91.361 1
76.923 1 95.273
ENSMUSG00000070107 Mir489 miRNA 1 72.546 1 96.897
1 88.41 1 92.466
ENSMUSG00000070108 Mir500 miRNA 1 64.014 1 96.847
NA NA 1 94.904
ENSMUSG00000070109 Mir497 miRNA 1 81.01 1 94.767 1

85.675	1	95.74
ENSMUSG00000070110	Mir504	miRNA	1	82.479	1	97.155
1	80.634	1	92.018
ENSMUSG00000070126	Mir199a-2	miRNA	1	82.543	1	93.654
1	82.903	1	95.35
ENSMUSG00000070128	Mir485	miRNA	1	84.865	1	96.268
1	80.381	1	96.358
ENSMUSG00000070129	Mir136	miRNA	1	82.231	1	93.863
1	88.161	1	94.174
ENSMUSG00000070130	Mir328	miRNA	0.718	33.745	1.23	90.092
1.128	67.562	1.175	80.573
ENSMUSG00000070133	Mir434	miRNA	1	85.499	1	96.843
NA	NA	1	94.016
ENSMUSG00000070136	Mir496	miRNA	1	86.333	1	95.211
1	64.024	1	88.797
ENSMUSG00000070139	Mir532	miRNA	1	85.77	1	95.088	1
71.232	1	90.865
ENSMUSG00000070140	Mir483	miRNA	1	85.129	NA	NA	1
86.666	1	94.904
ENSMUSG00000070141	Mir494	miRNA	1	76.968	1	90.378
1	78.498	1	93.685
ENSMUSG00000070144	NA	NA	NA	NA	1	95.708	1
85.757	1	96.248
ENSMUSG00000070150	n-R5s152	rRNA	1	83.968	1	96.68	1
87.658	1	95.273
ENSMUSG00000070166	Gm25821	snoRNA	0.442	18.487	1.333
95.809	1.699	50.375	1.641	73.939
ENSMUSG00000070167	Gm25822	snoRNA	0.878	75.115	1.071
96.68	1.067	84.724	0.805	90.611
ENSMUSG00000070170	Gm24163	snoRNA	1	81.465	1
97.155	1	86.985	1	93.734
ENSMUSG00000070173	Gm24164	snRNA	1	85.857	NA	NA	1
81.857	1	94.99
ENSMUSG00000070175	Gm24162	snRNA	1	84.049	1	90.712
1	79.325	1	96.183
ENSMUSG00000070178	n-R5s71	rRNA	1	83.823	1	96.013
1	83.857	1	93.316
ENSMUSG00000070182	Gm24817	snRNA	1	81.178	1	95.708
1	80.163	1	94.19
ENSMUSG00000070192	n-R5s58	rRNA	1	64.014	1	94.597
1	87.744	1	94.904
ENSMUSG00000070203	Gm22444	snRNA	1	83.333	1	96.335
1	78.713	1	96.139
ENSMUSG00000070209	n-R5s27	rRNA	1	84.661	1	96.847
1	84.552	1	96.086
ENSMUSG00000070214	Gm23058	snRNA	1	71.06	1	94.848	1
76.498	1	92.691
ENSMUSG00000070219	n-R5s13	rRNA	1	81.24	1	95.431	1
85.921	1	93.156
ENSMUSG00000070227	Gm24704	snRNA	1	78.428	NA	NA	1
86.602	1	95.273
ENSMUSG00000070235	Gm26329	misc_RNA	1	86.006	1
97.263	1	88.349	1	96.192
ENSMUSG00000070236	Gm26327	snRNA	1	83.968	1	96.847

1 81.032 1 95.832
ENSMUSG00000070240 Gm23516 snRNA 1 83.09 1 96.843 NA
NA 1 95.72
ENSMUSG00000070249 Gm23520 snRNA 1 86.172 NA NA 1
84.724 1 95.832
ENSMUSG00000070258 Gm25207 snRNA 1 86.172 1 96.138
1 77.888 1 96.287
ENSMUSG00000070263 Gm22365 snRNA 1 84.865 1 94.887
1 72.464 1 95.676
ENSMUSG00000070271 Gm13268 pseudogene 1 77.497 NA NA
1 73.428 1 95.676
ENSMUSG00000070280 Slc22a14 protein_coding 1.083 85.728
0.827 87.553 1.113 69.002 1.019 94.904
ENSMUSG00000070282 3000002C10Rik pseudogene 0.973 54.176
0.898 76.978 1.145 27.533 1.059 92.018
ENSMUSG00000070283 Ndufaf3 protein_coding 0.952 30.352
1.054 85.756 0.999 83.719 0.96 84.29
ENSMUSG00000070284 Gmppb protein_coding 0.968 64.893 1.117
79.261 0.921 56.822 1.081 70.624
ENSMUSG00000070287 Slc35g2 protein_coding 1.367 5.848 0.86
92.545 0.848 11.314 1.019 92.181
ENSMUSG00000070291 Ddx43 protein_coding 1.409 80.688 0.659
88.691 1.077 81.976 0.842 94.825
ENSMUSG00000070304 Scn2b protein_coding 1.223 46.875 0.883
92.355 0.924 64.155 0.975 95.72
ENSMUSG00000070305 Mpzl3 protein_coding 0.913 23.13 0.876 74.483
1.244 7.964 0.942 96.192
ENSMUSG00000070306 Ccdc153 protein_coding 0.802 68.955
0.918 96.216 1.36 84.83 0.826 93.964
ENSMUSG00000070311 Olfr894 protein_coding 1 79.577 1
96.138 1 81.29 1 91.872
ENSMUSG00000070313 A630095E13Rik protein_coding 1 73.734
1 91.661 1 88.601 1 95.74
ENSMUSG00000070315 4930581F22Rik processed_transcript 0.945
61.043 0.936 49.859 1.13 10.819 0.967 84.29
ENSMUSG00000070319 Eif3g protein_coding 0.918 5.084 0.953 67.941
1.145 7.024 1.035 83.067
ENSMUSG00000070323 Mmp27 protein_coding 1.389 5.253 0.769 48.565
0.938 87.173 1.031 96.183
ENSMUSG00000070324 Fbxw22 protein_coding 1 82.352 1
94.848 1 87.686 1 93.132
ENSMUSG00000070327 Rnf213 protein_coding 1.083 42.555
0.942 92.137 0.98 62.058 1.145 72.369
ENSMUSG00000070330 Tmem235 protein_coding 1 69.24 1
93.899 1 68.82 1 96.287
ENSMUSG00000070331 Qrich2 protein_coding 0.627 22.92 1.333
80.678 1.191 85.935 0.947 84.19
ENSMUSG00000070332 4933422H20Rik protein_coding 0.936 72.743
0.735 87.919 1.451 76.498 0.374 85.493
ENSMUSG00000070334 Krtap31-1 protein_coding 1 85.283 1
95.088 1 84.896 1 95.92
ENSMUSG00000070335 Krtap9-1 protein_coding 1 85.946 1
96.216 NA NA 1 96.248
ENSMUSG00000070336 Fbxo47 protein_coding 1.178 42.134

1.128 96.948 0.751 35.022 0.907 90.611
ENSMUSG00000070337 Gpr179 protein_coding 0.76 41.617
0.904 78.471 1.451 22.444 0.861 96.086
ENSMUSG00000070342 Gm10287 lincRNA 1 86.145 1
91.83 1 65.8 1 95.72
ENSMUSG00000070345 Hsf5 protein_coding 2.32 33.854 0.774
95.504 0.556 57.091 0.484 84.29
ENSMUSG00000070348 Ccnd1 protein_coding 0.976 86.843 0.914
53.434 1.117 48.935 1.147 77.245
ENSMUSG00000070354 NA NA NA NA 1 95.211 1
86.727 1 96.183
ENSMUSG00000070360 Amy2a1 protein_coding 1 76.396 1
96.378 1 76.754 1 95.405
ENSMUSG00000070366 Ppapdc1a protein_coding 1 69.985 1
96.407 1 83.925 1 92.018
ENSMUSG00000070371 Prss36 protein_coding 0.953 64.014
1.133 79.411 0.928 67.962 0.947 87.705
ENSMUSG00000070372 Capza1 protein_coding 1.104 3.713 0.944
88.691 0.962 11.116 1.074 53.923
ENSMUSG00000070374 Olfr59 protein_coding 1 84.977 1
96.68 1 86.985 1 96.086
ENSMUSG00000070375 Olfr406 protein_coding 1 86.199 1
96.847 1 87.744 1 95.35
ENSMUSG00000070377 Olfr43 protein_coding 1 84.564 NA
NA 1 82.161 1 96.183
ENSMUSG00000070378 Olfr403 protein_coding 1 81.24 1
96.216 NA NA 1 95.593
ENSMUSG00000070379 Olfr402 protein_coding 1 83.169 1
94.848 1 79.325 1 94.153
ENSMUSG00000070380 Olfr401 protein_coding 1 86.464 1
97.288 NA NA 1 94.153
ENSMUSG00000070382 Olfr391-ps pseudogene 1 86.199 1
96.68 NA NA 1 95.74
ENSMUSG00000070383 Olfr389 protein_coding 1 84.564 NA
NA 1 79.554 1 95.877
ENSMUSG00000070385 Ampd1 protein_coding 10.502 4.916 1.036
87.037 0.092 0.72 0.853 94.99
ENSMUSG00000070388 Fbxo39 protein_coding 1.177 72.85 0.933
86.604 0.907 86.475 0.756 93.264
ENSMUSG00000070390 Nlrp1b protein_coding 1.494 3.176 0.782
28.822 0.855 60.233 1.009 87.447
ENSMUSG00000070394 Tmem256 protein_coding 0.976 46.289
1.032 92.561 0.989 76.498 1.045 60.466
ENSMUSG00000070402 Gm12302 lincRNA 1 86.819 NA NA
1 84.434 1 95.92
ENSMUSG00000070407 Hs3st3b1 protein_coding 0.84 29.725
1.257 75.21 0.944 74.535 0.823 86.991
ENSMUSG00000070411 Olfr485 pseudogene 1 81.925 1
95.35 1 87.686 1 96.287
ENSMUSG00000070417 Olfr2 protein_coding 1 85.499 1
96.268 1 79.119 1 92.762
ENSMUSG00000070419 Cyp3a57 protein_coding 0.483 13.952
1.662 40.99 1.246 60.104 1.652 83.067
ENSMUSG00000070420 Zscan25 protein_coding 0.908 8.709 0.966

82.503 1.142 10.819 0.982 91.65
ENSMUSG00000070421 Olfr658 protein_coding 1 84.324 NA
NA 1 85.58 1 86.118
ENSMUSG00000070423 Olfr558 protein_coding 1.145 8.281 1.211
82.76 0.718 0.72 0.999 92.181
ENSMUSG00000070424 Art5 protein_coding 1.587 31.24 1.046 86.793
0.604 16.129 1.661 65.558
ENSMUSG00000070425 Trpc2 protein_coding 0.911 3.641 1.104 79.261
0.991 85.464 0.986 94.19
ENSMUSG00000070426 Rnf121 protein_coding 1.024 32.676
0.981 84.017 0.997 68.584 1.092 42.736
ENSMUSG00000070427 Il18bp protein_coding 0.96 66.406
1.111 79.367 0.935 48.387 1.133 64.513
ENSMUSG00000070436 Serpinh1 protein_coding 0.761 4.176 1.194
75.21 1.102 45.035 1.217 31.133
ENSMUSG00000070438 Olfr313 protein_coding 1 85.655 1
97.263 1 78.369 1 95.273
ENSMUSG00000070448 Vmn2r89 protein_coding 1 85.637 1
90.287 1 81.388 1 95.74
ENSMUSG00000070458 Vmn2r73 protein_coding 1 75.298 1
97.288 1 84.308 1 88.197
ENSMUSG00000070459 Olfr290 protein_coding 1 84.324 1
95.211 1 82.31 1 95.72
ENSMUSG00000070460 Olfr291 protein_coding 1 84.049 1
96.216 1 82.74 1 96.183
ENSMUSG00000070461 9230112E08Rik protein_coding 0.97 74.021
0.93 75.4 1.11 24.07 0.956 85.493
ENSMUSG00000070462 Mesdc1 protein_coding 0.93 14.849 1.18
71.222 0.913 68.266 0.963 86.209
ENSMUSG00000070469 Adamtsl3 protein_coding 1.04 54.44 0.92
38.699 1.048 54.809 0.986 95.832
ENSMUSG00000070471 Fam194a protein_coding 1.205 66 1.145
96.407 0.726 88.076 0.869 94.016
ENSMUSG00000070473 Cldn3 protein_coding 0.769 27.968 0.962
79.894 1.349 20.085 1.073 87.651
ENSMUSG00000070476 Fam217b protein_coding 1.067 74.958
1.257 72.546 0.744 0.879 1.101 82.328
ENSMUSG00000070489 4930527J03Rik pseudogene 1 81.24 1
96.763 1 75.009 1 95.676
ENSMUSG00000070493 Chchd2 protein_coding 0.996 71.36 0.93
79.807 1.076 28.997 0.99 86.991
ENSMUSG00000070495 Ctcfl protein_coding 0.667 4.385 0.956 87.322
1.561 6.202 0.926 94.468
ENSMUSG00000070498 Tmem132b protein_coding 0.872 53.679
0.631 81.443 1.821 17.412 0.726 92.31
ENSMUSG00000070501 BC094916 protein_coding 1.43 28.098
1.893 92.561 0.37 27.675 1.507 67.691
ENSMUSG00000070509 Rgma protein_coding 0.887 25.551 1.008
95.431 1.116 75.155 0.963 86.991
ENSMUSG00000070511 Gm10295 protein_coding 1 82.732 1
96.843 1 88.657 1 96.358
ENSMUSG00000070520 Ndnl2 protein_coding 0.963 80.751 0.97
80.463 1.07 33.042 0.987 96.183
ENSMUSG00000070522 Gm6505 protein_coding 1 71.217 1

96.843 1 82.31 1 95.877
ENSMUSG00000070524 Fcrlb protein_coding 0.921 86.112 0.876
95.088 1.241 87.658 1.031 93.156
ENSMUSG00000070526 Peg12 protein_coding 0.944 57.544 0.942
85.921 1.119 37.369 1.03 95.273
ENSMUSG00000070527 NA NA NA NA 1.053 87.041 0.99
70.328 0.976 87.218
ENSMUSG00000070529 Wfdc10 protein_coding 1 81.01 1
93.908 1 85.757 1 92.762
ENSMUSG00000070530 Wfdc16 protein_coding 1 75.858 1
95.211 1 84.308 1 95.74
ENSMUSG00000070531 Wfdc6b protein_coding 1 80.51 1
93.765 1 86.258 1 94.825
ENSMUSG00000070544 Top1 protein_coding 1.048 63.126 1.018
97.263 0.936 78.293 0.987 83.067
ENSMUSG00000070546 Mrgprb3 protein_coding 1 83.823 1
95.504 1 88.161 1 95.92
ENSMUSG00000070547 Mrgprb1 protein_coding 1.539 0.999 1.022
84.594 0.637 18.122 1.14 95.74
ENSMUSG00000070550 Mrgprb4 protein_coding 1 84.397 1
96.216 1 86.258 1 95.877
ENSMUSG00000070551 Mrgprb5 protein_coding 1 79.577 1
95.708 1 84.724 1 96.183
ENSMUSG00000070552 Mrgprx1 protein_coding 1 48.354 1
82.904 1 84.626 1 83.067
ENSMUSG00000070563 Spaca4 protein_coding 0.628 83.726
2.087 90.29 0.761 87.103 2.046 83.067
ENSMUSG00000070564 Ntn5 protein_coding 0.567 53.679 NA NA
1.545 60.5 1.419 70.36
ENSMUSG00000070565 Rasal2 protein_coding 0.954 35.894
0.883 70.864 1.187 10.76 NA NA
ENSMUSG00000070570 Slc17a7 protein_coding 1.191 59.211
0.849 81.284 0.985 80.089 0.767 90.611
ENSMUSG00000070574 2310016G11Rik protein_coding 1 53.138
1 85.985 1 72.464 1 91.625
ENSMUSG00000070576 Mn1 protein_coding 1.268 10.687 1.476
60.309 0.534 0.72 1.096 75.198
ENSMUSG00000070577 Gm572 protein_coding 3.742 7.501 0.491 85.95
0.545 27.675 0.528 85.493
ENSMUSG00000070583 Fv1 protein_coding 1.182 6.61 NA NA
0.868 49.967 0.968 87.182
ENSMUSG00000070594 Gm4788 protein_coding 0.892 58.077
1.888 83.104 0.592 62.533 0.705 96.183
ENSMUSG00000070601 Vmn2r84 protein_coding 1 84.294 1
96.138 1 86.602 1 93.734
ENSMUSG00000070604 Vsig10l protein_coding 0.952 36.259
1.051 79.261 0.997 83.811 0.951 87.331
ENSMUSG00000070605 Gm13251 protein_coding 1.333 2.651 0.757
32.512 0.987 86.475 1.07 66.068
ENSMUSG00000070606 Gm10298 pseudogene 1 71.915 1
96.897 1 82.903 1 94.297
ENSMUSG00000070609 Gm13177 protein_coding 1 86.112 1
95.708 1 86.258 1 96.192
ENSMUSG00000070610 Gm13127 pseudogene 1 84.452 1

95.088 1 87.274 1 86.445
ENSMUSG00000070616 Gm13040 protein_coding 1 85.637 1
96.847 NA NA 1 92.748
ENSMUSG00000070617 NA NA NA NA 1 96.407 NA NA
1 95.273
ENSMUSG00000070618 BC080695 protein_coding 1 84.168 1
92.631 NA NA 1 96.287
ENSMUSG00000070619 Gm13119 protein_coding 1 44.334 1
95.708 1 73.027 1 95.72
ENSMUSG00000070621 Vmn2r-ps60 pseudogene 1 86.819 1
97.097 NA NA 1 95.405
ENSMUSG00000070632 Btbd8 protein_coding 1.088 42.134 1.007
87.041 0.909 79.255 0.953 85.493
ENSMUSG00000070637 Gm694 protein_coding 1.131 36.686 0.889
84.594 0.992 67.962 0.937 85.493
ENSMUSG00000070639 Lrrc8b protein_coding 0.946 59.028 NA
NA 1.115 63.665 0.953 86.699
ENSMUSG00000070641 Gm16410 pseudogene 1 71.516 1
95.504 1 84.724 1 92.851
ENSMUSG00000070643 Sox13 protein_coding 0.893 24.748 1.063
89.014 1.055 41.806 0.974 88.136
ENSMUSG00000070644 Etnk2 protein_coding 1.489 16.959 1.164
93.863 0.579 6.858 1.077 88.755
ENSMUSG00000070667 Rpl31-ps10 pseudogene 1 30.877 1
90.116 1 83.538 1 95.273
ENSMUSG00000070677 D5Ertd577e protein_coding 1 76.396 1
95.088 1 84.308 1 96.358
ENSMUSG00000070683 Lactbl1 protein_coding 1 85.59 1
96.843 1 76.498 1 91.807
ENSMUSG00000070686 C87414 protein_coding 1 84.294 1
90.378 1 86.831 1 95.74
ENSMUSG00000070687 Htr1d protein_coding 1.076 58.896 1.264
64.123 0.734 1.764 0.891 86.445
ENSMUSG00000070690 5830473C10Rik protein_coding 1 85.203
1 96.68 1 85.352 1 96.183
ENSMUSG00000070691 Runx3 protein_coding 1.04 80.751 1.465
71.617 0.659 3.428 0.856 84.29
ENSMUSG00000070692 Rps12-ps10 pseudogene 1 65.733 NA NA
1 81.525 1 94.654
ENSMUSG00000070695 Cntnap5a protein_coding 1 33.002 1
92.561 1 69.315 1 95.273
ENSMUSG00000070697 Utp3 protein_coding 1.074 75.196 0.931
90.256 1.003 72.695 1.028 43.377
ENSMUSG00000070699 Sars2 protein_coding 0.796 1.379 1.157 79.579
1.086 28.979 1.1 78.948
ENSMUSG00000070702 Csn1s1 protein_coding 0.359 14.403
0.665 85.418 4.17 10.079 1.572 72.369
ENSMUSG00000070705 Eid2b protein_coding 0.954 33.002 1.019
93.529 1.031 69.196 0.946 83.724
ENSMUSG00000070708 Gtsf1l protein_coding 1 77.407 1
97.129 1 83.275 1 96.358
ENSMUSG00000070709 1700049G17Rik protein_coding 1.066 67.374
0.97 94.597 0.968 83.158 0.991 91.372
ENSMUSG00000070713 Gm10282 protein_coding 1 77.497 1

96.378 1 60.614 1 93.685
ENSMUSG00000070717 Gm10300 protein_coding 0.668 23.923
0.924 86.604 1.622 12.703 1.283 57.075
ENSMUSG00000070719 Pla2g4d protein_coding 1 85.857 1
93.046 1 84.308 1 96.192
ENSMUSG00000070720 Tmem200b protein_coding 1.091 15.456
0.898 72.399 1.024 84.669 1.021 93.156
ENSMUSG00000070729 Gm12966 pseudogene 1 84.564 1
92.253 1 65.409 1 86.699
ENSMUSG00000070730 Rmdn3 protein_coding 0.836 0.987 1.05 85.756
1.139 7.024 1.057 82.091
ENSMUSG00000070732 Rbm44 protein_coding 1.376 44.334 1.068
87.919 0.677 85.675 1.027 96.287
ENSMUSG00000070733 Fryl protein_coding 1.06 84.564 1
95.504 0.943 70.214 0.925 84.29
ENSMUSG00000070737 Gm12942 protein_coding 1.055 48.462
0.896 61.883 1.059 57.637 0.802 83.724
ENSMUSG00000070738 Dgkd protein_coding 0.976 68.846 0.916
82.848 1.117 36.011 0.96 91.372
ENSMUSG00000070777 Ceacam20 protein_coding 0.205 5.967 4.038
89.434 1.202 34.727 3.46 51.901
ENSMUSG00000070778 NA NA NA NA 1 95.708 1
84.504 1 92.018
ENSMUSG00000070780 Rbm47 protein_coding 0.942 82.231 1.12
72.642 0.944 28.237 0.937 83.724
ENSMUSG00000070794 BC016548 lincRNA 0.843 69.985 0.978
95.708 1.21 67.163 0.699 83.724
ENSMUSG00000070796 Psg21 protein_coding 1 85.637 1
95.708 1 86.666 1 96.183
ENSMUSG00000070797 Psg27 protein_coding 1 86.172 1
96.897 1 80.899 1 95.273
ENSMUSG00000070799 NA NA NA NA 1 96.68 NA NA 1
90.674
ENSMUSG00000070802 Pnmal2 protein_coding 1.152 42.555
1.018 91.584 0.854 22.009 1.071 80.497
ENSMUSG00000070803 Cited4 protein_coding 1.141 50.913
0.971 96.013 0.901 58.516 0.893 86.991
ENSMUSG00000070806 Zmynd12 protein_coding 0.769 53.679
0.778 79.261 1.668 31.975 1.041 85.493
ENSMUSG00000070808 Gltscr1 protein_coding 0.947 37.531
1.066 81.54 0.987 79.255 0.95 83.724
ENSMUSG00000070810 Sult2a6 protein_coding 1 84.534 1
96.948 1 86.258 1 96.358
ENSMUSG00000070811 Sult2a2 protein_coding 1 84.738 1
96.763 1 84.896 1 96.183
ENSMUSG00000070814 6330408A02Rik protein_coding 0.951 37.82
1.015 91.83 1.034 37.741 1.006 89.052
ENSMUSG00000070815 Vmn1r87 protein_coding 1 86.658 1
95.504 1 88.729 1 95.676
ENSMUSG00000070816 Vmn1r86 protein_coding 1 72.85 1
95.504 1 80.163 1 95.74
ENSMUSG00000070817 Vmn1r85 protein_coding 1 81.196 NA
NA 1 84.896 1 95.92
ENSMUSG00000070820 Olfr1339 protein_coding 1 83.09 1

95.211 1 80.899 1 95.593
ENSMUSG00000070821 Olfr1340 protein_coding 1 74.299 1
96.308 1 84.071 1 93.964
ENSMUSG00000070822 Zscan18 protein_coding 0.901 70.597
0.922 79.475 1.198 33.802 0.992 87.735
ENSMUSG00000070823 Gm1043 protein_coding 1 59.358 NA
NA 1 81.032 1 96.042
ENSMUSG00000070828 Zscan4f protein_coding 1 85.391 1
95.301 NA NA 1 96.248
ENSMUSG00000070834 Gm13015 pseudogene 1 79.068 1
87.452 1 81.29 1 95.877
ENSMUSG00000070837 Aurkc protein_coding 0.745 72.546 1.415
82.904 0.95 66.201 1.74 69.404
ENSMUSG00000070841 Vmn2r34 protein_coding 1 76.135 1
96.001 NA NA 1 95.72
ENSMUSG00000070844 Vmn2r42 protein_coding 1 83.333 1
95.301 1 88.601 1 96.192
ENSMUSG00000070852 Olfr1121 protein_coding 1 85.832 1
94.96 1 83.925 1 96.358
ENSMUSG00000070853 Olfr1120 protein_coding 1 85.637 1
97.263 1 88.693 1 95.74
ENSMUSG00000070855 NA NA NA NA 1 96.843 1
85.464 1 94.904
ENSMUSG00000070856 Olfr1115 protein_coding 1 83.823 1
96.948 1 86.084 1 93.316
ENSMUSG00000070857 Olfr1113 protein_coding 1 85.946 NA
NA 1 83.857 1 92.762
ENSMUSG00000070858 Gm1673 protein_coding 1.18 78.538
0.812 97.155 1.039 84.724 0.748 91.291
ENSMUSG00000070867 Trabd2b protein_coding 1.165 84.661
1.228 71.617 0.699 44.678 1.642 44.679
ENSMUSG00000070868 Skint3 protein_coding 1.517 13.292 0.75
64.624 0.88 87.431 0.816 83.952
ENSMUSG00000070870 Cryge protein_coding 1 85.391 NA NA
1 87.744 1 94.752
ENSMUSG00000070871 Ccnyl1 protein_coding 1.133 6.683 1.108
80.775 0.796 3.428 1.052 83.067
ENSMUSG00000070873 Lilra5 protein_coding 1.056 83.169
1.179 82.76 0.803 39.079 0.742 93.964
ENSMUSG00000070875 Olfr1100 protein_coding 1 84.324 1
96.407 1 88.161 1 96.287
ENSMUSG00000070877 Ldlrad1 protein_coding 0.802 70.213
0.836 82.947 1.486 45.667 1.157 95.273
ENSMUSG00000070883 Ccdc173 protein_coding 0.969 68.846
0.957 90.744 1.075 38.099 0.969 89.152
ENSMUSG00000070886 Gm10304 protein_coding 1 69.24 1
95.809 1 87.971 1 96.287
ENSMUSG00000070891 Gm12689 protein_coding 0.905 38.555
0.907 96.138 1.22 41.235 0.739 91.625
ENSMUSG00000070896 Rpl23a-ps1 protein_coding 1 85.129 NA
NA 1 86.258 1 95.593
ENSMUSG00000070900 Gm10306 protein_coding 0.735 41.021
1.211 83.104 NA NA 0.892 95.35
ENSMUSG00000070902 Zfp352 protein_coding 0.968 82.705

0.919 96.216 1.122 84.724 1.643 95.273
ENSMUSG00000070904 Ifna4 protein_coding 1 85.946 1
96.001 1 82.436 1 96.287
ENSMUSG00000070906 Ifna11 protein_coding 1 78.428 1
94.848 1 71.232 1 92.52
ENSMUSG00000070907 Ifna7 protein_coding 1 84.263 1
96.216 1 87.173 1 96.184
ENSMUSG00000070923 Klhl9 protein_coding 1.119 0 0.98 84.594
0.914 3.428 1.013 71.008
ENSMUSG00000070933 Speer4d protein_coding 0.639 10.739 0.81
97.155 1.932 27.047 1.287 96.192
ENSMUSG00000070934 Rraga protein_coding 0.998 74.147 1.062
80.678 0.938 19.765 0.998 92.675
ENSMUSG00000070939 Tgfbrap1 protein_coding 1.014 33.854 1.08
71.617 0.916 6.858 1.04 77.854
ENSMUSG00000070942 Il1rl2 protein_coding 1.397 0.577 0.899
81.731 0.796 4.23 1.121 48.119
ENSMUSG00000070943 Olfr356 protein_coding 1 71.142 1
91.643 1 83.066 1 94.19
ENSMUSG00000070953 Rabepk protein_coding 0.898 16.02 0.92
74.851 1.211 8.93 1.089 59.464
ENSMUSG00000070972 Dnajc25 protein_coding 1.032 84.049
0.955 92.931 1.011 82.575 1.108 60.968
ENSMUSG00000070977 Defb44-ps pseudogene 1 71.674 1
96.68 1 88.274 1 94.153
ENSMUSG00000070979 Actl7a protein_coding 1 62.007 1
96.268 1 38.799 1 87.542
ENSMUSG00000070983 Olfr270 protein_coding 1 85.092 NA
NA 1 88.161 1 92.181
ENSMUSG00000070984 4930552N02Rik processed_transcript 1
85.637 NA NA 1 86.475 1 95.273
ENSMUSG00000070985 Acnat1 protein_coding 0.525 11.769
0.766 82.503 2.476 17.412 1.428 91.525
ENSMUSG00000070990 Foxe1 protein_coding 1 73.292 1
95.735 1 65.8 1 87.7
ENSMUSG00000070995 Gm6313 lincRNA 1 84.263 1
97.155 1 87.274 1 96.086
ENSMUSG00000070997 1700055D18Rik protein_coding 1.21 3.598
0.906 79.807 0.911 25.512 0.927 83.724
ENSMUSG00000070999 Ccin protein_coding 1.14 60.168 0.837
88.705 1.043 87.744 0.836 90.691
ENSMUSG00000071000 Olfr155 protein_coding 1 86.145 1
97.097 1 79.776 1 95.74
ENSMUSG00000071001 Hrct1 protein_coding 0.873 8.709 0.985 93.654
1.158 11.665 0.991 95.877
ENSMUSG00000071005 Ccl19 protein_coding 1.646 0.976 0.935 97.155
0.647 0.72 0.971 91.783
ENSMUSG00000071014 Ndufb6 protein_coding 0.765 1.15 1.038
81.039 1.256 22.517 1 96.287
ENSMUSG00000071015 Gm136 protein_coding 1 77.916 1
90.712 1 67.648 1 91.928
ENSMUSG00000071019 Sdr16c6 protein_coding 1 81.01 1
95.735 NA NA 1 93.316
ENSMUSG00000071036 Gm10309 protein_coding 0.659 38.983

0.789 87.998 1.92 24.07 1.309 83.067
ENSMUSG00000071037 Camkmt protein_coding 0.99 73.401
1.021 90.116 0.985 37.103 1.087 88.755
ENSMUSG00000071040 Gm9386 pseudogene 1 73.585 1
94.48 1 74.082 1 96.139
ENSMUSG00000071041 Gm15210 pseudogene 2.122 22.633 1.389
95.088 0.339 27.321 0.603 85.493
ENSMUSG00000071042 Rasgrp3 protein_coding 1.077 7.553 1.19
71.617 0.778 0.72 0.932 83.724
ENSMUSG00000071047 Ces1a protein_coding 0.984 57.38 NA NA
0.543 68.82 1.305 92.748
ENSMUSG00000071054 Safb protein_coding 0.951 41.759 1.065
92.111 NA NA 0.93 85.493
ENSMUSG00000071064 Zfp827 protein_coding 0.855 10.704
1.016 87.029 1.151 24.791 0.96 92.12
ENSMUSG00000071065 Olfr806 protein_coding 1 86.843 1
94.848 1 83.538 1 93.964
ENSMUSG00000071068 Treml2 protein_coding 1.193 84.865
0.868 88.705 0.963 87.686 1.409 58.721
ENSMUSG00000071072 Ptges3 protein_coding 1.112 14.205
1.078 97.263 0.833 29.451 0.965 93.117
ENSMUSG00000071073 Lrrc73 protein_coding 1.173 58.896 1.03
95.329 0.825 69.583 1.129 86.445
ENSMUSG00000071074 Yipf3 protein_coding 0.921 10.143 1.024
93.574 1.062 57.286 0.999 89.587
ENSMUSG00000071076 Jund protein_coding 1.083 81.24 1.017 93.529
0.91 53.224 0.881 96.086
ENSMUSG00000071078 Nr2c2ap protein_coding 1.029 79.853
1.021 92.561 0.948 48.577 0.995 93.156
ENSMUSG00000071083 Gm10311 protein_coding 1.225 45.61 0.818
94.48 1 87.274 0.794 85.493
ENSMUSG00000071084 Olfr761 protein_coding 1 85.355 NA
NA NA NA 1 96.192
ENSMUSG00000071089 Trim75 protein_coding 1 80.688 1
97.263 1 82.436 1 95.832
ENSMUSG00000071102 Gm10313 pseudogene 1 85.832 1
96.984 1 84.896 1 94.825
ENSMUSG00000071103 1700029J07Rik protein_coding 0.912 64.284
0.922 68.848 1.186 26.498 0.916 85.493
ENSMUSG00000071112 B230216G23Rik protein_coding 1.006 53.138
1.09 88.91 0.91 83.811 0.949 93.264
ENSMUSG00000071138 Tex24 protein_coding 1 82.231 1
96.948 1 87.898 1 95.72
ENSMUSG00000071141 Rpl36a-ps3 pseudogene 1 61.887 1
96.68 1 66.928 1 94.215
ENSMUSG00000071147 Tas2r140 protein_coding 1 85.77 1
97.129 1 88.601 1 92.903
ENSMUSG00000071149 Tas2r115 protein_coding 1 79.93 1
97.155 1 84.229 1 96.248
ENSMUSG00000071150 Tas2r121 protein_coding 1 80.608 1
94.102 1 86.258 1 96.042
ENSMUSG00000071151 Gm4799 protein_coding 1 45.746 NA
NA 1 65.409 1 91.372
ENSMUSG00000071165 Gm6040 protein_coding 1 84.049 1

96.948 1 82.31 1 92.381
ENSMUSG00000071169 Defb46 protein_coding 1 85.203 1
96.216 1 88.161 1 96.192
ENSMUSG00000071172 Srsf3 protein_coding 1.22 0 0.971 76.431
0.847 1.898 1.047 83.067
ENSMUSG00000071176 Arhgef10 protein_coding 1.032 72 0.931
87.452 1.038 84.308 1.001 93.132
ENSMUSG00000071178 Serpina1b protein_coding 1.45 56.311
0.764 86.604 0.903 76.64 1.073 86.118
ENSMUSG00000071179 Gm46 protein_coding 1 69.985 1
93.146 1 80.381 1 88.755
ENSMUSG00000071180 Smim15 protein_coding 1.077 1.689 0.951
83.392 0.976 21.431 1.051 76.347
ENSMUSG00000071181 3830408C21Rik protein_coding 0.969 47.677
0.772 74.483 1.339 20.556 0.866 93.685
ENSMUSG00000071185 Olfr1357 protein_coding 1 84.534 1
95.504 1 87.658 1 95.273
ENSMUSG00000071192 Wfikkn1 protein_coding 1.079 42.722
0.614 79.807 1.504 18.208 0.939 79.545
ENSMUSG00000071193 Gm4938 pseudogene 0.957 83.169 NA NA
1.116 86.985 1.773 87.134
ENSMUSG00000071195 Gm10318 protein_coding 1 86.843 1
95.301 1 83.397 1 95.832
ENSMUSG00000071202 Ccdc78 protein_coding 0.762 51.056
1.151 97.263 1.141 66.269 0.758 86.699
ENSMUSG00000071203 Naip5 protein_coding 1.491 0 0.877 32.358
0.762 6.858 1.012 95.559
ENSMUSG00000071204 Gm10319 pseudogene 1 78.224 1
96.138 1 86.985 1 92.12
ENSMUSG00000071207 Gm8587 pseudogene 1 83.169 NA NA
1 75.009 1 94.904
ENSMUSG00000071217 Gm2022 pseudogene 1 29.811 1
91.532 1 78.045 1 95.405
ENSMUSG00000071226 Cecr2 protein_coding 0.866 28.998 0.993
96.843 1.156 52.432 0.891 84.29
ENSMUSG00000071230 Npw protein_coding 1.337 66.652 0.928
95.301 0.806 76.754 1.111 93.964
ENSMUSG00000071235 Vrtn protein_coding 1 74.745 NA NA
1 85.792 1 95.74
ENSMUSG00000071237 Gm6253 pseudogene 1 72.372 NA NA
NA NA 1 94.82
ENSMUSG00000071252 2210408I21Rik protein_coding 1.339 0.461
0.997 92.404 0.746 1.898 0.959 95.593
ENSMUSG00000071253 Slc25a16 protein_coding 1.085 6.438 0.985
95.211 0.936 36.786 0.921 83.724
ENSMUSG00000071256 Zfp213 protein_coding 1.013 70.213 NA
NA 1.012 87.658 1.052 82.328
ENSMUSG00000071262 Zfp957 protein_coding 1.564 42.255
0.459 94.887 1.389 72.269 0.659 85.493
ENSMUSG00000071266 Zfp946 protein_coding 1.196 28.693
0.926 83.58 0.903 44.678 1.1 83.581
ENSMUSG00000071267 Zfp942 protein_coding 1.172 1.395 0.873
37.187 0.979 64.778 0.995 96.287
ENSMUSG00000071273 Gm5145 protein_coding 1 84.324 NA

NA	1	87.336	1	96.358
ENSMUSG00000071275	NA	NA	NA	NA	NA	NA	1	83.066
1	95.273
ENSMUSG00000071276	Fpr-rs7	protein_coding	1	84.049	1
97.263	1	79.897	1	96.042
ENSMUSG00000071280	Gm10322	protein_coding	1	78.538	1
88.705	1	76.754	1	94.904
ENSMUSG00000071281	Zfp71-rs1	protein_coding	1.198	2.723	0.886
79.475	0.944	35.815	1.021	95.877
ENSMUSG00000071286	Sowahc	pseudogene	1	86.843	1
95.735	1	84.724	1	95.273
ENSMUSG00000071291	Zfp58	protein_coding	1.034	30.497	0.932
83.321	1.038	84.669	1.036	91.979
ENSMUSG00000071294	Gm5196	pseudogene	1	86.819	NA	NA
NA	NA	1	96.287
ENSMUSG00000071295	Gm10323	protein_coding	1	64.014	1
97.263	1	86.552	1	93.264
ENSMUSG00000071302	2610044O15Rik8	protein_coding	1.159	26.77	NA
NA	0.859	16.719	1.238	46.412
ENSMUSG00000071303	Rps8-ps1	pseudogene	1	65.199	1
89.434	1	88.657	1	93.316
ENSMUSG00000071311	Gpr31b	protein_coding	1.659	76.546
0.495	88.704	1.214	83.925	0.559	92.181
ENSMUSG00000071317	Bves	protein_coding	1.94	12.072	NA	NA
0.639	6.858	1.168	79.442
ENSMUSG00000071324	Armc2	protein_coding	0.942	83.658	0.899
90.138	1.178	65.235	1.218	87.088
ENSMUSG00000071332	E230015B07Rik	processed_transcript	0.817
58.56	0.782	97.155	1.563	74.082	0.989	91.872
ENSMUSG00000071335	G630090E17Rik	protein_coding	1.09	86.464
0.904	95.708	1.011	88.076	1.006	95.72
ENSMUSG00000071337	Tia1	protein_coding	0.814	1.031	0.985	90.053
1.249	5.864	0.984	96.071
ENSMUSG00000071342	Lsmem1	protein_coding	5.737	6.026	NA	NA
0.151	1.061	0.792	94.904
ENSMUSG00000071343	Gm10327	pseudogene	1	64.893	1
95.088	1	54.673	1	94.752
ENSMUSG00000071347	C1qtnf9	protein_coding	0.893	32.749
0.947	85.418	1.179	13.956	1.029	85.577
ENSMUSG00000071350	Setdb2	protein_coding	0.902	83.263	0.9
77.598	1.23	36.578	1.156	78.379
ENSMUSG00000071359	Tbpl1	protein_coding	1.106	4.925	0.921	85.116
0.982	38.434	1.069	57.1
ENSMUSG00000071361	Mcpt9	protein_coding	1	77.497	1
96.268	1	87.744	1	94.825
ENSMUSG00000071369	Map3k5	protein_coding	1.013	37.414
0.962	88.897	1.027	78.713	1.127	61.984
ENSMUSG00000071379	Hpcal1	protein_coding	1.182	0	0.916
63.773	0.924	24.523	1.037	93.964
ENSMUSG00000071398	2410004P03Rik	protein_coding	1.232	64.956
0.857	84.594	0.95	80.015	0.934	87.735
ENSMUSG00000071414	Gm6736	pseudogene	1	85.283	1
93.908	1	85.921	1	93.261
ENSMUSG00000071415	Rpl23	protein_coding	1.129	0.2	0.891	11.228

0.992 71.734 1.111 48.177
ENSMUSG00000071419 Rps15-ps2 pseudogene 1 81.125 NA NA
1 86.258 1 93.457
ENSMUSG00000071424 Grid2 protein_coding 1.58 44.061 1.043
97.263 0.605 31.676 0.794 85.493
ENSMUSG00000071428 Vmn1r27 protein_coding 1 80.608 1
95.504 1 85.792 1 94.904
ENSMUSG00000071434 9230019H11Rik protein_coding 1 85.728
1 91.83 NA NA 1 96.183
ENSMUSG00000071451 Psmg4 protein_coding 1.092 35.787 0.994
96.216 0.923 23.212 0.957 86.991
ENSMUSG00000071452 Gm11397 protein_coding 1 86.74 1
92.793 1 86.666 1 92.903
ENSMUSG00000071454 Dtnb protein_coding 0.882 25.3 0.928 93.654
1.223 39.471 1.041 92.12
ENSMUSG00000071456 1110002L01Rik protein_coding 0.863 2.524
1.021 94.848 1.13 7.635 1.045 87.7
ENSMUSG00000071466 Gm11360 protein_coding 1 76.827 NA
NA 1 86.552 1 96.287
ENSMUSG00000071470 Ccnb1ip1 protein_coding 1 77.497 1
90.378 1 80.634 1 96.183
ENSMUSG00000071471 Krtap26-1 protein_coding 1 76.686 1
95.211 1 82.575 1 92.762
ENSMUSG00000071475 Rpl21-ps5 pseudogene 1 85.77 1 96.216
1 83.925 1 95.273
ENSMUSG00000071477 Zfp777 protein_coding 0.991 51.447 0.95
89.713 1.061 52.8 0.97 88.245
ENSMUSG00000071478 Hist1h2ad protein_coding 0.341 28.242 1.56
90.389 1.885 63.301 1.764 83.067
ENSMUSG00000071481 Olfr437 protein_coding 1 81.01 NA NA
1 83.158 1 95.273
ENSMUSG00000071489 Ptgdr protein_coding 1.037 60.003 1.296
92.931 0.742 82.903 1.23 94.153
ENSMUSG00000071490 Vmn1r212 protein_coding 1 75.196 NA
NA 1 83.158 1 96.248
ENSMUSG00000071491 Vmn1r209 protein_coding 1 84.397 1
96.216 1 83.925 1 91.078
ENSMUSG00000071493 Vmn1r208 protein_coding 1 85.391 1
95.504 1 83.857 1 92.903
ENSMUSG00000071494 Olfr455 protein_coding 1 82.982 1
88.881 1 57.907 1 93.612
ENSMUSG00000071495 Vmn1r206 protein_coding 1 81.178 NA
NA 1 86.831 1 93.734
ENSMUSG00000071506 Tmem139 protein_coding 0.942 86.474
0.862 62.322 1.228 56.708 0.802 85.493
ENSMUSG00000071510 Olfr172 protein_coding 1 86.658 1
95.708 1 86.666 1 92.748
ENSMUSG00000071517 Gm10334 protein_coding 1 85.203 1
96.68 1 84.308 1 92.018
ENSMUSG00000071519 Prss3 protein_coding 1 85.832 1
97.129 NA NA 1 95.877
ENSMUSG00000071521 Try10 protein_coding 1 79.93 1 97.288
1 88.601 1 96.358
ENSMUSG00000071522 Olfr263 protein_coding 1 60.168 NA

NA	1	88.601	1	92.762
ENSMUSG00000071528	Usmg5	protein_coding	0.938	31.11	1.021	96.843
1.04	49.384	1.071	83.067
ENSMUSG00000071531	Gprin2	protein_coding	0.784	38.555
1.146	96.407	1.113	41.235	0.873	94.654
ENSMUSG00000071532	Gm10335	protein_coding	1	83.523	1
95.708	1	84.229	1	92.851
ENSMUSG00000071533	Pcnp	protein_coding	1.119	3.151	0.911	48.565
0.983	70.214	1.004	93.457
ENSMUSG00000071537	Klrg2	protein_coding	1.296	35.787	0.775
79.807	0.999	86.666	0.955	90.455
ENSMUSG00000071540	3425401B19Rik	protein_coding	5.474	11.638
2.252	83.104	0.081	0.72	0.986	96.287
ENSMUSG00000071547	Nt5dc2	protein_coding	1.244	1.079	1.027
82.848	0.779	41.506	1.157	44.679
ENSMUSG00000071550	Wdr52	protein_coding	2.2	22.025	0.681
83.876	0.668	54.521	0.478	83.952
ENSMUSG00000071551	Akr1c19	protein_coding	1.375	14.008
0.889	67.513	0.816	67.648	0.98	95.877
ENSMUSG00000071552	Tigit	protein_coding	1.307	49.202	0.909
89.277	0.844	83.607	0.653	86.699
ENSMUSG00000071553	Cpa2	protein_coding	1.365	81.125	1.094
95.431	0.671	56.822	0.772	95.72
ENSMUSG00000071561	BC100530	protein_coding	1	84.324	1
91.361	1	87.37	1	93.156
ENSMUSG00000071562	Stfa1	protein_coding	1	78.909	1
87.857	1	70.9	1	94.384
ENSMUSG00000071567	A830039N20Rik	lincRNA	1.985	7.174	0.744
78.877	0.674	45.799	0.753	83.724
ENSMUSG00000071568	Gm5874	pseudogene	1	86.351	NA	NA
NA	NA	1	95.777
ENSMUSG00000071573	Rnls	protein_coding	0.724	25.3	0.993	90.744
1.391	25.93	1.045	96.358
ENSMUSG00000071584	Atf7	protein_coding	1	82.092	1
95.301	1	62.648	1	96.183
ENSMUSG00000071586	Gm10337	protein_coding	0.818	77.615
1.067	96.897	1.147	88.203	1.2	96.358
ENSMUSG00000071591	Gm10339	protein_coding	1	76.042	1
94.597	1	87.173	1	92.181
ENSMUSG00000071604	Fam189a2	protein_coding	0.927	85.857
0.917	88.897	1.173	78.498	0.972	90.289
ENSMUSG00000071613	Gm10340	protein_coding	1	83.263	1
95.301	1	87.744	1	96.248
ENSMUSG00000071629	Olfr1477	protein_coding	1	86.57	1
95.504	1	88.729	1	94.654
ENSMUSG00000071632	2510002D24Rik	protein_coding	1.083	23.254
0.999	86.538	0.922	27.86	0.906	83.724
ENSMUSG00000071636	Rimbp3	protein_coding	0.77	77.407
0.919	90.378	1.412	57.637	0.826	96.248
ENSMUSG00000071637	Cebpd	pseudogene	0.897	68.353	0.899	87.998
1.233	23.41	1.045	93.964
ENSMUSG00000071644	Eef1g	protein_coding	1.055	52.429	0.962
67.513	0.988	88.729	1.083	46.846
ENSMUSG00000071645	Tut1	protein_coding	0.981	56.768	0.968

86.861 1.054 48.772 0.999 92.263
ENSMUSG00000071646 Mta2 protein_coding 1.005 84.324 1.015
81.039 0.977 10.934 0.983 88.053
ENSMUSG00000071647 Eml3 protein_coding 0.987 76.968 0.987
91.361 1.028 62.237 0.966 85.493
ENSMUSG00000071648 Rom1 protein_coding 1.026 67.608 0.932
73.766 1.041 84.961 1.012 92.018
ENSMUSG00000071649 B3gat3 protein_coding 0.974 16.472
0.998 95.431 1.031 38.152 0.935 85.493
ENSMUSG00000071650 Ganab protein_coding 0.956 40.568 1.018
83.104 1.028 71.615 1.013 93.316
ENSMUSG00000071652 Ints5 protein_coding 1.061 34.861 0.987
93.146 0.952 21.431 1.013 93.964
ENSMUSG00000071653 1810009A15Rik protein_coding 0.898 21.658
1.078 90.68 1.028 42.199 0.954 86.699
ENSMUSG00000071654 AI462493 protein_coding 0.938 45.728
0.975 83.733 1.095 22.131 0.989 84.29
ENSMUSG00000071655 Ubxn1 protein_coding 1.015 86.74 0.955 60.678
1.029 39.471 0.976 86.699
ENSMUSG00000071656 Lrrn4cl protein_coding 1.194 0.2 1.172
89.494 0.713 15.904 0.942 90.95
ENSMUSG00000071657 Bscl2 protein_coding 0.907 2.436 1.095 79.261
1.004 72.853 0.919 85.493
ENSMUSG00000071658 Gng3 protein_coding 1.023 75.196 0.858
87.147 1.136 83.066 0.934 92.675
ENSMUSG00000071659 Hnrnpul2 protein_coding 0.965 36.945
1.011 96.216 1.025 52.574 1.007 80.497
ENSMUSG00000071660 Ttc9c protein_coding 1.031 29.596 1.038
90.116 0.936 24.523 0.975 86.445
ENSMUSG00000071661 Zbtb3 protein_coding 0.947 81.465 1.21
72.642 0.873 22.009 1.039 88.512
ENSMUSG00000071662 Polr2g protein_coding 1.038 84.294
0.952 79.475 1.014 56.497 1.057 70.539
ENSMUSG00000071669 Snx29 protein_coding 1.036 62.689 0.925
75.493 1.044 78.498 0.859 84.29
ENSMUSG00000071679 Zcchc16 protein_coding 1 86.658 1
82.947 1 69.196 1 96.042
ENSMUSG00000071680 Gm7123 pseudogene 1 73.292 NA NA
1 88.349 1 96.183
ENSMUSG00000071686 Tex13a protein_coding 1 83.418 1
96.948 1 63.665 1 94.904
ENSMUSG00000071690 Gm10344 protein_coding 1.181 86.474
1.199 92.062 0.707 75.967 0.563 95.74
ENSMUSG00000071691 Gm960 protein_coding 0.77 68.955 0.951
77.598 1.367 17.279 1.027 94.153
ENSMUSG00000071708 Sms protein_coding 1.275 8.847 0.874 93.654
0.9 21.431 0.998 95.74
ENSMUSG00000071711 Mpst protein_coding 0.969 48.036 0.942
91.275 1.096 38.683 0.879 84.29
ENSMUSG00000071713 Csf2rb protein_coding 1.083 58.38 0.889
84.858 1.035 78.369 0.929 83.724
ENSMUSG00000071714 Csf2rb2 protein_coding 1.144 1.441 0.96
90.744 0.908 3.123 0.99 94.654
ENSMUSG00000071715 Ncf4 protein_coding 1.32 5.527 1.043 96.216

0.724 17.662 0.962 94.904
ENSMUSG00000071719 Tmem28 protein_coding 1.184 29.536 1.35
83.104 0.623 11.751 1.307 70.624
ENSMUSG00000071721 1700010D01Rik protein_coding 1 80.751
1 95.504 1 88.657 1 92.181
ENSMUSG00000071722 Spin4 protein_coding 0.942 54.176 1.118
82.904 0.952 87.744 0.991 94.904
ENSMUSG00000071723 Gspt2 protein_coding 1.146 59.211 0.868
60.678 1.007 70.623 1.007 83.276
ENSMUSG00000071724 Smpd5 protein_coding 1.006 69.985 0.978
96.948 1.019 78.498 0.812 83.724
ENSMUSG00000071726 Gm5941 protein_coding 1 83.169 1
95.301 1 85.58 1 96.248
ENSMUSG00000071738 Gm4937 protein_coding 1 75.858 NA
NA 1 88.349 1 95.74
ENSMUSG00000071749 4933412E24Rik protein_coding 1 81.465
1 96.216 1 85.21 1 93.734
ENSMUSG00000071750 Gm7713 pseudogene 1 76.968 NA NA
1 84.229 1 91.525
ENSMUSG00000071753 C230004F18Rik processed_transcript 1
83.523 1 96.948 1 82.685 1 95.676
ENSMUSG00000071757 Zhx2 protein_coding 1.018 60.853 1.062
83.104 0.925 25.512 0.968 88.552
ENSMUSG00000071763 4933416I08Rik pseudogene 1 67.334 NA
NA 1 87.744 1 94.174
ENSMUSG00000071764 Olfr1320 protein_coding 1 85.655 1
95.088 1 87.898 1 93.612
ENSMUSG00000071766 Rhox12 protein_coding 1 83.823 1
96.013 1 72.378 1 92.389
ENSMUSG00000071767 Rhox7 protein_coding 1 76.827 NA NA
NA NA 1 94.752
ENSMUSG00000071769 Rhox3h protein_coding 1 84.294 NA
NA 1 88.693 1 95.273
ENSMUSG00000071770 NA NA NA NA 1 96.984 1
88.601 1 94.468
ENSMUSG00000071771 Rhox4b protein_coding 1 73.585 1
94.367 NA NA 1 91.928
ENSMUSG00000071772 Rhox3a protein_coding 1 73.292 NA
NA 1 74.535 1 93.722
ENSMUSG00000071773 Rhox1 protein_coding 1 84.865 1
95.301 1 81.976 1 92.181
ENSMUSG00000071788 Gm14525 protein_coding 1 64.014 1
96.268 NA NA 1 96.287
ENSMUSG00000071793 2610005L07Rik pseudogene 0.94 39.612
0.892 8.462 1.195 5.864 0.893 83.724
ENSMUSG00000071796 6820431F20Rik pseudogene 1.07 23.019 0.96
60.678 0.974 69.196 0.957 93.584
ENSMUSG00000071815 Fthl17 protein_coding 1 85.499 1
95.35 1 81.976 1 96.183
ENSMUSG00000071816 Ssxb5 protein_coding 1 73.292 1
96.407 1 82.31 1 82.328
ENSMUSG00000071818 4930417O22Rik antisense 1 86.351 1
97.155 1 84.896 1 95.35
ENSMUSG00000071847 Apcdd1 protein_coding 0.984 72 1.032

95.431 0.986 69.583 1.034 87.651
ENSMUSG00000071855 Ccdc112 protein_coding 0.997 80.51 0.924
90.053 1.084 69.728 0.968 94.654
ENSMUSG00000071856 Mcc protein_coding 0.942 65.388 0.977
87.857 1.086 66.928 0.974 84.19
ENSMUSG00000071858 Gm94 protein_coding 1 68.727 1
95.941 1 86.084 1 95.74
ENSMUSG00000071860 2900055J20Rik protein_coding 1 46.533
1 94.48 1 66.753 1 92.561
ENSMUSG00000071866 Ppia protein_coding 0.983 60.569 0.994
95.088 1.026 55.109 1.064 46.412
ENSMUSG00000071890 Mroh9 protein_coding 1 85.129 1
92.474 1 72.695 1 93.457
ENSMUSG00000071893 Vmn1r4 protein_coding 0.761 64.589
1.612 90.29 NA NA 0.736 86.991
ENSMUSG00000071909 Gm806 protein_coding 1 59.674 1
93.529 1 86.985 1 95.832
ENSMUSG00000071952 Gm10351 protein_coding 1 77.291 1
95.708 1 88.161 1 96.183
ENSMUSG00000071984 Fndc1 protein_coding 0.957 81.01 1.264 83.464
0.825 36.499 0.959 86.699
ENSMUSG00000072049 Vmn2r121 protein_coding 1 86.474 1
95.708 1 88.41 1 92.748
ENSMUSG00000072066 6720489N17Rik protein_coding 1.641 13.105
0.998 83.733 0.608 12.907 0.863 90.157
ENSMUSG00000072068 Gm16261 pseudogene 1 85.637 1
92.474 1 85.792 1 94.904
ENSMUSG00000072082 Ccnf protein_coding 1.3 29.811 0.921
87.857 0.837 67.029 1.29 83.581
ENSMUSG00000072100 Gm16390 protein_coding 1 84.819 NA
NA 1 79.554 1 95.273
ENSMUSG00000072109 A530040E14Rik antisense 1.466 34.696
0.903 85.116 0.752 75.637 1.125 92.018
ENSMUSG00000072115 Ang protein_coding 1.249 12.295 0.737
49.266 1.086 71.232 1.03 87.134
ENSMUSG00000072145 BC061237 protein_coding 1 86.474 1
96.407 1 87.658 1 94.99
ENSMUSG00000072188 Gm10354 protein_coding 0.689 36.823 0.98
96.216 1.476 41.235 0.876 96.183
ENSMUSG00000072214 5-Sep protein_coding 0.986 69.985 1.002
96.268 1.015 76.498 1.109 57.1
ENSMUSG00000072235 Tuba1a protein_coding 0.687 3.07 1.396
71.617 1.044 78.369 1.216 44.213
ENSMUSG00000072244 Trim6 protein_coding 0.962 43.816 0.806
81.284 1.288 22.444 1.085 88.657
ENSMUSG00000072249 Gm5635 protein_coding 1 79.93 1
95.35 1 84.961 1 94.904
ENSMUSG00000072255 Rpl31-ps17 pseudogene 1 61.887 1
96.407 1 79.119 1 95.877
ENSMUSG00000072258 Taf1a protein_coding 1.038 42.255 0.977
82.947 0.986 88.161 1.079 82.85
ENSMUSG00000072259 Gm5592 protein_coding 1 84.977 1
96.138 1 72.148 1 92.748
ENSMUSG00000072294 Klf12 protein_coding 0.948 80.251 0.846

94.102 1.249 58.414 0.83 93.612
ENSMUSG00000072295 Als2cr11 protein_coding 1 58.896 1
93.908 1 86.985 1 85.577
ENSMUSG00000072324 Gm8420 protein_coding 1 81.01 1
95.708 1 82.778 1 95.74
ENSMUSG00000072381 Gm10355 protein_coding 1 86.843 NA
NA 1 85.757 1 94.567
ENSMUSG00000072407 Gm6419 pseudogene 1 73.734 1
97.155 1 80.015 1 93.117
ENSMUSG00000072419 Dppa2 protein_coding 1 78.224 1
95.211 1 65.8 1 93.734
ENSMUSG00000072421 Gm10357 pseudogene 1 85.203 1
82.947 1 73.759 1 95.273
ENSMUSG00000072423 Psmb11 protein_coding 1 85.655 1
95.088 1 63.301 1 94.904
ENSMUSG00000072432 Gm10358 pseudogene 1 85.728 1
95.35 1 76.225 1 94.904
ENSMUSG00000072437 Nanos1 protein_coding 1.103 82.352
0.957 95.504 0.944 76.64 1.451 57.1
ENSMUSG00000072460 Atp5l-ps1 protein_coding 1 83.658 NA
NA 1 87.686 1 91.105
ENSMUSG00000072467 Gm16451 pseudogene 1 85.391 NA NA
1 73.096 1 95.877
ENSMUSG00000072473 NA NA NA NA NA NA 1 87.173
1 95.92
ENSMUSG00000072476 Gm9008 protein_coding 1 83.968 1
94.48 1 85.935 1 94.904
ENSMUSG00000072477 Gm10360 pseudogene 1 81.196 1
95.941 1 88.601 1 96.183
ENSMUSG00000072492 Gm10364 pseudogene 2.494 15.662 0.928
85.95 0.433 40.19 0.469 95.593
ENSMUSG00000072494 Ppp1r3e protein_coding 0.82 2.009 1.141
60.309 1.068 53.134 1.053 87.624
ENSMUSG00000072501 Phf20l1 protein_coding 0.957 53.138
1.044 97.155 1.002 56.708 1.005 93.413
ENSMUSG00000072511 Hhla1 protein_coding 1 84.168 1
95.708 1 87.431 1 96.183
ENSMUSG00000072541 Gm5046 pseudogene 1 63.126 1
94.48 1 84.724 1 94.904
ENSMUSG00000072553 Gm525 protein_coding 0.682 24.344 1.116
96.68 1.311 48.451 0.907 92.748
ENSMUSG00000072568 Fam84b protein_coding 1.007 71.516
1.089 93.27 0.911 40.19 0.848 83.724
ENSMUSG00000072571 Tmem253 protein_coding 0.829 48.146
1.043 96.268 1.16 47.512 1.03 96.192
ENSMUSG00000072572 Slc39a2 protein_coding 0.746 3.513 1.119
87.609 1.196 21.462 1.122 72.826
ENSMUSG00000072573 Gm10369 protein_coding 1.518 62.689
0.585 55.265 1.122 55.109 0.999 95.74
ENSMUSG00000072574 Gm17184 pseudogene 1 81.52 1 94.367
1 87.971 1 93.964
ENSMUSG00000072582 Ptrh2 protein_coding 0.905 15.912 0.936
91.361 1.176 11.665 1.104 54.153
ENSMUSG00000072584 Gm7489 protein_coding 1 82.732 1

97.263 1 82.74 1 95.273
ENSMUSG00000072591 5930412G12Rik processed_transcript 0.731
5.946 1.162 79.367 1.175 32.524 1.021 96.086
ENSMUSG00000072594 Gm16439 pseudogene 1 68.846 1
96.948 1 86.084 1 95.832
ENSMUSG00000072595 4930503E14Rik protein_coding 1 80.751
1 95.708 1 85.921 1 93.584
ENSMUSG00000072596 Ear2 protein_coding 1.271 28.927 0.816
42.923 0.965 74.686 1.019 91.807
ENSMUSG00000072598 Ang6 protein_coding 1 86.333 1
96.407 1 84.961 1 94.82
ENSMUSG00000072599 Ear-ps2 pseudogene 1 86.658 1
96.138 1 85.792 1 93.117
ENSMUSG00000072600 Ear-ps9 pseudogene 1 67.163 1
96.68 1 81.976 1 95.832
ENSMUSG00000072605 Gm10376 protein_coding 1 85.499 1
95.708 1 81.032 1 95.273
ENSMUSG00000072612 Gm10382 protein_coding 0.82 34.954 1.1
96.407 1.112 53.449 0.943 92.742
ENSMUSG00000072613 1700049E17Rik2 protein_coding 1 84.397
1 96.268 1 74.179 1 94.99
ENSMUSG00000072616 Gm7853 protein_coding 1 84.819 NA
NA 1 84.669 1 92.12
ENSMUSG00000072618 Gm10384 protein_coding 1.293 53.562 0.98
86.071 0.789 72.269 1.101 88.197
ENSMUSG00000072620 Slfn2 protein_coding 1.562 0 0.966 90.046
0.664 2.889 1.095 70.36
ENSMUSG00000072621 Slfn10-ps pseudogene 0.889 83.418 1.076
95.735 1.046 86.985 1.232 95.676
ENSMUSG00000072623 Zfp9 protein_coding 1.066 37.702 1.131
84.007 0.827 15.63 1.06 76.046
ENSMUSG00000072624 Gm5460 protein_coding 1.306 0.984 0.874
51.277 0.875 44.678 0.818 86.991
ENSMUSG00000072640 Lyrm9 protein_coding 0.993 77.113 0.904
89.996 1.114 48.935 0.926 86.699
ENSMUSG00000072641 Cux2 pseudogene 1 80.251 1 95.708
NA NA 1 95.273
ENSMUSG00000072647 Adam1a protein_coding 0.767 3.895 1.359
74.663 0.955 84.896 1.08 83.581
ENSMUSG00000072653 Zfp783 pseudogene 1.089 42.555 0.979
92.631 0.939 53.7 0.936 85.493
ENSMUSG00000072660 Gm6288 antisense 0.883 33.002 1.144
89.494 0.988 76.225 0.904 85.493
ENSMUSG00000072662 Mansc4 protein_coding 0.807 66.897
0.755 92.355 1.642 69.002 1.667 85.577
ENSMUSG00000072663 Spef2 protein_coding 1.48 39.851 0.574
53.434 1.174 43.307 0.765 91.372
ENSMUSG00000072664 Ugt3a1 protein_coding 1 83.333 1
96.843 1 80.381 1 96.071
ENSMUSG00000072672 Duxbl3 protein_coding 1 68.955 NA
NA 1 84.669 1 96.139
ENSMUSG00000072673 Gm10392 protein_coding 0.811 7.58 0.946
88.881 1.302 11.782 0.956 90.611
ENSMUSG00000072674 Plac9b protein_coding 1 75.671 1

95.431 1 70.214 1 94.468
ENSMUSG00000072675 Duxbl2 protein_coding 1 80.608 1
96.948 NA NA 1 96.358
ENSMUSG00000072676 Tmem254a protein_coding 1 83.333 1
94.848 NA NA 1 95.593
ENSMUSG00000072677 Gm10396 sense_intronic 1 73.401 1
92.137 1 85.58 1 95.72
ENSMUSG00000072679 D6Ertd474e lincRNA 2.231 61.725 0.521
95.088 0.863 85.675 0.867 95.877
ENSMUSG00000072680 Tmem254c protein_coding 1 73.292 1
92.545 1 86.985 1 95.877
ENSMUSG00000072683 Gm7457 processed_transcript 1 72.985
NA NA 1 86.985 1 94.825
ENSMUSG00000072686 Gm10398 lincRNA 1 85.832 1
91.275 1 84.961 1 96.358
ENSMUSG00000072688 Gm10399 protein_coding 1.065 72.985
0.832 85.075 1.126 44.787 0.511 87.992
ENSMUSG00000072692 Gm13826 pseudogene 1.116 61.725 0.921
95.504 0.969 84.434 0.981 91.105
ENSMUSG00000072693 Gm10401 protein_coding 0.747 30.637
0.919 96.138 1.457 34.285 0.804 84.29
ENSMUSG00000072694 1500011B03Rik protein_coding 0.979 51.089
0.921 84.017 1.112 36.839 0.954 90.805
ENSMUSG00000072704 2700089E24Rik protein_coding 1.005 79.853
0.961 90.256 1.029 74.082 1.05 55.841
ENSMUSG00000072707 Olfr31 protein_coding 1 81.01 1
95.809 1 83.925 1 95.35
ENSMUSG00000072708 Olfr381 protein_coding 1 75.858 NA
NA 1 86.831 1 95.35
ENSMUSG00000072709 Olfr380 protein_coding 1 85.59 1
95.504 1 84.896 1 95.877
ENSMUSG00000072714 Rpl21-ps4 protein_coding 1 86.843 1
93.817 1 88.349 1 95.74
ENSMUSG00000072717 Klra11-ps pseudogene 1 76.396 1
96.68 1 88.693 1 92.675
ENSMUSG00000072718 Klra10 protein_coding 0.544 71.36 1.351
96.216 1.355 85.352 0.928 92.018
ENSMUSG00000072720 Myo18b protein_coding 5.437 10.858
1.242 83.104 0.148 0.72 0.858 95.92
ENSMUSG00000072721 Klra14-ps pseudogene 0.586 38.286 1.622
88.823 1.054 84.626 1.313 83.067
ENSMUSG00000072722 Gm6588 protein_coding 1 73.401 1
97.155 1 87.336 1 89.892
ENSMUSG00000072723 Gm10044 processed_transcript 0.763 73.585
0.745 67.739 1.759 25.425 1.413 68.354
ENSMUSG00000072726 Gm5797 protein_coding 1 63.804 1
96.847 1 79.524 1 95.72
ENSMUSG00000072731 Gm3715 pseudogene 1 79.93 1 95.735
NA NA 1 93.413
ENSMUSG00000072738 Gm16440 protein_coding 1 81.465 1
90.454 1 87.744 1 92.762
ENSMUSG00000072739 Gm10408 protein_coding 1 82.231 NA
NA 1 83.397 1 96.287
ENSMUSG00000072753 9230020A06Rik lincRNA 1 50.377 1

87.919	NA	NA	1	95.832
ENSMUSG00000072754	Lrcol1	protein_coding	1	79.577	1
95.708	1	80.089	1	95.877
ENSMUSG00000072761	Gm6712	protein_coding	1.107	51.737
1.011	95.329	0.894	44.865	1.121	95.273
ENSMUSG00000072762	4930522L14Rik	protein_coding	1.255	1.846
1.066	95.504	0.748	14.206	0.882	84.29
ENSMUSG00000072763	5430403G16Rik	protein_coding	0.861	85.59
1.182	95.735	0.984	77.037	0.973	94.001
ENSMUSG00000072768	Gm10418	pseudogene	0.917	51.56	1.054	90.952
1.038	76.225	0.763	83.724
ENSMUSG00000072769	Gm10419	protein_coding	0.942	74.021
1.049	95.196	1.009	88.203	0.729	83.724
ENSMUSG00000072770	Acrbp	protein_coding	0.804	2.174	1.271	82.904
0.973	79.119	0.932	84.19
ENSMUSG00000072772	Grcc10	protein_coding	0.905	16.183
0.946	85.116	1.164	28.997	0.994	96.248
ENSMUSG00000072774	Zfp951	protein_coding	1.237	14.34	0.995
95.504	0.813	16.719	1.086	96.248
ENSMUSG00000072778	Vmn2r27	protein_coding	1	61.887	1
82.904	1	86.144	1	89.152
ENSMUSG00000072780	Vmn2r24	protein_coding	1	75.399	1
93.765	NA	NA	1	91.928
ENSMUSG00000072789	Gm10420	pseudogene	1	79.165	1
96.216	1	75.809	1	94.825
ENSMUSG00000072791	Abcb5	protein_coding	1	84.865	1
95.301	1	86.084	1	95.273
ENSMUSG00000072809	9330160F10Rik	protein_coding	0.895	81.465
1.078	96.843	NA	NA	0.924	86.699
ENSMUSG00000072812	Ahnak2	protein_coding	1.136	73.841
0.819	91.955	1.071	80.381	0.873	83.724
ENSMUSG00000072813	E330014E10Rik	protein_coding	1	73.292
1	95.504	1	77.888	1	95.273
ENSMUSG00000072814	Gm7982	protein_coding	1	86.199	1
96.268	1	86.475	1	95.676
ENSMUSG00000072821	Gm6351	protein_coding	1	82.543	1
96.268	NA	NA	1	93.734
ENSMUSG00000072822	BC061212	protein_coding	1	78.299	1
95.504	1	87.37	1	93.734
ENSMUSG00000072825	Cep170b	protein_coding	0.924	37.82	0.987
96.013	1.1	42.522	0.912	83.724
ENSMUSG00000072829	NA	NA	NA	NA	1	96.138	1	70.9
1	96.183
ENSMUSG00000072834	Gm12298	antisense	1	81.196	NA	NA
1	70.44	1	93.016
ENSMUSG00000072837	Mir546	miRNA	1	69.777	NA	NA	1
76.768	1	96.183
ENSMUSG00000072844	G530011O06Rik	processed_transcript	1.296
24.689	1.125	85.501	0.683	8.626	0.982	94.016
ENSMUSG00000072845	Tmprss11a	protein_coding	1.547	18.388
2.174	93.146	0.298	14.409	3.691	42.736
ENSMUSG00000072847	A530017D24Rik	lincRNA	0.982	77.615
0.923	82.503	1.099	36.578	0.965	93.685
ENSMUSG00000072852	2310040G07Rik	antisense	0.967	82.352

0.957 96.013 1.077 82.778 0.822 90.674
ENSMUSG00000072855 Gm10430 protein_coding 1 66.944 NA
NA 1 87.971 1 93.264
ENSMUSG00000072872 Rybp protein_coding 1.214 0.2 1.005 95.735
0.821 1.764 1.072 95.273
ENSMUSG00000072874 Gm6116 lincRNA 1 79.277 1
83.321 1 67.823 1 96.086
ENSMUSG00000072875 Gpr27 protein_coding 1.054 65.622 0.889
85.116 1.064 49.898 0.992 90.177
ENSMUSG00000072884 Gm10433 processed_transcript 0.962 86.172
1.109 94.767 0.933 54.521 0.723 84.29
ENSMUSG00000072888 Samt2 protein_coding 1 84.564 1
93.908 1 76.754 1 94.904
ENSMUSG00000072889 Nfxl1 protein_coding 1.181 3.061 0.921 74.483
0.918 33.971 1.084 57.711
ENSMUSG00000072893 4933439C10Rik processed_transcript 0.796
1.175 0.996 94.102 1.265 6.202 0.822 85.493
ENSMUSG00000072894 Ppp1r2-ps2 pseudogene 1 78.428 1
95.708 1 86.727 1 95.676
ENSMUSG00000072898 Gm10434 pseudogene 0.86 62.437 0.948
75.493 1.221 31.479 0.887 86.991
ENSMUSG00000072900 Mir540 miRNA 1 67.334 1 96.407
NA NA 1 88.797
ENSMUSG00000072905 Gm2016 protein_coding 1 57.949 1
91.361 NA NA 1 91.291
ENSMUSG00000072915 Gm12258 protein_coding 0.974 84.397 0.91
87.313 1.123 68.425 1.129 86.445
ENSMUSG00000072917 Gm1965 protein_coding 1 83.09 1
95.708 1 86.985 1 96.248
ENSMUSG00000072918 1700022K14Rik antisense 0.823 50.768
0.814 82.073 1.491 36.95 1.001 86.991
ENSMUSG00000072919 Noxred1 protein_coding 0.858 71.763
1.409 79.261 0.828 22.735 0.958 95.559
ENSMUSG00000072923 Gm10439 protein_coding 1 82.479 1
95.088 1 88.601 1 96.183
ENSMUSG00000072929 Gm15109 pseudogene 1 85.59 1 96.652
1 86.258 1 93.964
ENSMUSG00000072930 Gm15107 protein_coding 1 73.585 1
96.68 1 83.397 1 95.559
ENSMUSG00000072931 Gm15080 protein_coding 1 79.165 1
94.48 1 82.575 1 93.132
ENSMUSG00000072941 Sod3 protein_coding 0.96 85.637 1.08
79.055 0.961 55.214 1.121 94.567
ENSMUSG00000072944 Nup62cl protein_coding 1.485 61.171
1.112 95.196 0.607 63.504 0.776 92.181
ENSMUSG00000072946 Ptgr2 protein_coding 0.976 45.894 0.979
95.301 1.048 36.95 1 93.627
ENSMUSG00000072949 Acot1 protein_coding 0.853 54.983 0.677
25.341 1.737 6.202 0.964 93.156
ENSMUSG00000072950 Gm10444 pseudogene 1 58.709 1
91.532 1 84.626 1 95.273
ENSMUSG00000072960 BC065397 protein_coding 0.805 49.749 0.99
87.919 1.255 47.31 0.768 86.991
ENSMUSG00000072962 Gm16401 protein_coding 1.056 75.671 NA

NA	0.876	73.874	0.603	83.724
ENSMUSG00000072963	Gm10447	protein_coding	0.587	39.174
0.888	90.053	1.914	24.791	1.145	96.192
ENSMUSG00000072964	Bhlhb9	protein_coding	0.93	65.869
0.937	85.418	1.141	23.41	0.993	86.301
ENSMUSG00000072966	Gprasp2	protein_coding	1.133	42.134
0.872	84.594	1.011	87.898	0.896	86.991
ENSMUSG00000072968	Gm17728	protein_coding	1	81.196	1
96.268	1	84.308	1	95.273
ENSMUSG00000072969	Armcx5	protein_coding	1.054	72.655
0.972	85.418	0.971	81.492	1	96.358
ENSMUSG00000072972	Adam4	protein_coding	1.292	55.462	1.12
85.756	0.692	15.376	0.948	96.248
ENSMUSG00000072974	Gm4787	protein_coding	1.225	59.527
0.843	87.041	0.966	85.21	0.891	95.832
ENSMUSG00000072978	Gm5830	protein_coding	1	76.396	1
95.504	1	78.954	1	94.654
ENSMUSG00000072980	Oip5	protein_coding	1.222	32.411	1.198
96.847	0.684	27.321	0.994	92.018
ENSMUSG00000072982	9230009I02Rik	protein_coding	1	81.925
1	95.211	1	86.985	1	96.358
ENSMUSG00000072983	4933414I15Rik	protein_coding	1	70.36	NA
NA	1	86.666	1	95.676
ENSMUSG00000072995	Cpxcr1	protein_coding	1	84.049	1
95.431	1	85.757	1	95.877
ENSMUSG00000072999	Gm15401	protein_coding	0.684	11.726
1.124	94.767	1.296	34.07	1.011	87.447
ENSMUSG00000073000	Gm10451	protein_coding	0.954	64.284
1.447	83.104	0.721	73.598	1.058	95.273
ENSMUSG00000073001	Cylc1	protein_coding	1	81.178	1
96.308	1	70.214	1	93.584
ENSMUSG00000073002	Vamp5	protein_coding	0.894	76.968	1.094
88.823	1.022	83.811	1.062	95.72
ENSMUSG00000073006	Gm732	protein_coding	1	80.51	1	96.216
1	88.076	1	95.74
ENSMUSG00000073008	Gpr174	protein_coding	1.036	81.925
1.413	83.104	0.68	54.521	1.133	92.762
ENSMUSG00000073010	Gm5127	protein_coding	1.203	81.76	0.75
95.708	1.11	88.601	1.131	82.091
ENSMUSG00000073012	Fnd3c2	protein_coding	1	82.479	1
95.329	1	87.274	1	87.992
ENSMUSG00000073016	Uprt	protein_coding	0.926	41.021	0.962
94.597	1.118	52.432	1.008	92.12
ENSMUSG00000073018	Gm1070	protein_coding	1.511	59.527
0.671	95.504	0.987	71.734	1.188	90.463
ENSMUSG00000073027	Dmrtc1b	protein_coding	1	74.745	1
96.897	1	88.203	1	96.192
ENSMUSG00000073028	Igkv4-71	protein_coding	1	80.815	NA
NA	1	85.134	1	93.016
ENSMUSG00000073043	Atoh1	protein_coding	0.969	85.129	1.318
92.931	0.781	80.015	0.926	93.612
ENSMUSG00000073045	Gm5570	lincRNA	1	80.51	NA	NA	1
84.552	1	96.287
ENSMUSG00000073051	Gm14812	lincRNA	1.435	17.336	1.285

83.104 0.54 1.898 1.083 96.183
ENSMUSG00000073057 C530030P08Rik pseudogene 1 61.887 1
96.013 1 67.163 1 96.086
ENSMUSG00000073058 Gm10463 protein_coding 1.111 51.189
0.773 84.594 1.166 81.976 0.849 96.287
ENSMUSG00000073062 Zxdb protein_coding 1.043 56.085 0.936
88.691 1.024 83.275 1.003 90.674
ENSMUSG00000073067 9130019P16Rik processed_transcript 1.181
79.068 0.881 95.504 0.96 59.726 0.709 83.724
ENSMUSG00000073069 Mageb2 protein_coding 1 79.165 1
96.847 NA NA 1 94.825
ENSMUSG00000073072 G930045G22Rik protein_coding 1 79.068
1 91.83 1 69.196 1 96.192
ENSMUSG00000073073 Gm8098 processed_transcript 0.848 49.944
0.91 95.088 1.3 62.995 1.035 89.152
ENSMUSG00000073077 Gm7173 protein_coding 0.223 23.923
2.733 83.104 1.64 59.274 1.233 95.593
ENSMUSG00000073079 Srp54a protein_coding 1.01 54.344
1.001 88.897 0.986 85.921 0.894 83.724
ENSMUSG00000073085 4930428D18Rik protein_coding 1 80.608
1 93.654 1 85.21 1 91.625
ENSMUSG00000073090 Gm7932 protein_coding 0.495 64.284
2.207 75.62 0.917 76.768 3.599 50.74
ENSMUSG00000073096 Lrrc61 protein_coding 0.782 13.581
0.987 88.705 1.289 17.412 1.024 89.498
ENSMUSG00000073101 5730522E02Rik protein_coding 1 64.014
NA NA 1 88.601 1 92.675
ENSMUSG00000073102 Drc1 protein_coding 1.027 64.401 0.976
93.908 0.994 84.071 0.938 84.29
ENSMUSG00000073103 Gm10466 lincRNA 1 81.465 1
95.211 1 84.961 1 95.72
ENSMUSG00000073110 Olfr444 protein_coding 1 80.51 1
94.102 1 85.935 1 94.608
ENSMUSG00000073111 Olfr446 protein_coding 1 85.77 1
92.355 1 74.686 1 95.273
ENSMUSG00000073116 Gm10471 protein_coding 1 78.428 1
95.708 1 79.776 1 92.762
ENSMUSG00000073117 NA NA NA NA 1 95.504 1
78.045 1 93.734
ENSMUSG00000073119 Speer4a protein_coding 1 66.944 1
95.301 1 87.686 1 96.183
ENSMUSG00000073125 Xlr3b protein_coding 1.089 79.709 1.193
87.282 0.77 68.584 1.118 92.748
ENSMUSG00000073130 Gm1141 protein_coding 1.049 85.283
0.915 97.155 1.039 81.62 0.971 96.183
ENSMUSG00000073131 Vma21 protein_coding 0.974 81.465 0.991
93.094 1.038 67.823 1.034 71.305
ENSMUSG00000073139 BC023829 protein_coding 0.964 61.334
1.105 71.222 0.936 23.938 1.061 90.865
ENSMUSG00000073141 4930567H17Rik protein_coding 1 85.203
NA NA 1 82.436 1 94.567
ENSMUSG00000073144 4930599N23Rik antisense 1.137 40.065
0.917 71.889 0.962 88.274 0.904 86.991
ENSMUSG00000073147 5031425E22Rik protein_coding 1.027 81.465

1.006 95.301 0.968 81.976 0.874 83.724
ENSMUSG00000073152 Gm10475 antisense 0.901 83.169 0.856
90.138 1.295 70.9 0.995 96.183
ENSMUSG00000073154 9330158H04Rik processed_transcript 1
85.391 1 95.088 1 84.071 1 92.263
ENSMUSG00000073155 1810058I24Rik lincRNA 0.934 18.379
1.109 71.222 0.965 53.7 1.036 88.197
ENSMUSG00000073158 9030624G23Rik protein_coding 0.886 78.909
1.209 97.129 0.934 79.625 1.251 59.464
ENSMUSG00000073164 2410018L13Rik lincRNA 0.961 85.203
0.679 79.261 1.532 48.304 1.223 92.31
ENSMUSG00000073173 Gm10477 protein_coding 1 78.299 1
94.597 1 88.657 1 95.273
ENSMUSG00000073174 Magi2 protein_coding 1.207 81.178 0.598
87.041 1.386 85.757 0.927 95.92
ENSMUSG00000073176 Zfp449 protein_coding 1.209 18.556
0.966 94.597 0.856 30.024 1.144 46.412
ENSMUSG00000073177 Gm773 protein_coding 1 78.299 1
95.504 1 73.309 1 92.748
ENSMUSG00000073179 Gm10478 pseudogene 1.23 35.748 0.921
96.68 0.882 22.279 0.958 86.991
ENSMUSG00000073184 Gm10479 protein_coding 1 84.564 NA
NA 1 88.729 1 93.612
ENSMUSG00000073197 5730507C01Rik protein_coding 1.167 72.27
0.894 84.017 0.958 84.961 1.191 92.691
ENSMUSG00000073207 Ccdc160 protein_coding 0.749 9.081 0.817
50.664 1.636 5.864 0.807 86.445
ENSMUSG00000073209 Klf14 protein_coding 1.184 50.111 1.253
88.092 0.675 23.86 1.122 82.091
ENSMUSG00000073226 Gm10482 protein_coding 1 81.465 1
95.708 1 88.203 1 96.287
ENSMUSG00000073231 Gm10483 protein_coding 1 81.01 1
96.843 1 87.103 1 93.156
ENSMUSG00000073233 Gm9144 pseudogene 1 68.727 1
97.263 1 88.161 1 94.904
ENSMUSG00000073234 Gm8773 protein_coding 0.55 34.795
1.619 83.464 1.125 81.857 1.697 38.624
ENSMUSG00000073236 2500004C02Rik processed_transcript 0.995
86.112 1.039 93.899 0.963 73.874 0.897 83.724
ENSMUSG00000073245 Gm14819 protein_coding 1 80.688 1
96.268 1 84.434 1 96.183
ENSMUSG00000073247 Gm10486 protein_coding 1 81.76 1
97.155 1 84.961 1 96.248
ENSMUSG00000073255 Gm14632 protein_coding 1 83.169 1
96.308 1 81.976 1 94.99
ENSMUSG00000073257 Gm10488 protein_coding 1 84.977 NA
NA 1 87.786 1 93.964
ENSMUSG00000073267 Gm1993 protein_coding 1 84.977 1
96.268 NA NA 1 92.018
ENSMUSG00000073274 Gm14636 processed_transcript 0.694 78.299
1.085 91.532 1.332 86.831 1.485 77.245
ENSMUSG00000073293 Nudt10 protein_coding 0.767 77.763
2.137 85.756 0.608 65.902 0.901 96.086
ENSMUSG00000073294 AU022751 protein_coding 1 85.59 1

96.652	NA	NA	1	95.273
ENSMUSG00000073295	Nudt11	protein_coding	0.896	77.113
1.437	93.994	0.776	88.349	1.125	83.581
ENSMUSG00000073367	Gm10493	pseudogene	1	83.169	1
86.728	1	78.369	1	95.877
ENSMUSG00000073371	Gm6594	protein_coding	1	82.092	NA
NA	1	84.724	1	96.287
ENSMUSG00000073374	C030034I22Rik	protein_coding	1.007	81.657
1.009	96.68	0.986	64.778	0.899	85.493
ENSMUSG00000073377	AU016765	protein_coding	1	85.857	1
96.013	1	79.554	1	95.92
ENSMUSG00000073380	Arrdc5	protein_coding	1	83.726	NA
NA	1	60.5	1	94.904
ENSMUSG00000073388	A330017A19Rik	protein_coding	0.794	61.887
NA	NA	1.251	54.809	1.078	96.248
ENSMUSG00000073394	Gm10497	antisense	0.798	19.023	1.136
82.904	1.102	84.961	0.996	95.273
ENSMUSG00000073396	Gm6084	protein_coding	1	71.763	1
97.155	1	80.899	1	96.358
ENSMUSG00000073399	Trim40	protein_coding	1	82.231	1
90.046	1	87.744	1	94.567
ENSMUSG00000073400	Trim10	protein_coding	1.328	69.408	0.9
96.013	0.837	81.62	1.302	94.904
ENSMUSG00000073402	Gm8909	protein_coding	1.338	82.705	0.98
92.404	0.762	84.626	0.703	92.181
ENSMUSG00000073403	Gm10499	pseudogene	1.148	84.977	1.08
89.494	0.807	27.994	0.888	96.183
ENSMUSG00000073405	C920025E04Rik	pseudogene	1.457	1.412	0.703
24.911	0.977	65.409	1.213	60.31
ENSMUSG00000073406	H2-Bl	polymorphic_pseudogene	0.772	42.922
0.498	82.073	2.595	17.279	1.072	91.011
ENSMUSG00000073407	Gm6034	protein_coding	0.954	84.865	NA
NA	1.195	88.693	1.152	96.183
ENSMUSG00000073408	Dpcr1	protein_coding	0.814	75.115	1.08
96.013	1.132	79.625	0.906	96.139
ENSMUSG00000073409	H2-Q6	protein_coding	0.949	82.231	1.078
83.819	0.974	58.775	0.878	83.724
ENSMUSG00000073411	H2-D1	protein_coding	0.97	48.354	0.945
94.848	1.092	30.948	0.922	84.19
ENSMUSG00000073412	Lst1	protein_coding	1.042	33.555	1.321
85.756	0.724	17.662	0.974	94.001
ENSMUSG00000073413	Ly6g6d	protein_coding	0.871	66.129
0.834	75.4	1.376	19.609	0.8	84.29
ENSMUSG00000073414	AU023871	protein_coding	1.003	71.896
1.057	82.904	0.938	21.331	0.709	83.724
ENSMUSG00000073415	Gm10501	antisense	0.824	14.403	1.11
96.897	1.095	46.575	1.152	61.984
ENSMUSG00000073418	C4b	protein_coding	1.427	0	0.806	55.71
0.871	19.765	1.047	62.594
ENSMUSG00000073421	H2-Ab1	protein_coding	0.965	75.761
1.214	93.899	0.853	87.744	0.753	83.724
ENSMUSG00000073423	Zfp414	protein_coding	0.882	17.705
1.054	79.411	1.078	64.305	0.955	90.157
ENSMUSG00000073424	Cyp4f15	protein_coding	1.525	14.966

0.826 90.053 0.792 26.057 0.728 95.832
ENSMUSG00000073427 Gm4924 protein_coding 1.182 16.959
1.255 97.155 0.674 31.135 1.008 95.72
ENSMUSG00000073429 C130040N14Rik protein_coding 0.902 56.242
1.057 80.806 1.048 86.346 0.953 84.29
ENSMUSG00000073430 Gm10505 lincRNA 0.167 22.92 1.609 89.434
3.714 57.286 3.498 80.573
ENSMUSG00000073433 Arhgdig protein_coding 0.881 71.674
0.986 85.242 1.153 27.431 1.106 93.156
ENSMUSG00000073434 Wdr90 protein_coding 1.003 84.168 0.972
91.661 1.023 84.626 1.005 94.654
ENSMUSG00000073435 Nme3 protein_coding 1 84.738 1
94.367 1 88.729 1 94.904
ENSMUSG00000073436 Eme2 protein_coding 0.953 24.748 1.085
82.904 0.967 83.066 0.98 96.183
ENSMUSG00000073437 D330041H03Rik processed_transcript 0.963
23.923 0.936 87.998 1.105 13.956 0.866 86.699
ENSMUSG00000073448 Gm10509 protein_coding 1.34 16.74 1.115
96.843 0.67 10.003 0.884 87.735
ENSMUSG00000073455 9030025P20Rik protein_coding 0.885 42.922
0.937 78.471 1.207 9.301 0.971 95.273
ENSMUSG00000073457 NA NA NA NA 1 96.216 1
73.531 1 90.95
ENSMUSG00000073458 Smok2a protein_coding 1 85.129 1
95.211 1 81.492 1 96.287
ENSMUSG00000073460 Pnldc1 protein_coding 1.01 81.01 1.218
92.111 0.81 71.999 1.005 93.964
ENSMUSG00000073468 Sft2d1 protein_coding 0.975 79.853 0.99
95.708 1.037 87.898 1.014 94.82
ENSMUSG00000073471 Rsph3a protein_coding 0.813 4.874 1.242
60.309 0.993 87.519 0.973 83.724
ENSMUSG00000073478 D730003I15Rik processed_transcript 1.009
86.918 1.04 96.843 0.954 72.464 1.254 60.968
ENSMUSG00000073481 2-Mar protein_coding 0.862 1.991 1.015 93.654
1.143 10.615 NA NA
ENSMUSG00000073482 Gm10517 protein_coding 0.57 48.036 2.08
88.91 0.846 81.976 0.98 95.74
ENSMUSG00000073485 Gm16067 antisense 0.808 26.483 0.85
60.806 1.454 7.24 1.06 93.261
ENSMUSG00000073486 Gm10518 protein_coding 0.765 36.034
1.125 92.253 1.164 66.647 0.936 86.991
ENSMUSG00000073489 Ifi204 protein_coding 1.316 0.577 0.873
81.731 0.872 34.21 1.229 40.127
ENSMUSG00000073490 AI607873 protein_coding 1.534 10.19 0.824
62.71 0.792 50.184 1.09 78.042
ENSMUSG00000073492 Gm10521 protein_coding 1.059 59.211
0.638 27.551 1.474 22.131 1.323 66.068
ENSMUSG00000073494 Sh2d1b2 protein_coding 1 82.982 NA
NA 1 85.921 1 88.505
ENSMUSG00000073497 AA792892 protein_coding 1 81.01 1
95.088 1 80.749 1 94.825
ENSMUSG00000073510 Gm7647 protein_coding 1 76.546 1
90.29 1 76.498 1 91.985
ENSMUSG00000073514 Dok6 protein_coding 0.955 53.679 0.893

95.211 1.174 40.875 1.082 93.261
ENSMUSG00000073528 Gm10530 protein_coding 1 74.689 1
93.899 1 72.695 1 96.358
ENSMUSG00000073529 F830208F22Rik processed_transcript 0.935
74.482 NA NA 1.012 88.203 1.211 87.134
ENSMUSG00000073530 Pappa2 protein_coding 1.635 8.281 0.701
92.914 0.87 33.137 0.76 86.699
ENSMUSG00000073535 Gm5532 lincRNA 1.196 46.533 0.997
96.407 0.839 54.25 0.834 84.29
ENSMUSG00000073538 E330020D12Rik protein_coding 2.265 0.461
0.775 85.671 0.57 8.907 0.98 73.597
ENSMUSG00000073542 Cep76 protein_coding 1.037 69.408 NA NA
0.937 72.269 1.043 71.653
ENSMUSG00000073554 Gm6960 pseudogene 1 68.955 1
95.504 1 88.41 1 89.428
ENSMUSG00000073555 Gm4951 protein_coding 1.361 3.932 0.962
94.102 0.764 11.751 0.911 86.699
ENSMUSG00000073557 Ppp1r12b protein_coding 1.087 29.596
1.018 96.897 0.903 23.48 1.015 93.117
ENSMUSG00000073559 Gm8618 pseudogene 1 82.231 1
94.848 NA NA 1 95.877
ENSMUSG00000073563 Csnk1g3 protein_coding 0.958 71.674 NA
NA 0.985 85.921 0.998 91.625
ENSMUSG00000073565 Prr16 protein_coding 0.821 21.957 1.2
79.411 1.017 77.532 1.242 46.846
ENSMUSG00000073568 Arl14epl protein_coding 2.838 13.492
0.736 83.392 0.478 66.201 0.763 85.493
ENSMUSG00000073569 Gm10540 pseudogene 1 83.968 1
92.404 1 87.744 1 96.358
ENSMUSG00000073572 Gm10542 protein_coding 1 84.865 1
93.654 1 87.37 1 94.384
ENSMUSG00000073573 Spink11 protein_coding 1 85.637 1
95.708 1 86.666 1 94.904
ENSMUSG00000073574 Grxcr2 protein_coding 1 83.726 NA
NA 1 86.602 1 94.654
ENSMUSG00000073576 9630014M24Rik protein_coding 1 83.169
1 96.138 1 86.475 1 93.156
ENSMUSG00000073586 Gm10543 pseudogene 1 83.333 1
94.102 1 69.196 1 94.174
ENSMUSG00000073590 3222401L13Rik protein_coding 1.192 55.124
0.918 90.116 0.91 41.662 0.802 91.078
ENSMUSG00000073591 Pcdhb22 protein_coding 1.038 73.734 1.1
82.904 0.874 26.057 0.996 96.358
ENSMUSG00000073594 Gm10545 protein_coding 1 78.224 1
93.654 1 80.015 1 92.564
ENSMUSG00000073596 2900060B14Rik protein_coding 1 72.85 1
96.843 1 55.701 1 94.001
ENSMUSG00000073598 1700066B19Rik protein_coding 1.148 82.982
1.025 96.013 0.853 74.686 1.11 96.287
ENSMUSG00000073599 Ecscr protein_coding 0.921 60.707 1.094
92.111 0.994 86.258 1.017 86.301
ENSMUSG00000073600 Prob1 protein_coding 1.326 32.873 0.905
88.704 0.834 49.733 0.775 91.078
ENSMUSG00000073601 Serpinb3c protein_coding 1 75.671 1

87.919 1 80.381 1 83.067
ENSMUSG00000073602 Serpinb3b protein_coding 1 70.121 1
95.708 1 87.173 1 95.74
ENSMUSG00000073607 Gm10548 pseudogene 1.01 84.397 0.664
62.692 1.488 22.517 0.954 96.183
ENSMUSG00000073608 Gm6086 protein_coding 0.73 72.139
1.195 95.708 1.149 71.839 0.865 86.991
ENSMUSG00000073609 D2hgdh protein_coding 0.938 30.352
1.049 96.68 1.017 47.512 0.969 92.181
ENSMUSG00000073616 Myeov2 protein_coding 0.911 10.29 0.955
85.242 1.147 10.819 1.064 83.276
ENSMUSG00000073617 Hdac4 protein_coding 1.085 80.688 0.905
93.654 1.015 85.792 1.09 50.452
ENSMUSG00000073627 C130036L24Rik processed_transcript 0.754
17.062 1.167 85.756 1.139 62.648 0.827 87.735
ENSMUSG00000073628 Gm10552 antisense 1.108 69.089 NA NA
0.559 33.402 0.546 85.493
ENSMUSG00000073631 Gm10553 protein_coding 1 84.865 1
97.155 1 88.41 1 91.217
ENSMUSG00000073633 Fbxo36 protein_coding 1.084 74.482
0.859 83.58 1.069 80.089 0.842 83.724
ENSMUSG00000073639 Rab18 protein_coding 1.04 31.477 0.949
67.165 1.014 82.74 1.022 67.691
ENSMUSG00000073643 Wdfy1 protein_coding 0.992 85.499 0.936
84.594 1.072 50.851 1.032 94.752
ENSMUSG00000073647 Gm10557 pseudogene 0.923 68.955 1.119
96.407 0.968 88.461 0.985 96.183
ENSMUSG00000073650 Gm216 protein_coding 1.19 42.555 0.858
93.046 0.981 77.294 0.868 95.405
ENSMUSG00000073652 Apol7d protein_coding 0.732 18.907 NA
NA 1.366 26.978 0.884 90.611
ENSMUSG00000073658 Crygb protein_coding 1 84.324 1
94.262 1 85.935 1 94.654
ENSMUSG00000073664 Nbeal1 protein_coding 0.917 10.143
1.007 94.89 1.085 35.441 0.958 85.493
ENSMUSG00000073672 Rnf223 pseudogene 1.752 10.595 1.409
82.904 0.405 4.963 1.646 93.964
ENSMUSG00000073676 Hspe1 protein_coding 0.847 30.877 1.053
95.809 1.122 26.663 1.077 93.132
ENSMUSG00000073678 Pgap1 protein_coding 0.893 35.449 0.899
90.378 1.242 36.388 1.077 92.181
ENSMUSG00000073679 Gm10562 antisense 0.841 48.646 1.055
96.897 1.128 72.695 0.934 88.387
ENSMUSG00000073680 Tmem88b protein_coding 1.161 25.421
0.939 91.532 0.921 38.434 0.944 86.699
ENSMUSG00000073682 Gm10563 protein_coding 1.012 78.909
1.027 92.931 0.964 79.255 1.178 91.807
ENSMUSG00000073684 2610002J02Rik protein_coding 0.912 34.795
0.997 96.216 1.097 28.055 1.005 85.493
ENSMUSG00000073686 Gm10564 protein_coding 1 74.482 1
85.075 1 66.647 NA NA
ENSMUSG00000073699 Gm833 lincRNA 1 81.925 1 95.708
1 88.693 1 93.261
ENSMUSG00000073700 Klhl21 protein_coding 1.058 43.654 0.95

68.818 0.992 81.492 0.978 88.245
ENSMUSG00000073702 Rpl31 protein_coding 1.017 66.272 0.941
60.678 1.042 38.975 0.973 91.269
ENSMUSG00000073705 Apitd1 protein_coding 1.157 57.655 0.84
86.277 1.028 83.857 1.072 83.067
ENSMUSG00000073716 Gm13241 pseudogene 0.63 34.954 1.19
87.282 1.335 63.665 1.223 96.248
ENSMUSG00000073719 Vps13d protein_coding 1 83.263 1
96.407 NA NA 1 93.156
ENSMUSG00000073721 Gm13125 protein_coding 1 72.372 1
96.68 1 83.925 1 93.413
ENSMUSG00000073723 Gm13102 protein_coding 1 75.671 1
93.654 1 73.027 1 96.086
ENSMUSG00000073724 Pramel4 protein_coding 1 84.738 1
95.708 1 85.935 1 95.273
ENSMUSG00000073725 Lmbrd1 protein_coding 1.089 3.893 0.993
91.532 0.926 6.202 0.965 83.724
ENSMUSG00000073728 Tmem51os1 processed_transcript 0.905 50.229
0.923 81.731 1.197 26.663 0.808 91.105
ENSMUSG00000073731 AI507597 antisense 0.535 19.985 1.239
82.904 1.503 84.504 0.757 83.724
ENSMUSG00000073733 Rsg1 protein_coding 0.992 54.176 1.349
78.235 0.748 59.125 1.075 93.722
ENSMUSG00000073735 Defb18 protein_coding 1 86.658 1
94.367 1 84.552 1 94.567
ENSMUSG00000073737 Gm10566 pseudogene 1 77.763 1
96.216 1 84.961 1 88.797
ENSMUSG00000073738 Gm10567 protein_coding 1 83.169 1
94.848 1 81.857 1 95.593
ENSMUSG00000073739 Gm16287 antisense 1.567 31.477 0.62
78.877 1.026 86.475 0.777 87.496
ENSMUSG00000073741 4732440D04Rik protein_coding 1.052 72.743
1.013 95.35 0.939 76.498 0.606 85.493
ENSMUSG00000073747 Gm7534 protein_coding 1 78.224 1
94.96 1 88.657 1 96.358
ENSMUSG00000073752 Gm10570 protein_coding 0.312 75.567
0.496 89.996 6.459 30.948 2.817 92.52
ENSMUSG00000073753 Gm10571 pseudogene 0.539 15.456 1.906
84.832 0.975 48.451 0.887 89.428
ENSMUSG00000073755 5730409E04Rik protein_coding 1.047 41.454
0.965 80.052 0.992 79.897 1.014 56.079
ENSMUSG00000073758 Sh3d21 protein_coding 0.818 7.574 0.885
83.58 1.387 7.143 1.012 93.261
ENSMUSG00000073759 4933407E24Rik lincRNA 1 81.76 1
93.654 1 77.294 1 95.593
ENSMUSG00000073764 Gm12888 protein_coding 3.814 0.984 0.27
68.115 0.967 44.019 0.907 84.29
ENSMUSG00000073768 Olfr1330 protein_coding 1 82.982 1
95.211 1 87.103 1 89.498
ENSMUSG00000073769 Olfr1331 protein_coding 1 82.092 1
96.68 1 86.985 1 94.654
ENSMUSG00000073770 Olfr1333 protein_coding 1 72.985 1
95.735 1 88.693 1 95.273
ENSMUSG00000073771 Btbd19 protein_coding 0.998 71.36 1.008

94.597 0.99 61.841 1.063 58.721
ENSMUSG00000073775 Kti12 protein_coding 0.984 82.092 NA NA
1.016 87.519 0.999 96.192
ENSMUSG00000073778 Gm10574 antisense 0.796 8.405 1.074 95.708
1.172 17.751 0.879 87.651
ENSMUSG00000073779 B230314M03Rik protein_coding 1 71.915
NA NA 1 86.144 1 86.301
ENSMUSG00000073781 Gm6471 protein_coding 1 86.145 1
95.329 1 87.898 1 94.904
ENSMUSG00000073785 Krtap5-5 protein_coding 1 82.092 1
91.661 1 81.388 1 96.071
ENSMUSG00000073786 Gm7579 protein_coding 1 82.732 1
97.155 1 82.778 1 96.183
ENSMUSG00000073787 Gm10575 processed_transcript 0.763 5.778
1.057 96.763 1.243 24.383 0.9 86.991
ENSMUSG00000073788 Gm10576 protein_coding 1 86.333 1
96.407 NA NA 1 94.904
ENSMUSG00000073789 Gm10577 protein_coding 1 77.182 NA
NA 1 86.602 1 94.904
ENSMUSG00000073791 Efcab7 protein_coding 1.036 80.03 0.886
72.576 1.092 47.512 1.088 94.904
ENSMUSG00000073792 Alg6 protein_coding 1.106 20.296 0.919
79.807 0.985 79.255 0.982 94.174
ENSMUSG00000073794 I0C0044D17Rik protein_coding 1 79.93 1
95.708 1 69.315 1 92.181
ENSMUSG00000073795 6430531B16Rik protein_coding 1.12 64.193
0.772 72.399 1.157 68.129 0.751 86.991
ENSMUSG00000073797 Gm12650 pseudogene 1 83.726 1
97.049 1 77.532 1 96.358
ENSMUSG00000073802 Cdkn2b protein_coding 0.793 22.92 1.276
83.104 0.989 67.823 0.9 92.181
ENSMUSG00000073803 4930544L04Rik protein_coding 1 77.113
1 95.504 1 81.169 1 93.132
ENSMUSG00000073804 Nps protein_coding 0.671 61.887 0.629
91.83 2.356 47.776 1.575 87.447
ENSMUSG00000073805 Fam196a protein_coding 0.74 32.749
1.354 82.76 0.996 88.41 0.641 85.493
ENSMUSG00000073809 Gm10583 pseudogene 1 79.165 1
95.196 1 88.274 1 88.797
ENSMUSG00000073811 Ifna12 protein_coding 1 84.865 NA
NA 1 84.83 1 95.593
ENSMUSG00000073821 8030451A03Rik processed_transcript 0.852
74.021 NA NA 1.158 86.985 0.956 95.273
ENSMUSG00000073830 Mup14 protein_coding 1.164 86.658 0.716
95.088 NA NA 0.564 84.29
ENSMUSG00000073835 Mup-ps12 pseudogene 1 85.391 1
96.407 1 85.21 1 94.904
ENSMUSG00000073838 Tufm protein_coding 0.879 0.987 1.046 79.367
1.083 55.109 1.074 76.831
ENSMUSG00000073840 Mup-ps2 pseudogene 1 82.479 1
97.155 1 86.112 1 94.825
ENSMUSG00000073842 Mup7 protein_coding 0.32 0.987 1.039 94.262
3.012 5.864 1.193 90.455
ENSMUSG00000073844 Gm21957 protein_coding 1 81.823 NA

NA 1 84.308 1 88.797
ENSMUSG00000073854 AI427809 protein_coding 1.493 4.228 1.028
93.654 0.649 30.827 1.266 72.616
ENSMUSG00000073856 Iqck protein_coding 0.842 15.76 1.058 84.999
1.119 43.481 0.922 83.724
ENSMUSG00000073860 Gm829 lincRNA 1 71.36 1 95.708 1
60.233 1 94.904
ENSMUSG00000073862 Gm5600 protein_coding 1 86.843 1
96.68 1 87.786 1 96.086
ENSMUSG00000073866 B430319F04Rik protein_coding 1.153 72.372
1.216 89.996 0.711 38.534 0.874 87.331
ENSMUSG00000073867 AA474408 protein_coding 1.381 70.741
0.873 93.046 0.826 80.015 0.867 92.018
ENSMUSG00000073876 Gm13305 protein_coding 1 76.827 1
96.843 1 85.792 1 78.948
ENSMUSG00000073877 Gm13306 protein_coding 1 76.686 1
97.263 1 60.789 1 96.184
ENSMUSG00000073878 Gm13304 protein_coding 1 81.465 1
96.216 NA NA 1 95.35
ENSMUSG00000073879 Gm5859 pseudogene 1 83.333 1
95.708 1 81.857 1 95.74
ENSMUSG00000073888 Ccl27a protein_coding 0.835 20.116
1.073 91.065 1.112 40.575 1.025 92.742
ENSMUSG00000073889 Il11ra1 protein_coding 1.073 21.703 1.05
83.104 0.888 1.764 1.18 0
ENSMUSG00000073893 Olfr472 protein_coding 1 80.608 1
96.763 1 80.015 1 94.654
ENSMUSG00000073894 Rbmxl2 protein_coding 1 83.169 1
91.584 NA NA 1 96.287
ENSMUSG00000073896 Olfr716 protein_coding 1 81.196 1
97.263 1 88.274 1 96.358
ENSMUSG00000073897 Olfr17 protein_coding 1 86.918 1
94.848 1 79.325 1 95.559
ENSMUSG00000073898 Olfr713 protein_coding 1 86.74 1
96.407 1 84.308 1 96.071
ENSMUSG00000073899 Olfr1532-ps1 protein_coding 1 80.751
1 93.817 1 88.693 1 95.72
ENSMUSG00000073900 Olfr704 protein_coding 1 76.396 1
97.155 1 80.899 1 95.593
ENSMUSG00000073901 Olfr703 protein_coding 1 79.277 1
96.843 1 75.809 1 95.72
ENSMUSG00000073906 Olfr692 protein_coding 1 81.24 1
96.138 1 88.601 1 96.287
ENSMUSG00000073907 Olfr689 protein_coding 1 85.637 1
94.89 1 75.009 1 94.153
ENSMUSG00000073908 Olfr687 protein_coding 1 74.021 NA
NA 1 84.724 1 95.273
ENSMUSG00000073909 Olfr688 protein_coding 1 73.401 1
91.83 1 84.724 1 94.608
ENSMUSG00000073910 Mob3b protein_coding 1.186 13.105 1.004
88.533 0.838 7.329 0.907 83.952
ENSMUSG00000073913 Olfr678 protein_coding 1 73.292 NA
NA 1 84.896 1 91.625
ENSMUSG00000073914 Olfr677 protein_coding 1 81.925 1

						96.68	1	78.954	1	93.722
ENSMUSG00000073915	Olfr676	protein_coding	1	85.857	1	96.001	1	79.426	1	95.273
ENSMUSG00000073916	Olfr669	protein_coding	1	84.263	NA	NA	1	87.336	1	92.389
ENSMUSG00000073917	Olfr665	protein_coding	1	84.819	1	96.847	1	84.504	1	95.74
ENSMUSG00000073919	Olfr663	pseudogene	1	81.196	1	89.713	1	84.308	1	96.248
ENSMUSG00000073920	Olfr661	protein_coding	1	84.738	1	96.268	1	88.693	1	95.877
ENSMUSG00000073922	Olfr659	protein_coding	1	81.01	1	95.431	1	83.538	1	96.248
ENSMUSG00000073923	Olfr657	protein_coding	1	86.57	1	95.708	1	84.071	1	96.183
ENSMUSG00000073924	Olfr656	protein_coding	1	78.299	NA	NA	1	80.089	1	94.904
ENSMUSG00000073925	Olfr654	protein_coding	1	82.231	1	96.378	1	80.5	1	96.358
ENSMUSG00000073926	Olfr653	protein_coding	1	68.727	1	96.68	1	84.071	1	93.685
ENSMUSG00000073927	Olfr652	protein_coding	1	82.092	NA	NA	1	69.315	1	94.567
ENSMUSG00000073928	Olfr651	protein_coding	1	60.435	1	92.355	1	88.693	1	95.92
ENSMUSG00000073929	Trim30e-ps1	pseudogene	1.812	75.399	0.543	65.819	1.015	42.522	0.802	93.722
ENSMUSG00000073931	Olfr646	protein_coding	1	86.464	1	94.848	1	88.693	1	96.358
ENSMUSG00000073932	Olfr641	protein_coding	1	86.145	1	91.661	1	86.666	1	95.777
ENSMUSG00000073937	Olfr633	protein_coding	1	85.355	1	96.216	NA	NA	1	95.74
ENSMUSG00000073938	Olfr632	protein_coding	1	82.982	1	95.708	1	73.309	1	94.82
ENSMUSG00000073940	Hbb-bt	protein_coding	1.085	61.725	0.769	64.123	1.201	58.051	1.218	87.7
ENSMUSG00000073943	Olfr625-ps1	protein_coding	1	85.728	1	92.545	NA	NA	1	93.685
ENSMUSG00000073944	Olfr619	protein_coding	1	79.277	1	95.504	1	86.144	1	94.654
ENSMUSG00000073945	Olfr618	protein_coding	1	83.169	1	96.268	1	86.475	1	93.316
ENSMUSG00000073946	Olfr617	protein_coding	1	81.52	NA	NA	1	88.41	1	96.071
ENSMUSG00000073947	Olfr615	protein_coding	1	85.391	1	95.735	1	79.524	1	95.72
ENSMUSG00000073948	Olfr608	protein_coding	1	85.637	1	96.948	1	83.275	1	93.584
ENSMUSG00000073949	Olfr606	protein_coding	1	70.121	NA	NA	1	74.686	1	95.832
ENSMUSG00000073950	Olfr599	protein_coding	1	78.054	1	95.708	1	87.274	1	96.042
ENSMUSG00000073951	Olfr598	protein_coding	1	83.658	NA					

NA 1 85.58 1 95.273
ENSMUSG00000073952 Olfr597 protein_coding 1 84.865 1
97.263 1 84.308 1 93.612
ENSMUSG00000073953 Olfr596 protein_coding 1 83.09 1
97.049 1 88.274 1 95.559
ENSMUSG00000073954 Olfr594 protein_coding 1 82.732 1
95.301 NA NA 1 95.273
ENSMUSG00000073955 Olfr593 protein_coding 1 86.333 1
95.301 1 88.601 1 95.877
ENSMUSG00000073956 Olfr592 protein_coding 1 82.982 1
96.843 1 84.724 1 94.752
ENSMUSG00000073959 Olfr584 protein_coding 1 80.408 1
97.263 1 88.693 1 93.316
ENSMUSG00000073960 Olfr583 protein_coding 1 81.24 1
96.847 1 83.607 1 88.552
ENSMUSG00000073961 Olfr582 protein_coding 1 74.147 NA
NA 1 88.203 1 94.384
ENSMUSG00000073962 Olfr576 protein_coding 1 80.815 1
95.301 1 87.898 1 94.904
ENSMUSG00000073963 Olfr572 protein_coding 1 75.858 NA
NA 1 79.426 1 96.358
ENSMUSG00000073964 Olfr570 protein_coding 1 84.263 NA
NA 1 86.602 1 96.183
ENSMUSG00000073965 Olfr568 protein_coding 1 83.523 1
95.504 NA NA 1 92.12
ENSMUSG00000073966 Olfr561 protein_coding 1 72.85 1
95.329 1 79.776 1 93.132
ENSMUSG00000073967 Olfr557 protein_coding 1 74.8 1
95.329 1 86.144 1 95.35
ENSMUSG00000073968 Trim68 protein_coding 0.847 0.987 1.093
89.494 1.083 6.202 0.928 87.992
ENSMUSG00000073969 Olfr556 protein_coding 1 82.092 1
93.046 1 86.144 1 94.752
ENSMUSG00000073970 Olfr555 protein_coding 1 84.738 1
95.809 NA NA 1 92.748
ENSMUSG00000073971 Olfr554 protein_coding 1 81.01 1
95.504 1 85.58 1 93.316
ENSMUSG00000073972 Olfr553 protein_coding 1 83.418 1
97.155 1 86.144 1 95.72
ENSMUSG00000073973 Olfr552 protein_coding 1 74.8 1
96.68 1 86.112 1 95.832
ENSMUSG00000073974 Olfr551 protein_coding 1 84.294 1
93.469 1 75.809 1 94.904
ENSMUSG00000073976 1700026J12Rik lincRNA 1 68.485 NA
NA 1 84.308 1 95.593
ENSMUSG00000073977 Olfr549 protein_coding 1 77.407 NA
NA NA NA 1 95.74
ENSMUSG00000073978 Olfr548-ps1 protein_coding 1 85.728 1
96.138 1 88.41 1 95.676
ENSMUSG00000073979 Olfr547 protein_coding 1 83.823 1
95.301 1 79.325 1 92.018
ENSMUSG00000073980 Gm9574 pseudogene 0.902 62.158 0.932
96.216 1.19 68.425 0.951 91.525
ENSMUSG00000073982 Rhog protein_coding 1.152 1.643 1.021 95.504

0.846 6.935 0.977 96.183
ENSMUSG00000073985 Gm10602 pseudogene 0.921 24 1.112 84.768
0.977 83.275 0.853 86.991
ENSMUSG00000073987 Ggh protein_coding 1.094 18.388 0.91
84.594 1.007 74.179 1.013 90.611
ENSMUSG00000073988 Ttpa protein_coding 0.65 4.099 1.095 95.809
1.407 11.151 1.085 91.872
ENSMUSG00000073991 Cnbd1 protein_coding 1 83.169 1
96.407 NA NA 1 91.525
ENSMUSG00000073995 Gm10604 protein_coding 0.595 84.534 2.11
83.104 0.796 63.919 1.197 96.071
ENSMUSG00000073997 Olfr521 protein_coding 1 84.819 1
95.504 1 79.897 1 95.74
ENSMUSG00000073998 Olfr520 protein_coding 0.68 63.283 1.4
93.146 1.051 86.985 0.514 83.724
ENSMUSG00000074001 Klhl40 protein_coding 4.001 7.083 6.131
83.104 0.041 3.969 0.569 94.654
ENSMUSG00000074003 Gucy2d protein_coding 0.736 83.333
2.677 80.678 0.506 59.125 1.205 96.139
ENSMUSG00000074004 B3gnt6 protein_coding 1.892 19.746
0.712 81.731 0.741 71.388 0.775 92.31
ENSMUSG00000074006 Omp protein_coding 2.089 1.441 0.581 64.123
0.823 52.658 0.962 91.807
ENSMUSG00000074011 Gm7682 protein_coding 1 81.178 1
95.708 1 85.921 1 94.99
ENSMUSG00000074024 4632427E13Rik antisense 0.997 82.982
0.894 87.147 1.124 81.857 0.967 87.496
ENSMUSG00000074026 Gm6091 pseudogene 1 84.397 1
97.263 NA NA 1 93.584
ENSMUSG00000074029 Gm10608 protein_coding 0.62 47.677
1.356 93.707 1.191 57.907 0.727 93.584
ENSMUSG00000074030 Exoc8 protein_coding 0.961 65.388 1.113
82.219 0.937 16.129 0.986 94.174
ENSMUSG00000074034 Gm5921 pseudogene 1 85.832 1
95.301 1 81.857 1 95.74
ENSMUSG00000074037 Mc1r protein_coding 1 58.38 1 90.053
1 86.831 1 93.156
ENSMUSG00000074039 4930520O04Rik processed_transcript 0.802
53.407 1.401 84.768 0.886 87.971 0.892 85.493
ENSMUSG00000074056 Gm10615 protein_coding 0.943 76.686 0.99
96.308 1.068 82.778 0.985 95.72
ENSMUSG00000074059 Fbxw18 protein_coding 1 80.03 NA NA
1 84.896 1 95.877
ENSMUSG00000074060 Fbxw15 protein_coding 1 85.857 1
91.584 NA NA 1 94.82
ENSMUSG00000074061 Fbxw19 protein_coding 1 86.658 1
97.097 1 81.388 1 96.184
ENSMUSG00000074062 Fbxw16 protein_coding 1 80.608 1
96.308 1 84.961 1 95.273
ENSMUSG00000074063 Osgin1 protein_coding 0.792 21.957
0.964 95.708 1.313 28.301 1.262 62.918
ENSMUSG00000074064 Mlycd protein_coding 0.972 64.284 0.974
90.378 1.056 61.418 0.931 86.699
ENSMUSG00000074065 Gm10617 pseudogene 0.601 59.028 2.35

75.612 0.709 73.598 1.245 93.734
ENSMUSG00000074067 Gm10619 protein_coding 1 82.231 NA
NA 1 84.896 1 94.016
ENSMUSG00000074071 Fam169b lincRNA 0.862 37.82 1.182 80.806
0.981 80.5 0.847 92.181
ENSMUSG00000074072 Gm10620 pseudogene 1 84.977 1
96.013 1 84.896 1 96.287
ENSMUSG00000074075 Gm10621 protein_coding 0.894 65.869
0.993 95.504 1.129 54.163 0.823 83.724
ENSMUSG00000074088 Snrnp40 protein_coding 1.041 13.492 0.97
73.766 0.99 63.075 1.042 91.525
ENSMUSG00000074092 Gm6181 pseudogene 1 71.142 1
95.431 1 75.809 1 91.872
ENSMUSG00000074093 Svip protein_coding 0.887 3.325 1.04 77.085
1.085 41.6 1.077 82.85
ENSMUSG00000074102 Rbm15b protein_coding 1.02 72 1.124
85.756 0.87 21.331 0.996 91.291
ENSMUSG00000074109 Mrgprx2 protein_coding 1.684 3.598 0.706
68.115 0.84 49.217 1.026 94.654
ENSMUSG00000074111 Mrgpra9 protein_coding 1 86.351 1
96.268 1 84.896 1 94.608
ENSMUSG00000074115 Saa1 protein_coding 5.62 16.436 0.263
68.818 0.674 84.552 1.203 96.192
ENSMUSG00000074121 Ntf5 protein_coding 0.915 50.519 1.043
89.494 1.049 74.325 0.93 85.493
ENSMUSG00000074123 7420426K07Rik protein_coding 1 69.985
1 95.211 1 88.601 1 94.904
ENSMUSG00000074127 Cmtm2a protein_coding 1 85.283 1
95.211 1 87.274 1 90.865
ENSMUSG00000074128 Gm10631 antisense 1 85.499 1
96.407 1 84.896 1 94.904
ENSMUSG00000074129 Rpl13a protein_coding 0.946 66.129
0.981 83.58 1.075 36.011 1.055 80.77
ENSMUSG00000074136 4930513N10Rik processed_transcript 0.889
36.034 1.089 95.088 1.036 49.601 0.935 86.991
ENSMUSG00000074139 1700057G04Rik protein_coding 1 81.76 NA
NA 1 86.985 1 91.525
ENSMUSG00000074140 Zfp319 protein_coding 1.02 63.385
1.061 92.561 0.919 29.217 0.959 85.493
ENSMUSG00000074141 Il4i1 protein_coding 0.905 20.799 0.998
97.155 1.104 28.979 0.787 83.724
ENSMUSG00000074146 4930579C12Rik protein_coding 1 74.147
1 95.088 1 85.464 1 95.676
ENSMUSG00000074149 Gm10634 protein_coding 1 80.688 NA
NA 1 86.475 1 96.287
ENSMUSG00000074151 Nlrc5 protein_coding 1.011 78.428 1.162
60.309 0.853 3.969 0.974 86.445
ENSMUSG00000074155 Klk5 protein_coding 1 85.728 1
95.211 NA NA 1 95.273
ENSMUSG00000074156 Ces1h protein_coding 1 72.743 NA NA
1 87.686 1 94.654
ENSMUSG00000074158 9830147E19Rik protein_coding 1.113 40.88 1.02
90.952 0.881 17.662 1.002 93.722
ENSMUSG00000074164 Vmn2r-ps54 pseudogene 1 51.56 1 96.843

1 80.749 1 93.045
ENSMUSG00000074165 Zfp788 protein_coding 1.074 24.253 0.94
65.165 NA NA 1.095 92.018
ENSMUSG00000074166 AW146154 protein_coding 1.171 6.135 0.872
60.678 0.98 71.999 0.907 85.493
ENSMUSG00000074170 Plekhf1 protein_coding 1.159 42.134
0.901 70.29 0.952 78.954 0.982 90.674
ENSMUSG00000074171 Gm6658 protein_coding 0.943 64.284
1.258 96.984 0.844 87.686 0.806 84.29
ENSMUSG00000074178 Gm10638 protein_coding 0.861 53.679
1.258 83.104 0.925 86.727 0.846 84.29
ENSMUSG00000074179 NA NA NA NA 1 96.138 1
83.066 1 83.067
ENSMUSG00000074182 Znhit6 protein_coding 1.039 32.676
0.892 41.268 1.075 57.227 1.265 48.857
ENSMUSG00000074183 Gsta1 protein_coding 1 85.832 1
93.654 1 82.575 1 91.525
ENSMUSG00000074184 Smt3h2-ps2 pseudogene 1 82.231 1
91.361 1 81.492 1 92.381
ENSMUSG00000074194 Zfp791 protein_coding 0.924 51.447 0.82
67.739 1.321 24.691 0.679 87.651
ENSMUSG00000074195 AI747448 protein_coding 1 86.006 1
90.053 1 84.724 1 92.762
ENSMUSG00000074196 Gm6289 protein_coding 1 86.658 1
95.708 1 86.985 1 93.156
ENSMUSG00000074199 Krtdap protein_coding 0.978 75.298 0.98
95.504 1.044 83.719 0.758 89.475
ENSMUSG00000074203 G430095P16Rik protein_coding 0.944 54.983
1.184 83.104 0.895 73.874 0.847 86.699
ENSMUSG00000074206 Adh6b protein_coding 1.126 80.608 1.109
93.529 0.802 67.648 0.967 89.72
ENSMUSG00000074207 Adh1 protein_coding 0.825 24 0.941 94.597
1.29 20.968 1.196 48.119
ENSMUSG00000074210 E130208F15Rik protein_coding 0.739 8.709
1.134 83.104 1.196 38.26 0.984 90.854
ENSMUSG00000074211 Sdhaf1 protein_coding 0.997 38.286
1.026 91.456 0.976 82.436 0.966 94.297
ENSMUSG00000074212 Dnajb14 protein_coding 0.966 15.76 0.986
96.268 1.053 30.561 0.931 83.724
ENSMUSG00000074213 Gm10642 protein_coding 0.63 14.086
1.306 82.904 1.212 40.575 1.057 96.183
ENSMUSG00000074215 Gm10643 protein_coding 0.729 21.957
1.059 96.407 1.296 44.361 1.207 60.31
ENSMUSG00000074217 2210011C24Rik protein_coding 0.91 66.652
0.656 82.947 1.676 20.315 0.824 96.248
ENSMUSG00000074218 Cox7a1 protein_coding 0.643 30.136
1.512 82.904 1.027 73.309 1.374 87.886
ENSMUSG00000074219 Gm10644 protein_coding 1 74.689 1.02
95.809 0.982 88.601 0.864 84.19
ENSMUSG00000074220 Zfp382 protein_coding 0.99 86.74 0.937
95.301 1.08 83.397 1.116 77.245
ENSMUSG00000074221 Zfp568 protein_coding 1.063 73.401
1.029 82.904 0.915 23.607 1.048 94.904
ENSMUSG00000074224 4932431P20Rik protein_coding 0.39 73.292

0.963 95.211 2.657 65.235 2.459 83.067
ENSMUSG00000074227 Spint2 protein_coding 0.899 41.021
1.017 91.361 1.095 23.454 0.938 86.445
ENSMUSG00000074228 Gm10645 protein_coding 1.04 76.686
0.982 91.361 0.981 50.091 0.737 85.493
ENSMUSG00000074232 Gm10647 protein_coding 0.688 39.712
0.911 68.818 1.597 10.819 1.318 85.577
ENSMUSG00000074235 Gm10649 processed_transcript 1.071 67.163
0.58 73.166 1.607 44.125 1.09 93.685
ENSMUSG00000074236 Gm9387 pseudogene 1 86.57 1 96.843
1 85.352 1 94.904
ENSMUSG00000074237 Gm10650 protein_coding 1 77.615 NA
NA 1 74.082 1 96.183
ENSMUSG00000074238 Ap1ar protein_coding 1.028 38.195 1.001
92.253 0.975 29.765 0.961 83.724
ENSMUSG00000074247 Dda1 protein_coding 1.001 63.736 1.084
61.943 0.922 8.634 1.075 83.276
ENSMUSG00000074248 NA NA NA NA 1 96.268 NA NA
1 96.184
ENSMUSG00000074250 Gm10653 pseudogene 1.097 74.516 0.914
85.921 NA NA 0.746 83.952
ENSMUSG00000074252 Gm10654 protein_coding 0.368 14.645
1.103 96.843 2.456 33.287 1.784 83.067
ENSMUSG00000074254 Cyp2a4 protein_coding 1 82.479 1
92.404 1 84.308 1 94.016
ENSMUSG00000074256 Gm10655 protein_coding 0.486 45.728
3.343 82.904 0.616 83.607 0.981 87.7
ENSMUSG00000074259 Gramd2 protein_coding 0.556 19.595
0.864 87.452 2.086 5.964 1.518 71.653
ENSMUSG00000074261 Gm7092 protein_coding 0.835 86.57 1.749
90.895 0.684 62.058 1.402 94.82
ENSMUSG00000074264 Amy1 protein_coding 1.126 86.464 0.88
85.116 1.011 42.522 0.93 92.675
ENSMUSG00000074268 Amy2a5 protein_coding 1 83.823 1
94.887 1 88.203 1 95.92
ENSMUSG00000074269 2410076I21Rik protein_coding 0.727 27.202
1.317 82.904 1.042 85.757 1.066 93.964
ENSMUSG00000074272 Ceacam1 protein_coding 1.002 84.738
1.016 96.138 0.981 88.693 1.061 74.607
ENSMUSG00000074273 Gm10657 protein_coding 0.724 42.034
1.072 97.049 1.288 62.995 1.167 90.674
ENSMUSG00000074277 Phldb3 protein_coding 0.757 6.952 0.972
95.504 1.355 15.049 0.829 84.29
ENSMUSG00000074281 Gm12522 processed_transcript 0.529 2.142
0.961 97.155 1.971 13.174 0.915 90.611
ENSMUSG00000074282 Zfp94 protein_coding 0.939 64.589 0.962
85.116 1.104 35.894 1.112 93.156
ENSMUSG00000074283 Zfp109 protein_coding 0.896 45.894
1.154 85.756 0.967 85.352 1.052 94.82
ENSMUSG00000074284 Gm10658 processed_transcript 0.824 29.725
0.98 95.196 1.236 54.809 1 95.92
ENSMUSG00000074289 Vmn1r173 protein_coding 1 82.479 1
96.897 1 85.921 1 93.627
ENSMUSG00000074291 Vmn1r168 protein_coding 1 81.125 1

95.211 1 81.29 1 96.183
ENSMUSG00000074292 Gm10660 lincRNA 1 80.408 1
95.211 1 88.274 1 95.74
ENSMUSG00000074300 BC030870 protein_coding 0.877 84.049
0.802 60.678 1.418 47.935 0.932 86.699
ENSMUSG00000074302 Gm10663 protein_coding 1 85.129 1
95.735 1 87.658 1 94.215
ENSMUSG00000074305 C230081A13Rik protein_coding 1.029 34.077
1.026 93.574 0.943 23.212 1.088 91.872
ENSMUSG00000074311 Vmn1r139 protein_coding 1 85.655 NA
NA 1 79.426 1 96.183
ENSMUSG00000074322 Vmn1r132 protein_coding 1 86.57 1
96.268 1 86.552 1 95.273
ENSMUSG00000074340 Ovgp1 protein_coding 0.821 10.143 0.963
81.443 1.268 8.339 1 92.675
ENSMUSG00000074342 I830077J02Rik protein_coding 1.479 2.723
0.902 90.378 0.747 14.788 1.074 78.042
ENSMUSG00000074343 1700104A03Rik antisense 1 71.896 NA
NA 1 85.757 1 94.654
ENSMUSG00000074345 Tnfaip8l3 protein_coding 1.412 7.083 0.884
84.017 0.804 28.237 1.165 92.52
ENSMUSG00000074354 9230115E21Rik antisense 0.72 18.602
1.651 74.663 0.839 66.201 0.904 94.904
ENSMUSG00000074355 Gm10676 protein_coding 0.937 27.836
1.143 78.235 0.936 87.898 1.036 88.657
ENSMUSG00000074357 AA386476 protein_coding 0.627 3.763 1.084
96.216 1.466 17.662 1.142 60.968
ENSMUSG00000074358 Ccdc61 protein_coding 0.907 21.658
1.052 93.574 1.044 51.977 0.967 86.699
ENSMUSG00000074359 Psg23 protein_coding 0.884 78.054 0.972
96.216 1.159 67.962 1.066 93.612
ENSMUSG00000074361 C5ar2 protein_coding 1.254 30.788 0.922
85.671 0.864 63.919 0.909 86.991
ENSMUSG00000074364 Ehd2 protein_coding 0.85 1.867 1.153 62.898
1.022 73.759 0.994 91.335
ENSMUSG00000074365 Crxos1 protein_coding 1 83.333 1
95.088 1 87.971 1 92.181
ENSMUSG00000074366 Obox5 protein_coding 1 79.93 1 97.288
1 77.17 1 95.74
ENSMUSG00000074369 Obox2 protein_coding 1 75.115 1
96.652 1 71.839 1 95.92
ENSMUSG00000074373 Gm10680 antisense 1 85.832 1
95.211 1 81.388 1 93.413
ENSMUSG00000074375 Sult2a3 protein_coding 1 81.01 NA NA
1 86.985 1 96.358
ENSMUSG00000074377 Sult2a4 protein_coding 1 77.916 1
95.329 1 78.369 1 95.273
ENSMUSG00000074378 Bsph1 protein_coding 1 81.657 NA NA
NA NA 1 95.832
ENSMUSG00000074384 AI429214 protein_coding 0.829 21.957
1.043 94.367 1.159 45.667 1.04 92.762
ENSMUSG00000074385 Gm10684 protein_coding 0.682 31.333 1.47
88.91 0.997 69.825 1.079 96.287
ENSMUSG00000074398 Gm15441 processed_transcript 0.65 11.232

0.876 35.072 1.752 5.864 0.794 83.724
ENSMUSG00000074401 Vmn1r59 protein_coding 1 84.661 NA
NA NA NA 1 92.561
ENSMUSG00000074402 Vmn1r55 protein_coding 1 84.397 1
95.735 1 88.693 1 96.071
ENSMUSG00000074404 Gm1698 lincRNA 1 84.263 1
85.501 1 76.768 1 90.854
ENSMUSG00000074405 Zfp865 protein_coding 0.898 7.574 1.047
82.904 1.062 50.636 0.962 83.724
ENSMUSG00000074406 Zfp628 protein_coding 0.892 38.983
1.045 89.996 1.071 81.032 0.986 92.748
ENSMUSG00000074414 Gm10691 protein_coding 1.436 51.189
0.958 93.654 0.723 62.533 0.859 93.584
ENSMUSG00000074415 2610203C20Rik processed_transcript 1.021
67.608 1.119 96.847 0.876 69.974 0.908 93.316
ENSMUSG00000074417 Gm14548 protein_coding 1.516 8.537 0.847
88.897 0.78 32.963 1.358 69.708
ENSMUSG00000074418 Gm15930 pseudogene 1 86.843 1
96.013 NA NA 1 93.156
ENSMUSG00000074419 Gm15448 protein_coding 1.334 41.177
0.535 69.611 1.4 70.623 1.07 91.525
ENSMUSG00000074424 Gm10696 protein_coding 1 80.251 1
96.138 1 84.669 1 95.777
ENSMUSG00000074429 Gm9116 protein_coding 1 84.049 NA
NA 1 84.504 1 95.877
ENSMUSG00000074433 Lce3e protein_coding 1 70.36 1 93.908
1 88.601 1 94.904
ENSMUSG00000074434 Gm6696 protein_coding 1 85.637 1
95.211 1 88.076 1 95.832
ENSMUSG00000074435 Smcp protein_coding 1 81.465 1
96.68 1 80.089 1 96.287
ENSMUSG00000074436 Defa-ps18 pseudogene 1 83.823 1
97.263 NA NA 1 96.071
ENSMUSG00000074437 Defa-rs1 protein_coding 1 81.125 NA
NA 1 85.921 1 94.153
ENSMUSG00000074439 Defa5 protein_coding 1 79.577 1
92.914 1 85.757 1 95.92
ENSMUSG00000074440 Defa3 protein_coding 1 68.955 1
95.504 1 84.308 1 95.273
ENSMUSG00000074441 Gm15292 protein_coding 1 63.385 1
96.216 1 84.504 1 96.183
ENSMUSG00000074442 Defa-rs7 protein_coding 1 82.352 1
96.138 1 81.976 1 94.016
ENSMUSG00000074443 Defa22 protein_coding 1 85.59 NA NA
1 87.658 1 96.287
ENSMUSG00000074444 Gm15284 protein_coding 1 84.564 1
95.431 1 79.625 1 93.156
ENSMUSG00000074445 Sprr2a3 protein_coding 3.912 56.907 0.23
88.704 1.114 78.713 1.409 93.413
ENSMUSG00000074446 Defa23 protein_coding 1 80.03 1
96.138 NA NA 1 96.071
ENSMUSG00000074447 Defa21 protein_coding 1 84.397 1
95.504 1 88.693 1 96.071
ENSMUSG00000074448 Gm5615 protein_coding 1 72.372 1

91.661 1 81.388 1 96.192
ENSMUSG00000074449 Gm15319 protein_coding 1 48.939 1
96.843 1 71.615 1 95.405
ENSMUSG00000074452 Pate2 protein_coding 0.684 15.06 NA NA 1.43
31.4 0.92 93.156
ENSMUSG00000074454 Defb33 protein_coding 1 85.728 1
92.631 1 88.203 1 96.183
ENSMUSG00000074457 S100a16 protein_coding 0.909 74.689
1.014 97.155 1.082 68.425 1.149 68.696
ENSMUSG00000074461 Gm10699 protein_coding 1.166 44.334
1.016 94.48 0.842 67.962 1.092 95.832
ENSMUSG00000074465 Gm10701 antisense 1 79.277 1
94.102 1 76.64 1 94.001
ENSMUSG00000074466 Gm15417 antisense 0.886 15.912 1.032
93.146 1.096 45.208 0.948 83.724
ENSMUSG00000074467 Gm10702 pseudogene 0.817 31.614 0.881
94.597 1.39 24.029 0.89 87.182
ENSMUSG00000074472 Zfp872 protein_coding 1.235 21.836
1.262 72.642 0.643 6.858 1.017 87.447
ENSMUSG00000074473 A230072I06Rik protein_coding 1 86.333
1 95.735 1 86.727 1 87.134
ENSMUSG00000074476 Spc24 protein_coding 1.426 13.734 0.813
83.733 0.86 70.791 2.046 57.1
ENSMUSG00000074479 Gm10704 protein_coding 1 66.652 NA
NA 1 70.791 1 96.248
ENSMUSG00000074480 Mex3a protein_coding 0.715 31.614 1.078
96.001 1.293 19.106 1.186 96.183
ENSMUSG00000074482 Gm16589 pseudogene 1 68.955 1
96.407 1 86.144 1 96.086
ENSMUSG00000074483 Bglap protein_coding 1.525 81.925 0.375
96.897 1.746 80.381 0.588 88.505
ENSMUSG00000074485 Gm7381 pseudogene 1 80.251 1
96.68 1 86.666 1 96.248
ENSMUSG00000074486 Bglap2 protein_coding 1 86.145 1
97.155 1 88.729 1 96.042
ENSMUSG00000074489 Bglap3 protein_coding 1.151 31.732
1.141 92.931 0.762 11.314 0.875 84.29
ENSMUSG00000074497 A430078G23Rik protein_coding 0.916 57.227
0.94 79.261 1.156 27.047 1.135 59.394
ENSMUSG00000074500 Zfp558 protein_coding 1.076 83.263
0.944 90.29 0.986 88.161 1.08 90.177
ENSMUSG00000074501 Gm5612 protein_coding 1 77.615 1
94.48 1 87.744 1 96.086
ENSMUSG00000074502 Ubtfl1 protein_coding 1 83.263 1
93.765 1 85.921 1 93.722
ENSMUSG00000074505 Fat3 protein_coding 1.208 58.709 0.82
96.897 1.007 76.388 0.783 85.493
ENSMUSG00000074506 Gm10705 pseudogene 1 81.823 1
96.407 1 85.921 1 94.567
ENSMUSG00000074507 Gm14340 lincRNA 1 78.538 1
96.335 1 88.729 1 92.561
ENSMUSG00000074513 Arfip1 protein_coding 1.055 1.949 0.907
47.727 1.042 86.258 1.043 76.831
ENSMUSG00000074516 Gm10709 protein_coding 0.749 2.174 1.091

91.584 1.225 29.777 1.266 31.133
ENSMUSG00000074517 Gm10710 protein_coding 1.226 39.851
0.861 94.262 0.95 67.648 1.745 94.297
ENSMUSG00000074518 Gm10711 protein_coding 1 82.231 1
93.094 NA NA 1 96.248
ENSMUSG00000074519 Etohi1 protein_coding 1.098 63.804
1.036 95.504 0.882 80.163 1.001 83.581
ENSMUSG00000074521 Gm14327 protein_coding 3.051 15.178 NA
NA 0.455 16.949 0.752 84.29
ENSMUSG00000074527 Gm14296 protein_coding 1 83.09 1
92.404 1 65.8 1 93.132
ENSMUSG00000074529 C330013J21Rik protein_coding 0.769 57.014
0.511 68.818 2.548 19.452 0.529 84.29
ENSMUSG00000074547 Gm10712 pseudogene 1 78.639 1
95.504 1 72.464 1 92.181
ENSMUSG00000074553 Gm10713 pseudogene 1 86.74 NA NA 1
80.899 1 95.832
ENSMUSG00000074555 Gm10714 antisense 1 82.543 1
81.731 1 65.235 1 95.676
ENSMUSG00000074564 Gm10720 protein_coding 1 74.516 1
95.196 1 79.524 1 93.964
ENSMUSG00000074569 Gcnt7 protein_coding 0.835 85.59 0.742 87.998
1.613 59.274 0.351 83.724
ENSMUSG00000074570 Cass4 protein_coding 1.274 20.296 1.137
89.434 0.691 10.726 1.079 80.77
ENSMUSG00000074571 1700101G07Rik protein_coding 1 84.819
1 95.708 NA NA 1 96.183
ENSMUSG00000074575 Kcng1 protein_coding 1.546 38.195 NA NA
0.748 48.577 1.386 92.903
ENSMUSG00000074576 Mocs3 protein_coding 0.945 70.213 1.074
96.407 0.985 84.896 1.035 94.608
ENSMUSG00000074577 Fam65c protein_coding 0.804 54.043
1.191 88.092 1.042 75.155 1.455 31.133
ENSMUSG00000074578 1500012F01Rik protein_coding 1.003 39.295
0.788 0 1.265 5.864 1.043 92.748
ENSMUSG00000074579 Lekr1 protein_coding 1.089 44.061 0.833
67.513 1.101 70.328 0.946 93.685
ENSMUSG00000074580 4931440P22Rik protein_coding 0.841 46.413
1.251 95.431 0.948 65.034 0.821 92.181
ENSMUSG00000074582 Arfgef2 protein_coding 1.001 86.658
0.949 66.651 1.05 35.441 0.975 91.65
ENSMUSG00000074589 4930449A18Rik protein_coding 1 67.867
1 96.216 1 88.076 1 96.183
ENSMUSG00000074592 F830104G03Rik protein_coding 1 84.977
1 94.102 NA NA 1 96.139
ENSMUSG00000074593 Spint5 protein_coding 1 79.433 1
96.843 1 86.985 1 95.273
ENSMUSG00000074594 Wfdc9 protein_coding 1 80.751 NA NA
1 72.695 1 96.071
ENSMUSG00000074595 Wfdc6a protein_coding 1 83.169 1
97.288 1 79.255 1 89.892
ENSMUSG00000074596 Spint3 protein_coding 1 86.172 1
95.329 1 81.29 1 94.904
ENSMUSG00000074602 Gm10728 pseudogene 1 76.042 1

84.594	1	79.897	1	93.316
ENSMUSG00000074604	Mgst2	protein_coding	0.89	71.915	1.007
93.046	1.119	54.363	1.007	85.493
ENSMUSG00000074607	Tox2	protein_coding	0.851	72.743	1.391
67.23	0.846	12.288	0.96	92.181
ENSMUSG00000074608	Zfp850	protein_coding	1.16	66	NA	NA
1.073	73.598	0.869	91.979
ENSMUSG00000074619	1700034I23Rik	pseudogene	1	72.655	1
95.504	1	82.436	1	93.612
ENSMUSG00000074622	Mafb	protein_coding	1.124	17.582	0.923
74.483	0.961	73.027	1.255	49.514
ENSMUSG00000074623	Gm826	protein_coding	1.008	72.546	1.068
87.313	0.925	41.331	0.741	92.748
ENSMUSG00000074625	Arhgap40	protein_coding	0.651	0.987	1.206
75.612	1.271	15.049	0.997	95.74
ENSMUSG00000074627	Mroh8	protein_coding	0.863	39.174	1.018
97.155	1.14	49.898	0.922	96.248
ENSMUSG00000074628	Tldc2	protein_coding	0.622	43.435	1.021
96.407	1.568	84.83	0.963	94.99
ENSMUSG00000074629	4930518I15Rik	protein_coding	1.002	83.169
1.091	82.76	0.916	54.037	1.066	92.31
ENSMUSG00000074634	Gm7120	protein_coding	1.07	85.203	1.14
86.34	0.82	59.383	0.693	83.724
ENSMUSG00000074635	3110070M22Rik	protein_coding	1.031	81.01
1.165	95.088	0.833	75.353	0.833	83.724
ENSMUSG00000074637	Sox2	protein_coding	0.669	34.795	0.971
95.504	1.54	29.777	1.5	60.31
ENSMUSG00000074639	BC089597	protein_coding	1	85.59	1
93.765	1	85.792	1	92.12
ENSMUSG00000074643	Cpne1	protein_coding	0.893	67.867	0.968
95.708	1.158	55.81	1.248	86.445
ENSMUSG00000074646	6430550D23Rik	protein_coding	0.8	16.325
1.077	96.68	1.164	23.668	0.857	86.991
ENSMUSG00000074647	Fam83c	protein_coding	0.803	73.585
0.666	87.147	1.86	44.515	1.084	92.762
ENSMUSG00000074649	BC029722	processed_transcript	0.897	6.409
1.081	82.76	1.029	37.741	0.954	86.445
ENSMUSG00000074650	Gm10735	protein_coding	0.438	2.559	1.323
79.29	1.722	38.26	1.073	96.287
ENSMUSG00000074651	Mcidas	protein_coding	1	67.991	1
95.708	1	88.203	1	92.466
ENSMUSG00000074652	Myh7b	protein_coding	1.047	66.944	1.243
87.037	0.77	75.353	1.293	65.558
ENSMUSG00000074653	Lrrc31	protein_coding	1	82.479	1
88.704	1	66.043	1	91.525
ENSMUSG00000074655	Gm1527	protein_coding	1	81.178	1
95.211	1	88.657	1	95.273
ENSMUSG00000074656	Eif2s2	protein_coding	1.015	40.333
0.948	64.123	1.04	72.853	1.115	44.679
ENSMUSG00000074657	Kif5a	protein_coding	0.811	0.987	1.004	95.211
1.228	5.864	1.001	92.31
ENSMUSG00000074661	Pde4d	protein_coding	1.013	66	0.789	85.116
1.245	82.778	1.218	90.177
ENSMUSG00000074662	Gm10737	pseudogene	1.536	61.725	0.551

78.877 1.18 44.787 0.591 90.611
ENSMUSG00000074671 Tspyl3 protein_coding 1.164 19.746
0.847 72.399 NA NA 0.969 96.287
ENSMUSG00000074673 Ttll9 protein_coding 0.656 62.158 1.784
92.793 0.855 87.519 1.145 93.734
ENSMUSG00000074674 Gm10742 protein_coding 1 84.865 1
92.137 1 75.009 1 95.405
ENSMUSG00000074676 Foxs1 protein_coding 0.741 1.017 0.877 93.654
1.541 5.864 0.848 86.699
ENSMUSG00000074677 Sirpb1c protein_coding 1.589 24.253 NA
NA 0.466 22.009 1.135 60.466
ENSMUSG00000074678 Defb25 protein_coding 2.286 25.207
0.563 85.921 0.779 86.666 0.985 93.156
ENSMUSG00000074679 Defb28 protein_coding 1 85.129 1
92.545 1 87.898 1 76.831
ENSMUSG00000074680 Defb26 protein_coding 1 80.146 NA
NA NA NA 1 94.904
ENSMUSG00000074682 Zcchc3 protein_coding 1.019 71.516 NA
NA 0.937 58.271 0.994 86.991
ENSMUSG00000074695 Il22 protein_coding 1 83.169 1
96.897 1 86.475 1 95.593
ENSMUSG00000074698 Csnk2a1 protein_coding 1.081 0.944 0.986
91.661 0.94 20.712 1.015 60.968
ENSMUSG00000074704 Rad21l protein_coding 1 64.714 1
86.071 1 87.37 1 95.877
ENSMUSG00000074715 Ccl28 protein_coding 0.965 70.213 0.982
97.155 NA NA 0.894 86.699
ENSMUSG00000074733 5830428H23Rik processed_transcript 1.055
13.633 NA NA 0.947 45.093 0.95 92.675
ENSMUSG00000074734 4933416C03Rik protein_coding 1 86.57 1
97.155 1 87.658 1 94.82
ENSMUSG00000074735 Gm21994 protein_coding 1 73.292 NA
NA 1 70.534 1 94.82
ENSMUSG00000074737 C530025M09Rik protein_coding 1 82.543
NA NA 1 86.985 1 96.086
ENSMUSG00000074738 B930041F14Rik protein_coding 1 76.396
1 93.765 1 88.41 1 95.92
ENSMUSG00000074739 Gm10750 protein_coding 1 72.139 1
95.708 1 87.37 1 96.358
ENSMUSG00000074743 Thbd protein_coding 0.925 70.213 1.034
94.367 NA NA 1.21 56.863
ENSMUSG00000074746 Pdzd8 protein_coding 1.058 52.134 0.978
74.851 NA NA 0.988 94.82
ENSMUSG00000074748 Atxn7l3b protein_coding 1.029 15.178
1.046 83.104 0.93 2.189 1.013 79.442
ENSMUSG00000074749 Plk1s1 protein_coding 1.088 40.88 0.88
79.807 NA NA 1.016 96.248
ENSMUSG00000074753 Gm14092 antisense 1 80.251 1
93.094 1 83.857 1 94.001
ENSMUSG00000074754 Gm561 protein_coding 1.047 65.733 0.967
86.728 0.982 85.921 1.015 92.691
ENSMUSG00000074758 Gm5535 lincRNA 2.735 18.556 0.464
87.857 0.787 74.535 1.58 83.067
ENSMUSG00000074763 Gm14105 antisense 1 71.674 1

92.561 1 87.898 1 92.018
ENSMUSG00000074764 Sel1l2 protein_coding 1 72.655 1
78.471 1 73.309 1 96.287
ENSMUSG00000074766 Ism1 protein_coding 0.79 8.302 1.011 96.013
1.248 10.079 1.21 57.075
ENSMUSG00000074771 Ankef1 protein_coding 0.906 43.495
0.906 85.921 1.212 45.94 0.91 86.991
ENSMUSG00000074776 Gm10754 protein_coding 1 73.585 1
96.216 1 70.623 1 96.071
ENSMUSG00000074780 Anapc15-ps protein_coding 1 85.203 1
92.137 1 77.888 1 94.904
ENSMUSG00000074781 Ube2n protein_coding 1.038 21.278 1.005
97.263 0.958 25.052 1.024 59.394
ENSMUSG00000074782 4833422C13Rik antisense 0.873 50.229
1.027 95.809 1.117 69.002 0.71 83.724
ENSMUSG00000074785 Plxnc1 protein_coding 1.358 1.949 0.878
78.51 0.84 23.48 0.865 86.445
ENSMUSG00000074793 Hspa12b protein_coding 0.717 0.987 0.998
83.876 1.403 5.864 0.915 92.018
ENSMUSG00000074794 Arrdc3 protein_coding 1.232 50.913
0.581 62.322 1.399 5.864 1.043 87.7
ENSMUSG00000074796 Slc4a11 protein_coding 0.957 49.944
1.032 95.211 1.015 68.425 1.017 91.807
ENSMUSG00000074797 Itpa protein_coding 0.962 47.677 1.067
87.604 NA NA 1.014 89.052
ENSMUSG00000074798 Gm10760 protein_coding 1.409 86.351 0.5
78.877 1.424 44.361 0.719 89.475
ENSMUSG00000074800 Gm4149 pseudogene 1 85.092 1
95.088 1 86.985 1 87.134
ENSMUSG00000074802 Gas2l3 protein_coding 1.14 22.205
0.943 88.705 0.933 36.248 0.993 94.567
ENSMUSG00000074804 NA NA NA NA 1 95.211 1
86.666 1 92.691
ENSMUSG00000074805 Gm14047 antisense 1.143 84.452 1.105
95.431 0.792 70.44 0.653 91.65
ENSMUSG00000074807 Gm10762 protein_coding 0.869 37.82 1.135
82.904 1.011 84.552 1.04 94.904
ENSMUSG00000074811 Hps6 protein_coding 0.934 32.557 1.067
88.551 1.003 51.977 0.99 95.273
ENSMUSG00000074812 Gm355 protein_coding 1 83.169 1
87.553 1 75.809 1 90.177
ENSMUSG00000074813 Gm14005 processed_transcript 1.021 83.263
0.992 86.604 0.983 75.809 1.044 93.117
ENSMUSG00000074817 Papolb protein_coding 1.162 60.853
1.346 90.895 0.637 39.528 0.718 85.493
ENSMUSG00000074818 Pdzd7 protein_coding 0.783 8.709 1.209 92.931
1.057 35.894 0.864 83.724
ENSMUSG00000074822 Gm10766 protein_coding 1.025 74.516
0.708 79.475 1.375 76.225 0.98 84.29
ENSMUSG00000074824 Rslcan18 protein_coding 1.007 76.135
1.156 82.904 0.86 59.726 0.959 96.358
ENSMUSG00000074825 Itpripl1 protein_coding 1.239 0.2 1.006
89.996 0.805 1.898 1.057 88.755
ENSMUSG00000074826 Gm10767 protein_coding 0.667 2.536 1.009

89.996 1.481 10.76 0.927 84.29
ENSMUSG00000074829 2010315B03Rik protein_coding 1.092 14.008
0.937 81.284 0.974 48.851 0.937 85.493
ENSMUSG00000074830 Zfp640 protein_coding 1 86.172 1
95.941 1 74.535 1 95.273
ENSMUSG00000074832 2410141K09Rik protein_coding 1 75.761
1 93.094 1 87.336 1 95.593
ENSMUSG00000074837 Gm10770 protein_coding 1 84.534 1
96.216 1 86.602 1 91.872
ENSMUSG00000074841 Gm6917 pseudogene 1 74.482 1
97.155 1 80.015 1 95.273
ENSMUSG00000074846 Gm10774 protein_coding 1 86.145 1
96.68 1 81.169 1 95.35
ENSMUSG00000074849 Spata31d1c protein_coding 1 73.585 1
95.088 1 78.498 1 90.674
ENSMUSG00000074852 Hpse2 protein_coding 1.408 36.686 1.191
96.763 0.594 28.237 0.859 89.827
ENSMUSG00000074862 BC025920 protein_coding 0.916 36.259
1.104 82.76 0.99 76.768 1.048 88.797
ENSMUSG00000074863 Gm5665 lincRNA 1.239 39.851 1.14
94.102 0.706 57.091 0.807 92.675
ENSMUSG00000074865 Zfp934 protein_coding 1.29 13.557
1.052 88.136 0.738 6.858 0.897 92.748
ENSMUSG00000074867 Zfp808 protein_coding 1.584 1.43 NA NA
0.632 2.333 0.94 85.493
ENSMUSG00000074870 Cts3 protein_coding 1 83.418 1
96.843 1 88.601 1 96.192
ENSMUSG00000074871 Ctsm protein_coding 1 85.637 1
97.155 1 78.713 1 87.447
ENSMUSG00000074872 Ctxn2 protein_coding 1 71.217 1
94.367 1 67.339 1 91.372
ENSMUSG00000074873 AI606181 protein_coding 0.89 17.276
1.037 92.561 1.081 54.673 1.054 93.964
ENSMUSG00000074874 Ctla2b protein_coding 1.16 16.959
0.824 66.09 1.046 79.325 1.037 96.183
ENSMUSG00000074876 Spata5l1 processed_transcript 0.86 10.903
1.154 71.617 1.009 85.792 0.976 92.181
ENSMUSG00000074877 Gm10780 pseudogene 1 85.946 1
95.088 1 85.675 1 95.72
ENSMUSG00000074882 Cyp2c68 protein_coding 1 82.092 1
96.138 1 81.492 1 96.183
ENSMUSG00000074884 Serf2 protein_coding 0.952 25.111 1.034
82.904 1.018 78.498 1.04 48.857
ENSMUSG00000074885 Gm10782 protein_coding 1 82.891 1
95.211 1 84.83 1 96.071
ENSMUSG00000074886 Grk6 protein_coding 1.052 52.134 0.959
94.102 0.992 67.648 0.97 93.132
ENSMUSG00000074887 EU599041 protein_coding 1 86.74 NA NA
1 88.601 1 92.466
ENSMUSG00000074890 Lcmt2 protein_coding 0.878 18.602 1.003
95.708 1.132 30.213 1.027 96.042
ENSMUSG00000074892 B3galt5 protein_coding 1.53 33.099
0.979 96.847 0.67 34.21 0.525 83.724
ENSMUSG00000074895 Eif4e1b protein_coding 1 79.068 1

96.216	1	85.352	1	92.564
ENSMUSG00000074896	Ifit3	protein_coding	1.169	5.932	1.385	83.104
0.617	15.63	1.731	43.377
ENSMUSG00000074899	Sptbn5	protein_coding	0.573	0.987	0.954
96.652	1.834	5.964	1.079	86.445
ENSMUSG00000074903	Gm2058	protein_coding	1	83.523	1
95.504	1	84.896	1	94.904
ENSMUSG00000074909	Ranbp6	protein_coding	0.992	69.985
0.965	96.013	1.045	61.418	1.016	96.358
ENSMUSG00000074912	Gm14207	processed_transcript	0.783	49.749
1.139	85.756	1.117	85.935	1.046	93.261
ENSMUSG00000074913	Gm815	protein_coding	1	80.688	1
95.708	1	86.666	1	93.316
ENSMUSG00000074916	Chst14	protein_coding	1.029	42.134
1.042	92.931	0.931	17.381	0.984	94.567
ENSMUSG00000074918	5430417L22Rik	antisense	0.962	40.568
1.165	60.309	0.894	15.904	1.072	86.118
ENSMUSG00000074919	Gm10787	protein_coding	1	86.918	NA
NA	1	83.719	1	96.358
ENSMUSG00000074922	Fam122a	protein_coding	1.004	61.334
1.047	79.261	0.953	81.169	0.879	83.952
ENSMUSG00000074923	Pak6	protein_coding	1.179	45.307	0.759
60.806	1.119	53.882	0.949	88.505
ENSMUSG00000074925	Ptar1	protein_coding	0.672	19.985	0.889
83.321	1.668	5.864	0.978	96.183
ENSMUSG00000074928	Krtap14	protein_coding	1	68.485	1
93.654	1	85.464	1	94.904
ENSMUSG00000074930	Gm13981	pseudogene	1	86.918	1
96.138	1	86.602	1	93.722
ENSMUSG00000074934	Grem1	protein_coding	1	83.658	1
85.242	1	42.199	1	83.276
ENSMUSG00000074943	Olfr1316	protein_coding	1	86.843	1
95.211	1	86.346	1	91.872
ENSMUSG00000074945	Olfr1314	protein_coding	1	86.918	1
96.216	1	88.349	1	95.92
ENSMUSG00000074946	Olfr1313	protein_coding	1	76.968	1
97.155	1	84.669	1	96.042
ENSMUSG00000074947	Olfr1312	protein_coding	1	81.465	1
95.708	1	83.158	1	94.016
ENSMUSG00000074948	Olfr1311	protein_coding	1	73.734	1
93.908	1	88.203	1	93.964
ENSMUSG00000074949	Olfr1310	protein_coding	1	85.391	1
96.847	1	85.935	1	94.904
ENSMUSG00000074951	Olfr1309	protein_coding	1	80.03	1
92.137	1	82.436	1	96.071
ENSMUSG00000074952	Olfr1308	protein_coding	1	83.333	NA
NA	1	84.724	1	93.132
ENSMUSG00000074955	Olfr1305	protein_coding	1	72.546	1
97.263	1	86.666	1	93.457
ENSMUSG00000074957	Olfr1299	protein_coding	1	85.946	1
96.68	NA	NA	1	96.139
ENSMUSG00000074959	Olfr1295	protein_coding	1	84.819	1
90.287	1	75.809	1	95.74
ENSMUSG00000074965	Olfr1277	protein_coding	1	86.57	1

97.155 1 82.436 1 93.584
ENSMUSG00000074966 NA NA NA NA NA NA 1 86.666
1 93.964
ENSMUSG00000074968 Ano3 protein_coding 1.151 23.493 0.858
93.654 1.01 69.445 1.295 55.841
ENSMUSG00000074970 Gm10794 pseudogene 1 86.351 1
95.211 1 80.015 1 94.468
ENSMUSG00000074971 Fibin protein_coding 0.804 54.87 1.041 96.847
1.188 45.035 1.153 56.339
ENSMUSG00000074973 Gm11382 pseudogene 1 74.689 1
96.407 1 88.693 1 92.762
ENSMUSG00000074978 Actg-ps1 pseudogene 1 83.169 1
95.708 1 86.258 1 94.904
ENSMUSG00000074985 Olfr1452-ps1 pseudogene 1 82.705 1
96.013 1 81.857 1 94.904
ENSMUSG00000074987 AI314831 antisense 1 86.474 1
96.138 1 80.089 1 95.676
ENSMUSG00000074994 Qser1 protein_coding 0.998 85.832 NA NA
0.981 74.179 1.026 92.564
ENSMUSG00000074995 Olfr201 protein_coding 1 82.982 NA
NA 1 84.308 1 95.72
ENSMUSG00000074996 Olfr199 protein_coding 1 82.231 NA
NA NA NA 1 96.183
ENSMUSG00000074999 Gm10797 protein_coding 1 84.263 1
96.948 1 58.775 1 93.045
ENSMUSG00000075000 Nrbf2 protein_coding 1.098 30.984 0.979
94.102 0.93 17.898 0.991 93.964
ENSMUSG00000075002 Gm813 protein_coding 1 83.823 1
95.211 1 87.971 1 94.654
ENSMUSG00000075006 Gm10799 protein_coding 1 86.843 1
95.329 1 86.727 1 95.877
ENSMUSG00000075010 AW112010 protein_coding 1.21 24.147
0.995 96.138 0.828 40.338 0.815 91.269
ENSMUSG00000075012 Fjx1 protein_coding 0.924 47.174 1.117
83.104 0.968 86.985 0.948 96.248
ENSMUSG00000075014 Gm10800 protein_coding 1 78.909 1
96.847 1 53.587 1 96.183
ENSMUSG00000075015 Gm10801 protein_coding 1 83.333 1
97.263 1 87.744 1 96.071
ENSMUSG00000075021 Gm13889 pseudogene 1 67.334 1
95.211 1 76.754 1 91.625
ENSMUSG00000075023 Accsl protein_coding 0.93 86.74 1.128 90.287
0.957 68.425 1.122 93.132
ENSMUSG00000075024 Gm10803 protein_coding 1 84.397 1
95.504 1 83.275 1 93.734
ENSMUSG00000075027 4631405J19Rik processed_transcript 0.484 25.3
1.038 96.216 1.986 44.125 1.202 95.92
ENSMUSG00000075028 Prdm11 protein_coding 0.939 33.002
1.043 96.68 1.022 41.806 0.848 84.29
ENSMUSG00000075031 Hist1h2bb protein_coding 0.752 44.252
1.182 95.735 1.123 84.308 0.776 85.493
ENSMUSG00000075033 Nxpe3 protein_coding 1.023 81.24 0.949 96.308
1.032 87.336 0.91 90.611
ENSMUSG00000075036 Gm10805 pseudogene 1 84.452 1

93.994 1 87.786 1 95.74
ENSMUSG00000075040 Zfp408 protein_coding 0.912 14.086
1.014 90.287 1.082 9.301 1.047 74.193
ENSMUSG00000075042 4930431P03Rik processed_transcript 1.451
50.111 0.592 67.165 1.166 74.686 0.659 83.724
ENSMUSG00000075043 Tmem223 protein_coding 1 65.199 1
92.253 1 81.857 1 94.904
ENSMUSG00000075044 Slc22a29 protein_coding 0.533 16.895
1.004 95.809 1.86 29.819 1.06 93.722
ENSMUSG00000075046 4933403O03Rik pseudogene 1 47.266 1
96.407 1 72.378 1 95.877
ENSMUSG00000075054 Yae1d1 protein_coding 0.992 83.823
1.011 95.504 0.998 79.119 0.986 93.156
ENSMUSG00000075061 Olfr1272 protein_coding 1 85.728 1
97.155 1 73.874 1 96.042
ENSMUSG00000075062 Olfr1271 protein_coding 1 84.294 1
95.211 NA NA 1 93.457
ENSMUSG00000075063 Olfr142 protein_coding 1 78.299 NA
NA 1 84.229 1 95.273
ENSMUSG00000075064 Olfr1506 protein_coding 1 78.744 1
96.013 1 87.658 1 95.74
ENSMUSG00000075065 Olfr1270 protein_coding 1 81.01 1
96.013 1 87.744 1 95.74
ENSMUSG00000075066 Olfr32 protein_coding 1 78.299 1
94.48 1 77.294 1 93.413
ENSMUSG00000075067 Olfr1269 protein_coding 1 85.77 1
96.335 1 81.857 1 93.964
ENSMUSG00000075068 Olfr140 protein_coding 1 84.294 1
97.263 1 84.961 1 95.273
ENSMUSG00000075069 Olfr1264 protein_coding 1 80.03 1
96.268 1 68.82 1 95.74
ENSMUSG00000075072 Olfr48 protein_coding 1 81.01 1
95.708 1 82.436 1 95.74
ENSMUSG00000075073 Olfr1256 protein_coding 1 85.728 1
93.654 NA NA 1 96.287
ENSMUSG00000075074 Olfr1254 protein_coding 1 86.199 1
97.097 1 84.724 1 95.92
ENSMUSG00000075075 Olfr1253 protein_coding 1 66.58 1
91.532 1 88.693 1 92.748
ENSMUSG00000075076 Olfr1252 protein_coding 1 81.01 1
95.708 1 86.666 1 96.086
ENSMUSG00000075077 Olfr1251 protein_coding 1 78.224 1
96.138 1 81.032 1 95.777
ENSMUSG00000075078 Olfr1250 protein_coding 1 74.021 1
95.504 1 85.134 1 92.675
ENSMUSG00000075079 Olfr1249 protein_coding 1 80.146 NA
NA 1 84.961 1 96.071
ENSMUSG00000075080 Olfr1248 protein_coding 1 81.925 1
94.89 1 86.144 1 91.492
ENSMUSG00000075081 Olfr1247 protein_coding 1 84.294 1
95.708 1 84.669 1 96.192
ENSMUSG00000075082 Olfr1246 protein_coding 1 79.853 1
96.268 1 84.308 1 96.358
ENSMUSG00000075083 Olfr1245 protein_coding 1 80.03 1

93.27 1 83.857 1 93.316
ENSMUSG00000075084 Olfr1243 protein_coding 1 80.408 1
93.817 1 88.461 1 89.892
ENSMUSG00000075085 Olfr1242 protein_coding 1 86.145 1
96.843 1 83.857 1 94.904
ENSMUSG00000075086 Olfr1241 protein_coding 1 84.865 1
95.329 1 83.397 1 94.904
ENSMUSG00000075087 Olfr1240 protein_coding 1 77.615 1
92.062 1 86.831 1 94.654
ENSMUSG00000075088 NA NA NA NA 1 93.654 1
86.475 1 96.192
ENSMUSG00000075089 Olfr1238 protein_coding 1 84.819 1
94.96 1 81.857 1 94.654
ENSMUSG00000075090 Olfr1234 protein_coding 1 86.474 1
96.843 NA NA 1 96.183
ENSMUSG00000075091 Olfr1233 protein_coding 1 85.59 1
93.046 1 75.809 1 94.752
ENSMUSG00000075092 Olfr1232 protein_coding 1 83.418 1
97.097 1 79.119 1 96.183
ENSMUSG00000075093 Olfr1231 protein_coding 1 80.815 NA
NA 1 87.786 1 94.904
ENSMUSG00000075094 Olfr1230 protein_coding 1 73.487 1
96.763 1 88.601 1 93.722
ENSMUSG00000075095 Olfr1229 protein_coding 1 84.977 1
95.504 1 84.896 1 96.287
ENSMUSG00000075096 Olfr1228 protein_coding 1 84.397 1
96.847 1 88.274 1 93.156
ENSMUSG00000075097 Olfr1226 protein_coding 1 82.982 1
93.908 1 79.426 1 92.748
ENSMUSG00000075098 Olfr1225 protein_coding 1 85.728 1
97.263 1 83.811 1 93.156
ENSMUSG00000075099 Olfr1224-ps1 protein_coding 1 84.168
1 96.138 1 88.203 1 95.74
ENSMUSG00000075100 Olfr1223 protein_coding 1 84.865 1
96.948 1 88.349 1 94.153
ENSMUSG00000075101 Olfr1222 protein_coding 1 58.264 1
87.998 1 86.666 1 92.675
ENSMUSG00000075102 Olfr1221 protein_coding 1 81.76 1
97.288 1 88.693 1 96.248
ENSMUSG00000075103 Olfr1220 protein_coding 1 81.657 1
97.263 1 86.727 1 94.654
ENSMUSG00000075104 Olfr1219 protein_coding 1 82.231 1
97.097 1 80.899 1 93.316
ENSMUSG00000075105 Olfr1218 protein_coding 1 86.658 1
97.263 1 82.161 1 96.248
ENSMUSG00000075106 Olfr1217 protein_coding 1 83.09 1
95.708 1 86.985 1 92.181
ENSMUSG00000075107 Olfr1216 protein_coding 1 86.464 1
96.68 1 88.657 1 94.654
ENSMUSG00000075108 Olfr1215 protein_coding 1 83.263 1
96.68 1 86.144 1 94.904
ENSMUSG00000075110 Olfr1214 protein_coding 1 80.751 1
96.138 NA NA 1 96.287
ENSMUSG00000075111 Gm13762 processed_transcript 1 84.534

1 96.763 1 86.144 1 96.183
ENSMUSG00000075112 Olfr1211 protein_coding 1 85.129 1
95.211 1 72.853 1 95.877
ENSMUSG00000075113 Olfr1209 protein_coding 1 74.147 1
97.129 1 88.693 1 96.192
ENSMUSG00000075114 Olfr1208 protein_coding 1 80.608 1
90.138 1 79.524 1 95.74
ENSMUSG00000075115 Olfr1200 protein_coding 1 86.172 1
96.268 1 88.693 1 96.183
ENSMUSG00000075116 NA NA NA NA NA NA 1 87.274
1 92.748
ENSMUSG00000075117 Olfr1198 protein_coding 1 83.09 1
95.088 1 84.434 1 96.071
ENSMUSG00000075119 Olfr1197 protein_coding 1 86.112 1
94.48 1 88.693 1 91.525
ENSMUSG00000075120 Olfr1196 protein_coding 1 86.006 1
95.504 1 80.163 1 92.389
ENSMUSG00000075121 Olfr1195 protein_coding 1 81.196 1
95.708 NA NA 1 92.389
ENSMUSG00000075122 Cd80 protein_coding 1.531 0 0.959 93.654
0.683 8.634 1.169 63.114
ENSMUSG00000075124 Olfr1182 protein_coding 1 79.853 NA
NA 1 78.498 1 96.086
ENSMUSG00000075125 Olfr1181 protein_coding 1 82.982 NA
NA 1 87.744 1 96.086
ENSMUSG00000075126 Olfr1180 protein_coding 1 83.263 1
96.216 1 84.961 1 93.685
ENSMUSG00000075127 Olfr1179 protein_coding 1 85.283 1
95.708 1 87.336 1 92.748
ENSMUSG00000075128 Olfr1177-ps pseudogene 1 79.709 NA NA
1 80.015 1 95.74
ENSMUSG00000075132 Olfr1173 protein_coding 1 79.709 NA
NA 1 81.976 1 95.273
ENSMUSG00000075133 Olfr1170 protein_coding 1 86.145 1
96.378 1 88.349 1 95.72
ENSMUSG00000075134 Olfr1167 protein_coding 1 81.196 1
96.216 NA NA 1 94.608
ENSMUSG00000075135 Olfr1166 protein_coding 1 74.958 NA
NA 1 78.369 1 91.525
ENSMUSG00000075136 Olfr1164 protein_coding 1 71.674 1
97.129 1 86.666 1 96.071
ENSMUSG00000075137 Olfr1163 protein_coding 1 71.36 1
96.652 1 84.896 1 96.358
ENSMUSG00000075139 Olfr1162 protein_coding 1 74.516 1
96.407 1 83.857 1 94.654
ENSMUSG00000075140 Olfr73 protein_coding 1 82.982 1
96.013 1 88.693 1 96.287
ENSMUSG00000075141 Olfr1160 protein_coding 1 75.761 1
91.361 1 83.607 1 96.248
ENSMUSG00000075142 Olfr74 protein_coding 1 76.135 1
96.763 1 88.161 1 93.722
ENSMUSG00000075143 Olfr1157 protein_coding 1 79.165 1
96.216 1 84.896 1 95.559
ENSMUSG00000075144 NA NA NA NA 1 96.847 1

87.971 1 96.287
ENSMUSG00000075145 Olfr1155 protein_coding 1 83.09 1
95.708 1 88.601 1 93.734
ENSMUSG00000075146 Olfr1154 protein_coding 1 86.112 NA
NA 1 87.173 1 96.071
ENSMUSG00000075148 Olfr1141 protein_coding 1 84.049 1
93.654 1 87.37 1 96.358
ENSMUSG00000075149 Olfr1138 protein_coding 1 84.263 NA
NA 1 86.666 1 95.877
ENSMUSG00000075150 Olfr1137 protein_coding 1 86.464 1
96.407 1 88.729 1 95.559
ENSMUSG00000075151 Olfr1136 protein_coding 1 85.499 1
96.847 1 84.724 1 94.904
ENSMUSG00000075153 Olfr1135 protein_coding 1 86.918 1
92.793 NA NA 1 95.676
ENSMUSG00000075154 Olfr1134 protein_coding 1 82.479 NA
NA 1 88.274 1 95.273
ENSMUSG00000075155 Olfr1133 protein_coding 1 75.858 1
96.763 1 82.575 1 96.183
ENSMUSG00000075156 Olfr1128 protein_coding 1 79.277 1
93.046 1 66.043 NA NA
ENSMUSG00000075158 Olfr1111 protein_coding 1 85.728 1
90.895 1 87.898 1 95.593
ENSMUSG00000075159 Olfr1110 protein_coding 1 70.597 1
96.001 1 88.601 1 93.156
ENSMUSG00000075160 Olfr259 protein_coding 1 82.732 1
96.216 1 84.896 1 94.904
ENSMUSG00000075161 Olfr1109 protein_coding 1 81.823 1
92.545 1 80.899 1 95.72
ENSMUSG00000075162 Gm10812 pseudogene 0.982 68.353 0.987
97.155 1.034 85.757 0.804 88.387
ENSMUSG00000075163 Olfr1107 protein_coding 1 82.732 NA
NA 1 84.626 1 95.832
ENSMUSG00000075164 Olfr1106 protein_coding 1 81.125 1
94.848 1 88.729 1 95.74
ENSMUSG00000075165 Olfr1105 protein_coding 1 79.165 1
96.216 1 88.461 1 96.071
ENSMUSG00000075166 Olfr1104 protein_coding 1 71.674 NA
NA 1 86.666 1 96.192
ENSMUSG00000075167 Olfr1101 protein_coding 1 77.615 1
96.897 1 76.923 1 94.99
ENSMUSG00000075168 Olfr1099 protein_coding 1 84.324 1
96.216 1 83.857 1 93.132
ENSMUSG00000075169 Olfr1098 protein_coding 1 85.728 1
94.848 1 83.275 1 95.273
ENSMUSG00000075170 Olfr1097 protein_coding 1 82.352 1
95.088 1 82.778 1 95.877
ENSMUSG00000075171 Olfr1095 protein_coding 1 84.049 1
95.301 1 85.757 1 90.177
ENSMUSG00000075172 NA NA NA NA 1 96.216 1
88.076 1 94.384
ENSMUSG00000075173 Olfr1089 protein_coding 1 82.231 1
94.597 1 78.155 1 95.273
ENSMUSG00000075174 Olfr1087 protein_coding 1 74.8 NA NA

1	84.308	1	92.31
ENSMUSG00000075175	Olfr1086	protein_coding	1	78.909	1
95.211	1	86.666	1	96.042
ENSMUSG00000075176	Olfr1085	protein_coding	1	82.092	1
95.708	1	86.666	1	96.358
ENSMUSG00000075177	Olfr1084	protein_coding	1	85.129	1
96.763	1	88.601	1	95.777
ENSMUSG00000075178	Olfr1080	protein_coding	1	72.985	1
96.268	NA	NA	1	96.183
ENSMUSG00000075179	Olfr1079	protein_coding	1	85.59	1
97.155	1	85.58	1	94.153
ENSMUSG00000075180	Olfr228	protein_coding	1	64.014	1
97.263	1	79.524	1	94.19
ENSMUSG00000075181	Olfr1066	protein_coding	1	85.857	1
96.847	1	76.498	1	93.316
ENSMUSG00000075182	Olfr1065	protein_coding	1	79.93	1
95.088	1	84.724	1	96.287
ENSMUSG00000075185	Olfr1061	protein_coding	1	80.815	1
94.96	1	83.158	1	96.248
ENSMUSG00000075186	Olfr1058	protein_coding	1	80.251	1
96.847	NA	NA	1	95.74
ENSMUSG00000075187	Olfr1057	protein_coding	1	84.452	1
93.654	1	87.274	1	91.078
ENSMUSG00000075188	Olfr1056	protein_coding	1	85.355	1
94.848	1	87.103	1	96.086
ENSMUSG00000075189	Olfr1055	protein_coding	1	83.169	1
96.847	1	87.658	1	94.19
ENSMUSG00000075190	Olfr1054	protein_coding	1	80.03	NA	NA
1	80.634	1	93.612
ENSMUSG00000075192	Olfr1053	protein_coding	1	85.728	1
97.155	1	79.625	1	96.287
ENSMUSG00000075193	Olfr1051	protein_coding	1	78.639	1
96.216	1	86.985	1	88.955
ENSMUSG00000075194	Olfr1049	protein_coding	1	84.865	1
96.308	NA	NA	1	95.273
ENSMUSG00000075195	Olfr1048	protein_coding	1	79.709	1
96.652	1	81.29	1	94.654
ENSMUSG00000075196	Olfr1047	protein_coding	1	86.351	1
96.216	1	82.903	1	93.261
ENSMUSG00000075197	Olfr1046	protein_coding	1	81.01	1
93.817	1	86.112	1	96.071
ENSMUSG00000075198	Olfr1045	protein_coding	1	85.832	1
96.013	1	84.896	1	95.92
ENSMUSG00000075199	Olfr52	protein_coding	1	84.534	1
93.654	1	88.729	1	96.183
ENSMUSG00000075200	Olfr1044	protein_coding	1	84.738	1
91.955	1	81.62	1	92.381
ENSMUSG00000075201	Olfr1043	protein_coding	1	86.464	NA
NA	1	87.274	1	96.287
ENSMUSG00000075202	Olfr1042	protein_coding	1	84.263	1
97.155	1	86.258	1	96.358
ENSMUSG00000075203	Olfr1040	protein_coding	1	85.77	NA	NA
1	79.776	1	95.74
ENSMUSG00000075204	Olfr1039	protein_coding	1	85.499	1

95.941	1	84.896	1	95.273
ENSMUSG00000075205	Olfr1037	protein_coding	1	82.479	1
96.013	1	84.308	1	96.184
ENSMUSG00000075206	Olfr1024	protein_coding	1	77.615	1
95.504	1	84.308	1	96.183
ENSMUSG00000075208	Olfr1019	protein_coding	1	82.352	NA
NA	1	87.274	1	95.832
ENSMUSG00000075209	Olfr1016	protein_coding	1	84.564	NA
NA	1	87.658	1	93.132
ENSMUSG00000075210	Olfr1012	protein_coding	1	85.857	1
96.216	1	81.525	1	92.018
ENSMUSG00000075211	Olfr1006	protein_coding	1	85.129	1
96.013	1	82.161	1	96.248
ENSMUSG00000075212	Olfr154	protein_coding	1	83.726	NA
NA	1	84.626	1	95.72
ENSMUSG00000075214	NA	NA	NA	NA	1	91.661	1
67.562	1	96.287
ENSMUSG00000075215	Olfr1000	protein_coding	1	86.464	1
95.35 NA	NA	1	92.748
ENSMUSG00000075217	4833423E24Rik	protein_coding	1	85.728
1	96.216	NA	NA	1	95.74
ENSMUSG00000075218	Olfr995	protein_coding	1	82.543	1
96.843	NA	NA	1	95.273
ENSMUSG00000075219	Olfr994	protein_coding	1	84.534	1
95.431	1	81.032	1	95.405
ENSMUSG00000075220	Olfr993	protein_coding	1	80.751	1
97.263	1	83.719	1	96.139
ENSMUSG00000075221	Olfr992	protein_coding	1	86.351	NA
NA	1	84.504	1	94.016
ENSMUSG00000075222	Olfr988	protein_coding	1	84.564	1
94.848	1	87.173	1	92.748
ENSMUSG00000075223	Olfr987	protein_coding	1	67.608	NA
NA	NA	NA	1	96.358
ENSMUSG00000075224	Lrrc55	protein_coding	1.434 40.635
1.119 96.216	0.622 42.376	0.828 90.674
ENSMUSG00000075225	Ccdc162	protein_coding	0.723 17.664
0.971 96.897	1.421 31.479	0.936 92.181
ENSMUSG00000075227	Znhit2	protein_coding	0.933 8.614 1.005
96.843	1.067 32.776	0.95	85.493
ENSMUSG00000075228	Gm10813	pseudogene	1	80.815	NA	NA
1	83.925	1	94.99
ENSMUSG00000075229	Ccdc58	protein_coding	0.991 77.113
0.945 65.165	1.065 30.107	1.06	83.276
ENSMUSG00000075232	Amd1	protein_coding	0.861 21.658	1.005
96.847	1.157 41.6	0.845 96.071
ENSMUSG00000075245	Gm6043	pseudogene	1	85.77 NA	NA	1
86.144	1	96.248
ENSMUSG00000075249	Fsip2 protein_coding	1	86.819	NA	NA
1	86.666	1	96.358
ENSMUSG00000075254	Heg1	protein_coding	0.966 86.351	1.041
87.857	0.993 70.9	1.031 79.268
ENSMUSG00000075256	Cerkl protein_coding	1.188 26.77 1.067 96.013
0.789 51.715	0.928 91.625
ENSMUSG00000075265	Gm10818	antisense	1.184 71.217	1.02

95.735 0.83 45.799 0.859 91.985
ENSMUSG00000075266 Cenpw protein_coding 1.125 60.168 0.908
91.661 0.978 87.898 1.063 92.691
ENSMUSG00000075268 Gm10819 pseudogene 1 86.464 1
95.708 1 70.534 1 95.593
ENSMUSG00000075270 Pde11a protein_coding 1.239 27.32 0.991
92.404 0.814 52.213 1.231 72.369
ENSMUSG00000075271 Ttc30a1 protein_coding 1.198 21.146 NA
NA 0.836 19.552 0.934 96.287
ENSMUSG00000075272 Ttc30a2 protein_coding 1.013 36.686
0.939 95.708 1.054 87.173 1.04 95.35
ENSMUSG00000075273 Ttc30b protein_coding 0.921 42.034
1.128 81.127 0.959 64.155 1.017 95.92
ENSMUSG00000075276 Gm13653 pseudogene 1 79.93 1 95.708
NA NA 1 96.086
ENSMUSG00000075277 6720416L17Rik antisense 0.67 59.028
0.755 95.35 1.969 69.002 1.348 90.674
ENSMUSG00000075284 Wipf1 protein_coding 1.131 0 1.035 93.654
0.856 3.432 0.997 96.287
ENSMUSG00000075288 Gm6913 pseudogene 1 86.112 1
96.984 1 83.719 1 95.777
ENSMUSG00000075289 Carns1 protein_coding 0.98 80.146
1.015 88.705 1.006 68.129 0.978 92.742
ENSMUSG00000075296 Aldh3b2 protein_coding 0.749 25.111
0.897 95.301 1.492 31.975 1.134 46.846
ENSMUSG00000075297 H60b protein_coding 0.594 15.486 1.235
92.793 1.361 42.065 1.213 93.457
ENSMUSG00000075302 Erich2 protein_coding 1.173 72.85 1.305
91.361 0.656 88.693 0.505 83.724
ENSMUSG00000075307 Klhl41 protein_coding 3.848 10.464
1.315 83.104 0.197 0.879 1.047 93.734
ENSMUSG00000075314 Gm1322 lincRNA 1 83.658 NA NA
1 86.144 1 95.273
ENSMUSG00000075318 Scn2a1 protein_coding 1.181 68.727 NA
NA 0.975 85.58 1.127 56.863
ENSMUSG00000075324 Fign protein_coding 0.896 43.435 1.091
85.756 1.025 70.623 0.843 95.832
ENSMUSG00000075327 Zbtb2 protein_coding 0.949 57.818 1.064
93.899 0.992 85.921 0.951 85.493
ENSMUSG00000075334 Rprm protein_coding 1.373 68.727 1.278
93.899 0.568 37.906 0.994 92.181
ENSMUSG00000075335 4930588K23Rik antisense 1 85.946 NA
NA 1 78.498 1 91.625
ENSMUSG00000075359 Gm20775 protein_coding 1 84.397 1
96.843 1 86.144 1 93.627
ENSMUSG00000075376 Rc3h2 protein_coding 1.013 60.168 1.043
92.111 0.948 36.786 0.923 83.724
ENSMUSG00000075377 Olfr362 protein_coding 1 85.728 1
95.941 1 87.898 1 96.042
ENSMUSG00000075378 Olfr361 protein_coding 1 85.946 1
95.211 1 86.144 1 94.904
ENSMUSG00000075379 Olfr358 protein_coding 1 82.092 1
96.216 1 83.811 1 95.273
ENSMUSG00000075380 Olfr355 protein_coding 1 86.351 1

96.68 1 80.163 1 95.35
ENSMUSG00000075382 Olfr353 protein_coding 1 84.263 1
96.407 1 86.084 1 91.928
ENSMUSG00000075383 Olfr351 protein_coding 1 77.291 1
91.661 1 86.144 1 94.752
ENSMUSG00000075384 Olfr3 protein_coding 1 82.543 1
93.146 1 86.346 1 94.904
ENSMUSG00000075387 Olfr341 protein_coding 1 75.196 NA
NA 1 87.431 1 95.35
ENSMUSG00000075389 2810410L24Rik processed_transcript 0.703
1.031 1.012 95.735 1.409 5.977 0.889 83.724
ENSMUSG00000075390 Rpl36-ps8 pseudogene 1 71.217 1
97.155 NA NA 1 94.825
ENSMUSG00000075394 Hoxc4 protein_coding 0.931 42.361 1.117
79.579 0.963 74.853 1.026 92.018
ENSMUSG00000075395 A630010A05Rik protein_coding 1 81.52 NA
NA 1 86.112 1 94.384
ENSMUSG00000075402 Krt76 protein_coding 1 84.263 1
96.68 1 87.971 1 92.018
ENSMUSG00000075405 9430097D07Rik protein_coding 1 60.569
1 81.127 1 88.729 1 86.301
ENSMUSG00000075408 6030408B16Rik protein_coding 0.807 86.74
1.361 96.843 0.911 83.607 0.945 85.493
ENSMUSG00000075410 Gm11744 protein_coding 1.016 70.741
0.941 95.708 1.046 88.693 0.859 83.724
ENSMUSG00000075415 Fnbp1 protein_coding 1.135 9.435 0.941 73.766
0.934 34.592 0.897 83.724
ENSMUSG00000075416 Gm14488 processed_transcript 1.172 81.178
0.693 86.604 1.231 78.369 0.971 86.301
ENSMUSG00000075419 Dolk protein_coding 0.851 6.677 1.088 72.546
1.083 31.761 0.919 83.724
ENSMUSG00000075420 Smim6 protein_coding 0.873 77.291 0.749
72.399 1.528 49.601 0.896 85.493
ENSMUSG00000075425 Gm13547 protein_coding 0.633 21.658
1.003 95.735 1.58 38.975 0.889 92.12
ENSMUSG00000075427 Olfr288 protein_coding 1 85.77 NA NA
NA NA 1 92.691
ENSMUSG00000075431 Gm11691 lincRNA 1 78.299 1
96.138 1 82.575 1 95.273
ENSMUSG00000075437 NA NA NA NA 1 97.129 1
88.657 1 92.181
ENSMUSG00000075463 4930594M22Rik processed_transcript 0.584
10.29 1.214 95.708 1.406 15.185 1.116 95.777
ENSMUSG00000075465 Gm10837 protein_coding 1 71.06 NA NA
NA NA 1 94.904
ENSMUSG00000075467 Dnlz protein_coding 0.928 8.709 1.055 79.411
1.021 69.728 0.946 88.505
ENSMUSG00000075469 Gm17205 pseudogene 1 85.832 1
94.102 1 88.729 1 93.132
ENSMUSG00000075470 Alg10b protein_coding 1 86.172
0.938 70.29 1.067 44.787 0.983 96.358
ENSMUSG00000075478 Slitrk1 protein_coding 2.019 6.853 0.865
96.138 0.572 7.329 1.559 64.513
ENSMUSG00000075485 Gm10842 protein_coding 1 83.333 NA

NA 1 84.961 NA NA
ENSMUSG00000075486 Commd6 protein_coding 1.01 66.652
0.981 90.378 1.009 79.426 1.017 71.305
ENSMUSG00000075502 Zbtbd6 protein_coding 1.34 29.536
0.944 94.597 0.791 59.922 1.292 85.577
ENSMUSG00000075510 Fam187a protein_coding 1 64.193 1
96.013 1 87.519 1 94.19
ENSMUSG00000075511 1700001L05Rik lincRNA 0.852 30.035 0.97
95.708 1.208 44.515 1.235 57.1
ENSMUSG00000075512 Gm1587 protein_coding 1 84.534 1
95.708 NA NA 1 96.183
ENSMUSG00000075514 Gm13375 antisense 0.868 28.804 1.074
80.806 1.07 78.369 1.01 96.287
ENSMUSG00000075517 Cyp2d37-ps protein_coding 1 82.732 1
96.68 1 86.258 1 95.273
ENSMUSG00000075520 Gm13318 protein_coding 1 77.763 1
92.631 1 73.096 1 96.183
ENSMUSG00000075524 4930407I10Rik protein_coding 1 67.163
NA NA 1 73.531 1 95.92
ENSMUSG00000075528 Aarsd1 protein_coding 0.859 60.569
1.163 90.454 1.001 87.274 0.829 94.99
ENSMUSG00000075529 BC028471 processed_transcript 0.962 76.396
1.083 95.088 0.962 84.308 0.766 86.699
ENSMUSG00000075538 Gm10855 protein_coding 0.817 80.146
1.252 95.211 NA NA 0.618 92.31
ENSMUSG00000075543 Urad protein_coding 1 69.943 1
94.48 1 84.308 1 94.654
ENSMUSG00000075551 Cyp3a41a protein_coding 1 84.819 1
96.407 1 84.724 1 93.734
ENSMUSG00000075552 Cyp3a41b protein_coding 1 72.985 1
95.708 1 80.089 1 96.042
ENSMUSG00000075553 Gm5464 protein_coding 0.942 43.816
1.229 79.579 0.865 64.155 0.677 94.608
ENSMUSG00000075555 Gm10863 antisense 0.775 21.176 0.91
95.504 1.412 20.886 1.046 96.287
ENSMUSG00000075558 Gm17151 antisense 1 84.865 1
87.857 1 84.308 1 93.964
ENSMUSG00000075566 Krtap4-6 protein_coding 1 85.857 1
94.102 1 87.744 1 96.183
ENSMUSG00000075567 Krtap1-4 protein_coding 1 83.418 NA
NA 1 78.369 1 96.192
ENSMUSG00000075569 Rsph10b protein_coding 0.959 72.985
0.886 79.475 1.178 19.877 0.987 87.735
ENSMUSG00000075570 Krt26 protein_coding 1 86.843 1
84.017 1 72.148 1 92.748
ENSMUSG00000075572 Defb43 protein_coding 1 82.479 NA
NA 1 78.155 1 93.156
ENSMUSG00000075573 Defb47 protein_coding 1 79.93 1
94.597 1 81.976 1 89.892
ENSMUSG00000075574 Defb48 protein_coding 1 85.655 1
97.097 1 87.103 1 95.273
ENSMUSG00000075576 Gm12359 antisense 0.932 78.744 1.241
82.904 0.868 53.224 0.741 83.724
ENSMUSG00000075581 Gm16409 pseudogene 1 35.449 1

83.104	1	68.266	1	93.413
ENSMUSG00000075585	6330403L08Rik	protein_coding	0.765	11.726
1.291	60.309	1.011	80.163	0.996	96.358
ENSMUSG00000075586	Gm11529	processed_transcript	1	68.485
1	96.984	1	87.336	1	96.287
ENSMUSG00000075587	Gm11531	pseudogene	1	79.709	1
95.708	1	84.896	1	96.183
ENSMUSG00000075588	Hoxb2	protein_coding	0.828	8.709	1.061	85.756
1.138	36.388	0.968	85.493
ENSMUSG00000075589	Gm11536	antisense	0.671	3.009	1.009	83.104
1.479	15.945	1.093	92.018
ENSMUSG00000075590	Nrbp2	protein_coding	0.898	14.086	1.045
79.864	1.062	85.464	0.967	85.493
ENSMUSG00000075591	Gm10874	protein_coding	1	83.658	1
96.216	1	80.089	1	92.381
ENSMUSG00000075592	Nynrin	protein_coding	1.059	12.72	1.047
89.996	0.9	6.858	1.061	87.134
ENSMUSG00000075593	Gal3st4	protein_coding	1	81.01	1
97.129	1	69.825	1	93.584
ENSMUSG00000075595	Zfp652	protein_coding	1.004	80.51	0.941
81.731	1.055	50.091	0.935	83.724
ENSMUSG00000075596	B130006D01Rik	protein_coding	0.545	49.368
2.758	71.617	0.667	48.387	1.377	92.762
ENSMUSG00000075598	NA	NA	NA	NA	1	95.211	1
83.607	1	96.086
ENSMUSG00000075599	Smok3a	protein_coding	1	76.396	1
97.155	1	84.83	1	95.273
ENSMUSG00000075600	Zc3h3	protein_coding	0.986	50.519	0.978
95.504	1.032	61.418	1.011	96.042
ENSMUSG00000075602	Ly6a	protein_coding	1.317	0	0.918	70.693
0.827	32.082	1.264	46.412
ENSMUSG00000075604	Cyp11b1	protein_coding	1	86.658	1
96.68	1	88.461	1	93.132
ENSMUSG00000075610	Tmem92	protein_coding	1	86.819	1
97.263	1	74.535	1	90.805
ENSMUSG00000075611	Gm11545	pseudogene	1.666	37.414	0.63
74.851	0.955	87.336	0.984	96.183
ENSMUSG00000075700	Selt	protein_coding	1.028	26.77	1.001	97.155
0.971	21.117	1.075	92.018
ENSMUSG00000075701	Vimp	protein_coding	1.002	85.391	1.006
92.931	0.989	83.158	1.052	69.965
ENSMUSG00000075702	Selm	protein_coding	1.013	81.196	1.045
83.104	0.949	35.559	1.043	86.118
ENSMUSG00000075703	Ept1	protein_coding	1.078	19.132	0.978
95.211	0.949	35.815	0.989	95.832
ENSMUSG00000075704	Txnrd2	protein_coding	0.873	5.544	1.025
91.643	1.121	45.94	1.001	93.584
ENSMUSG00000075705	Msrb1	protein_coding	0.812	8.002	1.089	85.985
1.129	61.171	1.109	83.276
ENSMUSG00000075706	Gpx4	protein_coding	0.66	0.987	1.109	71.617
1.362	5.864	1.104	84.31
ENSMUSG00000075707	Dio3	protein_coding	0.292	48.146	1.696
83.104	NA	NA	17.659	57.075
ENSMUSG00000075719	Gm23741	snRNA	1	54.604	1	96.68	1

85.757 1 96.358
ENSMUSG00000075726 Gm23254 snoRNA 1 85.129 1
96.407 NA NA 1 95.273
ENSMUSG00000075760 Gm25414 snRNA 1 83.726 1 96.847
1 86.258 1 96.183
ENSMUSG00000075770 Gm22589 snoRNA 1 80.815 1
94.102 1 86.258 1 96.183
ENSMUSG00000075826 Gm23691 snRNA 1 83.823 1 95.735
1 87.274 1 96.184
ENSMUSG00000075829 Gm23692 snRNA 1 86.006 1 93.908
1 81.29 1 94.904
ENSMUSG00000075832 Gm22004 snRNA 1 81.76 1 97.263 NA
NA 1 96.287
ENSMUSG00000075833 Gm22003 snRNA 1 83.968 1 97.049
1 86.258 1 95.559
ENSMUSG00000075837 Gm22005 snRNA 1 65.284 1 96.013
1 85.352 1 93.132
ENSMUSG00000075864 Gm26002 snRNA 1 85.203 1 97.155
1 86.666 1 94.752
ENSMUSG00000075874 n-R5s1 rRNA 1 79.165 1 95.504
1 84.896 1 95.273
ENSMUSG00000075893 Gm25538 snRNA 1 86.57 1 97.263 1
88.601 1 93.964
ENSMUSG00000075903 Gm23941 snRNA 1 79.433 1 95.088
1 84.308 1 92.748
ENSMUSG00000075906 Gm23942 snRNA 1 74.8 1 96.407 NA
NA 1 94.608
ENSMUSG00000075908 Gm23940 snRNA 1 85.857 1 95.504
1 84.83 1 93.584
ENSMUSG00000075918 n-R5s2 rRNA 1 85.499 1 96.843
1 88.203 1 88.136
ENSMUSG00000075934 Gm24423 snRNA 1 84.661 1 96.847
1 88.693 1 94.904
ENSMUSG00000075952 Gm23422 snRNA 1 79.433 1 95.708
1 86.475 1 96.071
ENSMUSG00000075958 Gm23423 snRNA 1 78.744 1 95.708
NA NA 1 95.72
ENSMUSG00000075968 Gm25089 snoRNA 1 86.474 1
96.378 1 84.626 1 92.903
ENSMUSG00000075973 n-R5s64 rRNA 1 80.815 1 96.138
1 81.857 1 94.654
ENSMUSG00000075983 Gm24121 snRNA 1 81.01 1 95.708 1
86.084 1 94.174
ENSMUSG00000076005 Mir547 miRNA 1 83.169 1 96.897
NA NA 1 96.071
ENSMUSG00000076006 Mir376b miRNA 1 86.464 1 96.68 1
87.744 1 94.752
ENSMUSG00000076009 Mir688 miRNA 0.815 69.24 0.639 95.708
1.923 79.554 1.287 92.748
ENSMUSG00000076010 Mir615 miRNA 0.518 40.422 1.722 97.263
1.118 56.931 0.897 95.72
ENSMUSG00000076011 Mir652 miRNA 0.43 75.858 NA NA
2.663 80.015 5.486 74.193
ENSMUSG00000076012 Mir705 miRNA 1 83.823 1 90.053

							1	84.071	1	95.273
ENSMUSG00000076014	Mir669a-2	miRNA	1	84.049	1	96.268	1	87.274	1	95.74
ENSMUSG00000076028	Mir669a-3	miRNA	1	79.709	NA	NA	1	84.724	1	93.316
ENSMUSG00000076030	Gm22750	miRNA	1	82.543	1	93.654	1	86.475	1	92.263
ENSMUSG00000076031	Gm22751	miRNA	1	81.52	1	96.843	1	86.258	1	95.273
ENSMUSG00000076033	Mir692-2b	miRNA	1	85.77	1	96.843	1	80.015	1	95.676
ENSMUSG00000076036	Gm22133	miRNA	1	86.112	1	95.708	1	83.275	1	96.358
ENSMUSG00000076037	Gm22752	miRNA	1	82.891	1	96.68	1	80.381	1	93.964
ENSMUSG00000076038	Gm22774	miRNA	1	86.464	1	95.504	1	86.112	1	95.273
ENSMUSG00000076039	Gm22137	miRNA	1	66	1	97.049	1	84.071	1	95.273
ENSMUSG00000076041	Mir20b	miRNA	1	75.399	1	95.301	1	88.076	1	95.273
ENSMUSG00000076042	Mir710	miRNA	1	82.231	1	95.301	1	85.757	1	93.016
ENSMUSG00000076049	Mir598	miRNA	1	78.744	NA	NA	1	79.524	1	96.248
ENSMUSG00000076050	Mir450b	miRNA	1	77.497	NA	NA	1	82.903	1	94.608
ENSMUSG00000076051	Mir707	miRNA	1	85.092	1	95.504	NA	NA	1	94.654
ENSMUSG00000076060	Mir704	miRNA	1	82.092	1	95.301	1	86.112	1	94.904
ENSMUSG00000076062	Mir92-1	miRNA	1	85.283	1	96.216	1	85.464	1	95.777
ENSMUSG00000076063	Mir539	miRNA	1	80.608	1	95.504	1	79.897	1	93.734
ENSMUSG00000076064	Mir690	miRNA	1	82.231	1	95.504	1	84.724	1	95.877
ENSMUSG00000076065	Mir681	miRNA	1	75.567	1	96.138	1	86.602	1	94.153
ENSMUSG00000076066	Mir223	miRNA	1	85.092	1	96.68	1	83.857	1	87.886
ENSMUSG00000076067	Mir592	miRNA	1	83.169	1	94.597	1	85.921	1	91.928
ENSMUSG00000076068	Mir678	miRNA	1	74.8	1	95.708	1	80.5	1	96.358
ENSMUSG00000076117	Mir680-2	miRNA	1	81.52	NA	NA	NA	NA	1	93.156
ENSMUSG00000076118	Mir669c	miRNA	1	83.968	1	95.504	1	82.161	1	94.654
ENSMUSG00000076119	Mir698	miRNA	1	81.01	1	96.013	1	86.475	1	96.139
ENSMUSG00000076120	Mir551b	miRNA	1	86.843	1	96.013	1	84.83	1	92.691
ENSMUSG00000076121	Mir687	miRNA	1	84.452	1	96.897				

							1	83.811	1	90.157
ENSMUSG00000076122	Mir503	miRNA	1	85.129	1	96.68	1	86.084	1	95.777
ENSMUSG00000076123	Mir700	miRNA	1	74.8	1	96.948	1	64.024	1	91.011
ENSMUSG00000076126	Mir669b	miRNA	1	86.006	1	93.654	1	76.754	1	95.832
ENSMUSG00000076127	Mir718	miRNA	1	85.655	1	93.094	1	71.615	1	96.248
ENSMUSG00000076128	Mir686	miRNA	1	78.639	1	91.955	1	87.274	1	95.92
ENSMUSG00000076129	Gm22920	miRNA	1	85.728	NA	NA	1	88.161	1	96.248
ENSMUSG00000076135	Gm24276	miRNA	1	72	1	96.138	1	81.525	1	94.904
ENSMUSG00000076136	Gm24951	miRNA	1	74.482	1	96.216	1	87.898	1	93.612
ENSMUSG00000076138	Gm26377	miRNA	1	83.523	NA	NA	1	86.346	1	94.654
ENSMUSG00000076140	Mir542	miRNA	1	78.299	1	94.597	1	85.935	1	96.183
ENSMUSG00000076141	Mir693	miRNA	1	86.843	1	97.263	1	87.658	1	92.181
ENSMUSG00000076142	NA	NA	NA	NA	1	79.894	1	48.772	1	94.904
ENSMUSG00000076143	Mir708	miRNA	1.031	72.985	0.722	97.263	1.347	86.144	0.7	86.699
ENSMUSG00000076144	Mir709	miRNA	0.684	45.502	1.1	96.68	1.332	58.616	1.03	96.287
ENSMUSG00000076145	Mir679	miRNA	1	74.8	1	94.262	1	88.657	1	96.183
ENSMUSG00000076146	Mir449c	miRNA	1	85.59	1	93.654	1	73.874	1	96.358
ENSMUSG00000076147	Mir694	miRNA	1	83.09	1	93.654	1	70.623	1	92.018
ENSMUSG00000076163	Mir701	miRNA	1	79.93	1	95.708	1	86.985	1	92.762
ENSMUSG00000076190	Gm23256	miRNA	1	78.744	1	90.053	1	86.831	1	95.273
ENSMUSG00000076199	Gm23255	miRNA	1	77.182	1	96.216	1	81.492	1	96.086
ENSMUSG00000076212	Mir680-1	miRNA	1	86.172	1	95.708	1	86.258	1	94.904
ENSMUSG00000076214	Mir717	miRNA	1	81.196	NA	NA	1	82.685	1	95.72
ENSMUSG00000076216	Mir695	miRNA	1	77.182	1	90.744	1	52.432	1	94.99
ENSMUSG00000076217	Mir706	miRNA	1	81.01	1	95.329	1	79.625	1	96.248
ENSMUSG00000076218	Mir692-1	miRNA	1	82.543	1	96.407	1	84.308	1	96.287
ENSMUSG00000076219	Mir487b	miRNA	1	85.391	1	95.504	1	83.925	1	92.748
ENSMUSG00000076222	Mir297b	miRNA	1	81.76	1	96.216	1			

84.896 1 96.248
ENSMUSG00000076227 Gm25593 miRNA 1 82.479 1 95.329
1 80.015 1 95.676
ENSMUSG00000076228 Gm25595 miRNA 1 84.819 1 96.847
1 75.809 1 93.156
ENSMUSG00000076229 Gm25594 miRNA 1 84.865 1 96.216
1 72.853 1 94.82
ENSMUSG00000076230 Gm22758 miRNA 1 85.129 1 95.941
1 86.144 1 95.273
ENSMUSG00000076231 Gm22759 miRNA 1 81.01 1 95.708 1
85.757 1 93.964
ENSMUSG00000076232 Gm22760 miRNA 1 80.146 1 96.407
1 77.704 1 94.654
ENSMUSG00000076233 Gm22761 miRNA 1 74.482 NA NA 1
86.602 1 96.358
ENSMUSG00000076234 Gm22757 miRNA 1 86.199 1 95.196
1 87.274 1 94.153
ENSMUSG00000076236 Mir682 miRNA 1 85.59 NA NA 1
87.37 1 94.153
ENSMUSG00000076237 Gm26300 miRNA 1 81.01 1 95.301 1
83.397 1 94.82
ENSMUSG00000076238 Gm22582 miRNA 1 86.843 1 95.735
1 80.5 1 94.608
ENSMUSG00000076240 Mir702 miRNA 1 83.658 1 95.708
1 88.349 1 96.183
ENSMUSG00000076241 Mir543 miRNA 1 86.918 1 94.48 1
80.015 1 88.512
ENSMUSG00000076246 NA NA NA NA 1 95.708 1
84.504 1 87.7
ENSMUSG00000076251 Mir501 miRNA 1 86.474 1 96.216
1 84.552 1 96.358
ENSMUSG00000076252 Mir302b miRNA 1 86.819 1 94.597
1 83.538 1 94.904
ENSMUSG00000076253 Mir680-3 miRNA 1 86.464 1 93.908
1 86.144 1 96.358
ENSMUSG00000076255 Mir92b miRNA 1 86.145 1 96.216
1 71.734 1 93.316
ENSMUSG00000076256 Mir19b-1 miRNA 1 86.006 1 96.843
1 86.666 1 96.248
ENSMUSG00000076258 Gm23935 miRNA 0.832 18.795 0.921 73.766
1.308 9.301 1.27 51.901
ENSMUSG00000076269 Mir374 miRNA 1.219 72.372 0.63 95.708
1.297 62.791 0.578 87.651
ENSMUSG00000076270 Mir719 miRNA 1 83.263 1 95.708
1 79.255 1 96.086
ENSMUSG00000076271 Mir697 miRNA 0.854 61.334 0.774 91.897
1.517 40.955 0.957 95.273
ENSMUSG00000076272 Mir666 miRNA 1 82.982 1 95.708
NA NA 1 93.734
ENSMUSG00000076274 Gm25083 miRNA 1 71.915 1 96.847
1 64.024 1 93.457
ENSMUSG00000076275 Mir675 miRNA 1 74.8 NA NA 1
87.519 1 94.654
ENSMUSG00000076281 Gm24270 miRNA 0.822 10.955 0.851 83.58

1.431 5.864 1.135 74.047
ENSMUSG00000076282 Gm24271 miRNA 1 85.092 1 96.378
1 80.749 1 95.593
ENSMUSG00000076284 Gm24272 miRNA 1 85.092 1 93.529
1 84.896 1 94.904
ENSMUSG00000076286 Mir672 miRNA 1 81.196 NA NA 1
76.64 1 93.016
ENSMUSG00000076287 Gm24273 miRNA 1 84.168 1 96.216
1 87.336 1 92.181
ENSMUSG00000076288 Mir301b miRNA 1 86.006 1 87.919
1 86.084 1 95.877
ENSMUSG00000076294 Gm26154 miRNA 1 83.418 1 91.776
1 85.352 1 93.413
ENSMUSG00000076300 NA NA NA NA 1 97.263 1
83.925 1 95.273
ENSMUSG00000076303 Gm23061 miRNA 1 84.865 1 96.216
1 73.531 1 96.358
ENSMUSG00000076305 Gm23063 miRNA 1 81.823 1 93.654
1 79.255 1 95.273
ENSMUSG00000076309 Mir147 miRNA 1 84.294 1 95.708
1 84.308 1 95.74
ENSMUSG00000076312 Mir499 miRNA 1 79.068 1 92.062
1 84.434 1 91.928
ENSMUSG00000076315 Mir343 miRNA 0.989 57.544 1.074 82.904
0.942 42.621 0.813 83.724
ENSMUSG00000076316 NA NA NA NA 1 95.504 1
87.686 1 88.797
ENSMUSG00000076318 Mir216b miRNA 1 82.705 NA NA 1
79.897 1 95.74
ENSMUSG00000076320 Gm26340 miRNA 1 85.355 1 90.378
1 85.464 1 96.192
ENSMUSG00000076323 Gm26339 miRNA 1 82.479 NA NA 1
84.724 1 95.74
ENSMUSG00000076327 Gm26338 miRNA 1 74.8 1 96.763 NA
NA 1 93.734
ENSMUSG00000076332 Gm24715 miRNA 1 71.763 1 95.504
1 78.155 1 94.608
ENSMUSG00000076333 Mir599 miRNA 1 84.977 NA NA 1
84.83 1 96.287
ENSMUSG00000076335 Mir670 miRNA 1 82.479 NA NA 1
86.258 1 93.457
ENSMUSG00000076337 Gm24714 miRNA 1 82.092 1 90.744
1 84.308 1 96.183
ENSMUSG00000076343 Gm25213 miRNA 1 83.169 1 97.263
1 87.686 1 96.358
ENSMUSG00000076355 Gm23526 miRNA 1 83.658 NA NA NA
NA 1 96.358
ENSMUSG00000076357 Mir653 miRNA 1 83.418 1 96.847
NA NA 1 96.086
ENSMUSG00000076360 Gm24047 miRNA 1 85.355 1 96.984
1 83.538 1 94.904
ENSMUSG00000076361 Mir182 miRNA 1 83.418 1 93.765
1 88.274 1 96.071
ENSMUSG00000076364 Gm24049 miRNA 1 85.203 1 96.763

1 87.173 1 95.273
ENSMUSG00000076366 Gm24048 miRNA 1 74.689 1 97.155
NA NA 1 95.676
ENSMUSG00000076370 Gm22369 miRNA 1 82.479 1 96.652
NA NA 1 94.99
ENSMUSG00000076371 Gm22368 miRNA 1 79.709 NA NA NA
NA 1 96.358
ENSMUSG00000076372 Mir568 miRNA 1 85.391 NA NA 1
86.831 1 96.183
ENSMUSG00000076377 Gm22370 miRNA 1 81.657 1 92.914
1 80.163 1 93.964
ENSMUSG00000076379 Mir190 miRNA 1 70.597 1 96.138
1 87.103 1 76.467
ENSMUSG00000076382 Gm23193 miRNA 1 75.671 1 95.301
1 67.029 1 96.183
ENSMUSG00000076385 Gm24462 miRNA 1 80.408 NA NA 1
88.693 1 95.273
ENSMUSG00000076387 Mir671 miRNA 1 86.112 1 96.216
1 86.144 1 96.192
ENSMUSG00000076389 Gm23192 miRNA 1 74.482 NA NA 1
87.336 1 96.139
ENSMUSG00000076394 Gm25991 miRNA 1 86.464 NA NA 1
83.719 1 95.273
ENSMUSG00000076395 Gm25990 miRNA 1 83.726 1 93.899
1 87.744 1 96.358
ENSMUSG00000076396 Mir667 miRNA 1 74.482 1 92.111
1 86.144 1 96.358
ENSMUSG00000076398 Mir676 miRNA 1 63.126 1 96.216
1 87.336 1 92.181
ENSMUSG00000076399 Mir450-2 miRNA 1 78.639 1 96.216
1 79.554 1 95.74
ENSMUSG00000076407 Gm22689 miRNA 1 70.121 1 95.708
1 87.744 1 93.413
ENSMUSG00000076424 Gm25986 miRNA 1 84.819 1 97.155
NA NA 1 93.612
ENSMUSG00000076430 Hus1b protein_coding 0.631 73.585 NA NA
1.157 86.084 1.259 93.964
ENSMUSG00000076431 Sox4 protein_coding 0.969 49.368 0.734
74.483 1.407 15.945 0.737 83.724
ENSMUSG00000076432 Ywhaq protein_coding 0.887 0.987 1.059 72.546
1.069 7.24 0.97 95.35
ENSMUSG00000076433 BC100451 protein_coding 1.003 86.464
0.914 90.287 1.092 72.378 1.045 82.85
ENSMUSG00000076434 Wfdc3 protein_coding 1.979 10.595 1.042
74.483 0.483 71.734 1.394 72.204
ENSMUSG00000076435 Acsf2 protein_coding 0.905 8.139 1.062 83.104
1.041 50.636 0.909 85.493
ENSMUSG00000076436 Oxct2a protein_coding 0.67 34.795
0.802 93.654 1.857 30.213 1.244 90.125
ENSMUSG00000076437 2700094K13Rik protein_coding 1.029 75.196
0.975 92.137 0.995 86.258 0.933 94.99
ENSMUSG00000076438 Oxct2b protein_coding 1.785 83.523
0.414 68.115 1.348 30.948 0.83 89.152
ENSMUSG00000076441 Ass1 protein_coding 1.023 79.709 0.975

95.504 0.997 80.634 1.126 64.905
ENSMUSG00000076444 Mir761 miRNA 1 79.277 NA NA 1
88.601 1 95.72
ENSMUSG00000076445 Gm24865 miRNA 1 86.172 1 91.361
1 87.519 1 94.608
ENSMUSG00000076448 Mir763 miRNA 1 83.658 1 95.735
NA NA 1 96.287
ENSMUSG00000076450 Mir804 miRNA 1 80.815 1 96.013
1 88.076 1 94.19
ENSMUSG00000076451 Mir770 miRNA 1 85.283 1 96.68 1
86.831 1 90.865
ENSMUSG00000076453 Mir759 miRNA 1 86.333 1 94.262
1 85.935 1 94.468
ENSMUSG00000076454 Mir762 miRNA 0.904 59.358 1.072 91.776
1.033 73.309 0.872 91.985
ENSMUSG00000076455 Mir767 miRNA 1 84.661 1 97.263
1 86.666 1 92.018
ENSMUSG00000076457 Mir802 miRNA 1 82.092 NA NA 1
87.898 1 94.904
ENSMUSG00000076458 Mir764 miRNA 1 76.686 1 95.431
1 84.308 1 96.287
ENSMUSG00000076459 Mir758 miRNA 1 81.52 1 94.48 1
82.903 1 91.625
ENSMUSG00000076460 Mir744 miRNA 1 79.277 1 95.301
1 86.552 1 93.584
ENSMUSG00000076462 Trbv2 IG_LV_gene 0.256 17.705 0.84 93.654
4.627 17.751 2.744 71.653
ENSMUSG00000076463 Trbv3 IG_LV_gene 0.467 26.77 2.291 73.092
0.935 87.103 1.348 90.455
ENSMUSG00000076477 Trbv21 IG_LV_gene 1 74.147 1
95.329 1 82.161 1 95.832
ENSMUSG00000076478 Trbv23 IG_LV_gene 1 85.499 1
96.216 1 80.015 1 95.35
ENSMUSG00000076479 Trbv26 IG_LV_gene 1 85.355 1
92.137 1 79.625 1 93.964
ENSMUSG00000076481 Trbv30 IG_LV_gene 1 75.761 NA NA
1 87.744 1 96.071
ENSMUSG00000076485 Trbj1-3 IG_J_gene 0.949 64.893 0.976
89.014 1.076 78.642 1.027 92.762
ENSMUSG00000076486 Trbj1-4 IG_J_gene 1.052 85.59 0.438 83.321
2.173 70.623 1.487 90.463
ENSMUSG00000076487 Trbj1-5 IG_J_gene 0.495 49.944 2.362
82.76 0.857 78.954 1.045 96.086
ENSMUSG00000076490 Trbc1 IG_C_gene 0.872 71.915 1.262 80.678
0.905 25.688 0.869 85.493
ENSMUSG00000076495 Trbj2-4 IG_J_gene 1 76.686 1
96.308 1 73.428 1 93.964
ENSMUSG00000076496 Trbj2-5 IG_J_gene 1 85.203 1
96.843 1 86.346 1 91.928
ENSMUSG00000076498 Trbc2 IG_C_gene 0.869 86.74 1.17 90.29 0.987
63.075 0.797 84.29
ENSMUSG00000076500 Gm20730 IG_LV_gene 1 73.585 1
96.948 1 81.525 1 94.904
ENSMUSG00000076505 Igkv1-131 IG_LV_gene 1 84.738 1

95.735 1 84.626 1 96.248
ENSMUSG00000076512 Igkv9-123 IG_LV_gene 1 71.516 NA NA
1 76.64 1 93.264
ENSMUSG00000076518 Igkv2-112 IG_LV_gene 1 67.163 1
87.857 1 86.666 1 92.691
ENSMUSG00000076525 Igkv1-99 IG_LV_gene 1 80.751 1
96.843 1 86.666 1 96.183
ENSMUSG00000076526 Igkv12-98 IG_LV_gene 1 71.217 1
94.597 1 81.62 1 88.505
ENSMUSG00000076530 Igkv19-93 pseudogene 1 81.196 1
93.765 1 87.744 1 93.156
ENSMUSG00000076531 Igkv4-92 IG_LV_gene 1 85.283 NA NA
1 79.426 1 95.676
ENSMUSG00000076533 Igkv4-90 IG_LV_gene 1 86.819 1
91.661 1 71.232 1 95.35
ENSMUSG00000076538 Igkv13-84 IG_LV_gene 1 75.858 1
94.597 1 86.475 1 95.74
ENSMUSG00000076539 Igkv4-81 IG_LV_gene 1 75.858 1
93.765 1 85.792 1 93.156
ENSMUSG00000076540 Igkv4-80 IG_LV_gene 1 65.733 NA NA
1 60.614 1 85.493
ENSMUSG00000076547 Igkv4-70 IG_LV_gene 1 74.482 1
95.735 1 84.229 1 95.273
ENSMUSG00000076548 Igkv4-69 protein_coding 1 66.272 1
96.843 1 71.734 1 87.992
ENSMUSG00000076550 NA NA NA NA 1 94.262 1
88.076 1 88.755
ENSMUSG00000076557 Igkv4-56 IG_LV_gene 1 84.534 1
96.216 1 88.601 1 95.273
ENSMUSG00000076570 Igkv12-38 IG_LV_gene 1 86.006 1
96.843 1 76.225 1 95.72
ENSMUSG00000076571 Igkv5-37 IG_LV_gene 1 71.896 1
96.216 1 69.196 1 89.892
ENSMUSG00000076572 Igkv18-36 IG_LV_gene 1 85.355 1
97.155 1 88.693 1 96.248
ENSMUSG00000076573 Igkv1-35 IG_LV_gene 1 74.299 1
95.708 1 87.786 1 96.248
ENSMUSG00000076575 Igkv7-33 IG_LV_gene 1 72.655 1
96.216 1 74.535 1 96.358
ENSMUSG00000076578 Igkv6-29 IG_LV_gene 1 74.147 NA NA
1 86.144 1 94.297
ENSMUSG00000076581 Igkv8-26 IG_LV_gene 1 86.172 1
95.504 1 86.144 1 96.358
ENSMUSG00000076589 Igkv8-18 IG_LV_gene 1 86.74 1 91.661
1 72.853 1 95.74
ENSMUSG00000076594 Igkv6-13 IG_LV_gene 1 37.941 NA NA
1 48.577 1 86.699
ENSMUSG00000076609 Igkc IG_C_gene 1.94 10.464 1.497 83.104
0.343 0.72 0.939 94.904
ENSMUSG00000076617 Ighm IG_C_gene 1.114 74.745 1.382 79.261
0.646 35.815 0.964 91.625
ENSMUSG00000076618 Ighj4 IG_J_gene 1.327 54.717 0.881 82.671
0.851 88.161 0.827 85.493
ENSMUSG00000076619 Ighj3 IG_J_gene 1.28 51.189 0.684 73.461

1.14 87.898 0.679 84.29
ENSMUSG00000076620 Ighj2 IG_J_gene 1.251 62.689 0.819 92.355
0.971 86.112 0.657 83.724
ENSMUSG00000076621 Ighj1 IG_J_gene 1.458 21.599 0.733 60.678
0.934 84.83 0.739 90.691
ENSMUSG00000076630 Ighd1-1 IG_D_gene 1 58.198 NA NA
1 84.83 1 94.904
ENSMUSG00000076632 Gm16968 IG_D_gene 1 84.564 1
95.196 1 78.713 1 95.273
ENSMUSG00000076646 Ighv2-6-8 IG_LV_gene 1 79.709 NA NA
1 86.602 1 90.95
ENSMUSG00000076653 Ighv7-2 IG_LV_gene 1 73.292 1
94.848 1 81.857 1 93.964
ENSMUSG00000076661 Ighv16-1 IG_LV_gene 1 86.172 1
96.138 1 87.898 1 95.559
ENSMUSG00000076668 Ighv7-4 IG_LV_gene 1 83.658 1
94.767 1 88.274 1 96.358
ENSMUSG00000076670 Ighv3-5 IG_LV_gene 1 75.858 1
96.216 1 79.119 1 94.567
ENSMUSG00000076671 Ighv13-1 IG_V_gene 1 80.03 NA NA 1
84.434 1 96.183
ENSMUSG00000076674 Ighv3-8 IG_LV_gene 1 81.196 NA NA
1 79.897 1 96.358
ENSMUSG00000076676 Ighv12-3 IG_LV_gene 1 84.564 1
83.104 1 57.091 1 95.35
ENSMUSG00000076680 Ighv6-6 IG_LV_gene 1 68.846 1
95.735 1 86.552 1 89.498
ENSMUSG00000076688 Ighv15-2 IG_LV_gene 1 79.709 1
96.763 1 84.724 1 94.99
ENSMUSG00000076694 Ighv1-30 IG_LV_gene 1 68.353 1
92.355 NA NA 1 96.183
ENSMUSG00000076695 Ighv1-18 IG_LV_gene 1 67.163 1
96.68 1 53.224 1 84.29
ENSMUSG00000076709 Ighv1-47 IG_LV_gene 1 83.09 1 95.088
NA NA 1 91.928
ENSMUSG00000076710 Ighv1-49 IG_LV_gene 1 84.324 NA NA
1 83.607 1 96.287
ENSMUSG00000076731 Ighv8-12 IG_LV_gene 1 82.982 1
88.705 1 86.475 1 93.964
ENSMUSG00000076733 Ighv8-13 IG_LV_gene 1 82.543 1
94.767 1 83.275 1 95.273
ENSMUSG00000076744 Tcrg-V7 IG_LV_gene 1 85.355 NA NA
1 84.308 1 92.181
ENSMUSG00000076747 Tcrg-V5 IG_LV_gene 1 74.958 1
93.899 1 88.729 1 96.086
ENSMUSG00000076749 Tcrg-C1 IG_C_gene 2.407 45.307 0.85
96.013 0.489 44.678 0.892 91.372
ENSMUSG00000076750 Tcrg-V3 IG_LV_gene 1 86.199 1
96.268 1 88.693 1 95.74
ENSMUSG00000076752 Tcrg-C2 IG_C_gene 0.924 73.124 1.154
95.941 0.936 84.071 0.942 90.854
ENSMUSG00000076755 Gm16602 IG_LV_gene 1 83.263 1
91.456 1 86.346 1 96.183
ENSMUSG00000076757 Tcrg-C4 IG_C_gene 3.031 16.249 0.543

81.443 0.609 49.967 1.177 86.991
ENSMUSG00000076758 Trav1 TR_V_gene 1 86.843 1 94.887
1 88.601 1 93.964
ENSMUSG00000076760 Trav3-1 IG_LV_gene 1 86.74 1 96.68 1
83.397 1 92.181
ENSMUSG00000076761 Trav5-1 IG_LV_gene 1 85.728 1
96.407 1 87.971 1 92.31
ENSMUSG00000076762 Trav6-1 IG_LV_gene 1 79.068 1
96.68 1 86.346 1 95.273
ENSMUSG00000076764 Trav6-2 IG_LV_gene 1 78.299 1
96.948 1 80.899 1 96.042
ENSMUSG00000076770 Trav8d-1 TR_V_gene 1 85.728 1
96.216 1 83.857 1 95.877
ENSMUSG00000076776 Trav7d-5 TR_V_gene 1 61.171 1
95.504 1 85.675 1 96.287
ENSMUSG00000076778 Trav13d-1 TR_V_gene 1 86.112 1
91.361 NA NA 1 93.316
ENSMUSG00000076795 Trav8d-2 IG_LV_gene 1 71.763 1
96.216 1 87.103 1 93.132
ENSMUSG00000076796 Trav16d-dv11 IG_LV_gene 1 82.705 NA
NA 1 86.666 1 78.948
ENSMUSG00000076800 Trav6n-5 IG_LV_gene 1 83.658 1
95.431 NA NA 1 93.132
ENSMUSG00000076802 Trav6n-6 IG_LV_gene 1 83.418 1
95.504 1 84.504 1 93.612
ENSMUSG00000076821 Trav8n-2 IG_LV_gene 1 48.462 1
95.708 1 48.577 1 92.181
ENSMUSG00000076822 Trav16n IG_LV_gene 1 66.944 1
96.407 1 65.688 1 86.699
ENSMUSG00000076823 Trav13n-4 IG_LV_gene 1 82.231 1
93.529 NA NA 1 96.183
ENSMUSG00000076826 Trav4-2 TR_V_gene 1 85.637 1
95.504 1 86.831 1 91.928
ENSMUSG00000076831 Trav8-1 TR_V_gene 1 56.085 1
94.48 1 52.972 1 94.19
ENSMUSG00000076840 Trav14-1 IG_LV_gene 1 79.277 1
88.704 1 85.935 1 96.071
ENSMUSG00000076848 Trav15-2-dv6-2 TR_V_gene 1 82.092 NA
NA 1 82.74 1 96.287
ENSMUSG00000076855 Trav16 TR_V_gene 1 81.465 NA NA
1 88.274 1 92.903
ENSMUSG00000076858 Trav3-4 TR_V_gene 1 85.499 1
94.848 1 79.119 1 96.287
ENSMUSG00000076861 Trav18 TR_V_gene 1 82.231 NA NA
1 88.274 1 92.748
ENSMUSG00000076862 Trav19 TR_V_gene 1 77.291 1
95.708 1 87.274 1 93.685
ENSMUSG00000076863 Trav21-dv12 TR_V_gene 1 84.819 1
94.848 1 86.144 1 96.287
ENSMUSG00000076864 Trdv1 TR_V_gene 1 75.567 1 91.532
1 84.308 1 94.904
ENSMUSG00000076865 Trdv2-1 IG_LV_gene 1 85.832 1
97.263 1 83.538 1 95.74
ENSMUSG00000076867 Trdv4 IG_LV_gene 0.709 30.352 0.671 87.857

2.094 16.446 0.419 84.29
ENSMUSG00000076871 Trdj2 IG_J_gene 1 77.182 1 96.216
1 85.134 1 95.273
ENSMUSG00000076873 Trdv5 IG_LV_gene 1 69.777 1 93.994
1 54.964 1 90.289
ENSMUSG00000076874 Traj59 IG_J_gene 1 83.333 1
93.765 1 86.602 1 94.82
ENSMUSG00000076875 Traj58 IG_J_gene 1 75.399 1
93.765 1 83.066 1 93.156
ENSMUSG00000076876 Traj57 IG_J_gene 1 81.24 1 96.407
1 87.686 1 93.132
ENSMUSG00000076877 Traj56 IG_J_gene 1 86.474 1
96.847 1 87.786 1 94.904
ENSMUSG00000076878 Traj53 IG_J_gene 1 82.891 1
95.088 1 88.693 1 96.287
ENSMUSG00000076879 Traj52 IG_J_gene 1 84.977 1
97.155 1 87.519 1 96.248
ENSMUSG00000076880 Traj51 IG_J_gene 1 85.391 NA NA
1 87.744 1 95.877
ENSMUSG00000076881 Traj50 IG_J_gene 1 75.298 1
96.216 1 63.801 1 95.877
ENSMUSG00000076882 Traj49 IG_J_gene 1 85.283 1
95.941 1 82.31 1 92.748
ENSMUSG00000076883 Traj48 IG_J_gene 1 79.577 1
94.597 1 84.961 1 96.192
ENSMUSG00000076884 Traj46 IG_J_gene 1 86.112 1
96.68 1 87.744 1 96.139
ENSMUSG00000076885 Traj45 IG_J_gene 1 82.092 1
97.129 1 87.786 1 92.31
ENSMUSG00000076886 Traj44 IG_J_gene 1 77.113 NA NA
1 85.935 1 92.018
ENSMUSG00000076887 Traj43 IG_J_gene 1 72 1 95.211
1 76.225 NA NA
ENSMUSG00000076888 Traj42 IG_J_gene 1 76.042 1
96.407 1 79.897 1 95.273
ENSMUSG00000076889 Traj41 IG_J_gene 1 84.977 NA NA
1 74.325 1 95.559
ENSMUSG00000076890 Traj40 IG_J_gene 1 84.977 NA NA
1 84.071 1 95.877
ENSMUSG00000076891 Traj39 IG_J_gene 1 84.397 1
93.574 NA NA 1 95.72
ENSMUSG00000076892 Traj38 IG_J_gene 1 82.092 1
97.263 1 87.103 1 96.086
ENSMUSG00000076893 Traj37 IG_J_gene 1 78.639 1
94.887 1 81.169 1 94.19
ENSMUSG00000076894 NA NA NA NA 1 96.216 1
82.778 1 96.086
ENSMUSG00000076895 Traj34 IG_J_gene 1 84.397 1
97.155 1 84.434 1 94.904
ENSMUSG00000076896 Traj33 IG_J_gene 1 86.351 NA NA
NA NA 1 95.273
ENSMUSG00000076897 Traj32 IG_J_gene 1 64.956 1
90.576 1 86.475 1 94.016
ENSMUSG00000076898 Traj31 IG_J_gene 1 83.263 NA NA

1 81.857 1 95.74
ENSMUSG00000076899 Traj30 IG_J_gene 1 81.178 1
96.013 1 80.749 1 93.612
ENSMUSG00000076900 Traj29 IG_J_gene 1 84.294 1
95.504 1 87.37 1 93.722
ENSMUSG00000076901 Traj28 IG_J_gene 1 86.145 1
94.597 1 68.82 1 96.358
ENSMUSG00000076903 Traj26 IG_J_gene 1 72 NA NA 1
87.744 1 94.608
ENSMUSG00000076904 Traj25 IG_J_gene 1 83.726 1
96.897 1 84.724 1 93.156
ENSMUSG00000076905 Traj24 IG_J_gene 1 85.092 1
91.661 1 82.685 1 96.086
ENSMUSG00000076906 Traj23 IG_J_gene 1 80.751 1
92.404 1 83.925 1 96.287
ENSMUSG00000076907 Traj22 IG_J_gene 1 86.843 1
95.809 1 87.103 1 94.904
ENSMUSG00000076908 Traj21 IG_J_gene 1 74.516 1
95.504 1 85.921 1 94.904
ENSMUSG00000076909 Traj20 IG_J_gene 1 83.169 1
96.268 1 68.425 1 94.904
ENSMUSG00000076910 Traj19 IG_J_gene 1 82.543 1
96.138 1 65.8 1 95.72
ENSMUSG00000076911 Traj18 IG_J_gene 1 76.968 1
88.691 1 67.823 1 95.877
ENSMUSG00000076912 Traj17 IG_J_gene 1 78.538 1
96.847 1 69.825 1 89.525
ENSMUSG00000076913 Traj16 IG_J_gene 1 80.51 NA NA 1
88.274 1 89.152
ENSMUSG00000076914 Traj15 IG_J_gene 1 83.169 1
96.216 1 78.864 1 96.248
ENSMUSG00000076915 Traj14 IG_J_gene 1 86.006 1
97.155 1 75.527 1 92.381
ENSMUSG00000076916 Traj13 IG_J_gene 1 74.8 1 97.129
1 84.896 1 89.428
ENSMUSG00000076917 Traj12 IG_J_gene 1 85.655 1
95.708 1 70.534 1 96.086
ENSMUSG00000076918 Traj11 IG_J_gene 1 80.251 1
93.654 NA NA 1 96.192
ENSMUSG00000076919 Traj9 IG_J_gene 1 82.705 1 96.763
1 83.538 1 93.685
ENSMUSG00000076920 Traj8 IG_J_gene 1 78.054 1 95.431
1 77.532 1 95.273
ENSMUSG00000076921 Traj7 IG_J_gene 1 81.925 1 95.504
1 86.112 1 96.192
ENSMUSG00000076922 Traj6 IG_J_gene 1 81.647 1 95.708
NA NA 1 96.183
ENSMUSG00000076923 Traj5 IG_J_gene 1 85.728 NA NA 1
85.464 1 83.581
ENSMUSG00000076924 Traj4 IG_J_gene 1 45.746 1 95.504
1 81.62 1 90.628
ENSMUSG00000076925 Traj3 IG_J_gene 1 86.918 1 97.097
1 77.888 1 96.358
ENSMUSG00000076927 Traj1 IG_J_gene 1 81.01 1 96.68 1

79.897 1 92.748
ENSMUSG00000076928 Trac IG_C_gene 0.861 72.85 1.394 90.29 0.834
40.338 0.863 86.699
ENSMUSG00000076941 Gm22065 miRNA 1 84.534 1 97.155
1 79.625 1 95.273
ENSMUSG00000076946 Mir582 miRNA 1 53.407 NA NA 1
87.686 1 94.752
ENSMUSG00000076948 Mir467e miRNA 1 74.482 NA NA 1
84.308 1 95.273
ENSMUSG00000076949 Gm22066 miRNA 1 76.686 1 92.474
1 84.724 1 94.82
ENSMUSG00000076950 Gm24933 miRNA 1 74.689 1 95.735
1 85.792 1 95.273
ENSMUSG00000076951 Gm24932 miRNA 1 73.487 1 94.887
1 88.729 1 95.777
ENSMUSG00000076952 Gm24934 miRNA 1 82.092 1 96.843
1 86.112 1 96.192
ENSMUSG00000076956 Mir297a-3 miRNA 1 74.482 1 92.404
1 83.066 1 91.525
ENSMUSG00000076958 Gm24935 miRNA 1 86.819 1 95.941
1 80.634 1 96.287
ENSMUSG00000076959 Mir669l miRNA 1 85.655 1 93.094
1 81.857 1 93.722
ENSMUSG00000076961 Gm23251 miRNA 0.773 64.284 1.098 86.793
1.178 85.21 0.94 89.827
ENSMUSG00000076964 Gm23252 miRNA 1 71.142 NA NA 1
88.349 1 96.183
ENSMUSG00000076966 Mir466b-2 miRNA 1 85.637 1 95.504
1 77.532 1 96.358
ENSMUSG00000076972 Mir193b miRNA 1 66 1 95.301 1
86.727 1 93.156
ENSMUSG00000076976 Mir297c miRNA 1 77.916 1 95.329
1 78.293 1 93.612
ENSMUSG00000076977 Gm26067 miRNA 1 81.178 1 96.407
1 81.525 1 94.174
ENSMUSG00000076983 Gm24114 miRNA 1 85.857 1 96.268
1 88.693 NA NA
ENSMUSG00000076987 Gm24115 miRNA 1 74.958 1 97.263
1 86.985 1 95.74
ENSMUSG00000076994 Mir466b-3 miRNA 1 82.092 NA NA 1
76.768 1 95.273
ENSMUSG00000076999 Gm22426 miRNA 1 77.916 1 88.345
1 73.428 1 96.358
ENSMUSG00000077001 Mir544 miRNA 1 85.655 NA NA 1
87.744 1 95.559
ENSMUSG00000077004 Gm23876 miRNA 1 85.655 NA NA 1
87.37 1 96.183
ENSMUSG00000077010 Gm22189 miRNA 1 81.52 1 95.708 NA
NA 1 96.287
ENSMUSG00000077015 Gm22190 miRNA 1 76.827 1 97.049
1 87.786 1 95.74
ENSMUSG00000077019 Gm22191 miRNA 1 84.263 NA NA 1
81.032 1 93.264
ENSMUSG00000077021 Mir467d miRNA 1 83.169 1 96.407

1 84.896 1 96.358
ENSMUSG00000077022 Gm22707 miRNA 1 80.815 NA NA 1
80.015 1 93.734
ENSMUSG00000077038 Mir669f miRNA 1 81.01 1 96.308 NA
NA 1 95.35
ENSMUSG00000077039 Gm25537 miRNA 1 83.823 1 94.767
1 88.693 1 92.748
ENSMUSG00000077040 Gm26209 miRNA 1 78.054 1 97.263
1 86.084 1 93.964
ENSMUSG00000077042 Mir574 miRNA 1 82.982 1 96.763
1 83.857 1 94.153
ENSMUSG00000077049 Mir467c miRNA 1 85.355 1 96.138
1 84.308 1 96.358
ENSMUSG00000077050 Gm24544 miRNA 1 81.76 1 97.097 1
88.601 1 96.192
ENSMUSG00000077055 Gm24543 miRNA 1 83.726 1 96.897
1 87.658 1 96.358
ENSMUSG00000077058 Gm24542 miRNA 1 82.231 1 97.097
1 75.637 1 95.74
ENSMUSG00000077059 Gm24541 miRNA 1 83.523 1 96.897
1 87.898 1 92.181
ENSMUSG00000077065 Gm25040 miRNA 1 76.827 1 97.263
1 67.339 1 96.071
ENSMUSG00000077081 Gm23701 miRNA 1 79.853 NA NA 1
81.976 1 95.559
ENSMUSG00000077082 Gm23766 miRNA 1 84.168 1 96.013
1 80.634 1 93.722
ENSMUSG00000077085 Gm23697 miRNA 1 81.01 1 95.708 1
84.961 1 92.762
ENSMUSG00000077086 Mir669o miRNA 1 78.224 1 95.211
1 87.173 1 95.877
ENSMUSG00000077090 Gm22117 miRNA 1 84.263 1 91.065
1 83.607 1 95.74
ENSMUSG00000077092 Mir742 miRNA 1 72.655 1 96.68 1
87.786 1 95.273
ENSMUSG00000077095 Gm24342 miRNA 1 85.857 NA NA 1
84.83 1 96.248
ENSMUSG00000077096 Gm22015 miRNA 1 79.165 1 96.216
1 88.601 1 96.184
ENSMUSG00000077104 Gm25263 miRNA 1 86.843 NA NA 1
88.41 1 94.468
ENSMUSG00000077107 Mir344c miRNA 1 82.231 1 96.216
1 85.921 1 96.139
ENSMUSG00000077110 Gm22427 miRNA 1 79.165 NA NA 1
87.37 1 94.825
ENSMUSG00000077111 Mir18b miRNA 1 82.982 NA NA 1
83.158 1 96.358
ENSMUSG00000077113 Mir466p miRNA 1 80.688 1 96.843
1 79.554 1 96.183
ENSMUSG00000077119 Mir741 miRNA 1 85.77 1 97.155 1
84.896 1 95.593
ENSMUSG00000077125 Gm26399 miRNA 1 81.823 1 91.897
1 81.032 1 95.74
ENSMUSG00000077127 Mir743 miRNA 1 84.324 NA NA 1

85.935	1	96.086
ENSMUSG00000077131	Gm23578	miRNA 1	80.688	NA	NA	NA
NA	1	96.287
ENSMUSG00000077138	Gm23577	miRNA 1	84.263	1	95.708
1	83.925	1	94.904
ENSMUSG00000077142	Gm22937	snoRNA	1	84.397	1
96.138	1	88.41 1	95.877
ENSMUSG00000077143	Gm22936	snoRNA	1	86.199	1
97.263	1	83.158	1	92.181
ENSMUSG00000077144	Gm22938	snoRNA	1	85.637	1
95.504	1	76.388	1	93.722
ENSMUSG00000077146	Gm22939	snoRNA	1	81.125	1
92.404	1	82.161	1	93.612
ENSMUSG00000077148	Gm22935	snRNA 0.981 83.418	0.622 85.075
1.64	38.926	1.328 91.492
ENSMUSG00000077149	Gm22934	snoRNA	1	80.688	1
97.155	NA	NA	1	94.654
ENSMUSG00000077150	Gm24581	snoRNA	1	82.982	1
95.088	1	84.896	1	94.752
ENSMUSG00000077152	Gm24579	snoRNA	1	81.52 NA	NA	1
84.724	1	95.273
ENSMUSG00000077153	Gm24580	snoRNA	1	83.823	NA	NA
1	87.658	1	96.183
ENSMUSG00000077155	Gm24582	snRNA 1	73.487	1	94.597
1	84.229	1	94.297
ENSMUSG00000077156	n-R5s90	rRNA	1	70.36 1	95.088	1
88.076	1	96.183
ENSMUSG00000077158	Gm24578	snoRNA	1	81.647	1
96.407	1	76.64 1	94.297
ENSMUSG00000077160	Gm24118	snoRNA	1	81.465	1
96.138	1	76.923	1	94.215
ENSMUSG00000077161	Gm24117	snoRNA	1	81.647	1
96.268	1	88.161	1	93.156
ENSMUSG00000077163	Gm24116	snRNA 1	85.637	1	95.211
1	80.749	1	96.192
ENSMUSG00000077166	Gm24120	snoRNA	1	79.277	1
95.504	1	87.898	1	96.287
ENSMUSG00000077167	Gm24119	snoRNA	0.942 50.519	0.955
96.268	1.111 54.809	0.817 93.584
ENSMUSG00000077169	Gm25396	snoRNA	1	74.299	1
96.984	1	84.504	1	93.584
ENSMUSG00000077171	Gm25761	snoRNA	1	86.57 1	95.504
1	87.274	1	95.559
ENSMUSG00000077172	Gm25762	snoRNA	1	69.408	1
96.216	1	88.693	1	91.372
ENSMUSG00000077174	Gm25757	snoRNA	1	84.294	1
95.301	NA	NA	1	95.273
ENSMUSG00000077175	Gm25758	snoRNA	1	80.751	1
93.707	1	87.658	1	93.964
ENSMUSG00000077176	Gm25759	snoRNA	1	80.608	1
96.843	1	76.923	1	96.358
ENSMUSG00000077177	Gm25760	snoRNA	1	81.465	1
96.843	NA	NA	1	93.264
ENSMUSG00000077178	Gm25756	snoRNA	1	72.655	1

96.843 1 86.831 1 95.593
ENSMUSG00000077180 Gm25094 snoRNA 1 80.688 1
94.848 1 86.144 1 95.676
ENSMUSG00000077181 Gm25093 snoRNA 1 76.319 NA NA
1 83.925 1 95.593
ENSMUSG00000077184 Gm25092 snoRNA 1 86.172 1
96.138 1 84.626 1 95.72
ENSMUSG00000077185 Gm25091 snoRNA 1 84.324 NA NA
NA NA 1 96.287
ENSMUSG00000077188 Gm25090 snoRNA 1 80.03 1 97.155
1 85.792 1 94.825
ENSMUSG00000077190 Gm22269 snoRNA 1 81.01 1 96.138
1 87.658 1 90.865
ENSMUSG00000077191 Snord64 snoRNA 0.707 46.693 0.687
95.504 2.06 34.285 2.667 60.968
ENSMUSG00000077192 Snora17 snoRNA 1 86.658 NA NA
1 78.498 1 94.825
ENSMUSG00000077194 Gm23431 snRNA 1 86.658 1 94.848
1 88.601 1 93.117
ENSMUSG00000077196 Gm22267 snoRNA 1 79.93 1 95.708
1 79.776 1 94.904
ENSMUSG00000077197 Gm22268 snoRNA 1 66.897 1
96.013 1 87.274 1 90.289
ENSMUSG00000077199 Gm22266 snoRNA 1 79.853 1
94.48 1 86.666 1 95.74
ENSMUSG00000077200 Gm25613 snoRNA 1 85.092 1
94.48 1 85.58 1 95.877
ENSMUSG00000077201 Gm25614 snoRNA 1 84.049 1
96.897 1 87.274 1 96.183
ENSMUSG00000077202 Gm25612 snoRNA 1.222 59.211 1.457
82.904 0.56 27.994 0.881 91.291
ENSMUSG00000077204 Gm25610 snoRNA 1 81.01 1 93.529
1 84.83 1 95.593
ENSMUSG00000077205 Gm25611 snoRNA 1 74.745 1
94.887 1 82.778 1 96.183
ENSMUSG00000077207 Gm25609 snRNA 1 84.819 1 96.652
1 86.258 1 96.287
ENSMUSG00000077208 Gm25607 snoRNA 1 66.129 1
86.387 1 88.461 1 96.086
ENSMUSG00000077209 Gm25608 snoRNA 1 83.658 1
92.931 1 88.657 1 96.071
ENSMUSG00000077212 Snord69 snoRNA 1.354 34.696 0.743
68.848 NA NA 0.95 94.904
ENSMUSG00000077214 Gm23950 snoRNA 1 74.689 1
96.216 1 87.744 1 92.742
ENSMUSG00000077215 Gm23949 snoRNA 1 73.124 1
96.216 1 88.601 1 96.183
ENSMUSG00000077217 Gm23951 snoRNA 1 84.534 1
95.211 1 81.857 1 96.287
ENSMUSG00000077218 Gm23948 snoRNA 1 84.452 1
95.708 NA NA 1 93.722
ENSMUSG00000077220 Gm23436 snoRNA 1 84.324 1
95.504 1 85.921 1 95.832
ENSMUSG00000077221 Snord67 snoRNA 1 82.231 1

95.708 1 80.015 1 94.752
ENSMUSG00000077222 Gm22270 snoRNA 1.114 75.671 0.922
94.597 0.968 85.757 1.032 81.56
ENSMUSG00000077223 Gm22271 snoRNA 1 84.865 1
96.216 NA NA 1 92.12
ENSMUSG00000077224 Gm23437 snoRNA 1 81.925 1
96.407 1 88.657 1 94.904
ENSMUSG00000077227 Gm22272 snoRNA 1 86.351 NA NA
1 84.83 1 91.105
ENSMUSG00000077228 Gm22273 snoRNA 1 81.823 NA NA
1 88.076 1 95.273
ENSMUSG00000077230 Gm25097 snRNA 1 84.294 1 95.431
1 87.431 1 95.74
ENSMUSG00000077231 Gm25096 snoRNA 1 83.169 1
94.848 1 81.388 1 95.92
ENSMUSG00000077235 Gm25095 snoRNA 1 78.054 1
94.848 1 84.724 1 96.086
ENSMUSG00000077236 Gm22439 snoRNA 1 78.054 1
95.708 1 86.144 NA NA
ENSMUSG00000077237 Gm22438 snoRNA 1 85.832 1
95.708 1 88.601 1 96.248
ENSMUSG00000077239 Snord66 snoRNA 0.73 66.129 0.56
95.088 2.453 71.232 1.752 91.872
ENSMUSG00000077240 Gm23271 snoRNA 1 85.283 1
97.155 1 75.809 1 95.559
ENSMUSG00000077241 Gm23272 snoRNA 1 74.482 NA NA
1 87.519 1 92.748
ENSMUSG00000077243 Gm23270 snoRNA 1 84.819 1
95.708 1 87.103 1 94.752
ENSMUSG00000077244 NA NA NA NA 1 96.407 1
85.352 1 95.74
ENSMUSG00000077246 Gm23273 snoRNA 1 81.125 1
96.138 1 84.626 1 96.358
ENSMUSG00000077248 Gm23275 snRNA 1 83.418 1 95.809
1 84.626 1 93.722
ENSMUSG00000077250 Gm26075 snoRNA 1 41.33 1 83.104
1 83.719 1 92.389
ENSMUSG00000077251 Gm26074 snoRNA 1 82.231 1
96.652 1 80.749 1 96.183
ENSMUSG00000077252 Gm26077 snoRNA 1 85.283 1
96.308 1 87.744 1 95.74
ENSMUSG00000077253 Gm26076 snRNA 1 84.294 1 96.216
1 77.17 1 94.825
ENSMUSG00000077254 Gm26079 snoRNA 1 85.203 1
92.137 1 78.864 1 94.297
ENSMUSG00000077255 Gm26078 snoRNA 1 86.112 1
95.708 1 76.768 1 95.72
ENSMUSG00000077256 Gm26081 snoRNA 1 76.827 1
91.955 1 86.112 1 96.192
ENSMUSG00000077257 Gm26080 snoRNA 1 84.865 1
96.378 NA NA 1 95.832
ENSMUSG00000077258 Gm26082 snoRNA 1 79.165 1
96.897 1 84.83 1 95.676
ENSMUSG00000077259 Gm23979 snoRNA 1 83.523 NA NA

1 86.258 1 96.071
ENSMUSG00000077260 Gm24869 snoRNA 1 77.407 1
96.216 1 81.525 1 95.273
ENSMUSG00000077261 Gm24431 snoRNA 1 76.968 NA NA
1 86.831 1 95.74
ENSMUSG00000077262 NA NA NA NA 1 96.216 1
86.258 1 92.018
ENSMUSG00000077263 Gm24432 snoRNA 1 80.408 NA NA
1 84.626 1 93.413
ENSMUSG00000077264 Gm24429 snoRNA 1 86.006 NA NA
1 83.538 1 96.183
ENSMUSG00000077268 Gm24433 snoRNA 1 83.523 1
95.708 1 85.921 1 95.676
ENSMUSG00000077269 Gm24434 snoRNA 1 78.538 1
96.138 1 86.727 1 95.405
ENSMUSG00000077270 Gm22782 snoRNA 1 80.251 1
91.661 1 79.625 1 93.413
ENSMUSG00000077272 Gm22780 snoRNA 1 80.815 NA NA
NA NA 1 95.405
ENSMUSG00000077273 Gm22779 snRNA 1 81.125 NA NA NA
NA 1 92.748
ENSMUSG00000077274 Gm22786 snoRNA 1 83.658 NA NA
1 88.601 1 86.445
ENSMUSG00000077275 Gm22785 snoRNA 1 84.049 1
96.847 1 85.757 1 94.215
ENSMUSG00000077276 Gm22784 snoRNA 1 79.93 1 95.708
1 88.076 1 96.183
ENSMUSG00000077277 Gm22783 snRNA 1 83.726 1 88.897
1 82.74 1 95.74
ENSMUSG00000077278 Gm22778 snoRNA 1 84.819 1
93.654 1 51.851 1 87.134
ENSMUSG00000077279 Gm22777 snoRNA 1 81.196 1
97.155 1 87.686 1 96.287
ENSMUSG00000077280 Gm25764 snoRNA 1 84.294 1
96.138 NA NA 1 95.832
ENSMUSG00000077281 Gm26396 snoRNA 1 86.333 NA NA
1 85.58 1 95.74
ENSMUSG00000077282 Gm26395 snoRNA 1 82.732 1
97.129 NA NA 1 94.608
ENSMUSG00000077283 n-R5s158 rRNA 1 75.567 1 92.793
1 87.173 1 92.31
ENSMUSG00000077287 Gm25763 snoRNA 1 67.163 NA NA
1 81.29 1 94.82
ENSMUSG00000077288 Gm25765 snoRNA 1 82.732 1
96.68 1 80.899 1 96.086
ENSMUSG00000077289 Gm25766 snoRNA 1 80.608 1
95.708 1 88.729 1 94.654
ENSMUSG00000077290 Gm25407 snoRNA 1 74.482 1
95.504 1 86.084 1 92.181
ENSMUSG00000077291 Gm24124 snoRNA 1 83.169 1
97.263 NA NA 1 96.071
ENSMUSG00000077292 Gm25408 snoRNA 1 81.24 1 94.848
1 81.29 1 95.273
ENSMUSG00000077293 Gm24125 snoRNA 1 86.658 1

96.763	1	79.426	1	95.777
ENSMUSG00000077294	Gm24123	snoRNA	1	86.464	1
96.897	NA	NA	1	96.358
ENSMUSG00000077295	Gm24122	snoRNA	1	78.224	1
97.049	1	85.935	1	95.74
ENSMUSG00000077298	Gm24127	snoRNA	1	68.485	1
95.329	1	81.032	1	93.457
ENSMUSG00000077299	Gm24126	snRNA	1	86.199	1	96.013
1	87.744	1	95.74
ENSMUSG00000077302	Gm22473	snoRNA	1	72.27	1	96.268
1	88.41	1	96.192
ENSMUSG00000077303	Gm22472	snRNA	1	84.168	NA	NA	1
83.607	1	86.991
ENSMUSG00000077304	Gm22471	snoRNA	1	82.982	1
96.268	1	84.724	1	96.287
ENSMUSG00000077305	Gm22470	snoRNA	1	83.418	NA	NA
NA	NA	1	95.593
ENSMUSG00000077306	Gm22469	snoRNA	1	83.169	1
94.597	1	84.229	1	93.964
ENSMUSG00000077309	n-R5s8	rRNA	1	80.688	1	87.919
1	58.616	1	94.904
ENSMUSG00000077312	Gm24170	snoRNA	1	82.092	1
95.196	NA	NA	1	95.273
ENSMUSG00000077314	Gm24171	snoRNA	1	81.925	1
95.708	1	87.786	1	95.405
ENSMUSG00000077315	Gm24172	snoRNA	1	81.125	1
96.013	1	81.62	1	93.722
ENSMUSG00000077316	n-R5s171	rRNA	1	80.251	1	96.308
1	88.203	1	93.457
ENSMUSG00000077317	Gm25785	snRNA	1	86.172	1	95.504
1	88.203	1	93.264
ENSMUSG00000077318	Gm24173	snoRNA	1	81.178	NA	NA
1	79.255	1	96.287
ENSMUSG00000077320	Gm22284	snoRNA	1	86.333	1
96.847	1	74.179	1	96.287
ENSMUSG00000077323	Rnu11	snRNA	1	71.896	1	81.731	1
70.328	1	87.447
ENSMUSG00000077325	Gm25833	snoRNA	1	81.178	NA	NA
1	86.831	1	93.117
ENSMUSG00000077327	Gm25938	snRNA	1	80.751	NA	NA	1
87.744	1	95.273
ENSMUSG00000077328	Gm25832	snoRNA	1	85.283	1
92.404	1	87.898	1	94.82
ENSMUSG00000077332	Gm23014	snoRNA	1	82.891	1
96.216	1	86.346	1	93.156
ENSMUSG00000077333	Gm23015	snoRNA	1	84.865	1
96.897	1	85.792	1	93.722
ENSMUSG00000077334	Gm23013	snRNA	1	73.401	1	96.897
1	84.724	1	96.248
ENSMUSG00000077336	Gm23011	snoRNA	1	81.178	1
94.262	1	87.274	1	90.691
ENSMUSG00000077337	Gm23012	snoRNA	1	86.351	1
96.216	1	85.21	1	96.071
ENSMUSG00000077339	Gm23010	snoRNA	1	84.534	1

96.268 1 84.83 1 94.016
ENSMUSG00000077342 Gm24670 snoRNA 1 84.397 1
95.504 1 79.625 1 96.183
ENSMUSG00000077344 Gm24326 snoRNA 1 72.985 NA NA
1 83.857 1 96.042
ENSMUSG00000077348 Gm24665 snoRNA 1 74.482 NA NA
1 84.896 1 94.99
ENSMUSG00000077349 Gm24867 snRNA 1 50.519 1 92.062
1 72.853 1 70.539
ENSMUSG00000077350 n-R5s31 rRNA 1 65.537 1 81.731
1 68.129 1 93.316
ENSMUSG00000077351 Gm24350 snoRNA 1 83.823 1
93.654 1 84.308 1 95.593
ENSMUSG00000077354 Gm26286 snoRNA 1 72.655 1
96.407 1 55.9 1 92.675
ENSMUSG00000077356 Gm26288 snoRNA 1 74.482 NA NA
1 67.648 1 96.358
ENSMUSG00000077357 Gm26289 snoRNA 1 85.203 NA NA
1 81.032 1 96.183
ENSMUSG00000077359 Gm25308 snRNA 1 79.709 1 93.654
1 88.601 1 92.181
ENSMUSG00000077360 Gm23482 snoRNA 0.574 40.422 1.958
83.104 0.888 76.64 1.919 91.492
ENSMUSG00000077361 Gm23481 snoRNA 1 75.399 1
95.504 1 88.729 NA NA
ENSMUSG00000077366 Gm23484 snoRNA 1 82.732 1
96.843 1 84.83 1 93.261
ENSMUSG00000077367 Gm23483 snRNA 1 82.732 1 96.138
1 85.757 1 95.777
ENSMUSG00000077368 Gm26273 snoRNA 1 83.726 NA NA
1 85.58 1 96.287
ENSMUSG00000077369 Gm23485 snRNA 1 78.744 1 91.361
1 81.976 1 96.192
ENSMUSG00000077376 Gm25167 snoRNA 1 83.968 1
97.263 1 83.607 1 94.82
ENSMUSG00000077377 Gm25168 snoRNA 1 83.823 1
97.263 1 76.754 1 94.904
ENSMUSG00000077378 Gm25169 snoRNA 1 85.203 1
92.914 1 81.525 1 96.183
ENSMUSG00000077379 Gm25170 snoRNA 1 86.464 1
97.155 1 82.31 1 94.19
ENSMUSG00000077380 Gm22661 snoRNA 0.74 51.089 0.739
97.263 1.823 56.708 0.697 85.493
ENSMUSG00000077381 Gm22660 snoRNA 1 83.169 1
95.504 1 78.642 1 94.82
ENSMUSG00000077382 n-R5s200 rRNA 0.891 67.334 0.629 94.48
1.776 35.13 1.463 90.177
ENSMUSG00000077383 Gm22659 snoRNA 1 78.299 1
91.83 NA NA 1 93.156
ENSMUSG00000077384 Gm22658 snRNA 1 64.893 1 95.504
1 74.686 1 93.964
ENSMUSG00000077385 Gm22657 snoRNA 1 84.294 1
97.263 1 83.275 1 94.904
ENSMUSG00000077391 Gm24336 snoRNA 1.049 84.168 0.385

84.594 2.468 45.94 0.757 92.181
ENSMUSG00000077392 Gm24337 snoRNA 1 85.391 NA NA
1 88.076 1 96.086
ENSMUSG00000077393 Gm24338 snoRNA 1 74.516 1
96.68 1 74.853 1 95.593
ENSMUSG00000077394 Gm24339 snoRNA 0.858 86.57 1.28 96.001
0.909 88.601 0.99 94.904
ENSMUSG00000077396 Gm24340 snRNA 1 85.637 1 96.138
1 82.161 1 93.264
ENSMUSG00000077397 Gm24341 snoRNA 1 85.391 1
97.155 1 87.274 1 95.593
ENSMUSG00000077398 Gm24334 snoRNA 1 73.734 1
91.361 1 88.693 1 95.559
ENSMUSG00000077399 Gm24335 snoRNA 1 83.169 1
96.847 NA NA 1 96.183
ENSMUSG00000077400 Gm25417 snoRNA 1 83.823 1
96.407 1 83.857 1 93.117
ENSMUSG00000077401 n-R5s82 rRNA 1 69.777 1 93.654
NA NA 1 93.612
ENSMUSG00000077402 Gm25418 snoRNA 1 83.418 1
96.847 1 88.601 1 95.273
ENSMUSG00000077403 Gm25419 snoRNA 1 73.841 1
96.013 1 67.962 1 95.877
ENSMUSG00000077404 Gm25420 snoRNA 1 75.115 1
95.196 1 84.434 NA NA
ENSMUSG00000077406 Gm25421 snRNA 1 81.76 1 95.504 1
85.58 1 95.593
ENSMUSG00000077407 NA NA NA NA 1 94.887 1
74.325 1 95.35
ENSMUSG00000077408 Gm25415 snoRNA 1 78.639 1
96.843 1 85.757 1 95.74
ENSMUSG00000077409 Gm25416 snRNA 1 80.03 1 90.256 1
82.161 1 92.381
ENSMUSG00000077410 Gm23750 snoRNA 1 81.24 1 95.708
1 84.229 1 95.74
ENSMUSG00000077411 Gm23749 snoRNA 1 84.977 1
96.847 1 77.704 1 95.35
ENSMUSG00000077413 Gm23389 snoRNA 1 85.857 1
96.843 1 87.898 1 92.31
ENSMUSG00000077415 Gm23748 snoRNA 1 86.918 1
96.013 1 78.155 1 94.608
ENSMUSG00000077417 Gm23746 snoRNA 1 79.433 1
97.129 1 80.015 1 95.35
ENSMUSG00000077418 Gm23745 snoRNA 1 83.263 1
94.887 1 79.426 1 95.877
ENSMUSG00000077419 Gm23744 snRNA 1 84.049 1 95.809
1 87.744 1 95.877
ENSMUSG00000077420 Gm24282 miRNA 1 84.661 1 92.931
1 71.734 1 88.505
ENSMUSG00000077421 Gm24283 snRNA 1 85.499 1 94.89 1
86.346 1 95.273
ENSMUSG00000077422 Gm24281 snoRNA 1 83.823 1
95.196 1 77.17 1 96.183
ENSMUSG00000077424 NA NA NA NA 1 97.155 1

81.169	1	93.316
ENSMUSG00000077425	Gm24280	snoRNA	1	76.319	1
97.288	1	87.336	1	94.654
ENSMUSG00000077426	Gm26387	snoRNA	1.301	54.44	1.018	93.817
0.756	73.531	0.894	93.316
ENSMUSG00000077427	Gm24278	snoRNA	1	80.146	1
96.308	1	87.898	1	93.734
ENSMUSG00000077428	Gm24284	snoRNA	1	84.661	1
97.288	1	85.134	1	94.215
ENSMUSG00000077430	Gm22592	snoRNA	1	86.112	1
96.407	1	82.161	1	92.903
ENSMUSG00000077431	Gm22591	snRNA	1	86.351	1	94.102
1	87.898	1	91.217
ENSMUSG00000077433	Gm22593	snRNA	1	81.647	1	95.708
1	88.274	1	94.567
ENSMUSG00000077435	Gm22590	snoRNA	1	85.499	1
95.35	1	82.903	1	95.35
ENSMUSG00000077437	n-R5s178	rRNA	1	78.538	1	95.431
1	86.602	1	96.287
ENSMUSG00000077438	Gm22595	snoRNA	1	75.761	1
97.288	1	88.461	1	94.19
ENSMUSG00000077439	Gm22594	snoRNA	1	86.333	1
97.288	1	83.607	1	96.287
ENSMUSG00000077440	Gm23130	snoRNA	1.133	51.826	0.876
53.434	1.004	57.637	0.925	91.372
ENSMUSG00000077441	Gm23131	snoRNA	1	74.745	1
95.708	1	88.076	1	95.273
ENSMUSG00000077442	Gm23132	snoRNA	1	69.408	1
96.847	NA	NA	1	94.82
ENSMUSG00000077443	Gm23133	snRNA	1	83.658	1	97.155
1	71.839	1	95.273
ENSMUSG00000077445	Gm23128	snoRNA	1	85.637	1
96.948	1	87.658	1	95.877
ENSMUSG00000077447	Gm23129	snoRNA	1	74.482	1
93.654	1	85.675	1	93.964
ENSMUSG00000077448	Gm23126	snoRNA	1	72.985	1
95.431	1	81.976	1	92.742
ENSMUSG00000077450	Rab11b	protein_coding	0.956	8.45	1.049
82.904	0.998	65.235	0.968	84.29
ENSMUSG00000077451	Gm25916	snoRNA	1	79.068	1
96.216	1	81.525	1	96.192
ENSMUSG00000077453	Gm25915	snoRNA	1	79.853	NA	NA
1	88.349	1	96.183
ENSMUSG00000077454	Gm25918	snRNA	1	73.841	1	96.843
1	88.41	1	96.042
ENSMUSG00000077455	Gm25917	snoRNA	1	71.516	NA	NA
NA	NA	1	94.174
ENSMUSG00000077456	Gm22927	snoRNA	1	78.744	1
91.532	1	73.027	1	96.183
ENSMUSG00000077459	Gm25919	snoRNA	1	86.172	1
95.708	1	79.897	1	94.904
ENSMUSG00000077460	Gm26419	snoRNA	1	79.577	1
97.097	1	83.066	1	95.676
ENSMUSG00000077461	Gm26420	snRNA	1	86.474	1	96.216

1 88.41 1 96.358
ENSMUSG00000077462 Gm26417 snoRNA 1 84.263 NA NA
1 88.274 1 96.287
ENSMUSG00000077463 Gm26418 snoRNA 1 85.355 1
95.35 1 88.657 1 92.748
ENSMUSG00000077464 Gm26422 snoRNA 1 84.865 NA NA
1 87.686 1 96.287
ENSMUSG00000077465 Gm26423 snoRNA 1 83.333 1
96.216 1 81.525 1 96.071
ENSMUSG00000077467 Gm26421 snoRNA 1 86.199 1
96.68 NA NA 1 95.273
ENSMUSG00000077468 Gm26416 snoRNA 1 79.277 1
97.155 1 87.173 1 95.676
ENSMUSG00000077469 Gm25922 snoRNA 1 84.263 1
96.843 1 79.325 1 95.273
ENSMUSG00000077471 Gm24788 snRNA 1 86.006 1 95.708
1 87.173 1 94.153
ENSMUSG00000077472 Gm24791 snoRNA 1 78.909 1
95.809 1 88.693 1 95.593
ENSMUSG00000077473 Gm24790 snoRNA 1 86.351 NA NA
1 79.897 1 95.777
ENSMUSG00000077474 Gm24792 snoRNA 1 84.977 1
93.654 1 88.693 1 93.156
ENSMUSG00000077476 Gm24794 snoRNA 1 79.577 1
96.407 1 88.41 1 93.156
ENSMUSG00000077477 Gm24793 snoRNA 1 81.823 1
93.765 1 70.44 1 96.042
ENSMUSG00000077480 Gm23439 snoRNA 1 86.351 1
92.931 1 83.158 1 93.612
ENSMUSG00000077481 Gm25272 snoRNA 1 85.655 1
97.155 1 85.921 1 96.183
ENSMUSG00000077483 Gm25273 snoRNA 1 86.006 1
95.708 1 62.995 1 94.904
ENSMUSG00000077484 Gm25274 snoRNA 1 85.655 1
96.843 1 84.724 1 94.82
ENSMUSG00000077485 Gm25275 snoRNA 1 80.146 1
97.263 1 80.015 1 95.877
ENSMUSG00000077487 Gm23440 snoRNA 1 81.01 NA NA 1
81.525 1 96.287
ENSMUSG00000077488 Gm25276 snoRNA 1 83.169 NA NA
1 84.83 1 93.016
ENSMUSG00000077492 Gm23596 snoRNA 1 80.408 NA NA
NA NA 1 95.92
ENSMUSG00000077493 Snord91a snoRNA 1.174 72.27 0.835 84.017
1.023 75.527 1.021 93.457
ENSMUSG00000077494 Gm23595 snoRNA 1 85.857 NA NA
1 83.538 1 96.183
ENSMUSG00000077495 Gm23594 snoRNA 1 85.728 1
94.597 1 84.961 1 92.52
ENSMUSG00000077497 Gm23593 snoRNA 1 73.292 1
95.708 1 53.882 1 76.831
ENSMUSG00000077499 Gm23604 snoRNA 1 85.391 1
93.908 1 71.615 1 94.567
ENSMUSG00000077501 Gm24230 snoRNA 1 84.294 1

97.263 1 85.757 1 94.752
ENSMUSG00000077502 Gm24232 snoRNA 1 85.728 1
95.504 1 83.857 1 94.153
ENSMUSG00000077503 Gm24231 snoRNA 1 85.655 1
94.597 1 87.744 1 95.273
ENSMUSG00000077504 Gm24234 snRNA 1 84.564 NA NA NA
NA 1 95.273
ENSMUSG00000077507 NA NA NA NA 1 93.529 1
81.492 1 95.877
ENSMUSG00000077511 Gm25875 snRNA 1 84.452 NA NA 1
88.693 1 94.904
ENSMUSG00000077514 Gm25877 snoRNA 1 81.823 1
95.941 1 88.203 1 92.466
ENSMUSG00000077515 Gm25878 snoRNA 1 85.499 1
96.68 1 82.575 1 95.35
ENSMUSG00000077516 n-R5s219 rRNA 1 82.231 1 96.652
1 83.607 1 96.358
ENSMUSG00000077517 Gm25876 snoRNA 1 82.732 1
95.504 1 86.985 1 94.99
ENSMUSG00000077518 Gm25879 snRNA 1 86.172 1 96.843
1 86.144 1 94.19
ENSMUSG00000077519 Gm25880 snoRNA 1 85.355 1
95.088 1 86.666 1 95.72
ENSMUSG00000077521 Gm25378 snoRNA 1 81.01 1 96.68 1
73.309 1 96.287
ENSMUSG00000077522 Gm25377 snoRNA 1 84.049 1
96.268 1 87.686 1 95.676
ENSMUSG00000077526 Gm25379 snoRNA 1 81.647 1
95.088 1 86.144 1 95.92
ENSMUSG00000077527 Gm25506 snoRNA 1 74.299 1
94.887 1 78.155 1 93.156
ENSMUSG00000077528 Gm25376 snoRNA 1 78.054 1
91.532 1 83.397 1 92.903
ENSMUSG00000077535 Gm22543 snRNA 1 79.577 1 95.211
1 87.744 1 92.381
ENSMUSG00000077537 Gm22544 snoRNA 1 86.464 NA NA
1 82.778 1 94.153
ENSMUSG00000077538 Gm22545 snoRNA 1 84.738 1
95.708 NA NA 1 92.12
ENSMUSG00000077539 Gm22546 snoRNA 1 86.658 1
95.35 1 83.158 1 95.593
ENSMUSG00000077541 Gm22021 snRNA 1 79.709 1 95.088
1 88.349 1 96.287
ENSMUSG00000077542 Gm22023 snoRNA 1 73.734 1
91.897 1 73.027 1 96.042
ENSMUSG00000077543 Gm22022 snoRNA 1 80.03 1 88.823
1 70.791 1 94.384
ENSMUSG00000077546 Gm22020 snoRNA 1 86.74 1 96.268
1 69.002 1 95.273
ENSMUSG00000077547 Gm22019 snRNA 1 65.869 1 96.68 1
77.17 1 79.268
ENSMUSG00000077549 Snord71 snoRNA 0.506 51.56 NA NA
1.795 62.995 1.183 94.825
ENSMUSG00000077552 Gm23709 snoRNA 1 86.172 1

96.138 1 87.744 1 93.264
ENSMUSG00000077553 Gm23176 snoRNA 1 85.499 1
95.708 1 88.203 1 95.593
ENSMUSG00000077554 Gm23707 snoRNA 1 85.728 1
97.155 1 77.888 1 94.904
ENSMUSG00000077555 Gm23708 snoRNA 1 83.823 NA NA
1 82.74 1 96.192
ENSMUSG00000077558 Gm23705 snoRNA 1 79.277 1
90.256 1 84.308 1 95.273
ENSMUSG00000077559 Gm23175 snoRNA 1 60.853 1
96.843 1 85.675 1 94.608
ENSMUSG00000077561 Gm24607 snoRNA 1 82.352 1
87.322 1 64.624 1 96.183
ENSMUSG00000077562 Gm23203 snRNA 1 86.199 1 95.35 1
87.274 1 95.832
ENSMUSG00000077563 Snora68 snoRNA 1.171 81.178 1.487
96.407 0.572 68.584 0.916 96.358
ENSMUSG00000077564 Gm23202 snoRNA 1 85.857 NA NA
NA NA 1 94.904
ENSMUSG00000077565 Gm23201 snoRNA 1.522 20.799 0.529
34.058 1.237 37.259 1.461 50.74
ENSMUSG00000077569 Gm24608 snoRNA 0.675 69.943 0.988
96.68 1.493 88.693 0.885 96.184
ENSMUSG00000077572 Gm24886 snoRNA 1 82.543 1
95.211 1 86.602 1 95.593
ENSMUSG00000077575 Gm24888 snoRNA 0.973 81.52 1.027 92.404
1.002 58.915 1.036 92.762
ENSMUSG00000077576 Gm24889 snoRNA 1 73.585 1
95.211 1 86.144 1 96.248
ENSMUSG00000077577 Gm24890 snRNA 1 67.163 1 96.378
1 80.089 1 95.877
ENSMUSG00000077578 Gm25631 snoRNA 1 82.891 1
95.708 1 86.346 1 94.904
ENSMUSG00000077579 NA NA NA NA 1 95.708 1
72.148 1 93.627
ENSMUSG00000077581 Gm24373 snoRNA 1 84.819 1
95.088 1 86.084 1 92.742
ENSMUSG00000077582 Gm24374 snRNA 1 84.294 NA NA 1
83.607 1 93.457
ENSMUSG00000077585 Gm24375 snoRNA 1 82.092 1
83.876 1 78.155 1 94.904
ENSMUSG00000077586 Gm24377 snoRNA 1 84.397 1
96.138 1 81.388 1 95.559
ENSMUSG00000077587 Gm24376 snoRNA 1 78.538 1
95.196 1 88.461 1 96.358
ENSMUSG00000077588 Gm24372 misc_RNA 1 81.657 NA NA
1 86.258 1 94.904
ENSMUSG00000077590 Gm26015 snoRNA 1 84.661 1
95.504 1 84.896 1 96.248
ENSMUSG00000077591 Gm26016 snoRNA 1 79.93 NA NA 1
86.258 1 96.086
ENSMUSG00000077592 Gm26013 snoRNA 1 74.482 1
90.378 1 81.857 1 93.612
ENSMUSG00000077593 Gm26014 snoRNA 1 79.577 1

96.984 1 86.112 1 96.287
ENSMUSG00000077594 Gm26018 snoRNA 1 84.977 1
95.735 NA NA 1 96.183
ENSMUSG00000077595 Gm26019 snoRNA 1 85.355 1
94.597 1 84.961 1 95.832
ENSMUSG00000077597 Gm26017 snRNA 1 86.145 1 92.631
1 86.258 1 94.99
ENSMUSG00000077598 Gm26011 snRNA 1 77.113 1 95.211
1 88.161 1 96.248
ENSMUSG00000077599 Gm26012 snoRNA 1 74.299 1
96.68 NA NA 1 94.82
ENSMUSG00000077601 Gm22674 snoRNA 1 85.355 1
95.708 1 78.498 1 95.72
ENSMUSG00000077603 Gm22673 snoRNA 1 84.661 NA NA
1 81.29 1 94.608
ENSMUSG00000077604 Snord1b snoRNA 1 85.391 1
95.504 1 86.112 1 93.734
ENSMUSG00000077607 Gm25803 snoRNA 1 71.763 1
92.404 1 55.512 1 93.261
ENSMUSG00000077608 Gm22671 snoRNA 1 84.263 1
93.654 1 88.657 1 92.389
ENSMUSG00000077609 Gm25795 snoRNA 1 83.09 NA NA 1
84.308 1 95.72
ENSMUSG00000077612 Gm25498 snoRNA 1 74.482 NA NA
1 86.666 1 96.042
ENSMUSG00000077613 Gm25497 snoRNA 1 76.827 1
94.597 1 85.675 1 95.35
ENSMUSG00000077614 NA NA NA NA 0.722 79.807 1.186
67.823 1.002 91.872
ENSMUSG00000077615 Gm25499 snoRNA 1 81.01 1 95.708
1 87.686 1 94.608
ENSMUSG00000077616 Gm25502 snoRNA 1 86.57 1 96.013
1 82.575 1 92.018
ENSMUSG00000077617 Gm25501 snoRNA 1 65.388 1
90.29 1 71.004 1 95.273
ENSMUSG00000077618 Gm25496 snoRNA 1 85.946 1
96.216 1 85.921 1 95.92
ENSMUSG00000077619 Gm25495 snoRNA 1 84.324 1
93.469 1 83.925 1 93.734
ENSMUSG00000077620 n-R5s9 rRNA 1 77.763 1 97.263
1 83.857 1 94.016
ENSMUSG00000077621 Gm23772 snoRNA 1 83.169 1
96.652 1 86.112 1 93.722
ENSMUSG00000077622 Gm23836 snoRNA 1 81.647 1
97.288 1 83.719 1 94.82
ENSMUSG00000077624 Gm23834 snoRNA 1 78.428 1
93.529 1 86.666 1 95.72
ENSMUSG00000077625 Snord4a snoRNA 0.974 84.263 1.13
84.768 0.911 50.184 1.206 92.389
ENSMUSG00000077627 Gm23835 snoRNA 1 85.092 1
94.48 1 88.349 1 96.184
ENSMUSG00000077628 Gm23768 snoRNA 1 81.178 1
95.708 1 81.492 1 94.904
ENSMUSG00000077629 Gm23769 snoRNA 1 86.199 1

97.263 1 87.37 1 96.183
ENSMUSG00000077630 Gm22149 snoRNA 1 80.608 1
96.216 1 88.203 1 96.139
ENSMUSG00000077633 Gm22148 snRNA 1 80.251 1 96.68 1
88.203 1 96.183
ENSMUSG00000077635 Gm22150 snoRNA 1 84.564 1
96.138 NA NA 1 96.192
ENSMUSG00000077637 Gm22771 snoRNA 0.67 27 1.144 82.103
1.303 86.985 1.092 90.125
ENSMUSG00000077639 Gm22772 snoRNA 1 37.414 1
91.361 1 80.5 1 93.612
ENSMUSG00000077640 Gm24986 snoRNA 1 68.485 1
95.088 1 75.009 1 95.273
ENSMUSG00000077641 Gm24987 snoRNA 1 85.728 1
96.984 1 88.461 1 92.748
ENSMUSG00000077642 Gm24984 snoRNA 1 73.487 1
96.216 1 86.258 1 96.358
ENSMUSG00000077643 Gm24985 snoRNA 1 72.985 1
95.504 1 76.923 1 96.183
ENSMUSG00000077644 Gm24982 snoRNA 1 83.823 1
93.094 1 86.985 1 95.832
ENSMUSG00000077645 Gm24983 snoRNA 1 83.333 1
92.474 1 87.744 1 93.156
ENSMUSG00000077647 Gm24981 snoRNA 1 85.946 1
97.155 1 77.704 1 92.389
ENSMUSG00000077648 Gm24988 snoRNA 1 70.213 1
95.735 1 78.498 1 93.261
ENSMUSG00000077649 Gm24989 snoRNA 1 85.655 1
95.211 1 87.274 1 94.904
ENSMUSG00000077650 Gm23320 snoRNA 1 85.857 1
95.301 1 76.388 1 92.748
ENSMUSG00000077653 Gm23321 snoRNA 1 83.658 1
97.155 1 84.896 1 92.181
ENSMUSG00000077655 Gm23319 snoRNA 1 82.705 1
91.955 1 84.896 1 96.358
ENSMUSG00000077659 Gm23323 snoRNA 1 81.465 1
96.763 NA NA 1 90.397
ENSMUSG00000077660 Gm24257 snoRNA 1 83.333 1
96.216 1 76.225 1 95.832
ENSMUSG00000077661 n-R5s51 rRNA 1 84.977 1 97.288
1 88.349 1 96.183
ENSMUSG00000077663 Gm26144 snoRNA 1 78.744 1
97.155 1 84.896 1 96.358
ENSMUSG00000077664 Gm26145 snoRNA 1 86.474 1
95.431 1 83.275 1 94.608
ENSMUSG00000077666 Gm26146 snRNA 1 81.76 1 97.049 1
88.461 1 96.086
ENSMUSG00000077668 Gm26143 snRNA 1 85.499 1 96.138
1 86.144 1 94.904
ENSMUSG00000077669 Gm24255 snoRNA 1 82.732 1
95.708 1 72.148 1 92.181
ENSMUSG00000077671 Gm24473 snoRNA 1 85.946 1
96.68 NA NA 1 95.877
ENSMUSG00000077672 Gm24472 snoRNA 1 85.203 1

95.941 1 84.724 1 93.413
ENSMUSG00000077673 Gm24471 snoRNA 1 83.09 1 95.708
1 83.538 1 94.297
ENSMUSG00000077674 Gm24470 snoRNA 1 83.169 NA NA
1 88.203 1 92.31
ENSMUSG00000077675 Gm24469 snoRNA 1 73.124 1
90.68 1 87.37 1 95.559
ENSMUSG00000077677 Gm24468 snRNA 1 86.658 1 95.211
1 88.203 1 93.584
ENSMUSG00000077678 Gm24318 snoRNA 1 79.277 1
95.088 1 84.724 1 96.183
ENSMUSG00000077680 Gm22495 snoRNA 1 83.726 NA NA
NA NA 1 90.95
ENSMUSG00000077681 Gm22496 snoRNA 1 84.534 NA NA
NA NA 1 96.183
ENSMUSG00000077682 Gm22493 snoRNA 1 79.433 1
93.094 1 88.657 1 93.457
ENSMUSG00000077683 Gm22494 snoRNA 1 82.982 NA NA
1 86.084 1 92.181
ENSMUSG00000077685 Gm22498 snoRNA 1 84.049 1
96.013 1 88.349 1 93.132
ENSMUSG00000077687 Gm22497 snoRNA 1 86.474 1
95.504 1 88.729 1 96.183
ENSMUSG00000077691 Gm25344 snoRNA 1 69.408 1
95.301 1 86.666 1 95.832
ENSMUSG00000077693 Gm25345 snoRNA 1 78.909 1
95.708 1 88.601 1 96.358
ENSMUSG00000077697 Gm25346 snoRNA 1 85.092 1
96.897 1 82.436 1 95.273
ENSMUSG00000077698 Snord12 snoRNA 1 81.925 1
93.765 NA NA 1 94.825
ENSMUSG00000077700 Gm24069 snoRNA 1 86.333 1
96.216 1 81.032 1 96.183
ENSMUSG00000077701 Gm24068 snoRNA 1 85.283 1
96.216 1 85.352 1 96.086
ENSMUSG00000077702 Gm24067 snoRNA 1 81.925 1
96.843 1 70.214 1 96.192
ENSMUSG00000077703 Gm24066 snoRNA 1 75.567 1
96.847 1 86.985 1 94.654
ENSMUSG00000077704 Snord89 snoRNA 0.82 62.437 0.941
94.96 1.297 69.825 1.317 78.948
ENSMUSG00000077706 n-R5s72 rRNA 1 82.732 NA NA 1
80.899 1 94.904
ENSMUSG00000077707 Gm24070 snoRNA 1 85.728 1
95.196 NA NA 1 94.001
ENSMUSG00000077708 Gm24071 snoRNA 1 83.263 1
96.68 NA NA 1 96.358
ENSMUSG00000077709 Snora64 snoRNA 1 86.474 1
95.088 1 87.37 1 96.192
ENSMUSG00000077710 Gm25727 snoRNA 1 85.391 1
96.216 1 82.575 1 96.192
ENSMUSG00000077711 AF357399 snoRNA 1.155 86.333 0.642
73.094 1.341 42.199 1.079 81.251
ENSMUSG00000077713 Gm25728 snRNA 1 84.534 1 96.407

1 83.925 1 93.457
ENSMUSG00000077714 Snord17 snoRNA 0.582 59.674 1.037
95.35 1.648 78.498 1.554 95.92
ENSMUSG00000077716 Gm25725 snoRNA 1 81.647 1
93.654 1 87.103 1 95.273
ENSMUSG00000077717 Gm25726 snoRNA 1 81.01 1 96.652
1 84.961 1 92.748
ENSMUSG00000077718 Gm25723 snoRNA 1 81.01 1 96.843
1 79.325 1 96.086
ENSMUSG00000077719 Gm25724 snoRNA 1 82.891 1
97.097 1 81.388 1 96.183
ENSMUSG00000077726 Gm22900 snoRNA 1 66.76 1 96.843
1 72.269 1 95.676
ENSMUSG00000077728 Gm22899 snoRNA 1 84.819 NA NA
NA NA 1 93.264
ENSMUSG00000077730 Gm23609 snoRNA 1 82.092 1
96.984 1 77.704 1 87.447
ENSMUSG00000077734 Snord83b snoRNA 0.697 70.597 0.952
93.817 1.503 57.286 1.623 69.404
ENSMUSG00000077735 Gm24556 snoRNA 1 82.092 1
97.263 1 86.084 1 96.287
ENSMUSG00000077736 Gm24555 snoRNA 1 74.745 1
95.504 NA NA 1 95.92
ENSMUSG00000077737 Snord72 snoRNA 1.43 55.789 0.642
85.116 1.091 82.161 0.972 92.181
ENSMUSG00000077738 Gm23607 snoRNA 1 80.751 1
96.216 NA NA 1 96.183
ENSMUSG00000077741 Gm26344 snoRNA 1 83.968 1
95.211 1 87.744 1 94.904
ENSMUSG00000077743 n-R5s16 rRNA 1 81.823 1 97.263
1 79.524 1 96.287
ENSMUSG00000077744 Gm26343 snoRNA 1 63.804 NA NA
1 74.179 1 92.181
ENSMUSG00000077745 Gm26342 snoRNA 1 77.182 NA NA
1 87.274 1 88.657
ENSMUSG00000077749 Gm26341 snoRNA 1 84.049 1
93.899 1 88.729 1 96.287
ENSMUSG00000077750 Gm23529 snoRNA 1 85.499 1
96.216 1 88.601 1 96.358
ENSMUSG00000077752 Gm23530 snoRNA 1 85.857 NA NA
1 87.971 1 94.608
ENSMUSG00000077755 Gm23531 snoRNA 1 84.865 NA NA
1 87.898 1 94.82
ENSMUSG00000077756 Snord90 snoRNA 1 73.487 1
96.216 1 86.727 1 95.405
ENSMUSG00000077757 Gm23532 snoRNA 1 85.637 1
93.654 1 86.144 1 95.273
ENSMUSG00000077758 Gm23527 snoRNA 1 81.925 1
91.361 1 79.554 1 95.92
ENSMUSG00000077759 Gm23528 snoRNA 1 75.298 1
94.848 1 87.173 1 93.964
ENSMUSG00000077761 Gm25214 snoRNA 1 85.77 1 91.532
1 72.853 1 92.389
ENSMUSG00000077767 Snora35 snoRNA 1 82.092 1

95.504 1 76.225 1 96.042
ENSMUSG00000077768 Gm25216 snoRNA 1 86.658 NA NA
1 84.961 1 95.35
ENSMUSG00000077769 Gm25215 snoRNA 1 82.092 1
95.211 1 81.492 1 95.559
ENSMUSG00000077771 Gm22374 snoRNA 1 82.705 1
95.809 1 87.103 1 95.35
ENSMUSG00000077772 Gm22373 snoRNA 1 79.277 1
96.843 1 84.229 1 95.273
ENSMUSG00000077776 Gm22371 snoRNA 1 84.263 1
90.712 1 88.693 1 96.086
ENSMUSG00000077777 Gm22372 snoRNA 1 60.853 1
94.262 1 78.369 1 95.273
ENSMUSG00000077780 Gm24161 snoRNA 1 86.57 1 95.504
1 84.724 1 96.358
ENSMUSG00000077782 Gm24160 snoRNA 1 81.196 1
96.013 1 87.431 1 96.287
ENSMUSG00000077783 Gm23907 snRNA 1 82.092 1 93.908
1 83.066 1 94.82
ENSMUSG00000077784 Gm23911 snoRNA 1 86.843 1
95.504 1 85.757 1 95.877
ENSMUSG00000077785 Gm23910 snoRNA 1 85.391 NA NA
1 82.778 1 95.593
ENSMUSG00000077786 Gm23909 snoRNA 1 85.857 NA NA
1 88.274 1 93.722
ENSMUSG00000077787 Gm23908 snoRNA 0.628 61.633 0.774
65.819 2.056 22.994 1.375 78.379
ENSMUSG00000077789 n-R5s26 rRNA 1 86.199 1 93.908
1 88.349 1 95.593
ENSMUSG00000077791 Gm25563 snRNA 1 76.042 1 90.378
1 62.307 1 95.273
ENSMUSG00000077792 Gm25564 snoRNA 1 73.734 1
95.504 1 80.015 1 95.273
ENSMUSG00000077793 Gm25565 snRNA 1 82.891 1 95.708
1 81.29 1 95.676
ENSMUSG00000077794 Gm25560 snRNA 1 85.832 1 96.68 1
88.41 1 96.183
ENSMUSG00000077795 Gm25561 snoRNA 1 84.865 1
96.268 1 88.349 1 95.273
ENSMUSG00000077796 Gm25562 snoRNA 1 71.915 1
95.431 1 81.492 1 94.904
ENSMUSG00000077797 Snord19 snoRNA 0.999 70.213 0.783
96.138 1.28 77.532 0.928 95.405
ENSMUSG00000077799 Gm25148 snoRNA 1 72.546 1
97.263 1 83.857 1 92.762
ENSMUSG00000077802 n-R5s52 rRNA 1 82.982 NA NA 1
84.626 1 95.273
ENSMUSG00000077804 Gm24403 snoRNA 1 76.686 1
83.104 1 77.294 1 90.865
ENSMUSG00000077807 Gm24404 miRNA 1 83.169 1 94.48 1
86.475 1 93.612
ENSMUSG00000077812 Gm22734 miRNA 1 79.577 1 95.809
1 84.308 1 94.904
ENSMUSG00000077815 Mir290 miRNA 1 86.819 1 96.68 1

85.134 1 90.463
ENSMUSG00000077816 Gm22735 miRNA 1 85.728 1 96.847
1 73.874 1 93.612
ENSMUSG00000077821 Gm23235 miRNA 1 80.815 1 96.847
1 88.076 1 95.74
ENSMUSG00000077829 Gm23237 miRNA 1 85.857 1 95.708
1 68.584 1 94.904
ENSMUSG00000077830 Gm26050 miRNA 1 81.823 1 95.211
1 84.896 1 94.608
ENSMUSG00000077832 Gm26051 miRNA 1 84.819 1 95.088
1 86.258 1 92.389
ENSMUSG00000077834 NA NA NA NA 1 96.268 1
78.864 1 94.904
ENSMUSG00000077836 Gm26052 miRNA 1 78.538 1 95.211
1 86.258 1 96.071
ENSMUSG00000077839 Gm26053 miRNA 1 86.006 NA NA 1
87.658 1 94.904
ENSMUSG00000077841 Gm22230 miRNA 1 76.042 NA NA 1
84.504 1 95.273
ENSMUSG00000077844 Gm22231 miRNA 1 68.485 1 93.654
1 74.179 1 94.468
ENSMUSG00000077846 Gm22232 miRNA 1 77.763 NA NA 1
88.601 1 95.273
ENSMUSG00000077847 Mir105 miRNA 1 72.655 1 93.817
1 87.103 1 95.877
ENSMUSG00000077851 Mir876 miRNA 1 86.199 NA NA 1
76.754 1 94.001
ENSMUSG00000077852 Gm25062 miRNA 1 84.324 1 95.708
NA NA 1 95.273
ENSMUSG00000077857 Gm25061 snRNA 1 84.865 NA NA 1
88.601 1 94.904
ENSMUSG00000077858 Gm25063 miRNA 1 78.909 1 96.268
1 79.897 1 95.273
ENSMUSG00000077860 Gm25568 miRNA 1 72.985 NA NA 1
81.62 1 96.184
ENSMUSG00000077863 Gm25567 miRNA 1 86.819 1 94.597
1 73.309 1 96.248
ENSMUSG00000077869 Mir344e miRNA 1 81.01 1 97.155 1
83.397 1 95.273
ENSMUSG00000077870 Gm23914 miRNA 1 84.564 1 97.155
1 78.498 1 96.248
ENSMUSG00000077872 Mir878 miRNA 1 84.865 1 96.216
1 74.179 1 96.183
ENSMUSG00000077876 Gm23913 miRNA 1 78.299 1 96.216
1 86.552 1 94.904
ENSMUSG00000077881 Mir882 miRNA 1 70.862 1 96.843
1 86.258 1 95.273
ENSMUSG00000077884 Gm24557 miRNA 1 81.657 1 96.407
NA NA 1 93.734
ENSMUSG00000077886 Mir295 miRNA 1 80.608 1 95.329
1 81.032 1 96.287
ENSMUSG00000077890 Mir875 miRNA 1 84.168 NA NA 1
88.274 1 95.92
ENSMUSG00000077892 Gm22901 miRNA 1 75.567 1 96.847

1 83.397 1 96.358
ENSMUSG00000077896 Mir879 miRNA 1 81.657 1 96.138
1 84.961 1 96.358
ENSMUSG00000077902 Gm25787 miRNA 1 81.76 NA NA 1
78.642 1 96.183
ENSMUSG00000077903 Mir294 miRNA 1 78.538 1 95.809
1 85.464 1 95.35
ENSMUSG00000077921 Mir872 miRNA 1 82.891 1 91.897
1 85.757 1 93.156
ENSMUSG00000077923 Gm22441 miRNA 1 78.299 1 90.29 1
88.41 1 96.287
ENSMUSG00000077928 Mir208b miRNA 1 84.819 NA NA 1
74.179 1 94.82
ENSMUSG00000077931 Mir877 miRNA 1 84.738 1 91.897
1 88.693 1 96.183
ENSMUSG00000077938 Gm24132 miRNA 1 84.168 NA NA NA
NA 1 95.74
ENSMUSG00000077939 Gm24133 miRNA 1 83.823 1 96.843
1 81.032 1 95.72
ENSMUSG00000077941 Mir466h miRNA 1 86.843 NA NA 1
88.076 1 95.74
ENSMUSG00000077948 Gm23438 miRNA 1 85.728 1 96.68 1
85.21 1 96.287
ENSMUSG00000077954 Mir297a-4 miRNA 1 85.655 1 95.504
NA NA 1 92.903
ENSMUSG00000077962 Mir874 miRNA 1 75.399 1 93.094
1 86.985 1 92.381
ENSMUSG00000077965 Gm24605 miRNA 1 86.199 1 91.361
1 86.346 1 95.676
ENSMUSG00000077966 Gm24604 miRNA 1 86.464 NA NA 1
88.461 1 96.248
ENSMUSG00000077967 Gm24603 miRNA 1 67.867 NA NA 1
72.853 1 93.964
ENSMUSG00000077971 Gm26259 miRNA 1 84.865 1 91.661
1 82.161 1 95.74
ENSMUSG00000077972 Mir466f-1 miRNA 1 84.865 NA NA 1
88.601 1 96.287
ENSMUSG00000077973 Gm26260 miRNA 1 72.743 1 95.708
1 80.015 1 96.071
ENSMUSG00000077976 Gm26258 miRNA 1 85.203 1 97.155
1 86.831 1 90.95
ENSMUSG00000077980 Mir654 miRNA 1 83.169 1 96.847
1 87.274 1 95.35
ENSMUSG00000077981 Mir466f-3 miRNA 1 79.577 NA NA 1
87.744 1 93.734
ENSMUSG00000077990 Gm23134 miRNA 0.523 5.544 1.066 88.136
1.793 20.315 1.132 66.944
ENSMUSG00000077991 Mir466n miRNA 1 77.497 1 97.155
NA NA 1 94.825
ENSMUSG00000077995 Gm23937 miRNA 1 85.728 NA NA 1
84.434 1 93.612
ENSMUSG00000077996 Mir190b miRNA 1 85.092 1 93.817
1 82.575 1 96.358
ENSMUSG00000077998 Mir466f-2 miRNA 1 83.726 1 95.708

1 84.83 1 95.777
ENSMUSG00000078002 Mir880 miRNA 1 83.418 NA NA 1
84.071 1 95.92
ENSMUSG00000078006 Gm26301 miRNA 1 84.977 NA NA 1
79.255 1 95.273
ENSMUSG00000078008 Mir291a miRNA 1 62.574 1 95.708
1 84.961 1 96.183
ENSMUSG00000078010 Gm23489 miRNA 0.791 67.867 1.377 88.533
0.916 83.857 0.506 85.493
ENSMUSG00000078012 NA NA NA NA 1 95.088 1
86.346 1 94.825
ENSMUSG00000078017 Mir883a miRNA 1 79.277 1 96.984
1 82.31 1 96.248
ENSMUSG00000078020 Gm23029 miRNA 1 86.333 1 95.504
1 84.504 1 95.273
ENSMUSG00000078024 Mir883b miRNA 1 73.734 NA NA NA
NA 1 92.389
ENSMUSG00000078025 Mir466g miRNA 1 70.213 1 96.001
1 88.461 1 94.608
ENSMUSG00000078026 Mir4660 miRNA 1 68.353 1 95.211
1 82.74 1 94.904
ENSMUSG00000078027 Mir449b miRNA 1 83.658 1 95.301
1 86.602 1 94.825
ENSMUSG00000078031 Mir466d miRNA 1 76.827 1 95.211
1 86.258 1 94.016
ENSMUSG00000078032 Mir291b miRNA 1 70.597 NA NA NA
NA 1 96.248
ENSMUSG00000078034 Gm24683 miRNA 1 75.761 1 95.504
NA NA 1 96.086
ENSMUSG00000078035 Mir293 miRNA 1 81.823 1 92.914
1 88.657 1 93.964
ENSMUSG00000078036 Gm24684 miRNA 1 79.577 1 96.138
1 88.693 1 94.384
ENSMUSG00000078038 Mir881 miRNA 1 77.763 NA NA 1
86.475 1 95.877
ENSMUSG00000078041 Mir292 miRNA 1 84.661 1 95.504
1 80.163 1 91.372
ENSMUSG00000078042 Gm24855 miRNA 1 75.761 NA NA 1
75.809 1 95.273
ENSMUSG00000078057 Mir873 miRNA 1 83.263 NA NA NA
NA 1 95.593
ENSMUSG00000078060 Mir466l miRNA 1 85.391 1 97.155
NA NA 1 95.35
ENSMUSG00000078061 Gm22824 miRNA 1 85.946 1 97.155
1 72.695 1 93.627
ENSMUSG00000078062 Gm25172 miRNA 1 83.09 NA NA 1
86.831 1 89.498
ENSMUSG00000078080 Olfr585 protein_coding 1 84.977 1
95.708 1 88.601 1 94.19
ENSMUSG00000078087 Rps12l1 pseudogene 1 85.092 1
97.155 1 72.695 1 96.184
ENSMUSG00000078096 Golgb1 protein_coding 1 79.853 1
96.847 1 86.346 NA NA
ENSMUSG00000078097 Gm10913 pseudogene 1 79.277 1

97.263 1 86.602 1 95.74
ENSMUSG00000078116 Olfr828 protein_coding 1 85.391 1
96.948 1 86.666 NA NA
ENSMUSG00000078117 Gm16485 protein_coding 0.857 64.014
1.169 82.76 0.998 71.999 1.558 74.193
ENSMUSG00000078118 Olfr483 protein_coding 1 86.658 1
96.652 1 68.425 1 96.139
ENSMUSG00000078122 F630028O10Rik antisense 0.96 50.913
1.019 83.58 1.018 85.21 1.22 59.464
ENSMUSG00000078123 Gm5071 pseudogene 1 81.52 1 96.216
1 87.431 1 93.117
ENSMUSG00000078126 Rpl23a-ps3 protein_coding 1 69.777 1
95.708 1 64.427 1 88.136
ENSMUSG00000078127 Fam170b protein_coding 0.955 67.163 1.47
85.501 0.712 82.685 0.317 83.724
ENSMUSG00000078128 Gm2178 protein_coding 1 83.169 1
96.843 1 84.724 1 92.851
ENSMUSG00000078130 Gm11555 protein_coding 1 83.726 1
96.407 1 82.436 1 96.358
ENSMUSG00000078131 Krtap1-3 protein_coding 1 85.728 1
96.216 1 87.744 1 92.381
ENSMUSG00000078132 Gm11939 protein_coding 1 81.465 1
95.211 1 86.144 1 93.584
ENSMUSG00000078137 Ankrd63 protein_coding 0.455 48.354
1.468 89.434 1.493 84.961 2.97 48.119
ENSMUSG00000078143 Gm17344 antisense 0.893 44.562 0.981
92.545 1.139 47.776 1.019 92.675
ENSMUSG00000078144 Capns2 protein_coding 0.984 67.374
1.242 95.431 0.817 38.434 0.952 93.584
ENSMUSG00000078153 Psme2b protein_coding 0.929 81.823
1.076 97.155 0.997 82.685 1.168 78.042
ENSMUSG00000078154 Gm12184 protein_coding 1.64 44.061
1.508 96.308 0.405 33.137 1.44 79.268
ENSMUSG00000078160 Gm16503 protein_coding 0.601 5.778 0.993
91.275 1.677 6.202 1.052 92.018
ENSMUSG00000078161 BC007180 protein_coding 1.062 61.725 1.36
93.899 0.693 52.658 0.692 85.493
ENSMUSG00000078162 Gm2574 pseudogene 1 83.726 NA NA
1 76.768 1 88.111
ENSMUSG00000078169 Gm16499 protein_coding 0.92 84.865 0.79
78.471 1.377 66.269 0.564 83.724
ENSMUSG00000078173 Lenep protein_coding 1 79.165 1
93.899 1 69.315 1 92.381
ENSMUSG00000078177 Gm4877 protein_coding 1 84.168 1
95.088 1 88.601 1 93.584
ENSMUSG00000078179 Rnf148 protein_coding 1 73.841 1
97.155 1 59.726 1 93.316
ENSMUSG00000078180 Gm20900 pseudogene 1 86.74 1 92.561
1 88.601 1 96.183
ENSMUSG00000078182 Gm6083 pseudogene 1 77.407 1
93.994 1 87.274 1 94.82
ENSMUSG00000078184 B020018G12Rik protein_coding 1 81.823
1 95.088 1 86.144 1 93.264
ENSMUSG00000078185 Chml protein_coding 0.965 33.599 1.114

91.643 0.93 74.325 0.913 89.525
ENSMUSG00000078186 Gm13821 protein_coding 1 82.891 1
95.708 1 87.103 1 95.74
ENSMUSG00000078190 Dnm3os antisense 1.494 39.416 0.881
89.996 0.757 50.184 0.823 83.581
ENSMUSG00000078192 Gm7290 pseudogene 1 81.76 1 96.847
1 83.607 1 95.273
ENSMUSG00000078193 Gm2000 protein_coding 1.675 0 0.848
73.766 0.702 4.23 1.127 85.577
ENSMUSG00000078197 Gm17374 protein_coding 1 85.637 1
94.102 1 82.161 1 96.183
ENSMUSG00000078198 Olfr364-ps1 protein_coding 1 84.977 1
95.708 NA NA 1 94.384
ENSMUSG00000078201 Tmem203 protein_coding 0.983 74.8 0.974
86.538 1.045 40.575 0.957 83.724
ENSMUSG00000078202 Nrarp protein_coding 0.664 4.385 1.115 89.996
1.345 9.502 0.843 84.29
ENSMUSG00000078206 Gm14458 protein_coding 1 84.738 NA
NA 1 87.744 1 95.273
ENSMUSG00000078208 Gm5634 protein_coding 1 86.172 1
97.263 1 73.309 1 95.877
ENSMUSG00000078210 Gm5924 pseudogene 1 82.705 1
94.96 1 77.294 1 95.72
ENSMUSG00000078212 Gm3757 pseudogene 1 76.968 NA NA
1 84.669 1 91.372
ENSMUSG00000078213 Gm3750 protein_coding 1 77.113 NA
NA 1 88.461 1 93.685
ENSMUSG00000078214 Gm5925 pseudogene 1 84.819 1
96.138 NA NA 1 94.654
ENSMUSG00000078218 Gm3706 protein_coding 1 86.918 1
96.216 1 86.258 1 93.132
ENSMUSG00000078234 Klhdc7a protein_coding 0.875 11.707 NA
NA 1.15 22.131 1.037 88.657
ENSMUSG00000078235 Fam43b protein_coding 0.628 79.709
0.881 93.046 1.807 76.498 1.728 93.132
ENSMUSG00000078238 Gm12854 pseudogene 0.677 15.227 1.345
82.904 1.095 69.196 0.999 95.72
ENSMUSG00000078247 Airn antisense 1.182 10.595 1.088 97.155
0.779 16.719 0.95 86.699
ENSMUSG00000078251 Gm16487 pseudogene 1 85.832 1
95.504 1 65.235 1 96.192
ENSMUSG00000078253 Krtap16-1 protein_coding 1 84.564 1
93.654 1 84.626 1 95.92
ENSMUSG00000078254 Krtap29-1 protein_coding 1 85.092 1
95.708 1 85.675 1 92.675
ENSMUSG00000078255 Krtap9-5 protein_coding 1 85.77 1
95.211 1 81.62 1 92.762
ENSMUSG00000078256 Gm11565 protein_coding 1 81.01 1
96.847 1 84.504 1 94.904
ENSMUSG00000078257 2300003K06Rik protein_coding 1 85.59 NA
NA 1 85.757 1 95.92
ENSMUSG00000078258 Gm11564 protein_coding 1 82.982 1
92.137 1 79.554 1 92.748
ENSMUSG00000078259 Gm11554 protein_coding 1 84.534 1

96.652 1 83.397 1 93.685
ENSMUSG00000078260 Gm11569 protein_coding 1 78.428 1
95.211 1 87.744 1 96.358
ENSMUSG00000078261 Gm11596 protein_coding 1 66.76 1
95.504 NA NA 1 95.273
ENSMUSG00000078262 Krtap4-9 protein_coding 1 81.76 1
95.504 1 81.169 1 94.608
ENSMUSG00000078263 Gm14188 pseudogene 1 82.705 NA NA
1 86.602 1 92.851
ENSMUSG00000078269 NA NA NA NA 1 97.155 1
73.428 1 94.001
ENSMUSG00000078270 Gm14193 pseudogene 1 77.291 1
90.053 NA NA 1 93.964
ENSMUSG00000078276 Gm14190 protein_coding 1 82.092 1
96.68 1 79.554 1 96.183
ENSMUSG00000078280 Tas2r122 protein_coding 1 85.637 1
96.652 1 73.309 1 95.877
ENSMUSG00000078284 Cdc73 protein_coding 1 84.324 1
95.708 1 78.293 1 96.248
ENSMUSG00000078291 Gm3940 protein_coding 1 84.819 1
94.597 1 84.896 1 92.762
ENSMUSG00000078300 Gm2606 pseudogene 1 82.479 1
97.263 1 88.657 1 95.273
ENSMUSG00000078302 Foxd1 protein_coding 0.651 25.111 1.517
83.104 1.009 87.274 0.951 90.289
ENSMUSG00000078306 Gm16493 protein_coding 0.895 59.028
1.016 85.985 1.104 66.928 0.911 86.991
ENSMUSG00000078308 Gm7293 protein_coding 1 84.564 1
96.268 1 83.811 1 91.525
ENSMUSG00000078314 Gm14762 lincRNA 1 70.597 NA NA
1.008 78.864 1.345 93.156
ENSMUSG00000078315 Fam47c protein_coding 1 86.172 1
97.263 NA NA 1 93.964
ENSMUSG00000078317 F8a protein_coding 0.882 11.726 1.146
72.642 0.99 84.896 0.937 85.493
ENSMUSG00000078320 Gm362 protein_coding 1 78.299 1
96.68 1 85.935 1 94.297
ENSMUSG00000078324 Gm2933 protein_coding 1 83.726 1
95.941 1 86.666 1 96.183
ENSMUSG00000078346 Gm5132 protein_coding 1 80.146 1
95.708 1 79.255 1 95.676
ENSMUSG00000078348 Sf3b5 protein_coding 0.935 11.924 1.057
84.007 1.014 84.896 1.001 96.139
ENSMUSG00000078349 AW011738 protein_coding 0.897 35.894
1.084 82.103 1.027 87.786 0.981 93.261
ENSMUSG00000078350 Smim1 protein_coding 0.919 8.139 1.092 79.367
0.999 61.418 1.013 83.067
ENSMUSG00000078353 Ifnab protein_coding 1 80.408 1
95.708 1 81.525 1 95.92
ENSMUSG00000078354 Ifna2 protein_coding 1 76.396 1
96.68 1 87.686 1 95.92
ENSMUSG00000078355 Gm13280 protein_coding 1 81.647 1
95.941 1 87.686 NA NA
ENSMUSG00000078365 Mos protein_coding 0.832 65.869 1.373

83.104 0.873 88.349 0.417 86.699
ENSMUSG00000078370 Gm6316 pseudogene 1 84.397 1
93.146 1 69.583 1 95.74
ENSMUSG00000078384 Gm10935 protein_coding 0.728 45.502
1.387 73.092 0.991 80.5 0.659 83.724
ENSMUSG00000078420 Olfr141 protein_coding 1 77.497 1
96.216 NA NA 1 96.248
ENSMUSG00000078427 Sarnp protein_coding 1.089 30.497 0.994
95.504 0.926 40.338 1.036 52.804
ENSMUSG00000078429 Ctdsp2 protein_coding 0.984 86.351 0.99
92.931 1.029 87.971 1.031 93.964
ENSMUSG00000078430 Gm10936 protein_coding 1 85.59 1
96.138 1 81.976 1 94.654
ENSMUSG00000078435 AU041133 protein_coding 1.202 26.338
1.093 87.609 0.758 17.662 0.989 93.734
ENSMUSG00000078439 2210404O07Rik protein_coding 1.101 60.435
0.964 88.705 0.94 84.724 0.841 85.493
ENSMUSG00000078440 Dohh protein_coding 0.878 9.863 1.067 90.454
1.067 29.777 1.007 96.183
ENSMUSG00000078441 Scamp4 protein_coding 1.648 9.259 0.568
71.41 1.066 83.607 0.718 83.952
ENSMUSG00000078444 Gm10941 protein_coding 0.842 67.608
1.069 78.471 1.109 69.315 1.387 96.287
ENSMUSG00000078451 Ppil6 protein_coding 0.854 34.954 0.803
75.052 1.459 11.932 1.117 94.904
ENSMUSG00000078453 Abracl protein_coding 1.166 7.049 0.884
72.576 0.972 67.339 1.147 60.968
ENSMUSG00000078481 Gm10944 protein_coding 1 86.819 1
91.361 1 86.144 1 95.35
ENSMUSG00000078484 Klhl17 protein_coding 0.949 7.218 0.986
95.735 1.065 14.376 0.977 84.29
ENSMUSG00000078485 Plekhn1 protein_coding 0.854 41.759
1.022 95.708 1.144 40.575 0.922 83.724
ENSMUSG00000078486 2310042D19Rik protein_coding 1.198 44.694
NA NA 0.883 57.091 1.206 89.72
ENSMUSG00000078487 Ankrd65 protein_coding 0.859 84.049 1.08
90.29 1.078 85.352 NA NA
ENSMUSG00000078488 Gm10945 protein_coding 1 82.231 NA
NA 1 67.823 1 92.181
ENSMUSG00000078489 Gm17106 processed_transcript 0.719 39.712
0.736 87.919 1.885 41.235 1.086 96.358
ENSMUSG00000078490 C030017K20Rik protein_coding 0.613 20.369
1.915 71.617 0.852 68.266 0.882 95.832
ENSMUSG00000078491 Gm13090 protein_coding 1 79.165 1
93.994 1 84.669 1 96.183
ENSMUSG00000078492 1700045H11Rik antisense 1 47.677 1
88.551 1 64.305 1 95.676
ENSMUSG00000078493 A930039A15Rik protein_coding 1.236 0.664
0.956 85.921 0.848 4.23 0.901 83.724
ENSMUSG00000078494 Gm13201 antisense 0.673 30.877 1.252
85.501 1.188 75.155 0.882 89.892
ENSMUSG00000078495 Gm13157 protein_coding 1.399 0 0.84
28.822 0.848 18.122 0.995 91.807
ENSMUSG00000078496 Gm13152 protein_coding 1.165 50.111 NA

NA 0.923 33.402 0.924 93.264
ENSMUSG00000078498 Gm13151 protein_coding 1 77.763 1
97.049 1 87.658 1 92.903
ENSMUSG00000078500 Gm13235 protein_coding 1 76.968 1
86.861 1 80.381 1 96.183
ENSMUSG00000078502 Gm13212 protein_coding 1.765 0 0.714
15.364 0.791 25.625 1.115 90.125
ENSMUSG00000078503 Gm13225 protein_coding 1 55.124 1
93.046 1 88.601 1 92.12
ENSMUSG00000078504 Gm438 protein_coding 1 80.608 1
96.847 1 85.757 1 95.676
ENSMUSG00000078505 Gm436 protein_coding 1 81.76 1 96.268
NA NA 1 91.269
ENSMUSG00000078506 Gm13124 protein_coding 1 84.168 1
96.138 1 84.896 1 96.358
ENSMUSG00000078507 Aadacl3 protein_coding 1 81.465 1
91.661 1 83.066 1 95.273
ENSMUSG00000078508 Gm13128 protein_coding 1 83.418 NA
NA 1 83.811 1 94.904
ENSMUSG00000078509 Pramef17 protein_coding 1 67.334 1
96.68 1 83.066 1 95.35
ENSMUSG00000078510 Gm13101 protein_coding 1 67.991 1
95.088 1 88.693 1 96.287
ENSMUSG00000078511 Gm13109 protein_coding 1 82.231 1
95.504 1 82.685 1 96.248
ENSMUSG00000078512 Pramef6 protein_coding 1 81.647 1
92.137 1 84.83 1 96.086
ENSMUSG00000078513 Gm13088 protein_coding 1 80.408 1
96.013 1 87.658 1 96.287
ENSMUSG00000078515 Ddi2 protein_coding 0.873 2.142 0.946 62.692
1.21 5.864 0.975 86.254
ENSMUSG00000078517 Emc1 protein_coding 0.902 0.987 1.041 71.617
1.062 22.366 1.001 93.734
ENSMUSG00000078519 2310026L22Rik lincRNA 0.751 81.647
1.044 96.843 1.279 87.744 1.554 95.273
ENSMUSG00000078520 Gm13011 protein_coding 1 64.714 1
94.848 NA NA 1 93.261
ENSMUSG00000078521 Aunip protein_coding 1.851 26.136 0.741
72.399 0.727 61.288 1.576 83.067
ENSMUSG00000078532 Nkain1 protein_coding 0.974 85.203
1.257 71.98 0.815 33.971 1.727 61.984
ENSMUSG00000078537 B020031M17Rik protein_coding 1 57.227
1 95.504 1 74.325 1 76.831
ENSMUSG00000078546 2210404O09Rik protein_coding 1.362 2.569
0.793 37.426 0.922 66.201 0.984 95.832
ENSMUSG00000078550 Dear1 pseudogene 1.158 77.497 0.867 92.404
0.995 86.258 0.746 85.493
ENSMUSG00000078552 Dcdc2b protein_coding 0.833 20.799
1.124 86.793 1.068 80.899 1.006 88.505
ENSMUSG00000078561 Ebf1 pseudogene 1 86.819 1 91.532
1 88.203 1 95.74
ENSMUSG00000078566 Bnip3 protein_coding 1.052 82.352 NA NA
0.916 86.666 0.914 83.724
ENSMUSG00000078570 1110065P20Rik protein_coding 0.856 19.595

1.191 79.579 0.983 84.229 0.919 85.493
ENSMUSG00000078572 1810043H04Rik protein_coding 0.928 67.163
1.016 96.216 1.061 63.301 0.968 93.316
ENSMUSG00000078576 Gm12886 protein_coding 1 86.112 1
96.268 1 79.897 1 92.263
ENSMUSG00000078577 Tmco2 protein_coding 1 72 1 91.83 NA
NA 1 93.964
ENSMUSG00000078578 Ube2d3 protein_coding 0.99 76.042
1.088 63.504 0.924 0.72 0.99 96.248
ENSMUSG00000078580 E430018J23Rik protein_coding 0.817 10.704
1.023 91.361 1.198 5.864 0.915 90.691
ENSMUSG00000078582 Hivep3 protein_coding 0.721 69.715 NA
NA 1.332 84.308 2.747 50.452
ENSMUSG00000078584 AU022252 protein_coding 0.907 10.29 1.071
82.904 1.028 63.301 0.997 89.892
ENSMUSG00000078586 Gm11735 pseudogene 1 77.497 1
97.155 1 87.431 1 94.654
ENSMUSG00000078588 Ccdc24 protein_coding 0.751 56.477
1.114 96.948 1.192 64.024 0.992 96.248
ENSMUSG00000078592 Gm4609 pseudogene 1 86.474 1
89.713 1 80.899 1 96.183
ENSMUSG00000078593 1700042G07Rik protein_coding 1 85.355
1 96.68 1 81.29 1 96.358
ENSMUSG00000078597 Cyp4a12b protein_coding 1 86.172 NA
NA 1 86.831 1 93.413
ENSMUSG00000078598 NA NA NA NA 1 94.597 1
86.144 1 94.82
ENSMUSG00000078599 Skint8 protein_coding 1 85.59 1
96.843 1 63.075 1 90.397
ENSMUSG00000078600 1700092K14Rik processed_transcript 1
84.049 1 93.863 1 79.426 1 95.273
ENSMUSG00000078601 Gm12525 antisense 0.862 60.003 3.811
67.462 0.305 24.918 0.301 83.724
ENSMUSG00000078604 Gm10961 protein_coding 0.644 81.01 1.308
96.843 1.184 84.308 1.196 92.52
ENSMUSG00000078605 E030025P04Rik protein_coding 1.725 25.421
NA NA 0.412 32.297 0.409 84.29
ENSMUSG00000078606 Gm4070 protein_coding 1.038 47.466
0.942 95.504 1.021 70.214 1.079 93.612
ENSMUSG00000078607 1810010H24Rik antisense 1.086 56.907
0.854 64.123 1.078 62.995 1.112 92.748
ENSMUSG00000078611 Gm5901 protein_coding 0.361 2.436 1.146
97.155 2.42 6.449 1.258 93.612
ENSMUSG00000078612 1700024P16Rik protein_coding 0.497 53.679
NA NA 1.691 43.586 1.85 49.806
ENSMUSG00000078616 Trim30c protein_coding 1.998 0.999 0.964
87.452 0.519 6.935 1.349 57.839
ENSMUSG00000078618 4930456L15Rik lincRNA 1 81.52 1
96.948 1 81.525 1 95.593
ENSMUSG00000078619 Smarcd2 protein_coding 0.928 23.13 0.981
75.618 1.098 9.429 1.014 95.676
ENSMUSG00000078620 Gm10964 protein_coding 1 72 1
96.68 1 52.213 1 88.245
ENSMUSG00000078621 Hbb-bh2 protein_coding 1 81.647 1

96.216 1 88.41 1 96.248
ENSMUSG00000078622 Ccdc47 protein_coding 1.037 81.125
0.996 93.094 0.968 80.381 1.035 56.863
ENSMUSG00000078624 Olfr613 protein_coding 0.812 18.379
0.934 96.897 1.313 38.038 0.898 85.493
ENSMUSG00000078625 Gm12789 protein_coding 1 79.853 1
93.654 1 84.669 1 94.654
ENSMUSG00000078626 Gm12790 protein_coding 1 78.299 1
96.407 1 77.17 1 91.65
ENSMUSG00000078627 10-Mar protein_coding 0.983 76.135
0.892 91.361 1.142 45.667 0.729 89.827
ENSMUSG00000078630 Tomt protein_coding 0.738 21.658 1.065
96.763 1.27 23.668 0.957 91.105
ENSMUSG00000078632 Lrrc37a protein_coding 1 83.169 1
96.843 1 83.607 1 95.92
ENSMUSG00000078636 Gm7336 pseudogene 1 77.497 1
96.138 1 87.37 1 92.762
ENSMUSG00000078639 Gm12695 protein_coding 1 84.263 1
95.735 1 85.757 1 94.567
ENSMUSG00000078640 Gm11627 protein_coding 1.286 40.333
1.186 93.899 0.654 17.258 1.084 95.35
ENSMUSG00000078648 Gm17546 protein_coding 0.674 65.869
0.829 86.277 1.794 43.586 1.032 93.627
ENSMUSG00000078650 G6pc protein_coding 1.908 30.497 0.618
55.71 0.847 57.637 0.467 83.724
ENSMUSG00000078651 Aoc2 protein_coding 0.805 0.987 0.927 90.378
1.336 10.615 0.969 90.289
ENSMUSG00000078652 Psme3 protein_coding 0.983 64.714 NA NA
1.004 63.075 1.07 57.1
ENSMUSG00000078653 Cntd1 protein_coding 0.781 13.062 NA NA
1.197 18.565 0.905 87.542
ENSMUSG00000078655 Gm10972 protein_coding 1 85.637 1
95.088 1 66.928 1 95.832
ENSMUSG00000078656 Vps25 protein_coding 0.996 77.497 1.081
97.263 0.928 73.531 0.893 93.612
ENSMUSG00000078657 Crnn protein_coding 1 82.352 1
94.597 1 86.144 1 96.183
ENSMUSG00000078658 Lce1d protein_coding 1 74.8 NA NA 1
81.169 1 94.174
ENSMUSG00000078664 Sprr2a1 protein_coding 1 69.777 1
88.691 1 86.602 1 95.74
ENSMUSG00000078667 1700094D03Rik protein_coding 1.007 68.846
NA NA 0.959 79.897 0.908 87.992
ENSMUSG00000078668 Gm11595 protein_coding 1 81.01 1
96.013 1 85.757 1 95.35
ENSMUSG00000078670 Fam174b protein_coding 0.975 86.819
0.925 79.475 1.103 69.196 1.001 85.493
ENSMUSG00000078671 Chd2 protein_coding 1.035 51.826 0.89
49.859 1.085 15.411 0.997 86.991
ENSMUSG00000078676 Casc3 protein_coding 1.02 46.097 0.992
95.504 0.989 66.201 0.976 83.724
ENSMUSG00000078677 Gm10974 protein_coding 1 84.049 1
97.263 NA NA 1 86.991
ENSMUSG00000078681 Tm2d3 protein_coding 1.018 50.627 1.012

91.532	0.972	69.445	0.942	83.724
ENSMUSG00000078683	Mup1	protein_coding	0.679	48.646	1.131
96.407	1.302	48.935	0.796	86.699
ENSMUSG00000078684	5830417I10Rik	pseudogene	0.993	79.165
0.981	85.116	1.029	38.038	1.027	78.546
ENSMUSG00000078685	Mup-ps4	pseudogene	1	85.129	1
90.053	1	82.685	1	95.273
ENSMUSG00000078686	Mup9	protein_coding	0.7	33.415	1.798
77.618	0.793	86.112	0.568	89.294
ENSMUSG00000078689	Mup6	protein_coding	0.486	44.787	1.381
96.68	1.492	77.532	1.212	95.832
ENSMUSG00000078691	Gm6821	protein_coding	1	72.27	1
97.155	1	87.971	1	96.358
ENSMUSG00000078695	Cisd3	protein_coding	0.872	28.998	1.251
72.546	0.915	58.775	1.084	91.872
ENSMUSG00000078698	Mrgpra3	protein_coding	1	85.946	1
97.155	1	72.148	1	95.273
ENSMUSG00000078700	D030028A08Rik	processed_transcript	0.812
19.023	1.03	97.129	1.191	18.411	0.951	96.183
ENSMUSG00000078706	Gm53	protein_coding	1.012	74.516	1.245
87.609	0.796	41.662	1.027	93.156
ENSMUSG00000078713	Tomm5	protein_coding	0.978	78.909	NA	NA
NA	NA	1.054	88.512
ENSMUSG00000078716	Tmem8b	protein_coding	1.004	75.115
0.974	96.268	1.024	83.719	0.96	87.182
ENSMUSG00000078719	Msmp	protein_coding	0.677	5.204	0.963	97.155
1.536	15.411	1	93.264
ENSMUSG00000078721	Gm12429	protein_coding	1	80.608	1
92.062	1	86.727	1	96.086
ENSMUSG00000078735	Il11ra2	protein_coding	1	80.815	NA
NA	1	84.071	1	91.269
ENSMUSG00000078737	Gm2128	protein_coding	1	79.433	1
96.407	1	79.776	1	96.183
ENSMUSG00000078742	Gm10985	protein_coding	1	85.637	NA
NA	1	82.74	1	96.183
ENSMUSG00000078746	Gm20938	protein_coding	1	72.985	1
95.504	1	74.853	1	95.273
ENSMUSG00000078747	Gm20878	protein_coding	1	48.354	1
96.216	1	86.666	1	95.72
ENSMUSG00000078753	Scgb1b24	protein_coding	1	78.538	1
96.013	1	88.461	1	94.904
ENSMUSG00000078754	Scgb2b3	protein_coding	1	77.182	1
95.211	1	80.381	1	94.904
ENSMUSG00000078757	Scgb1b29	protein_coding	1	81.125	1
97.097	1	86.144	1	96.183
ENSMUSG00000078759	Scgb1b7	protein_coding	1	85.092	NA
NA	1	86.144	1	95.877
ENSMUSG00000078762	Haus5	protein_coding	1.016	72.372	1.102
87.029	0.893	40.794	1.021	92.466
ENSMUSG00000078763	Slfn1	protein_coding	2.083	0.976	0.791	80.052
0.606	35.815	1.834	40.894
ENSMUSG00000078765	U2af1l4	protein_coding	0.867	1.239	1.055
73.092	1.094	47.935	1.006	96.183
ENSMUSG00000078768	Zfp566	protein_coding	0.941	79.165	NA

NA 1.129 84.896 1.206 52.324
ENSMUSG00000078771 Evi2a protein_coding 1.556 0 0.99 87.857
0.648 0.879 1.146 63.708
ENSMUSG00000078772 Gm12353 protein_coding 1.021 84.661
1.405 85.756 0.699 59.383 0.685 84.29
ENSMUSG00000078773 Rad54b protein_coding 1.297 54.717
0.866 94.597 0.887 83.158 1.513 64.513
ENSMUSG00000078776 9530053A07Rik protein_coding 0.974 76.319
0.987 96.847 1.042 86.552 0.745 85.493
ENSMUSG00000078779 Zfp59 protein_coding 1.155 25.944 0.876
73.461 0.989 81.976 1.083 83.067
ENSMUSG00000078780 Gm5150 protein_coding 2.022 19.315
2.086 89.494 0.237 4.935 1.089 87.7
ENSMUSG00000078784 1810022K09Rik protein_coding 0.981 85.655
0.982 96.847 1.04 88.601 1.104 61.984
ENSMUSG00000078786 BC024978 protein_coding 0.88 3.47 1.06
79.29 1.071 57.227 1.038 94.384
ENSMUSG00000078787 Cyp2t4 protein_coding 0.637 18.487
1.106 95.708 1.422 16.205 0.827 93.316
ENSMUSG00000078789 Dph1 protein_coding 1.02 84.452 0.938
91.361 1.047 79.325 0.989 86.699
ENSMUSG00000078794 Dact3 protein_coding 1.095 48.462 1.034
89.434 0.885 20.751 1.022 96.358
ENSMUSG00000078795 Ceacam15 protein_coding 1 85.77 1
75.052 1 43.586 1 90.397
ENSMUSG00000078796 Zfp541 protein_coding 0.957 72.985
1.373 97.155 0.759 84.552 0.663 94.608
ENSMUSG00000078798 Sult2a1 protein_coding 1 86.57 1
92.137 NA NA 1 95.273
ENSMUSG00000078799 Sult2a5 protein_coding 1 83.09 1
96.216 NA NA 1 94.904
ENSMUSG00000078800 Bsph2 protein_coding 1 84.819 1
96.68 1 87.274 1 96.071
ENSMUSG00000078808 Vmn1r58 protein_coding 1.023 65.733
0.813 91.361 1.198 87.786 0.949 86.991
ENSMUSG00000078810 Gp6 protein_coding 1 75.671 1
83.321 1 81.29 1 94.904
ENSMUSG00000078812 Eif5a protein_coding 0.882 0.987 1.043 71.617
1.089 39.925 1.097 59.464
ENSMUSG00000078813 Leng1 protein_coding 0.914 15.456 1.071
82.904 1.023 83.857 1.01 96.358
ENSMUSG00000078815 Cacng6 protein_coding 5.862 5.932 1.368
85.756 0.125 0.72 0.762 95.877
ENSMUSG00000078816 Prkcg protein_coding 0.798 6.409 0.957 95.088
1.308 7.635 1.042 96.192
ENSMUSG00000078821 Gm17414 lincRNA 0.384 8.709 0.838 91.361
3.114 5.864 1.376 78.042
ENSMUSG00000078838 Gm17382 lincRNA 1.466 57.38 1.29 83.104
0.529 26.057 0.786 95.72
ENSMUSG00000078840 Gm10999 protein_coding 1 82.543 1
95.504 1 75.527 1 91.872
ENSMUSG00000078847 Gm6746 pseudogene 1 65.622 1
91.661 1 84.896 1 96.192
ENSMUSG00000078850 Gm2348 pseudogene 1 82.891 1

97.155 1 82.74 1 94.904
ENSMUSG00000078851 Hist3h2a protein_coding 0.926 65.869
1.062 94.48 1.018 55.109 1.009 92.748
ENSMUSG00000078853 Igtp protein_coding 1.047 81.24 1.39 71.222
0.688 8.634 0.922 96.287
ENSMUSG00000078861 Zfp931 protein_coding 0.962 78.744
1.121 82.904 0.927 39.528 0.949 86.699
ENSMUSG00000078862 Gm14326 protein_coding 1.283 3.932 NA NA
0.818 5.884 0.996 96.086
ENSMUSG00000078864 Gm14322 protein_coding 1.103 64.193
0.916 91.955 0.986 78.642 1.037 88.197
ENSMUSG00000078865 Gm14406 protein_coding 1 86.112 1
92.793 1 78.642 1 86.991
ENSMUSG00000078866 Gm14420 protein_coding 0.966 83.418
0.973 88.881 1.059 32.173 1.282 32.045
ENSMUSG00000078867 Gm14418 protein_coding 1.508 57.655
0.872 87.322 0.762 58.271 0.927 94.82
ENSMUSG00000078868 Gm14412 protein_coding 1.116 64.956
0.973 96.407 0.919 42.997 0.87 95.832
ENSMUSG00000078869 Gm14409 protein_coding 1.203 84.397 0.98
86.277 0.848 57.446 1.479 83.067
ENSMUSG00000078870 Gm14410 protein_coding 1.088 66.76 1.067
96.407 0.863 36.499 1.344 68.696
ENSMUSG00000078875 Gm14419 protein_coding 1 79.577 1
96.216 1 84.626 1 94.468
ENSMUSG00000078876 Gm14408 protein_coding 1 85.355 1
95.504 1 88.41 1 96.358
ENSMUSG00000078877 Gm14295 protein_coding 1.245 2.969 NA NA
0.797 0.945 1.016 78.948
ENSMUSG00000078878 Gm14305 protein_coding 0.99 67.374
1.145 95.735 0.883 28.237 1.036 93.156
ENSMUSG00000078879 NA NA NA NA 1.783 90.29 0.391 60.233
0.561 84.29
ENSMUSG00000078880 Gm14308 protein_coding 1 83.823 1
94.96 1 84.308 1 95.877
ENSMUSG00000078881 Gm14434 protein_coding 1 86.464 1
95.329 1 88.349 1 94.752
ENSMUSG00000078886 Gm2026 protein_coding 1 81.01 1
96.138 NA NA 1 91.928
ENSMUSG00000078887 Gm6710 protein_coding 1 78.054 1
96.843 1 80.899 1 96.183
ENSMUSG00000078889 Gm14288 protein_coding 1 86.333 1
91.897 NA NA 1 93.261
ENSMUSG00000078891 Gm11008 lincRNA 1 85.728 NA NA
1 88.601 1 96.183
ENSMUSG00000078894 2210418O10Rik protein_coding 1 75.567
1 95.088 1 87.898 1 96.287
ENSMUSG00000078895 Gm11009 protein_coding 1 81.76 1
95.088 1 85.935 1 96.183
ENSMUSG00000078896 Gm8923 protein_coding 0.958 52.9 1.155
97.129 0.904 74.686 1.027 94.016
ENSMUSG00000078897 Gm4724 protein_coding 1 72.655 1
93.654 1 85.134 1 96.183
ENSMUSG00000078898 Gm4723 protein_coding 1 83.523 1

96.68 1 88.349 1 96.248
ENSMUSG00000078899 Gm4631 protein_coding 0.974 43.019
0.963 85.793 1.068 86.346 1.221 83.276
ENSMUSG00000078901 Gm14440 protein_coding 1 80.146 1
96.216 1 65.034 1 94.904
ENSMUSG00000078902 Gm14443 protein_coding 0.935 64.589
0.748 62.322 1.432 15.241 1.313 59.441
ENSMUSG00000078903 Gm14391 protein_coding 1 82.705 1
95.431 1 77.888 1 96.139
ENSMUSG00000078905 Gm14393 protein_coding 1.236 69.489
1.163 96.308 0.693 48.577 0.879 92.181
ENSMUSG00000078906 Gm14444 protein_coding 1 81.823 1
92.793 1 85.352 1 96.086
ENSMUSG00000078907 Fam186b protein_coding 2.244 33.325
0.686 84.594 0.648 73.531 0.667 89.475
ENSMUSG00000078908 Mon1b protein_coding 1.017 76.968 1.075
71.98 0.914 23.607 0.953 83.724
ENSMUSG00000078912 Gm11011 protein_coding 1 81.01 1
90.138 1 87.431 1 95.35
ENSMUSG00000078917 Gm11281 pseudogene 1 82.982 1
95.211 1 88.657 1 93.584
ENSMUSG00000078919 Dpm1 protein_coding 0.921 24.748 0.897
88.897 1.205 23.604 1.012 94.654
ENSMUSG00000078920 Ifi47 protein_coding 1.361 0.577 1.276 89.996
0.575 4.23 1.159 70.85
ENSMUSG00000078921 Tgtp2 protein_coding 0.974 85.832 1.455
71.617 0.707 6.858 1.006 91.783
ENSMUSG00000078922 Tgtp1 protein_coding 0.781 29.454 1.596
79.261 0.801 49.217 0.823 83.724
ENSMUSG00000078923 Ube2v1 protein_coding 0.992 58.077
0.959 93.146 1.053 86.258 1.085 94.016
ENSMUSG00000078924 Gm12169 protein_coding 1 73.585 1
93.654 1 87.336 1 92.748
ENSMUSG00000078931 Pdf protein_coding 0.816 28.998 0.962
87.998 1.277 16.808 1.018 88.245
ENSMUSG00000078933 Lrrc70 protein_coding 1.018 85.832
0.994 96.216 NA NA 0.824 86.699
ENSMUSG00000078934 9230113P08Rik protein_coding 1 86.464
1 95.708 1 84.308 1 93.132
ENSMUSG00000078935 1700025C18Rik protein_coding 1 82.479
1 96.216 1 88.693 1 94.384
ENSMUSG00000078936 Trappc13 protein_coding 1 86.006 NA
NA NA NA 1 94.215
ENSMUSG00000078937 Cpt1b protein_coding 0.811 51.056 1.054
87.037 1.167 84.896 1.15 95.877
ENSMUSG00000078938 Syce3 protein_coding 1 85.129 1
96.763 1 76.923 1 90.865
ENSMUSG00000078940 Wfdc11 protein_coding 1 84.397 1
92.914 1 77.17 1 95.273
ENSMUSG00000078941 Taf9 protein_coding 0.943 51.56 1.068 94.89
0.994 84.626 1.083 60.466
ENSMUSG00000078942 Naip6 protein_coding 1.407 0 0.772 32.512
0.923 51.443 1.002 94.654
ENSMUSG00000078945 Naip2 protein_coding 1.567 0 0.898 55.71

0.707 6.858 1.085 95.74
ENSMUSG00000078949 R3hdml protein_coding 1.063 76.135 0.83
75.993 1.136 51.243 1.183 74.607
ENSMUSG00000078952 Gm2373 lincRNA 0.879 81.823 0.682
88.897 1.671 48.772 0.964 91.807
ENSMUSG00000078953 Gm11020 protein_coding 1 60.339 1
90.378 1 43.481 1 93.964
ENSMUSG00000078954 Arhgap8 protein_coding 1.143 1.828 0.88
82.503 0.989 42.376 0.895 83.724
ENSMUSG00000078955 Gm14222 sense_intronic 1 81.925 1
96.216 1 87.686 1 96.183
ENSMUSG00000078956 Gm14221 protein_coding 1.043 72 0.975
94.848 0.98 84.071 0.846 88.552
ENSMUSG00000078958 Atp6ap1l protein_coding 1 74.482 1
96.308 1 85.792 1 90.865
ENSMUSG00000078960 Tob2 protein_coding 0.856 73.487 0.915
96.013 1.279 57.637 1.133 94.904
ENSMUSG00000078962 4932414J04Rik lincRNA 1 81.196 1
94.848 1 88.161 1 95.676
ENSMUSG00000078963 Hsbp1l1 protein_coding 1.062 71.674 0.81
89.713 1.156 54.163 0.856 96.183
ENSMUSG00000078964 Ces1b protein_coding 1 86.112 NA NA
1 82.903 1 96.183
ENSMUSG00000078965 Gm12033 pseudogene 1 85.283 1
89.277 1 66.928 1 87.7
ENSMUSG00000078967 Gm3272 pseudogene 1 82.352 1
95.504 1 88.601 1 92.561
ENSMUSG00000078970 Wdr92 protein_coding 1.067 38.195 1.033
90.116 0.907 9.573 0.966 91.372
ENSMUSG00000078972 Gm15557 protein_coding 0.256 24.817
1.214 95.809 3.215 31.4 2.437 87.447
ENSMUSG00000078974 Sec61g protein_coding 1.061 57.38 0.909
85.418 1.039 85.792 1.158 38.624
ENSMUSG00000078982 Gm11025 pseudogene 1 65.622 1
96.897 1 75.809 1 94.904
ENSMUSG00000078984 Gm11027 protein_coding 1 85.832 1
96.68 1 74.535 NA NA
ENSMUSG00000078991 Ighv1-2 IG_LV_gene 1 81.196 1
95.211 1 86.258 1 96.358
ENSMUSG00000078994 Zfp429 protein_coding 1.155 11.459
0.892 69.611 0.969 62.791 1.031 96.287
ENSMUSG00000078995 Zfp456 protein_coding 1.162 45.746
0.899 88.897 0.955 77.294 1.042 87.886
ENSMUSG00000078998 NA NA NA NA 1 95.735 NA NA
1 95.273
ENSMUSG00000079000 7530422B04Rik antisense 0.902 65.869
0.805 88.897 1.373 47.512 0.815 90.611
ENSMUSG00000079001 4930404H24Rik protein_coding 1 78.538
1 96.216 1 80.381 1 96.183
ENSMUSG00000079002 C030006K11Rik protein_coding 0.973 60.569
1.09 87.029 0.945 78.713 0.894 83.724
ENSMUSG00000079003 Samd1 protein_coding 1.012 78.639 1.065
78.629 0.922 27.194 1.026 87.447
ENSMUSG00000079005 Gm14147 protein_coding 1 81.823 1

96.407 1 87.686 1 92.018
ENSMUSG00000079006 Gm14151 protein_coding 1 78.299 1
96.847 1 88.274 1 91.217
ENSMUSG00000079007 Tcl1b4 protein_coding 1 81.823 1
95.504 1 84.724 1 93.316
ENSMUSG00000079008 Gm14124 protein_coding 0.708 83.169
0.592 64.123 2.379 33.802 0.877 89.892
ENSMUSG00000079010 Gm11032 protein_coding 1 84.977 NA
NA 1 87.898 1 95.92
ENSMUSG00000079011 Gm11033 lincRNA 1 80.251 NA NA
1 84.83 1 93.156
ENSMUSG00000079012 Serpina3m protein_coding 0.404 19.365 NA
NA 2.164 22.517 1.619 69.965
ENSMUSG00000079013 Serpina3j protein_coding 0.463 18.731
0.964 96.013 2.242 42.522 0.457 88.505
ENSMUSG00000079014 Serpina3i protein_coding 0.752 24.577
1.275 86.156 1.038 74.686 1.003 78.546
ENSMUSG00000079016 Gm11034 protein_coding 1 69.777 1
92.404 1 84.724 1 96.358
ENSMUSG00000079017 Ifi27l2a protein_coding 1.292 30.497
1.137 79.367 0.681 3.863 0.892 91.105
ENSMUSG00000079018 Ly6c1 protein_coding 1.07 44.694 NA NA
0.977 75.353 1.103 59.441
ENSMUSG00000079019 Insl3 protein_coding 0.801 16.325 1.094
92.793 1.143 43.185 1.051 91.807
ENSMUSG00000079020 Slc45a4 protein_coding 1.044 82.732
0.983 95.211 0.975 86.985 0.956 88.505
ENSMUSG00000079021 Flrt3 protein_coding 1 79.853 1
95.809 1 88.693 1 92.12
ENSMUSG00000079022 Col22a1 protein_coding 0.986 84.049 NA
NA 1.186 81.492 1.526 71.305
ENSMUSG00000079024 Gm21961 protein_coding 1 85.092 1
97.263 1 86.258 1 95.877
ENSMUSG00000079025 Gsdmc protein_coding 1 67.163 1
93.654 1 69.315 1 83.067
ENSMUSG00000079030 Gm2046 protein_coding 1 84.977 1
96.847 1 87.744 1 94.384
ENSMUSG00000079031 BB287469 protein_coding 1 77.497 1
91.897 NA NA 1 96.287
ENSMUSG00000079033 Mef2b protein_coding 1 85.832 NA NA
1 81.169 1 94.904
ENSMUSG00000079036 Alkbh1 protein_coding 1.077 21.146
0.922 56.781 1.007 66.647 1.02 91.928
ENSMUSG00000079037 NA NA NA NA 0.926 84.594 1.089
51.596 1.018 94.904
ENSMUSG00000079038 D130040H23Rik protein_coding 1.533 22.349
1.381 93.899 0.473 14.583 0.771 86.991
ENSMUSG00000079039 Gm11037 protein_coding 0.687 49.047
0.832 82.073 1.755 18.208 NA NA
ENSMUSG00000079042 Trim61 protein_coding 1 85.857 1
85.418 1 87.37 1 96.287
ENSMUSG00000079043 Fastkd5 protein_coding 1.228 31.732
1.125 97.049 0.721 23.607 0.919 96.042
ENSMUSG00000079048 4933413L06Rik antisense 1 86.843 1

96.763 1 85.352 1 95.273
ENSMUSG00000079051 Gm14025 protein_coding 1.053 85.129
0.725 79.475 1.304 55.512 0.606 85.493
ENSMUSG00000079055 Slc8a3 protein_coding 1.803 13.734
1.445 83.104 0.382 2.294 1.196 93.627
ENSMUSG00000079056 Kcnip3 protein_coding 0.744 46.693
1.101 92.561 1.218 71.839 1.18 76.697
ENSMUSG00000079057 Cyp4v3 protein_coding 1.05 48.462 NA
NA 0.945 48.851 1.121 65.558
ENSMUSG00000079058 Adam34 protein_coding 1 85.655 1
96.216 1 84.626 1 96.086
ENSMUSG00000079061 Gm11042 protein_coding 0.843 79.93 1.64
92.253 0.721 85.675 0.642 90.289
ENSMUSG00000079064 Vmn1r-ps123 protein_coding 1 82.982 1
91.661 1 86.552 1 96.358
ENSMUSG00000079065 BC005561 protein_coding 0.923 44.562
1.006 94.767 1.075 72.148 0.994 92.851
ENSMUSG00000079067 Hmgn2-ps1 pseudogene 1.914 15.662 1.484
96.68 0.351 11.751 0.833 84.29
ENSMUSG00000079070 Gm3985 lincRNA 1 84.819 1
94.262 1 87.658 1 94.016
ENSMUSG00000079076 Gm3086 protein_coding 0.431 14.526 NA
NA 2.475 11.348 0.827 93.156
ENSMUSG00000079083 Jrkl protein_coding 1 81.24 NA NA
0.972 83.397 1.079 64.513
ENSMUSG00000079084 Ccdc82 protein_coding 1.106 15.662
0.968 87.919 0.934 56.625 0.925 96.192
ENSMUSG00000079091 Gm3404 protein_coding 1 83.333 1
96.68 1 84.896 1 93.964
ENSMUSG00000079092 Prl2c2 protein_coding 1 67.163 1
95.708 1 78.713 1 96.287
ENSMUSG00000079093 4930449I24Rik protein_coding 1 84.263
1 96.948 1 78.498 1 95.35
ENSMUSG00000079101 Gm2904 pseudogene 1 84.168 1
93.654 1 75.353 1 95.72
ENSMUSG00000079102 Dab2 protein_coding 1 83.169 1
95.708 NA NA 1 95.777
ENSMUSG00000079104 Prps1l3 protein_coding 1.24 9.983 0.954
86.728 0.847 35.264 1.016 81.061
ENSMUSG00000079105 C7 protein_coding 1.348 0.824 0.849 66.09 0.87
23.48 0.984 89.892
ENSMUSG00000079108 Srp54b protein_coding 1 50.913 1
82.503 1 82.161 1 95.92
ENSMUSG00000079109 Pms2 protein_coding 1.023 41.211 0.945
74.851 1.032 78.864 1.063 68.696
ENSMUSG00000079110 Capn3 protein_coding 2.625 4.675 0.899 96.216
0.424 1.871 0.782 94.174
ENSMUSG00000079111 Kdelr2 protein_coding 1.159 1.643 1.032
82.904 0.836 0.945 1.075 69.918
ENSMUSG00000079112 C86695 protein_coding 0.542 65.869 NA
NA 2.327 67.823 0.351 88.053
ENSMUSG00000079113 Gm7861 protein_coding 1 84.263 1
95.088 1 87.971 1 94.904
ENSMUSG00000079114 Gm7849 protein_coding 1 85.655 1

94.367 1 66.753 1 95.35
ENSMUSG00000079116 Gm15293 protein_coding 1 80.251 1
97.155 1 84.308 1 96.086
ENSMUSG00000079120 AY761185 protein_coding 1 84.865 NA
NA 1 79.426 1 96.287
ENSMUSG00000079137 Rpl27-ps1 pseudogene 1.257 82.705 1.157
95.941 0.69 79.776 0.711 92.675
ENSMUSG00000079138 Gm8818 pseudogene 1 75.761 1
96.378 1 81.29 1 76.347
ENSMUSG00000079139 Gm4204 protein_coding 1.08 62.007
0.959 95.809 0.966 48.387 1.066 93.722
ENSMUSG00000079140 Gm6374 pseudogene 1 86.819 1
96.216 NA NA 1 96.086
ENSMUSG00000079143 Gm11052 antisense 1 71.896 1
88.704 1 74.325 1 94.468
ENSMUSG00000079144 A130010J15Rik protein_coding 1.03 53.228
0.939 94.848 1.029 66.425 1.047 83.067
ENSMUSG00000079146 Gm15353 antisense 1.287 85.129 1.75
90.116 0.444 59.726 0.303 83.724
ENSMUSG00000079156 Smok3b protein_coding 1 83.726 1
94.848 1 78.498 1 94.654
ENSMUSG00000079157 Fam155a protein_coding 1.067 68.727 1.05
96.984 0.893 86.112 0.69 94.654
ENSMUSG00000079162 Trim43b protein_coding 1 63.126 NA
NA 1 58.616 1 96.183
ENSMUSG00000079163 Gm15498 lincRNA 1.53 66.944 0.776
95.35 0.843 73.598 0.857 92.181
ENSMUSG00000079164 Tlr5 protein_coding 1.248 2.352 0.863 47.831
0.928 44.678 1.129 91.011
ENSMUSG00000079165 Sap25 protein_coding 1 84.865 1
97.155 1 85.792 1 96.183
ENSMUSG00000079168 Cd209g protein_coding 1.132 30.497
0.952 92.404 0.928 46.071 1.106 96.248
ENSMUSG00000079169 Gm15130 protein_coding 1 79.433 NA
NA 1 87.103 1 96.183
ENSMUSG00000079170 Gm13941 protein_coding 1 82.479 1
88.881 NA NA 1 96.086
ENSMUSG00000079171 Gm13942 lincRNA 1 70.741 1
96.216 1 86.144 1 95.676
ENSMUSG00000079173 Zan protein_coding 0.502 25.551 1.174
91.456 1.702 48.772 1.757 82.66
ENSMUSG00000079174 Gm3054 protein_coding 1 84.263 1
91.661 1 84.434 1 93.964
ENSMUSG00000079175 Gm11060 protein_coding 1 80.815 1
96.407 NA NA 1 96.192
ENSMUSG00000079177 Fam228a protein_coding 1.222 82.543
1.077 87.609 0.759 71.004 1.118 94.016
ENSMUSG00000079179 1700012B15Rik antisense 0.954 29.21 0.959
79.807 1.089 9.301 0.927 83.724
ENSMUSG00000079180 Mptx2 protein_coding 1 81.657 1
91.361 1 70.214 1 91.985
ENSMUSG00000079183 C030005K15Rik protein_coding 1 71.896
1 93.654 1 84.83 1 96.248
ENSMUSG00000079184 Mphosph8 protein_coding 0.97 85.355 0.96

75.618 1.074 41.331 1.114 44.679
ENSMUSG00000079197 Psme2 protein_coding 0.98 83.418 1.037
83.104 0.987 48.851 1.001 89.498
ENSMUSG00000079215 Zfp664 protein_coding 0.769 11.384
0.789 40.946 1.652 6.202 1.127 86.133
ENSMUSG00000079223 Gm8778 pseudogene 1 82.352 1
90.378 1 83.066 1 96.183
ENSMUSG00000079224 Gm6565 pseudogene 1 81.24 1 96.216
1 87.274 1 96.183
ENSMUSG00000079225 Gm9531 pseudogene 1 84.564 1
96.843 NA NA 1 95.273
ENSMUSG00000079227 Ccr5 protein_coding 1.475 1.689 0.871 92.914
0.778 7.083 1.035 79.442
ENSMUSG00000079230 Gm11065 pseudogene 1 78.639 1
93.469 1 85.58 1 95.593
ENSMUSG00000079235 Ccdc13 protein_coding 0.889 83.263
1.471 90.29 0.762 70.791 1.023 90.611
ENSMUSG00000079239 Olfr1082 protein_coding 1 85.391 1
96.843 NA NA 1 95.877
ENSMUSG00000079242 C730034F03Rik protein_coding 0.849 22.481
0.927 96.216 1.264 45.94 0.786 83.724
ENSMUSG00000079243 Xirp1 protein_coding 2.845 3.536 1.53 82.904
0.229 6.858 0.634 94.608
ENSMUSG00000079244 Gm5622 protein_coding 1 86.918 1
93.654 1 86.602 1 96.183
ENSMUSG00000079246 Slc35f4 protein_coding 1 78.639 1
95.941 1 85.757 1 94.468
ENSMUSG00000079247 Gm13691 protein_coding 1 81.125 1
95.708 1 84.071 1 95.676
ENSMUSG00000079254 Itprip protein_coding 1.142 39.416
0.888 92.631 0.983 61.288 0.875 85.493
ENSMUSG00000079258 NA NA NA NA 1 93.654 1
85.792 1 95.72
ENSMUSG00000079259 Trim71 protein_coding 1 84.049 1
92.111 1 88.274 1 93.016
ENSMUSG00000079260 Tmppe protein_coding 1.081 18.635 1.034
87.609 0.896 4.23 0.931 92.181
ENSMUSG00000079261 Gm15217 protein_coding 1 84.168 NA
NA 1 81.29 1 94.752
ENSMUSG00000079262 Slco1a6 protein_coding 0.747 67.477
0.654 68.818 2.046 25.564 1.36 83.067
ENSMUSG00000079263 Gm6614 protein_coding 1 79.277 1
97.129 1 83.925 1 93.457
ENSMUSG00000079265 Gm8257 protein_coding 1 86.199 1
94.48 1 78.713 1 96.248
ENSMUSG00000079266 Gm6490 pseudogene 1 83.333 NA NA
1 84.669 1 92.466
ENSMUSG00000079267 Gm5930 protein_coding 1 86.74 1
95.941 1 86.552 1 93.722
ENSMUSG00000079269 Gm3676 protein_coding 1 78.538 1
96.001 1 80.381 1 95.593
ENSMUSG00000079271 1700049E17Rik1 protein_coding 1 86.819
1 93.529 1 86.985 1 94.567
ENSMUSG00000079272 Gm7152 pseudogene 1 84.564 1

96.138	1	86.144	1	95.74
ENSMUSG00000079273	Gm17165	pseudogene	1	80.146	1
96.216	1	86.258	1	95.559
ENSMUSG00000079276	Gm4736	protein_coding	1	84.049	1
95.211	1	84.669	1	93.584
ENSMUSG00000079277	Hoxd3	protein_coding	0.962	42.722	0.985
97.263	1.052	40.875	0.967	85.493
ENSMUSG00000079278	Tmem233	protein_coding	9.975	4.465	1.139
85.378	0.088	0.72	1.102	94.904
ENSMUSG00000079283	2310009B15Rik	protein_coding	0.9	33.745
1.032	94.367	1.077	61.743	0.985	93.045
ENSMUSG00000079284	Fbxw28	protein_coding	1	85.728	1
94.262	1	82.161	1	95.877
ENSMUSG00000079293	Clec7a	polymorphic_pseudogene	1.456	32.676
0.852	82.671	0.803	66.753	0.438	86.699
ENSMUSG00000079295	Klrb1-ps1	pseudogene	1	48.462	1
96.984	1	69.134	1	94.215
ENSMUSG00000079299	Klrb1	protein_coding	1	86.112	NA	NA
1	84.83	1	91.105
ENSMUSG00000079311	Gm3222	pseudogene	1	85.77	1	96.138
1	83.538	1	95.35
ENSMUSG00000079316	Rab9	protein_coding	0.995	86.199	0.948
82.073	1.056	54.673	1.037	86.133
ENSMUSG00000079317	Trappc2	protein_coding	0.97	63.736
1.067	83.104	0.965	71.999	0.928	85.493
ENSMUSG00000079323	Gm20661	protein_coding	0.888	81.465	NA
NA	1.037	76.754	0.644	89.892
ENSMUSG00000079324	4932414N04Rik	protein_coding	1	74.689
1	95.708	1	81.032	1	95.72
ENSMUSG00000079326	Gm15262	lincRNA	1	83.823	1
93.654	1	84.504	1	95.559
ENSMUSG00000079333	Gm11096	protein_coding	1	64.956	1
96.847	1	82.31	1	96.358
ENSMUSG00000079334	Nat6	protein_coding	0.928	4.874	1.087	71.617
0.99	69.196	1.037	61.984
ENSMUSG00000079339	Gm14446	protein_coding	2.709	1.441	1.057
96.897	0.349	6.858	1.064	83.865
ENSMUSG00000079342	Gm5097	protein_coding	1	81.196	1
95.708	NA	NA	1	95.676
ENSMUSG00000079343	Gm5077	protein_coding	1.339	51.189
1.058	96.897	0.704	20.751	0.768	90.805
ENSMUSG00000079344	Lipo4	protein_coding	1	78.299	1
94.597	1	86.552	1	94.153
ENSMUSG00000079346	1700013D24Rik	protein_coding	1	79.577
1	96.138	1	86.112	1	95.74
ENSMUSG00000079349	Magea5	protein_coding	1	64.014	1
96.407	1	88.203	1	95.74
ENSMUSG00000079350	Magea8	protein_coding	1	83.418	1
97.049	1	84.724	1	93.627
ENSMUSG00000079353	Gm11099	protein_coding	1	81.01	1
96.652	1	87.744	1	94.904
ENSMUSG00000079355	Ackr4	protein_coding	0.757	5.204	0.966	83.876
1.368	5.864	0.915	86.445
ENSMUSG00000079357	Gm11100	antisense	0.72	33.599	0.927

96.013 1.497 29.819 1.167 96.071
ENSMUSG00000079359 Gm9006 pseudogene 1 79.577 1
95.504 NA NA 1 95.832
ENSMUSG00000079360 Gm3839 pseudogene 1 81.465 1
96.407 1 83.857 1 94.752
ENSMUSG00000079362 Gbp6 protein_coding 0.994 83.169 1.166
79.29 0.865 45.093 1.083 96.192
ENSMUSG00000079363 Gbp4 protein_coding 0.981 83.09 1.305 82.904
0.781 35.559 0.926 89.294
ENSMUSG00000079364 Gm3558 protein_coding 0.755 14.403
0.992 96.216 1.333 27.475 0.816 84.29
ENSMUSG00000079371 Gm3476 protein_coding 1 86.474 1
96.407 1 84.961 1 94.215
ENSMUSG00000079374 Gm8334 protein_coding 1 83.169 1
96.763 1 77.294 1 94.608
ENSMUSG00000079378 Gm8279 protein_coding 1 81.465 NA
NA NA NA 1 95.877
ENSMUSG00000079379 NA NA NA NA NA NA 1 83.275
1 92.018
ENSMUSG00000079380 Gm8271 protein_coding 1 80.03 NA NA
1 86.985 1 94.654
ENSMUSG00000079382 Gm3443 protein_coding 1 83.263 1
95.708 1 87.898 1 94.904
ENSMUSG00000079383 Gm3298 protein_coding 1 73.841 1
91.661 1 80.634 1 95.273
ENSMUSG00000079386 Gm3173 protein_coding 1 72.743 1
94.262 1 83.066 1 95.273
ENSMUSG00000079387 Luzp4 protein_coding 1 86.145 1
95.088 1 88.274 1 95.832
ENSMUSG00000079389 Gm3149 protein_coding 1 83.09 NA NA
1 72.695 1 93.156
ENSMUSG00000079391 Gm2974 protein_coding 1 85.728 1
96.897 1 86.552 1 96.248
ENSMUSG00000079392 Gm17170 pseudogene 1 85.655 1
96.138 1 79.625 1 96.183
ENSMUSG00000079395 Gm15114 protein_coding 1 75.399 1
96.847 1 87.658 1 95.74
ENSMUSG00000079396 Gm3411 protein_coding 0.825 85.637
2.455 92.931 0.493 86.727 0.814 93.685
ENSMUSG00000079402 Gm3020 protein_coding 1 81.01 NA NA
1 86.602 1 90.455
ENSMUSG00000079407 1700110I01Rik protein_coding 1 83.333
1 92.931 1 86.602 1 95.273
ENSMUSG00000079409 Gm5795 protein_coding 1 73.734 1
96.407 1 86.727 1 94.654
ENSMUSG00000079410 Gm2897 protein_coding 0.666 3.07 1.219
82.904 1.229 32.173 1.044 91.783
ENSMUSG00000079414 Gm11110 protein_coding 0.805 31.614
1.163 82.904 1.066 74.325 1.007 94.297
ENSMUSG00000079415 Cntf protein_coding 0.872 62.158 0.789
94.887 1.455 57.907 0.988 94.904
ENSMUSG00000079416 Gm11111 protein_coding 1 78.538 1
97.263 1 86.727 1 96.184
ENSMUSG00000079418 Atg4a protein_coding 0.864 14.526 0.931

74.633 1.242 7.635 0.815 95.92
ENSMUSG00000079419 Ms4a6c protein_coding 1.595 0.461 0.883
60.678 0.709 33.402 1.397 52.324
ENSMUSG00000079421 4930402F06Rik protein_coding 1 86.112
1 90.378 1 83.066 1 95.273
ENSMUSG00000079423 Gm3286 protein_coding 1 86.199 1
97.263 1 82.778 1 93.964
ENSMUSG00000079424 Gm3259 protein_coding 1 82.092 1
95.708 1 88.161 1 96.287
ENSMUSG00000079426 Arpc4 protein_coding 1.175 0.2 0.988 95.504
0.862 1.061 0.974 83.865
ENSMUSG00000079427 Gm2382 protein_coding 1.011 51.189
0.987 73.166 1.003 67.562 0.989 95.832
ENSMUSG00000079428 Tceal7 protein_coding 1.495 29.372
0.807 91.776 0.825 18.539 0.754 92.181
ENSMUSG00000079429 Mroh2a protein_coding 0.623 15.456
1.143 96.407 1.407 24.691 1.054 95.273
ENSMUSG00000079432 Gm15023 protein_coding 1 85.092 1
96.268 1 86.666 1 96.183
ENSMUSG00000079433 Gm11114 protein_coding 1 86.464 1
96.216 1 84.308 1 96.192
ENSMUSG00000079434 Neu2 protein_coding 0.959 85.857 1.074
85.756 0.968 74.179 0.833 94.384
ENSMUSG00000079435 Rpl36a protein_coding 1.051 24.689
0.932 38.923 1.019 79.625 1.089 78.948
ENSMUSG00000079436 Kcnj13 protein_coding 1.442 65.537
0.805 83.58 0.86 79.426 0.825 91.872
ENSMUSG00000079437 Tmem179b protein_coding 0.977 44.787
1.086 71.617 0.941 52.213 0.955 92.466
ENSMUSG00000079438 Gm11115 protein_coding 1 74.299 1
96.984 1 87.37 1 96.248
ENSMUSG00000079439 Gm11116 protein_coding 1 85.203 1
97.097 1 84.308 1 95.593
ENSMUSG00000079440 Alpi protein_coding 1 85.728 1
96.407 1 83.158 1 91.372
ENSMUSG00000079442 St6galnac4 protein_coding 1.192 5.313 0.984
94.102 0.852 23.86 1.079 79.268
ENSMUSG00000079444 Gm21981 protein_coding 0.716 45.229
1.389 83.104 1.003 87.898 0.998 94.904
ENSMUSG00000079445 B3gnt7 protein_coding 1.568 18.314
0.605 25.341 1.048 75.809 0.893 93.612
ENSMUSG00000079450 3110007F17Rik protein_coding 1.204 8.996
1.128 83.104 0.736 2.333 0.949 92.12
ENSMUSG00000079451 Tmprss11g protein_coding 1 84.865 1
94.102 1 83.607 1 90.865
ENSMUSG00000079457 Gm7609 pseudogene 1.822 0 0.888 62.322
0.618 7.329 1.377 59.394
ENSMUSG00000079460 4933403O08Rik protein_coding 1 80.608
1 95.708 1 84.896 1 93.316
ENSMUSG00000079462 Gm15737 protein_coding 0.85 55.939
1.207 87.604 0.974 85.58 1.004 86.699
ENSMUSG00000079465 Col4a3 protein_coding 1.228 67.163
1.006 93.863 0.809 59.922 0.967 96.358
ENSMUSG00000079466 Prdm12 protein_coding 1 81.925 1

97.288 1 87.173 1 94.608
ENSMUSG00000079467 Gm14966 antisense 0.893 64.014 0.997
96.948 1.12 55.109 0.996 92.181
ENSMUSG00000079469 Pigb protein_coding 0.9 37.531 NA NA
1.053 43.307 0.922 85.493
ENSMUSG00000079470 Utp14b protein_coding 1.29 21.703
1.105 71.617 0.699 4.759 0.938 95.593
ENSMUSG00000079476 1700011M02Rik protein_coding 1 80.608
1 93.574 1 79.776 1 95.273
ENSMUSG00000079477 Rab7 protein_coding 1.043 68.485 1.019
90.116 0.945 44.678 1.086 65.558
ENSMUSG00000079478 Sssca1 protein_coding 0.93 19.83 1.043
90.29 1.032 53.882 1.043 71.305
ENSMUSG00000079479 Gm9112 protein_coding 1 80.51 1
93.899 1 88.693 1 96.287
ENSMUSG00000079480 Pin4 protein_coding 1.045 77.497 0.91
79.475 1.053 65.567 1.105 69.404
ENSMUSG00000079481 Nhsl2 protein_coding 1.111 20.444 0.938
87.857 0.955 31.135 0.981 94.19
ENSMUSG00000079484 Phyhd1 protein_coding 0.882 42.361
0.674 38.699 1.685 5.864 1.148 59.464
ENSMUSG00000079487 Med12 protein_coding 0.95 17.705 0.99
91.361 1.063 24.07 0.982 88.505
ENSMUSG00000079488 Cd47 pseudogene 1 81.125 1 97.288
1 86.985 1 95.72
ENSMUSG00000079489 C030013D06Rik sense_intronic 0.844 71.915
0.779 89.509 1.517 56.708 0.872 92.018
ENSMUSG00000079491 H2-T10 polymorphic_pseudogene 1.045 51.826
0.971 92.631 0.981 83.397 0.976 94.608
ENSMUSG00000079492 Gm11127 protein_coding 0.967 84.738
1.342 88.136 0.768 49.217 0.857 85.493
ENSMUSG00000079494 Cml5 protein_coding 1.835 86.351 0.534
96.013 1.015 88.693 0.785 90.674
ENSMUSG00000079495 Gm11128 protein_coding 1.018 85.77 0.938
95.708 1.045 84.669 1.433 95.559
ENSMUSG00000079499 6530402F18Rik processed_transcript 0.967
84.865 1.233 73.092 0.84 20.751 1.113 83.067
ENSMUSG00000079501 Gm5138 pseudogene 1 86.145 NA NA
1 86.552 1 95.777
ENSMUSG00000079502 1700101E01Rik protein_coding 1.19 68.727
0.926 90.053 0.905 85.21 1.553 71.855
ENSMUSG00000079505 Gm11131 antisense 0.997 67.477 1.004
93.899 0.998 87.744 0.874 84.29
ENSMUSG00000079507 H2-Q1 protein_coding 0.902 73.585 1.285
93.899 0.864 88.601 1 95.593
ENSMUSG00000079508 Apoo protein_coding 1.024 53.138 1.084
85.378 0.901 87.37 1.031 80.573
ENSMUSG00000079509 Zfx protein_coding 1.073 0.2 0.967 76.978
0.966 44.678 0.993 93.261
ENSMUSG00000079510 Mdga2 protein_coding 1 86.006 1
88.345 1 85.675 1 96.184
ENSMUSG00000079511 Ccdc142 protein_coding 0.964 52.258
0.993 95.504 1.043 49.898 1.02 96.358
ENSMUSG00000079513 4932429P05Rik protein_coding 1 74.689

1	96.652	1	73.309	1	96.086
ENSMUSG00000079516	Reg3a	protein_coding	1	82.092	NA	NA
1	88.274	1	93.964
ENSMUSG00000079517	Gm8787	protein_coding	1.664	41.951
0.735	73.766	0.818	86.727	0.831	96.192
ENSMUSG00000079519	Gm14743	protein_coding	1	84.738	1
93.469	1	83.275	1	95.273
ENSMUSG00000079521	Gm5938	protein_coding	1	86.145	1
96.216	1	88.161	1	95.273
ENSMUSG00000079522	Gm14744	protein_coding	1	71.142	1
94.767	1	81.976	1	94.215
ENSMUSG00000079523	Tmsb10	protein_coding	1.174	27.731
0.912	74.483	0.936	59.383	1.089	76.697
ENSMUSG00000079524	Gm15402	antisense	1	67.163	1
95.504	1	86.831	1	93.685
ENSMUSG00000079525	4930468A15Rik	protein_coding	1	77.182
1	93.817	1	84.626	1	95.593
ENSMUSG00000079528	1700021O21Rik	antisense	1	71.763	1
94.597	1	88.203	1	96.139
ENSMUSG00000079531	Gm5936	protein_coding	1	63.804	1
87.322	1	85.464	1	96.287
ENSMUSG00000079532	Gm6890	protein_coding	1	77.497	1
96.013	1	87.103	1	95.593
ENSMUSG00000079536	Gm6880	protein_coding	1	85.832	1
94.102	1	82.903	1	87.886
ENSMUSG00000079537	C030048H21Rik	protein_coding	0.796	31.333
0.94	96.216	1.338	37.259	0.827	91.372
ENSMUSG00000079542	Gm11143	protein_coding	1	80.815	1
95.809	1	85.921	1	92.903
ENSMUSG00000079543	NA	NA	NA	NA	1	96.216	1
86.475	1	91.269
ENSMUSG00000079544	Igkv13-87	protein_coding	1	86.464	1
96.268	1	87.37	1	95.92
ENSMUSG00000079546	Gm11146	protein_coding	1	78.299	1
93.146	1	88.461	1	94.904
ENSMUSG00000079547	H2-DMb1	protein_coding	1.201	34.861
0.911	70.864	0.91	85.675	0.818	84.29
ENSMUSG00000079550	Mpp4	protein_coding	1.153	57.133	0.608
43.505	1.426	37.741	0.746	86.699
ENSMUSG00000079553	Kifc1	protein_coding	1.151	37.941	0.738
51.277	1.181	81.29	1.309	83.067
ENSMUSG00000079554	Aox2	protein_coding	2.33	17.761	1.139
95.211	0.377	29.451	1.177	95.273
ENSMUSG00000079555	Haus3	protein_coding	0.93	38.286	0.966
97.263	1.111	43.307	1.115	69.708
ENSMUSG00000079557	2-Mar	protein_coding	0.983	45.502	0.989
94.597	1.027	40.875	0.978	86.445
ENSMUSG00000079559	Gm684	protein_coding	0.991	56.768	1.015
94.89	0.995	82.778	1.085	74.896
ENSMUSG00000079560	Hoxa3	protein_coding	0.857	27.664	1.028
96.763	1.135	49.058	0.985	96.248
ENSMUSG00000079562	Maea	protein_coding	1.034	41.211	0.98
85.418	0.982	78.864	1.017	83.067
ENSMUSG00000079563	Pglyrp2	protein_coding	0.656	53.138

1.723 72.642 0.88 66.753 1.361 83.067
ENSMUSG00000079564 Gm11149 protein_coding 0.782 64.284
1.187 91.643 NA NA 0.721 96.183
ENSMUSG00000079566 Spin2d protein_coding 1 78.054 1
96.984 1 80.015 1 93.734
ENSMUSG00000079575 Rbpsuh-rs3 pseudogene 1 81.01 1 94.262
1 76.498 1 93.685
ENSMUSG00000079577 Gm14692 protein_coding 1 84.263 1
93.094 1 83.607 1 93.117
ENSMUSG00000079578 Gm1140 protein_coding 1 86.333 1
95.504 1 55.352 1 93.627
ENSMUSG00000079579 Gm6760 protein_coding 1 81.196 1
91.661 NA NA 1 93.964
ENSMUSG00000079583 Gm7073 protein_coding 1 85.655 1
97.263 1 86.084 1 93.964
ENSMUSG00000079584 Gm364 protein_coding 1 85.832 1
87.041 1 76.923 1 95.273
ENSMUSG00000079588 Tmem182 protein_coding 0.77 3.623 1.308
71.98 0.994 81.492 1.047 92.762
ENSMUSG00000079592 C1qtnf5 protein_coding 1 84.263 NA
NA 1 71.004 1 94.904
ENSMUSG00000079593 Gm14597 protein_coding 0.669 4.115 1.306
79.411 1.147 55.109 0.865 95.92
ENSMUSG00000079594 BC117090 protein_coding 1 78.538 1
93.817 1 85.675 1 96.086
ENSMUSG00000079595 Gm4758 protein_coding 1 84.324 1
93.654 1 88.076 1 95.72
ENSMUSG00000079597 Gm5483 protein_coding 1 78.054 NA
NA 1 85.792 1 93.734
ENSMUSG00000079598 Clec2l protein_coding 0.75 72.546
0.686 95.35 1.935 74.325 0.864 92.12
ENSMUSG00000079600 Gm17604 protein_coding 1 78.428 1
94.102 1 81.857 1 86.209
ENSMUSG00000079602 9230102O04Rik protein_coding 0.624 12.624
NA NA 1.429 26.912 0.881 84.29
ENSMUSG00000079603 Gm13218 lincRNA 1 84.865 1
96.216 1 83.719 1 95.559
ENSMUSG00000079604 Gm13219 lincRNA 0.921 82.982 0.979
96.68 1.107 87.519 0.822 83.724
ENSMUSG00000079605 Zbtb9 protein_coding 1 70.741 1
96.138 1 86.666 1 91.291
ENSMUSG00000079608 Stard6 protein_coding 1.089 66.944
0.968 92.631 0.946 70.44 1.098 76.467
ENSMUSG00000079610 Ankrd39 protein_coding 0.984 71.36 1.061
81.039 0.958 41.154 0.976 91.985
ENSMUSG00000079614 Seh1l protein_coding 1.03 26.77 1.003 92.931
0.965 15.778 0.963 87.651
ENSMUSG00000079615 Hspa14 protein_coding 1 76.396 1
95.196 1 88.203 1 87.7
ENSMUSG00000079619 Cypt15 protein_coding 1 81.178 1
94.48 1 88.349 1 93.722
ENSMUSG00000079620 Muc4 protein_coding 1.478 71.516 NA NA
0.773 73.027 0.814 86.991
ENSMUSG00000079625 Tm4sf19 protein_coding 0.93 79.577

1.269 92.931 0.847 84.434 0.74 96.287
ENSMUSG00000079626 Gm14543 protein_coding 1 77.763 NA
NA 1 78.369 1 95.72
ENSMUSG00000079627 Rhox2h protein_coding 1 83.09 1
95.708 1 84.83 1 95.593
ENSMUSG00000079628 Rhox4g protein_coding 1 85.355 1
96.216 1 76.498 1 93.734
ENSMUSG00000079629 Rhox2g protein_coding 1 75.399 1
96.268 1 87.971 1 92.851
ENSMUSG00000079630 Rhox4f protein_coding 1 81.01 1
95.708 1 81.62 1 95.74
ENSMUSG00000079633 Rhox4d protein_coding 1 84.865 1
93.654 1 87.658 1 94.001
ENSMUSG00000079635 Rhox4c protein_coding 1 78.909 NA
NA 1 74.535 1 92.389
ENSMUSG00000079636 Rhox3c protein_coding 1 86.819 1
96.68 NA NA 1 95.92
ENSMUSG00000079637 Rhox2c protein_coding 1 83.263 1
95.708 1 86.258 1 96.183
ENSMUSG00000079638 Rhox2b protein_coding 1 82.092 1
95.504 1 87.173 1 96.248
ENSMUSG00000079639 Rhox4a protein_coding 1 61.49 NA NA
1 79.625 1 94.904
ENSMUSG00000079641 Rpl39 protein_coding 1.141 20.444 0.927
46.764 0.947 83.158 0.991 91.872
ENSMUSG00000079642 Gm6268 protein_coding 1 81.01 1
97.263 1 86.666 1 96.358
ENSMUSG00000079644 Gm1110 protein_coding 0.515 57.014
1.015 95.211 1.909 46.575 0.865 95.92
ENSMUSG00000079645 Gm17193 sense_intronic 0.999 84.534
1.077 96.216 0.931 88.461 0.848 95.72
ENSMUSG00000079649 Gm2892 pseudogene 1 85.832 1
96.68 1 87.37 1 96.184
ENSMUSG00000079652 Fam71f2 protein_coding 1.005 82.705
0.797 80.463 1.25 49.384 1.009 93.156
ENSMUSG00000079654 Prrt4 protein_coding 0.546 2.668 1.12 96.68
1.642 10.819 0.891 96.183
ENSMUSG00000079655 Gm5168 protein_coding 1 78.909 1
95.708 1 75.009 1 93.457
ENSMUSG00000079657 Rab26 protein_coding 0.918 71.763 0.775
72.399 1.403 36.011 0.971 93.964
ENSMUSG00000079658 Tceb1 protein_coding 1.019 80.03 0.904 74.483
1.084 39.471 1.066 62.918
ENSMUSG00000079659 Tmem243 protein_coding 0.97 84.049 NA
NA 0.973 86.084 1.049 96.248
ENSMUSG00000079662 Ntn3 protein_coding 0.799 11.726 1.121
80.775 1.119 52.8 0.948 86.699
ENSMUSG00000079666 1700015F17Rik protein_coding 1 47.174
1 97.263 1 72.853 1 94.904
ENSMUSG00000079669 Gm17396 processed_transcript 0.825 43.816
1.206 79.055 1.008 84.071 0.917 89.587
ENSMUSG00000079671 2610203C22Rik protein_coding 0.886 59.358
1.069 91.065 1.059 79.897 0.983 91.979
ENSMUSG00000079677 Fdx1l protein_coding 0.901 12.084 1.063

79.864	1.045	50.636	1.051	86.301
ENSMUSG00000079679	Vwde	protein_coding	1	60.707	1
89.494	1	85.792	1	94.82
ENSMUSG00000079681	Zglp1	protein_coding	0.802	77.615	1.702
86.34	0.729	46.956	1.001	91.078
ENSMUSG00000079685	Ulbp1	protein_coding	0.849	56.242	1.191
83.104	0.988	86.552	1.539	40.729
ENSMUSG00000079693	Vmn1r-ps150	pseudogene	1	78.909	1
97.155	1	80.163	1	95.35
ENSMUSG00000079698	Vmn2r93	protein_coding	1	86.918	NA
NA	1	88.693	1	92.181
ENSMUSG00000079699	Gm6592	protein_coding	1	74.8	1
87.857	1	70.623	1	94.82
ENSMUSG00000079700	Fpr3	protein_coding	1	86.145	1
93.707	NA	NA	1	95.559
ENSMUSG00000079701	Ssxb3	protein_coding	1	86.474	1
96.763	1	86.602	1	95.877
ENSMUSG00000079702	Ssxb6	protein_coding	1	82.092	1
91.361	NA	NA	1	95.877
ENSMUSG00000079703	Ssxb8	protein_coding	1	81.01	1	96.216
1	86.666	1	95.877
ENSMUSG00000079704	Gm14459	protein_coding	1	84.324	1
95.35	1	88.461	1	95.877
ENSMUSG00000079705	Ssxb1	protein_coding	1	69.777	1
96.652	1	88.274	1	93.627
ENSMUSG00000079707	Tcte3	protein_coding	1	60.707	NA	NA
1	74.686	1	94.297
ENSMUSG00000079709	Gm3435	protein_coding	1.305	20.296
0.742	73.166	1.032	83.811	0.766	85.493
ENSMUSG00000079710	Gm3448	protein_coding	0.869	67.477
0.763	69.611	1.516	20.886	1.164	83.067
ENSMUSG00000079711	Smok4a	protein_coding	0.653	84.294
1.916	87.029	0.8	41.662	0.623	83.724
ENSMUSG00000079712	Gm8597	protein_coding	1	62.354	1
88.881	1	77.532	1	95.593
ENSMUSG00000079716	Map3k4	protein_coding	0.885	30.136
1.051	90.092	1.072	55.109	0.834	83.724
ENSMUSG00000079722	Ttll2	protein_coding	1.954	8.847	0.563	86.728
0.912	66.425	0.658	84.19
ENSMUSG00000079733	Tmem181b-ps	protein_coding	0.984	85.59	1.063
95.088	0.952	84.669	0.887	87.992
ENSMUSG00000079737	3110001I22Rik	protein_coding	1.133	67.608
0.978	96.407	0.904	56.625	1.02	86.133
ENSMUSG00000079740	Gm11172	protein_coding	1	81.465	1
95.301	1	83.925	1	96.287
ENSMUSG00000079741	Nlrp4g	protein_coding	1	76.135	1
95.431	1	79.524	1	96.183
ENSMUSG00000079797	Gm21784	pseudogene	1	82.705	NA	NA
1	83.925	1	92.018
ENSMUSG00000079806	Gm21719	protein_coding	1	81.01	NA	NA
1	86.831	1	95.832
ENSMUSG00000079842	Spag11a	protein_coding	1	86.464	1
95.708	1	80.163	1	94.904
ENSMUSG00000079845	Xlr4a	protein_coding	1.066	71.763	0.843

						96.843	1.112	77.888	1.451	44.679
ENSMUSG00000079852	Klra4	protein_coding	0.736	66.897	1.58	79.29	0.86	68.584	0.924	96.248
ENSMUSG00000079875	Olfr22-ps1	pseudogene	1	83.823	1	96.847	1	87.786	1	94.654
ENSMUSG00000079884	Gm10698	pseudogene	1	84.263	NA	NA	1	82.74	1	96.086
ENSMUSG00000079890	Gm12106	pseudogene	1	83.169	1	96.897	1	80.381	1	92.018
ENSMUSG00000079892	Scgb1b22-ps	pseudogene	1	80.608	1	96.847	1	87.431	1	96.086
ENSMUSG00000079893	Scgb1b23-ps	pseudogene	1	83.523	NA	NA	1	86.552	1	94.82
ENSMUSG00000079906	Gm15846	pseudogene	0.363	40.422	1.594	94.767	1.732	43.842	1.674	79.442
ENSMUSG00000079941	Gm11273	protein_coding	1	76.968	1	92.137	NA	NA	1	88.755
ENSMUSG00000079962	Gm12643	pseudogene	1	81.76	1	93.654	1	72.695	1	94.153
ENSMUSG00000079963	Gm7146	pseudogene	1	85.832	1	91.897	1	86.552	1	93.413
ENSMUSG00000079966	Gm14058	pseudogene	1	81.465	1	97.155	1	88.461	1	90.397
ENSMUSG00000079988	Gm11970	pseudogene	1	85.728	1	91.955	1	86.602	1	94.904
ENSMUSG00000079998	Gm12947	pseudogene	1	83.658	1	96.216	1	82.685	1	93.722
ENSMUSG00000080002	Gm5566	pseudogene	1	85.129	1	96.138	1	88.161	1	96.287
ENSMUSG00000080006	Rps19-ps7	pseudogene	1	83.418	1	91.361	1	88.601	1	93.734
ENSMUSG00000080021	Gm5915	pseudogene	1	75.298	1	95.504	1	76.923	1	81.251
ENSMUSG00000080046	Gm15108	pseudogene	1	76.546	1	97.263	1	80.089	1	95.877
ENSMUSG00000080050	Gm14864	pseudogene	1	72.546	1	92.474	1	84.308	1	92.691
ENSMUSG00000080051	Gm14510	pseudogene	1	86.145	1	95.735	1	81.032	1	96.071
ENSMUSG00000080057	Gm12378	pseudogene	1	76.135	1	95.211	1	83.538	1	93.457
ENSMUSG00000080058	Gm11175	protein_coding	1.062	68.846	1.088	92.561	0.861	87.744	0.822	94.654
ENSMUSG00000080059	Rps19-ps3	pseudogene	0.869	54.87	0.806	82.503	1.429	15.411	1.072	96.183
ENSMUSG00000080065	Gm11864	pseudogene	1	81.196	NA	NA	1	83.719	1	85.629
ENSMUSG00000080069	Gm41	pseudogene	1	86.464	1	96.335	1	84.552	1	95.777
ENSMUSG00000080071	Gm15125	pseudogene	1	82.231	1	94.848	1	87.898	1	96.358
ENSMUSG00000080072	Spin2-ps2	pseudogene	1	78.639	1	95.735	1	82.575	1	92.748
ENSMUSG00000080073	Gm9427	pseudogene	1	84.324	1					

94.597	1	87.744	1	95.74
ENSMUSG00000080076	Hist1h2aj	pseudogene	1	78.639	1
84.858	1	70.623	1	96.248
ENSMUSG00000080078	Gm15583	pseudogene	1	77.497	1
93.654	1	84.229	1	95.832
ENSMUSG00000080115	Mettl21b	protein_coding	0.896	56.768
1.289	79.579	0.867	68.82	1.207	63.708
ENSMUSG00000080142	Gm12838	pseudogene	1	80.146	1
95.431	1	80.015	1	92.762
ENSMUSG00000080155	Gm14777	pseudogene	1	83.09	1	95.708
NA	NA	1	95.273
ENSMUSG00000080158	Gm14385	pseudogene	1	85.59	NA	NA	1
85.58	1	92.762
ENSMUSG00000080175	Tpm3-rs2	pseudogene	1	84.661	1
96.843	1	86.552	1	92.018
ENSMUSG00000080180	Gm17235	pseudogene	1	78.909	1
96.138	1	72.148	1	96.183
ENSMUSG00000080186	Gm14448	pseudogene	1	86.843	1
97.263	1	86.727	1	94.904
ENSMUSG00000080193	Gm14756	pseudogene	1	69.943	1
95.211	1	87.744	1	96.071
ENSMUSG00000080198	Gm13573	pseudogene	0.605	48.146	0.898
78.877	1.837	20.968	0.673	87.992
ENSMUSG00000080205	Gm15526	pseudogene	0.59	80.251	0.767
85.116	2.217	39.471	0.929	90.674
ENSMUSG00000080222	Gm11486	pseudogene	1	85.77	1	96.378
1	84.724	1	95.74
ENSMUSG00000080223	Gm13768	pseudogene	1	84.661	1
91.661	1	81.388	1	92.748
ENSMUSG00000080227	Gm13343	pseudogene	1	81.01	1	95.504
1	82.161	1	87.7
ENSMUSG00000080241	Gm6269	pseudogene	1	86.351	1
95.809	1	87.786	1	93.734
ENSMUSG00000080250	Gm16400	pseudogene	1	81.01	1	94.597
1	78.293	1	95.273
ENSMUSG00000080266	Gm14772	pseudogene	1	78.538	1
95.708	1	86.475	1	96.248
ENSMUSG00000080268	Brms1	protein_coding	0.931	14.221	1.003
95.809	1.066	41.6	1.014	83.067
ENSMUSG00000080298	Gm14807	pseudogene	1	74.516	1
93.654	1	85.792	1	96.086
ENSMUSG00000080299	Gm14350	pseudogene	1	72.743	1
97.263	1	88.729	1	94.82
ENSMUSG00000080300	Spin2-ps1	pseudogene	1	83.418	NA	NA
1	84.961	1	96.183
ENSMUSG00000080303	Gm12419	pseudogene	1	86.172	1
97.155	1	87.519	1	95.74
ENSMUSG00000080316	4930546H06Rik	protein_coding	0.876	0.987	0.96
96.652	1.19	28.979	1.055	74.607
ENSMUSG00000080317	Vmn2r-ps94	pseudogene	1	79.853	1
96.216	1	84.308	1	95.74
ENSMUSG00000080321	Gm14644	pseudogene	1	69.24	1	95.211
1	88.601	1	93.264
ENSMUSG00000080326	Gm23425	miRNA	1	71.516	1	95.211

1 87.971 1 93.457
ENSMUSG00000080330 Gm22468 miRNA 1 82.352 1 93.654
NA NA 1 92.181
ENSMUSG00000080331 Mir1298 miRNA 1 81.823 1 93.654
1 83.811 1 96.183
ENSMUSG00000080336 Gm25111 miRNA 1 86.112 1 96.763
1 86.258 1 95.273
ENSMUSG00000080342 NA NA NA NA 1 94.102 1
88.076 1 94.19
ENSMUSG00000080345 Gm24586 miRNA 1 79.709 1 95.708
1 85.58 1 96.086
ENSMUSG00000080346 Gm24588 miRNA 1 74.8 1 94.597 1
87.786 1 90.125
ENSMUSG00000080347 Gm24587 miRNA 1 86.464 1 96.68 1
82.778 1 96.192
ENSMUSG00000080348 Gm24591 miRNA 1 80.251 1 90.389
1 86.985 1 95.559
ENSMUSG00000080349 Gm24590 miRNA 1 78.224 NA NA 1
86.666 1 96.071
ENSMUSG00000080350 Gm26248 miRNA 1 84.534 1 92.474
1 88.161 1 93.734
ENSMUSG00000080352 Gm26247 snoRNA 1 84.661 1
96.407 1 80.089 1 94.904
ENSMUSG00000080356 Mir1247 miRNA 1 82.352 1 72.399
1 70.328 1 91.492
ENSMUSG00000080358 Gm26249 snRNA 1 76.546 1 87.452
1 62.648 1 96.042
ENSMUSG00000080359 Gm26250 miRNA 1 76.396 1 93.765
1 85.792 1 96.183
ENSMUSG00000080360 Gm25779 miRNA 1 84.324 1 96.216
1 84.308 1 94.904
ENSMUSG00000080363 Gm25778 miRNA 1 86.172 1 96.847
1 86.144 1 96.071
ENSMUSG00000080364 Gm25777 snoRNA 1.005 79.577 0.929
88.345 1.068 64.024 1.084 81.56
ENSMUSG00000080365 Gm25776 snoRNA 1.138 66.58 0.942 73.766
0.934 76.225 0.769 86.991
ENSMUSG00000080366 Gm25775 miRNA 1 82.352 1 91.897
1 72.695 1 95.877
ENSMUSG00000080367 Gm25774 miRNA 1 81.925 1 93.654
1 86.144 1 93.156
ENSMUSG00000080368 Gm25773 miRNA 1 73.585 1 95.329
1 85.792 1 94.904
ENSMUSG00000080370 Gm22952 snoRNA 1.333 84.865 1.016
97.263 0.737 88.076 0.541 88.245
ENSMUSG00000080374 Gm22953 miRNA 0.901 49.621 0.931 95.088
1.194 65.235 0.911 85.493
ENSMUSG00000080378 Gm25309 snRNA 1 84.049 1 97.155
1 84.83 1 95.273
ENSMUSG00000080380 Gm22102 miRNA 1 86.658 NA NA 1
82.685 1 96.183
ENSMUSG00000080383 Gm22103 miRNA 1 79.433 1 95.088
1 81.492 1 91.807
ENSMUSG00000080384 Gm22105 snRNA 1 85.857 1 97.263

1 86.144 1 92.31
ENSMUSG00000080385 Gm22104 miRNA 1 82.231 1 87.452
1 79.255 1 94.904
ENSMUSG00000080390 Gm23784 miRNA 1 84.819 1 95.708
1 88.693 1 91.525
ENSMUSG00000080391 Gm23785 miRNA 1 83.169 1 93.654
1 78.498 1 92.018
ENSMUSG00000080394 Gm23786 snoRNA 1 77.497 1
96.216 1 83.925 1 95.273
ENSMUSG00000080401 Gm23653 miRNA 1 81.01 NA NA 1
87.274 1 94.608
ENSMUSG00000080402 Gm23652 miRNA 1 77.407 1 95.431
NA NA 1 94.904
ENSMUSG00000080405 Gm23651 miRNA 1 64.401 NA NA 1
84.961 1 96.248
ENSMUSG00000080409 Mir467h miRNA 1 86.819 NA NA 1
87.658 1 90.95
ENSMUSG00000080410 Gm26472 miRNA 1 68.353 1 95.708
1 87.898 1 92.675
ENSMUSG00000080411 Mir1193 miRNA 1 71.915 1 94.848
1 62.307 1 96.183
ENSMUSG00000080412 Gm26474 miRNA 1 85.355 1 90.454
1 85.757 1 95.593
ENSMUSG00000080413 Gm26473 snRNA 1 65.622 1 95.504
1 79.897 1 95.877
ENSMUSG00000080415 Gm26469 miRNA 1 84.534 NA NA 1
87.744 1 94.215
ENSMUSG00000080419 Gm26475 miRNA 1 82.231 1 95.504
1 86.144 1 95.676
ENSMUSG00000080424 NA NA NA NA 1 96.897 NA NA
1 96.287
ENSMUSG00000080425 Gm24833 snRNA 1 81.01 1 96.763 1
81.169 1 93.457
ENSMUSG00000080426 Gm24834 miRNA 1 80.815 1 95.708
1 64.024 1 96.183
ENSMUSG00000080427 Gm24835 miRNA 1 82.982 1 96.138
1 84.669 1 95.273
ENSMUSG00000080428 Gm24831 snRNA 1 78.299 1 96.407
1 86.602 1 94.752
ENSMUSG00000080430 Gm24275 miRNA 1 78.744 1 96.013
1 85.792 1 94.904
ENSMUSG00000080432 Gm23178 miRNA 1 82.352 1 92.914
1 86.144 1 90.397
ENSMUSG00000080434 Gm23177 miRNA 1 85.355 1 96.843
1 85.134 1 95.593
ENSMUSG00000080437 Gm24274 snRNA 1 66.897 NA NA 1
88.203 1 93.156
ENSMUSG00000080440 Gm25848 snoRNA 0.633 35.646 0.609
96.68 2.596 57.286 1.343 64.513
ENSMUSG00000080441 Mir1249 miRNA 1 85.355 1 92.631
1 87.744 1 95.35
ENSMUSG00000080443 Gm25847 miRNA 1 82.982 NA NA 1
88.729 1 95.273
ENSMUSG00000080445 Gm25850 miRNA 1 84.168 1 94.262

1 88.203 1 95.92
ENSMUSG00000080451 Gm24193 miRNA 1 86.819 NA NA NA
NA 1 92.018
ENSMUSG00000080452 Mir1306 miRNA 1 85.728 1 95.708
1 84.308 1 93.612
ENSMUSG00000080454 Gm24195 miRNA 1.133 32.873 0.764 85.116
1.158 87.519 0.995 94.19
ENSMUSG00000080455 Gm24194 miRNA 1 84.977 1 95.211
1 86.258 1 94.904
ENSMUSG00000080459 Mir669g miRNA 1 84.397 1 96.407
1 65.567 1 95.559
ENSMUSG00000080463 Gm22489 snoRNA 1 79.165 1
95.708 1 87.103 1 96.192
ENSMUSG00000080465 Gm22486 snoRNA 1.026 76.042 0.858
75.345 1.137 75.009 0.81 86.699
ENSMUSG00000080467 Gm22487 miRNA 1 85.655 1 96.335
1 84.308 1 95.74
ENSMUSG00000080468 Mir1195 miRNA 1 72 NA NA 1
80.015 1 95.593
ENSMUSG00000080469 Snord98 snoRNA 0.431 6.677 0.633 74.483
3.666 6.202 1.79 69.965
ENSMUSG00000080473 Gm25315 miRNA 1 85.728 1 95.708
1 84.724 1 96.287
ENSMUSG00000080474 Gm25317 miRNA 1 79.165 NA NA NA
NA 1 94.215
ENSMUSG00000080476 Gm25316 miRNA 1 73.401 1 95.301
1 83.719 1 95.832
ENSMUSG00000080478 Snord23 snoRNA 0.942 80.51 0.757 91.361
1.404 71.232 0.627 85.493
ENSMUSG00000080480 Gm23494 snRNA 1 78.224 1 91.532
1 77.17 1 95.273
ENSMUSG00000080485 Gm23493 miRNA 0.957 69.24 1.452 96.847
0.721 85.352 0.849 94.174
ENSMUSG00000080486 Snord100 snoRNA 1.339 71.516 1.319
93.654 0.565 81.525 1.233 92.381
ENSMUSG00000080487 Gm23492 miRNA 1 85.857 1 96.335
1 81.525 1 87.651
ENSMUSG00000080495 Gm26303 miRNA 1 69.943 1 96.763
1 85.792 1 95.832
ENSMUSG00000080498 Gm26302 miRNA 1 78.428 1 96.138
1 86.831 1 95.273
ENSMUSG00000080499 Gm25338 snRNA 1 58.264 1 96.216
1 84.724 1 94.904
ENSMUSG00000080500 Gm22072 miRNA 1 85.391 NA NA 1
81.857 1 96.183
ENSMUSG00000080504 Mir669d-2 miRNA 1 83.418 1 95.301
1 79.554 1 96.287
ENSMUSG00000080506 Gm25042 miRNA 1 82.231 1 94.367
1 84.896 1 93.132
ENSMUSG00000080507 Gm22071 miRNA 1 81.24 NA NA 1 80.5
1 91.269
ENSMUSG00000080508 Gm25041 miRNA 1 85.832 1 94.597
1 84.308 1 96.358
ENSMUSG00000080511 Gm22195 miRNA 1 76.827 1 96.216

1 87.971 1 94.82
ENSMUSG00000080515 Gm22196 miRNA 1 84.738 1 96.138
1 81.857 1 93.722
ENSMUSG00000080516 Gm22197 miRNA 1 81.925 1 95.941
1 85.757 1 92.561
ENSMUSG00000080517 Gm23109 snRNA 1 69.777 1 96.216
1 84.552 1 95.74
ENSMUSG00000080518 Gm22193 snoRNA 1 69.408 1
96.138 1 73.027 1 96.183
ENSMUSG00000080519 Gm22194 miRNA 1 80.408 NA NA NA
NA 1 95.74
ENSMUSG00000080522 Gm23881 miRNA 1 83.333 1 94.848
1 88.729 1 94.82
ENSMUSG00000080524 Gm23879 snRNA 1 85.832 1 96.268
1 83.857 1 95.72
ENSMUSG00000080526 Mir466j miRNA 1 85.857 1 94.597
NA NA 1 93.156
ENSMUSG00000080527 Gm23880 miRNA 0.799 68.955 1.103 93.654
1.131 72.378 0.668 86.991
ENSMUSG00000080529 Gm23882 miRNA 1 83.333 1 96.984
1 81.976 1 93.156
ENSMUSG00000080530 Gm25540 miRNA 1.287 81.196 1.311 96.68
0.591 73.027 1.118 92.389
ENSMUSG00000080533 Mir1191 miRNA 1.074 74.482 0.988 90.29
0.942 58.271 1.115 74.896
ENSMUSG00000080536 Gm25539 snoRNA 1 68.955 1
97.263 1 88.693 1 95.35
ENSMUSG00000080538 Gm25541 snRNA 1 85.655 NA NA 1
83.066 1 96.358
ENSMUSG00000080539 Gm25542 snRNA 1 78.744 1 95.504
1 78.713 1 94.567
ENSMUSG00000080540 Gm22711 snoRNA 0.874 69.985 NA NA
1.22 69.728 0.665 95.72
ENSMUSG00000080543 Gm22709 miRNA 1 81.647 1 96.843
1 82.31 1 90.463
ENSMUSG00000080545 Gm22713 snRNA 1 84.324 1 95.708
NA NA 1 94.654
ENSMUSG00000080549 NA NA NA NA 1 93.654 1
84.504 1 94.82
ENSMUSG00000080555 Gm24396 miRNA 1 86.819 1 96.013
1 87.37 1 94.608
ENSMUSG00000080556 Mir467g miRNA 1 86.006 NA NA 1
84.724 1 91.807
ENSMUSG00000080557 Gm24397 miRNA 1 83.823 NA NA 1
80.634 1 91.625
ENSMUSG00000080558 Gm22091 miRNA 1 84.865 1 96.897
1 82.161 1 96.192
ENSMUSG00000080561 Gm26042 miRNA 1 80.408 1 95.504
NA NA 1 96.358
ENSMUSG00000080562 Gm26043 miRNA 1 86.74 1 96.216 1
79.897 1 94.904
ENSMUSG00000080566 Gm26044 snRNA 1 85.946 1 96.216
1 79.554 1 96.071
ENSMUSG00000080574 Mir466i miRNA 1 81.01 1 82.947 1

57.286 1 94.608
ENSMUSG00000080575 Mir1190 miRNA 1 82.479 1 95.211
1 58.051 1 96.086
ENSMUSG00000080577 Gm23226 snRNA 1 80.146 NA NA 1
87.103 1 94.468
ENSMUSG00000080580 Mir466f-4 miRNA 1 77.407 NA NA 1
86.475 1 94.904
ENSMUSG00000080582 Gm24907 snoRNA 0.334 47.677 0.847
95.196 3.549 54.363 3.498 74.193
ENSMUSG00000080586 Mir1187 miRNA 1 85.499 NA NA 1
87.744 1 96.358
ENSMUSG00000080590 Gm22029 miRNA 1 84.564 NA NA 1
84.552 1 96.287
ENSMUSG00000080592 Gm22030 miRNA 1 85.857 NA NA 1
85.134 1 95.593
ENSMUSG00000080594 Mir669h miRNA 1 86.819 1 96.216
1 82.436 1 95.877
ENSMUSG00000080595 Gm22031 snRNA 1 61.043 1 96.138
1 76.498 1 96.071
ENSMUSG00000080596 Gm22032 miRNA 1 85.283 NA NA 1
87.744 1 91.625
ENSMUSG00000080597 Gm22033 miRNA 1 84.865 1 97.263
1 76.768 1 96.042
ENSMUSG00000080600 Gm23840 miRNA 1 77.763 1 95.504
1 83.925 1 96.071
ENSMUSG00000080601 Gm23841 miRNA 1 85.832 1 96.013
1 87.274 1 90.289
ENSMUSG00000080602 Gm23842 miRNA 1 84.397 1 97.097
1 81.032 1 95.74
ENSMUSG00000080607 Gm23839 snoRNA 1 76.135 1
93.654 NA NA 1 96.183
ENSMUSG00000080609 Gm23844 miRNA 1 85.59 1 96.138 1
83.538 1 94.825
ENSMUSG00000080613 Gm22151 miRNA 1 86.351 1 95.211
1 82.74 1 95.72
ENSMUSG00000080615 Snord99 snoRNA 1 73.401 1
95.301 1 82.685 1 96.248
ENSMUSG00000080616 Gm22152 snoRNA 1 85.59 1 95.35 1
86.258 1 96.086
ENSMUSG00000080622 Gm22676 snoRNA 1 81.647 1
96.216 NA NA 1 95.777
ENSMUSG00000080624 Gm22678 miRNA 1 74.689 1 95.301
NA NA 1 96.248
ENSMUSG00000080625 Gm22679 miRNA 1 77.113 1 96.843
1 80.381 1 96.358
ENSMUSG00000080626 Mir1192 miRNA 1.317 74.516 0.963 97.263
0.787 72.269 0.35 83.724
ENSMUSG00000080627 Gm22677 miRNA 1 76.042 1 97.097
1 66.269 1 96.248
ENSMUSG00000080631 Gm25507 miRNA 1 77.615 NA NA 1
87.971 1 95.832
ENSMUSG00000080632 Gm25508 miRNA 1 78.538 1 96.216
1 80.634 1 89.108
ENSMUSG00000080635 Gm25509 miRNA 1 76.686 1 96.897

1 78.045 1 92.181
ENSMUSG00000080636 Gm25510 miRNA 1 83.09 1 95.211 NA
NA 1 94.016
ENSMUSG00000080645 Mir1198 miRNA 1.965 22.205 0.391 37.426
1.305 55.109 1.057 91.335
ENSMUSG00000080649 Gm26156 miRNA 1 85.857 1 96.68 1
87.658 1 94.608
ENSMUSG00000080651 Gm24483 miRNA 1 79.577 1 93.654
1 83.538 1 95.559
ENSMUSG00000080653 Mir669e miRNA 1 72 1 95.504 1
84.896 1 95.74
ENSMUSG00000080655 Gm24482 miRNA 1 85.129 1 96.216
NA NA 1 92.018
ENSMUSG00000080657 Mir669i miRNA 1 81.178 1 92.474
1 86.552 1 91.979
ENSMUSG00000080661 Gm25003 miRNA 1 82.891 1 95.211
1 84.669 1 95.74
ENSMUSG00000080662 Mir1b miRNA 1 84.294 1 97.129 1
84.434 1 88.797
ENSMUSG00000080663 Gm25002 miRNA 1 84.049 1 97.155
1 87.173 1 92.762
ENSMUSG00000080666 Gm25000 miRNA 1 81.196 NA NA 1
70.534 1 96.287
ENSMUSG00000080668 Gm24999 miRNA 1 79.853 NA NA 1
88.657 1 93.685
ENSMUSG00000080669 Mir1224 miRNA 1 81.01 1 96.138 1
87.898 1 95.593
ENSMUSG00000080671 Gm23335 miRNA 1 74.021 1 97.155
1 84.504 1 95.35
ENSMUSG00000080673 Gm23336 snRNA 1 86.819 NA NA NA
NA 1 93.132
ENSMUSG00000080676 Gm25347 miRNA 1 84.738 1 95.504
1 78.498 1 91.492
ENSMUSG00000080678 Gm23334 miRNA 1 84.534 NA NA 1
82.778 1 93.964
ENSMUSG00000080680 Gm23989 miRNA 1 84.168 1 94.96 1
85.134 1 95.273
ENSMUSG00000080681 Gm23990 miRNA 1 83.968 NA NA 1
86.258 1 92.762
ENSMUSG00000080683 Gm23991 snoRNA 1 82.543 1
95.708 1 87.173 1 94.297
ENSMUSG00000080684 Gm23988 miRNA 1 86.333 1 92.137
1 82.575 1 96.358
ENSMUSG00000080686 Gm24806 miRNA 1 84.661 1 96.763
1 78.045 1 95.676
ENSMUSG00000080687 Mir1197 miRNA 1 51.737 NA NA NA
NA 1 96.183
ENSMUSG00000080689 Gm23987 snoRNA 1 84.865 1
93.654 1 88.693 1 95.74
ENSMUSG00000080694 Gm11621 pseudogene 1 81.823 1
95.504 1 86.985 1 95.676
ENSMUSG00000080695 Gm5394 pseudogene 1 85.637 1
94.367 1 85.464 1 91.807
ENSMUSG00000080696 Gm12825 pseudogene 1 86.172 1

92.474 1 87.744 1 94.016
ENSMUSG00000080697 Vmn1r-ps107 pseudogene 1 85.499 1
96.268 1 88.076 1 95.74
ENSMUSG00000080698 Gm11333 pseudogene 1 86.74 1 94.48 1
86.727 1 96.071
ENSMUSG00000080701 Gm12340 pseudogene 1 82.891 1
92.793 1 82.685 1 92.12
ENSMUSG00000080704 Gm6031 pseudogene 1 84.819 1
95.708 1 88.274 1 95.676
ENSMUSG00000080705 Gm15439 pseudogene 1 76.546 1
94.848 1 86.666 1 94.99
ENSMUSG00000080706 Gm12890 pseudogene 1 83.263 1
96.847 1 86.346 1 92.564
ENSMUSG00000080707 Gm13823 pseudogene 1 85.391 1
97.263 1 78.155 1 95.273
ENSMUSG00000080708 Ccnd3-ps pseudogene 1 84.977 1
94.597 1 76.225 1 87.088
ENSMUSG00000080709 Gm14324 pseudogene 1 86.464 1
96.001 1 87.173 1 94.19
ENSMUSG00000080710 Gm8107 pseudogene 1 81.01 1 97.263
1 84.724 1 96.287
ENSMUSG00000080711 Gm11686 pseudogene 1 81.178 1
92.137 1 85.935 1 94.904
ENSMUSG00000080713 Olfr1174-ps pseudogene 1 77.113 NA NA
1 88.274 1 96.192
ENSMUSG00000080714 Gm14602 pseudogene 1 80.146 1
95.088 1 84.896 1 92.31
ENSMUSG00000080715 Gm5406 pseudogene 1.826 86.351 NA NA
0.506 85.675 0.788 93.413
ENSMUSG00000080716 Gm13503 pseudogene 1 84.865 1
97.288 1 85.921 1 91.492
ENSMUSG00000080717 B230307C23Rik protein_coding 0.704 43.435
NA NA 1.87 41.331 1.512 73.823
ENSMUSG00000080718 Gm8019 pseudogene 1 81.647 1
95.196 1 86.666 1 96.183
ENSMUSG00000080719 Gm15532 pseudogene 1 69.408 1
93.908 1 85.58 1 96.358
ENSMUSG00000080720 Gm16077 pseudogene 1 54.44 1 96.763
1 86.985 1 95.877
ENSMUSG00000080721 Gm14944 pseudogene 1 84.865 NA NA
1 87.431 1 96.248
ENSMUSG00000080722 Defa-ps7 pseudogene 1 85.832 1
92.931 NA NA 1 95.273
ENSMUSG00000080723 Gm12564 pseudogene 1 86.464 1
96.652 1 87.519 1 96.287
ENSMUSG00000080725 NA NA NA NA 1 95.088 1
79.119 1 96.071
ENSMUSG00000080726 Gm15366 pseudogene 1 80.146 NA NA
1 73.428 1 92.018
ENSMUSG00000080727 C920021L13Rik protein_coding 1.005 82.982
0.831 61.883 1.192 30.292 1.073 92.018
ENSMUSG00000080728 Gm13616 pseudogene 1 77.497 1
96.68 1 76.225 1 93.457
ENSMUSG00000080729 Gm8084 pseudogene 1 86.658 1

90.053	1	84.434	1	87.134
ENSMUSG00000080730	Gm11313	pseudogene	1	78.224	1
95.211	1	84.229	1	95.35
ENSMUSG00000080731	Rps2-ps9	pseudogene	1	39.174	1
96.843	1	46.575	1	96.287
ENSMUSG00000080732	Gm13222	pseudogene	1	82.092	1
95.941	1	85.464	1	95.35
ENSMUSG00000080733	Gm14867	pseudogene	1	86.918	1
97.155	1	87.103	1	96.358
ENSMUSG00000080734	Gm12006	pseudogene	1	86.172	1
92.561	1	86.552	1	94.001
ENSMUSG00000080735	NA	NA	NA	NA	NA	NA	1	85.757
1	95.74
ENSMUSG00000080736	Gm15617	pseudogene	1	82.543	1
93.994	1	72.148	1	92.181
ENSMUSG00000080737	Gm6982	pseudogene	1	83.726	NA	NA
1	84.229	1	95.273
ENSMUSG00000080738	Mup-ps22	pseudogene	1	71.674	1
95.708	1	87.519	1	95.273
ENSMUSG00000080739	Gm13795	pseudogene	1	85.77	1	96.407
1	82.436	1	94.904
ENSMUSG00000080741	Gm11398	pseudogene	1	83.823	NA	NA
1	71.232	1	96.358
ENSMUSG00000080742	Gm14035	pseudogene	1	82.231	1
96.652	1	87.971	1	95.92
ENSMUSG00000080743	Gm7150	pseudogene	1	84.977	1
96.843	1	84.896	NA	NA
ENSMUSG00000080744	Gm12710	pseudogene	1	74.8	1	96.763
NA	NA	1	91.372
ENSMUSG00000080748	Gm15661	pseudogene	1	79.068	1
96.847	1	87.744	1	95.832
ENSMUSG00000080749	Gm12134	pseudogene	1	84.049	1
96.138	1	86.112	1	96.358
ENSMUSG00000080750	Gm15553	pseudogene	1	82.092	1
94.597	1	88.161	1	96.287
ENSMUSG00000080751	Olfr634-ps1	pseudogene	1	79.853	1
88.705	1	85.464	1	92.466
ENSMUSG00000080752	Olfr1069-ps1	pseudogene	1	80.51	1
96.652	1	85.792	1	94.99
ENSMUSG00000080753	Gm15613	pseudogene	1	82.352	1
90.287	1	86.144	1	93.457
ENSMUSG00000080754	Rpl36-ps7	pseudogene	1	81.178	1
95.708	1	77.532	1	95.877
ENSMUSG00000080755	Gm15062	pseudogene	1	70.862	NA	NA
1	84.961	1	95.74
ENSMUSG00000080756	Gm12633	pseudogene	1	86.333	1
95.431	1	83.066	1	95.273
ENSMUSG00000080757	Gm15029	pseudogene	1	85.77	1	96.216
1	84.961	1	94.904
ENSMUSG00000080758	Gm5392	pseudogene	1	68.955	1
95.708	1	86.258	1	93.964
ENSMUSG00000080759	Gm15573	pseudogene	1	47.174	NA	NA
1	52.087	1	85.493
ENSMUSG00000080760	Gm7571	pseudogene	1	86.819	1

91.532 1 81.976 1 95.405
ENSMUSG00000080762 Gm11879 pseudogene 1 66.76 1 95.504
1 82.74 1 92.762
ENSMUSG00000080763 Gm12341 pseudogene 1 80.608 1
94.848 1 86.112 1 94.904
ENSMUSG00000080764 Gm14606 pseudogene 1 85.092 1
95.504 1 88.41 1 95.832
ENSMUSG00000080765 Gm8908 pseudogene 1 86.474 1
95.329 1 85.134 1 94.904
ENSMUSG00000080766 Gm13593 pseudogene 1 83.968 1
95.708 1 87.898 1 95.273
ENSMUSG00000080767 Gm15595 pseudogene 1 72.139 1
95.088 1 85.21 1 96.184
ENSMUSG00000080770 Gm11278 pseudogene 1 80.608 1
96.68 1 83.158 1 93.261
ENSMUSG00000080771 Gm11925 pseudogene 1 81.178 1
97.263 1 85.792 1 94.904
ENSMUSG00000080773 Gm12955 pseudogene 1.756 48.462 0.685
84.594 0.831 86.258 0.74 92.181
ENSMUSG00000080774 Gm9143 pseudogene 1 83.169 1
96.216 1 88.076 1 93.264
ENSMUSG00000080775 Gm6368 pseudogene 1 84.397 1
96.138 1 88.161 1 93.457
ENSMUSG00000080777 Gm15365 pseudogene 1 83.263 1
95.504 1 83.607 1 94.19
ENSMUSG00000080778 Gm7199 pseudogene 1 82.705 1
97.129 1 80.899 NA NA
ENSMUSG00000080779 Gm8731 pseudogene 1 77.615 1
92.137 1 63.301 1 91.807
ENSMUSG00000080780 Gm11252 pseudogene 1 86.57 1 93.908
1 84.961 1 96.184
ENSMUSG00000080781 Olfr388-ps1 pseudogene 1 79.93 NA NA 1
88.349 1 92.018
ENSMUSG00000080784 Gm13765 pseudogene 1 76.686 1
96.897 1 84.071 1 91.011
ENSMUSG00000080785 Gm11591 pseudogene 1 82.092 1
93.765 1 83.607 1 91.492
ENSMUSG00000080788 Gm7841 pseudogene 1 77.497 1
96.001 NA NA 1 95.832
ENSMUSG00000080789 Gm14007 pseudogene 1 72.985 1
97.155 1 83.925 1 94.825
ENSMUSG00000080790 Gm7638 pseudogene 0.72 69.985 1.027
91.275 1.352 44.515 0.65 83.724
ENSMUSG00000080791 Gm11442 pseudogene 1 77.615 1
96.335 1 71.004 1 95.273
ENSMUSG00000080792 Gm14367 pseudogene 1 86.57 NA NA NA
NA 1 92.12
ENSMUSG00000080793 Cbx3-ps3 pseudogene 1 71.36 1 93.27 1
72.853 1 95.74
ENSMUSG00000080794 Vmn1r-ps132 pseudogene 1 79.93 1 93.654
1 87.686 1 94.654
ENSMUSG00000080795 Gm6783 pseudogene 1 72.743 1
97.049 1 83.158 1 96.248
ENSMUSG00000080797 Gm15760 pseudogene 0.63 62.437 0.861

88.881 1.841 28.74 1.796 82.66
ENSMUSG00000080798 Gm5135 pseudogene 1 75.196 NA NA
1 73.874 1 96.183
ENSMUSG00000080799 Gm15166 pseudogene 1 81.925 1
93.654 1 84.071 1 93.261
ENSMUSG00000080800 Gm11582 pseudogene 1 66 1 87.857
1 73.759 1 95.74
ENSMUSG00000080801 Gm13310 pseudogene 1 72.743 1
96.216 1 84.504 1 94.825
ENSMUSG00000080802 Gm13804 pseudogene 1 82.352 NA NA
NA NA 1 95.273
ENSMUSG00000080803 Gm12683 pseudogene 1 80.146 1
94.597 1 87.686 1 95.273
ENSMUSG00000080805 Gm11219 pseudogene 1 80.608 1
95.088 1 84.724 1 91.372
ENSMUSG00000080807 Gm13117 pseudogene 1 81.178 1
95.708 1 84.961 1 95.72
ENSMUSG00000080808 Gm12608 pseudogene 1 86.474 NA NA
1 88.076 1 95.593
ENSMUSG00000080809 Olfr1273-ps pseudogene 1 85.637 NA NA
1 84.552 1 96.086
ENSMUSG00000080811 Gm14513 pseudogene 1 84.168 1
95.301 1 86.258 1 96.086
ENSMUSG00000080812 NA NA NA NA 1 96.68 1 88.274
1 96.183
ENSMUSG00000080813 Gm14828 pseudogene 1 63.534 1
93.469 1 88.161 1 95.676
ENSMUSG00000080814 Gm12314 pseudogene 1 72.139 1
96.68 1 83.275 1 96.183
ENSMUSG00000080815 Hmgb1-rs18 pseudogene 1 77.113 1
94.597 1 82.436 1 94.904
ENSMUSG00000080816 Gm12447 pseudogene 1 66.897 1
95.431 1 66.425 1 94.153
ENSMUSG00000080817 Gm13751 pseudogene 1 86.145 1
91.83 1 76.498 1 81.901
ENSMUSG00000080818 Gm9434 pseudogene 1 76.968 1
92.793 1 86.475 1 95.92
ENSMUSG00000080819 Gm2005 pseudogene 1 66.652 1
88.881 1 88.601 1 94.654
ENSMUSG00000080820 Gm13197 pseudogene 1 83.169 1
96.897 1 84.724 1 94.608
ENSMUSG00000080822 Gm14694 pseudogene 1 74.8 1 95.708
1 83.158 1 94.016
ENSMUSG00000080823 Gm11477 pseudogene 1 73.401 1
91.661 1 88.203 1 94.99
ENSMUSG00000080824 Gm9001 pseudogene 1 86.464 1
96.216 1 73.027 1 95.676
ENSMUSG00000080825 Gm14892 pseudogene 1 83.823 1
97.155 1 87.658 1 93.722
ENSMUSG00000080826 Gm12019 pseudogene 1 83.418 NA NA
1 86.258 1 96.183
ENSMUSG00000080827 Gm12086 pseudogene 1 85.655 1
95.088 NA NA 1 96.184
ENSMUSG00000080828 Gm14669 pseudogene 1 72.985 1

96.138 1 84.229 1 96.184
ENSMUSG00000080829 Gm8545 pseudogene 1 84.564 1
95.504 1 86.144 1 95.676
ENSMUSG00000080831 Gm12529 pseudogene 1 84.397 1
95.211 1 73.309 1 83.067
ENSMUSG00000080832 M6pr-ps pseudogene 1 84.564 NA NA
1 67.029 1 88.955
ENSMUSG00000080833 Gm11976 pseudogene 1 82.092 1
92.137 1 87.744 1 95.273
ENSMUSG00000080834 Gm15216 pseudogene 1 82.352 NA NA
1 80.899 1 96.358
ENSMUSG00000080835 Gm12137 pseudogene 1 85.728 1
95.504 1 87.173 1 94.153
ENSMUSG00000080836 Gm6444 pseudogene 1.574 28.927 2.89
78.494 0.22 6.751 1.093 91.492
ENSMUSG00000080839 NA NA NA NA 1 91.361 1
84.626 1 93.685
ENSMUSG00000080840 Gm11349 pseudogene 1 71.142 1
97.097 1 86.831 1 94.297
ENSMUSG00000080841 Gm14880 pseudogene 1 86.819 1
97.155 1 84.896 1 96.358
ENSMUSG00000080842 Gm15400 pseudogene 1 82.705 1
95.301 1 82.74 1 95.273
ENSMUSG00000080843 Gm14726 pseudogene 1 86.199 1
88.881 1 78.713 1 95.273
ENSMUSG00000080844 Gm14107 pseudogene 1 85.655 1
96.407 1 82.903 1 94.016
ENSMUSG00000080845 Gm9115 pseudogene 1 72.743 1
97.263 1 88.203 1 94.904
ENSMUSG00000080846 Gm13379 pseudogene 1 83.523 1
96.847 1 84.308 1 93.627
ENSMUSG00000080847 Gm4773 pseudogene 1 80.03 1 96.013
1 86.666 1 96.086
ENSMUSG00000080849 Gm15702 pseudogene 1 84.397 1
97.097 1 87.519 1 92.561
ENSMUSG00000080850 Gm12439 pseudogene 1.602 27.545 0.652
81.731 0.958 74.179 0.965 96.248
ENSMUSG00000080851 Gm12094 pseudogene 1 71.36 1 97.155
1 80.089 1 93.584
ENSMUSG00000080852 Gm14918 pseudogene 1 84.263 1
96.68 1 86.475 1 95.877
ENSMUSG00000080855 Rpl35-ps1 pseudogene 1 86.172 1
94.767 1 85.757 NA NA
ENSMUSG00000080857 Gm12859 pseudogene 1 83.823 1
96.897 1 76.498 1 93.261
ENSMUSG00000080859 Rpl10-ps1 pseudogene 1 82.231 1
95.708 1 81.29 1 93.156
ENSMUSG00000080860 Gm13815 pseudogene 1 85.203 1
90.053 1 68.643 1 94.654
ENSMUSG00000080861 Gm11325 pseudogene 1 85.832 1
94.597 1 87.898 1 94.99
ENSMUSG00000080863 Gm14787 pseudogene 1 86.57 1 96.68 1
88.274 1 96.287
ENSMUSG00000080865 Gm13637 pseudogene 1 79.433 1

96.268	1	82.685	1	94.153
ENSMUSG00000080867	Gm14079	pseudogene	1	69.408	NA	NA
1	88.693	1	96.183
ENSMUSG00000080868	Gm15745	pseudogene	1	81.01	1	97.049
1	76.388	1	94.904
ENSMUSG00000080869	Gm11856	pseudogene	1	81.52	1	96.013
1	86.475	1	95.593
ENSMUSG00000080870	Gm14111	pseudogene	1	86.145	1
96.268	NA	NA	1	92.31
ENSMUSG00000080871	Gm12875	pseudogene	1	85.499	NA	NA
1	86.144	1	94.297
ENSMUSG00000080872	Gm11506	pseudogene	1	86.145	1
96.138	1	88.657	1	92.181
ENSMUSG00000080873	Rpl30-ps3	pseudogene	1	64.014	1
97.263	1	87.898	1	95.777
ENSMUSG00000080874	Gm15010	pseudogene	1	78.224	NA	NA
1	87.786	1	95.877
ENSMUSG00000080875	Gm7332	pseudogene	1	86.843	1
96.843	1	84.552	1	92.389
ENSMUSG00000080876	Gm14332	pseudogene	1	85.946	1
97.155	1	85.921	1	92.742
ENSMUSG00000080877	Rpl22-ps1	pseudogene	1	80.03	1	93.654
1	88.657	1	96.248
ENSMUSG00000080878	Gm15618	pseudogene	1	78.299	1
95.708	1	86.475	1	95.559
ENSMUSG00000080880	Gm14278	pseudogene	1	71.674	1
93.765	1	66.928	1	83.067
ENSMUSG00000080884	Gm12674	pseudogene	1	86.172	1
96.847	1	76.923	1	93.964
ENSMUSG00000080886	Gm14847	pseudogene	1	81.52	NA	NA	1
67.029	1	95.35
ENSMUSG00000080887	Gm14831	pseudogene	1	81.125	1
94.102	1	87.658	1	96.086
ENSMUSG00000080889	Gm7974	pseudogene	1	84.977	1
96.843	1	81.29	1	94.567
ENSMUSG00000080890	Gm16458	pseudogene	1	86.006	1
96.847	1	82.778	1	95.72
ENSMUSG00000080891	Olfr587-ps1	pseudogene	1	86.474	1
96.68	1	85.21	1	92.748
ENSMUSG00000080892	Gm9266	pseudogene	1	73.841	1
91.661	1	81.032	1	90.628
ENSMUSG00000080894	Defa-ps15	pseudogene	1	86.464	1
96.001	1	84.896	1	92.181
ENSMUSG00000080895	Gm11645	pseudogene	1	82.705	1
94.848	1	87.103	1	94.384
ENSMUSG00000080896	Gm14567	pseudogene	1	79.853	NA	NA
1	87.971	1	94.82
ENSMUSG00000080897	Gm14500	pseudogene	1	82.732	1
96.948	1	87.971	1	96.287
ENSMUSG00000080898	Gm14836	pseudogene	1	85.728	1
96.013	1	86.112	1	92.181
ENSMUSG00000080899	Gm9109	pseudogene	1	83.418	1
97.263	1	75.809	1	95.92
ENSMUSG00000080902	Gm11226	pseudogene	1	80.815	1

92.914	1	71.839	1	94.654
ENSMUSG00000080903	Gm16383	pseudogene	1	84.397	1
95.708	1	86.552	1	95.273
ENSMUSG00000080905	Gm13295	pseudogene	1	85.946	NA	NA
1	88.693	1	93.457
ENSMUSG00000080906	Gm12095	pseudogene	1	81.24	1	85.671
NA	NA	1	90.854
ENSMUSG00000080907	4930455H04Rik	protein_coding	0.708	20.369
1.15	95.088	1.225	62.237	0.814	85.493
ENSMUSG00000080909	Gm13044	pseudogene	1	81.01	1	93.654
1	87.686	1	94.825
ENSMUSG00000080910	Gm14821	pseudogene	1	86.819	1
96.138	NA	NA	1	94.654
ENSMUSG00000080911	Gm11918	pseudogene	1	86.145	1
95.809	1	85.921	1	96.192
ENSMUSG00000080912	Gm12269	pseudogene	1	85.655	NA	NA
1	77.888	1	96.248
ENSMUSG00000080913	Gm12719	pseudogene	1	85.655	1
96.407	1	85.792	1	95.676
ENSMUSG00000080914	Gm7129	pseudogene	1	80.251	1
90.68	1	67.339	1	96.139
ENSMUSG00000080915	Gm6446	pseudogene	1	73.585	NA	NA
1	88.601	1	92.381
ENSMUSG00000080916	Gm12463	pseudogene	2.175	85.946	0.419
95.211	1.095	63.801	0.821	94.904
ENSMUSG00000080917	Gm15925	pseudogene	1	71.217	1
82.947	1	73.759	1	94.99
ENSMUSG00000080918	Gm12202	pseudogene	1	86.918	1
95.708	1	81.976	1	93.132
ENSMUSG00000080919	Gm11854	pseudogene	1	83.333	1
95.329	1	85.21	1	94.016
ENSMUSG00000080922	Gm2214	pseudogene	1	81.196	1
96.268	1	84.896	1	95.877
ENSMUSG00000080923	Gm5400	pseudogene	1	84.865	NA	NA
1	88.601	1	95.273
ENSMUSG00000080924	Olfr1078-ps1	pseudogene	1	75.671	1
96.897	1	87.519	1	95.273
ENSMUSG00000080925	Gm13682	pseudogene	1	74.147	1
91.83	1	82.161	1	94.608
ENSMUSG00000080927	Gm11430	pseudogene	1	84.977	NA	NA
1	83.925	1	95.877
ENSMUSG00000080929	Gm12891	pseudogene	1	84.397	1
92.137	1	66.269	1	95.273
ENSMUSG00000080930	Gm13367	pseudogene	1	82.092	1
96.335	1	82.161	1	96.183
ENSMUSG00000080933	Rhox3g	protein_coding	1	86.74	1
95.708	1	84.071	1	94.016
ENSMUSG00000080935	Got2-ps1	pseudogene	1	84.865	1
94.597	1	74.686	1	96.183
ENSMUSG00000080936	Olfr337-ps1	pseudogene	1	84.294	1
95.708	1	88.601	1	96.192
ENSMUSG00000080937	Gm13510	pseudogene	1	43.435	NA	NA
1	76.754	1	94.567
ENSMUSG00000080938	Gm6062	pseudogene	1	78.744	1

96.138 1 84.669 1 95.777
ENSMUSG00000080939 Gm9208 pseudogene 1 84.324 1
94.887 1 87.37 1 95.74
ENSMUSG00000080940 Gm15145 pseudogene 1 85.637 1
97.155 1 87.898 1 96.086
ENSMUSG00000080941 Gm16056 pseudogene 1 81.01 1 95.708
1 79.625 1 95.273
ENSMUSG00000080942 Gm14556 pseudogene 1.03 84.564 1.407
97.263 0.691 68.584 1.281 86.209
ENSMUSG00000080943 Gm13130 pseudogene 1 83.263 NA NA
1 87.519 1 94.654
ENSMUSG00000080944 Gm12967 pseudogene 1 83.726 1
95.941 NA NA 1 93.964
ENSMUSG00000080947 Gm12779 pseudogene 1 84.324 NA NA
1 84.229 1 93.156
ENSMUSG00000080948 Gm14481 pseudogene 1 84.977 1
95.088 1 76.768 1 95.273
ENSMUSG00000080949 Gm13683 pseudogene 1 72.85 1 96.216
1 72.378 1 93.156
ENSMUSG00000080951 Gm12208 pseudogene 1.136 66.944 0.792
70.864 1.109 65.567 1.086 91.525
ENSMUSG00000080952 Gm5379 pseudogene 1 71.896 1
96.013 1 88.461 1 96.086
ENSMUSG00000080953 Gm15025 pseudogene 1 84.819 NA NA
1 87.519 1 96.358
ENSMUSG00000080954 Gm14919 pseudogene 1 79.93 1 95.504
1 86.552 1 91.335
ENSMUSG00000080955 Gm14263 pseudogene 1 84.294 1
97.155 1 88.349 1 96.042
ENSMUSG00000080956 Gm16179 pseudogene 1 84.819 1
95.504 1 71.615 1 94.904
ENSMUSG00000080958 Gm11902 pseudogene 1 75.399 1
97.155 NA NA 1 94.016
ENSMUSG00000080959 Gm2003 pseudogene 1 84.564 1
94.887 1 84.626 1 96.287
ENSMUSG00000080960 Olfr343-ps1 pseudogene 1 81.52 1 96.407
NA NA 1 95.74
ENSMUSG00000080961 Gm11798 pseudogene 1 78.054 NA NA
1 80.5 1 94.567
ENSMUSG00000080962 Gm11453 pseudogene 1 83.726 1
96.68 1 85.935 1 96.248
ENSMUSG00000080964 Ifna-ps1 pseudogene 1 77.497 1
96.138 1 74.179 1 95.832
ENSMUSG00000080965 Gm6461 pseudogene 1 81.76 1 96.68 1
84.961 1 95.559
ENSMUSG00000080966 Gm6263 pseudogene 1 85.129 1
96.216 1 71.839 1 92.851
ENSMUSG00000080967 Gm12706 pseudogene 1 77.497 1
91.361 1 88.41 1 94.904
ENSMUSG00000080968 Gm1862 pseudogene 1 84.324 1
97.263 1 85.58 1 95.676
ENSMUSG00000080969 Gm12372 pseudogene 1 81.24 1 96.897
1 87.686 1 96.358
ENSMUSG00000080970 Gm12173 pseudogene 1 65.199 1

86.277 1 88.729 1 95.676
ENSMUSG00000080974 Gm9009 pseudogene 1 85.391 NA NA
1 87.274 1 96.358
ENSMUSG00000080976 Gm11490 pseudogene 1 83.523 1
95.708 1 88.601 1 95.832
ENSMUSG00000080977 Gm13772 pseudogene 1 75.567 1
95.809 1 87.898 1 95.74
ENSMUSG00000080978 Gm12297 pseudogene 1 67.374 1
97.155 1 88.693 1 95.559
ENSMUSG00000080979 Gm13675 pseudogene 1 82.231 1
96.216 1 87.786 1 91.625
ENSMUSG00000080980 1700071K01Rik pseudogene 1 85.499 1
96.948 1 88.729 1 96.358
ENSMUSG00000080981 Gm12161 pseudogene 1 84.168 1
96.138 1 86.666 1 91.011
ENSMUSG00000080982 Gm13549 pseudogene 1 77.182 1
97.049 NA NA 1 94.99
ENSMUSG00000080983 Gm5419 pseudogene 1 83.726 1
96.897 1 88.349 1 92.762
ENSMUSG00000080984 Gm14469 pseudogene 1 85.857 1
93.765 1 84.896 1 90.691
ENSMUSG00000080985 Gm13559 pseudogene 1 86.843 1
96.138 1 86.144 1 95.92
ENSMUSG00000080986 Olfr1081-ps1 pseudogene 1 74.8 1
94.848 1 84.626 1 95.273
ENSMUSG00000080987 Gm12073 pseudogene 1 82.231 1
95.708 1 84.504 1 90.674
ENSMUSG00000080990 Olfr118 protein_coding 1 79.709 1
95.504 1 86.831 1 93.316
ENSMUSG00000080992 Psmb5-ps pseudogene 1 81.647 NA NA
1 88.729 1 95.35
ENSMUSG00000080993 Gm12903 pseudogene 0.543 44.467 0.981
97.097 1.875 40.511 1.03 94.016
ENSMUSG00000080995 Gm15165 pseudogene 1 84.819 1
95.708 1 83.538 1 94.654
ENSMUSG00000080996 Gm4986 pseudogene 1 86.74 1 96.763
1 85.792 1 93.734
ENSMUSG00000080997 Gm11885 pseudogene 1 81.465 1
96.843 1 80.015 1 90.611
ENSMUSG00000080998 Gm5384 pseudogene 1 79.577 1
97.155 1 74.082 1 95.74
ENSMUSG00000081000 Gm11200 pseudogene 1 85.728 1
97.155 1 87.744 1 93.316
ENSMUSG00000081001 Gm14678 pseudogene 1 78.428 1
94.96 1 82.161 1 96.287
ENSMUSG00000081002 Gm14240 pseudogene 1 83.726 1
95.504 1 88.076 1 93.316
ENSMUSG00000081003 Gm14301 pseudogene 1 84.564 NA NA
1 83.607 1 95.72
ENSMUSG00000081004 Gm9157 pseudogene 1.509 85.355 0.852
94.48 0.776 86.144 0.92 91.492
ENSMUSG00000081006 Gm12832 pseudogene 1 76.827 1
96.001 1 68.266 1 89.892
ENSMUSG00000081007 Gm11543 pseudogene 1 72.546 1

95.504 1 84.669 1 94.904
ENSMUSG00000081008 Gm13314 pseudogene 1 82.891 1
96.407 1 84.961 1 92.31
ENSMUSG00000081009 Gm14065 pseudogene 1 82.543 1
94.48 1 88.076 1 93.264
ENSMUSG00000081010 Gm13880 pseudogene 1 74.516 1
92.914 NA NA 1 95.832
ENSMUSG00000081011 Gm11913 pseudogene 1 84.397 1
91.361 1 84.626 1 96.358
ENSMUSG00000081012 Gm15224 pseudogene 1 69.777 1
97.155 1 83.811 1 96.184
ENSMUSG00000081013 Gm14497 pseudogene 1 77.182 NA NA
NA NA 1 96.287
ENSMUSG00000081014 Gm13714 pseudogene 1 80.815 1
95.088 1 87.103 1 93.722
ENSMUSG00000081015 Gm13805 pseudogene 1 86.333 1
96.847 1 84.071 1 96.287
ENSMUSG00000081016 Olfr1397-ps1 pseudogene 1 83.726 NA
NA 1 73.428 1 96.287
ENSMUSG00000081017 Gm15000 pseudogene 1 86.351 NA NA
1 87.37 1 95.273
ENSMUSG00000081018 Gm15012 pseudogene 1 71.142 NA NA
1 87.744 1 96.183
ENSMUSG00000081020 Gm14509 pseudogene 1 83.418 1
97.155 1 88.274 1 95.92
ENSMUSG00000081021 Gm11964 pseudogene 1 74.689 1
97.155 1 84.669 1 96.358
ENSMUSG00000081022 Gm15936 pseudogene 1 84.534 NA NA
NA NA 1 93.132
ENSMUSG00000081023 Gm15766 pseudogene 1 84.324 1
96.308 1 87.898 1 94.567
ENSMUSG00000081024 Gm14627 pseudogene 1 84.564 1
96.268 1 82.685 1 96.287
ENSMUSG00000081025 Gm11943 pseudogene 1 66.76 1 95.809
1 74.179 1 92.181
ENSMUSG00000081026 NA NA NA NA 1 97.155 1
84.896 1 95.273
ENSMUSG00000081027 Olfr1077-ps1 pseudogene 1 73.401 1
97.263 1 86.144 1 95.72
ENSMUSG00000081029 Gm15077 pseudogene 1 69.777 1
95.504 1 67.339 1 95.273
ENSMUSG00000081030 Gm12704 pseudogene 1 86.006 1
90.053 1 77.17 1 94.99
ENSMUSG00000081033 Gm14797 pseudogene 1 78.909 NA NA
1 84.724 1 96.358
ENSMUSG00000081034 Gm5944 pseudogene 1 79.93 1 95.431
NA NA 1 95.273
ENSMUSG00000081035 Olfr1300-ps1 pseudogene 1 82.982 1
97.049 1 83.275 1 93.627
ENSMUSG00000081036 Gm14628 pseudogene 1 86.918 1
91.661 1 81.976 1 93.457
ENSMUSG00000081037 Gm13929 pseudogene 1 86.199 1
96.897 1 68.266 1 96.192
ENSMUSG00000081038 Olfr626-ps1 pseudogene 1 86.474 NA NA

1 80.899 1 94.904
ENSMUSG00000081041 Nlk-ps1 pseudogene 1 82.732 1
93.899 1 83.857 1 94.904
ENSMUSG00000081043 Gm11512 pseudogene 1 80.608 1
97.288 1 82.778 1 96.086
ENSMUSG00000081044 AU015836 protein_coding 1 80.408 NA
NA 1 86.985 1 95.777
ENSMUSG00000081045 Gm12797 pseudogene 1 85.832 1
96.897 1 83.158 1 95.273
ENSMUSG00000081047 Olfr997-ps1 pseudogene 1 78.224 1
97.155 1 77.294 1 95.676
ENSMUSG00000081048 Gm11760 pseudogene 1 80.608 1
96.216 1 85.921 1 92.748
ENSMUSG00000081050 Gm12461 pseudogene 1 74.299 1
92.062 1 88.693 1 96.358
ENSMUSG00000081051 Gm15427 pseudogene 15.756 0 0.984
95.301 0.065 0.72 1.065 94.752
ENSMUSG00000081052 Gm6305 pseudogene 1.23 85.59 0.419 88.691
1.932 78.155 0.889 95.35
ENSMUSG00000081053 Mup-ps17 pseudogene 1 83.968 1
97.288 1 81.976 1 95.593
ENSMUSG00000081054 Gm12025 pseudogene 1 83.658 1
96.68 1 83.158 1 93.964
ENSMUSG00000081055 Gm6987 pseudogene 1 85.637 1
94.597 1 88.729 1 94.904
ENSMUSG00000081056 Gm13998 pseudogene 1 85.129 NA NA
1 81.492 1 96.192
ENSMUSG00000081057 Olfr1017-ps1 pseudogene 1 84.661 1
96.216 1 87.103 1 94.153
ENSMUSG00000081058 Hist2h3c2 protein_coding 0.779 33.002
1.241 82.904 1.031 85.935 1.041 96.183
ENSMUSG00000081059 Gm11945 pseudogene 1 66.406 1
87.037 1 88.729 1 94.82
ENSMUSG00000081060 Gm11318 pseudogene 1 81.823 1
94.48 1 86.144 1 96.192
ENSMUSG00000081061 Gm14712 pseudogene 1 76.135 1
93.654 1 84.724 1 91.525
ENSMUSG00000081062 Gm13640 pseudogene 1 76.319 1
95.708 1 83.275 1 96.287
ENSMUSG00000081063 Gm11825 pseudogene 1 83.169 1
97.263 1 84.552 1 94.904
ENSMUSG00000081064 Gm6071 pseudogene 1 80.815 1
97.263 1 83.925 1 93.734
ENSMUSG00000081065 Gm13576 pseudogene 1 85.499 1
95.708 1 80.089 1 95.273
ENSMUSG00000081066 Gm12817 pseudogene 1 81.52 NA NA 1
78.642 1 94.567
ENSMUSG00000081067 Gm12912 pseudogene 1 85.355 1
96.378 1 85.935 1 91.872
ENSMUSG00000081068 Gm12804 pseudogene 1 77.497 1
96.407 1 81.976 1 93.264
ENSMUSG00000081069 Gm13928 pseudogene 1 81.657 1
96.268 1 86.346 1 94.752
ENSMUSG00000081072 Gm14977 pseudogene 1 81.178 1

95.211 1 73.428 1 96.248
ENSMUSG00000081073 Gm13758 pseudogene 1 77.291 1
93.529 1 86.831 1 95.74
ENSMUSG00000081076 Rpsa-ps4 pseudogene 1 86.333 1
96.216 1 86.666 1 96.192
ENSMUSG00000081077 Gm13899 pseudogene 1 82.705 1
94.597 1 86.552 1 96.183
ENSMUSG00000081078 Gm12568 pseudogene 1 85.499 1
84.594 1 43.842 1 96.042
ENSMUSG00000081079 Gm13059 pseudogene 1 81.657 1
96.407 1 85.792 1 95.273
ENSMUSG00000081080 Vmn1r-ps102 pseudogene 1 86.57 1 95.088
1 78.498 1 89.72
ENSMUSG00000081081 Gm14856 pseudogene 1 67.608 1
96.216 1 88.729 1 93.264
ENSMUSG00000081082 Gm14937 pseudogene 1 85.391 NA NA
1 88.274 1 95.676
ENSMUSG00000081083 Gm6079 pseudogene 1 86.006 1
97.263 1 88.41 1 93.734
ENSMUSG00000081084 Gm5638 pseudogene 1 75.567 1
96.138 1 86.666 1 92.564
ENSMUSG00000081085 Gm13463 pseudogene 1 80.408 NA NA
1 86.552 1 96.184
ENSMUSG00000081086 Gm9506 pseudogene 1 82.732 1
92.561 1 56.822 1 96.086
ENSMUSG00000081088 Gm11679 pseudogene 1 84.865 1
93.908 1 84.308 1 95.74
ENSMUSG00000081089 Gm14624 pseudogene 1 72.139 1
96.897 1 84.83 1 96.358
ENSMUSG00000081092 Gm14128 pseudogene 1 83.418 NA NA
1 87.274 1 92.52
ENSMUSG00000081093 Gm12565 pseudogene 1 80.408 1
96.216 1 88.657 1 93.156
ENSMUSG00000081094 Rpl19-ps11 pseudogene 1 77.763 1
85.756 1 88.729 1 93.316
ENSMUSG00000081095 Gm14946 pseudogene 1 85.77 NA NA 1
80.089 1 94.904
ENSMUSG00000081096 Gm12691 pseudogene 1 82.352 1
83.733 1 83.857 1 94.904
ENSMUSG00000081097 Gm12135 pseudogene 1 66.652 1
91.955 1 87.744 1 95.72
ENSMUSG00000081098 Gm12220 pseudogene 1 83.523 1
95.941 1 86.258 1 96.183
ENSMUSG00000081099 Gm5270 pseudogene 1 80.51 1 96.763
1 87.971 1 96.287
ENSMUSG00000081100 Gm14630 pseudogene 1 82.891 NA NA
1 78.369 1 95.273
ENSMUSG00000081101 Gm12024 pseudogene 1 85.832 1
96.378 NA NA 1 94.904
ENSMUSG00000081103 Rps12-ps26 pseudogene 0.883 84.397 1.296
93.574 0.876 67.029 1.22 87.886
ENSMUSG00000081105 Gm14087 pseudogene 1 84.738 1
95.504 1 88.41 NA NA
ENSMUSG00000081106 Gm7155 pseudogene 1 79.709 1

96.268 1 86.258 1 96.086
ENSMUSG00000081107 Gm12235 pseudogene 1 85.391 1
91.275 1 78.369 1 88.512
ENSMUSG00000081108 Gm4234 pseudogene 1 62.354 1
90.116 1 88.203 1 96.183
ENSMUSG00000081110 NA NA NA NA 1 96.847 1
84.626 1 95.273
ENSMUSG00000081111 Gm5913 pseudogene 1 84.452 1
94.597 1 88.203 1 95.273
ENSMUSG00000081112 Gm12140 pseudogene 1 84.452 NA NA
1 85.675 1 90.611
ENSMUSG00000081113 Gm7308 pseudogene 1 80.688 1
95.504 1 87.658 1 95.273
ENSMUSG00000081114 Gm15390 pseudogene 1 85.59 1 95.211
1 87.898 1 96.287
ENSMUSG00000081115 Gm15913 pseudogene 1 85.857 1
94.848 1 81.976 1 93.734
ENSMUSG00000081116 Gm13329 pseudogene 1 79.433 1
97.155 1 86.475 1 96.184
ENSMUSG00000081117 Gm11233 pseudogene 1 82.732 1
96.407 1 81.388 1 95.273
ENSMUSG00000081118 Gm6758 pseudogene 1 86.112 1
96.216 1 86.602 1 95.273
ENSMUSG00000081119 Gm14601 pseudogene 1 81.24 NA NA 1
88.657 1 95.74
ENSMUSG00000081120 Gm12458 pseudogene 1 84.977 1
96.138 1 85.792 1 93.156
ENSMUSG00000081121 Gm12791 pseudogene 1 64.193 1
92.631 1 81.169 1 93.132
ENSMUSG00000081122 Gm13678 pseudogene 1 86.474 NA NA
1 74.535 1 95.74
ENSMUSG00000081124 Gm14906 pseudogene 1 85.77 1 96.216
1 86.831 1 95.559
ENSMUSG00000081125 Rpl26-ps5 pseudogene 1 82.352 1
96.138 1 85.464 1 94.608
ENSMUSG00000081126 NA NA NA NA 1.088 96.847 NA NA
0.792 88.505
ENSMUSG00000081127 Gm12677 pseudogene 1 82.705 1
96.138 1 78.045 1 95.593
ENSMUSG00000081128 Gm13328 pseudogene 1 71.217 1
93.654 1 71.734 1 93.612
ENSMUSG00000081129 Gm14605 pseudogene 1 85.355 1
96.013 1 80.089 1 92.903
ENSMUSG00000081130 Gm8217 pseudogene 1 80.408 NA NA
1 85.921 1 94.001
ENSMUSG00000081131 Gm14833 pseudogene 1 84.977 1
93.707 1 88.161 1 95.877
ENSMUSG00000081132 Gm13744 pseudogene 1 79.853 1
95.708 1 85.675 1 92.675
ENSMUSG00000081133 Gm14544 pseudogene 1 86.145 1
97.155 1 86.144 1 96.192
ENSMUSG00000081134 Gm14913 pseudogene 1 83.523 1
97.155 1 79.554 1 96.358
ENSMUSG00000081135 Gm14800 pseudogene 1 85.499 1

93.469 1 81.976 1 90.177
ENSMUSG00000081137 BC022960 pseudogene 1.246 50.913 0.896
91.361 0.893 49.967 0.721 85.493
ENSMUSG00000081138 Gm12834 pseudogene 1 81.823 NA NA
1 82.778 1 95.273
ENSMUSG00000081139 Gm13333 pseudogene 1 83.823 1
96.847 1 84.724 1 95.877
ENSMUSG00000081141 Gm11501 pseudogene 1 79.165 NA NA
NA NA 1 93.722
ENSMUSG00000081143 Gm15823 pseudogene 1 78.054 1
95.431 1 88.203 1 94.904
ENSMUSG00000081144 Vmn2r-ps2 pseudogene 1 79.577 NA NA
1 84.724 1 94.904
ENSMUSG00000081145 Gm14585 pseudogene 1 86.006 1
97.263 1 86.084 1 96.287
ENSMUSG00000081146 Gm6207 pseudogene 1 85.946 1
95.196 1 87.786 1 96.287
ENSMUSG00000081147 Gm6024 pseudogene 1 76.827 1
96.001 1 78.369 1 96.192
ENSMUSG00000081148 Gm11891 pseudogene 1 85.355 1
96.268 1 87.898 1 96.248
ENSMUSG00000081150 NA NA NA NA 1 97.097 1
88.601 1 95.676
ENSMUSG00000081151 Gm11448 pseudogene 1 83.418 1
90.576 1 87.336 1 96.086
ENSMUSG00000081152 Gm12430 pseudogene 1 77.916 1
95.431 1 56.625 1 92.561
ENSMUSG00000081153 Gm11401 pseudogene 1 79.709 1
94.48 1 85.352 1 94.468
ENSMUSG00000081154 Defa-ps13 pseudogene 1 82.732 NA NA
1 85.792 1 96.183
ENSMUSG00000081155 Gm12771 pseudogene 1 82.705 1
95.708 1 80.089 1 95.273
ENSMUSG00000081156 Gm14425 pseudogene 1 64.589 1
94.597 1 76.754 1 86.133
ENSMUSG00000081157 Gm13552 pseudogene 1 82.705 1
95.211 1 79.325 1 96.192
ENSMUSG00000081158 Gm13521 pseudogene 1 74.482 1
95.735 1 86.258 1 95.832
ENSMUSG00000081159 Gm6023 pseudogene 1 81.178 NA NA
1 86.831 1 95.273
ENSMUSG00000081160 Gm14897 pseudogene 1 77.763 1
93.817 1 81.388 1 95.877
ENSMUSG00000081161 Gm12783 pseudogene 0.181 32.749 0.999
92.631 5.549 24.691 4.32 57.1
ENSMUSG00000081162 Olfr400-ps1 pseudogene 1 81.196 1
96.407 1 87.744 1 92.381
ENSMUSG00000081163 Defa-ps12 pseudogene 1 84.865 NA NA
1 88.274 1 96.287
ENSMUSG00000081164 Gm8722 pseudogene 1 77.182 1
93.863 1 88.274 1 94.99
ENSMUSG00000081165 Gm11783 pseudogene 1.661 38.195 0.47
79.475 1.284 80.634 0.938 91.217
ENSMUSG00000081166 Gm12819 pseudogene 1 85.655 1

96.268 NA NA 1 87.7
ENSMUSG00000081167 Gm8880 pseudogene 1 74.482 1
96.216 NA NA 1 94.654
ENSMUSG00000081168 Gm13730 pseudogene 1 86.199 1
95.329 1 88.729 1 96.183
ENSMUSG00000081171 Gm7485 pseudogene 1 83.263 1
96.138 1 83.857 1 89.152
ENSMUSG00000081172 Gm12849 pseudogene 1 85.655 1
96.216 1 86.666 1 94.904
ENSMUSG00000081173 Gm13500 pseudogene 1 76.396 NA NA
1 80.5 1 96.248
ENSMUSG00000081174 Gm13439 pseudogene 1 82.543 NA NA
1 88.274 1 95.273
ENSMUSG00000081175 Gm5793 pseudogene 1 85.832 NA NA
1 82.436 1 94.99
ENSMUSG00000081177 Gm12509 pseudogene 1 64.893 1
95.504 1 73.309 1 95.559
ENSMUSG00000081179 Gm13136 pseudogene 1 83.968 NA NA
1 82.436 1 92.31
ENSMUSG00000081181 Gm13353 pseudogene 1 85.77 1 90.454
1 84.724 1 95.593
ENSMUSG00000081183 Gm12449 pseudogene 1 86.918 1
95.735 1 85.464 1 92.762
ENSMUSG00000081184 Gm12685 pseudogene 1 83.726 1
95.301 1 72.853 1 94.904
ENSMUSG00000081185 Gm4852 pseudogene 1 70.36 1 93.654
1 84.961 1 94.904
ENSMUSG00000081186 Gm12578 pseudogene 1 85.637 NA NA
1 88.274 1 96.287
ENSMUSG00000081187 3110067C02Rik pseudogene 0.825 66.652
1.207 95.211 1.002 87.898 0.743 91.217
ENSMUSG00000081188 Gm14621 pseudogene 1 82.982 1
95.708 1 77.888 1 96.287
ENSMUSG00000081189 Gm12725 pseudogene 1.095 73.124 0.967
85.793 0.94 79.897 0.826 86.699
ENSMUSG00000081194 Gm8424 pseudogene 3.496 10.595 3.151
85.756 0.091 0.945 0.61 96.139
ENSMUSG00000081195 Gm6310 pseudogene 1 85.499 1
96.013 1 78.954 1 95.74
ENSMUSG00000081197 Gm15076 pseudogene 1 81.196 1
97.263 1 86.985 NA NA
ENSMUSG00000081199 Gm16407 pseudogene 1 75.761 1
97.155 1 84.626 1 95.74
ENSMUSG00000081200 Gm15615 pseudogene 1 63.804 1
93.654 1 83.158 1 92.561
ENSMUSG00000081201 Smt3h2-ps4 pseudogene 1 85.129 1
97.263 1 83.811 1 95.273
ENSMUSG00000081202 Gm13220 pseudogene 1 74.516 NA NA
1 79.776 1 95.273
ENSMUSG00000081206 Gm11935 pseudogene 1 83.169 NA NA
1 86.144 1 93.316
ENSMUSG00000081208 Gm11400 pseudogene 0.797 44.467 0.79
68.848 1.582 10.819 1.259 70.624
ENSMUSG00000081209 Gm14890 pseudogene 1 85.857 1

95.708	1	79.325	1	96.287
ENSMUSG00000081210	Gm12933	pseudogene	1	86.172	NA	NA
1	71.839	1	92.851
ENSMUSG00000081211	Gm14886	pseudogene	1	84.661	NA	NA
1	88.41	1	94.654
ENSMUSG00000081212	Gm6964	pseudogene	1	84.263	1
96.68	1	84.308	1	89.498
ENSMUSG00000081214	Rpl35a-ps2	pseudogene	13.655	0	1.124
95.211	0.065	0.72	1.007	92.748
ENSMUSG00000081215	Gm11446	pseudogene	1	76.968	1
96.68	1	80.381	1	94.904
ENSMUSG00000081216	Gm6744	pseudogene	1	82.982	1
95.211	1	85.464	1	92.903
ENSMUSG00000081218	Gm4297	protein_coding	1	84.294	NA
NA	1	86.985	1	91.525
ENSMUSG00000081219	Bambi-ps1	pseudogene	1.202	14.34	0.991	96.68
0.841	16.719	0.97	95.877
ENSMUSG00000081220	Gm14542	pseudogene	1	82.732	1
96.216	1	81.976	1	93.264
ENSMUSG00000081221	Gm14760	pseudogene	1	81.01	1	95.211
1	86.144	1	95.273
ENSMUSG00000081222	Gm15173	pseudogene	1	77.182	1
96.216	1	85.675	1	96.183
ENSMUSG00000081223	Gm12247	pseudogene	0.988	82.092	1.735
84.007	0.583	29.217	2.275	64.905
ENSMUSG00000081224	Gm2066	pseudogene	1	72.985	1
95.088	1	85.21	1	92.675
ENSMUSG00000081226	Gm8802	pseudogene	1	84.324	1
95.708	1	88.41	1	95.273
ENSMUSG00000081227	Gm13082	pseudogene	1	86.006	1
95.809	1	87.744	1	94.608
ENSMUSG00000081229	Lamr1-ps1	pseudogene	1	75.399	1
95.211	1	84.961	1	93.261
ENSMUSG00000081230	NA	NA	NA	NA	1	96.335	1
87.173	1	95.273
ENSMUSG00000081231	Tcp1-ps1	pseudogene	1	80.51	1	95.735
1	76.64	1	92.12
ENSMUSG00000081232	Gm14373	pseudogene	1	67.991	NA	NA
1	80.163	1	90.611
ENSMUSG00000081233	Gm12857	pseudogene	1	68.955	1
94.887	1	45.208	NA	NA
ENSMUSG00000081234	Olfr1150-ps1	pseudogene	1	84.263	1
94.848	1	82.685	1	96.358
ENSMUSG00000081235	Gm14788	pseudogene	1	85.203	1
96.138	1	80.015	1	96.086
ENSMUSG00000081236	Gm13574	pseudogene	1	66.406	1
96.138	1	80.899	1	93.156
ENSMUSG00000081237	Gm11385	pseudogene	1	85.283	1
96.216	1	86.144	1	94.654
ENSMUSG00000081238	NA	NA	NA	NA	1	82.848	1
82.436	1	95.74
ENSMUSG00000081239	Gm11836	pseudogene	1	79.93	1	96.216
1	84.308	1	95.273
ENSMUSG00000081240	Gm16511	pseudogene	1	71.915	1

95.504 1 86.475 1 92.748
ENSMUSG00000081242 Gm15719 pseudogene 1 79.709 1
95.504 1 88.349 1 92.52
ENSMUSG00000081243 Gm14037 pseudogene 1 71.142 1
92.111 1 87.898 1 96.139
ENSMUSG00000081245 Gm6587 pseudogene 1 72.85 NA NA 1
88.461 1 93.685
ENSMUSG00000081248 Gm13787 pseudogene 1.378 53.809 0.717
91.532 1.014 79.255 0.724 95.405
ENSMUSG00000081249 Gm11517 pseudogene 1.11 86.333 0.949
95.708 0.948 81.29 1.065 93.457
ENSMUSG00000081250 Gm15554 pseudogene 1 86.145 NA NA
1 84.626 1 96.358
ENSMUSG00000081251 Gm12164 pseudogene 1 79.853 1
95.504 1 80.5 1 92.31
ENSMUSG00000081252 Gm16032 pseudogene 1 77.763 1
96.763 1 84.83 1 86.301
ENSMUSG00000081253 Gm8501 pseudogene 1 73.124 1
95.35 1 82.575 1 96.071
ENSMUSG00000081254 Gm12112 pseudogene 1 86.464 1
96.335 1 87.431 1 84.246
ENSMUSG00000081258 Gm14013 pseudogene 1 83.726 1
96.001 1 87.336 1 92.851
ENSMUSG00000081259 Gm6893 pseudogene 1 77.182 1
96.268 1 84.626 1 96.358
ENSMUSG00000081260 Gm13149 pseudogene 1 83.09 1 96.216
1 84.896 1 94.825
ENSMUSG00000081262 Gm12261 pseudogene 1 81.01 1 97.155
1 78.954 1 93.964
ENSMUSG00000081263 Gm14859 pseudogene 1 86.145 1
95.35 1 70.44 1 96.184
ENSMUSG00000081264 Gm14021 pseudogene 1 81.647 1
93.574 1 81.032 1 94.904
ENSMUSG00000081266 Gm15820 pseudogene 1 84.534 1
91.661 1 87.658 1 94.016
ENSMUSG00000081268 Gm14909 pseudogene 1 82.092 1
95.35 1 87.898 1 95.92
ENSMUSG00000081269 Gm14659 pseudogene 1 68.16 1 96.843
NA NA 1 94.99
ENSMUSG00000081271 Gm14883 pseudogene 1 85.946 1
95.708 1 87.173 1 96.358
ENSMUSG00000081272 Gm13509 pseudogene 0.978 80.751 1.131
83.464 0.907 43.426 1.172 81.251
ENSMUSG00000081273 Gm15896 pseudogene 1 75.858 1
96.407 NA NA 1 95.877
ENSMUSG00000081274 Gm15727 pseudogene 0.405 14.427 1.043
90.454 2.361 24.383 1.027 96.248
ENSMUSG00000081275 Gm13875 pseudogene 1 84.452 1
95.504 1 87.744 1 93.261
ENSMUSG00000081276 Gm13132 pseudogene 1 66.652 1
95.504 1 64.024 1 95.877
ENSMUSG00000081277 Gm15285 pseudogene 1 77.763 1
92.914 1 60.5 1 92.381
ENSMUSG00000081278 Gm13186 pseudogene 1 77.407 1

90.053 1 85.757 1 96.086
ENSMUSG00000081279 Vmn1r-ps2 pseudogene 1 74.147 1
96.843 1 85.464 1 93.156
ENSMUSG00000081280 Gm9516 pseudogene 1 84.049 1
93.654 1 86.258 1 92.018
ENSMUSG00000081281 Gm6274 pseudogene 1 83.09 1 95.088
1 87.658 1 93.132
ENSMUSG00000081282 Gm13836 pseudogene 3.029 56.311 0.502
94.102 0.66 71.734 1.293 94.904
ENSMUSG00000081283 Vmn1r-ps104 pseudogene 1 80.03 1 95.431
1 81.388 1 96.248
ENSMUSG00000081284 Gm13673 pseudogene 1 75.298 NA NA
1 88.657 1 94.904
ENSMUSG00000081285 Gm18859 pseudogene 1 83.658 1
96.68 1 88.461 1 94.904
ENSMUSG00000081286 Gm14995 pseudogene 1 74.516 1
92.545 1 88.601 1 93.584
ENSMUSG00000081287 Olfr333-ps1 pseudogene 1 77.497 1
96.216 1 82.575 1 94.016
ENSMUSG00000081288 Gm11386 pseudogene 1 70.597 1
96.268 1 84.308 1 95.676
ENSMUSG00000081289 Gm14857 pseudogene 1 68.16 NA NA 1
87.971 1 94.153
ENSMUSG00000081290 Gm12520 pseudogene 1 82.982 1
95.431 1 74.686 1 96.183
ENSMUSG00000081291 Gm14738 pseudogene 1 83.418 NA NA
1 87.686 1 93.132
ENSMUSG00000081292 Gm15018 pseudogene 1 82.891 1
95.329 1 81.29 1 94.608
ENSMUSG00000081293 Gm11673 pseudogene 1 85.499 1
96.138 1 87.274 1 96.358
ENSMUSG00000081295 Gm6275 pseudogene 1 76.319 1
97.155 1 88.076 1 96.358
ENSMUSG00000081296 Gm12203 pseudogene 1 86.658 1
96.335 1 87.786 1 94.904
ENSMUSG00000081297 Gm12209 pseudogene 1 53.679 1
95.504 1 88.461 1 96.183
ENSMUSG00000081298 Gm14622 pseudogene 1 84.977 1
95.708 1 80.015 1 96.287
ENSMUSG00000081299 Gm13603 pseudogene 1 82.982 1
95.504 1 83.607 1 96.248
ENSMUSG00000081300 Gm14587 pseudogene 1 84.865 1
96.652 NA NA 1 95.273
ENSMUSG00000081301 Gm15951 pseudogene 1 77.497 NA NA
1 87.274 1 96.248
ENSMUSG00000081302 Gm12020 pseudogene 1 83.09 1 96.216
1 81.492 1 96.358
ENSMUSG00000081304 Gm11509 pseudogene 1 83.658 1
93.908 1 71.232 1 94.215
ENSMUSG00000081305 Gm12879 pseudogene 1 80.408 1
92.253 1 87.744 1 95.877
ENSMUSG00000081306 Gm15266 pseudogene 1 72.985 1
94.848 1 70.534 1 87.447
ENSMUSG00000081307 Gm13627 pseudogene 1 79.433 1

91.532 1 80.634 1 95.74
ENSMUSG00000081309 Gm12397 pseudogene 1 86.112 1
96.948 1 88.729 1 95.35
ENSMUSG00000081310 Gm14054 pseudogene 1 85.092 1
96.216 1 84.83 1 93.261
ENSMUSG00000081311 Gm12197 pseudogene 1 67.163 1
94.262 1 73.096 1 93.316
ENSMUSG00000081312 Gm14520 pseudogene 1 86.843 1
96.001 1 85.464 1 95.35
ENSMUSG00000081313 Gm14213 pseudogene 1 82.479 1
92.474 1 86.602 1 95.777
ENSMUSG00000081314 Gm14132 pseudogene 1 86.006 1
96.843 1 76.754 1 92.675
ENSMUSG00000081315 Olfr621-ps1 pseudogene 1 84.738 1
95.504 1 87.971 1 95.72
ENSMUSG00000081316 Gm16205 pseudogene 1 80.815 1
95.211 1 88.203 1 96.086
ENSMUSG00000081317 Gm12757 pseudogene 1 85.946 1
93.899 1 81.857 1 96.183
ENSMUSG00000081318 Rpl36-ps1 pseudogene 1 86.918 1
96.013 1 85.58 1 96.358
ENSMUSG00000081319 Gm12936 pseudogene 1 86.658 1
96.013 1 79.119 1 93.413
ENSMUSG00000081320 Gm15034 pseudogene 1 80.688 1
95.504 1 82.778 1 95.559
ENSMUSG00000081321 Gm15304 pseudogene 1 85.946 1
95.708 1 86.144 1 96.358
ENSMUSG00000081323 NA NA NA NA 1 96.268 1
84.961 1 93.627
ENSMUSG00000081324 Gm14550 pseudogene 1 85.283 NA NA
1 85.134 1 92.12
ENSMUSG00000081325 Gm12599 pseudogene 1 75.567 1
94.48 1 78.498 1 95.559
ENSMUSG00000081326 Gm14417 pseudogene 1 85.728 1
95.088 1 85.757 1 95.72
ENSMUSG00000081327 Gm11819 pseudogene 1 82.705 1
95.504 1 84.626 1 96.287
ENSMUSG00000081328 Olfr1001-ps1 pseudogene 1 72.372 1
96.013 1 88.693 1 89.152
ENSMUSG00000081329 Gm12492 pseudogene 1 85.832 1
96.268 1 88.203 1 94.904
ENSMUSG00000081330 Gm13013 pseudogene 1 72.743 1
95.708 1 79.554 1 92.018
ENSMUSG00000081331 Gm15151 pseudogene 1 84.819 1
95.088 1 61.743 1 94.904
ENSMUSG00000081332 Gm12664 pseudogene 1 85.092 1
97.129 1 84.308 1 95.832
ENSMUSG00000081333 Gm12307 pseudogene 1 82.092 1
91.897 1 85.921 1 96.358
ENSMUSG00000081334 Vmn1r-ps94 pseudogene 1 86.199 NA NA
1 87.686 1 94.016
ENSMUSG00000081335 Gm15786 pseudogene 1 84.168 1
95.504 1 84.504 1 93.457
ENSMUSG00000081336 Gm15073 pseudogene 1 66.652 1

96.308 1 86.346 1 94.904
ENSMUSG00000081339 Gm16044 pseudogene 0.931 81.465 NA NA
0.953 81.032 1.452 93.016
ENSMUSG00000081340 Gm12275 pseudogene 0.544 46.289 0.797
79.475 2.309 16.666 2.885 44.213
ENSMUSG00000081341 Gm14891 pseudogene 1 83.333 1
96.652 1 81.492 1 96.287
ENSMUSG00000081342 Gm11434 pseudogene 1 75.196 1
91.361 1 76.754 1 91.291
ENSMUSG00000081343 Gm16071 pseudogene 1.202 80.251 0.976
87.029 0.85 44.678 0.662 84.19
ENSMUSG00000081344 Gm14303 pseudogene 23.894 0 1.847
83.104 0.023 0.72 0.994 92.31
ENSMUSG00000081345 Olfr1159-ps1 pseudogene 1 78.639 NA
NA 1 81.032 1 94.904
ENSMUSG00000081346 Gm12030 pseudogene 1 79.068 1
96.897 1 79.426 1 94.904
ENSMUSG00000081347 Gm14702 pseudogene 1 79.93 1 97.263
1 82.778 1 95.273
ENSMUSG00000081348 Gm13878 pseudogene 1 85.832 NA NA
1 84.724 1 96.086
ENSMUSG00000081349 Gm15041 pseudogene 1 70.121 NA NA
1 86.985 1 94.297
ENSMUSG00000081350 Gm5388 pseudogene 1 86.145 1
95.708 1 78.954 1 95.72
ENSMUSG00000081352 Gm12389 pseudogene 1 79.709 1
93.707 1 79.325 1 96.183
ENSMUSG00000081353 Gm13818 pseudogene 1 80.608 1
95.708 1 65.8 1 91.872
ENSMUSG00000081354 Gm14894 pseudogene 1 86.351 1
97.263 1 85.21 1 96.183
ENSMUSG00000081358 Gm14746 pseudogene 1 80.815 1
96.897 1 81.976 1 94.297
ENSMUSG00000081359 Cox20-ps pseudogene 1 78.224 1
93.654 1 85.58 1 95.877
ENSMUSG00000081360 Gm11718 pseudogene 1 84.738 1
95.708 1 82.161 1 96.071
ENSMUSG00000081361 Gm11813 pseudogene 1 86.112 1
93.469 1 79.554 1 93.722
ENSMUSG00000081362 Cyp2j7-ps protein_coding 1 66.652 NA
NA 1 84.071 1 93.627
ENSMUSG00000081363 Gm11500 pseudogene 1 86.57 1 96.847
1 86.727 1 88.197
ENSMUSG00000081364 Gm8595 pseudogene 1 81.178 1
92.631 1 75.353 1 94.99
ENSMUSG00000081365 Gm15031 pseudogene 1 77.916 NA NA
1 84.308 1 93.264
ENSMUSG00000081367 Gm13265 pseudogene 1 86.74 NA NA 1
88.203 1 95.676
ENSMUSG00000081369 Gm11896 pseudogene 1 83.263 1
95.504 1 87.103 1 95.92
ENSMUSG00000081370 Gm9105 pseudogene 1 86.918 1
95.708 1 86.112 1 96.086
ENSMUSG00000081373 Gm16436 pseudogene 1 85.637 1

						96.843	1	80.899	1	96.183
ENSMUSG00000081374	Gm15011	pseudogene	1	80.51	1	97.155	1	88.657	1	95.273
ENSMUSG00000081375	Gm14686	pseudogene	1	85.203	1	97.263	1	88.076	1	88.197
ENSMUSG00000081377	Gm12877	pseudogene	1	81.178	1	95.211	1	79.255	1	94.608
ENSMUSG00000081378	Rps13-ps4	pseudogene	0.866	77.407	1.269	90.092	0.908	70.44	1.215	90.177
ENSMUSG00000081379	Gm14771	pseudogene	1	85.832	1	95.504	1	86.666	1	96.184
ENSMUSG00000081381	Gm5752	pseudogene	1	78.224	1	93.908	1	82.685	1	95.676
ENSMUSG00000081383	NA	NA	NA	NA	1	91.275	1	80.089	1	96.287
ENSMUSG00000081384	Gm12752	pseudogene	1	78.224	NA	NA	1	86.258	1	96.183
ENSMUSG00000081386	Gm15160	pseudogene	1	77.763	1	95.504	1	85.921	1	95.405
ENSMUSG00000081388	Gm11801	pseudogene	1	73.487	1	94.48	1	86.985	1	95.74
ENSMUSG00000081389	Gm13368	pseudogene	1	74.299	NA	NA	1	83.857	1	95.273
ENSMUSG00000081390	Gm14538	pseudogene	1	86.112	1	97.129	1	87.103	1	96.358
ENSMUSG00000081391	Gm11765	pseudogene	1	83.726	1	97.155	1	85.935	1	95.273
ENSMUSG00000081394	Gm13215	pseudogene	1	78.538	1	96.847	1	87.686	1	93.964
ENSMUSG00000081395	Gm14879	pseudogene	1	86.112	NA	NA	1	78.864	1	96.287
ENSMUSG00000081396	Gm15132	pseudogene	1.504	55.789	1.119	88.551	0.593	42.621	0.518	84.29
ENSMUSG00000081397	Gm14815	pseudogene	1	78.744	NA	NA	1	87.431	1	96.287
ENSMUSG00000081398	NA	NA	NA	NA	1	97.049	1	87.744	1	96.358
ENSMUSG00000081401	Gm14889	pseudogene	1	84.977	1	97.263	1	84.724	1	95.273
ENSMUSG00000081403	Gm14658	pseudogene	1	83.169	1	96.216	1	79.255	1	96.086
ENSMUSG00000081404	Gm15694	pseudogene	3.281	63.126	0.359	81.731	0.849	80.015	1.537	83.067
ENSMUSG00000081405	Gm13142	pseudogene	1	73.401	1	96.847	1	48.133	1	92.742
ENSMUSG00000081407	Gm14568	pseudogene	1	83.169	NA	NA	1	83.607	1	96.287
ENSMUSG00000081408	Gm15047	pseudogene	1	84.564	1	96.138	1	84.724	1	94.825
ENSMUSG00000081409	Gm8215	pseudogene	1	82.891	1	96.138	1	85.792	1	94.19
ENSMUSG00000081410	Gm6797	pseudogene	1	86.918	1	96.001	1	84.229	1	95.273
ENSMUSG00000081412	Gm15403	pseudogene	1	60.707	1					

92.111 1 87.971 1 96.071
ENSMUSG00000081413 Gm14088 pseudogene 1 79.165 1
97.263 1 87.173 1 95.35
ENSMUSG00000081414 Gm3943 pseudogene 1 57.227 1
96.763 1 77.17 1 92.381
ENSMUSG00000081415 Gm15176 pseudogene 1 76.135 NA NA
1 86.144 1 93.734
ENSMUSG00000081416 Gm14383 pseudogene 1 83.333 1
92.404 1 84.896 1 93.156
ENSMUSG00000081417 Gm15603 pseudogene 1 85.832 NA NA
1 87.898 1 94.904
ENSMUSG00000081418 Gm14830 pseudogene 0.978 74.689 0.82
96.138 1.247 70.534 0.706 83.724
ENSMUSG00000081420 Olfr29-ps1 pseudogene 1 77.113 1
95.35 1 85.352 1 96.358
ENSMUSG00000081421 Gm13445 pseudogene 1 83.823 1
96.407 1 88.729 1 93.413
ENSMUSG00000081422 Gm14282 pseudogene 1 82.732 1
92.793 1 80.015 1 94.384
ENSMUSG00000081423 Gm12882 pseudogene 1 83.658 1
93.899 1 88.41 1 94.001
ENSMUSG00000081424 Gm14774 pseudogene 1 47.677 1
93.899 1 75.155 1 93.722
ENSMUSG00000081426 Gm15714 pseudogene 1 85.355 NA NA
1 83.607 1 93.584
ENSMUSG00000081427 Gm12382 pseudogene 1 85.728 1
90.256 1 85.935 1 96.287
ENSMUSG00000081428 Tpt1-ps2 pseudogene 1 86.658 1
96.268 1 84.896 1 96.287
ENSMUSG00000081429 Gm11603 pseudogene 1 85.728 1
95.211 1 88.349 1 91.011
ENSMUSG00000081432 Gm12593 pseudogene 1 82.732 1
97.049 1 88.461 1 96.086
ENSMUSG00000081433 Serpinb1-ps1 pseudogene 1 80.251 1
78.51 1 48.451 1 93.722
ENSMUSG00000081434 Gm14165 pseudogene 6.947 3.152 0.562 95.504
0.256 10.003 2.082 52.366
ENSMUSG00000081435 Gm11403 pseudogene 1 84.977 1
96.138 1 88.349 1 95.877
ENSMUSG00000081436 Gm5912 pseudogene 1 84.738 1
95.211 1 70.623 1 96.192
ENSMUSG00000081437 Gm12402 pseudogene 1 73.401 1
93.817 1 84.229 1 96.139
ENSMUSG00000081438 Gm11254 pseudogene 1 81.823 1
95.211 1 86.258 1 92.748
ENSMUSG00000081439 Gm7840 pseudogene 1 85.637 1
95.708 1 80.381 1 96.086
ENSMUSG00000081440 Gm12464 pseudogene 1 86.658 1
95.941 1 76.754 1 94.825
ENSMUSG00000081441 Gm6161 pseudogene 0.668 28.584 0.869
79.475 1.726 14.449 1.109 73.939
ENSMUSG00000081442 Gm14682 pseudogene 1 86.819 1
96.268 1 87.744 1 95.72
ENSMUSG00000081443 Gm3880 pseudogene 1 84.263 1

95.708 1 77.704 1 93.612
ENSMUSG00000081444 Gm15803 pseudogene 1 84.738 1
96.138 1 86.831 1 95.72
ENSMUSG00000081445 Rpl23a-ps4 pseudogene 1 85.655 1
97.263 NA NA 1 91.625
ENSMUSG00000081446 Gm14068 pseudogene 1 84.819 1
96.763 1 88.203 1 93.734
ENSMUSG00000081447 Gm13870 pseudogene 1 75.671 1
87.452 1 69.825 1 95.877
ENSMUSG00000081449 Gm11432 pseudogene 1 71.915 1
95.329 1 69.825 1 96.248
ENSMUSG00000081450 Gm11700 pseudogene 1 71.674 1
97.155 1 79.426 1 95.92
ENSMUSG00000081452 Gm14761 pseudogene 1 84.452 NA NA
1 87.686 1 92.181
ENSMUSG00000081453 Gm6767 pseudogene 1 84.397 1
96.216 1 78.864 1 88.111
ENSMUSG00000081455 Hmgb1-ps3 pseudogene 1 67.608 1
96.335 1 86.985 1 96.248
ENSMUSG00000081456 Gm7781 pseudogene 1 66.406 1
96.843 1 87.431 1 96.183
ENSMUSG00000081457 Gm13488 pseudogene 1 72.372 NA NA
1 87.431 1 96.071
ENSMUSG00000081458 Gm14796 pseudogene 1 85.129 1
97.263 1 88.41 1 93.734
ENSMUSG00000081459 Llph-ps1 pseudogene 1 83.523 1
96.68 1 85.21 1 94.825
ENSMUSG00000081460 Gm13168 pseudogene 1 81.465 1
95.735 1 41.928 1 96.183
ENSMUSG00000081461 Rpl30-ps11 pseudogene 1 84.738 1
96.216 1 77.037 1 96.139
ENSMUSG00000081463 Gm15049 pseudogene 1 86.74 1 97.263
1 85.935 1 95.273
ENSMUSG00000081464 Gm14732 pseudogene 1 86.74 1 95.088
1 82.31 1 95.72
ENSMUSG00000081465 Gm12884 pseudogene 1 78.299 1
97.155 1 84.961 1 92.903
ENSMUSG00000081466 Gm14912 pseudogene 1 81.647 1
96.216 1 86.666 1 93.156
ENSMUSG00000081467 Gm13337 pseudogene 1 82.092 1
97.263 1 87.519 1 95.72
ENSMUSG00000081469 Vmn1r-ps95 pseudogene 1 76.042 1
96.216 1 81.976 1 94.153
ENSMUSG00000081470 Gm14131 pseudogene 1 81.01 1 94.848
1 86.666 1 91.928
ENSMUSG00000081471 Gm14735 pseudogene 1 80.146 1
96.948 1 80.089 1 92.31
ENSMUSG00000081473 Gm4853 pseudogene 1 79.709 1
93.817 1 87.274 1 91.372
ENSMUSG00000081474 Gm12855 pseudogene 1 81.823 1
95.708 1 80.089 1 95.593
ENSMUSG00000081475 Gm14558 pseudogene 1 81.823 1
93.765 1 74.535 1 95.777
ENSMUSG00000081476 Itpa-ps1 pseudogene 1 78.428 1

88.881 1 87.336 1 95.74
ENSMUSG00000081477 Gm7916 pseudogene 1 85.77 1 95.088
1 73.309 1 93.413
ENSMUSG00000081478 AA623943 pseudogene 1 81.196 1
95.35 1 83.397 1 93.584
ENSMUSG00000081480 Gm13608 pseudogene 1 84.534 1
87.037 1 88.601 1 95.273
ENSMUSG00000081481 Gm14293 pseudogene 1 78.744 NA NA
1 84.504 1 96.248
ENSMUSG00000081483 Gm12445 pseudogene 1 82.231 NA NA
1 80.381 1 93.612
ENSMUSG00000081484 Gm13492 pseudogene 1 83.09 1 96.268
1 82.778 1 93.264
ENSMUSG00000081486 Gm14452 pseudogene 1 79.433 1
96.216 1 77.532 1 95.74
ENSMUSG00000081487 Gm13689 pseudogene 1.493 55.124 0.849
97.288 0.788 52.658 0.579 83.724
ENSMUSG00000081489 Gm14672 pseudogene 1 78.744 NA NA
1 82.575 1 95.35
ENSMUSG00000081491 Gm12778 pseudogene 1 84.564 1
96.843 1 87.786 1 86.699
ENSMUSG00000081492 Gm9311 pseudogene 1 78.538 1
89.713 1 44.787 1 89.498
ENSMUSG00000081494 Gm14130 pseudogene 1 77.113 1
95.708 NA NA 1 92.748
ENSMUSG00000081495 Vmn1r-ps108 pseudogene 1 80.51 1 96.68 1
88.729 1 93.685
ENSMUSG00000081496 Gm13428 pseudogene 1 79.165 1
96.013 1 86.144 1 93.722
ENSMUSG00000081498 Olfr407-ps1 pseudogene 1 86.333 1
97.263 NA NA 1 91.807
ENSMUSG00000081499 Gm12517 pseudogene 1 82.092 1
94.848 1 83.066 1 95.74
ENSMUSG00000081500 Gm15233 pseudogene 1 84.738 NA NA
NA NA 1 91.525
ENSMUSG00000081502 Gm5646 pseudogene 1 86.57 1 95.708
1 87.336 1 93.156
ENSMUSG00000081504 Gm12508 pseudogene 1 86.464 NA NA
1 87.786 1 95.74
ENSMUSG00000081505 Gm8644 pseudogene 1 75.298 1
91.532 1 87.37 1 94.654
ENSMUSG00000081506 Amy2-ps1 pseudogene 1 81.465 NA NA
1 80.634 1 96.071
ENSMUSG00000081507 Gm14993 pseudogene 1 75.298 1
96.001 1 87.519 1 96.183
ENSMUSG00000081508 Gm12654 pseudogene 1 83.418 1
95.211 NA NA 1 93.156
ENSMUSG00000081510 Gm12768 pseudogene 1 84.661 1
94.597 1 84.896 1 88.797
ENSMUSG00000081512 Gm15821 protein_coding 0.782 17.276
1.295 79.864 0.99 70.534 0.885 84.29
ENSMUSG00000081513 Gm11623 pseudogene 1 81.76 1 95.431
1 84.504 1 96.287
ENSMUSG00000081514 Gm14673 pseudogene 1 82.982 NA NA

1 78.642 1 96.183
ENSMUSG00000081515 Gm12311 pseudogene 1 77.615 1
96.138 1 60.5 1 91.525
ENSMUSG00000081517 Gm16204 pseudogene 1 81.823 NA NA
1 84.669 1 93.132
ENSMUSG00000081518 Gm12084 pseudogene 1 84.294 1
97.049 1 80.089 1 95.676
ENSMUSG00000081519 Gm13331 pseudogene 0.631 28.584 1.121
91.584 1.412 44.515 0.638 87.735
ENSMUSG00000081520 Gm16200 pseudogene 0.5 72.546 1.468
92.931 1.357 75.009 1.398 85.629
ENSMUSG00000081522 Gm13170 pseudogene 1 66.652 1
96.652 1 84.308 1 93.156
ENSMUSG00000081523 Gm13058 pseudogene 1 86.199 1
95.708 1 84.308 1 96.192
ENSMUSG00000081524 Gm11658 pseudogene 1 65.284 1
95.088 1 70.44 1 93.264
ENSMUSG00000081525 Gm13325 pseudogene 1 67.163 1
95.809 1 80.634 1 93.316
ENSMUSG00000081526 Gm7219 pseudogene 1 80.608 1
95.211 1 76.225 1 96.086
ENSMUSG00000081528 Gm12924 pseudogene 1 82.479 NA NA
1 84.83 1 93.722
ENSMUSG00000081529 Gm14884 pseudogene 1 85.77 1 95.35 1
86.112 1 94.904
ENSMUSG00000081530 Gm12646 pseudogene 1 71.217 1
93.654 1 88.076 1 96.287
ENSMUSG00000081531 Vmn1r-ps113 pseudogene 1 84.452 1
96.897 1 84.626 1 94.567
ENSMUSG00000081532 Gm15781 pseudogene 1 85.728 1
95.504 1 80.015 1 93.156
ENSMUSG00000081533 Gm15111 pseudogene 1 85.946 1
96.652 1 88.693 1 94.82
ENSMUSG00000081534 Slc48a1 protein_coding 0.896 2.009 1.02
79.579 1.089 52.8 0.981 95.273
ENSMUSG00000081535 Gm15453 pseudogene 1.03 79.709 0.91
95.211 1.065 52.432 1.837 43.377
ENSMUSG00000081536 Gm15193 pseudogene 1 77.916 1
96.268 1 87.744 1 93.261
ENSMUSG00000081537 Gm15235 pseudogene 1 83.968 NA NA
1 84.552 1 96.192
ENSMUSG00000081538 Gm13636 pseudogene 1 85.129 1
96.984 1 86.258 1 94.904
ENSMUSG00000081539 Gm11990 pseudogene 1 84.049 1
95.708 1 83.925 1 92.748
ENSMUSG00000081540 Gm12538 pseudogene 1 83.263 1
96.68 1 86.602 1 96.248
ENSMUSG00000081541 Gm13940 pseudogene 1 80.751 1
96.652 NA NA 1 95.405
ENSMUSG00000081542 Gm12512 pseudogene 1 80.146 1
95.708 1 88.693 1 96.358
ENSMUSG00000081543 Gm12733 pseudogene 1 79.709 1
96.335 1 80.5 1 96.287
ENSMUSG00000081544 Gm13077 pseudogene 1 85.59 1 97.263

1 84.308 1 96.358
ENSMUSG00000081546 Gm14927 pseudogene 1 71.763 1
96.216 1 81.169 1 95.777
ENSMUSG00000081547 Gm11553 pseudogene 1 85.946 1
93.765 NA NA 1 94.215
ENSMUSG00000081548 Gm11692 pseudogene 1 61.171 1
97.155 1 84.504 1 96.287
ENSMUSG00000081550 Gm15877 pseudogene 1 74.021 1
84.768 1 85.352 1 92.389
ENSMUSG00000081551 Gm14654 pseudogene 1 86.172 NA NA
1 88.693 1 96.287
ENSMUSG00000081552 Gm12328 pseudogene 1 79.93 1 95.504
1 87.744 1 95.832
ENSMUSG00000081553 Gm12699 pseudogene 1 84.865 1
96.847 NA NA 1 96.358
ENSMUSG00000081555 Gm6427 pseudogene 1 77.763 1
91.955 1 86.258 1 96.248
ENSMUSG00000081556 Gm15778 pseudogene 1 86.843 1
95.504 1 79.897 1 94.608
ENSMUSG00000081557 Gm5697 pseudogene 1 76.686 1
96.138 1 81.525 1 94.904
ENSMUSG00000081559 Gm12411 pseudogene 1 50.229 1
96.843 1 86.346 1 96.192
ENSMUSG00000081560 Gm9218 pseudogene 1 82.705 1
94.848 1 86.144 1 92.762
ENSMUSG00000081561 Gm12425 pseudogene 1 73.487 1
95.708 1 80.015 1 95.559
ENSMUSG00000081562 Gm11575 pseudogene 1 83.263 NA NA
1 87.103 1 91.928
ENSMUSG00000081563 Gm12635 pseudogene 1 85.857 1
95.301 1 88.461 1 92.018
ENSMUSG00000081564 Gm13717 pseudogene 0.971 74.482 NA NA
1.05 82.436 0.969 93.964
ENSMUSG00000081565 Rps12-ps5 pseudogene 1 83.333 1
92.137 1 85.792 1 95.273
ENSMUSG00000081566 Gm13756 pseudogene 1 86.112 1
90.712 1 83.925 1 96.358
ENSMUSG00000081567 Gm14838 pseudogene 1 84.452 1
96.216 1 86.727 1 94.19
ENSMUSG00000081568 Gm15032 pseudogene 1 84.397 1
94.597 1 87.103 1 94.825
ENSMUSG00000081570 Gm12192 pseudogene 1 85.637 1
91.955 1 87.519 1 93.261
ENSMUSG00000081571 Gm12125 pseudogene 1 84.738 1
96.843 1 82.436 1 94.904
ENSMUSG00000081572 Gm14138 pseudogene 1 79.577 1
96.138 1 79.776 1 88.245
ENSMUSG00000081573 Gm15164 pseudogene 1 81.01 1 92.474
1 86.084 1 94.904
ENSMUSG00000081574 Gm14429 pseudogene 0.545 60.569 1.417
91.456 1.291 75.009 1.407 91.011
ENSMUSG00000081575 Gm12090 pseudogene 1 74.689 1
97.263 1 79.625 1 93.413
ENSMUSG00000081576 Gm11279 pseudogene 1 85.59 NA NA 1

76.498	1	95.777
ENSMUSG00000081577	Gm13455	pseudogene	1	68.727	NA	NA
NA	NA	1	96.358
ENSMUSG00000081578	Gm12611	pseudogene	1	55.641	1
83.104	1	84.308	1	93.734
ENSMUSG00000081579	Gm11909	pseudogene	1	79.853	1
95.504	1	83.066	1	93.156
ENSMUSG00000081580	Gm14129	pseudogene	1	85.129	1
96.843	1	74.082	1	93.261
ENSMUSG00000081581	Gm14312	pseudogene	1	80.815	1
96.001	1	88.349	1	96.139
ENSMUSG00000081584	Gm12716	pseudogene	1	76.827	1
90.378	1	82.575	1	95.74
ENSMUSG00000081585	Gm12926	pseudogene	1	65.106	NA	NA
1	78.498	NA	NA
ENSMUSG00000081586	Gm11544	pseudogene	1	84.294	1
95.708	1	87.898	1	91.928
ENSMUSG00000081587	Gm14926	pseudogene	1	82.231	1
96.268	1	87.274	1	96.086
ENSMUSG00000081588	Gm11661	pseudogene	1	75.858	1
96.138	1	79.119	1	96.358
ENSMUSG00000081589	Gm14353	pseudogene	1	86.918	1
93.574	1	79.119	1	93.685
ENSMUSG00000081590	Gm14413	pseudogene	1	79.577	1
95.809	1	79.255	1	90.157
ENSMUSG00000081591	Gm13440	pseudogene	1	86.199	1
96.68 1	87.431	1	96.086
ENSMUSG00000081592	Gm11936	pseudogene	1	65.388	1
96.001	1	79.119	1	94.468
ENSMUSG00000081593	Gm11841	pseudogene	0.773 61.633	0.828
88.705	1.555 54.163	1.249 95.877
ENSMUSG00000081594	Gm15467	pseudogene	1	41.617	1
95.431	1	88.461	1	83.067
ENSMUSG00000081595	Gm13036	pseudogene	1	81.01 1	96.268
1	71.839	1	95.72
ENSMUSG00000081596	Gm15968	pseudogene	1	76.396	1
96.268	1	84.896	1	95.35
ENSMUSG00000081597	Gm14506	pseudogene	1	85.946	1
96.268	1	83.925	1	96.184
ENSMUSG00000081598	Gm8312	pseudogene	1	74.689	1
97.155	1	87.103	1	96.358
ENSMUSG00000081599	Gm14390	pseudogene	1	85.391	1
95.504	1	76.923	1	95.273
ENSMUSG00000081601	Gm13065	pseudogene	1	86.333	NA	NA
1	87.173	1	91.525
ENSMUSG00000081602	Gm15369	pseudogene	1	83.169	NA	NA
1	86.258	1	94.904
ENSMUSG00000081603	Gm14681	pseudogene	1	85.203	1
96.68 1	83.538	1	94.297
ENSMUSG00000081604	Gm11518	pseudogene	1	86.658	1
90.053	1	84.626	1	86.209
ENSMUSG00000081605	Gm15953	pseudogene	0.688 28.804	1.325
82.219	1.097 77.704	0.718 85.493
ENSMUSG00000081606	Gm9059	pseudogene	1	81.01 1	96.138

1 87.519 1 94.82
ENSMUSG00000081607 Gm15294 protein_coding 1 85.655 NA
NA 1 80.163 1 96.183
ENSMUSG00000081608 Gm16210 pseudogene 1 83.523 1
93.765 1 66.043 1 94.99
ENSMUSG00000081609 Gm12939 pseudogene 1 85.203 1
97.263 NA NA 1 95.72
ENSMUSG00000081610 Gm13733 pseudogene 1 80.751 1
96.68 1 85.58 1 96.071
ENSMUSG00000081611 Gm13651 pseudogene 1 84.819 1
95.196 1 84.83 1 91.525
ENSMUSG00000081612 NA NA NA NA 1 95.088 1
82.436 1 96.183
ENSMUSG00000081613 Gm12109 pseudogene 1 69.777 1
92.355 1 86.144 1 96.287
ENSMUSG00000081614 Gm9436 pseudogene 1 77.497 1
97.155 1 86.727 1 96.086
ENSMUSG00000081615 Gm12539 pseudogene 1 81.647 NA NA
1 86.258 1 92.903
ENSMUSG00000081616 Gm14247 pseudogene 1 83.823 1
95.211 1 79.625 1 95.74
ENSMUSG00000081617 Gm11601 pseudogene 1 66.76 1 96.68 1
88.461 1 96.287
ENSMUSG00000081618 5033423K11Rik pseudogene 1 84.263 1
95.504 NA NA 1 95.92
ENSMUSG00000081620 Gm14633 pseudogene 1 84.819 1
95.941 1 88.693 1 83.067
ENSMUSG00000081622 Gm5937 pseudogene 1 79.165 1
91.361 1 81.976 1 96.183
ENSMUSG00000081623 Gm8809 pseudogene 1 76.968 NA NA
NA NA 1 92.52
ENSMUSG00000081624 Gm6912 pseudogene 1 81.76 1 96.216
1 80.634 1 94.825
ENSMUSG00000081625 Rps12-ps7 pseudogene 1 69.985 1
95.735 1 80.381 1 96.358
ENSMUSG00000081626 Gm14369 pseudogene 1 84.977 1
96.138 1 88.41 1 94.016
ENSMUSG00000081627 Gm13158 pseudogene 1 86.172 1
95.504 NA NA 1 95.777
ENSMUSG00000081628 Gm14804 pseudogene 1 85.129 NA NA
1 88.601 1 96.248
ENSMUSG00000081629 Eif3s6-ps1 pseudogene 1 83.726 1
96.843 1 88.161 1 95.273
ENSMUSG00000081630 Gm11498 pseudogene 1 80.815 1
95.708 1 87.971 1 93.316
ENSMUSG00000081631 Gm12005 pseudogene 1 82.732 NA NA
1 86.112 1 92.691
ENSMUSG00000081632 Gm11443 pseudogene 1 80.608 1
96.652 1 87.173 1 95.877
ENSMUSG00000081634 Gm13621 pseudogene 1 45.61 1 95.211
1 73.096 1 88.387
ENSMUSG00000081635 Gm15071 pseudogene 1 85.728 1
96.68 1 73.309 1 96.358
ENSMUSG00000081636 Gm15901 pseudogene 1 71.915 1

94.887 1 73.309 1 96.358
ENSMUSG00000081638 Gm14594 pseudogene 1 85.355 1
95.504 1 85.935 1 94.297
ENSMUSG00000081639 Gm14973 pseudogene 1 81.01 1 95.504
1 85.757 1 93.734
ENSMUSG00000081640 Rplp0-ps1 pseudogene 1 81.178 1
96.847 1 84.308 1 92.31
ENSMUSG00000081641 Gm9439 pseudogene 1 85.857 1
96.216 NA NA 1 94.99
ENSMUSG00000081642 Gm13532 pseudogene 1 84.977 1
95.708 1 88.601 1 92.181
ENSMUSG00000081643 Gm11605 pseudogene 1 86.474 1
95.301 NA NA 1 95.777
ENSMUSG00000081646 Gm15060 pseudogene 1 74.299 1
96.68 1 82.778 1 93.156
ENSMUSG00000081648 Gm13423 pseudogene 1.213 83.523 0.776
79.807 1.061 64.024 1.163 79.268
ENSMUSG00000081649 Olfr213 protein_coding 1 82.732 1
90.712 1 88.274 1 94.384
ENSMUSG00000081650 Gm16181 protein_coding 0.69 25.551
0.962 91.361 1.499 26.179 1.097 91.625
ENSMUSG00000081651 Gm15530 pseudogene 3.274 52.134 0.209
82.073 1.453 75.809 1.088 74.047
ENSMUSG00000081652 Gm14283 pseudogene 1 82.352 1
97.155 1 84.229 1 95.877
ENSMUSG00000081653 Gm11354 pseudogene 1 82.982 1
96.68 1 83.607 1 95.593
ENSMUSG00000081654 Gm5383 pseudogene 1 86.351 1
93.654 NA NA 1 94.608
ENSMUSG00000081656 Gm11246 pseudogene 1 73.734 NA NA
1 86.602 1 94.153
ENSMUSG00000081658 Gm12077 pseudogene 1 82.543 1
97.263 1 80.015 1 92.762
ENSMUSG00000081659 Gm14917 pseudogene 1 82.705 NA NA
1 88.461 1 95.74
ENSMUSG00000081660 Gm11787 pseudogene 1 75.567 NA NA
1 83.158 1 95.72
ENSMUSG00000081661 NA NA NA NA 1 95.708 1
85.464 1 95.405
ENSMUSG00000081662 Gm14098 pseudogene 1 73.401 1
94.848 1 84.724 1 92.748
ENSMUSG00000081663 Gm11207 pseudogene 1 85.832 1
96.68 1 88.461 1 92.903
ENSMUSG00000081664 Gm15544 pseudogene 1 85.129 1
92.111 1 76.768 1 95.72
ENSMUSG00000081665 Gm15922 pseudogene 1.034 79.277 2.12
87.037 0.454 45.799 0.702 88.245
ENSMUSG00000081666 Gm11677 pseudogene 1 79.433 1
97.129 1 71.615 1 95.273
ENSMUSG00000081667 Gm12883 pseudogene 1 84.865 1
95.504 1 84.724 1 95.273
ENSMUSG00000081668 Gm7869 pseudogene 1 85.499 NA NA
1 55.9 1 95.74
ENSMUSG00000081669 Npm3-ps1 pseudogene 1 74.745 1

90.712	1	81.492	1	96.183
ENSMUSG00000081670	Gm15697	pseudogene	0.987	79.709	1.025
91.643	0.988	88.657	0.918	95.593
ENSMUSG00000081671	Gm13167	pseudogene	1	81.647	1
95.35	1	84.504	1	94.825
ENSMUSG00000081672	Gm13000	pseudogene	1	84.294	1
97.097	1	60.614	1	92.742
ENSMUSG00000081673	Gm14794	pseudogene	1	85.655	1
93.654	1	88.161	1	96.287
ENSMUSG00000081675	NA	NA	NA	NA	0.778	96.013	1.34
81.29	0.535	83.952
ENSMUSG00000081676	Gm11643	pseudogene	1	73.841	1
96.897	1	84.724	1	96.183
ENSMUSG00000081677	Mdk-ps1	pseudogene	1	77.291	1
96.216	NA	NA	1	94.99
ENSMUSG00000081678	Gm12959	pseudogene	1	77.113	1
96.652	1	72.378	1	96.071
ENSMUSG00000081679	Gm11874	pseudogene	0.511	12.084	1.381
84.768	1.412	63.665	0.887	94.654
ENSMUSG00000081680	Gm14473	pseudogene	1	85.092	1
94.848	1	87.971	1	94.904
ENSMUSG00000081681	Gm14868	pseudogene	1	80.688	1
94.597	1	88.461	1	96.086
ENSMUSG00000081683	Fzd10	protein_coding	0.772	33.599	1.313
87.604	0.982	70.9	0.989	91.872
ENSMUSG00000081685	Gm12647	pseudogene	1	67.163	1
97.263	1	78.293	1	95.72
ENSMUSG00000081686	Gm11774	pseudogene	1	85.092	1
91.361	1	78.155	1	92.018
ENSMUSG00000081687	Gm13996	pseudogene	1	85.391	1
92.931	1	73.428	1	95.273
ENSMUSG00000081688	Gm12499	pseudogene	1	80.146	NA	NA
1	86.258	1	94.468
ENSMUSG00000081689	Gm9166	pseudogene	1	86.843	1
94.597	1	77.888	1	94.297
ENSMUSG00000081691	Gm11965	pseudogene	1	81.196	1
91.83	1	84.626	1	92.561
ENSMUSG00000081692	Gm15971	pseudogene	1	86.333	1
93.654	1	68.643	1	94.825
ENSMUSG00000081693	Gm13297	pseudogene	1	81.01	1	95.504
NA	NA	1	96.183
ENSMUSG00000081694	Olfr660-ps1	pseudogene	1	85.499	1
93.094	1	78.954	1	93.132
ENSMUSG00000081695	Rpsa-ps5	pseudogene	1	84.865	1
93.046	1	82.778	1	93.132
ENSMUSG00000081696	Gm12717	pseudogene	1	85.499	1
96.268	1	84.504	1	94.608
ENSMUSG00000081697	Gm14714	pseudogene	1	81.24	1	96.68	1
85.134	1	89.892
ENSMUSG00000081698	Gm12329	pseudogene	1	85.728	1
86.793	1	71.004	1	93.964
ENSMUSG00000081699	Gm15213	pseudogene	1	85.499	1
95.504	1	86.985	1	95.92
ENSMUSG00000081700	Gm14337	pseudogene	1	85.499	1

96.897 1 84.896 1 95.877
ENSMUSG00000081701 Gm13890 pseudogene 1 82.891 1
96.763 1 87.786 1 90.674
ENSMUSG00000081703 NA NA NA NA 1 85.242 1
66.647 1 93.734
ENSMUSG00000081704 Gm14603 pseudogene 1 76.042 NA NA
1 86.727 1 96.248
ENSMUSG00000081705 Gm12582 pseudogene 1 85.391 1
95.504 1 84.724 1 90.865
ENSMUSG00000081706 Gm7927 pseudogene 1 84.564 1
96.013 1 82.685 1 93.964
ENSMUSG00000081707 Gm11343 pseudogene 1 84.865 1
89.996 1 66.043 1 95.92
ENSMUSG00000081709 Gm15022 pseudogene 1 81.178 NA NA
1 87.431 1 95.74
ENSMUSG00000081711 Gm11785 pseudogene 1 85.832 1
94.597 1 88.41 1 94.654
ENSMUSG00000081713 Gm12170 pseudogene 1 84.263 1
97.263 1 81.976 1 93.964
ENSMUSG00000081714 Gm12782 pseudogene 1 66 1 95.708
1 86.475 1 94.567
ENSMUSG00000081715 Rps11-ps3 pseudogene 1 74.021 1
94.48 1 55.81 1 94.752
ENSMUSG00000081716 Gm14839 pseudogene 1 82.982 1
97.288 1 84.724 1 96.086
ENSMUSG00000081717 Vmn1r-ps106 pseudogene 1 85.946 NA NA
1 88.729 1 94.297
ENSMUSG00000081718 Gm8261 pseudogene 1 83.658 1
95.708 1 81.169 1 95.273
ENSMUSG00000081719 Gm13726 pseudogene 1 83.658 1
95.708 1 84.896 1 93.627
ENSMUSG00000081721 Gm13882 pseudogene 1 85.832 1
96.138 1 76.923 1 95.676
ENSMUSG00000081722 Gm11763 pseudogene 1 72.743 1
96.407 1 87.519 1 93.964
ENSMUSG00000081723 Gm15931 pseudogene 2.019 0.355 1.39 87.604
0.357 1.342 1.175 92.564
ENSMUSG00000081724 Olfr129 protein_coding 1 86.819 1
96.843 1 84.896 1 93.964
ENSMUSG00000081728 Gm14930 pseudogene 1 79.165 NA NA
1 85.792 1 93.457
ENSMUSG00000081729 Hspa9-ps1 pseudogene 1 86.199 NA NA
1 88.729 1 91.372
ENSMUSG00000081730 Gm13785 pseudogene 0.975 81.76 2.266 71.98
0.452 20.712 1.746 79.442
ENSMUSG00000081731 Gm14078 pseudogene 1 77.182 1
95.708 1 88.693 1 94.82
ENSMUSG00000081732 Gm15495 pseudogene 1 64.589 1
93.994 1 85.792 1 96.287
ENSMUSG00000081733 Gm12852 pseudogene 1 82.982 1
96.68 1 85.921 1 94.904
ENSMUSG00000081734 Gm12740 pseudogene 1 80.03 1 97.155
1 85.757 1 96.248
ENSMUSG00000081735 Gm14530 pseudogene 1 79.93 1 91.897

1 75.527 1 95.92
ENSMUSG00000081736 Gm14971 pseudogene 1 85.637 1
94.48 1 85.792 1 92.675
ENSMUSG00000081737 Olfr359-ps1 pseudogene 1 77.916 1
94.96 1 79.325 1 93.156
ENSMUSG00000081738 Hmgb1-ps2 pseudogene 1 82.479 1
95.735 1 83.397 1 93.584
ENSMUSG00000081739 Mdm4-ps pseudogene 1 82.891 1
95.431 1 84.961 1 90.177
ENSMUSG00000081741 NA NA NA NA 1 95.504 1
86.258 1 93.734
ENSMUSG00000081742 Gm9051 pseudogene 1 85.946 1
96.138 1 87.971 1 95.92
ENSMUSG00000081743 Gm4911 pseudogene 1 81.01 1 92.137
1 80.5 1 96.183
ENSMUSG00000081744 Gm5721 pseudogene 1 81.01 1 95.708
1 88.076 1 94.567
ENSMUSG00000081746 Gm13925 pseudogene 1 63.126 1
94.597 1 86.666 1 96.183
ENSMUSG00000081747 Rpl7a-ps9 pseudogene 1 34.162 1
86.277 1 79.119 1 93.734
ENSMUSG00000081749 Gm12241 pseudogene 1 86.199 1
97.263 1 88.161 1 93.685
ENSMUSG00000081750 Gm4995 pseudogene 1 86.199 1
95.088 1 85.464 1 96.086
ENSMUSG00000081752 Gm14680 pseudogene 1 80.408 1
97.288 1 87.431 1 95.273
ENSMUSG00000081755 Gm12574 pseudogene 1 85.59 NA NA 1
86.666 1 95.273
ENSMUSG00000081756 Gm12291 pseudogene 1 86.843 NA NA
NA NA 1 93.132
ENSMUSG00000081757 Gm13798 pseudogene 1 81.52 1 95.504
1 84.308 1 94.99
ENSMUSG00000081761 Gm6298 pseudogene 1 67.163 1
97.263 1 87.744 1 95.273
ENSMUSG00000081762 Gm13738 pseudogene 1 76.968 1
93.817 1 77.17 1 91.525
ENSMUSG00000081763 Gm9435 pseudogene 1 83.523 1
88.691 1 78.498 1 96.183
ENSMUSG00000081765 Gm14677 pseudogene 1 83.823 1
94.597 1 76.498 1 95.405
ENSMUSG00000081766 2210409E12Rik pseudogene 1 82.231 1
94.597 1 82.74 1 96.192
ENSMUSG00000081767 Gm12067 pseudogene 1 72.546 1
95.088 1 86.831 1 95.877
ENSMUSG00000081768 Gm12755 pseudogene 1 84.397 1
91.361 1 83.857 1 95.72
ENSMUSG00000081769 Gm12216 protein_coding 1.057 40.88 1.082
82.904 0.871 1.92 0.911 83.724
ENSMUSG00000081770 Rbpsuh-ps1 pseudogene 1 81.823 1
96.216 1 85.58 1 92.675
ENSMUSG00000081771 Gm15381 pseudogene 1 61.334 1
94.48 1 67.562 1 93.117
ENSMUSG00000081772 Gm13131 pseudogene 1 85.77 1 97.129

1 85.134 1 95.832
ENSMUSG00000081773 Gm8302 pseudogene 1 82.982 1
83.733 1 67.163 1 92.52
ENSMUSG00000081774 Gm13866 pseudogene 1 63.804 1
95.708 1 86.602 1 90.125
ENSMUSG00000081775 Gm12420 pseudogene 1 81.76 1 95.088
1 76.754 1 96.287
ENSMUSG00000081777 Gm14159 pseudogene 1 82.479 1
92.137 1 69.196 1 93.261
ENSMUSG00000081778 Gm6325 pseudogene 1 86.172 NA NA
1 88.349 1 94.153
ENSMUSG00000081779 Gm21998 pseudogene 1 86.843 1
96.216 1 78.713 1 94.215
ENSMUSG00000081781 Gm14753 pseudogene 1 82.231 1
93.654 1 88.601 1 95.74
ENSMUSG00000081782 Gm15152 pseudogene 1 85.129 NA NA
1 86.346 1 93.964
ENSMUSG00000081783 Gm15599 pseudogene 1 86.145 1
91.532 1 71.839 1 96.086
ENSMUSG00000081784 Gm13281 pseudogene 1 69.489 NA NA
1 76.768 1 93.964
ENSMUSG00000081786 Gm11897 pseudogene 1 74.8 1 96.68 1
87.37 1 96.183
ENSMUSG00000081787 Gm13991 pseudogene 1 86.474 1
94.262 1 87.686 1 96.248
ENSMUSG00000081788 Gm5898 pseudogene 1 71.217 1
95.941 NA NA 1 95.877
ENSMUSG00000081789 Gm11893 pseudogene 1 84.324 1
96.216 NA NA 1 93.457
ENSMUSG00000081791 Gm12190 pseudogene 1 85.857 1
97.097 1 86.985 1 95.72
ENSMUSG00000081792 Anp32b-ps1 pseudogene 1.727 27.32 NA NA
0.624 35.559 0.537 85.493
ENSMUSG00000081796 Gm9005 pseudogene 1 67.163 1
97.155 NA NA 1 95.74
ENSMUSG00000081797 Gm8662 pseudogene 1 86.57 1 96.68 1
87.898 1 94.001
ENSMUSG00000081799 Gm14071 pseudogene 1 74.958 1
96.138 1 86.666 1 95.832
ENSMUSG00000081800 Gm14200 pseudogene 1 86.112 1
96.268 1 88.076 1 93.132
ENSMUSG00000081801 Dnmt3l-ps1 pseudogene 1 86.333 1
87.452 1 55.512 1 94.384
ENSMUSG00000081803 Gm15282 pseudogene 1 69.985 NA NA
1 86.985 1 94.904
ENSMUSG00000081804 Xlr5d-ps pseudogene 1 73.487 1
95.301 1 81.29 1 94.654
ENSMUSG00000081806 Gm14901 pseudogene 1 79.709 1
95.708 1 88.076 1 96.042
ENSMUSG00000081807 Gm14849 pseudogene 1 82.982 1
96.763 1 88.41 1 96.358
ENSMUSG00000081809 Gm15539 pseudogene 1.038 83.823 0.922
94.102 1.046 68.129 0.977 95.273
ENSMUSG00000081810 Gm11878 pseudogene 1 86.918 1

96.897 1 83.158 1 95.559
ENSMUSG00000081812 Gm4789 pseudogene 1 85.77 NA NA 1
70.1 1 93.264
ENSMUSG00000081813 Gm12806 pseudogene 1 77.497 1
96.216 NA NA 1 96.287
ENSMUSG00000081814 Gm14695 pseudogene 1 86.843 1
96.335 NA NA 1 94.215
ENSMUSG00000081817 Gm13590 pseudogene 1 78.538 1
91.83 1 83.719 1 93.612
ENSMUSG00000081818 Gm12652 pseudogene 1 84.865 1
91.532 1 83.811 1 95.273
ENSMUSG00000081819 Gm12722 pseudogene 1 82.479 1
93.654 NA NA 1 96.183
ENSMUSG00000081820 Gm12029 pseudogene 1 79.93 NA NA 1
81.62 1 94.904
ENSMUSG00000081821 Gm15158 pseudogene 1 84.294 1
90.138 1 84.896 1 92.851
ENSMUSG00000081822 Gm15626 pseudogene 1 85.59 1 95.211
1 85.757 1 96.183
ENSMUSG00000081823 Cyp4a28-ps pseudogene 1 73.401 1
96.68 1 79.897 NA NA
ENSMUSG00000081824 BC002163 pseudogene 1.89 34.861 2.761
71.98 0.192 8.634 1.043 85.629
ENSMUSG00000081827 Gm15103 pseudogene 1 84.738 1
96.013 NA NA 1 90.674
ENSMUSG00000081830 Gm13475 pseudogene 1 81.925 1
96.897 1 87.336 1 96.248
ENSMUSG00000081831 Gm7405 pseudogene 1 82.092 1
97.263 1 88.601 1 96.192
ENSMUSG00000081832 Gm6897 pseudogene 1 71.217 1
95.301 1 87.971 1 95.676
ENSMUSG00000081833 Gm13669 pseudogene 2.312 12.072 1.049
94.848 0.41 16.051 0.307 83.724
ENSMUSG00000081834 Gm13770 pseudogene 1 73.401 NA NA
1 78.713 1 96.358
ENSMUSG00000081836 Olfr1192-ps1 pseudogene 1 85.129 NA
NA NA NA 1 96.183
ENSMUSG00000081837 Gm14649 pseudogene 1 78.299 NA NA
1 87.37 1 96.287
ENSMUSG00000081838 Gm13038 pseudogene 1 70.862 NA NA
1 84.434 1 96.287
ENSMUSG00000081839 Gm13454 pseudogene 1 81.465 1
96.138 1 82.31 1 96.183
ENSMUSG00000081840 Gm15220 pseudogene 1 85.499 1
96.138 1 81.29 1 96.248
ENSMUSG00000081841 Gm13444 pseudogene 1 75.858 NA NA
1 85.757 1 96.192
ENSMUSG00000081842 Rap1a-ps2 pseudogene 1 86.006 NA NA
1 87.898 1 89.827
ENSMUSG00000081843 Gm7851 pseudogene 1 82.982 1
95.735 1 81.976 1 95.74
ENSMUSG00000081844 Gm11241 pseudogene 1 83.968 NA NA
1 73.874 1 96.042
ENSMUSG00000081845 Prkaca-ps1 pseudogene 1 80.688 1

95.941 1 84.504 1 92.12
ENSMUSG00000081847 Gm12142 pseudogene 1 85.59 1 96.138
1 83.397 1 96.192
ENSMUSG00000081848 Gm14925 pseudogene 1 86.006 1
97.155 1 88.203 1 94.174
ENSMUSG00000081849 Gm15847 pseudogene 1 86.199 1
97.263 1 86.552 1 95.676
ENSMUSG00000081851 Gm13323 pseudogene 1 84.294 1
96.216 1 87.103 1 96.042
ENSMUSG00000081853 Gm13735 pseudogene 1 85.355 1
96.948 1 85.464 1 95.273
ENSMUSG00000081854 Gm11447 pseudogene 1 78.054 1
96.68 NA NA 1 92.181
ENSMUSG00000081856 Gm14580 pseudogene 1 82.092 1
95.708 1 86.112 1 94.82
ENSMUSG00000081857 Gm13624 pseudogene 1 65.388 1
95.301 1 74.325 1 92.381
ENSMUSG00000081858 Gm14514 pseudogene 1 82.732 NA NA
1 85.921 1 95.777
ENSMUSG00000081859 Olfr650-ps1 pseudogene 1 86.843 1
95.504 1 79.625 1 95.559
ENSMUSG00000081860 Gm11533 pseudogene 1 73.841 1
96.984 1 78.642 1 86.991
ENSMUSG00000081861 Gm14441 pseudogene 1 66.652 1
97.263 1 80.089 1 96.358
ENSMUSG00000081862 Gm12413 pseudogene 1 70.121 1
87.857 1 84.229 1 95.74
ENSMUSG00000081863 Gm12780 pseudogene 1 81.125 NA NA
1 84.83 1 95.877
ENSMUSG00000081864 Olfr405-ps1 pseudogene 1 85.832 1
97.155 1 85.58 1 92.675
ENSMUSG00000081865 Gm15484 pseudogene 1 82.479 1
94.262 1 87.519 1 94.384
ENSMUSG00000081866 Gm12401 pseudogene 1 66.944 1
96.407 1 85.921 1 93.734
ENSMUSG00000081868 Gm12039 pseudogene 1 64.401 1
91.661 1 72.148 1 96.358
ENSMUSG00000081869 Gm15002 pseudogene 1 78.538 NA NA
1 83.719 1 96.183
ENSMUSG00000081870 Gm15141 pseudogene 1 66 1 96.843
1 84.724 1 96.086
ENSMUSG00000081871 Gm11488 pseudogene 1 86.918 1
95.211 1 85.134 1 95.74
ENSMUSG00000081872 Gm15572 pseudogene 1 62.354 1
95.708 1 84.896 1 89.72
ENSMUSG00000081873 Gm15197 pseudogene 0.584 48.146 0.865
95.504 1.965 53.882 1.413 96.287
ENSMUSG00000081874 Gm14983 pseudogene 1 83.263 NA NA
NA NA 1 93.132
ENSMUSG00000081875 Gm15223 pseudogene 1 77.497 NA NA
1 83.811 1 92.762
ENSMUSG00000081876 Gm12644 pseudogene 1 70.36 1 93.765
1 82.778 1 95.72
ENSMUSG00000081877 Gm8858 pseudogene 1 72.655 NA NA

1 87.744 1 95.777
ENSMUSG00000081878 Gm13739 pseudogene 1 79.577 NA NA
1 88.693 1 96.358
ENSMUSG00000081879 Gm11933 pseudogene 1 72.743 1
96.335 1 79.897 1 93.264
ENSMUSG00000081880 Gm5385 pseudogene 1 86.145 1
94.597 1 86.144 1 96.183
ENSMUSG00000081881 Gm14752 pseudogene 1 86.464 1
93.654 1 84.504 1 91.625
ENSMUSG00000081882 Gm14294 pseudogene 1 81.465 1
96.268 1 87.786 1 94.215
ENSMUSG00000081884 Gm11821 pseudogene 2.041 35.499 0.344
55.265 1.425 58.616 0.946 92.691
ENSMUSG00000081885 Gm13231 pseudogene 1 84.661 NA NA
NA NA 1 91.372
ENSMUSG00000081886 Gm6175 pseudogene 1 84.294 1
96.652 1 80.899 1 94.468
ENSMUSG00000081887 Gm12097 pseudogene 1.299 51.826 0.764
91.532 1.008 87.431 0.769 85.493
ENSMUSG00000081888 Spcs2-ps pseudogene 1 82.231 NA NA
1 85.352 1 95.74
ENSMUSG00000081889 Gm13188 pseudogene 1 68.955 1
96.138 1 80.163 1 95.74
ENSMUSG00000081890 Gm11243 pseudogene 1 85.832 1
97.263 1 83.925 1 95.273
ENSMUSG00000081891 Gm14660 pseudogene 1 81.465 1
94.48 1 87.658 1 94.654
ENSMUSG00000081892 Gm8864 pseudogene 1 82.092 NA NA
1 88.601 1 95.273
ENSMUSG00000081893 Gm13273 pseudogene 1 66.76 1 93.994
1 79.255 1 96.248
ENSMUSG00000081894 Gm14212 pseudogene 1 84.263 1
91.584 1 82.903 1 92.31
ENSMUSG00000081895 Gm10294 pseudogene 1 72.139 1
95.735 1 87.37 1 93.016
ENSMUSG00000081896 Gm5389 pseudogene 1 84.738 1
95.708 1 78.498 1 96.248
ENSMUSG00000081897 Gm15067 pseudogene 1 82.479 1
96.847 1 85.757 1 95.72
ENSMUSG00000081898 Gm5754 pseudogene 1 61.887 1
90.29 1 87.274 1 92.018
ENSMUSG00000081899 Gm13606 pseudogene 1 57.544 1
94.767 1 87.744 1 95.559
ENSMUSG00000081900 Gm14985 pseudogene 1 77.916 1
96.216 1 84.308 1 96.183
ENSMUSG00000081901 Gm15357 pseudogene 1 86.843 NA NA
1 76.388 1 93.413
ENSMUSG00000081903 Gm11387 pseudogene 1 85.59 NA NA 1
84.308 1 95.72
ENSMUSG00000081904 Gm13419 pseudogene 1 75.196 1
97.155 NA NA 1 93.734
ENSMUSG00000081905 Gm11370 pseudogene 1 82.982 1
97.049 1 75.527 1 93.457
ENSMUSG00000081906 Rpl9-ps1 protein_coding 1 83.823 1

90.378 1 86.552 1 93.734
ENSMUSG00000081907 Gm13731 pseudogene 1 82.891 1
96.013 1 84.724 1 94.82
ENSMUSG00000081908 Olfr1147-ps1 pseudogene 1 86.74 1
96.897 1 81.857 1 95.273
ENSMUSG00000081909 Gm11341 pseudogene 1 85.728 1
96.847 1 76.388 1 96.071
ENSMUSG00000081910 Gm15986 pseudogene 1 85.391 1
97.263 1 87.519 1 96.248
ENSMUSG00000081911 Mrps36-ps2 pseudogene 1 82.352 NA NA
1 85.935 1 93.264
ENSMUSG00000081912 Vmn1r-ps127 pseudogene 1 84.397 NA NA
1 82.74 1 96.248
ENSMUSG00000081913 Gm11803 pseudogene 1 81.647 1
96.68 1 84.552 1 96.248
ENSMUSG00000081914 Vmn1r-ps109 pseudogene 1 86.172 1
95.708 1 87.658 1 94.016
ENSMUSG00000081915 Gm15174 pseudogene 1 77.916 1
96.897 1 80.163 1 96.287
ENSMUSG00000081916 Gm12899 pseudogene 1 82.092 NA NA
1 68.129 1 96.086
ENSMUSG00000081917 Gm15209 pseudogene 1 78.538 1
96.68 1 87.744 1 96.183
ENSMUSG00000081918 Gm14613 pseudogene 1 84.865 1
96.847 1 87.744 1 94.904
ENSMUSG00000081919 Gm12399 pseudogene 1 86.006 1
93.908 1 86.144 1 95.72
ENSMUSG00000081920 Gm15359 pseudogene 1 85.655 1
93.094 1 72.695 1 94.19
ENSMUSG00000081921 Gm12858 pseudogene 1 76.396 1
95.35 1 80.015 1 93.457
ENSMUSG00000081922 Gm14646 pseudogene 1 68.846 1
96.216 1 86.144 1 95.273
ENSMUSG00000081923 Gm9062 pseudogene 1 73.292 1
95.708 NA NA 1 92.564
ENSMUSG00000081924 Gm11191 pseudogene 1 86.006 1
85.671 1 83.719 1 94.608
ENSMUSG00000081925 Rpl31-ps22 pseudogene 1 84.738 1
95.708 1 83.397 1 92.564
ENSMUSG00000081928 Gm15105 pseudogene 1 75.399 1
88.691 1 60.5 1 91.807
ENSMUSG00000081929 Rps11-ps2 pseudogene 1 81.01 1 94.597
1 67.163 1 96.358
ENSMUSG00000081930 Gm12345 pseudogene 1.563 77.497 1.024
95.708 0.625 87.686 1.038 96.358
ENSMUSG00000081931 Gm15211 pseudogene 1 84.819 1
97.155 1 85.464 1 95.832
ENSMUSG00000081932 Gm12331 pseudogene 1 84.865 1
96.68 1 87.898 1 96.248
ENSMUSG00000081933 Gm14767 pseudogene 1 73.487 1
95.504 1 83.397 1 91.928
ENSMUSG00000081935 Gm16186 pseudogene 1 72.27 1 96.847
1 83.925 1 96.086
ENSMUSG00000081936 Gm11244 pseudogene 1 85.59 1 96.216

1 87.744 1 96.287
ENSMUSG00000081937 Gm14599 pseudogene 1 84.865 1
96.138 1 86.602 1 95.273
ENSMUSG00000081938 Gm15039 pseudogene 1 85.391 1
95.504 1 86.112 1 96.183
ENSMUSG00000081939 Gm15399 pseudogene 1 85.655 1
96.407 1 85.464 1 95.877
ENSMUSG00000081940 Xlr3e-ps pseudogene 1 77.291 1
92.062 1 82.903 1 96.287
ENSMUSG00000081941 Gm11218 pseudogene 1 81.196 1
95.504 1 81.976 1 95.273
ENSMUSG00000081942 Gm15149 pseudogene 1 82.092 1
96.216 1 88.729 1 96.086
ENSMUSG00000081943 Gm9078 pseudogene 1 85.832 NA NA
1 86.258 1 94.82
ENSMUSG00000081944 Gm12276 pseudogene 0.761 10.29 1.29 71.617
1.019 88.693 1.243 49.514
ENSMUSG00000081945 Gm15121 pseudogene 1 84.294 1
96.013 1 86.727 1 96.183
ENSMUSG00000081946 Gm11472 pseudogene 1 86.843 1
94.48 1 77.888 1 93.457
ENSMUSG00000081947 Gm14512 pseudogene 1 85.857 1
97.049 1 85.792 1 94.904
ENSMUSG00000081948 Olfr1191-ps1 pseudogene 1 81.823 1
96.984 1 87.519 1 96.183
ENSMUSG00000081949 Gm14392 pseudogene 1 85.728 1
96.68 1 88.693 1 78.948
ENSMUSG00000081950 Gm11600 pseudogene 1 85.637 1
90.454 1 69.583 1 96.248
ENSMUSG00000081951 Gm15352 pseudogene 1 51.297 1
80.052 1 86.346 1 92.31
ENSMUSG00000081952 Gm12178 pseudogene 1 77.182 1
93.654 1 67.648 1 96.358
ENSMUSG00000081954 Gm13626 pseudogene 1 48.857 1
91.532 NA NA 1 93.612
ENSMUSG00000081955 Olfr999-ps1 pseudogene 1 81.76 1 97.263
1 78.155 1 93.722
ENSMUSG00000081957 Ak3l2-ps pseudogene 1 86.843 1
96.847 1 87.744 1 94.904
ENSMUSG00000081958 Gm11594 pseudogene 1 84.865 1
96.138 1 69.825 1 94.82
ENSMUSG00000081959 Gm16480 pseudogene 1 83.09 1 95.211
1 75.527 1 92.561
ENSMUSG00000081960 Mageb17-ps pseudogene 1 81.647 NA NA
1 81.29 1 96.358
ENSMUSG00000081961 Olfr383-ps1 pseudogene 1 85.499 1
95.708 1 80.089 1 95.35
ENSMUSG00000081962 Gm12773 pseudogene 1 83.333 1
96.897 1 83.607 1 95.35
ENSMUSG00000081964 Gm11873 pseudogene 1 81.24 1 96.652
1 84.071 1 95.273
ENSMUSG00000081965 Gm11620 pseudogene 0.546 17.062 1.02
95.211 1.796 16.446 0.618 95.74
ENSMUSG00000081966 Gm13900 pseudogene 1 81.823 1

97.155 1 87.37 1 95.74
ENSMUSG00000081967 Gm14017 pseudogene 1 74.299 1
96.847 1 87.519 1 91.928
ENSMUSG00000081968 Rpl23a-ps2 pseudogene 1.039 83.658 1.007
88.533 0.954 76.64 0.849 86.991
ENSMUSG00000081969 Gm16471 pseudogene 1 86.351 1
94.48 1 85.464 1 94.001
ENSMUSG00000081970 Gm16057 pseudogene 1 80.751 1
96.216 NA NA 1 93.964
ENSMUSG00000081971 Gm14115 pseudogene 1 80.51 1 94.102
1 86.475 1 95.273
ENSMUSG00000081973 Gm13600 pseudogene 1 81.657 1
93.707 1 87.686 1 95.92
ENSMUSG00000081975 NA NA NA NA 1 96.68 1 85.675
1 95.593
ENSMUSG00000081976 Gm14823 pseudogene 1 83.169 1
96.897 1 86.727 1 93.734
ENSMUSG00000081977 Flg processed_transcript 1 78.224 1
96.68 1 84.724 1 96.183
ENSMUSG00000081978 Gm14895 pseudogene 1 84.324 NA NA
1 87.103 1 94.825
ENSMUSG00000081979 Gm8078 pseudogene 1 85.203 1
89.494 1 74.853 1 94.82
ENSMUSG00000081980 Gm13702 pseudogene 1 80.03 1 94.102
1 85.352 1 95.74
ENSMUSG00000081982 Gm6168 pseudogene 1 83.968 1
93.654 1 83.811 1 96.183
ENSMUSG00000081984 Dnajb3 protein_coding 0.983 81.24 1.285
95.708 0.794 83.811 1 96.248
ENSMUSG00000081985 Gng2-ps1 pseudogene 1 85.283 1
96.216 NA NA 1 96.183
ENSMUSG00000081986 Gm14841 pseudogene 1 84.049 1
94.597 1 64.024 1 94.82
ENSMUSG00000081987 NA NA NA NA 1 96.68 1 81.388
1 96.183
ENSMUSG00000081990 NA NA NA NA 1 95.431 1
85.58 1 96.086
ENSMUSG00000081991 Gm13859 pseudogene 0.228 53.407 0.945
92.914 4.627 58.051 3.389 92.018
ENSMUSG00000081993 Gm8475 pseudogene 1 84.534 NA NA
1 77.704 1 93.457
ENSMUSG00000081994 Gm14517 pseudogene 1 65.537 1
97.049 1 78.713 1 95.273
ENSMUSG00000081995 Gm11719 pseudogene 1 73.841 1
95.504 1 87.274 1 88.657
ENSMUSG00000081997 Gm11886 pseudogene 1 84.294 NA NA
1 81.032 1 95.405
ENSMUSG00000081998 Gm13245 pseudogene 0.903 76.396 0.697
85.116 1.593 26.912 1.047 95.92
[truncated: 460,863 more chars]
